# Evidence of Physiological Comodulation During Human–Animal Interaction: A Systematic Review

**DOI:** 10.1111/nyas.70299

**Published:** 2026-06-04

**Authors:** Ginevra Bargigli, Lorenzo Frassineti, Paolo Baragli, Chiara Scopa, Aglaia Vignoli, Antonio Lanata

**Affiliations:** ^1^ ComPBioS, Department of Information Engineering University of Florence Florence Italy; ^2^ Department of Veterinary Sciences University of Pisa Pisa Italy; ^3^ Department of Health Sciences University of Milan Milan Italy

**Keywords:** comodulation, cortisol, HAI, heart rate, human–animal interaction, physiological, synchrony

## Abstract

This review examines the evidence in the literature for physiological co‐modulation during human–animal interaction, identifying studies that assessed comodulation via simultaneous measurement of physiological signals in both species, performing quantitative comparisons. We searched (last search: August 5, 2025) PubMed, EMBASE, Scopus, Google Scholar, Animal Studies Repository, Cochrane, and the Consensus App academic search engine. Risk of bias was assessed using an adapted version of the ROBINS‐I V2 tool. The results, grouped by data analysis method, interaction context, and physiological parameter, were synthesized narratively in structured tables. Thirty‐seven studies were included, focusing on dogs (*n* = 22) and horses (*n* = 15), framed primarily within the interaction contexts of animal‐assisted therapy/intervention and companionship. Cardiac and hormonal measures were most frequently assessed. Most studies (*n* = 20) performed correlation analyses. Sample sizes ranged from ≤10 to ≥130 dyads. Comodulation was significant in 16 studies, partial in 16, and absent in 5. Time‐series coupling methods yielded more consistent evidence than discrete‐time correlations. Evidence, while not conclusive, supports physiological comodulation during human–animal interactions. However, the studies' heterogeneity limits generalizability; findings suggest comodulation may emerge under specific biological and methodological conditions, and future research should explicitly test its presence across contexts.

## Graphical abstract

From thirty‐seven studies on physiological comodulation in human–animal interaction, dogs and horses emerged as the most studied species, primarily in therapeutic and companionship settings. Cardiac and hormonal signals dominated the analyses, with correlation methods prevailing. Comodulation was significant in most cases, yet methodological diversity limits generalization. The evidence suggests that physiological synchrony may emerge under specific biological and contextual conditions.

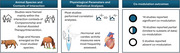

## 1 Introduction

### 1.1 Rationale

The present review was conducted to examine what evidence the literature provides in support of the existence of physiological synchrony—or, more conservatively, physiological comodulation [1]—between interacting humans and animals. In human–animal interaction (HAI) research, this phenomenon has been increasingly investigated as a potential marker of emotional attunement, mutual regulation, and relational bonding, echoing findings from human studies [2, 3, 4, 5, 6]. However, the conceptual and methodological landscape remains fragmented. First, while this phenomenon has been established in human research as an index of such processes, its adoption in HAI research should be approached with caution, as neither its presence nor its role in bonding processes have been conclusively established in this context.

Furthermore, studies frequently adopt the term *synchrony* in a broad manner, applying it interchangeably to time‐resolved coupling and static associations, and relying on heterogeneous analytical approaches. Consequently, a recent study proposed the term *comodulation* to denote reciprocal physiological influences observed over extended temporal windows, without implying moment‐to‐moment temporal alignment or the availability of densely sampled time‐series required for classical synchrony metrics (e.g., coherence or phase‐based analyses), while still preserving temporal overlap between interacting partners [1]. This broader conceptualization accommodates the diversity of physiological signals and interaction contexts observed in HAI studies, ranging from therapeutic interventions and companionship to sport and working environments. Despite growing interest, the question of whether physiological comodulation may represent a generalizable feature of HAI or a context‐dependent phenomenon shaped by species, interaction context, sampling modality, and analytical method remains unresolved.

### 1.2 Objectives

To address this gap, we conducted a systematic review focused on studies that simultaneously measured physiological signals in humans and animals during interaction, and performed quantitative comparisons. By synthesizing existing evidence, our aim was to map the presence and methodological characteristics of physiological comodulation across diverse interaction contexts, species, and analytical frameworks. The aim of this study is coherently represented through the PICO framework (Box 1), which provides a structured approach to delineating the population, intervention, comparison, and outcomes of a systematic review [7].

1 **BOX 1|PICO framework for this review**

- Population: Humans and animals engaged in interaction.
- Intervention/Exposure: Simultaneous measurement of physiological parameters during HAI.
- Comparator: No interaction, asynchronous measurement, or baseline.
- Outcome: Reported evidence of physiological comodulation.

## 2 Methods

### 2.1 Eligibility Criteria

Eligibility criteria were defined based on the review's research question, as well as the PICO framework of this work (Box 1). Neither the human nor the animal populations to be included in the studies were subjected to any restrictive criteria: studies involving any human participants (regardless of age, sex, or health status) and any animal species were considered eligible. Only studies that simultaneously measured physiological parameters in both humans and animals during an interaction were considered eligible. Simultaneity was broadly interpreted as physiological data being recorded in overlapping time windows, with a temporal resolution sufficiently similar to allow a comparison. No minimum or maximum duration of physiological recording was required for inclusion. Specifically, in the present review, simultaneity refers to the overlapping acquisition of physiological signals in humans and animals during an interaction, irrespective of whether the data allow fine‐grained time‐series analyses or only comparisons at a coarser, aggregated level. No restrictions were applied regarding study design; both observational and experimental studies were considered eligible. Moreover, the type of interaction, as well as the setting, were not subject to any restriction criteria. No restrictions were applied to the specific physiological parameters measured or the technical features of data acquisition. However, the search strategy included terms related to commonly used physiological measures (e.g., electroencephalography [EEG], photoplethysmography [PPG], functional near‐infrared spectroscopy [fNIRS], heart rate, oxytocin, cortisol, breath) to enhance sensitivity. No formal comparator was required for inclusion. Notably, comparators were not consistently present across included studies. When present, although classifiable in general as “no interaction” conditions, comparators were heterogeneous and highly specific to individual study designs, further limiting their utility for synthesis purposes. Studies were included if they performed any quantitative analysis to assess comodulation, interpreted as any comparative statistical evaluation between variations of human and animal physiological signals. Thus, papers that measured physiological parameters in both humans and animals but did not quantitatively compare those measures, performing separate analysis pipelines, were excluded. Comodulation was broadly defined to include both time‐resolved coupling and static associations.

To ensure methodological rigor and data availability, papers that had not been peer reviewed, master's or bachelor's theses, unpublished papers, conference papers, and papers written in languages other than English were excluded. In performing citation searching, a filter for papers published after 2024 was applied in order to limit the inclusion to the most recent advances on the subject. No other restrictions were applied regarding the year of publication. Studies in which the outcome of interest was not measured at all were excluded in the title‐screening step of the selection; exclusions attributed to the absence of outcome explicit report were performed in the text‐screening step of the selection.

No formal protocol was registered for this review. However, all methodological decisions, including post hoc amendments, were transparently reported in accordance with PRISMA 2020 guidelines [8]. For the synthesis, data were grouped according to three main dimensions: the data analysis method (e.g., time‐series coupling analysis, regression analysis, correlation analysis), the context of the interaction (i.e., animal‐assisted intervention/ animal‐assisted therapy [AAT/AAI], companionship, competitive sport, noncompetitive sport, working animals), and the measured physiological parameter (e.g., heart rate variability [HRV], beats per minute [bpm], cortisol, oxytocin). It is important to note that although animal‐assisted therapy interactions are encompassed within the broader AAI framework [9], the AAT label was retained when explicitly used by authors to preserve intervention‐specific detail and to avoid unnecessary loss of granularity. Detailed operational definitions of all interaction categories are available in the Data Dictionary (File S4).

As the included literature reports a wide and highly heterogeneous set of physiological parameters, we presented the distribution of physiological measures across studies (see Section 3.3) by aggregating individual parameters into physiological parameter classes (Box 2). This grouping was employed to enhance comparability and highlight cross‐study patterns; classes were defined to reflect (i) broad biological interpretation and (ii) the temporal granularity of the sampled signal (i.e., continuous time‐series vs. discrete sampling). To further explore the heterogeneity of the included studies along the physiological parameter dimension, a supplementary table was compiled to report the occurrence of different sampling methods for molecular physiological parameters (see Table S6). For the context of interaction and physiological parameter dimensions, a further subdivision was made based on the animal species enrolled in the studies (e.g., dog, horse, others).

1 **BOX 2|Physiological parameter classes**

- Cardiac activity (e.g., heart rate variability [HRV], beats per minute [bpm])
- Hormones (e.g., cortisol, oxytocin)
- Multiparameter (combinations of cardiac and hormonal measures)
- Other (e.g., EEG, breathing rate, testosterone, CgA, ACTH, β‐endorphin, epinephrine, norepinephrine, T3, T4, biochemical parameters (Na,K, CREA, urea, TP, ALB, Mg, AP, CK, ALT, AST), hematological parameters (WBC, RBC, HGB, HCT, MCV, MCH, MCHC, PLT, LYM, GRA)
*Note*: Full names of abbreviations are provided in the accompanying Data Dictionary (see File S4).

These dimensions were adopted to adequately represent the heterogeneity observed across studies, particularly along three dimensions of the PICO framework (Box 1): Population (animal species), Intervention (context of interaction and physiological parameter measured), and Outcome (data analysis method). All narrative syntheses, structured tables, and graphical visualizations were used to present the distribution of outcomes across key methodological dimensions without drawing inferential conclusions, as the performed narrative synthesis does not aim to quantify effect sizes or test hypotheses.

### 2.2 Information Sources

The following databases were searched: PubMed, EMBASE, Scopus, Google Scholar, Animal Studies Repository, and Cochrane. Additionally, the AI‐powered academic search engine Consensus App (https://consensus.app/) was interrogated. Although not a traditional bibliographic database, Consensus App was included to enhance sensitivity and identify potentially relevant studies not indexed in standard databases [10].

**FIGURE 1:**
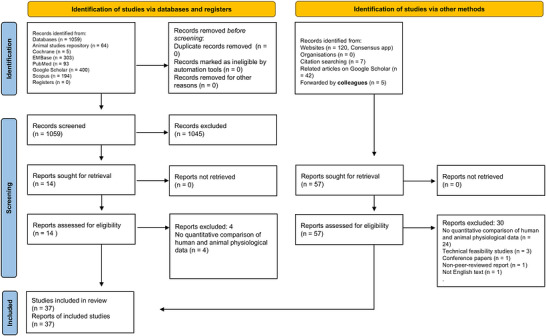
PRISMA 2020 flow diagram —the diagram illustrates the number of records identified, screened, and included in this review.

A citations search was also performed for each eligible entry identified through database and Consensus App tool interrogation (via Google Scholar web interface), and experts from the CoMPBioS (Department of Information Engineering, University of Florence, Firenze, Italy) network were consulted to identify additional eligible studies. All details on the information sources are reported in the PRISMA flow diagram (Figure 1) and PRISMA search strategy document of this review (see File S1).

### 2.3 Search Strategy

All searches were run through the web interfaces of each source using simple Boolean keyword combinations entered manually (without field tags, advanced syntax, or controlled vocabulary, e.g., “human animal interaction” AND “EEG”), and the Consensus App was queried using natural‐language question prompts. Initial terms and prompts were derived from the Intervention element of the PICO (Box 1); the term “physiological” was interpreted as defined by Frize et al. [11], and the term “correlate” was chosen to capture any quantitative assessment of comodulation from simultaneous human–animal measurements. Screening of preliminary search results indicated that relevant literature extended beyond AAT/AAI contexts, in which both the terms “human–animal interaction” and its acronym HAI are well established [12]. This finding was in contrast to the a priori assumption that relevant studies would be situated within these contexts, thus revealing a potential sensitivity limitation of the initial keyword strategy. To improve sensitivity, additional searches used specific physiological signal terms (e.g., EEG, PPG, fNIRS, heart rate, oxytocin, cortisol, breathing). All detailed information on the search strategy is reported in the PRISMA Search strategy document of this review (see File S1).

### 2.4 Selection Process

Two reviewers conducted independent searches using the same procedure; the second reviewer retrieved one additional record [13], later excluded at full‐text screening for not meeting the comodulation analysis criterion. No automation tools were used. In total, 1179 titles were screened and 71 full texts were assessed. A total of 37 studies were ultimately included, showing substantial inter‐reviewer agreement (Cohen's kappa = 0.94). Search results were saved as a Zotero collection (see File S2). The PRISMA flow diagram (Figure 1) and PRISMA search strategy document (see File S1) for this review provide a complete overview of the process.

### 2.5 Data Collection Process

Data were extracted from the full texts of the included studies using a structured Excel spreadsheet, which served as the data collection form. The data collection form, including the extracted data, and the accompanying data dictionary are available as Supplementary Material S3 and S4.

The first reviewer manually performed the data extraction. All extracted items were subsequently checked by the second reviewer. Discrepancies or uncertainties were resolved through iterative reading and cross‐checking of the source documents. No translations were required. No data were extracted from figures using software tools. Information regarding the presence of behavioral and psychological assessments was extracted, but was not included in the analysis, as the focus of the review was the qualitative comparison of human and animal physiological parameters.

### 2.6 Data Items

#### 2.6.1 Outcomes

The outcome domain was defined as the presence of physiological comodulation between human and animal subjects, assessed during interaction. This was operationalized as a dynamic alignment of physiological signals between individuals during interaction, assessed with any comparative statistical evaluation between variations of human and animal physiological signals. Comodulation analysis outcomes were classified into three categories: significant, partial (i.e., limited to specific conditions or subsets of data), or absent, based on the authors' reporting of their statistical analyses. No formal assessment of the validity of comodulation analysis methods was performed; all methods were accepted accordingly to the eligibility criteria of this review.

When multiple comodulation analyses were reported within the same study, the primary outcome reported in the synthesis was defined according to a predefined hierarchy, based on the complexity of the investigated relationship between physiological variables: time‐series coupling analyses were prioritized, followed by regression and correlation analyses.

When multiple physiological measures analysis results were reported within a study, only the presence of statistically significant comodulation between human and animal signals for each and every parameter was classified as a “significant” outcome. The presence of statistically significant comodulation between human and animal signals for a subset of parameters was classified as a “partial” outcome.

#### 2.6.2 Other Variables

In addition to the primary outcome, the following data items were extracted from each included study: name; year; animal species involved; number of human and animal participants; nonhealthy (human)/nonwild‐type (animal) participants; interaction context; behavioral measures; measured physiological parameter(s); sampling; index used; analysis description; data analysis category; outcome. When information was missing or unclear, no assumptions were made and the corresponding data fields were left blank. All extracted variables, including missing data, are documented in the data collection form. Definitions and coding rules for each variable are provided in the accompanying data dictionary (see File S3 and File S4).

### 2.7 Risk of Bias, Effect Measures, Certainty, and Sensitivity Assessment

The risk of bias assessment was conducted using a categorization derived and adapted from the ROBINS‐I V2 tool (Risk Of Bias In Non‐randomized Studies ‐ of Interventions [14], Version 2 (https://www.riskofbias.info/welcome/robins‐i‐v2). Given the heterogeneity of the included studies and the specific context of human–animal physiological and behavioral research, the original structure of the tool was modified to better align with the methodological characteristics of the analyzed studies. Specifically, five evaluation domains were defined (Box 3).

Each domain was rated qualitatively (Low, Moderate, High), with a textual justification. The overall risk of bias was categorized according to a predefined rule: studies with all domains rated as Low were classified as “Low”; those with at least one Moderate rating as “Low to Moderate”; those with at least two Moderate ratings as “Moderate”; those with at least one High rating as “Moderate to High”; those with at least two High ratings as “High.” This adapted categorization enabled a context‐sensitive evaluation of risk of bias, preserving the conceptual structure of ROBINS‐I V2 while tailoring it to the specific needs of the review. Discrepancies were resolved through discussion. No automation tools were used in the assessment process. A unified assessment of the risk of bias for the included studies is provided in Section 3.2. The individual assessments for each study are presented in Table 1 and detailed motivations for each rating are provided in Table S5.

**TABLE 1:** Risk of bias assessment for included studies.

Study	Bias due to confounding	Bias in sample selection	Bias in physiological measurement	Bias in statistical analysis	Bias in outcome reporting	Overall risk of bias
Baldwin et al., 2021 [15]	Moderate	Moderate	Low	High	Low	Moderate to High
A. L. Baldwin et al., 2023 [16]	Moderate	Moderate	Low	High	Low	Moderate to High
Buttner et al., 2015 [17]	Moderate	Moderate	Low	Low	Low	Moderate
Byrne and Arnott, 2024 [18]	Moderate	Moderate	Low	Low	Low	Moderate
Callara et al., 2024 [19]	Moderate	Moderate	Low	Low	Low	Moderate
Friend et al., 2023 [20]	Moderate	Moderate	Low	Low	Low	Moderate
Gnanadesikan, King, et al., 2024 [21]	Moderate	Moderate	Low	Low	Low	Moderate
Grigg et al., 2022 [22]	Moderate	Moderate	Low	Moderate	Low	Moderate
Guidi et al., 2016 [23]	Moderate	High	Low	Low	Low	Moderate to High
Handlin et al., 2012 [24]	Moderate	High	Low	Moderate	Low	Moderate to High
Harvie et al., 2021 [25]	Moderate	Moderate	Low	Low	Low	Moderate
Hockenhull et al., 2015 [26]	Moderate	Moderate	Low	Low	Low	Moderate
Holder et al., 2024 [27]	Moderate	Moderate	Low	Low	Low	Moderate
Janczarek et al., 2013 [28]	Moderate	High	Low	Moderate	Low	Moderate to High
Jones and Josephs, 2006 [29]	Moderate	Moderate	Low	Low	Low	Moderate
Kang and Yun, 2016 [30]	Moderate	Moderate	Low	Moderate	Low	Moderate
Katayama et al., 2019 [31]	Moderate	Moderate	Low	Low	Low	Moderate
Koskela et al., 2024 [1]	Moderate	Moderate	Low	Low	Low	Moderate
Lanata et al., 2016 [32]	Moderate	High	Low	High	Low	High
Lanata et al., 2017 [33]	Moderate	High	Low	High	Low	High
McDuffee et al., 2024 [34]	Moderate	Moderate	Low	Low	Low	Moderate
Naber et al., 2025 [35]	Moderate	Moderate	Low	Low	Low	Moderate
Nagasawa et al., 2015 [36]	Moderate	Moderate	Low	Moderate	Moderate	Moderate
Nagasawa et al., 2023 [37]	Moderate	Moderate	Moderate	High	High	High
Nomoto et al., 2024 [38]	Moderate	Moderate	Low	Low	Moderate	Moderate
Rankins et al., 2025a [39]	Moderate	Moderate	Low	Low	Moderate	Moderate
Ren et al., 2024 [40]	Moderate	Moderate	Low	Low	Moderate	Moderate
Risvanli et al., 2025 [41]	Moderate	Moderate	Low	Low	Moderate	Moderate
Ryan et al., 2019 [42]	Moderate	Moderate	Low	Low	Moderate	Moderate
Schöberl et al., 2012 [43]	Moderate	Moderate	Low	Low	High	Moderate to High
Schöberl et al., 2017 [44]	Moderate	Moderate	Low	Low	Low	Moderate
Strzelec et al., 2013 [45]	Moderate	Moderate	Low	Moderate	Moderate	Moderate
Sundman et al., 2019 [46]	Moderate	Moderate	Low	Low	Low	Moderate
Wienhold et al., 2025 [47]	Moderate	Moderate	Low	Low	Low	Moderate
Wojtaś et al., 2020 [48]	Moderate	Moderate	Low	Moderate	Low	Moderate
Wojtaś et al., 2022 [49]	Moderate	Moderate	Low	Low	Low	Moderate
Yorke et al., 2013 [50]	High	High	Moderate	Moderate	Low	High

Overall risk of bias was derived by combining the five domain ratings as follows: “Low” if all domains were rated as Low; “Low to Moderate” when at least one domain was rated as Moderate; “Moderate” when at least two domains were rated as Moderate; “Moderate to High” when at least one domain was rated as High; “High” when at least two domains were rated as High. Detailed motivations for each rating are provided in the supplemental risk of bias Table S5.

No standardized effect measures (e.g., risk ratios, mean differences) were used, as the review focused on the categorical outcome (i.e., presence, partial presence, or absence of comodulation), rather than on numerical effect estimates. Accordingly, rather than quantifying a specific effect size, the performed narrative synthesis of experimental evidence maps the available literature and evaluates its methodological heterogeneity across studies. While statistical significance and effect directions were often reported in the primary studies as displayed in the individual studies results table (Table 3), no selection or filtering was applied based on these characteristics during data extraction or synthesis. No conversion or manipulation of the reported outcomes was performed prior to data extraction. Each and every entry reported explicitly the outcome of interest (presence, partial presence, or absence of physiological comodulation). No formal assessment of reporting bias was conducted. The adapted ROBINS‐I V2 tool included an explicit evaluation of the risk of bias in outcome reporting (see Box 3, Tables 1 and S5). No formal assessment of the certainty (or confidence) in the body of evidence was conducted. However, methodological robustness was addressed by explicitly acknowledging the risk of bias associated with included studies in each synthesis. No formal sensitivity analysis was conducted. Potential bias from missing results was mitigated through a comprehensive search strategy, including citation tracking and expert consultation, to maximize evidence completeness.

1 **BOX 3|The five domains used in risk of bias evaluation**

- Bias due to confounding: includes the presence of uncontrolled variables that may influence the association between intervention and outcome.
- Bias in sample selection: assesses the representativeness, randomization, and generalizability of the participants.
- Bias in physiological measurement: considers the validity, reliability, and standardization of instruments and data collection protocols.
- Bias in statistical analysis: evaluates the appropriateness of statistical tests, assumption checking, and use of corrections.
- Bias in outcome reporting: assesses transparency in reporting results, including nonsignificant findings, and consistency with stated objectives.

### 2.8 Synthesis Methods

#### 2.8.1 Eligibility for Synthesis

All included studies were considered eligible for synthesis, regardless of species, context of interaction, or physiological parameter measured. No subgrouping or exclusion was applied at this stage. Studies assessed as being at high risk of bias (see Tables 1, 4‐6 and S5) were included in the synthesis; however, their risk of bias was explicitly acknowledged and considered during the interpretation of the results. No sensitivity analyses were conducted, as the synthesis was descriptive and based on categorical outcomes without quantitative pooling. Patterns of heterogeneity were explored through structured categorization and descriptive synthesis, rather than through statistical modeling, deemed inappropriate due to the substantial heterogeneity in study designs, outcome measures, and analytical approaches. Nevertheless, in the presentation of synthesis results, particular attention was given to transparently displaying heterogeneity along the PICO‐derived dimensions (Box 1), using supplementary structured tables to facilitate visual comparison across studies. A narrative and interpretative discussion of these patterns and subgroup structures is provided in Section 4.2.

## 3 Results

### 3.1 Individual Studies Characteristics and Results

**FIGURE 2:**
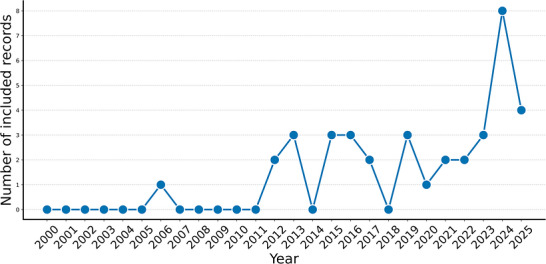
Publications per year for the included records —The line plot presents the number of publications per year for the records included in this review, highlighting a growth in the number of studies that assess the presence of physiological comodulation during human–animal interactions.

Characteristics of included studies are presented in Table 2, and the table notes display whether the existence of physiological comodulation is explicitly addressed in each study, along with references to the specific terminology employed to describe the phenomenon. A brief summary of each one is available as Supplementary Material S7. Individual study results are presented in Table 3, which provides a summary of the measured parameters, analysis methods, and outcomes for each included study. Overall, the included records indicate an increase over time in studies examining physiological comodulation during HAIs, as shown in Figure 2.

**TABLE 2:** Characteristics of individual studies table.

**Study**	**Animal species**	**Sample size** H: Human(s) A: Animal(s)	**Interaction context**	**Physiological parameter**	**Data analysis Method category**
Baldwin et al., 2023 [16]	Horse	24 H, 3 A	AAI	Cardiac activity	Time‐series coupling^a^
Baldwin et al., 2021 [15]	Horse	24 H, 1 A	AAT	Cardiac activity	Time‐series coupling^a^
Buttner et al., 2015 [17]	Dog	58 H, 58 A	Competitive sport	Cortisol (dogs) Testosterone (humans)	Regression^a^
Byrne and Arnott, 2024 [18]	Dog	28 H, 28 A	Companionship	Cardiac activity	Regression^c^
Callara et al., 2024 [19]	Horse	22 H, 20 A	AAI	Cardiac activity	Time‐series coupling^a^
Friend et al., 2023 [20]	Horse	18 H, 4 A	AAT	Cardiac activity Cortisol	Discrete‐time correlation analysis^b^
Gnanadesikan, et al., 2024 [21]	Dog	55 H, 55 A	Companionship	Oxytocin	Regression^c^
Grigg et al., 2022 [22]	Dog	40 H, 40 A	Companionship	Cardiac activity	Discrete‐time correlation analysis^b^
Guidi et al., 2016 [23]	Horse	14 H, 1 A	AAI	Cardiac activity	Time‐series coupling^b^
Handlin et al., 2012 [24]	Dog	10 H, 10 A	Companionship	Cortisol, Oxytocin	Discrete‐time correlation analysis^c^
Harvie et al., 2021 [25]	Dog	68 H, 68 A	Companionship	Cardiac activity Cortisol	Regression^b^
Hockenhull et al., 2015 [26]	Horse	17 H, 17 A	Noncompetitive sport	Cardiac activity	Discrete‐time correlation analysis^a^
Holder et al., 2024 [27]	Dog	8 H, 4 A	Companionship	Cardiac activity	Time‐series coupling^a^
Janczarek et al., 2013 [28]	Horse	2 H, 40 A	Noncompetitive sport	Cardiac activity	Discrete‐time correlation analysis^b^
Jones and Josephs, 2006 [29]	Dog	83 H, 83 A	Competitive sport	Cortisol (dogs) Testosterone (humans)	Regression^c^
Kang and Yun, 2016 [30]	Horse	23 H, 24 A	Competitive sport	Cortisol	Discrete‐time correlation analysis^c^
Katayama et al., 2019 [31]	Dog	34 H, 34 A	Companionship	Cardiac activity	Time‐series correlation^a^
Koskela et al., 2024 [1]	Dog	29 H, 29 A	Companionship	Cardiac activity	Regression^b^
Lanata et al., 2016 [32]	Horse	11 H, 1 A	AAI	Cardiac activity	Time‐series coupling^a^
Lanata et al., 2017 [33]	Horse	11 H, 1 A	AAI	Cardiac activity	Time‐series coupling^b^
McDuffee et al., 2024 [34]	Horse	16 H, 8 A	AAI	Cardiac activity Cortisol, Oxytocin	Time‐series correlation^a^
Naber et al., 2025 [35]	Horse	10 H, 4 A	AAT	Cardiac activity Cortisol	Discrete‐time correlation analysis^a^
Nagasawa et al., 2015 [36]	Dog, Wolf	30 H, 41 A	Companionship	Oxytocin	Discrete‐time correlation analysis^c^
Nagasawa et al., 2023 [37]	Dog	22 H, 22 A	Companionship	Cardiac activity	Time‐series correlation^a^
Nomoto et al., 2024 [38]	Dog	6 H, 6 A	Companionship	Heart rhythm	Regression^a^
Rankins et al., 2025a [39]	Horse	9 H, 12 A	AAI	Cardiac activity	Discrete‐time correlation analysis^b^
Ren et al., 2024 [40]	Dog	1 H, 19 A	Companionship	EEG	Time‐series coupling^a^
Risvanli et al., 2025 [41]	Horse	40 H, 40 A	Competitive sport	Hormones Biochemical Hematological^*^	Discrete‐time correlation analysis^c^
Ryan et al., 2019 [42]	Dog	29 H, 29 A	Companionship	Cortisol Cg A	Discrete‐time correlation analysis^a^
Schöberl et al., 2012 [43]	Dog	22 H, 22 A	Companionship	Cortisol	Discrete‐time correlation analysis^c^
Schöberl et al., 2017 [44]	Dog	132 H, 132 A	Companionship	Cortisol	Discrete‐time correlation analysis^c^
Strzelec et al., 2013 [45]	Horse	36 H, 36 A	Competitive sport	Cortisol	Discrete‐time correlation analysis^c^
Sundman et al., 2019 [46]	Dog	58 H, 58 A	Competitive sport	Cortisol	Regression^a^
Wienhold et al., 2025 [47]	Horse	45 H, 4 A	AAT	Cardiac activity	Time‐series coupling^a^
Wojtaś et al., 2020 [48]	Dog	41 H, 41 A	Working animals	Cortisol	Discrete‐time correlation analysis^c^
Wojtaś et al., 2022 [49]	Dog, Cat	25 H, 100 A	Companionship	Cortisol	Discrete‐time correlation analysis^c^
Yorke et al., 2013 [50]	Horse	4 H, 4 A	AAT	Cortisol	Cross‐correlation^c^

*Notes*: ^a^Comodulation (Synchronization) is regarded as supported by previous studies and adopted as the theoretical basis of the research, which aims to quantify its actual occurrence in the specific experimental context. ^b^Comodulation (Alternative variants, i.e., Association [22], Comodulation [1], Coupling [20, 23, 33], Correlation [25, 28, 39], Interplay [33], Similarity [33]) is regarded as supported by previous studies and adopted as the theoretical basis of the research, which aims to quantify its actual occurrence in the specific experimental context. ^c^The study does not assume a priori the existence of physiological comodulation between humans and animals and considers the experimental question regarding the actual presence of this phenomenon (although described as: Association [21], Correlation [24, 36, 41, 43, 45, 48, 49], Co‐variation [21], Cross‐correlation [50], Influence [30], Prediction or Influence of the variance in the parameters of one subject based on the variance in the parameters of the other [18, 29, 44], Synchronicity [50].) ^*^Hormones: (cortisol, ACTH, β‐endorphin, epinephrine, norepinephrine, T_3_, T_4_). Biochemical: Na, K, CREA, urea, TP, ALB, Mg, AP, CK, ALT, AST). Hematological: (WBC, RBC, HGB, HCT, MCV, MCH, MCHC, PLT, LYM, GRA). The full names of all abbreviations, as well as definitions for each characteristic, are listed in File S4.

The table presents main characteristics of each study. Abbreviations: AAT, animal‐assisted therapy; AAI, animal‐assisted intervention

**TABLE 3:** Individual studies results table.

**Name**	**Physiological parameters**	**Analysis description**	**Outcome**	**Statistical significance and/or effect size when available (NA: not applicable)**
Baldwin et al., 2023 [16]	Cardiac activity	Peak frequency analysis after FFT	Partial	NA
Baldwin et al., 2021 [15]	Cardiac activity	Fast Fourier transform (FFT) used to identify heart rate Variability frequency peaks. Coupling was defined as matching HRV frequency peaks (to 3–4 decimal places) between horse and human.	Partial	NA
Buttner et al., 2015 [17]	Cortisol testosterone	Pearson correlation, structural equation modeling (SEM) to test: human cortisol Variation influence on dog cortisol Variation; dog cortisol Variation influence on human cortisol Variation	Significant	In the first model, handlers' Δ CORT significantly predicted dogs' Δ CORT. Overall, the model fit the data well: $\chi^{2}$ (11) = 8.90, *p* ≤ 0.05, CFI = 1.00, RMSEA = 0.08, SRMR = 0.06, explaining 39% of variance in dogs' cortisol change. In the alternative model, dogs' Δ CORT significantly predicted handlers' Δ CORT; the model explained 37% of variance in handlers' cortisol change and had acceptable fit: $\chi^{2}$ (12) = 14.67, *p* ≤ 0.05, CFI = 0.95, RMSEA = 0.09, SRMR = 0.08. A $\chi^{2}$ difference test indicated that the first model fit better: $\chi^{2}$ (1) = 5.88, *p* ≤ 0.05.
Byrne and Arnott, 2024 [18]	Cardiac activity	Linear regression: human heart rate variation influence on dog heart rate variation.	Significant	Changes in owner mean HR significantly predicted changes in dog mean HR: *F*(1, 21) = 8.21, *p* = 0.009, accounting for 28.1% of variation in dog mean HR difference, with adjusted $R^{2}$ = 0.247, a large effect size according to Cohen.
Callara et al., 2024 [19]	Cardiac activity	Directed coherence (DC – directional physiological coupling in the frequency domain).	Significant	NA
Friend et al., 2023 [20]	Cardiac activity; cortisol;	Spearman correlation	Partial	A tendency toward a moderate negative correlation was found between changes in human and horse cortisol concentrations throughout treatment: *r* = −0.34, *p* = 0.10. However, a strong negative correlation was found over the second week: *r* = −0.90, *p* ≤ 0.01. No correlations between human and horse heart rates were found.
Gnanadesikan, et al., 2024 [21]	Oxytocin	Bayesian model: fit univariate models using dog's Area Under the Curve (AUCi) as the outcome variable and child's Area Under the Curve (AUCi) as a predictor variable	Partial	Child and dog salivary oxytocin had a moderate positive association in the unfamiliar‐dog condition: β_child_ AUCi = 0.19, CI: 0.01 ‐0.41. A weak negative association was estimated in the pet‐dog condition: β_child_ AUCi = ‐0.11, CI: ‐0.34 to 0.13. Minimal evidence was found for an association in urinary measures in the unfamiliar‐dog condition: β_child_ AUCi = 0.08, CI: ‐0.15 to 0.31. A moderate association was found in the pet‐dog condition: β_child_ AUCi = 0.29, CI: 0.00 ‐0.58.
Grigg et al., 2022 [22]	Cardiac activity	Pearson correlation	Partial	No significant correlations were found between human and canine cardiac activity parameters during session 1; during session 2, canine RMSSD was significantly correlated with human HF: (*r* = 0.432, *p* = 0.0001).
Guidi et al., 2016 [23]	Cardiac activity	Dynamic time warping	Significant*	NA
Handlin et al., 2012 [24]	Cortisol; oxytocin	Spearman correlation	Partial	A positive correlation was found between owners' oxytocin levels during the interaction experiment and dogs' oxytocin levels at the end of the experiment: *r* = 0.783, *p* ≤ 0.013. No significant correlations were found between owners' and dogs' cortisol levels.
Harvie et al., 2021 [25]	Cardiac activity; cortisol	Linear regression on heart rate difference between dogs and owners (calculated as the variation between the experimental time points).	Absent	No significant influence of owner HR on dog HR was found: Timepoint 1, control: *F*(1, 7) = 0.322, *p* = 0.588; experimental: *F*(1, 9) = 0.626, *p* = 0.449. Timepoint 2, control: *F*(1, 7) = 0.0897, *p* = 0.773; experimental: *F*(1, 9) = 0.0513, *p* = 0.826. Timepoint 3, control: *F*(1, 7) = 1.88, *p* = 0.212; experimental: *F*(1, 9) = 1.34, *p* = 0.276. Cortisol levels of dog‐owner dyads showed no significant regression in the experimental group, *F*(1, 31) = 1.96, *p* = 0.171, *R* ^2^ = 0.06, or control group, *F*(1, 25) = 1.84, *p* = 0.188, *R* ^2^ = 0.068.
Hockenhull et al., 2015 [26]	Cardiac activity	Spearman's rank order correlations of heart rate time‐ series	Partial	HR in four horses correlated significantly with HR in the familiar handler: Horse 3, *r* = ‐0.68, *n* = 16, *p* = 0.004; Horse 5, *r* = 0.55, *n* = 15, *p* = 0.035; Horse 7, *r* = 0.52, *n* = 15, *p* = 0.048; Horse 10, *r* = 0.63, *n* = 15, *p* = 0.011. HR in two horses correlated significantly with HR in the unfamiliar handler: Horse 7, *r* = 0.77, *n* = 15, *p* = 0.001; Horse 16, *r* = ‐0.56, *n* = 15, *p* = 0.029.
Holder et al., 2024 [27]	Cardiac activity	Pearson correlation; dynamic time‐warping methodology,	Absent	NA
Janczarek et al., 2013 [28]	Cardiac activity	Pearson correlation	Partial	Significant correlations within three training elements were recorded for associations between colt and trainer HR values, *p* ≤ 0.05 but not for mean HR; values are displayed in a table.
Jones and Josephs, 2006 [29]	Cortisol (dogs); testosterone (humans)	Linear regression	Partial	Handlers' change in testosterone (T) from pre‐ to post‐competition, controlling for pre‐competition T, significantly predicted change in dogs' cortisol from pre‐ to post‐competition: *t* = ‐2.349, *p* = 0.024, *R* ^2^ = 0.127. It accounted for less variance than handlers' pre‐competition T: *R* ^2^ = 0.127 vs. *R* ^2^ = 0.509. After a win, handlers' change in T was not a significant predictor of dogs' cortisol change: *t* = ‐1.713, *p* = 0.094, *R* ^2^ = 0.007.
Kang and Yun, 2016 [30]	Cortisol	Pearson correlation	Partial	Horses' T1 and riders' T2 salivary cortisol concentrations were positively related: *r* = 0.522, *p* < 0.001. T2 and T3 were also positively correlated: *r* = 0.435, *p* < 0.01.
Katayama et al., 2019 [31]	Cardiac activity (HRV)	dog–owner HRV time‐series correlation coefficients	Significant*	NA
Koskela et al., 2024 [1]	Cardiac activity	Spearman correlation multivariate linear regression	Significant	Multivariate linear regression analysis of owner overall RMSSD showed *R* ^2^ = 0.212 and Cohen's *f* ^2^ = 0.27. Higher RMSSD in dogs was associated with higher RMSSD in owners: β = 0.051, 95% CI: 0.008 –0.095, *p* = 0.024.
Lanata et al., 2016 [32]	Cardiac activity	Coherence (magnitude squared coherence – MSC, mean phase coherence – MPC); dynamic time warping	Significant*	NA
Lanata et al., 2017 [33]	Cardiac activity	Nonlinear coupling and similarity measures: Cross‐Correntropy; Cross information Potential; Correntropy coefficient;	Significant*	NA
McDuffee et al., 2024 [34]	Cardiac activity; cortisol; oxytocin	Bland–Altman plots of heart rate Variability (HRV) indices Sympathetic nervous system (SNS) to parasympathetic nervous system (PNS) ratio (SNS/PNS ratio)	Significant	NA
Naber et al., 2025 [35]	Cardiac activity; cortisol	Spearman correlation	Partial	Horse‐client dyads: No significant relation was found among HRV, cortisol, and HR. However, HR was significantly correlated with familiar horses: *r* = 0.38, *p* = 0.007; no significant correlation was found with unfamiliar horses: *r* = 0.06, *p* = 0.659. Horse‐therapist dyads: No significant correlation was found for HRV parameters or cortisol. However, HR was significantly correlated: *r* = 0.53, *p* < 0.001. The correlation was stronger with familiar horses, *r* = 0.61, *p* < 0.001, than with unfamiliar horses, *r* = 0.55, *p* < 0.001.
Nagasawa et al., 2015 [36]	Oxytocin	Spearman's rank correlation coefficient (two‐tailed)	Significant	The oxytocin change ratio in owners correlated significantly with that of dogs: *r* = 0.847 *p* ≤ 0.01.
Nagasawa et al., 2023 [37]	Cardiac activity	Correlation coefficients (not specified)	Significant*	NA; no details were provided beyond the correlation coefficient of mean RRI being higher in OW episodes: *t* = 4.098, *p* < 0.001.
Nomoto et al., 2024 [38]	Breath rhythm	Generalized linear mixed model (GLMM) to test the effect of human breathing on dog breathing. Fixed factors: human delta‐breathing‐interval (delta‐BI), condition. Random effect: dog–owner pair.	Significant	The effect of human breathing on dog breathing was significant: fixed‐effect estimate = 0.57, *t* = 3.94, *df* = 18.6, *p* < 0.01; *p* values were calculated with Satterthwaite *df*.
Rankins et al., 2025a [39]	Cardiac activity	ANOVA for group comparisons, Pearson between human/horse indices + Bonferroni	Absent	No significant correlations, *p* geq 0.0082, were observed between AH horse and veteran heart rates or HRV measures (LF/HF, RMSSD) after Bonferroni correction
Ren et al., 2024 [40]	EEG	Interbrain correlation (Pearson correlation of EEG power). Generalized partial directed coherence (GPDC) – directionality of coupling. Theta/beta ratio (TBR) – attention index	Significant	Interbrain correlations across pairs of human‐dog dyads from different sessions were significantly lower than those from the same interacting sessions: *p* < 0.05, Mann‐Whitney *U* test.
Risvanli et al., 2025 [41]	Hormones; biochemical; hematological	Spearman correlation for horse‐rider parameters pooled, separate analysis for pre and post competition and winners/losers.	Partial	Positive correlations for pre‐competition winner dyads are shown as examples: RDW‐CV and HGB, ρ = 0.527, *p* < 0.05; LYM% and MID, ρ = 0.654, *p* < 0.01; LYM% and MID%, ρ = 0.532, *p* < 0.05; LYM% and MCHC, ρ = 0.654, *p* < 0.01; RBC and RDW‐SD, ρ = 0.525, *p* < 0.05; MPV and PDW%, ρ = 0.616, *p* < 0.05.
Ryan et al., 2019 [42]	Cortisol; Cg A	Pearson correlation	Partial	Final CORT levels for dogs were positively correlated with their owners' final CORT levels: *r*_p_ = 0.62, *p* = 0.005, *N* = 17. Dog initial CgA was marginally correlated with owner initial CORT: *r*_p_ = 0.58, *p* = 0.018, *N* = 14. Owner CgA levels did not correlate with either dog CORT or dog CgA.
Schöberl et al., 2012 [43]	Cortisol	Spearman correlation	Absent	No correlations between owner and dog morning salivary cortisol values were found over control days: Spearman's correlation, *n* = 22, *r*_s_ = 0.18, *p* = 0.43.
Schöberl et al., 2017 [44]	Cortisol	Spearman correlation of individual coefficients of variance of cortisol (iCVs)	Absent	The owner's individual coefficient of variation of cortisol (iCV) did not correlate with the dog's iCV: Spearman's correlation, *n* = 105, *r*_s_ = 0.043, *p* = 0.663.
Strzelec et al., 2013 [45]	Cortisol	Pearson correlation	Partial	Correlation coefficients between riders' and horses' salivary cortisol concentrations in subsequent competition phases were: after dressage, *r* = 0.347, *p* = 0.052; after cross‐country, *r* = 0.195, *p* = 0.26; after show jumping, *r* = 0.161, *p* = 0.35.
Sundman et al., 2019 [46]	Cortisol	Generalized linear model	Significant	Human hair cortisol concentration (HCC) from both summer and winter samplings correlated strongly with dog HCC: summer, *N* = 57, $\chi^{2}$ = 23.697, *p* < 0.001, β = 0.235; winter, *N* = 55, $\chi^{2}$ = 13.796, *p* < 0.001, β = 0.027.
Wienhold et al., 2025 [47]	Cardiac activity	Cross‐wavelet power analyses	Significant	Final model predicting horse‐participant HF‐HRV synchronization: marginal *R*_2_ = 0.38 and conditional *R*_2_ = 0.52; fixed effects explained 38% of variance. The full model, including random effects, accounted for 52%. Adding HF‐HRV synchronization between therapy horse and riding therapist to the reference model significantly improved model fit: $\chi^{2}$ = 757.87, *p* < 0.01. The association appeared positive: estimate = 0.36, CI: 0.33 –0.38, *p* < 0.01.
Wojtaś et al., 2020 [48]	Cortisol	Spearman correlation	Partial	A significant positive correlation was found between cortisol levels in dogs and handlers before the examination: *r*_s_ = 0.34, *p* = 0.032; but not after the examination: *r*_s_ = 0.18, *p* = 0.406; or for the increase in cortisol levels: *r*_s_ = 0.13, *p* = 0.406.
Wojtaś et al., 2022 [49]	Cortisol	Spearman correlation	Partial	No significant relationship was found between hair cortisol levels of owners and pets: *r* = ‐0.033, *t*(*N* − 2) = ‐0.325, *p* = 0.746. A significant positive correlation was found between human and cat cortisol levels when cats were never kissed: *r* = 0.686, *p* = 0.0096. A significant positive correlation was observed for dogs in which the owner performed grooming treatments once a week: *r* = 0.836, *p* = 0.005.
Yorke et al., 2013 [50]	Cortisol	Zero‐lag cross‐correlation for each child–horse pair	Significant	The weighted mean cross‐correlation, controlling for autocorrelation, was 0.23, *Z* = 3.03, with an approximate 95% CI of 0.08–0.38.

*Note*: The table displays results for each retrieved study, summarizing measured parameters, analysis methods, and outcomes. A detailed narrative description of each study is available as a Supplementary Material (see File S7).

* Comodulation was significantly and consistently different when quantitatively compared between different experimental phases of scenarios, but its presence was assumed a priori.

### 3.2 Risk of Bias in Contributing Studies

The performed narrative synthesis is based on the complete set of included studies. Accordingly, a unified assessment of risk of bias is provided. Additionally, risk of bias judgments for each domain and study are presented in Table 1 and detailed justifications for each judgment are provided as a Supplementary Material Table S5.

The most frequent rating for the “Bias due to confounding” domain was “Moderate.” This was primarily attributed to the lack of control over individual‐level variables such as prior experience with animals, emotional state, and physical fitness. In several studies, the use of a single animal in interaction protocols further limited generalizability and introduced potential confounding effects specific to that individual (see Table S5).

The “Bias in sample selection” domain was also most frequently rated as “Moderate.” Many studies relied on self‐selected participants from homogeneous populations, often with limited demographic diversity. Small sample sizes and the use of convenience sampling were common, and in some cases, the inclusion of single‐individual animal models further restricted the external validity of the findings.

In contrast, physiological measurement was generally robust across studies, with most receiving a “Low” risk of bias rating. Validated devices and standardized protocols were widely used for the collection of cardiac activity, cortisol, and oxytocin. Signal processing and artifact removal procedures were typically well‐documented and appropriate.

Statistical analysis was similarly strong, with most studies employing suitable models and tests, and explicitly addressing their assumptions.

Outcome reporting was generally clear, with most studies presenting both significant and nonsignificant results and noting limitations. However, the lack of preregistration raises concerns about selective emphasis on positives. Overall, while internal validity was supported by sound methods, external validity and confounding control remain major limitations.

Many studies relied on small and homogeneous samples. Moreover, the animal species involved were almost exclusively domesticated, limiting the applicability of findings to broader HAI contexts.

Interaction protocols varied considerably in duration, setting, and type of engagement, and were often tailored to specific experimental aims rather than standardized across studies. Similarly, control conditions intended to represent “Noninteraction” baselines were highly heterogeneous and context‐dependent, reflecting the constraints of individual designs. These variations complicate cross‐study comparisons and may introduce uncontrolled variability in physiological responses.

These limitations, while relevant, do not invalidate the present synthesis, which aims to explore the presence and nature of experimental evidence for physiological comodulation in HAIs.

### 3.3 Results of Syntheses

#### 3.3.1 Contexts of Interaction Across Studies

Twelve studies (32.4%^1^) were conducted in the context of AAI or AAT, each of them enrolled horses (AAT refers to clinically framed interventions with explicit therapeutic aims, while AAI is used as the broader umbrella for structured, goal‐oriented programs involving animals that are not explicitly labeled as “therapy”). Seven of these studies reported significant comodulation outcomes [19, 23, 32, 33, 34, 47, 50]. Four reported partial comodulation [15, 16, 20, 35]. One reported absent comodulation [39].

Sixteen studies (43.2%) were conducted in the context of companionship, all of them enrolled dogs. Six of these studies reported significant comodulation [1, 18, 31, 37, 38, 40]. Four of them reported partial comodulation [21, 22, 24, 42]. Four of them reported absent comodulation [25, 27, 43, 44]. One study additionally enrolled wolves and reported significant comodulation [36]. One study additionally enrolled cats and reported partial comodulation [49]. Six studies (16.2%) were conducted in the context of competitive sport (sport activities involving formal competition). Three of them enrolled dogs: two reported significant comodulation [17, 46], while one reported partial comodulation [29]. Three of them enrolled horses and reported partial comodulation [30, 41, 45]. Two studies (5.4%) were conducted in the context of noncompetitive sport (sport‐related activities conducted without formal competition, e.g., training), both of them enrolled horses and reported partial comodulation [26, 28]. One study (2.7%) was conducted in the context of working animals training, enrolling search and rescue dogs, and reported partial comodulation [48]. Results of the synthesis for the “interaction context” dimension are additionally presented in Table 4.

**TABLE 4:** Tabular synthesis based on interaction context.

**Interaction context**	**Animal specie(s)**	**Nonhealthy humans/nonwild‐type animals**	**Comodulation outcome**	**Overall risk of bias**
AAT/AAI (*n* = 12)	Horse (*n* = 12)	Two studies enrolled veterans with PTSD One study enrolled children with PTSD One study enrolled people undergoing treatment for substance use disorder (SUD) One study enrolled women with intellectual disabilities One study enrolled people with age‐related conditions One study enrolled people with various mental health conditions	Significant: seven studies. Partial: four studies. Absent: one study.	High: ● ● ● Moderate to High: ● ● ● Moderate: ● ● ● ● ● ●
Companionship (*n* = 16)	Dog (*n* = 16)^a^	One study enrolled children from an NIH clinical trial (as a subset) One study enrolled Shank‐3 mutated dogs as autism spectrum disorder (ASD) models	Significant: seven studies. Partial: five studies. Absent: four studies.	High: ● Moderate to High: ● ● Moderate: ● ● ● ● ● ● ● ● ● ● ● ● ●
Competitive sport (*n* = 6)	Horse (*n* = 3)	—	Partial: three studies.	Moderate: ● ● ●
	Dog (*n* = 3)	—	Significant: two studies. Partial: one study.	Moderate: ● ● ●
Noncompetitive sport (*n* = 2)	Horse (*n* = 2)	—	Partial: two studies.	Moderate to High: ● Moderate: ●
Working animals (*n* = 1)	Dog (*n* = 1)	—	Partial: one study.	Moderate: ●

The table illustrates the animal species enrolled, the presence or absence of nonhealthy human subjects/nonwild‐type (wt) animal subjects, the comodulation outcomes, and the overall risk of bias for each context of interaction. Risk of bias legend: High = Red bullets; Moderate to High = Orange bullets; Moderate = Yellow bullets. ^a^One study additionally included cats; another study additionally included wolves.

#### 3.3.2 Physiological Parameters Across Studies

Sixteen studies (43.2%) compared human and animal cardiac activity signals. Six of them enrolled dogs: four studies reported significant comodulation [1, 18, 31, 37], one reported partial comodulation [22], one reported absent comodulation [27]. Ten of them enrolled horses: five studies reported significant comodulation [19, 23, 32, 33, 47], four studies reported partial comodulation [15, 16, 26, 28], one study reported absent comodulation [39]. Within this group, two studies limited their analysis to heart rate (bpm) characterization, rather than include HRV indices as well [26, 28].

Eight studies (21.6%) compared human and animal cortisol levels. Five of them enrolled dogs: one reported significant comodulation [46], one reported partial comodulation [48], two reported absent comodulation [43, 44]. The fifth, which additionally enrolled cats, reported partial comodulation [49]. Three of them enrolled horses: one reported significant comodulation [50], two reported partial comodulation [30, 45].

Two studies (5.4%) compared human and animal oxytocin levels, one enrolled dogs and reported partial comodulation [21], and the other additionally enrolled wolves and reported significant comodulation [36].

Three studies (8.1%) sampled both heart rate and cortisol levels: one enrolled dogs and reported absent comodulation [25]; two enrolled horses and reported partial comodulation [20, 35]. One study (2.7%) sampled both cortisol and oxytocin levels in dogs, reporting partial comodulation [24]. One study (2.7%) sampled heart rate, cortisol level, and oxytocin level in horses, reporting significant comodulation [34]. Two studies (5.4%) measured salivary testosterone in humans and correlated it to cortisol levels in dogs, one reporting significant comodulation [17] and one reporting partial comodulation [29]. One study (2.7%) measured, along with cortisol levels, salivary CgA levels in both humans and dogs, reporting partial comodulation [42].

One study (2.7%) measured, in both humans and horses for each parameter, the plasma concentration of many hormones (cortisol, ACTH, β‐endorphin, epinephrine, norepinephrine, T3, T4), various other biochemical (Na,K, CREA, urea, TP, ALB, Mg, AP, CK, ALT, AST) and hematological (WBC, RBC, HGB, HCT, MCV, MCH, MCHC, PLT, LYM, GRA) parameters (expanded forms of all abbreviations are available in File S4). This study reported partial comodulation [41]. One study (2.7%) measured breath rhythm in dogs and reported significant comodulation [38]. One study (2.7%) measured brain connectivity (EEG) in dogs and reported significant comodulation [40].

Results of the narrative synthesis for the “physiological parameter” dimension are additionally presented in Table 5. Finally, Table S6 presents the different sampling methods for the molecular physiological parameters across studies.

**TABLE 5:** Tabular synthesis based on physiological parameter.

**Physiological parameters**	**Animal specie(s)**	**Interaction context**	**Comodulation outcome**	**Overall risk of bias**
**Cardiac activity**				
Heart rate (*n* = 2) and heart rate variability (*n* = 14)	Horse (*n* = 10)	AAI/AAT: eight studies. Noncompetitive sport: two studies.	Significant: five studies. Partial: four studies. Absent: one study.	High: ● ● Moderate to High: ● ● ● ● Moderate: ● ● ● ●
	Dog (*n* = 6)	Companionship: six studies.	Significant: four studies. Partial: one study. Absent: one study.	High: ● Moderate: ● ● ● ● ●
**Hormones**				
Cortisol (*n* = 8)	Horse (*n* = 3)	AAI/AAT: one study. Competitive sport: two studies.	Significant: one study. Partial: two studies.	High: ● Moderate: ● ●
	Dog (*n* = 5)^a^	Companionship: three studies. Competitive sport: one study. Working animals: one study.	Significant: one study. Partial: two studies. Absent: two studies.	Moderate to High: ● Moderate: ● ● ● ●
Oxytocin (*n* = 2)	Dog (*n* = 2)^b^	Companionship: two studies.	Significant: one study. Partial: one study.	Moderate: ● ●
**Multiparameter analysis**				
Cardiac activity and cortisol (*n* = 3)	Horse (*n* = 2)	AAT/AAI: two studies.	Partial: two studies.	Moderate: ● ●
	Dog (*n* = 1)	Companionship: one study.	Absent: one study.	Moderate: ●
Cortisol and oxytocin (*n* = 1)	Dog (*n* = 1)	Companionship: one study.	Partial: one study.	Moderate to High: ●
Cardiac activity, cortisol, and oxytocin (*n* = 1)	Horse (*n* = 1)	AAT/AAI: one study.	Significant: one study.	Moderate: ●
**Other physiological parameters**				
Testosterone (*humans*) and cortisol (*dogs*) (*n* = 2)	Dog (*n* = 2)	Competitive sport: two studies.	Significant: one study. Partial: one study.	Moderate: ● ●
Cortisol and chromogranin A (*n* = 1)	Dog (*n* = 1)	Companionship: one study.	Partial: one study.	Moderate: ●
Hormonal, biochemical, and hematological parameters (*n* = 1)	Horse (*n* = 1)	Competitive sport: one study.	Partial: one study.	Moderate: ●
Breath rhythm (*n* = 1)	Dog (*n* = 1)	Companionship: one study.	Significant: one study.	Moderate: ●
Brain connectivity (*n* = 1)	Dog (*n* = 1)	Companionship: one study.	Significant: one study.	Moderate: ●

The table illustrates the animal species enrolled, the interaction context, the comodulation outcomes, and the overall risk of bias for physiological parameters classes (see Box 2). Risk of bias legend: Red bullets = High risk; Orange bullets = Moderate to High risk; Yellow bullets = Moderate risk. ^a^ One study also included cats. ^b^ One study also included wolves.

#### 3.3.3 Data Analysis Methods Across Studies

Nine studies (24.3%) performed time‐series coupling analysis. Six of them reported significant comodulation [19, 23, 32, 33, 40, 47]. Two reported partial comodulation [15, 16]. One work reported absence of comodulation [27].

Eight studies (21.6%) performed regression analyses. In particular, one study employed structural equation modeling and reported significant comodulation [17].

Three studies employed general linear mixed models; one reported significant comodulation [38], one reported partial comodulation [21], one reported absence of comodulation [25].

One study employed generalized linear models and reported significant comodulation [46].

Three studies employed (univariate or multivariate) linear regressions; two of them reported significant comodulation [1, 18] while one reported partial comodulation [29].

Twenty studies (54%) performed correlation analyses. In particular, one study (2.7%) performed a cross‐correlation analysis and reported significant comodulation [50].

Three studies (8.1%) performed time‐series correlation analyses and reported significant comodulation [31, 34, 37].

Sixteen studies (43.2%) performed discrete‐time correlation analyses. One of them reported significant comodulation outcomes [36]. Twelve of them reported partial comodulation [20, 22, 24, 26, 28, 30, 35, 41, 42, 45, 48, 49]. Three of them reported absence of comodulation [39, 43, 44]. Notably, within three of these studies, correlation was performed over a single time point with the assumption of sampling time‐accumulated effects between humans and animals with a history of interaction (e.g., owner–pet and horse–rider dyads), then performing significance and/or effect analyses on populations of dyads [41, 46, 48].

Table 6 summarizes results of the narrative synthesis for the “data analysis method” dimension, providing a structured overview of the comodulation outcomes and corresponding risk of bias assessments across all included studies, grouped by analytical approach.

**TABLE 6:** Tabular synthesis based on data analysis methods.

**Data analysis Method category**	**Data analysis Method subcategory**	**Comodulation outcome**	**Overall risk of bias**
Time‐series coupling analysis (*n* = 9)	—	Significant (*n* = 6)	High: ● ● Moderate to High: ● Moderate: ● ● ●
		Partial (*n* = 2)	Moderate to High: ● ●
		Absent (*n* = 1)	Moderate: ●
Regression analysis (*n* = 8)	Structural equation modeling (SEM) (*n* = 1)	Significant (*n* = 1)	Moderate: ●
	Generalized linear mixed models (GLMMs) (*n* = 3)	Significant (*n* = 1)	Moderate: ●
		Partial (*n* = 1)	Moderate: ●
		Absent (*n* = 1)	Moderate: ●
	Generalized linear models (GLMs) (*n* = 1)	Significant (*n* = 1)	Moderate: ●
	Linear regressions (*n* = 3)	Significant (*n* = 2)	Moderate: ● ●
		Partial (*n* = 1)	Moderate: ●
Correlation analysis (*n* = 20)	Cross‐correlation (*n* = 1)	Significant (*n* = 1)	High: ●
	Time‐series correlation (*n* = 3)	Significant (*n* = 3)	High: ● Moderate: ● ●
	Discrete‐time correlation (*n* = 16)	Significant (*n* = 1)	Moderate: ●
		Partial (*n* = 12)	Moderate to High: ● ● Moderate: ● ● ● ● ● ● ● ● ● ●
		Absent (*n* = 3)	Moderate to High: ● Moderate: ● ●

The table illustrates comodulation outcomes for each data analysis methods category and subcategory. Risk of bias legend: High = Red bullets; Moderate to High = Orange bullets; Moderate = Yellow bullets.

To thoroughly represent the methodological heterogeneity observed, particularly in terms of experimental design and statistical modeling, a brief narrative description of each study is presented in File S7. These narrative descriptions follow the same order of presentation as Table 6. In addition, studies are listed chronologically by year of publication and sorted by decreasing overall risk of bias to facilitate interpretability and comparison.

## 4 Discussion

### 4.1 General Interpretation of the Results

The narrative synthesis of the categorical comodulation outcomes for the included studies reveals a growing number of studies (Figure 2) investigating physiological comodulation between humans and animals during interaction. Across diverse contexts—ranging from AAT and companionship to sport and working environments—most studies report statistically significant associations between human and animal physiological signals, particularly in cardiac activity and hormonal measures (see Tables 3–5).

This is aligned with theoretical models of interspecies emotional attunement and mutual regulation, and echo similar patterns observed in human–human dyads, such as parent–child dyads or romantic partners [2, 3, 4, 5, 6]. However, the evidence remains fragmented. A substantial share of studies reported significant comodulation (16 out of 37), yet an equal number found only partial evidence (16 out of 37) and a smaller subset reported absence of comodulation (5 out of 37), particularly when hormonal parameters were involved or the interaction settings were less structured (see Tables 4 and 5).

This variability suggests that comodulation may be context‐dependent, influenced by factors such as the type of interaction, the species involved, and the physiological parameter measured. For instance, time‐series coupling methods applied to cardiac activity data yielded relatively more consistent evidence of comodulation than discrete‐time correlation analyses of hormonal signals (see Tables 5 and 6).

### 4.2 Limitations of the Evidence

Nevertheless, it should be noted that these findings emerge from a highly heterogeneous set of studies which differ substantially in terms of interaction context, animal species, physiological parameters measured, and analytical methods employed (see Table 2). This diversity calls for caution in interpretation: the presence of comodulation cannot yet be considered a generalizable phenomenon across HAIs. In particular, given the lack of consistent evidence for the existence of comodulation as a general effect, it was neither feasible nor meaningful to quantitatively analyze the distribution of outcomes across synthesis categories in search of trends.

Notably, the studies that reported the most consistent evidence of comodulation—particularly those employing time‐series coupling techniques—were also among those with the highest overall risk of bias (see Tables 1, 6, and S5), especially in the domains of confounding (e.g., uncontrolled emotional or experiential variables) and sample selection (e.g., small or homogeneous samples). Despite their analytical sophistication, these studies often lacked methodological safeguards that would support generalizability.

More broadly, the methodological robustness and risk of bias varied considerably across the included studies (see Tables 1 and S5). As detailed in Section 3.2, although physiological measurement and statistical analysis were generally sound, the overall methodological robustness varied, with most studies rated as “Moderate” in the overall risk of bias, and a substantial number rated as “Moderate to High.”

However, the synthesis suggests several ways in which researchers should approach this evidence prudently, by critically evaluating the methodological assumptions, contextual constraints, and analytical frameworks that shape each study's conclusions. As an example, the terminology used to describe interspecies physiological alignment is often inconsistent. The term synchrony is frequently employed in the retrieved papers, yet its operationalization varies significantly as it ranges from strict time‐resolved coupling based on densely sampled time‐series to broader statistical associations derived from aggregated or discretely sampled data (see Table 2). This variability complicates cross‐study comparisons and may conceal the underlying mechanisms of interaction. As proposed by Koskela et al. [1], the term comodulation, adopted in this review, may offer a more accurate and inclusive descriptor, capturing mutual physiological influence over extended temporal windows while retaining temporal overlap between interacting partners, and not implying moment‐to‐moment alignment or the use of specific signal processing techniques typically associated with the concept of synchrony.

Moreover, as reflected qualitatively in the synthesis results (see Table 3), the specific physiological parameter measured, as well as its sampling modality, strongly influences the feasibility of different comodulation analysis protocols. Continuous signals such as cardiac activity or EEG are well‐suited for time‐series coupling techniques, enabling fine‐grained analysis of temporal dynamics. In contrast, discrete measures like hormonal (e.g., cortisol, oxytocin) concentrations pose challenges due to their considerably lower temporal resolution. Even within hormonal measures, the biological matrix used (saliva, plasma, urine, hair—see Table S6) affects both the temporal window captured and the biological interpretation of the signal. For example, hair cortisol reflects long‐term accumulation, while salivary cortisol captures acute stress responses. In general, the onset and temporal dynamics of the physiological signal vary considerably depending on the sampling modality [51] introducing important differences in how comodulation can be biologically interpreted and generalized across studies.

These discrepancies must be taken into account not only when selecting analytical approaches, but especially when attempting to generalize the presence or absence of comodulation across HAIs and provide a corresponding biological interpretation. Without accounting for the temporal and biological specificity of each parameter, conclusions about the existence of comodulation risk being context‐bound and nontransferable.

Additionally, the synthesis results (see Tables 2 and 5) qualitatively suggest that the sampling modality may be shaped by the interaction context. For example, in therapeutic settings such as AAT or AAI, invasive procedures like blood draws may induce stress and compromise the ecological validity of the interaction [52]. This can alter the physiological signal itself, introducing stress responses unrelated to the interaction, and thereby compromise the validity of comodulation analysis, which may end up capturing the effects of the sampling procedure rather than interaction dynamics.

Conversely, in sport contexts, where blood sampling is already part of routine practice [53], plasma samples can be obtained without further altering the interaction context. Notably, the establishment of robust connections between HAI research and competitive sport environments has the potential to facilitate the creation of scientific datasets of relevance.

Furthermore, the interaction context influences not only the sampling strategy but also the type of subjects involved (see Table 4). In AAI, human participants are often nonhealthy individuals (e.g., patients with post traumatic stress disorder, physical and/or intellectual disabilities, or age‐related conditions), whereas sport studies typically involve healthy, trained individuals.

Similarly, the animal species enrolled are predominantly domesticated, primarily dogs and horses. Their domestication has made them central to therapeutic, recreational, and working contexts, which in turn facilitates funding and data availability. Additionally, domesticated animals do offer practical advantages for data collection, particularly in terms of compliance with wearable devices and tolerance to experimental procedures [52]. Nonetheless, the ecological generalizability of findings remains limited, and future research should aim to include wild and laboratory animals to broaden the scope of inference.

Finally, to advance the field, it is essential to design protocols that explicitly test whether significant comodulation occurs, rather than assuming its presence a priori. In many studies, the main research question is framed around highly specific scenarios, without addressing a broader and more foundational issue: whether significant comodulation between humans and animals is occurring at all (see Table 2). A more rigorous approach would involve framing the presence of comodulation as a primary research question, supported by appropriate control conditions and statistical testing.

Moreover, the concept of comodulation should be systematically contextualized with respect to the type of interaction, the experimental setting, the physiological parameters measured, the species involved, and the analytical methods used. This is particularly necessary due to the fact that the development of this field of study seems to be characterized by the adoption of increasingly complex physiological parameters combinations and data analysis procedures (see Table 2).

While this framework, illustrated by the present synthesis, offers one possible way to structure the evidence, it is not intended as exhaustive or prescriptive. Its further development may nonetheless facilitate future generalizations and meta‐analyses.

### 4.3 Limitations of the Review Processes

The first key limitation of this review lies in the initial search strategy, which relied on the compound keyword “human–animal interaction.” This choice was influenced by an implicit availability bias: the expectation that most relevant studies would be situated within the domain of AAT or AAI, where the term and its acronym (HAI) are commonly used [12]. However, this assumption may have inadvertently reduced the sensitivity of the search, potentially overlooking studies from other important contexts such as sport and companionship‐related ones. The subsequent broadening of the search strategy to include multiple Boolean keyword combinations targeting specific physiological parameters (see File S1), motivated by the need to enhance sensitivity, may have incidentally contributed to balancing the contextual limitations introduced by the initial keyword choice. Additionally, the use of the Consensus App and of citation tracking helped identify studies not indexed under standard HAI terminology, thereby enhancing the comprehensiveness of the evidence base.

Furthermore, as was discussed in the preceding sections (see Methods and Results), it is imperative to acknowledge many additional limitations: the review does not include a quantitative meta‐analysis; the outcomes were categorized qualitatively; no pooled effect estimates were calculated; statistical assessments of heterogeneity, as well as sensitivity analyses, and formal evaluations of reporting bias, which are typically employed in meta‐analytic frameworks, have not been performed.

Additionally, the adopted synthesis dimensions, despite being specifically selected to represent the heterogeneity observed across the PICO framework elements (Box 1), were not prespecified.

Finally, the risk of bias was assessed using a customized framework adapted from ROBINS‐I V2, tailored to the specific methodological features of HAI studies. While this approach aimed to preserve conceptual rigor, it does not follow a standardized or validated protocol.

### 4.4 Implications of the Results

In conclusion, this review presents preliminary evidence of physiological comodulation between humans and animals, without determining whether the phenomenon depends on specific combinations of interaction context, species, physiological parameter, and analytical method. Both interpretations remain plausible: comodulation may be a general feature of interspecies interaction, or it may emerge only under certain biological and methodological conditions.

While the synthesis supports the plausibility of physiological comodulation, its application in real‐world settings should invite caution. In therapeutic contexts, for example, it is premature to use comodulation as a parameter for session tuning or outcome evaluation. Similarly, in companionship studies, interpreting physiological alignment as a proxy for bonding still lacks definitive empirical grounding. Until the phenomenon is better characterized, it is advisable for practical implementations to avoid assuming a priori its occurrence, as well as treating it as a universally established mechanism.

Future studies, rather than assuming generalizability, should aim to explicitly test the presence of comodulation as a primary outcome, using appropriate control conditions and analytical methods. Expanding the scope to include nondomesticated species, nontherapeutic contexts, and datasets structured from sport environments may help clarify the conditions under which comodulation occurs.

At the policy level, the current evidence base remains insufficient to support standardized frameworks for physiological monitoring in HAI. Further conceptual and methodological consolidation is needed before translating these findings into practice or regulation.

### Author Contributions

Ginevra Bargigli: Conceptualization; formal analysis; investigation (first reviewer); visualization; writing – original draft preparation; writing – review and editing. Lorenzo Frassineti: Conceptualization; formal analysis; investigation (second reviewer); validation; writing – review and editing. Paolo Baragli: Conceptualization; funding acquisition; validation; writing – review and editing. Chiara Scopa: Conceptualization; validation; writing – review and editing. Aglaia Vignoli: Funding acquisition; validation; writing – review and editing. Antonio Lanatà: Conceptualization; funding acquisition; validation; writing – review and editing.

### Funding

We acknowledge financial support under the National Recovery and Resilience Plan (NRRP), Mission 4, Component 2, Investment 1.1, Call for tender No. 104 published on 02/02/2022 by the Italian Ministry of University and Research (MUR), funded by the European Union (NextGenerationEU). Project Title: “One welfare, one emotion: a look Inside the interaction. Bioengineering solution for the human‐horse emotional transfer in neuro‐psychological, and social perspectives. (OneFeeL)”; CUP: B53C24007430006; Grant Assignment Decree No. 104 adopted on 02/02/2022 by the Italian Ministry of University and Research (MUR).

### Conflicts of Interest

No conflicts of interest are reported. There are no personal, professional, or financial relationships that could be perceived as influencing the conduct or reporting of this systematic review.

### Registration and Protocol

This systematic review was not formally registered in a public repository. The review was conceived as an exploratory synthesis of emerging literature in a novel research area. Given its preliminary nature and evolving scope, formal registration was not pursued. However, all methodological decisions were transparently documented, in accordance with PRISMA 2020 guidelines.

## Supporting information




**Supplementary Materials**: Supp1‐Search‐Strategy‐Document.pdf

**Supplementary Materials**: Supp2‐Zotero‐Collection.zip

**Supplementary Materials**: Supp3‐Data‐Collection‐Form.xlsx

**Supplementary Materials**: Supp4‐Data‐Dictionary.pdf

**Supplementary Materials**: Supp5‐risk‐of‐bias‐table.xlsx

**Supplementary Materials**: Supp6‐Molecular‐Sampling‐Table.pdf

**Supplementary Materials**: Supp7‐Summary‐of‐Individual‐Studies.pdf

**Supplementary Materials**: Supp8‐PRISMA‐2020‐checklist‐compiled.docx

**Supplementary Materials**: Supp9‐PRISMA‐2020‐abstract‐checklist‐compiled.docx

**Supplementary Materials**: Supp10‐PRISMA‐S Checklist‐compiled.docx

## Footnotes

1 All percentages are calculated from the total of 37 studies.
