# Supplementary material for: Evidence of Physiological Comodulation During Human–Animal Interaction: A Systematic Review
Source: Ann N Y Acad Sci. 2026 Jun 4;1560(1):e70299. doi: 10.1111/nyas.70299 (PMC13238372; doi:10.1111/nyas.70299)
Supplement: Supplementary file 1 — Supplementary Materials: Supp1‐Search‐Strategy‐Document.pdf [file NYAS-1560-0-s005.pdf]

Evidence of Physiological Co-Modulation During Human-Animal Interaction: A Systematic Review - Search Strategy Document (S1)

The following databases have been separately consulted:

- PubMed (via NCBI interface; coverage: 1946–present, as per MEDLINE)
- EMBASE (via Elsevier; coverage: 1947–present)
- Scopus (via Elsevier; coverage: 1788–present, citation data from 1970)
- Google Scholar (via web interface; coverage not explicitly defined)
- Animal Studies Repository (via institutional repository interface; coverage: repository active since 2014, includes archival material).

No study registries have been consulted. Additionally, the research tool "Consensus app" (`https://consensus.app/`) was screened. Although not a traditional bibliographic database, the Consensus App was included to enhance sensitivity and identify potentially relevant studies not indexed in standard databases. Its use was exploratory and the top-ranked results were screened with the same eligibility criteria.
Consensus App was interrogated with prompts written in natural language and structured as questions, with whom this tool best performs. The search was conservatively limited to the first twenty results, due to the fact that, on average, less than 10 results of the output of this tool are properly related to the question asked.

Citation searching was performed for each eligible entry identified through database and tool interrogation (via Google Scholar web interface).
In performing citation search a filter for papers published after 2024 was applied in order to limit the inclusion to the most recent advances on the

subject. No other restrictions were applied regarding the year of publication. Additionally, the ‘related articles’ function in the Google Scholar web interface was performed, for each eligible entry identified through database and tool interrogation, to identify potentially relevant studies.

To identify additional relevant studies, faculty members, postdoctoral researchers, and research fellow affiliated with the CoMPBioS research group from the Department of Information engineering and related units at the University of Florence, as well as external collaborators and affiliated researchers working with CoMPBioS on related projects, were contacted and invited to share any publications they were aware of that met the inclusion criteria of this review.

For each database separate searches for the following eight keywords boolean combinations have been run:

- "human animal interaction" AND "physiological measures"
- "human animal interaction" AND "EEG"
- "human animal interaction" AND "PPG"
- "human animal interaction" AND "fNIRS"
- “human animal interaction" AND "heart rate"
- "human animal interaction" AND "oxytocin"
- "human animal interaction" AND "cortisol"
- "human animal interaction" AND "breath"

As an example, the following query was entered directly into the search bar of each database:

```
"human animal interaction" AND "EEG"
```

Separate searches were conducted for each keyword combination to facilitate workload distribution, maintain syntactic clarity, and ensure reproducibility. This approach also allowed for better control over search results,

as some databases handle complex Boolean logic differently, and minimized the risk of retrieving irrelevant records due to overly broad Boolean logic.

Consensus App was interrogated with the following prompts (this tool best performs with prompts written in natural language and structured as questions):

- "are there publications which correlate animal and human EEG measures in the context of human-animal interaction?"
- "are there publications which correlate animal and human PPG measures in the context of human-animal interaction?"
- "are there publications which correlate animal and human fNIRS measures in the context of human-animal interaction?"
- "are there publications which correlate animal and human heart rate measures in the context of human-animal interaction?"
- "are there publications which correlate animal and human oxytocin measures in the context of human-animal interaction?"
- "are there publications which correlate animal and human cortisol measures in the context of human-animal interaction?"
- "are there publications which correlate animal and human breath measures in the context of human-animal interaction?".

The screening on Consensus App was conservatively limited to the first twenty results, due to the fact that, on average, less than 10 results of the output of this tool are properly related to the question asked.
The term "correlate" was used in a broad sense to capture studies that simultaneously measured physiological signals in humans and animals during interaction and performed any quantitative analysis assessing their co-modulation (e.g., time-series coupling, correlation).

The first keywords combination ("human animal interaction" AND "physiological measures") and the first prompt ("are there publications which correlate animal and human physiological measures measures in the context of human-animal interaction?") were strictly based on the "Intervention" feature of the PICO for this review (Simultaneous measurement of physiological

parameters during human-animal interaction).
The term "physiological" has been interpreted as "*physical quantities such as temperature, cardiac activity, blood pressure, chemistry values in the blood and urine, enzymes, and proteins*"[1].
The choice of the composite keyword "human animal interaction" was based on its extensive use in the context of Animal Assisted Therapy (AAT) and Intervention (AAI) literature.
The decision to restrict the keyword strategy to the "Intervention" component of the PICO was aimed at maximizing the coverage of the search, in order to capture a wider spectrum of potentially relevant studies.
Screening of preliminary search results indicated that relevant literature extended beyond AAT/AAI contexts, in which both the terms "human-animal interaction" and its acronym HAI are well established[2]. This finding was in contrast to the a priori assumption that relevant studies would be situated within these contexts, thus revealing a potential sensitivity limitation of the initial keyword strategy. Thus, in order to enhance the sensitivity of the search, additional queries were performed using more specific alternatives to the general term "physiological parameters", namely the most frequently used physiological signals in current human-animal interaction research (EEG, PPG, fNIRS, heart rate, oxytocin, cortisol, breath).

To ensure methodological rigour and data availability, papers that had not been peer reviewed, master or bachelor thesis, unpublished papers, conference papers and papers written in languages other than English have been excluded.
In performing citation search a filter for papers published after 2024 was applied in order to limit the inclusion to the most recent advances on the subject. No other restrictions were applied regarding the year of publication.
All searches were conducted manually by entering each Boolean keyword combination directly into the search bar of each database's web interface, without the use of advanced syntax, field tags, or controlled vocabulary. No additional filters (e.g., language, date, species) were applied, except for limiting results to the category "article". This restriction was applied to exclude grey literature and ensure inclusion of peer-reviewed primary research, in line with the eligibility criteria of this review.
No search strategies from other literature reviews were adapted, reused or updated in this search.
All databases and tools were last consulted by the first reviewer on the 17th

of July 2025 in an additional final screening, with the aim of checking for wrongly ignored entries (which were not found).
Finally, all databases were last checked between the 1 and 5 of August 2025 by the second reviewer.

Citing and related papers were last consulted by the first reviewer on the 18th of July 2025 in an additional final screening, with the aim of checking for wrongly ignored entries (which were not found).
Finally, citing and related papers were last checked between the 1 and 5 of August 2025 by the second reviewer.

This search strategy has not undergone any peer review process.

Titles for a total of 1179 records were screened (1059 from bibliographic databases: Animal studies repository (n = 64), Cochrane (n = 5), EMBase (n = 303), PubMed (n = 93), Google Scholar (n = 400), Scopus (n = 194); and 120 from the Consensus App). Titles that clearly referred exclusively to behavioural measures, or to physiological parameters measured in either humans or animals only, were excluded.
Titles that did not explicitly mention physiological measures in both species, but also did not clearly indicate behavioural or single-species data, were further assessed at the abstract level.
Only studies that reported physiological measurements collected simultaneously in both humans and animals during interaction were included.

A total of 22 entries (14 from databases, 3 from consensus, 5 shared by the individuals who had been invited to contribute) were included.
Citation searching was performed for each eligible entry identified as well as interrogation of the 'related articles' tools in Google Scholar.
Additional 49 entries (7 from citation searching, 42 from 'related articles') that reported physiological measurements collected simultaneously in both humans and animals were included.

Search results have been saved in a Zotero collection (see: Supplementary File S2). All title-screened entries are stored in the folder "title screened", divided in subfolders that identify entries from each database, from citation searching, from related articles search and shared entries. All text-screened entries are stored in the folder "text screened", divided in subfolders that identify entries from each database, from citation searching, from related articles search and shared entries.

Three studies were excluded for being technological feasibility studies with no representative sample ($n \leq 2$) and did not focus on analysis of measured data[3,4,5]. One conference paper[6], one non-peer-reviewed report[7] and one paper written in Japanese[8] were excluded due to aforementioned eligibility criteria. Twenty-four papers were excluded because they did not directly and quantitatively compare physiological signals from human and animal subjects[9,10,11,12,13,14,15,16,17,18,19,20,21,22,23,24,25,26,27,28,29,30,31,32]. Thirty-seven studies that simultaneously measured physiological parameters in both humans and animals during an interaction and performed a co-modulation statistical analysis (defined as any quantitative analysis assessing their co-modulation) between the measured human and animal physiological signals, were included.

During the review phase an additional consultation of databases has been performed to look for entries in the specific HAI contexts[2] of animal-assisted therapy and animal-assisted intervention:
Specifically, we conducted additional Searches, with the boolean combination of keywords:("animal-assisted therapy" OR "animal-assisted intervention") AND ("physiological measures" OR "EEG" OR "PPG" OR "fNIRS" OR "heart rate" OR "oxytocin" OR "cortisol" OR "breath")

- EMBase (limited to 2020-2025 and limited to the category "article"): 76 results
- PubMed: 90 studies retrieved. 1 text-screened.
- scopus (limited to 2020-2025 and limited to title and abstract): 108 retrieved. 0 text screened.
- Scholar (with the adapted syntax: intitle:"animal-assisted" (EEG OR PPG OR fNIRS OR oxytocin OR cortisol OR "heart rate"): 113 results. 0 text screened.
- Animal Study Repository : 0 results.
- Consensus App (limitated to the first 20 results for the queries: "are there publications which correlate animal and human physiological measures such as EEG, PPG, fNIRS, heart rate, oxytocin, cortisol or breath in the context of animal-assisted therapy?" and "are there publications which correlate animal and human physiological measures such

as EEG, PPG, fNIRS, heart rate, oxytocin, cortisol or breath in the context of animal-assisted intervention?"): 40 retrieved. 0 included.

The two text screened studies were not eligible as:[33] did not directly and quantitatively compare physiological signals from human and animal subjects while[34] was eligible but was published on November 2025, after the last search of this review.

No automation processes have been used for the deduplication. A list of the eligible records has been constantly updated during the search, so that pre-identified eligible records were not recorded more than once.

References

[1] M. Frize, Measuring Physiological Variables in Humans, in: Health Care Engineering Part I, Springer International Publishing, Cham, 2014. doi:10.1007/978-3-031-01657-8_2.

[2] F. L. Green, M. L. Dahlman, A. Lomness, J.-T. Binfet, For the love of acronyms: An analysis of terminology and acronyms used in AAI research 2013–2023, Human-Animal Interactions (Jul. 2024). doi:10.1079/hai.2024.0024.

[3] M. Foster, E. Beppler, T. Holder, J. Dieffenderfer, P. Erb, K. Everette, M. Gruen, T. Somers, T. Evans, M. Daniele, D. L. Roberts, A. Bozkurt, A System for Assessment of Canine-Human Interaction during Animal-Assisted Therapies, in: 2018 40th Annual International Conference of the IEEE Engineering in Medicine and Biology Society (EMBC), IEEE, Honolulu, HI, 2018. doi:10.1109/EMBC.2018.8513384.

[4] A. Patel, M. Foster, T. Torfs, P. Ahmmed, I. Castro, T. Holder, A. Bozkurt, Noncontact Electrophysiology Monitoring Systems for Assessment of Canine-Human Interactions, 2021 IEEE Sensors (Oct. 2021). doi:10.1109/SENSORS47087.2021.9639748.

[5] T. Holder, M. Rahman, E. Summers, D. Roberts, C.-W. Wong, A. Bozkurt, Contact-Free Simultaneous Sensing of Human Heart Rate and Canine Breathing Rate for Animal Assisted Interactions, in: Proceedings of the Ninth International Conference on Animal-Computer Interaction, ACM, Newcastle-upon-Tyne United Kingdom, 2022. doi:10.1145/3565995.3566039.

[6] T. R. N. Holder, C. Nichols, E. Summers, D. L. Roberts, A. Bozkurt, Towards a Multimodal Synchronized System for Quantifying Psychophysiological States in Canine Assisted Interactions, in: The Tenth International Conference on Animal-Computer Interaction, ACM, Raleigh NC USA, 2023. doi:10.1145/3637882.3637886.

[7] E. K. Gehrke, The horse-human heart connection: Results of studies using heart rate variability, NAHRA's Strides, Spring (2010).

[8] T. Kikusui, Oxytocin bonds between human and dog, Japanese Journal of Animal Psychology, The Japanese Journal of Animal Psychology (2017). doi:10.2502/janip.67.1.1.

[9] K. Kotrschal, I. Schöberl, B. Bauer, A.-M. Thibeaut, M. Wedl, Dyadic relationships and operational performance of male and female owners and their male dogs, Behavioural Processes (Jul. 2009). doi:10.1016/j.beproc.2009.04.001.

[10] S. D. Clark, J. M. Smidt, B. A. Bauer, Therapy Dogs' and Handlers' Behavior and Salivary Cortisol During Initial Visits in a Complex Medical Institution: A Pilot Study (Nov. 2020). doi:10.3389/fvets.2020.564201.

[11] H. Hama, M. Yogo, Y. Matsuyama, Effects of stroking horses on both humans' and horses' heart rate responses[1], The Japanese psychological research (May 1996). doi:10.1111/j.1468-5884.1996.tb00009.x.

[12] A. Meinert, T. Deschner, F. Schaebs, S. Marshall-Pescini, F. Range, A. Gaugg, The Role of Oxytocin in the Dog–Owner Relationship, Animals : an Open Access Journal from MDPI (Oct. 2019). doi:10.3390/ani9100792.

[13] K. Merkies, A. Sievers, E. Zakrajsek, H. MacGregor, R. Bergeron, U. K. Von Borstel, Preliminary results suggest an influence of psychological and physiological stress in humans on horse heart rate and behavior, Journal of Veterinary Behavior (Sep. 2014). doi:10.1016/j.jveb.2014.06.003.

[14] A. Müller-Klein, M. N. Braun, D. S. Ferreira De Sá, T. Michael, U. Link-Dorner, J. Lass-Hennemann, A Relaxed Horse—A Relaxed Client? An Experimental Investigation of the Effects of Therapy Horses' Stress on Clients' Stress, Mood, and Anxiety, Animals (Feb. 2024). doi:10.3390/ani14040604.

[15] F. Pirrone, A. Ripamonti, E. C. Garoni, S. Stradiotti, M. Albertini, Measuring social synchrony and stress in the handler-dog dyad during animal-assisted activities: A pilot study, Journal of Veterinary Behavior (Sep. 2017). doi:10.1016/j.jveb.2017.07.004.

[16] M. Yoon, Y. Jung, The Effects of Human–Horse Interactions on Oxytocin and Cortisol Levels in Humans and Horses, Animals : an Open Access Journal from MDPI (Mar. 2025). doi:10.3390/ani15070905.

[17] J. Akiyama, M. Ohta, Hormonal and Neurological Aspects of Dog Walking for Dog Owners and Pet Dogs, Animals (Sep. 2021). doi:10.3390/ani11092732.

[18] N. Ille, M. Von Lewinski, R. Erber, M. Wulf, J. Aurich, E. Möstl, C. Aurich, Effects of the level of experience of horses and their riders on Cortisol release, heart rate and heart-rate variability during a jumping course (Nov. 2013). doi:10.7120/09627286.22.4.457.

[19] N. Ille, C. Aurich, R. Erber, M. Wulf, R. Palme, J. Aurich, M. Von Lewinski, Physiological stress responses and horse rider interactions in horses ridden by male and female riders, CEP (Jan. 2014). doi:10.3920/cep143001.

[20] L. Handlin, E. Hydbring-Sandberg, A. Nilsson, M. Ejdebäck, A. Jansson, K. Uvnäs-Moberg, Short-Term Interaction between Dogs and Their Owners: Effects on Oxytocin, Cortisol, Insulin and Heart Rate—An Exploratory Study, Anthrozoös (Sep. 2011). doi:10.2752/175303711X13045914865385.

[21] D. K. Haubenhofer, S. Kirchengast, 'Dog Handlers' and Dogs' Emotional and Cortisol Secretion Responses Associated with Animal-Aassisted Therapy Sessions, Soc Animals (2007). doi:10.1163/156853007x187090.

[22] M. Von Lewinski, S. Biau, R. Erber, N. Ille, J. Aurich, J.-M. Faure, E. Möstl, C. Aurich, Cortisol release, heart rate and heart rate variability in the horse and its rider: Different responses to training and performance, The Veterinary Journal (Aug. 2013). doi:10.1016/j.tvjl.2012.12.025.

[23] M. Peeters, C. Closson, J.-F. Beckers, M. Vandenheede, Rider and Horse Salivary Cortisol Levels During Competition and Impact on Performance, Journal of Equine Veterinary Science (Mar. 2013). doi:10.1016/j.jevs.2012.05.073.

[24] M. D. Ayala, A. Carrillo, P. Iniesta, P. Ferrer, Pilot Study of the Influence of Equine Assisted Therapy on Physiological and Behavioral Parameters Related to Welfare of Horses and Patients, Animals (Dec. 2021). doi:10.3390/ani11123527.

[25] D. Crews, The Bond Between a Horse and a Human, Nat Prec (Jul. 2009). doi:10.1038/npre.2009.3454.1.

[26] G. E. Gnanadesikan, E. Carranza, K. M. King, A. C. Flyer, G. Ossello, P. G. Smith, N. G. Steklis, H. D. Steklis, J. J. Connelly, M. Barnett, N. Gee, S. Tecot, E. L. MacLean, Glucocorticoid response to naturalistic interactions between children and dogs, Hormones and Behavior (May 2024). doi:10.1016/j.yhbeh.2024.105523.

[27] K. Malinowski, C. Yee, J. M. Tevlin, E. K. Birks, M. M. Durando, H. Pournajafi-Nazarloo, A. A. Cavaiola, K. H. McKeever, The Effects of Equine Assisted Therapy on Plasma Cortisol and Oxytocin Concentrations and Heart Rate Variability in Horses and Measures of Symptoms of Post-Traumatic Stress Disorder in Veterans, Journal of Equine Veterinary Science (May 2018). doi:10.1016/j.jevs.2018.01.011.

[28] C. C. Munsters, K. E. Visser, J. Van Den Broek, M. M. Sloet Van Oldruitenborgh-Oosterbaan, The influence of challenging objects and horse-rider matching on heart rate, heart rate variability and behavioural score in riding horses, The Veterinary Journal (Apr. 2012). doi:10.1016/j.tvjl.2011.04.011.

[29] J. Odendaal, R. Meintjes, Neurophysiological Correlates of Affiliative Behaviour between Humans and Dogs, The Veterinary Journal (May 2003). doi:10.1016/S1090-0233(02)00237-X.

[30] J. Odendaal, Animal-assisted therapy — magic or medicine?, Journal of Psychosomatic Research (Oct. 2000). doi:10.1016/S0022-3999(00)00183-5.

[31] D. Haubenhofer, E. Möstl, S. Kirchengast, Cortisol concentrations in saliva of humans and their dogs during intensive training courses in animal-assisted therapy, Wiener Tierarztliche Monatsschrift (Jan. 2005).

[32] N. A. Dreschel, D. A. Granger, Physiological and behavioral reactivity to stress in thunderstorm-phobic dogs and their caregivers, Applied Animal Behaviour Science (Dec. 2005). doi:10.1016/j.applanim.2005.04.009.

[33] V. O. Giuliano, L. Sacchettino, A. S. Rusu, D. Ciccarelli, V. Gazzano, M. De Cesare, M. Visone, V. Mizzoni, F. Napolitano, D. d'Angelo, Well-Being Indicators in Autistic Children and Therapy Dogs During a Group Intervention: A Pilot Study, Animals (Jul. 2025). doi:10.3390/ani15142032.

[34] A. Helmer, A. Hacohen, O. Bart, Child horse harmony in motion: A preliminary study to explore heart rate synchronization in equine assisted therapy for neurotypical and ADHD children, Scientific reports (2025). doi:10.1038/s41598-025-29330-6.
