# Supplementary material for: Evidence of Physiological Comodulation During Human–Animal Interaction: A Systematic Review
Source: Ann N Y Acad Sci. 2026 Jun 4;1560(1):e70299. doi: 10.1111/nyas.70299 (PMC13238372; doi:10.1111/nyas.70299)
Supplement: Supplementary file 2 — Supplementary Materials: Supp2‐Zotero‐Collection.zip [file NYAS-1560-0-s002.zip › Supp2_Zotero_Collection/new searches/Animal Study Repository.htm]

Zotero Report


- ## Searching: WBI Studies Repository

  |  |  |
  | --- | --- |
  | Item Type | Web Page |
  | URL | https://www.wellbeingintlstudiesrepository.org/do/search/?q=abstract%3A(%20%22animal-assisted%20therapy%22%20OR%20%22animal-assisted%20intervention%22%20AND%20(%22physiological%20measures%22%20OR%20%22EEG%22%20OR%20%22PPG%22%20OR%20%22fNIRS%22%20OR%20%22heart%20rate%22%20OR%20%22oxytocin%22%20OR%20%22cortisol%22%20OR%20%22breath%22%20)&start=0&context=4157404&facet= |
  | Accessed | 05/02/2026, 17:36:06 |
  | Date Added | 05/02/2026, 17:36:06 |
  | Modified | 05/02/2026, 17:36:06 |

  ### Attachments

  - Searching: WBI Studies Repository
