# Supplementary material for: Evidence of Physiological Comodulation During Human–Animal Interaction: A Systematic Review
Source: Ann N Y Acad Sci. 2026 Jun 4;1560(1):e70299. doi: 10.1111/nyas.70299 (PMC13238372; doi:10.1111/nyas.70299)
Supplement: Supplementary file 2 — Supplementary Materials: Supp2‐Zotero‐Collection.zip [file NYAS-1560-0-s002.zip › Supp2_Zotero_Collection/new searches/Consensus.htm]

Zotero Report


- ## are there publications which correlate animal and human physiological measures such as EEG, PPG, fNIRS, heart rate, oxytocin, cortisol or breath in the context of animal-assisted intervention - 05 feb 2026

  |  |  |
  | --- | --- |
  | Item Type | Attachment |
  | Date Added | 05/02/2026, 18:44:19 |
  | Modified | 05/02/2026, 18:44:19 |
- ## are there publications which correlate animal and human physiological measures such as EEG, PPG, fNIRS, heart rate, oxytocin, cortisol or breath in the context of animal-assisted therapy - 05 feb 2026

  |  |  |
  | --- | --- |
  | Item Type | Attachment |
  | Date Added | 05/02/2026, 18:44:19 |
  | Modified | 05/02/2026, 18:44:19 |
