# Supplementary material for: Evidence of Physiological Comodulation During Human–Animal Interaction: A Systematic Review
Source: Ann N Y Acad Sci. 2026 Jun 4;1560(1):e70299. doi: 10.1111/nyas.70299 (PMC13238372; doi:10.1111/nyas.70299)
Supplement: Supplementary file 2 — Supplementary Materials: Supp2‐Zotero‐Collection.zip [file NYAS-1560-0-s002.zip › Supp2_Zotero_Collection/new searches/EMBase.htm]

Zotero Report


- ## Robot therapy aids mental health in patients with hematological malignancy during hematopoietic stem cell transplantation in a protective isolation unit

  |  |  |
  | --- | --- |
  | Item Type | Journal Article |
  | Author | A. Yamada |
  | Author | D. Akahane |
  | Author | S. Takeuchi |
  | Author | K. Miyata |
  | Author | T. Sato |
  | Author | A. Gotoh |
  | Abstract | Patients with hematological malignancy experience physical and psychological pain, such as a sense of isolation and confinement due to intensive chemotherapy in a protective isolation unit (PIU). We examined whether the intervention of a robotic puppy, aibo (manufactured by Sony), could improve patients' mental health as an alternative therapy for pet therapy, which is not feasible in PIU. This study included 21 patients undergoing allogeneic hematopoietic stem cell transplantation (HSCT) (n = 16) or autologous HSCT (n = 5). The patients were randomly divided into the aibo and control groups. Psychological effects were regularly assessed by measuring the levels of salivary stress hormone chromogranin A (CgA), serum oxytocin, and serum cortisol and the quick Inventory of Depressive Symptomatology Self-Report (QIDS-SR) scores. The aibo group demonstrated a significant decrease in CgA level, while the control group showed the opposite trend. In addition, changes in serum oxytocin and cortisol levels indicated that aibo helped reduce stress. There was no significant difference in the QIDS-SR scores between the two groups; however, the psychomotor activity in the aibo group improved significantly. These findings suggest that aibo intervention during a stay in a PIU can improve the mental health of patients with hematological malignancies who have undergone HSCT. |
  | Date | 2024 |
  | Language | English |
  | Archive | Medline |
  | URL | https://www.embase.com/search/results?subaction=viewrecord&id=L643621309&from=export |
  | Volume | 14 |
  | Pages | 4737 |
  | Publication | Scientific reports |
  | DOI | 10.1038/s41598-024-54286-4 |
  | Issue | 1 |
  | Journal Abbr | Sci Rep |
  | ISSN | 2045-2322 |
  | Date Added | 05/02/2026, 17:58:39 |
  | Modified | 05/02/2026, 17:58:39 |

  ### Tags:

  - therapy
  - article
  - female
  - male
  - human
  - hydrocortisone
  - adult
  - controlled study
  - randomized controlled trial
  - quality of life
  - major clinical study
  - clinical article
  - chromogranin A
  - hydrocortisone blood level
  - mental health
  - pet therapy
  - puppy
  - oxytocin blood level
  - stress hormone
  - allogeneic hematopoietic stem cell transplantation
  - autologous hematopoietic stem cell transplantation
  - chromogranin
  - hematologic malignancy
  - hematopoietic stem cell transplantation
  - psychomotor activity
  - quick inventory of depressive symptomatology

  ### Attachments

  - Full Text (HTML)
- ## Can dog-assisted intervention decrease anxiety level and autonomic agitation in patients with anxiety disorders?

  |  |  |
  | --- | --- |
  | Item Type | Journal Article |
  | Author | D. Wołyńczyk-Gmaj |
  | Author | A. Ziółkowska |
  | Author | P. Rogala |
  | Author | D. Ścigała |
  | Author | L. Bryła |
  | Author | B. Gmaj |
  | Author | M. Wojnar |
  | Abstract | Few studies have explored the influence of an Animal-Assisted Intervention on patients with mental disorders. We investigated it’s impact on anxiety symptoms. We divided 51 patients with anxiety symptoms into two groups—treatment group, that went for a short 15–20 min’ walk with a dog, his handler and a researcher and control group, that went for a walk only with a researcher. We used State-Trait Anxiety Inventory (STAI), Visual Analogue Scale (VAS) of fear, Beck Depression Inventory (BDI), Ford Insomnia Response to Stress (FIRST), Brief symptom Inventory (BSI) and VAS of satisfaction after trial to assess. We also checked the resting blood pressure and resting heart rate before and after performing psychological tests while sitting. We have obtained full data of 21 people from the research group and 26 people from the control group. After the intervention, the treatment group reported lower anxiety levels as a state (Mean (M) = 34.35; Standard Deviation (SD) = 6.9 vs. M = 40.94; SD = 8.6) and fear (M = 1.05; SD = 1.0 vs. M = 2.04; SD = 2.2) than the control group. After a walk with a dog, trait anxiety (M = 34.35; SD = 6.9 vs. M = 46.3; SD = 9.6), state anxiety (M = 48.9; SD = 7.2 vs. M = 53.9; SD = 7.8), fear (M = 1.05; SD= 1.0 vs. M = 2.57; SD = 2.3) and resting heart rate (M = 71.05; SD = 12.3 vs. M = 73.67; SD = 13.1) decreased significantly, while walking without a dog only reduced state anxiety (M = 47.24; SD = 11.0 vs. M = 40.94; SD = 8.6). Multivariate analysis of variance showed that after the walk, state anxiety was significantly lower in the treatment group than in the control group, F(1.35) = 6.706, p <0.05, η2 = 0.161. Among those who walked with a dog, the intervention also led to significant decreases in fear and resting heart rate, F(1.44) = 11.694, p < 0.01, η2 = 0.210 and F(1.45) = 8.503; p < 0.01; η2 = 0.159, respectively. For anxious patients, a short walk with a dog is more beneficial than a walk without one. We found significant positive effects of a dog’s company on vegetative arousal and mental comfort. This is another study confirming the possible therapeutic effect of the animal on anxiety symptoms. Further research is required, especially in the large groups of patients, as recommendations on the use of Animal Assisted Interventions (AAI) are needed. |
  | Date | 2021 |
  | Language | English |
  | Archive | Embase |
  | URL | https://www.embase.com/search/results?subaction=viewrecord&id=L2014393225&from=export |
  | Volume | 10 |
  | Publication | Journal of Clinical Medicine |
  | DOI | 10.3390/jcm10215171 |
  | Issue | 21 |
  | Journal Abbr | J. Clin. Med. |
  | ISSN | 2077-0383 |
  | Date Added | 05/02/2026, 17:58:51 |
  | Modified | 05/02/2026, 17:58:51 |

  ### Tags:

  - blood pressure
  - agitation
  - article
  - female
  - male
  - nonhuman
  - physiological stress
  - human
  - adult
  - Brief Symptom Inventory
  - controlled study
  - questionnaire
  - group therapy
  - rating scale
  - visual analog scale
  - clinical article
  - fear
  - sitting
  - satisfaction
  - animal assisted therapy
  - service dog
  - State Trait Anxiety Inventory
  - therapy effect
  - arousal
  - walking
  - anxiety disorder
  - Beck Depression Inventory
  - psychologic test
  - insomnia
  - resting heart rate

  ### Attachments

  - Full Text (HTML)
- ## The effects of Animal Assisted Therapy on autonomic and endocrine activity in adults with autism spectrum disorder: A randomized controlled trial

  |  |  |
  | --- | --- |
  | Item Type | Journal Article |
  | Author | C. Wijker |
  | Author | N. Kupper |
  | Author | R. Leontjevas |
  | Author | A. Spek |
  | Author | M.-J. Enders-Slegers |
  | Abstract | Objective: Stress and its sequelae are very common in adults with autism spectrum disorder (ASD) without an intellectual disability (ID). Animal-assisted therapy (AAT) has shown physiological stress-reductive effects in children with ASD. The aim of the current study was to examine the acute psychophysiological response to an AAT session, and to examine the longer-term stress-physiological effects of the intervention, up until 10 weeks post-treatment, in comparison to waiting-list controls. Method: A randomized controlled trial with pre-intervention (T0), post-intervention (T1: 10 weeks) and follow-up (T2: 20 weeks) measurements of neuroendocrine and cardiovascular measures, was conducted in 53 adults with ASD (N = 27 in intervention arm; N = 26 in control arm). Within the intervention group, stress-physiological data were collected during the 5th therapy session (acute effects). Data were analyzed with mixed models for outcome measures cortisol, alpha-amylase, heart rate variability and sympathetic activity. Results: The AAT interventional session was significantly associated with reduced cortisol levels (β = −0.41, p = .010), while parasympathetic and sympathetic cardiovascular activity remained unaltered. No significant changes were found for stress-physiological measures at post-treatment time points. Conclusions: Acute stress reduction, reflected in significant reduction in cortisol levels, was found during an AAT session in adults with ASD, without ID. More research is needed to explore to what extent the specific factors of AAT have contributed to the decrease in cortisol and whether stress reduction is possible for the longer-term. |
  | Date | 2021 |
  | Language | English |
  | Archive | Embase |
  | URL | https://www.embase.com/search/results?subaction=viewrecord&id=L2013403572&from=export |
  | Volume | 72 |
  | Pages | 36-44 |
  | Publication | General Hospital Psychiatry |
  | DOI | 10.1016/j.genhosppsych.2021.05.003 |
  | Issue | (Wijker C., carolien.wijker@ou.nl) GGZ Oost Brabant, Berlicumseweg 8, 5248 NT Rosmalen, Netherlands |
  | Journal Abbr | Gen. Hosp. Psychiatry |
  | ISSN | 1873-7714 |
  | Date Added | 05/02/2026, 17:58:52 |
  | Modified | 05/02/2026, 17:58:52 |

  ### Tags:

  - heart rate variability
  - article
  - female
  - male
  - nonhuman
  - physiological stress
  - human
  - hydrocortisone
  - adult
  - controlled study
  - follow up
  - randomized controlled trial
  - autism
  - major clinical study
  - outcome assessment
  - amylase
  - animal assisted therapy
  - hydrocortisone blood level
  - neuroendocrine system
  - autonomic nervous system function
  - single blind procedure
  - 5938
  - biofeedback
  - cardiovascular function
  - endocrine function
  - sex ratio
  - sympathetic tone

  ### Attachments

  - Full Text (HTML)
- ## Breast Cancer: How Hippotherapy Bridges the Gap between Healing and Recovery—A Randomized Controlled Clinical Trial

  |  |  |
  | --- | --- |
  | Item Type | Journal Article |
  | Author | H. Viruega |
  | Author | C. Galy |
  | Author | C. Loriette |
  | Author | S. Jacquot |
  | Author | J.L. Houpeau |
  | Author | M. Gaviria |
  | Abstract | Background: Breast cancer is the most diagnosed women’s cancer, and has a high survival rate. Despite great progress in detection and treatment, life reconstruction requires comprehensive cross-sectoral approaches between different disciplines and deeper consideration of the patient’s challenges. Hippotherapy is an emerging specialized rehabilitation approach, performed by accredited health professionals and equine specialists, on specially trained horses via their movement, activating major paths for physical, mental, psychic and social reinforcement, and is synergistic to rehabilitative and supportive care. Methods: We conducted a randomized open, prospective, two-armed, controlled trial on the effectiveness of hippotherapy versus conventional supportive care on adult women with a diagnosis of breast cancer, after the period of primary treatment (surgery, chemotherapy, radiotherapy). The 6-month program included, in the treated group, an initial 1-week daily hippotherapy session, followed by three short 2-day sessions with an interval of 2 months between each, where the patients received conventional supportive care. The control group received 6 months of conventional supportive care. The primary end point was quality of life. Cognitive performances, fatigue, anxiety, depression, and body image were the secondary end points. Measurements were done through self-reported questionnaires. Results: We observed statistical differences in the evolution of the measured parameters over time between the two groups. The hippotherapy group showed a much faster, favorable and continuous improvement until the end of the program for each function assessed. The most striking improvements were observed in global quality of life, and fatigue, while breast cancer-specific quality of life, cognitive performance, anxiety and depression and body image showed a less marked but still statistically significant difference at the final post-treatment evaluation. Conclusions: We demonstrate the therapeutic relevance of hippotherapy, a one-health approach, as a key initial stage after cancer diagnosis and treatment to foster recovery. Furthermore, hippotherapy has a strong impact on cancer treatments’ efficiency and reconstruction of patient’s life and ecosystem. This work reveals a layer of complexity that needs to be broadly considered. Trial registration: ClincalTrials.gov NCT04350398 accessed on 1 January 2022. Registered 17 April 2020, retrospectively registered; French Clinical Trials in Cancer Register RECF3818. Registered 18 March 2019, retrospectively registered. |
  | Date | 2023 |
  | Language | English |
  | Archive | Embase |
  | URL | https://www.embase.com/search/results?subaction=viewrecord&id=L2021799509&from=export |
  | Volume | 15 |
  | Publication | Cancers |
  | DOI | 10.3390/cancers15041317 |
  | Issue | 4 |
  | Journal Abbr | Cancers |
  | ISSN | 2072-6694 |
  | Date Added | 05/02/2026, 17:58:44 |
  | Modified | 05/02/2026, 17:58:44 |

  ### Tags:

  - anxiety
  - cognition
  - article
  - female
  - human
  - adult
  - hippotherapy
  - randomized controlled trial
  - psychologic assessment
  - quality of life
  - major clinical study
  - mental performance
  - depression
  - Likert scale
  - fatigue
  - histology
  - diarrhea
  - vomiting
  - Beck Depression Inventory
  - prospective study
  - breathing exercise
  - working memory
  - body image
  - breast cancer
  - cancer staging
  - constipation
  - ductal breast carcinoma in situ
  - dyspnea
  - Hospital Anxiety and Depression Scale
  - insomnia
  - invasive ductal breast carcinoma
  - lobular carcinoma
  - lobular carcinoma in situ
  - mastectomy
  - Multidimensional Fatigue Inventory
  - NCT04350398
  - triple negative breast cancer

  ### Attachments

  - Full Text (HTML)
- ## Effects of Essential Animal Visitation Program (AVP) Components on Students’ Salivary α-Amylase and Amylase-to-Cortisol Ratios

  |  |  |
  | --- | --- |
  | Item Type | Journal Article |
  | Author | J.L. Vandagriff |
  | Author | A.M. Carr |
  | Author | S.M. Roeter Smith |
  | Author | P. Pendry |
  | Abstract | While efficacy trials suggest that Animal Visitation Programs (AVPs) relieve university student stress, their essential components are unknown. Students were randomly assigned to one of four 10-min conditions: AVP touch (n = 73), AVP proximity (n = 62), AVP imagery (n = 57), or AVP waitlist (n = 57). Participants collected salivary cortisol (Cort) and α-amylase (sAA) upon waking and at 15 and 25 min post-condition from which parameters indicating adaptive physiological functioning were calculated. Multiple linear regression analyses showed that students in all three comparison conditions had lower posttest sAA (β proximity = −0.175, p = 0.017; β imagery = −0.214, p = 0.003; β waitlist = −0.138, p = 0.051), lower sAA-to-Cort ratios (AOCs) from pretest to posttest (β proximity = −0.277, p < 0.001; β imagery = −0.307, p < 0.001; β waitlist = −0.172, p = 0.014), lower AOCs from wakeup to posttest (β proximity = −0.135, p = 0.010; β imagery = −0.150, p = 0.004; β waitlist = −0.117, p = 0.021), and a smaller sAA increase from wakeup to posttest (β proximity = −0.216, p = 0.001; β imagery = −0.247, p < 0.001; β waitlist = −0.130, p = 0.033) compared with the AVP touch condition, indicating greater autonomic arousal (sAA) and coordination of stress systems (AOCs) in AVP touch participants. These results suggest that touch is the primary AVP component facilitating adaptive stress-related physiological states among participating university students. |
  | Date | 2022 |
  | Language | English |
  | Archive | Embase |
  | URL | https://www.embase.com/search/results?subaction=viewrecord&id=L2014306471&from=export |
  | Volume | 35 |
  | Pages | 443-461 |
  | Publication | Anthrozoos |
  | DOI | 10.1080/08927936.2021.1996025 |
  | Issue | 3 |
  | Journal Abbr | Anthrozoos |
  | ISSN | 1753-0377 |
  | Date Added | 05/02/2026, 17:58:50 |
  | Modified | 05/02/2026, 17:58:50 |

  ### Tags:

  - human-animal interaction
  - autonomic nervous system
  - anxiety
  - article
  - female
  - male
  - physiological stress
  - human
  - hydrocortisone
  - adult
  - human experiment
  - normal human
  - touch
  - young adult
  - amylase
  - hypothalamus hypophysis adrenal system
  - arousal
  - depression
  - mental health
  - emotionality
  - saliva level
  - saliva analysis
  - adrenal medullary cell
  - animal visitation program
  - animal visitation program imagery
  - animal visitation program proximity
  - animal visitation program waitlist
  - Beck Anxiety Inventory
  - Beck Depression Inventory
  - catecholamine
  - health care organization
  - health survey
  - Penn State Worry Questionnaire
  - Perceived Stress Scale
  - pretest posttest design
  - sympathetic reflex
  - university student

  ### Attachments

  - Full Text (HTML)
- ## Do service dogs for veterans with ptsd mount a cortisol response in response to training?

  |  |  |
  | --- | --- |
  | Item Type | Journal Article |
  | Author | E.A.E. van Houtert |
  | Author | N. Endenburg |
  | Author | T. Bas Rodenburg |
  | Author | E. Vermetten |
  | Abstract | Only a few studies have investigated the welfare of animals participating in animal-assisted interventions (AAIs). Most of these studies focus on dogs in therapeutic settings. There are, however, also dogs—service dogs—that are employed to continuously support a single human. Because the welfare of these service dogs is important for the sustainability of their role, the aim of this study was to investigate their stress response to service dog training sessions. To do this, we took repeated salivary cortisol samples from dogs who participated in a training session (n = 19). Samples were taken just after arrival at the training ground, before training, after training, and after a period of free play. Our results showed that mean cortisol levels in all samples were relatively low (between 1.55 ± 1.10 and 2.73 ± 1.47 nmol/L) compared to similar studies. Analysis further showed that samples taken before and after participation in the training’s session did not differ from one another. Mean cortisol levels in both situations were additionally lower than those upon arrival at the training site and after a period of free play. This led to the conclusion that the dogs in our study did not seem to experience training as stressful. |
  | Date | 2021 |
  | Language | English |
  | Archive | Embase |
  | URL | https://www.embase.com/search/results?subaction=viewrecord&id=L2006118482&from=export |
  | Volume | 11 |
  | Pages | 1-9 |
  | Publication | Animals |
  | DOI | 10.3390/ani11030650 |
  | Issue | 3 |
  | Journal Abbr | Animals |
  | ISSN | 2076-2615 |
  | Date Added | 05/02/2026, 17:58:53 |
  | Modified | 05/02/2026, 17:58:53 |

  ### Tags:

  - veteran
  - animal welfare
  - animal experiment
  - article
  - female
  - male
  - nonhuman
  - hydrocortisone
  - posttraumatic stress disorder
  - exercise
  - training
  - service dog
  - immunoassay
  - saliva analysis
  - animal assisted intervention

  ### Attachments

  - Full Text (HTML)
- ## Can a Facility Dog Improve Inpatient Rehabilitation Engagement? A Randomized Controlled Crossover Trial

  |  |  |
  | --- | --- |
  | Item Type | Journal Article |
  | Author | H. Tropiano |
  | Author | J. Robertson |
  | Author | A. Rathbun |
  | Author | B.T. Yates |
  | Author | N.M. Fromm |
  | Abstract | Facility dogs offer novel ways to surmount barriers common to inpatient rehabilitation. The purpose of this study was to conduct the first investigation of the effects of a facility dog in physical therapy. In this randomized controlled crossover trial, 69 inpatients recovering from diverse acute conditions were introduced to a facility dog and engaged in one of five common rehabilitation tasks in collaboration with the dog. Participants were assigned randomly to interact with the facility dog on the first or second trial of the same task during a rehabilitation session. Linear mixed effects models, accounting for repeated measures within participants and other study design parameters, were used to assess associations between the presence of a facility dog and task persistence, self-rated pain and exertion, and physiological measures. Participants spent approximately 2.6 more minutes (95% CI [1.78, 3.41]; p < 0.001) engaged in goal-directed rehabilitation tasks when interacting with the facility dog, handler, and physical therapist compared with sessions with only the physical therapist and handler. Similarly, participants without a dog at home persisted significantly longer on tasks with the facility dog (2.00 min; 95% CI [−3.61, −0.39]; p = 0.015) compared with those who did not currently own a dog. Pain and heart rate varied by task order and facility dog presence: both were lower during the first task with the dog but significantly higher during the second, suggesting a shared physiological response to cumulative effort or arousal. These findings suggest that the presence of a facility dog may enhance patient participation through cognitive mechanisms such as distraction and increased engagement, likely supported by the positive emotional response elicited during therapy. |
  | Date | 2025 |
  | Language | English |
  | Archive | Embase |
  | URL | https://www.embase.com/search/results?subaction=viewrecord&id=L2035428893&from=export |
  | Volume | 38 |
  | Pages | 851-867 |
  | Publication | Anthrozoos |
  | DOI | 10.1080/08927936.2025.2529681 |
  | Issue | 5 |
  | Journal Abbr | Anthrozoos |
  | ISSN | 1753-0377 |
  | Date Added | 05/02/2026, 17:58:31 |
  | Modified | 05/02/2026, 17:58:31 |

  ### Tags:

  - human-animal interaction
  - heart rate
  - dog
  - emotion
  - cognition
  - article
  - female
  - male
  - nonhuman
  - human
  - aged
  - controlled study
  - data analysis software
  - physiotherapy
  - randomized controlled trial
  - crossover procedure
  - physiotherapist
  - pain
  - diastolic blood pressure
  - systolic blood pressure
  - arousal
  - G Power 3.1.9.6
  - hospital patient
  - numeric rating scale
  - patient engagement
  - patient participation

  ### Attachments

  - Full Text (HTML)
- ## Best practices for physiological data collection in youth with autism and co-occurring mental health diagnoses: Implications for human-animal intervention research

  |  |  |
  | --- | --- |
  | Item Type | Journal Article |
  | Author | C.M. Smith |
  | Author | K. Weimann |
  | Author | M. Widick |
  | Author | T. Merritt |
  | Author | H. Christensen |
  | Author | M. Siegel |
  | Author | Z. Pan |
  | Author | R.L. Gabriels |
  | Abstract | The purpose of this paper is to serve as a catalyst for the human-animal interaction research field to improve scientific rigor and accelerate the knowledge of field-based physiological responses during equine-assisted services in youth with autism spectrum disorder. This paper outlines the best practices for collecting and analyzing electrocardiogram and electrodermal activity in youth with autism spectrum disorder, utilized during a 10-week therapeutic horseback riding intervention. • Motivation strategies such as device choice, reward systems, and a visual schedule should be implemented to improve participant compliance. In addition, devices should be secured to the participant following implementation of appropriate desensitization techniques. • Time-domain heart rate variability analyses are more appropriate during therapeutic horseback riding data collection compared to frequency-domain approaches. For electrodermal activity, tonic responses should be assessed as opposed to phasic analyses. • An effective data monitoring team including the Data Collection Research Personnel, Site Principal Investigator, Physiologist, and Therapeutic Riding Center Intervention Lead are key to increasing the quality of usable data in equine-assisted service research environments. |
  | Date | 2025 |
  | Language | English |
  | Archive | Embase |
  | URL | https://www.embase.com/search/results?subaction=viewrecord&id=L2038140847&from=export |
  | Volume | 14 |
  | Publication | MethodsX |
  | DOI | 10.1016/j.mex.2025.103284 |
  | Issue | (Smith C.M., Cory\_M\_Smith@Baylor.edu) Baylor University Waco, TX Robbins College of Health and Human Sciences, United States |
  | Journal Abbr | MethodsX |
  | ISSN | 2215-0161 |
  | Date Added | 05/02/2026, 17:58:28 |
  | Modified | 05/02/2026, 17:58:28 |

  ### Tags:

  - heart rate variability
  - child
  - article
  - female
  - male
  - human
  - controlled study
  - data analysis software
  - hippotherapy
  - horseback riding
  - randomized controlled trial
  - information processing
  - sensor
  - motivation
  - adolescent
  - autism
  - best practice
  - desensitization
  - devices
  - electrocardiograph
  - electrocardiography
  - electrode
  - electrodermal response
  - information processing device
  - juvenile
  - major clinical study
  - mental disease
  - NCT04606966
  - patient compliance
  - personnel
  - reward

  ### Attachments

  - Full Text (HTML)
- ## Role of pets and animal assisted therapy in suicide prevention

  |  |  |
  | --- | --- |
  | Item Type | Journal Article |
  | Author | S. shoib |
  | Author | S.S. Hussaini |
  | Author | M. Chandradasa |
  | Author | F. Saeed |
  | Author | T. Khan |
  | Author | S. Swed |
  | Author | A. Lengvenyte |
  | Date | 2022 |
  | Language | English |
  | Archive | Embase |
  | URL | https://www.embase.com/search/results?subaction=viewrecord&id=L2019572715&from=export |
  | Volume | 80 |
  | Publication | Annals of Medicine and Surgery |
  | DOI | 10.1016/j.amsu.2022.104153 |
  | Issue | (shoib S., Sheikhshoib22@gmail.com) Department of Psychiatry, Jawahar Lal Nehru Memorial Hospital, Kashmir, Srinagar, India |
  | Journal Abbr | Ann. Med. Surg. |
  | ISSN | 2049-0801 |
  | Date Added | 05/02/2026, 17:53:32 |
  | Modified | 05/02/2026, 17:53:32 |

  ### Tags:

  - blood pressure
  - article
  - nonhuman
  - human
  - hydrocortisone
  - epinephrine
  - posttraumatic stress disorder
  - pet animal
  - autism
  - mental disease
  - mental stress
  - animal assisted therapy
  - cat
  - mental health
  - dementia
  - public health
  - schizophrenia
  - aldosterone
  - automutilation
  - cause of death
  - primary medical care
  - psychiatric treatment
  - suicide
  - suicide attempt
  - World Health Organization

  ### Attachments

  - Full Text (HTML)
- ## Non-randomized controlled trial examining the effects of livestock on motivation and anxiety in patients with chronic psychiatric disorders

  |  |  |
  | --- | --- |
  | Item Type | Journal Article |
  | Author | N. Shimizu |
  | Author | C. Yamazaki |
  | Author | K. Asano |
  | Author | S. Ohe |
  | Author | M. Ishida |
  | Abstract | Objectives: Patients with chronic schizophrenia exhibit negative symptoms, including decreased work motivation. Animal-assisted therapy programs have been reported to benefit such patients; hence, there is a possibility that sheep-rearing, rather than conventional employment training, may motivate these patients. Therefore, we investigated the effects of a one-day experiential learning program of sheep-rearing on the work motivation and anxiety of patients with chronic schizophrenia. Methods: Fourteen patients were included in a non-randomized controlled trial conducted between August 2018 and October 2018. The patients’ participation in the sheep-rearing experiential learning (one day; intervention day) and normal day care (one day; control day) programs were compared. The salivary cortisol and testosterone levels and State-Trait Anxiety Inventory (STAI) scores of the patients were analyzed. Results: The patients’ salivary testosterone was significantly higher on the intervention day (p = 0.04) than on the control day (p = 0.02). Their salivary cortisol was lower on the control day than on the intervention day, although the difference was not significant. Regression analysis was performed based on the change in salivary cortisol levels and STAI-Trait scores (p = 0.006), and a regression equation was established. Conclusions: The study revealed that participation in sheep-rearing may have promoted the testosterone production but did not increase anxiety in patients with schizophrenia. Additionally, regression equations for salivary cortisol levels in such patients may provide information on individual differences in anxiety levels. |
  | Date | 2023 |
  | Language | English |
  | Archive | Embase |
  | URL | https://www.embase.com/search/results?subaction=viewrecord&id=L2023366262&from=export |
  | Volume | 11 |
  | Publication | SAGE Open Medicine |
  | DOI | 10.1177/20503121231175291 |
  | Issue | (Shimizu N., n-shimizu@pu-toyama.ac.jp; Yamazaki C.) Faculty of Nursing, Toyama Prefectural University, Toyama, Toyama, Japan |
  | Journal Abbr | SAGE Open Med. |
  | ISSN | 2050-3121 |
  | Date Added | 05/02/2026, 17:53:30 |
  | Modified | 05/02/2026, 17:53:30 |

  ### Tags:

  - testosterone
  - article
  - female
  - male
  - nonhuman
  - human
  - hydrocortisone
  - adult
  - aged
  - controlled study
  - motivation
  - mental stress
  - clinical article
  - middle aged
  - animal assisted therapy
  - feeding
  - State Trait Anxiety Inventory
  - livestock
  - anxiety disorder
  - machine learning
  - schizophrenia
  - aripiprazole
  - controlled clinical trial
  - day care
  - levomepromazine
  - lithium carbonate
  - machine learning software
  - negative syndrome
  - olanzapine
  - risperidone
  - sheep rearing
  - UMIN000050224
  - valproic acid
  - zotepine

  ### Attachments

  - Full Text (HTML)
- ## Respiratory Sinus Arrhythmia Change during Trauma-Focused Cognitive-Behavioral Therapy: Results from a Randomized Controlled Feasibility Trial

  |  |  |
  | --- | --- |
  | Item Type | Journal Article |
  | Author | C.E. Shenk |
  | Author | B. Allen |
  | Author | N.A. Dreschel |
  | Author | M. Wang |
  | Author | J.M. Felt |
  | Author | M.P. Brown |
  | Author | A.M. Bucher |
  | Author | M.J. Chen |
  | Author | A.E. Olson |
  | Abstract | Trauma-Focused Cognitive-Behavioral Therapy (TF-CBT) is a well-established treatment for pediatric posttraumatic stress disorder (PTSD). Animal-assisted therapy (AAT) has been proposed as an adjunct to TF-CBT that may improve treatment effects through enhanced targeting of affect regulation, as indexed by specific changes in the respiratory sinus arrhythmia (RSA). The current study reports results from a randomized controlled feasibility trial (N = 33; Mage = 11.79 [SD = 3.08]; 64% White; 67% female) that measured RSA during Sessions 1, 4, 8, and 12 of a twelve-session TF-CBT protocol and tested whether: 1) TF-CBT + AAT achieved higher average RSA amplitudes relative to TF-CBT alone, and 2) RSA regulation, defined as less variability around person-specific RSA slopes during treatment, explained variation in post-treatment PTSD symptoms. Multilevel modeling failed to support an effect for TF-CBT + AAT on RSA amplitudes (δ001 = 0.08, p = 0.844). However, regardless of treatment condition, greater RSA withdrawal was observed within Sessions 4 (γ11 = -.01, p < .001) and 12 (γ13 = -.01, p = .015) relative to the Session 1 baseline. The average level of RSA amplitude in Session 8 was also significantly lower compared to Session 1 (γ02 = -0.70, p = .046). Intraindividual regression models demonstrated that greater RSA regulation predicted improved PTSD symptoms at post-treatment after adjusting for pre-treatment levels (b3 = 20.00, p = .012). These preliminary results offer support for future confirmatory trials testing whether affect regulation, as indexed by changes in RSA, is a mechanism of action for TF-CBT in the treatment of pediatric PTSD. |
  | Date | 2022 |
  | Language | English |
  | Archive | Medline |
  | URL | https://www.embase.com/search/results?subaction=viewrecord&id=L638222701&from=export |
  | Volume | 50 |
  | Pages | 1487-1499 |
  | Publication | Research on child and adolescent psychopathology |
  | DOI | 10.1007/s10802-022-00946-w |
  | Issue | 11 |
  | Journal Abbr | Res Child Adolesc Psychopathol |
  | ISSN | 2730-7174 |
  | Date Added | 05/02/2026, 17:53:31 |
  | Modified | 05/02/2026, 17:53:31 |

  ### Tags:

  - physiology
  - female
  - male
  - human
  - controlled study
  - posttraumatic stress disorder
  - randomized controlled trial
  - animal assisted therapy
  - procedures
  - cognitive behavioral therapy
  - feasibility study
  - respiratory sinus arrhythmia
  - sinus arrhythmia

  ### Attachments

  - Full Text (HTML)
- ## Acute salivary cortisol response in children with ADHD during psychosocial intervention with and without therapy dogs

  |  |  |
  | --- | --- |
  | Item Type | Journal Article |
  | Author | S.E.B. Schuck |
  | Author | C.N. Zeiler |
  | Author | A. Stehli |
  | Author | L.A. Steinhoff |
  | Author | R.Y. Stokes |
  | Author | S.E. Jeffrey |
  | Author | D.A. Granger |
  | Abstract | Introduction: Children with Attention Deficit/Hyperactivity Disorder (ADHD) participated in a randomized clinical trial comparing animal-assisted intervention (AAI) to psychosocial treatment as usual (TAU). This brief report describes effects of AAI on acute HPA axis reactivity and regulation. Saliva was collected before, during, and after psychosocial intervention sessions with and without therapy dogs and later assayed for cortisol (ug/dL). Methodology: Thirty-nine participants (n = 39) with ADHD, aged 7-9 years (79% male) provided saliva at 3 points during 90-minute sessions; (i) upon arrival, (ii) +20 minutes, and (iii) 15 minutes prior to departure, on 3 occasions across an 8-week intervention (weeks 1, 4, and 8). Cortisol slopes calculated within each session were compared across the intervention weeks to determine within subject and between group effect sizes. Spearman’s correlations between baseline individual neurodevelopmental symptoms and in-session acute cortisol responses were also evaluated. Results: No significant between group differences were observed in cortisol responsiveness at week-1. By week-4, in-session changes in cortisol were evident, with significantly greater decreases in the AAI group (Cohen’s d = -.40). This pattern was also observed at week-8, with an even stronger effect-size (d = -0.60). Concurrent symptoms of autism were associated with the in-session acute cortisol response. Specifically, higher parent-reported symptom scores were associated with steeper decreases in cortisol across the session at week 1 (r = -0.42, p <.01) and week-8 (r = -0.34 p =.05). At week-8 this association was stronger in the AAI group (r = -0.53) versus TAU (r = -0.25), with Cohen’s q = 0.413). Discussion: AAI may influence acute HPA reactivity and regulation for children with ADHD. Concurrent symptoms of ADHD and autism may be related to individual differences in the nature of the effect. Implications of these findings for AAI as an alternative, or complementary intervention for ADHD are discussed. Clinical trial registration: ClinicalTrials.gov, identifier NCT05102344. |
  | Date | 2024 |
  | Language | English |
  | Archive | Embase |
  | URL | https://www.embase.com/search/results?subaction=viewrecord&id=L2032157811&from=export |
  | Volume | 15 |
  | Publication | Frontiers in Psychiatry |
  | DOI | 10.3389/fpsyt.2024.1476522 |
  | Issue | (Schuck S.E.B., sabrina@uci.edu; Zeiler C.N.; Stehli A.; Steinhoff L.A.; Stokes R.Y.; Jeffrey S.E.) Pediatrics, University of California, Irvine, Irvine, CA, United States |
  | Journal Abbr | Front. Psychiatry |
  | ISSN | 1664-0640 |
  | Date Added | 05/02/2026, 17:53:22 |
  | Modified | 05/02/2026, 17:53:22 |

  ### Tags:

  - animal welfare
  - dog
  - saliva
  - child
  - article
  - coronavirus disease 2019
  - female
  - male
  - nonhuman
  - human
  - hydrocortisone
  - self concept
  - adult
  - pilot study
  - golden retriever
  - autism
  - middle aged
  - randomized controlled trial (topic)
  - attention deficit hyperactivity disorder
  - saliva collector
  - immunoassay
  - hyperactivity
  - schizophrenia
  - autism symptomatology
  - autoregulation
  - behavior counselor
  - cohort analysis
  - counselor
  - NCT05102344
  - psychosocial intervention
  - Salimetrics
  - symptomatology

  ### Attachments

  - Full Text (HTML)
- ## Human Enjoyment in Tactile Interaction With Horses and Dogs: A Comparative Study

  |  |  |
  | --- | --- |
  | Item Type | Journal Article |
  | Author | A. Sarrafchi |
  | Author | E. Lassallette |
  | Author | N. de Zwaan |
  | Author | M. Tucker |
  | Author | K. Merkies |
  | Abstract | Animal-assisted interventions (AAIs) are increasingly integrated into human healthcare due to their positive effects on mental and physical health. This study examined the impact of touch interactions on human physiological and emotional responses in two experiments with 10 horses and 18 dogs involved in AAI. Human heart rate (HR), heart rate variability (HRV), and positive emotional responses (assessed by the Positive Affect Scale (PAS) survey with three categories: activated, relaxed, and safe) were measured. The horse experiment also assessed how human experience with horses influenced these responses. Forty-nine participants interacted individually with horses, and 44 interacted individually with dogs under two conditions: forced touch (animals were restricted and required to interact) and consensual touch (animals were free to choose interaction). Human HR and HRV were recorded via heart rate monitors, and participants completed PAS surveys immediately after each interaction. Statistical analysis used a GLIMMIX model with repeated measures, and Pearson correlations examined human–horse HR relationships. During forced touch with horses, human HR was higher (p = 0.0001) and HRV lower (p = 0.0065) than during consensual touch. Experience with horses did not affect human HR (p = 0.3043) or HRV (p = 0.1366) but influenced PAS scores: the more experienced participants felt less "activated" (p = 0.0058) and the more "relaxed" (p = 0.0275) and "safe" (p = 0.0343). No significant correlation was found between human and horse HR (r = 0.09). In the dog study, touch treatment did not affect human HR (p = 0.2513), HRV (p = 0.1691), or PAS scores (all p > 0.0953). Descriptive results indicated that participants perceived dog interactions more positively than horse interactions. These findings suggest that allowing animals, especially horses, a choice to interact may reduce human physiological arousal during AAIs, with species-specific effects warranting further study. |
  | Date | 2025 |
  | Language | English |
  | Archive | Embase |
  | URL | https://www.embase.com/search/results?subaction=viewrecord&id=L2041400760&from=export |
  | Volume | 38 |
  | Pages | 1015-1031 |
  | Publication | Anthrozoos |
  | DOI | 10.1080/08927936.2025.2578073 |
  | Issue | 6 |
  | Journal Abbr | Anthrozoos |
  | ISSN | 1753-0377 |
  | Date Added | 05/02/2026, 17:53:15 |
  | Modified | 05/02/2026, 17:53:15 |

  ### Tags:

  - human-animal interaction
  - horse
  - heart rate variability
  - heart rate
  - dog
  - emotion
  - article
  - female
  - male
  - nonhuman
  - human
  - adult
  - controlled study
  - wellbeing
  - human-animal bond
  - arousal
  - comparative study

  ### Attachments

  - Full Text (HTML)
- ## Animal Assisted Activities (AAAs) with Dogs in a Dialysis Center in Southern Italy: Evaluation of Serotonin and Oxytocin Values in Involved Patients

  |  |  |
  | --- | --- |
  | Item Type | Journal Article |
  | Author | A. Santaniello |
  | Author | G. Perruolo |
  | Author | A. Amato |
  | Author | S. Garzillo |
  | Author | F. Mormone |
  | Author | C. Morelli |
  | Author | P. Formisano |
  | Author | M. Sansone |
  | Author | A. Fioretti |
  | Author | F. Oriente |
  | Abstract | Background/Objectives: In the present study, the changes in oxytocin (OXT) and serotonin (5-HT), as hormones involved in social relationships and mood regulation, respectively, were measured in dialysis patients involved in Animal Assisted Activity (AAA) interventions. Methods: Thirty patients (15 men and 15 women) with chronic kidney disease, undergoing hemodialysis three times per week, for 4 h, were enrolled. The patients were divided into three groups: two experimental groups who received the AAA intervention and a control group that never received the AAA intervention. A specific dog-zootherapist vet pair was assigned for each experimental group. All sessions of the two experimental groups were performed weekly, for a total period of 3 months (12 sessions). Blood samples were collected at the beginning and end of each session (T0 and T1), lasting about one hour. The interaction time with the dog was approximately 40 min. The samples were then analyzed to measure the levels of oxytocin and serotonin and processed using analysis of variance with mixed effects models. Results: The results obtained showed that both dog-zootherapist vet dyads caused a statistically significant overall effect of both oxytocin and serotonin, increasing during the sessions, compared to the control group. In addition, it was observed progressively increasing effect between two consecutive weeks. Conclusions: The results from this study showed that the AAA represents a positive stimulus for patients on dialysis. Thus, our study suggests that structured AAA intervention in a hemodialysis center can improve patients’ quality of life during the dialysis cycle. |
  | Date | 2025 |
  | Language | English |
  | Archive | Embase |
  | URL | https://www.embase.com/search/results?subaction=viewrecord&id=L2042135810&from=export |
  | Volume | 13 |
  | Publication | Biomedicines |
  | DOI | 10.3390/biomedicines13122944 |
  | Issue | 12 |
  | Journal Abbr | Biomedicines |
  | ISSN | 2227-9059 |
  | Date Added | 05/02/2026, 17:53:07 |
  | Modified | 05/02/2026, 17:53:07 |

  ### Tags:

  - oxytocin
  - dog
  - anxiety
  - article
  - female
  - male
  - social interaction
  - human
  - adult
  - blood sampling
  - controlled study
  - data analysis software
  - ELISA kit
  - randomized controlled trial
  - creatinine
  - quality of life
  - clinical article
  - middle aged
  - alkaline phosphatase
  - enzyme linked immunosorbent assay
  - animal assisted therapy
  - Labrador retriever
  - chronic kidney failure
  - hemodialysis
  - serotonin
  - urea nitrogen blood level
  - animal assisted activity
  - Italy
  - chlorhexidine
  - dialysis
  - gluconate zinc
  - glycerol
  - R ver. 4.4.3
  - Triturus semi-automated ELISA processor
  - Triturus software ver. 4.01b

  ### Attachments

  - Full Text (HTML)
- ## “No SMA can hold”: Nursing care for children with spinal muscular atrophy. Descriptive analysis of two case studies

  |  |  |
  | --- | --- |
  | Item Type | Journal Article |
  | Author | F. Ruta |
  | Author | P. Ferrara |
  | Author | F. Dal Mas |
  | Abstract | Background: Spinal muscular atrophy (SMA) is one of the most common genetic causes of death in children affecting about one in 10,000 live births, while its prevalence is about 1–2 per 100,000 (live births). Recently, the European Commission (EU) approved a novel gene therapy based on the onasemnogen abeparvovec (Zolgensma) for the treatment of patients with SMA. In addition to drug treatment, it is essential that children with SMA apply self-care methods to maintain their health, monitor their weight and food intake, and use appropriate remedies. Self-care and co-production of health care services are crucial in the modern ecosystem, as they can improve survival and prevent hospitalizations. The aim of this work is to support healthcare professionals who may have to deal with patients affected by this disease. Methods: The article uses two case studies of children with spinal muscular atrophy through the creation of a multi-professional research group composed of health professionals who provide direct care to SMA children. The collection and analysis of the data were carried out by involving different figures who interact with SMA children. Specifically, physicians, nurses, parents, physical therapists, social workers, and teachers were individually interviewed. Results: The study aims to provide suggestions on assessing child self-care, believing it to be a valuable method to gather information on how the child performs daily activities and how much the surrounding environment affects self-care. This paper highlights how self-management behaviors depend on four basic aspects: the person (individual, cognitive, and social perceptions), the patient's family (level of knowledge of pathology, involvement in the management and quality of relationship with the patient), the community (relationships with external social contexts, such as school and other organizations), and the healthcare system (availability of resources and the degree of evolution of healthcare). Conclusions: The experience conducted may be helpful to other health institutions to make the approach to children with SMA as most effective as possible, creating internal workgroups and collaboration with external experts on the subject. Moreover, it provides valuable information on caring for families with children with SMA. |
  | Date | 2024 |
  | Language | English |
  | Archive | Embase |
  | URL | https://www.embase.com/search/results?subaction=viewrecord&id=L2022438682&from=export |
  | Volume | 60 |
  | Publication | Revista Cientifica de la Sociedad Espanola de Enfermeria Neurologica |
  | DOI | 10.1016/j.sedene.2022.11.002 |
  | Issue | (Ruta F., federicorutabat@gmail.com) General Direction, ASL BAT (Health Agency), Andria, Italy |
  | Journal Abbr | Rev. Cient. Soc. Esp. Enferm. Neurol. |
  | ISSN | 2013-5246 |
  | Date Added | 05/02/2026, 17:53:18 |
  | Modified | 05/02/2026, 17:53:18 |

  ### Tags:

  - child
  - article
  - female
  - human
  - hippotherapy
  - occupational therapy
  - motor performance
  - physiotherapist
  - spinal muscular atrophy
  - clinical article
  - knowledge
  - health care system
  - health service
  - gene mutation
  - survival
  - case report
  - parent
  - breathing exercise
  - artificial ventilation
  - bilevel positive airway pressure
  - chronic respiratory failure
  - exon
  - gastroesophageal reflux
  - genetic counseling
  - health care quality
  - hydrotherapy
  - lung infection
  - medical research
  - muscle hypotonia
  - nurse
  - nursing care
  - physician
  - self care
  - social environment
  - social worker
  - speech therapy
  - teacher
  - tracheostomy
  - vaccine

  ### Attachments

  - Full Text (HTML)
- ## A Pre-Screening Tool to Assess Dog Suitability for Animal-Assisted Interventions: Preliminary Results for Dog-Suitability Tests (SuiTe)

  |  |  |
  | --- | --- |
  | Item Type | Journal Article |
  | Author | G. Russo |
  | Author | C. Borrelli |
  | Author | G. Riggio |
  | Author | E. Rosson |
  | Author | M. Bentivoglio |
  | Author | C. Mariti |
  | Abstract | Animal-assisted interventions (AAIs) or Services (AAS) may cause stress in participating dogs, making the selection of suitable individuals essential to prevent strain. Different non-standardized approaches currently exist to assess dogs’ suitability for AAIs. This preliminary study aimed at evaluating two combined tools, a behavioural aptitude test (SuiTe) and an ad hoc revised questionnaire incorporating C-BARQ, for pre-screening dog suitability for AAIs, also in relation to salivary cortisol measured by enzyme immunoassay in N = 38 dogs. Dogs’ behavioural responses to environmental and social stimuli were scored on an X-Y scale and classified by two independent evaluators as suitable (S), pending suitability (P), or unsuitable (U). Non-parametric tests were performed (p < 0.05). Results indicated significant differences between dogs classified as S or P versus U, both in SuiTe valence scores (higher in S and P) and in separation, attachment, and fear/anxiety behaviours assessed by the questionnaire (higher in U). However, suitability in the SuiTe was lower than that assessed by caregivers through an open question. Our study highlights the complexity of this assessment and the limited awareness of caregivers regarding the situations their dogs face every day. Future analyses will refine this multiparametric approach within a One Welfare perspective, ensuring the welfare of both animals and humans involved in AAIs. |
  | Date | 2025 |
  | Language | English |
  | Archive | Embase |
  | URL | https://www.embase.com/search/results?subaction=viewrecord&id=L2042119198&from=export |
  | Volume | 12 |
  | Publication | Veterinary Sciences |
  | DOI | 10.3390/vetsci12121110 |
  | Issue | 12 |
  | Journal Abbr | Vet. Sci. |
  | ISSN | 2306-7381 |
  | Date Added | 05/02/2026, 17:53:06 |
  | Modified | 05/02/2026, 17:53:06 |

  ### Tags:

  - dog
  - animal-assisted intervention
  - animal experiment
  - article
  - female
  - male
  - nonhuman
  - controlled study
  - data analysis software
  - questionnaire
  - caregiver
  - training
  - qualitative analysis
  - saliva collector
  - Salivette
  - enzyme immunoassay
  - adhesive tape
  - aptitude test
  - behavioural aptitude test
  - cortisol test kit
  - intervention study
  - RStudio software Version 2024.12.0+467
  - Salimetrics Cortisol Enzyme Immunoassay Kit

  ### Attachments

  - Full Text (HTML)
- ## Urinary oxytocin levels in children meeting a Hospital Dog®

  |  |  |
  | --- | --- |
  | Item Type | Journal Article |
  | Author | A. Risberg |
  | Author | A. Larsson |
  | Author | U. Bodén |
  | Author | A. Edner |
  | Abstract | Abstract: There has been growing interest in animal-assisted therapy (AAT) in recent decades due to increasing reports indicating its health benefits for adult patients. These benefits are partly attributed to changes, usually increased levels of the neuropeptide oxytocin. Aim: To investigate changes in oxytocin levels in hospitalized children meeting a certified Hospital Dog®. Method: Urine samples were collected between 25/02/2016 and 24/05/2017 from 35 hospitalized children (3–17 years) before and after each participant had a session with the Hospital Dog®. Oxytocin levels were analysed with an acetylcholinesterase (AChE) competitive enzyme-linked immunosorbent assay (ELISA). Creatinine levels were measured to determine the subject’s fluid intake and then divided by the hormonal concentration (uOT pg/ml). Results: The mean level of uOT was 186.0 ± 236.7 (51.5–1349.5) pg/ml before and 137.3 ± 121.5 (30.7–591.3) pg/ml after the dog session (p = 0.010). Conclusion: Decreased levels of uOT were recorded during the study in which hospitalized children met a Hospital Dog®. The decreased OT levels are potentially the result of the intense activity the subject experienced with the dog during the interaction. |
  | Date | 2025 |
  | Language | English |
  | Archive | Embase |
  | URL | https://www.embase.com/search/results?subaction=viewrecord&id=L2036016695&from=export |
  | Volume | 25 |
  | Publication | BMC Complementary Medicine and Therapies |
  | DOI | 10.1186/s12906-025-05076-6 |
  | Issue | 1 |
  | Journal Abbr | BMC Compl. Med.Therapies |
  | ISSN | 2662-7671 |
  | Date Added | 05/02/2026, 17:45:13 |
  | Modified | 05/02/2026, 17:45:13 |

  ### Tags:

  - oxytocin
  - dog
  - anxiety
  - child
  - article
  - female
  - male
  - physiological stress
  - human
  - hydrocortisone
  - adult
  - length of stay
  - questionnaire
  - adolescent
  - major clinical study
  - enzyme linked immunosorbent assay
  - disease severity
  - hospital

  ### Attachments

  - Full Text (HTML)
- ## Behavioral and physiological responses of horses to ground-based adaptive horsemanship lessons for veterans with post-traumatic stress disorder (PTSD)

  |  |  |
  | --- | --- |
  | Item Type | Journal Article |
  | Author | E.M. Rankins |
  | Author | K.H. McKeever |
  | Author | K. Malinowski |
  | Abstract | Little literature exists on horses in adaptive horsemanship (AH) despite concerns about their well-being. The study objective was to evaluate behavioral and physiological responses of horses to ground-based AH lessons for veterans with post-traumatic stress disorder (PTSD). Lessons were expected to alter horses’ hormone concentrations, behavior, and muscle activity. Geldings were assigned to AH (n=6; 20.3 ± 1.9 yrs., mean ± SE) or control (CON; stall in arena, n=6; 13.8 ± 1.7 yrs.) conditions for 8-week sessions based on current occupation (AH = equine-assisted services; CON = recreational riding). Plasma cortisol, epinephrine, norepinephrine, and oxytocin concentrations from samples at 0 (start of lesson), 3, 5, 25, and 30 (end) min were determined using assays validated in horses. Surface electromyography (sEMG) (masseter and brachiocephalic; Noraxon, Scottsdale, AZ, USA) and video were recorded continuously. Average rectified values (ARV) and median frequency (MF) were calculated (100 ms) after sEMG data were normalized, rectified, and filtered. The number, number of unique, and duration of stress related behaviors (ethogram) were recorded by three trained (ĸ ≥ 0.7) observers. Data were analyzed with repeated measures ANOVAs (significance P ≤ 0.05) with fixed effects of treatment, time point, week, and their interactions as appropriate and random effect of horse. CON horses had elevated cortisol concentrations (P = 0.0023) at 25 and 30 min. AH horses displayed fewer (P ≤ 0.0491) stress related and unique behaviors. CON horses were described as more (P < 0.0001) anxious, nervous, and stressed than AH horses (calm, comfortable, patient, and relaxed) in qualitative behavior analysis (22 observers). AH horses were less stressed than CON horses. |
  | Date | 2024 |
  | Language | English |
  | Archive | Embase |
  | URL | https://www.embase.com/search/results?subaction=viewrecord&id=L2031199778&from=export |
  | Volume | 135 |
  | Publication | Journal of Equine Veterinary Science |
  | DOI | 10.1016/j.jevs.2024.105049 |
  | Issue | (Rankins E.M., ellen.rankins@colostate.edu; McKeever K.H., mckeever@sebs.rutgers.edu; Malinowski K., karynmal@njaes.rutgers.edu) Equine Science Center, Department of Animal Sciences, Rutgers, The State University of New Jersey, New Brunswick, NJ, United States |
  | Journal Abbr | J. Equine Vet. Sci. |
  | ISSN | 0737-0806 |
  | Date Added | 05/02/2026, 17:53:20 |
  | Modified | 05/02/2026, 17:53:20 |

  ### Tags:

  - oxytocin
  - horse
  - veteran
  - animal behavior
  - animal experiment
  - article
  - male
  - nonhuman
  - physiological stress
  - human
  - hydrocortisone
  - adult
  - blood sampling
  - controlled study
  - epinephrine
  - hippotherapy
  - muscle contraction
  - noradrenalin
  - posttraumatic stress disorder
  - surface electromyography
  - Ultium EMG
  - behavioral observation
  - behavior assessment
  - videorecording
  - clinical article
  - enzyme linked immunosorbent assay
  - qualitative analysis
  - adrenalin blood level
  - hydrocortisone blood level
  - noradrenalin blood level
  - mental health
  - camera
  - oxytocin blood level
  - gelding
  - 3 Hero7
  - brachiocephalic trunk
  - electromyograph electrode
  - impedance analysis
  - masseter muscle
  - military service
  - Ninox 300C
  - Quarter horse
  - Standardbred horse
  - Thoroughbred horse

  ### Attachments

  - Full Text (HTML)
- ## Ground-based adaptive horsemanship lessons for veterans with post-traumatic stress disorder: a randomized controlled pilot study

  |  |  |
  | --- | --- |
  | Item Type | Journal Article |
  | Author | E.M. Rankins |
  | Author | A. Quinn |
  | Author | K.H. McKeever |
  | Author | K. Malinowski |
  | Abstract | Introduction: Equine-assisted services (EAS) has received attention as a potential treatment strategy for post-traumatic stress disorder (PTSD), as existing literature indicates that symptoms may decrease following EAS. Relatively little is known about the mechanisms at play during lessons and if physiological measures are impacted. The objectives of this pilot study were to 1) explore the effects of adaptive horsemanship (AH) lessons on symptoms of PTSD, hormone concentrations, and social motor synchrony; 2) determine if physiological changes occur as veterans interact with horses; and 3) explore if the interaction between veteran and horse changes over the 8-week session. Methods: Veterans with PTSD were randomly assigned to control (CON, n = 3) or AH (n = 6) groups for an 8-week period (clinical trial; NCT04850573; clinicaltrials.gov). Veterans completed the PTSD Checklist (PCL-5) and Brief Symptom Inventory (BSI) at pre-, post-, and 2- and 6-month follow-up time points. They also completed a social motor synchrony test (pendulum swinging) and blood draw at pre- and post-time points. In weeks 1, 4, and 8, blood samples were drawn at 0 min, 3 min, 5 min, 25 min, and 30 min during the 30-min AH lessons. Veterans completed the Human-Animal Interaction Scale (HAIS) after each lesson. Blood samples were assayed for plasma cortisol, epinephrine, norepinephrine, and oxytocin. Data were analyzed with repeated measure ANOVAs. Changes in PTSD symptoms from pre- to post-time point were analyzed with paired t-tests. Results: Changes in PCL-5 scores tended to differ (p = 0.0989), and global BSI scores differed (p = 0.0266) between AH (−11.5 ± 5.5, mean ± SE; −0.5 ± 0.2) and CON (5.3 ± 5.4; 0.4 ± 0.2) groups. Social motor synchrony and hormone concentrations did not differ between groups or time points (p > 0.05). Cortisol, norepinephrine, and oxytocin concentrations did not differ across sessions (p > 0.05). Epinephrine concentrations tended (p = 0.0744) to decrease from week 1 to 4 of sessions. HAIS scores increased (p ≥ 0.0437) in week 3 and remained elevated as compared to week 1. Discussion: Participant recruitment was the greatest challenge. These preliminary results agree with the literature suggesting that EAS can reduce symptoms of PTSD. |
  | Date | 2024 |
  | Language | English |
  | Archive | Embase |
  | URL | https://www.embase.com/search/results?subaction=viewrecord&id=L2030160775&from=export |
  | Volume | 15 |
  | Publication | Frontiers in Psychiatry |
  | DOI | 10.3389/fpsyt.2024.1390212 |
  | Issue | (Rankins E.M., ellen.rankins@colostate.edu; McKeever K.H.; Malinowski K.) Equine Science Center, Department of Animal Sciences, Rutgers University, New Brunswick, NJ, United States |
  | Journal Abbr | Front. Psychiatry |
  | ISSN | 1664-0640 |
  | Date Added | 05/02/2026, 17:53:22 |
  | Modified | 05/02/2026, 17:53:22 |

  ### Tags:

  - oxytocin
  - human-animal interaction
  - veteran
  - animal behavior
  - article
  - female
  - male
  - human
  - hydrocortisone
  - 9.4
  - adaptive behavior
  - adaptive horsemanship
  - adult
  - aged
  - Autoguard
  - bioinformatics software
  - biomedical software
  - blood collection tube
  - blood sampling
  - body weight
  - Brief Symptom Inventory
  - checklist
  - competitive ELISA
  - computer
  - controlled study
  - data analysis software
  - electromyograph
  - ELISA kit
  - epinephrine
  - Excel
  - feedback system
  - follow up
  - Fourier transform
  - grooming
  - gyroscope sensor
  - hippotherapy
  - hormone determination
  - horseback riding
  - Human Animal Interaction Scale
  - Insyte
  - intravenous catheter
  - iPad mini 2 OS v12.5.4
  - Life Events Checklist 5
  - MARS v 3.20
  - MATLAB 2022b
  - Mobile Precision 3541
  - muscle contraction
  - myoMUSCLE
  - NCT04850573
  - noradrenalin
  - occupational therapy
  - physiotherapy
  - pilot study
  - post traumatic stress disorder checklist 5
  - posttraumatic stress disorder
  - randomized controlled trial
  - range of vision
  - scoring system
  - silver electrode
  - social motor synchrony
  - social synchronization
  - surface electromyography
  - tablet computer
  - Ultium EMG
  - Vacutainer 23 GA
  - vein puncture
  - visual system parameters

  ### Attachments

  - Full Text (HTML)
- ## Comparison of contingent and noncontingent access to therapy dogs during academic tasks in children with autism spectrum disorder

  |  |  |
  | --- | --- |
  | Item Type | Journal Article |
  | Author | A. Protopopova |
  | Author | A.L. Matter |
  | Author | B.N. Harris |
  | Author | K.M. Wiskow |
  | Author | J.M. Donaldson |
  | Abstract | This study compared contingent and noncontingent access to therapy dogs during educational tasks for children with autism spectrum disorder using a multielement design. The experimenters assessed whether initial preference for the dog predicted reinforcer efficacy and how preference changed across time. A higher response rate during contingent dog sessions than baseline sessions occurred for 4 out of 5 participants, suggesting that the dog functioned as a reinforcer. One participant engaged in a high rate of responding in both contingent and noncontingent dog conditions. Preference assessments revealed idiosyncrasies, suggesting that further research is needed into the predictive nature of initial preference assessments with animals as part of the stimulus array. The experimenters also analyzed salivary cortisol before and after sessions to determine if learning about the upcoming interaction with a dog reduced salivary cortisol in children. Cortisol was variable across participants, with only some deriving a potential physiological benefit from expecting to interact with the dog. |
  | Date | 2020 |
  | Language | English |
  | Archive | Medline |
  | URL | https://www.embase.com/search/results?subaction=viewrecord&id=L629026419&from=export |
  | Volume | 53 |
  | Pages | 811-834 |
  | Publication | Journal of applied behavior analysis |
  | DOI | 10.1002/jaba.619 |
  | Issue | 2 |
  | Journal Abbr | J Appl Behav Anal |
  | ISSN | 1938-3703 |
  | Date Added | 05/02/2026, 17:58:57 |
  | Modified | 05/02/2026, 17:58:57 |

  ### Tags:

  - dog
  - saliva
  - animal experiment
  - article
  - female
  - male
  - nonhuman
  - hydrocortisone
  - controlled study
  - animal model
  - autism
  - learning
  - comparative effectiveness
  - drug efficacy
  - stimulus

  ### Attachments

  - Full Text (HTML)
- ## Evaluation of stress markers in horses during hippotherapy sessions in comparison to being ridden by beginners

  |  |  |
  | --- | --- |
  | Item Type | Journal Article |
  | Author | J.F.N. Potier |
  | Author | V. Louzier |
  | Abstract | Hippotherapy has been used for decades and its benefits to human patients have largely been proven, whether being applied to those with physical or mental disabilities. There have been a plethora of animal welfare studies recently, pertaining especially to ridden horses. This study aimed to investigate stress markers in horses during hippotherapy sessions to address the ethical considerations raised by using horses for therapy. A ridden stress ethogram was established and validated specifically for this study via subjective observation and video recording of a ridden session involving intermediate-level riders. The experiment entailed eight healthy horses undergoing two ridden sessions on separate days, one with disabled riders and one with beginners. Several parameters associated with physiological responses to stress were evaluated at rest, such as heart rate, plasma adrenocorticotropic hormone [ACTH], serum and salivary cortisol. These parameters as well as the behavioural stress score from the ethogram scale were measured during both sessions. No significant differences were found between heart rate, plasma ACTH, and stress scores. Serum and salivary cortisol were significantly lower during the hippotherapy session than during the session with beginners. The current study found no evidence of compromised welfare when horses were used as a therapeutic aid during hippotherapy sessions compared to their usual ridden activity. Although these results indicate that hippotherapy may be ethically justified as it benefits humans without causing harm to the horses, the present study was small, and the results should be interpreted with caution. |
  | Date | 2023 |
  | Language | English |
  | Archive | Embase |
  | URL | https://www.embase.com/search/results?subaction=viewrecord&id=L2022751646&from=export |
  | Volume | 32 |
  | Publication | Animal Welfare |
  | DOI | 10.1017/awf.2023.6 |
  | Issue | (Potier J.F.N., julie.potier@theleh.co.uk) Liphook Equine Hospital, Hampshire, United Kingdom |
  | Journal Abbr | Anim. Welf. |
  | ISSN | 0962-7286 |
  | Date Added | 05/02/2026, 17:53:29 |
  | Modified | 05/02/2026, 17:53:29 |

  ### Tags:

  - horse
  - animal welfare
  - heart rate
  - animal experiment
  - article
  - female
  - male
  - nonhuman
  - human
  - hydrocortisone
  - adult
  - controlled study
  - hippotherapy
  - horseback riding
  - corticotropin
  - videorecording
  - electrocardiogram
  - smartphone
  - hydrocortisone blood level
  - saliva collector
  - behavioral stress
  - corticotropin blood level
  - disabled person
  - evaluation study
  - medical ethics
  - rest

  ### Attachments

  - Full Text (HTML)
- ## Clinical EEG of Rett Syndrome: Group Analysis Supplemented with Longitudinal Case Report

  |  |  |
  | --- | --- |
  | Item Type | Journal Article |
  | Author | G. Portnova |
  | Author | A. Neklyudova |
  | Author | V. Voinova |
  | Author | O. Sysoeva |
  | Abstract | Rett syndrome (RTT), a severe neurodevelopmental disorder caused by MECP2 gene abnormalities, is characterized by atypical EEG activity, and its detailed examination is lacking. We combined the comparison of one-time eyes open EEG resting state activity from 32 girls with RTT and their 41 typically developing peers (age 2–16 years old) with longitudinal following of one girl with RTT to reveal EEG parameters which correspond to the RTT progression. Traditional measures, such as epileptiform abnormalities, generalized background activity, beta activity and the sensorimotor rhythm, were supplemented by a new frequency rate index measured as the ratio between high- and low-frequency power of sensorimotor rhythm. Almost all studied EEG parameters differentiated the groups; however, only the elevated generalized background slowing and decrease in our newly introduced frequency rate index which reflects attenuation in the proportion of the upper band of sensorimotor rhythm in RTT showed significant relation with RTT progression both in longitudinal case and group analysis. Moreover, only this novel index was linked to the breathing irregularities RTT symptom. The percentage of epileptiform activity was unrelated to RTT severity, confirming previous studies. Thus, resting EEG can provide information about the pathophysiological changes caused by MECP2 abnormalities and disease progression. |
  | Date | 2022 |
  | Language | English |
  | Archive | Embase |
  | URL | https://www.embase.com/search/results?subaction=viewrecord&id=L2020730682&from=export |
  | Volume | 12 |
  | Publication | Journal of Personalized Medicine |
  | DOI | 10.3390/jpm12121973 |
  | Issue | 12 |
  | Journal Abbr | J. Pers. Med. |
  | ISSN | 2075-4426 |
  | Date Added | 05/02/2026, 17:53:30 |
  | Modified | 05/02/2026, 17:53:30 |

  ### Tags:

  - physical activity
  - article
  - female
  - play
  - human
  - hippotherapy
  - physiotherapy
  - assessment of humans
  - clinical trial (topic)
  - electroencephalogram
  - beta rhythm
  - facial expression
  - massage
  - newborn
  - motor performance
  - clinical article
  - fear
  - sitting
  - standing
  - feeding
  - disease severity
  - socialization
  - pathologic nystagmus
  - motor activity
  - speech
  - hypersalivation
  - pregnancy
  - cognitive development
  - human activities
  - case report
  - speech therapy
  - disease exacerbation
  - active babbling
  - Apgar score
  - atypical absences
  - Bayley Scales of Infant Development
  - belly
  - birth length
  - birth weight
  - clear episodes of breath holding
  - clinical feature
  - clinical observation
  - commands
  - crawling
  - daily living
  - delay in expressive and receptive language development
  - delayed motor development
  - displeasure sounds
  - drug dose increase
  - echography
  - epileptic electroencephalogram abnormality
  - epileptiform activity
  - epileptiform discharges
  - episodes of shared attention
  - eye contact
  - freezing of gait
  - frequent mouthing of the hands
  - functional play
  - general slowing
  - grimaces
  - hand movement
  - hand soaking
  - hypoventilation
  - lamotrigine
  - laughter
  - mild developmental regression
  - mouth examination
  - muscle weakness
  - NCT02790034
  - nervous system parameters
  - opsoclonus
  - over all delay in all gross motor skills
  - peabody developmental motor scale 2
  - pincer grasp
  - pleasure sounds
  - polyspike waves
  - poor saliva control
  - preschool language scale
  - remission
  - representational gesture
  - Rett syndrome
  - sarizotan
  - scoliosis
  - sensorimotor rhythm
  - sit independently
  - slight hypotonia
  - stand with support
  - sundowning of the eyes
  - swimming
  - trembling at awakenings
  - very limited ways of communication
  - Vineland Adaptive Behavior Scale
  - voluntary movement
  - walk with support

  ### Attachments

  - Full Text (HTML)
- ## Effects of human and animal-assisted skills training on oxytocin und cortisol levels in patients with borderline personality disorder

  |  |  |
  | --- | --- |
  | Item Type | Journal Article |
  | Author | O. Plett |
  | Author | V. Flasbeck |
  | Author | M. Brüne |
  | Abstract | Objective: Borderline Personality Disorder (BPD) is characterised, among other symptoms, by emotional instability and difficulties in regulating proximity to significant others. Many with BPD have difficulties in establishing a trustful therapeutic relationship, which often develop before a background of adverse childhood experiences with caregivers. One way to facilitate therapeutic interaction in psychotherapy incorporates pet animals as “door openers”. No study exists, however, that has examined the effect of animal-assisted versus human-guided skills training on neurobiological correlates of affiliation and stress regulation, i.e. oxytocin and cortisol. Methods: Twenty in-patients diagnosed with BPD were recruited to participate in an animal-assisted skills-training. Another 20 in-patients participated in a human-guided skills-training. Salivary samples of both groups were taken for determining oxytocin and cortisol before and immediately after 3 therapeutic sessions at least one week apart from one another. In addition, borderline symptom severity (BSL-23), impulsivity (BIS-15), alexithymia (TAS-20), and fear of compassion (FOCS) were determined by self-rating questionnaires before and after the 6-week interventions. Results: Both therapeutic interventions led to a significant reduction in cortisol and an (non-significant) increase in oxytocin, respectively. Importantly, there was a statistically significant interaction between changes in cortisol and oxytocin, independent of group. Both groups further showed clinical improvement as measured using the above-listed questionnaires. Conclusion: Our findings suggest that both animal-assisted and human-guided interventions have measurable short-term effects on affiliative and stress hormones, with no approach being superior to the other in this regard. |
  | Date | 2023 |
  | Language | English |
  | Archive | Embase |
  | URL | https://www.embase.com/search/results?subaction=viewrecord&id=L2024293435&from=export |
  | Volume | 162 |
  | Pages | 156-160 |
  | Publication | Journal of Psychiatric Research |
  | DOI | 10.1016/j.jpsychires.2023.05.004 |
  | Issue | (Plett O.; Flasbeck V.; Brüne M., martin.bruene@rub.de) LWL University Hospital Bochum, Division of Social Neuropsychiatry and Evolutionary Medicine, Ruhr University, NRW, Bochum, Germany |
  | Journal Abbr | J. Psychiatr. Res. |
  | ISSN | 1879-1379 |
  | Date Added | 05/02/2026, 17:58:43 |
  | Modified | 05/02/2026, 17:58:43 |

  ### Tags:

  - oxytocin
  - saliva
  - article
  - human
  - hydrocortisone
  - self report
  - controlled study
  - hospitalization
  - questionnaire
  - clinical article
  - animal assisted therapy
  - hydrocortisone blood level
  - therapy effect
  - sample size
  - disease severity
  - impulsiveness
  - oxytocin blood level
  - alexithymia
  - antidepressant agent
  - Barratt Impulsiveness Scale
  - borderline state
  - comorbidity
  - general condition improvement
  - human guided skill training
  - medical education
  - methylphenidate
  - neuroleptic agent
  - sample
  - self compassion
  - Toronto Alexithymia scale

  ### Attachments

  - Full Text (HTML)
- ## Dog-assisted therapy for control of anxiety in pediatric dentistry

  |  |  |
  | --- | --- |
  | Item Type | Journal Article |
  | Author | S.L. Pinheiro |
  | Author | C. Silva |
  | Author | L. Luiz |
  | Author | N. Silva |
  | Author | R. Fonseca |
  | Author | T. Velásquez |
  | Author | D.R. Grandizoli |
  | Abstract | Anxiety is common in pediatric dental care, and affects the behavioral management of children. Animal-assisted therapy (AAT) has been shown to improve children's behavior. However, few studies have applied this technique in dentistry. The aim of the present study was to evaluate the applicability of dog-assisted therapy to control anxiety during pediatric dental treatment. Twenty children were selected from the Pediatric Dentistry Clinic of the Pontifical Catholic University of Campinas (PUC-Campinas), Brazil. Participants were divided into two groups: Control (n = 11; visits = 16), in which children were conditioned by methods routinely used in the clinic; and AAT (n = 9; visits = 23), in which children had contact with a dog therapist first at the reception desk and then again inside the office. The dog therapist stayed beside the dental chair with the child throughout the procedures. Corah's Dental Anxiety Scale (CS) and heart rate (HR) were used for evaluation of child anxiety. The results were tested for normality of distribution with the Shapiro-Wilk method, and subsequently analyzed in BioEstat 5.0. HR results were compared by Analysis of Variance (ANOVA) with Tukey's test, and CS scores, with the Wilcoxon test. There was a significant reduction in HR in the AAT group (p = 0.0069). In the Control group, HR did not change before, during, or after treatment (p = 0.6052). Controls showed a significant increase in anxiety measured by CS before and after treatment (p = 0.0455). In the AAT group, there was no change in CS scores before and after treatment (p = 0.3739). AAT could be an alternative to reduce anxiety during pediatric dental care. |
  | Date | 2023 |
  | Language | English |
  | Archive | Medline |
  | URL | https://www.embase.com/search/results?subaction=viewrecord&id=L642825196&from=export |
  | Volume | 47 |
  | Pages | 38-43 |
  | Publication | The Journal of clinical pediatric dentistry |
  | DOI | 10.22514/jocpd.2023.080 |
  | Issue | 6 |
  | Journal Abbr | J Clin Pediatr Dent |
  | ISSN | 1053-4628 |
  | Date Added | 05/02/2026, 17:53:25 |
  | Modified | 05/02/2026, 17:53:25 |

  ### Tags:

  - dog
  - anxiety
  - child
  - human
  - animal
  - animal assisted therapy
  - Brazil
  - pediatric dentistry
  - dental anxiety
  - prevention and control

  ### Attachments

  - Full Text (HTML)
- ## Heart Rate Variability Spectral Analysis for Monitoring Autonomic Activation in a Donkey Involved in Animal-Assisted Therapy: A Single Subject Design During Animal-Assisted Therapy Sessions

  |  |  |
  | --- | --- |
  | Item Type | Journal Article |
  | Author | M. Panzera |
  | Author | A. Statelli |
  | Abstract | Background: Only a limited number of studies have investigated objective indicators to assess donkey welfare during Animal-Assisted Services. Objective: The present research follows a single-subject design and its objective is to evaluate the neurovegetative indicators of the well-being of a donkey through spectral analysis of the R-R signal in the frequency domain. Methods: The experimental protocol of the Animal-Assisted Therapy project involved one donkey, previously selected through behavioral protocol evaluation, and ten patients with a diagnosis of paranoid schizophrenia. Spectral analysis of the R-R signal in the frequency domain was performed, providing objective data on the activity of the sympathetic and parasympathetic nervous systems of the donkey (before, during, and after the sessions). Results: The significance of the variations, both statistically significant and not, supports the hypothesis that the affiliative human–donkey interaction within the context of AAS is associated with modifications in the neurovegetative components of the donkey involved in AAT. Conclusions: These findings highlight the importance of objective and non-invasive monitoring tools to detect early signs of discomfort in donkeys involved in AAT, supporting the development of selection and management strategies that safeguard animal welfare. |
  | Date | 2025 |
  | Language | English |
  | Archive | Embase |
  | URL | https://www.embase.com/search/results?subaction=viewrecord&id=L2042119207&from=export |
  | Volume | 12 |
  | Publication | Veterinary Sciences |
  | DOI | 10.3390/vetsci12121131 |
  | Issue | 12 |
  | Journal Abbr | Vet. Sci. |
  | ISSN | 2306-7381 |
  | Date Added | 05/02/2026, 17:53:07 |
  | Modified | 05/02/2026, 17:53:07 |

  ### Tags:

  - human-animal interaction
  - animal welfare
  - heart rate variability
  - behavior
  - heart rate
  - animal experiment
  - article
  - female
  - male
  - nonhuman
  - human
  - adult
  - data analysis software
  - cholinergic system
  - electrocardiogram
  - middle aged
  - adrenergic system
  - animal assisted therapy
  - spectroscopy
  - laboratory device
  - donkey
  - heart rate monitor
  - ICD-11
  - Kubios HRV 2.1
  - Kubios Premium software
  - monitoring
  - paranoid schizophrenia
  - Polar S610i
  - Polar V800S Polar heart rate monitor

  ### Attachments

  - Full Text (HTML)
- ## Effects of stress on cognitive performance

  |  |  |
  | --- | --- |
  | Item Type | Journal Article |
  | Author | D.C. Nwikwe |
  | Abstract | Stress remains a pervasive challenge in modern life, exerting significant impacts on cognitive performance and overall well-being by triggering release of stress hormones like adrenaline and cortisol. It has profound implications for education, work performance, and everyday life, impacting cognitive performance, health outcomes, and social relationships. It does this by impacting memory, attention and focus, informed decision-making, developmental and cognitive performance, work and educational performance, genetic and epigenetic influence, and public health. When a stressor is perceived, the hypothalamus in the brain signals the pituitary gland to release adrenocorticotropic hormone, hence adrenaline is quickly released into the bloodstream, causing immediate physiological changes and thus releasing cortisol gradually to help maintain the body's response to stress over a longer period through the hypothalamic-pituitary-adrenal and sympathetic-adrenomedullar axis. The impacts can be short-term or long-term focusing on the working memory, pre-frontal cortex, amygdala, and hippocampus. By recognizing these implications and implementing targeted interventions, we can foster environments that support resilience, optimize performance, and enhance overall well-being across diverse contexts. This chapter also highlighted some mitigation strategies to reduce stress-related activities and improve cognitive performance, such as cognitive-behavioral therapy, mindfulness-based stress reduction, healthy lifestyle adoption, pet therapy, time management and prioritization, and workplace interventions. |
  | Date | 2025 |
  | Language | English |
  | Archive | Embase |
  | URL | https://www.embase.com/search/results?subaction=viewrecord&id=L2037394863&from=export |
  | Volume | 291 |
  | Pages | 109-135 |
  | Publication | Progress in Brain Research |
  | DOI | 10.1016/bs.pbr.2025.01.015 |
  | Issue | (Nwikwe D.C., davidnwikwe@gmail.com) Department of Chemical Sciences (Biochemistry Unit), Faculty of Science, Kings University, Osun State, Odeomu, Nigeria |
  | Journal Abbr | Prog. Brain Res. |
  | ISSN | 1875-7855 |
  | Date Added | 05/02/2026, 17:53:14 |
  | Modified | 05/02/2026, 17:53:14 |

  ### Tags:

  - behavior
  - physiology
  - article
  - physiological stress
  - human
  - hydrocortisone
  - epinephrine
  - posttraumatic stress disorder
  - brain function
  - mindfulness-based stress reduction
  - wellbeing
  - mental stress
  - psychology
  - job stress
  - mental performance
  - health
  - hypothalamus hypophysis adrenal system
  - mental health
  - pet therapy
  - social connectedness
  - memory
  - chronic stress
  - acute stress
  - yoga
  - adrenal medullary cell
  - brain
  - attention
  - psychological resilience
  - environmental stress
  - prevalence
  - adrenal gland
  - cognitive behavioral therapy
  - academic achievement
  - aerobic exercise
  - biology
  - breathing exercise
  - decision making
  - dorsolateral prefrontal cortex
  - healthy lifestyle
  - problem solving
  - stress hormone
  - symptom
  - Tai Chi
  - time management
  - working memory
  - workplace

  ### Attachments

  - Full Text (HTML)
- ## Equine spinal kinematics derived from different riding positions during asymmetrical bareback riding

  |  |  |
  | --- | --- |
  | Item Type | Journal Article |
  | Author | N. Nuchprayoon |
  | Author | P. Ritruechai |
  | Author | K. Watchararat |
  | Author | W. Limroongruengrat |
  | Author | T. Wongtawan |
  | Author | N. Arya |
  | Abstract | Hippotherapy in patients with neuromuscular dysfunction creates high focal pressure on the pony’s back due to bareback riding and an asymmetrical riding position. This study aimed to investigate the acute effect of asymmetrical bareback riding on the pony’s spinal kinematics, blood lactate, serum creatine kinase, heart rate, and temperament score. Eight ponies were selected, and they were walked on a treadmill for 45 min on each experimental day, including warm-up (5 min), weight-loading by mannequin (30 min), and cool-down (10 min) sessions. During the weight-loading session, three different weight distributions on the pony’s back were applied between the left and right side: 50:50 (treatment M), 70:30 (treatment L), and 30:70 (treatment R) on the first, second, and third day of the experiment, respectively. The spinal kinematics at the end of the weight-loading session revealed a slight reduction in range of motion in both flexion-extension and lateral bending during treatment R. Stride length and stride duration showed no differences between treatments. The levels of blood lactate and serum creatine kinase and results of a back examination were normal. Heart rates and temperament scores revealed that all ponies were calm throughout loading of the mannequin. This information suggests that asymmetrical bareback riding did not cause acute or serious back injury, which indicates good equine welfare in ponies used for hippotherapy. |
  | Date | 2021 |
  | Language | English |
  | Archive | Embase |
  | URL | https://www.embase.com/search/results?subaction=viewrecord&id=L2013765605&from=export |
  | Volume | 32 |
  | Pages | 81-89 |
  | Publication | Journal of Equine Science |
  | DOI | 10.1294/jes.32.81 |
  | Issue | 3 |
  | Journal Abbr | J. Equine Sci. |
  | ISSN | 1347-7501 |
  | Date Added | 05/02/2026, 17:58:53 |
  | Modified | 05/02/2026, 17:58:53 |

  ### Tags:

  - welfare
  - heart rate
  - animal experiment
  - article
  - defecation
  - nonhuman
  - human
  - body weight
  - hippotherapy
  - horseback riding
  - information processing
  - exercise
  - animal tissue
  - Equus
  - lactate blood level
  - stress assessment
  - heart rate measurement
  - temperament
  - animal lameness
  - body height
  - pony
  - stride length
  - barebacking
  - biomechanics
  - cardiotachometer
  - cool down
  - creatine kinase blood level
  - experimental design
  - force
  - force gauge
  - injury
  - kinematics
  - knee function
  - Kruskal Wallis test
  - manikin
  - motion analysis system
  - neuromuscular disease
  - polar H7
  - range of motion
  - spatiotemporal analysis
  - spine
  - treadmill
  - warm up

  ### Attachments

  - Full Text (HTML)
- ## Heart rate and salivary cortisol as indicators of arousal and synchrony in clients, therapy horses and therapist in equine-assisted therapy

  |  |  |
  | --- | --- |
  | Item Type | Journal Article |
  | Author | A. Naber |
  | Author | L. Kreuzer |
  | Author | R. Zink |
  | Author | E. Millesi |
  | Author | R. Palme |
  | Author | K. Hediger |
  | Author | L.M. Glenk |
  | Abstract | BACKGROUND: This exploratory study aimed to analyse physiological interaction processes in equine-assisted-therapy (EAT) between client, therapy horse and therapist. METHODS: We measured heart rate (HR), heart rate variability (HRV) and cortisol levels before, during and after a standardized therapy session and a control condition in one therapist, four therapy horses and ten female clients in emerging adulthood (Mn = 21.8 years, SD = 3.39). The clients were diagnosed with mild (N = 5) to moderate (N = 5) intellectual disability (ID). RESULTS: There was no significant change in the client's HR, HRV and cortisol levels during an EAT session. No difference was observed between therapy sessions with or without a therapy horse, except during the challenge phase of the EAT protocol, where clients had a significantly lower HR when interacting with the therapy horse. HR between therapist and client correlated significantly, as well as between therapist and horse. This effect was greater when therapists interacted with a familiar horse. Clients' and horses' HRs also correlated, but only when the horse was the clients' familiar and preferred horse. CONCLUSIONS: These results indicate that relationship intensity is an important factor for the synchronization process. Moreover, the inclusion of horses in a therapeutic setting can lead to a decreased HR in young adults with intellectual disability while mastering a challenge. Future research should investigate this potential benefit of EAT, considering the reciprocal influences and the relationship between client, therapist and horse. |
  | Date | 2025 |
  | Language | English |
  | Archive | Medline |
  | URL | https://www.embase.com/search/results?subaction=viewrecord&id=L646361603&from=export |
  | Volume | 59 |
  | Pages | 101937 |
  | Publication | Complementary therapies in clinical practice |
  | DOI | 10.1016/j.ctcp.2025.101937 |
  | Issue | (Naber A., anna.naber@lichtblickhof.at; Zink R., roswitha.zink@lichtblickhof.at) Vienna, Austria |
  | Journal Abbr | Complement Ther Clin Pract |
  | ISSN | 1873-6947 |
  | Date Added | 05/02/2026, 17:53:13 |
  | Modified | 05/02/2026, 17:53:13 |

  ### Tags:

  - horse
  - physiology
  - heart rate
  - saliva
  - therapy
  - female
  - male
  - human
  - hydrocortisone
  - adult
  - hippotherapy
  - animal
  - young adult
  - chemistry
  - arousal
  - metabolism
  - procedures
  - intellectual impairment

  ### Attachments

  - Full Text (HTML)
- ## A Relaxed Horse—A Relaxed Client? An Experimental Investigation of the Effects of Therapy Horses’ Stress on Clients’ Stress, Mood, and Anxiety

  |  |  |
  | --- | --- |
  | Item Type | Journal Article |
  | Author | A. Müller-Klein |
  | Author | M.N. Braun |
  | Author | D.S. Ferreira de Sá |
  | Author | T. Michael |
  | Author | U. Link-Dorner |
  | Author | J. Lass-Hennemann |
  | Abstract | Equine-assisted therapies are becoming increasingly popular for addressing physical and psychological disabilities in clients. The role of the horse’s welfare in equine-assisted service receives increasing attention in research. Several studies have shown that horses are able to perceive human emotions and respond to human stress responses. However, no research has yet looked at the other side of the coin—whether and how humans perceive and react to equine stress levels during equine-assisted services. To fill this gap in the research, we employed a within-subjects design, in which horse-naïve participants had a standardized interaction with both an experimentally stressed horse and an experimentally relaxed horse. We assessed physiological indicators of stress (heart rate, heart rate variability, and salivary cortisol) in participants and horses, as well as psychological indicators of stress (state anxiety and positive and negative affect) in participants. Although our stress and relaxation manipulations were successful (indicated by horses’ physiological indicators of stress), we did not find any difference in the participants’ physiological or psychological indicators of stress between the interaction with a stressed and the interaction with a relaxed horse. Together with results from previous studies, this suggests that humans cannot intuitively recognize the (physiological) stress level of horses, which has important implications for effective communication and bonding between humans and horses and for the safety of equine activities. |
  | Date | 2024 |
  | Language | English |
  | Archive | Embase |
  | URL | https://www.embase.com/search/results?subaction=viewrecord&id=L2028662269&from=export |
  | Volume | 14 |
  | Publication | Animals |
  | DOI | 10.3390/ani14040604 |
  | Issue | 4 |
  | Journal Abbr | Animals |
  | ISSN | 2076-2615 |
  | Date Added | 05/02/2026, 17:53:21 |
  | Modified | 05/02/2026, 17:53:21 |

  ### Tags:

  - horse
  - heart rate variability
  - heart rate
  - anxiety
  - animal experiment
  - article
  - nonhuman
  - physiological stress
  - human
  - hydrocortisone
  - adult
  - biomedical software
  - Brief Symptom Inventory
  - data analysis software
  - hippotherapy
  - scoring system
  - human experiment
  - normal human
  - monitor
  - questionnaire
  - electrocardiograph
  - electrode
  - mood
  - rating scale
  - electrocardiogram
  - Positive and Negative Affect Schedule
  - young adult
  - State Trait Anxiety Inventory
  - cotton swab
  - immunoassay
  - tube
  - Beck Depression Inventory
  - salivary cortisol
  - heart rate monitor
  - patient participation
  - BioSignalsPlux
  - fluorometry
  - interpersonal reactivity index
  - Kendall H124SG
  - Kubios HRV Standard 3.4.1
  - lexington attachment to pets scale
  - pet attitude scale
  - polar V800
  - relaxed horse
  - salivary gland
  - salivette tube
  - sampling
  - telephone interview

  ### Attachments

  - Full Text (HTML)
- ## Employing Siamese Networks as Quantitative Biomarker for Assessing the Effect of Dolphin-Assisted Therapy on Pediatric Cerebral Palsy

  |  |  |
  | --- | --- |
  | Item Type | Journal Article |
  | Author | J.J. Moreno Escobar |
  | Author | O. Morales Matamoros |
  | Author | E.Y. Aguilar del Villar |
  | Author | H. Quintana Espinosa |
  | Author | L. Chanona Hernández |
  | Abstract | This study explores the potential of using a Siamese Network as a biomarker for assessing the effectiveness of Dolphin-Assisted Therapy (DAT) in children with Spastic Cerebral Palsy (SCP). The problem statement revolves around the need for objective measures to evaluate the impact of DAT on patients with SCP, considering the subjective nature of traditional assessment methods. The methodology involves training a Siamese network, a type of neural network designed to compare similarities between inputs, using data collected from SCP patients undergoing DAT sessions. The study employed Event-Related Potential (ERP) and Fast Fourier Transform (FFT) analyses to examine cerebral activity and brain rhythms, proposing the use of SNN to compare electroencephalographic (EEG) signals of children with cerebral palsy before and after Dolphin-Assisted Therapy. Testing on samples from four children yielded a high average similarity index of 0.9150, indicating consistent similarity metrics before and after therapy. The network is trained to learn patterns and similarities between pre- and post-therapy evaluations, in order to identify biomarkers indicative of therapy effectiveness. Notably, the Siamese Network’s architecture ensures that comparisons are made within the same feature space, allowing for more accurate assessments. The results of the study demonstrate promising findings, indicating different patterns in the output of the Siamese Network that correlate with improvements in symptoms of SCP post-DAT. Confirming these observations will require large, longitudinal studies but such findings would suggest that the Siamese Network could have utility as a biomarker in monitoring treatment responses for children with SCP who undergo DAT and offer them more objective as well as quantifiable manners of assessing therapeutic interventions. Great discrepancies in neuronal voltage perturbations, 7.9825 dB on average at the specific samples compared to the whole dataset (6.2838 dB), imply a noted deviation from resting activity. These findings indicate that Dolphin-Assisted Therapy activates particular brain regions specifically during the intervention. |
  | Date | 2024 |
  | Language | English |
  | Archive | Embase |
  | URL | https://www.embase.com/search/results?subaction=viewrecord&id=L2031139495&from=export |
  | Volume | 14 |
  | Publication | Brain Sciences |
  | DOI | 10.3390/brainsci14080778 |
  | Issue | 8 |
  | Journal Abbr | Brain Sci. |
  | ISSN | 2076-3425 |
  | Date Added | 05/02/2026, 17:53:18 |
  | Modified | 05/02/2026, 17:53:18 |

  ### Tags:

  - child
  - article
  - human
  - data analysis software
  - Fourier transform
  - electroencephalogram
  - electroencephalography
  - human experiment
  - normal human
  - electroencephalograph electrode
  - cerebral palsy
  - deep learning
  - biological marker
  - animal assisted therapy
  - health status
  - therapy effect
  - comparative study
  - longitudinal study
  - artificial neural network
  - bottlenose dolphin
  - convolutional neural network
  - dolphin assisted therapy
  - event related potential
  - feature extraction
  - functional connectivity
  - learning algorithm
  - left hemisphere
  - mathematical model
  - Matlab R2024a
  - nerve cell network
  - power spectrum
  - reference electrode
  - right hemisphere
  - siamese network
  - spastic cerebral palsy
  - spastic paralysis
  - spiking neural network

  ### Attachments

  - Full Text (HTML)
- ## THE EFFECT OF THERAPEUTIC RIDING ON HEMODYNAMIC PARAMETERS OF PEOPLE

  |  |  |
  | --- | --- |
  | Item Type | Journal Article |
  | Author | E. Mlyneková |
  | Author | M. Halo |
  | Author | I. Imrich |
  | Author | M. Šmondrková |
  | Abstract | Hippotherapy has become part of therapeutic procedures that use the complex therapeutic effect of the horse on the client. The aim of the work was to analyse hemodynamic indicators of people during therapeutic riding. Changes in heart rate before, during and after riding and blood pressure before and after riding were evaluated in 68 probands. Probands were divided into groups according to riding performance level, age, gender and systolic and diastolic blood pressure values. Therapeutic riding represented the average load of the organism in the participating probands. When comparing probands according to performance level, there were significant differences in the heart rate before and after riding (P < 0.05) at the lower advanced performance level. Significant changes in the heart rate occurred during riding only in the age group from 16 to 40 years (P < 0.05). Both men and women achieved similar changes in the heart rate. No significant differences in the heart rate were found in people with low and high systolic blood pressure. There were statistically significant changes (P < 0.05) in the heart rate before and after riding only in probands with normal diastolic blood pressure. From the evaluation of the results of the effect of therapeutic driving on the level of systolic blood pressure, we found the highest values (133.92–135.92 mmHg) in the group of beginners. We recorded a decrease in systolic blood pressure after riding only in the group of less advanced probands. For them, the training load did not represent a significant physical load or mental discomfort. The highest values of systolic blood pressure depending on age were in probands aged 41–60 years. Statistically significantly higher values of systolic blood pressure were found in men (P < 0.01). The results of the analysis of diastolic blood pressure values confirm the beneficial effect of riding on the cardiovascular system of probands. Therapeutic riding decreased the level of diastolic pressure in probands with high blood pressure and increased it in probands with low diastolic pressure (P < 0.001). In the horses used in the therapeutic process, there was a statistically significant increase in the heart rate during training (P < 0.001). Based on the achieved results, we can state that the mutual interaction between the horse and the human significantly affects changes in hemodynamic parameters of clients. The interconnection of the mental well-being of the horse, the client and the hippo-rehabilitation team is the basis of success. |
  | Date | 2024 |
  | Language | English |
  | Archive | Embase |
  | URL | https://www.embase.com/search/results?subaction=viewrecord&id=L2036571715&from=export |
  | Volume | 82(1) |
  | Pages | 150-150 |
  | Publication | Veterinarija ir Zootechnika |
  | Issue | (Mlyneková E., eva.mlynekova@uniag.sk; Halo M.; Imrich I.; Šmondrková M.) Slovak University of Agriculture in Nitra, Faculty of Agrobiology and Food Resources, Institute of Animal Husbandry, Slovakia |
  | Journal Abbr | Vet. Zootech. |
  | ISSN | 1392-2130 |
  | Date Added | 05/02/2026, 17:53:23 |
  | Modified | 05/02/2026, 17:53:23 |

  ### Tags:

  - blood pressure
  - horse
  - heart rate
  - article
  - human
  - hippotherapy
  - horseback riding
  - diastolic blood pressure
  - psychological well-being
  - systolic blood pressure
  - therapy effect
  - cardiovascular system
  - hemodynamic parameters
  - therapeutic riding
- ## Investigation of Physiological and Behavioral Responses in Dogs Participating in Animal-Assisted Therapy with Children Diagnosed with Attention-Deficit Hyperactivity Disorder

  |  |  |
  | --- | --- |
  | Item Type | Journal Article |
  | Author | A.L. Melco |
  | Author | L. Goldman |
  | Author | A.H. Fine |
  | Author | J.M. Peralta |
  | Abstract | This study evaluated the impact that participation in sessions with children with Attention-Deficit Hyperactivity Disorder (ADHD) has on therapy dogs. Nine certified therapy dogs were paired for 6 sessions with groups of 3–4 children. Sessions consisted of 5 different activities. Activities 1 and 5 involved interactions solely with each dog and their owner, as a control. Activities 2–4 consisted of interactions with the dogs and the children which included social skills training, dog training, and reading in the company of dogs. One-zero interval sampling of stress-associated behaviors was conducted at 20-second intervals for a 10-minute duration during each of the 5 activities. At the end of each activity, heart rate was monitored, and a saliva sample was obtained for cortisol analysis. Dogs demonstrated only occasional behavioral responses and no significant findings related to cortisol or heart rate when the different activities were compared. The results indicate that with proper supervision and well-trained therapy staff, including suitable therapy dogs and their handlers, canine stress can be minimal in a therapy setting. |
  | Date | 2020 |
  | Language | English |
  | Archive | Embase |
  | URL | https://www.embase.com/search/results?subaction=viewrecord&id=L624685756&from=export |
  | Volume | 23 |
  | Pages | 10-28 |
  | Publication | Journal of Applied Animal Welfare Science |
  | DOI | 10.1080/10888705.2018.1536979 |
  | Issue | 1 |
  | Journal Abbr | J. Appl. Anim. Welf. Sci. |
  | ISSN | 1532-7604 |
  | Date Added | 05/02/2026, 17:58:59 |
  | Modified | 05/02/2026, 17:58:59 |

  ### Tags:

  - behavior
  - reading
  - heart rate
  - dog
  - anxiety
  - child
  - article
  - female
  - glucocorticoid
  - male
  - nonhuman
  - human
  - hydrocortisone
  - controlled study
  - human experiment
  - exercise
  - questionnaire
  - adrenergic system
  - training
  - animal assisted therapy
  - attention deficit hyperactivity disorder
  - hydrocortisone blood level
  - saliva analysis
  - social competence
  - vocalization
  - hyperactivity
  - cortisol test kit
  - Canis
  - cell interaction
  - frequency
  - heart auscultation
  - mesenchymal stem cell
  - musculoskeletal function
  - resident
  - salivation
  - wet dog shakes
  - yawning

  ### Attachments

  - Full Text (HTML)
- ## An observational evaluation of stress in horses during therapeutic riding sessions

  |  |  |
  | --- | --- |
  | Item Type | Journal Article |
  | Author | L. McDuffee |
  | Author | L. Carr |
  | Author | W. Montelpare |
  | Abstract | Therapeutic riding (TR) provides benefits to participants with cognitive and physical disabilities. Horses participating in TR programs are typically selected because of their calm temperament and may not show obvious signs of stress. However, the welfare of horses in TR programs is an important aspect when evaluating the delivery of the program, to ensure sustainability. The aim of this study was to assess stress levels in TR horses during scheduled sessions. The research was carried out during normal therapeutic horseback riding lessons designed for participants with intellectual and developmental disabilities within a certified program. There were 4 horses in the study who participated in multiple lessons each week over an 8-week period. Behavior scores, salivary cortisol, and heart rate variability measures were analyzed as measures of stress during baseline, mounting and dismounting. Results indicated that physiologic and behavioral measures of stress were increased during mounting and dismounting compared to baseline levels. Ethologically sound solutions to alleviate stress during TR activities is warranted to improve equine welfare. |
  | Date | 2022 |
  | Language | English |
  | Archive | Embase |
  | URL | https://www.embase.com/search/results?subaction=viewrecord&id=L2016674848&from=export |
  | Volume | 49 |
  | Pages | 53-64 |
  | Publication | Journal of Veterinary Behavior |
  | DOI | 10.1016/j.jveb.2021.11.009 |
  | Issue | (McDuffee L., lmcduffee@upei.ca; Carr L.; Montelpare W.) Departments of Health Management (McDuffee and Carr) and Applied Human Sciences (Montelpare) University of Prince Edward Island, Charlottetown, Prince Edward Island, Canada |
  | Journal Abbr | J. Vet. Behav. |
  | ISSN | 1558-7878 |
  | Date Added | 05/02/2026, 17:53:33 |
  | Modified | 05/02/2026, 17:53:33 |

  ### Tags:

  - horse
  - animal welfare
  - heart rate variability
  - animal behavior
  - article
  - female
  - male
  - nonhuman
  - physiological stress
  - human
  - hydrocortisone
  - adult
  - controlled study
  - hippotherapy
  - scoring system
  - adolescent
  - young adult
  - Equus
  - observational study
  - saliva level
  - intellectual impairment
  - health program
  - cohort analysis
  - developmental disorder
  - ethology

  ### Attachments

  - Full Text (HTML)
- ## Stroking a Real Horse Versus Stroking a Toy Horse: Effects on the Frontopolar Area of the Human Brain

  |  |  |
  | --- | --- |
  | Item Type | Journal Article |
  | Author | A. Matsuura |
  | Author | N. Aiba |
  | Author | H. Yamamoto |
  | Author | M. Takahashi |
  | Author | H. Kido |
  | Author | T. Suzuki |
  | Author | Y. Bando |
  | Abstract | The effects of animal-assisted therapy (AAT) on human cerebral activity are not clearly understood, although many studies have reported psychological and physiotherapeutic benefits associated with it. Any benefits of AAT are likely to be closely related to brain activity. The aim of the present study was to investigate the effects of stroking a horse on brain activity in humans, specifically the frontopolar area, to evaluate the significance of using real animals in AAT. In a randomized cross-over trial, 30 healthy adults saw and stroked a stuffed toy (Session T) followed by a miniature horse (Session A), or vice versa. Participants saw and stroked the object (the stuffed toy or the real animal) for 10 minutes, followed by seeing and stroking another object (the real animal or the stuffed toy) for 10 minutes, with 20 minutes for measurements and resting. We measured changes in oxygenated hemoglobin (oxy-Hb) in the frontopolar area using near-infrared spectroscopy (NIRS). Three-way repeated measures ANOVA was used to compare oxy-Hb changes between the object used (stuffed toy versus real animal), contact method (see versus stroke), and laterality (left versus right). Oxy-Hb changes were larger in the right frontopolar area compared with those in the left frontopolar area when participants stroked a real horse (p < 0.05). Binominal tests showed that, in participants who reported that they “loved horses,” the laterality of oxy-Hb changes when participants stroked a real horse was significant (p = 0.031), but this was not seen in those who reported that they only “kind of liked” horses. These findings suggest that stroking a real horse activates the right frontopolar area, but this effect is not achieved when stroking a stuffed toy. Using horses in AAT that aims to activate human brain activity could be useful. |
  | Date | 2020 |
  | Language | English |
  | Archive | Embase |
  | URL | https://www.embase.com/search/results?subaction=viewrecord&id=L2006096521&from=export |
  | Volume | 33 |
  | Pages | 673-683 |
  | Publication | Anthrozoos |
  | DOI | 10.1080/08927936.2020.1799564 |
  | Issue | 5 |
  | Journal Abbr | Anthrozoos |
  | ISSN | 1753-0377 |
  | Date Added | 05/02/2026, 17:58:53 |
  | Modified | 05/02/2026, 17:58:53 |

  ### Tags:

  - horse
  - article
  - female
  - male
  - human
  - adult
  - controlled study
  - physiotherapy
  - randomized controlled trial
  - crossover procedure
  - electroencephalogram
  - hemoglobin
  - human experiment
  - animal assisted therapy
  - prefrontal cortex
  - task performance
  - therapy effect
  - accuracy
  - cerebrovascular accident
  - near infrared spectroscopy
  - signal processing

  ### Attachments

  - Full Text (HTML)
- ## Neurodynamics of patients during a dolphin-assisted therapy by means of a fractal intraneural analysis

  |  |  |
  | --- | --- |
  | Item Type | Journal Article |
  | Author | O.M. Matamoros |
  | Author | J.J.M. Escobar |
  | Author | R.T. Padilla |
  | Author | I.L. Reyes |
  | Abstract | The recent proliferation of sensor technology applications in therapies for children’s disabilities to promote positive behavior among such children has produced optimistic results in developing a variety of skills and abilities in them. Dolphin-Assisted Therapy (DAT) has also become a topic of public and research interest for these disorders’ intervention and treatment. This work exposes the development of a system that controls brain–computer interaction when a patient with different abilities undergoes a DAT. To develop the proposed system, TGAM1, i.e., ThinkGear-AM1 series of NeuroSky company, was used, connecting it to an isolated Bluetooth 4.0 communication protocol from a brackish and humid environment, and a Notch Filter was applied to reduce the input noise. In this way, at Definiti Ixtapa-Mexico facilities, we explored the behavior of three children with Infantile Spastic Cerebral Palsy (Experiment 1), as well as the behavior of Obsessive Compulsive Disorder and neurotypic children (Experiment 2). This was done applying the Power Spectrum Density (PSD) and the Self-Affine Analysis (SSA) from Electroencephalogram (EEG) biosignals. The EEG Raw data were time series showing the cerebral brain activity (voltage versus time) before and during DAT for the Experiment 1, and before, during DAT and after for the Experiment 2. Likewise, the EEW RAW data were recorded by the first frontopolar electrode (FP1) by means of an EEG biosensor TGAM1 Module. From the PSD we found that in all child patients a huge increment of brain activity during DAT regarding the before and after therapy periods around 376.28 %. Moreover, from the SSA we found that the structure function of the all five child patients displayed an antipersistent behavior, characterized by σ ∝ (δt )H, for before, during DAT and after. Nonetheless, we propose that one way to assess whether a DAT is being efficient to the child patients is to increase the during DAT time when the samples are collected, supposing the data fitting by a power law will raise the time, displaying a persistent behavior or positive correlations, until a crossover appears and the curve tends to be horizontal, pointing out that our system has reached a stationary state. |
  | Date | 2020 |
  | Language | English |
  | Archive | Embase |
  | URL | https://www.embase.com/search/results?subaction=viewrecord&id=L2004617932&from=export |
  | Volume | 10 |
  | Pages | 1-23 |
  | Publication | Brain Sciences |
  | DOI | 10.3390/brainsci10060403 |
  | Issue | 6 |
  | Journal Abbr | Brain Sci. |
  | ISSN | 2076-3425 |
  | Date Added | 05/02/2026, 17:58:55 |
  | Modified | 05/02/2026, 17:58:55 |

  ### Tags:

  - child
  - article
  - female
  - male
  - human
  - Fourier transform
  - electroencephalography
  - cerebral palsy
  - learning
  - clinical article
  - animal assisted therapy
  - working memory
  - mathematical model
  - power spectrum
  - brain computer interface
  - dolphinb assisted therapy
  - fractal analysis
  - human computer interaction
  - molecular dynamics
  - obsessive compulsive disorder
  - self affine analysis

  ### Attachments

  - Full Text (HTML)
- ## Evaluating effects of animal-assisted therapy on pediatric dental care patients: A pilot clinical trial

  |  |  |
  | --- | --- |
  | Item Type | Journal Article |
  | Author | J. Massouda |
  | Author | N. Ghaltakhchyan |
  | Author | J. Judd |
  | Author | C. Bocklage |
  | Author | R. Selden |
  | Author | O. TumSuden |
  | Author | E. Nanney |
  | Author | J. Lee |
  | Author | J. Ginnis |
  | Author | T. Strauman |
  | Author | C. Sawicki |
  | Author | E.A. Hodges |
  | Author | C. Graves |
  | Author | K. Divaris |
  | Author | L. Jacox |
  | Abstract | BACKGROUND: An estimated 6% through 22% of children have dental anxiety, which can contribute to disruptive behavior and oral health care avoidance. Evidence from medical settings indicate reductions in pain and stress after therapy dog implementation. To identify a low-risk, nonpharmacologic approach for anxiety and pain management in dentistry, a pilot prospective clinical trial was conducted to determine best practices for evaluating the efficacy of animal-assisted therapy (AAT). METHODS: The effects of AAT on pediatric patients were measured through physiological, objective measures (ie, heart rate, salivary cortisol and α-amylase, and video coding) and validated self-reported scales of anxiety, fear, and pain, during an invasive dental procedure. Children aged 7 through 14 years were consecutively enrolled into an AAT (n = 18) or control (n = 21) group. Participants underwent an operative or surgical (eg, extraction) dental procedure. Descriptive and bivariate statistics were used. RESULTS: Participants in the AAT group reported significantly less postoperative pain than those in the control group (P = .001). The heart rates of AAT participants dropped after key events and had less variation than control participants. Objective video coding revealed that AAT participants had significantly longer durations of relaxed lower bodies than control participants (P = .204). No differences were seen in salivary cortisol and α-amylase. These physiological and self-report measures are feasible for use in future studies. CONCLUSIONS: AAT may be an effective therapy for alleviating anxiety and pain in pediatric dental patients and warrants additional study using both objective physiological end points and subjective self-report measures. PRACTICAL IMPLICATIONS: Pending further research, canine therapy may be a valuable addition to dental clinics for anxiety and pain management. This clinical trial was registered at ClinicalTrials.gov. The registration number is NCT04708028. |
  | Date | 2025 |
  | Language | English |
  | Archive | Medline |
  | URL | https://www.embase.com/search/results?subaction=viewrecord&id=L647616830&from=export |
  | Volume | 156 |
  | Pages | 447-457 |
  | Publication | Journal of the American Dental Association (1939) |
  | DOI | 10.1016/j.adaj.2025.03.006 |
  | Issue | 6 |
  | Journal Abbr | J Am Dent Assoc |
  | ISSN | 1943-4723 |
  | Date Added | 05/02/2026, 17:53:13 |
  | Modified | 05/02/2026, 17:53:13 |

  ### Tags:

  - physiology
  - heart rate
  - dog
  - saliva
  - therapy
  - child
  - female
  - male
  - human
  - hydrocortisone
  - pilot study
  - adolescent
  - animal
  - psychology
  - clinical trial
  - chemistry
  - amylase
  - animal assisted therapy
  - procedures
  - prospective study
  - dental anxiety
  - dental procedure
  - pain measurement
  - prevention and control
  - analgesia
  - NCT04708028

  ### Attachments

  - Full Text (HTML)
- ## Effects of contact with a dog on prefrontal brain activation in patients in a minimally conscious state: A controlled crossover trial

  |  |  |
  | --- | --- |
  | Item Type | Journal Article |
  | Author | R. Marti |
  | Author | M. Petignat |
  | Author | V.L. Marcar |
  | Author | J. Hattendorf |
  | Author | M. Wolf |
  | Author | M. Hund-Georgiadis |
  | Author | K. Hediger |
  | Abstract | The first studies have indicated that animal-assisted therapy benefits patients in a minimally conscious state (MCS), but the evidence is scarce. It is thus crucial to understand how these patients react to animal contact. This study aimed to measure the prefrontal brain activation in MCS patients during contact with a dog compared with a plush animal using functional near-infrared spectroscopy (fNIRS). We conducted a controlled crossover trial with 22 MCS patients, who each participated in six sessions. Patients interacted with a dog in three sessions and with a plush animal in three control sessions. Each session consisted of five 2-minute phases with a neutral phase at the start and the end. The contact intensity with the dog or the plush animal increased from the second to the fourth phase. The fNIRS parameters did not differ between the conditions. The mean heart rate was significantly higher in the dog condition than in the control. In both conditions, prefrontal brain activation, mean heart rate, and one heart rate variability parameter increased with the increased intensity of contact with the dog and plush animal. The results show that MCS patients react with the same prefrontal brain activation during contact with a dog and a plush animal but have increased heart rate in contact with the dog, indicating physiological arousal. These findings suggest that the incorporation of animals into MCS therapy has the potential to stimulate patients, thereby facilitating greater participation. However, more research is needed to understand the effects of animals on brain activation. |
  | Date | 2025 |
  | Language | English |
  | Archive | Embase |
  | URL | https://www.embase.com/search/results?subaction=viewrecord&id=L2038827911&from=export |
  | Volume | 577 |
  | Pages | 175-189 |
  | Publication | Neuroscience |
  | DOI | 10.1016/j.neuroscience.2025.05.014 |
  | Issue | (Marti R.; Petignat M.; Hediger K., karin.hediger@unilu.ch) Faculty of Psychology, University of Basel, Basel, Switzerland |
  | Journal Abbr | Neuroscience |
  | ISSN | 1873-7544 |
  | Date Added | 05/02/2026, 17:53:11 |
  | Modified | 05/02/2026, 17:53:11 |

  ### Tags:

  - human-animal interaction
  - physiology
  - heart rate
  - dog
  - animal experiment
  - article
  - female
  - male
  - nonhuman
  - controlled study
  - data analysis software
  - assessment of humans
  - basler vegetative state assessment
  - body temperature
  - brain function
  - brain injury
  - clinical trial (topic)
  - coma remission scale revised
  - crossover procedure
  - deoxygenated hemoglobin
  - electroencephalogram
  - Erprec-EEG/ERP recording Software version 2.0.7.1
  - functional near infrared spectrometer
  - functional near-infrared spectroscopy
  - Glasgow coma scale
  - golden retriever
  - hemoglobin
  - hemoglobin determination
  - imaging software
  - Kubios HRV Standard version 3.1.0–3.5.0
  - minimally conscious state
  - Model 8004CA Sensors Adhesive
  - NCT03341325
  - neurorehabilitation
  - oxygen saturation
  - oxygenated hemoglobin
  - prefrontal brain activation
  - R 4.1.0
  - R package lme4
  - sensitivity analysis
  - SenSmart Model X- 100
  - SenSmart software version 1.0.1.0
  - sensor
  - spectrometer
  - unclassified drug

  ### Attachments

  - Full Text (HTML)
- ## A Text-Mining Analysis of Research Trends in Animal-Assisted Therapy

  |  |  |
  | --- | --- |
  | Item Type | Journal Article |
  | Author | S.-J. Lee |
  | Author | G.-H. Kim |
  | Author | Y.-H. Moon |
  | Author | S.-S. Lee |
  | Abstract | Text-mining techniques were used to provide basic data to related policy stakeholders and academic researchers by collecting and analyzing research trends related to animal-mediated healing in a short time. A total of 776 studies were collected using the keyword “animal-assisted therapy” (AAT) in the search engine PubMed, which covers a wide range of topics related to health sciences, biomedical research, and health psychology. Four analysis methods were employed. “Dog” was the most commonly utilized animal in AAT. This study also identified individuals with autism spectrum disorder and post-traumatic stress disorder as the primary research participants. Finally, the terms “health care” and “blood pressure” were identified, indicating that AAT has a positive impact on improving blood pressure and enhancing heart rate. These findings demonstrate that AAT research is being actively pursued in various fields, such as social sciences, medicine, and psychology. |
  | Date | 2023 |
  | Language | English |
  | Archive | Embase |
  | URL | https://www.embase.com/search/results?subaction=viewrecord&id=L2026038337&from=export |
  | Volume | 13 |
  | Publication | Animals |
  | DOI | 10.3390/ani13193133 |
  | Issue | 19 |
  | Journal Abbr | Animals |
  | ISSN | 2076-2615 |
  | Date Added | 05/02/2026, 17:53:25 |
  | Modified | 05/02/2026, 17:53:25 |

  ### Tags:

  - blood pressure
  - anxiety
  - article
  - nonhuman
  - physiotherapy
  - posttraumatic stress disorder
  - autism
  - psychology
  - animal assisted therapy
  - attention deficit hyperactivity disorder
  - dementia
  - medical research
  - data mining
  - trend study

  ### Attachments

  - Full Text (HTML)
- ## The effects of a therapy dog intervention on dental fear and anxiety in adult patients undergoing dental procedures: a pilot study

  |  |  |
  | --- | --- |
  | Item Type | Journal Article |
  | Author | D. Lam |
  | Author | D.A. D'Anthony |
  | Author | S.A. Chilcutt |
  | Author | A. O'Connor |
  | Author | A.J. Avillo |
  | Author | N.J. Hamlin |
  | Author | J.E. Schmidt |
  | Abstract | Dental anxiety poses challenges for providing effective oral healthcare. While therapy dogs have shown promise in various medical and mental health contexts, their use for alleviating dental anxiety in adults remains underexplored. This study aimed to investigate the emotional and physiologic effects of therapy dogs on self-reported dental anxiety. Adults with dental anxiety were randomly assigned to an intervention group (DOG; n = 19) or a standard care group (SC; n = 14). Standard self-report measures were used to assess dental anxiety (Index of Dental Anxiety and Fear [IDAF-4C+]), depression (Patient Health Questionnaire 9), and generalized anxiety (Generalized Anxiety Disorder 7) prior to the intervention. Participants in the DOG group received a 10-minute therapy dog intervention before dental procedures in sessions 1 and 2, while participants in the SC group rested quietly for 10 minutes before their procedure. The SC participants received the 10-minute therapy dog intervention before dental procedures in the third and final session, while patients in the DOG group received no intervention prior to their third procedure. After the dental procedures, patients completed a questionnaire about their satisfaction with the dog therapy (Therapy Satisfaction Scale) and recorded their anxiety and comfort levels on visual analog scales. Continuous electrocardiographic recording measured heart rate variability during the intervention and dental procedure. Prior to the intervention, most participants (90.9%) met the IDAF-4C+ criteria for dental anxiety, with 7 (21.2%) meeting the criteria for dental phobia. The DOG group participants expressed high satisfaction with the therapy dog intervention. No significant differences in heart rate variability were observed between the groups during dental procedures. Therapy dogs can effectively manage dental anxiety in adults with mild to moderate dental anxiety, offering potential benefits for oral healthcare. |
  | Date | 2024 |
  | Language | English |
  | Archive | Medline |
  | URL | https://www.embase.com/search/results?subaction=viewrecord&id=L644586392&from=export |
  | Volume | 72 |
  | Pages | 44-49 |
  | Publication | General dentistry |
  | Issue | 4 |
  | Journal Abbr | Gen Dent |
  | ISSN | 0363-6771 |
  | Date Added | 05/02/2026, 17:45:26 |
  | Modified | 05/02/2026, 17:45:26 |

  ### Tags:

  - dog
  - female
  - male
  - human
  - adult
  - controlled study
  - pilot study
  - randomized controlled trial
  - animal
  - psychology
  - middle aged
  - animal assisted therapy
  - procedures
  - dental anxiety
  - dental procedure
  - prevention and control
- ## Treating Agitation in Patients with Dementia with a Therapy Dog in a Milieu Therapy Setting on a Geropsychiatric Ward

  |  |  |
  | --- | --- |
  | Item Type | Journal Article |
  | Author | J. Krüger |
  | Author | R. Izgi |
  | Author | R. Hellweg |
  | Author | A. Ströhle |
  | Author | M.C. Jockers-Scherübl |
  | Abstract | Background: Animal-assisted intervention has become a common therapeutic practice used for patients with dementia in home-dwelling and institutions. The most established procedure is a visiting service by specially trained dogs and their owners to improve social interactions and reduce symptoms of agitation. Objectives: The study aims to investigate the effects of a therapy dog on agitation of inpatients with dementia in a gerontopsychiatric ward. Materials and Methods: The severity of agitation was assessed by a rater blinded for the presence of the dog via the Overt Agitation Severity Scale (OASS). The scale was conducted on 1 day with the dog and his handler present (resident doctor on the ward) and on another day with only the handler present. Each patient was his/her own control. Heart rate variability (HRV) and serum level of brain-derived neurotrophic factor (BDNF) of the patients were measured on both days. 26 patients with the Mini-Mental Status Examination (MMSE) score <21 and the diagnosis of dementia were included in the study. Results: A significant reduction of agitation in the OASS could be shown when the dog was present (p = 0.006). The data neither demonstrated a difference in the HRV for the parameters mean heart rate (p = 0.65), root mean square of successive differences (p = 0.63), and high frequencies (p = 0.27) nor in serum BDNF concentrations (p = 0.42). Discussion: Therapy dogs can be implemented as a therapeutic tool in a gerontopsychiatric ward to reduce symptoms of agitation in patients with dementia. The study was registered in the German Clinical Trials Register (DRKS00024093). |
  | Date | 2022 |
  | Language | English |
  | Archive | Embase |
  | URL | https://www.embase.com/search/results?subaction=viewrecord&id=L2016341600&from=export |
  | Volume | 50 |
  | Pages | 541-547 |
  | Publication | Dementia and Geriatric Cognitive Disorders |
  | DOI | 10.1159/000520881 |
  | Issue | 6 |
  | Journal Abbr | Dementia Geriatr. Cogn. Disord. |
  | ISSN | 1421-9824 |
  | Date Added | 05/02/2026, 17:53:32 |
  | Modified | 05/02/2026, 17:53:32 |

  ### Tags:

  - heart rate variability
  - heart rate
  - dog
  - article
  - female
  - male
  - nonhuman
  - human
  - aged
  - controlled study
  - clinical article
  - disease severity
  - pet therapy
  - dementia
  - brain derived neurotrophic factor
  - protein blood level
  - restlessness
  - single blind procedure
  - hospital patient
  - controlled clinical trial
  - antibiotic agent
  - antibiotic therapy
  - delirium
  - disease severity assessment
  - DRKS00024093
  - infection
  - milieu therapy
  - Mini Mental State Examination
  - pneumonia
  - psychiatric department

  ### Attachments

  - Full Text (HTML)
- ## Veterans Training Service Dogs for Other Veterans: An Animal-Assisted Intervention for Post-Traumatic Stress Disorder

  |  |  |
  | --- | --- |
  | Item Type | Journal Article |
  | Author | C.A. Krause-Parello |
  | Author | E. Friedmann |
  | Author | D. Taber |
  | Author | H. Zhu |
  | Author | A. Quintero |
  | Author | R. Yount |
  | Abstract | Research on the post-deployment reintegration needs of women veterans is limited. Non-traditional support may enhance mental health. Relationships with animals and volunteering may aid those with post-traumatic stress disorder (PTSD). Using the biopsychosocial model, we examined whether participation in an 8-week service dog training program (SDTP) affected telomere length (TL), heart rate variability (HRV), PTSD symptom severity, perceived stress, and anxiety in female veterans with PTSD, as well as whether combat exposure influenced these relationships. Female veterans (ages 32–72, M = 45.9, SD = 11.8) with PTSD were randomized to either the SDTP group (n = 13) or a comparison group (n = 15) that received dog training video content. The interventions lasted one hour weekly for 8 weeks. Outcomes were assessed pre-, mid-, and post-intervention. Linear mixed models with random intercepts examined changes from pre- to post-intervention and compared changes by group and combat exposure. TL changes differed [F(1,11.65) = 3.543, p = 0.085] by intervention. In the SDTP group, TL increased, indicating reduced cellular senescence (i.e., slower biological aging), whereas TL decreased in the CI group. Combat exposure moderated these changes [F(1,12.36) = 5.41, p = 0.038]. HRV changed by intervention group [F(1,389.08) = 10.623, p = 0.001]. HRV decreased (stress increased) in the SDTP group but not in the CI group. Combat exposure did not moderate HRV changes. PTSD symptom severity [F(1,48.04) = 19.22, p < 0.001], perceived stress [F(1,48.48) = 14.65, p < 0.001], and anxiety [F(1,47.30) = 6.624, p = 0.013] decreased significantly from pre- to post-interventions; the decreases did not differ by intervention or combat exposure. |
  | Date | 2025 |
  | Language | English |
  | Archive | Embase |
  | URL | https://www.embase.com/search/results?subaction=viewrecord&id=L2036363447&from=export |
  | Volume | 15 |
  | Publication | Behavioral Sciences |
  | DOI | 10.3390/bs15091180 |
  | Issue | 9 |
  | Journal Abbr | Behavioral Sciences |
  | ISSN | 2076-328X |
  | Date Added | 05/02/2026, 17:45:17 |
  | Modified | 05/02/2026, 17:45:17 |

  ### Tags:

  - veteran
  - heart rate variability
  - social support
  - heart rate
  - dog
  - anxiety
  - article
  - female
  - physiological stress
  - human
  - adult
  - aged
  - controlled study
  - posttraumatic stress disorder
  - human experiment
  - exercise
  - questionnaire
  - outcome assessment
  - clinical article
  - training
  - education
  - army
  - disease severity
  - depression
  - mental health
  - ethnicity
  - aging
  - general device
  - telomere
  - animal assisted intervention
  - intervention study
  - cognitive behavioral therapy
  - cell aging
  - eating disorder
  - general software
  - heart rate sensor
  - marriage
  - PCR assay kit
  - polar monitor
  - polar software
  - Polar V800 GPS Sports Watch
  - posttraumatic stress disorder symptom severity
  - PROMIS Anxiety Short Form 8a
  - PROMIS v2.0
  - psychoeducation
  - race
  - residence characteristics
  - RR interval
  - senescence
  - smart watch
  - telomere length

  ### Attachments

  - Full Text (HTML)
- ## Are therapy animals the key to happier dental visits for children?

  |  |  |
  | --- | --- |
  | Item Type | Journal Article |
  | Author | S. Khan |
  | Abstract | A COMMENTARY ON: Massouda J, Ghaltakhchyan N, Judd J, Bocklage C, Selden R, TumSuden O, Nanney E, Lee J, Ginnis J, Strauman T, Sawicki C, Hodges EA, Graves C, Divaris K, Jacox L. Evaluating effects of animal-assisted therapy on paediatric dental care patients: A pilot clinical trial. J Am Dent Assoc. 2025;156:447-457. https://doi.org/10.1016/j.adaj.2025.03.006 DATA SOURCES: This commentary is based on the published pilot clinical trial by Massouda et al. (2025) evaluating animal-assisted therapy (AAT) in paediatric dental care. STUDY SELECTION: The study included children aged 7-14 undergoing invasive dental procedures, allocated to either an AAT or control group. DATA EXTRACTION AND SYNTHESIS: Outcome measures included validated self-reported anxiety and pain scales, physiological stress markers, and behavioural observations. Data was synthesised narratively given pilot design and small sample size. DESIGN: A prospective, non-randomised pilot trial carried out in a university paediatric dental setting assessed the practicality and initial effects of integrating animal-assisted therapy (AAT) into dental treatment. CASE SELECTION: Thirty-nine children aged 7-14 years scheduled for invasive dental procedures were enroled. Participants were allocated to an AAT group (n = 18) or a control group (n = 21). Inclusion criteria included the ability to assent and a willingness to interact with a certified therapy animal. Exclusion criteria included previous traumatic experiences with AAT, or significant developmental or behavioural disorders affecting cooperation. DATA ANALYSIS: Physiological and psychological measures were recorded at baseline, during treatment, and post-operatively. Primary outcomes included self-reported pain and anxiety scores using validated scales. Secondary outcomes included heart rate monitoring, salivary cortisol, α-amylase, and video-coded behavioural relaxation. Given the small sample size, statistical analyses employed nonparametric 35 tests with significance set at P < 0.05. RESULTS: Children exposed to the therapy dog reported significantly lower postoperative pain scores (P = 0.001) and demonstrated smaller heart rate fluctuations during stressful procedural moments, suggesting lower physiological stress responses. Behavioural observations indicated longer periods of relaxed posture in the AAT group, though this did not reach statistical significance (P = 0.204). No significant differences were detected in salivary cortisol or α-amylase. AAT was reported as safe, well-tolerated, and feasible to implement. CONCLUSIONS: AAT shows potential for reducing distress in paediatric dental settings, but larger, randomised studies are required. Within the limits of a pilot design, animal-assisted therapy may reduce pain and physiological stress in paediatric dental patients. While encouraging, larger randomised trials are needed to confirm these effects, explore mechanisms, and assess practicality and cost in routine dental practice. |
  | Date | 2025 |
  | Language | English |
  | Archive | Medline |
  | URL | https://www.embase.com/search/results?subaction=viewrecord&id=L649474030&from=export |
  | Volume | 26 |
  | Pages | 174-175 |
  | Publication | Evidence-based dentistry |
  | DOI | 10.1038/s41432-025-01197-6 |
  | Issue | 4 |
  | Journal Abbr | Evid Based Dent |
  | ISSN | 1476-5446 |
  | Date Added | 05/02/2026, 17:58:23 |
  | Modified | 05/02/2026, 17:58:23 |

  ### Tags:

  - child
  - female
  - male
  - human
  - pilot study
  - adolescent
  - animal
  - psychology
  - animal assisted therapy
  - procedures
  - prospective study
  - dental anxiety
  - dental procedure
  - pain measurement
  - prevention and control

  ### Attachments

  - Full Text (HTML)
- ## Effects of animal-assisted therapy on dental anxiety, behavior, and perceptions in young pediatric patients: a blinded randomized controlled trial

  |  |  |
  | --- | --- |
  | Item Type | Journal Article |
  | Author | G. Kapov |
  | Author | K. Linton |
  | Author | C. Gatewood |
  | Author | C. Liu |
  | Author | T. Strauman |
  | Author | E. Hodges |
  | Author | C. Graves |
  | Author | C. Sawicki |
  | Author | D. Wu |
  | Author | K. Divaris |
  | Author | L.A. Jacox |
  | Abstract | Background: Between 6 and 22% of children are affected by dental anxiety. Dental anxiety is a significant barrier to dental care and is associated with dental avoidance and negative oral health outcomes. Pharmacological methods of anxiety management are costly, carry risks of adverse outcomes, and may not be acceptable to some families. Alternative non-pharmacological methods are needed for the safe and effective delivery of dental care. Although there is an abundance of literature regarding animal-assisted therapy (AAT) in medicine, only preliminary studies on AAT exist in dentistry. To identify optimal outcome measures for evaluating AAT in pediatric dental contexts, a randomized controlled trial protocol was developed. Methods: A prospective randomized controlled trial protocol was developed to examine the impact of AAT on objective (heart rate, salivary stress and pain markers, and observational coding) and subjective self-reported measures of anxiety, pain, and dental expectations in pediatric patients. The study is designed to enroll 180 pediatric patients (4–8 years old), randomized into three arms (n = 60 per arm) with stratification by age (< 6.5 vs ≥ 6.5) and gender (block size = 4). Two therapy protocols (+ Short AAT and + Long AAT exposures) will be compared relative to an active control (coloring a dog picture) during a diagnostic dental visit consisting of an oral exam, dental cleaning, and simulated bitewing intraoral radiographs. Discussion: This study will provide information on optimal outcome measures to evaluate the impact of AAT on dental anxiety and behavior in pediatric dental patients. Determining the effects of AAT in pediatric dental care may provide a safe, non-pharmacological method of anxiety and behavior management, with broad translational impact. Trial registration: This trial was registered on ClinicalTrials.gov with number NCT05464888, on 15 July 2022 (first submitted to ClinicalTrials.gov) and 19 July 2022 (first posted to ClinicalTrials.gov). |
  | Date | 2025 |
  | Language | English |
  | Archive | Embase |
  | URL | https://www.embase.com/search/results?subaction=viewrecord&id=L2035640937&from=export |
  | Volume | 26 |
  | Publication | Trials |
  | DOI | 10.1186/s13063-025-08970-z |
  | Issue | 1 |
  | Journal Abbr | Trials |
  | ISSN | 1745-6215 |
  | Date Added | 05/02/2026, 17:53:04 |
  | Modified | 05/02/2026, 17:53:04 |

  ### Tags:

  - behavior
  - heart rate
  - anxiety
  - child
  - article
  - human
  - controlled study
  - randomized controlled trial
  - major clinical study
  - outcome assessment
  - animal assisted therapy
  - perception
  - avoidance behavior
  - prospective study
  - pediatric dentistry
  - dental anxiety
  - dental procedure
  - dental prophylaxis
  - dentistry
  - NCT05464888
  - pediatric patient
  - single blind procedure
  - treatment protocol

  ### Attachments

  - Full Text (HTML)
- ## Companion Animals and Health in Older Populations: A Systematic Review

  |  |  |
  | --- | --- |
  | Item Type | Journal Article |
  | Author | M.J. Hughes |
  | Author | M.-L. Verreynne |
  | Author | P. Harpur |
  | Author | N.A. Pachana |
  | Abstract | OBJECTIVES: . The aim of this systematic literature review (SLR) was to investigate the effect of companion animals (whether simply as pets or used in more formal intervention approaches) on the physical and mental health of older adults (aged 60+). METHODS: . The reviewers identified key search terms and conducted a systematic search of the PsycINFO and PubMed databases. The 70 articles reviewed were evaluated through tabular and thematic analysis. RESULTS: . In 52 of the studies examined, companion animals positively contributed to the mental and/or physical health of older adults. With respect to mental health, involvement with a companion animal improved participant quality of life and effectively attenuated symptoms of depression, anxiety, cognitive impairment, and the behavioral and psychiatric symptoms of dementia (BPSD). In relation to physical health, marked increases in physical activity and improvements in blood pressure and heart rate variability were the only consistent physical health improvements observed from companion animal interactions. CONCLUSIONS: . Animal companionship can benefit the mental and physical health of older adults, although more and better controlled research on this topic is required. CLINICAL IMPLICATIONS: . Use of companion animals has the potential to be an effective treatment or adjunct therapy to improve the health status and quality of life of older individuals. |
  | Date | 2020 |
  | Language | English |
  | Archive | Medline |
  | URL | https://www.embase.com/search/results?subaction=viewrecord&id=L629108090&from=export |
  | Volume | 43 |
  | Pages | 365-377 |
  | Publication | Clinical gerontologist |
  | DOI | 10.1080/07317115.2019.1650863 |
  | Issue | 4 |
  | Journal Abbr | Clin Gerontol |
  | ISSN | 1545-2301 |
  | Date Added | 05/02/2026, 17:58:54 |
  | Modified | 05/02/2026, 17:58:54 |

  ### Tags:

  - anxiety
  - human
  - aged
  - pet animal
  - quality of life
  - animal
  - animal assisted therapy
  - mental health
  - cognitive defect

  ### Attachments

  - Full Text (HTML)
- ## Scoping review of the role of equine assisted psychotherapy and learning in opioid abuse treatment

  |  |  |
  | --- | --- |
  | Item Type | Journal Article |
  | Author | K. Holtcamp |
  | Author | K. Galarneau |
  | Author | M.C. Nicodemus |
  | Author | T. Phillips |
  | Author | D. Christiansen |
  | Author | B.J. Rude |
  | Author | P.L. Ryan |
  | Abstract | Opioid abuse is an epidemic in the United States with the number of deaths associated with this addiction growing each year, and yet, identification of a successful treatment plan is a major limitation to addressing this epidemic. Traditional treatment programs do not always fit for all addicts; however, equine assisted psychotherapy and learning (EAPL) shows promise as an alternative treatment option for those individuals where traditional treatment options have failed. Nevertheless, due to the novelty of this treatment option, literature concerning EAPL is limited. The purpose of this scoping review was to identify articles that address the characteristics and methodologies found in EAPL programs as it relates to opioid abuse treatment. Through the use of this scoping review, literature covering four key topics were gathered: 1) addiction treatment curriculums, 2) EAPL programs, 3) physiological effect of addiction treatment for humans, and 4) physiological effect of EAPL participation on the human and horse. Research associated with physiological effect was the most lacking of the topics covered during this review of literature. Reviewed literature pointed to a variety of populations that participate in addiction treatment programs that range from inpatient to long term recovery. EAPL programs offer an assortment of activity options to combat the challenges presented by the range of populations treated. Vital signs were mentioned in the literature reviewed as the leading way to track physiological changes but were not discussed in relation to addiction-specific treatment. Physiological measures showed change in both horses and humans who participated in equine programming but did not dictate concrete cause and effect instances. The limited literature identified through this scoping review validates the need to further explore the intentional relationship between EAPL and opioid abuse treatment programming. |
  | Date | 2024 |
  | Language | English |
  | Archive | Embase |
  | URL | https://www.embase.com/search/results?subaction=viewrecord&id=L2033496881&from=export |
  | Volume | 74 |
  | Pages | 1-10 |
  | Publication | Journal of Veterinary Behavior |
  | DOI | 10.1016/j.jveb.2024.06.010 |
  | Issue | (Holtcamp K.; Galarneau K.; Christiansen D.) College of Veterinary Medicine, Mississippi State University, Mississippi State, Mississippi, United States |
  | Journal Abbr | J. Vet. Behav. |
  | ISSN | 1558-7878 |
  | Date Added | 05/02/2026, 17:58:37 |
  | Modified | 05/02/2026, 17:58:37 |

  ### Tags:

  - horse
  - article
  - nonhuman
  - human
  - hippotherapy
  - therapy effect
  - physiological process
  - patient participation
  - curriculum
  - data extraction
  - data quality assessment
  - equine assisted psychotherapy and learning
  - health program
  - medical literature
  - methodology
  - opiate addiction
  - physiological effect
  - scoping review
  - vital sign

  ### Attachments

  - Full Text (HTML)
- ## Reducing Anxiety and Stress among Youth in a CBT-Based Equine-Assisted Adaptive Riding Program

  |  |  |
  | --- | --- |
  | Item Type | Journal Article |
  | Author | K. Hoagwood |
  | Author | A. Vincent |
  | Author | M. Acri |
  | Author | M. Morrissey |
  | Author | L. Seibel |
  | Author | F. Guo |
  | Author | C. Flores |
  | Author | D. Seag |
  | Author | R. Peth Pierce |
  | Author | S. Horwitz |
  | Abstract | Reining in Anxiety (RiA) is a therapeutic program for youth with mild to moderate anxiety delivered in a therapeutic riding setting by Certified Therapeutic Riding Instructors. RiA was developed after a review of the evidence base for youth anxiety, is manualized, and includes five core CBT components: in vivo exposure, cognitive restructuring, youth psychoeducation, relaxation, and caregiver psychoeducation about anxiety. This study extended findings from a prior RCT that examined (1) the feasibility of collecting saliva samples from horses and children to measure stress (cortisol) and relaxation (oxytocin); (2) whether changes in stress and relaxation occurred both during each lesson and over the course of the 10-week intervention for horses and youth; (3) whether changes in anxiety symptoms, emotional regulation, and self-efficacy found in the first trial were comparable; and (4) if fidelity to the program was reliable. Youth participants (n = 39) ages 6–17 with caregiver-identified mild-to-moderate anxiety participated in a ten-week therapeutic intervention (RiA), which combined adaptive riding and components of CBT. Physiological data and self-report measures were taken at weeks one, four, seven, and ten for the youth and horses. Saliva assays assessed cortisol as a physiological marker of stress and anxiety, and oxytocin as a measure of relaxation. Fidelity data were recorded per session. Anxiety, as measured by caregiver self-reporting, significantly decreased from pre- to post-test, while emotional regulation scores increased. No significant changes in self-efficacy from pre- to post-test were observed. Saliva samples obtained from participants before and after riding sessions showed a consistent decrease in cortisol and a significant increase in oxytocin at two of the four timepoints (Week 1 and Week 7), but no overall pre- to post-test changes. Horse saliva data were collected using a modified bit; there were no significant changes in oxytocin or cortisol, suggesting that the horses did not have an increase in stress from the intervention. RiA may be a promising approach for reducing anxiety and stress among youth, as measured both by self-reported and by physiological measures. Collection of salivary assays for both youth and horses is feasible, and the intervention does not increase stress in the horses. Importantly, RiA can be delivered by adaptive/therapeutic horseback riding instructors in naturalistic (e.g., non-clinic-based) settings. As youth anxiety is a growing public health problem, novel interventions, such as RiA, that can be delivered naturalistically may have the potential to reach more youth and thus improve their quality of life. Further research is needed to examine the comparative value of RiA with other animal-assisted interventions and to assess its cost-effectiveness. |
  | Date | 2022 |
  | Language | English |
  | Archive | Embase |
  | URL | https://www.embase.com/search/results?subaction=viewrecord&id=L2019524640&from=export |
  | Volume | 12 |
  | Publication | Animals |
  | DOI | 10.3390/ani12192491 |
  | Issue | 19 |
  | Journal Abbr | Animals |
  | ISSN | 2076-2615 |
  | Date Added | 05/02/2026, 17:58:46 |
  | Modified | 05/02/2026, 17:58:46 |

  ### Tags:

  - oxytocin
  - saliva
  - anxiety
  - emotion regulation
  - article
  - physiological stress
  - human
  - hydrocortisone
  - hippotherapy
  - quality of life
  - caregiver
  - outcome assessment
  - training
  - mental health
  - distress syndrome
  - pretest posttest design
  - public health
  - cognitive behavioral therapy
  - psychoeducation
  - feasibility study
  - psychosocial intervention
  - burnout
  - cost effectiveness analysis
  - generalized anxiety disorder
  - hypothalamus
  - interrater reliability
  - mental health service
  - psychometry
  - public health problem

  ### Attachments

  - Full Text (HTML)
- ## Child horse harmony in motion: a preliminary study to explore heart rate synchronization in equine assisted therapy for neurotypical and ADHD children

  |  |  |
  | --- | --- |
  | Item Type | Journal Article |
  | Author | A. Helmer |
  | Author | A. Hacohen |
  | Author | O. Bart |
  | Abstract | Equine-Assisted Services (EAS) encompass a range of therapeutic interventions utilizing equine interactions to achieve therapeutic goals. This study explores heart rate synchronization between horses and riders during mounted and unmounted interactions, focusing on its potential implications for emotional regulation. A total of 25 participants aged 6-12 took part in the study, which included two groups: novice riders diagnosed with Attention Deficit Hyperactivity Disorder (ADHD) (n = 15) and experienced neurotypical riders (n = 10). Heart rate measurements were obtained using Polar® Equine and Verity Sense Optical Heart Rate Sensors. Results indicate mutual heart rate synchronization between horses and riders, suggesting a potential mechanism for emotional regulation. The neurotypical group showed high levels of synchronization, suggesting that rider experience influences the physiological connection between horse and rider., while notably, children with ADHD demonstrated above-average synchronization by their fourth to sixth EAS session. These findings underscore the significance of EAS in promoting physiological and emotional well-being, particularly for individuals with ADHD. This study contributes to the understanding of the physiological mechanisms underlying therapeutic effect of EAS interventions and highlights their potential in clinical practice. Further research is needed to examine the mechanisms, stability, and therapeutic significance of horse-human physiological synchronization over time, including how synchronization patterns evolve with rider experience and influence therapeutic outcomes in children with ADHD. |
  | Date | 2025 |
  | Language | English |
  | Archive | Medline |
  | URL | https://www.embase.com/search/results?subaction=viewrecord&id=L649322714&from=export |
  | Volume | 15 |
  | Pages | 45312 |
  | Publication | Scientific reports |
  | DOI | 10.1038/s41598-025-29330-6 |
  | Issue | 1 |
  | Journal Abbr | Sci Rep |
  | ISSN | 2045-2322 |
  | Date Added | 05/02/2026, 17:58:23 |
  | Modified | 05/02/2026, 17:58:23 |

  ### Tags:

  - horse
  - physiology
  - heart rate
  - therapy
  - emotion
  - child
  - female
  - male
  - human
  - hippotherapy
  - animal
  - psychology
  - attention deficit hyperactivity disorder
  - procedures
  - pathophysiology

  ### Attachments

  - Full Text (HTML)
  - PDF
- ## Protocol for a systematic review and meta-analysis on the effect of hippotherapy and related equine-assisted therapies on motor capabilities in children with cerebral palsy

  |  |  |
  | --- | --- |
  | Item Type | Journal Article |
  | Author | M. Häusler |
  | Author | N. Heussen |
  | Abstract | Background: Equine-assisted treatments of the motor system appear to have an effect on the neuromuscular system and aim to improve the pathological condition of children with cerebral palsy. Hippotherapy is a distinct form of equine-assisted therapy where certified physiotherapists use the horse as a dynamic tool in a medical treatment setting. The objective of the proposed review is to summarize and critically appraise the evidence on the effect of equine-assisted treatments on motor capabilities of children with cerebral palsy. Methods: We will identify trials through systematic searches of PubMed, Embase, Web of Science, and the Cochrane Central Register of Controlled Trials (CENTRAL). Quality assessment of retrieved articles will be conducted using the criteria outlined in the revised tool to assess risk of bias in randomized trials (RoB 2.0) or the ROBINS-I tool (Risk Of Bias In Non-randomized Studies - of interventions), respectively. Quantitative data synthesis will be performed if treatments, participants, and the underlying clinical question are homogenous and provide adequate outcome data for meta-analysis. Otherwise, data will be synthesized, using the narrative synthesis approach. Conclusion: This review will provide a critical summary of the evidence regarding the impact of equine-assisted treatments on motor capabilities of children with cerebral palsy. The result from this review will help to inform healthcare practitioners and policymakers on the additional effect of equine-assisted treatments on reducing the burden of cerebral palsy among children. Systematic review registration: This systematic review protocol is registered with the International Prospective Register of Systematic Reviews (PROSPERO). Registration number: CRD42018096403. This protocol was prepared using the Preferred Reporting Items for Systematic Reviews and Meta-Analyses for Protocols checklist (PRISMA-P). |
  | Date | 2020 |
  | Language | English |
  | Archive | Embase |
  | URL | https://www.embase.com/search/results?subaction=viewrecord&id=L631125335&from=export |
  | Volume | 9 |
  | Publication | Systematic Reviews |
  | DOI | 10.1186/s13643-020-01297-7 |
  | Issue | 1 |
  | Journal Abbr | Syst. Rev. |
  | ISSN | 2046-4053 |
  | Date Added | 05/02/2026, 17:58:58 |
  | Modified | 05/02/2026, 17:58:58 |

  ### Tags:

  - horse
  - heart rate variability
  - heart rate
  - physical activity
  - article
  - human
  - follow up
  - hippotherapy
  - quality of life
  - cerebral palsy
  - motor performance
  - outcome assessment
  - physiotherapist
  - randomized controlled trial (topic)
  - systematic review
  - priority journal
  - comparative study
  - psychometry
  - Gross Motor Function Classification System
  - meta analysis
  - mortality
  - motor system
  - neuromuscular system
  - Newcastle-Ottawa scale
  - quality control

  ### Attachments

  - Full Text (HTML)
- ## Group Changes in Cortisol and Heart Rate Variability of Children with Down Syndrome and Children with Autism Spectrum Disorder during Dog-Assisted Therapy

  |  |  |
  | --- | --- |
  | Item Type | Journal Article |
  | Author | R.E. Griffioen |
  | Author | G.J.M. van Boxtel |
  | Author | T. Verheggen |
  | Author | M.-J. Enders-Slegers |
  | Author | S. Van Der Steen |
  | Abstract | Dog-assisted therapy is hypothesized to lower stress in children with autism spectrum disorder (ASD) and children with Down syndrome (DS), which may be visible on a physiological level. In this study, we measured heart rate variability (HRV) and salivary cortisol of 20 children with DS or ASD at the beginning and end of six weekly sessions of dog-assisted therapy. We found a decrease of cortisol levels during single sessions, but no overall effect after six sessions (six weeks). The effect of dog-assisted therapy on the increase of HRV could not be confirmed. This study is one of the first to use physiological measurements to test the effects of DAT. |
  | Date | 2023 |
  | Language | English |
  | Archive | Embase |
  | URL | https://www.embase.com/search/results?subaction=viewrecord&id=L2024748133&from=export |
  | Volume | 10 |
  | Publication | Children |
  | DOI | 10.3390/children10071200 |
  | Issue | 7 |
  | Journal Abbr | Child. |
  | ISSN | 2227-9067 |
  | Date Added | 05/02/2026, 17:58:43 |
  | Modified | 05/02/2026, 17:58:43 |

  ### Tags:

  - heart rate variability
  - child
  - article
  - female
  - male
  - human
  - hydrocortisone
  - controlled study
  - adolescent
  - autism
  - mental stress
  - treatment outcome
  - clinical article
  - animal assisted therapy
  - therapy effect
  - saliva level
  - school child
  - saliva analysis
  - heart rate measurement
  - time factor
  - pediatric patient
  - dog assisted therapy
  - Down syndrome
  - hydrocortisone saliva level
  - treatment duration

  ### Attachments

  - Full Text (HTML)
- ## Well-Being Indicators in Autistic Children and Therapy Dogs During a Group Intervention: A Pilot Study

  |  |  |
  | --- | --- |
  | Item Type | Journal Article |
  | Author | V.O. Giuliano |
  | Author | L. Sacchettino |
  | Author | A.S. Rusu |
  | Author | D. Ciccarelli |
  | Author | V. Gazzano |
  | Author | M. de Cesare |
  | Author | M. Visone |
  | Author | V. Mizzoni |
  | Author | F. Napolitano |
  | Author | D. d’Angelo |
  | Abstract | Animal-assisted services (AAS) have been shown in multiple studies to improve a range of human psychological and physical health benefits. The aim of this pilot study is to investigate simultaneously two psycho-physiological indicators of the valence of interactions in the context of dog-assisted activities in children diagnosed with autism spectrum disorder. Ten children and four dogs experienced in AAS were involved, lasting 90 days, in weekly one-hour sessions. Before and after each session, saliva was taken in both dogs and children for determination of salivary oxytocin and cortisol levels. In addition, at the end of the program, a questionnaire was administered to both parents and dog handlers to assess the impact of AAS in children and dogs. Our results revealed no statistically significant change in cortisol and oxytocin levels in dogs enrolled throughout the sessions, while an increasing trend was noted for salivary oxytocin in 50% of the dogs and for salivary cortisol in all dogs at the end of the AAS, when compared to the pre-AAS. Salivary cortisol measurement in children with an autistic neurotype highlighted a statistically significant increase at the end of the AAS when compared to the pre-AAS, but this was not observed for oxytocin level evaluations. Regarding the perception of the children’s parents about the effects of the program, our data reported an improvement in sociability of the children in 100 percent of the cases. Furthermore, dog handlers reported an absence of signs of stress in their dogs during the sessions. Although the perceived effectiveness and quality of AAS has been demonstrated in the literature, the need to carefully select the dogs involved, considering their skills and needs, is critical to ensure their well-being in various therapeutic settings. |
  | Date | 2025 |
  | Language | English |
  | Archive | Embase |
  | URL | https://www.embase.com/search/results?subaction=viewrecord&id=L2035477463&from=export |
  | Volume | 15 |
  | Publication | Animals |
  | DOI | 10.3390/ani15142032 |
  | Issue | 14 |
  | Journal Abbr | Animals |
  | ISSN | 2076-2615 |
  | Date Added | 05/02/2026, 17:58:26 |
  | Modified | 05/02/2026, 17:58:26 |

  ### Tags:

  - oxytocin
  - behavior
  - dog
  - saliva
  - emotion
  - child
  - article
  - female
  - male
  - nonhuman
  - physiological stress
  - human
  - hydrocortisone
  - controlled study
  - pilot study
  - quality of life
  - questionnaire
  - autism
  - clinical article
  - training
  - cotton swab
  - saliva collector
  - Salivette
  - psychoeducation
  - animal-assisted service
  - sociability

  ### Attachments

  - Full Text (HTML)
- ## Hippotherapy in the Treatment of CMD and Bruxism in Dentistry

  |  |  |
  | --- | --- |
  | Item Type | Journal Article |
  | Author | M.-A. Geibel |
  | Author | D. Kildal |
  | Author | A.M. Geibel |
  | Author | S. Ott |
  | Abstract | Dysfunctions and disorders of the craniomandibular system are accompanied by pathophysiological changes of muscle groups in the throat/neck and facial area, e.g., pain in the jaw and muscles of mastication and disturbance of occlusion, leading to teeth injury (loss of dental hard tissue, fractures/sensibility disorders, etc.). For muscular dysfunctions, even in the context of psychosomatic disorders and chronic stress, hippotherapy is particularly suitable, since it helps actively to relieve muscle tensions. In the current project we combined hippotherapy with progressive muscle relaxation (PMR) to achieve a synergistic effect. The horses used for therapy (two mares and five geldings between seven and twenty-one years old) were especially suitable because of their calm temperament. In two cases, trained therapy horses were used; in five other cases, the patients used their own horses, which were not specially trained. Right from the beginning, the project was accompanied by veterinary support. Conditions of horse keeping (active stable, same-sex groups, no boxes) were assessed as well as the horses themselves prior to, during, and after each therapy unit. In patients, cortisol, as a quantifiable parameter for stress, was measured before and after each therapy unit. From before the start until the end of each therapy unit of 15 min, the heart rate variability (HRV) of both patients and horses was registered continuously and synchronously. In addition, the behavior of the horses was monitored and recorded on video by an experienced coach and a veterinarian. The stress load during the tension phases in the therapy units was low, perceivable in the horses lifting their heads and a slightly shortened stride length. Likewise, the horses reflected the patients’ relaxation phases, so that at the end of the units the horses were physically and psychically relaxed, too, noticeable by lowering their necks, free ear movement, and a decreasing heart frequency (HF). Altogether, the horses benefited from the treatment, too. Obvious stress signs like unrest, head tossing, tail swishing, or tense facial expressions were not noticed at any time. Twenty jumpers served as a control group in different situations (training, tournament, and leisure riding). |
  | Date | 2025 |
  | Language | English |
  | Archive | Embase |
  | URL | https://www.embase.com/search/results?subaction=viewrecord&id=L2036075732&from=export |
  | Volume | 15 |
  | Publication | Animals |
  | DOI | 10.3390/ani15172587 |
  | Issue | 17 |
  | Journal Abbr | Animals |
  | ISSN | 2076-2615 |
  | Date Added | 05/02/2026, 17:58:24 |
  | Modified | 05/02/2026, 17:58:24 |

  ### Tags:

  - horse
  - heart rate
  - anxiety
  - article
  - female
  - male
  - physiological stress
  - human
  - hydrocortisone
  - adult
  - follow up
  - hippotherapy
  - physiotherapy
  - exercise
  - quality of life
  - chronic stress
  - temperament
  - dentistry
  - bruxism
  - cognitive behavioral therapy
  - enamel
  - mastication
  - muscle relaxation
  - muscle tone

  ### Attachments

  - Full Text (HTML)
- ## Distraction-focused interventions on examination stress in nursing students: Effects on psychological stress and biomarker levels. A randomized controlled trial

  |  |  |
  | --- | --- |
  | Item Type | Journal Article |
  | Author | V. Gebhart |
  | Author | W. Buchberger |
  | Author | I. Klotz |
  | Author | S. Neururer |
  | Author | C. Rungg |
  | Author | G. Tucek |
  | Author | C. Zenzmaier |
  | Author | S. Perkhofer |
  | Abstract | BACKGROUND: Nursing students all over the world experience high levels of stress with negative impacts on their health, emotional state and performance. AIM: This study aimed to investigate the effects of distraction-focused interventions on examination stress and anxiety in nursing students. METHODS: A randomized controlled, parallel trial design was conducted from January to June 2016. After baseline measurement, 72 participants were randomized to one of the following groups (n = 18 each): (i) animal-assisted therapy; (ii) music therapy; (iii) mandala painting; (iv) control group. Outcomes of all groups in terms of stress-reduction were compared by measuring self-reported perceived stress (STAI-State and visual analogue stress scale) and salivary biomarker levels (Cortisol and Immunoglobulin A). RESULTS: Fifty-seven complete data sets (n = 12-16 for each group) were analysed. All distraction-focused interventions showed stress and anxiety reduction in everyday school situations. By contrast, on days with examinations, stress reductions did not reach statistical significance in regard to self-reported psychological stress. At the same time, interventions resulted in significantly decreased levels of stress biomarkers (P < .001). CONCLUSIONS: Our preliminary findings suggest positive but situation-dependent effects of distraction-focused interventions in academic settings. Further research should investigate the complex relationship between physiological and psychological stress parameters. |
  | Date | 2020 |
  | Language | English |
  | Archive | Medline |
  | URL | https://www.embase.com/search/results?subaction=viewrecord&id=L629865075&from=export |
  | Volume | 26 |
  | Pages | e12788 |
  | Publication | International journal of nursing practice |
  | DOI | 10.1111/ijn.12788 |
  | Issue | 1 |
  | Journal Abbr | Int J Nurs Pract |
  | ISSN | 1440-172X |
  | Date Added | 05/02/2026, 17:58:59 |
  | Modified | 05/02/2026, 17:58:59 |

  ### Tags:

  - saliva
  - anxiety
  - female
  - male
  - human
  - hydrocortisone
  - adult
  - controlled study
  - randomized controlled trial
  - adolescent
  - mental stress
  - psychology
  - young adult
  - middle aged
  - biological marker
  - animal assisted therapy
  - immunoglobulin A
  - education
  - metabolism
  - prevention and control
  - music therapy
  - art therapy
  - nursing student

  ### Attachments

  - Full Text (HTML)
- ## Equine-assisted therapeutic activities and their influence on the heart rate variability: A systematic review

  |  |  |
  | --- | --- |
  | Item Type | Journal Article |
  | Author | A. García-Gómez |
  | Author | E. Guerrero-Barona |
  | Author | I. García-Peña |
  | Author | M. Rodríguez-Jiménez |
  | Author | J.M. Moreno-Manso |
  | Abstract | OBJECTIVE: To examine the effect of equine-assisted therapeutic interventions on users' heart rate variability, using this said variability as an objective biological variable related to stress levels. METHOD: A systematic review has been carried out using the methodology suggested in the PRISMA declaration following systematic searches in academic databases. RESULTS: 432 registers were initially identified; however, in the screening and suitability process, nine papers were included in the review. With one exception, all of them reported that equine-assisted therapeutic activities had a favourable effect on users' heart rate variability as such activities favour a state of relaxation by activating the Parasympathetic Nervous System. The analysis of the quality of the evidence and the confirmation of the bias in the works indicate that these results must be considered with caution. DISCUSSION: Although these preliminary results are promising, more rigorous clinical trials are necessary to overcome the methodological limitations of the works. |
  | Date | 2020 |
  | Language | English |
  | Archive | Medline |
  | URL | https://www.embase.com/search/results?subaction=viewrecord&id=L631706208&from=export |
  | Volume | 39 |
  | Pages | 101167 |
  | Publication | Complementary therapies in clinical practice |
  | DOI | 10.1016/j.ctcp.2020.101167 |
  | Issue | (García-Gómez A.) Department of Educational Sciences, University of Extremadura, Spain |
  | Journal Abbr | Complement Ther Clin Pract |
  | ISSN | 1873-6947 |
  | Date Added | 05/02/2026, 17:58:57 |
  | Modified | 05/02/2026, 17:58:57 |

  ### Tags:

  - horse
  - autonomic nervous system
  - physiology
  - heart rate
  - therapy
  - human
  - animal
  - mental stress
  - animal assisted therapy
  - procedures

  ### Attachments

  - Full Text (HTML)
- ## Ultrasonographic examination of equine fetal growth parameters throughout gestation in pony for Equine-Assisted Therapy

  |  |  |
  | --- | --- |
  | Item Type | Journal Article |
  | Author | Y. Gao |
  | Author | M.A. Hannan |
  | Author | K. Murata |
  | Author | R. Rajabi-Toustani |
  | Author | Y. Nambo |
  | Abstract | Equine-Assisted Therapy (EAT) is gaining popularity. Ultrasound examination is used to decrease the abortion rate in horses. In this study, to monitor fetal well-being throughout the gestation for EAT, we measured fetal heart rate (FHR), fetal eye orbit (FEO), fetal gonad length (FGL), fetal kidney length (FKL), and the combined thickness of the uterus and placenta (CTUP) by ultrasonography in pony mares. Additionally, we measured the plasma progesterone (P4) and estradiol (E2) concentrations in pregnant horses using enzyme immunoassay. The FGL peaked at week 32 and then decreased to term, and a strong correlation (r=0.72, P<0.001) between the FGL and E2 concentration was observed. A strong correlation with gestational age was detected among the FEO (r=0.96, P<0.001), FKL (r=0.85, P<0.001), and CTUP (r=0.96, P<0.001). The P4 concentration peaked at week 10, decreased to low levels (below 5 ng/ml), and peaked before parturition. In conclusion, this study provides information on fetal growth throughout gestation in pony mares for EAT. In addition, it revealed the relationship between ultrasonographic profile and plasma hormone concentrations during gestation. |
  | Date | 2022 |
  | Language | English |
  | Archive | Embase |
  | URL | https://www.embase.com/search/results?subaction=viewrecord&id=L2015213259&from=export |
  | Volume | 84 |
  | Pages | 74-81 |
  | Publication | Journal of Veterinary Medical Science |
  | DOI | 10.1292/jvms.21-0301 |
  | Issue | 1 |
  | Journal Abbr | J. Vet. Med. Sci. |
  | ISSN | 1347-7439 |
  | Date Added | 05/02/2026, 17:58:50 |
  | Modified | 05/02/2026, 17:58:50 |

  ### Tags:

  - animal experiment
  - article
  - female
  - nonhuman
  - hippotherapy
  - mare
  - enzyme immunoassay
  - pregnancy
  - artificial insemination
  - embryo transfer
  - echography
  - convex transducer
  - estradiol blood level
  - fetal gonad length
  - fetal well being
  - fetus eye
  - fetus growth
  - fetus heart rate
  - fetus kidney
  - gestational age
  - hormone blood level
  - Noblus
  - placenta
  - placenta thickness
  - pony
  - progesterone blood level
  - rectal ultrasound transducer
  - ultrasound transducer
  - uterus
  - uterus thickness

  ### Attachments

  - Full Text (HTML)
- ## The Impact of Animal-Assisted Therapy on Changes in Autonomic Nervous Activity in Terminal Cancer Patients

  |  |  |
  | --- | --- |
  | Item Type | Journal Article |
  | Author | H. Fujisawa |
  | Author | K. Yamamura |
  | Abstract | Purpose: This study aimed to objectively verify the effectiveness of Animal-assisted therapy (AAT) as a complementary and alternative therapy based on its effects. Design: This was a cross-sectional, observational, and nonrandomized comparative study conducted. Materials and Methods: Changes in autonomic nerve activity, visual analog scale (VAS), interviews with patients, and general condition of patients and healthy individuals were analyzed. Additionally, differences in the patterns were analyzed between the two groups. Result: 10 patients and 9 healthy individuals were included in this study. The low frequency to high frequency (LF/HF) ratio showed a downward trend in the patient group. High frequency in normalized units (HFnu) showed a significant increase in the patient group. A comparison of the change patterns of HFnu values between the patient and healthy groups showed significant changes in the patient group. Discussion: These results demonstrate that AAT can relieve mental distress in patients with terminal cancer. These findings indicate that AAT is a complementary and alternative therapy that can improve the Quality of life (QOL) of patients with terminal cancer and support palliative care. Conclusion: AAT has the effect of reducing pain and maintaining and improving the QOL of patients with terminal cancer, even in the short period before death. |
  | Date | 2024 |
  | Language | English |
  | Archive | Embase |
  | URL | https://www.embase.com/search/results?subaction=viewrecord&id=L2035239213&from=export |
  | Volume | 31 |
  | Pages | 169-173 |
  | Publication | International Medical Journal |
  | Issue | 6 |
  | Journal Abbr | Int. Med. J. |
  | ISSN | 2436-3294 |
  | Date Added | 05/02/2026, 17:58:41 |
  | Modified | 05/02/2026, 17:58:41 |

  ### Tags:

  - heart rate
  - physical activity
  - article
  - coronavirus disease 2019
  - female
  - male
  - human
  - data analysis software
  - oxygen saturation
  - normal human
  - electrocardiography
  - visual analog scale
  - electrocardiogram
  - diastolic blood pressure
  - systolic blood pressure
  - animal assisted therapy
  - observational study
  - diabetes mellitus
  - autonomic nervous system function
  - comparative study
  - hemodialysis
  - cross-sectional study
  - analysis of variance
  - alternative medicine
  - autonomic nerve
  - cerebrovascular disease
  - colorectal cancer
  - consciousness
  - disease exacerbation
  - heart disease
  - hypotension
  - liver cell carcinoma
  - lung cancer
  - pain assessment
  - Parkinson disease
  - patient satisfaction
  - SPSS version 23
  - stomach cancer
- ## How the presence of a dog and types of interaction affect physiological responses to experimental heat pain induction in healthy humans - a randomized controlled study

  |  |  |
  | --- | --- |
  | Item Type | Journal Article |
  | Author | L.H. Fuglsang-Damgaard |
  | Author | S.J. Lunde |
  | Author | J.W. Christensen |
  | Author | L. Vase |
  | Author | P.B. Videbech |
  | Author | N.R. Gee |
  | Author | K. Thodberg |
  | Abstract | It has become increasingly popular to include dogs as a complement to regular therapy, with the expectation that they offer, among other benefits, pain-relieving effects. Meanwhile, studies covering the topic of painful situations within the field of animal-assisted interventions (AAI) present conflicting results and rarely consider the type and duration of interaction with the dog. Thus, the impact of human-dog interactions on physiological measurements during painful situations is largely unknown. Basic research is needed on the effects of interacting with a dog, using commonly applied immediate physiological measurements in healthy humans during experimental pain induction to fill this gap in the literature. The present study investigated how AAI influences physiological measurements when healthy humans are subjected to experimental heat pain induction. Simultaneously, the study explored how the duration of different types of interaction with a human companion or a dog as well as dog behavior during experimental heat pain induction affected physiological measurements. Fifty-eight healthy participants (14 men, 44 women, age: 18-66 years) were randomly assigned to one of two intervention groups: 1) a dog and a human companion or 2) a human companion only. Both intervention groups underwent two test conditions in a balanced order: an active test condition with their allocated intervention and a control test condition without their allocated intervention. The participants were exposed to a 5-minute heat pain induction trial in both test conditions with a 20-minute break between trials. Heart rate (HR), heart rate variability (HRV) and skin conductance (SC), analyzed as tonic level (SCL) and peak counts (SCR), were continuously recorded. Blood pressure (BP) and salivary cortisol (s-cortisol) were collected as pre- and post-measurements for each test condition. Behavioral interactions between the participant, dog and human companion as well as behavior of the dog were recorded and the influence of the behavioral interactions on each physiological measure was analyzed. Linear Mixed Models were applied. HR was higher for the intervention group with a dog and a human companion compared to a human companion only (p=0.013). Additionally, within-subject comparison showed an increased HR during the active condition compared to the control condition in both intervention groups (dog and human companion: p<0.001 and human companion only: p=0.025). None of the other physiological measurements were influenced by the mere presence of a dog in either the between- nor within-subject comparisons. Within the human companion only group, SCL and SCR were higher during the active condition compared to the control (both p<0.001). The duration of the behavioral interaction between the participant and human companion variously influenced HRV, SBP, s-cortisol and SCL during the active condition in both intervention groups. Further, the duration of the behavioral interactions with the dog and dog behavior variously influenced HR, HRV, s-cortisol and SCL during the active condition in the human companion and the dog intervention group. In conclusion, this study shows that the presence of a dog in addition to a human companion during experimental heat pain induction results in an increased HR compared to the presence of a human companion only. Thus, despite previously reported pain-relieving effects of the presence of a dog, this study suggests that dog presence may also induce a certain level of arousal and further studies are needed to explore the causal mechanisms. Interactions with a dog or a human companion influenced several physiological measurements and it is therefore important to quantify the type and duration of human-animal interaction in AAI-studies. |
  | Date | 2025 |
  | Language | English |
  | Archive | Embase |
  | URL | https://www.embase.com/search/results?subaction=viewrecord&id=L2040407909&from=export |
  | Volume | 302 |
  | Publication | Physiology and Behavior |
  | DOI | 10.1016/j.physbeh.2025.115097 |
  | Issue | (Fuglsang-Damgaard L.H., Fuglsang@anivet.au.dk; Christensen J.W.; Thodberg K.) Department of Animal and Veterinary Sciences, Aarhus University, Denmark |
  | Journal Abbr | Physiol. Behav. |
  | ISSN | 1873-507X |
  | Date Added | 05/02/2026, 17:58:19 |
  | Modified | 05/02/2026, 17:58:19 |

  ### Tags:

  - oxytocin
  - human-animal interaction
  - heart rate variability
  - heart rate
  - dog
  - animal behavior
  - animal experiment
  - article
  - female
  - male
  - nonhuman
  - human
  - hydrocortisone
  - adult
  - controlled study
  - pilot study
  - randomized controlled trial
  - silver electrode
  - body position
  - human experiment
  - normal human
  - behavioral observation
  - electrodermal response
  - visual analog scale
  - electrocardiogram
  - enzyme linked immunosorbent assay
  - diastolic blood pressure
  - systolic blood pressure
  - animal assisted therapy
  - State Trait Anxiety Inventory
  - saliva level
  - blood pressure measurement
  - avoidance behavior
  - additive effect
  - basic research
  - body mass
  - displacement behavior
  - electrocardiograph electrode
  - experimental pain
  - heat hyperalgesia
  - NCT04866173
  - pain intensity
  - skin conductance
  - upper arm

  ### Attachments

  - Full Text (HTML)
- ## The Effect of Therapy Dogs on Preoperative Anxiety

  |  |  |
  | --- | --- |
  | Item Type | Journal Article |
  | Author | P. Foerder |
  | Author | M. Royer |
  | Abstract | Animal-assisted interventions have been used to improve human psychological and physiological wellbeing. Research studies on the effects of therapy dogs have found that the companionship of a dog significantly decreases stress levels in many different situations, including health care facilities. We investigated the effects of therapy dogs on adults’ anxiety before going into day surgery. Participants were patients from a Surgical Ambulatory Care Unit. Four conditions were compared to examine the effects of dogs on the patients’ stress: a therapy dog and handler team, a stuffed dog and handler, a person without a dog, or no visitor. Before and after these visits, the heart rate and blood pressure of the patient was taken. Patients also completed the State-Trait Anxiety Inventory short form (STAI) and a brief demographics survey. No change was found in blood pressure or heart rate in any of the conditions. Based on the STAI scores, we found a statistically significant reduction in anxiety for any intervention over no visit at all. We also found that, although there was no statistical difference between the effects of the real dog and the stuffed toy dog, the stuffed dog condition lowered anxiety enough to show a statistically significant reduction compared with no visitor. Our research is one of the few studies to compare a live dog to a similar size and breed stuffed toy dog. Our results indicate that, in the absence of a real dog, a stuffed toy dog may have equivalent therapeutic value. Our study provides further knowledge on the effects of dog-assisted interventions on medical patients and indicates avenues for future research. |
  | Date | 2021 |
  | Language | English |
  | Archive | Embase |
  | URL | https://www.embase.com/search/results?subaction=viewrecord&id=L2011401725&from=export |
  | Volume | 34 |
  | Pages | 659-670 |
  | Publication | Anthrozoos |
  | DOI | 10.1080/08927936.2021.1914440 |
  | Issue | 5 |
  | Journal Abbr | Anthrozoos |
  | ISSN | 1753-0377 |
  | Date Added | 05/02/2026, 17:58:53 |
  | Modified | 05/02/2026, 17:58:53 |

  ### Tags:

  - blood pressure
  - human-animal interaction
  - heart rate
  - dog
  - animal experiment
  - article
  - female
  - male
  - nonhuman
  - physiological stress
  - human
  - adult
  - controlled study
  - animal model
  - wellbeing
  - health care facility
  - ambulatory care
  - ambulatory surgery
  - breed
  - demography
  - State Trait Anxiety Inventory
  - surgery
  - therapy effect

  ### Attachments

  - Full Text (HTML)
- ## Effects on Wellbeing of Exposure to Dog Videos Before a Stressor

  |  |  |
  | --- | --- |
  | Item Type | Journal Article |
  | Author | N. Ein |
  | Author | J. Gervasio |
  | Author | M.J. Reed |
  | Author | K. Vickers |
  | Abstract | Animal-assisted intervention (AAI) has been used as a means of stress relief in clinical and general settings; however, animals are not always allowed in certain spaces. Adapting AAI to video or virtual mediums could improve accessibility and is temporally relevant given the recent shift to online interventions. The current study explored: (1) whether an active video (dog or nature) watched before a stressor would improve wellbeing more than tranquil videos; (2) whether exposure to a dog video improves wellbeing more than a nature video; and (3) whether exposure to either a dog or nature video improves outcomes more than exposure to a control video. One hundred and seven undergraduates were randomly assigned to watch one of five videos (active dog, tranquil dog, active nature, tranquil nature, and control) for 3 minutes and then complete a 3-minute stress task. Subjective (anxiety, stress, happiness, relaxation, positive affect, and negative affect) and physiological (blood pressure and heart rate) outcomes were collected at baseline, video, stressor, and recovery time points. Results showed that the activity level of the dog in the video did not influence outcomes. However, relative to the control group, the dog-video condition showed decreases in stress from baseline to video and a smaller decrease in stress from stressor to recovery. Additionally, relative to the nature-video condition, the dog-video condition showed a slightly higher increase in happiness scores from baseline to video. Lastly, relative to the control group, the nature-video condition showed increased relaxation scores from baseline to video and a larger decrease in relaxation scores from video to stressor. This research may inform the development of alternate modes of AAIs. |
  | Date | 2023 |
  | Language | English |
  | Archive | Embase |
  | URL | https://www.embase.com/search/results?subaction=viewrecord&id=L2020468718&from=export |
  | Volume | 36 |
  | Pages | 349-367 |
  | Publication | Anthrozoos |
  | DOI | 10.1080/08927936.2022.2149925 |
  | Issue | 3 |
  | Journal Abbr | Anthrozoos |
  | ISSN | 1753-0377 |
  | Date Added | 05/02/2026, 17:58:44 |
  | Modified | 05/02/2026, 17:58:44 |

  ### Tags:

  - blood pressure
  - human-animal interaction
  - heart rate
  - dog
  - anxiety
  - animal experiment
  - article
  - nonhuman
  - physiological stress
  - human
  - controlled study
  - leisure
  - wellbeing
  - videorecording
  - happiness
  - web-based intervention

  ### Attachments

  - Full Text (HTML)
- ## The Effect of Dog Videos on Subjective and Physiological Responses to Stress

  |  |  |
  | --- | --- |
  | Item Type | Journal Article |
  | Author | N. Ein |
  | Author | M.J. Reed |
  | Author | K. Vickers |
  | Abstract | Research indicates that animal-assisted therapy programs can reduce stress responses. However, animals are not always permitted in public settings. Thus, alternative forms to the physical presence of an animal could be beneficial. The objective of this study was to determine (1) whether exposure to an active-dog video can help improve subjective and physiological responses to stress more than a tranquil-dog video, (2) whether exposure to dog videos can improve subjective and physiological stress responses more than nature videos, and (3) whether exposure to dog (and nature) videos can improve subjective and physiological stress responses more than a control video. Participants (n = 103; female = 78, male = 25) completed a stressful task and were randomly assigned to watch one of five videos: active dog (dog playing with a toy), tranquil dog (dog lying down quietly), active nature (fast-paced waterfall in a forest), tranquil nature (slow-moving stream in a forest), or blank screen (control video; a video of a black screen). Improvements in subjective (i.e., decrease in stress, anxiety, negative affect, and/or increase in happiness, relaxation, positive affect) and physiological (decrease in heart rate and blood pressure) responses to the stressor were examined. This study found no evidence that the active-dog video improved subjective or physiological responses more than the tranquil-dog video. However, this study found evidence that dog videos can decrease subjective anxiety and increase positive affect more than nature videos. Similarly, this study also found that dog videos can decrease subjective anxiety and increase happiness and positive affect more than can the control video. The effects of the dog videos and nature videos (and control video) on the remaining subjective measures and all physiological responses did not differ. Together, the results show some evidence that dog videos may be better at improving subjective anxiety, happiness, and positive affect responses than nature and/or control videos. However, the results did not show evidence that dog videos could alleviate any physiological responses more than the other videos. Practical applications of these findings include how to improve subjective anxiety and affect responses in public settings (e.g., universities) when animals are not allowed. |
  | Date | 2022 |
  | Language | English |
  | Archive | Embase |
  | URL | https://www.embase.com/search/results?subaction=viewrecord&id=L2014244928&from=export |
  | Volume | 35 |
  | Pages | 463-482 |
  | Publication | Anthrozoos |
  | DOI | 10.1080/08927936.2021.1999606 |
  | Issue | 3 |
  | Journal Abbr | Anthrozoos |
  | ISSN | 1753-0377 |
  | Date Added | 05/02/2026, 17:58:49 |
  | Modified | 05/02/2026, 17:58:49 |

  ### Tags:

  - blood pressure
  - human-animal interaction
  - heart rate
  - dog
  - anxiety
  - animal experiment
  - article
  - female
  - male
  - nonhuman
  - physiological stress
  - human
  - adult
  - controlled study
  - animal model
  - leisure
  - videorecording
  - happiness
  - forest

  ### Attachments

  - Full Text (HTML)
- ## Animal-Assisted Stress Management for Veterinary Staff

  |  |  |
  | --- | --- |
  | Item Type | Journal Article |
  | Author | Y.M. Eaton-Stull |
  | Author | C. Streidl |
  | Author | B.G. Jaffe |
  | Author | S. Kuehn |
  | Author | A. Kaufman |
  | Abstract | High levels of stress have a detrimental impact on veterinary staff, negatively influencing their mental health and contributing to high rates of suicide. Veterinary social workers are tasked with providing interventions to reverse these consequences and support the professional's health and well-being. Twenty-one veterinary staff participated in a study to evaluate the impact of animal-assisted support. Over three months, participants attended one or two therapy dog visits per month, interacting with therapy dogs for a minimum of 10 minutes each session. Pre- and postmeasures of blood pressure, heart rate, and self-reported stress were taken at each session. A measure to assess compassion fatigue and satisfaction was administered at the end of each session, and at the conclusion of the study participants' thoughts about the visits from the therapy dogs were assessed. Significant reductions in systolic and diastolic blood pressure were found as well as significantly lower ratings of self-reported stress. Additionally, participants looked forward to these visits stating they made them happy, helped them feel better, and took their mind off their troubles. Considering the growing utilization of therapy dogs, this timely study adds to the body of evidence, highlighting the benefits of animal-assisted interventions with veterinarian staff. |
  | Date | 2024 |
  | Language | English |
  | Archive | Medline |
  | URL | https://www.embase.com/search/results?subaction=viewrecord&id=L645269223&from=export |
  | Volume | 49 |
  | Pages | 219-226 |
  | Publication | Health & social work |
  | DOI | 10.1093/hsw/hlae025 |
  | Issue | 4 |
  | Journal Abbr | Health Soc Work |
  | ISSN | 1545-6854 |
  | Date Added | 05/02/2026, 17:58:34 |
  | Modified | 05/02/2026, 17:58:34 |

  ### Tags:

  - blood pressure
  - heart rate
  - dog
  - female
  - male
  - human
  - adult
  - animal
  - mental stress
  - psychology
  - middle aged
  - job stress
  - animal assisted therapy
  - veterinarian

  ### Attachments

  - Full Text (HTML)
- ## BLOOD CORTISOL INDICATORS AND WORKLOAD IN C ANISTHERAPY DOGS

  |  |  |
  | --- | --- |
  | Item Type | Journal Article |
  | Author | A. Dovidė |
  | Author | J. Kučinskienė |
  | Author | V. Ribikauskas |
  | Abstract | Blood serum cortisol level was measured in 5 therapy assisting dogs (3 Siberian huskies, 1 golden retriever, 1 mixed breed dog) before and after their work sessions at the beginning of a working season and 2 months after the beginning. An average cortisol level for first measurements was 1.33 ug/dL (SD 0.42, max 1.99, min 0.89); meanwhile, after an animal assisted therapy session, the cortisol level increased to 1.47 (SD 0.52, max 2.28, min 0.95). After 2 months of a working season, before a therapy session, an average cortisol level was 1.35 (SD 0.45, max 2.05, min 0.90); meanwhile, after an animal assisted therapy session, the cortisol level increased to 1.40 (SD 0.48, max 2.11, min 0.91). The results showed that the cortisol level after having a therapy class increased less in two months compared with the initial situation. It shows how well dogs adapt to theoretically stressful conditions and feel no more long-lasting stress. Another experiment was carried out for eight weeks with two Siberian huskies in order to estimate the workload (completed distances) of animals during therapy working sessions compared with their usual daily walking and sled sports training. The results revealed that during the therapy work with children dogs covered 12% to 22% of all their usual daily distance. Such workload for dogs is not high, does not exceed their daily physical activity and does not endanger their welfare. |
  | Date | 2024 |
  | Language | English |
  | Archive | Embase |
  | URL | https://www.embase.com/search/results?subaction=viewrecord&id=L2036571848&from=export |
  | Volume | 82(1) |
  | Pages | 168-168 |
  | Publication | Veterinarija ir Zootechnika |
  | Issue | (Dovidė A., vytautas.ribikauskas@lsmu.lt; Kučinskienė J.; Ribikauskas V.) Lithuanian University of Health Sciences, Lithuania |
  | Journal Abbr | Vet. Zootech. |
  | ISSN | 1392-2130 |
  | Date Added | 05/02/2026, 17:53:24 |
  | Modified | 05/02/2026, 17:53:24 |

  ### Tags:

  - physical activity
  - dog
  - article
  - nonhuman
  - hydrocortisone
  - training
  - animal assisted therapy
  - hydrocortisone blood level
  - walking
  - canistherapy
  - workload
- ## BLOOD CORTISOL INDICATORS AND WORKLOAD IN C ANISTHERAPY DOGS

  |  |  |
  | --- | --- |
  | Item Type | Journal Article |
  | Author | A. Dovidė |
  | Author | J. Kučinskienė |
  | Author | V. Ribikauskas |
  | Abstract | Blood serum cortisol level was measured in 5 therapy assisting dogs (3 Siberian huskies, 1 golden retriever, 1 mixed breed dog) before and after their work sessions at the beginning of a working season and 2 months after the beginning. An average cortisol level for first measurements was 1.33 ug/dL (SD 0.42, max 1.99, min 0.89); meanwhile, after an animal assisted therapy session, the cortisol level increased to 1.47 (SD 0.52, max 2.28, min 0.95). After 2 months of a working season, before a therapy session, an average cortisol level was 1.35 (SD 0.45, max 2.05, min 0.90); meanwhile, after an animal assisted therapy session, the cortisol level increased to 1.40 (SD 0.48, max 2.11, min 0.91). The results showed that the cortisol level after having a therapy class increased less in two months compared with the initial situation. It shows how well dogs adapt to theoretically stressful conditions and feel no more long-lasting stress. Another experiment was carried out for eight weeks with two Siberian huskies in order to estimate the workload (completed distances) of animals during therapy working sessions compared with their usual daily walking and sled sports training. The results revealed that during the therapy work with children dogs covered 12% to 22% of all their usual daily distance. Such workload for dogs is not high, does not exceed their daily physical activity and does not endanger their welfare. |
  | Date | 2024 |
  | Language | English |
  | Archive | Embase |
  | URL | https://www.embase.com/search/results?subaction=viewrecord&id=L2036571848&from=export |
  | Volume | 82(1) |
  | Pages | 168-168 |
  | Publication | Veterinarija ir Zootechnika |
  | Issue | (Dovidė A., vytautas.ribikauskas@lsmu.lt; Kučinskienė J.; Ribikauskas V.) Lithuanian University of Health Sciences, Lithuania |
  | Journal Abbr | Vet. Zootech. |
  | ISSN | 1392-2130 |
  | Date Added | 05/02/2026, 17:58:41 |
  | Modified | 05/02/2026, 17:58:41 |

  ### Tags:

  - physical activity
  - dog
  - article
  - nonhuman
  - hydrocortisone
  - training
  - animal assisted therapy
  - hydrocortisone blood level
  - walking
  - canistherapy
  - workload
- ## Adaptation and acceptability testing of the Expector® vibration vest in horses

  |  |  |
  | --- | --- |
  | Item Type | Journal Article |
  | Author | G.N. de Oliveira |
  | Author | S.C. da Silva |
  | Author | M. Zibordi |
  | Author | A.M.C. de Barros |
  | Author | C.B. Belli |
  | Abstract | The project is based on a test of a thoracic vibration vest prototype, adapted to equines by the Expector® vest’s company, on healthy animals. Ten (10) equines were used in the project, male or female, adults, healthy, belonging to FMVZ-USP or private owners. Each animal went through two phases: A and B. Phase A consisted of the placement of the vest without turning on the vibrators, evaluating the animal’s acceptability, facility, adaptation to the animal’s body, and discomfort due to the vest’s use. Phase B included the placement of the vest and turning on the vibrators, evaluating the animal’s acceptability, reaction to the vibrators, and, if present, to which velocity/type of vibration, and the presence of adverse effects. Both phases were done three times on separate days. The behavioral parameters: “placement facility” and “adaptation to the animal’s body” were observed. In phase B, the response to the vibration was classified from 0 to 5. The answer was evaluated on low and high intensities for the four vibration types. The heart rate (HR) and respiratory rate (RR) were also evaluated at the beginning and end of each repetition. The animals’ HR was kept on normal, except for one animal on one day of the test. Concerning the RR, most animals presented moments of tachypnea. On the experiment’s first day, 100% of grade Great to “facility of placement” and “adaptation to the animal’s body” was obtained, but on days 2 and 3 this value dropped to 90% due to alterations in one animal’s responses. Regarding vibration’s responses, 77.3% were evaluated as no discomfort (grade 0), 17.1% little discomfort (grade 1), 3.3% medium discomfort (grade 2), 0.4% great discomfort (grade 3), 0.21% extreme discomfort (grade 4), and 1.6% non-acceptance of the vest (grade 5). Some possible changes on the prototype were also verified to be suggested to the manufacturer, such as the change of the buckle and the use of wireless control. Vest use appears promising for equine respiratory physiotherapy, considering the acceptability was good, and its efficiency on the expectoration of diseased animals must be tested. |
  | Date | 2022 |
  | Language | English |
  | Archive | Embase |
  | URL | https://www.embase.com/search/results?subaction=viewrecord&id=L2015998791&from=export |
  | Volume | 59 |
  | Publication | Brazilian Journal of Veterinary Research and Animal Science |
  | DOI | 10.11606/issn.1678-4456.bjvras.2022.181942 |
  | Issue | (de Oliveira G.N., gabrielanovodeoliveira@gmail.com; Zibordi M.; de Barros A.M.C.; Belli C.B.) Universidade de São Paulo, Faculdade de Medicina Veterinária e Zootecnia, Departamento de Clínica Médica, SP, São Paulo, Brazil |
  | Journal Abbr | Braz. J. Vet. Res. Anim. Sci. |
  | ISSN | 1678-4456 |
  | Date Added | 05/02/2026, 17:58:48 |
  | Modified | 05/02/2026, 17:58:48 |

  ### Tags:

  - horse
  - behavior
  - heart rate
  - animal experiment
  - article
  - female
  - male
  - nonhuman
  - adult
  - controlled study
  - hippotherapy
  - physiotherapy
  - breathing rate
  - Equus
  - breed
  - breathing exercise
  - adaptation
  - airway clearance device
  - bone vibrator
  - erythema
  - Expector
  - head movement
  - incidence
  - program acceptability
  - prototype
  - sputum
  - tachypnea
  - thorax pain
  - tympanometry
  - vibration
  - vibrator

  ### Attachments

  - Full Text (HTML)
- ## The combined use of salivary cortisol concentrations, heart rate, and respiratory rate for the welfare assessment of dogs involved in AAI programs

  |  |  |
  | --- | --- |
  | Item Type | Journal Article |
  | Author | I.R. de Carvalho |
  | Author | T. Nunes |
  | Author | L. de Sousa |
  | Author | V. Almeida |
  | Abstract | Animal-assisted interventions (AAIs) are increasingly popular throughout the world, with a growing trend. Despite all their potential benefits, there are certain risks to both humans and animals involved in AAI programs that should not be disregarded, such as the burnout of the animals. This study investigated the welfare of dogs during AAIs in Portugal. Three welfare indicators related to stress were assessed in nineteen dogs of nineteen human-dog dyads: salivary cortisol concentration, heart rate (HR), and respiratory rate. These measurements were carried out at two time points: at home and after session. This combined assessment allows for the evaluation of the two main neurohormonal pathways related to stress responses: the hypothalamic-pituitary-adrenal axis and the sympathetic-adrenal-medullary axis. Along with monitoring these indicators, a questionnaire was given to each animal handler immediately before the sample collection, to collect demographic data about his/her dog and to characterize the AAI session they were involved in. Statistically significant differences were found (P < 0.05) among the indicators' mean/median values at home and after an AAI session, with the postsession values being higher. Dogs involved in animal-assisted activities had significantly higher mean HR values after session when compared with the ones involved in animal-assisted therapies (P = 0.015), despite all of them being within the normal range. The dogs subjected to a transport time of longer than fifty minutes also had significantly higher mean HR (P = 0.007) and median respiratory rate (P = 0.0001) after session. Most of the dogs (n = 15) had salivary cortisol concentration values within what is considered to be the normal range (<0.3 μg/dL) with only one dog having an extremely high value (1.101 μg/dL) that was attributed to the entrance protocol in the AAI site and to the characteristics of the session itself. The analysis of the three indicators did not raise severe concern about the welfare of the monitored dogs, but some practices that should be avoided were identified: long transport to the AAI site (≥50 minutes); performing AAI in excessively warm rooms; and entering schools during children's playtime. |
  | Date | 2020 |
  | Language | English |
  | Archive | Embase |
  | URL | https://www.embase.com/search/results?subaction=viewrecord&id=L2005534956&from=export |
  | Volume | 36 |
  | Pages | 26-33 |
  | Publication | Journal of Veterinary Behavior |
  | DOI | 10.1016/j.jveb.2019.10.011 |
  | Issue | (de Carvalho I.R., ircarvalho@campus.ul.pt; Nunes T.; Almeida V.) Faculty of Veterinary Medicine, University of Lisbon, Avenida da Universidade Técnica, Lisboa, Portugal |
  | Journal Abbr | J. Vet. Behav. |
  | ISSN | 1558-7878 |
  | Date Added | 05/02/2026, 17:58:58 |
  | Modified | 05/02/2026, 17:58:58 |

  ### Tags:

  - animal welfare
  - heart rate
  - dog
  - saliva
  - animal behavior
  - animal experiment
  - article
  - female
  - male
  - nonhuman
  - physiological stress
  - human
  - hydrocortisone
  - controlled study
  - questionnaire
  - breathing rate
  - hypothalamus hypophysis adrenal system
  - demography
  - concentration (parameter)
  - animal assisted intervention
  - burnout
  - health care
  - neurohormone
  - Portugal
  - risk benefit analysis

  ### Attachments

  - Full Text (HTML)
- ## Effects of multiple-dose intranasal oxytocin administration on social responsiveness in children with autism: a randomized, placebo-controlled trial

  |  |  |
  | --- | --- |
  | Item Type | Journal Article |
  | Author | N. Daniels |
  | Author | M. Moerkerke |
  | Author | J. Steyaert |
  | Author | A. Bamps |
  | Author | E. Debbaut |
  | Author | J. Prinsen |
  | Author | T. Tang |
  | Author | S. Van der Donck |
  | Author | B. Boets |
  | Author | K. Alaerts |
  | Abstract | Background: Intranasal administration of oxytocin is increasingly explored as a new approach to facilitate social development and reduce disability associated with a diagnosis of autism spectrum disorder (ASD). The efficacy of multiple-dose oxytocin administration in children with ASD is, however, not well established. Methods: A double-blind, randomized, placebo-controlled trial with parallel design explored the effects of a 4-week intranasal oxytocin administration (12 IU, twice daily) on parent-rated social responsiveness (Social Responsiveness Scale: SRS-2) in pre-pubertal school-aged children (aged 8–12 years, 61 boys, 16 girls). Secondary outcomes included a questionnaire-based assessment of repetitive behaviors, anxiety, and attachment. Effects of oxytocin were assessed immediately after the administration period and at a follow-up, 4 weeks after the last administration. The double-blind phase was followed by a 4-week single-blind phase during which all participants received intranasal oxytocin. Results: In the double-blind phase, both the oxytocin and placebo group displayed significant pre-to-post-improvements in social responsiveness and secondary questionnaires, but improvements were not specific to the intranasal oxytocin. Notably, in the single-blind phase, participants who were first allocated to intranasal placebo and later changed to intranasal oxytocin displayed a significant improvement in social responsiveness, over and above the placebo-induced improvements noted in the first phase. Participants receiving oxytocin in the first phase also showed a significant further improvement upon receiving a second course of oxytocin, but only at the 4-week follow-up. Further, exploratory moderator analyses indicated that children who received psychosocial trainings (3 or more sessions per month) along with oxytocin administration displayed a more pronounced improvement in social responsiveness. Limitations: Future studies using larger cohorts and more explicitly controlled concurrent psychosocial trainings are warranted to further explore the preliminary moderator effects, also including understudied populations within the autism spectrum, such as children with co-occurring intellectual disabilities. Conclusions: Four weeks of oxytocin administration did not induce treatment-specific improvements in social responsiveness in school-aged children with ASD. Future studies are warranted to further explore the clinical efficacy of oxytocin administration paired with targeted psychosocial trainings that stimulate socio-communicative behaviors. Trial registration The trial was registered with the European Clinical Trial Registry (EudraCT 2018-000769-35) on June 7th, 2018 (https://www.clinicaltrialsregister.eu/ctr-search/trial/2018-000769-35/BE). |
  | Date | 2023 |
  | Language | English |
  | Archive | Embase |
  | URL | https://www.embase.com/search/results?subaction=viewrecord&id=L2022707424&from=export |
  | Volume | 14 |
  | Publication | Molecular Autism |
  | DOI | 10.1186/s13229-023-00546-5 |
  | Issue | 1 |
  | Journal Abbr | Mol. Autism |
  | ISSN | 2040-2392 |
  | Date Added | 05/02/2026, 17:58:41 |
  | Modified | 05/02/2026, 17:58:41 |

  ### Tags:

  - oxytocin
  - anxiety
  - child
  - article
  - female
  - male
  - human
  - controlled study
  - follow up
  - hippotherapy
  - randomized controlled trial
  - motivation
  - questionnaire
  - autism
  - major clinical study
  - outcome assessment
  - awareness
  - exploratory research
  - attention deficit hyperactivity disorder
  - psychotherapy
  - depression
  - social cognition
  - nose spray
  - social adaptation
  - compulsion
  - intelligence quotient
  - cognitive behavioral therapy
  - 2018-000769-35
  - Autism Diagnostic Observation Schedule
  - double blind procedure
  - DSM-5
  - dyscalculia
  - dyslexia
  - good clinical practice
  - intention to treat analysis
  - music therapy
  - placebo
  - rigidity
  - Social Responsiveness Scale-second edition
  - syntocinon
  - Wechsler intelligence scale for children

  ### Attachments

  - Full Text (HTML)
- ## Therapeutic Riding Horses: using a hypothalamic-pituitary-adrenal axis measure to assess the physiological stress response to different riders

  |  |  |
  | --- | --- |
  | Item Type | Journal Article |
  | Author | C. Cravana |
  | Author | E. Fazio |
  | Author | A. Ferlazzo |
  | Author | P. Medica |
  | Abstract | In this study, circulating β-endorphin, ACTH and cortisol concentrations were chosen as physiological markers of the hypothalamic-pituitary-adrenal (HPA) stress-related response in Therapeutic Riding Horses (TRHs). The aim of this research was to investigate HPA axis hormonal response in TRHs and check the effects of different kind of riders (mentally-impaired and able-minded riders). The main hypothesis was that, in TRHs, the HPA axis reaction would be less responsive to mentally-impaired than to able-minded riders, due to increasing levels of horse control by the able-minded riders. 6 TRHs were randomly ridden in the same setting by 2 groups of riders, adult mentally-impaired riders (Treatment Group A) and able-minded adult riders (Treatment Group B). Both groups consisted of inexperienced riders. Each rider rode 2 times a week for 6 consecutive weeks. The horses performed the same gaits and exercises at all sessions, with both Treatment Group A and Treatment Group B riders. All hormones’ concentrations were determined on a resting day (baseline, T0), before the session (T1), and at 5 min (T2) and 30 min (T3) after each session. Statistical analysis showed significant effects of the different Treatment Groups on the cortisol concentrations only, with lower cortisol concentrations both at T2 and at T3 in Treatment Group A than in Treatment Group B. Related to changes over the sampling time, ccortisol concentrations in horses were significantly higher at T1 than at T0 in Treatment Group A, and at T1, T2 and T3 than at T0, in Treatment Group B. ACTH concentrations were significantly higher at T1, T2 and T3 than at T0, only in Treatment Group B. β-endorphin concentrations at T3 were significantly higher than at T0, only in Treatment Group B. Results showed differences in the TRHs' stress levels between rider groups, with significantly lower cortisol concentrations in Treatment Group A than in Treatment Group B. Thus, although sessions’ workload stress was equivalent, the minor response of HPA axis could be related to different effects of horse-human interaction. |
  | Date | 2021 |
  | Language | English |
  | Archive | Embase |
  | URL | https://www.embase.com/search/results?subaction=viewrecord&id=L2014722826&from=export |
  | Volume | 46 |
  | Pages | 18-23 |
  | Publication | Journal of Veterinary Behavior |
  | DOI | 10.1016/j.jveb.2021.07.013 |
  | Issue | (Cravana C., ccravana@unime.it; Fazio E.; Ferlazzo A.; Medica P.) Department of Veterinary Sciences, Unit of Veterinary Physiology, University of Messina, Polo Universitario Annunziata, Messina, Italy |
  | Journal Abbr | J. Vet. Behav. |
  | ISSN | 1558-7878 |
  | Date Added | 05/02/2026, 17:58:52 |
  | Modified | 05/02/2026, 17:58:52 |

  ### Tags:

  - article
  - male
  - physiological stress
  - human
  - hydrocortisone
  - adult
  - hippotherapy
  - corticotropin
  - exercise
  - clinical article
  - middle aged
  - beta endorphin
  - hypothalamus hypophysis adrenal system
  - hydrocortisone blood level
  - hormone response
  - corticotropin blood level
  - gait
  - cognitive defect
  - hormone blood level
  - patient monitoring

  ### Attachments

  - Full Text (HTML)
- ## The Experience of Animal Assisted Therapy on Patients in an Acute Care Setting

  |  |  |
  | --- | --- |
  | Item Type | Journal Article |
  | Author | A.B. Coakley |
  | Author | C.D. Annese |
  | Author | J.H. Empoliti |
  | Author | J.M. Flanagan |
  | Abstract | Animal assisted therapy (AAT) programs are popular and there has been a proliferation of programs across settings. However, the research to support this intervention has not kept pace. This is particularly so for people who are hospitalized. This investigation aimed to explore the effects of the AAT dog visitation program on patients. A single group pre-post quasi-experimental design evaluated the effect of pet therapy on patients. Measures included salivary cortisol, anxiety, wellbeing, comfort, respiratory and heart rate. Analysis indicates a significant reduction in heart and respiratory rates p < .01 and level of anxiety p < .000 with improved levels of comfort and well-being p < .000. The salivary cortisol result was non-significant p = .623. This ATT dog program resulted in reduced anxiety levels and decreased heart and respiratory rates while improving subjective measures of comfort and wellbeing. |
  | Date | 2021 |
  | Language | English |
  | Archive | Medline |
  | URL | https://www.embase.com/search/results?subaction=viewrecord&id=L633538662&from=export |
  | Volume | 30 |
  | Pages | 401-405 |
  | Publication | Clinical nursing research |
  | DOI | 10.1177/1054773820977198 |
  | Issue | 4 |
  | Journal Abbr | Clin Nurs Res |
  | ISSN | 1552-3799 |
  | Date Added | 05/02/2026, 17:58:52 |
  | Modified | 05/02/2026, 17:58:52 |

  ### Tags:

  - heart rate
  - dog
  - anxiety
  - human
  - animal
  - animal assisted therapy
  - intensive care

  ### Attachments

  - Full Text (HTML)
- ## The Impact of a 20-Minute Animal-Assisted Activity Session on the Physiological and Emotional States in Patients With Fibromyalgia

  |  |  |
  | --- | --- |
  | Item Type | Journal Article |
  | Author | S. Clark |
  | Author | F. Martin |
  | Author | R.T.S. McGowan |
  | Author | J. Smidt |
  | Author | R. Anderson |
  | Author | L. Wang |
  | Author | T. Turpin |
  | Author | N. Langenfeld-McCoy |
  | Author | B. Bauer |
  | Author | A.B. Mohabbat |
  | Abstract | Objective: To study the direct physiological and emotional impact of an animal-assisted activity (AAA) session (a form of complementary and integrative medicine) in patients with fibromyalgia (FM). Patients and Methods: The study population consisted of 221 participants with FM who were attending Mayo Clinic's Fibromyalgia Treatment Program between August 5, 2017, and September 1, 2018. This was a randomized controlled trial. Participants were randomly assigned to either the treatment group (a 20-minute session with a certified therapy dog and handler) or the control group (a 20-minute session with a handler only). To gain a better understanding of the direct physiological and emotional effects of AAA in patients with FM, we used multiple noninvasive physiologic-emotional biomarkers, including salivary cortisol and oxytocin concentrations, tympanic membrane temperatures, and various cardiac parameters, in addition to standardized pain and mood-based questionnaires. Results: Results show a decrease in heart rate, an increase in heart rate variability, an increase in well-being survey scores, an increase in salivary oxytocin, and subsequent tympanic membrane temperature changes, suggesting that participants in the treatment group were in a more positive emotional-physiologic state as a result of the AAA session compared with the control group. Conclusion: Our results suggest that a 20-minute therapy dog visit in an outpatient setting can significantly and positively impact the physical and mental health of patients with FM. |
  | Date | 2020 |
  | Language | English |
  | Archive | Embase |
  | URL | https://www.embase.com/search/results?subaction=viewrecord&id=L2007661740&from=export |
  | Volume | 95 |
  | Pages | 2442-2461 |
  | Publication | Mayo Clinic Proceedings |
  | DOI | 10.1016/j.mayocp.2020.04.037 |
  | Issue | 11 |
  | Journal Abbr | Mayo Clin. Proc. |
  | ISSN | 1942-5546 |
  | Date Added | 05/02/2026, 17:58:53 |
  | Modified | 05/02/2026, 17:58:53 |

  ### Tags:

  - oxytocin
  - heart rate variability
  - physiology
  - heart rate
  - dog
  - emotion
  - article
  - female
  - male
  - nonhuman
  - human
  - hydrocortisone
  - adult
  - aged
  - biomedical software
  - controlled study
  - randomized controlled trial
  - body temperature
  - questionnaire
  - wellbeing
  - major clinical study
  - mood
  - visual analog scale
  - pain
  - biological marker
  - animal assisted therapy
  - health
  - therapy effect
  - mental health
  - saliva level
  - outpatient
  - fibromyalgia
  - mass spectrometer
  - immunoassay analyzer
  - AB Sciex 6500+
  - Analyst 1.6.3
  - Braun ThermoScan PRO 6000
  - cardiograph
  - cardiovascular parameters
  - clinical chemistry analyzer
  - cobas e411
  - eardrum
  - Fibromyalgia Impact Questionnaire
  - Kubios HRV Standard Version 3.1.0
  - Lexington Pet Attachment Scale
  - Nexera X2
  - non invasive procedure
  - patient
  - Pet Attitude Scale
  - Polar Flow
  - Polar V800
  - tympanic thermometer

  ### Attachments

  - Full Text (HTML)
- ## Physiological state of therapy dogs during animal-assisted activities in an outpatient setting

  |  |  |
  | --- | --- |
  | Item Type | Journal Article |
  | Author | S.D. Clark |
  | Author | F. Martin |
  | Author | R.T.S. McGowan |
  | Author | J.M. Smidt |
  | Author | R. Anderson |
  | Author | L. Wang |
  | Author | T. Turpin |
  | Author | N. Langenfeld-Mccoy |
  | Author | B.A. Bauer |
  | Author | A.B. Mohabbat |
  | Abstract | Therapy dogs are increasingly being incorporated into numerous clinical settings. However, there are only a handful of studies that have focused on the impact of animal-assisted activity or therapy sessions on the wellbeing of the therapy dogs. Furthermore, these studies show mixed results. The goal of this study was to provide an in-depth picture of the effects of these interactions on the dogs involved by considering multiple physiological measures known to be associated with emotional state (continuous heart rate, heart rate variability, pre-and post-session tympanic membrane temperatures, and salivary cortisol and oxytocin concentrations). Nineteen Mayo Clinic Caring Canine therapy dogs completed five 20-minute animal-assisted activity (AAA) visits each in an outpatient clinical setting (Mayo Clinic Fibromyalgia and Chronic Fatigue Clinic). From a physiological perspective, the dogs showed a neutral to positive response to the AAA sessions. Heart rate (HR) was significantly lower at the end of the session compared with the beginning of the session (F = 17.26, df1 = 1, df2 = 29.7, p = 0.0003). The right tympanic membrane temperature was lower post-session (F = 8.87, df1 = 1, df2 = 107, p = 0.003). All other emotional indicators remained stable between pre-and post-session. These results suggest that the dogs involved were not negatively affected by their participation in the AAA. Moreover, there was some evidence suggesting the dogs may have been in a more relaxed state at the end of the session (lower HR and lower right tympanic membrane temperature) compared to the beginning of the session. |
  | Date | 2020 |
  | Language | English |
  | Archive | Embase |
  | URL | https://www.embase.com/search/results?subaction=viewrecord&id=L2004338710&from=export |
  | Volume | 10 |
  | Publication | Animals |
  | DOI | 10.3390/ani10050819 |
  | Issue | 5 |
  | Journal Abbr | Animals |
  | ISSN | 2076-2615 |
  | Date Added | 05/02/2026, 17:58:56 |
  | Modified | 05/02/2026, 17:58:56 |

  ### Tags:

  - heart rate
  - dog
  - article
  - female
  - male
  - nonhuman
  - social interaction
  - human
  - adult
  - aged
  - controlled study
  - chemiluminescence immunoassay
  - volunteer
  - major clinical study
  - animal assisted therapy
  - tympanic temperature
  - saliva analysis
  - liquid chromatography-mass spectrometry
  - mass spectrometer
  - immunoassay analyzer
  - animal assisted activity
  - Braun ThermoScan PRO 6000
  - Nexera X2
  - Polar V800
  - tympanic thermometer
  - AB Sciex 6500
  - Cobas e411
  - emotional stability
  - fetal heart monitor
  - liquid chromatograph
  - outpatient care

  ### Attachments

  - Full Text (HTML)
- ## The Effect of Equine-Assisted Activities in Children Aged 7-8 Years Inhibitory Control: An fNIRS Study

  |  |  |
  | --- | --- |
  | Item Type | Journal Article |
  | Author | X.D. Cheng |
  | Author | L. Qian |
  | Author | Y. Fan |
  | Author | Q. Tang |
  | Author | H. Wu |
  | Abstract | Background: Inhibitory control (IC), an important component of executive function, plays an important role in the overall development of children and has not been better studied in the field of equine-assisted activity (EAA). Therefore, this study investigated the effects of EAA on IC and the underlying brain neural mechanisms in children aged 7-8 years. Methods: Forty-eight healthy children aged 7-8 years from the Maple Leaf International School-Xi'an were randomly allocated to the equine-assisted activities group (EAAG) and control group (CG). The EAAG received 12 weeks of EAAG training from instructors at the MingLiu Horse Club while the CG continued their normal daily activities. The Flanker task was administered to both groups to assess IC pre- and post-intervention. Functional nearinfrared spectroscopy (fNIRS) data were collected during the Flanker task to examine the underlying neural mechanisms. Results: Our findings indicate that after 12 weeks of EAA, the EAAG performed significantly better on the Flanker tasks than the CG, with congruent and incongruent higher accuracy and faster reaction (p < 0.01). Importantly, fNIRS data analysis revealed increased oxyhemoglobin levels in the right dorsolateral prefrontal cortex (R-DLPFC) (p < 0.05) of the EAAG during the Flanker congruent task after the EAA intervention. Conclusions: Collectively, EAA demonstrated a positive impact on IC and could effectively activate R-DLPFC in children aged 7-8 years. Furthermore, it enhanced the activation of the brain regions related to IC and increased cognitive ability in children aged 7-8 years. |
  | Date | 2023 |
  | Language | English |
  | Archive | Embase |
  | URL | https://www.embase.com/search/results?subaction=viewrecord&id=L2026359003&from=export |
  | Volume | 22 |
  | Publication | Journal of Integrative Neuroscience |
  | DOI | 10.31083/j.jin2204089 |
  | Issue | 4 |
  | Journal Abbr | J. Integr. Neurosci. |
  | ISSN | 1757-448X |
  | Date Added | 05/02/2026, 17:53:26 |
  | Modified | 05/02/2026, 17:53:26 |

  ### Tags:

  - cognition
  - child
  - article
  - female
  - male
  - human
  - body weight
  - controlled study
  - hippotherapy
  - functional near-infrared spectroscopy
  - clinical article
  - prefrontal cortex
  - demography
  - perception
  - sport injury
  - body mass
  - blood oxygenation
  - body height
  - cognitive defect
  - executive function
  - exercise intensity
  - measurement accuracy
  - motor cortex
  - oxyhemoglobin

  ### Attachments

  - Full Text (HTML)
- ## The influence of pretreatment respiratory sinus arrhythmia dimensions on trauma-focused cognitive behavioral therapy outcomes: Findings from a randomized controlled feasibility trial

  |  |  |
  | --- | --- |
  | Item Type | Journal Article |
  | Author | M.P. Brown |
  | Author | C.E. Shenk |
  | Author | B. Allen |
  | Author | E.D. Dunning |
  | Author | M.A. Lombera |
  | Author | A.M. Bucher |
  | Author | N.A. Dreschel |
  | Abstract | Child maltreatment is associated with respiratory sinus arrhythmia (RSA) dysregulation, a physiological indicator of emotion regulation that predicts elevated posttraumatic stress disorder (PTSD) symptoms and may be a mechanism of action for exposure-based therapies, such as trauma-focused cognitive behavioral therapy (TF-CBT). Animal-assisted therapy (AAT) has been proposed as an adjunct to TF-CBT for improving emotion regulation following maltreatment. The current study reports findings from a randomized controlled feasibility trial (N = 33; Mage = 11.79 years, SD = 3.08; 63.6% White; 66.7% female) that measured youths' resting RSA, RSA reactivity, and RSA recovery in response to a pretreatment laboratory challenge. We tested whether (a) lower pretreatment resting RSA was associated with blunted RSA during the challenge; (b) either of the pretreatment RSA dimensions predicted more severe pretreatment PTSD symptoms; and (c) either of the pretreatment RSA dimensions predicted less severe posttreatment PTSD symptoms and, as an exploratory aim, whether this was moderated by treatment group (i.e., TF-CBT vs. TF-CBT + AAT). Results from multiple linear regression indicated that, after controlling for pretreatment symptom severity, there was a large effect size for higher resting RSA predicting less severe caregiver-reported posttreatment PTSD symptoms, β = -.52, p = .058, and higher RSA during recovery predicting less severe child-reported posttreatment PTSD symptoms, β = -.70, p = .056, although these findings were not significant. These preliminary results offer important insights for future studies to investigate how the ability to regulate RSA informs which children need additional support to benefit from psychotherapeutic treatment. |
  | Date | 2024 |
  | Language | English |
  | Archive | Medline |
  | URL | https://www.embase.com/search/results?subaction=viewrecord&id=L644266176&from=export |
  | Volume | 37 |
  | Pages | 850-863 |
  | Publication | Journal of traumatic stress |
  | DOI | 10.1002/jts.23053 |
  | Issue | 6 |
  | Journal Abbr | J Trauma Stress |
  | ISSN | 1573-6598 |
  | Date Added | 05/02/2026, 17:53:16 |
  | Modified | 05/02/2026, 17:53:16 |

  ### Tags:

  - physiology
  - therapy
  - emotion regulation
  - child
  - female
  - male
  - human
  - controlled study
  - posttraumatic stress disorder
  - randomized controlled trial
  - adolescent
  - psychology
  - treatment outcome
  - procedures
  - cognitive behavioral therapy
  - child abuse
  - feasibility study
  - respiratory sinus arrhythmia

  ### Attachments

  - Full Text (HTML)
- ## Animal-Assisted Activity in Critically Ill Older Adults: A Randomized Pilot and Feasibility Trial

  |  |  |
  | --- | --- |
  | Item Type | Journal Article |
  | Author | S. Branson |
  | Author | L. Boss |
  | Author | S. Hamlin |
  | Author | N.S. Padhye |
  | Abstract | BACKGROUND: Limited evidence suggests the efficacy of animal-assisted activities (AAA) in improving biobehavioral stress responses in older patients in intensive care units (ICUs). OBJECTIVES: To assess the feasibility of an AAA (dog) intervention for improving biobehavioral stress response, measured by self-reported stress and anxiety and salivary cortisol, C-reactive protein, and interleukin-1β in older ICU patients, we examined enrollment, attrition, completion, data collection, and biobehavioral stress responses. METHODS: ICU patients ≥60 years old were randomly assigned to a 10-min AAA intervention or control/usual ICU care. Attitudes toward pets were assessed before the intervention. Self-reported stress and anxiety and salivary stress biomarkers were collected before and after the intervention and the usual care condition. RESULTS: The majority of patients were ineligible due to lack of decisional capacity, younger age, inability to provide saliva specimens, or critical illness. Though 15 participants were randomly allocated (AAA = 9; control = 6), only 10 completed the study. All participants completed the questionnaires; however, saliva specimens were significantly limited by volume. AAA was associated with decreases in stress and anxiety. Biomarker results were variable and revealed no specific trends associated with stress responses. Conclusions: Barriers to recruitment included an insufficient number of patients eligible for AAA based on hospital policy, difficulty finding patients who met study eligibility criteria, and illness-related factors. Recommendations for future studies include larger samples, a stronger control intervention such as a visitor without a dog, greater control over the AAA intervention, and use of blood from indwelling catheters for biomarkers. |
  | Date | 2020 |
  | Language | English |
  | Archive | Medline |
  | URL | https://www.embase.com/search/results?subaction=viewrecord&id=L631599674&from=export |
  | Volume | 22 |
  | Pages | 412-417 |
  | Publication | Biological research for nursing |
  | DOI | 10.1177/1099800420920719 |
  | Issue | 3 |
  | Journal Abbr | Biol Res Nurs |
  | ISSN | 1552-4175 |
  | Date Added | 05/02/2026, 17:58:54 |
  | Modified | 05/02/2026, 17:58:54 |

  ### Tags:

  - dog
  - female
  - male
  - human
  - aged
  - pilot study
  - questionnaire
  - animal
  - mental stress
  - psychology
  - middle aged
  - animal assisted therapy
  - procedures
  - intensive care unit
  - feasibility study
  - critical illness
  - frail elderly
  - randomization
  - very elderly

  ### Attachments

  - Full Text (HTML)
- ## Is Animal-Assisted Therapy for Minimally Conscious State Beneficial? A Case Study

  |  |  |
  | --- | --- |
  | Item Type | Journal Article |
  | Author | J.P. Boitier |
  | Author | M. Huber |
  | Author | C. Saleh |
  | Author | M.J. Kerry |
  | Author | M. Hund-Georgiadis |
  | Author | K. Hediger |
  | Abstract | Objective: The goal of this single case study was to qualitatively investigate the effects of animal-assisted therapy in a patient in a minimally conscious state. Method: We present a 28-year-old female patient in a minimally conscious state following polytrauma after a sports accident leading to cerebral fat embolism causing multiple CNS ischemic lesions. She received eight animal-assisted therapy sessions and eight paralleled control therapy sessions over 4 weeks. We investigated the reactions of the patient during these sessions via qualitative behavior analysis. Results: The patient showed a broader variability and higher quality of behavior during animal-assisted therapy compared to control therapy sessions. Conclusion: The observed behavioral changes showed higher arousal and increased awareness in the presence of an animal. The presented case supports the assumption that animal-assisted therapy can be a beneficial treatment approach for patients in a minimally conscious state. |
  | Date | 2020 |
  | Language | English |
  | Archive | Embase |
  | URL | https://www.embase.com/search/results?subaction=viewrecord&id=L632043182&from=export |
  | Volume | 11 |
  | Publication | Frontiers in Psychiatry |
  | DOI | 10.3389/fpsyt.2020.00491 |
  | Issue | (Boitier J.P.; Huber M.; Kerry M.J.) Department of Health, Zurich University of Applied Sciences (ZHAW), Winterthur, Switzerland |
  | Journal Abbr | Front. Psychiatry |
  | ISSN | 1664-0640 |
  | Date Added | 05/02/2026, 17:58:56 |
  | Modified | 05/02/2026, 17:58:56 |

  ### Tags:

  - article
  - female
  - human
  - adult
  - occupational therapy
  - physiotherapy
  - scoring system
  - Glasgow coma scale
  - minimally conscious state
  - neurorehabilitation
  - electroencephalography
  - clinical article
  - animal assisted therapy
  - sport injury
  - case report
  - speech therapy
  - music therapy
  - baclofen
  - brain embolism
  - case study
  - clonidine
  - Coma Recovery Scale score
  - melatonin
  - multiple trauma
  - neurofeedback
  - percutaneous endoscopic gastrostomy tube
  - piracetam
  - slow brain wave

  ### Attachments

  - Full Text (HTML)
- ## Equine-assisted therapy as an adjunctive method in the treatment of recurrent depressive disorder – a case report

  |  |  |
  | --- | --- |
  | Item Type | Journal Article |
  | Author | K. Bartniak |
  | Author | K. Zaborska |
  | Author | M.E. Talarowska |
  | Abstract | Background: The term “animal-assisted therapy” refers to a range of methods in which animals accompany the treatment of patients with various somatic or mental disorders, while “equine-assisted psychotherapy” refers to sessions that involve a psychotherapist and an equine professional working alongside the animal, with the primary goal of improving the mental functioning of the patient. Materials and methods: The paper presents a case study of a 48-year-old female patient treated for symptoms of recurrent depressive disorder. A programme of five sessions was developed based on equine-assisted psychotherapy principles. The NEO Five-Factor Inventory, INTE Emotional Intelligence Questionnaire, Beck Depression Inventory II, and UWIST Mood Adjective Checklist were used. Conclusions: A series of five sessions based on the equineassisted psychotherapy principles led to a reduction in the severity of depressive symptoms as measured by the BDI-II scale and a positive change in dominant mood. |
  | Date | 2025 |
  | Language | English |
  | Archive | Embase |
  | URL | https://www.embase.com/search/results?subaction=viewrecord&id=L2042414733&from=export |
  | Volume | 25 |
  | Pages | 182-189 |
  | Publication | Psychiatria i Psychologia Kliniczna |
  | DOI | 10.15557/PiPK.2025.0023 |
  | Issue | 2 |
  | Journal Abbr | Psychiatr. Psychol. Klin. |
  | ISSN | 2451-0645 |
  | Date Added | 05/02/2026, 17:53:16 |
  | Modified | 05/02/2026, 17:53:16 |

  ### Tags:

  - emotion
  - article
  - female
  - human
  - adult
  - hippotherapy
  - motivation
  - clinical article
  - middle aged
  - disease severity
  - depression
  - neurosis
  - oxytocin receptor
  - anxiety disorder
  - valence (emotion)
  - fatigue
  - suicidal ideation
  - Beck Depression Inventory
  - body weight loss
  - avoidance behavior
  - psychologic test
  - irritability
  - sleep disorder
  - sleep quality
  - case report
  - intellectual impairment
  - anhedonia
  - emotional intelligence
  - extraversion
  - faxolet
  - headache
  - inte emotional intelligence questionnaire
  - intrusive thought
  - mental function
  - neo five factor inventory
  - psychopharmacotherapy
  - recurrent disease
  - Structured Clinical Interview for DSM Disorders
  - uwist mood adjective checklist
  - venlafaxine

  ### Attachments

  - Full Text (HTML)
- ## Pilot study of the influence of equine assisted therapy on physiological and behavioral parameters related to welfare of horses and patients

  |  |  |
  | --- | --- |
  | Item Type | Journal Article |
  | Author | M.D. Ayala |
  | Author | A. Carrillo |
  | Author | P. Iniesta |
  | Author | P. Ferrer |
  | Abstract | Different welfare indicators were studied in three patients with psychomotor alterations and in two horses throughout 9–10 equine assisted therapy sessions in each patient. In horses, heart and respiratory rates, blood pressure, temperature and behavioral signs were studied. In patients, heart rate, oxygen saturation, temperature, sleep quality, psychomotor and emotional parameters were analyzed. Data collection was recorded in the anticipatory phase (15 min before the start of the session), two interaction phases (after 30 min of horse-patient interaction on the ground and on horseback, respectively) and the recovery phase (15 min after the end of the session). During the anticipatory phase, most of physiological parameters of patients and horses and the stress behavioral signs of horses increased, followed by a relaxing phase during the horse-patient interaction on the ground. In horse-patient riding phase the heart and respiratory rates of the horses again increased. These results showed that the horses did not seem to suffer stress attributable to the therapy sessions, but only an increase in their parameters associated with activity and external stimuli. The patients improved their gross and fine motor skills, their cognitive and perceptual-sensitive parameters and it led to an improvement in the life quality of their families. |
  | Date | 2021 |
  | Language | English |
  | Archive | Embase |
  | URL | https://www.embase.com/search/results?subaction=viewrecord&id=L2014814744&from=export |
  | Volume | 11 |
  | Publication | Animals |
  | DOI | 10.3390/ani11123527 |
  | Issue | 12 |
  | Journal Abbr | Animals |
  | ISSN | 2076-2615 |
  | Date Added | 05/02/2026, 17:58:51 |
  | Modified | 05/02/2026, 17:58:51 |

  ### Tags:

  - blood pressure
  - welfare
  - horse
  - heart rate
  - article
  - female
  - male
  - nonhuman
  - physiological stress
  - human
  - adult
  - hippotherapy
  - pilot study
  - oxygen saturation
  - quality of life
  - wellbeing
  - motor performance
  - breathing rate
  - clinical article
  - sleep quality
  - case report
  - remission

  ### Attachments

  - Full Text (HTML)
- ## Effects of animal-assisted therapy on gait performance, respiratory function, and psychological variables in patients post-stroke

  |  |  |
  | --- | --- |
  | Item Type | Journal Article |
  | Author | H.-J. An |
  | Author | S.-J. Park |
  | Abstract | Background: Animal-assisted therapy using dogs is being administered to patients post-stroke for the purpose of recovering psychological and physical activity. Objective: This study was conducted to confirm the effect of animal-assisted therapy using dogs on gait performance, pulmonary function, and psychological variables in patients post-stroke. All outcomes were analyzed using two-way repeated-measure analysis. Methods: In total, 30 post-stroke patients were divided into an experimental group (gait training by animal-assisted activity, n = 15) and a control group (gait training, n = 15). Gait performance (cadence, gait speed, stride length, symmetric index), respiratory pulmonary function (forced expiratory volume in 1 second (FEV1), forced vital capacity (FVC), peak expiratory flow (PEF), maximal inspiratory pressure (MIP), maximal expiratory pressure (MEP)), and psychological variables (rehabilitation motivation and depression assessment) were measured before and after eight weeks of intervention. Results: Gait performance, respiratory pulmonary function, and psychological variables significantly increased in the experimental group compared to the control group. Conclusion: Based on this study, it was found that animal-assisted therapy using dogs is an effective intervention for recovery of psychological and physical activity in patients post-stroke. |
  | Date | 2021 |
  | Language | English |
  | Archive | Embase |
  | URL | https://www.embase.com/search/results?subaction=viewrecord&id=L2007343504&from=export |
  | Volume | 18 |
  | Publication | International Journal of Environmental Research and Public Health |
  | DOI | 10.3390/ijerph18115818 |
  | Issue | 11 |
  | Journal Abbr | Int. J. Environ. Res. Public Health |
  | ISSN | 1660-4601 |
  | Date Added | 05/02/2026, 17:58:52 |
  | Modified | 05/02/2026, 17:58:52 |

  ### Tags:

  - dog
  - animal experiment
  - article
  - female
  - male
  - nonhuman
  - human
  - adult
  - controlled study
  - motivation
  - animal model
  - animal assisted therapy
  - walking speed
  - rehabilitation
  - remission
  - depression assessment
  - forced expiratory volume
  - forced vital capacity
  - lung function
  - maximal expiratory pressure
  - maximal inspiratory pressure
  - peak expiratory flow
  - stride length
  - stroke patient

  ### Attachments

  - Full Text (HTML)
