# Supplementary material for: Evidence of Physiological Comodulation During Human–Animal Interaction: A Systematic Review
Source: Ann N Y Acad Sci. 2026 Jun 4;1560(1):e70299. doi: 10.1111/nyas.70299 (PMC13238372; doi:10.1111/nyas.70299)
Supplement: Supplementary file 2 — Supplementary Materials: Supp2‐Zotero‐Collection.zip [file NYAS-1560-0-s002.zip › Supp2_Zotero_Collection/new searches/Google Scholar.htm]

Zotero Report


- ## Effectiveness of animal-assisted therapy on pain in children: A systematic review and meta-analysis

  |  |  |
  | --- | --- |
  | Item Type | Journal Article |
  | Author | Yuanyuan Zhang |
  | Author | Fanghong Yan |
  | Author | Sijun Li |
  | Author | Yutan Wang |
  | Author | Yuxia Ma |
  | Date | 2021 |
  | Short Title | Effectiveness of animal-assisted therapy on pain in children |
  | Library Catalogue | Google Scholar |
  | URL | https://www.sciencedirect.com/science/article/pii/S2352013220301800 |
  | Accessed | 05/02/2026, 17:13:09 |
  | Volume | 8 |
  | Publisher | Elsevier |
  | Pages | 30–37 |
  | Publication | International journal of nursing sciences |
  | Issue | 1 |
  | Date Added | 05/02/2026, 17:13:35 |
  | Modified | 05/02/2026, 17:13:35 |
- ## Effectiveness of animal-assisted activities and therapies for autism spectrum disorder: A systematic review and meta-analysis

  |  |  |
  | --- | --- |
  | Item Type | Journal Article |
  | Author | Ningkun Xiao |
  | Author | Vaishnavi Bagayi |
  | Author | Dandan Yang |
  | Author | Xinlin Huang |
  | Author | Lei Zhong |
  | Author | Sergey Kiselev |
  | Author | Mikhail A. Bolkov |
  | Author | Irina A. Tuzankina |
  | Author | Valery A. Chereshnev |
  | Date | 2024 |
  | Short Title | Effectiveness of animal-assisted activities and therapies for autism spectrum disorder |
  | Library Catalogue | Google Scholar |
  | URL | https://www.frontiersin.org/articles/10.3389/fvets.2024.1403527/full |
  | Accessed | 05/02/2026, 17:13:30 |
  | Volume | 11 |
  | Publisher | Frontiers Media SA |
  | Pages | 1403527 |
  | Publication | Frontiers in Veterinary Science |
  | Date Added | 05/02/2026, 17:13:35 |
  | Modified | 05/02/2026, 17:13:35 |

  ### Attachments

  - Available Version (via Google Scholar)
- ## Dog welfare, well-being and behavior: considerations for selection, evaluation and suitability for animal-assisted therapy

  |  |  |
  | --- | --- |
  | Item Type | Journal Article |
  | Author | Melissa Winkle |
  | Author | Amy Johnson |
  | Author | Daniel Mills |
  | Date | 2020 |
  | Short Title | Dog welfare, well-being and behavior |
  | Library Catalogue | Google Scholar |
  | URL | https://www.mdpi.com/2076-2615/10/11/2188 |
  | Accessed | 05/02/2026, 17:25:08 |
  | Volume | 10 |
  | Publisher | MDPI |
  | Pages | 2188 |
  | Publication | Animals |
  | Issue | 11 |
  | Date Added | 05/02/2026, 17:25:16 |
  | Modified | 05/02/2026, 17:25:16 |
- ## Animal Assisted Therapy (AAT) and Animal Assisted Intervention (AAI) for individuals with Autism Spectrum Disorder (ASD): a systematic review and meta-analysis of randomised control trials (RCT's) and Control Trial Studies and; Man's best friend: What is the difference in outcomes (family functioning, quality of life, parental stress and child social communication) in families that have a dog present with children with Autism Spectrum Disorder (ASD): a control comparison study.

  |  |  |
  | --- | --- |
  | Item Type | Journal Article |
  | Author | Lianne White |
  | Date | 2021 |
  | Short Title | Animal Assisted Therapy (AAT) and Animal Assisted Intervention (AAI) for individuals with Autism Spectrum Disorder (ASD) |
  | Library Catalogue | Google Scholar |
  | URL | https://era.ed.ac.uk/handle/1842/38129 |
  | Accessed | 05/02/2026, 17:34:16 |
  | Publisher | The University of Edinburgh |
  | Date Added | 05/02/2026, 17:34:30 |
  | Modified | 05/02/2026, 17:34:30 |

  ### Attachments

  - Available Version (via Google Scholar)
- ## Specific and non-specific factors of animal-assisted interventions considered in research: A systematic review

  |  |  |
  | --- | --- |
  | Item Type | Journal Article |
  | Author | Cora Wagner |
  | Author | Carmina Grob |
  | Author | Karin Hediger |
  | Date | 2022 |
  | Short Title | Specific and non-specific factors of animal-assisted interventions considered in research |
  | Library Catalogue | Google Scholar |
  | URL | https://www.frontiersin.org/journals/psychology/articles/10.3389/fpsyg.2022.931347/full |
  | Accessed | 05/02/2026, 17:25:13 |
  | Volume | 13 |
  | Publisher | Frontiers Media SA |
  | Pages | 931347 |
  | Publication | Frontiers in psychology |
  | Date Added | 05/02/2026, 17:25:16 |
  | Modified | 05/02/2026, 17:25:16 |

  ### Attachments

  - Available Version (via Google Scholar)
- ## Effectiveness of animal-assisted therapy and pet-robot interventions in reducing depressive symptoms among older adults: A systematic review and meta-analysis

  |  |  |
  | --- | --- |
  | Item Type | Journal Article |
  | Author | David Villarreal-Zegarra |
  | Author | Teodoro Yllescas-Panta |
  | Author | Sofía Malaquias-Obregon |
  | Author | Andrea Dámaso-Román |
  | Author | Nikol Mayo-Puchoc |
  | Date | 2024 |
  | Short Title | Effectiveness of animal-assisted therapy and pet-robot interventions in reducing depressive symptoms among older adults |
  | Library Catalogue | Google Scholar |
  | URL | https://www.sciencedirect.com/science/article/pii/S0965229924000116 |
  | Accessed | 05/02/2026, 17:24:58 |
  | Volume | 80 |
  | Publisher | Elsevier |
  | Pages | 103023 |
  | Publication | Complementary therapies in medicine |
  | Date Added | 05/02/2026, 17:25:16 |
  | Modified | 05/02/2026, 17:25:16 |
- ## Effects of dog-based animal-assisted interventions in prison population: A systematic review

  |  |  |
  | --- | --- |
  | Item Type | Journal Article |
  | Author | Beatriz Villafaina-Domínguez |
  | Author | Daniel Collado-Mateo |
  | Author | Eugenio Merellano-Navarro |
  | Author | Santos Villafaina |
  | Date | 2020 |
  | Short Title | Effects of dog-based animal-assisted interventions in prison population |
  | Library Catalogue | Google Scholar |
  | URL | https://www.mdpi.com/2076-2615/10/11/2129 |
  | Accessed | 05/02/2026, 17:17:10 |
  | Volume | 10 |
  | Publisher | MDPI |
  | Pages | 2129 |
  | Publication | Animals |
  | Issue | 11 |
  | Date Added | 05/02/2026, 17:17:48 |
  | Modified | 05/02/2026, 17:17:48 |
- ## Benefits and challenges of animal-assisted therapy in older adults: a literature review

  |  |  |
  | --- | --- |
  | Item Type | Journal Article |
  | Author | Ashley Veilleux |
  | Date | 2021 |
  | Short Title | Benefits and challenges of animal-assisted therapy in older adults |
  | Library Catalogue | Google Scholar |
  | URL | https://search.proquest.com/openview/658ef1fd26b3639f8b0bfbe65e90fc1a/1?pq-origsite=gscholar&cbl=2042228 |
  | Accessed | 05/02/2026, 17:21:41 |
  | Volume | 36 |
  | Pages | 28–33 |
  | Publication | Nursing Standard |
  | Issue | 1 |
  | Date Added | 05/02/2026, 17:22:09 |
  | Modified | 05/02/2026, 17:22:09 |
- ## The Life Trajectory of Animal-Assisted Therapy Teams in Professional Mental Health Practice: Overview of Developmental and Practice Considerations

  |  |  |
  | --- | --- |
  | Item Type | Journal Article |
  | Author | Risë VanFleet |
  | Author | Aubrey H. Fine |
  | Author | Mary Rottier |
  | Author | Tracie Faa-Thompson |
  | Date | 2025 |
  | Short Title | The Life Trajectory of Animal-Assisted Therapy Teams in Professional Mental Health Practice |
  | Library Catalogue | Google Scholar |
  | URL | https://www.sciencedirect.com/science/article/pii/B9780443223464000202 |
  | Accessed | 05/02/2026, 17:28:18 |
  | Publisher | Elsevier |
  | Pages | 127–149 |
  | Publication | Handbook on Animal-Assisted Therapy |
  | Date Added | 05/02/2026, 17:28:56 |
  | Modified | 05/02/2026, 17:28:56 |
- ## Paper One: Outcomes and Implementation Characteristics Of College-Based Animal Assisted Activities: A Systematic Review of Randomized Trials

  |  |  |
  | --- | --- |
  | Item Type | Journal Article |
  | Author | Jaymie Lynne Vandagriff |
  | Author | Stephanie Kuzara |
  | Author | Patricia Pendry |
  | Date | 2021 |
  | Short Title | Paper One |
  | Library Catalogue | Google Scholar |
  | URL | https://rex.libraries.wsu.edu/view/pdfCoverPage?instCode=01ALLIANCE\_WSU&filePid=13354044520001842&download=true#page=52 |
  | Accessed | 05/02/2026, 17:28:50 |
  | Publisher | WASHINGTON STATE UNIVERSITY |
  | Pages | 42 |
  | Publication | EXAMINING ANIMAL ASSISTED ACTIVITIES AS STRESS PREVENTION AND MENTAL HEALTH PROMOTION STRATEGIES ON COLLEGE CAMPUSES |
  | Date Added | 05/02/2026, 17:28:56 |
  | Modified | 05/02/2026, 17:28:56 |

  ### Attachments

  - Available Version (via Google Scholar)
- ## Recognizing and mitigating canine stress during animal assisted interventions

  |  |  |
  | --- | --- |
  | Item Type | Journal Article |
  | Author | Lisa Townsend |
  | Author | Nancy R. Gee |
  | Date | 2021 |
  | Library Catalogue | Google Scholar |
  | URL | https://www.mdpi.com/2306-7381/8/11/254 |
  | Accessed | 05/02/2026, 17:21:47 |
  | Volume | 8 |
  | Publisher | MDPI |
  | Pages | 254 |
  | Publication | Veterinary Sciences |
  | Issue | 11 |
  | Date Added | 05/02/2026, 17:22:09 |
  | Modified | 05/02/2026, 17:22:09 |
- ## Effect of Animal Assisted Interventions on Inmates: A Systematic Review

  |  |  |
  | --- | --- |
  | Item Type | Journal Article |
  | Author | Zehra Su Topbaş |
  | Author | Nuray Şimşek |
  | Date | 2022 |
  | Short Title | Effect of Animal Assisted Interventions on Inmates |
  | Library Catalogue | Google Scholar |
  | URL | https://search.proquest.com/openview/b8977b03a653af4d69228927262fd5f9/1?pq-origsite=gscholar&cbl=166138 |
  | Accessed | 05/02/2026, 17:34:13 |
  | Volume | 14 |
  | Publisher | Psikiyatride Guncel Yaklasimlar: Current Approaches in Psychiatry |
  | Pages | 12–20 |
  | Publication | Psikiyatride Guncel Yaklasimlar |
  | Issue | 1 |
  | Date Added | 05/02/2026, 17:34:30 |
  | Modified | 05/02/2026, 17:34:30 |

  ### Attachments

  - Available Version (via Google Scholar)
- ## Animal assisted therapy

  |  |  |
  | --- | --- |
  | Item Type | Journal Article |
  | Author | Hayvan Destekli Tedavi |
  | Date | 2020 |
  | Library Catalogue | Google Scholar |
  | URL | https://www.ceeol.com/search/article-detail?id=848995 |
  | Accessed | 05/02/2026, 17:34:08 |
  | Volume | 12 |
  | Pages | 117–130 |
  | Publication | Current Approaches in Psychiatry |
  | Issue | 1 |
  | Date Added | 05/02/2026, 17:34:30 |
  | Modified | 05/02/2026, 17:34:30 |

  ### Attachments

  - Available Version (via Google Scholar)
- ## Therapeutic role of animals: a comprehensive literature review on the prevalent forms and species in animal-assisted interventions

  |  |  |
  | --- | --- |
  | Item Type | Journal Article |
  | Author | Dorota Szewczyk |
  | Author | Jakub Fiega |
  | Author | Milena Michalska |
  | Author | Urszula Żurek |
  | Author | Zuzanna Lubaszka |
  | Author | Ewa Sikorska |
  | Date | 2023 |
  | Short Title | Therapeutic role of animals |
  | Library Catalogue | Google Scholar |
  | URL | https://apcz.umk.pl/JEHS/article/view/45312 |
  | Accessed | 05/02/2026, 17:21:58 |
  | Volume | 45 |
  | Pages | 215–235 |
  | Publication | Journal of Education, Health and Sport |
  | Issue | 1 |
  | Date Added | 05/02/2026, 17:22:09 |
  | Modified | 05/02/2026, 17:22:09 |

  ### Attachments

  - Available Version (via Google Scholar)
- ## The role of physical activity in animal-assisted interventions for autism: A systematic review

  |  |  |
  | --- | --- |
  | Item Type | Journal Article |
  | Author | Ann-Marie Sylvia |
  | Author | Sofiya Alhassan |
  | Author | Katie Potter |
  | Abstract | Purpose: Animal-assisted interventions (AAIs) may have therapeutic effects for autism. Physical activity (PA) has many established benefits for autism. AAIs appear to involve PA, which may drive some of their beneficial effects. The purpose of this review was to quantify the extent to which AAIs involve PA and to describe the PA dose. Methods: A systematic search was conducted for relevant articles published between January 2015 and May 2022. A total of 1,949 articles were identified with 38 articles eligible for inclusion. PA involvement and dose were determined by matching the intervention description to PA values in the Compendium of Physical Activities. Results: PA was involved in 31 of the studies. Animal species included horses ( n = 17), dogs ( n = 17), dolphins ( n = 3), and cats ( n = 1). All horse, 70% of dog and 67% of dolphin studies included PA. PA dose varied; however, horse and dolphin studies involved moderate-to-vigorous intensity PA and dog studies involved light-intensity PA. PA was assessed in only one study. Conclusion: AAIs for autism involve PA but are not designed as PA interventions nor is PA measured. AAIs could be designed as animal-assisted PA interventions to capitalize on the benefits of PA for both overall health and characteristics of autism. |
  | Date | 01/2024 |
  | Language | en |
  | Short Title | The role of physical activity in animal-assisted interventions for autism |
  | Library Catalogue | DOI.org (Crossref) |
  | URL | https://journals.sagepub.com/doi/10.1177/27546330241249880 |
  | Accessed | 05/02/2026, 17:24:51 |
  | Volume | 2 |
  | Pages | 27546330241249880 |
  | Publication | Neurodiversity |
  | DOI | 10.1177/27546330241249880 |
  | Journal Abbr | Neurodiversity |
  | ISSN | 2754-6330, 2754-6330 |
  | Date Added | 05/02/2026, 17:25:16 |
  | Modified | 05/02/2026, 17:25:16 |

  ### Attachments

  - Available Version (via Google Scholar)
- ## Unconventional animal species participation in animal-assisted interventions and methods for measuring their experienced stress

  |  |  |
  | --- | --- |
  | Item Type | Journal Article |
  | Author | Éva Suba-Bokodi |
  | Author | István Nagy |
  | Author | Marcell Molnár |
  | Date | 2024 |
  | Library Catalogue | Google Scholar |
  | URL | https://www.mdpi.com/2076-2615/14/20/2935 |
  | Accessed | 05/02/2026, 17:13:03 |
  | Volume | 14 |
  | Publisher | MDPI |
  | Pages | 2935 |
  | Publication | Animals |
  | Issue | 20 |
  | Date Added | 05/02/2026, 17:13:35 |
  | Modified | 05/02/2026, 17:13:35 |
- ## Use of animal-assisted interventions in relieving pain in healthcare settings: A systematic review

  |  |  |
  | --- | --- |
  | Item Type | Journal Article |
  | Author | Meredith L. Stensland |
  | Author | Don D. McGeary |
  | Date | 2022 |
  | Short Title | Use of animal-assisted interventions in relieving pain in healthcare settings |
  | Library Catalogue | Google Scholar |
  | URL | https://www.sciencedirect.com/science/article/pii/S1744388121002188 |
  | Accessed | 05/02/2026, 17:24:41 |
  | Volume | 46 |
  | Publisher | Elsevier |
  | Pages | 101519 |
  | Publication | Complementary Therapies in Clinical Practice |
  | Date Added | 05/02/2026, 17:25:16 |
  | Modified | 05/02/2026, 17:25:16 |
- ## <i>Calm with horses?</i> A systematic review of animal-assisted interventions for improving social functioning in children with autism

  |  |  |
  | --- | --- |
  | Item Type | Journal Article |
  | Author | Jon H Sissons |
  | Author | Elise Blakemore |
  | Author | Hannah Shafi |
  | Author | Naomi Skotny |
  | Author | Donna M Lloyd |
  | Abstract | The aim of this systematic review was to evaluate the effect of animal-assisted interventions on social functioning in children with autism spectrum disorder, based on evidence from randomized control trials. Included studies were articles published in English, with school aged children from 4 to 18 years with autism spectrum disorder. Databases searched were MEDLINE, PsycINFO, EMBASE, Web of Science, CINAHL and Zoological Record. Data extraction from included studies included demographics and sample features, interventions and controls descriptions, outcome measures, study funding and descriptive statistics. Risk of bias was assessed, considering randomization, allocation concealment, blinding, attrition, selective reporting and other sources of bias. Studies were synthesized narratively based on the animal approach taken and the use of waitlist versus active controls. Nine studies were included reporting across eight trials. Studies overall reported improvements in social functioning following equine-assisted services, with preliminary evidence suggesting improvements are sustained in the short and medium term. Insufficient evidence was available to draw conclusions on the efficacy of other animal-assisted interventions. Future research should aim to address the limitations common to included designs. Lay abstract Children with autism typically experience difficulties interacting socially with others when compared to their non-autistic peers. Establishing how effective interventions are for improving social functioning is important to help inform what should be offered to children with autism. This study reviewed how effective interventions that involved interaction with a live animal, known as animal-assisted interventions, are in improving social functioning in children with autism. A systematic search of the evidence on this topic found nine studies, which were explored for the effectiveness of animal-assisted interventions and the quality of methods used. Overall, these studies showed improvements in social functioning following equine-assisted or therapeutic horse-riding interventions, with initial evidence showing improvements are sustained in the short and medium term. However, several issues were identified, which limit the strength of any conclusions that can be drawn from this evidence. For example, in many studies people assessing the children were aware that they received the intervention or were in a control group. There was also not enough evidence available to draw conclusions on the effectiveness of other animal-assisted interventions. Future research should address the limitations that were common in the designs of these studies and investigate the potential benefit of other animal populations, such as dogs and cats. |
  | Date | 08/2022 |
  | Language | en |
  | Short Title | <i>Calm with horses?</i> |
  | Library Catalogue | DOI.org (Crossref) |
  | URL | https://journals.sagepub.com/doi/10.1177/13623613221085338 |
  | Accessed | 05/02/2026, 17:21:41 |
  | Volume | 26 |
  | Pages | 1320-1340 |
  | Publication | Autism |
  | DOI | 10.1177/13623613221085338 |
  | Issue | 6 |
  | Journal Abbr | Autism |
  | ISSN | 1362-3613, 1461-7005 |
  | Date Added | 05/02/2026, 17:22:09 |
  | Modified | 05/02/2026, 17:22:09 |

  ### Attachments

  - Available Version (via Google Scholar)
- ## Animal-assisted and robotic animal-assisted interventions within dementia care: A systematic review

  |  |  |
  | --- | --- |
  | Item Type | Journal Article |
  | Author | Emily Shoesmith |
  | Author | Claire Surr |
  | Author | Elena Ratschen |
  | Abstract | Background Animal-assisted interventions and robotic animal interventions are becoming increasingly popular to support the care of people with dementia and may have the potential to improve a range of psychosocial outcomes. This review aims to identify, describe, and compare animal-assisted and robotic animal interventions delivered to people with dementia, their characteristics, effectiveness, and the proposed mechanisms underlying any potential impact. Methods A systematic literature search was conducted in MEDLINE, AMED, EMBASE, PsycINFO, OVID Nursing, PubMed, CINAHL and Web of Science. Random-effects meta-analyses of randomised controlled trials (RCTs) were conducted to summarise studies that evaluated common outcomes (agitation, depression, quality of life). A narrative approach was used to synthesise other findings. Results Fifty-one studies were included: 18 RCTs; 12 non-randomised trials, 13 cohort studies, 7 qualitative studies and one mixed-methods study. Meta-analyses were conducted for a small number of RCTs, with effectiveness of animal-assisted interventions demonstrated for agitation. Narrative findings suggested animal-assisted and robotic animal interventions may be promising in improving depression, agitation, and quality of life. Three potential mechanisms of action were identified for both animal-assisted and robotic animal interventions, namely enhancing social connections, providing engaging and meaningful activities, and the affect-generating aspect of the human-animal bond. A fourth mechanism was identified for animal-assisted interventions only: promoting physical activity. Robotic animals appear to have a place in complex human-animal relationships, but a greater understanding of robotic animal interventions is required to harness the benefits that may be derived from their use. Conclusion Delivering these interventions appear promising in improving psychosocial outcomes for people with dementia. As most included studies had methodological limitations, these findings are preliminary, but contribute to the body of evidence providing an understanding in terms of intervention characteristics and mechanisms of action. When developing intervention guidance, attention should be given to potential mechanisms and fundamental characteristics such as session content, delivery format and facilitator role. |
  | Date | 04/2023 |
  | Language | en |
  | Short Title | Animal-assisted and robotic animal-assisted interventions within dementia care |
  | Library Catalogue | DOI.org (Crossref) |
  | URL | https://journals.sagepub.com/doi/10.1177/14713012231155985 |
  | Accessed | 05/02/2026, 17:24:55 |
  | Volume | 22 |
  | Pages | 664-693 |
  | Publication | Dementia |
  | DOI | 10.1177/14713012231155985 |
  | Issue | 3 |
  | Journal Abbr | Dementia |
  | ISSN | 1471-3012, 1741-2684 |
  | Date Added | 05/02/2026, 17:25:16 |
  | Modified | 05/02/2026, 17:25:16 |

  ### Attachments

  - Available Version (via Google Scholar)
- ## Animal-Assisted Green Care Farming for Patients With Mental and Physical Disorders: A Narrative Review

  |  |  |
  | --- | --- |
  | Item Type | Journal Article |
  | Author | Alex Sargsyan |
  | Author | Lora H Beebe |
  | Abstract | Green care is an umbrella term that includes numerous therapeutic interventions that immerse the patient into nature and natural environments. Animal-assisted therapy (AAT) with farm animals is a component of green care that may benefit patients with mental health disorders. While the majority of research exploring farm AATs originated in Europe, interest in this therapeutic intervention is emerging in the United States. While there are green care farms utilizing animal therapies in the United States, these therapeutic interventions have not been reported in the scientific literature. Further exploration of this topic in the United States may benefit various patient populations and contribute to overall better health care. A literature review found that farm AATs may be beneficial for patients with mental health disorders, but did not identify any studies addressing AAT with farm animals in the context of physical health disorders. This may be a desirable direction for future research. |
  | Date | 05/2023 |
  | Language | en |
  | Short Title | Animal-Assisted Green Care Farming for Patients With Mental and Physical Disorders |
  | Library Catalogue | DOI.org (Crossref) |
  | URL | https://journals.sagepub.com/doi/10.1177/10784535231195433 |
  | Accessed | 05/02/2026, 17:34:05 |
  | Volume | 29 |
  | Pages | 192-196 |
  | Publication | Creative Nursing |
  | DOI | 10.1177/10784535231195433 |
  | Issue | 2 |
  | Journal Abbr | Creative Nursing |
  | ISSN | 1078-4535, 1946-1895 |
  | Date Added | 05/02/2026, 17:34:30 |
  | Modified | 05/02/2026, 17:34:30 |
- ## The research of standardized protocols for dog involvement in animal-assisted therapy: A systematic review

  |  |  |
  | --- | --- |
  | Item Type | Journal Article |
  | Author | Antonio Santaniello |
  | Author | Susanne Garzillo |
  | Author | Serena Cristiano |
  | Author | Alessandro Fioretti |
  | Author | Lucia Francesca Menna |
  | Date | 2021 |
  | Short Title | The research of standardized protocols for dog involvement in animal-assisted therapy |
  | Library Catalogue | Google Scholar |
  | URL | https://www.mdpi.com/2076-2615/11/9/2576 |
  | Accessed | 05/02/2026, 17:21:39 |
  | Volume | 11 |
  | Publisher | MDPI |
  | Pages | 2576 |
  | Publication | Animals |
  | Issue | 9 |
  | Date Added | 05/02/2026, 17:22:09 |
  | Modified | 05/02/2026, 17:22:09 |
- ## Methodological and terminological issues in animal-assisted interventions: an umbrella review of systematic reviews

  |  |  |
  | --- | --- |
  | Item Type | Journal Article |
  | Author | Antonio Santaniello |
  | Author | Francesca Dicé |
  | Author | Roberta Claudia Carratú |
  | Author | Alessia Amato |
  | Author | Alessandro Fioretti |
  | Author | Lucia Francesca Menna |
  | Date | 2020 |
  | Short Title | Methodological and terminological issues in animal-assisted interventions |
  | Library Catalogue | Google Scholar |
  | URL | https://www.mdpi.com/2076-2615/10/5/759 |
  | Accessed | 05/02/2026, 17:21:49 |
  | Volume | 10 |
  | Publisher | MDPI |
  | Pages | 759 |
  | Publication | Animals |
  | Issue | 5 |
  | Date Added | 05/02/2026, 17:22:09 |
  | Modified | 05/02/2026, 17:22:09 |
- ## Zoonotic risk of Encephalitozoon cuniculi in animal-assisted interventions: laboratory strategies for the diagnosis of infections in humans and animals

  |  |  |
  | --- | --- |
  | Item Type | Journal Article |
  | Author | Antonio Santaniello |
  | Author | Ilaria Cimmino |
  | Author | Ludovico Dipineto |
  | Author | Ayewa Lawoe Agognon |
  | Author | Francesco Beguinot |
  | Author | Pietro Formisano |
  | Author | Alessandro Fioretti |
  | Author | Lucia Francesca Menna |
  | Author | Francesco Oriente |
  | Date | 2021 |
  | Short Title | Zoonotic risk of Encephalitozoon cuniculi in animal-assisted interventions |
  | Library Catalogue | Google Scholar |
  | URL | https://www.mdpi.com/1660-4601/18/17/9333 |
  | Accessed | 05/02/2026, 17:21:51 |
  | Volume | 18 |
  | Publisher | MDPI |
  | Pages | 9333 |
  | Publication | International Journal of Environmental Research and Public Health |
  | Issue | 17 |
  | Date Added | 05/02/2026, 17:22:09 |
  | Modified | 05/02/2026, 17:22:09 |
- ## Systematic review and meta-analysis of the occurrence of ESKAPE bacteria group in dogs, and the related zoonotic risk in animal-assisted therapy, and in animal-assisted activity in the health context

  |  |  |
  | --- | --- |
  | Item Type | Journal Article |
  | Author | Antonio Santaniello |
  | Author | Mario Sansone |
  | Author | Alessandro Fioretti |
  | Author | Lucia Francesca Menna |
  | Date | 2020 |
  | Library Catalogue | Google Scholar |
  | URL | https://www.mdpi.com/1660-4601/17/9/3278 |
  | Accessed | 05/02/2026, 17:24:52 |
  | Volume | 17 |
  | Publisher | MDPI |
  | Pages | 3278 |
  | Publication | International journal of environmental research and public health |
  | Issue | 9 |
  | Date Added | 05/02/2026, 17:25:16 |
  | Modified | 05/02/2026, 17:25:16 |
- ## A Transdisciplinary Perspective on Dog-Handler-Client Interactions in Animal Assisted Activities for Children, Youth and Young Adults

  |  |  |
  | --- | --- |
  | Item Type | Journal Article |
  | Author | Renata P. S. Roma |
  | Author | Christine Yvette Tardif-Williams |
  | Author | Shannon A. Moore |
  | Author | Sandra L. Bosacki |
  | Abstract | Abstract A growing body of research has linked the inclusion of dogs in Animal-Assisted Activities (AAA) for children and young adults to a diverse range of positive social emotional and cognitive outcomes. However, many studies have focused exclusively on aspects directly related to dog-client interactions. There is a need to gain a better understanding of how dog-handler teams have been described, conceptualized and incorporated into the analysis in previous research. In addition, few studies have investigated the mutual adjustments inherent to dog-handler-client triadic relationships. This paper explores if and how the unique characteristics of dog-handler teams have been conceptualized and measured in previous studies. First, this paper undertakes a scoping review to map what, if any, characteristics of dogs, handlers, and dog-handler teams have been described and incorporated into the assessment of AAAs from 2004 to 2019 including: demographic characteristics, formal training and certification, handlers’ or dogs’ behavioral and physiological responses to AAAs, handlers’ roles during activities, and configuration of AAA teams. This scoping review also highlights key features of AAA teams requiring further investigation. In addition, this paper proposes the incorporation of a transdisciplinary framework to the analysis of AAAs. Such a holistic framework can inform the field of human-animal interactions by prioritizing a relational and contextual focus to the study of AAAs. |
  | Date | 12/2021 |
  | Language | en |
  | Library Catalogue | DOI.org (Crossref) |
  | URL | http://www.cabidigitallibrary.org/doi/10.1079/hai.2021.0026 |
  | Accessed | 05/02/2026, 17:34:00 |
  | Pages | hai.2021.0026 |
  | Publication | Human-animal interaction bulletin |
  | DOI | 10.1079/hai.2021.0026 |
  | Journal Abbr | Human-animal interaction bulletin |
  | ISSN | 2333-522X |
  | Date Added | 05/02/2026, 17:34:30 |
  | Modified | 05/02/2026, 17:34:30 |

  ### Attachments

  - Full Text PDF
- ## Evidence of animal-assisted therapy in neurological diseases in adults: a systematic review

  |  |  |
  | --- | --- |
  | Item Type | Journal Article |
  | Author | María del Carmen Rodríguez-Martínez |
  | Author | Alba De la Plana Maestre |
  | Author | Juan Antonio Armenta-Peinado |
  | Author | Miguel Ángel Barbancho |
  | Author | Natalia García-Casares |
  | Date | 2021 |
  | Short Title | Evidence of animal-assisted therapy in neurological diseases in adults |
  | Library Catalogue | Google Scholar |
  | URL | https://www.mdpi.com/1660-4601/18/24/12882 |
  | Accessed | 05/02/2026, 17:17:24 |
  | Volume | 18 |
  | Publisher | MDPI |
  | Pages | 12882 |
  | Publication | International Journal of Environmental Research and Public Health |
  | Issue | 24 |
  | Date Added | 05/02/2026, 17:17:48 |
  | Modified | 05/02/2026, 17:17:48 |
- ## Characterizing stress during animal interaction: a focus on the human endocrine response during equine-assisted services

  |  |  |
  | --- | --- |
  | Item Type | Journal Article |
  | Author | Brandon R. Rigby |
  | Date | 2023 |
  | Short Title | Characterizing stress during animal interaction |
  | Library Catalogue | Google Scholar |
  | URL | https://www.frontiersin.org/journals/veterinary-science/articles/10.3389/fvets.2023.1303354/full |
  | Accessed | 05/02/2026, 17:13:12 |
  | Volume | 10 |
  | Publisher | Frontiers Media SA |
  | Pages | 1303354 |
  | Publication | Frontiers in Veterinary Science |
  | Date Added | 05/02/2026, 17:13:35 |
  | Modified | 05/02/2026, 17:13:35 |

  ### Attachments

  - Available Version (via Google Scholar)
- ## The effectiveness of animal-assisted therapy for children and adolescents with autism spectrum disorder: A systematic review

  |  |  |
  | --- | --- |
  | Item Type | Journal Article |
  | Author | Amy Kate Rehn |
  | Author | Victoria Rose Caruso |
  | Author | Saravana Kumar |
  | Date | 2023 |
  | Short Title | The effectiveness of animal-assisted therapy for children and adolescents with autism spectrum disorder |
  | Library Catalogue | Google Scholar |
  | URL | https://www.sciencedirect.com/science/article/pii/S1744388122001876 |
  | Accessed | 05/02/2026, 17:21:32 |
  | Volume | 50 |
  | Publisher | Elsevier |
  | Pages | 101719 |
  | Publication | Complementary Therapies in Clinical Practice |
  | Date Added | 05/02/2026, 17:22:09 |
  | Modified | 05/02/2026, 17:22:09 |
- ## Effect of Animal-Assisted Activities on Symptoms and Emotions of Children with Neoplastic Disease: A Systematic Review with Meta-Analysis

  |  |  |
  | --- | --- |
  | Item Type | Journal Article |
  | Author | Luca Giuseppe Re |
  | Author | Silvia Porcarelli |
  | Author | Camilla Ripari |
  | Author | Sara Marotta |
  | Short Title | Effect of Animal-Assisted Activities on Symptoms and Emotions of Children with Neoplastic Disease |
  | Library Catalogue | Google Scholar |
  | URL | https://www.researchgate.net/profile/Luca-Re/publication/382348092\_Effect\_of\_Animal-Assisted\_Activities\_on\_Symptoms\_and\_Emotions\_of\_Children\_with\_Neoplastic\_Disease\_A\_Systematic\_Review\_with\_Meta-Analysis/links/669a4d888dca9f441b883b4c/Effect-of-Animal-Assisted-Activities-on-Symptoms-and-Emotions-of-Children-with-Neoplastic-Disease-A-Systematic-Review-with-Meta-Analysis.pdf |
  | Accessed | 05/02/2026, 17:28:39 |
  | Date Added | 05/02/2026, 17:28:56 |
  | Modified | 05/02/2026, 17:28:56 |

  ### Attachments

  - Available Version (via Google Scholar)
- ## Animal assisted intervention for oncology and palliative care patients: A systematic review

  |  |  |
  | --- | --- |
  | Item Type | Journal Article |
  | Author | Karina Diniz Pinto |
  | Author | Claudia Teresa Vieira de Souza |
  | Author | Maria de Lourdes Benamor Teixeira |
  | Author | Maria Isabel Fragoso da Silveira Gouvêa |
  | Date | 2021 |
  | Short Title | Animal assisted intervention for oncology and palliative care patients |
  | Library Catalogue | Google Scholar |
  | URL | https://www.sciencedirect.com/science/article/pii/S1744388121000463 |
  | Accessed | 05/02/2026, 17:17:35 |
  | Volume | 43 |
  | Publisher | Elsevier |
  | Pages | 101347 |
  | Publication | Complementary Therapies in Clinical Practice |
  | Date Added | 05/02/2026, 17:17:48 |
  | Modified | 05/02/2026, 17:17:48 |
- ## Effects of Music and Animal-Assisted Therapy on Opioid Use Disorder

  |  |  |
  | --- | --- |
  | Item Type | Journal Article |
  | Author | Riley Perry |
  | Author | Emily Bushy |
  | Author | Emma Glover |
  | Author | Kathryn Jensen |
  | Author | Julia Pelkey |
  | Date | 2025 |
  | Library Catalogue | Google Scholar |
  | URL | https://digitalcommons.library.umaine.edu/student\_work/82/ |
  | Accessed | 05/02/2026, 17:34:27 |
  | Date Added | 05/02/2026, 17:34:30 |
  | Modified | 05/02/2026, 17:34:30 |

  ### Attachments

  - Available Version (via Google Scholar)
- ## Animal-assisted and pet-robot interventions for ameliorating behavioral and psychological symptoms of dementia: a systematic review and meta-analysis

  |  |  |
  | --- | --- |
  | Item Type | Journal Article |
  | Author | Sangki Park |
  | Author | Ahream Bak |
  | Author | Sujin Kim |
  | Author | Yunkwon Nam |
  | Author | Hyeon soo Kim |
  | Author | Doo-Han Yoo |
  | Author | Minho Moon |
  | Date | 2020 |
  | Short Title | Animal-assisted and pet-robot interventions for ameliorating behavioral and psychological symptoms of dementia |
  | Library Catalogue | Google Scholar |
  | URL | https://www.mdpi.com/2227-9059/8/6/150 |
  | Accessed | 05/02/2026, 17:17:43 |
  | Volume | 8 |
  | Publisher | MDPI |
  | Pages | 150 |
  | Publication | Biomedicines |
  | Issue | 6 |
  | Date Added | 05/02/2026, 17:17:48 |
  | Modified | 05/02/2026, 17:17:48 |
- ## Animal-assisted interventions for the improvement of mental health outcomes in higher education students: A systematic review of randomised controlled trials

  |  |  |
  | --- | --- |
  | Item Type | Journal Article |
  | Author | Charlotte Parbery-Clark |
  | Author | Marvellas Lubamba |
  | Author | Louise Tanner |
  | Author | Elaine McColl |
  | Date | 2021 |
  | Short Title | Animal-assisted interventions for the improvement of mental health outcomes in higher education students |
  | Library Catalogue | Google Scholar |
  | URL | https://www.mdpi.com/1660-4601/18/20/10768 |
  | Accessed | 05/02/2026, 17:22:01 |
  | Volume | 18 |
  | Publisher | MDPI |
  | Pages | 10768 |
  | Publication | International journal of environmental research and public health |
  | Issue | 20 |
  | Date Added | 05/02/2026, 17:22:09 |
  | Modified | 05/02/2026, 17:22:09 |
- ## The role of animal-assisted therapy in enhancing patients’ well-being: Systematic study of the qualitative and quantitative evidence

  |  |  |
  | --- | --- |
  | Item Type | Journal Article |
  | Author | Ramendra Pati Pandey |
  | Author | Riya Mukherjee |
  | Author | Chung-Ming Chang |
  | Date | 2024 |
  | Short Title | The role of animal-assisted therapy in enhancing patients’ well-being |
  | Library Catalogue | Google Scholar |
  | URL | https://xmed.jmir.org/2024/1/e51787/ |
  | Accessed | 05/02/2026, 17:13:07 |
  | Volume | 5 |
  | Publisher | JMIR Publications Inc., Toronto, Canada |
  | Pages | e51787 |
  | Publication | Jmirx med |
  | Issue | 1 |
  | Date Added | 05/02/2026, 17:13:35 |
  | Modified | 05/02/2026, 17:13:35 |
- ## Animal-Assisted Therapy in palliative care: a scoping review

  |  |  |
  | --- | --- |
  | Item Type | Journal Article |
  | Author | Laura Palomino-Lázaro |
  | Author | María Rueda-Extremera |
  | Author | María Cantero-García |
  | Date | 2024 |
  | Short Title | Animal-Assisted Therapy in palliative care |
  | Library Catalogue | Google Scholar |
  | URL | https://www.frontiersin.org/journals/psychology/articles/10.3389/fpsyg.2024.1478264/full |
  | Accessed | 05/02/2026, 17:17:19 |
  | Volume | 15 |
  | Publisher | Frontiers Media SA |
  | Pages | 1478264 |
  | Publication | Frontiers in Psychology |
  | Date Added | 05/02/2026, 17:17:48 |
  | Modified | 05/02/2026, 17:17:48 |

  ### Attachments

  - Available Version (via Google Scholar)
- ## Animal‐assisted interventions in adult hospital rehabilitation settings: A scoping review

  |  |  |
  | --- | --- |
  | Item Type | Journal Article |
  | Author | Mary O'Loughlin |
  | Author | Rachael Edwards |
  | Author | Em Bould |
  | Author | Sue Devine |
  | Author | Sandra Downing |
  | Abstract | Abstract Animal‐assisted interventions (AAIs) have the potential to enhance people's well‐being and function and are increasingly being implemented across a range of settings. This scoping review explored how AAIs have been used in adult hospital rehabilitative care. Using JBI and PRISMA‐ScR guidelines, a systematic search of four databases was undertaken. Inclusion criteria involved adults, aged >18 years, who had received AAIs in the hospital rehabilitation setting. Twenty‐two articles met the inclusion criteria. Results identified two intervention types: visitation activities ( n  = 8 studies) and structured therapeutic interventions ( n  = 14 studies). Dogs were the most common animal species. Improvements in social and emotional well‐being were reported across both types of interventions, with improvements in ambulation, motor skills, and verbal communication reported by those engaged in structured therapeutic interventions. Implementation challenges included a dependency on volunteer dog‐handlers; the need for better recording of interventions in medical records to enable evaluation; and cost, safety, infection control, and animal welfare considerations. Strengthening the planning of AAIs is fundamental for the realization of potential outcomes from human–animal interactions in hospital rehabilitative care. |
  | Date | 09/2024 |
  | Language | en |
  | Short Title | Animal‐assisted interventions in adult hospital rehabilitation settings |
  | Library Catalogue | DOI.org (Crossref) |
  | URL | https://onlinelibrary.wiley.com/doi/10.1111/nhs.13138 |
  | Accessed | 05/02/2026, 17:28:16 |
  | Volume | 26 |
  | Pages | e13138 |
  | Publication | Nursing & Health Sciences |
  | DOI | 10.1111/nhs.13138 |
  | Issue | 3 |
  | Journal Abbr | Nursing &amp; Health Sciences |
  | ISSN | 1441-0745, 1442-2018 |
  | Date Added | 05/02/2026, 17:28:56 |
  | Modified | 05/02/2026, 17:28:56 |

  ### Attachments

  - Full Text PDF
- ## Animal-Assisted Interventions for Autism Spectrum Disorder: A Systematic Review of the Literature from 2016 to 2020

  |  |  |
  | --- | --- |
  | Item Type | Journal Article |
  | Author | Leanne O. Nieforth |
  | Author | A. J. Schwichtenberg |
  | Author | Marguerite E. O’Haire |
  | Date | 06/2023 |
  | Language | en |
  | Short Title | Animal-Assisted Interventions for Autism Spectrum Disorder |
  | Library Catalogue | DOI.org (Crossref) |
  | URL | https://link.springer.com/10.1007/s40489-021-00291-6 |
  | Accessed | 05/02/2026, 17:17:42 |
  | Volume | 10 |
  | Pages | 255-280 |
  | Publication | Review Journal of Autism and Developmental Disorders |
  | DOI | 10.1007/s40489-021-00291-6 |
  | Issue | 2 |
  | Journal Abbr | Rev J Autism Dev Disord |
  | ISSN | 2195-7177, 2195-7185 |
  | Date Added | 05/02/2026, 17:17:48 |
  | Modified | 05/02/2026, 17:17:48 |
- ## A Reflection on the Current Status of Animal-Assisted Therapy in India

  |  |  |
  | --- | --- |
  | Item Type | Journal Article |
  | Author | Hemangi Narayan Narvekar |
  | Date | 12/2023 |
  | Language | en |
  | Library Catalogue | DOI.org (Crossref) |
  | URL | https://link.springer.com/10.1007/s42087-021-00250-x |
  | Accessed | 05/02/2026, 17:34:07 |
  | Volume | 6 |
  | Pages | 760-775 |
  | Publication | Human Arenas |
  | DOI | 10.1007/s42087-021-00250-x |
  | Issue | 4 |
  | Journal Abbr | Hu Arenas |
  | ISSN | 2522-5790, 2522-5804 |
  | Date Added | 05/02/2026, 17:34:30 |
  | Modified | 05/02/2026, 17:34:30 |

  ### Attachments

  - Available Version (via Google Scholar)
- ## Animal-Assisted Interventions: A Literature Review on Diverse Applications of Animal-Assisted Interventions in selected medical entities in different age groups

  |  |  |
  | --- | --- |
  | Item Type | Journal Article |
  | Author | Agnieszka Najdek |
  | Author | Dorota Szewczyk |
  | Author | Julia Nowak |
  | Author | Aleksandra Woźniak |
  | Author | Michał Jakub Cioch |
  | Author | Marcin Mycyk |
  | Author | Urszula Kaczmarska |
  | Author | Daria Oleksy |
  | Author | Katarzyna Doman |
  | Author | Kamil Hermanowicz |
  | Date | 2025 |
  | Short Title | Animal-Assisted Interventions |
  | Library Catalogue | Google Scholar |
  | URL | https://apcz.umk.pl/JEHS/article/view/57671 |
  | Accessed | 05/02/2026, 17:25:16 |
  | Volume | 78 |
  | Pages | 57671–57671 |
  | Publication | Journal of Education, Health and Sport |
  | Date Added | 05/02/2026, 17:25:16 |
  | Modified | 05/02/2026, 17:25:16 |

  ### Attachments

  - Available Version (via Google Scholar)
- ## Animal-Assisted Therapy in Dentistry: A Review

  |  |  |
  | --- | --- |
  | Item Type | Journal Article |
  | Author | Begüm Mutlu |
  | Author | Mine Keskin |
  | Author | Yelda Kasımoğlu |
  | Date | 2025 |
  | Short Title | Animal-Assisted Therapy in Dentistry |
  | Library Catalogue | Google Scholar |
  | URL | https://www.essentdent.org/index.php/pub/article/view/103 |
  | Accessed | 05/02/2026, 17:25:02 |
  | Volume | 4 |
  | Pages | 1–9 |
  | Publication | Essentials of Dentistry |
  | Issue | 1 |
  | Date Added | 05/02/2026, 17:25:16 |
  | Modified | 05/02/2026, 17:25:16 |

  ### Attachments

  - Available Version (via Google Scholar)
- ## Animal assisted education and social communication competency: systematic literature review

  |  |  |
  | --- | --- |
  | Item Type | Journal Article |
  | Author | Sinéad Morgan |
  | Date | 2024-11-29 |
  | Language | en |
  | Short Title | Animal assisted education and social communication competency |
  | Library Catalogue | DOI.org (Crossref) |
  | URL | https://link.springer.com/10.1007/s44217-024-00357-7 |
  | Accessed | 05/02/2026, 17:24:31 |
  | Volume | 3 |
  | Pages | 258 |
  | Publication | Discover Education |
  | DOI | 10.1007/s44217-024-00357-7 |
  | Issue | 1 |
  | Journal Abbr | Discov Educ |
  | ISSN | 2731-5525 |
  | Date Added | 05/02/2026, 17:25:16 |
  | Modified | 05/02/2026, 17:25:16 |

  ### Attachments

  - Available Version (via Google Scholar)
- ## Are there criteria for inclusion of dogs for animal assisted interventions at universities?

  |  |  |
  | --- | --- |
  | Item Type | Journal Article |
  | Author | Maria Cecília Pianaro Mores |
  | Author | Ana Carolina Rizzon Cintra |
  | Author | Carolina Zaghi Cavalcante |
  | Author | Cristina Santos Sotomaior |
  | Author | Ana Lúcia Lacerda Michelotto |
  | Date | 2021 |
  | Library Catalogue | Google Scholar |
  | URL | https://www.cabidigitallibrary.org/doi/full/10.5555/20220175785 |
  | Accessed | 05/02/2026, 17:34:23 |
  | Date Added | 05/02/2026, 17:34:30 |
  | Modified | 05/02/2026, 17:34:30 |
- ## Effectiveness of animal-assisted services for school-aged children: a systematic review

  |  |  |
  | --- | --- |
  | Item Type | Journal Article |
  | Author | Ingyin Moe |
  | Author | Pei Ju Ho |
  | Author | Maria Andersson |
  | Author | Sara Karlberg |
  | Author | Lena Lidfors |
  | Author | Filipa Sampaio |
  | Author | Inna Feldman |
  | Abstract | Abstract Animal-assisted services (AAS) have demonstrated potential benefits for children and adolescents, particularly in emotional development and educational outcomes. However, existing systematic reviews often focused on specific populations or types of animals, and overlook the quality of included studies. This review evaluates the effectiveness of animal-assisted services on the well-being and behavior of school-aged children, and assesses the risk of bias of the included studies. Following PRISMA Guidelines, a systematic search was conducted in MEDLINE, CINAHL, PsycARTICLES, PsycINFO via EBSCOhost, Cochrane Database of Systematic Reviews, and Web of Science, from database inception until August 15, 2023. Risk of bias was assessed using the Cochrane Risk of Bias 2 for randomized trials and ROBINS-I for non-randomized studies. Two reviewers (IM, PJH) assessed each record, and two additional reviewers (IF, FS) resolved disagreements. Data were synthesized narratively, and presented in tables and figures. From 2,380 initial articles, 30 met the inclusion criteria. Studies were primarily randomized controlled trials ( n  = 21), involving various populations, from children with autism spectrum disorders ( n  = 10) to general population ( n  = 8). Outcomes were grouped into abilities, attitudes, externalizing behaviors, internalizing behaviors, disorder-specific measures, and well-being. AAS showed positive impacts on socio-emotional functioning and behavior despite diverse intervention approaches. Nonetheless, all studies had study design problems, which caused high risk of bias. AAS appears to enhance cognitive, behavioral, and socio-emotional aspects, as well as learning in school-aged children. Further methodologically rigorous research and evidence, such as implementation studies and cost-effectiveness analyses comparing AAS to standard practices are needed to support decision-making. |
  | Date | 10/2025 |
  | Language | en |
  | Short Title | Effectiveness of animal-assisted services for school-aged children |
  | Library Catalogue | DOI.org (Crossref) |
  | URL | https://link.springer.com/10.1007/s00787-025-02740-7 |
  | Accessed | 05/02/2026, 17:13:33 |
  | Volume | 34 |
  | Pages | 3017-3033 |
  | Publication | European Child & Adolescent Psychiatry |
  | DOI | 10.1007/s00787-025-02740-7 |
  | Issue | 10 |
  | Journal Abbr | Eur Child Adolesc Psychiatry |
  | ISSN | 1018-8827, 1435-165X |
  | Date Added | 05/02/2026, 17:13:35 |
  | Modified | 05/02/2026, 17:13:35 |

  ### Attachments

  - Available Version (via Google Scholar)
- ## The effect of animal-assisted interventions on the course of neurological diseases: a systematic review

  |  |  |
  | --- | --- |
  | Item Type | Journal Article |
  | Author | Veronika Mittly |
  | Author | Cecilia Farkas-Kirov |
  | Author | Ágnes Zana |
  | Author | Kata Szabó |
  | Author | Veronika Ónodi-Szabó |
  | Author | György Purebl |
  | Abstract | Abstract Background In our experience, working with a therapy animal strengthens endurance, maintains motivation, provides a sense of achievement, and boosts overall mental resilience. The aims of this work were to summarize the results of quantitative research on the possibilities of animal-assisted intervention (AAI) among people with neurodegenerative and cerebrovascular diseases and to attempt to assess the effects of animal-assisted interventions in an objective manner and to find supporting evidence based on published literature. Methods Our target groups are people diagnosed with Parkinson’s disease, multiple sclerosis, or stroke. A systematic search of relevant articles was conducted by two independent researchers in April 2021 and August 2023. The search for studies was conducted using PubMed, Google Scholar, Web of Science, Scopus, and Ovid databases, specifying keywords and search criteria. The qualitative evaluation of the research reports was conducted by four independent researchers, using the Newcastle–Ottawa Quality Assessment Form. Results According to the scientific criteria and based on the Newcastle–Ottawa Quality Assessment Form, thirteen publications met the search criteria, out of which 9 publications were rated good and 4 publications were rated poor. Evaluating the publications we found evidence that AAI had a measurable impact on participants, as their physical and mental health status significantly improved; however, mental health improvement was more prominent. Conclusions By developing evidence-based research methodology and standardized research settings, AAI could be measured effectively as part of health care practice. This would bring significant benefits to the rehabilitation of patients in need. Systematic review registration PROSPERO CRD42021255776. |
  | Date | 2023-11-25 |
  | Language | en |
  | Short Title | The effect of animal-assisted interventions on the course of neurological diseases |
  | Library Catalogue | DOI.org (Crossref) |
  | URL | https://systematicreviewsjournal.biomedcentral.com/articles/10.1186/s13643-023-02387-y |
  | Accessed | 05/02/2026, 17:13:31 |
  | Volume | 12 |
  | Pages | 224 |
  | Publication | Systematic Reviews |
  | DOI | 10.1186/s13643-023-02387-y |
  | Issue | 1 |
  | Journal Abbr | Syst Rev |
  | ISSN | 2046-4053 |
  | Date Added | 05/02/2026, 17:13:35 |
  | Modified | 05/02/2026, 17:13:35 |

  ### Attachments

  - Available Version (via Google Scholar)
- ## Animal-assisted interventions with dogs in special education—A systematic review

  |  |  |
  | --- | --- |
  | Item Type | Journal Article |
  | Author | Jana Meixner |
  | Author | Kurt Kotrschal |
  | Date | 2022 |
  | Library Catalogue | Google Scholar |
  | URL | https://www.frontiersin.org/journals/psychology/articles/10.3389/fpsyg.2022.876290/full |
  | Accessed | 05/02/2026, 17:13:19 |
  | Volume | 13 |
  | Publisher | Frontiers Media SA |
  | Pages | 876290 |
  | Publication | Frontiers in psychology |
  | Date Added | 05/02/2026, 17:13:35 |
  | Modified | 05/02/2026, 17:13:35 |

  ### Attachments

  - Available Version (via Google Scholar)
- ## Animal-Assisted Therapies for Youth with or at risk for Mental Health Problems: A Systematic Review

  |  |  |
  | --- | --- |
  | Item Type | Journal Article |
  | Author | LMSW Meghan Morrissey |
  | Author | Robin Peth-Pierce |
  | Short Title | Animal-Assisted Therapies for Youth with or at risk for Mental Health Problems |
  | Library Catalogue | Google Scholar |
  | URL | https://www.academia.edu/download/99162317/pmc5546745.pdf |
  | Accessed | 05/02/2026, 17:28:49 |
  | Date Added | 05/02/2026, 17:28:56 |
  | Modified | 05/02/2026, 17:28:56 |

  ### Attachments

  - Available Version (via Google Scholar)
- ## Animal‐assisted services for adults with acquired neurogenic communication disorders: A scoping review

  |  |  |
  | --- | --- |
  | Item Type | Journal Article |
  | Author | Marie‐Pier McSween |
  | Author | Tasman Day |
  | Author | Jessica Hill |
  | Author | Sarah J. Wallace |
  | Abstract | Abstract Background There is increasing interest in the incorporation of animal‐assisted services (AAS) in therapy for adults with acquired neurogenic communication disorders. AAS have the potential to enhance speech and language therapy engagement and outcomes. However, a greater understanding of the nature and potential benefits of these interventions is needed. Aims To describe the existing evidence for the incorporation of AAS in therapy with adults with acquired neurogenic communication disorders and to identify areas for future research. Methods & Procedures A scoping review was conducted and reported in alignment with the Preferred Reporting Items for Systematic Reviews and Meta‐analyses checklist extension for scoping reviews (PRISMA‐ScR). Seven databases (PubMed, Embase, CINAHL, PsycINFO, Cochrane, Scopus, Web of Science) and grey literature (Google) were searched. Two reviewers independently screened titles, abstracts and full texts against eligibility criteria using Covidence software. The Template for Intervention Description and Replication (TIDieR) checklist guided extraction of intervention data. Main Contributions A total of 17 studies with adults with aphasia, apraxia of speech and cognitive–communication disorders were included. While terminology varied, most interventions met the definition of animal‐assisted therapy or animal‐assisted activity and used therapy dogs. Across studies, a range of outcomes were targeted, and positive benefits were reported for participant mood, emotions, motivation and satisfaction. There were mixed, but mostly positive, benefits on social behaviour, communication and participation. Conclusions & Implications: AAS has been incorporated in therapy for adults with acquired neurogenic communication disorders, predominately with people with dementia. Across studies, communication impairments and AAS interventions were insufficiently or inconsistently described. Improved reporting would assist understanding of the potential benefits of AAS as an adjunct therapy. A quality appraisal of existing studies, and meta‐analysis of findings, is needed to draw conclusions about the effectiveness of AAS as a complementary therapy for people with acquired neurogenic communication disorders. WHAT THIS PAPER ADDS What is already known on this subject There is increasing interest and research in AAS as an adjunct to traditional speech and language therapy. Several clinical populations have been shown to benefit from the incorporation of AAS as a complementary therapy approach, including adults with acquired neurogenic communication disorders. To date there has not been a comprehensive review of literature in the area. What this paper adds to the existing knowledge This review aimed to describe what is known about AAS as an adjunct intervention for adults with acquired neurogenic communication disorders. A total of 17 studies were identified, the majority conducted with people living with dementia. Overall, consistent positive benefits of AAS were reported on participant mood, positive emotions, motivation, and satisfaction. There were mixed, but mostly positive, benefits of AAS on social behaviour, including both verbal and non‐verbal communication outcomes, and participation. What are the practical and clinical implications of this work? These results suggest a potential therapeutic benefit for the inclusion of AAS into traditional modalities used by speech–language therapists with people with acquired neurogenic communication disorders. However, clinicians should view these findings with caution. Studies were often insufficiently or inconsistently reported, and a quality appraisal and meta‐analysis of existing studies would be needed to draw clear conclusions on the effectiveness of AAS as a complementary approach for people with acquired neurogenic communication disorders. |
  | Date | 11/2024 |
  | Language | en |
  | Short Title | Animal‐assisted services for adults with acquired neurogenic communication disorders |
  | Library Catalogue | DOI.org (Crossref) |
  | URL | https://onlinelibrary.wiley.com/doi/10.1111/1460-6984.13119 |
  | Accessed | 05/02/2026, 17:28:21 |
  | Volume | 59 |
  | Pages | 2858-2877 |
  | Publication | International Journal of Language & Communication Disorders |
  | DOI | 10.1111/1460-6984.13119 |
  | Issue | 6 |
  | Journal Abbr | Intl J Lang &amp; Comm Disor |
  | ISSN | 1368-2822, 1460-6984 |
  | Date Added | 05/02/2026, 17:28:56 |
  | Modified | 05/02/2026, 17:28:56 |
- ## Effectiveness of Animal-assisted Interventions (AAIs) in Treatment of Adults with Depressive Symptoms: A Systematic Review

  |  |  |
  | --- | --- |
  | Item Type | Journal Article |
  | Author | Chris McFalls-Steger |
  | Author | David Patterson |
  | Author | Phyllis Thompson |
  | Abstract | Abstract Animal-assisted interventions (AAIs) have become widespread, with programs targeting various populations and mental health conditions. Despite its popularity, AAI’s operational definition and its efficacy are unclear. This systematic review aims to assess the utility of AAIs in decreasing depressive symptoms in adults based upon results of empirically validated depression assessment scales used by researchers. A systematic published literature search was conducted using Web of Science, PsychInfo, PubMed, ProQuest, SCOPUS, CINAHL, Social Work Abstracts, Web of Science, and Google Scholar. Peer-reviewed research articles on the effectiveness of AAIs on depressive symptoms in adults using empirically validated depression scales published from 2010 through October 2020 were chosen for this systematic review. Search results were filtered to include only quantitative, peer-reviewed articles for adults 18 and over; those were reviewed, and only journal articles using an empirically established depression evaluation tool were chosen. A total of 10 quantitative articles met these inclusion criteria. Overall, research design quality was low, but AAI had a statistically significant effect on outcomes in most studies. Results are moderately favorable but more thorough, standardized, and controlled research is needed. |
  | Date | 12/2021 |
  | Language | en |
  | Short Title | Effectiveness of Animal-assisted Interventions (AAIs) in Treatment of Adults with Depressive Symptoms |
  | Library Catalogue | DOI.org (Crossref) |
  | URL | http://www.cabidigitallibrary.org/doi/10.1079/hai.2021.0007 |
  | Accessed | 05/02/2026, 17:28:25 |
  | Pages | hai.2021.0007 |
  | Publication | Human-animal interaction bulletin |
  | DOI | 10.1079/hai.2021.0007 |
  | Journal Abbr | Human-animal interaction bulletin |
  | ISSN | 2333-522X |
  | Date Added | 05/02/2026, 17:28:56 |
  | Modified | 05/02/2026, 17:28:56 |

  ### Attachments

  - Full Text PDF
- ## Animal-assisted interventions in intensive care delirium: a literature review

  |  |  |
  | --- | --- |
  | Item Type | Journal Article |
  | Author | Jahanzeb Malik |
  | Date | 2021 |
  | Short Title | Animal-assisted interventions in intensive care delirium |
  | Library Catalogue | Google Scholar |
  | URL | https://aacnjournals.org/aacnacconline/article-abstract/32/4/391/31636 |
  | Accessed | 05/02/2026, 17:17:37 |
  | Volume | 32 |
  | Publisher | American Association of Critical Care Nurses |
  | Pages | 391–397 |
  | Publication | AACN advanced critical care |
  | Issue | 4 |
  | Date Added | 05/02/2026, 17:17:48 |
  | Modified | 05/02/2026, 17:17:48 |

  ### Attachments

  - Available Version (via Google Scholar)
- ## Animal-assisted activities in the intensive care unit: a scoping review

  |  |  |
  | --- | --- |
  | Item Type | Journal Article |
  | Author | Tania Lovell |
  | Author | Kristen Ranse |
  | Date | 2022 |
  | Short Title | Animal-assisted activities in the intensive care unit |
  | Library Catalogue | Google Scholar |
  | URL | https://www.sciencedirect.com/science/article/pii/S0964339722001070 |
  | Accessed | 05/02/2026, 17:22:03 |
  | Volume | 73 |
  | Publisher | Elsevier |
  | Pages | 103304 |
  | Publication | Intensive and Critical Care Nursing |
  | Date Added | 05/02/2026, 17:22:09 |
  | Modified | 05/02/2026, 17:22:09 |
- ## Human-animal interaction in animal-assisted interventions (AAI) s: zoonosis risks, benefits, and future directions—a one health approach

  |  |  |
  | --- | --- |
  | Item Type | Journal Article |
  | Author | Giovanna Liguori |
  | Author | Anna Costagliola |
  | Author | Renato Lombardi |
  | Author | Orlando Paciello |
  | Author | Antonio Giordano |
  | Date | 2023 |
  | Short Title | Human-animal interaction in animal-assisted interventions (AAI) s |
  | Library Catalogue | Google Scholar |
  | URL | https://www.mdpi.com/2076-2615/13/10/1592 |
  | Accessed | 05/02/2026, 17:24:54 |
  | Volume | 13 |
  | Publisher | MDPI |
  | Pages | 1592 |
  | Publication | Animals |
  | Issue | 10 |
  | Date Added | 05/02/2026, 17:25:16 |
  | Modified | 05/02/2026, 17:25:16 |
- ## A text-mining analysis of research trends in animal-assisted therapy

  |  |  |
  | --- | --- |
  | Item Type | Journal Article |
  | Author | Shin-Ja Lee |
  | Author | Geun-Hyeon Kim |
  | Author | Yea-Hwang Moon |
  | Author | Sung-Sill Lee |
  | Date | 2023 |
  | Library Catalogue | Google Scholar |
  | URL | https://www.mdpi.com/2076-2615/13/19/3133 |
  | Accessed | 05/02/2026, 17:13:05 |
  | Volume | 13 |
  | Publisher | MDPI |
  | Pages | 3133 |
  | Publication | Animals |
  | Issue | 19 |
  | Date Added | 05/02/2026, 17:13:35 |
  | Modified | 05/02/2026, 17:13:35 |
- ## Emerging Therapies for Neurological Disorders: A Clinical Review of MANAGED (Music, Art, Nature-Based, Animal-Assisted, Game, Essential Oil, Dance) Care

  |  |  |
  | --- | --- |
  | Item Type | Journal Article |
  | Author | Alyssa Wan-Chei Lee |
  | Author | Rahim Hirani |
  | Author | Jonathan Ogulnick |
  | Author | Raj K. Tiwari |
  | Author | Mill Etienne |
  | Date | 2025 |
  | Short Title | Emerging Therapies for Neurological Disorders |
  | Library Catalogue | Google Scholar |
  | URL | https://www.mdpi.com/2673-4087/6/2/51 |
  | Accessed | 05/02/2026, 17:21:35 |
  | Volume | 6 |
  | Publisher | MDPI |
  | Pages | 51 |
  | Publication | NeuroSci |
  | Issue | 2 |
  | Date Added | 05/02/2026, 17:22:09 |
  | Modified | 05/02/2026, 17:22:09 |
- ## Benefits of animal-Assisted interventions in preschool children: A systematic review

  |  |  |
  | --- | --- |
  | Item Type | Journal Article |
  | Author | Ana Myriam Lavín-Pérez |
  | Author | Beatriz Rivera-Martín |
  | Author | Luis Lucio Lobato-Rincón |
  | Author | Santos Villafaina-Domínguez |
  | Author | Daniel Collado-Mateo |
  | Abstract | Animal-assisted interventions are frequently used to stimulate and improve different skills in children with and without disabilities. However, the heterogeneity of AAI studies in preschool children is large, including different health conditions, duration, outcomes, study design or therapy animals. Therefore, the current study aims to summarize all intervention procedures and provide an updated analysis of the effectiveness of AAI intervention in the early childhood. Following the Preferred Reporting Items for Systematic Reviews and Meta-Analyses guidelines (PRISMA), a systematic search was conducted in two databases: Pubmed (MedLine) and Web of Science. The risk of bias was assessed using the Evidence Project risk of bias tool. A total of 319 articles were identified and 17 were finally included in the qualitative synthesis. Due to the large heterogeneity in terms of study design, intervention, and sample characteristics, it was not possible to conduct a meta-analysis. Animal-assisted interventions might lead to a positive impact on physical, physiological, psychosocial, and language skills in preschool children. These enhancements may be observed both in healthy children and in those with different health conditions, such as intellectual disabilities, cerebral palsy, autism disorder, or Down syndrome. Results must be interpreted with caution due to the large heterogeneity and risk of bias in the included articles. |
  | Date | 04/2023 |
  | Language | en |
  | Short Title | Benefits of animal-Assisted interventions in preschool children |
  | Library Catalogue | DOI.org (Crossref) |
  | URL | http://journals.sagepub.com/doi/10.1177/13591045221142115 |
  | Accessed | 05/02/2026, 17:24:34 |
  | Volume | 28 |
  | Pages | 850-873 |
  | Publication | Clinical Child Psychology and Psychiatry |
  | DOI | 10.1177/13591045221142115 |
  | Issue | 2 |
  | Journal Abbr | Clin Child Psychol Psychiatry |
  | ISSN | 1359-1045, 1461-7021 |
  | Date Added | 05/02/2026, 17:25:16 |
  | Modified | 05/02/2026, 17:25:16 |
- ## The role of animal-assisted programs in physical health improvement of children and adolescents with special education needs - a systematic review

  |  |  |
  | --- | --- |
  | Item Type | Journal Article |
  | Author | Karolina Eszter Kovács |
  | Author | Éva Zita Balogh |
  | Author | Buda Lovas |
  | Author | Péter Boris |
  | Author | Beáta Erika Nagy |
  | Abstract | Abstract Programs involving animals in therapeutic programs are becoming increasingly prevalent. These programs can vary greatly in their approach, scope, and objectives, and they can significantly impact the development of healthy children and those with various disorders. In this systematic review, we sought to investigate the psychological ramifications of animal-assisted activities (AAA), therapies (AAT), and interventions (AAI). We searched for relevant studies using the EBSCO Discovery Service search engine across 85 databases, utilising appropriate keywords. Our search generated 262 results, of which 21 were selected for inclusion after title and abstract screening, as well as full-text analysis. Our findings indicate that dogs and horses are animal-assisted programs’ most commonly used animals. Additionally, autism, cerebral palsy, and ADHD were found to be overrepresented in these programs. Furthermore, the length of sessions and overall program duration exhibited considerable variation, regardless of patient age or disease type. The principal measures centred on the physiological variables related to the nervous system and motorium-related indicators. The studies were generally of exceptional methodological soundness. Frequently, the studies narrowed their scope to a single segment or just the child or adolescent, but the outcomes lacked contextual interpretation. Expanding the range of studies by comparing psychological and physiological indicators and conducting follow-up analysis with a longitudinal design would be beneficial. |
  | Date | 2024-03-15 |
  | Language | en |
  | Library Catalogue | DOI.org (Crossref) |
  | URL | https://bmcpublichealth.biomedcentral.com/articles/10.1186/s12889-024-18326-y |
  | Accessed | 05/02/2026, 17:17:36 |
  | Volume | 24 |
  | Pages | 824 |
  | Publication | BMC Public Health |
  | DOI | 10.1186/s12889-024-18326-y |
  | Issue | 1 |
  | Journal Abbr | BMC Public Health |
  | ISSN | 1471-2458 |
  | Date Added | 05/02/2026, 17:17:48 |
  | Modified | 05/02/2026, 17:17:48 |

  ### Attachments

  - Available Version (via Google Scholar)
- ## Neurological mechanisms of animal-assisted intervention in alzheimer’s disease: A hypothetical review

  |  |  |
  | --- | --- |
  | Item Type | Journal Article |
  | Author | Sujin Kim |
  | Author | Yunkwon Nam |
  | Author | Min-Joo Ham |
  | Author | Chisoo Park |
  | Author | Minho Moon |
  | Author | Doo-Han Yoo |
  | Date | 2021 |
  | Short Title | Neurological mechanisms of animal-assisted intervention in alzheimer’s disease |
  | Library Catalogue | Google Scholar |
  | URL | https://www.frontiersin.org/journals/aging-neuroscience/articles/10.3389/fnagi.2021.682308/full |
  | Accessed | 05/02/2026, 17:24:50 |
  | Volume | 13 |
  | Publisher | Frontiers Media SA |
  | Pages | 682308 |
  | Publication | Frontiers in Aging Neuroscience |
  | Date Added | 05/02/2026, 17:25:16 |
  | Modified | 05/02/2026, 17:25:16 |

  ### Attachments

  - Available Version (via Google Scholar)
- ## Does animal-assisted intervention work? Research review on the effectiveness of AAI with the use of different animal species.

  |  |  |
  | --- | --- |
  | Item Type | Journal Article |
  | Author | Joanna Kapustka |
  | Author | Monika Budzyńska |
  | Date | 2020 |
  | Short Title | Does animal-assisted intervention work? |
  | Library Catalogue | Google Scholar |
  | URL | http://hvm.bioflux.com.ro/docs/2020.135-141.pdf |
  | Accessed | 05/02/2026, 17:17:14 |
  | Date Added | 05/02/2026, 17:17:48 |
  | Modified | 05/02/2026, 17:17:48 |

  ### Attachments

  - Available Version (via Google Scholar)
- ## Farm-animal-assisted Interventions: A Systematic Scoping Review, Realist Synthesis, and Realist Evaluation

  |  |  |
  | --- | --- |
  | Item Type | Journal Article |
  | Author | Suzanne Johnston |
  | Date | 2021 |
  | Short Title | Farm-animal-assisted Interventions |
  | Library Catalogue | Google Scholar |
  | URL | https://pureadmin.qub.ac.uk/ws/portalfiles/portal/258131763/SJThesis.pdf |
  | Accessed | 05/02/2026, 17:34:18 |
  | Publisher | Queen's University Belfast |
  | Date Added | 05/02/2026, 17:34:30 |
  | Modified | 05/02/2026, 17:34:30 |

  ### Attachments

  - Available Version (via Google Scholar)
- ## Animal-Assisted Interventions Improve Mental, But Not Cognitive or Physiological Health Outcomes of Higher Education Students: a Systematic Review and Meta-analysis

  |  |  |
  | --- | --- |
  | Item Type | Journal Article |
  | Author | Annalena Huber |
  | Author | Stefanie J. Klug |
  | Author | Annette Abraham |
  | Author | Erica Westenberg |
  | Author | Veronika Schmidt |
  | Author | Andrea S. Winkler |
  | Abstract | Abstract Due to the high burden of mental health issues among students at higher education institutions world-wide, animal-assisted interventions (AAIs) are being used to relieve student stress. The objective of this study was to systematically review of the effects of AAIs on the mental, physiological, and cognitive outcomes of higher education students. Randomized controlled trials using any unfamiliar animal as the sole intervention tool were included in this review. Study quality was assessed using the Cochrane Risk-of-Bias tool. Where possible, effect sizes (Hedges’ g ) were pooled for individual outcomes using random-effects meta-analyses. Albatross plots were used to supplement the data synthesis. Of 2.494 identified studies, 35 were included. Almost all studies used dogs as the intervention animal. The quality of most included studies was rated as moderate. Studies showed an overall reduction of acute anxiety and stress. For other mental outcomes, studies showed smaller, but nonetheless beneficial effects. Studies showed no clear effect on physiological or cognitive outcomes. Strong methodological heterogeneity between studies limited the ability to draw clear conclusions. |
  | Date | 06/2024 |
  | Language | en |
  | Short Title | Animal-Assisted Interventions Improve Mental, But Not Cognitive or Physiological Health Outcomes of Higher Education Students |
  | Library Catalogue | DOI.org (Crossref) |
  | URL | https://link.springer.com/10.1007/s11469-022-00945-4 |
  | Accessed | 05/02/2026, 17:13:35 |
  | Volume | 22 |
  | Pages | 1597-1628 |
  | Publication | International Journal of Mental Health and Addiction |
  | DOI | 10.1007/s11469-022-00945-4 |
  | Issue | 3 |
  | Journal Abbr | Int J Ment Health Addiction |
  | ISSN | 1557-1874, 1557-1882 |
  | Date Added | 05/02/2026, 17:13:35 |
  | Modified | 05/02/2026, 17:13:35 |

  ### Attachments

  - Available Version (via Google Scholar)
- ## Animals in higher education settings: Do animal-assisted interventions improve mental and cognitive health outcomes of students? A systematic review and meta-analysis

  |  |  |
  | --- | --- |
  | Item Type | Journal Article |
  | Author | Annalena Huber |
  | Author | Stefanie J. Klug |
  | Author | Annette Abraham |
  | Author | Erica Westenberg |
  | Author | Veronika Schmidt |
  | Author | Andrea S. Winkler |
  | Date | 2022 |
  | Short Title | Animals in higher education settings |
  | Library Catalogue | Google Scholar |
  | URL | https://www.medrxiv.org/content/10.1101/2022.04.11.22273607.abstract |
  | Accessed | 05/02/2026, 17:21:43 |
  | Publisher | Cold Spring Harbor Laboratory Press |
  | Pages | 2022–04 |
  | Publication | medRxiv |
  | Date Added | 05/02/2026, 17:22:09 |
  | Modified | 05/02/2026, 17:22:09 |

  ### Attachments

  - Available Version (via Google Scholar)
- ## Animal assisted activities in the children's hospital: protoco l for a scoping review [version 1; peer review: 1 approved

  |  |  |
  | --- | --- |
  | Item Type | Journal Article |
  | Author | Rachel Howe |
  | Author | Sandra Nicholson |
  | Author | Attracta Lafferty |
  | Author | Carmel Davies |
  | Author | Diarmuid Stokes |
  | Author | Thilo Kroll |
  | Short Title | Animal assisted activities in the children's hospital |
  | Library Catalogue | Google Scholar |
  | URL | https://www.academia.edu/download/78679627/39492b32-7f15-4bda-bdda-475589f3f2f0\_13143\_-\_rachel\_howe.pdf |
  | Accessed | 05/02/2026, 17:28:56 |
  | Date Added | 05/02/2026, 17:28:56 |
  | Modified | 05/02/2026, 17:28:56 |

  ### Attachments

  - Available Version (via Google Scholar)
- ## Animal assisted interventions in the children's hospital: protocol for a scoping review [version 2; peer review: 2

  |  |  |
  | --- | --- |
  | Item Type | Journal Article |
  | Author | Rachel Howe |
  | Author | Sandra Nicholson |
  | Author | Attracta Lafferty |
  | Author | Carmel Davies |
  | Author | Diarmuid Stokes |
  | Author | Thilo Kroll |
  | Date | 2021 |
  | Short Title | Animal assisted interventions in the children's hospital |
  | Library Catalogue | Google Scholar |
  | URL | https://www.academia.edu/download/108146841/pdf.pdf |
  | Accessed | 05/02/2026, 17:34:02 |
  | Date Added | 05/02/2026, 17:34:30 |
  | Modified | 05/02/2026, 17:34:30 |

  ### Attachments

  - Available Version (via Google Scholar)
- ## A Systematic Literature Review of Animal-Assisted Interventions in Oncology (Part I): Methods and Results

  |  |  |
  | --- | --- |
  | Item Type | Journal Article |
  | Author | Timothy R. N. Holder |
  | Author | Margaret E. Gruen |
  | Author | David L. Roberts |
  | Author | Tamara Somers |
  | Author | Alper Bozkurt |
  | Abstract | Animal-assisted interventions (AAIs) use human-animal interactions to positive effect in various contexts including cancer care. As the first installment of a 2-part series, this systematic literature review focuses on the research methods and quantitative results of AAI studies in oncology. We find methodological consistency in the use of canines as therapy animals, in the types of high-risk patients excluded from studies, and in the infection precautions taken with therapy animals throughout cancer wards. The investigated patient endpoints are not significantly affected by AAI, with the exceptions of improvements in oxygen consumption, quality of life, depression, mood, and satisfaction with therapy. The AAI field in oncology has progressed significantly since its inception and has great potential to positively affect future patient outcomes. To advance the field, future research should consistently improve the methodological design of studies, report data more completely, and focus more on the therapy animal’s well-being. |
  | Date | 01/2020 |
  | Language | en |
  | Short Title | A Systematic Literature Review of Animal-Assisted Interventions in Oncology (Part I) |
  | Library Catalogue | DOI.org (Crossref) |
  | URL | https://journals.sagepub.com/doi/10.1177/1534735420943278 |
  | Accessed | 05/02/2026, 17:13:29 |
  | Volume | 19 |
  | Pages | 1534735420943278 |
  | Publication | Integrative Cancer Therapies |
  | DOI | 10.1177/1534735420943278 |
  | Journal Abbr | Integr Cancer Ther |
  | ISSN | 1534-7354, 1552-695X |
  | Date Added | 05/02/2026, 17:13:35 |
  | Modified | 05/02/2026, 17:13:35 |

  ### Attachments

  - Available Version (via Google Scholar)
- ## Compilation of animal-assisted therapy studies: A narrative review of clinical evidence

  |  |  |
  | --- | --- |
  | Item Type | Journal Article |
  | Author | Vanessa Herrera |
  | Author | Luis Gutierrez-Rojas |
  | Author | Miguel Angel Alvarez-Mon |
  | Date | 2025 |
  | Short Title | Compilation of animal-assisted therapy studies |
  | Library Catalogue | Google Scholar |
  | URL | https://www.sciencedirect.com/science/article/pii/S1134593425000405 |
  | Accessed | 05/02/2026, 17:13:17 |
  | Volume | 32 |
  | Publisher | Elsevier |
  | Pages | 100738 |
  | Publication | Psiquiatría Biológica |
  | Issue | 3 |
  | Date Added | 05/02/2026, 17:13:35 |
  | Modified | 05/02/2026, 17:13:35 |
- ## Effectiveness of animal-assisted interventions for children and adults with post-traumatic stress disorder symptoms: a systematic review and meta-analysis

  |  |  |
  | --- | --- |
  | Item Type | Journal Article |
  | Author | Karin Hediger |
  | Author | Julia Wagner |
  | Author | Pascale Künzi |
  | Author | Anna Haefeli |
  | Author | Felicitas Theis |
  | Author | Carmina Grob |
  | Author | Elena Pauli |
  | Author | Heike Gerger |
  | Date | 01/2021 |
  | Language | en |
  | Short Title | Effectiveness of animal-assisted interventions for children and adults with post-traumatic stress disorder symptoms |
  | Library Catalogue | DOI.org (Crossref) |
  | URL | https://www.tandfonline.com/doi/full/10.1080/20008198.2021.1879713 |
  | Accessed | 05/02/2026, 17:13:27 |
  | Volume | 12 |
  | Pages | 1879713 |
  | Publication | European Journal of Psychotraumatology |
  | DOI | 10.1080/20008198.2021.1879713 |
  | Issue | 1 |
  | Journal Abbr | European Journal of Psychotraumatology |
  | ISSN | 2000-8066 |
  | Date Added | 05/02/2026, 17:13:35 |
  | Modified | 05/02/2026, 17:13:35 |

  ### Attachments

  - Available Version (via Google Scholar)
- ## The role of animal-assisted interventions (AAI) in healthcare waiting rooms: A scoping review on enhancing patients’ well-being and experience

  |  |  |
  | --- | --- |
  | Item Type | Journal Article |
  | Author | Irene Hartigan |
  | Author | Yvonne Pennisi |
  | Author | Claire Harman |
  | Author | Claire Keating |
  | Author | Kate Fitzgerald |
  | Author | My Linh Truong |
  | Abstract | Abstract This scoping review investigates the role of snimal-sssisted interventions (AAI) in enhancing patient well-being and experience in healthcare waiting rooms. From an initial pool of 1689 articles across CINAHL, PUBMED, and ASSIA, 8 studies met the inclusion criteria. The review evaluates the psychological and physical impacts of AAI, considerations for its implementation, and its potential to complement patient-centered care. Findings revealed that AAI consistently improved psychological outcomes, such as reducing anxiety and depression, though effects on physical parameters like pain and blood pressure remain mixed. While the term “therapy dog” may be misleading, these animals play a vital role in providing emotional support and enhancing patient well-being in healthcare settings. Future research should further clarify the distinct functions of therapy versus service dogs and explore the long-term and population-specific impacts of AAI in patient-centered care. |
  | Date | 2025-06-19 |
  | Language | en |
  | Short Title | The role of animal-assisted interventions (AAI) in healthcare waiting rooms |
  | Library Catalogue | DOI.org (Crossref) |
  | URL | http://www.cabidigitallibrary.org/doi/10.1079/hai.2025.0030 |
  | Accessed | 05/02/2026, 17:25:10 |
  | Pages | 0030 |
  | Publication | Human-Animal Interactions |
  | DOI | 10.1079/hai.2025.0030 |
  | Journal Abbr | Human-Animal Interactions |
  | ISSN | 2957-9538 |
  | Date Added | 05/02/2026, 17:25:16 |
  | Modified | 05/02/2026, 17:25:16 |
- ## Parents’ Perspectives on the Benefits of Animal-Assisted Intervention: A Systematic Review

  |  |  |
  | --- | --- |
  | Item Type | Journal Article |
  | Author | Francisco González-Sala |
  | Author | Karel Llopiz-Guerra |
  | Author | Ainhoa Ferri |
  | Author | Manuel Martí-Vilar |
  | Date | 2025 |
  | Short Title | Parents’ Perspectives on the Benefits of Animal-Assisted Intervention |
  | Library Catalogue | Google Scholar |
  | URL | https://pmc.ncbi.nlm.nih.gov/articles/PMC12729349/ |
  | Accessed | 05/02/2026, 17:28:52 |
  | Volume | 15 |
  | Pages | 1663 |
  | Publication | Behavioral Sciences |
  | Issue | 12 |
  | Date Added | 05/02/2026, 17:28:56 |
  | Modified | 05/02/2026, 17:28:56 |
- ## ANIMAL ASSISTED INTERVENTIONS: REVISION AND EVALUATION OF LATIN AMERICAN STUDIES

  |  |  |
  | --- | --- |
  | Item Type | Journal Article |
  | Author | Crystian Moraes Silva GOMES |
  | Author | Amanda Doring SEMEDO |
  | Author | Maria Eduarda Teixeira CAETANO |
  | Author | Rosana Suemi TOKUMARU |
  | Date | 2023 |
  | Short Title | ANIMAL ASSISTED INTERVENTIONS |
  | Library Catalogue | Google Scholar |
  | URL | http://educa.fcc.org.br/scielo.php?pid=S1413-65382023000100410&script=sci\_abstract&tlng=en |
  | Accessed | 05/02/2026, 17:34:24 |
  | Volume | 29 |
  | Publication | Revista Brasileira de Educação Especial |
  | Date Added | 05/02/2026, 17:34:30 |
  | Modified | 05/02/2026, 17:34:30 |

  ### Attachments

  - Available Version (via Google Scholar)
- ## Animal assisted therapy for older adults in aged care facilities: A rapid review

  |  |  |
  | --- | --- |
  | Item Type | Journal Article |
  | Author | Mitchell A. Franklin |
  | Author | Tracey Parnell |
  | Author | Natasha Versi |
  | Author | Rodney Pope |
  | Date | 2022 |
  | Short Title | Animal assisted therapy for older adults in aged care facilities |
  | Library Catalogue | Google Scholar |
  | URL | https://nsuworks.nova.edu/ijahsp/vol20/iss1/14/ |
  | Accessed | 05/02/2026, 17:28:41 |
  | Volume | 20 |
  | Pages | 14 |
  | Publication | Internet Journal of Allied Health Sciences and Practice |
  | Issue | 1 |
  | Date Added | 05/02/2026, 17:28:56 |
  | Modified | 05/02/2026, 17:28:56 |

  ### Attachments

  - Available Version (via Google Scholar)
- ## The supporting role of dogs in the inpatient setting: a systematic review of the therapeutic effects of animal-assisted therapy with dogs for children and adolescents in an inpatient setting

  |  |  |
  | --- | --- |
  | Item Type | Journal Article |
  | Author | Dustin Fornefeld |
  | Author | Undine Zellin |
  | Author | Peter Schmidt |
  | Author | Oliver Fricke |
  | Abstract | Abstract Animal-assisted therapy (AAT) is becoming increasingly popular. The possibilities and guidelines for interventions and methods are very diverse. Currently, published studies mainly concentrate on effects in paediatrics, outpatient therapy and schools. Specific recommendations for AAT in the context of inpatient child and adolescent psychiatry do not exist. This systematic review will attempt to evaluate the existing studies in terms of their methodological quality and specify positive and negative effects, aiming to provide a decision-making aid for everyday clinical practice. A systematic literature search (PubMed/MEDLINE, APA PsycINFO, PubPsych, ProQuest, Google Scholar, and Cochrane Library) according to the PRISMA criteria resulted in 1,908 identified hits, of which 49 articles were reviewed in full text. Three raters contributed to the review of the articles using a criteria-guided codebook. This systematic review is listed in the PROSPERO database (CRD42022358909). Quality analysis was conducted using Effective Public Health Practice Project (EPHPP). Five studies were identified. The majority of these showed deficits in quality. Therapeutic effects and positive influences on the psychopathological status, interpersonal relationships and subjective well-being or attitudes towards canine-assisted therapy (CAT) could be identified. Current studies indicate positive therapeutic effects of CAT in the inpatient treatment of children and adolescents. A cautiously positive perspective is warranted, but a general recommendation for CAT cannot be given. CAT should be carefully considered, planned, and implemented by professionals. For the future, further randomised controlled studies including follow-up studies, larger subject groups and clinically evaluated interventions are necessary to validate the current results. |
  | Date | 01/2025 |
  | Language | en |
  | Short Title | The supporting role of dogs in the inpatient setting |
  | Library Catalogue | DOI.org (Crossref) |
  | URL | https://link.springer.com/10.1007/s00787-023-02326-1 |
  | Accessed | 05/02/2026, 17:28:17 |
  | Volume | 34 |
  | Pages | 3-17 |
  | Publication | European Child & Adolescent Psychiatry |
  | DOI | 10.1007/s00787-023-02326-1 |
  | Issue | 1 |
  | Journal Abbr | Eur Child Adolesc Psychiatry |
  | ISSN | 1018-8827, 1435-165X |
  | Date Added | 05/02/2026, 17:28:56 |
  | Modified | 05/02/2026, 17:28:56 |

  ### Attachments

  - Available Version (via Google Scholar)
- ## The effects of animal-assisted therapy on the health and well-being of military veterans: A systematic scoping review and recommendations for future research

  |  |  |
  | --- | --- |
  | Item Type | Journal Article |
  | Author | Brooke Fonseka |
  | Author | Fiona Marshall |
  | Author | Laura J. Edwards |
  | Date | 2022 |
  | Short Title | The effects of animal-assisted therapy on the health and well-being of military veterans |
  | Library Catalogue | Google Scholar |
  | URL | https://docs.lib.purdue.edu/paij/vol5/iss1/12/ |
  | Accessed | 05/02/2026, 17:17:27 |
  | Volume | 5 |
  | Pages | 12 |
  | Publication | People and Animals: The International Journal of Research and Practice |
  | Issue | 1 |
  | Date Added | 05/02/2026, 17:17:48 |
  | Modified | 05/02/2026, 17:17:48 |

  ### Attachments

  - Available Version (via Google Scholar)
- ## Risks and benefits of animal-assisted interventions for critically ill patients admitted to intensive care units

  |  |  |
  | --- | --- |
  | Item Type | Journal Article |
  | Author | Marco Fiore |
  | Author | Andrea Cortegiani |
  | Author | Giansaverio Friolo |
  | Author | Francesca Frigieri Covani |
  | Author | Luigi Cardia |
  | Author | Fausto Ferraro |
  | Author | Daniela Alampi |
  | Abstract | Abstract Background Pets offer significant health benefits, from decreased cardiovascular risks to anxiety and post-traumatic stress improvements. Animal-assisted interventions (AAI) are not frequently practiced in the intensive care unit (ICU) for fear of health risk for critical patients because there is a hypothetical risk of zoonoses. Objectives This systematic review aimed to collect and summarize available evidence about AAI in the ICU. The Review questions were “Do AAI improve the clinical outcome of Critically Ill Patients admitted to ICUs?” and “Are the zoonotic infections the cause of negative prognosis?”. Methods The following databases were searched on 5 January 2023: Cochrane Central Register of Controlled Trials (CENTRAL), EMBASE, and PubMed. All controlled studies (randomized controlled, quasi-experimental, and observational studies) were included. The systematic review protocol has been registered on the International Prospective Register of Systematic Review (CRD42022344539). Results A total of 1302 papers were retrieved, 1262 after the duplicate remotion. Of these, only 34 were assessed for eligibility and only 6 were included in the qualitative synthesis. In all the studies included the dog was the animal used for the AAI with a total of 118 cases and 128 controls. Studies have high variability, and no one has used increased survival or zoonotic risk as outcomes. Conclusions The evidence on the effectiveness of AAIs in ICU settings is scarce and no data are available on their safety. AAIs use in the ICU must be considered experimental and follow the related regulation until further data will be available. Given the potential positive impact on patient-centered outcomes, a research effort for high-quality studies seems to be justified. |
  | Date | 2023-05-31 |
  | Language | en |
  | Library Catalogue | DOI.org (Crossref) |
  | URL | https://janesthanalgcritcare.biomedcentral.com/articles/10.1186/s44158-023-00100-y |
  | Accessed | 05/02/2026, 17:13:21 |
  | Volume | 3 |
  | Pages | 15 |
  | Publication | Journal of Anesthesia, Analgesia and Critical Care |
  | DOI | 10.1186/s44158-023-00100-y |
  | Issue | 1 |
  | Journal Abbr | J Anesth Analg Crit Care |
  | ISSN | 2731-3786 |
  | Date Added | 05/02/2026, 17:13:35 |
  | Modified | 05/02/2026, 17:13:35 |

  ### Attachments

  - Available Version (via Google Scholar)
- ## Animal-assisted therapy in patients affected by schizophrenia and schizophrenic-related disorders: A scoping review

  |  |  |
  | --- | --- |
  | Item Type | Journal Article |
  | Author | Martina Finistrella |
  | Author | Paolo Flores |
  | Author | Gianpaolo Frediani |
  | Date | 2024 |
  | Short Title | Animal-assisted therapy in patients affected by schizophrenia and schizophrenic-related disorders |
  | Library Catalogue | Google Scholar |
  | URL | https://hal.science/hal-04567974/ |
  | Accessed | 05/02/2026, 17:21:55 |
  | Volume | 1 |
  | Pages | 53–61 |
  | Publication | Advances in Medicine, Psychology, and Public Health |
  | Issue | 2 |
  | Date Added | 05/02/2026, 17:22:09 |
  | Modified | 05/02/2026, 17:22:09 |

  ### Attachments

  - Available Version (via Google Scholar)
- ## Protecting Animal Welfare in Animal-Assisted Intervention: Our Ethical Obligation

  |  |  |
  | --- | --- |
  | Item Type | Journal Article |
  | Author | Aubrey H. Fine |
  | Author | Taylor Chastain Griffin |
  | Abstract | Abstract When incorporating therapy animals into clinical practice, there are essential ethical considerations that must be considered to protect the welfare of both the people and the animals who are involved in the intervention. The field of animal-assisted interventions (AAIs) and more specifically animal-assisted therapy (AAT) is just beginning to appreciate the critical role that animal welfare has in enhancing the quality of the entire process of working with a therapy animal. In this article, the authors will present ethical models that are incumbent for practitioners to consider prior to partnering with a therapy animal. Examples of how a speech-language pathologist (SLP) might work with a therapy animal will be integrated throughout the article to demonstrate applied awareness of how good welfare not only protects the animal but also the clients who engage in the intervention. Key aspects to consider at all stages of AAT will be described, including considerations of welfare as they relate to selecting and working with a therapy animal, preparing clients for AAT, and developing specific competencies as an AAT practitioner. Theoretical support for these recommendations will also be outlined, preparing AAT providers to not only incorporate the highest standards in AAT but to also serve as an advocate in championing these standards as the field develops. |
  | Date | 01/2022 |
  | Language | en |
  | Short Title | Protecting Animal Welfare in Animal-Assisted Intervention |
  | Library Catalogue | DOI.org (Crossref) |
  | URL | http://www.thieme-connect.de/DOI/DOI?10.1055/s-0041-1742099 |
  | Accessed | 05/02/2026, 17:24:42 |
  | Volume | 43 |
  | Pages | 008-023 |
  | Publication | Seminars in Speech and Language |
  | DOI | 10.1055/s-0041-1742099 |
  | Issue | 01 |
  | Journal Abbr | Semin Speech Lang |
  | ISSN | 0734-0478, 1098-9056 |
  | Date Added | 05/02/2026, 17:25:16 |
  | Modified | 05/02/2026, 17:25:16 |

  ### Attachments

  - Available Version (via Google Scholar)
- ## A commentary on the contemporary issues confronting animal assisted and equine assisted interactions

  |  |  |
  | --- | --- |
  | Item Type | Journal Article |
  | Author | Aubrey H. Fine |
  | Author | Sarah J. Andersen |
  | Date | 2021 |
  | Library Catalogue | Google Scholar |
  | URL | https://www.sciencedirect.com/science/article/pii/S0737080621000666 |
  | Accessed | 05/02/2026, 17:13:27 |
  | Volume | 100 |
  | Publisher | Elsevier |
  | Pages | 103436 |
  | Publication | Journal of Equine Veterinary Science |
  | Date Added | 05/02/2026, 17:13:35 |
  | Modified | 05/02/2026, 17:13:35 |
- ## Effects of animal-assisted therapy on hospitalized children and teenagers: A systematic review and meta-analysis

  |  |  |
  | --- | --- |
  | Item Type | Journal Article |
  | Author | Yongshen Feng |
  | Author | Yeqing Lin |
  | Author | Ningning Zhang |
  | Author | Xiaohan Jiang |
  | Author | Lifeng Zhang |
  | Date | 2021 |
  | Short Title | Effects of animal-assisted therapy on hospitalized children and teenagers |
  | Library Catalogue | Google Scholar |
  | URL | https://www.sciencedirect.com/science/article/pii/S0882596321000233 |
  | Accessed | 05/02/2026, 17:13:15 |
  | Volume | 60 |
  | Publisher | Elsevier |
  | Pages | 11–23 |
  | Publication | Journal of Pediatric Nursing |
  | Date Added | 05/02/2026, 17:13:35 |
  | Modified | 05/02/2026, 17:13:35 |

  ### Attachments

  - Available Version (via Google Scholar)
- ## A bibliometric study for global hotspots and trends in animal-assisted interventions (1983–2023)

  |  |  |
  | --- | --- |
  | Item Type | Journal Article |
  | Author | Xiaowei Feng |
  | Author | Shanguang Zhao |
  | Author | Dong Zhang |
  | Author | Qing Yi |
  | Author | Yanlan Chen |
  | Author | Xinding Zhang |
  | Date | 2025 |
  | Library Catalogue | Google Scholar |
  | URL | https://www.frontiersin.org/journals/psychiatry/articles/10.3389/fpsyt.2025.1490122/full |
  | Accessed | 05/02/2026, 17:17:20 |
  | Volume | 16 |
  | Publisher | Frontiers Media SA |
  | Pages | 1490122 |
  | Publication | Frontiers in Psychiatry |
  | Date Added | 05/02/2026, 17:17:48 |
  | Modified | 05/02/2026, 17:17:48 |

  ### Attachments

  - Available Version (via Google Scholar)
- ## PENGARUH ANIMAL ASSISTED THERAPY TERHADAP TINGKAT STRES: LITERATURE REVIEW

  |  |  |
  | --- | --- |
  | Item Type | Journal Article |
  | Author | INDAH FEBRIANTI |
  | Short Title | PENGARUH ANIMAL ASSISTED THERAPY TERHADAP TINGKAT STRES |
  | Library Catalogue | Google Scholar |
  | URL | http://repository.stikesmadani.ac.id/lampiran/1649120303-pengaruh-animal-assisted-therapi-terhadap-tingkat-stres-literature-review.pdf |
  | Accessed | 05/02/2026, 17:34:29 |
  | Date Added | 05/02/2026, 17:34:30 |
  | Modified | 05/02/2026, 17:34:30 |

  ### Attachments

  - Available Version (via Google Scholar)
- ## PIH21 A Targeted Review of Studies on Canine Animal Assisted Therapy in Paediatric Oncology Patients

  |  |  |
  | --- | --- |
  | Item Type | Journal Article |
  | Author | J. Farrington |
  | Author | C. W. Tallentire |
  | Author | A. Lenny |
  | Author | J. Singh |
  | Author | L. Longworth |
  | Date | 2020 |
  | Library Catalogue | Google Scholar |
  | URL | https://www.valueinhealthjournal.com/article/S1098-3015(20)33076-X/fulltext |
  | Accessed | 05/02/2026, 17:22:07 |
  | Volume | 23 |
  | Publisher | Elsevier |
  | Pages | S542 |
  | Publication | Value in Health |
  | Date Added | 05/02/2026, 17:22:09 |
  | Modified | 05/02/2026, 17:22:09 |
- ## Animal-Assisted Therapy for the Management of Anxiety in the Hospital Setting: A Systematic Review

  |  |  |
  | --- | --- |
  | Item Type | Journal Article |
  | Author | Leticia Antolín Esteve |
  | Author | Patricia López-Mases |
  | Author | Leticia E. Bartolomé Del Pino |
  | Author | Esther Lázaro |
  | Short Title | Animal-Assisted Therapy for the Management of Anxiety in the Hospital Setting |
  | Library Catalogue | Google Scholar |
  | URL | https://journals.lww.com/hnpjournal/fulltext/9900/animal\_assisted\_therapy\_for\_the\_management\_of.89.aspx |
  | Accessed | 05/02/2026, 17:22:05 |
  | Publisher | LWW |
  | Pages | 10–1097 |
  | Publication | Holistic Nursing Practice |
  | Date Added | 05/02/2026, 17:22:09 |
  | Modified | 05/02/2026, 17:22:09 |
- ## The neural and physiological mechanisms of animal assisted interventions (AAI)

  |  |  |
  | --- | --- |
  | Item Type | Journal Article |
  | Author | Neta Ehrlich |
  | Author | Leehe Peled-Avron |
  | Date | 2025 |
  | Library Catalogue | Google Scholar |
  | URL | https://osf.io/preprints/psyarxiv/dz4fy |
  | Accessed | 05/02/2026, 17:13:02 |
  | Publisher | OSF |
  | Date Added | 05/02/2026, 17:13:35 |
  | Modified | 05/02/2026, 17:13:35 |
- ## ANIMAL-ASSISTED THERAPY: A SUBJECT FIELD REVIEW

  |  |  |
  | --- | --- |
  | Item Type | Journal Article |
  | Author | Z. Dyachenko |
  | Date | 2022 |
  | Short Title | ANIMAL-ASSISTED THERAPY |
  | Library Catalogue | Google Scholar |
  | URL | https://elibrary.ru/item.asp?id=50318335 |
  | Accessed | 05/02/2026, 17:28:37 |
  | Date Added | 05/02/2026, 17:28:56 |
  | Modified | 05/02/2026, 17:28:56 |
- ## Animal assisted therapy in pediatric mental health conditions: A review

  |  |  |
  | --- | --- |
  | Item Type | Journal Article |
  | Author | Tam Doan |
  | Author | Deanna Pennewitt |
  | Author | Rohan Patel |
  | Date | 2023 |
  | Short Title | Animal assisted therapy in pediatric mental health conditions |
  | Library Catalogue | Google Scholar |
  | URL | https://www.sciencedirect.com/science/article/pii/S1538544223001554 |
  | Accessed | 05/02/2026, 17:24:43 |
  | Volume | 53 |
  | Publisher | Elsevier |
  | Pages | 101506 |
  | Publication | Current Problems in Pediatric and Adolescent Health Care |
  | Issue | 12 |
  | Date Added | 05/02/2026, 17:25:16 |
  | Modified | 05/02/2026, 17:25:16 |
- ## Assessment of changes in the saliva cortisol level of horses during different ways in recreational exploitation

  |  |  |
  | --- | --- |
  | Item Type | Journal Article |
  | Author | Bogusława Długosz |
  | Author | Tomasz Próchniak |
  | Author | Monika Stefaniuk-Szmukier |
  | Author | Marta Basiaga |
  | Author | Jarosław Łuszczyńśki |
  | Author | Magdalena Pieszka |
  | Date | 2020-08-18 |
  | Library Catalogue | DOI.org (Crossref) |
  | URL | https://journals.tubitak.gov.tr/veterinary/vol44/iss4/1 |
  | Accessed | 05/02/2026, 18:01:54 |
  | Volume | 44 |
  | Pages | 757-762 |
  | Publication | TURKISH JOURNAL OF VETERINARY AND ANIMAL SCIENCES |
  | DOI | 10.3906/vet-2003-57 |
  | Issue | 4 |
  | Journal Abbr | Turk J Vet Anim Sci |
  | ISSN | 13036181 |
  | Date Added | 05/02/2026, 18:01:54 |
  | Modified | 05/02/2026, 18:01:54 |
- ## Systematic Review The use of animal-assisted practices as a nursing intervention: A systematic review

  |  |  |
  | --- | --- |
  | Item Type | Journal Article |
  | Author | Şeyma Demiralay |
  | Author | İlkay Keser |
  | Author | Sibel Çaynak |
  | Date | 2020 |
  | Short Title | Systematic Review The use of animal-assisted practices as a nursing intervention |
  | Library Catalogue | Google Scholar |
  | URL | https://jag.journalagent.com/z4/download\_fulltext.asp?pdir=phd&ppdf=2&plng=eng&un=PHD-82474 |
  | Accessed | 05/02/2026, 17:28:54 |
  | Date Added | 05/02/2026, 17:28:56 |
  | Modified | 05/02/2026, 17:28:56 |

  ### Attachments

  - Available Version (via Google Scholar)
- ## The use of animal-assisted practices as a nursing intervention: A systematic review

  |  |  |
  | --- | --- |
  | Item Type | Journal Article |
  | Author | Seyma Demiralay |
  | Author | İlkay Keser |
  | Author | Sibel Çaynak |
  | Date | 2020 |
  | Short Title | The use of animal-assisted practices as a nursing intervention |
  | Library Catalogue | Google Scholar |
  | URL | https://avesis.akdeniz.edu.tr/yayin/94fc2ddc-4586-4a49-bf92-148e9c25ae4b/the-use-of-animal-assisted-practices-as-a-nursing-intervention-a-systematic-review |
  | Accessed | 05/02/2026, 17:34:06 |
  | Volume | 11 |
  | Publication | Journal of Psychiatric Nursing |
  | Issue | 3 |
  | Date Added | 05/02/2026, 17:34:30 |
  | Modified | 05/02/2026, 17:34:30 |

  ### Attachments

  - Available Version (via Google Scholar)
- ## Developing a research agenda on NATure-based and Animal-assisted Intervention Strategies (NATAIS) in people with neurodegenerative diseases with a specific focus on social isolation and loneliness: a group concept mapping procedure

  |  |  |
  | --- | --- |
  | Item Type | Journal Article |
  | Author | I. J. N. Declercq |
  | Author | R. Leontjevas |
  | Author | M.-J. Enders-Slegers |
  | Author | M. Molog |
  | Author | D. L. Gerritsen |
  | Author | K. Hediger |
  | Abstract | Abstract Background Social isolation and feelings of loneliness are very prevalent in people with neurodegenerative diseases and are associated with a lower quality of life and other negative outcomes. These problems were increased during the COVID-19 pandemic resulting in initiatives to address social isolation. Given the potential benefits of nature-based and animal-assisted intervention strategies (NATAIS), it is crucial to further investigate if and how these strategies might minimize negative effects of social isolation and feelings of loneliness in this population. Therefore, the aim of this project was to develop a research agenda for NATAIS in people with neurodegenerative diseases, especially during challenging times, such as pandemics. Methods This article outlines the process and results of a group concept mapping procedure aimed at developing a research agenda based on a logic model. In total, 19 work group members participated through a combination of in-person and online group meetings. Additionally, face-to-face group sessions were held at two international scientific conferences, during which feedback was solicited from 12 experts in the field of NATAIS and psychogeriatrics. Results The group concept mapping procedure resulted in 14 clusters describing various future research topics, which were further refined and detailed during group discussions. The remaining eleven clusters, encompassing important research themes within the field of NATAIS, were organized into a logic model and summarized into the research agenda. The overarching cluster ‘ethical issues, possible risk factors, and their solutions’ was considered the most relevant during times of increased social isolation, such as during a pandemic, along with the necessity for more accessible NATAIS. Conclusions This project resulted in a research agenda, directing future research and fostering collaboration between practitioners and researchers in the field of NATAIS. Such an enhanced partnership between science and practice has the potential to significantly contribute to the well-being of people with neurodegenerative diseases, in their daily lives and also during pandemics. |
  | Date | 2024-09-28 |
  | Language | en |
  | Short Title | Developing a research agenda on NATure-based and Animal-assisted Intervention Strategies (NATAIS) in people with neurodegenerative diseases with a specific focus on social isolation and loneliness |
  | Library Catalogue | DOI.org (Crossref) |
  | URL | https://bmcgeriatr.biomedcentral.com/articles/10.1186/s12877-024-05387-2 |
  | Accessed | 05/02/2026, 17:25:01 |
  | Volume | 24 |
  | Pages | 795 |
  | Publication | BMC Geriatrics |
  | DOI | 10.1186/s12877-024-05387-2 |
  | Issue | 1 |
  | Journal Abbr | BMC Geriatr |
  | ISSN | 1471-2318 |
  | Date Added | 05/02/2026, 17:25:16 |
  | Modified | 05/02/2026, 17:25:16 |

  ### Attachments

  - Available Version (via Google Scholar)
- ## Race, zoonoses and animal assisted interventions in pediatric cancer

  |  |  |
  | --- | --- |
  | Item Type | Journal Article |
  | Author | Crina Cotoc |
  | Author | Stephen Notaro |
  | Date | 2022 |
  | Library Catalogue | Google Scholar |
  | URL | https://www.mdpi.com/1660-4601/19/13/7772 |
  | Accessed | 05/02/2026, 17:17:48 |
  | Volume | 19 |
  | Publisher | MDPI |
  | Pages | 7772 |
  | Publication | International Journal of Environmental Research and Public Health |
  | Issue | 13 |
  | Date Added | 05/02/2026, 17:17:48 |
  | Modified | 05/02/2026, 17:17:48 |
- ## Improving the emotional distress and the experience of hospitalization in children and adolescent patients through animal assisted interventions: a systematic review

  |  |  |
  | --- | --- |
  | Item Type | Journal Article |
  | Author | Cinzia Correale |
  | Author | Marta Borgi |
  | Author | Barbara Collacchi |
  | Author | Chiara Falamesca |
  | Author | Simonetta Gentile |
  | Author | Federico Vigevano |
  | Author | Simona Cappelletti |
  | Author | Francesca Cirulli |
  | Date | 2022 |
  | Short Title | Improving the emotional distress and the experience of hospitalization in children and adolescent patients through animal assisted interventions |
  | Library Catalogue | Google Scholar |
  | URL | https://www.frontiersin.org/articles/10.3389/fpsyg.2022.840107/full |
  | Accessed | 05/02/2026, 17:17:12 |
  | Volume | 13 |
  | Publisher | Frontiers Media SA |
  | Pages | 840107 |
  | Publication | Frontiers in psychology |
  | Date Added | 05/02/2026, 17:17:48 |
  | Modified | 05/02/2026, 17:17:48 |

  ### Attachments

  - Available Version (via Google Scholar)
- ## Animal-assisted interventions in universities: A scoping review of implementation and associated outcomes

  |  |  |
  | --- | --- |
  | Item Type | Journal Article |
  | Author | Emily Cooke |
  | Author | Claire Henderson-Wilson |
  | Author | Elyse Warner |
  | Author | Anthony LaMontagne |
  | Date | 2023 |
  | Short Title | Animal-assisted interventions in universities |
  | Library Catalogue | Google Scholar |
  | URL | https://academic.oup.com/heapro/article-abstract/38/3/daac001/6517178 |
  | Accessed | 05/02/2026, 17:17:39 |
  | Volume | 38 |
  | Publisher | Oxford University Press |
  | Pages | daac001 |
  | Publication | Health Promotion International |
  | Issue | 3 |
  | Date Added | 05/02/2026, 17:17:48 |
  | Modified | 05/02/2026, 17:17:48 |

  ### Attachments

  - Available Version (via Google Scholar)
- ## Animal-assisted interventions and post-traumatic stress disorder of military workers and veterans: a systematic review

  |  |  |
  | --- | --- |
  | Item Type | Journal Article |
  | Author | Francesco Chirico |
  | Author | Ilaria Capitanelli |
  | Author | Behdin Nowrouzi-Kia |
  | Author | Aaron Howe |
  | Author | Kavita Batra |
  | Author | Manoj Sharma |
  | Author | Lukasz Szarpak |
  | Author | Michal Pruc |
  | Author | Gabriella Nucera |
  | Author | Giuseppe Ferrari |
  | Date | 2022 |
  | Short Title | Animal-assisted interventions and post-traumatic stress disorder of military workers and veterans |
  | Library Catalogue | Google Scholar |
  | URL | https://iris.unito.it/handle/2318/1888503 |
  | Accessed | 05/02/2026, 17:17:21 |
  | Volume | 7 |
  | Pages | 152–180 |
  | Publication | Journal of Health and Social Sciences |
  | Issue | 2 |
  | Date Added | 05/02/2026, 17:17:48 |
  | Modified | 05/02/2026, 17:17:48 |

  ### Attachments

  - Available Version (via Google Scholar)
- ## Effects of animal-assisted therapy on patients with dementia: A systematic review and meta-analysis of randomized controlled trials

  |  |  |
  | --- | --- |
  | Item Type | Journal Article |
  | Author | Hongyu Chen |
  | Author | Yuanyuan Wang |
  | Author | Minyi Zhang |
  | Author | Ning Wang |
  | Author | Yao Li |
  | Author | Yan Liu |
  | Date | 2022 |
  | Short Title | Effects of animal-assisted therapy on patients with dementia |
  | Library Catalogue | Google Scholar |
  | URL | https://www.sciencedirect.com/science/article/pii/S0165178122002207 |
  | Accessed | 05/02/2026, 17:17:45 |
  | Volume | 314 |
  | Publisher | Elsevier |
  | Pages | 114619 |
  | Publication | Psychiatry research |
  | Date Added | 05/02/2026, 17:17:48 |
  | Modified | 05/02/2026, 17:17:48 |
- ## Animal‐Assisted Therapy as an Intervention for Older Adults: A Systematic Review and Meta‐Analysis to Guide Evidence‐Based Practice

  |  |  |
  | --- | --- |
  | Item Type | Journal Article |
  | Author | Sun Ju Chang |
  | Author | Jongeun Lee |
  | Author | Hyeran An |
  | Author | Woi‐Hyun Hong |
  | Author | Joo Yun Lee |
  | Abstract | Abstract Background Animal‐assisted therapy (AAT) can ameliorate diverse health problems in older adults. However, applications of AAT have been limited because of the lack of intervention guidelines for older adults. Aims This study aimed to explore applications of AAT to older adults, analyze its health effects, and provide evidence for future interventions. Methods A systematic review and meta‐analysis were conducted based on the Preferred Reporting Items for Systematic Reviews and Meta‐Analyses checklist. Data were analyzed based on both a narrative synthesis and a meta‐analysis specifically for depression. Results A total of 47 studies were selected for analysis. About 45% focused on older adults with diseases such as dementia, and 57.4% selected dog(s) as an intervention animal. About 34.0% delivered interventions once a week, and the behavioral outcome domain was the most frequently investigated. The meta‐analysis showed that the effect sizes of the AAT group were −1.310 (95% CI [−1.900, −.721]). Linking Evidence to Action This review provides evidence for AAT as an intervention in the physiological, psychosocial, cognitive, and behavioral domains of older adults. When planning interventions for older adults, nurses should consider intended health outcomes, appropriate therapeutic animals, and the consequent intervention contents. |
  | Date | 02/2021 |
  | Language | en |
  | Short Title | Animal‐Assisted Therapy as an Intervention for Older Adults |
  | Library Catalogue | DOI.org (Crossref) |
  | URL | https://sigmapubs.onlinelibrary.wiley.com/doi/10.1111/wvn.12484 |
  | Accessed | 05/02/2026, 17:21:48 |
  | Volume | 18 |
  | Pages | 60-67 |
  | Publication | Worldviews on Evidence-Based Nursing |
  | DOI | 10.1111/wvn.12484 |
  | Issue | 1 |
  | Journal Abbr | Worldviews Ev Based Nurs |
  | ISSN | 1545-102X, 1741-6787 |
  | Date Added | 05/02/2026, 17:22:09 |
  | Modified | 05/02/2026, 17:22:09 |

  ### Attachments

  - Available Version (via Google Scholar)
- ## A review of studies conducted with animal assisted interventions for children with autism spectrum disorder

  |  |  |
  | --- | --- |
  | Item Type | Journal Article |
  | Author | Damla Çetin |
  | Author | Selmin Çuhadar |
  | Date | 2021 |
  | Library Catalogue | Google Scholar |
  | URL | http://www.cappsy.org/archives/vol13/no3/cap\_13\_03\_15\_en.pdf |
  | Accessed | 05/02/2026, 17:21:37 |
  | Volume | 13 |
  | Publisher | Psikiyatride Guncel Yaklasimlar: Current Approaches in Psychiatry |
  | Pages | 619–639 |
  | Publication | Psikiyatride Guncel Yaklasimlar |
  | Issue | 3 |
  | Date Added | 05/02/2026, 17:22:09 |
  | Modified | 05/02/2026, 17:22:09 |

  ### Attachments

  - Available Version (via Google Scholar)
- ## Stress assessment of co-therapist dogs in animal assisted interventions: a review

  |  |  |
  | --- | --- |
  | Item Type | Journal Article |
  | Author | Laura Ceglia |
  | Date | 2021 |
  | Short Title | Stress assessment of co-therapist dogs in animal assisted interventions |
  | Library Catalogue | Google Scholar |
  | URL | https://dogbehavior.it/dogbehavior/article/view/140 |
  | Accessed | 05/02/2026, 17:25:10 |
  | Volume | 7 |
  | Pages | 39–54 |
  | Publication | Dog behavior |
  | Issue | 2 |
  | Date Added | 05/02/2026, 17:25:16 |
  | Modified | 05/02/2026, 17:25:16 |

  ### Attachments

  - Available Version (via Google Scholar)
- ## Can Animal-assisted Therapy Aid Recovery from Alcohol and Drug Addiction? A Brief Review of the Literature

  |  |  |
  | --- | --- |
  | Item Type | Journal Article |
  | Author | Kirstie Broadfield |
  | Date | 2020 |
  | Short Title | Can Animal-assisted Therapy Aid Recovery from Alcohol and Drug Addiction? |
  | Library Catalogue | Google Scholar |
  | URL | https://www.researchgate.net/profile/Kirstie-Broadfield/publication/344678036\_Can\_Animal-assisted\_Therapy\_Aid\_Recovery\_from\_Alcohol\_and\_Drug\_Addiction\_A\_Brief\_Review\_of\_the\_Literature/links/5f88f19192851c14bccc18ad/Can-Animal-assisted-Therapy-Aid-Recovery-from-Alcohol-and-Drug-Addiction-A-Brief-Review-of-the-Literature.pdf |
  | Accessed | 05/02/2026, 17:28:43 |
  | Volume | 2 |
  | Publication | Research Gate |
  | Date Added | 05/02/2026, 17:28:56 |
  | Modified | 05/02/2026, 17:28:56 |

  ### Attachments

  - Available Version (via Google Scholar)
- ## Effects of Animal-Assisted Therapy for Anxiety Reduction in Children and Adolescents: A Systematic Review

  |  |  |
  | --- | --- |
  | Item Type | Journal Article |
  | Author | Constança Brandão |
  | Author | Maria Sampaio |
  | Author | Valéria Sousa-Gomes |
  | Author | Marisalva Fávero |
  | Author | Diana Moreira |
  | Date | 2025 |
  | Short Title | Effects of Animal-Assisted Therapy for Anxiety Reduction in Children and Adolescents |
  | Library Catalogue | Google Scholar |
  | URL | https://www.mdpi.com/2077-0383/14/1/287 |
  | Accessed | 05/02/2026, 17:21:31 |
  | Volume | 14 |
  | Publisher | MDPI |
  | Pages | 287 |
  | Publication | Journal of Clinical Medicine |
  | Issue | 1 |
  | Date Added | 05/02/2026, 17:22:09 |
  | Modified | 05/02/2026, 17:22:09 |
- ## Beyond the human touch: A critical review of the promise and challenges of animal-and robot-assisted therapy in loneliness and mental healthcare

  |  |  |
  | --- | --- |
  | Item Type | Journal Article |
  | Author | Lindsey Bertin |
  | Author | Jeffrey S. Katz |
  | Author | Gopikrishna Deshpande |
  | Author | Frank Krueger |
  | Date | 2025 |
  | Short Title | Beyond the human touch |
  | Library Catalogue | Google Scholar |
  | URL | https://www.sciencedirect.com/science/article/pii/S1876201825002588 |
  | Accessed | 05/02/2026, 17:13:21 |
  | Volume | 110 |
  | Publisher | Elsevier |
  | Pages | 104615 |
  | Publication | Asian journal of psychiatry |
  | Date Added | 05/02/2026, 17:13:35 |
  | Modified | 05/02/2026, 17:13:35 |
- ## Animal-assisted intervention for geriatric well-being: A comprehensive review: Healthy ageing

  |  |  |
  | --- | --- |
  | Item Type | Journal Article |
  | Author | Liegelin Kavitha Bernhardt |
  | Author | Asha Vashe |
  | Author | Grisilda Vidya Bernhardt |
  | Author | Janita Pinto |
  | Date | 2024 |
  | Short Title | Animal-assisted intervention for geriatric well-being |
  | Library Catalogue | Google Scholar |
  | URL | http://www.clinicaterapeutica.it/ojs/index.php/1/article/view/921 |
  | Accessed | 05/02/2026, 17:17:16 |
  | Volume | 175 |
  | Publication | La Clinica terapeutica |
  | Issue | 5 |
  | Date Added | 05/02/2026, 17:17:48 |
  | Modified | 05/02/2026, 17:17:48 |

  ### Attachments

  - Available Version (via Google Scholar)
- ## The role of connection in the efficacy of animal-assisted therapies: A scoping review

  |  |  |
  | --- | --- |
  | Item Type | Journal Article |
  | Author | Sharron Beggs |
  | Author | Rob Townsend |
  | Date | 2021 |
  | Short Title | The role of connection in the efficacy of animal-assisted therapies |
  | Library Catalogue | Google Scholar |
  | URL | https://search.informit.org/doi/abs/10.3316/informit.188131443230363 |
  | Accessed | 05/02/2026, 17:17:26 |
  | Volume | 33 |
  | Publisher | Aotearoa New Zealand Association of Social Workers Christchurch, NZ |
  | Pages | 34–47 |
  | Publication | Aotearoa New Zealand Social Work |
  | Issue | 3 |
  | Date Added | 05/02/2026, 17:17:48 |
  | Modified | 05/02/2026, 17:17:48 |

  ### Attachments

  - Available Version (via Google Scholar)
- ## Animal-assisted therapies for autism

  |  |  |
  | --- | --- |
  | Item Type | Journal Article |
  | Author | Anna Beavers |
  | Author | Antoinette Fleming |
  | Author | Jeffrey D. Shahidullah |
  | Date | 2023 |
  | Library Catalogue | Google Scholar |
  | URL | https://www.sciencedirect.com/science/article/pii/S153854422300127X |
  | Accessed | 05/02/2026, 17:17:31 |
  | Volume | 53 |
  | Publisher | Elsevier |
  | Pages | 101478 |
  | Publication | Current Problems in Pediatric and Adolescent Health Care |
  | Issue | 11 |
  | Date Added | 05/02/2026, 17:17:48 |
  | Modified | 05/02/2026, 17:17:48 |
- ## Effects of animal-assisted interventions for people with dementia: A systematic review and meta-analysis

  |  |  |
  | --- | --- |
  | Item Type | Journal Article |
  | Author | Sakti Oktaria Batubara |
  | Author | Santo Imanuel Tonapa |
  | Author | Ita Daryanti Saragih |
  | Author | Mulyadi Mulyadi |
  | Author | Bih-O. Lee |
  | Date | 2022 |
  | Short Title | Effects of animal-assisted interventions for people with dementia |
  | Library Catalogue | Google Scholar |
  | URL | https://www.sciencedirect.com/science/article/pii/S0197457221003360 |
  | Accessed | 05/02/2026, 17:21:59 |
  | Volume | 43 |
  | Publisher | Elsevier |
  | Pages | 26–37 |
  | Publication | Geriatric Nursing |
  | Date Added | 05/02/2026, 17:22:09 |
  | Modified | 05/02/2026, 17:22:09 |
- ## Terapia Ocupacional, Esquizofrenia y Terapia Asistida con Animales. Una revisión sistemática Occupational Therapy, Schizophrenia and Animal Assisted Therapy. A systematic review

  |  |  |
  | --- | --- |
  | Item Type | Journal Article |
  | Author | Lucía Rosón Barrio |
  | Library Catalogue | Google Scholar |
  | URL | https://zaguan.unizar.es/record/117885/files/TAZ-TFG-2022-796.pdf |
  | Accessed | 05/02/2026, 17:34:30 |
  | Date Added | 05/02/2026, 17:34:30 |
  | Modified | 05/02/2026, 17:34:30 |

  ### Attachments

  - Available Version (via Google Scholar)
- ## The Benefits of Animal-Assisted Interventions on Improving Mood in Elder Patients in Nursing Homes from a Volunteer’s Perspective

  |  |  |
  | --- | --- |
  | Item Type | Journal Article |
  | Author | Jessica Banuelos |
  | Date | 2025 |
  | Library Catalogue | Google Scholar |
  | URL | https://search.proquest.com/openview/3de8fe0b153fd0ecbf80226f6465f06e/1?pq-origsite=gscholar&cbl=18750&diss=y |
  | Accessed | 05/02/2026, 17:25:04 |
  | Publisher | The Chicago School of Professional Psychology |
  | Date Added | 05/02/2026, 17:25:16 |
  | Modified | 05/02/2026, 17:25:16 |
- ## A scoping review of campus-based animal-assisted interactions programs for college student mental health

  |  |  |
  | --- | --- |
  | Item Type | Journal Article |
  | Author | Tanya K. Bailey |
  | Date | 2023 |
  | Library Catalogue | Google Scholar |
  | URL | https://docs.lib.purdue.edu/paij/vol6/iss1/1/ |
  | Accessed | 05/02/2026, 17:22:09 |
  | Volume | 6 |
  | Pages | 1 |
  | Publication | People and Animals: The International Journal of Research and Practice |
  | Issue | 1 |
  | Date Added | 05/02/2026, 17:22:09 |
  | Modified | 05/02/2026, 17:22:09 |

  ### Attachments

  - Available Version (via Google Scholar)
- ## The Effect of Animal Assisted Therapy for Elementary Students-A Systematic Review

  |  |  |
  | --- | --- |
  | Item Type | Journal Article |
  | Author | Seung-Jong Bae |
  | Author | Dae-Sik Kim |
  | Author | Sun-Hee Lee |
  | Author | Juyeon Bae |
  | Date | 2022 |
  | Library Catalogue | Google Scholar |
  | URL | https://koreascience.kr/article/JAKO202226362341018.page |
  | Accessed | 05/02/2026, 17:34:20 |
  | Volume | 28 |
  | Publisher | Korean Society of Rural Planning |
  | Pages | 83–94 |
  | Publication | Journal of Korean Society of Rural Planning |
  | Issue | 3 |
  | Date Added | 05/02/2026, 17:34:30 |
  | Modified | 05/02/2026, 17:34:30 |
- ## Animal-Assisted Interventions for Dementia: A Systematic Review

  |  |  |
  | --- | --- |
  | Item Type | Journal Article |
  | Author | Jennifer R. Babka |
  | Author | Kari R. Lane |
  | Author | Rebecca A. Johnson |
  | Abstract | Behavioral and psychological symptoms of dementia (BPSD) can be devastating for people who have dementia and their families. More than 5 million Americans are living with dementia, and approximately 97% of these individuals have BPSD, including agitation, aggression, anxiety, depression, apathy, sleep disturbances, wandering, and disinhibition. Animal-assisted interventions (AAI) have increasingly been used to treat these symptoms in individuals with dementia and constitute an optimal nonpharmacological treatment strategy. The current review aims to explore the literature regarding AAI in treating BPSD. Of 3,573 publications reviewed, 15 articles met inclusion criteria. All studies included a type of AAI exploring BPSD within individuals with dementia. The systematic review unveils AAI may be beneficial for those with dementia; however, further research is necessary to address limitations in the existing literature. [ Research in Gerontological Nursing, 14 (6), 317–324.] |
  | Date | 11/2021 |
  | Language | en |
  | Short Title | Animal-Assisted Interventions for Dementia |
  | Library Catalogue | DOI.org (Crossref) |
  | URL | https://journals.healio.com/doi/10.3928/19404921-20210924-01 |
  | Accessed | 05/02/2026, 17:28:23 |
  | Volume | 14 |
  | Pages | 317-324 |
  | Publication | Research in Gerontological Nursing |
  | DOI | 10.3928/19404921-20210924-01 |
  | Issue | 6 |
  | Journal Abbr | Research in Gerontological Nursing |
  | ISSN | 1940-4921, 1938-2464 |
  | Date Added | 05/02/2026, 17:28:56 |
  | Modified | 05/02/2026, 17:28:56 |

  ### Attachments

  - Available Version (via Google Scholar)
- ## Addressing the Psychosocial Needs of Individuals with Communication Disorders: The Integration of Animal-Assisted Therapy within Counseling

  |  |  |
  | --- | --- |
  | Item Type | Journal Article |
  | Author | W. Leigh Atherton |
  | Author | Daniel Hudock |
  | Abstract | Abstract Individuals with speech, language, and communication disorders often present with psychosocial concerns that span the physiological, intrapersonal, and interpersonal domains of functioning. Despite this fact, the provision of counseling service by speech-language pathologist (SLP) that directly addresses clients' psychosocial needs is sparse. Research shows the primary counseling strategy used by therapists is psychoeducation, failing to effectively address the psychosocial concerns. Integrating complementary approaches to traditional counseling in SLP can enhance both the quality of therapeutic intervention and client outcomes. The purpose of this article is to demonstrate the potential of animal-assisted therapy (AAT) as an adjunctive or complementary approach for counseling within SLP. A review of literature demonstrates a need for improved counseling service provision within SLP treatment, as well as the benefits of integrating AAT. A framework for how AAT intentions and techniques fit within SLP Scope of Practice counseling activities is presented, along with case examples to demonstrate how AAT can be integrated within SLP treatment. It is concluded that the integration of AAT as a complementary approach to traditional SLP counseling can enhance both the frequency of counseling services provided and clients' psychosocial outcomes. |
  | Date | 01/2022 |
  | Language | en |
  | Short Title | Addressing the Psychosocial Needs of Individuals with Communication Disorders |
  | Library Catalogue | DOI.org (Crossref) |
  | URL | http://www.thieme-connect.de/DOI/DOI?10.1055/s-0041-1741556 |
  | Accessed | 05/02/2026, 17:24:40 |
  | Volume | 43 |
  | Pages | 024-034 |
  | Publication | Seminars in Speech and Language |
  | DOI | 10.1055/s-0041-1741556 |
  | Issue | 01 |
  | Journal Abbr | Semin Speech Lang |
  | ISSN | 0734-0478, 1098-9056 |
  | Date Added | 05/02/2026, 17:25:16 |
  | Modified | 05/02/2026, 17:25:16 |

  ### Attachments

  - Available Version (via Google Scholar)
- ## The role of animal assisted therapy in the rehabilitation of mental health disorders: A systematic literature review

  |  |  |
  | --- | --- |
  | Item Type | Journal Article |
  | Author | Denis Arsovski |
  | Date | 2024 |
  | Short Title | The role of animal assisted therapy in the rehabilitation of mental health disorders |
  | Library Catalogue | Google Scholar |
  | URL | https://www.integrmed.org/journal/view.php?number=55 |
  | Accessed | 05/02/2026, 17:13:13 |
  | Volume | 3 |
  | Publisher | Jaseng Medical Foundation |
  | Pages | 142–151 |
  | Publication | Perspectives on Integrative Medicine |
  | Issue | 3 |
  | Date Added | 05/02/2026, 17:13:35 |
  | Modified | 05/02/2026, 17:13:35 |
- ## Animal-Assisted Intervention in Speech-Language Pathology: Practical, Clinical, and Theoretical Considerations

  |  |  |
  | --- | --- |
  | Item Type | Journal Article |
  | Author | Sharon M. Antonucci |
  | Abstract | Abstract Interest in animal-assisted interventions within the field of communication sciences and disorders is growing. As more clinicians become interested in engaging in animal-assisted therapy (AAT) and more researchers begin to study its potential benefits, it will be critical for all stakeholders to demonstrate knowledge of the standards and qualifications for service delivery as well as the challenges that must be met in developing an evidence base for clinical practice. This review highlights some of the foundational information relevant to AAT in the context of speech-language pathology. |
  | Date | 01/2022 |
  | Language | en |
  | Short Title | Animal-Assisted Intervention in Speech-Language Pathology |
  | Library Catalogue | DOI.org (Crossref) |
  | URL | http://www.thieme-connect.de/DOI/DOI?10.1055/s-0041-1741555 |
  | Accessed | 05/02/2026, 17:28:19 |
  | Volume | 43 |
  | Pages | 001-007 |
  | Publication | Seminars in Speech and Language |
  | DOI | 10.1055/s-0041-1741555 |
  | Issue | 01 |
  | Journal Abbr | Semin Speech Lang |
  | ISSN | 0734-0478, 1098-9056 |
  | Date Added | 05/02/2026, 17:28:56 |
  | Modified | 05/02/2026, 17:28:56 |

  ### Attachments

  - Available Version (via Google Scholar)
- ## Healing Smiles: Enhancing Pediatric Dental Care through Animal-assisted Therapy–A Narrative Review

  |  |  |
  | --- | --- |
  | Item Type | Journal Article |
  | Author | Aishwarya Vineshkumar Antala |
  | Author | Pratik B. Kariya |
  | Date | 2023 |
  | Short Title | Healing Smiles |
  | Library Catalogue | Google Scholar |
  | URL | https://journals.lww.com/armh/fulltext/2023/11020/healing\_smiles\_\_enhancing\_pediatric\_dental\_care.19.aspx?context=latestarticles |
  | Accessed | 05/02/2026, 17:28:47 |
  | Volume | 11 |
  | Publisher | Medknow |
  | Pages | 258–262 |
  | Publication | Archives of Medicine and Health Sciences |
  | Issue | 2 |
  | Date Added | 05/02/2026, 17:28:56 |
  | Modified | 05/02/2026, 17:28:56 |
- ## Animal-assisted intervention and health care workers’ psychological health: a systematic review of the literature

  |  |  |
  | --- | --- |
  | Item Type | Journal Article |
  | Author | Daniela Acquadro Maran |
  | Author | Ilaria Capitanelli |
  | Author | Claudio Giovanni Cortese |
  | Author | Olayinka Stephen Ilesanmi |
  | Author | Maria Michela Gianino |
  | Author | Francesco Chirico |
  | Date | 2022 |
  | Short Title | Animal-assisted intervention and health care workers’ psychological health |
  | Library Catalogue | Google Scholar |
  | URL | https://www.mdpi.com/2076-2615/12/3/383 |
  | Accessed | 05/02/2026, 17:13:23 |
  | Volume | 12 |
  | Publisher | MDPI |
  | Pages | 383 |
  | Publication | Animals |
  | Issue | 3 |
  | Date Added | 05/02/2026, 17:13:35 |
  | Modified | 05/02/2026, 17:13:35 |
- ## Animal-Assisted Therapy in Pediatric Care: A qualitative descriptive literature review

  |  |  |
  | --- | --- |
  | Item Type | Journal Article |
  | Author | Romesha Abayanayake |
  | Author | Dilini Weerasooriya |
  | Date | 2024 |
  | Short Title | Animal-Assisted Therapy in Pediatric Care |
  | Library Catalogue | Google Scholar |
  | URL | https://www.theseus.fi/handle/10024/870592 |
  | Accessed | 05/02/2026, 17:28:45 |
  | Date Added | 05/02/2026, 17:28:56 |
  | Modified | 05/02/2026, 17:28:56 |

  ### Attachments

  - Available Version (via Google Scholar)
