# Supplementary material for: Evidence of Physiological Comodulation During Human–Animal Interaction: A Systematic Review
Source: Ann N Y Acad Sci. 2026 Jun 4;1560(1):e70299. doi: 10.1111/nyas.70299 (PMC13238372; doi:10.1111/nyas.70299)
Supplement: Supplementary file 2 — Supplementary Materials: Supp2‐Zotero‐Collection.zip [file NYAS-1560-0-s002.zip › Supp2_Zotero_Collection/new searches/PubMed.htm]

Zotero Report


- ## The impact of canine-assisted intervention on stress reduction among university students in Thailand

  |  |  |
  | --- | --- |
  | Item Type | Journal Article |
  | Author | Saengduean Yotanyamaneewong |
  | Author | Daranee Junla |
  | Author | Janine L. Brown |
  | Author | Nathida Siriapaipant |
  | Author | Naruedee Yodkamol |
  | Author | Worapat Prachasilchai |
  | Author | Adul Saengthong |
  | Author | Pratch Sanguansak |
  | Author | Thanapun Kankonsue |
  | Author | Veerasak Punyapornwithaya |
  | Author | Chalutwan Sansamur |
  | Author | Korakot Nganvongpanit |
  | Author | Jaruwan Khonmee |
  | Abstract | Stress negatively impacts university students, leading to adverse outcomes. While canine-assisted intervention (CAI) has been shown to reduce self-reported stress, no studies have investigated stress levels and associated biomarkers in dogs and students simultaneously. This study examined salivary cortisol, blood pressure, and pulse rate in 122 university students experiencing self-reported moderate to high stress before an encounter with a dog (T1), immediately before meeting a dog (T2), and after spending 15 minutes interacting with a dog (T3). Participants assessed their stress level using a visual analog scale, and blood pressure and pulse rate were measured at three time points. Salivary cortisol was also measured at T1 and T3. Six privately owned dogs, all in good health and comfortable with strangers, participated in the intervention sessions. Salivary and fecal cortisol samples from the dogs were collected in the morning before commencing activities, at noon, and in the evening after human interactions ended. The results showed that the expectation of interacting with dogs reduces self-reported stress, pulse rate, and salivary cortisol, which remained significantly lower after the interaction. Salivary cortisol concentrations in dogs did not differ throughout the day. By contrast, fecal glucocorticoid metabolite concentrations during the week dogs interacted with students were higher (P =  0.0012) than those during the week post-experiment, which, based on behavior, appeared to indicate positive stimulation. These findings highlight the potential of integrating CAI into university stress management programs. Future research could explore extending these benefits to community dogs, long-term effects, and enhancing accessibility to this form of stress relief. |
  | Date | 2025 |
  | Language | eng |
  | Library Catalogue | PubMed |
  | Volume | 20 |
  | Pages | e0318777 |
  | Publication | PloS One |
  | DOI | 10.1371/journal.pone.0318777 |
  | Issue | 3 |
  | Journal Abbr | PLoS One |
  | ISSN | 1932-6203 |
  | PMID | 40072925 |
  | PMCID | PMC11902293 |
  | Date Added | 05/02/2026, 16:23:23 |
  | Modified | 05/02/2026, 16:23:23 |

  ### Tags:

  - Hydrocortisone
  - Dogs
  - Animals
  - Female
  - Adult
  - Heart Rate
  - Humans
  - Male
  - Young Adult
  - Animal Assisted Therapy
  - Blood Pressure
  - Stress, Psychological
  - Saliva
  - Students
  - Feces
  - Thailand
  - Universities

  ### Attachments

  - PubMed entry
- ## Tracking positive and negative affect in PTSD inpatients during a service dog intervention

  |  |  |
  | --- | --- |
  | Item Type | Journal Article |
  | Author | Steven H. Woodward |
  | Author | Andrea L. Jamison |
  | Author | Sasha Gala |
  | Author | Catherine Lawlor |
  | Author | Diana Villasenor |
  | Author | Gisselle Tamayo |
  | Author | Melissa Puckett |
  | Abstract | Though popular across many audiences, engagement with a service dog has undergone limited empirical evaluation as a complementary or alternative treatment for posttraumatic stress disorder (PTSD). The present study took advantage of a service dog training intervention underway in a Department of Veterans Affairs residential PTSD treatment program to perform a within-subjects comparison of a range of phenotypic markers. The present report considers negative and positive affect, assessed throughout the day, contrasting weeks when participants were or were not accompanied by their service dog. Fifty-four veterans were studied for 2-6 weeks. Negative and positive affect were sampled five times per day using items from the Positive and Negative Affect Schedule. Participants also wore a single-patch ECG/activity recorder and slept on beds recording sleep actigraphically. Linear mixed effects regression was employed to estimate the effect of the presence of service dog on momentary affect in the context of other presumable influences. Missing data were managed using methods applicable to random and nonrandom missingness. In this sample, the presence of a service dog was associated with reduced negative and increased positive affect, with both effects diminishing over time. Only negative affect was associated with time in residential treatment, and only positive affect was associated with concurrent heart rate, activity, and the interaction of activity and prior-night actigraphic sleep efficiency. These results concur with prior reports of reduced PTSD symptomology in association with the presence of a service dog, and with the distinct neurocircuitries underlying defensive and appetitive emotion and motivation. Limitations derive from the artificial environment and brief duration of study. (PsycInfo Database Record (c) 2021 APA, all rights reserved). |
  | Date | 2021-06 |
  | Language | eng |
  | Library Catalogue | PubMed |
  | Volume | 89 |
  | Pages | 551-562 |
  | Publication | Journal of Consulting and Clinical Psychology |
  | DOI | 10.1037/ccp0000572 |
  | Issue | 6 |
  | Journal Abbr | J Consult Clin Psychol |
  | ISSN | 1939-2117 |
  | PMID | 34264702 |
  | Date Added | 05/02/2026, 16:23:23 |
  | Modified | 05/02/2026, 16:23:23 |

  ### Tags:

  - Dogs
  - Animals
  - Adult
  - Heart Rate
  - Humans
  - Male
  - Middle Aged
  - Animal Assisted Therapy
  - Stress Disorders, Post-Traumatic
  - Veterans
  - Affect
  - Inpatients
  - Linear Models
  - Residential Treatment
  - Service Animals
  - Sleep

  ### Attachments

  - PubMed entry
- ## Can Dog-Assisted Intervention Decrease Anxiety Level and Autonomic Agitation in Patients with Anxiety Disorders?

  |  |  |
  | --- | --- |
  | Item Type | Journal Article |
  | Author | Dorota Wołyńczyk-Gmaj |
  | Author | Aleksandra Ziółkowska |
  | Author | Piotr Rogala |
  | Author | Dawid Ścigała |
  | Author | Ludwik Bryła |
  | Author | Bartłomiej Gmaj |
  | Author | Marcin Wojnar |
  | Abstract | Few studies have explored the influence of an Animal-Assisted Intervention on patients with mental disorders. We investigated it's impact on anxiety symptoms. We divided 51 patients with anxiety symptoms into two groups-treatment group, that went for a short 15-20 min' walk with a dog, his handler and a researcher and control group, that went for a walk only with a researcher. We used State-Trait Anxiety Inventory (STAI), Visual Analogue Scale (VAS) of fear, Beck Depression Inventory (BDI), Ford Insomnia Response to Stress (FIRST), Brief symptom Inventory (BSI) and VAS of satisfaction after trial to assess. We also checked the resting blood pressure and resting heart rate before and after performing psychological tests while sitting. We have obtained full data of 21 people from the research group and 26 people from the control group. After the intervention, the treatment group reported lower anxiety levels as a state (Mean (M) = 34.35; Standard Deviation (SD) = 6.9 vs. M = 40.94; SD = 8.6) and fear (M = 1.05; SD = 1.0 vs. M = 2.04; SD = 2.2) than the control group. After a walk with a dog, trait anxiety (M = 34.35; SD = 6.9 vs. M = 46.3; SD = 9.6), state anxiety (M = 48.9; SD = 7.2 vs. M = 53.9; SD = 7.8), fear (M = 1.05; SD= 1.0 vs. M = 2.57; SD = 2.3) and resting heart rate (M = 71.05; SD = 12.3 vs. M = 73.67; SD = 13.1) decreased significantly, while walking without a dog only reduced state anxiety (M = 47.24; SD =&nbsp;11.0 vs. M = 40.94; SD =&nbsp;8.6). Multivariate analysis of variance showed that after the walk, state anxiety was significantly lower in the treatment group than in the control group, F(1.35) = 6.706, p <0.05, η2 = 0.161. Among those who walked with a dog, the intervention also led to significant decreases in fear and resting heart rate, F(1.44) = 11.694, p < 0.01, η2 = 0.210 and F(1.45) = 8.503; p < 0.01; η2 = 0.159, respectively. For anxious patients, a short walk with a dog is more beneficial than a walk without one. We found significant positive effects of a dog's company on vegetative arousal and mental comfort. This is another study confirming the possible therapeutic effect of the animal on anxiety symptoms. Further research is required, especially in the large groups of patients, as recommendations on the use of Animal Assisted Interventions (AAI) are needed. |
  | Date | 2021-11-04 |
  | Language | eng |
  | Library Catalogue | PubMed |
  | Volume | 10 |
  | Pages | 5171 |
  | Publication | Journal of Clinical Medicine |
  | DOI | 10.3390/jcm10215171 |
  | Issue | 21 |
  | Journal Abbr | J Clin Med |
  | ISSN | 2077-0383 |
  | PMID | 34768691 |
  | PMCID | PMC8584515 |
  | Date Added | 05/02/2026, 16:20:09 |
  | Modified | 05/02/2026, 16:20:09 |

  ### Tags:

  - dogs
  - heart rate
  - anxiety
  - animal assisted intervention (AAI)
  - anxiety disorders treatment
  - dogotheraphy

  ### Attachments

  - Full Text
  - PubMed entry
- ## Clinical and neurobiological effects of real-life and virtual animal-assisted interventions for patients with depression

  |  |  |
  | --- | --- |
  | Item Type | Journal Article |
  | Author | Julien Willms |
  | Author | Alice Sader |
  | Author | Georg Juckel |
  | Author | Paraskevi Mavrogiorgou |
  | Abstract | BACKGROUND: Animal-assisted interventions (AAIs) have been shown to exert beneficial effects on various mental disorders. However, organizational, hygienic, and ethical challenges often limit their implementation. As a result, virtual alternatives are gaining increasing relevance. Initial findings suggest that digital interactions with animals may also elicit affective and physiological responses, although systematic, controlled studies are lacking. This study aimed to systematically compare the effects of real and virtual AAIs on depressive symptoms and peripheral oxytocin concentrations in patients with depression and healthy controls. METHODS: In a mixed factorial design (N = 66), patients with depression and healthy controls each underwent three conditions in randomized order: interaction with a live therapy dog, a virtual therapy dog, and a virtual reality (VR)-based fantastical creature. Depressive symptoms (per the DASS) and salivary oxytocin concentrations were assessed. RESULTS: All interventions significantly reduced depressive symptoms in patients, with the strongest effects observed for the live therapy dog and the VR-based fantastical creature. Oxytocin levels increased significantly across all conditions, regardless of participant group. CONCLUSION: Virtual animal interactions can evoke psychophysiological effects comparable to real-life AAIs. They appear promising as accessible, technology-supported interventions for psychiatric populations, particularly when access to live animals is limited. |
  | Date | 2026-03 |
  | Language | eng |
  | Library Catalogue | PubMed |
  | Volume | 357 |
  | Pages | 116917 |
  | Publication | Psychiatry Research |
  | DOI | 10.1016/j.psychres.2025.116917 |
  | Journal Abbr | Psychiatry Res |
  | ISSN | 1872-7123 |
  | PMID | 41461126 |
  | Date Added | 05/02/2026, 16:23:23 |
  | Modified | 05/02/2026, 16:23:23 |

  ### Tags:

  - Oxytocin
  - Dogs
  - Animals
  - Female
  - Virtual reality
  - Virtual Reality
  - Adult
  - Humans
  - Male
  - Middle Aged
  - Young Adult
  - Animal Assisted Therapy
  - Saliva
  - Depression
  - Animal-assisted interventions
  - Digital therapeutic approaches
  - Social interaction
  - Therapy dog
  - Virtual reality therapy

  ### Attachments

  - PubMed entry
- ## The effects of Animal Assisted Therapy on autonomic and endocrine activity in adults with autism spectrum disorder: A randomized controlled trial

  |  |  |
  | --- | --- |
  | Item Type | Journal Article |
  | Author | Carolien Wijker |
  | Author | Nina Kupper |
  | Author | Ruslan Leontjevas |
  | Author | Annelies Spek |
  | Author | Marie-Jose Enders-Slegers |
  | Abstract | OBJECTIVE: Stress and its sequelae are very common in adults with autism spectrum disorder (ASD) without an intellectual disability (ID). Animal-assisted therapy (AAT) has shown physiological stress-reductive effects in children with ASD. The aim of the current study was to examine the acute psychophysiological response to an AAT session, and to examine the longer-term stress-physiological effects of the intervention, up until 10 weeks post-treatment, in comparison to waiting-list controls. METHOD: A randomized controlled trial with pre-intervention (T0), post-intervention (T1: 10 weeks) and follow-up (T2: 20 weeks) measurements of neuroendocrine and cardiovascular measures, was conducted in 53 adults with ASD (N = 27 in intervention arm; N = 26 in control arm). Within the intervention group, stress-physiological data were collected during the 5th therapy session (acute effects). Data were analyzed with mixed models for outcome measures cortisol, alpha-amylase, heart rate variability and sympathetic activity. RESULTS: The AAT interventional session was significantly associated with reduced cortisol levels (β = -0.41, p = .010), while parasympathetic and sympathetic cardiovascular activity remained unaltered. No significant changes were found for stress-physiological measures at post-treatment time points. CONCLUSIONS: Acute stress reduction, reflected in significant reduction in cortisol levels, was found during an AAT session in adults with ASD, without ID. More research is needed to explore to what extent the specific factors of AAT have contributed to the decrease in cortisol and whether stress reduction is possible for the longer-term. |
  | Date | 2021 |
  | Language | eng |
  | Short Title | The effects of Animal Assisted Therapy on autonomic and endocrine activity in adults with autism spectrum disorder |
  | Library Catalogue | PubMed |
  | Volume | 72 |
  | Pages | 36-44 |
  | Publication | General Hospital Psychiatry |
  | DOI | 10.1016/j.genhosppsych.2021.05.003 |
  | Journal Abbr | Gen Hosp Psychiatry |
  | ISSN | 1873-7714 |
  | PMID | 34237553 |
  | Date Added | 05/02/2026, 16:20:09 |
  | Modified | 05/02/2026, 16:20:09 |

  ### Tags:

  - Dogs
  - Animals
  - Heart Rate
  - Humans
  - Animal Assisted Therapy
  - Stress
  - Adults
  - Autism spectrum disorder
  - Autism Spectrum Disorder
  - Cardiac autonomic control
  - Endocrinology

  ### Attachments

  - PubMed entry
- ## [Animal-assisted interventions (AAI) in intensive care : Position of the German Society for Internal Intensive Care and Emergency Medicine]

  |  |  |
  | --- | --- |
  | Item Type | Journal Article |
  | Author | Nadine Weeverink |
  | Author | Carsten Hermes |
  | Author | Sebastian Schulz-Stübner |
  | Author | Tobias Ochmann |
  | Author | Matthias Kochanek |
  | Author | Uwe Janssens |
  | Abstract | Animal-assisted interventions (AAI) are increasingly recognized as nonpharmacological adjuncts to intensive care medicine. The aim of this paper by the German Society of Internal Intensive Care and Emergency Medicine (DGIIN) is to summarize the scientific rationale, safety considerations, and implementation recommendations for the use of AAI in intensive care units. Therapeutic effects are attributed to neuroendocrine mechanisms, particularly activation of the oxytocin system and reduction of cortisol levels, which can alleviate anxiety, pain perception, and psychological stress in critically ill patients. Early studies have reported significant reductions in anxiety without an associated increase in nosocomial infections. However, clinical implementation requires well-defined structural, hygienic, and ethical frameworks, including standardized risk analyses, binding hygiene and animal welfare standards, and multiprofessional collaboration integrating intensive care, nursing, infection control, and animal therapy. This paper presents the first systematic recommendations in Germany for the safe and quality-assured integration of animal-assisted interventions into intensive care practice and calls for scientifically accompanied implementation focusing on patient safety, animal welfare, and team well-being. |
  | Date | 2026-01-08 |
  | Language | ger |
  | Short Title | [Animal-assisted interventions (AAI) in intensive care |
  | Library Catalogue | PubMed |
  | Publication | Medizinische Klinik, Intensivmedizin Und Notfallmedizin |
  | DOI | 10.1007/s00063-025-01381-7 |
  | Journal Abbr | Med Klin Intensivmed Notfmed |
  | ISSN | 2193-6226 |
  | PMID | 41504779 |
  | Date Added | 05/02/2026, 16:23:23 |
  | Modified | 05/02/2026, 16:23:23 |

  ### Tags:

  - Animal assisted therapy
  - Complementary therapies
  - Critical care
  - Patient care team
  - Psychological stress

  ### Attachments

  - PubMed entry
- ## Can presence of a dog reduce pain and distress in children during venipuncture?

  |  |  |
  | --- | --- |
  | Item Type | Journal Article |
  | Author | Laura Vagnoli |
  | Author | Simona Caprilli |
  | Author | Chiara Vernucci |
  | Author | Silvia Zagni |
  | Author | Francesca Mugnai |
  | Author | Andrea Messeri |
  | Abstract | The aim of this study was to investigate the effectiveness of animal-assisted intervention as distraction for reducing children's pain and distress before, during, and after standard blood collection procedure. Fifty children (ages 4-11 years) undergoing venipuncture were randomly assigned to the experimental group (EG; n = 25) or to the control group (CG; n = 25). The blood collection procedure was carried on the children in the EG arm in the presence of a dog, whereas no dog was present when venipuncture was conducted on children in CG. In both cases, parents accompanied the child in the procedure room. Distress experienced by the child was measured with the Amended Observation Scale of Behavioral Distress, while perceived pain was measured with a visual analog scale or the Wong Baker Scale (Faces Scale); levels of cortisol in blood also were analyzed. Parental anxiety during the procedure was measured with State Trait Anxiety Inventory. Children assigned to the EG group reacted with less distress than children in the CG arm. Furthermore, cortisol levels were lower in the EG group compared with the CG group. There were no significant differences in pain ratings and in the level of parental anxiety. It appears that the presence of dogs during blood draw procedures reduces distress in children. |
  | Date | 2015-04 |
  | Language | eng |
  | Library Catalogue | PubMed |
  | Volume | 16 |
  | Pages | 89-95 |
  | Publication | Pain Management Nursing: Official Journal of the American Society of Pain Management Nurses |
  | DOI | 10.1016/j.pmn.2014.04.004 |
  | Issue | 2 |
  | Journal Abbr | Pain Manag Nurs |
  | ISSN | 1532-8635 |
  | PMID | 25439114 |
  | Date Added | 05/02/2026, 16:23:23 |
  | Modified | 05/02/2026, 16:23:23 |

  ### Tags:

  - Dogs
  - Animals
  - Female
  - Humans
  - Male
  - Animal Assisted Therapy
  - Stress, Psychological
  - Child
  - Child, Preschool
  - Pain Management
  - Phlebotomy

  ### Attachments

  - PubMed entry
- ## Psychophysiological mechanisms underlying the potential health benefits of human-dog interactions: A systematic literature review

  |  |  |
  | --- | --- |
  | Item Type | Journal Article |
  | Author | Jillian T. Teo |
  | Author | Stuart J. Johnstone |
  | Author | Stephanie S. Römer |
  | Author | Susan J. Thomas |
  | Abstract | While the symbiotic nature of human-dog relationships and perceived benefits to human health have attracted much scientific interest, the mechanisms through which human-dog interactions may confer health benefits to humans are still poorly understood. The aim of this systematic literature review was to synthesize evidence of physiological changes associated with human-dog interactions with relevance to human health. Electronic databases were systematically searched (PubMed, MEDLINE with full text, Scopus, PsycINFO, CINAHL Plus with Full Text, and Web of Science Core Collection) for relevant studies. Of the 13,072 studies identified, 129 met the inclusion criteria, with approximately half being randomized trials (Level 2) based on the Oxford Centre for Evidence Based Medicine level system. Measures employed to study human physiological changes associated with human-dog interactions most commonly involved cardiac parameters and hormones, with negligible research of brain activity. The main positive findings were increases in heart rate variability and oxytocin, and decreases in cortisol with human-dog interactions. These physiological indicators are consistent with activation of the parasympathetic nervous system (PNS) and oxytocinergic system (OTS), and down-regulation of hypothalamic-pituitary-adrenal (HPA) axis activity. These results provide evidence of specific pathways through which human-dog contact may confer health benefits, likely through relaxation, bonding, and stress reduction. However, these findings should be interpreted contextually due to limitations and methodological differences. Previous research using other biological variables was limited in quantity and quality, thus impeding firm conclusions on other possible mechanisms. Further research is needed in some psychophysiological domains, particularly electroencephalography, to better understand central nervous system (CNS) effects. The findings of this review have implications for human-dog interactions to positively affect several stress-sensitive physiological pathways and thus confer health benefits. This supports their incorporation in various clinical, non-clinical, and research settings to develop evidence-based interventions and practices for cost-effective and efficacious ways to improve human health. |
  | Date | 2022-10 |
  | Language | eng |
  | Short Title | Psychophysiological mechanisms underlying the potential health benefits of human-dog interactions |
  | Library Catalogue | PubMed |
  | Volume | 180 |
  | Pages | 27-48 |
  | Publication | International Journal of Psychophysiology: Official Journal of the International Organization of Psychophysiology |
  | DOI | 10.1016/j.ijpsycho.2022.07.007 |
  | Journal Abbr | Int J Psychophysiol |
  | ISSN | 1872-7697 |
  | PMID | 35901904 |
  | Date Added | 05/02/2026, 16:20:09 |
  | Modified | 05/02/2026, 16:20:09 |

  ### Tags:

  - Dogs
  - Animals
  - Humans
  - Psychophysiology
  - Animal-assisted intervention
  - Human-animal interaction
  - Systematic review

  ### Attachments

  - PubMed entry
- ## Effects of canine-assisted intervention on stress and depression in humans

  |  |  |
  | --- | --- |
  | Item Type | Journal Article |
  | Author | Yujin Song |
  | Author | Youngwook Jung |
  | Author | Yeonju Choi |
  | Author | Minjung Yoon |
  | Abstract | The demand for mental health support systems has been increasing because of the rising prevalence of mental health issues globally. These challenges related to mental health have been addressed through animal-assisted intervention. This approach has gained recognition as an effective method that enhances emotional stability and fosters social bonds. Canine-assisted intervention, a subset of animal-assisted intervention that involves dogs, is recognized for its effectiveness in managing stress and depression in humans. Despite the development of various canine-assisted intervention programs, there is insufficient scientific data evaluating the efficacy of each program. Customized programs that target individuals' symptoms and needs are necessary to effectively manage stress and depression. As such, generalizing the effects of canine-assisted intervention across diverse situations continues to be a challenge. This review aims to identify the most effective canine-assisted intervention programs for various target groups and suggest strategies that maximize the effects of canine-assisted intervention programs by consolidating various biometric indicators and physiological evaluation tools and by analyzing the effects of canine-assisted intervention through multiple approaches. It examines current studies demonstrating how interactions with therapy dogs lead to remarkable psychological and physiological changes, including measurable reductions in stress indicators (such as cortisol levels and heart rates) and notable improvements in overall mood and emotional well-being. Furthermore, this paper evaluates the effectiveness of canine-assisted intervention in various settings, highlighting its potential as a therapeutic intervention and preventive measure in mental health care. Based on previous findings, this review provides a comprehensive overview of the role of canine-assisted intervention in enhancing human mental health and its potential for broader implementation across diverse environments. |
  | Date | 2025-07 |
  | Language | eng |
  | Library Catalogue | PubMed |
  | Volume | 67 |
  | Pages | 719-736 |
  | Publication | Journal of Animal Science and Technology |
  | DOI | 10.5187/jast.2025.e5 |
  | Issue | 4 |
  | Journal Abbr | J Anim Sci Technol |
  | ISSN | 2055-0391 |
  | PMID | 40874008 |
  | PMCID | PMC12380019 |
  | Date Added | 05/02/2026, 16:20:09 |
  | Modified | 05/02/2026, 16:20:09 |

  ### Tags:

  - Depression
  - Stress
  - Canine-assisted intervention
  - Well-being

  ### Attachments

  - Full Text PDF
  - PubMed entry
- ## Can Dogs Assist Children with Severe Autism Spectrum Disorder in Complying with Challenging Demands? An Exploratory Experiment with a Live and a Robotic Dog

  |  |  |
  | --- | --- |
  | Item Type | Journal Article |
  | Author | Karine Silva |
  | Author | Mariely Lima |
  | Author | André Santos-Magalhães |
  | Author | Carla Fafiães |
  | Author | Liliana de Sousa |
  | Abstract | OBJECTIVES: Prompted by the need to find effective ways to enhance compliance in children with autism spectrum disorder (ASD), and building on the increasing interest in dog-assisted interventions for this population, this study provides an exploratory test on whether dogs may assist children with severe ASD in complying with challenging demands while also decreasing behavioral and cardiovascular distress. DESIGN: A within-subject design was used. Depending on condition, participants were allowed to engage with a particular stimulus-their preferred toy, a live dog, or a robotic dog-before being exposed to a demanding task in which they had to wait for permission to eat a desired food item ("prohibition task"). Although inactive, the stimulus remained present during the prohibition task. SUBJECTS AND SETTINGS: Ten male children, aged between 6 and 9 years and diagnosed with severe ASD, participated in this study. All were clinically referred as having serious compliance difficulties in everyday routines. Testing occurred at participants' homes. OUTCOME MEASURES: Participants' emotional expressions, latency to distress, compliance levels, and behaviors that were shown during committed compliance were assessed during the prohibition task. In addition, cardiovascular reactivity to the task was monitored. RESULTS: Obtained data revealed significant differences between conditions for some of the considered measures. Latency to distress was higher in the live dog than in the toy condition. Committed compliance was higher in the live dog than in the toy and robot conditions. Quiet waiting during committed compliance was higher in the live dog condition than in the toy condition, and tension release behaviors were lower. In addition, heart rate reactivity was lower in the live dog condition than in the toy condition. CONCLUSIONS: The live dog condition appeared to have a calming effect on the participants, hypothetically facilitating compliance. Although promising, these findings are only preliminary and their clinical significance needs to be assessed in future studies. |
  | Date | 2018-03 |
  | Language | eng |
  | Short Title | Can Dogs Assist Children with Severe Autism Spectrum Disorder in Complying with Challenging Demands? |
  | Library Catalogue | PubMed |
  | Volume | 24 |
  | Place | New York, N.Y. |
  | Pages | 238-242 |
  | Publication | Journal of Alternative and Complementary Medicine |
  | DOI | 10.1089/acm.2017.0254 |
  | Issue | 3 |
  | Journal Abbr | J Altern Complement Med |
  | ISSN | 1557-7708 |
  | PMID | 29116816 |
  | Date Added | 05/02/2026, 16:23:23 |
  | Modified | 05/02/2026, 16:23:23 |

  ### Tags:

  - Dogs
  - dogs
  - Animals
  - Female
  - Heart Rate
  - Humans
  - Male
  - Animal Assisted Therapy
  - heart rate variability
  - Emotions
  - Child
  - Robotics
  - autism spectrum disorder
  - Child Behavior
  - Autism Spectrum Disorder
  - compliance
  - Patient Preference
  - robotic dogs
  - Task Performance and Analysis

  ### Attachments

  - PubMed entry
- ## Impact of an animal-assisted therapy programme on physiological and psychosocial variables of paediatric oncology patients

  |  |  |
  | --- | --- |
  | Item Type | Journal Article |
  | Author | Nathiana B. Silva |
  | Author | Flávia L. Osório |
  | Abstract | The objective of this study was to propose an intervention and safety protocol for performing animal-assisted therapy (AAT) and evaluating its efficacy in children under outpatient oncological treatment based on psychological, physiological, and quality of life indicators for the children and caregivers. The sample consisted of 24 children diagnosed with leukaemia and solid tumours (58% girls with a mean age of 8.0 years) who underwent an AAT programme consisting of three 30-min sessions in an open group. Two dogs (one Labrador retriever and one golden retriever) were used, and activities such as sensory stimulation, gait training, and socialization were conducted. The exclusion criteria were severe mental problems, inability to answer the questions included in the instruments used, allergy to animals, unavailability/lack of interest, isolation precaution, surgical wound, use of invasive devices, ostomy, no current blood count for evaluation, neutropaenia, infection, fever, diarrhoea, vomiting, respiratory symptoms at the beginning of the intervention or 1 week before the intervention, hospitalization or scheduled surgery, and non-completion of the AAT programme. The variables analysed using validated self or other evaluations were stress, pain, mood, anxiety, depression, quality of life, heart rate, and blood pressure. A quasi-experimental study design was used. We observed a decrease in pain (p = 0.046, d = -0.894), irritation (p = 0.041, d = -0.917), and stress (p = 0.005; d = -1.404) and a tendency towards improvement of depressive symptoms (p = 0.069; d = -0.801). Among the caregivers, an improvement was observed in anxiety (p = 0.007, d = -1.312), mental confusion (p = 0.006, d = -1.350), and tension (p = 0.006, d = -1.361). Therefore, the selection criteria and care protocols used for the AAT programme in the oncological context were adequate, and the programme was effective. |
  | Date | 2018 |
  | Language | eng |
  | Library Catalogue | PubMed |
  | Volume | 13 |
  | Pages | e0194731 |
  | Publication | PloS One |
  | DOI | 10.1371/journal.pone.0194731 |
  | Issue | 4 |
  | Journal Abbr | PLoS One |
  | ISSN | 1932-6203 |
  | PMID | 29617398 |
  | PMCID | PMC5884536 |
  | Date Added | 05/02/2026, 16:20:09 |
  | Modified | 05/02/2026, 16:20:09 |

  ### Tags:

  - Dogs
  - Animals
  - Female
  - Heart Rate
  - Humans
  - Male
  - Animal Assisted Therapy
  - Stress, Psychological
  - Depression
  - Child
  - Anxiety
  - Neoplasms
  - Pain
  - Quality of Life
  - Caregivers
  - Program Evaluation

  ### Attachments

  - Full Text
  - PubMed entry
- ## Non-randomized controlled trial examining the effects of livestock on motivation and anxiety in patients with chronic psychiatric disorders

  |  |  |
  | --- | --- |
  | Item Type | Journal Article |
  | Author | Nobuko Shimizu |
  | Author | Chika Yamazaki |
  | Author | Keigo Asano |
  | Author | Shingo Ohe |
  | Author | Motohiko Ishida |
  | Abstract | OBJECTIVES: Patients with chronic schizophrenia exhibit negative symptoms, including decreased work motivation. Animal-assisted therapy programs have been reported to benefit such patients; hence, there is a possibility that sheep-rearing, rather than conventional employment training, may motivate these patients. Therefore, we investigated the effects of a one-day experiential learning program of sheep-rearing on the work motivation and anxiety of patients with chronic schizophrenia. METHODS: Fourteen patients were included in a non-randomized controlled trial conducted between August 2018 and October 2018. The patients' participation in the sheep-rearing experiential learning (one day; intervention day) and normal day care (one day; control day) programs were compared. The salivary cortisol and testosterone levels and State-Trait Anxiety Inventory (STAI) scores of the patients were analyzed. RESULTS: The patients' salivary testosterone was significantly higher on the intervention day (p = 0.04) than on the control day (p = 0.02). Their salivary cortisol was lower on the control day than on the intervention day, although the difference was not significant. Regression analysis was performed based on the change in salivary cortisol levels and STAI-Trait scores (p = 0.006), and a regression equation was established. CONCLUSIONS: The study revealed that participation in sheep-rearing may have promoted the testosterone production but did not increase anxiety in patients with schizophrenia. Additionally, regression equations for salivary cortisol levels in such patients may provide information on individual differences in anxiety levels. |
  | Date | 2023 |
  | Language | eng |
  | Library Catalogue | PubMed |
  | Volume | 11 |
  | Pages | 20503121231175291 |
  | Publication | SAGE open medicine |
  | DOI | 10.1177/20503121231175291 |
  | Journal Abbr | SAGE Open Med |
  | ISSN | 2050-3121 |
  | PMID | 37251360 |
  | PMCID | PMC10214043 |
  | Date Added | 05/02/2026, 16:23:23 |
  | Modified | 05/02/2026, 16:23:23 |

  ### Tags:

  - social support
  - motivation
  - schizophrenia
  - Anxiety
  - rehabilitation
  - non-randomized controlled trial

  ### Attachments

  - Full Text
  - PubMed entry
- ## Acute salivary cortisol response in children with ADHD during psychosocial intervention with and without therapy dogs

  |  |  |
  | --- | --- |
  | Item Type | Journal Article |
  | Author | Sabrina E. B. Schuck |
  | Author | Cassie N. Zeiler |
  | Author | Annamarie Stehli |
  | Author | Lydia A. Steinhoff |
  | Author | Rachel Y. Stokes |
  | Author | Sara E. Jeffrey |
  | Author | Douglas Alan Granger |
  | Abstract | INTRODUCTION: Children with Attention Deficit/Hyperactivity Disorder (ADHD) participated in a randomized clinical trial comparing animal-assisted intervention (AAI) to psychosocial treatment as usual (TAU). This brief report describes effects of AAI on acute HPA axis reactivity and regulation. Saliva was collected before, during, and after psychosocial intervention sessions with and without therapy dogs and later assayed for cortisol (ug/dL). METHODOLOGY: Thirty-nine participants (n = 39) with ADHD, aged 7-9 years (79% male) provided saliva at 3 points during 90-minute sessions; (i) upon arrival, (ii) +20 minutes, and (iii) 15 minutes prior to departure, on 3 occasions across an 8-week intervention (weeks 1, 4, and 8). Cortisol slopes calculated within each session were compared across the intervention weeks to determine within subject and between group effect sizes. Spearman's correlations between baseline individual neurodevelopmental symptoms and in-session acute cortisol responses were also evaluated. RESULTS: No significant between group differences were observed in cortisol responsiveness at week-1. By week-4, in-session changes in cortisol were evident, with significantly greater decreases in the AAI group (Cohen's d = -.40). This pattern was also observed at week-8, with an even stronger effect-size (d = -0.60). Concurrent symptoms of autism were associated with the in-session acute cortisol response. Specifically, higher parent-reported symptom scores were associated with steeper decreases in cortisol across the session at week 1 (r = -0.42, p <.01) and week-8 (r = -0.34 p = .05). At week-8 this association was stronger in the AAI group (r = -0.53) versus TAU (r = -0.25), with Cohen's q = 0.413). DISCUSSION: AAI may influence acute HPA reactivity and regulation for children with ADHD. Concurrent symptoms of ADHD and autism may be related to individual differences in the nature of the effect. Implications of these findings for AAI as an alternative, or complementary intervention for ADHD are discussed. CLINICAL TRIAL REGISTRATION: ClinicalTrials.gov, identifier NCT05102344. |
  | Date | 2024 |
  | Language | eng |
  | Library Catalogue | PubMed |
  | Volume | 15 |
  | Pages | 1476522 |
  | Publication | Frontiers in Psychiatry |
  | DOI | 10.3389/fpsyt.2024.1476522 |
  | Journal Abbr | Front Psychiatry |
  | ISSN | 1664-0640 |
  | PMID | 39512897 |
  | PMCID | PMC11540863 |
  | Date Added | 05/02/2026, 16:20:09 |
  | Modified | 05/02/2026, 16:20:09 |

  ### Tags:

  - therapy dogs
  - animal assisted interventions
  - attention deficit hyperactivity disorder (ADHD)
  - autism symptomatology
  - cortisol (Cor)
  - psychosocial skills intervention
  - school-based intervention

  ### Attachments

  - Full Text PDF
  - PubMed entry
- ## The effect of a service dog on salivary cortisol awakening response in a military population with posttraumatic stress disorder (PTSD)

  |  |  |
  | --- | --- |
  | Item Type | Journal Article |
  | Author | Kerri E. Rodriguez |
  | Author | Crystal I. Bryce |
  | Author | Douglas A. Granger |
  | Author | Marguerite E. O'Haire |
  | Abstract | Recent studies suggest a therapeutic effect of psychiatric service dogs for military veterans with posttraumatic stress disorder (PTSD), but are limited by self-report biases. The current study assessed the effect of PTSD service dogs on the salivary cortisol awakening response (CAR) and arousal-related functioning in a population of military veterans with PTSD. Participants included 73 post-9/11 military veterans with PTSD including 45 with a service dog and 28 on the waitlist to receive one. Saliva samples were collected on two consecutive weekday mornings at awakening and 30 min later to quantify the cortisol awakening response (CAR) and its area under the curve (AUCi) in addition to standardized survey measures of anxiety, anger, sleep quality and disturbance, and alcohol abuse. There was a significant main effect of having a service dog on both the CAR and the AUCi, with individuals with a service dog exhibiting a higher CAR and AUCi compared to those on the waitlist. Results also revealed that those with a service dog reported significantly lower anxiety, anger, and sleep disturbance as well as less alcohol abuse compared to those on the waitlist, with medium to large effect sizes. Although those with a service dog reported significantly less PTSD symptom severity, CAR was not significantly associated with PTSD symptoms within or across group. In conclusion, results indicate that the placement of a PTSD service dog may have a significant positive influence on both physiological and psychosocial indicators of wellbeing in military veterans with PTSD. Although clinical significance cannot be confirmed, a higher CAR/AUCi among those with a service dog may indicate better health and wellbeing in this population. Future within-subject, longitudinal research will be necessary to determine potential clinical significance and impact of individual differences. |
  | Date | 2018-12 |
  | Language | eng |
  | Library Catalogue | PubMed |
  | Volume | 98 |
  | Pages | 202-210 |
  | Publication | Psychoneuroendocrinology |
  | DOI | 10.1016/j.psyneuen.2018.04.026 |
  | Journal Abbr | Psychoneuroendocrinology |
  | ISSN | 1873-3360 |
  | PMID | 29907299 |
  | PMCID | PMC8454180 |
  | Date Added | 05/02/2026, 16:23:23 |
  | Modified | 05/02/2026, 16:23:23 |

  ### Tags:

  - Hydrocortisone
  - Dogs
  - PTSD
  - Animals
  - Female
  - Adult
  - Humans
  - Male
  - Middle Aged
  - Animal Assisted Therapy
  - Stress, Psychological
  - Human-animal interaction
  - Surveys and Questionnaires
  - Saliva
  - Cortisol awakening response
  - Military Personnel
  - Military veterans
  - Posttraumatic stress disorder
  - Service dogs
  - Stress Disorders, Post-Traumatic
  - Veterans

  ### Attachments

  - Accepted Version
  - PubMed entry
- ## Urinary oxytocin levels in children meeting a Hospital Dog®

  |  |  |
  | --- | --- |
  | Item Type | Journal Article |
  | Author | A. Risberg |
  | Author | A. Larsson |
  | Author | U. Bodén |
  | Author | A. Edner |
  | Abstract | There has been growing interest in animal-assisted therapy (AAT) in recent decades due to increasing reports indicating its health benefits for adult patients. These benefits are partly attributed to changes, usually increased levels of the neuropeptide oxytocin.  AIM: To investigate changes in oxytocin levels in hospitalized children meeting a certified Hospital Dog®. METHOD: Urine samples were collected between 25/02/2016 and 24/05/2017 from 35 hospitalized children (3-17 years) before and after each participant had a session with the Hospital Dog®. Oxytocin levels were analysed with an acetylcholinesterase (AChE) competitive enzyme-linked immunosorbent assay (ELISA). Creatinine levels were measured to determine the subject's fluid intake and then divided by the hormonal concentration (uOT pg/ml). RESULTS: The mean level of uOT was 186.0 ± 236.7 (51.5-1349.5) pg/ml before and 137.3 ± 121.5 (30.7-591.3) pg/ml after the dog session (p = 0.010). CONCLUSION: Decreased levels of uOT were recorded during the study in which hospitalized children met a Hospital Dog®. The decreased OT levels are potentially the result of the intense activity the subject experienced with the dog during the interaction. |
  | Date | 2025-09-08 |
  | Language | eng |
  | Library Catalogue | PubMed |
  | Volume | 25 |
  | Pages | 327 |
  | Publication | BMC complementary medicine and therapies |
  | DOI | 10.1186/s12906-025-05076-6 |
  | Issue | 1 |
  | Journal Abbr | BMC Complement Med Ther |
  | ISSN | 2662-7671 |
  | PMID | 40922012 |
  | PMCID | PMC12418696 |
  | Date Added | 05/02/2026, 16:23:23 |
  | Modified | 05/02/2026, 16:23:23 |

  ### Tags:

  - Oxytocin
  - Dogs
  - Animals
  - Female
  - Humans
  - Male
  - Animal Assisted Therapy
  - Child
  - Child, Preschool
  - Adolescent
  - Child, Hospitalized
  - Children
  - Dog therapy
  - Hospital Dog®
  - Urine oxytocin

  ### Attachments

  - PubMed entry
- ## The role of cortisol in the association of canine-companionship with blood pressure, glucose, and lipids: a systematic review

  |  |  |
  | --- | --- |
  | Item Type | Journal Article |
  | Author | D. Rathish |
  | Author | R. P. V. J. Rajapakse |
  | Author | K. G. a. D. Weerakoon |
  | Abstract | INTRODUCTION: The dog is known as man's best friend and canine-companionship is associated with positive effects on cardiovascular health. AIM: We aim to review the role of cortisol in the association of canine-companionship with human blood pressure, glucose and lipid profile. METHODS: Electronic databases, and reference lists of the selected articles were searched for original articles in English which evaluate the role of cortisol in the association of canine-companionship with human blood pressure, glucose and lipid profile. Appropriate tools from the National Institute of Health were used for the quality assessment. RESULTS: Of the 2108 articles included for screening, 6 studies fulfilled the selection criteria. The USA had produced the highest number of studies (50%, 3/6). Pre-post studies (83%, 5/6) were the predominant type, and the overall quality of the selected studies was acceptable. The majority of studies showed a significant reduction of cortisol with dog companionship or therapy (67%, 4/6) along with a significant reduction of heart rate (2/4), systolic blood pressure (1/4), mean arterial blood pressure (1/4), or total cholesterol level (1/4). The role of cortisol in the association of canine-companionship with human blood pressure, glucose and lipid profile was scarcely studied with no studies from lower-middle-income countries, where the disease burden is on the rise. CONCLUSION: A significant reduction of few cardiovascular risk factors was found along with a significant reduction of cortisol in canine companionship in few studies. Future comparative or higher-level studies are essential on the association between canine companionship, cortisol and cardiovascular risk factors, especially in lower-middle-income countries. |
  | Date | 2021-09 |
  | Language | eng |
  | Short Title | The role of cortisol in the association of canine-companionship with blood pressure, glucose, and lipids |
  | Library Catalogue | PubMed |
  | Volume | 28 |
  | Pages | 447-455 |
  | Publication | High Blood Pressure & Cardiovascular Prevention: The Official Journal of the Italian Society of Hypertension |
  | DOI | 10.1007/s40292-021-00469-3 |
  | Issue | 5 |
  | Journal Abbr | High Blood Press Cardiovasc Prev |
  | ISSN | 1179-1985 |
  | PMID | 34351596 |
  | Date Added | 05/02/2026, 16:20:09 |
  | Modified | 05/02/2026, 16:20:09 |

  ### Tags:

  - Hydrocortisone
  - Dogs
  - Animals
  - Human-Animal Bond
  - Humans
  - Blood Pressure
  - Blood Glucose
  - Animal-assisted therapy
  - Cardiovascular health
  - Diabetes
  - Dyslipidaemia
  - Hypertension
  - Lipids

  ### Attachments

  - PubMed entry
- ## Comparison of contingent and noncontingent access to therapy dogs during academic tasks in children with autism spectrum disorder

  |  |  |
  | --- | --- |
  | Item Type | Journal Article |
  | Author | Alexandra Protopopova |
  | Author | Ashley L. Matter |
  | Author | Breanna N. Harris |
  | Author | Katie M. Wiskow |
  | Author | Jeanne M. Donaldson |
  | Abstract | This study compared contingent and noncontingent access to therapy dogs during educational tasks for children with autism spectrum disorder using a multielement design. The experimenters assessed whether initial preference for the dog predicted reinforcer efficacy and how preference changed across time. A higher response rate during contingent dog sessions than baseline sessions occurred for 4 out of 5 participants, suggesting that the dog functioned as a reinforcer. One participant engaged in a high rate of responding in both contingent and noncontingent dog conditions. Preference assessments revealed idiosyncrasies, suggesting that further research is needed into the predictive nature of initial preference assessments with animals as part of the stimulus array. The experimenters also analyzed salivary cortisol before and after sessions to determine if learning about the upcoming interaction with a dog reduced salivary cortisol in children. Cortisol was variable across participants, with only some deriving a potential physiological benefit from expecting to interact with the dog. |
  | Date | 2020-04 |
  | Language | eng |
  | Library Catalogue | PubMed |
  | Volume | 53 |
  | Pages | 811-834 |
  | Publication | Journal of Applied Behavior Analysis |
  | DOI | 10.1002/jaba.619 |
  | Issue | 2 |
  | Journal Abbr | J Appl Behav Anal |
  | ISSN | 1938-3703 |
  | PMID | 31378932 |
  | Date Added | 05/02/2026, 16:23:23 |
  | Modified | 05/02/2026, 16:23:23 |

  ### Tags:

  - cortisol
  - Dogs
  - Animals
  - Humans
  - Child
  - animal-assisted intervention
  - autism
  - therapy dog
  - Autism Spectrum Disorder
  - Therapy Animals
  - Learning
  - preference assessment

  ### Attachments

  - PubMed entry
- ## Effects of human and animal-assisted skills training on oxytocin und cortisol levels in patients with borderline personality disorder

  |  |  |
  | --- | --- |
  | Item Type | Journal Article |
  | Author | Olivia Plett |
  | Author | Vera Flasbeck |
  | Author | Martin Brüne |
  | Abstract | OBJECTIVE: Borderline Personality Disorder (BPD) is characterised, among other symptoms, by emotional instability and difficulties in regulating proximity to significant others. Many with BPD have difficulties in establishing a trustful therapeutic relationship, which often develop before a background of adverse childhood experiences with caregivers. One way to facilitate therapeutic interaction in psychotherapy incorporates pet animals as "door openers". No study exists, however, that has examined the effect of animal-assisted versus human-guided skills training on neurobiological correlates of affiliation and stress regulation, i.e. oxytocin and cortisol. METHODS: Twenty in-patients diagnosed with BPD were recruited to participate in an animal-assisted skills-training. Another 20 in-patients participated in a human-guided skills-training. Salivary samples of both groups were taken for determining oxytocin and cortisol before and immediately after 3 therapeutic sessions at least one week apart from one another. In addition, borderline symptom severity (BSL-23), impulsivity (BIS-15), alexithymia (TAS-20), and fear of compassion (FOCS) were determined by self-rating questionnaires before and after the 6-week interventions. RESULTS: Both therapeutic interventions led to a significant reduction in cortisol and an (non-significant) increase in oxytocin, respectively. Importantly, there was a statistically significant interaction between changes in cortisol and oxytocin, independent of group. Both groups further showed clinical improvement as measured using the above-listed questionnaires. CONCLUSION: Our findings suggest that both animal-assisted and human-guided interventions have measurable short-term effects on affiliative and stress hormones, with no approach being superior to the other in this regard. |
  | Date | 2023-06 |
  | Language | eng |
  | Library Catalogue | PubMed |
  | Volume | 162 |
  | Pages | 156-160 |
  | Publication | Journal of Psychiatric Research |
  | DOI | 10.1016/j.jpsychires.2023.05.004 |
  | Journal Abbr | J Psychiatr Res |
  | ISSN | 1879-1379 |
  | PMID | 37156130 |
  | Date Added | 05/02/2026, 16:20:09 |
  | Modified | 05/02/2026, 16:20:09 |

  ### Tags:

  - Hydrocortisone
  - Oxytocin
  - Animals
  - Humans
  - Empathy
  - Cortisol
  - Animal-assisted therapy
  - Psychotherapy
  - Borderline personality disorder
  - Borderline Personality Disorder
  - Skills training

  ### Attachments

  - PubMed entry
- ## Dog-assisted therapy for control of anxiety in pediatric dentistry

  |  |  |
  | --- | --- |
  | Item Type | Journal Article |
  | Author | Sérgio Luiz Pinheiro |
  | Author | Camila Silva |
  | Author | Lidiane Luiz |
  | Author | Nubia Silva |
  | Author | Rafaela Fonseca |
  | Author | Thaís Velásquez |
  | Author | Diana Roberta Grandizoli |
  | Abstract | Anxiety is common in pediatric dental care, and affects the behavioral management of children. Animal-assisted therapy (AAT) has been shown to improve children's behavior. However, few studies have applied this technique in dentistry. The aim of the present study was to evaluate the applicability of dog-assisted therapy to control anxiety during pediatric dental treatment. Twenty children were selected from the Pediatric Dentistry Clinic of the Pontifical Catholic University of Campinas (PUC-Campinas), Brazil. Participants were divided into two groups: Control (n = 11; visits = 16), in which children were conditioned by methods routinely used in the clinic; and AAT (n = 9; visits = 23), in which children had contact with a dog therapist first at the reception desk and then again inside the office. The dog therapist stayed beside the dental chair with the child throughout the procedures. Corah's Dental Anxiety Scale (CS) and heart rate (HR) were used for evaluation of child anxiety. The results were tested for normality of distribution with the Shapiro-Wilk method, and subsequently analyzed in BioEstat 5.0. HR results were compared by Analysis of Variance (ANOVA) with Tukey's test, and CS scores, with the Wilcoxon test. There was a significant reduction in HR in the AAT group (p = 0.0069). In the Control group, HR did not change before, during, or after treatment (p = 0.6052). Controls showed a significant increase in anxiety measured by CS before and after treatment (p = 0.0455). In the AAT group, there was no change in CS scores before and after treatment (p = 0.3739). AAT could be an alternative to reduce anxiety during pediatric dental care. |
  | Date | 2023-11 |
  | Language | eng |
  | Library Catalogue | PubMed |
  | Volume | 47 |
  | Pages | 38-43 |
  | Publication | The Journal of Clinical Pediatric Dentistry |
  | DOI | 10.22514/jocpd.2023.080 |
  | Issue | 6 |
  | Journal Abbr | J Clin Pediatr Dent |
  | ISSN | 1053-4628 |
  | PMID | 37997233 |
  | Date Added | 05/02/2026, 16:20:09 |
  | Modified | 05/02/2026, 16:20:09 |

  ### Tags:

  - Dogs
  - Animals
  - Humans
  - Animal Assisted Therapy
  - Child
  - Brazil
  - Anxiety
  - Animal assisted therapy
  - Dental Anxiety
  - Pediatric dentistry
  - Pediatric Dentistry

  ### Attachments

  - Full Text
  - PubMed entry
- ## Dog companionship and cortisol levels in youth. A systematic review and meta-analysis

  |  |  |
  | --- | --- |
  | Item Type | Journal Article |
  | Author | Humberto Peña-Jorquera |
  | Author | Sam Hernández-Jaña |
  | Author | Javier Sanchez-Martinez |
  | Author | Juan Pablo Espinoza-Puelles |
  | Author | Ricardo Martínez-Flores |
  | Author | Felipe Barreto-Schuch |
  | Author | Rodrigo Yáñez-Sepúlveda |
  | Author | Pedro Delgado-Floody |
  | Author | Gerson Ferrari |
  | Author | Kabir P. Sadarangani |
  | Author | Jorge Cancino-López |
  | Author | Joao Bento-Torres |
  | Author | Alexis Espinoza-Salinas |
  | Author | Emmanuel Stamatakis |
  | Author | Carlos Cristi-Montero |
  | Abstract | OBJECTIVE: Traditional and non-traditional strategies have been employed to improve youth health. Dog-assisted interventions have been proposed as a novel strategy to regulate stress and its consequences across all age groups. This systematic review and multilevel meta-analysis assessed the influence of dog-assisted interventions on cortisol levels in youth and explored potential moderators. SOURCES: We conducted a comprehensive systematic search across multiple databases, including PubMed, Scopus, Web of Science, Cochrane Library, and ScienceDirect, up to June 17, 2024, to evaluate the impact of dog-assisted interventions on youth cortisol levels. Two reviewers independently extracted and verified data from eligible randomized clinical trials, with a third reviewer ensuring accuracy. Cochrane's RoB 2.0 tool was used to assess the risk of bias. Heterogeneity was analyzed using Q and I2 statistics. A random-effects model was employed to calculate effect sizes (ES) using R software. SUMMARY OF THE FINDINGS: Significant cortisol reductions were found for interventions lasting >15 min (ES: 0.65; p = .038), with a non-significant trend towards reduced cortisol in non-medical settings (ES: 0.46; p = .070). No significant effects were observed for shorter interventions, different control groups, or age-specific analyses. Meta-regression analysis revealed significant differences, showing better outcomes with longer intervention times and fewer female participants. CONCLUSION: Dog-assisted interventions lasting >15 min seem to be a promising and non-traditional strategy for regulating cortisol levels in children and adolescents in stressful situations. This study outlines gaps in the research and future directions. |
  | Date | 2025-03 |
  | Language | eng |
  | Library Catalogue | PubMed |
  | Volume | 369 |
  | Place | 1982 |
  | Pages | 117815 |
  | Publication | Social Science & Medicine |
  | DOI | 10.1016/j.socscimed.2025.117815 |
  | Journal Abbr | Soc Sci Med |
  | ISSN | 1873-5347 |
  | PMID | 39951871 |
  | Date Added | 05/02/2026, 16:20:09 |
  | Modified | 05/02/2026, 16:20:09 |

  ### Tags:

  - Hydrocortisone
  - Dogs
  - Animals
  - Female
  - Humans
  - Male
  - Animal Assisted Therapy
  - Stress, Psychological
  - Child
  - Stress
  - Adolescent
  - Animal-assisted therapy
  - Childhood
  - Health
  - Service dog

  ### Attachments

  - PubMed entry
- ## Heart Rate Variability Spectral Analysis for Monitoring Autonomic Activation in a Donkey Involved in Animal-Assisted Therapy: A Single Subject Design During Animal-Assisted Therapy Sessions

  |  |  |
  | --- | --- |
  | Item Type | Journal Article |
  | Author | Michele Panzera |
  | Author | Alessandra Statelli |
  | Abstract | Background: Only a limited number of studies have investigated objective indicators to assess donkey welfare during Animal-Assisted Services. Objective: The present research follows a single-subject design and its objective is to evaluate the neurovegetative indicators of the well-being of a donkey through spectral analysis of the R-R signal in the frequency domain. Methods: The experimental protocol of the Animal-Assisted Therapy project involved one donkey, previously selected through behavioral protocol evaluation, and ten patients with a diagnosis of paranoid schizophrenia. Spectral analysis of the R-R signal in the frequency domain was performed, providing objective data on the activity of the sympathetic and parasympathetic nervous systems of the donkey (before, during, and after the sessions). Results: The significance of the variations, both statistically significant and not, supports the hypothesis that the affiliative human-donkey interaction within the context of AAS is associated with modifications in the neurovegetative components of the donkey involved in AAT. Conclusions: These findings highlight the importance of objective and non-invasive monitoring tools to detect early signs of discomfort in donkeys involved in AAT, supporting the development of selection and management strategies that safeguard animal welfare. |
  | Date | 2025-11-28 |
  | Language | eng |
  | Short Title | Heart Rate Variability Spectral Analysis for Monitoring Autonomic Activation in a Donkey Involved in Animal-Assisted Therapy |
  | Library Catalogue | PubMed |
  | Volume | 12 |
  | Pages | 1131 |
  | Publication | Veterinary Sciences |
  | DOI | 10.3390/vetsci12121131 |
  | Issue | 12 |
  | Journal Abbr | Vet Sci |
  | ISSN | 2306-7381 |
  | PMID | 41472111 |
  | PMCID | PMC12737542 |
  | Date Added | 05/02/2026, 16:23:23 |
  | Modified | 05/02/2026, 16:23:23 |

  ### Tags:

  - human–animal interaction
  - animal welfare
  - AAS
  - donkeys

  ### Attachments

  - PubMed entry
- ## Companion Dog Foster Caregiver Program for Older Veterans at the VA Maryland Health Care System: A Feasibility Study

  |  |  |
  | --- | --- |
  | Item Type | Journal Article |
  | Author | Heidi K. Ortmeyer |
  | Author | Lynda C. Robey |
  | Abstract | Veterans experience mental health conditions at a disproportionate rate compared to their civilian counterparts, and approximately 60% of older veterans who receive their care through the United States Department of Veterans Affairs (VA) do not meet physical activity (PA) recommendations. We tested the Veterans as Foster Ambassadors program at the VA Maryland Health Care System to examine whether fostering a companion dog would improve PA and function, heart rate variability (HRV), balance, and quality of life (QOL) in older veterans. Participants wore an accelerometer for ≥10 days during each phase (30 day baseline vs. 60 day foster period) to measure daily PA (n = 4). Six-minute walk (6MW) and balance testing (n = 4) and 24 h heart rate (HR) and HRV (n = 2) were determined at baseline and during the foster period. Compared to baseline, there were significant increases in (a) distance during the 6MW, (b) daily steps, and (c) time spent in moderate activity during the foster period. 24 h HR decreased and time- and frequency-domain measures of HRV significantly increased in a veteran with post-traumatic stress disorder during the foster period compared to baseline. All veterans offered positive feedback about the program and indicated that it was beneficial to them. The results from this pilot study provide evidence that fostering a companion dog can improve PA, health, and QOL in older veterans. Future research conducted with a larger sample size to validate the results is warranted. |
  | Date | 2019-11-04 |
  | Language | eng |
  | Short Title | Companion Dog Foster Caregiver Program for Older Veterans at the VA Maryland Health Care System |
  | Library Catalogue | PubMed |
  | Volume | 16 |
  | Pages | 4285 |
  | Publication | International Journal of Environmental Research and Public Health |
  | DOI | 10.3390/ijerph16214285 |
  | Issue | 21 |
  | Journal Abbr | Int J Environ Res Public Health |
  | ISSN | 1660-4601 |
  | PMID | 31690056 |
  | PMCID | PMC6861960 |
  | Date Added | 05/02/2026, 16:23:23 |
  | Modified | 05/02/2026, 16:23:23 |

  ### Tags:

  - Dogs
  - veteran
  - Animals
  - Human-Animal Bond
  - Humans
  - Aged
  - Animal Assisted Therapy
  - heart rate variability
  - physical activity
  - United States
  - Veterans
  - Feasibility Studies
  - Pilot Projects
  - Quality of Life
  - Caregivers
  - Program Evaluation
  - Persons with Disabilities
  - United States Department of Veterans Affairs
  - accelerometry
  - companion dog

  ### Attachments

  - Full Text
  - PubMed entry
- ## Psychological Evaluation of Animal-assisted Intervention (AAI) Programs Involving Visiting Dogs and Cats for Alcohol Dependents: A Pilot Study

  |  |  |
  | --- | --- |
  | Item Type | Journal Article |
  | Author | Nobuyo Ohtani |
  | Author | Shin Narita |
  | Author | Eiji Yoshihara |
  | Author | Mitsuaki Ohta |
  | Author | Kazuhiko Iwahashi |
  | Abstract | The purpose of this study was to develop an evaluation method for animal-assisted intervention (AAI) programs involving Mood Check List-Short form.2 (MCL-S.2) and the State-Trait Anxiety Inventory (STAI) for psychiatric daycare of Japanese alcohol. dependents. A total of 36 alcohol dependents completed the study and questionnaires assessing their state. A single session of AAI reduced both subjective and physiological measures of state anxiety (A-State); and this program induced a significant reduction in the anxiety after an AAI program session with the dogs and cats involved in the intervention (p = 0.001). The Wilcoxon t-test showed that there were also significant differences in the "anxiety", "pleasantness", and "relaxation". scores for MCL-S.2 among the alcohol dependents, before and after AAI; a significantly decreased "anxiety" score (p = 0.006), and increased "pleasantness" (p = 0.002) and "relaxation" (p=0.012) scores for MCL-S.2 after AAI. The results of this study indicated that alcohol dependents who experienced a group AAI session-program exhibited significant improvements in their feeling; decreased anxiety, and increased pleasantness and relaxation. |
  | Date | 2015-12 |
  | Language | eng |
  | Short Title | Psychological Evaluation of Animal-assisted Intervention (AAI) Programs Involving Visiting Dogs and Cats for Alcohol Dependents |
  | Library Catalogue | PubMed |
  | Volume | 50 |
  | Pages | 289-295 |
  | Publication | Nihon Arukoru Yakubutsu Igakkai Zasshi = Japanese Journal of Alcohol Studies & Drug Dependence |
  | Issue | 6 |
  | Journal Abbr | Nihon Arukoru Yakubutsu Igakkai Zasshi |
  | ISSN | 1341-8963 |
  | PMID | 26964290 |
  | Date Added | 05/02/2026, 16:23:23 |
  | Modified | 05/02/2026, 16:23:23 |

  ### Tags:

  - Dogs
  - Relaxation
  - Animals
  - Female
  - Adult
  - Humans
  - Male
  - Middle Aged
  - Animal Assisted Therapy
  - Cats
  - Anxiety
  - Pilot Projects
  - Alcoholism

  ### Attachments

  - PubMed entry
- ## Animal-assisted therapy - magic or medicine?

  |  |  |
  | --- | --- |
  | Item Type | Journal Article |
  | Author | J. S. Odendaal |
  | Abstract | A sound theoretical basis supported by scientifically measured physiological parameters is needed to gain medical support for animal-assisted therapy. Six neurochemicals associated with a decrease in blood pressure were measured in humans (n=18) and dogs (n=18) before and after positive interaction. Results (P<.05) indicated that in both species the neurochemicals involved with attention-seeking or attentionis egens behavior have increased. This information can be used as a rationale for animal-assisted therapy. |
  | Date | 2000-10 |
  | Language | eng |
  | Library Catalogue | PubMed |
  | Volume | 49 |
  | Pages | 275-280 |
  | Publication | Journal of Psychosomatic Research |
  | DOI | 10.1016/s0022-3999(00)00183-5 |
  | Issue | 4 |
  | Journal Abbr | J Psychosom Res |
  | ISSN | 0022-3999 |
  | PMID | 11119784 |
  | Date Added | 05/02/2026, 16:20:09 |
  | Modified | 05/02/2026, 16:20:09 |

  ### Tags:

  - Hydrocortisone
  - Oxytocin
  - Dogs
  - Animals
  - Human-Animal Bond
  - Humans
  - Emotions
  - beta-Endorphin
  - Case-Control Studies
  - Dopamine
  - Love
  - Prolactin
  - Psychotherapy
  - Reading

  ### Attachments

  - PubMed entry
- ## [Agility in treatment of children with type 1 diabetes--pilot study]

  |  |  |
  | --- | --- |
  | Item Type | Journal Article |
  | Author | Monika Niewiadomska |
  | Author | Marija Radziejewska |
  | Author | Anita Horodnicka-Józwa |
  | Author | Elzbieta Petriczko |
  | Abstract | INTRODUCTION: Physical activity is a very important element in treatment of children with type 1 diabetes. However, it is difficult to find suitable exercises for the children due to their specific needs and psychophysiological condition. The aim of this study was to examine the effects of agility as a physical activity used to improve parameters of metabolic control in children with type 1 diabetes. Additionally, we hope that this form of recreation could induce a more health-oriented behavior in children. MATERIAL AND METHODS: The experimental group consisted of seven girls aged 8-10 years, the patients of the Clinic of Pediatrics, Endocrinology, Diabetology, Metabolic Disorders and Cardiology of the Developmental Age in Szczecin. The children were qualified for the study after the prior assessment of their metabolic control under the conditions of the one-day hospital stay program. The physical condition of the patients was controlled with a 6-minute walk test and the test of perceived exertion (Borg scale). All the patients were treated using a personal insulin pump and the basal-bolus therapy. The applied research method used the scheme of physical exercise performed 3 times a week, 45 min each, reaching the intensity of 75% of the maximum heart rate under effort performed by a given patient. Before the exercises and directly after their completion, sugar level was measured in the blood of the patients (Accu-chek Active). During the exercises, the heart rate was measured with a pulsometer Bauer PM 80. The exercises included outdoor games and agility sessions. Especially the latter received a positive response and high involvement. This form includes a dog going through an obstacle course, with a child as a guide. RESULTS AND CONCLUSIONS: After three months of the exercise scheme, the analysis of the collected results showed a statistically significant (p <0.05) decrease in the insulin doses (bolus) during morning hours (7-8 am), and in the evening at 9 pm and 10.30 pm, with an unchanged basal. No such decrease was observed in the control group. This study showed that an interesting physical activity resulted in a more eager and systematic effort among examined diabetic children. Its proper organization in time may help in the metabolic control in children with type 1 diabetes. |
  | Date | 2010 |
  | Language | pol |
  | Library Catalogue | PubMed |
  | Volume | 16 |
  | Pages | 89-93 |
  | Publication | Pediatric Endocrinology, Diabetes, and Metabolism |
  | Issue | 2 |
  | Journal Abbr | Pediatr Endocrinol Diabetes Metab |
  | ISSN | 2081-237X |
  | PMID | 20813085 |
  | Date Added | 05/02/2026, 16:23:23 |
  | Modified | 05/02/2026, 16:23:23 |

  ### Tags:

  - Dogs
  - Animals
  - Female
  - Humans
  - Animal Assisted Therapy
  - Child
  - Pilot Projects
  - Diabetes Mellitus, Type 1
  - Exercise
  - Exercise Therapy
  - Insulin

  ### Attachments

  - PubMed entry
- ## Survey of international academic centers and institutes focused on human-animal bond: Scope and landscape in 2021

  |  |  |
  | --- | --- |
  | Item Type | Journal Article |
  | Author | Leanne O. Nieforth |
  | Author | Sarah C. Leighton |
  | Author | Elise A. Miller |
  | Author | Marguerite E. O'Haire |
  | Abstract | Routine surveying of academic centers focused on the human-animal bond is critical to understand the trajectory of the field and to create an environment where centers can learn from one another and build collaborations. The purpose of this manuscript was to report the findings of a survey of these human-animal bond centers, to summarize the status of the field, and to identify changes within the field since 2016. Survey questions concentrated on the demographic characteristics, engagement programs, educational opportunities, and research focuses of the centers. Findings suggest that the field continues to grow as one-third of human-animal bond centers are less than 10 years old. The number of centers that participated in this survey increased by 31% compared to the previous survey (O'Haire et al., 2018). Centers have developed a variety of engagement programs, including animal-assisted intervention and companion animal education programs. About half of the centers (48%) offer degree programs and about one quarter of the centers (24%) offer certificate programs. Most centers (95%) focus their research on companion animals with the most studied companion animal being dogs (95%). The most frequent data collection method was surveys (86%). Qualitative analyses, behavior measures, and physiological measures were also common. The most notable changes from the 2016 survey include overall growth of the field (indicated by the establishment of new centers) and a shift in the specialty area of directors, moving from 44% of directors being veterinarians in 2016 survey to 90% having a human-focused specialty in the 2021 survey. Most centers' research focused on animal-assisted interventions which is consistent with the previous survey. As the field of the human-animal bond continues to grow and more centers emerge, ongoing evaluation of offerings is important to track changes, identify needs, and foster success. |
  | Date | 2022-12 |
  | Language | eng |
  | Short Title | Survey of international academic centers and institutes focused on human-animal bond |
  | Library Catalogue | PubMed |
  | Volume | 2022 |
  | Publication | Human-Animal Interactions |
  | DOI | 10.1079/hai.2022.0026 |
  | Journal Abbr | Hum Anim Interact |
  | ISSN | 2957-9538 |
  | PMID | 38894882 |
  | PMCID | PMC11184414 |
  | Date Added | 05/02/2026, 16:23:23 |
  | Modified | 05/02/2026, 16:23:23 |

  ### Attachments

  - Full Text PDF
  - PubMed entry
- ## Animal-Assisted Activity: Effects of a Complementary Intervention Program on Psychological and Physiological Variables

  |  |  |
  | --- | --- |
  | Item Type | Journal Article |
  | Author | Peggy Nepps |
  | Author | Charles N. Stewart |
  | Author | Stephen R. Bruckno |
  | Abstract | Animal-assisted activity is the use of trained animals for the therapeutic, motivational, or educational benefit of patients. Subjects of this study were 218 patients hospitalized on the mental health unit of a community hospital with an existing, complementary animal-assisted activity program. Half of the patients participated in a 1-hour session of animal-assisted activity. The other half, who served as a comparison group, participated in a 1-hour stress management program. It was hypothesized that an animal-assisted activity program would improve ratings of depression, anxiety, and pain and the associated physiological measures of stress and discomfort. Self-report ratings of depression, anxiety, and pain were collected before and after treatment sessions, and blood pressure, pulse, and salivary cortisol were measured. There were significant decreases in depression (P < .0001), anxiety (P < .0001), pain (P < .0001), and pulse (P < .04) after animal-assisted activity program, comparable to those in the more traditional stress management group. |
  | Date | 2014-07 |
  | Language | eng |
  | Short Title | Animal-Assisted Activity |
  | Library Catalogue | PubMed |
  | Volume | 19 |
  | Pages | 211-215 |
  | Publication | Journal of Evidence-Based Complementary & Alternative Medicine |
  | DOI | 10.1177/2156587214533570 |
  | Issue | 3 |
  | Journal Abbr | J Evid Based Complementary Altern Med |
  | ISSN | 2156-5899 |
  | PMID | 24789913 |
  | Date Added | 05/02/2026, 16:20:09 |
  | Modified | 05/02/2026, 16:20:09 |

  ### Tags:

  - animal-assisted therapy
  - animal-assisted intervention
  - anthrozoology

  ### Attachments

  - Full Text
  - PubMed entry
- ## The Importance of Evaluating Positive Welfare Characteristics and Temperament in Working Therapy Dogs

  |  |  |
  | --- | --- |
  | Item Type | Journal Article |
  | Author | Sharmaine L. Miller |
  | Author | James A. Serpell |
  | Author | Kathryn R. Dalton |
  | Author | Kaitlin B. Waite |
  | Author | Daniel O. Morris |
  | Author | Laurel E. Redding |
  | Author | Nancy A. Dreschel |
  | Author | Meghan F. Davis |
  | Abstract | To date, investigations of the welfare of therapy dogs have focused largely on examining physiological and behavioral measures that could indicate if the animal is experiencing stress or distress. However, this approach does not fully address the definition of welfare which is often described as existing on a continuum from negative (or stressful) to positive. With therapy dogs, it would be worth addressing if they experience positive emotional affect while working since the quality and efficacy of animal-assisted interventions for the human recipient is likely to be influenced by the animal's emotional state during the interaction. The purpose of this review is to articulate how objective measurements of the HPA axis and measurements of behavioral observations and standardized questions can be used to evaluate positive welfare in therapy dogs. A potentially relevant indicator of positive welfare is the peripheral concentration of the neurohormone oxytocin, which has been found to increase in systemic circulation within a variety of species during positive social and affiliative contexts, including during human-dog interaction. Oxytocin is also a negative-feedback regulator of the Hypothalamic-Pituitary-Adrenal (HPA) axis, which culminates with the production of the stress hormone cortisol. Cortisol is widely used as a physiological indicator to assess negative welfare states in animals, including therapy dogs. Observable behavior during interactions with humans that may convey enjoyment could provide indicators of positive welfare in dogs such as engagement in play, or human-directed affiliative behaviors including leaning against, nudging, or licking the patient. However, in assessing positive welfare, it is also critical to consider that all animal behavioral displays and physiological responses are dependent on the dog's individual (and breed) temperament. Temperament directly drives how the animal copes and responds to its current physical and social environment, including during stressful situations such as when therapy dogs interact with unfamiliar humans in novel healthcare settings. Coupled with both positive and negative physiological and behavioral welfare indicators, questionnaire data can provide further context to, and enhance interpretations of, therapy dog welfare assessment results. Overall, to date, no studies have measured all of these factors to assess therapy dog welfare. |
  | Date | 2022 |
  | Language | eng |
  | Library Catalogue | PubMed |
  | Volume | 9 |
  | Pages | 844252 |
  | Publication | Frontiers in Veterinary Science |
  | DOI | 10.3389/fvets.2022.844252 |
  | Journal Abbr | Front Vet Sci |
  | ISSN | 2297-1769 |
  | PMID | 35445102 |
  | PMCID | PMC9014261 |
  | Date Added | 05/02/2026, 16:20:09 |
  | Modified | 05/02/2026, 16:20:09 |

  ### Tags:

  - cortisol
  - oxytocin
  - Animal-Assisted Intervention (AAI)
  - Animal-Assisted Therapy (AAT)
  - dog behavior
  - human-dog interaction
  - positive welfare
  - therapy dog welfare

  ### Attachments

  - Full Text
  - PubMed entry
- ## Efficacy of animal-assisted therapy adapted to reality orientation therapy: measurement of salivary cortisol

  |  |  |
  | --- | --- |
  | Item Type | Journal Article |
  | Author | Lucia Francesca Menna |
  | Author | Antonio Santaniello |
  | Author | Federica Gerardi |
  | Author | Mario Sansone |
  | Author | Annamaria Di Maggio |
  | Author | Annalisa Di Palma |
  | Author | Giuseppe Perruolo |
  | Author | Vittoria D'Esposito |
  | Author | Pietro Formisano |
  | Date | 2019-09 |
  | Language | eng |
  | Short Title | Efficacy of animal-assisted therapy adapted to reality orientation therapy |
  | Library Catalogue | PubMed |
  | Volume | 19 |
  | Pages | 510-512 |
  | Publication | Psychogeriatrics: The Official Journal of the Japanese Psychogeriatric Society |
  | DOI | 10.1111/psyg.12418 |
  | Issue | 5 |
  | Journal Abbr | Psychogeriatrics |
  | ISSN | 1479-8301 |
  | PMID | 30740833 |
  | Date Added | 05/02/2026, 16:23:23 |
  | Modified | 05/02/2026, 16:23:23 |

  ### Tags:

  - Hydrocortisone
  - Animals
  - Female
  - Humans
  - Male
  - Aged
  - Animal Assisted Therapy
  - Saliva
  - Biomarkers
  - Treatment Outcome
  - Aged, 80 and over
  - Alzheimer Disease
  - Reality Therapy

  ### Attachments

  - PubMed entry
- ## Investigation of Physiological and Behavioral Responses in Dogs Participating in Animal-Assisted Therapy with Children Diagnosed with Attention-Deficit Hyperactivity Disorder

  |  |  |
  | --- | --- |
  | Item Type | Journal Article |
  | Author | Ashley L. Melco |
  | Author | Larry Goldman |
  | Author | Aubrey H. Fine |
  | Author | Jose M. Peralta |
  | Abstract | This study evaluated the impact that participation in sessions with children with Attention-Deficit Hyperactivity Disorder (ADHD) has on therapy dogs. Nine certified therapy dogs were paired for 6 sessions with groups of 3-4 children. Sessions consisted of 5 different activities. Activities 1 and 5 involved interactions solely with each dog and their owner, as a control. Activities 2-4 consisted of interactions with the dogs and the children which included social skills training, dog training, and reading in the company of dogs. One-zero interval sampling of stress-associated behaviors was conducted at 20-second intervals for a 10-minute duration during each of the 5 activities. At the end of each activity, heart rate was monitored, and a saliva sample was obtained for cortisol analysis. Dogs demonstrated only occasional behavioral responses and no significant findings related to cortisol or heart rate when the different activities were compared. The results indicate that with proper supervision and well-trained therapy staff, including suitable therapy dogs and their handlers, canine stress can be minimal in a therapy setting. |
  | Date | 2020 |
  | Language | eng |
  | Library Catalogue | PubMed |
  | Volume | 23 |
  | Pages | 10-28 |
  | Publication | Journal of applied animal welfare science: JAAWS |
  | DOI | 10.1080/10888705.2018.1536979 |
  | Issue | 1 |
  | Journal Abbr | J Appl Anim Welf Sci |
  | ISSN | 1532-7604 |
  | PMID | 30376724 |
  | Date Added | 05/02/2026, 16:20:09 |
  | Modified | 05/02/2026, 16:20:09 |

  ### Tags:

  - cortisol
  - Hydrocortisone
  - Dogs
  - stress
  - Animals
  - Behavior, Animal
  - Female
  - Adult
  - Heart Rate
  - Humans
  - Male
  - Animal Assisted Therapy
  - Stress, Psychological
  - behavior
  - Saliva
  - Child
  - dog
  - Animal Welfare
  - Animal-assisted therapy
  - Attention Deficit Disorder with Hyperactivity

  ### Attachments

  - PubMed entry
- ## Measuring the Effects of an Animal-Assisted Intervention for Pediatric Oncology Patients and Their Parents: A Multisite Randomized Controlled Trial [Formula: see text]

  |  |  |
  | --- | --- |
  | Item Type | Journal Article |
  | Author | Amy McCullough |
  | Author | Ashleigh Ruehrdanz |
  | Author | Molly A. Jenkins |
  | Author | Mary Jo Gilmer |
  | Author | Janice Olson |
  | Author | Anjali Pawar |
  | Author | Leslie Holley |
  | Author | Shirley Sierra-Rivera |
  | Author | Deborah E. Linder |
  | Author | Danielle Pichette |
  | Author | Neil J. Grossman |
  | Author | Cynthia Hellman |
  | Author | Noémi A. Guérin |
  | Author | Marguerite E. O'Haire |
  | Abstract | OBJECTIVE: This multicenter, parallel-group, randomized trial examined the effects of an animal-assisted intervention on the stress, anxiety, and health-related quality of life for children diagnosed with cancer and their parents. METHOD: Newly diagnosed patients, aged 3 to 17 years (n = 106), were randomized to receive either standard care plus regular visits from a therapy dog (intervention group), or standard care only (control group). Data were collected at set points over 4 months of the child's treatment. Measures included the State-Trait Anxiety Inventory™, Pediatric Quality of Life Inventory, Pediatric Inventory for Parents, and child blood pressure and heart rate. All instruments were completed by the child and/or his/her parent(s). RESULTS: Children in both groups experienced a significant reduction in state anxiety ( P < .001). Parents in the intervention group showed significantly decreased parenting stress ( P = .008), with no changes in stress among parents in the control group. However, no significant differences between groups over time on any measures were observed. CONCLUSIONS: Animal-assisted interventions may provide certain benefits for parents and families during the initial stages of pediatric cancer treatment. |
  | Date | 2018-05 |
  | Language | eng |
  | Short Title | Measuring the Effects of an Animal-Assisted Intervention for Pediatric Oncology Patients and Their Parents |
  | Library Catalogue | PubMed |
  | Volume | 35 |
  | Pages | 159-177 |
  | Publication | Journal of Pediatric Oncology Nursing: Official Journal of the Association of Pediatric Oncology Nurses |
  | DOI | 10.1177/1043454217748586 |
  | Issue | 3 |
  | Journal Abbr | J Pediatr Oncol Nurs |
  | ISSN | 1532-8457 |
  | PMID | 29268667 |
  | Date Added | 05/02/2026, 16:20:09 |
  | Modified | 05/02/2026, 16:20:09 |

  ### Tags:

  - stress
  - Female
  - Adult
  - Humans
  - Male
  - Animal Assisted Therapy
  - Stress, Psychological
  - Child
  - Child, Preschool
  - animal-assisted intervention
  - Adolescent
  - Anxiety
  - Neoplasms
  - Quality of Life
  - health-related quality of life
  - parent
  - Parents
  - Patients
  - pediatric oncology

  ### Attachments

  - PubMed entry
- ## Pet ownership and physical health

  |  |  |
  | --- | --- |
  | Item Type | Journal Article |
  | Author | Robert L. Matchock |
  | Abstract | PURPOSE OF REVIEW: Pet ownership and brief human-animal interactions can serve as a form of social support and convey a host of beneficial psychological and physiological health benefits. This article critically examines recent relevant literature on the pet-health connection. RECENT FINDINGS: Cross-sectional studies indicate correlations between pet ownership and numerous aspects of positive health outcomes, including improvements on cardiovascular measures and decreases in loneliness. Quasi-experimental studies and better controlled experimental studies corroborate these associations and suggest that owning and/or interacting with a pet may be causally related to some positive health outcomes. SUMMARY: The value of pet ownership and animal-assisted therapy (AAT), as a nonpharmacological treatment modality, augmentation to traditional treatment, and healthy preventive behavior (in the case of pet ownership), is starting to be realized. However, more investigations that employ randomized controlled trials with larger sample sizes and investigations that more closely examine the underlying mechanism of the pet-health effect, such as oxytocin, are needed. |
  | Date | 2015-09 |
  | Language | eng |
  | Library Catalogue | PubMed |
  | Volume | 28 |
  | Pages | 386-392 |
  | Publication | Current Opinion in Psychiatry |
  | DOI | 10.1097/YCO.0000000000000183 |
  | Issue | 5 |
  | Journal Abbr | Curr Opin Psychiatry |
  | ISSN | 1473-6578 |
  | PMID | 26164613 |
  | Date Added | 05/02/2026, 16:20:09 |
  | Modified | 05/02/2026, 16:20:09 |

  ### Tags:

  - Pets
  - Animals
  - Human-Animal Bond
  - Humans
  - Animal Assisted Therapy
  - Loneliness
  - Health Status
  - Mental Disorders
  - Ownership

  ### Attachments

  - PubMed entry
- ## Evaluating effects of animal-assisted therapy on pediatric dental care patients: A pilot clinical trial

  |  |  |
  | --- | --- |
  | Item Type | Journal Article |
  | Author | Jacqueline Massouda |
  | Author | Nare Ghaltakhchyan |
  | Author | Jennifer Judd |
  | Author | Clare Bocklage |
  | Author | Raven Selden |
  | Author | Olivia TumSuden |
  | Author | Eleanor Nanney |
  | Author | Jessica Lee |
  | Author | Jeannie Ginnis |
  | Author | Timothy Strauman |
  | Author | Caroline Sawicki |
  | Author | Eric A. Hodges |
  | Author | Christina Graves |
  | Author | Kimon Divaris |
  | Author | Laura Jacox |
  | Abstract | BACKGROUND: An estimated 6% through 22% of children have dental anxiety, which can contribute to disruptive behavior and oral health care avoidance. Evidence from medical settings indicate reductions in pain and stress after therapy dog implementation. To identify a low-risk, nonpharmacologic approach for anxiety and pain management in dentistry, a pilot prospective clinical trial was conducted to determine best practices for evaluating the efficacy of animal-assisted therapy (AAT). METHODS: The effects of AAT on pediatric patients were measured through physiological, objective measures (ie, heart rate, salivary cortisol and α-amylase, and video coding) and validated self-reported scales of anxiety, fear, and pain, during an invasive dental procedure. Children aged 7 through 14 years were consecutively enrolled into an AAT (n = 18) or control (n = 21) group. Participants underwent an operative or surgical (eg, extraction) dental procedure. Descriptive and bivariate statistics were used. RESULTS: Participants in the AAT group reported significantly less postoperative pain than those in the control group (P = .001). The heart rates of AAT participants dropped after key events and had less variation than control participants. Objective video coding revealed that AAT participants had significantly longer durations of relaxed lower bodies than control participants (P = .204). No differences were seen in salivary cortisol and α-amylase. These physiological and self-report measures are feasible for use in future studies. CONCLUSIONS: AAT may be an effective therapy for alleviating anxiety and pain in pediatric dental patients and warrants additional study using both objective physiological end points and subjective self-report measures. PRACTICAL IMPLICATIONS: Pending further research, canine therapy may be a valuable addition to dental clinics for anxiety and pain management. This clinical trial was registered at ClinicalTrials.gov. The registration number is NCT04708028. |
  | Date | 2025-06 |
  | Language | eng |
  | Short Title | Evaluating effects of animal-assisted therapy on pediatric dental care patients |
  | Library Catalogue | PubMed |
  | Volume | 156 |
  | Place | 1939 |
  | Pages | 447-457.e14 |
  | Publication | Journal of the American Dental Association |
  | DOI | 10.1016/j.adaj.2025.03.006 |
  | Issue | 6 |
  | Journal Abbr | J Am Dent Assoc |
  | ISSN | 1943-4723 |
  | PMID | 40467121 |
  | PMCID | PMC12356166 |
  | Date Added | 05/02/2026, 16:20:09 |
  | Modified | 05/02/2026, 16:20:09 |

  ### Tags:

  - Hydrocortisone
  - Dogs
  - Animals
  - Female
  - Heart Rate
  - Humans
  - Male
  - Animal Assisted Therapy
  - Saliva
  - Child
  - Adolescent
  - Pain Management
  - Dental Anxiety
  - Pilot Projects
  - Pain Measurement
  - Dental anxiety
  - Dental Care for Children
  - Prospective Studies
  - alpha-Amylases
  - animal therapy
  - pediatric dentistry

  ### Attachments

  - PubMed entry
- ## Effects of contact with a dog on prefrontal brain activation in patients in a minimally conscious state: A controlled crossover trial

  |  |  |
  | --- | --- |
  | Item Type | Journal Article |
  | Author | Rahel Marti |
  | Author | Milena Petignat |
  | Author | Valentine L. Marcar |
  | Author | Jan Hattendorf |
  | Author | Martin Wolf |
  | Author | Margret Hund-Georgiadis |
  | Author | Karin Hediger |
  | Abstract | The first studies have indicated that animal-assisted therapy benefits patients in a minimally conscious state (MCS), but the evidence is scarce. It is thus crucial to understand how these patients react to animal contact. This study aimed to measure the prefrontal brain activation in MCS patients during contact with a dog compared with a plush animal using functional near-infrared spectroscopy (fNIRS). We conducted a controlled crossover trial with 22 MCS patients, who each participated in six sessions. Patients interacted with a dog in three sessions and with a plush animal in three control sessions. Each session consisted of five 2-minute phases with a neutral phase at the start and the end. The contact intensity with the dog or the plush animal increased from the second to the fourth phase. The fNIRS parameters did not differ between the conditions. The mean heart rate was significantly higher in the dog condition than in the control. In both conditions, prefrontal brain activation, mean heart rate, and one heart rate variability parameter increased with the increased intensity of contact with the dog and plush animal. The results show that MCS patients react with the same prefrontal brain activation during contact with a dog and a plush animal but have increased heart rate in contact with the dog, indicating physiological arousal. These findings suggest that the incorporation of animals into MCS therapy has the potential to stimulate patients, thereby facilitating greater participation. However, more research is needed to understand the effects of animals on brain activation. |
  | Date | 2025-06-21 |
  | Language | eng |
  | Short Title | Effects of contact with a dog on prefrontal brain activation in patients in a minimally conscious state |
  | Library Catalogue | PubMed |
  | Volume | 577 |
  | Pages | 175-189 |
  | Publication | Neuroscience |
  | DOI | 10.1016/j.neuroscience.2025.05.014 |
  | Journal Abbr | Neuroscience |
  | ISSN | 1873-7544 |
  | PMID | 40360130 |
  | Date Added | 05/02/2026, 16:23:23 |
  | Modified | 05/02/2026, 16:23:23 |

  ### Tags:

  - Brain activation
  - Functional near-infrared spectroscopy
  - Heart rate
  - Human–animal interaction
  - Minimally conscious state
  - Neurorehabilitation
  - Dogs
  - Animals
  - Female
  - Adult
  - Heart Rate
  - Humans
  - Male
  - Middle Aged
  - Aged
  - Animal Assisted Therapy
  - Cross-Over Studies
  - Persistent Vegetative State
  - Prefrontal Cortex
  - Spectroscopy, Near-Infrared

  ### Attachments

  - PubMed entry
- ## Canine-Assisted Therapy Improves Well-Being in Nurses

  |  |  |
  | --- | --- |
  | Item Type | Journal Article |
  | Author | Kristýna Machová |
  | Author | Michaela Součková |
  | Author | Radka Procházková |
  | Author | Zdislava Vaníčková |
  | Author | Kamal Mezian |
  | Abstract | As nursing is one of the most stressful occupations worldwide, its management warrants more attention to identify possible ways to cope with its pressures. This study aims to evaluate whether animal-assisted therapy (AAT) with the presence of a dog affects the stress level of nurses. As a stress biomarker, we used salivary cortisol level testing. Twenty female nurses (mean age: 30) in physical medicine (PMR) (n = 11) and the department of internal medicine and long-term care (IM < C) (n = 9). On each of the three observed days, saliva was collected at 10 a.m. and then again after 50 min. The first sampling was performed during a normal working process without a break (Condition A), the second was carried out during a normal working process with a break of choice (Condition B), and the third sampling was performed during a normal working process with a break with AAT (Condition C). All participants were enrolled in all three interventional conditions in a randomized order. The results demonstrated the effect of a reduction of cortisol levels in Condition C, where AAT was included (p = 0.02) only in nurses recruited from the IM < C department. By way of explanation, nurses from the PMR department already showed low cortisol levels at baseline. We propose including AAT with a dog in healthcare facilities where nurses are at a high risk of stress. |
  | Date | 2019-09-30 |
  | Language | eng |
  | Library Catalogue | PubMed |
  | Volume | 16 |
  | Pages | 3670 |
  | Publication | International Journal of Environmental Research and Public Health |
  | DOI | 10.3390/ijerph16193670 |
  | Issue | 19 |
  | Journal Abbr | Int J Environ Res Public Health |
  | ISSN | 1660-4601 |
  | PMID | 31574899 |
  | PMCID | PMC6801790 |
  | Date Added | 05/02/2026, 16:20:09 |
  | Modified | 05/02/2026, 16:20:09 |

  ### Tags:

  - cortisol
  - Hydrocortisone
  - Dogs
  - stress
  - Animals
  - Female
  - Adult
  - Humans
  - Animal Assisted Therapy
  - animal-assisted therapy
  - Saliva
  - Occupational Stress
  - Czech Republic
  - dog-assisted therapy
  - Healthcare providers
  - Hospitals, Military
  - Nurses

  ### Attachments

  - Full Text
  - PubMed entry
- ## Effect of Animal-Assisted Therapy on Patients in the Department of Long-Term Care: A Pilot Study

  |  |  |
  | --- | --- |
  | Item Type | Journal Article |
  | Author | Kristýna Machová |
  | Author | Radka Procházková |
  | Author | Petra Eretová |
  | Author | Ivona Svobodová |
  | Author | Ilja Kotík |
  | Abstract | Long-term hospital stays might have a negative psychosocial impact on our patients. One way to positively activate hospitalized patients is to introduce animal-assisted therapy (AAT). A total of 72 individuals participated in this research. The experimental group comprised 33 patients (8 males, 25 females), while the control group contained 39 patients (11 men, 28 women). The participants in the control group were aged from 58 to 100 years and the experimental group featured participants aged from 51 to 95, for whom AAT was included alongside standard care. Blood pressure, heart rate, Barthel index, and general mood were measured in both groups. Results did not reveal any changes in blood pressure, heart rate, or Barthel index in comparison between groups. A great influence was noted in assessment of the mood of the patients. The inclusion of AAT did not affect physiological parameters, but it exerted a significant effect on the psychological well-being of the patients. |
  | Date | 2019-04-16 |
  | Language | eng |
  | Short Title | Effect of Animal-Assisted Therapy on Patients in the Department of Long-Term Care |
  | Library Catalogue | PubMed |
  | Volume | 16 |
  | Pages | 1362 |
  | Publication | International Journal of Environmental Research and Public Health |
  | DOI | 10.3390/ijerph16081362 |
  | Issue | 8 |
  | Journal Abbr | Int J Environ Res Public Health |
  | ISSN | 1660-4601 |
  | PMID | 31014022 |
  | PMCID | PMC6518374 |
  | Date Added | 05/02/2026, 16:20:09 |
  | Modified | 05/02/2026, 16:20:09 |

  ### Tags:

  - Female
  - Heart Rate
  - Humans
  - Male
  - Middle Aged
  - Aged
  - Animal Assisted Therapy
  - animal-assisted therapy
  - dog
  - elderly
  - Czech Republic
  - Aged, 80 and over
  - Pilot Projects
  - Affect
  - long-term care
  - Long-Term Care
  - well-being

  ### Attachments

  - Full Text
  - PubMed entry
- ## The Effect of Animal-Assisted Therapy on the State of Patients' Health After a Stroke: A Pilot Study

  |  |  |
  | --- | --- |
  | Item Type | Journal Article |
  | Author | Kristýna Machová |
  | Author | Radka Procházková |
  | Author | Michal Říha |
  | Author | Ivona Svobodová |
  | Abstract | A stroke is a condition that can give rise to consequences such as cognitive and physical constraints, which sometimes manifest in the psychological condition of the patient. Such patients commence rehabilitation as soon as is possible, which involves a multi-disciplinary approach to treatment. One aspect of complementary rehabilitation could be animal-assisted therapy (AAT). A total of 15 individuals were split into an experimental group comprising 6 patients (2 males, 4 females), and a control group of 9 patients (3 males, 6 females). The participants in the control group were aged from 43 to 87 years and the experimental group featured participants aged from 45 to 76 years. Both groups received standard physiotherapy and occupational therapy. In addition, the experimental group was supplemented with AAT, with the animal in question being a dog. The tools primarily applied to measure the outcomes were the Barthel index, blood pressure, and heart rate measurements, whereas the Likert scale was employed to discern the mood of the patients. The results showed that changes in the values for heart rate and blood pressure were insignificant. However, a statistically significant aspect of the research pertained to the patients confirming that they felt better after the AAT sessions. Hence, AAT could potentially bolster the effectiveness of other therapies. |
  | Date | 2019-09-06 |
  | Language | eng |
  | Short Title | The Effect of Animal-Assisted Therapy on the State of Patients' Health After a Stroke |
  | Library Catalogue | PubMed |
  | Volume | 16 |
  | Pages | 3272 |
  | Publication | International Journal of Environmental Research and Public Health |
  | DOI | 10.3390/ijerph16183272 |
  | Issue | 18 |
  | Journal Abbr | Int J Environ Res Public Health |
  | ISSN | 1660-4601 |
  | PMID | 31489875 |
  | PMCID | PMC6765888 |
  | Date Added | 05/02/2026, 16:20:09 |
  | Modified | 05/02/2026, 16:20:09 |

  ### Tags:

  - Dogs
  - Animals
  - Female
  - Heart Rate
  - Humans
  - Male
  - Middle Aged
  - Aged
  - Animal Assisted Therapy
  - dog
  - animal assisted therapy
  - Aged, 80 and over
  - Pilot Projects
  - Affect
  - Occupational Therapy
  - Physical Therapy Modalities
  - rehabilitation
  - stroke
  - Stroke
  - Stroke Rehabilitation

  ### Attachments

  - Full Text
  - PubMed entry
- ## Effect of Dog Presence on Stress Levels in Students under Psychological Strain: A Pilot Study

  |  |  |
  | --- | --- |
  | Item Type | Journal Article |
  | Author | Kristýna Machová |
  | Author | Radka Procházková |
  | Author | Mariana Vadroňová |
  | Author | Michaela Součková |
  | Author | Eliška Prouzová |
  | Abstract | As university students face many stressful situations, especially during the examination period, this study focused on the use of animal-assisted activities (AAAs) with a dog as a means of relieving students' stress before a final exam. The aim was to determine whether a 10-min interaction with a dog affected subjectively evaluated stress and mood, objective blood pressure, and heart rate. Ninety-three female students (mean age = 22.5 years; standard deviation = 3.8 years) were divided into three groups according to their preference. The first group underwent AAAs (n = 26), the second group chose a relaxation technique (n = 28), and the last one was a control group (n = 39). Physiological values were measured using a pressure gauge and the subjective feelings of stress and mood were evaluated by the Likert scale 1-5. The AAA group showed significant improvement after 10 min of interaction in both mood and stress, with no change in heart rate and blood pressure. The remaining groups showed a significant decrease in blood pressure, but not in heart rate, with different evaluations of mood and stress. AAAs with a dog appear to be effective in improving students' mood and stress without affecting their physiological parameters. |
  | Date | 2020-03-28 |
  | Language | eng |
  | Short Title | Effect of Dog Presence on Stress Levels in Students under Psychological Strain |
  | Library Catalogue | PubMed |
  | Volume | 17 |
  | Pages | 2286 |
  | Publication | International Journal of Environmental Research and Public Health |
  | DOI | 10.3390/ijerph17072286 |
  | Issue | 7 |
  | Journal Abbr | Int J Environ Res Public Health |
  | ISSN | 1660-4601 |
  | PMID | 32231132 |
  | PMCID | PMC7178231 |
  | Date Added | 05/02/2026, 16:23:23 |
  | Modified | 05/02/2026, 16:23:23 |

  ### Tags:

  - Dogs
  - stress
  - Animals
  - Female
  - Adult
  - Heart Rate
  - Humans
  - Young Adult
  - Animal Assisted Therapy
  - Blood Pressure
  - Stress, Psychological
  - dog
  - Students
  - Pilot Projects
  - animal assisted activity
  - Affect
  - Relaxation Therapy
  - students’ health

  ### Attachments

  - Full Text
  - PubMed entry
- ## Cognitive mechanisms and neurological foundations of companion animals' role in enhancing human psychological well-being

  |  |  |
  | --- | --- |
  | Item Type | Journal Article |
  | Author | Heng Liu |
  | Author | Jingyuan Lin |
  | Author | Wuji Lin |
  | Abstract | The impact of companion animals on human psychological health has garnered widespread attention. Research demonstrates that companion animals contribute positively in various ways, including reducing depression, anxiety, stress, and fostering positive emotions in humans. Recent studies have revealed significant changes in the activity levels of human emotion-related cortical areas (such as the frontal cortex and amygdala) and neurotransmitter (e.g., oxytocin, cortisol) secretion due to interaction with companion animals. However, research in this domain is still in a nascent stage, with many unknowns in the cognitive neural mechanisms involved. This paper proposes that to understand the cognitive mechanisms through which companion animals affect human psychological health, we need to examine changes in emotional cognitive processing. It aims to uncover the neurological underpinnings of how companion animals enhance human psychological well-being from the perspective of brain connectivity. This approach is expected to provide theoretical support and direction for future research and practical applications in this field. |
  | Date | 2024 |
  | Language | eng |
  | Library Catalogue | PubMed |
  | Volume | 15 |
  | Pages | 1354220 |
  | Publication | Frontiers in Psychology |
  | DOI | 10.3389/fpsyg.2024.1354220 |
  | Journal Abbr | Front Psychol |
  | ISSN | 1664-1078 |
  | PMID | 38721326 |
  | PMCID | PMC11076790 |
  | Date Added | 05/02/2026, 16:20:09 |
  | Modified | 05/02/2026, 16:20:09 |

  ### Tags:

  - human-animal interaction
  - animal-assisted therapy
  - cognitive neural mechanisms
  - companion animals
  - emotion regulation

  ### Attachments

  - Full Text
  - PubMed entry
- ## A Text-Mining Analysis of Research Trends in Animal-Assisted Therapy

  |  |  |
  | --- | --- |
  | Item Type | Journal Article |
  | Author | Shin-Ja Lee |
  | Author | Geun-Hyeon Kim |
  | Author | Yea-Hwang Moon |
  | Author | Sung-Sill Lee |
  | Abstract | Text-mining techniques were used to provide basic data to related policy stakeholders and academic researchers by collecting and analyzing research trends related to animal-mediated healing in a short time. A total of 776 studies were collected using the keyword "animal-assisted therapy" (AAT) in the search engine PubMed, which covers a wide range of topics related to health sciences, biomedical research, and health psychology. Four analysis methods were employed. "Dog" was the most commonly utilized animal in AAT. This study also identified individuals with autism spectrum disorder and post-traumatic stress disorder as the primary research participants. Finally, the terms "health care" and "blood pressure" were identified, indicating that AAT has a positive impact on improving blood pressure and enhancing heart rate. These findings demonstrate that AAT research is being actively pursued in various fields, such as social sciences, medicine, and psychology. |
  | Date | 2023-10-07 |
  | Language | eng |
  | Library Catalogue | PubMed |
  | Volume | 13 |
  | Pages | 3133 |
  | Publication | Animals: an open access journal from MDPI |
  | DOI | 10.3390/ani13193133 |
  | Issue | 19 |
  | Journal Abbr | Animals (Basel) |
  | ISSN | 2076-2615 |
  | PMID | 37835738 |
  | PMCID | PMC10571978 |
  | Date Added | 05/02/2026, 16:20:09 |
  | Modified | 05/02/2026, 16:20:09 |

  ### Tags:

  - animal-assisted therapy
  - big data
  - text mining

  ### Attachments

  - Full Text
  - PubMed entry
- ## Therapy Dogs as a Crisis Intervention After Traumatic Events? - An Experimental Study

  |  |  |
  | --- | --- |
  | Item Type | Journal Article |
  | Author | Johanna Lass-Hennemann |
  | Author | Sarah K. Schäfer |
  | Author | Sonja Römer |
  | Author | Elena Holz |
  | Author | Markus Streb |
  | Author | Tanja Michael |
  | Abstract | Animal-assisted therapy has been proposed as a treatment adjunct for traumatized patients. In animal-assisted crisis response, dogs are used directly after a traumatic event to reduce stress and anxiety. However, to date there are few controlled studies investigating the effects of therapy dogs on PTSD symptoms and to our knowledge there is no study investigating the effects of a therapy dog intervention directly after a traumatic event. In this study, 60 healthy female participants were randomly assigned to one of three groups: after exposure to a "traumatic" film clip (trauma-film paradigm), one group of participants interacted with a friendly dog for 15 min, another group of participants watched a film clip showing a person interacting with a friendly dog and the last group was instructed to relax. Participants who had interacted with the dog after the film reported lower anxiety levels, less negative affect, and more positive affect after the intervention as compared to the other two groups. However, the participants who interacted with the dog showed a smaller decrease in physiological arousal after the traumatic film clip compared to both other groups. There were no differences in intrusion symptoms between the three groups. Our results show that dogs are able to lessen subjectively experienced stress and anxiety after a "traumatic" stress situation. |
  | Date | 2018 |
  | Language | eng |
  | Short Title | Therapy Dogs as a Crisis Intervention After Traumatic Events? |
  | Library Catalogue | PubMed |
  | Volume | 9 |
  | Pages | 1627 |
  | Publication | Frontiers in Psychology |
  | DOI | 10.3389/fpsyg.2018.01627 |
  | Journal Abbr | Front Psychol |
  | ISSN | 1664-1078 |
  | PMID | 30233464 |
  | PMCID | PMC6132135 |
  | Date Added | 05/02/2026, 16:23:23 |
  | Modified | 05/02/2026, 16:23:23 |

  ### Tags:

  - cortisol
  - stress
  - PTSD
  - animal assisted therapy
  - service dogs
  - trauma film paradigm

  ### Attachments

  - Full Text
  - PubMed entry
- ## Presence of a dog reduces subjective but not physiological stress responses to an analog trauma

  |  |  |
  | --- | --- |
  | Item Type | Journal Article |
  | Author | Johanna Lass-Hennemann |
  | Author | Peter Peyk |
  | Author | Markus Streb |
  | Author | Elena Holz |
  | Author | Tanja Michael |
  | Abstract | Dogs are known to have stress and anxiety reducing effects. Several studies have shown that dogs are able to calm people during cognitive and performance stressors. Recently, therapy dogs have been proposed as a treatment adjunct for post-traumatic stress disorder patients. In this study we aimed to investigate, whether dogs also have anxiety- and stress reducing effect during "traumatic stressors." 80 healthy female participants were randomly assigned to one of four conditions. They were exposed to a "traumatic" film clip (trauma-film-paradigm). For one group of participants a friendly dog was present during the film, one group of participants was accompanied by a friendly human, another control group watched the film with a toy animal and the last group watched the film clip alone. Participants that were accompanied by the dog during the film reported lower anxiety ratings and less negative affect after the film clip as compared to the "toy dog group" and the "alone group." Results of the "dog group" were comparable to the group that was accompanied by a friendly human. There were no differences in physiological stress responses between the four conditions. Our results show that dogs are able to lessen subjectively experienced stress and anxiety during a "traumatic" stress situation. This effect was comparable to that of social support by a friendly person. Implications for PTSD patients are discussed. |
  | Date | 2014 |
  | Language | eng |
  | Library Catalogue | PubMed |
  | Volume | 5 |
  | Pages | 1010 |
  | Publication | Frontiers in Psychology |
  | DOI | 10.3389/fpsyg.2014.01010 |
  | Journal Abbr | Front Psychol |
  | ISSN | 1664-1078 |
  | PMID | 25250009 |
  | PMCID | PMC4158977 |
  | Date Added | 05/02/2026, 16:23:23 |
  | Modified | 05/02/2026, 16:23:23 |

  ### Tags:

  - cortisol
  - stress
  - PTSD
  - animal assisted therapy
  - service dogs
  - trauma-film-paradigm

  ### Attachments

  - Full Text
  - PubMed entry
- ## A Case for the Interspecies Transfer of Emotions: A Preliminary Investigation on How Humans Odors Modify Reactions of the Autonomic Nervous System in Horses

  |  |  |
  | --- | --- |
  | Item Type | Journal Article |
  | Author | Antonio Lanata |
  | Author | Mimma Nardelli |
  | Author | Gaetano Valenza |
  | Author | Paolo Baragli |
  | Author | Biagio DrAniello |
  | Author | Alessandra Alterisio |
  | Author | Anna Scandurra |
  | Author | Gun Refik Semin |
  | Author | Enzo Pasquale Scilingo |
  | Abstract | We examined the Autonomic Nervous System (ANS) activity of horses in response to human body odors (BOs) produced under happy and fear states. The ANS response of horses was analyzed in terms of Heart Rate Variability (HRV) features extracted in the frequency domain. Our results revealed that human BOs induce sympathetic and parasympathetic changes and stimulate horses emotionally, suggesting interspecies transfer of emotions via BOs. These preliminary findings open the way to measure changes in horse's ANS dynamics in response to human internal states via human BOs, and allow us to better understand unexpected animal behavior that could compromise human-horse interaction. Moreover, it becomes possible to design more effective strategies to manage animals across a range of situations in which a strict humananimal interaction is required, such as the well known Animal Assisted Therapy (AAT). |
  | Date | 2018-07 |
  | Language | eng |
  | Short Title | A Case for the Interspecies Transfer of Emotions |
  | Library Catalogue | PubMed |
  | Volume | 2018 |
  | Pages | 522-525 |
  | Publication | Annual International Conference of the IEEE Engineering in Medicine and Biology Society. IEEE Engineering in Medicine and Biology Society. Annual International Conference |
  | DOI | 10.1109/EMBC.2018.8512327 |
  | Journal Abbr | Annu Int Conf IEEE Eng Med Biol Soc |
  | ISSN | 2694-0604 |
  | PMID | 30440449 |
  | Date Added | 05/02/2026, 16:23:23 |
  | Modified | 05/02/2026, 16:23:23 |

  ### Tags:

  - Animals
  - Heart Rate
  - Humans
  - Animal Assisted Therapy
  - Emotions
  - Horses
  - Autonomic Nervous System
  - Odorants

  ### Attachments

  - PubMed entry
- ## The effects of a therapy dog intervention on dental fear and anxiety in adult patients undergoing dental procedures: a pilot study

  |  |  |
  | --- | --- |
  | Item Type | Journal Article |
  | Author | Doris Lam |
  | Author | Dominique A. D'Anthony |
  | Author | Sara A. Chilcutt |
  | Author | Amy O'Connor |
  | Author | Andrew J. Avillo |
  | Author | Nicholas J. Hamlin |
  | Author | John E. Schmidt |
  | Abstract | Dental anxiety poses challenges for providing effective oral healthcare. While therapy dogs have shown promise in various medical and mental health contexts, their use for alleviating dental anxiety in adults remains underexplored. This study aimed to investigate the emotional and physiologic effects of therapy dogs on self-reported dental anxiety. Adults with dental anxiety were randomly assigned to an intervention group (DOG; n = 19) or a standard care group (SC; n = 14). Standard self-report measures were used to assess dental anxiety (Index of Dental Anxiety and Fear [IDAF-4C+]), depression (Patient Health Questionnaire 9), and generalized anxiety (Generalized Anxiety Disorder 7) prior to the intervention. Participants in the DOG group received a 10-minute therapy dog intervention before dental procedures in sessions 1 and 2, while participants in the SC group rested quietly for 10 minutes before their procedure. The SC participants received the 10-minute therapy dog intervention before dental procedures in the third and final session, while patients in the DOG group received no intervention prior to their third procedure. After the dental procedures, patients completed a questionnaire about their satisfaction with the dog therapy (Therapy Satisfaction Scale) and recorded their anxiety and comfort levels on visual analog scales. Continuous electrocardiographic recording measured heart rate variability during the intervention and dental procedure. Prior to the intervention, most participants (90.9%) met the IDAF-4C+ criteria for dental anxiety, with 7 (21.2%) meeting the criteria for dental phobia. The DOG group participants expressed high satisfaction with the therapy dog intervention. No significant differences in heart rate variability were observed between the groups during dental procedures. Therapy dogs can effectively manage dental anxiety in adults with mild to moderate dental anxiety, offering potential benefits for oral healthcare. |
  | Date | 2024 |
  | Language | eng |
  | Short Title | The effects of a therapy dog intervention on dental fear and anxiety in adult patients undergoing dental procedures |
  | Library Catalogue | PubMed |
  | Volume | 72 |
  | Pages | 44-49 |
  | Publication | General Dentistry |
  | Issue | 4 |
  | Journal Abbr | Gen Dent |
  | ISSN | 0363-6771 |
  | PMID | 38905604 |
  | Date Added | 05/02/2026, 16:20:09 |
  | Modified | 05/02/2026, 16:20:09 |

  ### Tags:

  - Dogs
  - Animals
  - Female
  - Adult
  - Humans
  - Male
  - Middle Aged
  - Animal Assisted Therapy
  - animal-assisted therapy
  - dog
  - pet therapy
  - Dental Anxiety
  - Pilot Projects
  - dental anxiety
  - Dental Care
  - dental fear

  ### Attachments

  - PubMed entry
- ## Treating Agitation in Patients with Dementia with a Therapy Dog in a Milieu Therapy Setting on a Geropsychiatric Ward

  |  |  |
  | --- | --- |
  | Item Type | Journal Article |
  | Author | Jana Krüger |
  | Author | Reyhan Izgi |
  | Author | Rainer Hellweg |
  | Author | Andreas Ströhle |
  | Author | Maria C. Jockers-Scherübl |
  | Abstract | BACKGROUND: Animal-assisted intervention has become a common therapeutic practice used for patients with dementia in home-dwelling and institutions. The most established procedure is a visiting service by specially trained dogs and their owners to improve social interactions and reduce symptoms of agitation. OBJECTIVES: The study aims to investigate the effects of a therapy dog on agitation of inpatients with dementia in a gerontopsychiatric ward. MATERIALS AND METHODS: The severity of agitation was assessed by a rater blinded for the presence of the dog via the Overt Agitation Severity Scale (OASS). The scale was conducted on 1 day with the dog and his handler present (resident doctor on the ward) and on another day with only the handler present. Each patient was his/her own control. Heart rate variability (HRV) and serum level of brain-derived neurotrophic factor (BDNF) of the patients were measured on both days. 26 patients with the Mini-Mental Status Examination (MMSE) score <21 and the diagnosis of dementia were included in the study. RESULTS: A significant reduction of agitation in the OASS could be shown when the dog was present (p = 0.006). The data neither demonstrated a difference in the HRV for the parameters mean heart rate (p = 0.65), root mean square of successive differences (p = 0.63), and high frequencies (p = 0.27) nor in serum BDNF concentrations (p = 0.42). DISCUSSION: Therapy dogs can be implemented as a therapeutic tool in a gerontopsychiatric ward to reduce symptoms of agitation in patients with dementia. The study was registered in the German Clinical Trials Register (DRKS00024093). |
  | Date | 2021 |
  | Language | eng |
  | Library Catalogue | PubMed |
  | Volume | 50 |
  | Pages | 541-547 |
  | Publication | Dementia and Geriatric Cognitive Disorders |
  | DOI | 10.1159/000520881 |
  | Issue | 6 |
  | Journal Abbr | Dement Geriatr Cogn Disord |
  | ISSN | 1421-9824 |
  | PMID | 34965533 |
  | Date Added | 05/02/2026, 16:23:23 |
  | Modified | 05/02/2026, 16:23:23 |

  ### Tags:

  - Dogs
  - Animals
  - Female
  - Humans
  - Male
  - Hospital
  - Agitation
  - Animal-assisted-therapy
  - Brain-Derived Neurotrophic Factor
  - Dementia
  - Dog-assisted therapy
  - Milieu Therapy
  - Psychomotor Agitation
  - Therapy Animals

  ### Attachments

  - Full Text
  - PubMed entry
- ## Relation of post-traumatic stress disorder symptom severity to the efficacy of an animal-assisted intervention for stress reduction after military aeromedical evacuation

  |  |  |
  | --- | --- |
  | Item Type | Journal Article |
  | Author | Cheryl A. Krause-Parello |
  | Author | Erika Friedmann |
  | Author | Candy Wilson |
  | Author | Jennifer J. Hatzfeld |
  | Author | John Kolassa |
  | Author | Alisha Hackney |
  | Author | Kristie A. Morales |
  | Abstract | Animal-assisted interventions (AAIs) have been found to decrease stress in some settings, but it is not known if AAI is feasible in an aeromedical staging facility or effective in reducing stress following aeromedical evacuation (AE) of military personnel. An experimental design was used to evaluate the efficacy of AAI at reducing stress in AE military patients (N = 120). Patients participated in a 20-min AAI (n = 60) or 20-min informational session about assistance dogs as an attention-control group (n = 60). Demographics, post-traumatic stress symptom severity (PTSSS), and stress biomarkers (cortisol, alpha-amylase, and immunoglobulin A) were collected regular intervals. AAI was found feasible and efficacious at reducing stress. Cortisol decreased significantly (p < .05) in the AAI group compared with the attention-control group. PTSSS moderated the immunoglobulin A responses to AAI as demonstrated by the interaction of PTSD Checklist-Military Version score, group, and time, F(1, 111.23) = 4.15 p = .044; effect size: d = 0.31. This research supports AAI as a stress-reducing modality in AE patients, particularly those who report higher PTSSS. Implications for future research are discussed. |
  | Date | 2019-10 |
  | Language | eng |
  | Library Catalogue | PubMed |
  | Volume | 35 |
  | Pages | 480-490 |
  | Publication | Stress and Health: Journal of the International Society for the Investigation of Stress |
  | DOI | 10.1002/smi.2881 |
  | Issue | 4 |
  | Journal Abbr | Stress Health |
  | ISSN | 1532-2998 |
  | PMID | 31274219 |
  | Date Added | 05/02/2026, 16:20:09 |
  | Modified | 05/02/2026, 16:20:09 |

  ### Tags:

  - cortisol
  - Hydrocortisone
  - Dogs
  - Animals
  - Female
  - Humans
  - Male
  - Animal Assisted Therapy
  - Stress, Psychological
  - animal-assisted intervention
  - Biomarkers
  - immunoglobulin A
  - Military Personnel
  - Stress Disorders, Post-Traumatic
  - Immunoglobulin A
  - alpha-Amylases
  - Air Ambulances
  - alpha-amylase
  - biomarkers
  - Emergency Shelter
  - post-traumatic stress disorder

  ### Attachments

  - PubMed entry
- ## Effects of VA Facility Dog on Hospitalized Veterans Seen by a Palliative Care Psychologist: An Innovative Approach to Impacting Stress Indicators

  |  |  |
  | --- | --- |
  | Item Type | Journal Article |
  | Author | Cheryl A. Krause-Parello |
  | Author | Cari Levy |
  | Author | Elizabeth Holman |
  | Author | John E. Kolassa |
  | Abstract | The United States is home to 23 million veterans. In many instances, veterans with serious illness who seek healthcare at the VA receive care from a palliative care service. Animal-assisted intervention (AAI) is gaining attention as a therapeutic stress reducing modality; however, its effects have not been well studied in veterans receiving palliative care in an acute care setting. A crossover repeated-measures study was conducted to examine the effects of an animal-assisted intervention (AAI) in the form of a therapy dog on stress indicators in 25 veterans on the palliative care service at the VA Eastern Colorado Healthcare System in Denver, CO. Veterans had a visit from a therapy dog and the dog's handler, a clinical psychologist (experimental condition) and an unstructured visit with the clinical psychologist alone (control condition). Blood pressure, heart rate, and the salivary biomarkers cortisol, alpha-amylase, and immunoglobulin A were collected before, after, and 30-minutes after both the experimental and control conditions. Significant decreases in cortisol were found when the before time period was compared to the 30-minutes after time period for both the experimental ( p = 0.007) and control condition ( p = 0.036). A significant decrease in HR was also found when the before time period was compared to the 30-minutes after time period for both the experimental ( p = 0.0046) and control ( p = 0.0119) condition. Results of this study supported that a VA facility dog paired with a palliative care psychologist had a measurable impact on salivary cortisol levels and HR in veterans. |
  | Date | 2018-01 |
  | Language | eng |
  | Short Title | Effects of VA Facility Dog on Hospitalized Veterans Seen by a Palliative Care Psychologist |
  | Library Catalogue | PubMed |
  | Volume | 35 |
  | Pages | 5-14 |
  | Publication | The American Journal of Hospice & Palliative Care |
  | DOI | 10.1177/1049909116675571 |
  | Issue | 1 |
  | Journal Abbr | Am J Hosp Palliat Care |
  | ISSN | 1938-2715 |
  | PMID | 27895150 |
  | Date Added | 05/02/2026, 16:23:23 |
  | Modified | 05/02/2026, 16:23:23 |

  ### Tags:

  - cortisol
  - Hydrocortisone
  - Dogs
  - stress
  - Animals
  - Female
  - Adult
  - Heart Rate
  - Humans
  - Male
  - Middle Aged
  - Aged
  - Animal Assisted Therapy
  - Blood Pressure
  - Stress, Psychological
  - Saliva
  - heart rate
  - animal-assisted intervention
  - United States
  - Immunoglobulin A
  - Psychotherapy
  - therapy dog
  - Aged, 80 and over
  - Quality of Life
  - alpha-Amylases
  - Palliative Care
  - veterans
  - Colorado
  - Socioeconomic Factors
  - United States Department of Veterans Affairs

  ### Attachments

  - PubMed entry
- ## Veterans Training Service Dogs for Other Veterans: An Animal-Assisted Intervention for Post-Traumatic Stress Disorder

  |  |  |
  | --- | --- |
  | Item Type | Journal Article |
  | Author | Cheryl A. Krause-Parello |
  | Author | Erika Friedmann |
  | Author | Deborah Taber |
  | Author | Haidong Zhu |
  | Author | Alejandra Quintero |
  | Author | Rick Yount |
  | Abstract | Research on the post-deployment reintegration needs of women veterans is limited. Non-traditional support may enhance mental health. Relationships with animals and volunteering may aid those with post-traumatic stress disorder (PTSD). Using the biopsychosocial model, we examined whether participation in an 8-week service dog training program (SDTP) affected telomere length (TL), heart rate variability (HRV), PTSD symptom severity, perceived stress, and anxiety in female veterans with PTSD, as well as whether combat exposure influenced these relationships. Female veterans (ages 32-72, M = 45.9, SD = 11.8) with PTSD were randomized to either the SDTP group (n = 13) or a comparison group (n = 15) that received dog training video content. The interventions lasted one hour weekly for 8 weeks. Outcomes were assessed pre-, mid-, and post-intervention. Linear mixed models with random intercepts examined changes from pre- to post-intervention and compared changes by group and combat exposure. TL changes differed [F(1,11.65) = 3.543, p = 0.085] by intervention. In the SDTP group, TL increased, indicating reduced cellular senescence (i.e., slower biological aging), whereas TL decreased in the CI group. Combat exposure moderated these changes [F(1,12.36) = 5.41, p = 0.038]. HRV changed by intervention group [F(1,389.08) = 10.623, p = 0.001]. HRV decreased (stress increased) in the SDTP group but not in the CI group. Combat exposure did not moderate HRV changes. PTSD symptom severity [F(1,48.04) = 19.22, p < 0.001], perceived stress [F(1,48.48) = 14.65, p < 0.001], and anxiety [F(1,47.30) = 6.624, p = 0.013] decreased significantly from pre- to post-interventions; the decreases did not differ by intervention or combat exposure. |
  | Date | 2025-08-29 |
  | Language | eng |
  | Short Title | Veterans Training Service Dogs for Other Veterans |
  | Library Catalogue | PubMed |
  | Volume | 15 |
  | Place | Basel, Switzerland |
  | Pages | 1180 |
  | Publication | Behavioral Sciences |
  | DOI | 10.3390/bs15091180 |
  | Issue | 9 |
  | Journal Abbr | Behav Sci (Basel) |
  | ISSN | 2076-328X |
  | PMID | 41009210 |
  | PMCID | PMC12466435 |
  | Date Added | 05/02/2026, 16:23:23 |
  | Modified | 05/02/2026, 16:23:23 |

  ### Tags:

  - heart rate variability
  - cellular aging
  - combat exposure
  - female veterans
  - telomere

  ### Attachments

  - PubMed entry
- ## Examining the Effects of a Service-Trained Facility Dog on Stress in Children Undergoing Forensic Interview for Allegations of Child Sexual Abuse

  |  |  |
  | --- | --- |
  | Item Type | Journal Article |
  | Author | Cheryl A. Krause-Parello |
  | Author | Michele Thames |
  | Author | Colleen M. Ray |
  | Author | John Kolassa |
  | Abstract | Disclosure of child sexual abuse can be a stressful experience for the child. Gaining a better understanding of how best to serve the child, while preserving the quality of their disclosure, is an ever-evolving process. The data to answer this question come from 51 children aged 4-16 (M = 9.1, SD = 3.5), who were referred to a child advocacy center in Virginia for a forensic interview (FI) following allegations of sexual abuse. A repeated measures design was conducted to examine how the presence of a service-trained facility dog (e.g. animal-assisted intervention (AAI) may serve as a mode of lowering stress levels in children during their FIs. Children were randomized to one of the two FI conditions: experimental condition (service-trained facility dog present-AAI) or control condition (service-trained facility dog not present- standard forensic interview). Stress biomarkers salivary cortisol, alpha-amylase, immunoglobulin A (IgA), heart rate, and blood pressure, and Immunoglobulin A were collected before and after the FI. Self-report data were also collected. Results supported a significant decrease in heart rate for those in the experimental condition (p = .0086) vs the control condition (p = .4986). Regression models revealed a significant decrease in systolic and diastolic blood pressure in the experimental condition (p = .03285) and (p = .04381), respectively. Statistically significant changes in alpha-amylase and IgA were also found in relation to disclosure and type of offense. The results of this study support the stress reducing effects of a service-trained facility dog for children undergoing FI for allegations of child sexual abuse. |
  | Date | 2018-04 |
  | Language | eng |
  | Library Catalogue | PubMed |
  | Volume | 27 |
  | Pages | 305-320 |
  | Publication | Journal of Child Sexual Abuse |
  | DOI | 10.1080/10538712.2018.1443303 |
  | Issue | 3 |
  | Journal Abbr | J Child Sex Abus |
  | ISSN | 1547-0679 |
  | PMID | 29533149 |
  | Date Added | 05/02/2026, 16:23:23 |
  | Modified | 05/02/2026, 16:23:23 |

  ### Tags:

  - Hydrocortisone
  - Dogs
  - Animals
  - Female
  - Heart Rate
  - Human-Animal Bond
  - Humans
  - Male
  - Blood Pressure
  - Stress, Psychological
  - Saliva
  - children
  - Child
  - Child, Preschool
  - Adolescent
  - Child Abuse, Sexual
  - Forensic Psychiatry
  - Interview, Psychological
  - Child Advocacy
  - intervention
  - Salivary alpha-Amylases
  - Sexual abuse
  - sexual abuse disclosure
  - Truth Disclosure

  ### Attachments

  - PubMed entry
- ## Pet Therapy: Enhancing Social and Cardiovascular Wellness in Community Dwelling Older Adults

  |  |  |
  | --- | --- |
  | Item Type | Journal Article |
  | Author | Cheryl A. Krause-Parello |
  | Author | John Kolassa |
  | Abstract | Pet therapy can be therapeutic for older adults living in the community. A crossover design was used to examine changes in blood pressure and heart rate before and after a pet therapy visit versus a volunteer-only visit in 28 community dwelling older adults. Relationships among stress, pet attitude, social support, and health status were also examined. Study findings supported that pet therapy significantly decreased blood pressure and heart rate. Ultimately, the findings supported the notion that community health nurses should consider developing and implementing pet therapy programs in the communities they serve. Further implications for community health nurses are discussed. |
  | Date | 2016 |
  | Language | eng |
  | Short Title | Pet Therapy |
  | Library Catalogue | PubMed |
  | Volume | 33 |
  | Pages | 1-10 |
  | Publication | Journal of Community Health Nursing |
  | DOI | 10.1080/07370016.2016.1120587 |
  | Issue | 1 |
  | Journal Abbr | J Community Health Nurs |
  | ISSN | 1532-7655 |
  | PMID | 26813050 |
  | Date Added | 05/02/2026, 16:23:23 |
  | Modified | 05/02/2026, 16:23:23 |

  ### Tags:

  - Dogs
  - Animals
  - Female
  - Heart Rate
  - Humans
  - Male
  - Middle Aged
  - Aged
  - Animal Assisted Therapy
  - Cross-Over Studies
  - Blood Pressure
  - Social Support
  - Health Status
  - Aged, 80 and over
  - Cardiovascular Diseases
  - Independent Living

  ### Attachments

  - PubMed entry
- ## Forensic Interviews for Child Sexual Abuse Allegations: An Investigation into the Effects of Animal-Assisted Intervention on Stress Biomarkers

  |  |  |
  | --- | --- |
  | Item Type | Journal Article |
  | Author | Cheryl A. Krause-Parello |
  | Author | Elsie E. Gulick |
  | Abstract | The use of therapy animals during forensic interviews for child sexual abuse allegations is a recommendation by the Therapy Animals Supporting Kids Program to help ease children's discomfort during the forensic interview process. Based on this recommendation, this study incorporated a certified therapy canine into the forensic interview process for child sexual abuse allegations. This study investigated changes in salivary cortisol, immunoglobulin A, blood pressure, and heart rate as a result of forensic interview phenomenon (e.g., outcry) incorporating animal-assisted intervention versus a control condition in children (N = 42) interviewed for alleged child sexual abuse. The results supported significantly greater heart rate values for the control group (n = 23) who experienced sexual contact and/or indecency than the experience of aggravated sexual assault compared to no difference in HR for the intervention group (n = 19). The results suggest that the presence of the canine in the forensic interview may have acted as a buffer or safeguard for the children when disclosing details of sexual abuse. In the intervention group, children's HR was lower at the start of the forensic interview compared to the control group. Finding an effect of having a certified handler-canine team available during the forensic interview on physiological measures of stress has real-world value for children, child welfare personnel, and clinical therapists. It is suggested that animal-assisted intervention be expanded to children facing other types of trauma and to treatment programs for child survivors of sexual abuse. |
  | Date | 2015 |
  | Language | eng |
  | Short Title | Forensic Interviews for Child Sexual Abuse Allegations |
  | Library Catalogue | PubMed |
  | Volume | 24 |
  | Pages | 873-886 |
  | Publication | Journal of Child Sexual Abuse |
  | DOI | 10.1080/10538712.2015.1088916 |
  | Issue | 8 |
  | Journal Abbr | J Child Sex Abus |
  | ISSN | 1547-0679 |
  | PMID | 26701279 |
  | Date Added | 05/02/2026, 16:20:09 |
  | Modified | 05/02/2026, 16:20:09 |

  ### Tags:

  - cortisol
  - Hydrocortisone
  - Dogs
  - Animals
  - Female
  - Heart Rate
  - Humans
  - Animal Assisted Therapy
  - Blood Pressure
  - Stress, Physiological
  - Child
  - immunoglobulin A
  - Immunoglobulin A
  - Case-Control Studies
  - therapy dog
  - Child Abuse, Sexual
  - child advocacy center
  - Forensic Psychiatry
  - Interview, Psychological

  ### Attachments

  - PubMed entry
- ## Randomized Trial of Therapy Dogs Versus Deliberative Coloring (Art Therapy) to Reduce Stress in Emergency Medicine Providers

  |  |  |
  | --- | --- |
  | Item Type | Journal Article |
  | Author | Jeffrey A. Kline |
  | Author | Kimberly VanRyzin |
  | Author | Jacob C. Davis |
  | Author | Jonathan A. Parra |
  | Author | Maxwell L. Todd |
  | Author | Liza L. Shaw |
  | Author | Benjamin R. Haggard |
  | Author | Michelle A. Fisher |
  | Author | Katherine L. Pettit |
  | Author | Alan M. Beck |
  | Abstract | OBJECTIVE: Cognitive stress during shift work contributes to burnout in emergency department (ED) workers. We hypothesize that if physicians and nurses interact with a therapy dog for 5 minutes while on ED shift, both their perceived and their manifested stress levels will decrease. METHODS: In this single-center, prospective, randomized controlled clinical trial (NCT03628820), we tested the effectiveness of therapy dogs versus coloring a mandala and versus no intervention (control) on provider stress. Consenting emergency medicine physicians and nurses provided three self-reported assessments of stress and saliva samples at the start (T1), at the middle (T2), and near the end (T3) of shift. Thirty minutes prior to T2, participants were randomized to either interacting with a therapy dog or coloring for 5 minutes; controls had neither. Stress was assessed on visual analog scale (VAS, 0-100 mm) and with salivary cortisol (Salimetrics) and the modified Perceived Stress Scale (mPSS-10). To assess potential change in participant behavior, patients of providers in either group were asked to complete an internally derived survey of empathic behaviors displayed by providers at T1 and T3. RESULTS: We enrolled 122 providers (n = 39 control, n = 40 coloring, n = 43 dog); 48% were residents, and 60% enrolled on an evening shift. At T1, mean (±SD) VAS score was not different between groups (18.2 [±17.8] mm). At T3, VAS tended to increase with coloring (24.5 mm), remain unchanged in controls (20 mm), and decreased slightly with dogs (13.6 mm, p = 0.018 vs. coloring, Tukey's post hoc). Salivary cortisol levels were consistently highest at the beginning of each providers' shift and were significantly decreased versus control in both the dog and the coloring groups (p < 0.05, Tukey's). We observed no difference between groups for the mPSS-10 nor in patient reported survey of empathic behaviors. CONCLUSION: This randomized controlled clinical trial demonstrates preliminary evidence that a 5-minute therapy dog interaction while on shift can reduce provider stress in ED physicians and nurses. |
  | Date | 2020-04 |
  | Language | eng |
  | Library Catalogue | PubMed |
  | Volume | 27 |
  | Pages | 266-275 |
  | Publication | Academic Emergency Medicine: Official Journal of the Society for Academic Emergency Medicine |
  | DOI | 10.1111/acem.13939 |
  | Issue | 4 |
  | Journal Abbr | Acad Emerg Med |
  | ISSN | 1553-2712 |
  | PMID | 32266765 |
  | Date Added | 05/02/2026, 16:23:23 |
  | Modified | 05/02/2026, 16:23:23 |

  ### Tags:

  - Hydrocortisone
  - Dogs
  - Animals
  - Female
  - Adult
  - Humans
  - Male
  - Middle Aged
  - Aged
  - Animal Assisted Therapy
  - Surveys and Questionnaires
  - Emergency Service, Hospital
  - Prospective Studies
  - Art Therapy
  - Burnout, Professional
  - Nursing Staff, Hospital
  - Physicians
  - Visual Analog Scale

  ### Attachments

  - Full Text
  - PubMed entry
- ## Therapy Dogs at the Bedside: A Scoping Review of Pet Therapy in Adult Intensive Care Units

  |  |  |
  | --- | --- |
  | Item Type | Journal Article |
  | Author | Joseph L. Kim |
  | Abstract | Adult intensive care unit (ICU) patients experience significant anxiety, distress, and isolation. Pet therapy or animal-assisted interventions, often delivered by certified therapy dogs, are used in hospitals to improve comfort and engagement, but ICU-specific evidence remains limited. This scoping review maps the recent evidence on pet therapy in adult ICUs, with the aim of describing how it is used, what benefits are reported, what risks and barriers exist, and where the literature remains thin. Peer-reviewed publications from 2015 to 2025 were found through major biomedical databases (including PubMed, Cumulative Index to Nursing and Allied Health Literature (CINAHL), Embase, and Scopus) that reported live animal interactions in adult ICUs, along with any relevant reviews and implementation reports. The studies were independently screened according to the inclusion and exclusion criteria, and a total of 15 studies met eligibility criteria. Across the studies, patients showed immediate reductions in self-reported anxiety after visits, families often reported parallel relief, and satisfaction with the experience was high among patients, families, and staff. Pain scores improved modestly in some cohorts, while heart rate, blood pressure, and respiratory rate were generally unchanged, suggesting psychological benefit without physiologic destabilization. Pet therapy programs were feasible in the ICU when supported by clear policies and coordination, although common barriers included infection prevention concerns, scheduling and staffing needs, space constraints, and the limited availability of controlled data. The evidence was insufficient to determine effects on delirium, sedative exposure, length of stay, or mortality. Overall, pet therapy in adult ICUs appears feasible, well-accepted, and safe under structured protocols, with consistent short-term relief of anxiety and perceived distress and potential support for engagement in rehabilitation. Future work should include multicenter controlled studies, formal safety surveillance, standardized outcome sets that include delirium and longer-term psychological recovery, cost and implementation analyses, and evaluation of robotic or virtual animal options when live visits are not possible. |
  | Date | 2025-11 |
  | Language | eng |
  | Short Title | Therapy Dogs at the Bedside |
  | Library Catalogue | PubMed |
  | Volume | 17 |
  | Pages | e97092 |
  | Publication | Cureus |
  | DOI | 10.7759/cureus.97092 |
  | Issue | 11 |
  | Journal Abbr | Cureus |
  | ISSN | 2168-8184 |
  | PMID | 41255502 |
  | PMCID | PMC12622910 |
  | Date Added | 05/02/2026, 16:23:23 |
  | Modified | 05/02/2026, 16:23:23 |

  ### Tags:

  - animal assisted therapy
  - pet therapy
  - therapy dogs
  - animal assisted intervention
  - icu
  - intensive care unit
  - patient experience
  - pet assisted intervention

  ### Attachments

  - PubMed entry
- ## Are therapy animals the key to happier dental visits for children?

  |  |  |
  | --- | --- |
  | Item Type | Journal Article |
  | Author | Sara Khan |
  | Abstract | A COMMENTARY ON: Massouda J, Ghaltakhchyan N, Judd J, Bocklage C, Selden R, TumSuden O, Nanney E, Lee J, Ginnis J, Strauman T, Sawicki C, Hodges EA, Graves C, Divaris K, Jacox L. Evaluating effects of animal-assisted therapy on paediatric dental care patients: A pilot clinical trial. J Am Dent Assoc. 2025;156:447-457. https://doi.org/10.1016/j.adaj.2025.03.006 DATA SOURCES: This commentary is based on the published pilot clinical trial by Massouda et al. (2025) evaluating animal-assisted therapy (AAT) in paediatric dental care. STUDY SELECTION: The study included children aged 7-14 undergoing invasive dental procedures, allocated to either an AAT or control group. DATA EXTRACTION AND SYNTHESIS: Outcome measures included validated self-reported anxiety and pain scales, physiological stress markers, and behavioural observations. Data was synthesised narratively given pilot design and small sample size. DESIGN: A prospective, non-randomised pilot trial carried out in a university paediatric dental setting assessed the practicality and initial effects of integrating animal-assisted therapy (AAT) into dental treatment. CASE SELECTION: Thirty-nine children aged 7-14 years scheduled for invasive dental procedures were enroled. Participants were allocated to an AAT group (n = 18) or a control group (n = 21). Inclusion criteria included the ability to assent and a willingness to interact with a certified therapy animal. Exclusion criteria included previous traumatic experiences with AAT, or significant developmental or behavioural disorders affecting cooperation. DATA ANALYSIS: Physiological and psychological measures were recorded at baseline, during treatment, and post-operatively. Primary outcomes included self-reported pain and anxiety scores using validated scales. Secondary outcomes included heart rate monitoring, salivary cortisol, α-amylase, and video-coded behavioural relaxation. Given the small sample size, statistical analyses employed nonparametric 35 tests with significance set at P < 0.05. RESULTS: Children exposed to the therapy dog reported significantly lower postoperative pain scores (P = 0.001) and demonstrated smaller heart rate fluctuations during stressful procedural moments, suggesting lower physiological stress responses. Behavioural observations indicated longer periods of relaxed posture in the AAT group, though this did not reach statistical significance (P = 0.204). No significant differences were detected in salivary cortisol or α-amylase. AAT was reported as safe, well-tolerated, and feasible to implement. CONCLUSIONS: AAT shows potential for reducing distress in paediatric dental settings, but larger, randomised studies are required. Within the limits of a pilot design, animal-assisted therapy may reduce pain and physiological stress in paediatric dental patients. While encouraging, larger randomised trials are needed to confirm these effects, explore mechanisms, and assess practicality and cost in routine dental practice. |
  | Date | 2025-12 |
  | Language | eng |
  | Library Catalogue | PubMed |
  | Volume | 26 |
  | Pages | 174-175 |
  | Publication | Evidence-Based Dentistry |
  | DOI | 10.1038/s41432-025-01197-6 |
  | Issue | 4 |
  | Journal Abbr | Evid Based Dent |
  | ISSN | 1476-5446 |
  | PMID | 41361505 |
  | Date Added | 05/02/2026, 16:23:23 |
  | Modified | 05/02/2026, 16:23:23 |

  ### Tags:

  - Animals
  - Female
  - Humans
  - Male
  - Animal Assisted Therapy
  - Child
  - Adolescent
  - Dental Anxiety
  - Pilot Projects
  - Pain Measurement
  - Dental Care for Children
  - Prospective Studies
  - Clinical Trials as Topic

  ### Attachments

  - PubMed entry
- ## Therapy Dogs for Anxiety in Children in the Emergency Department: A Randomized Clinical Trial

  |  |  |
  | --- | --- |
  | Item Type | Journal Article |
  | Author | Heather P. Kelker |
  | Author | Huma K. Siddiqui |
  | Author | Alan M. Beck |
  | Author | Jeffrey A. Kline |
  | Abstract | IMPORTANCE: Prior evidence suggests that the use of therapy dogs in emergency care reduces anxiety in adults, but no trial has tested the use of therapy dogs in emergency care of children. OBJECTIVE: To examine whether adjunctive use of therapy dogs in standard child-life therapy reduces child-reported and parent-reported child anxiety in a pediatric emergency department (ED). DESIGN, SETTING, AND PARTICIPANTS: This randomized clinical trial was conducted from February 1, 2023, to June 30, 2024, at an academic pediatric ED. Children (aged 5-17 years) with suspected moderate to high anxiety were included. INTERVENTION: All participants received standard child-life therapy, and the intervention group was randomly assigned to have exposure to a therapy dog and handler for approximately 10 minutes. MAIN OUTCOMES AND MEASURES: Anxiety was measured using the 0- to 10-point FACES scale (with 0 indicating no anxiety and 10 indicating very severe anxiety) and salivary cortisol concentrations. Measurements were obtained at baseline (T0), 45 minutes (T1), and 120 minutes (T2) for both child and parents. RESULTS: A total of 80 patients (mean [SD] age, 10.9 [3.8] years; 45 [56%] female) were enrolled (40 in the control group and 40 in the intervention group). At T0, the mean (SD) FACES scores were 5.4 (2.8) for child report and 6.4 (2.4) for parent report; the means were not different between groups. From T0 to T1, child-reported anxiety changed by a mean (SD) of -1.5 (3.4) points in the control group vs -2.7 (2.5) points in the intervention group (P = .02, Mann-Whitney U test); similarly, mean (SD) parent-estimated child anxiety changed by -1.8 (2.7) points in the control group vs -3.2 (2.3) points in the intervention group (P = .008). A total of 9 children (23%) in the control group had a greater than 2.5-point decrease in FACES score vs 18 (46%) in intervention group (P = .04, Fisher test). At T2, mean (SD) child-reported FACES scores decreased to 3.6 (3.4) points in the control group and 3.0 (2.7) points in the intervention group (P = .70). A total of 14 control participants (35%) received ketamine, midazolam, lorazepam, or droperidol vs 7 (18%) in the intervention group (P = .08, Fisher test). Child and parent salivary cortisol decreased from T0 to T1 in both groups but was not different between groups. Parental salivary cortisol was significantly consistently higher than their children's salivary cortisol (P < .001, unpaired t test, for comparisons of child vs parent at T0 and T1 in both groups). CONCLUSIONS AND RELEVANCE: This study of adjunctive use of therapy dogs in standard child-life therapy found a modest but significantly greater reduction in both child-reported and parental-reported child anxiety in the pediatric ED for the intervention vs control group. These findings support the use of therapy dogs to help reduce pain and anxiety without the use of chemical or physical constraint. TRIAL REGISTRATION: ClinicalTrials.gov Identifier: NCT03784573. |
  | Date | 2025-03-03 |
  | Language | eng |
  | Short Title | Therapy Dogs for Anxiety in Children in the Emergency Department |
  | Library Catalogue | PubMed |
  | Volume | 8 |
  | Pages | e250636 |
  | Publication | JAMA network open |
  | DOI | 10.1001/jamanetworkopen.2025.0636 |
  | Issue | 3 |
  | Journal Abbr | JAMA Netw Open |
  | ISSN | 2574-3805 |
  | PMID | 40085085 |
  | PMCID | PMC11909607 |
  | Date Added | 05/02/2026, 16:20:09 |
  | Modified | 05/02/2026, 16:20:09 |

  ### Tags:

  - Hydrocortisone
  - Dogs
  - Animals
  - Female
  - Humans
  - Male
  - Animal Assisted Therapy
  - Saliva
  - Child
  - Child, Preschool
  - Adolescent
  - Anxiety
  - Emergency Service, Hospital
  - Treatment Outcome

  ### Attachments

  - PubMed entry
- ## Effects of animal-assisted therapy on dental anxiety, behavior, and perceptions in young pediatric patients: a blinded randomized controlled trial

  |  |  |
  | --- | --- |
  | Item Type | Journal Article |
  | Author | Grace Kapov |
  | Author | Kasey Linton |
  | Author | Christopher Gatewood |
  | Author | Chuwen Liu |
  | Author | Timothy Strauman |
  | Author | Eric Hodges |
  | Author | Christina Graves |
  | Author | Caroline Sawicki |
  | Author | Di Wu |
  | Author | Kimon Divaris |
  | Author | Laura Anne Jacox |
  | Abstract | BACKGROUND: Between 6 and 22% of children are affected by dental anxiety. Dental anxiety is a significant barrier to dental care and is associated with dental avoidance and negative oral health outcomes. Pharmacological methods of anxiety management are costly, carry risks of adverse outcomes, and may not be acceptable to some families. Alternative non-pharmacological methods are needed for the safe and effective delivery of dental care. Although there is an abundance of literature regarding animal-assisted therapy (AAT) in medicine, only preliminary studies on AAT exist in dentistry. To identify optimal outcome measures for evaluating AAT in pediatric dental contexts, a randomized controlled trial protocol was developed. METHODS: A prospective randomized controlled trial protocol was developed to examine the impact of AAT on objective (heart rate, salivary stress and pain markers, and observational coding) and subjective self-reported measures of anxiety, pain, and dental expectations in pediatric patients. The study is designed to enroll 180 pediatric patients (4-8 years old), randomized into three arms (n = 60 per arm) with stratification by age (< 6.5 vs ≥ 6.5) and gender (block size = 4). Two therapy protocols (+ Short AAT and + Long AAT exposures) will be compared relative to an active control (coloring a dog picture) during a diagnostic dental visit consisting of an oral exam, dental cleaning, and simulated bitewing intraoral radiographs. DISCUSSION: This study will provide information on optimal outcome measures to evaluate the impact of AAT on dental anxiety and behavior in pediatric dental patients. Determining the effects of AAT in pediatric dental care may provide a safe, non-pharmacological method of anxiety and behavior management, with broad translational impact. TRIAL REGISTRATION: This trial was registered on ClinicalTrials.gov with number NCT05464888, on 15 July 2022 (first submitted to ClinicalTrials.gov) and 19 July 2022 (first posted to ClinicalTrials.gov). |
  | Date | 2025-08-07 |
  | Language | eng |
  | Short Title | Effects of animal-assisted therapy on dental anxiety, behavior, and perceptions in young pediatric patients |
  | Library Catalogue | PubMed |
  | Volume | 26 |
  | Pages | 279 |
  | Publication | Trials |
  | DOI | 10.1186/s13063-025-08970-z |
  | Issue | 1 |
  | Journal Abbr | Trials |
  | ISSN | 1745-6215 |
  | PMID | 40775655 |
  | PMCID | PMC12330074 |
  | Date Added | 05/02/2026, 16:20:09 |
  | Modified | 05/02/2026, 16:20:09 |

  ### Tags:

  - Animals
  - Female
  - Heart Rate
  - Humans
  - Male
  - Animal Assisted Therapy
  - Saliva
  - Child
  - Child, Preschool
  - Dog
  - Animal-assisted therapy
  - Dental Anxiety
  - Pediatric dentistry
  - Treatment Outcome
  - Pain Measurement
  - Age Factors
  - Animal therapy
  - Behavior management
  - Child Behavior
  - Dental anxiety
  - Dental Care for Children
  - Dental fear
  - Dentistry
  - Perception
  - Prospective Studies
  - Randomized Controlled Trials as Topic

  ### Attachments

  - PubMed entry
- ## Effect of animal assisted interactions on activity and stress response in children in acute care settings

  |  |  |
  | --- | --- |
  | Item Type | Journal Article |
  | Author | Mary Lou Jennings |
  | Author | Douglas A. Granger |
  | Author | Crystal I. Bryce |
  | Author | Denice Twitchell |
  | Author | Kim Yeakel |
  | Author | Patricia A. Teaford |
  | Abstract | OBJECTIVE: Determine the effects of animal assisted interactions (AAI) on activity and stress response in pediatric acute care settings. DESIGN: Randomized treatment control design. SETTING: Inpatient pediatric acute care units (PICU, CVICU and Hematology/Oncology). PATIENTS: Eighty pediatric inpatients (49% male) age 2-19 years. INTERVENTION: The AAI experimental group patients interacted with therapy dog teams for 5-10 min and the comparison group patients continued their current activity without an AAI visit. MEASUREMENT AND RESULTS: Salivary cortisol, activity level, and mood were assessed before and after AAI. AAI was associated with a decrease in cortisol levels and increases in mood and activity. CONCLUSION: AAI benefits children in pediatric acute care units. |
  | Date | 2021-11 |
  | Language | eng |
  | Library Catalogue | PubMed |
  | Volume | 8 |
  | Pages | 100076 |
  | Publication | Comprehensive Psychoneuroendocrinology |
  | DOI | 10.1016/j.cpnec.2021.100076 |
  | Journal Abbr | Compr Psychoneuroendocrinol |
  | ISSN | 2666-4976 |
  | PMID | 35757663 |
  | PMCID | PMC9216416 |
  | Date Added | 05/02/2026, 16:20:09 |
  | Modified | 05/02/2026, 16:20:09 |

  ### Tags:

  - Activity
  - Salivary cortisol
  - Acute care
  - Animal assisted interaction or animal assisted therapy
  - Pediatric

  ### Attachments

  - Full Text
  - PubMed entry
- ## [A daycare program of animal assisted therapy for affective disorder patients during psychotropic drug therapy: evaluation of the relaxation effect by fNIRS (functional near-infrared spectroscopy)]

  |  |  |
  | --- | --- |
  | Item Type | Journal Article |
  | Author | Kazuhiko Iwahashi |
  | Author | Fumihiko Fukamauchi |
  | Author | Jun Aoki |
  | Author | Kouhei Kurihara |
  | Author | Eiji Yoshihara |
  | Author | Masao Inoue |
  | Author | Hiroko Shibanai |
  | Author | Jun Ishigooka |
  | Abstract | During daycare programs of animal assisted therapy (AAT), we collected data on the brain function of two affective disorder patients who received psychotropic drug therapy with fNIRS, after written informed consent was obtained. A male patient at first showed a bloodstream drop, seen in the lower inside part of frontal lobe. In both patients, at least a slight activation of the function of the frontal lobe was seen during the therapy. Therefore, an activation effect of AAT was seen at least objectively by fNIRS. |
  | Date | 2010-06 |
  | Language | jpn |
  | Short Title | [A daycare program of animal assisted therapy for affective disorder patients during psychotropic drug therapy |
  | Library Catalogue | PubMed |
  | Volume | 30 |
  | Pages | 129-134 |
  | Publication | Nihon Shinkei Seishin Yakurigaku Zasshi = Japanese Journal of Psychopharmacology |
  | Issue | 3 |
  | Journal Abbr | Nihon Shinkei Seishin Yakurigaku Zasshi |
  | ISSN | 1340-2544 |
  | PMID | 20666144 |
  | Date Added | 05/02/2026, 16:23:23 |
  | Modified | 05/02/2026, 16:23:23 |

  ### Tags:

  - Brain
  - Female
  - Adult
  - Humans
  - Male
  - Animal Assisted Therapy
  - Spectroscopy, Near-Infrared
  - Mood Disorders
  - Psychotropic Drugs

  ### Attachments

  - PubMed entry
- ## Companion Animals and Health in Older Populations: A Systematic Review

  |  |  |
  | --- | --- |
  | Item Type | Journal Article |
  | Author | Michael J. Hughes |
  | Author | Martie-Louise Verreynne |
  | Author | Paul Harpur |
  | Author | Nancy A. Pachana |
  | Abstract | OBJECTIVES: . The aim of this systematic literature review (SLR) was to investigate the effect of companion animals (whether simply as pets or used in more formal intervention approaches) on the physical and mental health of older adults (aged 60+). METHODS: . The reviewers identified key search terms and conducted a systematic search of the PsycINFO and PubMed databases. The 70 articles reviewed were evaluated through tabular and thematic analysis. RESULTS: . In 52 of the studies examined, companion animals positively contributed to the mental and/or physical health of older adults. With respect to mental health, involvement with a companion animal improved participant quality of life and effectively attenuated symptoms of depression, anxiety, cognitive impairment, and the behavioral and psychiatric symptoms of dementia (BPSD). In relation to physical health, marked increases in physical activity and improvements in blood pressure and heart rate variability were the only consistent physical health improvements observed from companion animal interactions. CONCLUSIONS: . Animal companionship can benefit the mental and physical health of older adults, although more and better controlled research on this topic is required. CLINICAL IMPLICATIONS: . Use of companion animals has the potential to be an effective treatment or adjunct therapy to improve the health status and quality of life of older individuals. |
  | Date | 2020 |
  | Language | eng |
  | Short Title | Companion Animals and Health in Older Populations |
  | Library Catalogue | PubMed |
  | Volume | 43 |
  | Pages | 365-377 |
  | Publication | Clinical Gerontologist |
  | DOI | 10.1080/07317115.2019.1650863 |
  | Issue | 4 |
  | Journal Abbr | Clin Gerontol |
  | ISSN | 1545-2301 |
  | PMID | 31423915 |
  | Date Added | 05/02/2026, 16:20:09 |
  | Modified | 05/02/2026, 16:20:09 |

  ### Tags:

  - Pets
  - Animals
  - Humans
  - Aged
  - Animal Assisted Therapy
  - pet ownership
  - health
  - mental health
  - aging
  - Anxiety
  - Animal assisted therapy
  - older adult
  - animal assisted activity
  - animal companionship
  - Cognitive Dysfunction
  - Mental Health
  - Quality of Life

  ### Attachments

  - PubMed entry
- ## Palliative Care Matters: Lessons From the Loss of a Facility Dog

  |  |  |
  | --- | --- |
  | Item Type | Journal Article |
  | Author | Elizabeth Holman |
  | Author | Cari Levy |
  | Author | Brenda Kennedy |
  | Abstract | Animal-assisted intervention is gaining attention as a stress reduction modality. Quantitative data demonstrate its effectiveness, as a recent study published in AJHPM supported that a Veterans Affairs (VA) hospital facility dog paired with a palliative care psychologist had a measurable impact on salivary cortisol levels and heart rate in hospitalized veterans. There remains an important role for qualitative insights. The Denver VA palliative care team learned a range of lessons from the sudden loss of their facility dog, many of which relate directly to palliative care. The importance of communication, adjusting to changing teams, and the need for consultation and support based in shared goals and values all became evident in the course of the facility dog's illness. After her death, lessons shifted to grief and loss and how providers, patients, and a community care for each other. People's connection to the facility dog ultimately proved to be a critical factor in helping them connect with one another. |
  | Date | 2018-10 |
  | Language | eng |
  | Short Title | Palliative Care Matters |
  | Library Catalogue | PubMed |
  | Volume | 35 |
  | Pages | 1362-1364 |
  | Publication | The American Journal of Hospice & Palliative Care |
  | DOI | 10.1177/1049909118761387 |
  | Issue | 10 |
  | Journal Abbr | Am J Hosp Palliat Care |
  | ISSN | 1938-2715 |
  | PMID | 29514488 |
  | Date Added | 05/02/2026, 16:20:09 |
  | Modified | 05/02/2026, 16:20:09 |

  ### Tags:

  - Dogs
  - Pets
  - stress
  - Animals
  - Female
  - Adult
  - Humans
  - Male
  - Middle Aged
  - Aged
  - Animal Assisted Therapy
  - Stress, Psychological
  - animal-assisted intervention
  - United States
  - grief
  - Veterans
  - Aged, 80 and over
  - facility dog
  - Grief
  - Hospice and Palliative Care Nursing
  - Hospitals, Veterans
  - loss
  - Palliative Care
  - palliative care team
  - veterans

  ### Attachments

  - PubMed entry
- ## Animal-assisted therapy for patients in a minimally conscious state: A randomized two treatment multi-period crossover trial

  |  |  |
  | --- | --- |
  | Item Type | Journal Article |
  | Author | Karin Hediger |
  | Author | Milena Petignat |
  | Author | Rahel Marti |
  | Author | Margret Hund-Georgiadis |
  | Abstract | OBJECTIVE: To investigate if animal-assisted therapy (AAT) leads to higher consciousness in patients in a minimally conscious state during a therapy session, measured via behavioral reactions, heart rate and heart rate variability. METHODS: In a randomized two treatment multi-period crossover trial, 10 patients in a minimally conscious state participated in eight AAT sessions and eight paralleled conventional therapy sessions, leading to 78 AAT and 73 analyzed control sessions. Patients' responses during sessions were assessed via behavioral video coding and the Basler Vegetative State Assessment (BAVESTA), heart rate and heart rate variability (SDNN, RMSSD, HF and LF). Data were analyzed with generalized linear mixed models. RESULTS: Patients showed more eye movements (IRR = 1.31, 95% CI: 1.23 to 1.40, p < 0.001) and active movements per tactile input during AAT compared to control sessions (IRR = 1.13, 95% CI: 1.02 to 1.25, p = 0.018). No difference was found for positive emotions. With BAVESTA, patients' overall behavioral reactions were rated higher during AAT (b = 0.11, 95% CI: 0.01 to 0.22, p = 0.038). AAT led to significantly higher LF (b = 5.82, 95% CI: 0.55 to 11.08, p = 0.031) and lower HF (b = -5.80, 95% CI: -11.06 to -0.57, p = 0.030), while heart rate, SDNN, RMSSD did not differ. CONCLUSIONS: Patients in a minimally conscious state showed more behavioral reactions and increased physiological arousal during AAT compared to control sessions. This might indicate increased consciousness during therapeutic sessions in the presence of an animal. TRIAL REGISTRATION: ClinicalTrials.gov NCT02629302. |
  | Date | 2019 |
  | Language | eng |
  | Short Title | Animal-assisted therapy for patients in a minimally conscious state |
  | Library Catalogue | PubMed |
  | Volume | 14 |
  | Pages | e0222846 |
  | Publication | PloS One |
  | DOI | 10.1371/journal.pone.0222846 |
  | Issue | 10 |
  | Journal Abbr | PLoS One |
  | ISSN | 1932-6203 |
  | PMID | 31574106 |
  | PMCID | PMC6772068 |
  | Date Added | 05/02/2026, 16:20:09 |
  | Modified | 05/02/2026, 16:20:09 |

  ### Tags:

  - Animals
  - Female
  - Adult
  - Heart Rate
  - Humans
  - Male
  - Middle Aged
  - Aged
  - Animal Assisted Therapy
  - Cross-Over Studies
  - Persistent Vegetative State
  - Adolescent
  - Consciousness

  ### Attachments

  - Full Text
  - PubMed entry
- ## Physiological arousal for companion dogs working with their owners in animal-assisted activities and animal-assisted therapy

  |  |  |
  | --- | --- |
  | Item Type | Journal Article |
  | Author | Dorit Karla Haubenhofer |
  | Author | Sylvia Kirchengast |
  | Abstract | This study investigated the physiological reactions of companion dogs (Canis familiaris) used in animal-assisted activities and animal-assisted therapy by measuring salivary cortisol concentrations. The dog caregivers (owners) collected saliva samples (a) at 3 control days without therapeutic work, (b) directly before and after each therapeutic session during 3 consecutive months, and (c) again at 3 control days without therapeutic work. The study used an enzyme immunoassay to analyze the samples. Cortisol concentrations were significantly higher during therapy days than on control days. Dogs working during the first half of the day produced higher cortisol concentrations after therapeutic sessions than before, whereas dogs working in the afternoon produced lower cortisol concentrations. Cortisol concentrations were higher in short sessions than in long ones and increased relative to the number of therapeutic sessions done during the sampling period. The results indicate that therapeutic work was physiologically arousing for the dogs in this study. Whether these physiological responses are indicative of potentially negative stress or of positive excitement remains an open question. |
  | Date | 2006 |
  | Language | eng |
  | Library Catalogue | PubMed |
  | Volume | 9 |
  | Pages | 165-172 |
  | Publication | Journal of applied animal welfare science: JAAWS |
  | DOI | 10.1207/s15327604jaws0902\_5 |
  | Issue | 2 |
  | Journal Abbr | J Appl Anim Welf Sci |
  | ISSN | 1088-8705 |
  | PMID | 16956319 |
  | Date Added | 05/02/2026, 16:23:23 |
  | Modified | 05/02/2026, 16:23:23 |

  ### Tags:

  - Hydrocortisone
  - Dogs
  - Animals
  - Female
  - Humans
  - Male
  - Stress, Psychological
  - Saliva
  - Work

  ### Attachments

  - PubMed entry
- ## Effects of Animal-Assisted Therapy (AAT) in Alzheimer's Disease: A Case Study

  |  |  |
  | --- | --- |
  | Item Type | Journal Article |
  | Author | Armando Gregorini |
  | Author | Angela Di Canio |
  | Author | Emanuele Palmucci |
  | Author | Marco Tomasetti |
  | Author | Marco B. L. Rocchi |
  | Author | Mariastella Colomba |
  | Abstract | Alzheimer's disease (AD) is a neurodegenerative disorder, characterized by cortical dementia and irreversibly progressive developments leading to a vegetative state and, finally, to death. Although many aspects of its etiology, diagnosis and treatment still remain obscure and the current approach to the disease mostly suffers from limited and low-efficiency therapeutic means, nevertheless, recent interventions have aimed at improving patients' quality of life through nonpharmacological approaches, including animal-assisted therapy (AAT), arousing growing interest. In order to assess the physiological and neuropsychological effects of AAT on AD, 24 residents of a rest house in northern Italy were enrolled. The intervention consisted of one 45-minute AAT session per week over ten weeks. Twelve residents (six AD and six non-AD) received AAT and twelve (six AD and six non-AD) were controls. In order to evaluate the physiological and clinical effect of AAT on AD residents, three cardiac parameters, including the systolic and diastolic blood pressure and heart rate, were measured. Moreover, the neurocognitive and depressive states were assessed by the Mini Mental State Examination and the Geriatric Depression Scale, respectively. Analyses were performed by a four-way ANOVA model (including two ways for repeated measures) considering each main effect and interaction possible in the design. Our findings, despite the small sample size, suggest that AAT has a positive significant effect on physiological parameters and neurocognitive impairment, while no effect was observed on the depression level. |
  | Date | 2022-03-18 |
  | Language | eng |
  | Short Title | Effects of Animal-Assisted Therapy (AAT) in Alzheimer's Disease |
  | Library Catalogue | PubMed |
  | Volume | 10 |
  | Place | Basel, Switzerland |
  | Pages | 567 |
  | Publication | Healthcare |
  | DOI | 10.3390/healthcare10030567 |
  | Issue | 3 |
  | Journal Abbr | Healthcare (Basel) |
  | ISSN | 2227-9032 |
  | PMID | 35327045 |
  | PMCID | PMC8950375 |
  | Date Added | 05/02/2026, 16:20:09 |
  | Modified | 05/02/2026, 16:20:09 |

  ### Tags:

  - heart rate
  - cognitive and behavioral interventions
  - GDS
  - MMSE
  - neurodegenerative disorders
  - systolic and diastolic blood pressure

  ### Attachments

  - Full Text
  - PubMed entry
- ## Do Animals Perceive Human Developmental Disabilities? Guinea Pigs' Behaviour with Children with Autism Spectrum Disorders and Children with Typical Development. A Pilot Study

  |  |  |
  | --- | --- |
  | Item Type | Journal Article |
  | Author | Marine Grandgeorge |
  | Author | Elodie Dubois |
  | Author | Zarrin Alavi |
  | Author | Yannig Bourreau |
  | Author | Martine Hausberger |
  | Abstract | Some cues used by humans and animals during human-animal interactions may have significant effects, modulating these interactions (e.g., gaze direction, heart rate). This study aimed to determine whether an animal in human-animal interactions is capable of "perceiving" its human partner's potential developmental "disabilities". To test this hypothesis, we studied guinea pigs (GP) behaviours in the presence of 44 6-to-12-year-old children with either typical development (TD children) or with autism spectrum disorders (ASD children). Thus, we recorded the GP behaviours during the entire session (to establish their time budget) and focused in particular on the onset and end of physical interactions. The GP behaviours (e.g., feeding, resting, self-grooming, exploring) were not significantly different between the two groups of children during the whole session. GP behaviours in the presence of children differed slightly when encountering ASD children versus TD children: more positive behaviours toward ASD children at the onset, more feeding and resting in the presence of TD children toward the end of an interaction. TD children showed longer-lasting interactions. One could explain this by GP curiosity toward ASD children behaviours (e.g., no marked behaviours such as attempts to touch), whereas GPs seemed calmer at the end with TD children (i.e., interacting with ASD children may be a little stressful). This partly gave support to our study's hypothesis. GPs seemed to perceive developmental disabilities during a first encounter with children and to adjust their behaviours to that of children. We discuss the issues of animal training, animals' well-being and acute stress, whether they are pets or used in animal-assisted interventions. Further studies (on pets or animal-assisted interventions) are warranted. |
  | Date | 2019-08-02 |
  | Language | eng |
  | Short Title | Do Animals Perceive Human Developmental Disabilities? |
  | Library Catalogue | PubMed |
  | Volume | 9 |
  | Pages | 522 |
  | Publication | Animals: an open access journal from MDPI |
  | DOI | 10.3390/ani9080522 |
  | Issue | 8 |
  | Journal Abbr | Animals (Basel) |
  | ISSN | 2076-2615 |
  | PMID | 31382429 |
  | PMCID | PMC6719160 |
  | Date Added | 05/02/2026, 16:23:23 |
  | Modified | 05/02/2026, 16:23:23 |

  ### Tags:

  - interaction
  - behaviour
  - children
  - animal assisted intervention
  - autism spectrum disorders
  - guinea pig
  - interspecific

  ### Attachments

  - Full Text
  - PubMed entry
- ## Therapy Dog Welfare Revisited: A Review of the Literature

  |  |  |
  | --- | --- |
  | Item Type | Journal Article |
  | Author | Lisa Maria Glenk |
  | Author | Sandra Foltin |
  | Abstract | During the past decade, the field of human-animal interaction(s) research has been characterized by a significant increase in scientific findings. These data have contributed to our current understanding of how humans may benefit from contact with animals. However, the animal experience of these interactions is still an under-researched area. This paper addresses the welfare of dogs who participate in animal-assisted interventions (AAIs) to improve health in human recipients. This paper builds on previous work by Glenk (2017) and provides an updated review of the literature on therapy dog welfare published from 2017-2021. New advances in scientific methodology, such as the determination of salivary oxytocin, breath rate and tympanic membrane temperature, are analyzed regarding their value and limitations for research in AAIs. Moreover, welfare-related social and environmental factors (e.g., freedom of choice, exploration of novel environments, inequity aversion, individual development, working experience, relationship with handler and handler skills) that profoundly influence dog perception and well-being are reviewed and discussed. Accounting for the globally increasing interest and the number of dogs utilized in AAIs, safeguarding therapy dog well-being, and identifying situations, circumstances and protocols that may challenge animal welfare remains an emerging and crucial area of scientific effort. |
  | Date | 2021-10-12 |
  | Language | eng |
  | Short Title | Therapy Dog Welfare Revisited |
  | Library Catalogue | PubMed |
  | Volume | 8 |
  | Pages | 226 |
  | Publication | Veterinary Sciences |
  | DOI | 10.3390/vetsci8100226 |
  | Issue | 10 |
  | Journal Abbr | Vet Sci |
  | ISSN | 2306-7381 |
  | PMID | 34679056 |
  | PMCID | PMC8538106 |
  | Date Added | 05/02/2026, 16:20:09 |
  | Modified | 05/02/2026, 16:20:09 |

  ### Tags:

  - welfare
  - stress
  - dog
  - animal-assisted intervention
  - canine
  - therapy

  ### Attachments

  - Full Text
  - PubMed entry
- ## Current Perspectives on Therapy Dog Welfare in Animal-Assisted Interventions

  |  |  |
  | --- | --- |
  | Item Type | Journal Article |
  | Author | Lisa Maria Glenk |
  | Abstract | Research into the effects of animal-assisted interventions (AAIs) has primarily addressed human health outcomes. In contrast, only few publications deal with the therapy dog experience of AAIs. This paper provides an overview on potential welfare threats that therapy dogs may encounter and presents the results of a review of available studies on welfare indicators for therapy dogs during AAIs. Previous investigations used physiological and behavioral welfare indicators and dog handler surveys to identify work-related stress. Research outcomes are discussed in the light of strengths and weaknesses of the methods used. Study results suggest that frequency and duration of AAI sessions, novelty of the environment, controllability, age and familiarity of recipients modulate animal welfare indicators. However, this review reveals that currently, clear conclusions on how the well-being of dogs is influenced by the performance in AAIs are lacking due to the heterogeneity of programs, recipient and session characteristics, small dog sample sizes and methodological limitations. This paper further aimed to identify unresolved difficulties in previous research to pave the way for future investigations supporting the applicability of scientific findings in practice. |
  | Date | 2017-02-01 |
  | Language | eng |
  | Library Catalogue | PubMed |
  | Volume | 7 |
  | Pages | 7 |
  | Publication | Animals: an open access journal from MDPI |
  | DOI | 10.3390/ani7020007 |
  | Issue | 2 |
  | Journal Abbr | Animals (Basel) |
  | ISSN | 2076-2615 |
  | PMID | 28157145 |
  | PMCID | PMC5332928 |
  | Date Added | 05/02/2026, 16:20:09 |
  | Modified | 05/02/2026, 16:20:09 |

  ### Tags:

  - cortisol
  - stress
  - animal welfare
  - animal-assisted therapy
  - behavior
  - animal-assisted intervention
  - animal-assisted activity
  - therapy dog

  ### Attachments

  - Full Text
  - PubMed entry
- ## Well-Being Indicators in Autistic Children and Therapy Dogs During a Group Intervention: A Pilot Study

  |  |  |
  | --- | --- |
  | Item Type | Journal Article |
  | Author | Viviana Orsola Giuliano |
  | Author | Luigi Sacchettino |
  | Author | Alina Simona Rusu |
  | Author | Davide Ciccarelli |
  | Author | Valentina Gazzano |
  | Author | Martina de Cesare |
  | Author | Michele Visone |
  | Author | Vincenzo Mizzoni |
  | Author | Francesco Napolitano |
  | Author | Danila d'Angelo |
  | Abstract | Animal-assisted services (AAS) have been shown in multiple studies to improve a range of human psychological and physical health benefits. The aim of this pilot study is to investigate simultaneously two psycho-physiological indicators of the valence of interactions in the context of dog-assisted activities in children diagnosed with autism spectrum disorder. Ten children and four dogs experienced in AAS were involved, lasting 90 days, in weekly one-hour sessions. Before and after each session, saliva was taken in both dogs and children for determination of salivary oxytocin and cortisol levels. In addition, at the end of the program, a questionnaire was administered to both parents and dog handlers to assess the impact of AAS in children and dogs. Our results revealed no statistically significant change in cortisol and oxytocin levels in dogs enrolled throughout the sessions, while an increasing trend was noted for salivary oxytocin in 50% of the dogs and for salivary cortisol in all dogs at the end of the AAS, when compared to the pre-AAS. Salivary cortisol measurement in children with an autistic neurotype highlighted a statistically significant increase at the end of the AAS when compared to the pre-AAS, but this was not observed for oxytocin level evaluations. Regarding the perception of the children's parents about the effects of the program, our data reported an improvement in sociability of the children in 100 percent of the cases. Furthermore, dog handlers reported an absence of signs of stress in their dogs during the sessions. Although the perceived effectiveness and quality of AAS has been demonstrated in the literature, the need to carefully select the dogs involved, considering their skills and needs, is critical to ensure their well-being in various therapeutic settings. |
  | Date | 2025-07-10 |
  | Language | eng |
  | Short Title | Well-Being Indicators in Autistic Children and Therapy Dogs During a Group Intervention |
  | Library Catalogue | PubMed |
  | Volume | 15 |
  | Pages | 2032 |
  | Publication | Animals: an open access journal from MDPI |
  | DOI | 10.3390/ani15142032 |
  | Issue | 14 |
  | Journal Abbr | Animals (Basel) |
  | ISSN | 2076-2615 |
  | PMID | 40723495 |
  | PMCID | PMC12291636 |
  | Date Added | 05/02/2026, 16:23:23 |
  | Modified | 05/02/2026, 16:23:23 |

  ### Tags:

  - cortisol
  - oxytocin
  - human–animal relationship
  - animal-assisted intervention
  - animal assisted services
  - dog welfare

  ### Attachments

  - PubMed entry
- ## Hippotherapy in the Treatment of CMD and Bruxism in Dentistry

  |  |  |
  | --- | --- |
  | Item Type | Journal Article |
  | Author | Margrit-Ann Geibel |
  | Author | Daniela Kildal |
  | Author | Amina Maria Geibel |
  | Author | Sibylle Ott |
  | Abstract | Dysfunctions and disorders of the craniomandibular system are accompanied by pathophysiological changes of muscle groups in the throat/neck and facial area, e.g., pain in the jaw and muscles of mastication and disturbance of occlusion, leading to teeth injury (loss of dental hard tissue, fractures/sensibility disorders, etc.). For muscular dysfunctions, even in the context of psychosomatic disorders and chronic stress, hippotherapy is particularly suitable, since it helps actively to relieve muscle tensions. In the current project we combined hippotherapy with progressive muscle relaxation (PMR) to achieve a synergistic effect. The horses used for therapy (two mares and five geldings between seven and twenty-one years old) were especially suitable because of their calm temperament. In two cases, trained therapy horses were used; in five other cases, the patients used their own horses, which were not specially trained. Right from the beginning, the project was accompanied by veterinary support. Conditions of horse keeping (active stable, same-sex groups, no boxes) were assessed as well as the horses themselves prior to, during, and after each therapy unit. In patients, cortisol, as a quantifiable parameter for stress, was measured before and after each therapy unit. From before the start until the end of each therapy unit of 15 min, the heart rate variability (HRV) of both patients and horses was registered continuously and synchronously. In addition, the behavior of the horses was monitored and recorded on video by an experienced coach and a veterinarian. The stress load during the tension phases in the therapy units was low, perceivable in the horses lifting their heads and a slightly shortened stride length. Likewise, the horses reflected the patients' relaxation phases, so that at the end of the units the horses were physically and psychically relaxed, too, noticeable by lowering their necks, free ear movement, and a decreasing heart frequency (HF). Altogether, the horses benefited from the treatment, too. Obvious stress signs like unrest, head tossing, tail swishing, or tense facial expressions were not noticed at any time. Twenty jumpers served as a control group in different situations (training, tournament, and leisure riding). |
  | Date | 2025-09-03 |
  | Language | eng |
  | Library Catalogue | PubMed |
  | Volume | 15 |
  | Pages | 2587 |
  | Publication | Animals: an open access journal from MDPI |
  | DOI | 10.3390/ani15172587 |
  | Issue | 17 |
  | Journal Abbr | Animals (Basel) |
  | ISSN | 2076-2615 |
  | PMID | 40941382 |
  | PMCID | PMC12427267 |
  | Date Added | 05/02/2026, 16:23:23 |
  | Modified | 05/02/2026, 16:23:23 |

  ### Tags:

  - animal welfare
  - stress reduction
  - hippotherapy
  - animal assisted therapy
  - bruxism in dentistry
  - CMD (craniomandibular dysfunction)
  - equine therapy
  - horse assisted therapy
  - HRV (heart rate variability)
  - stress index
  - stress signals

  ### Attachments

  - PubMed entry
- ## Distraction-focused interventions on examination stress in nursing students: Effects on psychological stress and biomarker levels. A randomized controlled trial

  |  |  |
  | --- | --- |
  | Item Type | Journal Article |
  | Author | Verena Gebhart |
  | Author | Waltraud Buchberger |
  | Author | Isabella Klotz |
  | Author | Sabrina Neururer |
  | Author | Christine Rungg |
  | Author | Gerhard Tucek |
  | Author | Christoph Zenzmaier |
  | Author | Susanne Perkhofer |
  | Abstract | BACKGROUND: Nursing students all over the world experience high levels of stress with negative impacts on their health, emotional state and performance. AIM: This study aimed to investigate the effects of distraction-focused interventions on examination stress and anxiety in nursing students. METHODS: A randomized controlled, parallel trial design was conducted from January to June 2016. After baseline measurement, 72 participants were randomized to one of the following groups (n = 18 each): (i) animal-assisted therapy; (ii) music therapy; (iii) mandala painting; (iv) control group. Outcomes of all groups in terms of stress-reduction were compared by measuring self-reported perceived stress (STAI-State and visual analogue stress scale) and salivary biomarker levels (Cortisol and Immunoglobulin A). RESULTS: Fifty-seven complete data sets (n = 12-16 for each group) were analysed. All distraction-focused interventions showed stress and anxiety reduction in everyday school situations. By contrast, on days with examinations, stress reductions did not reach statistical significance in regard to self-reported psychological stress. At the same time, interventions resulted in significantly decreased levels of stress biomarkers (P < .001). CONCLUSIONS: Our preliminary findings suggest positive but situation-dependent effects of distraction-focused interventions in academic settings. Further research should investigate the complex relationship between physiological and psychological stress parameters. |
  | Date | 2020-02 |
  | Language | eng |
  | Short Title | Distraction-focused interventions on examination stress in nursing students |
  | Library Catalogue | PubMed |
  | Volume | 26 |
  | Pages | e12788 |
  | Publication | International Journal of Nursing Practice |
  | DOI | 10.1111/ijn.12788 |
  | Issue | 1 |
  | Journal Abbr | Int J Nurs Pract |
  | ISSN | 1440-172X |
  | PMID | 31724291 |
  | Date Added | 05/02/2026, 16:23:23 |
  | Modified | 05/02/2026, 16:23:23 |

  ### Tags:

  - Hydrocortisone
  - Female
  - Adult
  - Humans
  - Male
  - Middle Aged
  - Young Adult
  - Animal Assisted Therapy
  - Stress, Psychological
  - Saliva
  - anxiety
  - Biomarkers
  - physiological stress
  - randomized controlled trial
  - Immunoglobulin A
  - Adolescent
  - Anxiety
  - Art Therapy
  - distraction
  - Educational Measurement
  - Music Therapy
  - nursing
  - psychological stress
  - Students, Nursing

  ### Attachments

  - PubMed entry
- ## Equine-assisted therapeutic activities and their influence on the heart rate variability: A systematic review

  |  |  |
  | --- | --- |
  | Item Type | Journal Article |
  | Author | Andrés García-Gómez |
  | Author | Eloísa Guerrero-Barona |
  | Author | Inés García-Peña |
  | Author | Marta Rodríguez-Jiménez |
  | Author | Juan Manuel Moreno-Manso |
  | Abstract | OBJECTIVE: To examine the effect of equine-assisted therapeutic interventions on users' heart rate variability, using this said variability as an objective biological variable related to stress levels. METHOD: A systematic review has been carried out using the methodology suggested in the PRISMA declaration following systematic searches in academic databases. RESULTS: 432 registers were initially identified; however, in the screening and suitability process, nine papers were included in the review. With one exception, all of them reported that equine-assisted therapeutic activities had a favourable effect on users' heart rate variability as such activities favour a state of relaxation by activating the Parasympathetic Nervous System. The analysis of the quality of the evidence and the confirmation of the bias in the works indicate that these results must be considered with caution. DISCUSSION: Although these preliminary results are promising, more rigorous clinical trials are necessary to overcome the methodological limitations of the works. |
  | Date | 2020-05 |
  | Language | eng |
  | Short Title | Equine-assisted therapeutic activities and their influence on the heart rate variability |
  | Library Catalogue | PubMed |
  | Volume | 39 |
  | Pages | 101167 |
  | Publication | Complementary Therapies in Clinical Practice |
  | DOI | 10.1016/j.ctcp.2020.101167 |
  | Journal Abbr | Complement Ther Clin Pract |
  | ISSN | 1873-6947 |
  | PMID | 32379693 |
  | Date Added | 05/02/2026, 16:20:09 |
  | Modified | 05/02/2026, 16:20:09 |

  ### Tags:

  - Heart rate variability
  - Animals
  - Heart Rate
  - Humans
  - Animal Assisted Therapy
  - Stress, Psychological
  - Systematic review
  - Horses
  - Stress
  - Autonomic Nervous System
  - Autonomous nervous system
  - Equine-assisted therapeutic activities

  ### Attachments

  - PubMed entry
- ## How the presence of a dog and types of interaction affect physiological responses to experimental heat pain induction in healthy humans - a randomized controlled study

  |  |  |
  | --- | --- |
  | Item Type | Journal Article |
  | Author | Lene Høeg Fuglsang-Damgaard |
  | Author | Sigrid Juhl Lunde |
  | Author | Janne Winther Christensen |
  | Author | Lene Vase |
  | Author | Poul B. Videbech |
  | Author | Nancy R. Gee |
  | Author | Karen Thodberg |
  | Abstract | It has become increasingly popular to include dogs as a complement to regular therapy, with the expectation that they offer, among other benefits, pain-relieving effects. Meanwhile, studies covering the topic of painful situations within the field of animal-assisted interventions (AAI) present conflicting results and rarely consider the type and duration of interaction with the dog. Thus, the impact of human-dog interactions on physiological measurements during painful situations is largely unknown. Basic research is needed on the effects of interacting with a dog, using commonly applied immediate physiological measurements in healthy humans during experimental pain induction to fill this gap in the literature. The present study investigated how AAI influences physiological measurements when healthy humans are subjected to experimental heat pain induction. Simultaneously, the study explored how the duration of different types of interaction with a human companion or a dog as well as dog behavior during experimental heat pain induction affected physiological measurements. Fifty-eight healthy participants (14 men, 44 women, age: 18-66 years) were randomly assigned to one of two intervention groups: 1) a dog and a human companion or 2) a human companion only. Both intervention groups underwent two test conditions in a balanced order: an active test condition with their allocated intervention and a control test condition without their allocated intervention. The participants were exposed to a 5-minute heat pain induction trial in both test conditions with a 20-minute break between trials. Heart rate (HR), heart rate variability (HRV) and skin conductance (SC), analyzed as tonic level (SCL) and peak counts (SCR), were continuously recorded. Blood pressure (BP) and salivary cortisol (s-cortisol) were collected as pre- and post-measurements for each test condition. Behavioral interactions between the participant, dog and human companion as well as behavior of the dog were recorded and the influence of the behavioral interactions on each physiological measure was analyzed. Linear Mixed Models were applied. HR was higher for the intervention group with a dog and a human companion compared to a human companion only (p=0.013). Additionally, within-subject comparison showed an increased HR during the active condition compared to the control condition in both intervention groups (dog and human companion: p<0.001 and human companion only: p=0.025). None of the other physiological measurements were influenced by the mere presence of a dog in either the between- nor within-subject comparisons. Within the human companion only group, SCL and SCR were higher during the active condition compared to the control (both p<0.001). The duration of the behavioral interaction between the participant and human companion variously influenced HRV, SBP, s-cortisol and SCL during the active condition in both intervention groups. Further, the duration of the behavioral interactions with the dog and dog behavior variously influenced HR, HRV, s-cortisol and SCL during the active condition in the human companion and the dog intervention group. In conclusion, this study shows that the presence of a dog in addition to a human companion during experimental heat pain induction results in an increased HR compared to the presence of a human companion only. Thus, despite previously reported pain-relieving effects of the presence of a dog, this study suggests that dog presence may also induce a certain level of arousal and further studies are needed to explore the causal mechanisms. Interactions with a dog or a human companion influenced several physiological measurements and it is therefore important to quantify the type and duration of human-animal interaction in AAI-studies. |
  | Date | 2025-12-01 |
  | Language | eng |
  | Library Catalogue | PubMed |
  | Volume | 302 |
  | Pages | 115097 |
  | Publication | Physiology & Behavior |
  | DOI | 10.1016/j.physbeh.2025.115097 |
  | Journal Abbr | Physiol Behav |
  | ISSN | 1873-507X |
  | PMID | 40939729 |
  | Date Added | 05/02/2026, 16:23:23 |
  | Modified | 05/02/2026, 16:23:23 |

  ### Tags:

  - Hydrocortisone
  - Dogs
  - Heart rate variability
  - Animals
  - Female
  - Adult
  - Heart Rate
  - Human-Animal Bond
  - Humans
  - Male
  - Middle Aged
  - Young Adult
  - Aged
  - Animal Assisted Therapy
  - Blood Pressure
  - Animal-assisted intervention
  - Human-animal interaction
  - Blood pressure
  - Salivary cortisol
  - Skin conductance
  - Adolescent
  - Pain
  - Pain Measurement
  - Galvanic Skin Response
  - Hot Temperature
  - Thermal pain stimuli

  ### Attachments

  - PubMed entry
- ## A System for Assessment of Canine-Human Interaction during Animal-Assisted Therapies

  |  |  |
  | --- | --- |
  | Item Type | Journal Article |
  | Author | Marc Foster |
  | Author | Eric Beppler |
  | Author | Timothy Holder |
  | Author | James Dieffenderfer |
  | Author | Patrick Erb |
  | Author | Kristy Everette |
  | Author | Margaret Gruen |
  | Author | Tamara Somers |
  | Author | Tom Evans |
  | Author | Michael Daniele |
  | Author | David L. Roberts |
  | Author | Alper Bozkurt |
  | Abstract | Animal-assisted therapies (AAT) are becoming increasingly common to help hospitalized patients, especially in oncology units. There is a critical need for methods and technologies that can enable a quantifiable understanding of AAT to objectively demonstrate its efficacy and improve its efficiency. In this paper, we present our preliminary efforts towards the development of wireless sensor systems to simultaneously detect the related behavioral (activity level, movement, stroking) and physiological signals (heart rate/variability) of humans and animals during their interaction. To detect heart rate, we tested two different techniques based on wearable or contactless electrocardiography. In this preliminary evaluation, we were able to assess these parameters successfully and identify the design challenges towards deployment of these systems in larger clinical studies. |
  | Date | 2018-07 |
  | Language | eng |
  | Library Catalogue | PubMed |
  | Volume | 2018 |
  | Pages | 4347-4350 |
  | Publication | Annual International Conference of the IEEE Engineering in Medicine and Biology Society. IEEE Engineering in Medicine and Biology Society. Annual International Conference |
  | DOI | 10.1109/EMBC.2018.8513384 |
  | Journal Abbr | Annu Int Conf IEEE Eng Med Biol Soc |
  | ISSN | 2694-0604 |
  | PMID | 30441316 |
  | Date Added | 05/02/2026, 16:23:23 |
  | Modified | 05/02/2026, 16:23:23 |

  ### Tags:

  - Dogs
  - Animals
  - Heart Rate
  - Humans
  - Animal Assisted Therapy
  - Electrocardiography

  ### Attachments

  - PubMed entry
- ## Effects of Animal-Assisted Therapy on Hospitalized Children and Teenagers: A Systematic Review and Meta-Analysis

  |  |  |
  | --- | --- |
  | Item Type | Journal Article |
  | Author | Yongshen Feng |
  | Author | Yeqing Lin |
  | Author | Ningning Zhang |
  | Author | Xiaohan Jiang |
  | Author | Lifeng Zhang |
  | Abstract | PROBLEM: Psychological and symptom disturbances seriously affect hospitalized children's subjective experiences of hospitalization and their prognosis. We systematically reviewed the effects of animal-assisted therapy (AAT) on pain, anxiety, depression, stress, blood pressure (BP), and heart rate (HR) in hospitalized children and teenagers. ELIGIBILITY CRITERIA: A systematic review and meta-analysis were conducted using the English-language electronic databases PubMed, EMBASE, Web of Science, the Cochrane Library, Clinical Trials, Science Direct, EBSCOhost, Open Grey and Google Scholar, and the Chinese databases CNKI, Sinomed, Vip, and WanFang. These databases were searched through July 15, 2020. SAMPLE: Eight studies, including four randomized controlled trials (RCTs) and four quasi-experimental studies were included, with a total of 348 participants. RESULTS: Hospitalized children and teenagers with AAT had less pain (standardized mean difference = -0.49; 95% confidence interval [CI], -0.77 to -0.22; P < 0.001), lower systolic blood pressure (mean difference [MD] = -4.85; 95% CI, -9.50 to -0.21; P= 0.04), higher diastolic blood pressure (MD = 4.95; 95% CI, 1.90 to 8.00; P = 0.001) than controls, while there was no significant difference in depression, anxiety, stress, or HR. CONCLUSION: As an adjuvant to traditional treatment, AAT was beneficial for controlling pain and BP in hospitalized children and teenagers. IMPLICATION: AAT may be an effective strategy for relieving pain and controlling BP in hospitalized children and teenagers, especially those with cancer. High-quality RCTs conducted or supported by nurses on the effects of AAT are needed. |
  | Date | 2021 |
  | Language | eng |
  | Short Title | Effects of Animal-Assisted Therapy on Hospitalized Children and Teenagers |
  | Library Catalogue | PubMed |
  | Volume | 60 |
  | Pages | 11-23 |
  | Publication | Journal of Pediatric Nursing |
  | DOI | 10.1016/j.pedn.2021.01.020 |
  | Journal Abbr | J Pediatr Nurs |
  | ISSN | 1532-8449 |
  | PMID | 33582447 |
  | Date Added | 05/02/2026, 16:20:09 |
  | Modified | 05/02/2026, 16:20:09 |

  ### Tags:

  - Animals
  - Humans
  - Animal Assisted Therapy
  - Child
  - Adolescent
  - Animal-assisted therapy
  - Anxiety
  - Child, Hospitalized
  - Hospitalization
  - Meta-analysis
  - Neoplasms
  - Pain
  - Pain Management

  ### Attachments

  - PubMed entry
- ## Animal-assisted therapy: paws with a cause

  |  |  |
  | --- | --- |
  | Item Type | Journal Article |
  | Author | Lorraine S. Ernst |
  | Abstract | Ranger, the cardiac Pet Therapy standard poodle, was called upon by a family member to visit Mrs. M, a patient hospitalized for worsening heart failure. Although short of breath, Mrs. M started talking to Ranger as he rested quietly on the bed beside her. She told him in a soft voice that she knew she was sick but "you, my friend, give me strength and courage." Mrs. M died 1 week later. Soon after, the family stated in a survey that the interaction between Mrs. M and Ranger was very important to Mrs. M and that she had looked forward to her visits with Ranger. Mrs. M indicated to her family that Ranger made her feel calm and protected as she faced her illness. |
  | Date | 2013-03 |
  | Language | eng |
  | Short Title | Animal-assisted therapy |
  | Library Catalogue | PubMed |
  | Volume | 44 |
  | Pages | 16-19; quiz 20 |
  | Publication | Nursing Management |
  | DOI | 10.1097/01.NUMA.0000427181.19436.19 |
  | Issue | 3 |
  | Journal Abbr | Nurs Manage |
  | ISSN | 1538-8670 |
  | PMID | 23392285 |
  | Date Added | 05/02/2026, 16:20:09 |
  | Modified | 05/02/2026, 16:20:09 |

  ### Tags:

  - Dogs
  - Animals
  - Female
  - Humans
  - Male
  - Animal Assisted Therapy
  - Stress, Psychological
  - Emotions
  - Communication
  - Treatment Outcome
  - Heart Failure
  - Patient Safety
  - Practice Guidelines as Topic
  - Program Development

  ### Attachments

  - PubMed entry
- ## The effect of pet therapy on the physiological and subjective stress response: A meta-analysis

  |  |  |
  | --- | --- |
  | Item Type | Journal Article |
  | Author | Natalie Ein |
  | Author | Lingqian Li |
  | Author | Kristin Vickers |
  | Abstract | Studies have reported that exposure to pet therapy (PT) can reduce physiological and subjective stress and anxiety levels. The aim of this meta-analysis is to examine the efficacy of PT as a method for reducing physiological stress levels (blood pressure and heart rate) and subjective stress and anxiety scores (self-reported stress/anxiety). Further, we examined the effects of sample characteristics and modifications to the PT (different age groups and health status of participants across samples, whether a stressor was present, and individual versus group PT) as potential moderators of the relationship between PT and stress reactivity. Our searches incorporated articles published from May 2017 and earlier in PsycINFO, MEDLINE, and PubMed. This meta-analysis included 28 articles with 34 independent samples and contained a total of 1,310 participants. Using a random effects model, we determined that significant differences occurred in heart rate, self-reported anxiety, and self-reported stress after PT exposure compared with before PT. However, we did not detect significant differences in blood pressure after PT. Sample characteristics and modifications to the PT significantly moderated the effect of PT on stress responses. Our results suggest that PT can be an effective program for reducing stress reactivity. |
  | Date | 2018-10 |
  | Language | eng |
  | Short Title | The effect of pet therapy on the physiological and subjective stress response |
  | Library Catalogue | PubMed |
  | Volume | 34 |
  | Pages | 477-489 |
  | Publication | Stress and Health: Journal of the International Society for the Investigation of Stress |
  | DOI | 10.1002/smi.2812 |
  | Issue | 4 |
  | Journal Abbr | Stress Health |
  | ISSN | 1532-2998 |
  | PMID | 29882342 |
  | Date Added | 05/02/2026, 16:20:09 |
  | Modified | 05/02/2026, 16:20:09 |

  ### Tags:

  - blood pressure
  - stress
  - Heart Rate
  - Humans
  - Animal Assisted Therapy
  - animal-assisted therapy
  - Blood Pressure
  - Stress, Psychological
  - heart rate
  - pet therapy
  - Anxiety
  - Outcome Assessment, Health Care

  ### Attachments

  - PubMed entry
- ## Animal-Assisted Stress Management for Veterinary Staff

  |  |  |
  | --- | --- |
  | Item Type | Journal Article |
  | Author | Yvonne M. Eaton-Stull |
  | Author | Christopher Streidl |
  | Author | Batya G. Jaffe |
  | Author | Sarah Kuehn |
  | Author | Alexandra Kaufman |
  | Abstract | High levels of stress have a detrimental impact on veterinary staff, negatively influencing their mental health and contributing to high rates of suicide. Veterinary social workers are tasked with providing interventions to reverse these consequences and support the professional's health and well-being. Twenty-one veterinary staff participated in a study to evaluate the impact of animal-assisted support. Over three months, participants attended one or two therapy dog visits per month, interacting with therapy dogs for a minimum of 10 minutes each session. Pre- and postmeasures of blood pressure, heart rate, and self-reported stress were taken at each session. A measure to assess compassion fatigue and satisfaction was administered at the end of each session, and at the conclusion of the study participants' thoughts about the visits from the therapy dogs were assessed. Significant reductions in systolic and diastolic blood pressure were found as well as significantly lower ratings of self-reported stress. Additionally, participants looked forward to these visits stating they made them happy, helped them feel better, and took their mind off their troubles. Considering the growing utilization of therapy dogs, this timely study adds to the body of evidence, highlighting the benefits of animal-assisted interventions with veterinarian staff. |
  | Date | 2024-11-01 |
  | Language | eng |
  | Library Catalogue | PubMed |
  | Volume | 49 |
  | Pages | 219-226 |
  | Publication | Health & Social Work |
  | DOI | 10.1093/hsw/hlae025 |
  | Issue | 4 |
  | Journal Abbr | Health Soc Work |
  | ISSN | 1545-6854 |
  | PMID | 39265989 |
  | Date Added | 05/02/2026, 16:20:09 |
  | Modified | 05/02/2026, 16:20:09 |

  ### Tags:

  - Dogs
  - Animals
  - Female
  - Adult
  - Heart Rate
  - Humans
  - Male
  - Middle Aged
  - Animal Assisted Therapy
  - Blood Pressure
  - Stress, Psychological
  - animal-assisted intervention
  - stress management
  - Occupational Stress
  - support
  - therapy dogs
  - Veterinarians
  - veterinary staff

  ### Attachments

  - PubMed entry
- ## Effect of Canine Play Interventions as a Stress Reduction Strategy in College Students

  |  |  |
  | --- | --- |
  | Item Type | Journal Article |
  | Author | Cheryl Delgado |
  | Author | Margaret Toukonen |
  | Author | Corinne Wheeler |
  | Abstract | Forty-eight students engaged with a therapy dog for 15 minutes during finals week to evaluate the effect on stress. Psychological (Perceived Stress Scale, visual analog scales) and physiologic stress (vital signs, salivary cortisol) measures were collected before and after the intervention. Paired t tests showed significant reductions in all psychological and physiologic measures except diastolic blood pressure. This supports animal-assisted therapy as an effective stress management strategy for nursing and other college students. |
  | Date | 2018 |
  | Language | eng |
  | Library Catalogue | PubMed |
  | Volume | 43 |
  | Pages | 149-153 |
  | Publication | Nurse Educator |
  | DOI | 10.1097/NNE.0000000000000451 |
  | Issue | 3 |
  | Journal Abbr | Nurse Educ |
  | ISSN | 1538-9855 |
  | PMID | 28857956 |
  | Date Added | 05/02/2026, 16:23:23 |
  | Modified | 05/02/2026, 16:23:23 |

  ### Tags:

  - Hydrocortisone
  - Dogs
  - Animals
  - Female
  - Adult
  - Humans
  - Male
  - Middle Aged
  - Young Adult
  - Animal Assisted Therapy
  - Stress, Psychological
  - Saliva
  - Stress, Physiological
  - Students
  - Adolescent
  - Treatment Outcome
  - Universities
  - Students, Nursing
  - Education, Nursing

  ### Attachments

  - PubMed entry
- ## Pawsitive Care: Canine-Assisted Intervention for Anxiety in ICU Patients and Family Members: A Single-Center, Single-Arm Study

  |  |  |
  | --- | --- |
  | Item Type | Journal Article |
  | Author | Kathleen Cook |
  | Author | Clare Robertson |
  | Author | Kiran Gudivada |
  | Author | Imogen Mitchell |
  | Author | Mary Nourse |
  | Author | Megan M. Hosey |
  | Author | Catherine Paterson |
  | Author | Sumeet Rai |
  | Abstract | OBJECTIVES: To investigate the effect of canine-assisted intervention (CAI) on anxiety symptoms among intensive care patients and their family members. DESIGN: Prospective, single-center, single-arm, nonrandomized, within-subject study design. SETTING: Tertiary hospital ICU. PATIENTS/SUBJECTS: Adult (≥ 16 yr) ICU patients and their family members. INTERVENTIONS: Individual CAI (therapy dog) sessions, lasting at least 15 minutes. MEASUREMENTS AND MAIN RESULTS: Primary outcome: change in Visual Analog Scale for Anxiety (VAS-A) among patients and family members; secondary outcomes (patient cohort): change in: 1) Numeric Pain Rating Scale, 2) physiologic vital signs, and 3) intervention-related adverse events. A total of 141 participants (70 patients and 71 family members) were recruited. The median (interquartile range [IQR]) age (yr) was 63 (49-71) for patients, and 51 (36-61) for family members. There was a significant reduction in anxiety scores after the intervention, with median (IQR) VAS-A scores changing from 5 (1-7) to 0 (0-4 [p < 0.001]) for the patient cohort and from 6 (5-8) to 3 (1-5 [p < 0.001]) for the family cohort. Majority of patients (56/70 [62%]) and family members (63/68 [93%]) demonstrated a greater than or equal to 2-point reduction in VAS-A scores. In terms of pain, median (IQR) scores among the patient cohort were also lower post-intervention (0 [0-5] vs. 0 [0-2]; p < 0.001). There were no statistically significant changes in physiologic vital signs (heart rate, respiratory rate, and systolic blood pressure) among patients following the intervention. Additionally, there were no reported dog bites, scratches, or other adverse events during CAI. CONCLUSIONS: CAI offers immediate therapeutic benefits in reducing anxiety symptoms in ICU patients and their family members with no observed adverse effects. It may also have a potential role as an adjunctive therapy for pain management in ICU patients. Further research should explore the influence on longer-term psychologic outcomes for ICU patients and family members. |
  | Date | 2025-05-01 |
  | Language | eng |
  | Short Title | Pawsitive Care |
  | Library Catalogue | PubMed |
  | Volume | 7 |
  | Pages | e1258 |
  | Publication | Critical Care Explorations |
  | DOI | 10.1097/CCE.0000000000001258 |
  | Issue | 5 |
  | Journal Abbr | Crit Care Explor |
  | ISSN | 2639-8028 |
  | PMID | 40293835 |
  | PMCID | PMC12040009 |
  | Date Added | 05/02/2026, 16:23:23 |
  | Modified | 05/02/2026, 16:23:23 |

  ### Tags:

  - Dogs
  - Animals
  - Female
  - Adult
  - Humans
  - Male
  - Middle Aged
  - Aged
  - Animal Assisted Therapy
  - animal-assisted intervention
  - anxiety
  - family
  - Anxiety
  - critical care
  - Intensive Care Units
  - Pain Measurement
  - Prospective Studies
  - Critical Care
  - canine-assisted intervention
  - Family

  ### Attachments

  - PubMed entry
- ## The Experience of Animal Assisted Therapy on Patients in an Acute Care Setting

  |  |  |
  | --- | --- |
  | Item Type | Journal Article |
  | Author | Amanda Bulette Coakley |
  | Author | Christine Donahue Annese |
  | Author | Joanne Hughes Empoliti |
  | Author | Jane M. Flanagan |
  | Abstract | Animal assisted therapy (AAT) programs are popular and there has been a proliferation of programs across settings. However, the research to support this intervention has not kept pace. This is particularly so for people who are hospitalized. This investigation aimed to explore the effects of the AAT dog visitation program on patients. A single group pre-post quasi-experimental design evaluated the effect of pet therapy on patients. Measures included salivary cortisol, anxiety, wellbeing, comfort, respiratory and heart rate. Analysis indicates a significant reduction in heart and respiratory rates p < .01 and level of anxiety p < .000 with improved levels of comfort and well-being p < .000. The salivary cortisol result was non-significant p = .623. This ATT dog program resulted in reduced anxiety levels and decreased heart and respiratory rates while improving subjective measures of comfort and wellbeing. |
  | Date | 2021-05 |
  | Language | eng |
  | Library Catalogue | PubMed |
  | Volume | 30 |
  | Pages | 401-405 |
  | Publication | Clinical Nursing Research |
  | DOI | 10.1177/1054773820977198 |
  | Issue | 4 |
  | Journal Abbr | Clin Nurs Res |
  | ISSN | 1552-3799 |
  | PMID | 33242977 |
  | Date Added | 05/02/2026, 16:20:09 |
  | Modified | 05/02/2026, 16:20:09 |

  ### Tags:

  - Dogs
  - Animals
  - Heart Rate
  - Humans
  - Animal Assisted Therapy
  - Anxiety
  - acute care setting
  - complementary therapies
  - Critical Care
  - health promotion

  ### Attachments

  - PubMed entry
- ## The Impact of a 20-Minute Animal-Assisted Activity Session on the Physiological and Emotional States in Patients With Fibromyalgia

  |  |  |
  | --- | --- |
  | Item Type | Journal Article |
  | Author | Stephanie Clark |
  | Author | François Martin |
  | Author | Ragen T. S. McGowan |
  | Author | Jessica Smidt |
  | Author | Rachel Anderson |
  | Author | Lei Wang |
  | Author | Tricia Turpin |
  | Author | Natalie Langenfeld-McCoy |
  | Author | Brent Bauer |
  | Author | Arya B. Mohabbat |
  | Abstract | OBJECTIVE: To study the direct physiological and emotional impact of an animal-assisted activity (AAA) session (a form of complementary and integrative medicine) in patients with fibromyalgia (FM). PATIENTS AND METHODS: The study population consisted of 221 participants with FM who were attending Mayo Clinic's Fibromyalgia Treatment Program between August 5, 2017, and September 1, 2018. This was a randomized controlled trial. Participants were randomly assigned to either the treatment group (a 20-minute session with a certified therapy dog and handler) or the control group (a 20-minute session with a handler only). To gain a better understanding of the direct physiological and emotional effects of AAA in patients with FM, we used multiple noninvasive physiologic-emotional biomarkers, including salivary cortisol and oxytocin concentrations, tympanic membrane temperatures, and various cardiac parameters, in addition to standardized pain and mood-based questionnaires. RESULTS: Results show a decrease in heart rate, an increase in heart rate variability, an increase in well-being survey scores, an increase in salivary oxytocin, and subsequent tympanic membrane temperature changes, suggesting that participants in the treatment group were in a more positive emotional-physiologic state as a result of the AAA session compared with the control group. CONCLUSION: Our results suggest that a 20-minute therapy dog visit in an outpatient setting can significantly and positively impact the physical and mental health of patients with FM. |
  | Date | 2020-11 |
  | Language | eng |
  | Library Catalogue | PubMed |
  | Volume | 95 |
  | Pages | 2442-2461 |
  | Publication | Mayo Clinic Proceedings |
  | DOI | 10.1016/j.mayocp.2020.04.037 |
  | Issue | 11 |
  | Journal Abbr | Mayo Clin Proc |
  | ISSN | 1942-5546 |
  | PMID | 32819740 |
  | Date Added | 05/02/2026, 16:20:09 |
  | Modified | 05/02/2026, 16:20:09 |

  ### Tags:

  - Hydrocortisone
  - Oxytocin
  - Dogs
  - Animals
  - Female
  - Adult
  - Humans
  - Male
  - Middle Aged
  - Young Adult
  - Aged
  - Animal Assisted Therapy
  - Saliva
  - Adolescent
  - Chronic Pain
  - Electrocardiography, Ambulatory
  - Fibromyalgia
  - Pain Measurement

  ### Attachments

  - Full Text PDF
  - PubMed entry
- ## Comparison of Cardiorespiratory Demand and Rate of Perceived Exertion During Propulsion in a Natural Environment With and Without the Use of a Mobility Assistance Dog in Manual Wheelchair Users

  |  |  |
  | --- | --- |
  | Item Type | Journal Article |
  | Author | Audrey Champagne |
  | Author | Dany H. Gagnon |
  | Author | Claude Vincent |
  | Abstract | OBJECTIVE: The aim of this study was to compare cardiorespiratory demand during manual wheelchair (MWC) propulsion among MWC users with a spinal cord injury (SCI) in a natural environment with and without the use of a trained mobility assistance dog (MAD). DESIGN: In this quasi-experimental repeated-measures analysis of difference, 13 experienced MWC users with an SCI propelled themselves with and without their trained MAD at a self-selected natural speed along a standardized 630-m course in a natural environment. Participants were equipped with a portable gas analyzer to measure their oxygen consumption, ventilation, tidal volume, respiratory quotient, respiratory rate, and heart rate before, during, and after completing the course. Participants also rated their perceived exertion on a modified Borg scale following each trial. RESULTS: All cardiorespiratory outcome measures decreased significantly with the use of a MAD (P ≤ 0.013; mean difference, -9% to -38%). Furthermore, most participants completed the course significantly faster (P ≤ 0.001; mean difference, -34%), while reporting considerably lower perceived exertion rates (P = 0.007; mean difference, -65%). CONCLUSIONS: A trained MAD decreases cardiorespiratory demand and rate of perceived exertion during MWC propulsion on a 630-m course among experienced MWC users with SCI. Trained MADs represent a valuable mobility assistive technology option for MWC users. |
  | Date | 2016-09 |
  | Language | eng |
  | Library Catalogue | PubMed |
  | Volume | 95 |
  | Pages | 685-691 |
  | Publication | American Journal of Physical Medicine & Rehabilitation |
  | DOI | 10.1097/PHM.0000000000000473 |
  | Issue | 9 |
  | Journal Abbr | Am J Phys Med Rehabil |
  | ISSN | 1537-7385 |
  | PMID | 26945223 |
  | Date Added | 05/02/2026, 16:20:09 |
  | Modified | 05/02/2026, 16:20:09 |

  ### Tags:

  - Dogs
  - Animals
  - Female
  - Adult
  - Heart Rate
  - Humans
  - Male
  - Animal Assisted Therapy
  - Oxygen Consumption
  - Persons with Disabilities
  - Physical Exertion
  - Respiratory Function Tests
  - Respiratory Rate
  - Spinal Cord Injuries
  - Wheelchairs

  ### Attachments

  - PubMed entry
- ## Animal Assisted Therapy (AAT) Program As a Useful Adjunct to Conventional Psychosocial Rehabilitation for Patients with Schizophrenia: Results of a Small-scale Randomized Controlled Trial

  |  |  |
  | --- | --- |
  | Item Type | Journal Article |
  | Author | Paula Calvo |
  | Author | Joan R. Fortuny |
  | Author | Sergio Guzmán |
  | Author | Cristina Macías |
  | Author | Jonathan Bowen |
  | Author | María L. García |
  | Author | Olivia Orejas |
  | Author | Ferran Molins |
  | Author | Asta Tvarijonaviciute |
  | Author | José J. Cerón |
  | Author | Antoni Bulbena |
  | Author | Jaume Fatjó |
  | Abstract | Currently, one of the main objectives of human-animal interaction research is to demonstrate the benefits of animal assisted therapy (AAT) for specific profiles of patients or participants. The aim of this study is to assess the effect of an AAT program as an adjunct to a conventional 6-month psychosocial rehabilitation program for people with schizophrenia. Our hypothesis is that the inclusion of AAT into psychosocial rehabilitation would contribute positively to the impact of the overall program on symptomology and quality of life, and that AAT would be a positive experience for patients. To test these hypotheses, we compared pre-program with post-program scores for the Positive and Negative Syndrome Scale (PANSS) and the EuroQoL-5 dimensions questionnaire (EuroQol-5D), pre-session with post-session salivary cortisol and alpha-amylase for the last four AAT sessions, and adherence rates between different elements of the program. We conducted a randomized, controlled study in a psychiatric care center in Spain. Twenty-two institutionalized patients with chronic schizophrenia completed the 6-month rehabilitation program, which included individual psychotherapy, group therapy, a functional program (intended to improve daily functioning), a community program (intended to facilitate community reintegration) and a family program. Each member of the control group (n = 8) participated in one activity from a range of therapeutic activities that were part of the functional program. In place of this functional program activity, the AAT-treatment group (n = 14) participated in twice-weekly 1-h sessions of AAT. All participants received the same weekly total number of hours of rehabilitation. At the end of the program, both groups (control and AAT-treatment) showed significant improvements in positive and overall symptomatology, as measured with PANSS, but only the AAT-treatment group showed a significant improvement in negative symptomatology. Adherence to the AAT-treatment was significantly higher than overall adherence to the control group's functional rehabilitation activities. Cortisol level was significantly reduced after participating in an AAT session, which could indicate that interaction with the therapy dogs reduced stress. In conclusion, the results of this small-scale RCT suggest that AAT could be considered a useful adjunct to conventional psychosocial rehabilitation for people with schizophrenia. |
  | Date | 2016 |
  | Language | eng |
  | Short Title | Animal Assisted Therapy (AAT) Program As a Useful Adjunct to Conventional Psychosocial Rehabilitation for Patients with Schizophrenia |
  | Library Catalogue | PubMed |
  | Volume | 7 |
  | Pages | 631 |
  | Publication | Frontiers in Psychology |
  | DOI | 10.3389/fpsyg.2016.00631 |
  | Journal Abbr | Front Psychol |
  | ISSN | 1664-1078 |
  | PMID | 27199859 |
  | PMCID | PMC4858645 |
  | Date Added | 05/02/2026, 16:20:09 |
  | Modified | 05/02/2026, 16:20:09 |

  ### Tags:

  - animal-assisted therapy
  - salivary cortisol
  - adherence to treatment
  - EuroQol-5 dimensions
  - PANSS
  - psychosocial rehabilitation
  - salivary alpha-amylase
  - schizophrenia

  ### Attachments

  - Full Text
  - PubMed entry
- ## Post-operative benefits of animal-assisted therapy in pediatric surgery: a randomised study

  |  |  |
  | --- | --- |
  | Item Type | Journal Article |
  | Author | Valeria Calcaterra |
  | Author | Pierangelo Veggiotti |
  | Author | Clara Palestrini |
  | Author | Valentina De Giorgis |
  | Author | Roberto Raschetti |
  | Author | Massimiliano Tumminelli |
  | Author | Simonetta Mencherini |
  | Author | Francesca Papotti |
  | Author | Catherine Klersy |
  | Author | Riccardo Albertini |
  | Author | Selene Ostuni |
  | Author | Gloria Pelizzo |
  | Abstract | BACKGROUND: Interest in animal-assisted therapy has been fuelled by studies supporting the many health benefits. The purpose of this study was to better understand the impact of an animal-assisted therapy program on children response to stress and pain in the immediate post-surgical period. PATIENTS AND METHODS: Forty children (3-17 years) were enrolled in the randomised open-label, controlled, pilot study. Patients were randomly assigned to the animal-assisted therapy-group (n = 20, who underwent a 20 min session with an animal-assisted therapy dog, after surgery) or the standard-group (n = 20, standard postoperative care). The study variables were determined in each patient, independently of the assigned group, by a researcher unblinded to the patient's group. The outcomes of the study were to define the neurological, cardiovascular and endocrinological impact of animal-assisted therapy in response to stress and pain. Electroencephalogram activity, heart rate, blood pressure, oxygen saturation, cerebral prefrontal oxygenation, salivary cortisol levels and the faces pain scale were considered as outcome measures. RESULTS: After entrance of the dog faster electroencephalogram diffuse beta-activity (> 14 Hz) was reported in all children of the animal-assisted therapy group; in the standard-group no beta-activity was recorded (100% vs 0%, p<0.001). During observation, some differences in the time profile between groups were observed for heart rate (test for interaction p = 0.018), oxygen saturation (test for interaction p = 0.06) and cerebral oxygenation (test for interaction p = 0.09). Systolic and diastolic blood pressure were influenced by animal-assisted therapy, though a higher variability in diastolic pressure was observed. Salivary cortisol levels did not show different behaviours over time between groups (p=0.70). Lower pain perception was noted in the animal-assisted group in comparison with the standard-group (p = 0.01). CONCLUSION: Animal-assisted therapy facilitated rapid recovery in vigilance and activity after anaesthesia, modified pain perception and induced emotional prefrontal responses. An adaptative cardiovascular response was also present. TRIAL REGISTRATION: ClinicalTrials.gov NCT02284100. |
  | Date | 2015 |
  | Language | eng |
  | Short Title | Post-operative benefits of animal-assisted therapy in pediatric surgery |
  | Library Catalogue | PubMed |
  | Volume | 10 |
  | Pages | e0125813 |
  | Publication | PloS One |
  | DOI | 10.1371/journal.pone.0125813 |
  | Issue | 6 |
  | Journal Abbr | PLoS One |
  | ISSN | 1932-6203 |
  | PMID | 26039494 |
  | PMCID | PMC4454536 |
  | Date Added | 05/02/2026, 16:20:09 |
  | Modified | 05/02/2026, 16:20:09 |

  ### Tags:

  - Dogs
  - Animals
  - Female
  - Humans
  - Male
  - Animal Assisted Therapy
  - Stress, Psychological
  - Child
  - Child, Preschool
  - Adolescent
  - Postoperative Pain
  - Postoperative Period

  ### Attachments

  - Full Text
  - PubMed entry
- ## Animal-assisted therapy as a pain relief intervention for children

  |  |  |
  | --- | --- |
  | Item Type | Journal Article |
  | Author | Carie Braun |
  | Author | Teresa Stangler |
  | Author | Jennifer Narveson |
  | Author | Sandra Pettingell |
  | Abstract | Animal-assisted therapy (AAT) is a healing modality involving a patient, an animal therapist, and handler with a goal of achieving a specified therapeutic outcome. Despite the myriad of studies documenting the benefits of AAT, no studies have yet determined the impact of animals on alleviation of pain in children. Therefore, a quasi-experimental intervention design was used to capture the change in pain and vital signs with (n=18) or without (n=39) AAT in children ages 3-17 in one acute care pediatric setting. The AAT intervention group experienced a significant reduction in pain level compared to the control group, t(55)=-2.86, p=.006. Although blood pressure and pulse were not impacted, respiratory rates became significantly higher in the AAT group (by an average of 2.22 breaths/min) as compared to the control group, t(55)=-2.63, p=.011. This study provides further support to the numerous health benefits of AAT, particularly for children in pain. |
  | Date | 2009-05 |
  | Language | eng |
  | Library Catalogue | PubMed |
  | Volume | 15 |
  | Pages | 105-109 |
  | Publication | Complementary Therapies in Clinical Practice |
  | DOI | 10.1016/j.ctcp.2009.02.008 |
  | Issue | 2 |
  | Journal Abbr | Complement Ther Clin Pract |
  | ISSN | 1873-6947 |
  | PMID | 19341990 |
  | Date Added | 05/02/2026, 16:23:23 |
  | Modified | 05/02/2026, 16:23:23 |

  ### Tags:

  - Dogs
  - Animals
  - Female
  - Heart Rate
  - Human-Animal Bond
  - Humans
  - Male
  - Blood Pressure
  - Child
  - Child, Preschool
  - Pain Management
  - Age Factors
  - Complementary Therapies
  - Immunocompromised Host
  - Respiration
  - Sex Factors

  ### Attachments

  - PubMed entry
- ## Animal-Assisted Activity in Critically Ill Older Adults: A Randomized Pilot and Feasibility Trial

  |  |  |
  | --- | --- |
  | Item Type | Journal Article |
  | Author | Sandy Branson |
  | Author | Lisa Boss |
  | Author | Shannan Hamlin |
  | Author | Nikhil S. Padhye |
  | Abstract | BACKGROUND: Limited evidence suggests the efficacy of animal-assisted activities (AAA) in improving biobehavioral stress responses in older patients in intensive care units (ICUs). OBJECTIVES: To assess the feasibility of an AAA (dog) intervention for improving biobehavioral stress response, measured by self-reported stress and anxiety and salivary cortisol, C-reactive protein, and interleukin-1β in older ICU patients, we examined enrollment, attrition, completion, data collection, and biobehavioral stress responses. METHODS: ICU patients ≥60 years old were randomly assigned to a 10-min AAA intervention or control/usual ICU care. Attitudes toward pets were assessed before the intervention. Self-reported stress and anxiety and salivary stress biomarkers were collected before and after the intervention and the usual care condition. RESULTS: The majority of patients were ineligible due to lack of decisional capacity, younger age, inability to provide saliva specimens, or critical illness. Though 15 participants were randomly allocated (AAA = 9; control = 6), only 10 completed the study. All participants completed the questionnaires; however, saliva specimens were significantly limited by volume. AAA was associated with decreases in stress and anxiety. Biomarker results were variable and revealed no specific trends associated with stress responses. Conclusions: Barriers to recruitment included an insufficient number of patients eligible for AAA based on hospital policy, difficulty finding patients who met study eligibility criteria, and illness-related factors. Recommendations for future studies include larger samples, a stronger control intervention such as a visitor without a dog, greater control over the AAA intervention, and use of blood from indwelling catheters for biomarkers. |
  | Date | 2020-07 |
  | Language | eng |
  | Short Title | Animal-Assisted Activity in Critically Ill Older Adults |
  | Library Catalogue | PubMed |
  | Volume | 22 |
  | Pages | 412-417 |
  | Publication | Biological Research for Nursing |
  | DOI | 10.1177/1099800420920719 |
  | Issue | 3 |
  | Journal Abbr | Biol Res Nurs |
  | ISSN | 1552-4175 |
  | PMID | 32319313 |
  | Date Added | 05/02/2026, 16:20:09 |
  | Modified | 05/02/2026, 16:20:09 |

  ### Tags:

  - Dogs
  - stress
  - Animals
  - Female
  - Humans
  - Male
  - Middle Aged
  - Aged
  - Animal Assisted Therapy
  - animal-assisted therapy
  - Stress, Psychological
  - Surveys and Questionnaires
  - anxiety
  - Aged, 80 and over
  - critical care
  - Critical Illness
  - Feasibility Studies
  - Frail Elderly
  - Intensive Care Units
  - older adult
  - Pilot Projects
  - Random Allocation

  ### Attachments

  - PubMed entry
- ## Effects of Animal-assisted Activities on Biobehavioral Stress Responses in Hospitalized Children: A Randomized Controlled Study

  |  |  |
  | --- | --- |
  | Item Type | Journal Article |
  | Author | Sandra M. Branson |
  | Author | Lisa Boss |
  | Author | Nikhil S. Padhye |
  | Author | Thea Trötscher |
  | Author | Alexandra Ward |
  | Abstract | PURPOSE: This study assessed the effectiveness of animal-assisted activities (AAA) on biobehavioral stress responses (anxiety, positive and negative affect, and salivary cortisol and C-reactive protein [CRP] levels) in hospitalized children. DESIGN AND METHODS: This was a randomized, controlled study. METHOD: Forty-eight participants were randomly assigned to receive a 10-minute AAA (n=24) or a control condition (n=24). Anxiety, positive and negative affect, and levels of salivary biomarkers were assessed before and after the intervention. RESULTS: Although increases in positive affect and decreases in negative affect were larger in the AAA condition, pre- and post-intervention differences between the AAA and control conditions were not significant. In addition, pre- and post-intervention differences between the conditions in salivary cortisol and CRP were not statistically significant. Baseline levels of anxiety, cortisol, and CRP had a significant and large correlation to the corresponding post-intervention measures. Scores on the Pet Attitude Scale were high but were not associated with changes in anxiety, positive affect, negative affect, or stress biomarkers. CONCLUSIONS: Although changes were in the expected direction, the magnitude of the effect was small. Future randomized controlled trials with larger recruitment are needed to determine the effectiveness of AAAs in reducing biobehavioral stress responses in hospitalized children. PRACTICE IMPLICATIONS: Nurses are positioned to recommend AAA as a beneficial and safe experience for hospitalized children. |
  | Date | 2017 |
  | Language | eng |
  | Short Title | Effects of Animal-assisted Activities on Biobehavioral Stress Responses in Hospitalized Children |
  | Library Catalogue | PubMed |
  | Volume | 36 |
  | Pages | 84-91 |
  | Publication | Journal of Pediatric Nursing |
  | DOI | 10.1016/j.pedn.2017.05.006 |
  | Journal Abbr | J Pediatr Nurs |
  | ISSN | 1532-8449 |
  | PMID | 28888516 |
  | Date Added | 05/02/2026, 16:20:09 |
  | Modified | 05/02/2026, 16:20:09 |

  ### Tags:

  - Animals
  - Female
  - Humans
  - Male
  - Animal Assisted Therapy
  - Adaptation, Psychological
  - Stress, Psychological
  - Child
  - Biomarkers
  - United States
  - Stress
  - Anxiety
  - Child, Hospitalized
  - Treatment Outcome
  - Child Behavior
  - Animal-assisted activities
  - Biobehavioral
  - Children
  - Confidence Intervals
  - Hospital
  - Multivariate Analysis
  - Reference Values

  ### Attachments

  - PubMed entry
- ## Developing effective animal-assisted intervention programs involving visiting dogs for institutionalized geriatric patients: a pilot study

  |  |  |
  | --- | --- |
  | Item Type | Journal Article |
  | Author | Alessandra Berry |
  | Author | Marta Borgi |
  | Author | Livia Terranova |
  | Author | Flavia Chiarotti |
  | Author | Enrico Alleva |
  | Author | Francesca Cirulli |
  | Abstract | AIM: An ever increasing interest in the therapeutic aspects of the human-animal bond has led to a proliferation of animal-assisted interventions (AAI) involving dogs. However, most of these programs lack a solid methodological structure, and basic evaluative research is needed. The purpose of this study was to test the value of dog-assisted interventions as an innovative tool to increase quality of life in the geriatric population. METHODS: Nineteen patients (men and women) with a mean age of 85 years participated in the study. Interactions between patients and visiting dogs occurred either in a social situation (socialization sessions) or in a therapeutic context (physical therapy sessions). We derived and characterized a specific ethogram of elderly-dog interactions aimed at evaluating the effectiveness of visiting dogs in improving mood, catalyzing social interactions and reducing their everyday apathetic state. Cortisol levels were also measured in the saliva, and depressive state was evaluated. RESULTS: Overall, results show a time-dependent increase in social behaviour and spontaneous interactions with the dogs. Dog-mediated interactions affected the daily increase in cortisol levels, thus having an 'activational effect', in contrast to the apathetic state of institutionalized elderly. CONCLUSIONS: Dog-mediated intervention programs appear to be promising tools to improve the social skills and enrich the daily activities of the institutionalized elderly. |
  | Date | 2012-09 |
  | Language | eng |
  | Short Title | Developing effective animal-assisted intervention programs involving visiting dogs for institutionalized geriatric patients |
  | Library Catalogue | PubMed |
  | Volume | 12 |
  | Pages | 143-150 |
  | Publication | Psychogeriatrics: The Official Journal of the Japanese Psychogeriatric Society |
  | DOI | 10.1111/j.1479-8301.2011.00393.x |
  | Issue | 3 |
  | Journal Abbr | Psychogeriatrics |
  | ISSN | 1479-8301 |
  | PMID | 22994611 |
  | Date Added | 05/02/2026, 16:20:09 |
  | Modified | 05/02/2026, 16:20:09 |

  ### Tags:

  - Hydrocortisone
  - Dogs
  - Animals
  - Female
  - Humans
  - Male
  - Aged
  - Animal Assisted Therapy
  - Saliva
  - Depression
  - Social Behavior
  - Aged, 80 and over
  - Pilot Projects
  - Quality of Life
  - Analysis of Variance
  - Follow-Up Studies
  - Geriatrics
  - Homes for the Aged
  - Institutionalization
  - Italy
  - Nursing Homes
  - Psychiatric Status Rating Scales

  ### Attachments

  - Full Text
  - PubMed entry
- ## Animal-assisted intervention for geriatric well-being: A comprehensive review

  |  |  |
  | --- | --- |
  | Item Type | Journal Article |
  | Author | L. K. Bernhardt |
  | Author | A. Vashe |
  | Author | G. V. Bernhardt |
  | Author | J. Pinto |
  | Abstract | BACKGROUND: In recent years, the size of geriatric population seems to have grown larger than that of younger children and is expected to grow even larger in few years from now. As older individuals are more vulnerable to health concerns and loneliness, it is necessary to focus on providing them opportunities for healthy ageing. Animal-assisted therapy (AAT) has emerged as one of the simple yet effective approach to enhance physical, psychological, and social well-being in older adults. METHODS: This review article presents collective information from various experiments on AAT's effectiveness in promoting healthy ageing. RESULTS: The physiological impacts of AAT, on cardiovascular health, mobility, and day to day activities have been discussed. The psychological benefits of AAT, such as improvement of mood, cognition, and alleviation of anxiety, loneliness, and depression, are explored. This review also presents the possible mechanisms underlying the effective-ness of AAT, such as release of oxytocin, dopamine, and endorphins, which contribute to emotional well-being along with reduction of stress. The human-animal bond established during AAT sessions is discussed as a significant factor in promoting positive outcomes. Challenges faced and limitations involved in employing therapy animals within ageing populations are also discussed. CONCLUSIONS: Avenues for future research and potential applications of AAT in diverse healthcare settings are proposed, emphasizing the need for further empirical investigation to fully elucidate the mechanisms and benefits of AAT for healthy ageing. Through this comprehensive review, we aim to highlight the potential of AAT as a holistic intervention to enhance the well-being of older adults, providing valuable insights for healthcare practitioners, researchers, and policymakers invested in promoting healthy ageing strategies. |
  | Date | 2024 |
  | Language | eng |
  | Short Title | Animal-assisted intervention for geriatric well-being |
  | Library Catalogue | PubMed |
  | Volume | 175 |
  | Pages | 362-369 |
  | Publication | La Clinica Terapeutica |
  | DOI | 10.7417/CT.2024.5126 |
  | Issue | 5 |
  | Journal Abbr | Clin Ter |
  | ISSN | 1972-6007 |
  | PMID | 39400102 |
  | Date Added | 05/02/2026, 16:20:09 |
  | Modified | 05/02/2026, 16:20:09 |

  ### Tags:

  - Animals
  - Human-Animal Bond
  - Humans
  - Aged
  - Animal Assisted Therapy
  - animal-assisted therapy
  - pet ownership
  - animal-assisted intervention
  - Loneliness
  - Anxiety
  - elderly
  - geriatric
  - healthy ageing
  - Healthy Aging
  - older adults
  - pet companionship
  - Pet therapy
  - SDG-3

  ### Attachments

  - PubMed entry
- ## Oxytocin levels and self-reported anxiety during interactions between humans and cows

  |  |  |
  | --- | --- |
  | Item Type | Journal Article |
  | Author | Bente Berget |
  | Author | Judit Vas |
  | Author | Gunn Pedersen |
  | Author | Kerstin Uvnäs-Moberg |
  | Author | Ruth C. Newberry |
  | Abstract | INTRODUCTION: Positive social interactions with farm animals may have therapeutic benefits on humans by increasing brain oxytocin secretion, as inferred from circulating oxytocin levels. The aim of this observational study was to investigate acute changes in human plasma oxytocin levels and state anxiety associated with interactions with dairy cows. METHODS: Data were collected from 18 healthy female nursing students who performed stroking and brushing of an unfamiliar cow for 15 min. Blood samples were drawn before entering the cowshed (T1, baseline), and after 5 (T2) and 15 (T3) min of interaction with a cow. At T1 and T3, the students filled out the Norwegian version of the Spielberger State-Trait Anxiety Inventory-State Subscale (STAI-SS). RESULTS: Across participants, no significant changes in average plasma oxytocin concentration were detected between time points (p>0.05). There was, however, a modest decline in the STAI-SS scores between T1 and T3 (p=0.015) and a positive correlation between the change in individual level of state anxiety between T1 and T3 and the change in OT concentration of the same individual between T2 and T3 (p = 0.045). DISCUSSION: The results suggest that friendly social interactions with cows are beneficial in lowering state anxiety, but any relationship with release of OT into the circulation was complex and variable across individuals. The acute reduction in state anxiety lends support to the value of interacting with farm animals in the context of Green Care for people with mental health challenges. |
  | Date | 2023 |
  | Language | eng |
  | Library Catalogue | PubMed |
  | Volume | 14 |
  | Pages | 1252463 |
  | Publication | Frontiers in Psychology |
  | DOI | 10.3389/fpsyg.2023.1252463 |
  | Journal Abbr | Front Psychol |
  | ISSN | 1664-1078 |
  | PMID | 37780173 |
  | PMCID | PMC10536144 |
  | Date Added | 05/02/2026, 16:20:09 |
  | Modified | 05/02/2026, 16:20:09 |

  ### Tags:

  - oxytocin
  - human–animal interaction
  - animal-assisted intervention
  - anxiety
  - cow
  - green care

  ### Attachments

  - Full Text
  - PubMed entry
- ## Psychosocial and psychophysiological effects of human-animal interactions: the possible role of oxytocin

  |  |  |
  | --- | --- |
  | Item Type | Journal Article |
  | Author | Andrea Beetz |
  | Author | Kerstin Uvnäs-Moberg |
  | Author | Henri Julius |
  | Author | Kurt Kotrschal |
  | Abstract | During the last decade it has become more widely accepted that pet ownership and animal assistance in therapy and education may have a multitude of positive effects on humans. Here, we review the evidence from 69 original studies on human-animal interactions (HAI) which met our inclusion criteria with regard to sample size, peer-review, and standard scientific research design. Among the well-documented effects of HAI in humans of different ages, with and without special medical, or mental health conditions are benefits for: social attention, social behavior, interpersonal interactions, and mood; stress-related parameters such as cortisol, heart rate, and blood pressure; self-reported fear and anxiety; and mental and physical health, especially cardiovascular diseases. Limited evidence exists for positive effects of HAI on: reduction of stress-related parameters such as epinephrine and norepinephrine; improvement of immune system functioning and pain management; increased trustworthiness of and trust toward other persons; reduced aggression; enhanced empathy and improved learning. We propose that the activation of the oxytocin system plays a key role in the majority of these reported psychological and psychophysiological effects of HAI. Oxytocin and HAI effects largely overlap, as documented by research in both, humans and animals, and first studies found that HAI affects the oxytocin system. As a common underlying mechanism, the activation of the oxytocin system does not only provide an explanation, but also allows an integrative view of the different effects of HAI. |
  | Date | 2012 |
  | Language | eng |
  | Short Title | Psychosocial and psychophysiological effects of human-animal interactions |
  | Library Catalogue | PubMed |
  | Volume | 3 |
  | Pages | 234 |
  | Publication | Frontiers in Psychology |
  | DOI | 10.3389/fpsyg.2012.00234 |
  | Journal Abbr | Front Psychol |
  | ISSN | 1664-1078 |
  | PMID | 22866043 |
  | PMCID | PMC3408111 |
  | Date Added | 05/02/2026, 16:20:09 |
  | Modified | 05/02/2026, 16:20:09 |

  ### Tags:

  - oxytocin
  - human-animal interaction
  - animal-assisted interventions
  - animal-assisted therapy
  - pet ownership
  - stress reduction

  ### Attachments

  - Full Text
  - PubMed entry
