# Supplementary material for: Evidence of Physiological Comodulation During Human–Animal Interaction: A Systematic Review
Source: Ann N Y Acad Sci. 2026 Jun 4;1560(1):e70299. doi: 10.1111/nyas.70299 (PMC13238372; doi:10.1111/nyas.70299)
Supplement: Supplementary file 2 — Supplementary Materials: Supp2‐Zotero‐Collection.zip [file NYAS-1560-0-s002.zip › Supp2_Zotero_Collection/new searches/scopus_export_Feb 5-2026_ed8094e3-abfa-443c-ac59-6666c7a10b31.htm]

Zotero Report


- ## Comparison of contingent and noncontingent access to therapy dogs during academic tasks in children with autism spectrum disorder

  |  |  |
  | --- | --- |
  | Item Type | Journal Article |
  | Author | A. Protopopova |
  | Author | A.L. Matter |
  | Author | B.N. Harris |
  | Author | K.M. Wiskow |
  | Author | J.M. Donaldson |
  | Date | 2020 |
  | Archive | Scopus |
  | URL | https://www.scopus.com/inward/record.uri?eid=2-s2.0-85083758103&doi=10.1002%2Fjaba.619&partnerID=40&md5=853f1eb5368b7ea0c2a9a2336fd5b22a |
  | Volume | 53 |
  | Pages | 811-834 |
  | Publication | Journal of Applied Behavior Analysis |
  | DOI | 10.1002/jaba.619 |
  | Issue | 2 |
  | Date Added | 05/02/2026, 16:52:06 |
  | Modified | 05/02/2026, 16:52:06 |

  ### Notes:

  - Export Date: 05 February 2026; Cited By: 14
- ## Dog-assisted intervention at a Spanish university: pilot study

  |  |  |
  | --- | --- |
  | Item Type | Journal Article |
  | Author | D. Peña Gil |
  | Author | M. García-García |
  | Author | C. Camilli-Trujillo |
  | Date | 2020 |
  | Archive | Scopus |
  | URL | https://www.scopus.com/inward/record.uri?eid=2-s2.0-85071592723&doi=10.1108%2FJARHE-03-2019-0067&partnerID=40&md5=c02a493faf2d82fa1a7518c6a165deb7 |
  | Volume | 12 |
  | Pages | 344-356 |
  | Publication | Journal of Applied Research in Higher Education |
  | DOI | 10.1108/JARHE-03-2019-0067 |
  | Issue | 2 |
  | Date Added | 05/02/2026, 16:52:06 |
  | Modified | 05/02/2026, 16:52:06 |

  ### Notes:

  - Export Date: 05 February 2026; Cited By: 7
- ## Investigation of Physiological and Behavioral Responses in Dogs Participating in Animal-Assisted Therapy with Children Diagnosed with Attention-Deficit Hyperactivity Disorder

  |  |  |
  | --- | --- |
  | Item Type | Journal Article |
  | Author | A.L. Melco |
  | Author | L. Goldman |
  | Author | A.H. Fine |
  | Author | J.M. Peralta |
  | Date | 2020 |
  | Archive | Scopus |
  | URL | https://www.scopus.com/inward/record.uri?eid=2-s2.0-85055644883&doi=10.1080%2F10888705.2018.1536979&partnerID=40&md5=a2281eb216306aefdd360f57f9368118 |
  | Volume | 23 |
  | Pages | 10-28 |
  | Publication | Journal of Applied Animal Welfare Science |
  | DOI | 10.1080/10888705.2018.1536979 |
  | Issue | 1 |
  | Date Added | 05/02/2026, 16:52:06 |
  | Modified | 05/02/2026, 16:52:06 |

  ### Notes:

  - Export Date: 05 February 2026; Cited By: 33
- ## Effect of dog presence on stress levels in students under psychological strain: A pilot study

  |  |  |
  | --- | --- |
  | Item Type | Journal Article |
  | Author | K. Kristýna |
  | Author | R. Procházková |
  | Author | M. Vadroňová |
  | Author | M. Součková |
  | Author | E. Prouzová |
  | Date | 2020 |
  | Archive | Scopus |
  | URL | https://www.scopus.com/inward/record.uri?eid=2-s2.0-85082791812&doi=10.3390%2Fijerph17072286&partnerID=40&md5=5f0fc954812219f7d9005a4b11598f0c |
  | Volume | 17 |
  | Publication | International Journal of Environmental Research and Public Health |
  | DOI | 10.3390/ijerph17072286 |
  | Issue | 7 |
  | Date Added | 05/02/2026, 16:52:06 |
  | Modified | 05/02/2026, 16:52:06 |

  ### Notes:

  - Export Date: 05 February 2026; Cited By: 16
- ## Randomized Trial of Therapy Dogs Versus Deliberative Coloring (Art Therapy) to Reduce Stress in Emergency Medicine Providers

  |  |  |
  | --- | --- |
  | Item Type | Journal Article |
  | Author | J.A. Kline |
  | Author | K. VanRyzin |
  | Author | J.C. Davis |
  | Author | J.A. Parra |
  | Author | M.L. Todd |
  | Author | L.L. Shaw |
  | Author | B.R. Haggard |
  | Author | M.A. Fisher |
  | Author | K.L. Pettit |
  | Author | A.M. Beck |
  | Date | 2020 |
  | Archive | Scopus |
  | URL | https://www.scopus.com/inward/record.uri?eid=2-s2.0-85082976378&doi=10.1111%2Facem.13939&partnerID=40&md5=a4d2b49e377499d04bf68cac310b51f7 |
  | Volume | 27 |
  | Pages | 266-275 |
  | Publication | Academic Emergency Medicine |
  | DOI | 10.1111/acem.13939 |
  | Issue | 4 |
  | Date Added | 05/02/2026, 16:52:06 |
  | Modified | 05/02/2026, 16:52:06 |

  ### Notes:

  - Export Date: 05 February 2026; Cited By: 39
- ## The impact of dog therapy on nursing students’ heart rates and ability to pay attention in class

  |  |  |
  | --- | --- |
  | Item Type | Journal Article |
  | Author | O. Griscti |
  | Author | L. Camilleri |
  | Date | 2020 |
  | Archive | Scopus |
  | URL | https://www.scopus.com/inward/record.uri?eid=2-s2.0-85075313969&doi=10.1016%2Fj.ijer.2019.101498&partnerID=40&md5=dac06cf85253ab625bc6b0a502564f18 |
  | Volume | 99 |
  | Publication | International Journal of Educational Research |
  | DOI | 10.1016/j.ijer.2019.101498 |
  | Date Added | 05/02/2026, 16:52:06 |
  | Modified | 05/02/2026, 16:52:06 |

  ### Notes:

  - Export Date: 05 February 2026; Cited By: 7
- ## Distraction-focused interventions on examination stress in nursing students: Effects on psychological stress and biomarker levels. A randomized controlled trial

  |  |  |
  | --- | --- |
  | Item Type | Journal Article |
  | Author | V. Gebhart |
  | Author | W. Buchberger |
  | Author | I. Klotz |
  | Author | S. Neururer |
  | Author | C. Rungg |
  | Author | G. Tucek |
  | Author | C. Zenzmaier |
  | Author | S. Perkhofer |
  | Date | 2020 |
  | Archive | Scopus |
  | URL | https://www.scopus.com/inward/record.uri?eid=2-s2.0-85074969047&doi=10.1111%2Fijn.12788&partnerID=40&md5=8de34f88b6c6b9d1eaeaec6a1d272803 |
  | Volume | 26 |
  | Publication | International Journal of Nursing Practice |
  | DOI | 10.1111/ijn.12788 |
  | Issue | 1 |
  | Date Added | 05/02/2026, 16:52:06 |
  | Modified | 05/02/2026, 16:52:06 |

  ### Notes:

  - Export Date: 05 February 2026; Cited By: 34
- ## The combined use of salivary cortisol concentrations, heart rate, and respiratory rate for the welfare assessment of dogs involved in AAI programs

  |  |  |
  | --- | --- |
  | Item Type | Journal Article |
  | Author | I.R. de Carvalho |
  | Author | T. Nunes |
  | Author | L. De Sousa |
  | Author | V. Almeida |
  | Date | 2020 |
  | Archive | Scopus |
  | URL | https://www.scopus.com/inward/record.uri?eid=2-s2.0-85083018432&doi=10.1016%2Fj.jveb.2019.10.011&partnerID=40&md5=e00124ae4ddb25456cbf06f61b506909 |
  | Volume | 36 |
  | Pages | 26-33 |
  | Publication | Journal of Veterinary Behavior |
  | DOI | 10.1016/j.jveb.2019.10.011 |
  | Date Added | 05/02/2026, 16:52:06 |
  | Modified | 05/02/2026, 16:52:06 |

  ### Notes:

  - Export Date: 05 February 2026; Cited By: 29
