# Supplementary material for: Evidence of Physiological Comodulation During Human–Animal Interaction: A Systematic Review
Source: Ann N Y Acad Sci. 2026 Jun 4;1560(1):e70299. doi: 10.1111/nyas.70299 (PMC13238372; doi:10.1111/nyas.70299)
Supplement: Supplementary file 2 — Supplementary Materials: Supp2‐Zotero‐Collection.zip [file NYAS-1560-0-s002.zip › Supp2_Zotero_Collection/new searches/scopus_export_Feb 5-2026_fa3ebad6-0b1a-4194-af46-609c1bd6c2a1.htm]

Zotero Report


- ## The impact of canine-assisted intervention on stress reduction among university students in Thailand

  |  |  |
  | --- | --- |
  | Item Type | Journal Article |
  | Author | S. Yotanyamaneewong |
  | Author | D. Junla |
  | Author | J.L. Brown |
  | Author | N. Siriapaipant |
  | Author | N. Yodkamol |
  | Author | W. Prachasilchai |
  | Author | A. Saengthong |
  | Author | P. Sanguansak |
  | Author | T. Kankonsue |
  | Author | V. Punyapornwithaya |
  | Author | C. Sansamur |
  | Author | K. Nganvongpanit |
  | Author | J. Khonmee |
  | Date | 2025 |
  | Archive | Scopus |
  | URL | https://www.scopus.com/inward/record.uri?eid=2-s2.0-86000770081&doi=10.1371%2Fjournal.pone.0318777&partnerID=40&md5=e2e4cc5fa5aa77733240c41fc13df9ec |
  | Volume | 20 |
  | Publication | PLoS ONE |
  | DOI | 10.1371/journal.pone.0318777 |
  | Issue | 3 March |
  | Date Added | 05/02/2026, 16:38:57 |
  | Modified | 05/02/2026, 16:38:57 |

  ### Notes:

  - Export Date: 05 February 2026; Cited By: 0
- ## Human-Animal Interaction

  |  |  |
  | --- | --- |
  | Item Type | Book Section |
  | Author | J. Yamasaki |
  | Date | 2022 |
  | Archive | Scopus |
  | URL | https://www.scopus.com/inward/record.uri?eid=2-s2.0-105026110855&doi=10.1002%2F9781119678816.iehc0582&partnerID=40&md5=e5330465297c841fbc7e5093b0fed113 |
  | Pages | 1-5 |
  | Book Title | The International Encyclopedia of Health Communication |
  | DOI | 10.1002/9781119678816.iehc0582 |
  | Date Added | 05/02/2026, 16:38:58 |
  | Modified | 05/02/2026, 16:38:58 |

  ### Notes:

  - Export Date: 05 February 2026; Cited By: 0
- ## Tracking positive and negative affect in PTSD inpatients during a service dog intervention.

  |  |  |
  | --- | --- |
  | Item Type | Journal Article |
  | Author | S.H. Woodward |
  | Author | A.L. Jamison |
  | Author | S. Gala |
  | Author | C. Lawlor |
  | Author | D. Villaseñor |
  | Author | G. Tamayo |
  | Author | M. Puckett |
  | Date | 2021 |
  | Archive | Scopus |
  | URL | https://www.scopus.com/inward/record.uri?eid=2-s2.0-85111755174&doi=10.1037%2Fccp0000572&partnerID=40&md5=c5bb3e8e5dfa8cff28e25563598594d6 |
  | Volume | 89 |
  | Pages | 551-562 |
  | Publication | Journal of Consulting and Clinical Psychology |
  | DOI | 10.1037/ccp0000572 |
  | Issue | 6 |
  | Date Added | 05/02/2026, 16:38:58 |
  | Modified | 05/02/2026, 16:38:58 |

  ### Notes:

  - Export Date: 05 February 2026; Cited By: 13
- ## Can dog-assisted intervention decrease anxiety level and autonomic agitation in patients with anxiety disorders?

  |  |  |
  | --- | --- |
  | Item Type | Journal Article |
  | Author | D. Wolyńczyk-Gmaj |
  | Author | A. Ziółkowska |
  | Author | P. Rogala |
  | Author | D. Scigała |
  | Author | L. Bryła |
  | Author | B. Gmaj |
  | Author | M. Wojnar |
  | Date | 2021 |
  | Archive | Scopus |
  | URL | https://www.scopus.com/inward/record.uri?eid=2-s2.0-85118355248&doi=10.3390%2Fjcm10215171&partnerID=40&md5=e63828a317b171dd19bb34d6692a082b |
  | Volume | 10 |
  | Publication | Journal of Clinical Medicine |
  | DOI | 10.3390/jcm10215171 |
  | Issue | 21 |
  | Date Added | 05/02/2026, 16:38:58 |
  | Modified | 05/02/2026, 16:38:58 |

  ### Notes:

  - Export Date: 05 February 2026; Cited By: 15
- ## Welfare of the therapy animal within facilitated youth psychotherapy: A scoping review

  |  |  |
  | --- | --- |
  | Item Type | Journal Article |
  | Author | T. Winton |
  | Author | M. Nicodemus |
  | Author | M. Friend |
  | Author | K. Holtcamp |
  | Author | D.D. Burnett |
  | Author | T. Smith |
  | Author | C. Hill |
  | Author | E. Memili |
  | Author | C. Cavinder |
  | Date | 2024 |
  | Archive | Scopus |
  | URL | https://www.scopus.com/inward/record.uri?eid=2-s2.0-85199187502&doi=10.1016%2Fj.applanim.2024.106357&partnerID=40&md5=75cf35e1691d515c095717eefe16738d |
  | Volume | 277 |
  | Publication | Applied Animal Behaviour Science |
  | DOI | 10.1016/j.applanim.2024.106357 |
  | Date Added | 05/02/2026, 16:38:58 |
  | Modified | 05/02/2026, 16:38:58 |

  ### Notes:

  - Export Date: 05 February 2026; Cited By: 3
- ## Animals in Animal-Assisted Services: Are They Volunteers or Professionals?

  |  |  |
  | --- | --- |
  | Item Type | Journal Article |
  | Author | B. Wijnen |
  | Author | P. Martens |
  | Date | 2022 |
  | Archive | Scopus |
  | URL | https://www.scopus.com/inward/record.uri?eid=2-s2.0-85139741947&doi=10.3390%2Fani12192564&partnerID=40&md5=45d3253d6b7c0d929249437934d14d79 |
  | Volume | 12 |
  | Publication | Animals |
  | DOI | 10.3390/ani12192564 |
  | Issue | 19 |
  | Date Added | 05/02/2026, 16:38:58 |
  | Modified | 05/02/2026, 16:38:58 |

  ### Notes:

  - Export Date: 05 February 2026; Cited By: 10
- ## The effects of Animal Assisted Therapy on autonomic and endocrine activity in adults with autism spectrum disorder: A randomized controlled trial

  |  |  |
  | --- | --- |
  | Item Type | Journal Article |
  | Author | C. Wijker |
  | Author | N. Kupper |
  | Author | R. Leontjevas |
  | Author | A. Spek |
  | Author | M.-J. Enders-Slegers |
  | Date | 2021 |
  | Archive | Scopus |
  | URL | https://www.scopus.com/inward/record.uri?eid=2-s2.0-85109090462&doi=10.1016%2Fj.genhosppsych.2021.05.003&partnerID=40&md5=43854d61ae34722e1542e9a8fa18a1ba |
  | Volume | 72 |
  | Pages | 36-44 |
  | Publication | General Hospital Psychiatry |
  | DOI | 10.1016/j.genhosppsych.2021.05.003 |
  | Date Added | 05/02/2026, 16:38:58 |
  | Modified | 05/02/2026, 16:38:58 |

  ### Notes:

  - Export Date: 05 February 2026; Cited By: 18
- ## Human-Animal Interaction in Immersive Virtual Reality: The Role of Social Presence and Positive Effects

  |  |  |
  | --- | --- |
  | Item Type | Conference Paper |
  | Author | W.A.A. Wanali |
  | Author | M. Dresel |
  | Author | N. Jochems |
  | Date | 2024 |
  | Archive | Scopus |
  | URL | https://www.scopus.com/inward/record.uri?eid=2-s2.0-85203671564&doi=10.1145%2F3670653.3670661&partnerID=40&md5=7a725b0df264eb0a94ce56e50d2d88f0 |
  | Pages | 342-359 |
  | Conference Name | ACM International Conference Proceeding Series |
  | DOI | 10.1145/3670653.3670661 |
  | Date Added | 05/02/2026, 16:38:57 |
  | Modified | 05/02/2026, 16:38:57 |

  ### Notes:

  - Export Date: 05 February 2026; Cited By: 2
- ## The Impact of Therapy Dogs on Prelicensure Baccalaureate Nursing Student Test Anxiety

  |  |  |
  | --- | --- |
  | Item Type | Journal Article |
  | Author | C. Walker |
  | Date | 2023 |
  | Archive | Scopus |
  | URL | https://www.scopus.com/inward/record.uri?eid=2-s2.0-85148324201&doi=10.1097%2F01.NEP.0000000000001042&partnerID=40&md5=8e458bbb67dbd2b07646368353bd6288 |
  | Volume | 44 |
  | Pages | 98-104 |
  | Publication | Nursing Education Perspectives |
  | DOI | 10.1097/01.NEP.0000000000001042 |
  | Issue | 2 |
  | Date Added | 05/02/2026, 16:38:58 |
  | Modified | 05/02/2026, 16:38:58 |

  ### Notes:

  - Export Date: 05 February 2026; Cited By: 5
- ## Therapy Dog Support in Pediatric Dentistry: A Social Welfare Intervention for Reducing Anticipatory Anxiety and Situational Fear in Children

  |  |  |
  | --- | --- |
  | Item Type | Journal Article |
  | Author | A. Vincent |
  | Author | M. Heima |
  | Author | K.J. Farkas |
  | Date | 2020 |
  | Archive | Scopus |
  | URL | https://www.scopus.com/inward/record.uri?eid=2-s2.0-85091006780&doi=10.1007%2Fs10560-020-00701-4&partnerID=40&md5=ac5fdf312b7d29c5eff41a1188d05947 |
  | Volume | 37 |
  | Pages | 615-629 |
  | Publication | Child and Adolescent Social Work Journal |
  | DOI | 10.1007/s10560-020-00701-4 |
  | Issue | 6 |
  | Date Added | 05/02/2026, 16:38:58 |
  | Modified | 05/02/2026, 16:38:58 |

  ### Notes:

  - Export Date: 05 February 2026; Cited By: 16
- ## Effects of Essential Animal Visitation Program (AVP) Components on Students’ Salivary α-Amylase and Amylase-to-Cortisol Ratios

  |  |  |
  | --- | --- |
  | Item Type | Journal Article |
  | Author | J.L. Vandagriff |
  | Author | A.M. Carr |
  | Author | S.M. Roeter Smith |
  | Author | P. Pendry |
  | Date | 2022 |
  | Archive | Scopus |
  | URL | https://www.scopus.com/inward/record.uri?eid=2-s2.0-85119954206&doi=10.1080%2F08927936.2021.1996025&partnerID=40&md5=87441eeb4a7e19e6cf16cab5018b2c48 |
  | Volume | 35 |
  | Pages | 443-461 |
  | Publication | Anthrozoos |
  | DOI | 10.1080/08927936.2021.1996025 |
  | Issue | 3 |
  | Date Added | 05/02/2026, 16:38:58 |
  | Modified | 05/02/2026, 16:38:58 |

  ### Notes:

  - Export Date: 05 February 2026; Cited By: 3
- ## Do service dogs for veterans with ptsd mount a cortisol response in response to training?

  |  |  |
  | --- | --- |
  | Item Type | Journal Article |
  | Author | E.A.E. van Houtert |
  | Author | N. Endenburg |
  | Author | T. Bas Rodenburg |
  | Author | E. Vermetten |
  | Date | 2021 |
  | Archive | Scopus |
  | URL | https://www.scopus.com/inward/record.uri?eid=2-s2.0-85101667692&doi=10.3390%2Fani11030650&partnerID=40&md5=36f3d3f527296f278bd745c40f38a7ba |
  | Volume | 11 |
  | Pages | 1-9 |
  | Publication | Animals |
  | DOI | 10.3390/ani11030650 |
  | Issue | 3 |
  | Date Added | 05/02/2026, 16:38:58 |
  | Modified | 05/02/2026, 16:38:58 |

  ### Notes:

  - Export Date: 05 February 2026; Cited By: 5
- ## Can a Facility Dog Improve Inpatient Rehabilitation Engagement? A Randomized Controlled Crossover Trial

  |  |  |
  | --- | --- |
  | Item Type | Journal Article |
  | Author | H. Tropiano |
  | Author | J. Robertson |
  | Author | A. Rathbun |
  | Author | B.T. Yates |
  | Author | N.M. Fromm |
  | Date | 2025 |
  | Archive | Scopus |
  | URL | https://www.scopus.com/inward/record.uri?eid=2-s2.0-105011283410&doi=10.1080%2F08927936.2025.2529681&partnerID=40&md5=b9b097d7cea396da8113ad801a6ab0cd |
  | Volume | 38 |
  | Pages | 851-867 |
  | Publication | Anthrozoos |
  | DOI | 10.1080/08927936.2025.2529681 |
  | Issue | 5 |
  | Date Added | 05/02/2026, 16:38:57 |
  | Modified | 05/02/2026, 16:38:57 |

  ### Notes:

  - Export Date: 05 February 2026; Cited By: 0
- ## Psychophysiological mechanisms underlying the potential health benefits of human-dog interactions: A systematic literature review

  |  |  |
  | --- | --- |
  | Item Type | Journal Article |
  | Author | J.T. Teo |
  | Author | S.J. Johnstone |
  | Author | S.S. Römer |
  | Author | S.J. Thomas |
  | Date | 2022 |
  | Archive | Scopus |
  | URL | https://www.scopus.com/inward/record.uri?eid=2-s2.0-85135390145&doi=10.1016%2Fj.ijpsycho.2022.07.007&partnerID=40&md5=4ac78c7e84332976d6afe333c9605fe7 |
  | Volume | 180 |
  | Pages | 27-48 |
  | Publication | International Journal of Psychophysiology |
  | DOI | 10.1016/j.ijpsycho.2022.07.007 |
  | Date Added | 05/02/2026, 16:38:58 |
  | Modified | 05/02/2026, 16:38:58 |

  ### Notes:

  - Export Date: 05 February 2026; Cited By: 31
- ## Unconventional Animal Species Participation in Animal-Assisted Interventions and Methods for Measuring Their Experienced Stress

  |  |  |
  | --- | --- |
  | Item Type | Journal Article |
  | Author | É. Suba-Bokodi |
  | Author | I. Nagy |
  | Author | M. Molnár |
  | Date | 2024 |
  | Archive | Scopus |
  | URL | https://www.scopus.com/inward/record.uri?eid=2-s2.0-85207652362&doi=10.3390%2Fani14202935&partnerID=40&md5=8a945f15f0c5f2531c715bd94e9f1e55 |
  | Volume | 14 |
  | Publication | Animals |
  | DOI | 10.3390/ani14202935 |
  | Issue | 20 |
  | Date Added | 05/02/2026, 16:38:57 |
  | Modified | 05/02/2026, 16:38:57 |

  ### Notes:

  - Export Date: 05 February 2026; Cited By: 3
- ## The Impact of Transportation on the Cortisol Level of Dwarf Rabbits Bred to Animal-Assisted Interventions

  |  |  |
  | --- | --- |
  | Item Type | Journal Article |
  | Author | É. Suba-Bokodi |
  | Author | I. Nagy |
  | Author | M. Molnár |
  | Date | 2024 |
  | Archive | Scopus |
  | URL | https://www.scopus.com/inward/record.uri?eid=2-s2.0-85187874688&doi=10.3390%2Fani14050664&partnerID=40&md5=ad23713963cb044dc0ffd02bd334e798 |
  | Volume | 14 |
  | Publication | Animals |
  | DOI | 10.3390/ani14050664 |
  | Issue | 5 |
  | Date Added | 05/02/2026, 16:38:58 |
  | Modified | 05/02/2026, 16:38:58 |

  ### Notes:

  - Export Date: 05 February 2026; Cited By: 6
- ## A Review of Creative Play Interventions to Improve Children’s Hospital Experience and Wellbeing

  |  |  |
  | --- | --- |
  | Item Type | Journal Article |
  | Author | C. Signorelli |
  | Author | E.G. Robertson |
  | Author | C. Valentin |
  | Author | J.E. Alchin |
  | Author | C. Treadgold |
  | Date | 2023 |
  | Archive | Scopus |
  | URL | https://www.scopus.com/inward/record.uri?eid=2-s2.0-85176762881&doi=10.1542%2Fhpeds.2022-006994&partnerID=40&md5=6ad12d83ad8215c3a626ca7a20b59547 |
  | Volume | 13 |
  | Pages | E355-E364 |
  | Publication | Hospital Pediatrics |
  | DOI | 10.1542/hpeds.2022-006994 |
  | Issue | 11 |
  | Date Added | 05/02/2026, 16:38:58 |
  | Modified | 05/02/2026, 16:38:58 |

  ### Notes:

  - Export Date: 05 February 2026; Cited By: 7
- ## Non-randomized controlled trial examining the effects of livestock on motivation and anxiety in patients with chronic psychiatric disorders

  |  |  |
  | --- | --- |
  | Item Type | Journal Article |
  | Author | N. Shimizu |
  | Author | C. Yamazaki |
  | Author | K. Asano |
  | Author | S. Ohe |
  | Author | M. Ishida |
  | Date | 2023 |
  | Archive | Scopus |
  | URL | https://www.scopus.com/inward/record.uri?eid=2-s2.0-85160413096&doi=10.1177%2F20503121231175291&partnerID=40&md5=5e36d82e8b52f60a94c73f62283bac44 |
  | Volume | 11 |
  | Publication | SAGE Open Medicine |
  | DOI | 10.1177/20503121231175291 |
  | Date Added | 05/02/2026, 16:38:58 |
  | Modified | 05/02/2026, 16:38:58 |

  ### Notes:

  - Export Date: 05 February 2026; Cited By: 2
- ## Acute salivary cortisol response in children with ADHD during psychosocial intervention with and without therapy dogs

  |  |  |
  | --- | --- |
  | Item Type | Journal Article |
  | Author | S.E.B. Schuck |
  | Author | C.N. Zeiler |
  | Author | A. Stehli |
  | Author | L.A. Steinhoff |
  | Author | R.Y. Stokes |
  | Author | S.E. Jeffrey |
  | Author | D.A. Granger |
  | Date | 2024 |
  | Archive | Scopus |
  | URL | https://www.scopus.com/inward/record.uri?eid=2-s2.0-85208633259&doi=10.3389%2Ffpsyt.2024.1476522&partnerID=40&md5=06e7816a3c38bfb8c4b529418332294f |
  | Volume | 15 |
  | Publication | Frontiers in Psychiatry |
  | DOI | 10.3389/fpsyt.2024.1476522 |
  | Date Added | 05/02/2026, 16:38:58 |
  | Modified | 05/02/2026, 16:38:58 |

  ### Notes:

  - Export Date: 05 February 2026; Cited By: 0
- ## Human Enjoyment in Tactile Interaction With Horses and Dogs: A Comparative Study

  |  |  |
  | --- | --- |
  | Item Type | Journal Article |
  | Author | A. Sarrafchi |
  | Author | E. Lassallette |
  | Author | N. de Zwaan |
  | Author | M. Tucker |
  | Author | K. Merkies |
  | Date | 2025 |
  | Archive | Scopus |
  | URL | https://www.scopus.com/inward/record.uri?eid=2-s2.0-105023897384&doi=10.1080%2F08927936.2025.2578073&partnerID=40&md5=c01d3a77514fdabd67dabc3ab3a8a8ef |
  | Volume | 38 |
  | Pages | 1015-1031 |
  | Publication | Anthrozoos |
  | DOI | 10.1080/08927936.2025.2578073 |
  | Issue | 6 |
  | Date Added | 05/02/2026, 16:38:57 |
  | Modified | 05/02/2026, 16:38:57 |

  ### Notes:

  - Export Date: 05 February 2026; Cited By: 0
- ## Animal Assisted Activities (AAAs) with Dogs in a Dialysis Center in Southern Italy: Evaluation of Serotonin and Oxytocin Values in Involved Patients

  |  |  |
  | --- | --- |
  | Item Type | Journal Article |
  | Author | A. Santaniello |
  | Author | G. Perruolo |
  | Author | A. Amato |
  | Author | S. Garzillo |
  | Author | F. Mormone |
  | Author | C. Morelli |
  | Author | P. Formisano |
  | Author | M. Sansone |
  | Author | A. Fioretti |
  | Author | F. Oriente |
  | Date | 2025 |
  | Archive | Scopus |
  | URL | https://www.scopus.com/inward/record.uri?eid=2-s2.0-105026494180&doi=10.3390%2Fbiomedicines13122944&partnerID=40&md5=1eef06bee04ff40ae5ae79124b7189be |
  | Volume | 13 |
  | Publication | Biomedicines |
  | DOI | 10.3390/biomedicines13122944 |
  | Issue | 12 |
  | Date Added | 05/02/2026, 16:38:57 |
  | Modified | 05/02/2026, 16:38:57 |

  ### Notes:

  - Export Date: 05 February 2026; Cited By: 0
- ## A Pre-Screening Tool to Assess Dog Suitability for Animal-Assisted Interventions: Preliminary Results for Dog-Suitability Tests (SuiTe)

  |  |  |
  | --- | --- |
  | Item Type | Journal Article |
  | Author | G. Russo |
  | Author | C. Borrelli |
  | Author | G. Riggio |
  | Author | E. Rosson |
  | Author | M. Bentivoglio |
  | Author | C. Mariti |
  | Date | 2025 |
  | Archive | Scopus |
  | URL | https://www.scopus.com/inward/record.uri?eid=2-s2.0-105026097043&doi=10.3390%2Fvetsci12121110&partnerID=40&md5=3902c8ac6f3b87b0855238eca5fd4f88 |
  | Volume | 12 |
  | Publication | Veterinary Sciences |
  | DOI | 10.3390/vetsci12121110 |
  | Issue | 12 |
  | Date Added | 05/02/2026, 16:38:57 |
  | Modified | 05/02/2026, 16:38:57 |

  ### Notes:

  - Export Date: 05 February 2026; Cited By: 0
- ## Sustained effects of animal-assisted crisis response on stress in school shooting survivors

  |  |  |
  | --- | --- |
  | Item Type | Book Section |
  | Author | A.E. Robino |
  | Author | D.M. Feldman |
  | Author | A.N. Stein |
  | Author | M.A. Schmaltz |
  | Author | H.A. Fitzpatrick |
  | Author | J.L. Tartar |
  | Author | F. Pizzo |
  | Author | M. Friedman |
  | Author | O. Feldman |
  | Date | 2024 |
  | Archive | Scopus |
  | URL | https://www.scopus.com/inward/record.uri?eid=2-s2.0-85213926833&doi=10.1079%2F9781800626539.0003&partnerID=40&md5=26f9b097f31a1e0820c878787b73eeb7 |
  | Pages | 27-41 |
  | Book Title | The Impact of Therapy and Pet Animals on Human Stress |
  | DOI | 10.1079/9781800626539.0003 |
  | Date Added | 05/02/2026, 16:38:57 |
  | Modified | 05/02/2026, 16:38:57 |

  ### Notes:

  - Export Date: 05 February 2026; Cited By: 0
- ## Sustained Effects of Animal-Assisted Crisis Response on Stress in School Shooting Survivors

  |  |  |
  | --- | --- |
  | Item Type | Journal Article |
  | Author | A.E. Robino |
  | Author | D.M. Feldman |
  | Author | A.N. Stein |
  | Author | M.A. Schmaltz |
  | Author | H.A. Fitzpatrick |
  | Author | J.L. Tartar |
  | Author | F. Pizzo |
  | Author | M. Friedman |
  | Author | O. Feldman |
  | Date | 2022 |
  | Archive | Scopus |
  | URL | https://www.scopus.com/inward/record.uri?eid=2-s2.0-85164856998&doi=10.1079%2Fhai.2022.0019&partnerID=40&md5=b675bb48b83daee0dd14f6a54a25f1e1 |
  | Volume | 12 |
  | Pages | 65-85 |
  | Publication | Human-Animal Interactions |
  | DOI | 10.1079/hai.2022.0019 |
  | Issue | 2 |
  | Date Added | 05/02/2026, 16:38:58 |
  | Modified | 05/02/2026, 16:38:58 |

  ### Notes:

  - Export Date: 05 February 2026; Cited By: 4
- ## Urinary oxytocin levels in children meeting a Hospital Dog®

  |  |  |
  | --- | --- |
  | Item Type | Journal Article |
  | Author | A. Risberg |
  | Author | A. Larsson |
  | Author | U. Bodén |
  | Author | A. Edner |
  | Date | 2025 |
  | Archive | Scopus |
  | URL | https://www.scopus.com/inward/record.uri?eid=2-s2.0-105015193868&doi=10.1186%2Fs12906-025-05076-6&partnerID=40&md5=1d56fd2409e47b2b82f8c8614aa9ad40 |
  | Volume | 25 |
  | Publication | BMC Complementary Medicine and Therapies |
  | DOI | 10.1186/s12906-025-05076-6 |
  | Issue | 1 |
  | Date Added | 05/02/2026, 16:38:57 |
  | Modified | 05/02/2026, 16:38:57 |

  ### Notes:

  - Export Date: 05 February 2026; Cited By: 0
- ## The role of cortisol in the association of canine-companionship with blood pressure, glucose, and lipids: a systematic review

  |  |  |
  | --- | --- |
  | Item Type | Journal Article |
  | Author | D. Rathish |
  | Author | R.P.V.J. Rajapakse |
  | Author | K.G.A.D. Weerakoon |
  | Date | 2021 |
  | Archive | Scopus |
  | URL | https://www.scopus.com/inward/record.uri?eid=2-s2.0-85111905880&doi=10.1007%2Fs40292-021-00469-3&partnerID=40&md5=f8f4a0b144f528ea9c7e178388aa6cc5 |
  | Volume | 28 |
  | Pages | 447-455 |
  | Publication | High Blood Pressure and Cardiovascular Prevention |
  | DOI | 10.1007/s40292-021-00469-3 |
  | Issue | 5 |
  | Date Added | 05/02/2026, 16:38:58 |
  | Modified | 05/02/2026, 16:38:58 |

  ### Notes:

  - Export Date: 05 February 2026; Cited By: 13
- ## Effects of human and animal-assisted skills training on oxytocin und cortisol levels in patients with borderline personality disorder

  |  |  |
  | --- | --- |
  | Item Type | Journal Article |
  | Author | O. Plett |
  | Author | V. Flasbeck |
  | Author | M. Brüne |
  | Date | 2023 |
  | Archive | Scopus |
  | URL | https://www.scopus.com/inward/record.uri?eid=2-s2.0-85156248478&doi=10.1016%2Fj.jpsychires.2023.05.004&partnerID=40&md5=46e5b1f33103bfb3ac0d5b3f08039a94 |
  | Volume | 162 |
  | Pages | 156-160 |
  | Publication | Journal of Psychiatric Research |
  | DOI | 10.1016/j.jpsychires.2023.05.004 |
  | Date Added | 05/02/2026, 16:38:58 |
  | Modified | 05/02/2026, 16:38:58 |

  ### Notes:

  - Export Date: 05 February 2026; Cited By: 4
- ## Dog-assisted therapy for control of anxiety in pediatric dentistry

  |  |  |
  | --- | --- |
  | Item Type | Journal Article |
  | Author | S.L. Pinheiro |
  | Author | C. Silva |
  | Author | L. Luiz |
  | Author | N. Silva |
  | Author | R. Fonseca |
  | Author | T. Velásquez |
  | Author | D.R. Grandizoli |
  | Date | 2023 |
  | Archive | Scopus |
  | URL | https://www.scopus.com/inward/record.uri?eid=2-s2.0-85176743541&doi=10.22514%2Fjocpd.2023.080&partnerID=40&md5=a414d49b487e557603924fdd4191ec08 |
  | Volume | 47 |
  | Pages | 38-43 |
  | Publication | Journal of Clinical Pediatric Dentistry |
  | DOI | 10.22514/jocpd.2023.080 |
  | Issue | 6 |
  | Date Added | 05/02/2026, 16:38:58 |
  | Modified | 05/02/2026, 16:38:58 |

  ### Notes:

  - Export Date: 05 February 2026; Cited By: 3
- ## Wildlife and wellbeing: Wildlife immersion experiences in veterans with PTSD

  |  |  |
  | --- | --- |
  | Item Type | Journal Article |
  | Author | D.J. Perry |
  | Author | S.L. Crawford |
  | Author | J.J. Averka |
  | Author | J.M. Mackin |
  | Author | D.A. Granger |
  | Author | D.A. Smelson |
  | Date | 2025 |
  | Archive | Scopus |
  | URL | https://www.scopus.com/inward/record.uri?eid=2-s2.0-105015062777&doi=10.1079%2Fhai.2025.0006&partnerID=40&md5=3946f6cda7a9a42024c98ceb4ee2ad54 |
  | Volume | 13 |
  | Publication | Human-Animal Interactions |
  | DOI | 10.1079/hai.2025.0006 |
  | Issue | 1 |
  | Date Added | 05/02/2026, 16:38:57 |
  | Modified | 05/02/2026, 16:38:57 |

  ### Notes:

  - Export Date: 05 February 2026; Cited By: 1
- ## Effects of University-Based AAIs: Conceptual Models Guiding Research on Active Treatment Components of AAIs on Stress-Related Outcomes

  |  |  |
  | --- | --- |
  | Item Type | Book Section |
  | Author | P. Pendry |
  | Author | A.M. Carr |
  | Date | 2023 |
  | Archive | Scopus |
  | URL | https://www.scopus.com/inward/record.uri?eid=2-s2.0-85159404327&doi=10.1007%2F978-3-031-29789-2\_5&partnerID=40&md5=74ac2226cc65b5f916491629c56bbba6 |
  | Volume | 69 |
  | Pages | 91-116 |
  | Book Title | Nebraska Symposium on Motivation |
  | DOI | 10.1007/978-3-031-29789-2\_5 |
  | Date Added | 05/02/2026, 16:38:58 |
  | Modified | 05/02/2026, 16:38:58 |

  ### Notes:

  - Export Date: 05 February 2026; Cited By: 1
- ## Dog companionship and cortisol levels in youth. A systematic review and meta-analysis

  |  |  |
  | --- | --- |
  | Item Type | Journal Article |
  | Author | H. Peña-Jorquera |
  | Author | S. Hernández-Jaña |
  | Author | J. Sanchez-Martinez |
  | Author | J.P. Espinoza-Puelles |
  | Author | R. Martinez-Flores |
  | Author | F. Schuch |
  | Author | R. Yánez-Sepúlveda |
  | Author | P. Delgado-Floody |
  | Author | G. Ferrari |
  | Author | K.P. Sadarangani |
  | Author | J. Cancino-López |
  | Author | J. Bento-Torres |
  | Author | A. Espinoza-Salinas |
  | Author | E. Stamatakis |
  | Author | C. Cristi-Montero |
  | Date | 2025 |
  | Archive | Scopus |
  | URL | https://www.scopus.com/inward/record.uri?eid=2-s2.0-85217709541&doi=10.1016%2Fj.socscimed.2025.117815&partnerID=40&md5=291b6d177bba8040248e259dd47ac8b6 |
  | Volume | 369 |
  | Publication | Social Science and Medicine |
  | DOI | 10.1016/j.socscimed.2025.117815 |
  | Date Added | 05/02/2026, 16:38:57 |
  | Modified | 05/02/2026, 16:38:57 |

  ### Notes:

  - Export Date: 05 February 2026; Cited By: 1
- ## Effects of a Canine-Assisted Intervention in Perceived and Physiological Stress of Spanish University Students

  |  |  |
  | --- | --- |
  | Item Type | Journal Article |
  | Author | D. Peña Gil |
  | Author | C. Camilli-Trujillo |
  | Author | M.G. García-García |
  | Date | 2023 |
  | Archive | Scopus |
  | URL | https://www.scopus.com/inward/record.uri?eid=2-s2.0-85172345975&doi=10.1163%2F15685306-bja10146&partnerID=40&md5=37f742f53776570bd643bfd33b85c432 |
  | Volume | 23 |
  | Pages | 1-20 |
  | Publication | Society and Animals |
  | DOI | 10.1163/15685306-bja10146 |
  | Issue | 2 |
  | Date Added | 05/02/2026, 16:38:58 |
  | Modified | 05/02/2026, 16:38:58 |

  ### Notes:

  - Export Date: 05 February 2026; Cited By: 1
- ## Ethological and physiological parameters assessment in donkeys used in animal assisted interventions

  |  |  |
  | --- | --- |
  | Item Type | Journal Article |
  | Author | M. Panzera |
  | Author | D. Alberghina |
  | Author | A. Statelli |
  | Date | 2020 |
  | Archive | Scopus |
  | URL | https://www.scopus.com/inward/record.uri?eid=2-s2.0-85092541771&doi=10.3390%2Fani10101867&partnerID=40&md5=23d17ad7cf37edd6031a041950bcf1a3 |
  | Volume | 10 |
  | Pages | 1-24 |
  | Publication | Animals |
  | DOI | 10.3390/ani10101867 |
  | Issue | 10 |
  | Date Added | 05/02/2026, 16:38:58 |
  | Modified | 05/02/2026, 16:38:58 |

  ### Notes:

  - Export Date: 05 February 2026; Cited By: 15
- ## Heart Rate Variability Spectral Analysis for Monitoring Autonomic Activation in a Donkey Involved in Animal-Assisted Therapy: A Single Subject Design During Animal-Assisted Therapy Sessions

  |  |  |
  | --- | --- |
  | Item Type | Journal Article |
  | Author | M. Panzera |
  | Author | A. Statelli |
  | Date | 2025 |
  | Archive | Scopus |
  | URL | https://www.scopus.com/inward/record.uri?eid=2-s2.0-105026083652&doi=10.3390%2Fvetsci12121131&partnerID=40&md5=425b259666214877e4db57588e908e65 |
  | Volume | 12 |
  | Publication | Veterinary Sciences |
  | DOI | 10.3390/vetsci12121131 |
  | Issue | 12 |
  | Date Added | 05/02/2026, 16:38:57 |
  | Modified | 05/02/2026, 16:38:57 |

  ### Notes:

  - Export Date: 05 February 2026; Cited By: 0
- ## The Effect of Interaction with a Dog on Heart Rate Variability based on Lorenz Plot Analysis

  |  |  |
  | --- | --- |
  | Item Type | Journal Article |
  | Author | I. Nose |
  | Author | K. Masamoto |
  | Author | A. Tsuchida |
  | Author | M. Hayashi |
  | Author | M. Irimajiri |
  | Author | M. Kakinuma |
  | Date | 2022 |
  | Archive | Scopus |
  | URL | https://www.scopus.com/inward/record.uri?eid=2-s2.0-85193840594&doi=10.1079%2Fhai.2022.0004&partnerID=40&md5=1b844d832b0edc42ed610f9fc4327b75 |
  | Volume | 10 |
  | Pages | 84-99 |
  | Publication | Human-Animal Interactions |
  | DOI | 10.1079/hai.2022.0004 |
  | Issue | 1 |
  | Date Added | 05/02/2026, 16:38:58 |
  | Modified | 05/02/2026, 16:38:58 |

  ### Notes:

  - Export Date: 05 February 2026; Cited By: 1
- ## Survey of international academic centers and institutes focused on the human-animal bond: Scope and landscape in 2021

  |  |  |
  | --- | --- |
  | Item Type | Journal Article |
  | Author | L.O. Nieforth |
  | Author | S.C. Leighton |
  | Author | E.A. Miller |
  | Author | M.E. O'Haire |
  | Date | 2022 |
  | Archive | Scopus |
  | URL | https://www.scopus.com/inward/record.uri?eid=2-s2.0-85192812959&doi=10.1079%2Fhai.2022.0026&partnerID=40&md5=ece2bfd3da0eaffcbac7ab2bf7421835 |
  | Volume | 2022 |
  | Publication | Human-Animal Interactions |
  | DOI | 10.1079/hai.2022.0026 |
  | Date Added | 05/02/2026, 16:38:58 |
  | Modified | 05/02/2026, 16:38:58 |

  ### Notes:

  - Export Date: 05 February 2026; Cited By: 5
- ## Editorial: Insights in animal behavior and welfare: 2021

  |  |  |
  | --- | --- |
  | Item Type | Journal Article |
  | Author | E. Narayan |
  | Date | 2022 |
  | Archive | Scopus |
  | URL | https://www.scopus.com/inward/record.uri?eid=2-s2.0-85136066284&doi=10.3389%2Ffvets.2022.988463&partnerID=40&md5=8f3c7b3c9a5dee6cae43a35394a203b6 |
  | Volume | 9 |
  | Publication | Frontiers in Veterinary Science |
  | DOI | 10.3389/fvets.2022.988463 |
  | Date Added | 05/02/2026, 16:38:58 |
  | Modified | 05/02/2026, 16:38:58 |

  ### Notes:

  - Export Date: 05 February 2026; Cited By: 0
- ## Neurodynamics of patients during a dolphin-assisted therapy by means of a fractal intraneural analysis

  |  |  |
  | --- | --- |
  | Item Type | Journal Article |
  | Author | O. Morales-Matamoros |
  | Author | J.J. Escobar |
  | Author | R.T. Padilla |
  | Author | I.L. Reyes |
  | Date | 2020 |
  | Archive | Scopus |
  | URL | https://www.scopus.com/inward/record.uri?eid=2-s2.0-85086914555&doi=10.3390%2Fbrainsci10060403&partnerID=40&md5=2048612c29667a30ab8d0bfaac6eb6b3 |
  | Volume | 10 |
  | Pages | 1-23 |
  | Publication | Brain Sciences |
  | DOI | 10.3390/brainsci10060403 |
  | Issue | 6 |
  | Date Added | 05/02/2026, 16:38:58 |
  | Modified | 05/02/2026, 16:38:58 |

  ### Notes:

  - Export Date: 05 February 2026; Cited By: 11
- ## The Importance of Evaluating Positive Welfare Characteristics and Temperament in Working Therapy Dogs

  |  |  |
  | --- | --- |
  | Item Type | Journal Article |
  | Author | S.L. Miller |
  | Author | J.A. Serpell |
  | Author | K.R. Dalton |
  | Author | K.B. Waite |
  | Author | D.O. Morris |
  | Author | L.E. Redding |
  | Author | N.A. Dreschel |
  | Author | M.F. Davis |
  | Date | 2022 |
  | Archive | Scopus |
  | URL | https://www.scopus.com/inward/record.uri?eid=2-s2.0-85128455598&doi=10.3389%2Ffvets.2022.844252&partnerID=40&md5=9a02c155daf0799b2a6a24ae589841f3 |
  | Volume | 9 |
  | Publication | Frontiers in Veterinary Science |
  | DOI | 10.3389/fvets.2022.844252 |
  | Date Added | 05/02/2026, 16:38:58 |
  | Modified | 05/02/2026, 16:38:58 |

  ### Notes:

  - Export Date: 05 February 2026; Cited By: 17
- ## Animal-Assisted Interventions With Dogs in Special Education—A Systematic Review

  |  |  |
  | --- | --- |
  | Item Type | Journal Article |
  | Author | J. Meixner |
  | Author | K. Kotrschal |
  | Date | 2022 |
  | Archive | Scopus |
  | URL | https://www.scopus.com/inward/record.uri?eid=2-s2.0-85132694943&doi=10.3389%2Ffpsyg.2022.876290&partnerID=40&md5=554c3cfd19f48626262df18fc6409c5a |
  | Volume | 13 |
  | Publication | Frontiers in Psychology |
  | DOI | 10.3389/fpsyg.2022.876290 |
  | Date Added | 05/02/2026, 16:38:58 |
  | Modified | 05/02/2026, 16:38:58 |

  ### Notes:

  - Export Date: 05 February 2026; Cited By: 14
- ## Can dogs reduce stress levels in school children? effects of dog-assisted interventions on salivary cortisol in children with and without special educational needs using randomized controlled trials

  |  |  |
  | --- | --- |
  | Item Type | Journal Article |
  | Author | K. Meints |
  | Author | V.L. Brelsford |
  | Author | M. Dimolareva |
  | Author | L. Maréchal |
  | Author | K. Pennington |
  | Author | E. Rowan |
  | Author | N.R. Gee |
  | Date | 2022 |
  | Archive | Scopus |
  | URL | https://www.scopus.com/inward/record.uri?eid=2-s2.0-85132078819&doi=10.1371%2Fjournal.pone.0269333&partnerID=40&md5=bdbfa15a4d8bd4cf01776316dfc5b6ad |
  | Volume | 17 |
  | Publication | PLoS ONE |
  | DOI | 10.1371/journal.pone.0269333 |
  | Issue | 6 June |
  | Date Added | 05/02/2026, 16:38:58 |
  | Modified | 05/02/2026, 16:38:58 |

  ### Notes:

  - Export Date: 05 February 2026; Cited By: 35
- ## Psychophysiological effects of equine-facilitated psychotherapy on Veterans with PTSD and their horse partners

  |  |  |
  | --- | --- |
  | Item Type | Journal Article |
  | Author | L.A. McDuffee |
  | Author | W.J. Montelpare |
  | Author | C. LeBlanc |
  | Date | 2024 |
  | Archive | Scopus |
  | URL | https://www.scopus.com/inward/record.uri?eid=2-s2.0-85199695694&doi=10.3138%2Fjmvfh-2023-0063&partnerID=40&md5=c7122f75765b310330e5619dd252e8ad |
  | Volume | 10 |
  | Pages | 135-147 |
  | Publication | Journal of Military, Veteran and Family Health |
  | DOI | 10.3138/jmvfh-2023-0063 |
  | Issue | 3 |
  | Date Added | 05/02/2026, 16:38:58 |
  | Modified | 05/02/2026, 16:38:58 |

  ### Notes:

  - Export Date: 05 February 2026; Cited By: 2
- ## Cortisol changes in bottlenose dolphins in the dolphin interactive program

  |  |  |
  | --- | --- |
  | Item Type | Journal Article |
  | Author | M. Matsushiro |
  | Author | H. Kurono |
  | Author | K. Yamamoto |
  | Author | T. Kooriyama |
  | Date | 2021 |
  | Archive | Scopus |
  | URL | https://www.scopus.com/inward/record.uri?eid=2-s2.0-85108988697&doi=10.14943%2Fjjvr.69.2.99&partnerID=40&md5=3fd816c7126cdcf33a691723473e442b |
  | Volume | 69 |
  | Pages | 99-108 |
  | Publication | Japanese Journal of Veterinary Research |
  | DOI | 10.14943/jjvr.69.2.99 |
  | Issue | 2 |
  | Date Added | 05/02/2026, 16:38:58 |
  | Modified | 05/02/2026, 16:38:58 |

  ### Notes:

  - Export Date: 05 February 2026; Cited By: 4
- ## Evaluating effects of animal-assisted therapy on pediatric dental care patients: A pilot clinical trial

  |  |  |
  | --- | --- |
  | Item Type | Journal Article |
  | Author | J. Massouda |
  | Author | N. Ghaltakhchyan |
  | Author | J. Judd |
  | Author | C. Bocklage |
  | Author | R. Selden |
  | Author | O. TumSuden |
  | Author | E. Nanney |
  | Author | J. Lee |
  | Author | J. Ginnis |
  | Author | T. Strauman |
  | Author | C. Sawicki |
  | Author | E. Hodges |
  | Author | C. Graves |
  | Author | K. Divaris |
  | Author | L.A. Jacox |
  | Date | 2025 |
  | Archive | Scopus |
  | URL | https://www.scopus.com/inward/record.uri?eid=2-s2.0-105005574697&doi=10.1016%2Fj.adaj.2025.03.006&partnerID=40&md5=8c1b989e7d055f291263e23d75eb8d59 |
  | Volume | 156 |
  | Pages | 447-457.e14 |
  | Publication | Journal of the American Dental Association |
  | DOI | 10.1016/j.adaj.2025.03.006 |
  | Issue | 6 |
  | Date Added | 05/02/2026, 16:38:57 |
  | Modified | 05/02/2026, 16:38:57 |

  ### Notes:

  - Export Date: 05 February 2026; Cited By: 2
- ## Effects of contact with a dog on prefrontal brain activation in patients in a minimally conscious state: A controlled crossover trial

  |  |  |
  | --- | --- |
  | Item Type | Journal Article |
  | Author | R. Marti |
  | Author | M. Petignat |
  | Author | V.L. Marcar |
  | Author | J. Hattendorf |
  | Author | M. Wolf |
  | Author | M. Hund-Georgiadis |
  | Author | K. Hediger |
  | Date | 2025 |
  | Archive | Scopus |
  | URL | https://www.scopus.com/inward/record.uri?eid=2-s2.0-105005653323&doi=10.1016%2Fj.neuroscience.2025.05.014&partnerID=40&md5=5af3b202bf61d1ae22c27e0b0ee92081 |
  | Volume | 577 |
  | Pages | 175-189 |
  | Publication | Neuroscience |
  | DOI | 10.1016/j.neuroscience.2025.05.014 |
  | Date Added | 05/02/2026, 16:38:57 |
  | Modified | 05/02/2026, 16:38:57 |

  ### Notes:

  - Export Date: 05 February 2026; Cited By: 0
- ## Cognitive mechanisms and neurological foundations of companion animals’ role in enhancing human psychological well-being

  |  |  |
  | --- | --- |
  | Item Type | Journal Article |
  | Author | H. Liu |
  | Author | J. Lin |
  | Author | W. Lin |
  | Date | 2024 |
  | Archive | Scopus |
  | URL | https://www.scopus.com/inward/record.uri?eid=2-s2.0-85192464255&doi=10.3389%2Ffpsyg.2024.1354220&partnerID=40&md5=e29f0357365b2bf6e8c86caac8502281 |
  | Volume | 15 |
  | Publication | Frontiers in Psychology |
  | DOI | 10.3389/fpsyg.2024.1354220 |
  | Date Added | 05/02/2026, 16:38:58 |
  | Modified | 05/02/2026, 16:38:58 |

  ### Notes:

  - Export Date: 05 February 2026; Cited By: 4
- ## A Text-Mining Analysis of Research Trends in Animal-Assisted Therapy

  |  |  |
  | --- | --- |
  | Item Type | Journal Article |
  | Author | S.-J. Lee |
  | Author | G.-H. Kim |
  | Author | Y.-H. Moon |
  | Author | S.-S. Lee |
  | Date | 2023 |
  | Archive | Scopus |
  | URL | https://www.scopus.com/inward/record.uri?eid=2-s2.0-85173896459&doi=10.3390%2Fani13193133&partnerID=40&md5=0644768851e38d662135b6a024ccfd86 |
  | Volume | 13 |
  | Publication | Animals |
  | DOI | 10.3390/ani13193133 |
  | Issue | 19 |
  | Date Added | 05/02/2026, 16:38:58 |
  | Modified | 05/02/2026, 16:38:58 |

  ### Notes:

  - Export Date: 05 February 2026; Cited By: 2
- ## The effects of a therapy dog intervention on dental fear and anxiety in adult patients undergoing dental procedures: a pilot study

  |  |  |
  | --- | --- |
  | Item Type | Journal Article |
  | Author | D. Lam |
  | Author | D.A. D’Anthony |
  | Author | S.A. Chilcutt |
  | Author | A. O’Connor |
  | Author | A.J. Avillo |
  | Author | N.J. Hamlin |
  | Author | J.E. Schmidt |
  | Date | 2024 |
  | Archive | Scopus |
  | URL | https://www.scopus.com/inward/record.uri?eid=2-s2.0-85196893362&partnerID=40&md5=8410a1f35812d1fc33e9c85337d4cc39 |
  | Volume | 72 |
  | Pages | 44-49 |
  | Publication | General Dentistry |
  | Issue | 4 |
  | Date Added | 05/02/2026, 16:38:58 |
  | Modified | 05/02/2026, 16:38:58 |

  ### Notes:

  - Export Date: 05 February 2026; Cited By: 0
- ## Treating Agitation in Patients with Dementia with a Therapy Dog in a Milieu Therapy Setting on a Geropsychiatric Ward

  |  |  |
  | --- | --- |
  | Item Type | Journal Article |
  | Author | J. Krüger |
  | Author | R. Izgi |
  | Author | R. Hellweg |
  | Author | A. Ströhle |
  | Author | M.C. Jockers-Scherübl |
  | Date | 2022 |
  | Archive | Scopus |
  | URL | https://www.scopus.com/inward/record.uri?eid=2-s2.0-85122326069&doi=10.1159%2F000520881&partnerID=40&md5=c4a26298ed6e0770e465ed1d7b9b9525 |
  | Volume | 50 |
  | Pages | 541-547 |
  | Publication | Dementia and Geriatric Cognitive Disorders |
  | DOI | 10.1159/000520881 |
  | Issue | 6 |
  | Date Added | 05/02/2026, 16:38:58 |
  | Modified | 05/02/2026, 16:38:58 |

  ### Notes:

  - Export Date: 05 February 2026; Cited By: 1
- ## Veterans Training Service Dogs for Other Veterans: An Animal-Assisted Intervention for Post-Traumatic Stress Disorder

  |  |  |
  | --- | --- |
  | Item Type | Journal Article |
  | Author | C.A. Krause-Parello |
  | Author | E. Friedmann |
  | Author | D. Taber |
  | Author | H. Zhu |
  | Author | A. Quintero |
  | Author | R. Yount |
  | Date | 2025 |
  | Archive | Scopus |
  | URL | https://www.scopus.com/inward/record.uri?eid=2-s2.0-105017429771&doi=10.3390%2Fbs15091180&partnerID=40&md5=6adc8e43c0c96ce8c3fa9b1bf814b06c |
  | Volume | 15 |
  | Publication | Behavioral Sciences |
  | DOI | 10.3390/bs15091180 |
  | Issue | 9 |
  | Date Added | 05/02/2026, 16:38:57 |
  | Modified | 05/02/2026, 16:38:57 |

  ### Notes:

  - Export Date: 05 February 2026; Cited By: 1
- ## Are therapy animals the key to happier dental visits for children?

  |  |  |
  | --- | --- |
  | Item Type | Journal Article |
  | Author | S. Khan |
  | Date | 2025 |
  | Archive | Scopus |
  | URL | https://www.scopus.com/inward/record.uri?eid=2-s2.0-105024333581&doi=10.1038%2Fs41432-025-01197-6&partnerID=40&md5=89ae04095ff51341ffc13f201614420f |
  | Volume | 26 |
  | Pages | 174-175 |
  | Publication | Evidence-Based Dentistry |
  | DOI | 10.1038/s41432-025-01197-6 |
  | Issue | 4 |
  | Date Added | 05/02/2026, 16:38:57 |
  | Modified | 05/02/2026, 16:38:57 |

  ### Notes:

  - Export Date: 05 February 2026; Cited By: 0
- ## Therapy Dogs for Anxiety in Children in the Emergency Department A Randomized Clinical Trial

  |  |  |
  | --- | --- |
  | Item Type | Journal Article |
  | Author | H.P. Kelker |
  | Author | H.K. Siddiqui |
  | Author | A.M. Beck |
  | Author | J.A. Kline |
  | Date | 2025 |
  | Archive | Scopus |
  | URL | https://www.scopus.com/inward/record.uri?eid=2-s2.0-105001222418&doi=10.1001%2Fjamanetworkopen.2025.0636&partnerID=40&md5=cbb2417e129007a786b8e85da79896ab |
  | Volume | 8 |
  | Publication | JAMA Network Open |
  | DOI | 10.1001/jamanetworkopen.2025.0636 |
  | Issue | 3 |
  | Date Added | 05/02/2026, 16:38:57 |
  | Modified | 05/02/2026, 16:38:57 |

  ### Notes:

  - Export Date: 05 February 2026; Cited By: 4
- ## Evaluation of cortisol levels and behavior in dogs during animal-assisted interventions in clinical practice

  |  |  |
  | --- | --- |
  | Item Type | Journal Article |
  | Author | K. Kateřina |
  | Author | M. Kristýna |
  | Author | P. Radka |
  | Author | M. Aneta |
  | Author | Z. Štěpán |
  | Author | S. Ivona |
  | Date | 2024 |
  | Archive | Scopus |
  | URL | https://www.scopus.com/inward/record.uri?eid=2-s2.0-85197028877&doi=10.1016%2Fj.applanim.2024.106321&partnerID=40&md5=5c4de80401383955fc71bfb40e785b9f |
  | Volume | 277 |
  | Publication | Applied Animal Behaviour Science |
  | DOI | 10.1016/j.applanim.2024.106321 |
  | Date Added | 05/02/2026, 16:38:58 |
  | Modified | 05/02/2026, 16:38:58 |

  ### Notes:

  - Export Date: 05 February 2026; Cited By: 4
- ## Effects of animal-assisted therapy on dental anxiety, behavior, and perceptions in young pediatric patients: a blinded randomized controlled trial

  |  |  |
  | --- | --- |
  | Item Type | Journal Article |
  | Author | G. Kapov |
  | Author | K. Linton |
  | Author | C. Gatewood |
  | Author | C. Liu |
  | Author | T. Strauman |
  | Author | E. Hodges |
  | Author | C. Graves |
  | Author | C. Sawicki |
  | Author | D. Wu |
  | Author | K. Divaris |
  | Author | L.A. Jacox |
  | Date | 2025 |
  | Archive | Scopus |
  | URL | https://www.scopus.com/inward/record.uri?eid=2-s2.0-105012853707&doi=10.1186%2Fs13063-025-08970-z&partnerID=40&md5=9272a39a0b4e496dd24a20028aea4a52 |
  | Volume | 26 |
  | Publication | Trials |
  | DOI | 10.1186/s13063-025-08970-z |
  | Issue | 1 |
  | Date Added | 05/02/2026, 16:38:57 |
  | Modified | 05/02/2026, 16:38:57 |

  ### Notes:

  - Export Date: 05 February 2026; Cited By: 1
- ## The Effects of Human–Horse Interactions on Oxytocin and Cortisol Levels in Humans and Horses

  |  |  |
  | --- | --- |
  | Item Type | Journal Article |
  | Author | Y. Jung |
  | Author | M. Yoon |
  | Date | 2025 |
  | Archive | Scopus |
  | URL | https://www.scopus.com/inward/record.uri?eid=2-s2.0-105002461479&doi=10.3390%2Fani15070905&partnerID=40&md5=6b051d28b087bf7034e1bc8c80cb21a6 |
  | Volume | 15 |
  | Publication | Animals |
  | DOI | 10.3390/ani15070905 |
  | Issue | 7 |
  | Date Added | 05/02/2026, 16:38:57 |
  | Modified | 05/02/2026, 16:38:57 |

  ### Notes:

  - Export Date: 05 February 2026; Cited By: 7
- ## Effect of animal assisted interactions on activity and stress response in children in acute care settings

  |  |  |
  | --- | --- |
  | Item Type | Journal Article |
  | Author | M.L. Jennings |
  | Author | D.A. Granger |
  | Author | C.I. Bryce |
  | Author | D. Twitchell |
  | Author | K. Yeakel |
  | Author | P.A. Teaford |
  | Date | 2021 |
  | Archive | Scopus |
  | URL | https://www.scopus.com/inward/record.uri?eid=2-s2.0-85137965791&doi=10.1016%2Fj.cpnec.2021.100076&partnerID=40&md5=d7671d201814b617e8eea3de5dac9c98 |
  | Volume | 8 |
  | Publication | Comprehensive Psychoneuroendocrinology |
  | DOI | 10.1016/j.cpnec.2021.100076 |
  | Date Added | 05/02/2026, 16:38:58 |
  | Modified | 05/02/2026, 16:38:58 |

  ### Notes:

  - Export Date: 05 February 2026; Cited By: 16
- ## Recovering from Trauma with the Support of Animals: Interventions for Adult Posttraumatic Stress and Child Maltreatment

  |  |  |
  | --- | --- |
  | Item Type | Book Section |
  | Author | M.A. Jenkins |
  | Author | P. Tedeschi |
  | Author | M.D. Olmert |
  | Author | R. Yount |
  | Author | N. Parish-Plass |
  | Author | A. Leslie |
  | Date | 2024 |
  | Archive | Scopus |
  | URL | https://www.scopus.com/inward/record.uri?eid=2-s2.0-85208870681&doi=10.1016%2FB978-0-443-22346-4.15010-3&partnerID=40&md5=7a7ecc5fee007017660d19dee296901e |
  | Pages | 401-422 |
  | Book Title | Handbook on Animal-Assisted Therapy |
  | DOI | 10.1016/B978-0-443-22346-4.15010-3 |
  | Date Added | 05/02/2026, 16:38:58 |
  | Modified | 05/02/2026, 16:38:58 |

  ### Notes:

  - Export Date: 05 February 2026; Cited By: 0
- ## Companion Animals and Health in Older Populations: A Systematic Review

  |  |  |
  | --- | --- |
  | Item Type | Journal Article |
  | Author | M.J. Hughes |
  | Author | M.-L. Verreynne |
  | Author | P. Harpur |
  | Author | N.A. Pachana |
  | Date | 2020 |
  | Archive | Scopus |
  | URL | https://www.scopus.com/inward/record.uri?eid=2-s2.0-85071034661&doi=10.1080%2F07317115.2019.1650863&partnerID=40&md5=5a622da6b8ace7a96c6868b39c241ab9 |
  | Volume | 43 |
  | Pages | 365-377 |
  | Publication | Clinical Gerontologist |
  | DOI | 10.1080/07317115.2019.1650863 |
  | Issue | 4 |
  | Date Added | 05/02/2026, 16:38:58 |
  | Modified | 05/02/2026, 16:38:58 |

  ### Notes:

  - Export Date: 05 February 2026; Cited By: 81
- ## Contact-Free Simultaneous Sensing of Human Heart Rate and Canine Breathing Rate for Animal Assisted Interactions

  |  |  |
  | --- | --- |
  | Item Type | Conference Paper |
  | Author | T. Holder |
  | Author | M. Rahman |
  | Author | E. Summers |
  | Author | D. Roberts |
  | Author | C.-W. Wong |
  | Author | A. Bozkurt |
  | Date | 2022 |
  | Archive | Scopus |
  | URL | https://www.scopus.com/inward/record.uri?eid=2-s2.0-85152192261&doi=10.1145%2F3565995.3566039&partnerID=40&md5=4532d9ebb1ddfaab126a2446893d6f26 |
  | Conference Name | ACM International Conference Proceeding Series |
  | DOI | 10.1145/3565995.3566039 |
  | Date Added | 05/02/2026, 16:38:58 |
  | Modified | 05/02/2026, 16:38:58 |

  ### Notes:

  - Export Date: 05 February 2026; Cited By: 3
- ## Reducing Anxiety and Stress among Youth in a CBT-Based Equine-Assisted Adaptive Riding Program

  |  |  |
  | --- | --- |
  | Item Type | Journal Article |
  | Author | K. Hoagwood |
  | Author | A. Vincent |
  | Author | M. Acri |
  | Author | M. Morrissey |
  | Author | L. Seibel |
  | Author | F. Guo |
  | Author | C. Flores |
  | Author | D. Seag |
  | Author | R. Peth-Pierce |
  | Author | S. Horwitz |
  | Date | 2022 |
  | Archive | Scopus |
  | URL | https://www.scopus.com/inward/record.uri?eid=2-s2.0-85139785963&doi=10.3390%2Fani12192491&partnerID=40&md5=1acce2952ae9c152c3fe9d9ba2d8167b |
  | Volume | 12 |
  | Publication | Animals |
  | DOI | 10.3390/ani12192491 |
  | Issue | 19 |
  | Date Added | 05/02/2026, 16:38:58 |
  | Modified | 05/02/2026, 16:38:58 |

  ### Notes:

  - Export Date: 05 February 2026; Cited By: 22
- ## Psychosocial Interventions Impact on Cardiometabolic, Neurobiological, Behavioral, and Immune Outcomes in People With a Serious Mental Illness: A Systematic Review

  |  |  |
  | --- | --- |
  | Item Type | Journal Article |
  | Author | J.C. Hill |
  | Author | C. Noller |
  | Author | P. Holtzheimer |
  | Author | K. DiSano |
  | Author | E. Klinsky |
  | Author | M. Pudasaini |
  | Author | H. Crowe-Cumella |
  | Author | K.L. Fortuna |
  | Date | 2025 |
  | Archive | Scopus |
  | URL | https://www.scopus.com/inward/record.uri?eid=2-s2.0-105011031538&doi=10.1002%2Fhsr2.70954&partnerID=40&md5=3fe071e19e76048037bcafc105b26677 |
  | Volume | 8 |
  | Publication | Health Science Reports |
  | DOI | 10.1002/hsr2.70954 |
  | Issue | 7 |
  | Date Added | 05/02/2026, 16:38:57 |
  | Modified | 05/02/2026, 16:38:57 |

  ### Notes:

  - Export Date: 05 February 2026; Cited By: 1
- ## Investigating Non-Pharmacological Stress Reduction Interventions in Pediatric Patients Confirmed with Salivary Cortisol Levels: A Systematic Review

  |  |  |
  | --- | --- |
  | Item Type | Journal Article |
  | Author | M. Grigoropoulou |
  | Author | E.I. Kapetanakis |
  | Author | A. Attilakos |
  | Author | A. Charalampopoulos |
  | Author | A. Dimopoulou |
  | Author | E. Vamvakas |
  | Author | E. Mavrigiannaki |
  | Author | N. Zavras |
  | Date | 2023 |
  | Archive | Scopus |
  | URL | https://www.scopus.com/inward/record.uri?eid=2-s2.0-85163668728&doi=10.3390%2Fpediatric15020031&partnerID=40&md5=9a1445d107587b2511caae53b3c2ede3 |
  | Volume | 15 |
  | Pages | 349-359 |
  | Publication | Pediatric Reports |
  | DOI | 10.3390/pediatric15020031 |
  | Issue | 2 |
  | Date Added | 05/02/2026, 16:38:58 |
  | Modified | 05/02/2026, 16:38:58 |

  ### Notes:

  - Export Date: 05 February 2026; Cited By: 3
- ## Group Changes in Cortisol and Heart Rate Variability of Children with Down Syndrome and Children with Autism Spectrum Disorder during Dog-Assisted Therapy

  |  |  |
  | --- | --- |
  | Item Type | Journal Article |
  | Author | R.E. Griffioen |
  | Author | G.J.M. van Boxtel |
  | Author | T. Verheggen |
  | Author | M.-J. Enders-Slegers |
  | Author | S. van der Steen |
  | Date | 2023 |
  | Archive | Scopus |
  | URL | https://www.scopus.com/inward/record.uri?eid=2-s2.0-85166280471&doi=10.3390%2Fchildren10071200&partnerID=40&md5=58716932845f3c0454e76e600bd9471a |
  | Volume | 10 |
  | Publication | Children |
  | DOI | 10.3390/children10071200 |
  | Issue | 7 |
  | Date Added | 05/02/2026, 16:38:58 |
  | Modified | 05/02/2026, 16:38:58 |

  ### Notes:

  - Export Date: 05 February 2026; Cited By: 2
- ## Effects of Animal-Assisted Therapy (AAT) in Alzheimer’s Disease: A Case Study

  |  |  |
  | --- | --- |
  | Item Type | Journal Article |
  | Author | A. Gregorini |
  | Author | A. Di Canio |
  | Author | E. Palmucci |
  | Author | M. Tomasetti |
  | Author | M.B.L. Rocchi |
  | Author | M. Colomba |
  | Date | 2022 |
  | Archive | Scopus |
  | URL | https://www.scopus.com/inward/record.uri?eid=2-s2.0-85127461969&doi=10.3390%2Fhealthcare10030567&partnerID=40&md5=593a5056d4f5f84cceea97934c3a4ec9 |
  | Volume | 10 |
  | Publication | Healthcare (Switzerland) |
  | DOI | 10.3390/healthcare10030567 |
  | Issue | 3 |
  | Date Added | 05/02/2026, 16:38:58 |
  | Modified | 05/02/2026, 16:38:58 |

  ### Notes:

  - Export Date: 05 February 2026; Cited By: 4
- ## The impact of music, play, and pet therapies in managing pain and anxiety in paediatric patients in hospital: a rapid systematic review

  |  |  |
  | --- | --- |
  | Item Type | Journal Article |
  | Author | K. Goren |
  | Author | Y. Cen |
  | Author | V. Montemurri |
  | Author | D. Moodley |
  | Author | A. Sutton |
  | Author | A. Ahmed |
  | Author | L. Alphonsus |
  | Author | P. Denezis |
  | Author | C. Fleming |
  | Author | H. Guertin |
  | Author | K. Hyland |
  | Author | A. Kalim |
  | Author | H.H. Kim |
  | Author | S. Krause |
  | Author | A. Liang |
  | Author | E. MacLean |
  | Author | P. Neocleous |
  | Author | A. Patel |
  | Author | S. Pritchard |
  | Author | V. Purcell |
  | Author | M. Qaqish |
  | Author | S. Ryall |
  | Author | K. Shum |
  | Author | K. Suwary |
  | Author | A. Vucetic |
  | Author | J. Skinner |
  | Author | A. Woolsey |
  | Author | E. Marcotte |
  | Date | 2023 |
  | Archive | Scopus |
  | URL | https://www.scopus.com/inward/record.uri?eid=2-s2.0-85162134462&doi=10.1093%2Fpch%2Fpxad010&partnerID=40&md5=18d4de737ae095b68d8ff62a7bf5f18d |
  | Volume | 28 |
  | Pages | 218-224 |
  | Publication | Paediatrics and Child Health (Canada) |
  | DOI | 10.1093/pch/pxad010 |
  | Issue | 4 |
  | Date Added | 05/02/2026, 16:38:58 |
  | Modified | 05/02/2026, 16:38:58 |

  ### Notes:

  - Export Date: 05 February 2026; Cited By: 9
- ## Therapy dog welfare revisited: A review of the literature

  |  |  |
  | --- | --- |
  | Item Type | Journal Article |
  | Author | L.-M. Glenk |
  | Author | S. Foltin |
  | Date | 2021 |
  | Archive | Scopus |
  | URL | https://www.scopus.com/inward/record.uri?eid=2-s2.0-85117594178&doi=10.3390%2Fvetsci8100226&partnerID=40&md5=cbd70e496910e0c7abf467f03bf4da3f |
  | Volume | 8 |
  | Publication | Veterinary Sciences |
  | DOI | 10.3390/vetsci8100226 |
  | Issue | 10 |
  | Date Added | 05/02/2026, 16:38:58 |
  | Modified | 05/02/2026, 16:38:58 |

  ### Notes:

  - Export Date: 05 February 2026; Cited By: 46
- ## Trends in Animal Welfare Research in Animal-Assisted Interventions

  |  |  |
  | --- | --- |
  | Item Type | Book Section |
  | Author | L.-M. Glenk |
  | Date | 2024 |
  | Archive | Scopus |
  | URL | https://www.scopus.com/inward/record.uri?eid=2-s2.0-85208863325&doi=10.1016%2FB978-0-443-22346-4.15004-8&partnerID=40&md5=daae286c90c2ca8600621911f4518849 |
  | Pages | 235-248 |
  | Book Title | Handbook on Animal-Assisted Therapy |
  | DOI | 10.1016/B978-0-443-22346-4.15004-8 |
  | Date Added | 05/02/2026, 16:38:58 |
  | Modified | 05/02/2026, 16:38:58 |

  ### Notes:

  - Export Date: 05 February 2026; Cited By: 2
- ## Well-Being Indicators in Autistic Children and Therapy Dogs During a Group Intervention: A Pilot Study

  |  |  |
  | --- | --- |
  | Item Type | Journal Article |
  | Author | V.O. Giuliano |
  | Author | L. Sacchettino |
  | Author | A.S. Rusu |
  | Author | D. Ciccarelli |
  | Author | V. Gazzano |
  | Author | M. de Cesare |
  | Author | M. Visone |
  | Author | V. Mizzoni |
  | Author | F. Napolitano |
  | Author | D. d’Angelo |
  | Date | 2025 |
  | Archive | Scopus |
  | URL | https://www.scopus.com/inward/record.uri?eid=2-s2.0-105011633890&doi=10.3390%2Fani15142032&partnerID=40&md5=53a8049c05a8b7820429f8e113b8d275 |
  | Volume | 15 |
  | Publication | Animals |
  | DOI | 10.3390/ani15142032 |
  | Issue | 14 |
  | Date Added | 05/02/2026, 16:38:57 |
  | Modified | 05/02/2026, 16:38:57 |

  ### Notes:

  - Export Date: 05 February 2026; Cited By: 0
- ## A pilot study into the effects of PTSD-assistance dogs’ work on their salivary cortisol levels and their handlers’ Quality of life

  |  |  |
  | --- | --- |
  | Item Type | Journal Article |
  | Author | K. Gerwisch |
  | Author | K. Weissenbacher |
  | Author | M. Proyer |
  | Author | R. Palme |
  | Author | L. Huber |
  | Date | 2025 |
  | Archive | Scopus |
  | URL | https://www.scopus.com/inward/record.uri?eid=2-s2.0-85171598448&doi=10.1080%2F10888705.2023.2259795&partnerID=40&md5=d0349d743b17de006a4e865199c210b7 |
  | Volume | 28 |
  | Pages | 288-300 |
  | Publication | Journal of Applied Animal Welfare Science |
  | DOI | 10.1080/10888705.2023.2259795 |
  | Issue | 2 |
  | Date Added | 05/02/2026, 16:38:57 |
  | Modified | 05/02/2026, 16:38:57 |

  ### Notes:

  - Export Date: 05 February 2026; Cited By: 4
- ## Hippotherapy in the Treatment of CMD and Bruxism in Dentistry

  |  |  |
  | --- | --- |
  | Item Type | Journal Article |
  | Author | M.-A. Geibel |
  | Author | D. Kildal |
  | Author | A.M. Geibel |
  | Author | S. Ott |
  | Date | 2025 |
  | Archive | Scopus |
  | URL | https://www.scopus.com/inward/record.uri?eid=2-s2.0-105015450707&doi=10.3390%2Fani15172587&partnerID=40&md5=7440d1652df6cec68a11804acd7610a2 |
  | Volume | 15 |
  | Publication | Animals |
  | DOI | 10.3390/ani15172587 |
  | Issue | 17 |
  | Date Added | 05/02/2026, 16:38:57 |
  | Modified | 05/02/2026, 16:38:57 |

  ### Notes:

  - Export Date: 05 February 2026; Cited By: 0
- ## Equine-assisted therapeutic activities and their influence on the heart rate variability: A systematic review

  |  |  |
  | --- | --- |
  | Item Type | Journal Article |
  | Author | A. García |
  | Author | E. Guerrero-Barona |
  | Author | I. García-Peña |
  | Author | M. Rodríguez-Jiménez |
  | Author | J.M. Moreno-Manso |
  | Date | 2020 |
  | Archive | Scopus |
  | URL | https://www.scopus.com/inward/record.uri?eid=2-s2.0-85082862359&doi=10.1016%2Fj.ctcp.2020.101167&partnerID=40&md5=de29a6e83f1f141ffcfe907b72712d5a |
  | Volume | 39 |
  | Publication | Complementary Therapies in Clinical Practice |
  | DOI | 10.1016/j.ctcp.2020.101167 |
  | Date Added | 05/02/2026, 16:38:58 |
  | Modified | 05/02/2026, 16:38:58 |

  ### Notes:

  - Export Date: 05 February 2026; Cited By: 16
- ## Molecular Biomarkers of Adult Human and Dog Stress during Canine-Assisted Interventions: A Systematic Scoping Review

  |  |  |
  | --- | --- |
  | Item Type | Journal Article |
  | Author | J. Gandenberger |
  | Author | E. Flynn |
  | Author | E. Moratto |
  | Author | A. Wendt |
  | Author | K.N. Morris |
  | Date | 2022 |
  | Archive | Scopus |
  | URL | https://www.scopus.com/inward/record.uri?eid=2-s2.0-85126061105&doi=10.3390%2Fani12050651&partnerID=40&md5=b0774c0fe562ba855e06c2e05f2de004 |
  | Volume | 12 |
  | Publication | Animals |
  | DOI | 10.3390/ani12050651 |
  | Issue | 5 |
  | Date Added | 05/02/2026, 16:38:58 |
  | Modified | 05/02/2026, 16:38:58 |

  ### Notes:

  - Export Date: 05 February 2026; Cited By: 11
- ## How the presence of a dog and types of interaction affect physiological responses to experimental heat pain induction in healthy humans - a randomized controlled study

  |  |  |
  | --- | --- |
  | Item Type | Journal Article |
  | Author | L.H. Fuglsang-Damgaard |
  | Author | S.J. Lunde |
  | Author | J.W. Christensen |
  | Author | L. Vase Toft |
  | Author | P.B. Videbech |
  | Author | N.R. Gee |
  | Author | K. Thodberg |
  | Date | 2025 |
  | Archive | Scopus |
  | URL | https://www.scopus.com/inward/record.uri?eid=2-s2.0-105015627858&doi=10.1016%2Fj.physbeh.2025.115097&partnerID=40&md5=a5be5fdbff7ae55ae1a6614a611f65d7 |
  | Volume | 302 |
  | Publication | Physiology and Behavior |
  | DOI | 10.1016/j.physbeh.2025.115097 |
  | Date Added | 05/02/2026, 16:38:57 |
  | Modified | 05/02/2026, 16:38:57 |

  ### Notes:

  - Export Date: 05 February 2026; Cited By: 0
- ## The Animal-Human Bond: Health and Wellness

  |  |  |
  | --- | --- |
  | Item Type | Book Section |
  | Author | E. Friedmann |
  | Date | 2024 |
  | Archive | Scopus |
  | URL | https://www.scopus.com/inward/record.uri?eid=2-s2.0-85208895309&doi=10.1016%2FB978-0-443-22346-4.00015-9&partnerID=40&md5=771f39704f233b9ae7204b76b208e789 |
  | Pages | 61-81 |
  | Book Title | Handbook on Animal-Assisted Therapy |
  | DOI | 10.1016/B978-0-443-22346-4.00015-9 |
  | Date Added | 05/02/2026, 16:38:58 |
  | Modified | 05/02/2026, 16:38:58 |

  ### Notes:

  - Export Date: 05 February 2026; Cited By: 3
- ## The Effect of Therapy Dogs on Preoperative Anxiety

  |  |  |
  | --- | --- |
  | Item Type | Journal Article |
  | Author | P. Foerder |
  | Author | M. Royer |
  | Date | 2021 |
  | Archive | Scopus |
  | URL | https://www.scopus.com/inward/record.uri?eid=2-s2.0-85105350660&doi=10.1080%2F08927936.2021.1914440&partnerID=40&md5=ff81e09fdf1dd3c3496a846aa6f6947c |
  | Volume | 34 |
  | Pages | 659-670 |
  | Publication | Anthrozoos |
  | DOI | 10.1080/08927936.2021.1914440 |
  | Issue | 5 |
  | Date Added | 05/02/2026, 16:38:58 |
  | Modified | 05/02/2026, 16:38:58 |

  ### Notes:

  - Export Date: 05 February 2026; Cited By: 5
- ## Effects of animal-assisted therapy on hospitalized children and teenagers: A systematic review and meta-analysis

  |  |  |
  | --- | --- |
  | Item Type | Journal Article |
  | Author | Y. Feng |
  | Author | Y. Lin |
  | Author | N. Zhang |
  | Author | X. Jiang |
  | Author | L. Zhang |
  | Date | 2021 |
  | Archive | Scopus |
  | URL | https://www.scopus.com/inward/record.uri?eid=2-s2.0-85100774002&doi=10.1016%2Fj.pedn.2021.01.020&partnerID=40&md5=5048c1ddad6b643649b616ab7916992e |
  | Volume | 60 |
  | Pages | 11-23 |
  | Publication | Journal of Pediatric Nursing |
  | DOI | 10.1016/j.pedn.2021.01.020 |
  | Date Added | 05/02/2026, 16:38:58 |
  | Modified | 05/02/2026, 16:38:58 |

  ### Notes:

  - Export Date: 05 February 2026; Cited By: 52
- ## Employing Siamese Networks as Quantitative Biomarker for Assessing the Effect of Dolphin-Assisted Therapy on Pediatric Cerebral Palsy

  |  |  |
  | --- | --- |
  | Item Type | Journal Article |
  | Author | J.J. Escobar |
  | Author | O. Morales-Matamoros |
  | Author | E.Y. Aguilar-Del-Villar |
  | Author | H. Quintana Espinosa |
  | Author | L. Chanona-Hernández |
  | Date | 2024 |
  | Archive | Scopus |
  | URL | https://www.scopus.com/inward/record.uri?eid=2-s2.0-85202636933&doi=10.3390%2Fbrainsci14080778&partnerID=40&md5=bf4da213500e6a1eef1c20624fec9608 |
  | Volume | 14 |
  | Publication | Brain Sciences |
  | DOI | 10.3390/brainsci14080778 |
  | Issue | 8 |
  | Date Added | 05/02/2026, 16:38:58 |
  | Modified | 05/02/2026, 16:38:58 |

  ### Notes:

  - Export Date: 05 February 2026; Cited By: 2
- ## Byron’s bear and dwarf rabbits in the classroom: a review of animals against academic stress

  |  |  |
  | --- | --- |
  | Item Type | Journal Article |
  | Author | T.C. Erren |
  | Author | F. Glenewinkel |
  | Author | U. Wild |
  | Author | J. Wallraff |
  | Author | P. Lewis |
  | Date | 2025 |
  | Archive | Scopus |
  | URL | https://www.scopus.com/inward/record.uri?eid=2-s2.0-105026286642&doi=10.3389%2Ffvets.2025.1693505&partnerID=40&md5=1de1030d9f47bf297627a131b4b55595 |
  | Volume | 12 |
  | Publication | Frontiers in Veterinary Science |
  | DOI | 10.3389/fvets.2025.1693505 |
  | Date Added | 05/02/2026, 16:38:57 |
  | Modified | 05/02/2026, 16:38:57 |

  ### Notes:

  - Export Date: 05 February 2026; Cited By: 0
- ## Effects on Wellbeing of Exposure to Dog Videos Before a Stressor

  |  |  |
  | --- | --- |
  | Item Type | Journal Article |
  | Author | N. Ein |
  | Author | J. Gervasio |
  | Author | M.J. Reed |
  | Author | K. Vickers |
  | Date | 2023 |
  | Archive | Scopus |
  | URL | https://www.scopus.com/inward/record.uri?eid=2-s2.0-85144045934&doi=10.1080%2F08927936.2022.2149925&partnerID=40&md5=9394d0b0934d9a4ea854e6e86cc24eed |
  | Volume | 36 |
  | Pages | 349-367 |
  | Publication | Anthrozoos |
  | DOI | 10.1080/08927936.2022.2149925 |
  | Issue | 3 |
  | Date Added | 05/02/2026, 16:38:58 |
  | Modified | 05/02/2026, 16:38:58 |

  ### Notes:

  - Export Date: 05 February 2026; Cited By: 7
- ## The Effect of Dog Videos on Subjective and Physiological Responses to Stress

  |  |  |
  | --- | --- |
  | Item Type | Journal Article |
  | Author | N. Ein |
  | Author | M.J. Reed |
  | Author | K. Vickers |
  | Date | 2022 |
  | Archive | Scopus |
  | URL | https://www.scopus.com/inward/record.uri?eid=2-s2.0-85119422477&doi=10.1080%2F08927936.2021.1999606&partnerID=40&md5=f5e3a933212a59bca54253667b03fec3 |
  | Volume | 35 |
  | Pages | 463-482 |
  | Publication | Anthrozoos |
  | DOI | 10.1080/08927936.2021.1999606 |
  | Issue | 3 |
  | Date Added | 05/02/2026, 16:38:58 |
  | Modified | 05/02/2026, 16:38:58 |

  ### Notes:

  - Export Date: 05 February 2026; Cited By: 12
- ## Animal-Assisted Stress Management for Veterinary Staff

  |  |  |
  | --- | --- |
  | Item Type | Journal Article |
  | Author | Y.M. Eaton-Stull |
  | Author | C. Streidl |
  | Author | B.G. Jaffe |
  | Author | S. Kuehn |
  | Author | A. Kaufman |
  | Date | 2024 |
  | Archive | Scopus |
  | URL | https://www.scopus.com/inward/record.uri?eid=2-s2.0-85207665050&doi=10.1093%2Fhsw%2Fhlae025&partnerID=40&md5=5f2d5d2b42a9dd0f5d804d34338eca92 |
  | Volume | 49 |
  | Pages | 219-226 |
  | Publication | Health and Social Work |
  | DOI | 10.1093/hsw/hlae025 |
  | Issue | 4 |
  | Date Added | 05/02/2026, 16:38:57 |
  | Modified | 05/02/2026, 16:38:57 |

  ### Notes:

  - Export Date: 05 February 2026; Cited By: 1
- ## BLOOD CORTISOL INDICATORS AND WORKLOAD IN C ANISTHERAPY DOGS

  |  |  |
  | --- | --- |
  | Item Type | Journal Article |
  | Author | A. Dovidė |
  | Author | J. Kučinskiene |
  | Author | V. Ribikauskas |
  | Date | 2024 |
  | Archive | Scopus |
  | URL | https://www.scopus.com/inward/record.uri?eid=2-s2.0-85212945455&partnerID=40&md5=313dc4571621d934b89140db2775d784 |
  | Volume | 82(1) |
  | Pages | 168-168 |
  | Publication | Veterinarija ir Zootechnika |
  | Date Added | 05/02/2026, 16:38:58 |
  | Modified | 05/02/2026, 16:38:58 |

  ### Notes:

  - Export Date: 05 February 2026; Cited By: 0
- ## Cortisol levels of shelter dogs in animal assisted interventions in a prison: An exploratory study

  |  |  |
  | --- | --- |
  | Item Type | Journal Article |
  | Author | D. d’Angelo |
  | Author | S. d'Ingeo |
  | Author | F. Ciani |
  | Author | M. Visone |
  | Author | L. Sacchettino |
  | Author | L. Avallone |
  | Author | A. Quaranta |
  | Date | 2021 |
  | Archive | Scopus |
  | URL | https://www.scopus.com/inward/record.uri?eid=2-s2.0-85099911592&doi=10.3390%2Fani11020345&partnerID=40&md5=cca4f4075ffd2b2e5dc762c64a06869a |
  | Volume | 11 |
  | Pages | 1-11 |
  | Publication | Animals |
  | DOI | 10.3390/ani11020345 |
  | Issue | 2 |
  | Date Added | 05/02/2026, 16:38:58 |
  | Modified | 05/02/2026, 16:38:58 |

  ### Notes:

  - Export Date: 05 February 2026; Cited By: 28
- ## Improving the Emotional Distress and the Experience of Hospitalization in Children and Adolescent Patients Through Animal Assisted Interventions: A Systematic Review

  |  |  |
  | --- | --- |
  | Item Type | Journal Article |
  | Author | C. Correale |
  | Author | M. Borgi |
  | Author | B. Collacchi |
  | Author | C. Falamesca |
  | Author | S. Gentile |
  | Author | F. Vigevano |
  | Author | S. Cappelletti |
  | Author | F. Cirulli |
  | Date | 2022 |
  | Archive | Scopus |
  | URL | https://www.scopus.com/inward/record.uri?eid=2-s2.0-85127437752&doi=10.3389%2Ffpsyg.2022.840107&partnerID=40&md5=1e735d773cff0684084d0bedba5b3643 |
  | Volume | 13 |
  | Publication | Frontiers in Psychology |
  | DOI | 10.3389/fpsyg.2022.840107 |
  | Date Added | 05/02/2026, 16:38:58 |
  | Modified | 05/02/2026, 16:38:58 |

  ### Notes:

  - Export Date: 05 February 2026; Cited By: 24
- ## Pawsitive Care: Canine-Assisted Intervention for Anxiety in ICU Patients and Family Members: A Single-Center, Single-Arm Study

  |  |  |
  | --- | --- |
  | Item Type | Journal Article |
  | Author | K. Cook |
  | Author | C. Robertson |
  | Author | K. Gudivada |
  | Author | I. Mitchell |
  | Author | M. Nourse |
  | Author | M.M. Hosey |
  | Author | C. Paterson |
  | Author | S. Rai |
  | Date | 2025 |
  | Archive | Scopus |
  | URL | https://www.scopus.com/inward/record.uri?eid=2-s2.0-105004219513&doi=10.1097%2FCCE.0000000000001258&partnerID=40&md5=6592b4daa9d826ac7db37d257fa8350b |
  | Volume | 7 |
  | Pages | e1258 |
  | Publication | Critical Care Explorations |
  | DOI | 10.1097/CCE.0000000000001258 |
  | Issue | 5 |
  | Date Added | 05/02/2026, 16:38:57 |
  | Modified | 05/02/2026, 16:38:57 |

  ### Notes:

  - Export Date: 05 February 2026; Cited By: 1
- ## The Experience of Animal Assisted Therapy on Patients in an Acute Care Setting

  |  |  |
  | --- | --- |
  | Item Type | Journal Article |
  | Author | A.B. Coakley |
  | Author | C.D. Annese |
  | Author | J.H. Empoliti |
  | Author | J.M. Flanagan |
  | Date | 2021 |
  | Archive | Scopus |
  | URL | https://www.scopus.com/inward/record.uri?eid=2-s2.0-85096824305&doi=10.1177%2F1054773820977198&partnerID=40&md5=291161efa383a2cecfd3f762066246f1 |
  | Volume | 30 |
  | Pages | 401-405 |
  | Publication | Clinical Nursing Research |
  | DOI | 10.1177/1054773820977198 |
  | Issue | 4 |
  | Date Added | 05/02/2026, 16:38:58 |
  | Modified | 05/02/2026, 16:38:58 |

  ### Notes:

  - Export Date: 05 February 2026; Cited By: 19
- ## The Impact of a 20-Minute Animal-Assisted Activity Session on the Physiological and Emotional States in Patients With Fibromyalgia

  |  |  |
  | --- | --- |
  | Item Type | Journal Article |
  | Author | S. Clark |
  | Author | F. Martin |
  | Author | R.T.S. McGowan |
  | Author | J. Smidt |
  | Author | R. Anderson |
  | Author | L. Wang |
  | Author | T. Turpin |
  | Author | N. Langenfeld-McCoy |
  | Author | B. Bauer |
  | Author | A.B. Mohabbat |
  | Date | 2020 |
  | Archive | Scopus |
  | URL | https://www.scopus.com/inward/record.uri?eid=2-s2.0-85084505049&doi=10.1016%2Fj.mayocp.2020.04.037&partnerID=40&md5=b1998401f4932f475e87b3d3c5df44bc |
  | Volume | 95 |
  | Pages | 2442-2461 |
  | Publication | Mayo Clinic Proceedings |
  | DOI | 10.1016/j.mayocp.2020.04.037 |
  | Issue | 11 |
  | Date Added | 05/02/2026, 16:38:58 |
  | Modified | 05/02/2026, 16:38:58 |

  ### Notes:

  - Export Date: 05 February 2026; Cited By: 25
- ## Physiological state of therapy dogs during animal-assisted activities in an outpatient setting

  |  |  |
  | --- | --- |
  | Item Type | Journal Article |
  | Author | S. Clark |
  | Author | F. Martin |
  | Author | R.T.S. McGowan |
  | Author | J. Smidt |
  | Author | R. Anderson |
  | Author | L. Wang |
  | Author | T. Turpin |
  | Author | N. Langenfeld-McCoy |
  | Author | B. Bauer |
  | Author | A.B. Mohabbat |
  | Date | 2020 |
  | Archive | Scopus |
  | URL | https://www.scopus.com/inward/record.uri?eid=2-s2.0-85084463388&doi=10.3390%2Fani10050819&partnerID=40&md5=2c1ad01ef6c9aae586fef3d453a60c0b |
  | Volume | 10 |
  | Publication | Animals |
  | DOI | 10.3390/ani10050819 |
  | Issue | 5 |
  | Date Added | 05/02/2026, 16:38:58 |
  | Modified | 05/02/2026, 16:38:58 |

  ### Notes:

  - Export Date: 05 February 2026; Cited By: 38
- ## Unveiling directional physiological coupling in human-horse interactions

  |  |  |
  | --- | --- |
  | Item Type | Journal Article |
  | Author | A.L. Callara |
  | Author | C. Scopa |
  | Author | L. Contalbrigo |
  | Author | A. Lanatà |
  | Author | E.P. Scilingo |
  | Author | P. Baragli |
  | Author | A. Greco |
  | Date | 2024 |
  | Archive | Scopus |
  | URL | https://www.scopus.com/inward/record.uri?eid=2-s2.0-85207814732&doi=10.1016%2Fj.isci.2024.110857&partnerID=40&md5=da46550d50bb68ce8284b064f33f85c6 |
  | Volume | 27 |
  | Publication | iScience |
  | DOI | 10.1016/j.isci.2024.110857 |
  | Issue | 9 |
  | Date Added | 05/02/2026, 16:38:57 |
  | Modified | 05/02/2026, 16:38:57 |

  ### Notes:

  - Export Date: 05 February 2026; Cited By: 3
- ## The Human-Animal Bond

  |  |  |
  | --- | --- |
  | Item Type | Book Section |
  | Author | S.G.J. Bybee |
  | Date | 2021 |
  | Archive | Scopus |
  | URL | https://www.scopus.com/inward/record.uri?eid=2-s2.0-85131998392&doi=10.4324%2F9781351069120-21&partnerID=40&md5=f546346285308546d255949b4b10d4c3 |
  | Pages | 133-135 |
  | Book Title | New Techniques of Grief Therapy: Bereavement and Beyond |
  | DOI | 10.4324/9781351069120-21 |
  | Date Added | 05/02/2026, 16:38:58 |
  | Modified | 05/02/2026, 16:38:58 |

  ### Notes:

  - Export Date: 05 February 2026; Cited By: 0
- ## Animal-Assisted Activity in Critically Ill Older Adults: A Randomized Pilot and Feasibility Trial

  |  |  |
  | --- | --- |
  | Item Type | Journal Article |
  | Author | S. Branson |
  | Author | L. Boss |
  | Author | S. Hamlin |
  | Author | N.S. Padhye |
  | Date | 2020 |
  | Archive | Scopus |
  | URL | https://www.scopus.com/inward/record.uri?eid=2-s2.0-85083773814&doi=10.1177%2F1099800420920719&partnerID=40&md5=d518b873e251a0c28983714cc73240c4 |
  | Volume | 22 |
  | Pages | 412-417 |
  | Publication | Biological Research for Nursing |
  | DOI | 10.1177/1099800420920719 |
  | Issue | 3 |
  | Date Added | 05/02/2026, 16:38:58 |
  | Modified | 05/02/2026, 16:38:58 |

  ### Notes:

  - Export Date: 05 February 2026; Cited By: 17
- ## Animal-assisted intervention for geriatric well-being: A comprehensive review

  |  |  |
  | --- | --- |
  | Item Type | Journal Article |
  | Author | L.K. Bernhardt |
  | Author | A. Vashe |
  | Author | G.V. Bernhardt |
  | Author | J. Pinto |
  | Date | 2024 |
  | Archive | Scopus |
  | URL | https://www.scopus.com/inward/record.uri?eid=2-s2.0-85206279006&doi=10.7417%2FCT.2024.5126&partnerID=40&md5=b36676c0127b7ab4b275c2cdc5c874b7 |
  | Volume | 175 |
  | Pages | 362-369 |
  | Publication | Clinica Terapeutica |
  | DOI | 10.7417/CT.2024.5126 |
  | Issue | 5 |
  | Date Added | 05/02/2026, 16:38:57 |
  | Modified | 05/02/2026, 16:38:57 |

  ### Notes:

  - Export Date: 05 February 2026; Cited By: 1
- ## Oxytocin levels and self-reported anxiety during interactions between humans and cows

  |  |  |
  | --- | --- |
  | Item Type | Journal Article |
  | Author | B. Berget |
  | Author | J. Vas |
  | Author | G. Pedersen |
  | Author | K. Uvnäs-Moberg |
  | Author | R.C. Newberry |
  | Date | 2023 |
  | Archive | Scopus |
  | URL | https://www.scopus.com/inward/record.uri?eid=2-s2.0-85172998589&doi=10.3389%2Ffpsyg.2023.1252463&partnerID=40&md5=f9acaaa9329abffbf137c7fa1c7bd085 |
  | Volume | 14 |
  | Publication | Frontiers in Psychology |
  | DOI | 10.3389/fpsyg.2023.1252463 |
  | Date Added | 05/02/2026, 16:38:58 |
  | Modified | 05/02/2026, 16:38:58 |

  ### Notes:

  - Export Date: 05 February 2026; Cited By: 3
- ## Animal Farm in healthcare: definitions, policies, laws and implications for health professionals

  |  |  |
  | --- | --- |
  | Item Type | Journal Article |
  | Author | E. Ben-Sefer |
  | Author | L. Shields |
  | Date | 2021 |
  | Archive | Scopus |
  | URL | https://www.scopus.com/inward/record.uri?eid=2-s2.0-85102125671&doi=10.1177%2F0141076821996003&partnerID=40&md5=e4329db1457e3b4f9431193fbae17074 |
  | Volume | 114 |
  | Pages | 171-177 |
  | Publication | Journal of the Royal Society of Medicine |
  | DOI | 10.1177/0141076821996003 |
  | Issue | 4 |
  | Date Added | 05/02/2026, 16:38:58 |
  | Modified | 05/02/2026, 16:38:58 |

  ### Notes:

  - Export Date: 05 February 2026; Cited By: 2
- ## Virtual Reality Zoo Therapy for Alzheimer’s Disease Using Real-Time Gesture Recognition

  |  |  |
  | --- | --- |
  | Item Type | Book Section |
  | Author | H. Ben Abdessalem |
  | Author | Y. Ai |
  | Author | K.S. Marulasidda Swamy |
  | Author | C. Frasson |
  | Date | 2021 |
  | Archive | Scopus |
  | URL | https://www.scopus.com/inward/record.uri?eid=2-s2.0-85122477216&doi=10.1007%2F978-3-030-78775-2\_12&partnerID=40&md5=7a588cba3468441383acc3aac4235988 |
  | Volume | 1338 |
  | Pages | 97-105 |
  | Book Title | Advances in Experimental Medicine and Biology |
  | DOI | 10.1007/978-3-030-78775-2\_12 |
  | Date Added | 05/02/2026, 16:38:58 |
  | Modified | 05/02/2026, 16:38:58 |

  ### Notes:

  - Export Date: 05 February 2026; Cited By: 10
- ## Equine-assisted therapy as an adjunctive method in the treatment of recurrent depressive disorder – a case report

  |  |  |
  | --- | --- |
  | Item Type | Journal Article |
  | Author | K. Bartniak |
  | Author | K. Zaborska |
  | Author | M.E. Talarowska |
  | Date | 2025 |
  | Short Title | Zastosowanie terapii z udziałem koni jako metody wspomagającej w leczeniu zaburzeń depresyjnych nawracających – opis przypadku |
  | Archive | Scopus |
  | URL | https://www.scopus.com/inward/record.uri?eid=2-s2.0-105026583090&doi=10.15557%2FPiPK.2025.0023&partnerID=40&md5=68982cdcc921df2f6723fe9c0f4750d0 |
  | Volume | 25 |
  | Pages | 182-189 |
  | Publication | Psychiatria i Psychologia Kliniczna |
  | DOI | 10.15557/PiPK.2025.0023 |
  | Issue | 2 |
  | Date Added | 05/02/2026, 16:38:57 |
  | Modified | 05/02/2026, 16:38:57 |

  ### Notes:

  - Export Date: 05 February 2026; Cited By: 0
- ## Animal-Assisted Interventions on a College Campus to Improve Wellness: Adventures With the Northern Michigan University Wildpups

  |  |  |
  | --- | --- |
  | Item Type | Journal Article |
  | Author | M. Andriacchi |
  | Author | C. Hopper |
  | Author | A. Stein |
  | Author | R. Nye |
  | Author | K. Taylor |
  | Date | 2023 |
  | Archive | Scopus |
  | URL | https://www.scopus.com/inward/record.uri?eid=2-s2.0-85176775783&doi=10.3928%2F01484834-20230906-05&partnerID=40&md5=e5c6c4ceeb12115b7b5c0383dc961bb1 |
  | Volume | 62 |
  | Pages | 631-637 |
  | Publication | Journal of Nursing Education |
  | DOI | 10.3928/01484834-20230906-05 |
  | Issue | 11 |
  | Date Added | 05/02/2026, 16:38:58 |
  | Modified | 05/02/2026, 16:38:58 |

  ### Notes:

  - Export Date: 05 February 2026; Cited By: 0
- ## Machine learning-based affect detection within the context of human-horse interaction

  |  |  |
  | --- | --- |
  | Item Type | Book Section |
  | Author | T. Althobaiti |
  | Author | S. Katsigiannis |
  | Author | D. West |
  | Author | H. Rabah |
  | Author | N. Ramzan |
  | Date | 2021 |
  | Archive | Scopus |
  | URL | https://www.scopus.com/inward/record.uri?eid=2-s2.0-85114583650&doi=10.1049%2FPBPC034E\_ch3&partnerID=40&md5=15e59043158daf0a97816fe153d27949 |
  | Pages | 45-61 |
  | Book Title | AI for Emerging Verticals: Human-robot computing, sensing and networking |
  | DOI | 10.1049/PBPC034E\_ch3 |
  | Date Added | 05/02/2026, 16:38:58 |
  | Modified | 05/02/2026, 16:38:58 |

  ### Notes:

  - Export Date: 05 February 2026; Cited By: 0
- ## Noncontact Electrophysiology Monitoring Systems for Assessment of Canine-Human Interactions

  |  |  |
  | --- | --- |
  | Item Type | Conference Paper |
  | Author | P. Ahmmed |
  | Author | T. Holder |
  | Author | M. Foster |
  | Author | I.D. Castro |
  | Author | A. Patel |
  | Author | T. Torfs |
  | Author | A. Bozkurt |
  | Date | 2021 |
  | Archive | Scopus |
  | URL | https://www.scopus.com/inward/record.uri?eid=2-s2.0-85123608863&doi=10.1109%2FSENSORS47087.2021.9639748&partnerID=40&md5=d3dddf466c5c8536fdb84c14b0ee2773 |
  | Volume | 2021-October |
  | Conference Name | Proceedings of IEEE Sensors |
  | DOI | 10.1109/SENSORS47087.2021.9639748 |
  | Date Added | 05/02/2026, 16:38:58 |
  | Modified | 05/02/2026, 16:38:58 |

  ### Notes:

  - Export Date: 05 February 2026; Cited By: 3
- ## Animal-assisted therapy for cardiac conditions

  |  |  |
  | --- | --- |
  | Item Type | Book Section |
  | Author | S.V. Abate |
  | Date | 2022 |
  | Archive | Scopus |
  | URL | https://www.scopus.com/inward/record.uri?eid=2-s2.0-85177464374&doi=10.1016%2FB978-0-323-98815-5.00010-0&partnerID=40&md5=14bac3a506679afbb1257b8c30e6f366 |
  | Pages | 147-164 |
  | Book Title | Animal Assisted Therapy Use Application by Condition |
  | DOI | 10.1016/B978-0-323-98815-5.00010-0 |
  | Date Added | 05/02/2026, 16:38:58 |
  | Modified | 05/02/2026, 16:38:58 |

  ### Notes:

  - Export Date: 05 February 2026; Cited By: 1
