# Supplementary material for: Evidence of Physiological Comodulation During Human–Animal Interaction: A Systematic Review
Source: Ann N Y Acad Sci. 2026 Jun 4;1560(1):e70299. doi: 10.1111/nyas.70299 (PMC13238372; doi:10.1111/nyas.70299)
Supplement: Supplementary file 2 — Supplementary Materials: Supp2‐Zotero‐Collection.zip [file NYAS-1560-0-s002.zip › Supp2_Zotero_Collection/new searches/text screened.htm]

Zotero Report


- ## Child horse harmony in motion: a preliminary study to explore heart rate synchronization in equine assisted therapy for neurotypical and ADHD children

  |  |  |
  | --- | --- |
  | Item Type | Journal Article |
  | Author | A. Helmer |
  | Author | A. Hacohen |
  | Author | O. Bart |
  | Abstract | Equine-Assisted Services (EAS) encompass a range of therapeutic interventions utilizing equine interactions to achieve therapeutic goals. This study explores heart rate synchronization between horses and riders during mounted and unmounted interactions, focusing on its potential implications for emotional regulation. A total of 25 participants aged 6-12 took part in the study, which included two groups: novice riders diagnosed with Attention Deficit Hyperactivity Disorder (ADHD) (n = 15) and experienced neurotypical riders (n = 10). Heart rate measurements were obtained using Polar® Equine and Verity Sense Optical Heart Rate Sensors. Results indicate mutual heart rate synchronization between horses and riders, suggesting a potential mechanism for emotional regulation. The neurotypical group showed high levels of synchronization, suggesting that rider experience influences the physiological connection between horse and rider., while notably, children with ADHD demonstrated above-average synchronization by their fourth to sixth EAS session. These findings underscore the significance of EAS in promoting physiological and emotional well-being, particularly for individuals with ADHD. This study contributes to the understanding of the physiological mechanisms underlying therapeutic effect of EAS interventions and highlights their potential in clinical practice. Further research is needed to examine the mechanisms, stability, and therapeutic significance of horse-human physiological synchronization over time, including how synchronization patterns evolve with rider experience and influence therapeutic outcomes in children with ADHD. |
  | Date | 2025 |
  | Language | English |
  | Archive | Medline |
  | URL | https://www.embase.com/search/results?subaction=viewrecord&id=L649322714&from=export |
  | Volume | 15 |
  | Pages | 45312 |
  | Publication | Scientific reports |
  | DOI | 10.1038/s41598-025-29330-6 |
  | Issue | 1 |
  | Journal Abbr | Sci Rep |
  | ISSN | 2045-2322 |
  | Date Added | 05/02/2026, 17:58:23 |
  | Modified | 05/02/2026, 17:58:23 |

  ### Tags:

  - horse
  - physiology
  - heart rate
  - therapy
  - emotion
  - child
  - female
  - male
  - human
  - hippotherapy
  - animal
  - psychology
  - attention deficit hyperactivity disorder
  - procedures
  - pathophysiology

  ### Attachments

  - Full Text (HTML)
  - PDF
- ## Well-Being Indicators in Autistic Children and Therapy Dogs During a Group Intervention: A Pilot Study

  |  |  |
  | --- | --- |
  | Item Type | Journal Article |
  | Author | Viviana Orsola Giuliano |
  | Author | Luigi Sacchettino |
  | Author | Alina Simona Rusu |
  | Author | Davide Ciccarelli |
  | Author | Valentina Gazzano |
  | Author | Martina De Cesare |
  | Author | Michele Visone |
  | Author | Vincenzo Mizzoni |
  | Author | Francesco Napolitano |
  | Author | Danila d’Angelo |
  | Abstract | Animal-assisted services (AAS) have been shown in multiple studies to improve a range of human psychological and physical health benefits. The aim of this pilot study is to investigate simultaneously two psycho-physiological indicators of the valence of interactions in the context of dog-assisted activities in children diagnosed with autism spectrum disorder. Ten children and four dogs experienced in AAS were involved, lasting 90 days, in weekly one-hour sessions. Before and after each session, saliva was taken in both dogs and children for determination of salivary oxytocin and cortisol levels. In addition, at the end of the program, a questionnaire was administered to both parents and dog handlers to assess the impact of AAS in children and dogs. Our results revealed no statistically significant change in cortisol and oxytocin levels in dogs enrolled throughout the sessions, while an increasing trend was noted for salivary oxytocin in 50% of the dogs and for salivary cortisol in all dogs at the end of the AAS, when compared to the pre-AAS. Salivary cortisol measurement in children with an autistic neurotype highlighted a statistically significant increase at the end of the AAS when compared to the pre-AAS, but this was not observed for oxytocin level evaluations. Regarding the perception of the children’s parents about the effects of the program, our data reported an improvement in sociability of the children in 100 percent of the cases. Furthermore, dog handlers reported an absence of signs of stress in their dogs during the sessions. Although the perceived effectiveness and quality of AAS has been demonstrated in the literature, the need to carefully select the dogs involved, considering their skills and needs, is critical to ensure their well-being in various therapeutic settings. |
  | Date | 2025-07-10 |
  | Language | en |
  | Short Title | Well-Being Indicators in Autistic Children and Therapy Dogs During a Group Intervention |
  | Library Catalogue | DOI.org (Crossref) |
  | URL | https://www.mdpi.com/2076-2615/15/14/2032 |
  | Accessed | 05/02/2026, 16:28:36 |
  | Volume | 15 |
  | Pages | 2032 |
  | Publication | Animals |
  | DOI | 10.3390/ani15142032 |
  | Issue | 14 |
  | Journal Abbr | Animals |
  | ISSN | 2076-2615 |
  | Date Added | 05/02/2026, 16:28:36 |
  | Modified | 05/02/2026, 16:28:36 |

  ### Notes:

  - From PubMed

  ### Attachments

  - PDF
