# Supplementary material for: Evidence of Physiological Comodulation During Human–Animal Interaction: A Systematic Review
Source: Ann N Y Acad Sci. 2026 Jun 4;1560(1):e70299. doi: 10.1111/nyas.70299 (PMC13238372; doi:10.1111/nyas.70299)
Supplement: Supplementary file 2 — Supplementary Materials: Supp2‐Zotero‐Collection.zip [file NYAS-1560-0-s002.zip › Supp2_Zotero_Collection/text screened/All text screened.htm]

Zotero Report


- ## Equine-assisted therapy and its impact on cortisol levels of children and horses: a pilot study and meta-analysis

  |  |  |
  | --- | --- |
  | Item Type | Journal Article |
  | Author | Jan Yorke |
  | Author | William Nugent |
  | Author | Elizabeth Strand |
  | Author | Rebecca Bolen |
  | Author | John New |
  | Author | Cindy Davis |
  | Date | 07/2013 |
  | Language | en |
  | Short Title | Equine-assisted therapy and its impact on cortisol levels of children and horses |
  | Library Catalogue | Crossref |
  | URL | https://www.tandfonline.com/doi/full/10.1080/03004430.2012.693486 |
  | Accessed | 09/07/2025, 12:07:46 |
  | Volume | 183 |
  | Publisher | Informa UK Limited |
  | Pages | 874-894 |
  | Publication | Early Child Development and Care |
  | DOI | 10.1080/03004430.2012.693486 |
  | Issue | 7 |
  | ISSN | 0300-4430, 1476-8275 |
  | Date Added | 09/07/2025, 12:07:46 |
  | Modified | 09/07/2025, 12:07:46 |

  ### Attachments

  - PDF
- ## The Effects of Human–Horse Interactions on Oxytocin and Cortisol Levels in Humans and Horses

  |  |  |
  | --- | --- |
  | Item Type | Journal Article |
  | Author | Minjung Yoon |
  | Author | Youngwook Jung |
  | Date | 2025-03-21 |
  | URL | https://consensus.app/papers/the-effects-of-human%E2%80%93horse-interactions-on-oxytocin-and-yoon-jung/0c72618bc22a5c9689bf2e621a450ac2/ |
  | Volume | 15 |
  | Publication | Animals : an Open Access Journal from MDPI |
  | DOI | 10.3390/ani15070905 |
  | Journal Abbr | Animals : an Open Access Journal from MDPI |
  | Date Added | 20/06/2025, 10:21:07 |
  | Modified | 20/06/2025, 10:21:07 |

  ### Attachments

  - PDF
- ## Emotional contagion: Dogs and humans show a similar physiological response to human infant crying

  |  |  |
  | --- | --- |
  | Item Type | Journal Article |
  | Author | Min Hooi Yong |
  | Author | Ted Ruffman |
  | Date | 10/2014 |
  | Language | en |
  | Short Title | Emotional contagion |
  | Library Catalogue | Crossref |
  | URL | https://linkinghub.elsevier.com/retrieve/pii/S0376635714002472 |
  | Accessed | 09/07/2025, 11:30:51 |
  | Rights | https://www.elsevier.com/tdm/userlicense/1.0/ |
  | Volume | 108 |
  | Publisher | Elsevier BV |
  | Pages | 155-165 |
  | Publication | Behavioural Processes |
  | DOI | 10.1016/j.beproc.2014.10.006 |
  | ISSN | 0376-6357 |
  | Date Added | 09/07/2025, 11:30:51 |
  | Modified | 09/07/2025, 11:30:51 |

  ### Attachments

  - PDF
- ## Salivary Cortisol Interactions in Search and Rescue Dogs and Their Handlers

  |  |  |
  | --- | --- |
  | Item Type | Journal Article |
  | Author | Justyna Wojtaś |
  | Author | Mirosław Karpiński |
  | Author | Piotr Czyżowski |
  | Abstract | Search and rescue dogs are an important link in the search for missing persons. The aim of the study was to assess exam stress in search and rescue dogs and their handlers. The study included 41 rescue teams taking exams of field and rubble specialties. The level of cortisol, which is the main glucocorticosteroid modulating stress reactions in humans and dogs, was analyzed. The biological material used to assess the hormone concentration was saliva collected in a non-invasive way. In total, 164 test samples were collected: two from the dog and two from the handler before and immediately after the exam. Rescue exams were shown to significantly increase salivary cortisol in both dogs and their handlers. Strong interactions between cortisol levels in human–dog teams were also found with a more pronounced effect in female dog–female handler dyads. |
  | Date | 2020-04-01 |
  | Language | en |
  | Library Catalogue | DOI.org (Crossref) |
  | URL | https://www.mdpi.com/2076-2615/10/4/595 |
  | Accessed | 01/07/2025, 15:48:02 |
  | Rights | https://creativecommons.org/licenses/by/4.0/ |
  | Volume | 10 |
  | Pages | 595 |
  | Publication | Animals |
  | DOI | 10.3390/ani10040595 |
  | Issue | 4 |
  | Journal Abbr | Animals |
  | ISSN | 2076-2615 |
  | Date Added | 01/07/2025, 15:48:02 |
  | Modified | 01/07/2025, 15:48:02 |

  ### Attachments

  - Full Text
- ## Are Hair Cortisol Levels of Humans, Cats, and Dogs from the Same Household Correlated?

  |  |  |
  | --- | --- |
  | Item Type | Journal Article |
  | Author | Justyna Wojtaś |
  | Author | Aleksandra Garbiec |
  | Author | Mirosław Karpiński |
  | Author | Patrycja Skowronek |
  | Author | Aneta Strachecka |
  | Abstract | Human–animal interactions and the emotional relationship of the owner with the pet are the subjects of many scientific studies and the constant interest of not only scientists but also pet owners. The aim of this study was to determine and compare the hair cortisol levels of dogs, cats, and their owners living in the same household. The owners were asked to complete a questionnaire concerning the frequency of their interactions with pets and emotional relationship with each of their cats and each of their dogs. The study involved 25 women who owned at least one dog and at least one cat. In total, 45 dogs and 55 cats from 25 households participated in the study. The average level of hair cortisol of the owners was 4.62 ng/mL, of the dogs 0.26 ng/mL, and in the hair of cats 0.45 ng/mL. There was no significant correlation between the hair cortisol level of the owner and dog or the owner and the cat and between dogs and cats living together. A significant positive correlation was observed between the hair cortisol level in the owner and the pet, for dogs in which the owner performs grooming treatments once a week and for cats which are never kissed. Although our study did not find many significant correlations, studies using other stress markers might have yielded different results. |
  | Date | 2022-06-06 |
  | Language | en |
  | Library Catalogue | Crossref |
  | URL | https://www.mdpi.com/2076-2615/12/11/1472 |
  | Accessed | 10/07/2025, 09:29:49 |
  | Rights | https://creativecommons.org/licenses/by/4.0/ |
  | Volume | 12 |
  | Publisher | MDPI AG |
  | Pages | 1472 |
  | Publication | Animals |
  | DOI | 10.3390/ani12111472 |
  | Issue | 11 |
  | ISSN | 2076-2615 |
  | Date Added | 10/07/2025, 09:29:49 |
  | Modified | 10/07/2025, 09:29:49 |

  ### Attachments

  - Full Text
- ## The relationship of early life adversity and physiological synchrony within the therapeutic triad in horse-assisted therapy

  |  |  |
  | --- | --- |
  | Item Type | Journal Article |
  | Author | Stella Wienhold |
  | Author | Larissa Bär |
  | Author | Zoe Ringleb |
  | Author | Victoria Zirpel |
  | Author | Annette Gomolla |
  | Author | Bernadette F. Denk |
  | Author | Nina Volkmer |
  | Author | Raphaela J. Gaertner |
  | Author | Elea S. C. Klink |
  | Author | Jens C. Pruessner |
  | Abstract | Abstract In any therapeutic setting, the outcome depends in part on the therapeutic alliance, characterized by mutual understanding, empathy and trust among the participants. This also manifests through physiological synchronization (PS) processes involving breathing, heart and brain. This study examined the dynamics of heart rate variability (HRV) synchronization patterns during horse-assisted therapy. We explored the correlations between the therapist’s horse preference, levels of early life adversity (ELA), and PS relationships within and across dyads of participants, therapists, and therapy horses. Our sample of 42 female participants engaged in standardized horse-assisted therapy sessions facilitated by three riding therapists and four therapy horses. PS was operationalized through cross-wavelet power analyses across the different dyads. The results showed, that stronger HRV synchronization between the therapist and horse was associated with stronger HRV synchronization between the horse and participant, as well as stronger HRV synchronization between the therapist and participant. We found a correlation between ELA and HRV synchronization between participants and therapists, with individuals experiencing higher levels of ELA showing lower synchronization. However, this effect of ELA was not observed for HRV synchronization between participants and horses. Furthermore, we found a negative correlation between the riding therapist’s preference for a particular therapy horse and the HRV synchronization between the therapist and that horse. These findings contribute to a better understanding of the correlational dynamics in horse-human interactions and may have potential implications for optimizing therapeutic interventions in clinical settings. |
  | Date | 2025-05-27 |
  | Language | en |
  | Library Catalogue | DOI.org (Crossref) |
  | URL | https://link.springer.com/10.1007/s00702-025-02947-7 |
  | Accessed | 01/07/2025, 17:35:46 |
  | Publication | Journal of Neural Transmission |
  | DOI | 10.1007/s00702-025-02947-7 |
  | Journal Abbr | J Neural Transm |
  | ISSN | 0300-9564, 1435-1463 |
  | Date Added | 01/07/2025, 17:35:46 |
  | Modified | 01/07/2025, 17:35:46 |

  ### Attachments

  - PDF
- ## Cortisol release, heart rate and heart rate variability in the horse and its rider: Different responses to training and performance

  |  |  |
  | --- | --- |
  | Item Type | Journal Article |
  | Author | Mareike Von Lewinski |
  | Author | Sophie Biau |
  | Author | Regina Erber |
  | Author | Natascha Ille |
  | Author | Jörg Aurich |
  | Author | Jean-Michel Faure |
  | Author | Erich Möstl |
  | Author | Christine Aurich |
  | Date | 08/2013 |
  | Language | en |
  | Short Title | Cortisol release, heart rate and heart rate variability in the horse and its rider |
  | Library Catalogue | DOI.org (Crossref) |
  | URL | https://linkinghub.elsevier.com/retrieve/pii/S1090023313000038 |
  | Accessed | 01/07/2025, 12:19:29 |
  | Volume | 197 |
  | Pages | 229-232 |
  | Publication | The Veterinary Journal |
  | DOI | 10.1016/j.tvjl.2012.12.025 |
  | Issue | 2 |
  | Journal Abbr | The Veterinary Journal |
  | ISSN | 10900233 |
  | Date Added | 01/07/2025, 12:19:29 |
  | Modified | 01/07/2025, 12:19:29 |

  ### Attachments

  - PDF
- ## Long-term stress levels are synchronized in dogs and their owners

  |  |  |
  | --- | --- |
  | Item Type | Journal Article |
  | Author | Ann-Sofie Sundman |
  | Author | Enya Van Poucke |
  | Author | Ann-Charlotte Svensson Holm |
  | Author | Åshild Faresjö |
  | Author | Elvar Theodorsson |
  | Author | Per Jensen |
  | Author | Lina S. V. Roth |
  | Abstract | Abstract This study reveals, for the first time, an interspecific synchronization in long-term stress levels. Previously, acute stress, has been shown to be highly contagious both among humans and between individuals of other species. Here, long-term stress synchronization in dogs and their owners was investigated. We studied 58 dog-human dyads and analyzed their hair cortisol concentrations (HCC) at two separate occasions, reflecting levels during previous summer and winter months. The personality traits of both dogs and their owners were determined through owner-completed Dog Personality Questionnaire (DPQ) and human Big Five Inventory (BFI) surveys. In addition, the dogs’ activity levels were continuously monitored with a remote cloud-based activity collar for one week. Shetland sheepdogs (N = 33) and border collies (N = 25), balanced for sex, participated, and both pet dogs and actively competing dogs (agility and obedience) were included to represent different lifestyles. The results showed significant interspecies correlations in long-term stress where human HCC from both summer and winter samplings correlated strongly with dog HCC (summer: N = 57, χ 2  = 23.697, P < 0.001, β = 0.235; winter: N = 55, χ 2  = 13.796, P < 0.001, β = 0.027). Interestingly, the dogs’ activity levels did not affect HCC, nor did the amount of training sessions per week, showing that the HCC levels were not related to general physical activity. Additionally, there was a seasonal effect in HCC. However, although dogs’ personalities had little effects on their HCC, the human personality traits neuroticism, conscientiousness, and openness significantly affected dog HCC. Hence, we suggest that dogs, to a great extent, mirror the stress level of their owners. |
  | Date | 2019-06-06 |
  | Language | en |
  | Library Catalogue | DOI.org (Crossref) |
  | URL | https://www.nature.com/articles/s41598-019-43851-x |
  | Accessed | 01/07/2025, 17:23:11 |
  | Volume | 9 |
  | Pages | 7391 |
  | Publication | Scientific Reports |
  | DOI | 10.1038/s41598-019-43851-x |
  | Issue | 1 |
  | Journal Abbr | Sci Rep |
  | ISSN | 2045-2322 |
  | Date Added | 01/07/2025, 17:23:11 |
  | Modified | 01/07/2025, 17:23:11 |

  ### Attachments

  - Full Text
- ## Salivary Cortisol Levels in Horses and their Riders During Three-Day-Events

  |  |  |
  | --- | --- |
  | Item Type | Journal Article |
  | Author | Katarzyna Strzelec |
  | Author | Witold Kędzierski |
  | Author | Andrzej Bereznowski |
  | Author | Iwona Janczarek |
  | Author | Krzysztof Bocian |
  | Author | Maciej Radosz |
  | Abstract | Abstract The group of 36 warm-blooded half-bred horses (18 stallions and 18 mares) and their riders (20 men and 16 women), who ended three-day-events, were selected for the study. The horses were aged 4 to 6 years, while the riders were 19 to 34-year-old. The saliva samples were collected after each phase of the competitions. The cortisol concentration was determined using an immunoassay method. The following factors were considered: type of competition, horse sex, and rider gender. In horses, the statistically important correlation was found between the results obtained for the dressage and cross-country, for the cross-country and show jumping, and for the dressage and show jumping. An analogous comparison for the riders suggests a statistically significant correlation between the data obtained for the cross-country and show jumping. Comparing the data of horses and their riders, a significant correlation coefficient was found for the cross-country group of woman and the dressage group of men. In conclusion, the salivary cortisol level in individual horses in each phase of three-day-event was found to be repetitive. Therefore, the salivary cortisol test is demonstrated to be a useful method to evaluate the horse response to each type of competition during three-day-events. |
  | Date | 2013-06-01 |
  | Language | en |
  | Library Catalogue | Crossref |
  | URL | https://www.sciendo.com/article/10.2478/bvip-2013-0042 |
  | Accessed | 08/07/2025, 13:01:45 |
  | Volume | 57 |
  | Publisher | Walter de Gruyter GmbH |
  | Pages | 237-241 |
  | Publication | Bulletin of the Veterinary Institute in Pulawy |
  | DOI | 10.2478/bvip-2013-0042 |
  | Issue | 2 |
  | ISSN | 2300-3235 |
  | Date Added | 08/07/2025, 13:01:45 |
  | Modified | 08/07/2025, 13:01:45 |

  ### Attachments

  - Full Text
- ## Psychobiological Factors Affecting Cortisol Variability in Human-Dog Dyads

  |  |  |
  | --- | --- |
  | Item Type | Journal Article |
  | Author | Iris Schöberl |
  | Author | Manuela Wedl |
  | Author | Andrea Beetz |
  | Author | Kurt Kotrschal |
  | Editor | Urs M. Nater |
  | Date | 2017-2-8 |
  | Language | en |
  | Library Catalogue | DOI.org (Crossref) |
  | URL | https://dx.plos.org/10.1371/journal.pone.0170707 |
  | Accessed | 27/06/2025, 16:34:57 |
  | Volume | 12 |
  | Pages | e0170707 |
  | Publication | PLOS ONE |
  | DOI | 10.1371/journal.pone.0170707 |
  | Issue | 2 |
  | Journal Abbr | PLoS ONE |
  | ISSN | 1932-6203 |
  | Date Added | 27/06/2025, 16:34:57 |
  | Modified | 27/06/2025, 16:34:57 |

  ### Attachments

  - Full Text
- ## Effects of Owner–Dog Relationship and Owner Personality on Cortisol Modulation in Human–Dog Dyads

  |  |  |
  | --- | --- |
  | Item Type | Journal Article |
  | Author | Iris Schöberl |
  | Author | Manuela Wedl |
  | Author | Barbara Bauer |
  | Author | Jon Day |
  | Author | Erich Möstl |
  | Author | Kurt Kotrschal |
  | Date | 06/2012 |
  | Language | en |
  | Library Catalogue | DOI.org (Crossref) |
  | URL | https://www.tandfonline.com/doi/full/10.2752/175303712X13316289505422 |
  | Accessed | 01/07/2025, 12:45:40 |
  | Volume | 25 |
  | Pages | 199-214 |
  | Publication | Anthrozoös |
  | DOI | 10.2752/175303712X13316289505422 |
  | Issue | 2 |
  | Journal Abbr | Anthrozoös |
  | ISSN | 0892-7936, 1753-0377 |
  | Date Added | 01/07/2025, 12:45:40 |
  | Modified | 01/07/2025, 12:45:40 |

  ### Attachments

  - PDF
- ## Physiological Indicators of Attachment in Domestic Dogs (Canis familiaris) and Their Owners in the Strange Situation Test

  |  |  |
  | --- | --- |
  | Item Type | Journal Article |
  | Author | Morag G. Ryan |
  | Author | Anne E. Storey |
  | Author | Rita E. Anderson |
  | Author | Carolyn J. Walsh |
  | Date | 2019-07-23 |
  | Library Catalogue | Crossref |
  | URL | https://www.frontiersin.org/article/10.3389/fnbeh.2019.00162/full |
  | Accessed | 08/07/2025, 12:09:49 |
  | Rights | https://creativecommons.org/licenses/by/4.0/ |
  | Volume | 13 |
  | Publisher | Frontiers Media SA |
  | Publication | Frontiers in Behavioral Neuroscience |
  | DOI | 10.3389/fnbeh.2019.00162 |
  | Journal Abbr | Front. Behav. Neurosci. |
  | ISSN | 1662-5153 |
  | Date Added | 08/07/2025, 12:09:49 |
  | Modified | 08/07/2025, 12:09:49 |

  ### Attachments

  - Full Text
- ## The Effect of Victory and Defeat on the Correlations of Stress Parameters Between the Horse and Rider in Kök‐Börü Equestrian Teams

  |  |  |
  | --- | --- |
  | Item Type | Journal Article |
  | Author | Ali Rişvanli |
  | Author | İsmail Şen |
  | Author | Kanat Canuzakov |
  | Author | Askarbek Tulobayev |
  | Author | Abuzer Taş |
  | Author | Ruslan Salykov |
  | Author | Nezahat Ceylan |
  | Author | Ünal Türkçapar |
  | Author | Ulanbek Alimov |
  | Author | Arina Kazakbayeva |
  | Author | Ayday Cunuşova |
  | Author | Nur Abdimnap Uulu |
  | Author | Burak Fatih Yuksel |
  | Author | Mert Turanli |
  | Author | Muhammed Uz |
  | Author | Metin Bayraktar |
  | Author | Nuriddin Ruzikulov |
  | Abstract | ABSTRACTThe presented study outlines a research plan aimed at determining the effects of winning and losing situations on the relationship levels between the rider and horse's stress, metabolic, and physiological parameters in Kök‐Börü, a traditional equestrian team game. For this purpose, blood samples were collected from both the horses and riders of four different teams participating in two different Kök‐Börü games before and after the games. Cortisol, ACTH, beta‐endorphin, epinephrine, norepinephrine, T3 and T4 analyses were performed on the collected blood samples using species‐specific commercial ELISA kits. Additionally, biochemical and haematological parameters in the same blood samples were tested using an autoanalyser. Based on the obtained data, it was found that there were both positive and negative correlations between most biochemical and haematological parameters of the winning teams' horses and riders before and after the game. However, when examining the correlations between the hormonal parameters of the winning teams' horses and riders before the game, only a negative correlation was found between ACTH and T4 (‐0.529, p < 0.05), and no positive correlation was identified among any hormonal parameters. In conclusion, it was interpreted that in the equestrian team sport of Kök‐Börü, there are significant changes in the hormonal parameters, especially before and after the game, between the horses and riders of the losing teams. Furthermore, it was concluded that winning and losing situations in Kök‐Börü games did not have a significant impact on the correlations between haematological and biochemical parameters before and after the game for both the horses and riders. |
  | Date | 05/2025 |
  | Language | en |
  | Library Catalogue | Crossref |
  | URL | https://onlinelibrary.wiley.com/doi/10.1002/vms3.70356 |
  | Accessed | 09/07/2025, 15:23:27 |
  | Rights | http://creativecommons.org/licenses/by/4.0/ |
  | Volume | 11 |
  | Publisher | Wiley |
  | Publication | Veterinary Medicine and Science |
  | DOI | 10.1002/vms3.70356 |
  | Issue | 3 |
  | Journal Abbr | Veterinary Medicine &amp; Sci |
  | ISSN | 2053-1095, 2053-1095 |
  | Date Added | 09/07/2025, 15:23:27 |
  | Modified | 09/07/2025, 15:23:27 |

  ### Attachments

  - PDF
- ## Disrupted Human–Dog Interbrain Neural Coupling in Autism‐Associated <i>Shank3</i> Mutant Dogs

  |  |  |
  | --- | --- |
  | Item Type | Journal Article |
  | Author | Wei Ren |
  | Author | Shan Yu |
  | Author | Kun Guo |
  | Author | Chunming Lu |
  | Author | Yong Q. Zhang |
  | Abstract | AbstractDogs interact with humans effectively and intimately. However, the neural underpinnings for such interspecies social communication are not understood. It is known that interbrain activity coupling, i.e., the synchronization of neural activity between individuals, represents the neural basis of social interactions. Here, previously unknown cross‐species interbrain activity coupling in interacting human–dog dyads is reported. By analyzing electroencephalography signals from both dogs and humans, it is found that mutual gaze and petting induce interbrain synchronization in the frontal and parietal regions of the human–dog dyads, respectively. The strength of the synchronization increases with growing familiarity of the human–dog dyad over five days, and the information flow analysis suggests that the human is the leader while the dog is the follower during human–dog interactions. Furthermore, dogs with Shank3 mutations, which represent a promising complementary animal model of autism spectrum disorders (ASD), show a loss of interbrain coupling and reduced attention during human–dog interactions. Such abnormalities are rescued by the psychedelic lysergic acid diethylamide (LSD). The results reveal previously unknown interbrain synchronizations within an interacting human–dog dyad which may underlie the interspecies communication, and suggest a potential of LSD for the amelioration of social impairment in patients with ASD. |
  | Date | 11/2024 |
  | Language | en |
  | Library Catalogue | Crossref |
  | URL | https://advanced.onlinelibrary.wiley.com/doi/10.1002/advs.202402493 |
  | Accessed | 11/07/2025, 13:23:15 |
  | Rights | http://creativecommons.org/licenses/by/4.0/ |
  | Volume | 11 |
  | Publisher | Wiley |
  | Publication | Advanced Science |
  | DOI | 10.1002/advs.202402493 |
  | Issue | 41 |
  | ISSN | 2198-3844, 2198-3844 |
  | Date Added | 11/07/2025, 13:23:15 |
  | Modified | 11/07/2025, 13:23:15 |

  ### Attachments

  - Full Text PDF
- ## Heart rate variability responses of horses and veterans with post-traumatic stress disorder to ground-based adaptive horsemanship lessons: a pilot study

  |  |  |
  | --- | --- |
  | Item Type | Journal Article |
  | Author | Ellen M Rankins |
  | Author | Boluwatife E Faremi |
  | Author | Kyle Hartmann |
  | Author | Andrea Quinn |
  | Author | Hugo F Posada-Quintero |
  | Author | Kenneth H McKeever |
  | Author | Karyn Malinowski |
  | Abstract | Abstract Heart rate variability (HRV) can be measured as an indicator of autonomic nervous system (ANS) balance and thus, stress and affective arousal. Mixed results have been reported in the limited literature addressing the effects of equine-assisted services (EAS) on HRV in the human and horse participants. The aims of the present study were to determine the effects of ground-based adaptive horsemanship (AH) lessons on veterans’ and horses’ HRV during weekly lessons as well as veterans’ resting HRV outside of lessons. Veterans with post-traumatic stress disorder (PTSD) were randomly assigned to 8 wk of AH or control (CON, no changes in treatments or activities) conditions. Horses were assigned to AH or control (CON, stall in arena) conditions based on previous experience (EAS or recreational riding). Electrocardiogram traces from the veteran and AH and CON horses were recorded during the 30-min lessons. Traces were recorded during resting conditions before (PRE) and after (POST) the 8-wk period from AH and CON veterans and healthy, non-veterans. Heart rate (HR), root mean square of successive differences (RMSSD) and low frequency to high frequency ratios (LF/HF) were calculated after identification of peaks with the Pan-Tompkins algorithm and manual correction during 5-min epochs. Data were analyzed with repeated measures, mixed model ANOVAs (SAS v9.4). HR was lowest (p ≤ 0.0323) in the horses and veterans during the first 10 min of the lessons. Veterans’ RMSSD was higher (p ≤ 0.0496) in weeks 4 and 6 than week 2. LF/HF was greater in veterans with PTSD than healthy, non-veterans across PRE and POST time points. Increased HR later in the lessons is likely a result of increased movement in the horses and veterans. An interval of decreased stress and increased resiliency might be present in veterans participating in multi-day AH sessions. Increased sympathetic arousal measured via LF/HF was not mitigated by 8 wk of AH. Horses appeared unstressed by the interactions as no changes in HRV were observed. |
  | Date | 2025-01-07 |
  | Language | en |
  | Short Title | Heart rate variability responses of horses and veterans with post-traumatic stress disorder to ground-based adaptive horsemanship lessons |
  | Library Catalogue | DOI.org (Crossref) |
  | URL | https://academic.oup.com/tas/article/doi/10.1093/tas/txaf019/8010614 |
  | Accessed | 01/07/2025, 17:51:41 |
  | Rights | https://creativecommons.org/licenses/by-nc-nd/4.0/ |
  | Volume | 9 |
  | Pages | txaf019 |
  | Publication | Translational Animal Science |
  | DOI | 10.1093/tas/txaf019 |
  | ISSN | 2573-2102 |
  | Date Added | 01/07/2025, 17:51:41 |
  | Modified | 01/07/2025, 17:51:41 |

  ### Attachments

  - Full Text PDF
- ## Measuring social synchrony and stress in the handler-dog dyad during animal-assisted activities: A pilot study

  |  |  |
  | --- | --- |
  | Item Type | Journal Article |
  | Author | Federica Pirrone |
  | Author | Alessandra Ripamonti |
  | Author | Elena C. Garoni |
  | Author | Sabrina Stradiotti |
  | Author | Mariangela Albertini |
  | Date | 09/2017 |
  | Language | en |
  | Short Title | Measuring social synchrony and stress in the handler-dog dyad during animal-assisted activities |
  | Library Catalogue | DOI.org (Crossref) |
  | URL | https://linkinghub.elsevier.com/retrieve/pii/S1558787817300527 |
  | Accessed | 01/07/2025, 16:49:07 |
  | Volume | 21 |
  | Pages | 45-52 |
  | Publication | Journal of Veterinary Behavior |
  | DOI | 10.1016/j.jveb.2017.07.004 |
  | Journal Abbr | Journal of Veterinary Behavior |
  | ISSN | 15587878 |
  | Date Added | 01/07/2025, 16:49:07 |
  | Modified | 01/07/2025, 16:49:07 |

  ### Attachments

  - Accepted Version
- ## Rider and Horse Salivary Cortisol Levels During Competition and Impact on Performance

  |  |  |
  | --- | --- |
  | Item Type | Journal Article |
  | Author | Marie Peeters |
  | Author | Coline Closson |
  | Author | Jean-François Beckers |
  | Author | Marc Vandenheede |
  | Date | 03/2013 |
  | Language | en |
  | Library Catalogue | DOI.org (Crossref) |
  | URL | https://linkinghub.elsevier.com/retrieve/pii/S0737080612002961 |
  | Accessed | 01/07/2025, 18:01:50 |
  | Rights | https://www.elsevier.com/tdm/userlicense/1.0/ |
  | Volume | 33 |
  | Pages | 155-160 |
  | Publication | Journal of Equine Veterinary Science |
  | DOI | 10.1016/j.jevs.2012.05.073 |
  | Issue | 3 |
  | Journal Abbr | Journal of Equine Veterinary Science |
  | ISSN | 07370806 |
  | Date Added | 01/07/2025, 18:01:50 |
  | Modified | 01/07/2025, 18:01:50 |

  ### Attachments

  - Full Text
- ## Noncontact Electrophysiology Monitoring Systems for Assessment of Canine-Human Interactions

  |  |  |
  | --- | --- |
  | Item Type | Journal Article |
  | Author | Aakash Patel |
  | Author | Marc Foster |
  | Author | T. Torfs |
  | Author | P. Ahmmed |
  | Author | I. Castro |
  | Author | Timothy Holder |
  | Author | A. Bozkurt |
  | Date | 2021-10-31 |
  | URL | https://consensus.app/papers/noncontact-electrophysiology-monitoring-systems-for-patel-foster/0eeed0f0fd9750729fde69f8f3929af0/ |
  | Pages | 1-4 |
  | Publication | 2021 IEEE Sensors |
  | DOI | 10.1109/SENSORS47087.2021.9639748 |
  | Journal Abbr | 2021 IEEE Sensors |
  | Date Added | 17/06/2025, 18:30:59 |
  | Modified | 17/06/2025, 18:30:59 |

  ### Attachments

  - PDF
- ## Neurophysiological Correlates of Affiliative Behaviour between Humans and Dogs

  |  |  |
  | --- | --- |
  | Item Type | Journal Article |
  | Author | J.S.J Odendaal |
  | Author | R.A Meintjes |
  | Abstract | Few physiological parameters for positive human–companion animal contact have been identiﬁed and those that are established have all been in humans. The implication is that if the physiological reactions are mutual, dogs would experience the same psychological beneﬁts from these neurophysiological changes as humans. Therefore, we have determined the role of certain neurochemicals during afﬁliation behaviour on an interspecies basis. Our results indicate that concentrations of b-endorphin, oxytocin, prolactin, b-phenylethylamine, and dopamine increased in both species after positive interspecies interaction, while that of cortisol decreased in the humans only. Indicators of mutual physiological changes during positive interaction between dog lovers and dogs may contribute to a better understanding of the human–animal bond in veterinary practice. |
  | Date | 5/2003 |
  | Language | en |
  | Library Catalogue | DOI.org (Crossref) |
  | URL | https://linkinghub.elsevier.com/retrieve/pii/S109002330200237X |
  | Accessed | 17/06/2025, 17:10:02 |
  | Rights | https://www.elsevier.com/tdm/userlicense/1.0/ |
  | Volume | 165 |
  | Pages | 296-301 |
  | Publication | The Veterinary Journal |
  | DOI | 10.1016/S1090-0233(02)00237-X |
  | Issue | 3 |
  | Journal Abbr | The Veterinary Journal |
  | ISSN | 10900233 |
  | Date Added | 17/06/2025, 17:10:02 |
  | Modified | 17/06/2025, 17:10:02 |

  ### Attachments

  - PDF
- ## Animal-assisted therapy — magic or medicine?

  |  |  |
  | --- | --- |
  | Item Type | Journal Article |
  | Author | J.S.J Odendaal |
  | Date | 10/2000 |
  | Language | en |
  | Library Catalogue | DOI.org (Crossref) |
  | URL | https://linkinghub.elsevier.com/retrieve/pii/S0022399900001835 |
  | Accessed | 17/06/2025, 17:08:24 |
  | Rights | https://www.elsevier.com/tdm/userlicense/1.0/ |
  | Volume | 49 |
  | Pages | 275-280 |
  | Publication | Journal of Psychosomatic Research |
  | DOI | 10.1016/S0022-3999(00)00183-5 |
  | Issue | 4 |
  | Journal Abbr | Journal of Psychosomatic Research |
  | ISSN | 00223999 |
  | Date Added | 17/06/2025, 17:08:24 |
  | Modified | 17/06/2025, 17:08:24 |

  ### Attachments

  - PDF
- ## Dog’s breath rhythm was drawn into owner’s breath rhythm

  |  |  |
  | --- | --- |
  | Item Type | Journal Article |
  | Author | Kensaku Nomoto |
  | Author | Tomoki Hashimoto |
  | Author | Miho Nagasawa |
  | Author | Takefumi Kikusui |
  | Date | 2024-07-17 |
  | Language | en |
  | Library Catalogue | Crossref |
  | URL | https://www.tandfonline.com/doi/full/10.1080/01691864.2024.2369795 |
  | Accessed | 10/07/2025, 13:53:08 |
  | Volume | 38 |
  | Publisher | Informa UK Limited |
  | Pages | 926-933 |
  | Publication | Advanced Robotics |
  | DOI | 10.1080/01691864.2024.2369795 |
  | Issue | 14 |
  | ISSN | 0169-1864, 1568-5535 |
  | Date Added | 10/07/2025, 13:53:08 |
  | Modified | 10/07/2025, 13:53:08 |

  ### Attachments

  - PDF
- ## Oxytocin-gaze positive loop and the coevolution of human-dog bonds

  |  |  |
  | --- | --- |
  | Item Type | Journal Article |
  | Author | Miho Nagasawa |
  | Author | Shouhei Mitsui |
  | Author | Shiori En |
  | Author | Nobuyo Ohtani |
  | Author | Mitsuaki Ohta |
  | Author | Yasuo Sakuma |
  | Author | Tatsushi Onaka |
  | Author | Kazutaka Mogi |
  | Author | Takefumi Kikusui |
  | Date | 2015 |
  | Language | en |
  | Library Catalogue | Zotero |
  | Date Added | 17/06/2025, 17:34:17 |
  | Modified | 15/07/2025, 09:33:15 |

  ### Attachments

  - 1261022-nagasawa-sm
  - PDF
- ## Dogs showed lower parasympathetic activity during mutual gazing while owners did not

  |  |  |
  | --- | --- |
  | Item Type | Journal Article |
  | Author | Miho Nagasawa |
  | Author | Maaya Saito |
  | Author | Haruka Hirasawa |
  | Author | Kazutaka Mogi |
  | Author | Takefumi Kikusui |
  | Date | 2023 |
  | Language | en |
  | Library Catalogue | DOI.org (Crossref) |
  | URL | https://linkinghub.elsevier.com/retrieve/pii/S1880654624000064 |
  | Accessed | 01/07/2025, 17:27:09 |
  | Volume | 73 |
  | Pages | 9 |
  | Publication | The Journal of Physiological Sciences |
  | DOI | 10.1186/s12576-023-00863-7 |
  | Issue | 1 |
  | Journal Abbr | The Journal of Physiological Sciences |
  | ISSN | 18806546 |
  | Date Added | 01/07/2025, 17:27:09 |
  | Modified | 01/07/2025, 17:27:09 |

  ### Attachments

  - Full Text
- ## Heart rate and salivary cortisol as indicators of arousal and synchrony in clients, therapy horses and therapist in equine-assisted therapy

  |  |  |
  | --- | --- |
  | Item Type | Journal Article |
  | Author | A. Naber |
  | Author | L. Kreuzer |
  | Author | R. Zink |
  | Author | E. Millesi |
  | Author | R. Palme |
  | Author | K. Hediger |
  | Author | L.M. Glenk |
  | Date | 05/2025 |
  | Language | en |
  | Library Catalogue | DOI.org (Crossref) |
  | URL | https://linkinghub.elsevier.com/retrieve/pii/S1744388125000027 |
  | Accessed | 27/06/2025, 17:18:09 |
  | Volume | 59 |
  | Pages | 101937 |
  | Publication | Complementary Therapies in Clinical Practice |
  | DOI | 10.1016/j.ctcp.2025.101937 |
  | Journal Abbr | Complementary Therapies in Clinical Practice |
  | ISSN | 17443881 |
  | Date Added | 27/06/2025, 17:18:09 |
  | Modified | 27/06/2025, 17:18:09 |

  ### Attachments

  - PDF
- ## The influence of challenging objects and horse-rider matching on heart rate, heart rate variability and behavioural score in riding horses

  |  |  |
  | --- | --- |
  | Item Type | Journal Article |
  | Author | Carolien C.B.M. Munsters |
  | Author | Kathalijne E.K. Visser |
  | Author | Jan Van Den Broek |
  | Author | Marianne M. Sloet Van Oldruitenborgh-Oosterbaan |
  | Date | 04/2012 |
  | Language | en |
  | Library Catalogue | Crossref |
  | URL | https://linkinghub.elsevier.com/retrieve/pii/S1090023311001493 |
  | Accessed | 08/07/2025, 11:51:07 |
  | Rights | https://www.elsevier.com/tdm/userlicense/1.0/ |
  | Volume | 192 |
  | Publisher | Elsevier BV |
  | Pages | 75-80 |
  | Publication | The Veterinary Journal |
  | DOI | 10.1016/j.tvjl.2011.04.011 |
  | Issue | 1 |
  | ISSN | 1090-0233 |
  | Date Added | 08/07/2025, 11:51:07 |
  | Modified | 08/07/2025, 11:51:07 |

  ### Attachments

  - PDF
- ## A Relaxed Horse—A Relaxed Client? An Experimental Investigation of the Effects of Therapy Horses’ Stress on Clients’ Stress, Mood, and Anxiety

  |  |  |
  | --- | --- |
  | Item Type | Journal Article |
  | Author | Alicia Müller-Klein |
  | Author | Moritz Nicolai Braun |
  | Author | Diana S. Ferreira De Sá |
  | Author | Tanja Michael |
  | Author | Ulrike Link-Dorner |
  | Author | Johanna Lass-Hennemann |
  | Abstract | Equine-assisted therapies are becoming increasingly popular for addressing physical and psychological disabilities in clients. The role of the horse’s welfare in equine-assisted service receives increasing attention in research. Several studies have shown that horses are able to perceive human emotions and respond to human stress responses. However, no research has yet looked at the other side of the coin—whether and how humans perceive and react to equine stress levels during equine-assisted services. To fill this gap in the research, we employed a within-subjects design, in which horse-naïve participants had a standardized interaction with both an experimentally stressed horse and an experimentally relaxed horse. We assessed physiological indicators of stress (heart rate, heart rate variability, and salivary cortisol) in participants and horses, as well as psychological indicators of stress (state anxiety and positive and negative affect) in participants. Although our stress and relaxation manipulations were successful (indicated by horses’ physiological indicators of stress), we did not find any difference in the participants’ physiological or psychological indicators of stress between the interaction with a stressed and the interaction with a relaxed horse. Together with results from previous studies, this suggests that humans cannot intuitively recognize the (physiological) stress level of horses, which has important implications for effective communication and bonding between humans and horses and for the safety of equine activities. |
  | Date | 2024-02-13 |
  | Language | en |
  | Short Title | A Relaxed Horse—A Relaxed Client? |
  | Library Catalogue | DOI.org (Crossref) |
  | URL | https://www.mdpi.com/2076-2615/14/4/604 |
  | Accessed | 01/07/2025, 13:10:56 |
  | Rights | https://creativecommons.org/licenses/by/4.0/ |
  | Volume | 14 |
  | Pages | 604 |
  | Publication | Animals |
  | DOI | 10.3390/ani14040604 |
  | Issue | 4 |
  | Journal Abbr | Animals |
  | ISSN | 2076-2615 |
  | Date Added | 01/07/2025, 13:10:56 |
  | Modified | 01/07/2025, 13:10:56 |

  ### Attachments

  - Full Text
- ## Preliminary results suggest an influence of psychological and physiological stress in humans on horse heart rate and behavior

  |  |  |
  | --- | --- |
  | Item Type | Journal Article |
  | Author | Katrina Merkies |
  | Author | Anja Sievers |
  | Author | Emily Zakrajsek |
  | Author | Helen MacGregor |
  | Author | Renée Bergeron |
  | Author | Uta König Von Borstel |
  | Date | 09/2014 |
  | Language | en |
  | Library Catalogue | DOI.org (Crossref) |
  | URL | https://linkinghub.elsevier.com/retrieve/pii/S1558787814000860 |
  | Accessed | 01/07/2025, 12:24:40 |
  | Volume | 9 |
  | Pages | 242-247 |
  | Publication | Journal of Veterinary Behavior |
  | DOI | 10.1016/j.jveb.2014.06.003 |
  | Issue | 5 |
  | Journal Abbr | Journal of Veterinary Behavior |
  | ISSN | 15587878 |
  | Date Added | 01/07/2025, 12:24:40 |
  | Modified | 01/07/2025, 12:24:40 |

  ### Attachments

  - PDF
- ## The Role of Oxytocin in the Dog–Owner Relationship

  |  |  |
  | --- | --- |
  | Item Type | Journal Article |
  | Author | Anne Meinert |
  | Author | T. Deschner |
  | Author | F. Schaebs |
  | Author | S. Marshall-Pescini |
  | Author | F. Range |
  | Author | Alina Gaugg |
  | Date | 2019-10-01 |
  | URL | https://consensus.app/papers/the-role-of-oxytocin-in-the-dog%E2%80%93owner-relationship-meinert-deschner/da7848f459b15165a232f855e1d6f051/ |
  | Volume | 9 |
  | Publication | Animals : an Open Access Journal from MDPI |
  | DOI | 10.3390/ani9100792 |
  | Journal Abbr | Animals : an Open Access Journal from MDPI |
  | Date Added | 20/06/2025, 10:21:07 |
  | Modified | 20/06/2025, 10:21:07 |

  ### Attachments

  - PDF
- ## Psychophysiological effects of equine-facilitated psychotherapy on Veterans with PTSD and their horse partners

  |  |  |
  | --- | --- |
  | Item Type | Journal Article |
  | Author | Laurie A. McDuffee |
  | Author | William J. Montelpare |
  | Author | Caroline LeBlanc |
  | Abstract | LAY SUMMARY Veterans with posttraumatic stress disorder (PTSD) often struggle with emotion and impulse control, resulting in an inability to appropriately handle even minimal stress, which can lead to physiological dysregulation. This study used a variety of measures to assess the effect of equine-facilitated psychotherapy on Veterans with PTSD. Given that maintaining practices of good welfare for animals is essential in supporting these types of therapeutic programs for Veterans, the study also evaluated the welfare of the horse participants. The results showed that equine-facilitated psychotherapy had a positive impact on the perceived post-program responses of human participants and that horses were not stressed during the sessions and likely perceived the sessions as a neutral stimulus. |
  | Date | 2024-06-01 |
  | Language | en |
  | Library Catalogue | Crossref |
  | URL | https://utppublishing.com/doi/10.3138/jmvfh-2023-0063 |
  | Accessed | 10/07/2025, 12:49:53 |
  | Volume | 10 |
  | Publisher | University of Toronto Press Inc. (UTPress) |
  | Pages | 135-147 |
  | Publication | Journal of Military, Veteran and Family Health |
  | DOI | 10.3138/jmvfh-2023-0063 |
  | Issue | 3 |
  | ISSN | 2368-7924, 2368-7924 |
  | Date Added | 10/07/2025, 12:49:53 |
  | Modified | 10/07/2025, 12:49:53 |

  ### Attachments

  - PDF
- ## The Effects of Equine Assisted Therapy on Plasma Cortisol and Oxytocin Concentrations and Heart Rate Variability in Horses and Measures of Symptoms of Post-Traumatic Stress Disorder in Veterans

  |  |  |
  | --- | --- |
  | Item Type | Journal Article |
  | Author | Karyn Malinowski |
  | Author | Chi Yee |
  | Author | Jenni M. Tevlin |
  | Author | Eric K. Birks |
  | Author | Mary M. Durando |
  | Author | Hossein Pournajafi-Nazarloo |
  | Author | Alan A. Cavaiola |
  | Author | Kenneth H. McKeever |
  | Date | 05/2018 |
  | Language | en |
  | Library Catalogue | Crossref |
  | URL | https://linkinghub.elsevier.com/retrieve/pii/S0737080617307761 |
  | Accessed | 08/07/2025, 12:39:03 |
  | Rights | https://www.elsevier.com/tdm/userlicense/1.0/ |
  | Volume | 64 |
  | Publisher | Elsevier BV |
  | Pages | 17-26 |
  | Publication | Journal of Equine Veterinary Science |
  | DOI | 10.1016/j.jevs.2018.01.011 |
  | ISSN | 0737-0806 |
  | Date Added | 08/07/2025, 12:39:03 |
  | Modified | 08/07/2025, 12:39:03 |

  ### Attachments

  - PDF
- ## Quantitative heartbeat coupling measures in human-horse interaction

  |  |  |
  | --- | --- |
  | Item Type | Conference Paper |
  | Author | Antonio Lanata |
  | Author | Andrea Guidi |
  | Author | Gaetano Valenza |
  | Author | Paolo Baragli |
  | Author | Enzo Pasquale Scilingo |
  | Date | 8/2016 |
  | Library Catalogue | DOI.org (Crossref) |
  | URL | http://ieeexplore.ieee.org/document/7591286/ |
  | Accessed | 20/06/2025, 10:03:28 |
  | Place | Orlando, FL, USA |
  | Publisher | IEEE |
  | ISBN | 978-1-4577-0220-4 |
  | Pages | 2696-2699 |
  | Proceedings Title | 2016 38th Annual International Conference of the IEEE Engineering in Medicine and Biology Society (EMBC) |
  | Conference Name | 2016 38th Annual International Conference of the IEEE Engineering in Medicine and Biology Society (EMBC) |
  | DOI | 10.1109/EMBC.2016.7591286 |
  | Date Added | 20/06/2025, 10:03:28 |
  | Modified | 20/06/2025, 10:03:28 |

  ### Attachments

  - PDF
- ## The role of nonlinear coupling in Human-Horse Interaction: A preliminary study

  |  |  |
  | --- | --- |
  | Item Type | Conference Paper |
  | Author | Antonio Lanata |
  | Author | Andrea Guidi |
  | Author | Gaetano Valenza |
  | Author | Paolo Baragli |
  | Author | Enzo Pasquale Scilingo |
  | Date | 7/2017 |
  | Short Title | The role of nonlinear coupling in Human-Horse Interaction |
  | Library Catalogue | DOI.org (Crossref) |
  | URL | https://ieeexplore.ieee.org/document/8037075/ |
  | Accessed | 20/06/2025, 10:04:42 |
  | Place | Seogwipo |
  | Publisher | IEEE |
  | ISBN | 978-1-5090-2809-2 |
  | Pages | 1320-1323 |
  | Proceedings Title | 2017 39th Annual International Conference of the IEEE Engineering in Medicine and Biology Society (EMBC) |
  | Conference Name | 2017 39th Annual International Conference of the IEEE Engineering in Medicine and Biology Society (EMBC) |
  | DOI | 10.1109/EMBC.2017.8037075 |
  | Date Added | 20/06/2025, 10:04:42 |
  | Modified | 20/06/2025, 10:04:42 |

  ### Attachments

  - PDF
- ## Dog-Owner Attachment Is Associated With Oxytocin Receptor Gene Polymorphisms in Both Parties. A Comparative Study on Austrian and Hungarian Border Collies

  |  |  |
  | --- | --- |
  | Item Type | Journal Article |
  | Author | Krisztina Kovács |
  | Author | Zsófia Virányi |
  | Author | Anna Kis |
  | Author | Borbála Turcsán |
  | Author | Ágnes Hudecz |
  | Author | Maria T. Marmota |
  | Author | Dóra Koller |
  | Author | Zsolt Rónai |
  | Author | Márta Gácsi |
  | Author | József Topál |
  | Date | 2018-4-5 |
  | Library Catalogue | DOI.org (Crossref) |
  | URL | http://journal.frontiersin.org/article/10.3389/fpsyg.2018.00435/full |
  | Accessed | 01/07/2025, 15:46:33 |
  | Volume | 9 |
  | Pages | 435 |
  | Publication | Frontiers in Psychology |
  | DOI | 10.3389/fpsyg.2018.00435 |
  | Journal Abbr | Front. Psychol. |
  | ISSN | 1664-1078 |
  | Date Added | 01/07/2025, 15:46:33 |
  | Modified | 01/07/2025, 15:46:33 |

  ### Attachments

  - Full Text
- ## Dyadic relationships and operational performance of male and female owners and their male dogs

  |  |  |
  | --- | --- |
  | Item Type | Journal Article |
  | Author | Kurt Kotrschal |
  | Author | Iris Schöberl |
  | Author | Barbara Bauer |
  | Author | Anne-Marie Thibeaut |
  | Author | Manuela Wedl |
  | Date | 07/2009 |
  | Language | en |
  | Library Catalogue | Crossref |
  | URL | https://linkinghub.elsevier.com/retrieve/pii/S037663570900103X |
  | Accessed | 08/07/2025, 16:18:57 |
  | Rights | https://www.elsevier.com/tdm/userlicense/1.0/ |
  | Volume | 81 |
  | Publisher | Elsevier BV |
  | Pages | 383-391 |
  | Publication | Behavioural Processes |
  | DOI | 10.1016/j.beproc.2009.04.001 |
  | Issue | 3 |
  | ISSN | 0376-6357 |
  | Date Added | 08/07/2025, 16:18:57 |
  | Modified | 08/07/2025, 16:18:57 |

  ### Attachments

  - PDF
- ## Behavioral and emotional co-modulation during dog–owner interaction measured by heart rate variability and activity

  |  |  |
  | --- | --- |
  | Item Type | Journal Article |
  | Author | Aija Koskela |
  | Author | Heini Törnqvist |
  | Author | Sanni Somppi |
  | Author | Katriina Tiira |
  | Author | Virpi-Liisa Kykyri |
  | Author | Laura Hänninen |
  | Author | Jan Kujala |
  | Author | Miho Nagasawa |
  | Author | Takefumi Kikusui |
  | Author | Miiamaaria V. Kujala |
  | Abstract | Behavioral and physiological synchrony facilitate emotional closeness in attachment relationships. The aim of this pseudorandomized cross-over study was to investigate the emotional and physiological link, designated as co-modulation, between dogs and their owners. We measured the heart rate variability (HRV) and physical activity of dogs belonging to co-operative breeds (n = 29) and their owners during resting baselines and positive interaction tasks (Stroking, Training, Sniffing, Playing) and collected survey data on owner temperament and dog–owner relationship. Although overall HRV and activity correlated between dogs and their owners across tasks, task-specific analyses showed that HRV of dogs and owners correlated during free behaving (Pre- and Post-Baseline), whereas the activity of dogs and owners correlated during predefined interaction tasks (Stroking and Playing). Dog overall HRV was the only predictive factor for owner overall HRV, while dog height, ownership duration, owner negative affectivity, and dog–owner interaction scale predicted dog overall HRV. Thus, the characteristics of dog, owner, and the relationship modified the HRV responses in dog–owner dyads. The physiology and behavior of dogs belonging to co-operative breeds and their owners were therefore co-modulated, demonstrating physiological and emotional connection comparable to those found in attachment relationships between humans. |
  | Date | 2024-10-24 |
  | Language | en |
  | Library Catalogue | DOI.org (Crossref) |
  | URL | https://www.nature.com/articles/s41598-024-76831-x |
  | Accessed | 17/06/2025, 16:27:02 |
  | Volume | 14 |
  | Pages | 25201 |
  | Publication | Scientific Reports |
  | DOI | 10.1038/s41598-024-76831-x |
  | Issue | 1 |
  | Journal Abbr | Sci Rep |
  | ISSN | 2045-2322 |
  | Date Added | 17/06/2025, 16:27:02 |
  | Modified | 17/06/2025, 16:27:03 |

  ### Attachments

  - PDF
- ## Oxytocin bonds between human and dog

  |  |  |
  | --- | --- |
  | Item Type | Journal Article |
  | Author | Takefumi Kikusui |
  | Date | 2017 |
  | Language | en |
  | Library Catalogue | Crossref |
  | URL | https://www.jstage.jst.go.jp/article/janip/67/1/67\_67.1.1/\_article |
  | Accessed | 09/07/2025, 11:29:03 |
  | Volume | 67 |
  | Publisher | Japanese Society of Animal Psychology |
  | Pages | 19-27 |
  | Publication | Japanese Journal of Animal Psychology |
  | DOI | 10.2502/janip.67.1.1 |
  | Issue | 1 |
  | Journal Abbr | Japanese Journal of Animal Psychology, The Japanese Journal of Animal Psychology |
  | ISSN | 0916-8419, 1880-9022 |
  | Date Added | 09/07/2025, 11:29:03 |
  | Modified | 09/07/2025, 11:29:03 |

  ### Attachments

  - Full Text
- ## Investigating horse–human interactions: The effect of a nervous human

  |  |  |
  | --- | --- |
  | Item Type | Journal Article |
  | Author | Linda J. Keeling |
  | Author | Liv Jonare |
  | Author | Lovisa Lanneborn |
  | Date | 7/2009 |
  | Language | en |
  | Short Title | Investigating horse–human interactions |
  | Library Catalogue | DOI.org (Crossref) |
  | URL | https://linkinghub.elsevier.com/retrieve/pii/S109002330900121X |
  | Accessed | 01/07/2025, 12:26:52 |
  | Rights | https://www.elsevier.com/tdm/userlicense/1.0/ |
  | Volume | 181 |
  | Pages | 70-71 |
  | Publication | The Veterinary Journal |
  | DOI | 10.1016/j.tvjl.2009.03.013 |
  | Issue | 1 |
  | Journal Abbr | The Veterinary Journal |
  | ISSN | 10900233 |
  | Date Added | 01/07/2025, 12:26:52 |
  | Modified | 01/07/2025, 12:26:52 |

  ### Attachments

  - PDF
- ## Emotional Contagion From Humans to Dogs Is Facilitated by Duration of Ownership

  |  |  |
  | --- | --- |
  | Item Type | Journal Article |
  | Author | Maki Katayama |
  | Author | Takatomi Kubo |
  | Author | Toshitaka Yamakawa |
  | Author | Koichi Fujiwara |
  | Author | Kensaku Nomoto |
  | Author | Kazushi Ikeda |
  | Author | Kazutaka Mogi |
  | Author | Miho Nagasawa |
  | Author | Takefumi Kikusui |
  | Date | 2019-07-19 |
  | Library Catalogue | Crossref |
  | URL | https://www.frontiersin.org/article/10.3389/fpsyg.2019.01678/full |
  | Accessed | 09/07/2025, 11:31:56 |
  | Rights | https://creativecommons.org/licenses/by/4.0/ |
  | Volume | 10 |
  | Publisher | Frontiers Media SA |
  | Publication | Frontiers in Psychology |
  | DOI | 10.3389/fpsyg.2019.01678 |
  | Journal Abbr | Front. Psychol. |
  | ISSN | 1664-1078 |
  | Date Added | 09/07/2025, 11:31:56 |
  | Modified | 09/07/2025, 11:31:56 |

  ### Attachments

  - Full Text
- ## Influence of Horse and Rider on Stress during Horse-riding Lesson Program

  |  |  |
  | --- | --- |
  | Item Type | Journal Article |
  | Author | Ok-Deuk Kang |
  | Author | Young-Min Yun |
  | Date | 2016-03-22 |
  | Language | en |
  | Library Catalogue | Crossref |
  | URL | http://ajas.info/journal/view.php?doi=10.5713/ajas.15.1068 |
  | Accessed | 08/07/2025, 12:59:30 |
  | Volume | 29 |
  | Publisher | Asian Australasian Association of Animal Production Societies |
  | Pages | 895-900 |
  | Publication | Asian-Australasian Journal of Animal Sciences |
  | DOI | 10.5713/ajas.15.1068 |
  | Issue | 6 |
  | Journal Abbr | Asian Australas. J. Anim. Sci |
  | ISSN | 1011-2367, 1976-5517 |
  | Date Added | 08/07/2025, 12:59:30 |
  | Modified | 08/07/2025, 12:59:30 |

  ### Attachments

  - Full Text
- ## Interspecies hormonal interactions between man and the domestic dog (Canis familiaris)

  |  |  |
  | --- | --- |
  | Item Type | Journal Article |
  | Author | Amanda C. Jones |
  | Author | Robert A. Josephs |
  | Date | 09/2006 |
  | Language | en |
  | Library Catalogue | Crossref |
  | URL | https://linkinghub.elsevier.com/retrieve/pii/S0018506X0600122X |
  | Accessed | 08/07/2025, 12:19:26 |
  | Rights | https://www.elsevier.com/tdm/userlicense/1.0/ |
  | Volume | 50 |
  | Publisher | Elsevier BV |
  | Pages | 393-400 |
  | Publication | Hormones and Behavior |
  | DOI | 10.1016/j.yhbeh.2006.04.007 |
  | Issue | 3 |
  | ISSN | 0018-506X |
  | Date Added | 08/07/2025, 12:19:26 |
  | Modified | 08/07/2025, 12:19:26 |

  ### Attachments

  - PDF
- ## Emotional reactions of horses and trainers during natural method training / Reakcje emocjonalne koni i trenerów podczas treningu metodami naturalnymi

  |  |  |
  | --- | --- |
  | Item Type | Journal Article |
  | Author | Iwona Janczarek |
  | Author | Witold Kędzierski |
  | Author | Anna Stachurska |
  | Author | Izabela Wilk |
  | Abstract | Abstract The first aim of the present study was to evaluate whether the trainer factor and the sex of the horse affect the heart rate (HR) of the trainer-horse pair. The second aim was to estimate the level of the trainer-horse pair’s emotions and to find the relationship of the HR between the trainer and the horse during the preliminary, natural-method training. The animals used in the study were 40 three-year-old purebred Arabian horses trained by two trainers from the Silversand Horsemanship School. Each trainer worked with 20 randomly selected horses, equally grouped by sex. The study was carried out during the first day of the training cycle. The aim was to have a horse accept a rider. The following items were subject to analysis: deconcentration, concentration, desensitizing, putting on the lungeing surcingle, and saddling. The emotional status of the horses and the trainers was evaluated based on HR variations which were measured by applying Polar S810 telemetric devices. The device produced continuous measurements with readings every 60 seconds. Two-factor analysis of variance and Pearson correlations were determined with the use of SAS software. Significance of differences between mean values was verified using Tukey’s test. The results obtained revealed that the sex of the trained horses was not an important factor in the evaluation of trainer’s emotions, despite the fact that fillies were characterized as having a more uniform HR. The trainer is very responsible for the emotions of a trained horse, especially at the beginning of training and during saddling. From a trainer’s point of view, it is important to complete the horse concentration task as quickly as possible. The lack of an emotional relationship in the trainer-horse pair during some training elements, suggests that it is not only the trainer’s experience, but mainly the trainer’s personality that determines the probable success in naturalmethod work. |
  | Date | 2013-03-1 |
  | Library Catalogue | DOI.org (Crossref) |
  | URL | https://content.sciendo.com/doi/10.2478/aoas-2013-0008 |
  | Accessed | 01/07/2025, 13:03:05 |
  | Volume | 13 |
  | Pages | 263-273 |
  | Publication | Annals of Animal Science |
  | DOI | 10.2478/aoas-2013-0008 |
  | Issue | 2 |
  | ISSN | 1642-3402 |
  | Date Added | 01/07/2025, 13:03:05 |
  | Modified | 01/07/2025, 13:03:05 |

  ### Attachments

  - Full Text PDF
- ## Effects of the level of experience of horses and their riders on Cortisol release, heart rate and heart-rate variability during a jumping course

  |  |  |
  | --- | --- |
  | Item Type | Journal Article |
  | Author | N Ille |
  | Author | M Von Lewinski |
  | Author | R Erber |
  | Author | M Wulf |
  | Author | J Aurich |
  | Author | E Möstl |
  | Author | C Aurich |
  | Abstract | Abstract Equestrian sports require the co-operation of two species, horses and humans, but it is unknown to what extent stress responses in the rider affect the horse. In this study, the stress response of experienced and less-experienced horses and riders at showjumping was analysed. Sixteen sport horses were divided into two groups (n = 8 each) by experience and were ridden by highly experienced professionals (n = 8) and less-experienced riders (n = 8). Riders jumped a course of obstacles with an experienced and a less-experienced horse and horses took part with an experienced and less-experienced rider. Salivary cortisol, heart rate and heart-rate variability (HRV) variables, standard deviation of RR interval (SDRR) and root mean square of successive RR differences (RMSSD) were analysed. Cortisol and heart rate increased and HRV decreased in all riders and horses. In less-experienced riders, cortisol release was higher on a less-experienced versus an experienced horse but the horses’ cortisol release was not affected by experience of their riders. Heart rate did not differ between groups of horses and was not affected by experience of the rider but was higher in less-experienced versus experienced riders. The HRV decreased in horses and riders and SDRR was lower in less-experienced versus experienced riders. Thus, lower experience of riders appears not to affect physiological stress parameters in their horses during a showjumping course. |
  | Date | 11/2013 |
  | Language | en |
  | Library Catalogue | DOI.org (Crossref) |
  | URL | https://www.cambridge.org/core/product/identifier/S0962728600005583/type/journal\_article |
  | Accessed | 01/07/2025, 12:22:13 |
  | Rights | https://www.cambridge.org/core/terms |
  | Volume | 22 |
  | Pages | 457-465 |
  | Publication | Animal Welfare |
  | DOI | 10.7120/09627286.22.4.457 |
  | Issue | 4 |
  | Journal Abbr | Anim. welf. |
  | ISSN | 0962-7286, 2054-1538 |
  | Date Added | 01/07/2025, 12:22:13 |
  | Modified | 01/07/2025, 12:22:13 |

  ### Attachments

  - PDF
- ## Physiological stress responses and horse rider interactions in horses ridden by male and female riders

  |  |  |
  | --- | --- |
  | Item Type | Journal Article |
  | Author | N. Ille |
  | Author | C. Aurich |
  | Author | R. Erber |
  | Author | M. Wulf |
  | Author | R. Palme |
  | Author | J. Aurich |
  | Author | M. Von Lewinski |
  | Abstract | Traditionally, horse riding has been restricted to men but today equestrian sports are dominated by women. We hypothesised that men and women differ with regard to riding and the response they evoke in their horse. Cortisol and heart rate variability (HRV) were studied in male (n=8) and female riders (n=8) and in horses (n=8) ridden by men and women over a jumping course. Saliva for cortisol analysis was collected, cardiac beat to beat (RR) intervals were recorded and heart rate and HRV variables SDRR (standard deviation of RR interval) and RMSSD (root mean square of successive RR differences) calculated. In another experiment, saddle pressure was compared between male and female riders (n=5 each). Cortisol did not differ between male and female riders and increased in horses (P<0.001) irrespective of the sex of the rider. Heart rate in riders increased from walk to jumping (P<0.001) while HRV decreased (P<0.001) to the same extent in men and women. In horses, heart rate increased (P<0.001) and SDRR and RMSSD decreased during walk and remained low at trot and canter (P<0.001) irrespective of the riders’ sex. In trot (P<0.05) and canter (P<0.01) saddle pressure was slightly lower in female versus male riders. This is due to weight differences and not to a different seat. In conclusion, no fundamental differences existed in the physical effort, stress response and seat between male and female riders and in the response of horses to men and women. |
  | Date | 2014-01-01 |
  | Library Catalogue | Crossref |
  | URL | https://brill.com/view/journals/cep/10/2/article-p131\_131.xml |
  | Accessed | 08/07/2025, 12:34:44 |
  | Volume | 10 |
  | Publisher | Walter de Gruyter GmbH |
  | Pages | 131-138 |
  | Publication | Comparative Exercise Physiology |
  | DOI | 10.3920/cep143001 |
  | Issue | 2 |
  | Journal Abbr | CEP |
  | ISSN | 1755-2540, 1755-2559 |
  | Date Added | 08/07/2025, 12:34:44 |
  | Modified | 08/07/2025, 12:34:44 |

  ### Attachments

  - PDF
- ## Oxytocin and Cortisol Levels in Dog Owners and Their Dogs Are Associated with Behavioral Patterns: An Exploratory Study

  |  |  |
  | --- | --- |
  | Item Type | Journal Article |
  | Author | E. Hydbring-Sandberg |
  | Author | Linda Handlin |
  | Author | Lise-Lotte Gustafson |
  | Author | K. Uvnäs-Moberg |
  | Author | Anne Nilsson |
  | Author | M. Petersson |
  | Date | 2017-10-13 |
  | URL | https://consensus.app/papers/oxytocin-and-cortisol-levels-in-dog-owners-and-their-dogs-hydbring-sandberg-handlin/bcc2b8f5b33c58e0bc8caddd9502c762/ |
  | Volume | 8 |
  | Publication | Frontiers in Psychology |
  | DOI | 10.3389/fpsyg.2017.01796 |
  | Journal Abbr | Frontiers in Psychology |
  | Date Added | 20/06/2025, 10:21:07 |
  | Modified | 20/06/2025, 10:21:07 |

  ### Attachments

  - PDF
- ## Exploring the Dynamics of Canine-Assisted Interactions: A Wearable Approach to Understanding Interspecies Well-Being

  |  |  |
  | --- | --- |
  | Item Type | Journal Article |
  | Author | Timothy R. N. Holder |
  | Author | Colt Nichols |
  | Author | Emily Summers |
  | Author | David L. Roberts |
  | Author | Alper Bozkurt |
  | Date | 2024 |
  | URL | https://www.scopus.com/inward/record.uri?eid=2-s2.0-85213375550&doi=10.3390%2fani14243628&partnerID=40&md5=f5eb4ca70a3271d1bbba5f9c56a68b0c |
  | Extra | Type: Article |
  | Volume | 14 |
  | Publication | Animals |
  | DOI | 10.3390/ani14243628 |
  | Issue | 24 |
  | Date Added | 19/06/2025, 15:40:13 |
  | Modified | 19/06/2025, 15:40:13 |

  ### Notes:

  - Cited by: 1; All Open Access, Gold Open Access

  ### Attachments

  - PDF
- ## Contact-Free Simultaneous Sensing of Human Heart Rate and Canine Breathing Rate for Animal Assisted Interactions

  |  |  |
  | --- | --- |
  | Item Type | Conference Paper |
  | Author | Timothy Holder |
  | Author | Mushfiqur Rahman |
  | Author | Emily Summers |
  | Author | David Roberts |
  | Author | Chau-Wai Wong |
  | Author | Alper Bozkurt |
  | Date | 2022-12-05 |
  | Language | en |
  | Library Catalogue | DOI.org (Crossref) |
  | URL | https://dl.acm.org/doi/10.1145/3565995.3566039 |
  | Accessed | 01/07/2025, 15:33:49 |
  | Place | Newcastle-upon-Tyne United Kingdom |
  | Publisher | ACM |
  | ISBN | 978-1-4503-9830-5 |
  | Pages | 1-10 |
  | Proceedings Title | Proceedings of the Ninth International Conference on Animal-Computer Interaction |
  | Conference Name | ACI'22: Ninth International Conference on Animal-Computer Interaction |
  | DOI | 10.1145/3565995.3566039 |
  | Date Added | 01/07/2025, 15:33:49 |
  | Modified | 01/07/2025, 15:33:49 |

  ### Attachments

  - Submitted Version
- ## Towards a Multimodal Synchronized System for Quantifying Psychophysiological States in Canine Assisted Interactions

  |  |  |
  | --- | --- |
  | Item Type | Conference Paper |
  | Author | Timothy R. N. Holder |
  | Author | Colt Nichols |
  | Author | Emily Summers |
  | Author | David L. Roberts |
  | Author | Alper Bozkurt |
  | Date | 2023-12-04 |
  | Language | en |
  | Library Catalogue | DOI.org (Crossref) |
  | URL | https://dl.acm.org/doi/10.1145/3637882.3637886 |
  | Accessed | 01/07/2025, 17:10:43 |
  | Place | Raleigh NC USA |
  | Publisher | ACM |
  | ISBN | 979-8-4007-1656-0 |
  | Pages | 1-13 |
  | Proceedings Title | The Tenth International Conference on Animal-Computer Interaction |
  | Conference Name | ACI '23: The Tenth International Conference on Animal-Computer Interaction |
  | DOI | 10.1145/3637882.3637886 |
  | Date Added | 01/07/2025, 17:10:43 |
  | Modified | 01/07/2025, 17:10:43 |

  ### Attachments

  - Full Text
- ## Exploring Synchronicity in the Heart Rates of Familiar and Unfamiliar Pairs of Horses and Humans Undertaking an In-Hand Task

  |  |  |
  | --- | --- |
  | Item Type | Journal Article |
  | Author | Jo Hockenhull |
  | Author | Tamsin J. Young |
  | Author | Sarah E. Redgate |
  | Author | Lynda Birke |
  | Date | 2015-09-02 |
  | Language | en |
  | Library Catalogue | DOI.org (Crossref) |
  | URL | https://www.tandfonline.com/doi/full/10.1080/08927936.2015.1052284 |
  | Accessed | 17/06/2025, 18:06:24 |
  | Volume | 28 |
  | Pages | 501-511 |
  | Publication | Anthrozoös |
  | DOI | 10.1080/08927936.2015.1052284 |
  | Issue | 3 |
  | Journal Abbr | Anthrozoös |
  | ISSN | 0892-7936, 1753-0377 |
  | Date Added | 17/06/2025, 18:06:24 |
  | Modified | 17/06/2025, 18:06:24 |

  ### Attachments

  - Accepted Version
- ## Cortisol concentrations in saliva of humans and their dogs during intensive training courses in animal-assisted therapy

  |  |  |
  | --- | --- |
  | Item Type | Journal Article |
  | Author | D. Haubenhofer |
  | Author | Erich Möstl |
  | Author | Sylvia Kirchengast |
  | Date | 2005-01-01 |
  | Volume | 92 |
  | Pages | 66-73 |
  | Publication | Wiener Tierarztliche Monatsschrift |
  | Journal Abbr | Wiener Tierarztliche Monatsschrift |
  | Date Added | 09/07/2025, 10:19:01 |
  | Modified | 09/07/2025, 10:19:01 |

  ### Attachments

  - PDF
- ## 'Dog Handlers' and Dogs' Emotional and Cortisol Secretion Responses Associated with Animal-Aassisted Therapy Sessions

  |  |  |
  | --- | --- |
  | Item Type | Journal Article |
  | Author | Dorit Karla Haubenhofer |
  | Author | Sylvia Kirchengast |
  | Abstract | AbstractThe study investigated 13 dog handlers and 18 companion dogs (Canis familiaris) working as teams in nonhuman animal-assisted service. The handlers described in questionnaires what emotions they chose to associate with their daily life and therapeutic work. They described their emotional condition before and after therapeutic sessions, giving analogous descriptions for their dogs. Handlers collected saliva samples from themselves and their dogs (6 non-therapeutic control days) during 3 months of therapeutic work) to measure cortisol concentrations using an enzyme-immunoassay. Handlers chose different emotions from the questionnaires for themselves and their dogs, differing from the cortisol sampling results. Handlers and dogs had increased cortisol concentrations on therapy days compared to control days. Handlers had significantly higher concentrations immediately before therapeutic sessions. In handlers, cortisol concentrations increased steadily with the duration of sessions; in dogs, with the number of sessions per week. Further study of the effects of recreation periods during therapy work days or of more days scheduled without therapy will help clarify what conditions for delivering animal-assisted service best safeguard the welfare of dog and dog handler teams. |
  | Date | 2007 |
  | Library Catalogue | Crossref |
  | URL | https://brill.com/view/journals/soan/15/2/article-p127\_3.xml |
  | Accessed | 08/07/2025, 12:06:51 |
  | Volume | 15 |
  | Publisher | Walter de Gruyter GmbH |
  | Pages | 127-150 |
  | Publication | Society & Animals |
  | DOI | 10.1163/156853007x187090 |
  | Issue | 2 |
  | Journal Abbr | Soc Animals |
  | ISSN | 1063-1119, 1568-5306 |
  | Date Added | 08/07/2025, 12:06:51 |
  | Modified | 08/07/2025, 12:06:51 |

  ### Attachments

  - PDF
- ## Does stress run through the leash? An examination of stress transmission between owners and dogs during a walk

  |  |  |
  | --- | --- |
  | Item Type | Journal Article |
  | Author | Helen Harvie |
  | Author | Alejandro Rodrigo |
  | Author | Candace Briggs |
  | Author | Shane Thiessen |
  | Author | Debbie M. Kelly |
  | Date | 03/2021 |
  | Language | en |
  | Short Title | Does stress run through the leash? |
  | Library Catalogue | DOI.org (Crossref) |
  | URL | https://link.springer.com/10.1007/s10071-020-01460-6 |
  | Accessed | 27/06/2025, 16:32:56 |
  | Volume | 24 |
  | Pages | 239-250 |
  | Publication | Animal Cognition |
  | DOI | 10.1007/s10071-020-01460-6 |
  | Issue | 2 |
  | Journal Abbr | Anim Cogn |
  | ISSN | 1435-9448, 1435-9456 |
  | Date Added | 27/06/2025, 16:32:56 |
  | Modified | 27/06/2025, 16:32:56 |

  ### Attachments

  - PDF
- ## Short-Term Interaction between Dogs and Their Owners: Effects on Oxytocin, Cortisol, Insulin and Heart Rate—An Exploratory Study

  |  |  |
  | --- | --- |
  | Item Type | Journal Article |
  | Author | Linda Handlin |
  | Author | Eva Hydbring-Sandberg |
  | Author | Anne Nilsson |
  | Author | Mikael Ejdebäck |
  | Author | Anna Jansson |
  | Author | Kerstin Uvnäs-Moberg |
  | Date | 09/2011 |
  | Language | en |
  | Short Title | Short-Term Interaction between Dogs and Their Owners |
  | Library Catalogue | DOI.org (Crossref) |
  | URL | https://www.tandfonline.com/doi/full/10.2752/175303711X13045914865385 |
  | Accessed | 17/06/2025, 17:35:50 |
  | Volume | 24 |
  | Pages | 301-315 |
  | Publication | Anthrozoös |
  | DOI | 10.2752/175303711X13045914865385 |
  | Issue | 3 |
  | Journal Abbr | Anthrozoös |
  | ISSN | 0892-7936, 1753-0377 |
  | Date Added | 17/06/2025, 17:35:50 |
  | Modified | 17/06/2025, 17:35:50 |

  ### Attachments

  - PDF
- ## Associations between the Psychological Characteristics of the Human–Dog Relationship and Oxytocin and Cortisol Levels

  |  |  |
  | --- | --- |
  | Item Type | Journal Article |
  | Author | Linda Handlin |
  | Author | K. Uvnäs-Moberg |
  | Author | E. Hydbring-Sandberg |
  | Author | Mikael Ejdebäck |
  | Author | Anne Nilsson |
  | Date | 2012-06-01 |
  | URL | https://consensus.app/papers/associations-between-the-psychological-characteristics-handlin-uvn%C3%A4s-moberg/682a01ff2b6d5aba8a9fbe2c78893f1d/ |
  | Volume | 25 |
  | Pages | 215-228 |
  | Publication | Anthrozoös |
  | DOI | 10.2752/175303712X13316289505468 |
  | Journal Abbr | Anthrozoös |
  | Date Added | 20/06/2025, 10:21:07 |
  | Modified | 20/06/2025, 10:21:07 |

  ### Attachments

  - PDF
- ## Effects of stroking horses on both humans' and horses' heart rate responses<sup>1</sup>

  |  |  |
  | --- | --- |
  | Item Type | Journal Article |
  | Author | Haruyo Hama |
  | Author | Masao Yogo |
  | Author | Yoshinori Matsuyama |
  | Abstract | The present study examined both human and horse heart rates (HRs) when humans stroked horses for 90 seconds; the subjective arousal levels of the humans were measured by the Tohoku Activation Deactivation Adjective Check List before and after stroking horses. Six male sublects with a positive attitude toward companion animals and 6 male subjects with a negative attitude were selected by their scores on the Pet Attitude Scale, and these two groups, together with a third group, of 6 subjects who were male members of the Doshisha University horse-riding club, participated in this experiment. The HRs of the human subjects during the first 10 seconds immediately after the stroking began were significantly higher than those obtained after that period, but these higher levels gradually returned to baseline levels. This tendency appears more clearly in the negative attitude group. The HRs of the horses increased during the first 20 seconds immediately after the human subjects of the NA group started stroking them, but gradually reduced as the stroking continued. The results of subjective arousal levels suggest a decrease in tension by stroking horses. These results suggest that a certain affectional interaction may exist between humans and companion animals. |
  | Date | 05/1996 |
  | Language | en |
  | Library Catalogue | DOI.org (Crossref) |
  | URL | https://onlinelibrary.wiley.com/doi/10.1111/j.1468-5884.1996.tb00009.x |
  | Accessed | 17/06/2025, 16:48:18 |
  | Rights | http://onlinelibrary.wiley.com/termsAndConditions#vor |
  | Volume | 38 |
  | Pages | 66-73 |
  | Publication | Japanese Psychological Research |
  | DOI | 10.1111/j.1468-5884.1996.tb00009.x |
  | Issue | 2 |
  | Journal Abbr | Jpn Psychol Res |
  | ISSN | 0021-5368, 1468-5884 |
  | Date Added | 17/06/2025, 16:48:18 |
  | Modified | 17/06/2025, 16:48:18 |

  ### Attachments

  - PDF
- ## A Wearable System for the Evaluation of the Human-Horse Interaction: A Preliminary Study

  |  |  |
  | --- | --- |
  | Item Type | Journal Article |
  | Author | Andrea Guidi |
  | Author | Antonio Lanata |
  | Author | Paolo Baragli |
  | Author | Gaetano Valenza |
  | Author | Enzo Scilingo |
  | Abstract | This study reports on a preliminary estimation of the human-horse interaction through the analysis of the heart rate variability (HRV) in both human and animal by using the dynamic time warping (DTW) algorithm. Here, we present a wearable system for HRV monitoring in horses. Specifically, we first present a validation of a wearable electrocardiographic (ECG) monitoring system for horses in terms of comfort and robustness, then we introduce a preliminary objective estimation of the human-horse interaction. The performance of the proposed wearable system for horses was compared with a standard system in terms of movement artifact (MA) percentage. Seven healthy horses were monitored without any movement constraints. As a result, the lower amount of MA% of the wearable system suggests that it could be profitably used for reliable measurement of physiological parameters related to the autonomic nervous system (ANS) activity in horses, such as the HRV. Human-horse interaction estimation was achieved through the analysis of their HRV time series. Specifically, DTW was applied to estimate dynamic coupling between human and horse in a group of fourteen human subjects and one horse. Moreover, a support vector machine (SVM) classifier was able to recognize the three classes of interaction with an accuracy greater than 78%. Preliminary significant results showed the discrimination of three distinct real human-animal interaction levels. These results open the measurement and characterization of the already empirically-proven relationship between human and horse. |
  | Date | 2016-09-26 |
  | Language | en |
  | Short Title | A Wearable System for the Evaluation of the Human-Horse Interaction |
  | Library Catalogue | Crossref |
  | URL | https://www.mdpi.com/2079-9292/5/4/63 |
  | Accessed | 11/07/2025, 13:24:23 |
  | Rights | https://creativecommons.org/licenses/by/4.0/ |
  | Volume | 5 |
  | Publisher | MDPI AG |
  | Pages | 63 |
  | Publication | Electronics |
  | DOI | 10.3390/electronics5040063 |
  | Issue | 4 |
  | ISSN | 2079-9292 |
  | Date Added | 11/07/2025, 13:24:23 |
  | Modified | 11/07/2025, 13:24:23 |

  ### Attachments

  - Full Text
- ## Assessing the Relationship Between Emotional States of Dogs and Their Human Handlers, Using Simultaneous Behavioral and Cardiac Measures

  |  |  |
  | --- | --- |
  | Item Type | Journal Article |
  | Author | Emma K. Grigg |
  | Author | Serene Liu |
  | Author | Denise G. Dempsey |
  | Author | Kylee Wong |
  | Author | Melissa Bain |
  | Author | John J. Sollers |
  | Author | Rani Haddock |
  | Author | Lori R. Kogan |
  | Author | Jennifer A. Barnhard |
  | Author | Ashley A. Tringali |
  | Author | Abigail P. Thigpen |
  | Author | Lynette A. Hart |
  | Date | 2022 |
  | URL | https://www.scopus.com/inward/record.uri?eid=2-s2.0-85134911057&doi=10.3389%2ffvets.2022.897287&partnerID=40&md5=dc1c7db926a688d9248b3adefe433302 |
  | Extra | Type: Article |
  | Volume | 9 |
  | Publication | Frontiers in Veterinary Science |
  | DOI | 10.3389/fvets.2022.897287 |
  | Date Added | 19/06/2025, 15:40:14 |
  | Modified | 19/06/2025, 15:40:14 |

  ### Notes:

  - Cited by: 4

  ### Attachments

  - PDF
- ## Effects of human-animal interaction on salivary and urinary oxytocin in children and dogs

  |  |  |
  | --- | --- |
  | Item Type | Journal Article |
  | Author | Gitanjali E. Gnanadesikan |
  | Author | Katherine M. King |
  | Author | Elizabeth Carranza |
  | Author | Abigail C. Flyer |
  | Author | Gianna Ossello |
  | Author | Paige G. Smith |
  | Author | Netzin G. Steklis |
  | Author | H. Dieter Steklis |
  | Author | C. Sue Carter |
  | Author | Jessica J. Connelly |
  | Author | Melissa Barnett |
  | Author | Nancy Gee |
  | Author | Stacey R. Tecot |
  | Author | Evan L. MacLean |
  | Date | 2024 |
  | URL | https://www.scopus.com/inward/record.uri?eid=2-s2.0-85199938687&doi=10.1016%2fj.psyneuen.2024.107147&partnerID=40&md5=678a7fea07f2a7f3ab186bbb2a1167ab |
  | Extra | Type: Article |
  | Volume | 169 |
  | Publication | Psychoneuroendocrinology |
  | DOI | 10.1016/j.psyneuen.2024.107147 |
  | Date Added | 20/06/2025, 09:37:47 |
  | Modified | 20/06/2025, 09:37:47 |

  ### Notes:

  - Cited by: 2

  ### Attachments

  - PDF
- ## Glucocorticoid response to naturalistic interactions between children and dogs

  |  |  |
  | --- | --- |
  | Item Type | Journal Article |
  | Author | Gitanjali E. Gnanadesikan |
  | Author | Elizabeth Carranza |
  | Author | Katherine M. King |
  | Author | Abigail C. Flyer |
  | Author | Gianna Ossello |
  | Author | Paige G. Smith |
  | Author | Netzin G. Steklis |
  | Author | H. Dieter Steklis |
  | Author | Jessica J. Connelly |
  | Author | Melissa Barnett |
  | Author | Nancy Gee |
  | Author | Stacey Tecot |
  | Author | Evan L. MacLean |
  | Date | 05/2024 |
  | Language | en |
  | Library Catalogue | Crossref |
  | URL | https://linkinghub.elsevier.com/retrieve/pii/S0018506X24000485 |
  | Accessed | 08/07/2025, 12:04:07 |
  | Rights | https://www.elsevier.com/tdm/userlicense/1.0/ |
  | Volume | 161 |
  | Publisher | Elsevier BV |
  | Pages | 105523 |
  | Publication | Hormones and Behavior |
  | DOI | 10.1016/j.yhbeh.2024.105523 |
  | ISSN | 0018-506X |
  | Date Added | 08/07/2025, 12:04:07 |
  | Modified | 08/07/2025, 12:04:07 |

  ### Attachments

  - PDF
- ## The horse-human heart connection: Results of studies using heart rate variability

  |  |  |
  | --- | --- |
  | Item Type | Journal Article |
  | Author | Ellen Kaye Gehrke |
  | Date | 2010 |
  | Short Title | The horse-human heart connection |
  | Library Catalogue | Google Scholar |
  | URL | http://www.mindfulhorsemindfulleader.com/wp-content/uploads/2013/01/Research\_The-Horse-Human-Heart-Connection-1.pdf |
  | Accessed | 01/07/2025, 13:06:35 |
  | Pages | 20–23 |
  | Publication | NAHRA’s Strides, Spring |
  | Date Added | 01/07/2025, 13:06:35 |
  | Modified | 01/07/2025, 13:06:35 |

  ### Attachments

  - Available Version (via Google Scholar)
- ## Physiology of human-horse interactions during substance withdrawal within psychotherapy participants

  |  |  |
  | --- | --- |
  | Item Type | Journal Article |
  | Author | M.M. Friend |
  | Author | M.C. Nicodemus |
  | Author | C.A. Cavinder |
  | Author | C.O. Lemley |
  | Author | P. Prince |
  | Author | K. Holtcamp |
  | Author | R.M. Swanson |
  | Abstract | AbstractPsychotherapy incorporating equine interaction (PIE) is emerging as an effective treatment for substance use disorder (SUD); however, research concerning physiological impacts of PIE during substance withdrawal is lacking. This study investigated impacts of PIE on salivary cortisol concentrations and heart rates in SUD patients during withdrawal. Heart rate and cortisol concentrations were also measured in horses to investigate potential human-horse coupling during PIE. Saliva samples and heart rates were collected from SUD patients (n = 18) and their therapy horses (n = 4) prior to the introduction of the horse and following equine interaction within a residential psychotherapy program during the substance withdrawal period. Without the presence of the horse, the equine environment during the first week of withdrawal produced lower () cortisol and heart rate measures than found in the equine interaction for the SUD patients. Human heart rates, however, decreased () in the second week in response to the equine interaction. A strong negative correlation (r = −0.9, ) was found within the changes in human and horse cortisol concentrations during week two as human cortisol concentrations decreased while horse cortisol concentrations increased. Results indicate equine interaction during psychotherapy is more effective in the second week than the first at mitigating stress for withdrawing residential SUD treatment program patients and the equid environment, even without the presence of a horse, can positively impact stress parameters in withdrawing SUD patients during the first week of treatment. |
  | Date | 2023-10-31 |
  | Library Catalogue | Crossref |
  | URL | https://brill.com/view/journals/cep/20/1/article-p55\_6.xml |
  | Accessed | 09/07/2025, 12:31:15 |
  | Volume | 20 |
  | Publisher | Walter de Gruyter GmbH |
  | Pages | 55-68 |
  | Publication | Comparative Exercise Physiology |
  | DOI | 10.1163/17552559-20230023 |
  | Issue | 1 |
  | Journal Abbr | Comp. Exerc. |
  | ISSN | 1755-2540, 1755-2559 |
  | Date Added | 09/07/2025, 12:31:15 |
  | Modified | 09/07/2025, 12:31:15 |

  ### Attachments

  - PDF
- ## A System for Assessment of Canine-Human Interaction during Animal-Assisted Therapies

  |  |  |
  | --- | --- |
  | Item Type | Conference Paper |
  | Author | Marc Foster |
  | Author | Eric Beppler |
  | Author | Timothy Holder |
  | Author | James Dieffenderfer |
  | Author | Patrick Erb |
  | Author | Kristy Everette |
  | Author | Margaret Gruen |
  | Author | Tamara Somers |
  | Author | Tom Evans |
  | Author | Michael Daniele |
  | Author | David L. Roberts |
  | Author | Alper Bozkurt |
  | Date | 7/2018 |
  | Library Catalogue | DOI.org (Crossref) |
  | URL | https://ieeexplore.ieee.org/document/8513384/ |
  | Accessed | 01/07/2025, 16:33:55 |
  | Place | Honolulu, HI |
  | Publisher | IEEE |
  | ISBN | 978-1-5386-3646-6 |
  | Pages | 4347-4350 |
  | Proceedings Title | 2018 40th Annual International Conference of the IEEE Engineering in Medicine and Biology Society (EMBC) |
  | Conference Name | 2018 40th Annual International Conference of the IEEE Engineering in Medicine and Biology Society (EMBC) |
  | DOI | 10.1109/EMBC.2018.8513384 |
  | Date Added | 01/07/2025, 16:33:55 |
  | Modified | 01/07/2025, 16:33:55 |

  ### Attachments

  - PDF
- ## Physiological and behavioral reactivity to stress in thunderstorm-phobic dogs and their caregivers

  |  |  |
  | --- | --- |
  | Item Type | Journal Article |
  | Author | Nancy A. Dreschel |
  | Author | Douglas A. Granger |
  | Date | 12/2005 |
  | Language | en |
  | Library Catalogue | Crossref |
  | URL | https://linkinghub.elsevier.com/retrieve/pii/S0168159105001152 |
  | Accessed | 08/07/2025, 16:51:28 |
  | Rights | https://www.elsevier.com/tdm/userlicense/1.0/ |
  | Volume | 95 |
  | Publisher | Elsevier BV |
  | Pages | 153-168 |
  | Publication | Applied Animal Behaviour Science |
  | DOI | 10.1016/j.applanim.2005.04.009 |
  | Issue | 3-4 |
  | ISSN | 0168-1591 |
  | Date Added | 08/07/2025, 16:51:28 |
  | Modified | 08/07/2025, 16:51:28 |

  ### Attachments

  - PDF
- ## The Bond Between a Horse and a Human

  |  |  |
  | --- | --- |
  | Item Type | Journal Article |
  | Author | Debbie Crews |
  | Abstract | The bond that exists between a horse and human was examined using EEG from the horse and human simultaneously. Three volunteers ranging from novice to elite horse experience participated with an unfamiliar horse. The elite participant was also recorded with her own horse. A dose-response effect was tested using 6 conditions requiring increasing interaction between the horse and human (baseline – apart, standing together, petting, grooming, sitting, and riding). |
  | Date | 2009-07-24 |
  | Language | en |
  | Library Catalogue | DOI.org (Crossref) |
  | URL | https://www.nature.com/articles/npre.2009.3454.1 |
  | Accessed | 24/06/2025, 09:07:15 |
  | Publication | Nature Precedings |
  | DOI | 10.1038/npre.2009.3454.1 |
  | Journal Abbr | Nat Prec |
  | ISSN | 1756-0357 |
  | Date Added | 24/06/2025, 09:07:15 |
  | Modified | 24/06/2025, 09:07:15 |

  ### Attachments

  - PDF
- ## Therapy Dogs' and Handlers' Behavior and Salivary Cortisol During Initial Visits in a Complex Medical Institution: A Pilot Study

  |  |  |
  | --- | --- |
  | Item Type | Journal Article |
  | Author | Stephanie D. Clark |
  | Author | Jessica M. Smidt |
  | Author | Brent A. Bauer |
  | Date | 2020-11-13 |
  | Short Title | Therapy Dogs' and Handlers' Behavior and Salivary Cortisol During Initial Visits in a Complex Medical Institution |
  | Library Catalogue | DOI.org (Crossref) |
  | URL | https://www.frontiersin.org/articles/10.3389/fvets.2020.564201/full |
  | Accessed | 01/07/2025, 16:45:22 |
  | Volume | 7 |
  | Pages | 564201 |
  | Publication | Frontiers in Veterinary Science |
  | DOI | 10.3389/fvets.2020.564201 |
  | Journal Abbr | Front. Vet. Sci. |
  | ISSN | 2297-1769 |
  | Date Added | 01/07/2025, 16:45:22 |
  | Modified | 01/07/2025, 16:45:22 |

  ### Attachments

  - Full Text
- ## Unveiling directional physiological coupling in human-horse interactions

  |  |  |
  | --- | --- |
  | Item Type | Journal Article |
  | Author | Alejandro Luis Callara |
  | Author | Chiara Scopa |
  | Author | Laura Contalbrigo |
  | Author | Antonio Lanatà |
  | Author | Enzo Pasquale Scilingo |
  | Author | Paolo Baragli |
  | Author | Alberto Greco |
  | Date | 09/2024 |
  | Language | en |
  | Library Catalogue | DOI.org (Crossref) |
  | URL | https://linkinghub.elsevier.com/retrieve/pii/S2589004224020820 |
  | Accessed | 20/06/2025, 10:02:13 |
  | Volume | 27 |
  | Pages | 110857 |
  | Publication | iScience |
  | DOI | 10.1016/j.isci.2024.110857 |
  | Issue | 9 |
  | Journal Abbr | iScience |
  | ISSN | 25890042 |
  | Date Added | 20/06/2025, 10:02:13 |
  | Modified | 20/06/2025, 10:02:13 |

  ### Attachments

  - Full Text
- ## Empathy or Apathy? Investigating the influence of owner stress on canine stress in a novel environment

  |  |  |
  | --- | --- |
  | Item Type | Journal Article |
  | Author | Aoife Byrne |
  | Author | Gareth Arnott |
  | Date | 10/2024 |
  | Language | en |
  | Short Title | Empathy or Apathy? |
  | Library Catalogue | DOI.org (Crossref) |
  | URL | https://linkinghub.elsevier.com/retrieve/pii/S016815912400251X |
  | Accessed | 01/07/2025, 16:43:41 |
  | Volume | 279 |
  | Pages | 106403 |
  | Publication | Applied Animal Behaviour Science |
  | DOI | 10.1016/j.applanim.2024.106403 |
  | Journal Abbr | Applied Animal Behaviour Science |
  | ISSN | 01681591 |
  | Date Added | 01/07/2025, 16:43:41 |
  | Modified | 01/07/2025, 16:43:41 |

  ### Attachments

  - PDF
- ## Evidence for a synchronization of hormonal states between humans and dogs during competition

  |  |  |
  | --- | --- |
  | Item Type | Journal Article |
  | Author | Alicia Phillips Buttner |
  | Author | Breanna Thompson |
  | Author | Rosemary Strasser |
  | Author | Jonathan Santo |
  | Date | 08/2015 |
  | Language | en |
  | Library Catalogue | DOI.org (Crossref) |
  | URL | https://linkinghub.elsevier.com/retrieve/pii/S003193841500205X |
  | Accessed | 01/07/2025, 12:48:46 |
  | Volume | 147 |
  | Pages | 54-62 |
  | Publication | Physiology & Behavior |
  | DOI | 10.1016/j.physbeh.2015.04.010 |
  | Journal Abbr | Physiology & Behavior |
  | ISSN | 00319384 |
  | Date Added | 01/07/2025, 12:48:46 |
  | Modified | 01/07/2025, 12:48:46 |

  ### Attachments

  - PDF
- ## Effects of Equine Interaction on Mutual Autonomic Nervous System Responses and Interoception in a Learning Program for Older Adults

  |  |  |
  | --- | --- |
  | Item Type | Journal Article |
  | Author | Ann L Baldwin |
  | Author | Lisa Walters |
  | Author | Barbara K Rector |
  | Author | Ann C Alden |
  | Abstract | Equine-assisted learning (EAL) may improve the health of older adults, but scientific data are sparse. This study investigated whether people aged 55 and older show increased heart rate variability (HRV) during EAL and awareness of bodily sensations that are overall pleasant. Subjects (n = 24) participated in mindful grooming during which they slowed their breathing and brushed a horse while noticing sensations in their body and watching the horse’s reactions. The subject’s and horse’s HRV were recorded simultaneously before, during, and after mindful grooming. For control, the same subjects performed mindful grooming with a plush simulation horse. During exit interviews, participants described their sensations. Words and gestures were categorized as positive, neutral, or negative. During mindful grooming, human heart rate and HRV (standard deviation of interbeat interval, SDRR) increased compared to baseline (paired t-test, t = –4.228, p < 0.001; t = –3.814, p = 0.001), as did the percent very low frequency (%VLF) component of HRV (t = –4.274, p < 0.001). Equine HRV values remained in the normal range, mostly VLF. In 10 cases, during mindful grooming, horse and human HRVs showed matching VLF frequencies. Grooming the simulation horse significantly elevated SDRR but did not alter %VLF. Exit interviews revealed significantly more positive gestures (t = –3.814, p = 0.031) and fewer negative gestures (Wilcoxon signed-rank test, Z-statistic = –2.12, p = 0.036, p < 0.05) when participants spoke about the real horse compared to the simulation. These findings demonstrate that during mindful grooming people aged 55 and older benefit by experiencing increased HRV, heightened awareness of pleasant bodily sensations, and often some synchronization of their HRV frequency spectrum with that of their horse, possibly reflecting emotional bonding. |
  | Date | 2023 |
  | Language | en |
  | Library Catalogue | Zotero |
  | Volume | 6 |
  | Issue | 1 |
  | Date Added | 01/07/2025, 15:37:46 |
  | Modified | 01/07/2025, 15:37:46 |

  ### Attachments

  - PDF
- ## Physiological and Behavioral Benefits for People and Horses during Guided Interactions at an Assisted Living Residence

  |  |  |
  | --- | --- |
  | Item Type | Journal Article |
  | Author | Ann Baldwin |
  | Author | Barbara Rector |
  | Author | Ann Alden |
  | Abstract | Assisted living is a fast-growing living option for seniors who require residence-based activities for maintaining mental and physical health. Guided equine interactions may benefit seniors, so an on-site equine program was started at Hacienda at the River senior living community. For research purposes, twenty-four residents and associates, aged fifty-five or over, consented to physiological measurements before, during and after four guided sessions of stroking one of three horses for 10 min over 4–6 weeks. Heart rate variability (HRV) was measured simultaneously in humans and horses during interactions. We hypothesized that human heart rate (HR) and HRV would increase during stroking and HRV power would shift toward the very low frequency (VLF) range common in horses, indicative of healthy function. During stroking, human HR increased (p < 0.05) but HRV (SDRR) and %VLF of HRV power did not change. Diastolic blood pressure (DBP), an exploratory measure, significantly increased after stroking, consistent with arousal. Two horses showed no significant changes in HR or HRV, but one relaxed. Sixteen horse–human pairs demonstrated synchronized HRV peak frequencies during sessions, suggestive of social connection. Participants used more positive than negative words describing their experience during exit interviews (p < 0.05). These data show that horses animate seniors without causing emotional stress and provide opportunities for social bonding. |
  | Date | 2021-09-23 |
  | Language | en |
  | Library Catalogue | DOI.org (Crossref) |
  | URL | https://www.mdpi.com/2076-328X/11/10/129 |
  | Accessed | 01/07/2025, 18:00:17 |
  | Rights | https://creativecommons.org/licenses/by/4.0/ |
  | Volume | 11 |
  | Pages | 129 |
  | Publication | Behavioral Sciences |
  | DOI | 10.3390/bs11100129 |
  | Issue | 10 |
  | Journal Abbr | Behavioral Sciences |
  | ISSN | 2076-328X |
  | Date Added | 01/07/2025, 18:00:17 |
  | Modified | 01/07/2025, 18:00:17 |

  ### Attachments

  - Full Text
- ## Pilot Study of the Influence of Equine Assisted Therapy on Physiological and Behavioral Parameters Related to Welfare of Horses and Patients

  |  |  |
  | --- | --- |
  | Item Type | Journal Article |
  | Author | María Dolores Ayala |
  | Author | Andrea Carrillo |
  | Author | Pilar Iniesta |
  | Author | Pedro Ferrer |
  | Abstract | Different welfare indicators were studied in three patients with psychomotor alterations and in two horses throughout 9–10 equine assisted therapy sessions in each patient. In horses, heart and respiratory rates, blood pressure, temperature and behavioral signs were studied. In patients, heart rate, oxygen saturation, temperature, sleep quality, psychomotor and emotional parameters were analyzed. Data collection was recorded in the anticipatory phase (15 min before the start of the session), two interaction phases (after 30 min of horse-patient interaction on the ground and on horseback, respectively) and the recovery phase (15 min after the end of the session). During the anticipatory phase, most of physiological parameters of patients and horses and the stress behavioral signs of horses increased, followed by a relaxing phase during the horse-patient interaction on the ground. In horse-patient riding phase the heart and respiratory rates of the horses again increased. These results showed that the horses did not seem to suffer stress attributable to the therapy sessions, but only an increase in their parameters associated with activity and external stimuli. The patients improved their gross and fine motor skills, their cognitive and perceptual-sensitive parameters and it led to an improvement in the life quality of their families. |
  | Date | 2021-12-10 |
  | Language | en |
  | Library Catalogue | Crossref |
  | URL | https://www.mdpi.com/2076-2615/11/12/3527 |
  | Accessed | 09/07/2025, 12:06:51 |
  | Rights | https://creativecommons.org/licenses/by/4.0/ |
  | Volume | 11 |
  | Publisher | MDPI AG |
  | Pages | 3527 |
  | Publication | Animals |
  | DOI | 10.3390/ani11123527 |
  | Issue | 12 |
  | ISSN | 2076-2615 |
  | Date Added | 09/07/2025, 12:06:51 |
  | Modified | 09/07/2025, 12:06:51 |

  ### Attachments

  - Full Text
- ## Hormonal and Neurological Aspects of Dog Walking for Dog Owners and Pet Dogs

  |  |  |
  | --- | --- |
  | Item Type | Journal Article |
  | Author | Junko Akiyama |
  | Author | Mitsuaki Ohta |
  | Abstract | The hormone oxytocin is involved in various aspects of the relationship between humans and animals. Dog walking is a common activity for dog owners and their dogs. The walk, of course, should be good for the health of the dog as well as its owner. In Experiment I, we assessed whether salivary oxytocin and cortisol in dog owners changed because of walking their dogs. Ten owners walked with their dogs and walked alone. Similar to other previous research, walking with a dog did not significantly change oxytocin and cortisol. Therefore, in Experiment II, we investigated the effect of dog walking on brain noradrenergic and GABAergic neural activity, as indicated by salivary MHPG and GABA, in 14 dog owners. Walking with a dog reduced salivary MHPG compared to walking alone, and MHPG was correlated negatively with GABA. Thus, dog walking activated GABAergic nerves in the brain and suppressed noradrenergic nerves, effectively relieving stress. |
  | Date | 2021-09-18 |
  | Language | en |
  | Library Catalogue | Crossref |
  | URL | https://www.mdpi.com/2076-2615/11/9/2732 |
  | Accessed | 09/07/2025, 10:26:41 |
  | Rights | https://creativecommons.org/licenses/by/4.0/ |
  | Volume | 11 |
  | Publisher | MDPI AG |
  | Pages | 2732 |
  | Publication | Animals |
  | DOI | 10.3390/ani11092732 |
  | Issue | 9 |
  | ISSN | 2076-2615 |
  | Date Added | 09/07/2025, 10:26:41 |
  | Modified | 09/07/2025, 10:26:41 |

  ### Attachments

  - Full Text
