# Supplementary material for: Evidence of Physiological Comodulation During Human–Animal Interaction: A Systematic Review
Source: Ann N Y Acad Sci. 2026 Jun 4;1560(1):e70299. doi: 10.1111/nyas.70299 (PMC13238372; doi:10.1111/nyas.70299)
Supplement: Supplementary file 2 — Supplementary Materials: Supp2‐Zotero‐Collection.zip [file NYAS-1560-0-s002.zip › Supp2_Zotero_Collection/text screened/Citing Papers 1.htm]

Zotero Report


- ## Heart rate variability responses of horses and veterans with post-traumatic stress disorder to ground-based adaptive horsemanship lessons: a pilot study

  |  |  |
  | --- | --- |
  | Item Type | Journal Article |
  | Author | Ellen M Rankins |
  | Author | Boluwatife E Faremi |
  | Author | Kyle Hartmann |
  | Author | Andrea Quinn |
  | Author | Hugo F Posada-Quintero |
  | Author | Kenneth H McKeever |
  | Author | Karyn Malinowski |
  | Abstract | Abstract Heart rate variability (HRV) can be measured as an indicator of autonomic nervous system (ANS) balance and thus, stress and affective arousal. Mixed results have been reported in the limited literature addressing the effects of equine-assisted services (EAS) on HRV in the human and horse participants. The aims of the present study were to determine the effects of ground-based adaptive horsemanship (AH) lessons on veterans’ and horses’ HRV during weekly lessons as well as veterans’ resting HRV outside of lessons. Veterans with post-traumatic stress disorder (PTSD) were randomly assigned to 8 wk of AH or control (CON, no changes in treatments or activities) conditions. Horses were assigned to AH or control (CON, stall in arena) conditions based on previous experience (EAS or recreational riding). Electrocardiogram traces from the veteran and AH and CON horses were recorded during the 30-min lessons. Traces were recorded during resting conditions before (PRE) and after (POST) the 8-wk period from AH and CON veterans and healthy, non-veterans. Heart rate (HR), root mean square of successive differences (RMSSD) and low frequency to high frequency ratios (LF/HF) were calculated after identification of peaks with the Pan-Tompkins algorithm and manual correction during 5-min epochs. Data were analyzed with repeated measures, mixed model ANOVAs (SAS v9.4). HR was lowest (p ≤ 0.0323) in the horses and veterans during the first 10 min of the lessons. Veterans’ RMSSD was higher (p ≤ 0.0496) in weeks 4 and 6 than week 2. LF/HF was greater in veterans with PTSD than healthy, non-veterans across PRE and POST time points. Increased HR later in the lessons is likely a result of increased movement in the horses and veterans. An interval of decreased stress and increased resiliency might be present in veterans participating in multi-day AH sessions. Increased sympathetic arousal measured via LF/HF was not mitigated by 8 wk of AH. Horses appeared unstressed by the interactions as no changes in HRV were observed. |
  | Date | 2025-01-07 |
  | Language | en |
  | Short Title | Heart rate variability responses of horses and veterans with post-traumatic stress disorder to ground-based adaptive horsemanship lessons |
  | Library Catalogue | DOI.org (Crossref) |
  | URL | https://academic.oup.com/tas/article/doi/10.1093/tas/txaf019/8010614 |
  | Accessed | 27/06/2025, 17:26:58 |
  | Rights | https://creativecommons.org/licenses/by-nc-nd/4.0/ |
  | Volume | 9 |
  | Pages | txaf019 |
  | Publication | Translational Animal Science |
  | DOI | 10.1093/tas/txaf019 |
  | ISSN | 2573-2102 |
  | Date Added | 27/06/2025, 17:26:58 |
  | Modified | 27/06/2025, 17:26:58 |

  ### Attachments

  - Full Text PDF
- ## Heart rate and salivary cortisol as indicators of arousal and synchrony in clients, therapy horses and therapist in equine-assisted therapy

  |  |  |
  | --- | --- |
  | Item Type | Journal Article |
  | Author | A. Naber |
  | Author | L. Kreuzer |
  | Author | R. Zink |
  | Author | E. Millesi |
  | Author | R. Palme |
  | Author | K. Hediger |
  | Author | L.M. Glenk |
  | Date | 05/2025 |
  | Language | en |
  | Library Catalogue | DOI.org (Crossref) |
  | URL | https://linkinghub.elsevier.com/retrieve/pii/S1744388125000027 |
  | Accessed | 27/06/2025, 17:18:09 |
  | Volume | 59 |
  | Pages | 101937 |
  | Publication | Complementary Therapies in Clinical Practice |
  | DOI | 10.1016/j.ctcp.2025.101937 |
  | Journal Abbr | Complementary Therapies in Clinical Practice |
  | ISSN | 17443881 |
  | Date Added | 27/06/2025, 17:18:09 |
  | Modified | 27/06/2025, 17:18:09 |

  ### Attachments

  - PDF
- ## The Effects of Human–Horse Interactions on Oxytocin and Cortisol Levels in Humans and Horses

  |  |  |
  | --- | --- |
  | Item Type | Journal Article |
  | Author | Youngwook Jung |
  | Author | Minjung Yoon |
  | Abstract | Therapeutic programs involving human–horse interactions are gaining popularity as a means of enhancing human well-being. Understanding the physiological responses of both humans and horses during these interactions is essential for evaluating the effectiveness of such programs. This study examined the effects of specific interactive activities on both humans and horses by monitoring changes in oxytocin and cortisol levels. Six participants and six horses took part in the study. The participants engaged in three distinct activities, each lasting 15 min: (1) resting alone without the horse (resting), (2) standing near the horse without physical contact (standing), and (3) gently rubbing the horse’s neck and withers (rubbing). Saliva samples from the participants and blood samples from the horses were collected at three time points for each activity: T0 (before the activity), T1 (at the end of the activity), and T2 (15 min after the activity ended). The results indicated that oxytocin levels significantly increased in horses at T2 following both the standing and rubbing activities, while cortisol levels remained unchanged in both humans and horses across all activities. These findings suggest that human–horse interactions, particularly standing and rubbing, may foster social bonding in horses without eliciting a stress response in either species. |
  | Date | 2025-03-21 |
  | Language | en |
  | Library Catalogue | DOI.org (Crossref) |
  | URL | https://www.mdpi.com/2076-2615/15/7/905 |
  | Accessed | 27/06/2025, 17:19:11 |
  | Rights | https://creativecommons.org/licenses/by/4.0/ |
  | Volume | 15 |
  | Pages | 905 |
  | Publication | Animals |
  | DOI | 10.3390/ani15070905 |
  | Issue | 7 |
  | Journal Abbr | Animals |
  | ISSN | 2076-2615 |
  | Date Added | 27/06/2025, 17:19:11 |
  | Modified | 27/06/2025, 17:19:11 |

  ### Attachments

  - Full Text PDF
- ## Contact-Free Simultaneous Sensing of Human Heart Rate and Canine Breathing Rate for Animal Assisted Interactions

  |  |  |
  | --- | --- |
  | Item Type | Conference Paper |
  | Author | Timothy Holder |
  | Author | Mushfiqur Rahman |
  | Author | Emily Summers |
  | Author | David Roberts |
  | Author | Chau-Wai Wong |
  | Author | Alper Bozkurt |
  | Date | 2022-12-05 |
  | Language | en |
  | Library Catalogue | DOI.org (Crossref) |
  | URL | https://dl.acm.org/doi/10.1145/3565995.3566039 |
  | Accessed | 01/07/2025, 15:33:49 |
  | Place | Newcastle-upon-Tyne United Kingdom |
  | Publisher | ACM |
  | ISBN | 978-1-4503-9830-5 |
  | Pages | 1-10 |
  | Proceedings Title | Proceedings of the Ninth International Conference on Animal-Computer Interaction |
  | Conference Name | ACI'22: Ninth International Conference on Animal-Computer Interaction |
  | DOI | 10.1145/3565995.3566039 |
  | Date Added | 01/07/2025, 15:33:49 |
  | Modified | 01/07/2025, 15:33:49 |

  ### Attachments

  - Submitted Version
