# Supplementary material for: Evidence of Physiological Comodulation During Human–Animal Interaction: A Systematic Review
Source: Ann N Y Acad Sci. 2026 Jun 4;1560(1):e70299. doi: 10.1111/nyas.70299 (PMC13238372; doi:10.1111/nyas.70299)
Supplement: Supplementary file 2 — Supplementary Materials: Supp2‐Zotero‐Collection.zip [file NYAS-1560-0-s002.zip › Supp2_Zotero_Collection/text screened/Citing Papers 2.htm]

Zotero Report


- ## Are Hair Cortisol Levels of Humans, Cats, and Dogs from the Same Household Correlated?

  |  |  |
  | --- | --- |
  | Item Type | Journal Article |
  | Author | Justyna Wojtaś |
  | Author | Aleksandra Garbiec |
  | Author | Mirosław Karpiński |
  | Author | Patrycja Skowronek |
  | Author | Aneta Strachecka |
  | Abstract | Human–animal interactions and the emotional relationship of the owner with the pet are the subjects of many scientific studies and the constant interest of not only scientists but also pet owners. The aim of this study was to determine and compare the hair cortisol levels of dogs, cats, and their owners living in the same household. The owners were asked to complete a questionnaire concerning the frequency of their interactions with pets and emotional relationship with each of their cats and each of their dogs. The study involved 25 women who owned at least one dog and at least one cat. In total, 45 dogs and 55 cats from 25 households participated in the study. The average level of hair cortisol of the owners was 4.62 ng/mL, of the dogs 0.26 ng/mL, and in the hair of cats 0.45 ng/mL. There was no significant correlation between the hair cortisol level of the owner and dog or the owner and the cat and between dogs and cats living together. A significant positive correlation was observed between the hair cortisol level in the owner and the pet, for dogs in which the owner performs grooming treatments once a week and for cats which are never kissed. Although our study did not find many significant correlations, studies using other stress markers might have yielded different results. |
  | Date | 2022-06-06 |
  | Language | en |
  | Library Catalogue | Crossref |
  | URL | https://www.mdpi.com/2076-2615/12/11/1472 |
  | Accessed | 10/07/2025, 09:29:49 |
  | Rights | https://creativecommons.org/licenses/by/4.0/ |
  | Volume | 12 |
  | Publisher | MDPI AG |
  | Pages | 1472 |
  | Publication | Animals |
  | DOI | 10.3390/ani12111472 |
  | Issue | 11 |
  | ISSN | 2076-2615 |
  | Date Added | 10/07/2025, 09:29:49 |
  | Modified | 10/07/2025, 09:29:49 |

  ### Attachments

  - Full Text
- ## The Effect of Victory and Defeat on the Correlations of Stress Parameters Between the Horse and Rider in Kök‐Börü Equestrian Teams

  |  |  |
  | --- | --- |
  | Item Type | Journal Article |
  | Author | Ali Rişvanli |
  | Author | İsmail Şen |
  | Author | Kanat Canuzakov |
  | Author | Askarbek Tulobayev |
  | Author | Abuzer Taş |
  | Author | Ruslan Salykov |
  | Author | Nezahat Ceylan |
  | Author | Ünal Türkçapar |
  | Author | Ulanbek Alimov |
  | Author | Arina Kazakbayeva |
  | Author | Ayday Cunuşova |
  | Author | Nur Abdimnap Uulu |
  | Author | Burak Fatih Yuksel |
  | Author | Mert Turanli |
  | Author | Muhammed Uz |
  | Author | Metin Bayraktar |
  | Author | Nuriddin Ruzikulov |
  | Abstract | ABSTRACTThe presented study outlines a research plan aimed at determining the effects of winning and losing situations on the relationship levels between the rider and horse's stress, metabolic, and physiological parameters in Kök‐Börü, a traditional equestrian team game. For this purpose, blood samples were collected from both the horses and riders of four different teams participating in two different Kök‐Börü games before and after the games. Cortisol, ACTH, beta‐endorphin, epinephrine, norepinephrine, T3 and T4 analyses were performed on the collected blood samples using species‐specific commercial ELISA kits. Additionally, biochemical and haematological parameters in the same blood samples were tested using an autoanalyser. Based on the obtained data, it was found that there were both positive and negative correlations between most biochemical and haematological parameters of the winning teams' horses and riders before and after the game. However, when examining the correlations between the hormonal parameters of the winning teams' horses and riders before the game, only a negative correlation was found between ACTH and T4 (‐0.529, p < 0.05), and no positive correlation was identified among any hormonal parameters. In conclusion, it was interpreted that in the equestrian team sport of Kök‐Börü, there are significant changes in the hormonal parameters, especially before and after the game, between the horses and riders of the losing teams. Furthermore, it was concluded that winning and losing situations in Kök‐Börü games did not have a significant impact on the correlations between haematological and biochemical parameters before and after the game for both the horses and riders. |
  | Date | 05/2025 |
  | Language | en |
  | Library Catalogue | Crossref |
  | URL | https://onlinelibrary.wiley.com/doi/10.1002/vms3.70356 |
  | Accessed | 09/07/2025, 15:23:27 |
  | Rights | http://creativecommons.org/licenses/by/4.0/ |
  | Volume | 11 |
  | Publisher | Wiley |
  | Publication | Veterinary Medicine and Science |
  | DOI | 10.1002/vms3.70356 |
  | Issue | 3 |
  | Journal Abbr | Veterinary Medicine &amp; Sci |
  | ISSN | 2053-1095, 2053-1095 |
  | Date Added | 09/07/2025, 15:23:27 |
  | Modified | 09/07/2025, 15:23:27 |

  ### Attachments

  - PDF
