# Supplementary material for: Evidence of Physiological Comodulation During Human–Animal Interaction: A Systematic Review
Source: Ann N Y Acad Sci. 2026 Jun 4;1560(1):e70299. doi: 10.1111/nyas.70299 (PMC13238372; doi:10.1111/nyas.70299)
Supplement: Supplementary file 2 — Supplementary Materials: Supp2‐Zotero‐Collection.zip [file NYAS-1560-0-s002.zip › Supp2_Zotero_Collection/text screened/Citing Papers 3.htm]

Zotero Report


- ## Dog’s breath rhythm was drawn into owner’s breath rhythm

  |  |  |
  | --- | --- |
  | Item Type | Journal Article |
  | Author | Kensaku Nomoto |
  | Author | Tomoki Hashimoto |
  | Author | Miho Nagasawa |
  | Author | Takefumi Kikusui |
  | Date | 2024-07-17 |
  | Language | en |
  | Library Catalogue | Crossref |
  | URL | https://www.tandfonline.com/doi/full/10.1080/01691864.2024.2369795 |
  | Accessed | 10/07/2025, 13:53:08 |
  | Volume | 38 |
  | Publisher | Informa UK Limited |
  | Pages | 926-933 |
  | Publication | Advanced Robotics |
  | DOI | 10.1080/01691864.2024.2369795 |
  | Issue | 14 |
  | ISSN | 0169-1864, 1568-5535 |
  | Date Added | 10/07/2025, 13:53:08 |
  | Modified | 10/07/2025, 13:53:08 |

  ### Attachments

  - PDF
