# Supplementary material for: Evidence of Physiological Comodulation During Human–Animal Interaction: A Systematic Review
Source: Ann N Y Acad Sci. 2026 Jun 4;1560(1):e70299. doi: 10.1111/nyas.70299 (PMC13238372; doi:10.1111/nyas.70299)
Supplement: Supplementary file 2 — Supplementary Materials: Supp2‐Zotero‐Collection.zip [file NYAS-1560-0-s002.zip › Supp2_Zotero_Collection/text screened/Consensus.htm]

Zotero Report


- ## Noncontact Electrophysiology Monitoring Systems for Assessment of Canine-Human Interactions

  |  |  |
  | --- | --- |
  | Item Type | Journal Article |
  | Author | Aakash Patel |
  | Author | Marc Foster |
  | Author | T. Torfs |
  | Author | P. Ahmmed |
  | Author | I. Castro |
  | Author | Timothy Holder |
  | Author | A. Bozkurt |
  | Date | 2021-10-31 |
  | URL | https://consensus.app/papers/noncontact-electrophysiology-monitoring-systems-for-patel-foster/0eeed0f0fd9750729fde69f8f3929af0/ |
  | Pages | 1-4 |
  | Publication | 2021 IEEE Sensors |
  | DOI | 10.1109/SENSORS47087.2021.9639748 |
  | Journal Abbr | 2021 IEEE Sensors |
  | Date Added | 17/06/2025, 18:30:59 |
  | Modified | 17/06/2025, 18:30:59 |

  ### Attachments

  - PDF
- ## The Role of Oxytocin in the Dog–Owner Relationship

  |  |  |
  | --- | --- |
  | Item Type | Journal Article |
  | Author | Anne Meinert |
  | Author | T. Deschner |
  | Author | F. Schaebs |
  | Author | S. Marshall-Pescini |
  | Author | F. Range |
  | Author | Alina Gaugg |
  | Date | 2019-10-01 |
  | URL | https://consensus.app/papers/the-role-of-oxytocin-in-the-dog%E2%80%93owner-relationship-meinert-deschner/da7848f459b15165a232f855e1d6f051/ |
  | Volume | 9 |
  | Publication | Animals : an Open Access Journal from MDPI |
  | DOI | 10.3390/ani9100792 |
  | Journal Abbr | Animals : an Open Access Journal from MDPI |
  | Date Added | 20/06/2025, 10:21:07 |
  | Modified | 20/06/2025, 10:21:07 |

  ### Attachments

  - PDF
- ## Oxytocin and Cortisol Levels in Dog Owners and Their Dogs Are Associated with Behavioral Patterns: An Exploratory Study

  |  |  |
  | --- | --- |
  | Item Type | Journal Article |
  | Author | E. Hydbring-Sandberg |
  | Author | Linda Handlin |
  | Author | Lise-Lotte Gustafson |
  | Author | K. Uvnäs-Moberg |
  | Author | Anne Nilsson |
  | Author | M. Petersson |
  | Date | 2017-10-13 |
  | URL | https://consensus.app/papers/oxytocin-and-cortisol-levels-in-dog-owners-and-their-dogs-hydbring-sandberg-handlin/bcc2b8f5b33c58e0bc8caddd9502c762/ |
  | Volume | 8 |
  | Publication | Frontiers in Psychology |
  | DOI | 10.3389/fpsyg.2017.01796 |
  | Journal Abbr | Frontiers in Psychology |
  | Date Added | 20/06/2025, 10:21:07 |
  | Modified | 20/06/2025, 10:21:07 |

  ### Attachments

  - PDF
