# Supplementary material for: Evidence of Physiological Comodulation During Human–Animal Interaction: A Systematic Review
Source: Ann N Y Acad Sci. 2026 Jun 4;1560(1):e70299. doi: 10.1111/nyas.70299 (PMC13238372; doi:10.1111/nyas.70299)
Supplement: Supplementary file 2 — Supplementary Materials: Supp2‐Zotero‐Collection.zip [file NYAS-1560-0-s002.zip › Supp2_Zotero_Collection/text screened/Correlated 1.htm]

Zotero Report


- ## Salivary Cortisol Interactions in Search and Rescue Dogs and Their Handlers

  |  |  |
  | --- | --- |
  | Item Type | Journal Article |
  | Author | Justyna Wojtaś |
  | Author | Mirosław Karpiński |
  | Author | Piotr Czyżowski |
  | Abstract | Search and rescue dogs are an important link in the search for missing persons. The aim of the study was to assess exam stress in search and rescue dogs and their handlers. The study included 41 rescue teams taking exams of field and rubble specialties. The level of cortisol, which is the main glucocorticosteroid modulating stress reactions in humans and dogs, was analyzed. The biological material used to assess the hormone concentration was saliva collected in a non-invasive way. In total, 164 test samples were collected: two from the dog and two from the handler before and immediately after the exam. Rescue exams were shown to significantly increase salivary cortisol in both dogs and their handlers. Strong interactions between cortisol levels in human–dog teams were also found with a more pronounced effect in female dog–female handler dyads. |
  | Date | 2020-04-01 |
  | Language | en |
  | Library Catalogue | DOI.org (Crossref) |
  | URL | https://www.mdpi.com/2076-2615/10/4/595 |
  | Accessed | 01/07/2025, 15:48:02 |
  | Rights | https://creativecommons.org/licenses/by/4.0/ |
  | Volume | 10 |
  | Pages | 595 |
  | Publication | Animals |
  | DOI | 10.3390/ani10040595 |
  | Issue | 4 |
  | Journal Abbr | Animals |
  | ISSN | 2076-2615 |
  | Date Added | 01/07/2025, 15:48:02 |
  | Modified | 01/07/2025, 15:48:02 |

  ### Attachments

  - Full Text
- ## The relationship of early life adversity and physiological synchrony within the therapeutic triad in horse-assisted therapy

  |  |  |
  | --- | --- |
  | Item Type | Journal Article |
  | Author | Stella Wienhold |
  | Author | Larissa Bär |
  | Author | Zoe Ringleb |
  | Author | Victoria Zirpel |
  | Author | Annette Gomolla |
  | Author | Bernadette F. Denk |
  | Author | Nina Volkmer |
  | Author | Raphaela J. Gaertner |
  | Author | Elea S. C. Klink |
  | Author | Jens C. Pruessner |
  | Abstract | Abstract In any therapeutic setting, the outcome depends in part on the therapeutic alliance, characterized by mutual understanding, empathy and trust among the participants. This also manifests through physiological synchronization (PS) processes involving breathing, heart and brain. This study examined the dynamics of heart rate variability (HRV) synchronization patterns during horse-assisted therapy. We explored the correlations between the therapist’s horse preference, levels of early life adversity (ELA), and PS relationships within and across dyads of participants, therapists, and therapy horses. Our sample of 42 female participants engaged in standardized horse-assisted therapy sessions facilitated by three riding therapists and four therapy horses. PS was operationalized through cross-wavelet power analyses across the different dyads. The results showed, that stronger HRV synchronization between the therapist and horse was associated with stronger HRV synchronization between the horse and participant, as well as stronger HRV synchronization between the therapist and participant. We found a correlation between ELA and HRV synchronization between participants and therapists, with individuals experiencing higher levels of ELA showing lower synchronization. However, this effect of ELA was not observed for HRV synchronization between participants and horses. Furthermore, we found a negative correlation between the riding therapist’s preference for a particular therapy horse and the HRV synchronization between the therapist and that horse. These findings contribute to a better understanding of the correlational dynamics in horse-human interactions and may have potential implications for optimizing therapeutic interventions in clinical settings. |
  | Date | 2025-05-27 |
  | Language | en |
  | Library Catalogue | DOI.org (Crossref) |
  | URL | https://link.springer.com/10.1007/s00702-025-02947-7 |
  | Accessed | 01/07/2025, 17:35:46 |
  | Publication | Journal of Neural Transmission |
  | DOI | 10.1007/s00702-025-02947-7 |
  | Journal Abbr | J Neural Transm |
  | ISSN | 0300-9564, 1435-1463 |
  | Date Added | 01/07/2025, 17:35:46 |
  | Modified | 01/07/2025, 17:35:46 |

  ### Attachments

  - PDF
- ## Cortisol release, heart rate and heart rate variability in the horse and its rider: Different responses to training and performance

  |  |  |
  | --- | --- |
  | Item Type | Journal Article |
  | Author | Mareike Von Lewinski |
  | Author | Sophie Biau |
  | Author | Regina Erber |
  | Author | Natascha Ille |
  | Author | Jörg Aurich |
  | Author | Jean-Michel Faure |
  | Author | Erich Möstl |
  | Author | Christine Aurich |
  | Date | 08/2013 |
  | Language | en |
  | Short Title | Cortisol release, heart rate and heart rate variability in the horse and its rider |
  | Library Catalogue | DOI.org (Crossref) |
  | URL | https://linkinghub.elsevier.com/retrieve/pii/S1090023313000038 |
  | Accessed | 01/07/2025, 12:19:29 |
  | Volume | 197 |
  | Pages | 229-232 |
  | Publication | The Veterinary Journal |
  | DOI | 10.1016/j.tvjl.2012.12.025 |
  | Issue | 2 |
  | Journal Abbr | The Veterinary Journal |
  | ISSN | 10900233 |
  | Date Added | 01/07/2025, 12:19:29 |
  | Modified | 01/07/2025, 12:19:29 |

  ### Attachments

  - PDF
- ## Long-term stress levels are synchronized in dogs and their owners

  |  |  |
  | --- | --- |
  | Item Type | Journal Article |
  | Author | Ann-Sofie Sundman |
  | Author | Enya Van Poucke |
  | Author | Ann-Charlotte Svensson Holm |
  | Author | Åshild Faresjö |
  | Author | Elvar Theodorsson |
  | Author | Per Jensen |
  | Author | Lina S. V. Roth |
  | Abstract | Abstract This study reveals, for the first time, an interspecific synchronization in long-term stress levels. Previously, acute stress, has been shown to be highly contagious both among humans and between individuals of other species. Here, long-term stress synchronization in dogs and their owners was investigated. We studied 58 dog-human dyads and analyzed their hair cortisol concentrations (HCC) at two separate occasions, reflecting levels during previous summer and winter months. The personality traits of both dogs and their owners were determined through owner-completed Dog Personality Questionnaire (DPQ) and human Big Five Inventory (BFI) surveys. In addition, the dogs’ activity levels were continuously monitored with a remote cloud-based activity collar for one week. Shetland sheepdogs (N = 33) and border collies (N = 25), balanced for sex, participated, and both pet dogs and actively competing dogs (agility and obedience) were included to represent different lifestyles. The results showed significant interspecies correlations in long-term stress where human HCC from both summer and winter samplings correlated strongly with dog HCC (summer: N = 57, χ 2  = 23.697, P < 0.001, β = 0.235; winter: N = 55, χ 2  = 13.796, P < 0.001, β = 0.027). Interestingly, the dogs’ activity levels did not affect HCC, nor did the amount of training sessions per week, showing that the HCC levels were not related to general physical activity. Additionally, there was a seasonal effect in HCC. However, although dogs’ personalities had little effects on their HCC, the human personality traits neuroticism, conscientiousness, and openness significantly affected dog HCC. Hence, we suggest that dogs, to a great extent, mirror the stress level of their owners. |
  | Date | 2019-06-06 |
  | Language | en |
  | Library Catalogue | DOI.org (Crossref) |
  | URL | https://www.nature.com/articles/s41598-019-43851-x |
  | Accessed | 01/07/2025, 17:23:11 |
  | Volume | 9 |
  | Pages | 7391 |
  | Publication | Scientific Reports |
  | DOI | 10.1038/s41598-019-43851-x |
  | Issue | 1 |
  | Journal Abbr | Sci Rep |
  | ISSN | 2045-2322 |
  | Date Added | 01/07/2025, 17:23:11 |
  | Modified | 01/07/2025, 17:23:11 |

  ### Attachments

  - Full Text
- ## Effects of Owner–Dog Relationship and Owner Personality on Cortisol Modulation in Human–Dog Dyads

  |  |  |
  | --- | --- |
  | Item Type | Journal Article |
  | Author | Iris Schöberl |
  | Author | Manuela Wedl |
  | Author | Barbara Bauer |
  | Author | Jon Day |
  | Author | Erich Möstl |
  | Author | Kurt Kotrschal |
  | Date | 06/2012 |
  | Language | en |
  | Library Catalogue | DOI.org (Crossref) |
  | URL | https://www.tandfonline.com/doi/full/10.2752/175303712X13316289505422 |
  | Accessed | 01/07/2025, 12:45:40 |
  | Volume | 25 |
  | Pages | 199-214 |
  | Publication | Anthrozoös |
  | DOI | 10.2752/175303712X13316289505422 |
  | Issue | 2 |
  | Journal Abbr | Anthrozoös |
  | ISSN | 0892-7936, 1753-0377 |
  | Date Added | 01/07/2025, 12:45:40 |
  | Modified | 01/07/2025, 12:45:40 |

  ### Attachments

  - PDF
- ## Measuring social synchrony and stress in the handler-dog dyad during animal-assisted activities: A pilot study

  |  |  |
  | --- | --- |
  | Item Type | Journal Article |
  | Author | Federica Pirrone |
  | Author | Alessandra Ripamonti |
  | Author | Elena C. Garoni |
  | Author | Sabrina Stradiotti |
  | Author | Mariangela Albertini |
  | Date | 09/2017 |
  | Language | en |
  | Short Title | Measuring social synchrony and stress in the handler-dog dyad during animal-assisted activities |
  | Library Catalogue | DOI.org (Crossref) |
  | URL | https://linkinghub.elsevier.com/retrieve/pii/S1558787817300527 |
  | Accessed | 01/07/2025, 16:49:07 |
  | Volume | 21 |
  | Pages | 45-52 |
  | Publication | Journal of Veterinary Behavior |
  | DOI | 10.1016/j.jveb.2017.07.004 |
  | Journal Abbr | Journal of Veterinary Behavior |
  | ISSN | 15587878 |
  | Date Added | 01/07/2025, 16:49:07 |
  | Modified | 01/07/2025, 16:49:07 |

  ### Attachments

  - Accepted Version
- ## Rider and Horse Salivary Cortisol Levels During Competition and Impact on Performance

  |  |  |
  | --- | --- |
  | Item Type | Journal Article |
  | Author | Marie Peeters |
  | Author | Coline Closson |
  | Author | Jean-François Beckers |
  | Author | Marc Vandenheede |
  | Date | 03/2013 |
  | Language | en |
  | Library Catalogue | DOI.org (Crossref) |
  | URL | https://linkinghub.elsevier.com/retrieve/pii/S0737080612002961 |
  | Accessed | 01/07/2025, 18:01:50 |
  | Rights | https://www.elsevier.com/tdm/userlicense/1.0/ |
  | Volume | 33 |
  | Pages | 155-160 |
  | Publication | Journal of Equine Veterinary Science |
  | DOI | 10.1016/j.jevs.2012.05.073 |
  | Issue | 3 |
  | Journal Abbr | Journal of Equine Veterinary Science |
  | ISSN | 07370806 |
  | Date Added | 01/07/2025, 18:01:50 |
  | Modified | 01/07/2025, 18:01:50 |

  ### Attachments

  - Full Text
- ## Dogs showed lower parasympathetic activity during mutual gazing while owners did not

  |  |  |
  | --- | --- |
  | Item Type | Journal Article |
  | Author | Miho Nagasawa |
  | Author | Maaya Saito |
  | Author | Haruka Hirasawa |
  | Author | Kazutaka Mogi |
  | Author | Takefumi Kikusui |
  | Date | 2023 |
  | Language | en |
  | Library Catalogue | DOI.org (Crossref) |
  | URL | https://linkinghub.elsevier.com/retrieve/pii/S1880654624000064 |
  | Accessed | 01/07/2025, 17:27:09 |
  | Volume | 73 |
  | Pages | 9 |
  | Publication | The Journal of Physiological Sciences |
  | DOI | 10.1186/s12576-023-00863-7 |
  | Issue | 1 |
  | Journal Abbr | The Journal of Physiological Sciences |
  | ISSN | 18806546 |
  | Date Added | 01/07/2025, 17:27:09 |
  | Modified | 01/07/2025, 17:27:09 |

  ### Attachments

  - Full Text
- ## A Relaxed Horse—A Relaxed Client? An Experimental Investigation of the Effects of Therapy Horses’ Stress on Clients’ Stress, Mood, and Anxiety

  |  |  |
  | --- | --- |
  | Item Type | Journal Article |
  | Author | Alicia Müller-Klein |
  | Author | Moritz Nicolai Braun |
  | Author | Diana S. Ferreira De Sá |
  | Author | Tanja Michael |
  | Author | Ulrike Link-Dorner |
  | Author | Johanna Lass-Hennemann |
  | Abstract | Equine-assisted therapies are becoming increasingly popular for addressing physical and psychological disabilities in clients. The role of the horse’s welfare in equine-assisted service receives increasing attention in research. Several studies have shown that horses are able to perceive human emotions and respond to human stress responses. However, no research has yet looked at the other side of the coin—whether and how humans perceive and react to equine stress levels during equine-assisted services. To fill this gap in the research, we employed a within-subjects design, in which horse-naïve participants had a standardized interaction with both an experimentally stressed horse and an experimentally relaxed horse. We assessed physiological indicators of stress (heart rate, heart rate variability, and salivary cortisol) in participants and horses, as well as psychological indicators of stress (state anxiety and positive and negative affect) in participants. Although our stress and relaxation manipulations were successful (indicated by horses’ physiological indicators of stress), we did not find any difference in the participants’ physiological or psychological indicators of stress between the interaction with a stressed and the interaction with a relaxed horse. Together with results from previous studies, this suggests that humans cannot intuitively recognize the (physiological) stress level of horses, which has important implications for effective communication and bonding between humans and horses and for the safety of equine activities. |
  | Date | 2024-02-13 |
  | Language | en |
  | Short Title | A Relaxed Horse—A Relaxed Client? |
  | Library Catalogue | DOI.org (Crossref) |
  | URL | https://www.mdpi.com/2076-2615/14/4/604 |
  | Accessed | 01/07/2025, 13:10:56 |
  | Rights | https://creativecommons.org/licenses/by/4.0/ |
  | Volume | 14 |
  | Pages | 604 |
  | Publication | Animals |
  | DOI | 10.3390/ani14040604 |
  | Issue | 4 |
  | Journal Abbr | Animals |
  | ISSN | 2076-2615 |
  | Date Added | 01/07/2025, 13:10:56 |
  | Modified | 01/07/2025, 13:10:56 |

  ### Attachments

  - Full Text
- ## Preliminary results suggest an influence of psychological and physiological stress in humans on horse heart rate and behavior

  |  |  |
  | --- | --- |
  | Item Type | Journal Article |
  | Author | Katrina Merkies |
  | Author | Anja Sievers |
  | Author | Emily Zakrajsek |
  | Author | Helen MacGregor |
  | Author | Renée Bergeron |
  | Author | Uta König Von Borstel |
  | Date | 09/2014 |
  | Language | en |
  | Library Catalogue | DOI.org (Crossref) |
  | URL | https://linkinghub.elsevier.com/retrieve/pii/S1558787814000860 |
  | Accessed | 01/07/2025, 12:24:40 |
  | Volume | 9 |
  | Pages | 242-247 |
  | Publication | Journal of Veterinary Behavior |
  | DOI | 10.1016/j.jveb.2014.06.003 |
  | Issue | 5 |
  | Journal Abbr | Journal of Veterinary Behavior |
  | ISSN | 15587878 |
  | Date Added | 01/07/2025, 12:24:40 |
  | Modified | 01/07/2025, 12:24:40 |

  ### Attachments

  - PDF
- ## Dog-Owner Attachment Is Associated With Oxytocin Receptor Gene Polymorphisms in Both Parties. A Comparative Study on Austrian and Hungarian Border Collies

  |  |  |
  | --- | --- |
  | Item Type | Journal Article |
  | Author | Krisztina Kovács |
  | Author | Zsófia Virányi |
  | Author | Anna Kis |
  | Author | Borbála Turcsán |
  | Author | Ágnes Hudecz |
  | Author | Maria T. Marmota |
  | Author | Dóra Koller |
  | Author | Zsolt Rónai |
  | Author | Márta Gácsi |
  | Author | József Topál |
  | Date | 2018-4-5 |
  | Library Catalogue | DOI.org (Crossref) |
  | URL | http://journal.frontiersin.org/article/10.3389/fpsyg.2018.00435/full |
  | Accessed | 01/07/2025, 15:46:33 |
  | Volume | 9 |
  | Pages | 435 |
  | Publication | Frontiers in Psychology |
  | DOI | 10.3389/fpsyg.2018.00435 |
  | Journal Abbr | Front. Psychol. |
  | ISSN | 1664-1078 |
  | Date Added | 01/07/2025, 15:46:33 |
  | Modified | 01/07/2025, 15:46:33 |

  ### Attachments

  - Full Text
- ## Investigating horse–human interactions: The effect of a nervous human

  |  |  |
  | --- | --- |
  | Item Type | Journal Article |
  | Author | Linda J. Keeling |
  | Author | Liv Jonare |
  | Author | Lovisa Lanneborn |
  | Date | 7/2009 |
  | Language | en |
  | Short Title | Investigating horse–human interactions |
  | Library Catalogue | DOI.org (Crossref) |
  | URL | https://linkinghub.elsevier.com/retrieve/pii/S109002330900121X |
  | Accessed | 01/07/2025, 12:26:52 |
  | Rights | https://www.elsevier.com/tdm/userlicense/1.0/ |
  | Volume | 181 |
  | Pages | 70-71 |
  | Publication | The Veterinary Journal |
  | DOI | 10.1016/j.tvjl.2009.03.013 |
  | Issue | 1 |
  | Journal Abbr | The Veterinary Journal |
  | ISSN | 10900233 |
  | Date Added | 01/07/2025, 12:26:52 |
  | Modified | 01/07/2025, 12:26:52 |

  ### Attachments

  - PDF
- ## Emotional reactions of horses and trainers during natural method training / Reakcje emocjonalne koni i trenerów podczas treningu metodami naturalnymi

  |  |  |
  | --- | --- |
  | Item Type | Journal Article |
  | Author | Iwona Janczarek |
  | Author | Witold Kędzierski |
  | Author | Anna Stachurska |
  | Author | Izabela Wilk |
  | Abstract | Abstract The first aim of the present study was to evaluate whether the trainer factor and the sex of the horse affect the heart rate (HR) of the trainer-horse pair. The second aim was to estimate the level of the trainer-horse pair’s emotions and to find the relationship of the HR between the trainer and the horse during the preliminary, natural-method training. The animals used in the study were 40 three-year-old purebred Arabian horses trained by two trainers from the Silversand Horsemanship School. Each trainer worked with 20 randomly selected horses, equally grouped by sex. The study was carried out during the first day of the training cycle. The aim was to have a horse accept a rider. The following items were subject to analysis: deconcentration, concentration, desensitizing, putting on the lungeing surcingle, and saddling. The emotional status of the horses and the trainers was evaluated based on HR variations which were measured by applying Polar S810 telemetric devices. The device produced continuous measurements with readings every 60 seconds. Two-factor analysis of variance and Pearson correlations were determined with the use of SAS software. Significance of differences between mean values was verified using Tukey’s test. The results obtained revealed that the sex of the trained horses was not an important factor in the evaluation of trainer’s emotions, despite the fact that fillies were characterized as having a more uniform HR. The trainer is very responsible for the emotions of a trained horse, especially at the beginning of training and during saddling. From a trainer’s point of view, it is important to complete the horse concentration task as quickly as possible. The lack of an emotional relationship in the trainer-horse pair during some training elements, suggests that it is not only the trainer’s experience, but mainly the trainer’s personality that determines the probable success in naturalmethod work. |
  | Date | 2013-03-1 |
  | Library Catalogue | DOI.org (Crossref) |
  | URL | https://content.sciendo.com/doi/10.2478/aoas-2013-0008 |
  | Accessed | 01/07/2025, 13:03:05 |
  | Volume | 13 |
  | Pages | 263-273 |
  | Publication | Annals of Animal Science |
  | DOI | 10.2478/aoas-2013-0008 |
  | Issue | 2 |
  | ISSN | 1642-3402 |
  | Date Added | 01/07/2025, 13:03:05 |
  | Modified | 01/07/2025, 13:03:05 |

  ### Attachments

  - Full Text PDF
- ## Effects of the level of experience of horses and their riders on Cortisol release, heart rate and heart-rate variability during a jumping course

  |  |  |
  | --- | --- |
  | Item Type | Journal Article |
  | Author | N Ille |
  | Author | M Von Lewinski |
  | Author | R Erber |
  | Author | M Wulf |
  | Author | J Aurich |
  | Author | E Möstl |
  | Author | C Aurich |
  | Abstract | Abstract Equestrian sports require the co-operation of two species, horses and humans, but it is unknown to what extent stress responses in the rider affect the horse. In this study, the stress response of experienced and less-experienced horses and riders at showjumping was analysed. Sixteen sport horses were divided into two groups (n = 8 each) by experience and were ridden by highly experienced professionals (n = 8) and less-experienced riders (n = 8). Riders jumped a course of obstacles with an experienced and a less-experienced horse and horses took part with an experienced and less-experienced rider. Salivary cortisol, heart rate and heart-rate variability (HRV) variables, standard deviation of RR interval (SDRR) and root mean square of successive RR differences (RMSSD) were analysed. Cortisol and heart rate increased and HRV decreased in all riders and horses. In less-experienced riders, cortisol release was higher on a less-experienced versus an experienced horse but the horses’ cortisol release was not affected by experience of their riders. Heart rate did not differ between groups of horses and was not affected by experience of the rider but was higher in less-experienced versus experienced riders. The HRV decreased in horses and riders and SDRR was lower in less-experienced versus experienced riders. Thus, lower experience of riders appears not to affect physiological stress parameters in their horses during a showjumping course. |
  | Date | 11/2013 |
  | Language | en |
  | Library Catalogue | DOI.org (Crossref) |
  | URL | https://www.cambridge.org/core/product/identifier/S0962728600005583/type/journal\_article |
  | Accessed | 01/07/2025, 12:22:13 |
  | Rights | https://www.cambridge.org/core/terms |
  | Volume | 22 |
  | Pages | 457-465 |
  | Publication | Animal Welfare |
  | DOI | 10.7120/09627286.22.4.457 |
  | Issue | 4 |
  | Journal Abbr | Anim. welf. |
  | ISSN | 0962-7286, 2054-1538 |
  | Date Added | 01/07/2025, 12:22:13 |
  | Modified | 01/07/2025, 12:22:13 |

  ### Attachments

  - PDF
- ## Towards a Multimodal Synchronized System for Quantifying Psychophysiological States in Canine Assisted Interactions

  |  |  |
  | --- | --- |
  | Item Type | Conference Paper |
  | Author | Timothy R. N. Holder |
  | Author | Colt Nichols |
  | Author | Emily Summers |
  | Author | David L. Roberts |
  | Author | Alper Bozkurt |
  | Date | 2023-12-04 |
  | Language | en |
  | Library Catalogue | DOI.org (Crossref) |
  | URL | https://dl.acm.org/doi/10.1145/3637882.3637886 |
  | Accessed | 01/07/2025, 17:10:43 |
  | Place | Raleigh NC USA |
  | Publisher | ACM |
  | ISBN | 979-8-4007-1656-0 |
  | Pages | 1-13 |
  | Proceedings Title | The Tenth International Conference on Animal-Computer Interaction |
  | Conference Name | ACI '23: The Tenth International Conference on Animal-Computer Interaction |
  | DOI | 10.1145/3637882.3637886 |
  | Date Added | 01/07/2025, 17:10:43 |
  | Modified | 01/07/2025, 17:10:43 |

  ### Attachments

  - Full Text
- ## The horse-human heart connection: Results of studies using heart rate variability

  |  |  |
  | --- | --- |
  | Item Type | Journal Article |
  | Author | Ellen Kaye Gehrke |
  | Date | 2010 |
  | Short Title | The horse-human heart connection |
  | Library Catalogue | Google Scholar |
  | URL | http://www.mindfulhorsemindfulleader.com/wp-content/uploads/2013/01/Research\_The-Horse-Human-Heart-Connection-1.pdf |
  | Accessed | 01/07/2025, 13:06:35 |
  | Pages | 20–23 |
  | Publication | NAHRA’s Strides, Spring |
  | Date Added | 01/07/2025, 13:06:35 |
  | Modified | 01/07/2025, 13:06:35 |

  ### Attachments

  - Available Version (via Google Scholar)
- ## A System for Assessment of Canine-Human Interaction during Animal-Assisted Therapies

  |  |  |
  | --- | --- |
  | Item Type | Conference Paper |
  | Author | Marc Foster |
  | Author | Eric Beppler |
  | Author | Timothy Holder |
  | Author | James Dieffenderfer |
  | Author | Patrick Erb |
  | Author | Kristy Everette |
  | Author | Margaret Gruen |
  | Author | Tamara Somers |
  | Author | Tom Evans |
  | Author | Michael Daniele |
  | Author | David L. Roberts |
  | Author | Alper Bozkurt |
  | Date | 7/2018 |
  | Library Catalogue | DOI.org (Crossref) |
  | URL | https://ieeexplore.ieee.org/document/8513384/ |
  | Accessed | 01/07/2025, 16:33:55 |
  | Place | Honolulu, HI |
  | Publisher | IEEE |
  | ISBN | 978-1-5386-3646-6 |
  | Pages | 4347-4350 |
  | Proceedings Title | 2018 40th Annual International Conference of the IEEE Engineering in Medicine and Biology Society (EMBC) |
  | Conference Name | 2018 40th Annual International Conference of the IEEE Engineering in Medicine and Biology Society (EMBC) |
  | DOI | 10.1109/EMBC.2018.8513384 |
  | Date Added | 01/07/2025, 16:33:55 |
  | Modified | 01/07/2025, 16:33:55 |

  ### Attachments

  - PDF
- ## Therapy Dogs' and Handlers' Behavior and Salivary Cortisol During Initial Visits in a Complex Medical Institution: A Pilot Study

  |  |  |
  | --- | --- |
  | Item Type | Journal Article |
  | Author | Stephanie D. Clark |
  | Author | Jessica M. Smidt |
  | Author | Brent A. Bauer |
  | Date | 2020-11-13 |
  | Short Title | Therapy Dogs' and Handlers' Behavior and Salivary Cortisol During Initial Visits in a Complex Medical Institution |
  | Library Catalogue | DOI.org (Crossref) |
  | URL | https://www.frontiersin.org/articles/10.3389/fvets.2020.564201/full |
  | Accessed | 01/07/2025, 16:45:22 |
  | Volume | 7 |
  | Pages | 564201 |
  | Publication | Frontiers in Veterinary Science |
  | DOI | 10.3389/fvets.2020.564201 |
  | Journal Abbr | Front. Vet. Sci. |
  | ISSN | 2297-1769 |
  | Date Added | 01/07/2025, 16:45:22 |
  | Modified | 01/07/2025, 16:45:22 |

  ### Attachments

  - Full Text
- ## Empathy or Apathy? Investigating the influence of owner stress on canine stress in a novel environment

  |  |  |
  | --- | --- |
  | Item Type | Journal Article |
  | Author | Aoife Byrne |
  | Author | Gareth Arnott |
  | Date | 10/2024 |
  | Language | en |
  | Short Title | Empathy or Apathy? |
  | Library Catalogue | DOI.org (Crossref) |
  | URL | https://linkinghub.elsevier.com/retrieve/pii/S016815912400251X |
  | Accessed | 01/07/2025, 16:43:41 |
  | Volume | 279 |
  | Pages | 106403 |
  | Publication | Applied Animal Behaviour Science |
  | DOI | 10.1016/j.applanim.2024.106403 |
  | Journal Abbr | Applied Animal Behaviour Science |
  | ISSN | 01681591 |
  | Date Added | 01/07/2025, 16:43:41 |
  | Modified | 01/07/2025, 16:43:41 |

  ### Attachments

  - PDF
- ## Evidence for a synchronization of hormonal states between humans and dogs during competition

  |  |  |
  | --- | --- |
  | Item Type | Journal Article |
  | Author | Alicia Phillips Buttner |
  | Author | Breanna Thompson |
  | Author | Rosemary Strasser |
  | Author | Jonathan Santo |
  | Date | 08/2015 |
  | Language | en |
  | Library Catalogue | DOI.org (Crossref) |
  | URL | https://linkinghub.elsevier.com/retrieve/pii/S003193841500205X |
  | Accessed | 01/07/2025, 12:48:46 |
  | Volume | 147 |
  | Pages | 54-62 |
  | Publication | Physiology & Behavior |
  | DOI | 10.1016/j.physbeh.2015.04.010 |
  | Journal Abbr | Physiology & Behavior |
  | ISSN | 00319384 |
  | Date Added | 01/07/2025, 12:48:46 |
  | Modified | 01/07/2025, 12:48:46 |

  ### Attachments

  - PDF
- ## Effects of Equine Interaction on Mutual Autonomic Nervous System Responses and Interoception in a Learning Program for Older Adults

  |  |  |
  | --- | --- |
  | Item Type | Journal Article |
  | Author | Ann L Baldwin |
  | Author | Lisa Walters |
  | Author | Barbara K Rector |
  | Author | Ann C Alden |
  | Abstract | Equine-assisted learning (EAL) may improve the health of older adults, but scientific data are sparse. This study investigated whether people aged 55 and older show increased heart rate variability (HRV) during EAL and awareness of bodily sensations that are overall pleasant. Subjects (n = 24) participated in mindful grooming during which they slowed their breathing and brushed a horse while noticing sensations in their body and watching the horse’s reactions. The subject’s and horse’s HRV were recorded simultaneously before, during, and after mindful grooming. For control, the same subjects performed mindful grooming with a plush simulation horse. During exit interviews, participants described their sensations. Words and gestures were categorized as positive, neutral, or negative. During mindful grooming, human heart rate and HRV (standard deviation of interbeat interval, SDRR) increased compared to baseline (paired t-test, t = –4.228, p < 0.001; t = –3.814, p = 0.001), as did the percent very low frequency (%VLF) component of HRV (t = –4.274, p < 0.001). Equine HRV values remained in the normal range, mostly VLF. In 10 cases, during mindful grooming, horse and human HRVs showed matching VLF frequencies. Grooming the simulation horse significantly elevated SDRR but did not alter %VLF. Exit interviews revealed significantly more positive gestures (t = –3.814, p = 0.031) and fewer negative gestures (Wilcoxon signed-rank test, Z-statistic = –2.12, p = 0.036, p < 0.05) when participants spoke about the real horse compared to the simulation. These findings demonstrate that during mindful grooming people aged 55 and older benefit by experiencing increased HRV, heightened awareness of pleasant bodily sensations, and often some synchronization of their HRV frequency spectrum with that of their horse, possibly reflecting emotional bonding. |
  | Date | 2023 |
  | Language | en |
  | Library Catalogue | Zotero |
  | Volume | 6 |
  | Issue | 1 |
  | Date Added | 01/07/2025, 15:37:46 |
  | Modified | 01/07/2025, 15:37:46 |

  ### Attachments

  - PDF
- ## Physiological and Behavioral Benefits for People and Horses during Guided Interactions at an Assisted Living Residence

  |  |  |
  | --- | --- |
  | Item Type | Journal Article |
  | Author | Ann Baldwin |
  | Author | Barbara Rector |
  | Author | Ann Alden |
  | Abstract | Assisted living is a fast-growing living option for seniors who require residence-based activities for maintaining mental and physical health. Guided equine interactions may benefit seniors, so an on-site equine program was started at Hacienda at the River senior living community. For research purposes, twenty-four residents and associates, aged fifty-five or over, consented to physiological measurements before, during and after four guided sessions of stroking one of three horses for 10 min over 4–6 weeks. Heart rate variability (HRV) was measured simultaneously in humans and horses during interactions. We hypothesized that human heart rate (HR) and HRV would increase during stroking and HRV power would shift toward the very low frequency (VLF) range common in horses, indicative of healthy function. During stroking, human HR increased (p < 0.05) but HRV (SDRR) and %VLF of HRV power did not change. Diastolic blood pressure (DBP), an exploratory measure, significantly increased after stroking, consistent with arousal. Two horses showed no significant changes in HR or HRV, but one relaxed. Sixteen horse–human pairs demonstrated synchronized HRV peak frequencies during sessions, suggestive of social connection. Participants used more positive than negative words describing their experience during exit interviews (p < 0.05). These data show that horses animate seniors without causing emotional stress and provide opportunities for social bonding. |
  | Date | 2021-09-23 |
  | Language | en |
  | Library Catalogue | DOI.org (Crossref) |
  | URL | https://www.mdpi.com/2076-328X/11/10/129 |
  | Accessed | 01/07/2025, 18:00:17 |
  | Rights | https://creativecommons.org/licenses/by/4.0/ |
  | Volume | 11 |
  | Pages | 129 |
  | Publication | Behavioral Sciences |
  | DOI | 10.3390/bs11100129 |
  | Issue | 10 |
  | Journal Abbr | Behavioral Sciences |
  | ISSN | 2076-328X |
  | Date Added | 01/07/2025, 18:00:17 |
  | Modified | 01/07/2025, 18:00:17 |

  ### Attachments

  - Full Text
