# Supplementary material for: Evidence of Physiological Comodulation During Human–Animal Interaction: A Systematic Review
Source: Ann N Y Acad Sci. 2026 Jun 4;1560(1):e70299. doi: 10.1111/nyas.70299 (PMC13238372; doi:10.1111/nyas.70299)
Supplement: Supplementary file 2 — Supplementary Materials: Supp2‐Zotero‐Collection.zip [file NYAS-1560-0-s002.zip › Supp2_Zotero_Collection/text screened/correlated 2.htm]

Zotero Report


- ## Equine-assisted therapy and its impact on cortisol levels of children and horses: a pilot study and meta-analysis

  |  |  |
  | --- | --- |
  | Item Type | Journal Article |
  | Author | Jan Yorke |
  | Author | William Nugent |
  | Author | Elizabeth Strand |
  | Author | Rebecca Bolen |
  | Author | John New |
  | Author | Cindy Davis |
  | Date | 07/2013 |
  | Language | en |
  | Short Title | Equine-assisted therapy and its impact on cortisol levels of children and horses |
  | Library Catalogue | Crossref |
  | URL | https://www.tandfonline.com/doi/full/10.1080/03004430.2012.693486 |
  | Accessed | 09/07/2025, 12:07:46 |
  | Volume | 183 |
  | Publisher | Informa UK Limited |
  | Pages | 874-894 |
  | Publication | Early Child Development and Care |
  | DOI | 10.1080/03004430.2012.693486 |
  | Issue | 7 |
  | ISSN | 0300-4430, 1476-8275 |
  | Date Added | 09/07/2025, 12:07:46 |
  | Modified | 09/07/2025, 12:07:46 |

  ### Attachments

  - PDF
- ## Emotional contagion: Dogs and humans show a similar physiological response to human infant crying

  |  |  |
  | --- | --- |
  | Item Type | Journal Article |
  | Author | Min Hooi Yong |
  | Author | Ted Ruffman |
  | Date | 10/2014 |
  | Language | en |
  | Short Title | Emotional contagion |
  | Library Catalogue | Crossref |
  | URL | https://linkinghub.elsevier.com/retrieve/pii/S0376635714002472 |
  | Accessed | 09/07/2025, 11:30:51 |
  | Rights | https://www.elsevier.com/tdm/userlicense/1.0/ |
  | Volume | 108 |
  | Publisher | Elsevier BV |
  | Pages | 155-165 |
  | Publication | Behavioural Processes |
  | DOI | 10.1016/j.beproc.2014.10.006 |
  | ISSN | 0376-6357 |
  | Date Added | 09/07/2025, 11:30:51 |
  | Modified | 09/07/2025, 11:30:51 |

  ### Attachments

  - PDF
- ## Salivary Cortisol Levels in Horses and their Riders During Three-Day-Events

  |  |  |
  | --- | --- |
  | Item Type | Journal Article |
  | Author | Katarzyna Strzelec |
  | Author | Witold Kędzierski |
  | Author | Andrzej Bereznowski |
  | Author | Iwona Janczarek |
  | Author | Krzysztof Bocian |
  | Author | Maciej Radosz |
  | Abstract | Abstract The group of 36 warm-blooded half-bred horses (18 stallions and 18 mares) and their riders (20 men and 16 women), who ended three-day-events, were selected for the study. The horses were aged 4 to 6 years, while the riders were 19 to 34-year-old. The saliva samples were collected after each phase of the competitions. The cortisol concentration was determined using an immunoassay method. The following factors were considered: type of competition, horse sex, and rider gender. In horses, the statistically important correlation was found between the results obtained for the dressage and cross-country, for the cross-country and show jumping, and for the dressage and show jumping. An analogous comparison for the riders suggests a statistically significant correlation between the data obtained for the cross-country and show jumping. Comparing the data of horses and their riders, a significant correlation coefficient was found for the cross-country group of woman and the dressage group of men. In conclusion, the salivary cortisol level in individual horses in each phase of three-day-event was found to be repetitive. Therefore, the salivary cortisol test is demonstrated to be a useful method to evaluate the horse response to each type of competition during three-day-events. |
  | Date | 2013-06-01 |
  | Language | en |
  | Library Catalogue | Crossref |
  | URL | https://www.sciendo.com/article/10.2478/bvip-2013-0042 |
  | Accessed | 08/07/2025, 13:01:45 |
  | Volume | 57 |
  | Publisher | Walter de Gruyter GmbH |
  | Pages | 237-241 |
  | Publication | Bulletin of the Veterinary Institute in Pulawy |
  | DOI | 10.2478/bvip-2013-0042 |
  | Issue | 2 |
  | ISSN | 2300-3235 |
  | Date Added | 08/07/2025, 13:01:45 |
  | Modified | 08/07/2025, 13:01:45 |

  ### Attachments

  - Full Text
- ## Physiological Indicators of Attachment in Domestic Dogs (Canis familiaris) and Their Owners in the Strange Situation Test

  |  |  |
  | --- | --- |
  | Item Type | Journal Article |
  | Author | Morag G. Ryan |
  | Author | Anne E. Storey |
  | Author | Rita E. Anderson |
  | Author | Carolyn J. Walsh |
  | Date | 2019-07-23 |
  | Library Catalogue | Crossref |
  | URL | https://www.frontiersin.org/article/10.3389/fnbeh.2019.00162/full |
  | Accessed | 08/07/2025, 12:09:49 |
  | Rights | https://creativecommons.org/licenses/by/4.0/ |
  | Volume | 13 |
  | Publisher | Frontiers Media SA |
  | Publication | Frontiers in Behavioral Neuroscience |
  | DOI | 10.3389/fnbeh.2019.00162 |
  | Journal Abbr | Front. Behav. Neurosci. |
  | ISSN | 1662-5153 |
  | Date Added | 08/07/2025, 12:09:49 |
  | Modified | 08/07/2025, 12:09:49 |

  ### Attachments

  - Full Text
- ## The influence of challenging objects and horse-rider matching on heart rate, heart rate variability and behavioural score in riding horses

  |  |  |
  | --- | --- |
  | Item Type | Journal Article |
  | Author | Carolien C.B.M. Munsters |
  | Author | Kathalijne E.K. Visser |
  | Author | Jan Van Den Broek |
  | Author | Marianne M. Sloet Van Oldruitenborgh-Oosterbaan |
  | Date | 04/2012 |
  | Language | en |
  | Library Catalogue | Crossref |
  | URL | https://linkinghub.elsevier.com/retrieve/pii/S1090023311001493 |
  | Accessed | 08/07/2025, 11:51:07 |
  | Rights | https://www.elsevier.com/tdm/userlicense/1.0/ |
  | Volume | 192 |
  | Publisher | Elsevier BV |
  | Pages | 75-80 |
  | Publication | The Veterinary Journal |
  | DOI | 10.1016/j.tvjl.2011.04.011 |
  | Issue | 1 |
  | ISSN | 1090-0233 |
  | Date Added | 08/07/2025, 11:51:07 |
  | Modified | 08/07/2025, 11:51:07 |

  ### Attachments

  - PDF
- ## The Effects of Equine Assisted Therapy on Plasma Cortisol and Oxytocin Concentrations and Heart Rate Variability in Horses and Measures of Symptoms of Post-Traumatic Stress Disorder in Veterans

  |  |  |
  | --- | --- |
  | Item Type | Journal Article |
  | Author | Karyn Malinowski |
  | Author | Chi Yee |
  | Author | Jenni M. Tevlin |
  | Author | Eric K. Birks |
  | Author | Mary M. Durando |
  | Author | Hossein Pournajafi-Nazarloo |
  | Author | Alan A. Cavaiola |
  | Author | Kenneth H. McKeever |
  | Date | 05/2018 |
  | Language | en |
  | Library Catalogue | Crossref |
  | URL | https://linkinghub.elsevier.com/retrieve/pii/S0737080617307761 |
  | Accessed | 08/07/2025, 12:39:03 |
  | Rights | https://www.elsevier.com/tdm/userlicense/1.0/ |
  | Volume | 64 |
  | Publisher | Elsevier BV |
  | Pages | 17-26 |
  | Publication | Journal of Equine Veterinary Science |
  | DOI | 10.1016/j.jevs.2018.01.011 |
  | ISSN | 0737-0806 |
  | Date Added | 08/07/2025, 12:39:03 |
  | Modified | 08/07/2025, 12:39:03 |

  ### Attachments

  - PDF
- ## Dyadic relationships and operational performance of male and female owners and their male dogs

  |  |  |
  | --- | --- |
  | Item Type | Journal Article |
  | Author | Kurt Kotrschal |
  | Author | Iris Schöberl |
  | Author | Barbara Bauer |
  | Author | Anne-Marie Thibeaut |
  | Author | Manuela Wedl |
  | Date | 07/2009 |
  | Language | en |
  | Library Catalogue | Crossref |
  | URL | https://linkinghub.elsevier.com/retrieve/pii/S037663570900103X |
  | Accessed | 08/07/2025, 16:18:57 |
  | Rights | https://www.elsevier.com/tdm/userlicense/1.0/ |
  | Volume | 81 |
  | Publisher | Elsevier BV |
  | Pages | 383-391 |
  | Publication | Behavioural Processes |
  | DOI | 10.1016/j.beproc.2009.04.001 |
  | Issue | 3 |
  | ISSN | 0376-6357 |
  | Date Added | 08/07/2025, 16:18:57 |
  | Modified | 08/07/2025, 16:18:57 |

  ### Attachments

  - PDF
- ## Oxytocin bonds between human and dog

  |  |  |
  | --- | --- |
  | Item Type | Journal Article |
  | Author | Takefumi Kikusui |
  | Date | 2017 |
  | Language | en |
  | Library Catalogue | Crossref |
  | URL | https://www.jstage.jst.go.jp/article/janip/67/1/67\_67.1.1/\_article |
  | Accessed | 09/07/2025, 11:29:03 |
  | Volume | 67 |
  | Publisher | Japanese Society of Animal Psychology |
  | Pages | 19-27 |
  | Publication | Japanese Journal of Animal Psychology |
  | DOI | 10.2502/janip.67.1.1 |
  | Issue | 1 |
  | Journal Abbr | Japanese Journal of Animal Psychology, The Japanese Journal of Animal Psychology |
  | ISSN | 0916-8419, 1880-9022 |
  | Date Added | 09/07/2025, 11:29:03 |
  | Modified | 09/07/2025, 11:29:03 |

  ### Attachments

  - Full Text
- ## Emotional Contagion From Humans to Dogs Is Facilitated by Duration of Ownership

  |  |  |
  | --- | --- |
  | Item Type | Journal Article |
  | Author | Maki Katayama |
  | Author | Takatomi Kubo |
  | Author | Toshitaka Yamakawa |
  | Author | Koichi Fujiwara |
  | Author | Kensaku Nomoto |
  | Author | Kazushi Ikeda |
  | Author | Kazutaka Mogi |
  | Author | Miho Nagasawa |
  | Author | Takefumi Kikusui |
  | Date | 2019-07-19 |
  | Library Catalogue | Crossref |
  | URL | https://www.frontiersin.org/article/10.3389/fpsyg.2019.01678/full |
  | Accessed | 09/07/2025, 11:31:56 |
  | Rights | https://creativecommons.org/licenses/by/4.0/ |
  | Volume | 10 |
  | Publisher | Frontiers Media SA |
  | Publication | Frontiers in Psychology |
  | DOI | 10.3389/fpsyg.2019.01678 |
  | Journal Abbr | Front. Psychol. |
  | ISSN | 1664-1078 |
  | Date Added | 09/07/2025, 11:31:56 |
  | Modified | 09/07/2025, 11:31:56 |

  ### Attachments

  - Full Text
- ## Influence of Horse and Rider on Stress during Horse-riding Lesson Program

  |  |  |
  | --- | --- |
  | Item Type | Journal Article |
  | Author | Ok-Deuk Kang |
  | Author | Young-Min Yun |
  | Date | 2016-03-22 |
  | Language | en |
  | Library Catalogue | Crossref |
  | URL | http://ajas.info/journal/view.php?doi=10.5713/ajas.15.1068 |
  | Accessed | 08/07/2025, 12:59:30 |
  | Volume | 29 |
  | Publisher | Asian Australasian Association of Animal Production Societies |
  | Pages | 895-900 |
  | Publication | Asian-Australasian Journal of Animal Sciences |
  | DOI | 10.5713/ajas.15.1068 |
  | Issue | 6 |
  | Journal Abbr | Asian Australas. J. Anim. Sci |
  | ISSN | 1011-2367, 1976-5517 |
  | Date Added | 08/07/2025, 12:59:30 |
  | Modified | 08/07/2025, 12:59:30 |

  ### Attachments

  - Full Text
- ## Interspecies hormonal interactions between man and the domestic dog (Canis familiaris)

  |  |  |
  | --- | --- |
  | Item Type | Journal Article |
  | Author | Amanda C. Jones |
  | Author | Robert A. Josephs |
  | Date | 09/2006 |
  | Language | en |
  | Library Catalogue | Crossref |
  | URL | https://linkinghub.elsevier.com/retrieve/pii/S0018506X0600122X |
  | Accessed | 08/07/2025, 12:19:26 |
  | Rights | https://www.elsevier.com/tdm/userlicense/1.0/ |
  | Volume | 50 |
  | Publisher | Elsevier BV |
  | Pages | 393-400 |
  | Publication | Hormones and Behavior |
  | DOI | 10.1016/j.yhbeh.2006.04.007 |
  | Issue | 3 |
  | ISSN | 0018-506X |
  | Date Added | 08/07/2025, 12:19:26 |
  | Modified | 08/07/2025, 12:19:26 |

  ### Attachments

  - PDF
- ## Physiological stress responses and horse rider interactions in horses ridden by male and female riders

  |  |  |
  | --- | --- |
  | Item Type | Journal Article |
  | Author | N. Ille |
  | Author | C. Aurich |
  | Author | R. Erber |
  | Author | M. Wulf |
  | Author | R. Palme |
  | Author | J. Aurich |
  | Author | M. Von Lewinski |
  | Abstract | Traditionally, horse riding has been restricted to men but today equestrian sports are dominated by women. We hypothesised that men and women differ with regard to riding and the response they evoke in their horse. Cortisol and heart rate variability (HRV) were studied in male (n=8) and female riders (n=8) and in horses (n=8) ridden by men and women over a jumping course. Saliva for cortisol analysis was collected, cardiac beat to beat (RR) intervals were recorded and heart rate and HRV variables SDRR (standard deviation of RR interval) and RMSSD (root mean square of successive RR differences) calculated. In another experiment, saddle pressure was compared between male and female riders (n=5 each). Cortisol did not differ between male and female riders and increased in horses (P<0.001) irrespective of the sex of the rider. Heart rate in riders increased from walk to jumping (P<0.001) while HRV decreased (P<0.001) to the same extent in men and women. In horses, heart rate increased (P<0.001) and SDRR and RMSSD decreased during walk and remained low at trot and canter (P<0.001) irrespective of the riders’ sex. In trot (P<0.05) and canter (P<0.01) saddle pressure was slightly lower in female versus male riders. This is due to weight differences and not to a different seat. In conclusion, no fundamental differences existed in the physical effort, stress response and seat between male and female riders and in the response of horses to men and women. |
  | Date | 2014-01-01 |
  | Library Catalogue | Crossref |
  | URL | https://brill.com/view/journals/cep/10/2/article-p131\_131.xml |
  | Accessed | 08/07/2025, 12:34:44 |
  | Volume | 10 |
  | Publisher | Walter de Gruyter GmbH |
  | Pages | 131-138 |
  | Publication | Comparative Exercise Physiology |
  | DOI | 10.3920/cep143001 |
  | Issue | 2 |
  | Journal Abbr | CEP |
  | ISSN | 1755-2540, 1755-2559 |
  | Date Added | 08/07/2025, 12:34:44 |
  | Modified | 08/07/2025, 12:34:44 |

  ### Attachments

  - PDF
- ## Cortisol concentrations in saliva of humans and their dogs during intensive training courses in animal-assisted therapy

  |  |  |
  | --- | --- |
  | Item Type | Journal Article |
  | Author | D. Haubenhofer |
  | Author | Erich Möstl |
  | Author | Sylvia Kirchengast |
  | Date | 2005-01-01 |
  | Volume | 92 |
  | Pages | 66-73 |
  | Publication | Wiener Tierarztliche Monatsschrift |
  | Journal Abbr | Wiener Tierarztliche Monatsschrift |
  | Date Added | 09/07/2025, 10:19:01 |
  | Modified | 09/07/2025, 10:19:01 |

  ### Attachments

  - PDF
- ## 'Dog Handlers' and Dogs' Emotional and Cortisol Secretion Responses Associated with Animal-Aassisted Therapy Sessions

  |  |  |
  | --- | --- |
  | Item Type | Journal Article |
  | Author | Dorit Karla Haubenhofer |
  | Author | Sylvia Kirchengast |
  | Abstract | AbstractThe study investigated 13 dog handlers and 18 companion dogs (Canis familiaris) working as teams in nonhuman animal-assisted service. The handlers described in questionnaires what emotions they chose to associate with their daily life and therapeutic work. They described their emotional condition before and after therapeutic sessions, giving analogous descriptions for their dogs. Handlers collected saliva samples from themselves and their dogs (6 non-therapeutic control days) during 3 months of therapeutic work) to measure cortisol concentrations using an enzyme-immunoassay. Handlers chose different emotions from the questionnaires for themselves and their dogs, differing from the cortisol sampling results. Handlers and dogs had increased cortisol concentrations on therapy days compared to control days. Handlers had significantly higher concentrations immediately before therapeutic sessions. In handlers, cortisol concentrations increased steadily with the duration of sessions; in dogs, with the number of sessions per week. Further study of the effects of recreation periods during therapy work days or of more days scheduled without therapy will help clarify what conditions for delivering animal-assisted service best safeguard the welfare of dog and dog handler teams. |
  | Date | 2007 |
  | Library Catalogue | Crossref |
  | URL | https://brill.com/view/journals/soan/15/2/article-p127\_3.xml |
  | Accessed | 08/07/2025, 12:06:51 |
  | Volume | 15 |
  | Publisher | Walter de Gruyter GmbH |
  | Pages | 127-150 |
  | Publication | Society & Animals |
  | DOI | 10.1163/156853007x187090 |
  | Issue | 2 |
  | Journal Abbr | Soc Animals |
  | ISSN | 1063-1119, 1568-5306 |
  | Date Added | 08/07/2025, 12:06:51 |
  | Modified | 08/07/2025, 12:06:51 |

  ### Attachments

  - PDF
- ## Glucocorticoid response to naturalistic interactions between children and dogs

  |  |  |
  | --- | --- |
  | Item Type | Journal Article |
  | Author | Gitanjali E. Gnanadesikan |
  | Author | Elizabeth Carranza |
  | Author | Katherine M. King |
  | Author | Abigail C. Flyer |
  | Author | Gianna Ossello |
  | Author | Paige G. Smith |
  | Author | Netzin G. Steklis |
  | Author | H. Dieter Steklis |
  | Author | Jessica J. Connelly |
  | Author | Melissa Barnett |
  | Author | Nancy Gee |
  | Author | Stacey Tecot |
  | Author | Evan L. MacLean |
  | Date | 05/2024 |
  | Language | en |
  | Library Catalogue | Crossref |
  | URL | https://linkinghub.elsevier.com/retrieve/pii/S0018506X24000485 |
  | Accessed | 08/07/2025, 12:04:07 |
  | Rights | https://www.elsevier.com/tdm/userlicense/1.0/ |
  | Volume | 161 |
  | Publisher | Elsevier BV |
  | Pages | 105523 |
  | Publication | Hormones and Behavior |
  | DOI | 10.1016/j.yhbeh.2024.105523 |
  | ISSN | 0018-506X |
  | Date Added | 08/07/2025, 12:04:07 |
  | Modified | 08/07/2025, 12:04:07 |

  ### Attachments

  - PDF
- ## Physiology of human-horse interactions during substance withdrawal within psychotherapy participants

  |  |  |
  | --- | --- |
  | Item Type | Journal Article |
  | Author | M.M. Friend |
  | Author | M.C. Nicodemus |
  | Author | C.A. Cavinder |
  | Author | C.O. Lemley |
  | Author | P. Prince |
  | Author | K. Holtcamp |
  | Author | R.M. Swanson |
  | Abstract | AbstractPsychotherapy incorporating equine interaction (PIE) is emerging as an effective treatment for substance use disorder (SUD); however, research concerning physiological impacts of PIE during substance withdrawal is lacking. This study investigated impacts of PIE on salivary cortisol concentrations and heart rates in SUD patients during withdrawal. Heart rate and cortisol concentrations were also measured in horses to investigate potential human-horse coupling during PIE. Saliva samples and heart rates were collected from SUD patients (n = 18) and their therapy horses (n = 4) prior to the introduction of the horse and following equine interaction within a residential psychotherapy program during the substance withdrawal period. Without the presence of the horse, the equine environment during the first week of withdrawal produced lower () cortisol and heart rate measures than found in the equine interaction for the SUD patients. Human heart rates, however, decreased () in the second week in response to the equine interaction. A strong negative correlation (r = −0.9, ) was found within the changes in human and horse cortisol concentrations during week two as human cortisol concentrations decreased while horse cortisol concentrations increased. Results indicate equine interaction during psychotherapy is more effective in the second week than the first at mitigating stress for withdrawing residential SUD treatment program patients and the equid environment, even without the presence of a horse, can positively impact stress parameters in withdrawing SUD patients during the first week of treatment. |
  | Date | 2023-10-31 |
  | Library Catalogue | Crossref |
  | URL | https://brill.com/view/journals/cep/20/1/article-p55\_6.xml |
  | Accessed | 09/07/2025, 12:31:15 |
  | Volume | 20 |
  | Publisher | Walter de Gruyter GmbH |
  | Pages | 55-68 |
  | Publication | Comparative Exercise Physiology |
  | DOI | 10.1163/17552559-20230023 |
  | Issue | 1 |
  | Journal Abbr | Comp. Exerc. |
  | ISSN | 1755-2540, 1755-2559 |
  | Date Added | 09/07/2025, 12:31:15 |
  | Modified | 09/07/2025, 12:31:15 |

  ### Attachments

  - PDF
- ## Physiological and behavioral reactivity to stress in thunderstorm-phobic dogs and their caregivers

  |  |  |
  | --- | --- |
  | Item Type | Journal Article |
  | Author | Nancy A. Dreschel |
  | Author | Douglas A. Granger |
  | Date | 12/2005 |
  | Language | en |
  | Library Catalogue | Crossref |
  | URL | https://linkinghub.elsevier.com/retrieve/pii/S0168159105001152 |
  | Accessed | 08/07/2025, 16:51:28 |
  | Rights | https://www.elsevier.com/tdm/userlicense/1.0/ |
  | Volume | 95 |
  | Publisher | Elsevier BV |
  | Pages | 153-168 |
  | Publication | Applied Animal Behaviour Science |
  | DOI | 10.1016/j.applanim.2005.04.009 |
  | Issue | 3-4 |
  | ISSN | 0168-1591 |
  | Date Added | 08/07/2025, 16:51:28 |
  | Modified | 08/07/2025, 16:51:28 |

  ### Attachments

  - PDF
- ## Pilot Study of the Influence of Equine Assisted Therapy on Physiological and Behavioral Parameters Related to Welfare of Horses and Patients

  |  |  |
  | --- | --- |
  | Item Type | Journal Article |
  | Author | María Dolores Ayala |
  | Author | Andrea Carrillo |
  | Author | Pilar Iniesta |
  | Author | Pedro Ferrer |
  | Abstract | Different welfare indicators were studied in three patients with psychomotor alterations and in two horses throughout 9–10 equine assisted therapy sessions in each patient. In horses, heart and respiratory rates, blood pressure, temperature and behavioral signs were studied. In patients, heart rate, oxygen saturation, temperature, sleep quality, psychomotor and emotional parameters were analyzed. Data collection was recorded in the anticipatory phase (15 min before the start of the session), two interaction phases (after 30 min of horse-patient interaction on the ground and on horseback, respectively) and the recovery phase (15 min after the end of the session). During the anticipatory phase, most of physiological parameters of patients and horses and the stress behavioral signs of horses increased, followed by a relaxing phase during the horse-patient interaction on the ground. In horse-patient riding phase the heart and respiratory rates of the horses again increased. These results showed that the horses did not seem to suffer stress attributable to the therapy sessions, but only an increase in their parameters associated with activity and external stimuli. The patients improved their gross and fine motor skills, their cognitive and perceptual-sensitive parameters and it led to an improvement in the life quality of their families. |
  | Date | 2021-12-10 |
  | Language | en |
  | Library Catalogue | Crossref |
  | URL | https://www.mdpi.com/2076-2615/11/12/3527 |
  | Accessed | 09/07/2025, 12:06:51 |
  | Rights | https://creativecommons.org/licenses/by/4.0/ |
  | Volume | 11 |
  | Publisher | MDPI AG |
  | Pages | 3527 |
  | Publication | Animals |
  | DOI | 10.3390/ani11123527 |
  | Issue | 12 |
  | ISSN | 2076-2615 |
  | Date Added | 09/07/2025, 12:06:51 |
  | Modified | 09/07/2025, 12:06:51 |

  ### Attachments

  - Full Text
- ## Hormonal and Neurological Aspects of Dog Walking for Dog Owners and Pet Dogs

  |  |  |
  | --- | --- |
  | Item Type | Journal Article |
  | Author | Junko Akiyama |
  | Author | Mitsuaki Ohta |
  | Abstract | The hormone oxytocin is involved in various aspects of the relationship between humans and animals. Dog walking is a common activity for dog owners and their dogs. The walk, of course, should be good for the health of the dog as well as its owner. In Experiment I, we assessed whether salivary oxytocin and cortisol in dog owners changed because of walking their dogs. Ten owners walked with their dogs and walked alone. Similar to other previous research, walking with a dog did not significantly change oxytocin and cortisol. Therefore, in Experiment II, we investigated the effect of dog walking on brain noradrenergic and GABAergic neural activity, as indicated by salivary MHPG and GABA, in 14 dog owners. Walking with a dog reduced salivary MHPG compared to walking alone, and MHPG was correlated negatively with GABA. Thus, dog walking activated GABAergic nerves in the brain and suppressed noradrenergic nerves, effectively relieving stress. |
  | Date | 2021-09-18 |
  | Language | en |
  | Library Catalogue | Crossref |
  | URL | https://www.mdpi.com/2076-2615/11/9/2732 |
  | Accessed | 09/07/2025, 10:26:41 |
  | Rights | https://creativecommons.org/licenses/by/4.0/ |
  | Volume | 11 |
  | Publisher | MDPI AG |
  | Pages | 2732 |
  | Publication | Animals |
  | DOI | 10.3390/ani11092732 |
  | Issue | 9 |
  | ISSN | 2076-2615 |
  | Date Added | 09/07/2025, 10:26:41 |
  | Modified | 09/07/2025, 10:26:41 |

  ### Attachments

  - Full Text
