# Supplementary material for: Evidence of Physiological Comodulation During Human–Animal Interaction: A Systematic Review
Source: Ann N Y Acad Sci. 2026 Jun 4;1560(1):e70299. doi: 10.1111/nyas.70299 (PMC13238372; doi:10.1111/nyas.70299)
Supplement: Supplementary file 2 — Supplementary Materials: Supp2‐Zotero‐Collection.zip [file NYAS-1560-0-s002.zip › Supp2_Zotero_Collection/text screened/Correlated 3.htm]

Zotero Report


- ## Psychophysiological effects of equine-facilitated psychotherapy on Veterans with PTSD and their horse partners

  |  |  |
  | --- | --- |
  | Item Type | Journal Article |
  | Author | Laurie A. McDuffee |
  | Author | William J. Montelpare |
  | Author | Caroline LeBlanc |
  | Abstract | LAY SUMMARY Veterans with posttraumatic stress disorder (PTSD) often struggle with emotion and impulse control, resulting in an inability to appropriately handle even minimal stress, which can lead to physiological dysregulation. This study used a variety of measures to assess the effect of equine-facilitated psychotherapy on Veterans with PTSD. Given that maintaining practices of good welfare for animals is essential in supporting these types of therapeutic programs for Veterans, the study also evaluated the welfare of the horse participants. The results showed that equine-facilitated psychotherapy had a positive impact on the perceived post-program responses of human participants and that horses were not stressed during the sessions and likely perceived the sessions as a neutral stimulus. |
  | Date | 2024-06-01 |
  | Language | en |
  | Library Catalogue | Crossref |
  | URL | https://utppublishing.com/doi/10.3138/jmvfh-2023-0063 |
  | Accessed | 10/07/2025, 12:49:53 |
  | Volume | 10 |
  | Publisher | University of Toronto Press Inc. (UTPress) |
  | Pages | 135-147 |
  | Publication | Journal of Military, Veteran and Family Health |
  | DOI | 10.3138/jmvfh-2023-0063 |
  | Issue | 3 |
  | ISSN | 2368-7924, 2368-7924 |
  | Date Added | 10/07/2025, 12:49:53 |
  | Modified | 10/07/2025, 12:49:53 |

  ### Attachments

  - PDF
