# Supplementary material for: Evidence of Physiological Comodulation During Human–Animal Interaction: A Systematic Review
Source: Ann N Y Acad Sci. 2026 Jun 4;1560(1):e70299. doi: 10.1111/nyas.70299 (PMC13238372; doi:10.1111/nyas.70299)
Supplement: Supplementary file 2 — Supplementary Materials: Supp2‐Zotero‐Collection.zip [file NYAS-1560-0-s002.zip › Supp2_Zotero_Collection/text screened/EMBase.htm]

Zotero Report


- ## Psychobiological Factors Affecting Cortisol Variability in Human-Dog Dyads

  |  |  |
  | --- | --- |
  | Item Type | Journal Article |
  | Author | Iris Schöberl |
  | Author | Manuela Wedl |
  | Author | Andrea Beetz |
  | Author | Kurt Kotrschal |
  | Editor | Urs M. Nater |
  | Date | 2017-2-8 |
  | Language | en |
  | Library Catalogue | DOI.org (Crossref) |
  | URL | https://dx.plos.org/10.1371/journal.pone.0170707 |
  | Accessed | 27/06/2025, 16:34:57 |
  | Volume | 12 |
  | Pages | e0170707 |
  | Publication | PLOS ONE |
  | DOI | 10.1371/journal.pone.0170707 |
  | Issue | 2 |
  | Journal Abbr | PLoS ONE |
  | ISSN | 1932-6203 |
  | Date Added | 27/06/2025, 16:34:57 |
  | Modified | 27/06/2025, 16:34:57 |

  ### Attachments

  - Full Text
- ## Does stress run through the leash? An examination of stress transmission between owners and dogs during a walk

  |  |  |
  | --- | --- |
  | Item Type | Journal Article |
  | Author | Helen Harvie |
  | Author | Alejandro Rodrigo |
  | Author | Candace Briggs |
  | Author | Shane Thiessen |
  | Author | Debbie M. Kelly |
  | Date | 03/2021 |
  | Language | en |
  | Short Title | Does stress run through the leash? |
  | Library Catalogue | DOI.org (Crossref) |
  | URL | https://link.springer.com/10.1007/s10071-020-01460-6 |
  | Accessed | 27/06/2025, 16:32:56 |
  | Volume | 24 |
  | Pages | 239-250 |
  | Publication | Animal Cognition |
  | DOI | 10.1007/s10071-020-01460-6 |
  | Issue | 2 |
  | Journal Abbr | Anim Cogn |
  | ISSN | 1435-9448, 1435-9456 |
  | Date Added | 27/06/2025, 16:32:56 |
  | Modified | 27/06/2025, 16:32:56 |

  ### Attachments

  - PDF
