# Supplementary material for: Evidence of Physiological Comodulation During Human–Animal Interaction: A Systematic Review
Source: Ann N Y Acad Sci. 2026 Jun 4;1560(1):e70299. doi: 10.1111/nyas.70299 (PMC13238372; doi:10.1111/nyas.70299)
Supplement: Supplementary file 2 — Supplementary Materials: Supp2‐Zotero‐Collection.zip [file NYAS-1560-0-s002.zip › Supp2_Zotero_Collection/text screened/Google Scholar.htm]

Zotero Report


- ## Neurophysiological Correlates of Affiliative Behaviour between Humans and Dogs

  |  |  |
  | --- | --- |
  | Item Type | Journal Article |
  | Author | J.S.J Odendaal |
  | Author | R.A Meintjes |
  | Abstract | Few physiological parameters for positive human–companion animal contact have been identiﬁed and those that are established have all been in humans. The implication is that if the physiological reactions are mutual, dogs would experience the same psychological beneﬁts from these neurophysiological changes as humans. Therefore, we have determined the role of certain neurochemicals during afﬁliation behaviour on an interspecies basis. Our results indicate that concentrations of b-endorphin, oxytocin, prolactin, b-phenylethylamine, and dopamine increased in both species after positive interspecies interaction, while that of cortisol decreased in the humans only. Indicators of mutual physiological changes during positive interaction between dog lovers and dogs may contribute to a better understanding of the human–animal bond in veterinary practice. |
  | Date | 5/2003 |
  | Language | en |
  | Library Catalogue | DOI.org (Crossref) |
  | URL | https://linkinghub.elsevier.com/retrieve/pii/S109002330200237X |
  | Accessed | 17/06/2025, 17:10:02 |
  | Rights | https://www.elsevier.com/tdm/userlicense/1.0/ |
  | Volume | 165 |
  | Pages | 296-301 |
  | Publication | The Veterinary Journal |
  | DOI | 10.1016/S1090-0233(02)00237-X |
  | Issue | 3 |
  | Journal Abbr | The Veterinary Journal |
  | ISSN | 10900233 |
  | Date Added | 17/06/2025, 17:10:02 |
  | Modified | 17/06/2025, 17:10:02 |

  ### Attachments

  - PDF
- ## Animal-assisted therapy — magic or medicine?

  |  |  |
  | --- | --- |
  | Item Type | Journal Article |
  | Author | J.S.J Odendaal |
  | Date | 10/2000 |
  | Language | en |
  | Library Catalogue | DOI.org (Crossref) |
  | URL | https://linkinghub.elsevier.com/retrieve/pii/S0022399900001835 |
  | Accessed | 17/06/2025, 17:08:24 |
  | Rights | https://www.elsevier.com/tdm/userlicense/1.0/ |
  | Volume | 49 |
  | Pages | 275-280 |
  | Publication | Journal of Psychosomatic Research |
  | DOI | 10.1016/S0022-3999(00)00183-5 |
  | Issue | 4 |
  | Journal Abbr | Journal of Psychosomatic Research |
  | ISSN | 00223999 |
  | Date Added | 17/06/2025, 17:08:24 |
  | Modified | 17/06/2025, 17:08:24 |

  ### Attachments

  - PDF
- ## Oxytocin-gaze positive loop and the coevolution of human-dog bonds

  |  |  |
  | --- | --- |
  | Item Type | Journal Article |
  | Author | Miho Nagasawa |
  | Author | Shouhei Mitsui |
  | Author | Shiori En |
  | Author | Nobuyo Ohtani |
  | Author | Mitsuaki Ohta |
  | Author | Yasuo Sakuma |
  | Author | Tatsushi Onaka |
  | Author | Kazutaka Mogi |
  | Author | Takefumi Kikusui |
  | Date | 2015 |
  | Language | en |
  | Library Catalogue | Zotero |
  | Date Added | 17/06/2025, 17:34:17 |
  | Modified | 15/07/2025, 09:33:15 |

  ### Attachments

  - 1261022-nagasawa-sm
  - PDF
- ## Exploring Synchronicity in the Heart Rates of Familiar and Unfamiliar Pairs of Horses and Humans Undertaking an In-Hand Task

  |  |  |
  | --- | --- |
  | Item Type | Journal Article |
  | Author | Jo Hockenhull |
  | Author | Tamsin J. Young |
  | Author | Sarah E. Redgate |
  | Author | Lynda Birke |
  | Date | 2015-09-02 |
  | Language | en |
  | Library Catalogue | DOI.org (Crossref) |
  | URL | https://www.tandfonline.com/doi/full/10.1080/08927936.2015.1052284 |
  | Accessed | 17/06/2025, 18:06:24 |
  | Volume | 28 |
  | Pages | 501-511 |
  | Publication | Anthrozoös |
  | DOI | 10.1080/08927936.2015.1052284 |
  | Issue | 3 |
  | Journal Abbr | Anthrozoös |
  | ISSN | 0892-7936, 1753-0377 |
  | Date Added | 17/06/2025, 18:06:24 |
  | Modified | 17/06/2025, 18:06:24 |

  ### Attachments

  - Accepted Version
- ## Short-Term Interaction between Dogs and Their Owners: Effects on Oxytocin, Cortisol, Insulin and Heart Rate—An Exploratory Study

  |  |  |
  | --- | --- |
  | Item Type | Journal Article |
  | Author | Linda Handlin |
  | Author | Eva Hydbring-Sandberg |
  | Author | Anne Nilsson |
  | Author | Mikael Ejdebäck |
  | Author | Anna Jansson |
  | Author | Kerstin Uvnäs-Moberg |
  | Date | 09/2011 |
  | Language | en |
  | Short Title | Short-Term Interaction between Dogs and Their Owners |
  | Library Catalogue | DOI.org (Crossref) |
  | URL | https://www.tandfonline.com/doi/full/10.2752/175303711X13045914865385 |
  | Accessed | 17/06/2025, 17:35:50 |
  | Volume | 24 |
  | Pages | 301-315 |
  | Publication | Anthrozoös |
  | DOI | 10.2752/175303711X13045914865385 |
  | Issue | 3 |
  | Journal Abbr | Anthrozoös |
  | ISSN | 0892-7936, 1753-0377 |
  | Date Added | 17/06/2025, 17:35:50 |
  | Modified | 17/06/2025, 17:35:50 |

  ### Attachments

  - PDF
- ## Associations between the Psychological Characteristics of the Human–Dog Relationship and Oxytocin and Cortisol Levels

  |  |  |
  | --- | --- |
  | Item Type | Journal Article |
  | Author | Linda Handlin |
  | Author | Anne Nilsson |
  | Author | Mikael Ejdebäck |
  | Author | Eva Hydbring-Sandberg |
  | Author | Kerstin Uvnäs-Moberg |
  | Date | 06/2012 |
  | Language | en |
  | Library Catalogue | Crossref |
  | URL | https://www.tandfonline.com/doi/full/10.2752/175303712X13316289505468 |
  | Accessed | 11/07/2025, 14:50:51 |
  | Volume | 25 |
  | Publisher | Informa UK Limited |
  | Pages | 215-228 |
  | Publication | Anthrozoös |
  | DOI | 10.2752/175303712x13316289505468 |
  | Issue | 2 |
  | ISSN | 0892-7936, 1753-0377 |
  | Date Added | 11/07/2025, 14:50:51 |
  | Modified | 11/07/2025, 14:50:51 |

  ### Attachments

  - PDF
- ## Effects of stroking horses on both humans' and horses' heart rate responses<sup>1</sup>

  |  |  |
  | --- | --- |
  | Item Type | Journal Article |
  | Author | Haruyo Hama |
  | Author | Masao Yogo |
  | Author | Yoshinori Matsuyama |
  | Abstract | The present study examined both human and horse heart rates (HRs) when humans stroked horses for 90 seconds; the subjective arousal levels of the humans were measured by the Tohoku Activation Deactivation Adjective Check List before and after stroking horses. Six male sublects with a positive attitude toward companion animals and 6 male subjects with a negative attitude were selected by their scores on the Pet Attitude Scale, and these two groups, together with a third group, of 6 subjects who were male members of the Doshisha University horse-riding club, participated in this experiment. The HRs of the human subjects during the first 10 seconds immediately after the stroking began were significantly higher than those obtained after that period, but these higher levels gradually returned to baseline levels. This tendency appears more clearly in the negative attitude group. The HRs of the horses increased during the first 20 seconds immediately after the human subjects of the NA group started stroking them, but gradually reduced as the stroking continued. The results of subjective arousal levels suggest a decrease in tension by stroking horses. These results suggest that a certain affectional interaction may exist between humans and companion animals. |
  | Date | 05/1996 |
  | Language | en |
  | Library Catalogue | DOI.org (Crossref) |
  | URL | https://onlinelibrary.wiley.com/doi/10.1111/j.1468-5884.1996.tb00009.x |
  | Accessed | 17/06/2025, 16:48:18 |
  | Rights | http://onlinelibrary.wiley.com/termsAndConditions#vor |
  | Volume | 38 |
  | Pages | 66-73 |
  | Publication | Japanese Psychological Research |
  | DOI | 10.1111/j.1468-5884.1996.tb00009.x |
  | Issue | 2 |
  | Journal Abbr | Jpn Psychol Res |
  | ISSN | 0021-5368, 1468-5884 |
  | Date Added | 17/06/2025, 16:48:18 |
  | Modified | 17/06/2025, 16:48:18 |

  ### Attachments

  - PDF
