# Supplementary material for: Evidence of Physiological Comodulation During Human–Animal Interaction: A Systematic Review
Source: Ann N Y Acad Sci. 2026 Jun 4;1560(1):e70299. doi: 10.1111/nyas.70299 (PMC13238372; doi:10.1111/nyas.70299)
Supplement: Supplementary file 2 — Supplementary Materials: Supp2‐Zotero‐Collection.zip [file NYAS-1560-0-s002.zip › Supp2_Zotero_Collection/text screened/Scopus.htm]

Zotero Report


- ## Behavioral and emotional co-modulation during dog–owner interaction measured by heart rate variability and activity

  |  |  |
  | --- | --- |
  | Item Type | Journal Article |
  | Author | Aija Koskela |
  | Author | Heini Törnqvist |
  | Author | Sanni Somppi |
  | Author | Katriina Tiira |
  | Author | Virpi-Liisa Kykyri |
  | Author | Laura Hänninen |
  | Author | Jan Kujala |
  | Author | Miho Nagasawa |
  | Author | Takefumi Kikusui |
  | Author | Miiamaaria V. Kujala |
  | Abstract | Behavioral and physiological synchrony facilitate emotional closeness in attachment relationships. The aim of this pseudorandomized cross-over study was to investigate the emotional and physiological link, designated as co-modulation, between dogs and their owners. We measured the heart rate variability (HRV) and physical activity of dogs belonging to co-operative breeds (n = 29) and their owners during resting baselines and positive interaction tasks (Stroking, Training, Sniffing, Playing) and collected survey data on owner temperament and dog–owner relationship. Although overall HRV and activity correlated between dogs and their owners across tasks, task-specific analyses showed that HRV of dogs and owners correlated during free behaving (Pre- and Post-Baseline), whereas the activity of dogs and owners correlated during predefined interaction tasks (Stroking and Playing). Dog overall HRV was the only predictive factor for owner overall HRV, while dog height, ownership duration, owner negative affectivity, and dog–owner interaction scale predicted dog overall HRV. Thus, the characteristics of dog, owner, and the relationship modified the HRV responses in dog–owner dyads. The physiology and behavior of dogs belonging to co-operative breeds and their owners were therefore co-modulated, demonstrating physiological and emotional connection comparable to those found in attachment relationships between humans. |
  | Date | 2024-10-24 |
  | Language | en |
  | Library Catalogue | DOI.org (Crossref) |
  | URL | https://www.nature.com/articles/s41598-024-76831-x |
  | Accessed | 17/06/2025, 16:27:02 |
  | Volume | 14 |
  | Pages | 25201 |
  | Publication | Scientific Reports |
  | DOI | 10.1038/s41598-024-76831-x |
  | Issue | 1 |
  | Journal Abbr | Sci Rep |
  | ISSN | 2045-2322 |
  | Date Added | 17/06/2025, 16:27:02 |
  | Modified | 17/06/2025, 16:27:03 |

  ### Attachments

  - PDF
- ## Exploring the Dynamics of Canine-Assisted Interactions: A Wearable Approach to Understanding Interspecies Well-Being

  |  |  |
  | --- | --- |
  | Item Type | Journal Article |
  | Author | Timothy R. N. Holder |
  | Author | Colt Nichols |
  | Author | Emily Summers |
  | Author | David L. Roberts |
  | Author | Alper Bozkurt |
  | Date | 2024 |
  | URL | https://www.scopus.com/inward/record.uri?eid=2-s2.0-85213375550&doi=10.3390%2fani14243628&partnerID=40&md5=f5eb4ca70a3271d1bbba5f9c56a68b0c |
  | Extra | Type: Article |
  | Volume | 14 |
  | Publication | Animals |
  | DOI | 10.3390/ani14243628 |
  | Issue | 24 |
  | Date Added | 19/06/2025, 15:40:13 |
  | Modified | 19/06/2025, 15:40:13 |

  ### Notes:

  - Cited by: 1; All Open Access, Gold Open Access

  ### Attachments

  - PDF
- ## A wearable system for the evaluation of the human-horse interaction: A preliminary study

  |  |  |
  | --- | --- |
  | Item Type | Journal Article |
  | Author | Andrea Guidi |
  | Author | Antonio Lanata |
  | Author | Paolo Baragli |
  | Author | Gaetano Valenza |
  | Author | Enzo Pasquale Scilingo |
  | Date | 2016 |
  | URL | https://www.scopus.com/inward/record.uri?eid=2-s2.0-84991698546&doi=10.3390%2felectronics5040063&partnerID=40&md5=be2f249cfcbf5995397e1aac3cffb6b8 |
  | Extra | Type: Article |
  | Volume | 5 |
  | Publication | Electronics (Switzerland) |
  | DOI | 10.3390/electronics5040063 |
  | Issue | 4 |
  | Date Added | 19/06/2025, 15:40:13 |
  | Modified | 19/06/2025, 15:40:13 |

  ### Notes:

  - Cited by: 35; All Open Access, Gold Open Access, Green Open Access

  ### Attachments

  - PDF
- ## Assessing the Relationship Between Emotional States of Dogs and Their Human Handlers, Using Simultaneous Behavioral and Cardiac Measures

  |  |  |
  | --- | --- |
  | Item Type | Journal Article |
  | Author | Emma K. Grigg |
  | Author | Serene Liu |
  | Author | Denise G. Dempsey |
  | Author | Kylee Wong |
  | Author | Melissa Bain |
  | Author | John J. Sollers |
  | Author | Rani Haddock |
  | Author | Lori R. Kogan |
  | Author | Jennifer A. Barnhard |
  | Author | Ashley A. Tringali |
  | Author | Abigail P. Thigpen |
  | Author | Lynette A. Hart |
  | Date | 2022 |
  | URL | https://www.scopus.com/inward/record.uri?eid=2-s2.0-85134911057&doi=10.3389%2ffvets.2022.897287&partnerID=40&md5=dc1c7db926a688d9248b3adefe433302 |
  | Extra | Type: Article |
  | Volume | 9 |
  | Publication | Frontiers in Veterinary Science |
  | DOI | 10.3389/fvets.2022.897287 |
  | Date Added | 19/06/2025, 15:40:14 |
  | Modified | 19/06/2025, 15:40:14 |

  ### Notes:

  - Cited by: 4

  ### Attachments

  - PDF
- ## Effects of human-animal interaction on salivary and urinary oxytocin in children and dogs

  |  |  |
  | --- | --- |
  | Item Type | Journal Article |
  | Author | Gitanjali E. Gnanadesikan |
  | Author | Katherine M. King |
  | Author | Elizabeth Carranza |
  | Author | Abigail C. Flyer |
  | Author | Gianna Ossello |
  | Author | Paige G. Smith |
  | Author | Netzin G. Steklis |
  | Author | H. Dieter Steklis |
  | Author | C. Sue Carter |
  | Author | Jessica J. Connelly |
  | Author | Melissa Barnett |
  | Author | Nancy Gee |
  | Author | Stacey R. Tecot |
  | Author | Evan L. MacLean |
  | Date | 2024 |
  | URL | https://www.scopus.com/inward/record.uri?eid=2-s2.0-85199938687&doi=10.1016%2fj.psyneuen.2024.107147&partnerID=40&md5=678a7fea07f2a7f3ab186bbb2a1167ab |
  | Extra | Type: Article |
  | Volume | 169 |
  | Publication | Psychoneuroendocrinology |
  | DOI | 10.1016/j.psyneuen.2024.107147 |
  | Date Added | 20/06/2025, 09:37:47 |
  | Modified | 20/06/2025, 09:37:47 |

  ### Notes:

  - Cited by: 2

  ### Attachments

  - PDF
