# Supplementary material for: Evidence of Physiological Comodulation During Human–Animal Interaction: A Systematic Review
Source: Ann N Y Acad Sci. 2026 Jun 4;1560(1):e70299. doi: 10.1111/nyas.70299 (PMC13238372; doi:10.1111/nyas.70299)
Supplement: Supplementary file 2 — Supplementary Materials: Supp2‐Zotero‐Collection.zip [file NYAS-1560-0-s002.zip › Supp2_Zotero_Collection/text screened/Shared.htm]

Zotero Report


- ## Disrupted Human–Dog Interbrain Neural Coupling in Autism‐Associated <i>Shank3</i> Mutant Dogs

  |  |  |
  | --- | --- |
  | Item Type | Journal Article |
  | Author | Wei Ren |
  | Author | Shan Yu |
  | Author | Kun Guo |
  | Author | Chunming Lu |
  | Author | Yong Q. Zhang |
  | Abstract | AbstractDogs interact with humans effectively and intimately. However, the neural underpinnings for such interspecies social communication are not understood. It is known that interbrain activity coupling, i.e., the synchronization of neural activity between individuals, represents the neural basis of social interactions. Here, previously unknown cross‐species interbrain activity coupling in interacting human–dog dyads is reported. By analyzing electroencephalography signals from both dogs and humans, it is found that mutual gaze and petting induce interbrain synchronization in the frontal and parietal regions of the human–dog dyads, respectively. The strength of the synchronization increases with growing familiarity of the human–dog dyad over five days, and the information flow analysis suggests that the human is the leader while the dog is the follower during human–dog interactions. Furthermore, dogs with Shank3 mutations, which represent a promising complementary animal model of autism spectrum disorders (ASD), show a loss of interbrain coupling and reduced attention during human–dog interactions. Such abnormalities are rescued by the psychedelic lysergic acid diethylamide (LSD). The results reveal previously unknown interbrain synchronizations within an interacting human–dog dyad which may underlie the interspecies communication, and suggest a potential of LSD for the amelioration of social impairment in patients with ASD. |
  | Date | 11/2024 |
  | Language | en |
  | Library Catalogue | Crossref |
  | URL | https://advanced.onlinelibrary.wiley.com/doi/10.1002/advs.202402493 |
  | Accessed | 11/07/2025, 13:23:15 |
  | Rights | http://creativecommons.org/licenses/by/4.0/ |
  | Volume | 11 |
  | Publisher | Wiley |
  | Publication | Advanced Science |
  | DOI | 10.1002/advs.202402493 |
  | Issue | 41 |
  | ISSN | 2198-3844, 2198-3844 |
  | Date Added | 11/07/2025, 13:23:15 |
  | Modified | 11/07/2025, 13:23:15 |

  ### Attachments

  - Full Text PDF
- ## Quantitative heartbeat coupling measures in human-horse interaction

  |  |  |
  | --- | --- |
  | Item Type | Conference Paper |
  | Author | Antonio Lanata |
  | Author | Andrea Guidi |
  | Author | Gaetano Valenza |
  | Author | Paolo Baragli |
  | Author | Enzo Pasquale Scilingo |
  | Date | 8/2016 |
  | Library Catalogue | DOI.org (Crossref) |
  | URL | http://ieeexplore.ieee.org/document/7591286/ |
  | Accessed | 20/06/2025, 10:03:28 |
  | Place | Orlando, FL, USA |
  | Publisher | IEEE |
  | ISBN | 978-1-4577-0220-4 |
  | Pages | 2696-2699 |
  | Proceedings Title | 2016 38th Annual International Conference of the IEEE Engineering in Medicine and Biology Society (EMBC) |
  | Conference Name | 2016 38th Annual International Conference of the IEEE Engineering in Medicine and Biology Society (EMBC) |
  | DOI | 10.1109/EMBC.2016.7591286 |
  | Date Added | 20/06/2025, 10:03:28 |
  | Modified | 20/06/2025, 10:03:28 |

  ### Attachments

  - PDF
- ## The role of nonlinear coupling in Human-Horse Interaction: A preliminary study

  |  |  |
  | --- | --- |
  | Item Type | Conference Paper |
  | Author | Antonio Lanata |
  | Author | Andrea Guidi |
  | Author | Gaetano Valenza |
  | Author | Paolo Baragli |
  | Author | Enzo Pasquale Scilingo |
  | Date | 7/2017 |
  | Short Title | The role of nonlinear coupling in Human-Horse Interaction |
  | Library Catalogue | DOI.org (Crossref) |
  | URL | https://ieeexplore.ieee.org/document/8037075/ |
  | Accessed | 20/06/2025, 10:04:42 |
  | Place | Seogwipo |
  | Publisher | IEEE |
  | ISBN | 978-1-5090-2809-2 |
  | Pages | 1320-1323 |
  | Proceedings Title | 2017 39th Annual International Conference of the IEEE Engineering in Medicine and Biology Society (EMBC) |
  | Conference Name | 2017 39th Annual International Conference of the IEEE Engineering in Medicine and Biology Society (EMBC) |
  | DOI | 10.1109/EMBC.2017.8037075 |
  | Date Added | 20/06/2025, 10:04:42 |
  | Modified | 20/06/2025, 10:04:42 |

  ### Attachments

  - PDF
- ## The Bond Between a Horse and a Human

  |  |  |
  | --- | --- |
  | Item Type | Journal Article |
  | Author | Debbie Crews |
  | Abstract | The bond that exists between a horse and human was examined using EEG from the horse and human simultaneously. Three volunteers ranging from novice to elite horse experience participated with an unfamiliar horse. The elite participant was also recorded with her own horse. A dose-response effect was tested using 6 conditions requiring increasing interaction between the horse and human (baseline – apart, standing together, petting, grooming, sitting, and riding). |
  | Date | 2009-07-24 |
  | Language | en |
  | Library Catalogue | DOI.org (Crossref) |
  | URL | https://www.nature.com/articles/npre.2009.3454.1 |
  | Accessed | 24/06/2025, 09:07:15 |
  | Publication | Nature Precedings |
  | DOI | 10.1038/npre.2009.3454.1 |
  | Journal Abbr | Nat Prec |
  | ISSN | 1756-0357 |
  | Date Added | 24/06/2025, 09:07:15 |
  | Modified | 24/06/2025, 09:07:15 |

  ### Attachments

  - PDF
- ## Unveiling directional physiological coupling in human-horse interactions

  |  |  |
  | --- | --- |
  | Item Type | Journal Article |
  | Author | Alejandro Luis Callara |
  | Author | Chiara Scopa |
  | Author | Laura Contalbrigo |
  | Author | Antonio Lanatà |
  | Author | Enzo Pasquale Scilingo |
  | Author | Paolo Baragli |
  | Author | Alberto Greco |
  | Date | 09/2024 |
  | Language | en |
  | Library Catalogue | DOI.org (Crossref) |
  | URL | https://linkinghub.elsevier.com/retrieve/pii/S2589004224020820 |
  | Accessed | 20/06/2025, 10:02:13 |
  | Volume | 27 |
  | Pages | 110857 |
  | Publication | iScience |
  | DOI | 10.1016/j.isci.2024.110857 |
  | Issue | 9 |
  | Journal Abbr | iScience |
  | ISSN | 25890042 |
  | Date Added | 20/06/2025, 10:02:13 |
  | Modified | 20/06/2025, 10:02:13 |

  ### Attachments

  - Full Text
