# Supplementary material for: Evidence of Physiological Comodulation During Human–Animal Interaction: A Systematic Review
Source: Ann N Y Acad Sci. 2026 Jun 4;1560(1):e70299. doi: 10.1111/nyas.70299 (PMC13238372; doi:10.1111/nyas.70299)
Supplement: Supplementary file 2 — Supplementary Materials: Supp2‐Zotero‐Collection.zip [file NYAS-1560-0-s002.zip › Supp2_Zotero_Collection/title screened/Animal Study Repository.htm]

Zotero Report


- ## Searching: WBI Studies Repository

  |  |  |
  | --- | --- |
  | Item Type | Web Page |
  | URL | https://www.wellbeingintlstudiesrepository.org/do/search/?q=%22human%20animal%20interaction%22%20AND%20%22fNIRS%22%20&start=0&context=4157404&facet= |
  | Accessed | 11/07/2025, 12:25:33 |
  | Date Added | 11/07/2025, 12:25:33 |
  | Modified | 11/07/2025, 12:25:33 |

  ### Attachments

  - Searching: WBI Studies Repository
- ## Searching: WBI Studies Repository

  |  |  |
  | --- | --- |
  | Item Type | Web Page |
  | URL | https://www.wellbeingintlstudiesrepository.org/do/search/?q=%22human%20animal%20interaction%22%20AND%20%22cortisol%22%20&start=0&context=4157404&facet= |
  | Accessed | 11/07/2025, 14:24:41 |
  | Date Added | 11/07/2025, 14:24:41 |
  | Modified | 11/07/2025, 14:24:41 |

  ### Attachments

  - Searching: WBI Studies Repository
- ## Searching: WBI Studies Repository

  |  |  |
  | --- | --- |
  | Item Type | Web Page |
  | URL | https://www.wellbeingintlstudiesrepository.org/do/search/?q=%22human%20animal%20interaction%22%20AND%20%22EEG%22&start=0&context=4157404&facet= |
  | Accessed | 16/07/2025, 12:02:45 |
  | Date Added | 16/07/2025, 12:02:45 |
  | Modified | 16/07/2025, 12:02:45 |

  ### Attachments

  - Searching: WBI Studies Repository
- ## Searching: WBI Studies Repository

  |  |  |
  | --- | --- |
  | Item Type | Web Page |
  | URL | https://www.wellbeingintlstudiesrepository.org/do/search/?q=%22human%20animal%20interaction%22%20AND%20%22PPG%22&start=0&context=4157404&facet= |
  | Accessed | 16/07/2025, 12:03:57 |
  | Date Added | 16/07/2025, 12:03:57 |
  | Modified | 16/07/2025, 12:03:57 |

  ### Attachments

  - Searching: WBI Studies Repository
- ## Searching: WBI Studies Repository

  |  |  |
  | --- | --- |
  | Item Type | Web Page |
  | URL | https://www.wellbeingintlstudiesrepository.org/do/search/?q=%22human%20animal%20interaction%22%20AND%20%22fNIRS%22&start=0&context=4157404&facet= |
  | Accessed | 16/07/2025, 12:04:42 |
  | Date Added | 16/07/2025, 12:04:42 |
  | Modified | 16/07/2025, 12:04:42 |

  ### Attachments

  - Searching: WBI Studies Repository
- ## Searching: WBI Studies Repository

  |  |  |
  | --- | --- |
  | Item Type | Web Page |
  | URL | https://www.wellbeingintlstudiesrepository.org/do/search/?q=%22human%20animal%20interaction%22%20AND%20%22heart%20rate%22&start=0&context=4157404&facet= |
  | Accessed | 16/07/2025, 12:05:22 |
  | Date Added | 16/07/2025, 12:05:22 |
  | Modified | 16/07/2025, 12:05:22 |

  ### Attachments

  - Searching: WBI Studies Repository
- ## Searching: WBI Studies Repository

  |  |  |
  | --- | --- |
  | Item Type | Web Page |
  | URL | https://www.wellbeingintlstudiesrepository.org/do/search/?q=%22human%20animal%20interaction%22%20AND%20%22oxytocin%22&start=0&context=4157404&facet= |
  | Accessed | 16/07/2025, 12:06:54 |
  | Date Added | 16/07/2025, 12:06:54 |
  | Modified | 16/07/2025, 12:06:54 |

  ### Attachments

  - Searching: WBI Studies Repository
- ## Searching: WBI Studies Repository

  |  |  |
  | --- | --- |
  | Item Type | Web Page |
  | URL | https://www.wellbeingintlstudiesrepository.org/do/search/?q=%22human%20animal%20interaction%22%20AND%20%22breath%22&start=0&context=4157404&facet= |
  | Accessed | 16/07/2025, 12:07:57 |
  | Date Added | 16/07/2025, 12:07:57 |
  | Modified | 16/07/2025, 12:07:57 |

  ### Attachments

  - Searching: WBI Studies Repository
