# Supplementary material for: Evidence of Physiological Comodulation During Human–Animal Interaction: A Systematic Review
Source: Ann N Y Acad Sci. 2026 Jun 4;1560(1):e70299. doi: 10.1111/nyas.70299 (PMC13238372; doi:10.1111/nyas.70299)
Supplement: Supplementary file 2 — Supplementary Materials: Supp2‐Zotero‐Collection.zip [file NYAS-1560-0-s002.zip › Supp2_Zotero_Collection/title screened/Cochrane.htm]

Zotero Report


- ## Search | Cochrane Library

  |  |  |
  | --- | --- |
  | Item Type | Web Page |
  | Language | en-US |
  | URL | https://www.cochranelibrary.com/search?p\_p\_id=scolarissearchresultsportlet\_WAR\_scolarissearchresults&p\_p\_lifecycle=0&\_scolarissearchresultsportlet\_WAR\_scolarissearchresults\_searchType=basic&\_scolarissearchresultsportlet\_WAR\_scolarissearchresults\_searchBy=1&\_scolarissearchresultsportlet\_WAR\_scolarissearchresults\_searchText=%22human+animal+interaction%22+AND+%22physiological+measures%22+ |
  | Accessed | 11/07/2025, 12:16:10 |
  | Date Added | 11/07/2025, 12:16:10 |
  | Modified | 11/07/2025, 12:16:10 |

  ### Attachments

  - Snapshot
- ## Search | Cochrane Library

  |  |  |
  | --- | --- |
  | Item Type | Web Page |
  | Language | en-US |
  | URL | https://www.cochranelibrary.com/search?p\_p\_id=scolarissearchresultsportlet\_WAR\_scolarissearchresults&p\_p\_lifecycle=0&\_scolarissearchresultsportlet\_WAR\_scolarissearchresults\_searchType=basic&\_scolarissearchresultsportlet\_WAR\_scolarissearchresults\_searchBy=1&\_scolarissearchresultsportlet\_WAR\_scolarissearchresults\_searchText=%22human+animal+interaction%22+AND+%22heart+rate%22+ |
  | Accessed | 11/07/2025, 12:17:36 |
  | Date Added | 11/07/2025, 12:17:36 |
  | Modified | 11/07/2025, 12:17:36 |

  ### Attachments

  - Snapshot
- ## Search | Cochrane Library

  |  |  |
  | --- | --- |
  | Item Type | Web Page |
  | Language | en-US |
  | URL | https://www.cochranelibrary.com/search?p\_p\_id=scolarissearchresultsportlet\_WAR\_scolarissearchresults&p\_p\_lifecycle=0&\_scolarissearchresultsportlet\_WAR\_scolarissearchresults\_searchType=basic&\_scolarissearchresultsportlet\_WAR\_scolarissearchresults\_searchBy=1&\_scolarissearchresultsportlet\_WAR\_scolarissearchresults\_searchText=%22human+animal+interaction%22+AND+%22EEG%22+ |
  | Accessed | 11/07/2025, 12:18:28 |
  | Date Added | 11/07/2025, 12:18:28 |
  | Modified | 11/07/2025, 12:18:28 |

  ### Attachments

  - Snapshot
- ## Search | Cochrane Library

  |  |  |
  | --- | --- |
  | Item Type | Web Page |
  | Language | en-US |
  | URL | https://www.cochranelibrary.com/search?p\_p\_id=scolarissearchresultsportlet\_WAR\_scolarissearchresults&p\_p\_lifecycle=0&\_scolarissearchresultsportlet\_WAR\_scolarissearchresults\_searchType=basic&\_scolarissearchresultsportlet\_WAR\_scolarissearchresults\_searchBy=1&\_scolarissearchresultsportlet\_WAR\_scolarissearchresults\_searchText=%22human+animal+interaction%22+AND+%22PPG%22 |
  | Accessed | 11/07/2025, 12:18:43 |
  | Date Added | 11/07/2025, 12:18:43 |
  | Modified | 11/07/2025, 12:18:43 |

  ### Attachments

  - Snapshot
- ## Search | Cochrane Library

  |  |  |
  | --- | --- |
  | Item Type | Web Page |
  | Language | en-US |
  | URL | https://www.cochranelibrary.com/search?p\_p\_id=scolarissearchresultsportlet\_WAR\_scolarissearchresults&p\_p\_lifecycle=0&\_scolarissearchresultsportlet\_WAR\_scolarissearchresults\_searchType=basic&\_scolarissearchresultsportlet\_WAR\_scolarissearchresults\_searchBy=1&\_scolarissearchresultsportlet\_WAR\_scolarissearchresults\_searchText=%22human+animal+interaction%22+AND+%22fNIRS%22 |
  | Accessed | 11/07/2025, 12:19:00 |
  | Date Added | 11/07/2025, 12:19:00 |
  | Modified | 11/07/2025, 12:19:00 |

  ### Attachments

  - Snapshot
- ## Search | Cochrane Library

  |  |  |
  | --- | --- |
  | Item Type | Web Page |
  | Language | en-US |
  | URL | https://www.cochranelibrary.com/search?p\_p\_id=scolarissearchresultsportlet\_WAR\_scolarissearchresults&p\_p\_lifecycle=0&\_scolarissearchresultsportlet\_WAR\_scolarissearchresults\_searchType=basic&\_scolarissearchresultsportlet\_WAR\_scolarissearchresults\_searchBy=1&\_scolarissearchresultsportlet\_WAR\_scolarissearchresults\_searchText=%22human+animal+interaction%22+AND+%22oxytocin |
  | Accessed | 11/07/2025, 12:19:27 |
  | Date Added | 11/07/2025, 12:19:27 |
  | Modified | 11/07/2025, 12:19:27 |

  ### Attachments

  - Snapshot
