# Supplementary material for: Evidence of Physiological Comodulation During Human–Animal Interaction: A Systematic Review
Source: Ann N Y Acad Sci. 2026 Jun 4;1560(1):e70299. doi: 10.1111/nyas.70299 (PMC13238372; doi:10.1111/nyas.70299)
Supplement: Supplementary file 2 — Supplementary Materials: Supp2‐Zotero‐Collection.zip [file NYAS-1560-0-s002.zip › Supp2_Zotero_Collection/title screened/Consensus - EEG prompt.htm]

Zotero Report


- ## Cross-Species Investigation on Resting State Electroencephalogram

  |  |  |
  | --- | --- |
  | Item Type | Journal Article |
  | Author | Fengrui Zhang |
  | Author | Feixue Wang |
  | Author | Lupeng Yue |
  | Author | Huijuan Zhang |
  | Author | W. Peng |
  | Author | Li Hu |
  | Date | 2019-07-04 |
  | URL | https://consensus.app/papers/crossspecies-investigation-on-resting-state-peng-hu/725a199f288f5aef9b7424546598a823/ |
  | Volume | 32 |
  | Pages | 808-824 |
  | Publication | Brain Topography |
  | DOI | 10.1007/s10548-019-00723-x |
  | Journal Abbr | Brain Topography |
  | Date Added | 11/07/2025, 13:09:23 |
  | Modified | 11/07/2025, 13:09:23 |
- ## The State of Research on Human–Animal Relations: Implications for Human Health

  |  |  |
  | --- | --- |
  | Item Type | Journal Article |
  | Author | D. Wells |
  | Date | 2019-03-04 |
  | URL | https://consensus.app/papers/the-state-of-research-on-human%E2%80%93animal-relations-wells/d57ae41124365ae0b531560b06a8400f/ |
  | Volume | 32 |
  | Pages | 169-181 |
  | Publication | Anthrozoös |
  | DOI | 10.1080/08927936.2019.1569902 |
  | Journal Abbr | Anthrozoös |
  | Date Added | 11/07/2025, 13:09:23 |
  | Modified | 11/07/2025, 13:09:23 |
- ## Psychometric Evaluation of the Comfort from Companion Animals Scale in a Sexual and Gender Minority Sample

  |  |  |
  | --- | --- |
  | Item Type | Journal Article |
  | Author | Camie Tomlinson |
  | Author | Sarah Pittman |
  | Author | Jennifer Murphy |
  | Author | Angela Matijczak |
  | Author | S. McDonald |
  | Date | 2021-08-26 |
  | URL | https://consensus.app/papers/psychometric-evaluation-of-the-comfort-from-companion-matijczak-mcdonald/c3c149ee4c17564f82a51f37e29354ab/ |
  | Volume | 35 |
  | Pages | 143-163 |
  | Publication | Anthrozoös |
  | DOI | 10.1080/08927936.2021.1963548 |
  | Journal Abbr | Anthrozoös |
  | Date Added | 11/07/2025, 13:09:23 |
  | Modified | 11/07/2025, 13:09:23 |
- ## The Power of a Positive Human–Animal Relationship for Animal Welfare

  |  |  |
  | --- | --- |
  | Item Type | Journal Article |
  | Author | J. Rault |
  | Author | S. Waiblinger |
  | Author | X. Boivin |
  | Author | P. Hemsworth |
  | Date | 2020-11-09 |
  | URL | https://consensus.app/papers/the-power-of-a-positive-human%E2%80%93animal-relationship-for-boivin-hemsworth/64c125aa43f55555ba71107faa821f1e/ |
  | Volume | 7 |
  | Publication | Frontiers in Veterinary Science |
  | DOI | 10.3389/fvets.2020.590867 |
  | Journal Abbr | Frontiers in Veterinary Science |
  | Date Added | 11/07/2025, 13:09:23 |
  | Modified | 11/07/2025, 13:09:23 |
- ## Neural Basis of Categorical Representations of Animal Body Silhouettes.

  |  |  |
  | --- | --- |
  | Item Type | Journal Article |
  | Author | Yue Pu |
  | Author | Shihui Han |
  | Date | 2024-07-26 |
  | URL | https://consensus.app/papers/neural-basis-of-categorical-representations-of-animal-han-pu/e57fe935e5b15f8082550397a4719bcb/ |
  | Publication | Neuroscience bulletin |
  | DOI | 10.1007/s12264-024-01268-1 |
  | Journal Abbr | Neuroscience bulletin |
  | Date Added | 11/07/2025, 13:09:23 |
  | Modified | 11/07/2025, 13:09:23 |
- ## The 2020 Five Domains Model: Including Human–Animal Interactions in Assessments of Animal Welfare

  |  |  |
  | --- | --- |
  | Item Type | Journal Article |
  | Author | D. Mellor |
  | Author | N. Beausoleil |
  | Author | K. Littlewood |
  | Author | A. McLean |
  | Author | P. McGreevy |
  | Author | B. Jones |
  | Author | C. Wilkins |
  | Date | 2020-10-01 |
  | URL | https://consensus.app/papers/the-2020-five-domains-model-including-human%E2%80%93animal-mellor-wilkins/c390aac677b45a2ebbde016e12b73522/ |
  | Volume | 10 |
  | Publication | Animals : an Open Access Journal from MDPI |
  | DOI | 10.3390/ani10101870 |
  | Journal Abbr | Animals : an Open Access Journal from MDPI |
  | Date Added | 11/07/2025, 13:09:23 |
  | Modified | 11/07/2025, 13:09:23 |
- ## Human-Animal Interactions

  |  |  |
  | --- | --- |
  | Item Type | Journal Article |
  | Author | Megan LaFollette |
  | Date | 2020-09-09 |
  | URL | https://consensus.app/papers/humananimal-interactions-lafollette/1f21ef9cfd055b07a0d7afebc6db76f8/ |
  | Publication | Animal-centric Care and Management |
  | DOI | 10.1201/9780429059544-1 |
  | Journal Abbr | Animal-centric Care and Management |
  | Date Added | 11/07/2025, 13:09:23 |
  | Modified | 11/07/2025, 13:09:23 |
- ## Electroencephalography Measurements in Awake Marmosets Listening to Conspecific Vocalizations.

  |  |  |
  | --- | --- |
  | Item Type | Journal Article |
  | Author | Naho Konoike |
  | Author | Miki Miwa |
  | Author | Kosuke Itoh |
  | Author | Katsuki Nakamura |
  | Date | 2024-07-26 |
  | URL | https://consensus.app/papers/electroencephalography-measurements-in-awake-marmosets-konoike-itoh/f242033d909c503aa9d60eb382153f0b/ |
  | Volume | 209 |
  | Publication | Journal of visualized experiments : JoVE |
  | DOI | 10.3791/66869 |
  | Journal Abbr | Journal of visualized experiments : JoVE |
  | Date Added | 11/07/2025, 13:09:23 |
  | Modified | 11/07/2025, 13:09:23 |
- ## The Human-Animal Interaction at Work Scale: Development and psychometric properties

  |  |  |
  | --- | --- |
  | Item Type | Journal Article |
  | Author | A. Junça‐Silva |
  | Date | 2024-07-01 |
  | URL | https://consensus.app/papers/the-humananimal-interaction-at-work-scale-development-and-jun%C3%A7a%E2%80%90silva/44d95ba5ee9459d8b057a28ea754c998/ |
  | Publication | Journal of Veterinary Behavior |
  | DOI | 10.1016/j.jveb.2024.06.007 |
  | Journal Abbr | Journal of Veterinary Behavior |
  | Date Added | 11/07/2025, 13:09:23 |
  | Modified | 11/07/2025, 13:09:23 |
- ## Measurement of attachment in human-animal interaction research

  |  |  |
  | --- | --- |
  | Item Type | Journal Article |
  | Author | Eli Halbreich |
  | Author | Tristen Hefner |
  | Author | Ashly Healy |
  | Author | Jason Van Allen |
  | Date | 2024-09-25 |
  | URL | https://consensus.app/papers/measurement-of-attachment-in-humananimal-interaction-allen-halbreich/54b1023aa33657699ba75874ecd76bfd/ |
  | Publication | Human-Animal Interactions |
  | DOI | 10.1079/hai.2024.0030 |
  | Journal Abbr | Human-Animal Interactions |
  | Date Added | 11/07/2025, 13:09:23 |
  | Modified | 11/07/2025, 13:09:23 |
- ## Variability in Human-Animal Interaction Research

  |  |  |
  | --- | --- |
  | Item Type | Journal Article |
  | Author | N. Gee |
  | Author | Kerri Rodriguez |
  | Author | H. Herzog |
  | Date | 2021-01-15 |
  | URL | https://consensus.app/papers/variability-in-humananimal-interaction-research-gee-rodriguez/f107e4d7aae8564db4918abed4d35d13/ |
  | Volume | 7 |
  | Publication | Frontiers in Veterinary Science |
  | DOI | 10.3389/fvets.2020.619600 |
  | Journal Abbr | Frontiers in Veterinary Science |
  | Date Added | 17/06/2025, 18:30:59 |
  | Modified | 04/01/2026, 11:26:12 |
- ## Human–Animal Interaction and Older Adults: An Overview

  |  |  |
  | --- | --- |
  | Item Type | Journal Article |
  | Author | N. Gee |
  | Author | M. Mueller |
  | Author | A. Curl |
  | Date | 2017-08-21 |
  | URL | https://consensus.app/papers/human%E2%80%93animal-interaction-and-older-adults-an-overview-curl-mueller/62cd68a37a515eba81be204222b6d800/ |
  | Volume | 8 |
  | Publication | Frontiers in Psychology |
  | DOI | 10.3389/fpsyg.2017.01416 |
  | Journal Abbr | Frontiers in Psychology |
  | Date Added | 11/07/2025, 13:09:23 |
  | Modified | 11/07/2025, 13:09:23 |
- ## A Systematic Review of Research on Pet Ownership and Animal Interactions among Older Adults

  |  |  |
  | --- | --- |
  | Item Type | Journal Article |
  | Author | N. Gee |
  | Author | M. Mueller |
  | Date | 2019-03-04 |
  | URL | https://consensus.app/papers/a-systematic-review-of-research-on-pet-ownership-and-animal-gee-mueller/a8fcdd6946a65fb09e8e634b91d4c228/ |
  | Volume | 32 |
  | Pages | 183-207 |
  | Publication | Anthrozoös |
  | DOI | 10.1080/08927936.2019.1569903 |
  | Journal Abbr | Anthrozoös |
  | Date Added | 11/07/2025, 13:09:23 |
  | Modified | 11/07/2025, 13:09:23 |
- ## The Human–Animal Interaction Scale: Development and Evaluation

  |  |  |
  | --- | --- |
  | Item Type | Journal Article |
  | Author | Angela Fournier |
  | Author | T. Berry |
  | Author | Elizabeth Letson |
  | Author | Ryan Chanen |
  | Date | 2016-08-17 |
  | URL | https://consensus.app/papers/the-human%E2%80%93animal-interaction-scale-development-and-fournier-letson/c4455d7449d7509c81fe89ac5f87a63c/ |
  | Volume | 29 |
  | Pages | 455-467 |
  | Publication | Anthrozoös |
  | DOI | 10.1080/08927936.2016.1181372 |
  | Journal Abbr | Anthrozoös |
  | Date Added | 11/07/2025, 13:09:23 |
  | Modified | 11/07/2025, 13:09:23 |
- ## Human–Animal Interaction Analysis

  |  |  |
  | --- | --- |
  | Item Type | Journal Article |
  | Author | Angela Fournier |
  | Date | 2019-01-01 |
  | URL | https://consensus.app/papers/human%E2%80%93animal-interaction-analysis-fournier/f0d745c27c6c512b956474c61ef450a8/ |
  | Publication | Animal-Assisted Intervention |
  | DOI | 10.1007/978-3-030-32972-3\_2 |
  | Journal Abbr | Animal-Assisted Intervention |
  | Date Added | 11/07/2025, 13:09:23 |
  | Modified | 11/07/2025, 13:09:23 |
- ## Pharmaco-EEG Studies in Animals: An Overview of Contemporary Translational Applications

  |  |  |
  | --- | --- |
  | Item Type | Journal Article |
  | Author | W. Drinkenburg |
  | Author | G. Ruigt |
  | Author | A. Ahnaou |
  | Date | 2016-02-01 |
  | URL | https://consensus.app/papers/pharmacoeeg-studies-in-animals-an-overview-of-drinkenburg-ruigt/c6537a3d636654b8a13f9352db77d87c/ |
  | Volume | 72 |
  | Pages | 151-164 |
  | Publication | Neuropsychobiology |
  | DOI | 10.1159/000442210 |
  | Journal Abbr | Neuropsychobiology |
  | Date Added | 11/07/2025, 13:09:23 |
  | Modified | 11/07/2025, 13:09:23 |
- ## Pharmaco-EEG Studies in Animals: A History-Based Introduction to Contemporary Translational Applications

  |  |  |
  | --- | --- |
  | Item Type | Journal Article |
  | Author | W. Drinkenburg |
  | Author | A. Ahnaou |
  | Author | G. Ruigt |
  | Date | 2016-02-01 |
  | URL | https://consensus.app/papers/pharmacoeeg-studies-in-animals-a-historybased-ahnaou-drinkenburg/e162c82fa11855308e70d1b44143f791/ |
  | Volume | 72 |
  | Pages | 139-150 |
  | Publication | Neuropsychobiology |
  | DOI | 10.1159/000443175 |
  | Journal Abbr | Neuropsychobiology |
  | Date Added | 11/07/2025, 13:09:23 |
  | Modified | 11/07/2025, 13:09:23 |
- ## How to Measure Human-Dog Interaction in Dog Assisted Interventions? A Scoping Review

  |  |  |
  | --- | --- |
  | Item Type | Journal Article |
  | Author | Marta De Santis |
  | Author | L. Filugelli |
  | Author | Alberto Mair |
  | Author | Simona Normando |
  | Author | F. Mutinelli |
  | Author | L. Contalbrigo |
  | Date | 2024-01-26 |
  | URL | https://consensus.app/papers/how-to-measure-humandog-interaction-in-dog-assisted-mutinelli-santis/165b6e269f695f6fa43345168729b0df/ |
  | Volume | 14 |
  | Publication | Animals : an Open Access Journal from MDPI |
  | DOI | 10.3390/ani14030410 |
  | Journal Abbr | Animals : an Open Access Journal from MDPI |
  | Date Added | 11/07/2025, 13:09:23 |
  | Modified | 11/07/2025, 13:09:23 |
- ## Solidarity with Animals: Assessing a Relevant Dimension of Social Identification with Animals

  |  |  |
  | --- | --- |
  | Item Type | Journal Article |
  | Author | Catherine Amiot |
  | Author | B. Bastian |
  | Date | 2017-01-03 |
  | URL | https://consensus.app/papers/solidarity-with-animals-assessing-a-relevant-dimension-of-bastian-amiot/ac8b6a7975d85afd957959cadd167336/ |
  | Volume | 12 |
  | Publication | PLoS ONE |
  | DOI | 10.1371/journal.pone.0168184 |
  | Journal Abbr | PLoS ONE |
  | Date Added | 11/07/2025, 13:09:23 |
  | Modified | 11/07/2025, 13:09:23 |
