# Supplementary material for: Evidence of Physiological Comodulation During Human–Animal Interaction: A Systematic Review
Source: Ann N Y Acad Sci. 2026 Jun 4;1560(1):e70299. doi: 10.1111/nyas.70299 (PMC13238372; doi:10.1111/nyas.70299)
Supplement: Supplementary file 2 — Supplementary Materials: Supp2‐Zotero‐Collection.zip [file NYAS-1560-0-s002.zip › Supp2_Zotero_Collection/title screened/Consensus - fNIRS prompt.htm]

Zotero Report


- ## A Synchrony-Dependent Influence of Sounds on Activity in Visual Cortex Measured Using Functional Near-Infrared Spectroscopy (fNIRS)

  |  |  |
  | --- | --- |
  | Item Type | Journal Article |
  | Author | I. Wiggins |
  | Author | D. Hartley |
  | Date | 2015-03-31 |
  | URL | https://consensus.app/papers/a-synchronydependent-influence-of-sounds-on-activity-in-hartley-wiggins/8d08e03c74745e9d950f1d01599089cd/ |
  | Volume | 10 |
  | Publication | PLoS ONE |
  | DOI | 10.1371/journal.pone.0122862 |
  | Journal Abbr | PLoS ONE |
  | Date Added | 11/07/2025, 13:11:55 |
  | Modified | 11/07/2025, 13:11:55 |
- ## The State of Research on Human–Animal Relations: Implications for Human Health

  |  |  |
  | --- | --- |
  | Item Type | Journal Article |
  | Author | D. Wells |
  | Date | 2019-03-04 |
  | URL | https://consensus.app/papers/the-state-of-research-on-human%E2%80%93animal-relations-wells/d57ae41124365ae0b531560b06a8400f/ |
  | Volume | 32 |
  | Pages | 169-181 |
  | Publication | Anthrozoös |
  | DOI | 10.1080/08927936.2019.1569902 |
  | Journal Abbr | Anthrozoös |
  | Date Added | 11/07/2025, 13:11:55 |
  | Modified | 11/07/2025, 13:11:55 |
- ## Perceiving emotions in human–human and human–animal interactions: Hemodynamic prefrontal activity (fNIRS) and empathic concern

  |  |  |
  | --- | --- |
  | Item Type | Journal Article |
  | Author | M. Vanutelli |
  | Author | M. Balconi |
  | Date | 2015-09-25 |
  | URL | https://consensus.app/papers/perceiving-emotions-in-human%E2%80%93human-and-human%E2%80%93animal-vanutelli-balconi/efc444907a2451af8d71e57c2b909ce0/ |
  | Volume | 605 |
  | Pages | 1-6 |
  | Publication | Neuroscience Letters |
  | DOI | 10.1016/j.neulet.2015.07.020 |
  | Journal Abbr | Neuroscience Letters |
  | Date Added | 11/07/2025, 13:11:55 |
  | Modified | 11/07/2025, 13:11:55 |
- ## Psychometric Evaluation of the Comfort from Companion Animals Scale in a Sexual and Gender Minority Sample

  |  |  |
  | --- | --- |
  | Item Type | Journal Article |
  | Author | Camie Tomlinson |
  | Author | Sarah Pittman |
  | Author | Jennifer Murphy |
  | Author | Angela Matijczak |
  | Author | S. McDonald |
  | Date | 2021-08-26 |
  | URL | https://consensus.app/papers/psychometric-evaluation-of-the-comfort-from-companion-matijczak-mcdonald/c3c149ee4c17564f82a51f37e29354ab/ |
  | Volume | 35 |
  | Pages | 143-163 |
  | Publication | Anthrozoös |
  | DOI | 10.1080/08927936.2021.1963548 |
  | Journal Abbr | Anthrozoös |
  | Date Added | 11/07/2025, 13:11:55 |
  | Modified | 11/07/2025, 13:11:55 |
- ## The Power of a Positive Human–Animal Relationship for Animal Welfare

  |  |  |
  | --- | --- |
  | Item Type | Journal Article |
  | Author | J. Rault |
  | Author | S. Waiblinger |
  | Author | X. Boivin |
  | Author | P. Hemsworth |
  | Date | 2020-11-09 |
  | URL | https://consensus.app/papers/the-power-of-a-positive-human%E2%80%93animal-relationship-for-boivin-hemsworth/64c125aa43f55555ba71107faa821f1e/ |
  | Volume | 7 |
  | Publication | Frontiers in Veterinary Science |
  | DOI | 10.3389/fvets.2020.590867 |
  | Journal Abbr | Frontiers in Veterinary Science |
  | Date Added | 11/07/2025, 13:11:55 |
  | Modified | 11/07/2025, 13:11:55 |
- ## Functional Near-Infrared Spectroscopy (fNIRS) for Assessing Cerebral Cortex Function During Human Behavior in Natural/Social Situations: A Concise Review

  |  |  |
  | --- | --- |
  | Item Type | Journal Article |
  | Author | V. Quaresima |
  | Author | M. Ferrari |
  | Date | 2016-07-18 |
  | URL | https://consensus.app/papers/functional-nearinfrared-spectroscopy-fnirs-for-quaresima-ferrari/b9c6e8c460445ac5bec610bafc80b64f/ |
  | Volume | 22 |
  | Pages | 46-68 |
  | Publication | Organizational Research Methods |
  | DOI | 10.1177/1094428116658959 |
  | Journal Abbr | Organizational Research Methods |
  | Date Added | 11/07/2025, 13:11:55 |
  | Modified | 11/07/2025, 13:11:55 |
- ## Analyzing Human-Animal Relationship Measures

  |  |  |
  | --- | --- |
  | Item Type | Journal Article |
  | Author | R. Poresky |
  | Date | 1989-12-01 |
  | URL | https://consensus.app/papers/analyzing-humananimal-relationship-measures-poresky/a559200da86a5a89ac84b66ad93e0d6e/ |
  | Volume | 2 |
  | Pages | 236-244 |
  | Publication | Anthrozoos |
  | DOI | 10.2752/089279389787057911 |
  | Journal Abbr | Anthrozoos |
  | Date Added | 11/07/2025, 13:11:55 |
  | Modified | 11/07/2025, 13:11:55 |
- ## Redefining human-animal relationships: an evaluation of methods to allow their empirical measurement in zoos

  |  |  |
  | --- | --- |
  | Item Type | Journal Article |
  | Author | F. Patel |
  | Author | K. Whitehouse-Tedd |
  | Author | S. Ward |
  | Date | 2019-08-01 |
  | URL | https://consensus.app/papers/redefining-humananimal-relationships-an-evaluation-of-ward-patel/00206af33edc515fabc0d6d8fe95286a/ |
  | Publication | Animal Welfare |
  | DOI | 10.7120/109627286.28.3.247 |
  | Journal Abbr | Animal Welfare |
  | Date Added | 11/07/2025, 13:11:55 |
  | Modified | 11/07/2025, 13:11:55 |
- ## The 2020 Five Domains Model: Including Human–Animal Interactions in Assessments of Animal Welfare

  |  |  |
  | --- | --- |
  | Item Type | Journal Article |
  | Author | D. Mellor |
  | Author | N. Beausoleil |
  | Author | K. Littlewood |
  | Author | A. McLean |
  | Author | P. McGreevy |
  | Author | B. Jones |
  | Author | C. Wilkins |
  | Date | 2020-10-01 |
  | URL | https://consensus.app/papers/the-2020-five-domains-model-including-human%E2%80%93animal-mellor-wilkins/c390aac677b45a2ebbde016e12b73522/ |
  | Volume | 10 |
  | Publication | Animals : an Open Access Journal from MDPI |
  | DOI | 10.3390/ani10101870 |
  | Journal Abbr | Animals : an Open Access Journal from MDPI |
  | Date Added | 11/07/2025, 13:11:55 |
  | Modified | 11/07/2025, 13:11:55 |
- ## Shining new light on sensory brain activation and physiological measurement in seals using wearable optical technology

  |  |  |
  | --- | --- |
  | Item Type | Journal Article |
  | Author | J. McKnight |
  | Author | Alexander Ruesch |
  | Author | K. Bennett |
  | Author | Mathijs Bronkhorst |
  | Author | Steve Balfour |
  | Author | S. Moss |
  | Author | R. Milne |
  | Author | P. Tyack |
  | Author | J. Kainerstorfer |
  | Author | G. Hastie |
  | Date | 2021-06-14 |
  | URL | https://consensus.app/papers/shining-new-light-on-sensory-brain-activation-and-mcknight-ruesch/a96672958db55fe2a4229756c4f7023d/ |
  | Volume | 376 |
  | Publication | Philosophical Transactions of the Royal Society B |
  | DOI | 10.1098/rstb.2020.0224 |
  | Journal Abbr | Philosophical Transactions of the Royal Society B |
  | Date Added | 11/07/2025, 13:11:55 |
  | Modified | 11/07/2025, 13:11:55 |
- ## Application of Functional Near-Infrared Spectroscopy to the Study of Brain Function in Humans and Animal Models

  |  |  |
  | --- | --- |
  | Item Type | Journal Article |
  | Author | H. Kim |
  | Author | Kain Seo |
  | Author | H. Jeon |
  | Author | Unjoo Lee |
  | Author | Hyosang Lee |
  | Date | 2017-08-01 |
  | URL | https://consensus.app/papers/application-of-functional-nearinfrared-spectroscopy-to-jeon-lee/b60ab03acc1d5027933a1e027df6fa03/ |
  | Volume | 40 |
  | Pages | 523-532 |
  | Publication | Molecules and Cells |
  | DOI | 10.14348/molcells.2017.0153 |
  | Journal Abbr | Molecules and Cells |
  | Date Added | 11/07/2025, 13:11:55 |
  | Modified | 11/07/2025, 13:11:55 |
- ## The Human-Animal Interaction at Work Scale: Development and psychometric properties

  |  |  |
  | --- | --- |
  | Item Type | Journal Article |
  | Author | A. Junça‐Silva |
  | Date | 2024-07-01 |
  | URL | https://consensus.app/papers/the-humananimal-interaction-at-work-scale-development-and-jun%C3%A7a%E2%80%90silva/44d95ba5ee9459d8b057a28ea754c998/ |
  | Publication | Journal of Veterinary Behavior |
  | DOI | 10.1016/j.jveb.2024.06.007 |
  | Journal Abbr | Journal of Veterinary Behavior |
  | Date Added | 11/07/2025, 13:11:55 |
  | Modified | 11/07/2025, 13:11:55 |
- ## Measurement of attachment in human-animal interaction research

  |  |  |
  | --- | --- |
  | Item Type | Journal Article |
  | Author | Eli Halbreich |
  | Author | Tristen Hefner |
  | Author | Ashly Healy |
  | Author | Jason Van Allen |
  | Date | 2024-09-25 |
  | URL | https://consensus.app/papers/measurement-of-attachment-in-humananimal-interaction-allen-halbreich/54b1023aa33657699ba75874ecd76bfd/ |
  | Publication | Human-Animal Interactions |
  | DOI | 10.1079/hai.2024.0030 |
  | Journal Abbr | Human-Animal Interactions |
  | Date Added | 11/07/2025, 13:11:55 |
  | Modified | 11/07/2025, 13:11:55 |
- ## Variability in Human-Animal Interaction Research

  |  |  |
  | --- | --- |
  | Item Type | Journal Article |
  | Author | N. Gee |
  | Author | Kerri Rodriguez |
  | Author | H. Herzog |
  | Date | 2021-01-15 |
  | URL | https://consensus.app/papers/variability-in-humananimal-interaction-research-gee-rodriguez/f107e4d7aae8564db4918abed4d35d13/ |
  | Volume | 7 |
  | Publication | Frontiers in Veterinary Science |
  | DOI | 10.3389/fvets.2020.619600 |
  | Journal Abbr | Frontiers in Veterinary Science |
  | Date Added | 17/06/2025, 18:30:59 |
  | Modified | 04/01/2026, 11:26:12 |
- ## The Human–Animal Interaction Scale: Development and Evaluation

  |  |  |
  | --- | --- |
  | Item Type | Journal Article |
  | Author | Angela Fournier |
  | Author | T. Berry |
  | Author | Elizabeth Letson |
  | Author | Ryan Chanen |
  | Date | 2016-08-17 |
  | URL | https://consensus.app/papers/the-human%E2%80%93animal-interaction-scale-development-and-fournier-letson/c4455d7449d7509c81fe89ac5f87a63c/ |
  | Volume | 29 |
  | Pages | 455-467 |
  | Publication | Anthrozoös |
  | DOI | 10.1080/08927936.2016.1181372 |
  | Journal Abbr | Anthrozoös |
  | Date Added | 11/07/2025, 13:11:55 |
  | Modified | 11/07/2025, 13:11:55 |
- ## Human–Animal Interaction Analysis

  |  |  |
  | --- | --- |
  | Item Type | Journal Article |
  | Author | Angela Fournier |
  | Date | 2019-01-01 |
  | URL | https://consensus.app/papers/human%E2%80%93animal-interaction-analysis-fournier/f0d745c27c6c512b956474c61ef450a8/ |
  | Publication | Animal-Assisted Intervention |
  | DOI | 10.1007/978-3-030-32972-3\_2 |
  | Journal Abbr | Animal-Assisted Intervention |
  | Date Added | 11/07/2025, 13:11:55 |
  | Modified | 11/07/2025, 13:11:55 |
- ## A brief review on the history of human functional near-infrared spectroscopy (fNIRS) development and fields of application

  |  |  |
  | --- | --- |
  | Item Type | Journal Article |
  | Author | M. Ferrari |
  | Author | V. Quaresima |
  | Date | 2012-11-01 |
  | URL | https://consensus.app/papers/a-brief-review-on-the-history-of-human-functional-ferrari-quaresima/c84e025da56956cd81512c25ce0a70af/ |
  | Volume | 63 |
  | Pages | 921-935 |
  | Publication | NeuroImage |
  | DOI | 10.1016/j.neuroimage.2012.03.049 |
  | Journal Abbr | NeuroImage |
  | Date Added | 11/07/2025, 13:11:55 |
  | Modified | 11/07/2025, 13:11:55 |
- ## Emotions and BIS/BAS components affect brain activity (ERPs and fNIRS) in observing intra-species and inter-species interactions

  |  |  |
  | --- | --- |
  | Item Type | Journal Article |
  | Author | M. Balconi |
  | Author | M. Vanutelli |
  | Date | 2016-09-01 |
  | URL | https://consensus.app/papers/emotions-and-bisbas-components-affect-brain-activity-erps-balconi-vanutelli/f0e081bc8a9c5bc6b42a74014c0d4f9c/ |
  | Volume | 10 |
  | Pages | 750-760 |
  | Publication | Brain Imaging and Behavior |
  | DOI | 10.1007/s11682-015-9443-z |
  | Journal Abbr | Brain Imaging and Behavior |
  | Date Added | 11/07/2025, 13:11:55 |
  | Modified | 11/07/2025, 13:11:55 |
- ## Hemodynamic (fNIRS) and EEG (N200) correlates of emotional inter-species interactions modulated by visual and auditory stimulation

  |  |  |
  | --- | --- |
  | Item Type | Journal Article |
  | Author | M. Balconi |
  | Author | M. Vanutelli |
  | Date | 2016-03-15 |
  | URL | https://consensus.app/papers/hemodynamic-fnirs-and-eeg-n200-correlates-of-emotional-balconi-vanutelli/7ecb080ad98a540fb0595b60bbd655ca/ |
  | Volume | 6 |
  | Publication | Scientific Reports |
  | DOI | 10.1038/srep23083 |
  | Journal Abbr | Scientific Reports |
  | Date Added | 11/07/2025, 13:11:55 |
  | Modified | 11/07/2025, 13:11:55 |
