# Supplementary material for: Evidence of Physiological Comodulation During Human–Animal Interaction: A Systematic Review
Source: Ann N Y Acad Sci. 2026 Jun 4;1560(1):e70299. doi: 10.1111/nyas.70299 (PMC13238372; doi:10.1111/nyas.70299)
Supplement: Supplementary file 2 — Supplementary Materials: Supp2‐Zotero‐Collection.zip [file NYAS-1560-0-s002.zip › Supp2_Zotero_Collection/title screened/Consensus - heart rate prompt.htm]

Zotero Report


- ## The State of Research on Human–Animal Relations: Implications for Human Health

  |  |  |
  | --- | --- |
  | Item Type | Journal Article |
  | Author | D. Wells |
  | Date | 2019-03-04 |
  | URL | https://consensus.app/papers/the-state-of-research-on-human%E2%80%93animal-relations-wells/d57ae41124365ae0b531560b06a8400f/ |
  | Volume | 32 |
  | Pages | 169-181 |
  | Publication | Anthrozoös |
  | DOI | 10.1080/08927936.2019.1569902 |
  | Journal Abbr | Anthrozoös |
  | Date Added | 11/07/2025, 13:13:12 |
  | Modified | 11/07/2025, 13:13:12 |
- ## Heart rate as a measure of emotional arousal in evolutionary biology

  |  |  |
  | --- | --- |
  | Item Type | Journal Article |
  | Author | C. Wascher |
  | Date | 2021-06-28 |
  | URL | https://consensus.app/papers/heart-rate-as-a-measure-of-emotional-arousal-in-wascher/98029954521653808a6c983631aa7ab3/ |
  | Volume | 376 |
  | Publication | Philosophical Transactions of the Royal Society B |
  | DOI | 10.1098/rstb.2020.0479 |
  | Journal Abbr | Philosophical Transactions of the Royal Society B |
  | Date Added | 11/07/2025, 13:13:12 |
  | Modified | 11/07/2025, 13:13:12 |
- ## Psychophysiological mechanisms underlying the potential health benefits of human-dog interactions: A systematic literature review.

  |  |  |
  | --- | --- |
  | Item Type | Journal Article |
  | Author | J. Teo |
  | Author | S. Johnstone |
  | Author | Stephanie Römer |
  | Author | Susan Thomas |
  | Date | 2022-07-01 |
  | URL | https://consensus.app/papers/psychophysiological-mechanisms-underlying-the-teo-johnstone/cf2e99df9704550d80c9c83dd45426b6/ |
  | Publication | International journal of psychophysiology : official journal of the International Organization of Psychophysiology |
  | DOI | 10.1016/j.ijpsycho.2022.07.007 |
  | Journal Abbr | International journal of psychophysiology : official journal of the International Organization of Psychophysiology |
  | Date Added | 11/07/2025, 13:13:12 |
  | Modified | 11/07/2025, 13:13:12 |
- ## Inside the Interaction: Contact With Familiar Humans Modulates Heart Rate Variability in Horses

  |  |  |
  | --- | --- |
  | Item Type | Journal Article |
  | Author | Chiara Scopa |
  | Author | A. Greco |
  | Author | L. Contalbrigo |
  | Author | Elisabetta Fratini |
  | Author | A. Lanatà |
  | Author | E. Scilingo |
  | Author | P. Baragli |
  | Date | 2020-11-30 |
  | URL | https://consensus.app/papers/inside-the-interaction-contact-with-familiar-humans-greco-fratini/b0eacee91eb65df9ac8c79d1aec26beb/ |
  | Volume | 7 |
  | Publication | Frontiers in Veterinary Science |
  | DOI | 10.3389/fvets.2020.582759 |
  | Journal Abbr | Frontiers in Veterinary Science |
  | Date Added | 11/07/2025, 13:13:12 |
  | Modified | 11/07/2025, 13:13:12 |
- ## The Power of a Positive Human–Animal Relationship for Animal Welfare

  |  |  |
  | --- | --- |
  | Item Type | Journal Article |
  | Author | J. Rault |
  | Author | S. Waiblinger |
  | Author | X. Boivin |
  | Author | P. Hemsworth |
  | Date | 2020-11-09 |
  | URL | https://consensus.app/papers/the-power-of-a-positive-human%E2%80%93animal-relationship-for-boivin-hemsworth/64c125aa43f55555ba71107faa821f1e/ |
  | Volume | 7 |
  | Publication | Frontiers in Veterinary Science |
  | DOI | 10.3389/fvets.2020.590867 |
  | Journal Abbr | Frontiers in Veterinary Science |
  | Date Added | 11/07/2025, 13:13:12 |
  | Modified | 11/07/2025, 13:13:12 |
- ## Effects of Interactions with Cats in Domestic Environment on the Psychological and Physiological State of Their Owners: Associations among Cortisol, Oxytocin, Heart Rate Variability, and Emotions

  |  |  |
  | --- | --- |
  | Item Type | Journal Article |
  | Author | T. Nagasawa |
  | Author | Y. Kimura |
  | Author | Koji Masuda |
  | Author | Hidehiko Uchiyama |
  | Date | 2023-06-26 |
  | URL | https://consensus.app/papers/effects-of-interactions-with-cats-in-domestic-environment-masuda-uchiyama/7f19dc80b3bb5296829214701253a4b4/ |
  | Volume | 13 |
  | Publication | Animals : an Open Access Journal from MDPI |
  | DOI | 10.3390/ani13132116 |
  | Journal Abbr | Animals : an Open Access Journal from MDPI |
  | Date Added | 11/07/2025, 13:13:12 |
  | Modified | 11/07/2025, 13:13:12 |
- ## The 2020 Five Domains Model: Including Human–Animal Interactions in Assessments of Animal Welfare

  |  |  |
  | --- | --- |
  | Item Type | Journal Article |
  | Author | D. Mellor |
  | Author | N. Beausoleil |
  | Author | K. Littlewood |
  | Author | A. McLean |
  | Author | P. McGreevy |
  | Author | B. Jones |
  | Author | C. Wilkins |
  | Date | 2020-10-01 |
  | URL | https://consensus.app/papers/the-2020-five-domains-model-including-human%E2%80%93animal-mellor-wilkins/c390aac677b45a2ebbde016e12b73522/ |
  | Volume | 10 |
  | Publication | Animals : an Open Access Journal from MDPI |
  | DOI | 10.3390/ani10101870 |
  | Journal Abbr | Animals : an Open Access Journal from MDPI |
  | Date Added | 11/07/2025, 13:13:12 |
  | Modified | 11/07/2025, 13:13:12 |
- ## First noncontact millimeter‐wave radar measurement of heart rate in great apes: Validation in chimpanzees (Pan troglodytes)

  |  |  |
  | --- | --- |
  | Item Type | Journal Article |
  | Author | Takuya Matsumoto |
  | Author | Itsuki Iwata |
  | Author | Takuya Sakamoto |
  | Author | Satoshi Hirata |
  | Date | 2024-05-22 |
  | URL | https://consensus.app/papers/first-noncontact-millimeter%E2%80%90wave-radar-measurement-of-hirata-matsumoto/0bd0bc8ad74350e9beeb5a93e41d4f80/ |
  | Volume | 86 |
  | Publication | American Journal of Primatology |
  | DOI | 10.1002/ajp.23633 |
  | Journal Abbr | American Journal of Primatology |
  | Date Added | 11/07/2025, 13:13:12 |
  | Modified | 11/07/2025, 13:13:12 |
- ## Pet ownership and physical health

  |  |  |
  | --- | --- |
  | Item Type | Journal Article |
  | Author | Robert Matchock |
  | Date | 2015-09-01 |
  | URL | https://consensus.app/papers/pet-ownership-and-physical-health-matchock/04e3751892ee503ab5e10e85f52f7253/ |
  | Volume | 28 |
  | Pages | 386 |
  | Publication | Current Opinion in Psychiatry |
  | DOI | 10.1097/YCO.0000000000000183 |
  | Journal Abbr | Current Opinion in Psychiatry |
  | Date Added | 11/07/2025, 13:13:12 |
  | Modified | 11/07/2025, 13:13:12 |
- ## Evolution of research on human–animal interaction: A review

  |  |  |
  | --- | --- |
  | Item Type | Journal Article |
  | Author | Vivekswar Khandai |
  | Author | P. Shrivastava |
  | Date | 2023-03-01 |
  | URL | https://consensus.app/papers/evolution-of-research-on-human%E2%80%93animal-interaction-a-shrivastava-khandai/fd9df718c67450919ccead2b23394aee/ |
  | Publication | Journal of Entomology and Zoology Studies |
  | DOI | 10.22271/j.ento.2023.v11.i2b.9165 |
  | Journal Abbr | Journal of Entomology and Zoology Studies |
  | Date Added | 11/07/2025, 13:13:12 |
  | Modified | 11/07/2025, 13:13:12 |
- ## Inverse Correlation between Heart Rate Variability and Heart Rate Demonstrated by Linear and Nonlinear Analysis

  |  |  |
  | --- | --- |
  | Item Type | Journal Article |
  | Author | S. Kazmi |
  | Author | Henggui Zhang |
  | Author | W. Aziz |
  | Author | O. Monfredi |
  | Author | Syed Ali Abbas |
  | Author | Saeed Arif Shah |
  | Author | S. Kazmi |
  | Author | W. Butt |
  | Date | 2016-06-23 |
  | URL | https://consensus.app/papers/inverse-correlation-between-heart-rate-variability-and-butt-shah/7baa8236bd9053518e875c77eb6fd265/ |
  | Volume | 11 |
  | Publication | PLoS ONE |
  | DOI | 10.1371/journal.pone.0157557 |
  | Journal Abbr | PLoS ONE |
  | Date Added | 11/07/2025, 13:13:12 |
  | Modified | 11/07/2025, 13:13:12 |
- ## A preliminary model of human–animal relationships in the zoo

  |  |  |
  | --- | --- |
  | Item Type | Journal Article |
  | Author | G. Hosey |
  | Date | 2008-02-01 |
  | URL | https://consensus.app/papers/a-preliminary-model-of-human%E2%80%93animal-relationships-in-the-hosey/5be7e8203a755f01b96ed7c0bf14b40a/ |
  | Volume | 109 |
  | Pages | 105-127 |
  | Publication | Applied Animal Behaviour Science |
  | DOI | 10.1016/J.APPLANIM.2007.04.013 |
  | Journal Abbr | Applied Animal Behaviour Science |
  | Date Added | 11/07/2025, 13:13:12 |
  | Modified | 11/07/2025, 13:13:12 |
- ## Transgenic rabbit models for cardiac disease research

  |  |  |
  | --- | --- |
  | Item Type | Journal Article |
  | Author | T. Hornyik |
  | Author | M. Rieder |
  | Author | A. Castiglione |
  | Author | P. Major |
  | Author | I. Baczkó |
  | Author | M. Brunner |
  | Author | G. Koren |
  | Author | K. Odening |
  | Date | 2021-04-06 |
  | URL | https://consensus.app/papers/transgenic-rabbit-models-for-cardiac-disease-research-castiglione-major/17bda76878185417a3f0ebd1407cb28a/ |
  | Volume | 179 |
  | Pages | 938-957 |
  | Publication | British Journal of Pharmacology |
  | DOI | 10.1111/bph.15484 |
  | Journal Abbr | British Journal of Pharmacology |
  | Date Added | 11/07/2025, 13:13:12 |
  | Modified | 11/07/2025, 13:13:12 |
- ## Contact-Free Simultaneous Sensing of Human Heart Rate and Canine Breathing Rate for Animal Assisted Interactions

  |  |  |
  | --- | --- |
  | Item Type | Journal Article |
  | Author | Timothy Holder |
  | Author | Mushfiqur Rahman |
  | Author | E. Summers |
  | Author | David Roberts |
  | Author | Chau-Wai Wong |
  | Author | A. Bozkurt |
  | Date | 2022-11-07 |
  | URL | https://consensus.app/papers/contactfree-simultaneous-sensing-of-human-heart-rate-and-roberts-bozkurt/9d41ff10bd375dc1825d23afdf1d970f/ |
  | Publication | Proceedings of the Ninth International Conference on Animal-Computer Interaction |
  | DOI | 10.1145/3565995.3566039 |
  | Journal Abbr | Proceedings of the Ninth International Conference on Animal-Computer Interaction |
  | Date Added | 11/07/2025, 13:13:12 |
  | Modified | 11/07/2025, 13:13:12 |
- ## Variability in Human-Animal Interaction Research

  |  |  |
  | --- | --- |
  | Item Type | Journal Article |
  | Author | N. Gee |
  | Author | Kerri Rodriguez |
  | Author | H. Herzog |
  | Date | 2021-01-15 |
  | URL | https://consensus.app/papers/variability-in-humananimal-interaction-research-gee-rodriguez/f107e4d7aae8564db4918abed4d35d13/ |
  | Volume | 7 |
  | Publication | Frontiers in Veterinary Science |
  | DOI | 10.3389/fvets.2020.619600 |
  | Journal Abbr | Frontiers in Veterinary Science |
  | Date Added | 17/06/2025, 18:30:59 |
  | Modified | 04/01/2026, 11:26:12 |
- ## A Systematic Review of Research on Pet Ownership and Animal Interactions among Older Adults

  |  |  |
  | --- | --- |
  | Item Type | Journal Article |
  | Author | N. Gee |
  | Author | M. Mueller |
  | Date | 2019-03-04 |
  | URL | https://consensus.app/papers/a-systematic-review-of-research-on-pet-ownership-and-animal-gee-mueller/a8fcdd6946a65fb09e8e634b91d4c228/ |
  | Volume | 32 |
  | Pages | 183-207 |
  | Publication | Anthrozoös |
  | DOI | 10.1080/08927936.2019.1569903 |
  | Journal Abbr | Anthrozoös |
  | Date Added | 11/07/2025, 13:13:12 |
  | Modified | 11/07/2025, 13:13:12 |
- ## Human–Animal Interaction and Human Prosociality: A Meta-Analytic Review of Experimental and Correlational Studies

  |  |  |
  | --- | --- |
  | Item Type | Journal Article |
  | Author | Nicole Chen |
  | Author | Nadyanna Majeed |
  | Author | Gloria Lai |
  | Author | Paye Shin Koh |
  | Author | K. Kasturiratna |
  | Author | Manmeet Kaur |
  | Author | Alycia Ho |
  | Author | Jose Yong |
  | Author | Andree Hartanto |
  | Date | 2023-12-15 |
  | URL | https://consensus.app/papers/human%E2%80%93animal-interaction-and-human-prosociality-a-chen-ho/e3164a5afc6557a2b4c4f38b22ca4760/ |
  | Volume | 37 |
  | Pages | 269-288 |
  | Publication | Anthrozoös |
  | DOI | 10.1080/08927936.2023.2288745 |
  | Journal Abbr | Anthrozoös |
  | Date Added | 11/07/2025, 13:13:12 |
  | Modified | 11/07/2025, 13:13:12 |
- ## Affective Implications of Human–Animal Relationship on Pig Welfare: Integrating Non-Linear Heart Rate Variability Measures

  |  |  |
  | --- | --- |
  | Item Type | Journal Article |
  | Author | J. Calderón-Amor |
  | Author | Belén Zuleta |
  | Author | M. Ceballos |
  | Author | Daniel Cartes |
  | Author | Christopher Byrd |
  | Author | Benjamin Lecorps |
  | Author | Rocío Palomo |
  | Author | S. Guzmán-Pino |
  | Author | Daniela Siel |
  | Author | Daniela Luna |
  | Date | 2024-07-31 |
  | URL | https://consensus.app/papers/affective-implications-of-human%E2%80%93animal-relationship-on-siel-lecorps/8f1259e116895236ae0f516d20877cdb/ |
  | Volume | 14 |
  | Publication | Animals : an Open Access Journal from MDPI |
  | DOI | 10.3390/ani14152217 |
  | Journal Abbr | Animals : an Open Access Journal from MDPI |
  | Date Added | 11/07/2025, 13:13:12 |
  | Modified | 11/07/2025, 13:13:12 |
- ## Heart rate responses to social interactions in free-moving rhesus macaques (Macaca mulatta): a pilot study.

  |  |  |
  | --- | --- |
  | Item Type | Journal Article |
  | Author | F. Aureli |
  | Author | Stephanie Preston |
  | Author | Frans De Waal |
  | Date | 1999-03-01 |
  | URL | https://consensus.app/papers/heart-rate-responses-to-social-interactions-in-freemoving-aureli-waal/82dc7fe8f6a7528681c1ef3d09a9a5cd/ |
  | Volume | 113 1 |
  | Pages | 59-65 |
  | Publication | Journal of comparative psychology |
  | DOI | 10.1037/0735-7036.113.1.59 |
  | Journal Abbr | Journal of comparative psychology |
  | Date Added | 11/07/2025, 13:13:12 |
  | Modified | 11/07/2025, 13:13:12 |
- ## Solidarity with Animals: Assessing a Relevant Dimension of Social Identification with Animals

  |  |  |
  | --- | --- |
  | Item Type | Journal Article |
  | Author | Catherine Amiot |
  | Author | B. Bastian |
  | Date | 2017-01-03 |
  | URL | https://consensus.app/papers/solidarity-with-animals-assessing-a-relevant-dimension-of-bastian-amiot/ac8b6a7975d85afd957959cadd167336/ |
  | Volume | 12 |
  | Publication | PLoS ONE |
  | DOI | 10.1371/journal.pone.0168184 |
  | Journal Abbr | PLoS ONE |
  | Date Added | 11/07/2025, 13:13:12 |
  | Modified | 11/07/2025, 13:13:12 |
