# Supplementary material for: Evidence of Physiological Comodulation During Human–Animal Interaction: A Systematic Review
Source: Ann N Y Acad Sci. 2026 Jun 4;1560(1):e70299. doi: 10.1111/nyas.70299 (PMC13238372; doi:10.1111/nyas.70299)
Supplement: Supplementary file 2 — Supplementary Materials: Supp2‐Zotero‐Collection.zip [file NYAS-1560-0-s002.zip › Supp2_Zotero_Collection/title screened/Consensus - Oxytocin prompt and Cortisol prompt.htm]

Zotero Report


- ## The Effects of Human–Horse Interactions on Oxytocin and Cortisol Levels in Humans and Horses

  |  |  |
  | --- | --- |
  | Item Type | Journal Article |
  | Author | Minjung Yoon |
  | Author | Youngwook Jung |
  | Date | 2025-03-21 |
  | URL | https://consensus.app/papers/the-effects-of-human%E2%80%93horse-interactions-on-oxytocin-and-yoon-jung/0c72618bc22a5c9689bf2e621a450ac2/ |
  | Volume | 15 |
  | Publication | Animals : an Open Access Journal from MDPI |
  | DOI | 10.3390/ani15070905 |
  | Journal Abbr | Animals : an Open Access Journal from MDPI |
  | Date Added | 20/06/2025, 10:21:07 |
  | Modified | 20/06/2025, 10:21:07 |

  ### Attachments

  - PDF
- ## Cortisol and oxytocin show independent activity during chimpanzee intergroup conflict

  |  |  |
  | --- | --- |
  | Item Type | Journal Article |
  | Author | R. Wittig |
  | Author | L. Samuni |
  | Author | T. Deschner |
  | Author | Catherine Crockford |
  | Author | A. Preis |
  | Date | 2019-06-01 |
  | URL | https://consensus.app/papers/cortisol-and-oxytocin-show-independent-activity-during-wittig-samuni/a076a4a1e81757abab387e8d71585206/ |
  | Volume | 104 |
  | Pages | 165-173 |
  | Publication | Psychoneuroendocrinology |
  | DOI | 10.1016/j.psyneuen.2019.02.007 |
  | Journal Abbr | Psychoneuroendocrinology |
  | Date Added | 20/06/2025, 10:21:07 |
  | Modified | 20/06/2025, 10:21:07 |
- ## Oxytocin and cortisol in romantically unattached young adults: associations with bonding and psychological distress.

  |  |  |
  | --- | --- |
  | Item Type | Journal Article |
  | Author | A. Weller |
  | Author | Ilanit Gordon |
  | Author | R. Feldman |
  | Author | O. Zagoory-Sharon |
  | Author | Inna Schneiderman |
  | Author | J. Leckman |
  | Date | 2008-05-01 |
  | URL | https://consensus.app/papers/oxytocin-and-cortisol-in-romantically-unattached-young-weller-gordon/50846f3ecc56552eb93ca2c953455399/ |
  | Volume | 45 3 |
  | Pages | 349-352 |
  | Publication | Psychophysiology |
  | DOI | 10.1111/j.1469-8986.2008.00649.x |
  | Journal Abbr | Psychophysiology |
  | Date Added | 20/06/2025, 10:21:07 |
  | Modified | 20/06/2025, 10:21:07 |
- ## Social Environment Affects Peripheral Oxytocin and Cortisol during Stress Responses in Guinea‐Pigs

  |  |  |
  | --- | --- |
  | Item Type | Journal Article |
  | Author | B. Wallner |
  | Author | I. Machatschke |
  | Author | J. Dittami |
  | Author | D. Schams |
  | Date | 2004-03-01 |
  | URL | https://consensus.app/papers/social-environment-affects-peripheral-oxytocin-and-wallner-machatschke/c96c428db747578aa58a5d03dc156b29/ |
  | Volume | 110 |
  | Pages | 161-176 |
  | Publication | Ethology |
  | DOI | 10.1111/J.1439-0310.2004.00966.X |
  | Journal Abbr | Ethology |
  | Date Added | 20/06/2025, 10:21:07 |
  | Modified | 20/06/2025, 10:21:07 |
- ## Psychosocial and Psychophysiological Effects of Human-Animal Interactions: The Possible Role of Oxytocin

  |  |  |
  | --- | --- |
  | Item Type | Journal Article |
  | Author | K. Uvnäs-Moberg |
  | Author | Henri Julius |
  | Author | K. Kotrschal |
  | Author | A. Beetz |
  | Date | 2012-07-09 |
  | URL | https://consensus.app/papers/psychosocial-and-psychophysiological-effects-of-uvn%C3%A4s-moberg-julius/c3e730ec42f856fdab39d61c9c937ccf/ |
  | Volume | 3 |
  | Publication | Frontiers in Psychology |
  | DOI | 10.3389/fpsyg.2012.00234 |
  | Journal Abbr | Frontiers in Psychology |
  | Date Added | 20/06/2025, 10:21:07 |
  | Modified | 20/06/2025, 10:21:07 |
- ## Effects of Stroking on Salivary Oxytocin and Cortisol in Guide Dogs: Preliminary Results

  |  |  |
  | --- | --- |
  | Item Type | Journal Article |
  | Author | V. Sergi |
  | Author | C. Mariti |
  | Author | A. Gazzano |
  | Author | P. Baragli |
  | Author | A. Ogi |
  | Date | 2020-04-01 |
  | URL | https://consensus.app/papers/effects-of-stroking-on-salivary-oxytocin-and-cortisol-in-sergi-mariti/84d0d1f7618057c5aa494b5886342fed/ |
  | Volume | 10 |
  | Publication | Animals : an Open Access Journal from MDPI |
  | DOI | 10.3390/ani10040708 |
  | Journal Abbr | Animals : an Open Access Journal from MDPI |
  | Date Added | 20/06/2025, 10:21:07 |
  | Modified | 20/06/2025, 10:21:07 |
- ## Brief communication: Plasma cortisol concentration is affected by lactation, but not intra-nasal oxytocin treatment, in beef cows

  |  |  |
  | --- | --- |
  | Item Type | Journal Article |
  | Author | A. Relling |
  | Author | A. Parker |
  | Author | J. Kieffer |
  | Author | B. Wagner |
  | Date | 2021-07-30 |
  | URL | https://consensus.app/papers/brief-communication-plasma-cortisol-concentration-is-relling-parker/9e104839ad845639a56bd6aa57d7a53c/ |
  | Volume | 16 |
  | Publication | PLoS ONE |
  | DOI | 10.1371/journal.pone.0249323 |
  | Journal Abbr | PLoS ONE |
  | Date Added | 20/06/2025, 10:21:07 |
  | Modified | 20/06/2025, 10:21:07 |
- ## Salivary oxytocin in pigs, cattle, and goats during positive human-animal interactions

  |  |  |
  | --- | --- |
  | Item Type | Journal Article |
  | Author | J. Rault |
  | Author | S. Lürzel |
  | Author | S. Waiblinger |
  | Author | Laura Bückendorf |
  | Date | 2020-03-03 |
  | URL | https://consensus.app/papers/salivary-oxytocin-in-pigs-cattle-and-goats-during-positive-rault-l%C3%BCrzel/b0c163c3ddbc5412ae16ddee892f24ad/ |
  | Volume | 115 |
  | Publication | Psychoneuroendocrinology |
  | DOI | 10.1016/j.psyneuen.2020.104636 |
  | Journal Abbr | Psychoneuroendocrinology |
  | Date Added | 20/06/2025, 10:21:07 |
  | Modified | 20/06/2025, 10:21:07 |
- ## Effects of positive and negative human contacts and intranasal oxytocin on cerebrospinal fluid oxytocin

  |  |  |
  | --- | --- |
  | Item Type | Journal Article |
  | Author | J. Rault |
  | Date | 2016-07-01 |
  | URL | https://consensus.app/papers/effects-of-positive-and-negative-human-contacts-and-rault/90cfa0c6d28257b183856a8bdc4f7ed5/ |
  | Volume | 69 |
  | Pages | 60-66 |
  | Publication | Psychoneuroendocrinology |
  | DOI | 10.1016/j.psyneuen.2016.03.015 |
  | Journal Abbr | Psychoneuroendocrinology |
  | Date Added | 20/06/2025, 10:21:07 |
  | Modified | 20/06/2025, 10:21:07 |
- ## Serum Oxytocin, Cortisol and Social Behavior in Calves: A Study in the Impossible Task Paradigm

  |  |  |
  | --- | --- |
  | Item Type | Journal Article |
  | Author | M. Pero |
  | Author | C. Pinelli |
  | Author | V. Mastellone |
  | Author | Alfredo Di Lucrezia |
  | Author | B. D’Aniello |
  | Author | P. Lombardi |
  | Author | N. Musco |
  | Author | F. Infascelli |
  | Author | P. Iommelli |
  | Author | R. Tudisco |
  | Author | Daria Lotito |
  | Author | Anna Scandurra |
  | Date | 2023-02-01 |
  | URL | https://consensus.app/papers/serum-oxytocin-cortisol-and-social-behavior-in-calves-a-pero-pinelli/396dffa0561e5b989b09ff5b52f1141b/ |
  | Volume | 13 |
  | Publication | Animals : an Open Access Journal from MDPI |
  | DOI | 10.3390/ani13040646 |
  | Journal Abbr | Animals : an Open Access Journal from MDPI |
  | Date Added | 20/06/2025, 10:21:07 |
  | Modified | 20/06/2025, 10:21:07 |
- ## The Urinary Hormonal State of Cats Associated With Social Interaction With Humans

  |  |  |
  | --- | --- |
  | Item Type | Journal Article |
  | Author | Mitsuaki Ohta |
  | Author | T. Nagasawa |
  | Author | Hidehiko Uchiyama |
  | Date | 2021-07-26 |
  | URL | https://consensus.app/papers/the-urinary-hormonal-state-of-cats-associated-with-social-ohta-nagasawa/ef5e075b0ab15735a0de184ac7851a9d/ |
  | Volume | 8 |
  | Publication | Frontiers in Veterinary Science |
  | DOI | 10.3389/fvets.2021.680843 |
  | Journal Abbr | Frontiers in Veterinary Science |
  | Date Added | 20/06/2025, 10:21:07 |
  | Modified | 20/06/2025, 10:21:07 |
- ## Neurophysiological correlates of affiliative behaviour between humans and dogs.

  |  |  |
  | --- | --- |
  | Item Type | Journal Article |
  | Author | J. Odendaal |
  | Author | R. Meintjes |
  | Date | 2003-05-01 |
  | URL | https://consensus.app/papers/neurophysiological-correlates-of-affiliative-behaviour-odendaal-meintjes/781e58a5471d57f2ba145df08fcf2d90/ |
  | Volume | 165 3 |
  | Pages | 296-301 |
  | Publication | Veterinary journal |
  | DOI | 10.1016/S1090-0233(02)00237-X |
  | Journal Abbr | Veterinary journal |
  | Date Added | 20/06/2025, 10:21:07 |
  | Modified | 20/06/2025, 10:21:07 |
- ## Oxytocin levels and self-reported anxiety during interactions between humans and cows

  |  |  |
  | --- | --- |
  | Item Type | Journal Article |
  | Author | Ruth Newberry |
  | Author | Gunn Pedersen |
  | Author | Bente Berget |
  | Author | Judit Vas |
  | Author | Kerstin Uvnäs-Moberg |
  | Date | 2023-09-14 |
  | URL | https://consensus.app/papers/oxytocin-levels-and-selfreported-anxiety-during-newberry-pedersen/d4a67490f4a05105a4c841de6059e3df/ |
  | Volume | 14 |
  | Publication | Frontiers in Psychology |
  | DOI | 10.3389/fpsyg.2023.1252463 |
  | Journal Abbr | Frontiers in Psychology |
  | Date Added | 20/06/2025, 10:21:07 |
  | Modified | 20/06/2025, 10:21:07 |
- ## Physiological Assessment of the Health and Welfare of Domestic Cats—An Exploration of Factors Affecting Urinary Cortisol and Oxytocin

  |  |  |
  | --- | --- |
  | Item Type | Journal Article |
  | Author | T. Nagasawa |
  | Author | Koji Masuda |
  | Author | Hidehiko Uchiyama |
  | Author | Y. Kimura |
  | Date | 2022-11-28 |
  | URL | https://consensus.app/papers/physiological-assessment-of-the-health-and-welfare-of-nagasawa-masuda/4638e92d261755cf8e8014e26a44e9c5/ |
  | Volume | 12 |
  | Publication | Animals : an Open Access Journal from MDPI |
  | DOI | 10.3390/ani12233330 |
  | Journal Abbr | Animals : an Open Access Journal from MDPI |
  | Date Added | 20/06/2025, 10:21:07 |
  | Modified | 20/06/2025, 10:21:07 |
- ## Salivary Cortisol, but Not Oxytocin, Varies With Social Challenges in Domestic Pigs: Implications for Measuring Emotions

  |  |  |
  | --- | --- |
  | Item Type | Journal Article |
  | Author | L. Moscovice |
  | Author | W. Otten |
  | Author | A. Eggert |
  | Author | U. Gimsa |
  | Date | 2022-05-23 |
  | URL | https://consensus.app/papers/salivary-cortisol-but-not-oxytocin-varies-with-social-moscovice-otten/c8b037b364ca5d899b1d9e874de0ce3e/ |
  | Volume | 16 |
  | Publication | Frontiers in Behavioral Neuroscience |
  | DOI | 10.3389/fnbeh.2022.899397 |
  | Journal Abbr | Frontiers in Behavioral Neuroscience |
  | Date Added | 20/06/2025, 10:21:07 |
  | Modified | 20/06/2025, 10:21:07 |
- ## Attachment-like behavioral expressions to humans in puppies are related to oxytocin and cortisol: A comparative study of Akitas and Labrador Retrievers

  |  |  |
  | --- | --- |
  | Item Type | Journal Article |
  | Author | K. Mogi |
  | Author | M. Nagasawa |
  | Author | Sakiko Tomori |
  | Author | T. Kikusui |
  | Date | 2024-04-01 |
  | URL | https://consensus.app/papers/attachmentlike-behavioral-expressions-to-humans-in-mogi-nagasawa/fb32e4587b6053a4ad030f6b8081e5c7/ |
  | Volume | 177 |
  | Publication | Peptides |
  | DOI | 10.1016/j.peptides.2024.171224 |
  | Journal Abbr | Peptides |
  | Date Added | 20/06/2025, 10:21:07 |
  | Modified | 20/06/2025, 10:21:07 |
- ## The Role of Oxytocin in the Dog–Owner Relationship

  |  |  |
  | --- | --- |
  | Item Type | Journal Article |
  | Author | Anne Meinert |
  | Author | T. Deschner |
  | Author | F. Schaebs |
  | Author | S. Marshall-Pescini |
  | Author | F. Range |
  | Author | Alina Gaugg |
  | Date | 2019-10-01 |
  | URL | https://consensus.app/papers/the-role-of-oxytocin-in-the-dog%E2%80%93owner-relationship-meinert-deschner/da7848f459b15165a232f855e1d6f051/ |
  | Volume | 9 |
  | Publication | Animals : an Open Access Journal from MDPI |
  | DOI | 10.3390/ani9100792 |
  | Journal Abbr | Animals : an Open Access Journal from MDPI |
  | Date Added | 20/06/2025, 10:21:07 |
  | Modified | 20/06/2025, 10:21:07 |

  ### Attachments

  - PDF
- ## Relations between plasma oxytocin and cortisol: The stress buffering role of social support

  |  |  |
  | --- | --- |
  | Item Type | Journal Article |
  | Author | R. McQuaid |
  | Author | Faisal Al‐Yawer |
  | Author | H. Anisman |
  | Author | O. McInnis |
  | Author | K. Matheson |
  | Author | Angela Paric |
  | Date | 2016-01-30 |
  | URL | https://consensus.app/papers/relations-between-plasma-oxytocin-and-cortisol-the-stress-mcquaid-al%E2%80%90yawer/827f0ed27b5051febb042e3c552f3a19/ |
  | Volume | 3 |
  | Pages | 52-60 |
  | Publication | Neurobiology of Stress |
  | DOI | 10.1016/j.ynstr.2016.01.001 |
  | Journal Abbr | Neurobiology of Stress |
  | Date Added | 20/06/2025, 10:21:07 |
  | Modified | 20/06/2025, 10:21:07 |
- ## Endocrine changes related to dog domestication: Comparing urinary cortisol and oxytocin in hand-raised, pack-living dogs and wolves

  |  |  |
  | --- | --- |
  | Item Type | Journal Article |
  | Author | S. Marshall-Pescini |
  | Author | G. Wirobski |
  | Author | F. Schaebs |
  | Author | R. Palme |
  | Author | T. Deschner |
  | Author | F. Range |
  | Date | 2020-11-24 |
  | URL | https://consensus.app/papers/endocrine-changes-related-to-dog-domestication-comparing-marshall-pescini-wirobski/afd4e192a2fa51dda1f93acb53adafd2/ |
  | Volume | 128 |
  | Publication | Hormones and Behavior |
  | DOI | 10.1016/j.yhbeh.2020.104901 |
  | Journal Abbr | Hormones and Behavior |
  | Date Added | 20/06/2025, 10:21:07 |
  | Modified | 20/06/2025, 10:21:07 |
- ## Effects of human-animal interaction on salivary and urinary oxytocin in children and dogs

  |  |  |
  | --- | --- |
  | Item Type | Journal Article |
  | Author | Evan MacLean |
  | Author | C. Carter |
  | Author | Melissa Barnett |
  | Author | Jessica Connelly |
  | Author | Gianna Ossello |
  | Author | Katherine King |
  | Author | Stacey Tecot |
  | Author | Elizabeth Carranza |
  | Author | Paige Smith |
  | Author | H. Steklis |
  | Author | Gitanjali Gnanadesikan |
  | Author | Nancy Gee |
  | Author | Abigail Flyer |
  | Author | Netzin Steklis |
  | Date | 2024-07-01 |
  | URL | https://consensus.app/papers/effects-of-humananimal-interaction-on-salivary-and-maclean-carter/8e2732d427945a918018eef3077f9072/ |
  | Volume | 169 |
  | Publication | Psychoneuroendocrinology |
  | DOI | 10.1016/j.psyneuen.2024.107147 |
  | Journal Abbr | Psychoneuroendocrinology |
  | Date Added | 20/06/2025, 10:21:07 |
  | Modified | 20/06/2025, 10:21:07 |
- ## Effects of Affiliative Human–Animal Interaction on Dog Salivary and Plasma Oxytocin and Vasopressin

  |  |  |
  | --- | --- |
  | Item Type | Journal Article |
  | Author | E. MacLean |
  | Author | N. Gee |
  | Author | N. Gee |
  | Author | W. Martin |
  | Author | Kerinne Levy |
  | Author | L. Gesquiere |
  | Author | C. Carter |
  | Date | 2017-09-20 |
  | URL | https://consensus.app/papers/effects-of-affiliative-human%E2%80%93animal-interaction-on-dog-maclean-gee/9d142804ec5e580fa146e41a1136b805/ |
  | Volume | 8 |
  | Publication | Frontiers in Psychology |
  | DOI | 10.3389/fpsyg.2017.01606 |
  | Journal Abbr | Frontiers in Psychology |
  | Date Added | 20/06/2025, 10:21:07 |
  | Modified | 20/06/2025, 10:21:07 |
- ## Basal plasma oxytocin & fecal cortisol concentrations are highly heritable and associated with individual differences in behavior & cognition in dog puppies

  |  |  |
  | --- | --- |
  | Item Type | Journal Article |
  | Author | Brenda Kennedy |
  | Author | Gitanjali Gnanadesikan |
  | Author | Kerinne Levy |
  | Author | Stacey Tecot |
  | Author | Evan MacLean |
  | Author | Emily Bray |
  | Author | L. Douglas |
  | Author | Erica Cook |
  | Date | 2024-08-07 |
  | URL | https://consensus.app/papers/basal-plasma-oxytocin-fecal-cortisol-concentrations-are-kennedy-gnanadesikan/ef2b9b2a191658dabc8800052617b5c5/ |
  | Volume | 165 |
  | Publication | Hormones and Behavior |
  | DOI | 10.1016/j.yhbeh.2024.105612 |
  | Journal Abbr | Hormones and Behavior |
  | Date Added | 20/06/2025, 10:21:07 |
  | Modified | 20/06/2025, 10:21:07 |
- ## Dogs' endocrine and behavioural responses at reunion are affected by how the human initiates contact

  |  |  |
  | --- | --- |
  | Item Type | Journal Article |
  | Author | L. Keeling |
  | Author | Linda Handlin |
  | Author | K. Uvnäs-Moberg |
  | Author | Therese Rehn |
  | Date | 2014-01-30 |
  | URL | https://consensus.app/papers/dogs-endocrine-and-behavioural-responses-at-reunion-are-keeling-handlin/def688292adc5c4c8381468fa6b7bafb/ |
  | Volume | 124 |
  | Pages | 45-53 |
  | Publication | Physiology & Behavior |
  | DOI | 10.1016/j.physbeh.2013.10.009 |
  | Journal Abbr | Physiology & Behavior |
  | Date Added | 20/06/2025, 10:21:07 |
  | Modified | 20/06/2025, 10:21:07 |
- ## Dogs' endocrine and behavioural responses at reunion are affected by how the human initiates contact.

  |  |  |
  | --- | --- |
  | Item Type | Journal Article |
  | Author | L. Keeling |
  | Author | K. Uvnäs-Moberg |
  | Author | Linda Handlin |
  | Author | Therese Rehn |
  | Date | 2014 |
  | URL | https://consensus.app/papers/dogs-endocrine-and-behavioural-responses-at-reunion-are-keeling-uvn%C3%A4s-moberg/f74d52e5ca1d5d36864c9a3dbb1c1356/ |
  | Volume | 124 |
  | Pages | 45-53 |
  | Publication | Physiology & behavior |
  | Journal Abbr | Physiology & behavior |
  | Date Added | 20/06/2025, 10:21:08 |
  | Modified | 20/06/2025, 10:21:08 |
- ## Oxytocin and Cortisol Levels in Dog Owners and Their Dogs Are Associated with Behavioral Patterns: An Exploratory Study

  |  |  |
  | --- | --- |
  | Item Type | Journal Article |
  | Author | E. Hydbring-Sandberg |
  | Author | Linda Handlin |
  | Author | Lise-Lotte Gustafson |
  | Author | K. Uvnäs-Moberg |
  | Author | Anne Nilsson |
  | Author | M. Petersson |
  | Date | 2017-10-13 |
  | URL | https://consensus.app/papers/oxytocin-and-cortisol-levels-in-dog-owners-and-their-dogs-hydbring-sandberg-handlin/bcc2b8f5b33c58e0bc8caddd9502c762/ |
  | Volume | 8 |
  | Publication | Frontiers in Psychology |
  | DOI | 10.3389/fpsyg.2017.01796 |
  | Journal Abbr | Frontiers in Psychology |
  | Date Added | 20/06/2025, 10:21:07 |
  | Modified | 20/06/2025, 10:21:07 |

  ### Attachments

  - PDF
- ## Intranasal Oxytocin Increases Positive Communication and Reduces Cortisol Levels During Couple Conflict

  |  |  |
  | --- | --- |
  | Item Type | Journal Article |
  | Author | M. Heinrichs |
  | Author | B. Ditzen |
  | Author | G. Bodenmann |
  | Author | Barbara Gabriel |
  | Author | U. Ehlert |
  | Author | M. Schaer |
  | Date | 2009-05-01 |
  | URL | https://consensus.app/papers/intranasal-oxytocin-increases-positive-communication-heinrichs-ditzen/3958ef56666c5485adab81f3fb002149/ |
  | Volume | 65 |
  | Pages | 728-731 |
  | Publication | Biological Psychiatry |
  | DOI | 10.1016/j.biopsych.2008.10.011 |
  | Journal Abbr | Biological Psychiatry |
  | Date Added | 20/06/2025, 10:21:07 |
  | Modified | 20/06/2025, 10:21:07 |
- ## Associations between the Psychological Characteristics of the Human–Dog Relationship and Oxytocin and Cortisol Levels

  |  |  |
  | --- | --- |
  | Item Type | Journal Article |
  | Author | Linda Handlin |
  | Author | K. Uvnäs-Moberg |
  | Author | E. Hydbring-Sandberg |
  | Author | Mikael Ejdebäck |
  | Author | Anne Nilsson |
  | Date | 2012-06-01 |
  | URL | https://consensus.app/papers/associations-between-the-psychological-characteristics-handlin-uvn%C3%A4s-moberg/682a01ff2b6d5aba8a9fbe2c78893f1d/ |
  | Volume | 25 |
  | Pages | 215-228 |
  | Publication | Anthrozoös |
  | DOI | 10.2752/175303712X13316289505468 |
  | Journal Abbr | Anthrozoös |
  | Date Added | 20/06/2025, 10:21:07 |
  | Modified | 20/06/2025, 10:21:07 |

  ### Attachments

  - PDF
- ## Short-Term Interaction between Dogs and Their Owners: Effects on Oxytocin, Cortisol, Insulin and Heart Rate—An Exploratory Study

  |  |  |
  | --- | --- |
  | Item Type | Journal Article |
  | Author | Linda Handlin |
  | Author | A. Jansson |
  | Author | Anne Nilsson |
  | Author | Mikael Ejdebäck |
  | Author | K. Uvnäs-Moberg |
  | Author | E. Hydbring-Sandberg |
  | Date | 2011-09-01 |
  | URL | https://consensus.app/papers/shortterm-interaction-between-dogs-and-their-owners-handlin-jansson/deb3624dcd6d5b50918ed737f0e883a7/ |
  | Volume | 24 |
  | Pages | 301-315 |
  | Publication | Anthrozoös |
  | DOI | 10.2752/175303711X13045914865385 |
  | Journal Abbr | Anthrozoös |
  | Date Added | 20/06/2025, 10:21:07 |
  | Modified | 20/06/2025, 10:21:07 |
- ## Human-human and human-animal interaction

  |  |  |
  | --- | --- |
  | Item Type | Journal Article |
  | Author | Linda Handlin |
  | Date | 2010-01-01 |
  | URL | https://consensus.app/papers/humanhuman-and-humananimal-interaction-handlin/a84379b7734a594787b21ca3aff8c7ca/ |
  | Date Added | 20/06/2025, 10:21:07 |
  | Modified | 20/06/2025, 10:21:07 |
- ## Human-Human and Human-Animal Interaction : Some Common Physiological and Psychological Effects

  |  |  |
  | --- | --- |
  | Item Type | Journal Article |
  | Author | Linda Handlin |
  | Date | 2010-01-01 |
  | URL | https://consensus.app/papers/humanhuman-and-humananimal-interaction-some-common-handlin/dc01a7b2afd25e7fb98845aff3fbe2de/ |
  | Date Added | 20/06/2025, 10:21:07 |
  | Modified | 20/06/2025, 10:21:07 |
- ## Exploring women’s oxytocin responses to interactions with their pet cats

  |  |  |
  | --- | --- |
  | Item Type | Journal Article |
  | Author | P. Gray |
  | Author | Elizabeth Johnson |
  | Author | Arianna Portillo |
  | Author | Nikki Bennett |
  | Date | 2021-11-12 |
  | URL | https://consensus.app/papers/exploring-women-%E2%80%99-s-oxytocin-responses-to-interactions-gray-johnson/f084871e8eac5b48a5e93893658df5c0/ |
  | Volume | 9 |
  | Publication | PeerJ |
  | DOI | 10.7717/peerj.12393 |
  | Journal Abbr | PeerJ |
  | Date Added | 20/06/2025, 10:21:07 |
  | Modified | 20/06/2025, 10:21:07 |
- ## Boosting recovery rather than buffering reactivity: Higher stress-induced oxytocin secretion is associated with increased cortisol reactivity and faster vagal recovery after acute psychosocial stress

  |  |  |
  | --- | --- |
  | Item Type | Journal Article |
  | Author | V. Engert |
  | Author | T. Singer |
  | Author | Anna Koester |
  | Author | A. Riepenhausen |
  | Date | 2016-12-01 |
  | URL | https://consensus.app/papers/boosting-recovery-rather-than-buffering-reactivity-engert-singer/6ab9efa9ad5b5786aa1ec9abfdcbd04e/ |
  | Volume | 74 |
  | Pages | 111-120 |
  | Publication | Psychoneuroendocrinology |
  | DOI | 10.1016/j.psyneuen.2016.08.029 |
  | Journal Abbr | Psychoneuroendocrinology |
  | Date Added | 20/06/2025, 10:21:07 |
  | Modified | 20/06/2025, 10:21:07 |
- ## Canine Endogenous Oxytocin Responses to Dog-Walking and Affiliative Human–Dog Interactions

  |  |  |
  | --- | --- |
  | Item Type | Journal Article |
  | Author | B. Drayton |
  | Author | Lauren Powell |
  | Author | A. Bauman |
  | Author | P. McGreevy |
  | Author | K. Edwards |
  | Author | E. Stamatakis |
  | Author | A. Guastella |
  | Date | 2019-02-01 |
  | URL | https://consensus.app/papers/canine-endogenous-oxytocin-responses-to-dogwalking-and-drayton-powell/31cf18c60db35636ba9b2c6c798cfc01/ |
  | Volume | 9 |
  | Publication | Animals : an Open Access Journal from MDPI |
  | DOI | 10.3390/ani9020051 |
  | Journal Abbr | Animals : an Open Access Journal from MDPI |
  | Date Added | 20/06/2025, 10:21:07 |
  | Modified | 20/06/2025, 10:21:07 |
- ## Oxytocin facilitates adaptive fear and attenuates anxiety responses in animal models and human studies—potential interaction with the corticotropin-releasing factor (CRF) system in the bed nucleus of the stria terminalis (BNST)

  |  |  |
  | --- | --- |
  | Item Type | Journal Article |
  | Author | J. Dabrowska |
  | Author | Michael Janeček |
  | Date | 2018-07-28 |
  | URL | https://consensus.app/papers/oxytocin-facilitates-adaptive-fear-and-attenuates-dabrowska-jane%C4%8Dek/82d3fa61cb38535a876fd84e05b9f06b/ |
  | Volume | 375 |
  | Pages | 143-172 |
  | Publication | Cell and Tissue Research |
  | DOI | 10.1007/s00441-018-2889-8 |
  | Journal Abbr | Cell and Tissue Research |
  | Date Added | 20/06/2025, 10:21:07 |
  | Modified | 20/06/2025, 10:21:07 |
- ## Serum Oxytocin in Cows Is Positively Correlated with Caregiver Interactions in the Impossible Task Paradigm

  |  |  |
  | --- | --- |
  | Item Type | Journal Article |
  | Author | B. D’Aniello |
  | Author | P. Lombardi |
  | Author | N. Musco |
  | Author | F. Infascelli |
  | Author | Alfredo Di Lucrezia |
  | Author | M. Pero |
  | Author | C. Pinelli |
  | Author | V. Mastellone |
  | Author | R. Tudisco |
  | Author | Anna Scandurra |
  | Date | 2022-01-23 |
  | URL | https://consensus.app/papers/serum-oxytocin-in-cows-is-positively-correlated-with-d%E2%80%99aniello-lombardi/af684ce8f8bd5b9e84f6f8fd6ab2eaf3/ |
  | Volume | 12 |
  | Publication | Animals : an Open Access Journal from MDPI |
  | DOI | 10.3390/ani12030276 |
  | Journal Abbr | Animals : an Open Access Journal from MDPI |
  | Date Added | 20/06/2025, 10:21:07 |
  | Modified | 20/06/2025, 10:21:07 |
- ## A meta-analytic review of the correlation between peripheral oxytocin and cortisol concentrations

  |  |  |
  | --- | --- |
  | Item Type | Journal Article |
  | Author | Christopher Cardoso |
  | Author | M. Ellenbogen |
  | Author | Christopher Brown |
  | Date | 2016-10-01 |
  | URL | https://consensus.app/papers/a-metaanalytic-review-of-the-correlation-between-cardoso-ellenbogen/259a9ca4bb455ffba161a6cc4bee4f8c/ |
  | Volume | 43 |
  | Pages | 19-27 |
  | Publication | Frontiers in Neuroendocrinology |
  | DOI | 10.1016/j.yfrne.2016.11.001 |
  | Journal Abbr | Frontiers in Neuroendocrinology |
  | Date Added | 20/06/2025, 10:21:07 |
  | Modified | 20/06/2025, 10:21:07 |
- ## Effects of human and animal-assisted skills training on oxytocin und cortisol levels in patients with borderline personality disorder.

  |  |  |
  | --- | --- |
  | Item Type | Journal Article |
  | Author | M. Brüne |
  | Author | Olivia Plett |
  | Author | V. Flasbeck |
  | Date | 2023-05-01 |
  | URL | https://consensus.app/papers/effects-of-human-and-animalassisted-skills-training-on-br%C3%BCne-plett/55c95d456dda54bdac2ed973811e10b3/ |
  | Volume | 162 |
  | Pages | 156-160 |
  | Publication | Journal of psychiatric research |
  | DOI | 10.1016/j.jpsychires.2023.05.004 |
  | Journal Abbr | Journal of psychiatric research |
  | Date Added | 20/06/2025, 10:21:07 |
  | Modified | 20/06/2025, 10:21:07 |
- ## Human–lamb bonding: Oxytocin, cortisol and behavioural responses of lambs to human contacts and social separation

  |  |  |
  | --- | --- |
  | Item Type | Journal Article |
  | Author | X. Boivin |
  | Author | R. Nowak |
  | Author | Christine Ravel |
  | Author | P. Marnet |
  | Author | M. Coulon |
  | Author | S. Andanson |
  | Author | A. Boissy |
  | Date | 2013-04-01 |
  | URL | https://consensus.app/papers/human%E2%80%93lamb-bonding-oxytocin-cortisol-and-behavioural-boivin-nowak/a5b71a97f2115e4ba661246b6529511b/ |
  | Volume | 38 |
  | Pages | 499-508 |
  | Publication | Psychoneuroendocrinology |
  | DOI | 10.1016/j.psyneuen.2012.07.008 |
  | Journal Abbr | Psychoneuroendocrinology |
  | Date Added | 20/06/2025, 10:21:07 |
  | Modified | 20/06/2025, 10:21:07 |
- ## Social support and oxytocin interact to suppress cortisol and subjective responses to psychosocial stress

  |  |  |
  | --- | --- |
  | Item Type | Journal Article |
  | Author | T. Baumgartner |
  | Author | C. Kirschbaum |
  | Author | U. Ehlert |
  | Author | M. Heinrichs |
  | Date | 2003-12-15 |
  | URL | https://consensus.app/papers/social-support-and-oxytocin-interact-to-suppress-cortisol-baumgartner-kirschbaum/c0136ada0b9456dba08c77d79255990e/ |
  | Volume | 54 |
  | Pages | 1389-1398 |
  | Publication | Biological Psychiatry |
  | DOI | 10.1016/S0006-3223(03)00465-7 |
  | Journal Abbr | Biological Psychiatry |
  | Date Added | 20/06/2025, 10:21:08 |
  | Modified | 20/06/2025, 10:21:08 |
- ## Oxytocin: a parenting hormone.

  |  |  |
  | --- | --- |
  | Item Type | Journal Article |
  | Author | M. Bakermans‐Kranenburg |
  | Author | R. Feldman |
  | Date | 2017-06-01 |
  | URL | https://consensus.app/papers/oxytocin-a-parenting-hormone-bakermans%E2%80%90kranenburg-feldman/f09351e37bba566fb2faf25b02200a45/ |
  | Volume | 15 |
  | Pages | 13-18 |
  | Publication | Current opinion in psychology |
  | DOI | 10.1016/j.copsyc.2017.02.011 |
  | Journal Abbr | Current opinion in psychology |
  | Date Added | 20/06/2025, 10:21:07 |
  | Modified | 20/06/2025, 10:21:07 |
