# Supplementary material for: Evidence of Physiological Comodulation During Human–Animal Interaction: A Systematic Review
Source: Ann N Y Acad Sci. 2026 Jun 4;1560(1):e70299. doi: 10.1111/nyas.70299 (PMC13238372; doi:10.1111/nyas.70299)
Supplement: Supplementary file 2 — Supplementary Materials: Supp2‐Zotero‐Collection.zip [file NYAS-1560-0-s002.zip › Supp2_Zotero_Collection/title screened/Consensus - PPG prompt.htm]

Zotero Report


- ## An Approach towards Motion-Tolerant PPG-Based Algorithm for Real-Time Heart Rate Monitoring of Moving Pigs

  |  |  |
  | --- | --- |
  | Item Type | Journal Article |
  | Author | A. Youssef |
  | Author | Alberto Peña Fernández |
  | Author | Laura Wassermann |
  | Author | S. Biernot |
  | Author | Eva-Maria Wittauer |
  | Author | A. Bleich |
  | Author | J. Hartung |
  | Author | D. Berckmans |
  | Author | Tomas Norton |
  | Date | 2020-07-26 |
  | URL | https://consensus.app/papers/an-approach-towards-motiontolerant-ppgbased-algorithm-biernot-youssef/d8019dab57e058eb964807931a13edc8/ |
  | Volume | 20 |
  | Publication | Sensors (Basel, Switzerland) |
  | DOI | 10.3390/s20154251 |
  | Journal Abbr | Sensors (Basel, Switzerland) |
  | Date Added | 11/07/2025, 13:10:54 |
  | Modified | 11/07/2025, 13:10:54 |
- ## Challenges in Designing Human-Animal Interaction Research

  |  |  |
  | --- | --- |
  | Item Type | Journal Article |
  | Author | Cindy Wilson |
  | Author | S. Barker |
  | Date | 2003-09-01 |
  | URL | https://consensus.app/papers/challenges-in-designing-humananimal-interaction-wilson-barker/33c9f77e531552e587c21a274e8136d4/ |
  | Volume | 47 |
  | Pages | 16-28 |
  | Publication | American Behavioral Scientist |
  | DOI | 10.1177/0002764203255208 |
  | Journal Abbr | American Behavioral Scientist |
  | Date Added | 11/07/2025, 13:10:54 |
  | Modified | 11/07/2025, 13:10:54 |
- ## The State of Research on Human–Animal Relations: Implications for Human Health

  |  |  |
  | --- | --- |
  | Item Type | Journal Article |
  | Author | D. Wells |
  | Date | 2019-03-04 |
  | URL | https://consensus.app/papers/the-state-of-research-on-human%E2%80%93animal-relations-wells/d57ae41124365ae0b531560b06a8400f/ |
  | Volume | 32 |
  | Pages | 169-181 |
  | Publication | Anthrozoös |
  | DOI | 10.1080/08927936.2019.1569902 |
  | Journal Abbr | Anthrozoös |
  | Date Added | 11/07/2025, 13:10:54 |
  | Modified | 11/07/2025, 13:10:54 |
- ## Psychometric Evaluation of the Comfort from Companion Animals Scale in a Sexual and Gender Minority Sample

  |  |  |
  | --- | --- |
  | Item Type | Journal Article |
  | Author | Camie Tomlinson |
  | Author | Sarah Pittman |
  | Author | Jennifer Murphy |
  | Author | Angela Matijczak |
  | Author | S. McDonald |
  | Date | 2021-08-26 |
  | URL | https://consensus.app/papers/psychometric-evaluation-of-the-comfort-from-companion-matijczak-mcdonald/c3c149ee4c17564f82a51f37e29354ab/ |
  | Volume | 35 |
  | Pages | 143-163 |
  | Publication | Anthrozoös |
  | DOI | 10.1080/08927936.2021.1963548 |
  | Journal Abbr | Anthrozoös |
  | Date Added | 11/07/2025, 13:10:54 |
  | Modified | 11/07/2025, 13:10:54 |
- ## The Power of a Positive Human–Animal Relationship for Animal Welfare

  |  |  |
  | --- | --- |
  | Item Type | Journal Article |
  | Author | J. Rault |
  | Author | S. Waiblinger |
  | Author | X. Boivin |
  | Author | P. Hemsworth |
  | Date | 2020-11-09 |
  | URL | https://consensus.app/papers/the-power-of-a-positive-human%E2%80%93animal-relationship-for-boivin-hemsworth/64c125aa43f55555ba71107faa821f1e/ |
  | Volume | 7 |
  | Publication | Frontiers in Veterinary Science |
  | DOI | 10.3389/fvets.2020.590867 |
  | Journal Abbr | Frontiers in Veterinary Science |
  | Date Added | 11/07/2025, 13:10:54 |
  | Modified | 11/07/2025, 13:10:54 |
- ## Prediction of state anxiety by machine learning applied to photoplethysmography data

  |  |  |
  | --- | --- |
  | Item Type | Journal Article |
  | Author | D. Perpetuini |
  | Author | A. Chiarelli |
  | Author | D. Cardone |
  | Author | C. Filippini |
  | Author | Sergio Rinella |
  | Author | Simona Massimino |
  | Author | F. Bianco |
  | Author | V. Bucciarelli |
  | Author | V. Vinciguerra |
  | Author | P. Fallica |
  | Author | V. Perciavalle |
  | Author | S. Gallina |
  | Author | S. Conoci |
  | Author | A. Merla |
  | Date | 2021-01-15 |
  | URL | https://consensus.app/papers/prediction-of-state-anxiety-by-machine-learning-applied-to-fallica-chiarelli/9850ffe0af1957ff8803056263df0572/ |
  | Volume | 9 |
  | Publication | PeerJ |
  | DOI | 10.7717/peerj.10448 |
  | Journal Abbr | PeerJ |
  | Date Added | 11/07/2025, 13:10:54 |
  | Modified | 11/07/2025, 13:10:54 |
- ## The 2020 Five Domains Model: Including Human–Animal Interactions in Assessments of Animal Welfare

  |  |  |
  | --- | --- |
  | Item Type | Journal Article |
  | Author | D. Mellor |
  | Author | N. Beausoleil |
  | Author | K. Littlewood |
  | Author | A. McLean |
  | Author | P. McGreevy |
  | Author | B. Jones |
  | Author | C. Wilkins |
  | Date | 2020-10-01 |
  | URL | https://consensus.app/papers/the-2020-five-domains-model-including-human%E2%80%93animal-mellor-wilkins/c390aac677b45a2ebbde016e12b73522/ |
  | Volume | 10 |
  | Publication | Animals : an Open Access Journal from MDPI |
  | DOI | 10.3390/ani10101870 |
  | Journal Abbr | Animals : an Open Access Journal from MDPI |
  | Date Added | 11/07/2025, 13:10:54 |
  | Modified | 11/07/2025, 13:10:54 |
- ## Salivary oxytocin in pigs, cattle, and goats during positive human-animal interactions

  |  |  |
  | --- | --- |
  | Item Type | Journal Article |
  | Author | S. Lürzel |
  | Author | Laura Bückendorf |
  | Author | S. Waiblinger |
  | Author | J. Rault |
  | Date | 2020-03-03 |
  | URL | https://consensus.app/papers/salivary-oxytocin-in-pigs-cattle-and-goats-during-positive-rault-l%C3%BCrzel/b0c163c3ddbc5412ae16ddee892f24ad/ |
  | Volume | 115 |
  | Publication | Psychoneuroendocrinology |
  | DOI | 10.1016/j.psyneuen.2020.104636 |
  | Journal Abbr | Psychoneuroendocrinology |
  | Date Added | 11/07/2025, 13:10:54 |
  | Modified | 11/07/2025, 13:10:54 |
- ## Human-Animal Interactions

  |  |  |
  | --- | --- |
  | Item Type | Journal Article |
  | Author | Megan LaFollette |
  | Date | 2020-09-09 |
  | URL | https://consensus.app/papers/humananimal-interactions-lafollette/1f21ef9cfd055b07a0d7afebc6db76f8/ |
  | Publication | Animal-centric Care and Management |
  | DOI | 10.1201/9780429059544-1 |
  | Journal Abbr | Animal-centric Care and Management |
  | Date Added | 11/07/2025, 13:10:54 |
  | Modified | 11/07/2025, 13:10:54 |
- ## The Human-Animal Interaction at Work Scale: Development and psychometric properties

  |  |  |
  | --- | --- |
  | Item Type | Journal Article |
  | Author | A. Junça‐Silva |
  | Date | 2024-07-01 |
  | URL | https://consensus.app/papers/the-humananimal-interaction-at-work-scale-development-and-jun%C3%A7a%E2%80%90silva/44d95ba5ee9459d8b057a28ea754c998/ |
  | Publication | Journal of Veterinary Behavior |
  | DOI | 10.1016/j.jveb.2024.06.007 |
  | Journal Abbr | Journal of Veterinary Behavior |
  | Date Added | 11/07/2025, 13:10:54 |
  | Modified | 11/07/2025, 13:10:54 |
- ## Measurement of attachment in human-animal interaction research

  |  |  |
  | --- | --- |
  | Item Type | Journal Article |
  | Author | Eli Halbreich |
  | Author | Tristen Hefner |
  | Author | Ashly Healy |
  | Author | Jason Van Allen |
  | Date | 2024-09-25 |
  | URL | https://consensus.app/papers/measurement-of-attachment-in-humananimal-interaction-allen-halbreich/54b1023aa33657699ba75874ecd76bfd/ |
  | Publication | Human-Animal Interactions |
  | DOI | 10.1079/hai.2024.0030 |
  | Journal Abbr | Human-Animal Interactions |
  | Date Added | 11/07/2025, 13:10:54 |
  | Modified | 11/07/2025, 13:10:54 |
- ## Human-Animal Interaction Research: Progress and Possibilities

  |  |  |
  | --- | --- |
  | Item Type | Journal Article |
  | Author | J. Griffin |
  | Author | K. Hurley |
  | Author | S. McCune |
  | Date | 2019-12-20 |
  | URL | https://consensus.app/papers/humananimal-interaction-research-progress-and-mccune-hurley/a0acdde9b990569aa91ffb26ec73c2ea/ |
  | Volume | 10 |
  | Publication | Frontiers in Psychology |
  | DOI | 10.3389/fpsyg.2019.02803 |
  | Journal Abbr | Frontiers in Psychology |
  | Date Added | 11/07/2025, 13:10:54 |
  | Modified | 11/07/2025, 13:10:54 |
- ## Variability in Human-Animal Interaction Research

  |  |  |
  | --- | --- |
  | Item Type | Journal Article |
  | Author | N. Gee |
  | Author | Kerri Rodriguez |
  | Author | H. Herzog |
  | Date | 2021-01-15 |
  | URL | https://consensus.app/papers/variability-in-humananimal-interaction-research-gee-rodriguez/f107e4d7aae8564db4918abed4d35d13/ |
  | Volume | 7 |
  | Publication | Frontiers in Veterinary Science |
  | DOI | 10.3389/fvets.2020.619600 |
  | Journal Abbr | Frontiers in Veterinary Science |
  | Date Added | 17/06/2025, 18:30:59 |
  | Modified | 04/01/2026, 11:26:12 |
- ## A Systematic Review of Research on Pet Ownership and Animal Interactions among Older Adults

  |  |  |
  | --- | --- |
  | Item Type | Journal Article |
  | Author | N. Gee |
  | Author | M. Mueller |
  | Date | 2019-03-04 |
  | URL | https://consensus.app/papers/a-systematic-review-of-research-on-pet-ownership-and-animal-gee-mueller/a8fcdd6946a65fb09e8e634b91d4c228/ |
  | Volume | 32 |
  | Pages | 183-207 |
  | Publication | Anthrozoös |
  | DOI | 10.1080/08927936.2019.1569903 |
  | Journal Abbr | Anthrozoös |
  | Date Added | 11/07/2025, 13:10:54 |
  | Modified | 11/07/2025, 13:10:54 |
- ## The Human–Animal Interaction Scale: Development and Evaluation

  |  |  |
  | --- | --- |
  | Item Type | Journal Article |
  | Author | Angela Fournier |
  | Author | T. Berry |
  | Author | Elizabeth Letson |
  | Author | Ryan Chanen |
  | Date | 2016-08-17 |
  | URL | https://consensus.app/papers/the-human%E2%80%93animal-interaction-scale-development-and-fournier-letson/c4455d7449d7509c81fe89ac5f87a63c/ |
  | Volume | 29 |
  | Pages | 455-467 |
  | Publication | Anthrozoös |
  | DOI | 10.1080/08927936.2016.1181372 |
  | Journal Abbr | Anthrozoös |
  | Date Added | 11/07/2025, 13:10:54 |
  | Modified | 11/07/2025, 13:10:54 |
- ## How to Measure Human-Dog Interaction in Dog Assisted Interventions? A Scoping Review

  |  |  |
  | --- | --- |
  | Item Type | Journal Article |
  | Author | Marta De Santis |
  | Author | L. Filugelli |
  | Author | Alberto Mair |
  | Author | Simona Normando |
  | Author | F. Mutinelli |
  | Author | L. Contalbrigo |
  | Date | 2024-01-26 |
  | URL | https://consensus.app/papers/how-to-measure-humandog-interaction-in-dog-assisted-mutinelli-santis/165b6e269f695f6fa43345168729b0df/ |
  | Volume | 14 |
  | Publication | Animals : an Open Access Journal from MDPI |
  | DOI | 10.3390/ani14030410 |
  | Journal Abbr | Animals : an Open Access Journal from MDPI |
  | Date Added | 11/07/2025, 13:10:54 |
  | Modified | 11/07/2025, 13:10:54 |
- ## Measurement in the study of human exposure to animal feces: A systematic review and audit.

  |  |  |
  | --- | --- |
  | Item Type | Journal Article |
  | Author | A. Ballard |
  | Author | Nicholas Laramee |
  | Author | R. Haardörfer |
  | Author | M. Freeman |
  | Author | K. Levy |
  | Author | B. Caruso |
  | Date | 2023-03-01 |
  | URL | https://consensus.app/papers/measurement-in-the-study-of-human-exposure-to-animal-feces-a-levy-ballard/56434451d0f55a18942b4f68c5121e3b/ |
  | Volume | 249 |
  | Pages | 114146 |
  | Publication | International journal of hygiene and environmental health |
  | DOI | 10.1016/j.ijheh.2023.114146 |
  | Journal Abbr | International journal of hygiene and environmental health |
  | Date Added | 11/07/2025, 13:10:54 |
  | Modified | 11/07/2025, 13:10:54 |
- ## Solidarity with Animals: Assessing a Relevant Dimension of Social Identification with Animals

  |  |  |
  | --- | --- |
  | Item Type | Journal Article |
  | Author | Catherine Amiot |
  | Author | B. Bastian |
  | Date | 2017-01-03 |
  | URL | https://consensus.app/papers/solidarity-with-animals-assessing-a-relevant-dimension-of-bastian-amiot/ac8b6a7975d85afd957959cadd167336/ |
  | Volume | 12 |
  | Publication | PLoS ONE |
  | DOI | 10.1371/journal.pone.0168184 |
  | Journal Abbr | PLoS ONE |
  | Date Added | 11/07/2025, 13:10:54 |
  | Modified | 11/07/2025, 13:10:54 |
- ## Protein Binding in Translational Antimicrobial Development-Focus on Interspecies Differences

  |  |  |
  | --- | --- |
  | Item Type | Journal Article |
  | Author | Hifza Ahmed |
  | Author | F. Bergmann |
  | Author | M. Zeitlinger |
  | Date | 2022-07-01 |
  | URL | https://consensus.app/papers/protein-binding-in-translational-antimicrobial-zeitlinger-bergmann/841ad70b3d6a5b56be12daae1a47e51e/ |
  | Volume | 11 |
  | Publication | Antibiotics |
  | DOI | 10.3390/antibiotics11070923 |
  | Journal Abbr | Antibiotics |
  | Date Added | 11/07/2025, 13:10:54 |
  | Modified | 11/07/2025, 13:10:54 |
