# Supplementary material for: Evidence of Physiological Comodulation During Human–Animal Interaction: A Systematic Review
Source: Ann N Y Acad Sci. 2026 Jun 4;1560(1):e70299. doi: 10.1111/nyas.70299 (PMC13238372; doi:10.1111/nyas.70299)
Supplement: Supplementary file 2 — Supplementary Materials: Supp2‐Zotero‐Collection.zip [file NYAS-1560-0-s002.zip › Supp2_Zotero_Collection/title screened/Consensus - Breath prompt.htm]

Zotero Report


- ## Human–Animal Interactions in Disaster Settings: A Systematic Review

  |  |  |
  | --- | --- |
  | Item Type | Journal Article |
  | Author | Haorui Wu |
  | Author | M. Yung |
  | Author | Maryam Schneider |
  | Author | Lindsay Heyland |
  | Date | 2023-06-01 |
  | URL | https://consensus.app/papers/human%E2%80%93animal-interactions-in-disaster-settings-a-wu-yung/9a69fdae373a50f0b3b3cde27cabbea1/ |
  | Volume | 14 |
  | Pages | 369-381 |
  | Publication | International Journal of Disaster Risk Science |
  | DOI | 10.1007/s13753-023-00496-9 |
  | Journal Abbr | International Journal of Disaster Risk Science |
  | Date Added | 17/06/2025, 18:30:59 |
  | Modified | 17/06/2025, 18:30:59 |
- ## The State of Research on Human–Animal Relations: Implications for Human Health

  |  |  |
  | --- | --- |
  | Item Type | Journal Article |
  | Author | D. Wells |
  | Date | 2019-03-04 |
  | URL | https://consensus.app/papers/the-state-of-research-on-human%E2%80%93animal-relations-wells/d57ae41124365ae0b531560b06a8400f/ |
  | Volume | 32 |
  | Pages | 169-181 |
  | Publication | Anthrozoös |
  | DOI | 10.1080/08927936.2019.1569902 |
  | Journal Abbr | Anthrozoös |
  | Date Added | 17/06/2025, 18:30:59 |
  | Modified | 17/06/2025, 18:30:59 |
- ## Heart rate as a measure of emotional arousal in evolutionary biology

  |  |  |
  | --- | --- |
  | Item Type | Journal Article |
  | Author | C. Wascher |
  | Date | 2021-06-28 |
  | URL | https://consensus.app/papers/heart-rate-as-a-measure-of-emotional-arousal-in-wascher/98029954521653808a6c983631aa7ab3/ |
  | Volume | 376 |
  | Publication | Philosophical Transactions of the Royal Society B |
  | DOI | 10.1098/rstb.2020.0479 |
  | Journal Abbr | Philosophical Transactions of the Royal Society B |
  | Date Added | 17/06/2025, 18:30:59 |
  | Modified | 17/06/2025, 18:30:59 |
- ## Measurement of attachment in human-animal interaction research

  |  |  |
  | --- | --- |
  | Item Type | Journal Article |
  | Author | Jason Van Allen |
  | Author | Eli Halbreich |
  | Author | Tristen Hefner |
  | Author | Ashly Healy |
  | Date | 2024-09-25 |
  | URL | https://consensus.app/papers/measurement-of-attachment-in-humananimal-interaction-allen-halbreich/54b1023aa33657699ba75874ecd76bfd/ |
  | Publication | Human-Animal Interactions |
  | DOI | 10.1079/hai.2024.0030 |
  | Journal Abbr | Human-Animal Interactions |
  | Date Added | 17/06/2025, 18:30:59 |
  | Modified | 17/06/2025, 18:30:59 |
- ## The rat adequately reflects human responses to exercise in blood biochemical profile: a comparative study

  |  |  |
  | --- | --- |
  | Item Type | Journal Article |
  | Author | Aikaterini Tzioura |
  | Author | V. Paschalis |
  | Author | I. Vrabas |
  | Author | Aristidis Veskoukis |
  | Author | N. Margaritelis |
  | Author | A. Zafeiridis |
  | Author | Georgios Goutianos |
  | Author | M. Nikolaidis |
  | Author | K. Dipla |
  | Author | A. Kyparos |
  | Date | 2015-02-01 |
  | URL | https://consensus.app/papers/the-rat-adequately-reflects-human-responses-to-exercise-in-tzioura-paschalis/0e47758a66b45a489987969c5715b889/ |
  | Volume | 3 |
  | Publication | Physiological Reports |
  | DOI | 10.14814/phy2.12293 |
  | Journal Abbr | Physiological Reports |
  | Date Added | 17/06/2025, 18:30:59 |
  | Modified | 17/06/2025, 18:30:59 |
- ## Future trends in measuring physiology in free-living animals

  |  |  |
  | --- | --- |
  | Item Type | Journal Article |
  | Author | J. Shipley |
  | Author | M. Wikelski |
  | Author | H. Williams |
  | Author | C. Rutz |
  | Author | L. Hawkes |
  | Author | M. Wilkes |
  | Date | 2021-06-28 |
  | URL | https://consensus.app/papers/future-trends-in-measuring-physiology-in-freeliving-shipley-wikelski/1b3502741e985b88afe1ad769b8f9052/ |
  | Volume | 376 |
  | Publication | Philosophical Transactions of the Royal Society B |
  | DOI | 10.1098/rstb.2020.0230 |
  | Journal Abbr | Philosophical Transactions of the Royal Society B |
  | Date Added | 17/06/2025, 18:30:59 |
  | Modified | 17/06/2025, 18:30:59 |
- ## Analyzing Human-Animal Relationship Measures

  |  |  |
  | --- | --- |
  | Item Type | Journal Article |
  | Author | R. Poresky |
  | Date | 1989-12-01 |
  | URL | https://consensus.app/papers/analyzing-humananimal-relationship-measures-poresky/a559200da86a5a89ac84b66ad93e0d6e/ |
  | Volume | 2 |
  | Pages | 236-244 |
  | Publication | Anthrozoos |
  | DOI | 10.2752/089279389787057911 |
  | Journal Abbr | Anthrozoos |
  | Date Added | 17/06/2025, 18:30:59 |
  | Modified | 17/06/2025, 18:30:59 |
- ## Salivary Studies of the Social Neuroscience of Human–Animal Interaction

  |  |  |
  | --- | --- |
  | Item Type | Journal Article |
  | Author | Patricia Pendry |
  | Author | Jaymie Vandagriff |
  | Date | 2020-01-01 |
  | URL | https://consensus.app/papers/salivary-studies-of-the-social-neuroscience-of-pendry-vandagriff/b7b920ab9d625ce48ba9f8784e6aca97/ |
  | Pages | 555-581 |
  | DOI | 10.1007/978-3-030-35784-9\_23 |
  | Date Added | 17/06/2025, 18:30:59 |
  | Modified | 17/06/2025, 18:30:59 |
- ## Noncontact Electrophysiology Monitoring Systems for Assessment of Canine-Human Interactions

  |  |  |
  | --- | --- |
  | Item Type | Journal Article |
  | Author | Aakash Patel |
  | Author | Marc Foster |
  | Author | T. Torfs |
  | Author | P. Ahmmed |
  | Author | I. Castro |
  | Author | Timothy Holder |
  | Author | A. Bozkurt |
  | Date | 2021-10-31 |
  | URL | https://consensus.app/papers/noncontact-electrophysiology-monitoring-systems-for-patel-foster/0eeed0f0fd9750729fde69f8f3929af0/ |
  | Pages | 1-4 |
  | Publication | 2021 IEEE Sensors |
  | DOI | 10.1109/SENSORS47087.2021.9639748 |
  | Journal Abbr | 2021 IEEE Sensors |
  | Date Added | 17/06/2025, 18:30:59 |
  | Modified | 17/06/2025, 18:30:59 |

  ### Attachments

  - PDF
- ## The 2020 Five Domains Model: Including Human–Animal Interactions in Assessments of Animal Welfare

  |  |  |
  | --- | --- |
  | Item Type | Journal Article |
  | Author | D. Mellor |
  | Author | C. Wilkins |
  | Author | P. McGreevy |
  | Author | A. McLean |
  | Author | B. Jones |
  | Author | K. Littlewood |
  | Author | N. Beausoleil |
  | Date | 2020-10-01 |
  | URL | https://consensus.app/papers/the-2020-five-domains-model-including-human%E2%80%93animal-mellor-wilkins/c390aac677b45a2ebbde016e12b73522/ |
  | Volume | 10 |
  | Publication | Animals : an Open Access Journal from MDPI |
  | DOI | 10.3390/ani10101870 |
  | Journal Abbr | Animals : an Open Access Journal from MDPI |
  | Date Added | 17/06/2025, 18:30:59 |
  | Modified | 17/06/2025, 18:30:59 |
- ## Human-Animal Interactions

  |  |  |
  | --- | --- |
  | Item Type | Journal Article |
  | Author | Megan LaFollette |
  | Date | 2020-09-09 |
  | URL | https://consensus.app/papers/humananimal-interactions-lafollette/1f21ef9cfd055b07a0d7afebc6db76f8/ |
  | Publication | Animal-centric Care and Management |
  | DOI | 10.1201/9780429059544-1 |
  | Journal Abbr | Animal-centric Care and Management |
  | Date Added | 17/06/2025, 18:30:59 |
  | Modified | 17/06/2025, 18:30:59 |
- ## The Human-Animal Interaction at Work Scale: Development and psychometric properties

  |  |  |
  | --- | --- |
  | Item Type | Journal Article |
  | Author | A. Junça‐Silva |
  | Date | 2024-07-01 |
  | URL | https://consensus.app/papers/the-humananimal-interaction-at-work-scale-development-and-jun%C3%A7a%E2%80%90silva/44d95ba5ee9459d8b057a28ea754c998/ |
  | Publication | Journal of Veterinary Behavior |
  | DOI | 10.1016/j.jveb.2024.06.007 |
  | Journal Abbr | Journal of Veterinary Behavior |
  | Date Added | 17/06/2025, 18:30:59 |
  | Modified | 17/06/2025, 18:30:59 |
- ## Variability in Human-Animal Interaction Research

  |  |  |
  | --- | --- |
  | Item Type | Journal Article |
  | Author | N. Gee |
  | Author | Kerri Rodriguez |
  | Author | H. Herzog |
  | Date | 2021-01-15 |
  | URL | https://consensus.app/papers/variability-in-humananimal-interaction-research-gee-rodriguez/f107e4d7aae8564db4918abed4d35d13/ |
  | Volume | 7 |
  | Publication | Frontiers in Veterinary Science |
  | DOI | 10.3389/fvets.2020.619600 |
  | Journal Abbr | Frontiers in Veterinary Science |
  | Date Added | 17/06/2025, 18:30:59 |
  | Modified | 04/01/2026, 11:26:12 |
- ## Human–Animal Interaction Analysis

  |  |  |
  | --- | --- |
  | Item Type | Journal Article |
  | Author | Angela Fournier |
  | Date | 2019-01-01 |
  | URL | https://consensus.app/papers/human%E2%80%93animal-interaction-analysis-fournier/f0d745c27c6c512b956474c61ef450a8/ |
  | Publication | Animal-Assisted Intervention |
  | DOI | 10.1007/978-3-030-32972-3\_2 |
  | Journal Abbr | Animal-Assisted Intervention |
  | Date Added | 17/06/2025, 18:30:59 |
  | Modified | 17/06/2025, 18:30:59 |
- ## Human–Animal Interaction and Older Adults: An Overview

  |  |  |
  | --- | --- |
  | Item Type | Journal Article |
  | Author | A. Curl |
  | Author | M. Mueller |
  | Author | N. Gee |
  | Date | 2017-08-21 |
  | URL | https://consensus.app/papers/human%E2%80%93animal-interaction-and-older-adults-an-overview-curl-mueller/62cd68a37a515eba81be204222b6d800/ |
  | Volume | 8 |
  | Publication | Frontiers in Psychology |
  | DOI | 10.3389/fpsyg.2017.01416 |
  | Journal Abbr | Frontiers in Psychology |
  | Date Added | 17/06/2025, 18:30:59 |
  | Modified | 17/06/2025, 18:30:59 |
- ## The Animal-Human Interface in Farm Animal Production: Animal Fear, Stress, Reproduction and Welfare

  |  |  |
  | --- | --- |
  | Item Type | Journal Article |
  | Author | G. Coleman |
  | Author | P. Hemsworth |
  | Author | R. Acharya |
  | Author | J. Kinder |
  | Date | 2022-02-01 |
  | URL | https://consensus.app/papers/the-animalhuman-interface-in-farm-animal-production-coleman-hemsworth/b1a6ef44c8705219b13595fb204c1bb2/ |
  | Volume | 12 |
  | Publication | Animals : an Open Access Journal from MDPI |
  | DOI | 10.3390/ani12040487 |
  | Journal Abbr | Animals : an Open Access Journal from MDPI |
  | Date Added | 17/06/2025, 18:30:59 |
  | Modified | 17/06/2025, 18:30:59 |
- ## Human–Animal Interaction and Human Prosociality: A Meta-Analytic Review of Experimental and Correlational Studies

  |  |  |
  | --- | --- |
  | Item Type | Journal Article |
  | Author | Nicole Chen |
  | Author | Alycia Ho |
  | Author | Andree Hartanto |
  | Author | K. Kasturiratna |
  | Author | Manmeet Kaur |
  | Author | Nadyanna Majeed |
  | Author | Gloria Lai |
  | Author | Paye Shin Koh |
  | Author | Jose Yong |
  | Date | 2023-12-15 |
  | URL | https://consensus.app/papers/human%E2%80%93animal-interaction-and-human-prosociality-a-chen-ho/e3164a5afc6557a2b4c4f38b22ca4760/ |
  | Volume | 37 |
  | Pages | 269-288 |
  | Publication | Anthrozoös |
  | DOI | 10.1080/08927936.2023.2288745 |
  | Journal Abbr | Anthrozoös |
  | Date Added | 17/06/2025, 18:30:59 |
  | Modified | 17/06/2025, 18:30:59 |
- ## Well-Being over the Life Course: Incorporating Human-Animal Interaction

  |  |  |
  | --- | --- |
  | Item Type | Journal Article |
  | Author | R. Bures |
  | Date | 2020-12-14 |
  | URL | https://consensus.app/papers/wellbeing-over-the-life-course-incorporating-humananimal-bures/db65a83830335c1586ad4981a5a80456/ |
  | Pages | 1-9 |
  | DOI | 10.1007/978-3-030-64085-9\_1 |
  | Date Added | 17/06/2025, 18:30:59 |
  | Modified | 17/06/2025, 18:30:59 |
- ## The Power of a Positive Human–Animal Relationship for Animal Welfare

  |  |  |
  | --- | --- |
  | Item Type | Journal Article |
  | Author | X. Boivin |
  | Author | P. Hemsworth |
  | Author | J. Rault |
  | Author | S. Waiblinger |
  | Date | 2020-11-09 |
  | URL | https://consensus.app/papers/the-power-of-a-positive-human%E2%80%93animal-relationship-for-boivin-hemsworth/64c125aa43f55555ba71107faa821f1e/ |
  | Volume | 7 |
  | Publication | Frontiers in Veterinary Science |
  | DOI | 10.3389/fvets.2020.590867 |
  | Journal Abbr | Frontiers in Veterinary Science |
  | Date Added | 17/06/2025, 18:30:59 |
  | Modified | 17/06/2025, 18:30:59 |
- ## Toward a psychology of human-animal relations.

  |  |  |
  | --- | --- |
  | Item Type | Journal Article |
  | Author | Catherine Amiot |
  | Author | B. Bastian |
  | Date | 2014-11-03 |
  | URL | https://consensus.app/papers/toward-a-psychology-of-humananimal-relations-amiot-bastian/06f7acb38e205c65a64e7e0022944aa8/ |
  | Volume | 141 1 |
  | Pages | 6-47 |
  | Publication | Psychological bulletin |
  | DOI | 10.1037/a0038147 |
  | Journal Abbr | Psychological bulletin |
  | Date Added | 17/06/2025, 18:30:59 |
  | Modified | 17/06/2025, 18:30:59 |
