# Supplementary material for: Evidence of Physiological Comodulation During Human–Animal Interaction: A Systematic Review
Source: Ann N Y Acad Sci. 2026 Jun 4;1560(1):e70299. doi: 10.1111/nyas.70299 (PMC13238372; doi:10.1111/nyas.70299)
Supplement: Supplementary file 2 — Supplementary Materials: Supp2‐Zotero‐Collection.zip [file NYAS-1560-0-s002.zip › Supp2_Zotero_Collection/title screened/EMBase.htm]

Zotero Report


- ## Physical Therapy for Montana Children With Neuromotor Disorders Using Hippotherapy and the Equine Environment

  |  |  |
  | --- | --- |
  | Item Type | Journal Article |
  | Abstract | Brief Summary The goal of this pilot clinical trial is to determine if physical therapy incorporating horses can improve the motor skills of the arms in children 6-17 years old with neuromotor disorders (such as cerebral palsy, spinal muscular atrophy, or spina bifida) compared to standard play-based physical therapy. The main questions it aims to answer are: 1. Is the study protocol feasible and acceptable for participants, that investigators could apply them to a larger trial? 2. Do participants make improvements toward their goals for motor function, arm use, and participation in life situations following treatment, and is it different between the experimental and comparative intervention groups? 3. What are the physiological, behavioral, and emotional responses of children receiving physical therapy incorporating horses, versus those receiving standard physical therapy? Researchers will compare the experimental group who receive physical therapy incorporating horses to the comparative intervention group who receive standard play-based physical therapy to see if there is a difference in outcomes. Participants will complete a pre- and post-intervention assessment of their motor function and participation in life situations. Participants will receive physical therapy twice a week for 8 weeks for the intervention. In both groups, physiological, behavioral, and emotional responses to the interventions will be measured in 4 total sessions, 1 each at weeks 2, 4, 6, and 8. Detailed Description The long-term goal is to increase the variety of effective PT interventions for children with NMD, to achieve optimum outcomes for all. The investigators have already completed a pilot feasibility randomized-controlled trial (RCT) with waitlist control of an intervention targeting upper extremity function and participation in children with neuromotor disorders. The intervention protocol was feasible, but certain aspects of the RCT protocol needed to be revised. Aim 1 of this proposal evaluates the feasibility of these revisions by: 1) establishing an appropriate control condition, 2) evaluating replacement outcome measures for those that were not feasible; and 3) determining effects of a different treatment dosage on retention and attendance. Aim 2 will explore preliminary effects of the intervention on motor function and participation and Aim 3 will explore behavioral and physiological responses. Aim 1: Evaluate the feasibility of the RCT protocol comparing an 8-week PT intervention using hippotherapy and the equine environment with a play-based PT control group targeting motor function and participation for children with NMD. Inestigators will track recruitment, retention, attendance, outcome measure completion, and reasons for absences and withdrawal. Investigators will assess fidelity and acceptability of the treatments for both groups. This will inform design of a large-scale RCT with full statistical power. Investigators hypothesize that they will recruit and randomize 24 participants with \>80% retention, treatment attendance, and outcome measure completion in both groups; and both groups will meet \>80% intervention fidelity and demonstrate acceptability. Aim 2: Identify preliminary effects of an 8-week PT intervention using hippotherapy and the equine environment on motor function and participation compared to play-based PT for children with NMD. Investigators will measure motor function (BOT-3, ABILHANDKIDS), individual goal attainment (GAS), and participation (PEM-CY) before and after the 8-week treatment period for all participants and compare the average change score between the treatment and control groups. Investigators hypothesize that group average scores will improve from pre- to post-treatment for both groups, with a greater change in the treatment group compared to the control group. Aim 3: Compare physiological, behavioral, and emotional responses in children with NMD during PT sessions incorporating equine interaction versus play-based PT. Investigators will calculate changes in high-frequency heart rate variability (HF-HRV) - the gold standard method of measuring parasympathetic nervous system activity - before, during, and after a treatment session. Investigators will measure levels of salivary oxytocin and cortisol before and after a treatment session. Investigators will assess engagement during treatment using behavioral coding and an observational rating scale. Enjoyment will be measured via patient report on a visual-analog scale following treatment. These measures will be collected during 4 treatment sessions (every 2 weeks) throughout the intervention. Investigators hypothesize that the intervention group will demonstrate an immediate increase in HF-HRV and salivary oxytocin, and a decrease in salivary cortisol after a single 60-minute session and sustained over the course of the 8-week intervention; participants will demonstrate sustained high engagement and enjoyment over the course of the intervention, and ratings of engagement and enjoyment will be greater than in the control condition; and physiological changes and behavioral ratings will be significantly positively correlated with the outcomes measured in Aim 2. Background and Rationale There is a growing number of children with NMD across the United States. In rural areas like Montana, access and adherence to PT is especially challenging. Offering PT services in an equine environment can increase access to rural populations and provide PT in setting that might be more motivating and engaging for children, however there is a need to further evaluate its efficacy for improving important outcomes, like motor function and participation. The investigators began to address this need with a previous feasibility RCT which is linked in References. In Aim 1 investigators will implement changes identified to improve feasibility of the RCT protocol: 1) add additional screening to confirm that participants can complete all measures; 2) assess the feasibility of a play-based PT control condition; 3) test the reduced frequency of 2x/ week with a larger sample, and 4) replace the outcome measures that had low completion rates. Results from Aim 1 will inform the design of a large-scale clinical efficacy trial. In Aim 2, investigators will evaluate preliminary efficacy of the PT intervention using hippotherapy and the equine environment. This will provide the justification for a large-scale efficacy trial, and data for a power analysis. PT using hippotherapy and the equine environment involves interaction between the patient and the equine. Emerging research has shown positive physiological and behavioral impacts of human-animal interactions. This PI's pilot study, which is not yet published, is the first known study to evaluate patient behavioral and physiological responses to HEI during a PT session. In Aim 3, investigators will evaluate these responses longitudinally, compare them to a control intervention, and investigate their potential moderation of patient outcomes. Study Design A pilot, randomized-controlled trial with 24 children with NMD who will be assigned to either the PT intervention using hippotherapy and the equine environment or a standard PT control condition. Participants Investigators will recruit 24 children from rehabilitation clinics, schools, and recreational centers around Western Montana. Screening A research assistant will screen interested participants by phone. Those who meet initial inclusion criteria will attend a screening visit to ensure they can safely mount and dismount the equine, can provide a saliva sample, can follow simple verbal instructions, and can ambulate independently. Sample Size The study design was determined using guidance in the NIH document Pilot Studies: Common Uses and Misuses. Investigators concluded that 80%+ power is not necessary for the current proposal because intervention efficacy is not being tested, which would require a larger sample size and length of time than this funding mechanism accommodates. Rather, the sample size is "based on practical considerations including participant flow, budgetary constraints, and the number of participants needed to reasonably evaluate goals."36 A sample size of 24 will allow sufficient participant variability to assess the feasibility of changes made to the RCT protocol and provide insight into preliminary effects of the intervention (see Preliminary Data). The investigators have assembled a strong network of recruitment partners and treatment sites (see Letters of Support) to ensure that completion of study aims with a sample of 24 subjects is achievable within the 12-month timeline. Setting Participant baseline and post-intervention assessments and the control group treatment sessions will take place at the Pediatric Rehabilitation and Active Lifestyle (PALS) Lab at the University of Montana. The PALS lab is a 1000 ft2 space with two private assessment spaces and a waiting area for caregivers and families. The equine intervention will occur at Trotting Horse Therapeutic Riding (THTR) in Missoula, Hobby Horse Place in Polson, and Two Bear Therapeutic Riding Center in Whitefish (see Letters of Support). The facilities have accessible indoor arenas and trained therapy equines and equine professionals. Therapists Therapists for the equine treatment are licensed physical therapists who have the recommended training to incorporate hippotherapy and the equine environment into treatment (American Hippotherapy Association (AHA) Inc. Hippotherapy Treatment Principles Part I and II). Each therapist will work with an equine professional with PATH Intl. Therapeutic Riding Instructor Certification, to ensure that therapists adhere to industry standards for safety and handling of the equines. The standard PT control intervention will be delivered by Doctor of Physical Therapy (DPT) students under the direct supervision of a licensed physical therapist. Outcomes Assessments Pre- and post-outcomes assessments will be completed by DPT students who are trained on standardized application of all measures and under direct supervision of a licensed PT (study PI). The assessors will be blinded to subject group assignments. Measures The following measures will be applied to all participants: Feasibility Fidelity Delivery of intervention Receipt of intervention Reliability: Coders for fidelity will establish ≥85% reliability by coding the same video, comparing codes, discussing any discrepancies, and reaching a consensus on any item where codes were not in agreement. This process will be repeated until the codes are congruent for ≥85% of items for sufficient reliability. Once reliability is established, the remaining videos will be divided amongst coders. Acceptability: The engagement (PRIME-O) and enjoyment (How Happy Scale) measures described below will be used as measures of participant acceptability of the intervention. The following measures will be collected at baseline (week 0) and post-intervention (week 9) for both groups: GMFM-88 ABILHAND-KIDS GAS PEM-CY The following measures will be collected at weeks 2, 4, 6, and 8 for both groups: PRIME-O HF-HRV Salivary Oxytocin Salivary Cortisol Adaptation of How Happy Scale Behavioral Coding Analysis Aim 1: Descriptive statistics wil be used to calculate rates of enrollment, attendance, attrition, fidelity, and outcome measure completion. Reasons for study withdrawal, missed therapy sessions, or incomplete outcome assessments will be documented. Rates will be compared between the treatment and control groups using independent samples t-tests for attendance, attrition, and measure completion and GLMMRM for fidelity. Aim 2: Changes in outcome scores from pre- to post-intervention will be compared between groups using generalized linear mixed models for repeated measures (GLMMRM). Where available, the percent of participants who meet a minimal clinically important difference (MCID) from pre- to post-intervention will be reported. Aim 3: HF-HRV will be calculated for the pre-, during-, and post-session intervals and compare these means. Pre-post session changes in salivary oxytocin and cortisol will be compared. HF-HRV pre-to during-session mean change and oxytocin and cortisol pre- to post-session mean changes between groups will be compared. For the behavioral data, the percentage of scans where behaviors indicate a positive or negative mood will be calculated and the average frequency of each determined and compare between groups. Longitudinal changes will be compared for all variables throughout the intervention. GLMMRM will be used for all comparisons. |
  | Date | 2025 |
  | Language | English |
  | URL | https://www.embase.com/search/results?subaction=viewrecord&id=LNCT06887647&from=export |
  | Publication | clinicaltrials.gov |
  | Journal Abbr | clinicaltrials.gov |
  | Date Added | 11/07/2025, 11:53:16 |
  | Modified | 11/07/2025, 11:53:16 |

  ### Tags:

  - oxytocin
  - human-animal interaction
  - horse
  - heart rate variability
  - emotion
  - child
  - human
  - hydrocortisone
  - controlled study
  - hippotherapy
  - physiotherapy
  - pilot study
  - randomized controlled trial
  - leisure
  - caregiver
  - cerebral palsy
  - certification
  - cholinergic system
  - clinical trial
  - coregonine
  - goal attainment
  - Gross Motor Function Measure
  - group therapy
  - minimal clinically important difference
  - Montana
  - mood
  - motoneuron
  - motor performance
  - neural tube defect
  - outcome assessment
  - participation
  - pediatric rehabilitation
  - physiotherapist
  - power analysis
  - preliminary data
  - rating scale
  - rehabilitation center
  - reliability
  - rural area
  - rural population
  - spinal dysraphism
  - spinal muscular atrophy
  - treatment outcome
  - upper limb
  - videorecording
  - visual analog scale

  ### Attachments

  - Full Text (HTML)
- ## Physical Therapy for Montana Children With Neuromotor Disorders Using Hippotherapy and the Equine Environment

  |  |  |
  | --- | --- |
  | Item Type | Journal Article |
  | Abstract | Brief Summary The goal of this pilot clinical trial is to determine if physical therapy incorporating horses can improve the motor skills of the arms in children 6-17 years old with neuromotor disorders (such as cerebral palsy, spinal muscular atrophy, or spina bifida) compared to standard play-based physical therapy. The main questions it aims to answer are: 1. Is the study protocol feasible and acceptable for participants, that investigators could apply them to a larger trial? 2. Do participants make improvements toward their goals for motor function, arm use, and participation in life situations following treatment, and is it different between the experimental and comparative intervention groups? 3. What are the physiological, behavioral, and emotional responses of children receiving physical therapy incorporating horses, versus those receiving standard physical therapy? Researchers will compare the experimental group who receive physical therapy incorporating horses to the comparative intervention group who receive standard play-based physical therapy to see if there is a difference in outcomes. Participants will complete a pre- and post-intervention assessment of their motor function and participation in life situations. Participants will receive physical therapy twice a week for 8 weeks for the intervention. In both groups, physiological, behavioral, and emotional responses to the interventions will be measured in 4 total sessions, 1 each at weeks 2, 4, 6, and 8. Detailed Description The long-term goal is to increase the variety of effective PT interventions for children with NMD, to achieve optimum outcomes for all. The investigators have already completed a pilot feasibility randomized-controlled trial (RCT) with waitlist control of an intervention targeting upper extremity function and participation in children with neuromotor disorders. The intervention protocol was feasible, but certain aspects of the RCT protocol needed to be revised. Aim 1 of this proposal evaluates the feasibility of these revisions by: 1) establishing an appropriate control condition, 2) evaluating replacement outcome measures for those that were not feasible; and 3) determining effects of a different treatment dosage on retention and attendance. Aim 2 will explore preliminary effects of the intervention on motor function and participation and Aim 3 will explore behavioral and physiological responses. Aim 1: Evaluate the feasibility of the RCT protocol comparing an 8-week PT intervention using hippotherapy and the equine environment with a play-based PT control group targeting motor function and participation for children with NMD. Inestigators will track recruitment, retention, attendance, outcome measure completion, and reasons for absences and withdrawal. Investigators will assess fidelity and acceptability of the treatments for both groups. This will inform design of a large-scale RCT with full statistical power. Investigators hypothesize that they will recruit and randomize 24 participants with \>80% retention, treatment attendance, and outcome measure completion in both groups; and both groups will meet \>80% intervention fidelity and demonstrate acceptability. Aim 2: Identify preliminary effects of an 8-week PT intervention using hippotherapy and the equine environment on motor function and participation compared to play-based PT for children with NMD. Investigators will measure motor function (BOT-3, ABILHANDKIDS), individual goal attainment (GAS), and participation (PEM-CY) before and after the 8-week treatment period for all participants and compare the average change score between the treatment and control groups. Investigators hypothesize that group average scores will improve from pre- to post-treatment for both groups, with a greater change in the treatment group compared to the control group. Aim 3: Compare physiological, behavioral, and emotional responses in children with NMD during PT sessions incorporating equine interaction versus play-based PT. Investigators will calculate changes in high-frequency heart rate variability (HF-HRV) - the gold standard method of measuring parasympathetic nervous system activity - before, during, and after a treatment session. Investigators will measure levels of salivary oxytocin and cortisol before and after a treatment session. Investigators will assess engagement during treatment using behavioral coding and an observational rating scale. Enjoyment will be measured via patient report on a visual-analog scale following treatment. These measures will be collected during 4 treatment sessions (every 2 weeks) throughout the intervention. Investigators hypothesize that the intervention group will demonstrate an immediate increase in HF-HRV and salivary oxytocin, and a decrease in salivary cortisol after a single 60-minute session and sustained over the course of the 8-week intervention; participants will demonstrate sustained high engagement and enjoyment over the course of the intervention, and ratings of engagement and enjoyment will be greater than in the control condition; and physiological changes and behavioral ratings will be significantly positively correlated with the outcomes measured in Aim 2. Background and Rationale There is a growing number of children with NMD across the United States. In rural areas like Montana, access and adherence to PT is especially challenging. Offering PT services in an equine environment can increase access to rural populations and provide PT in setting that might be more motivating and engaging for children, however there is a need to further evaluate its efficacy for improving important outcomes, like motor function and participation. The investigators began to address this need with a previous feasibility RCT which is linked in References. In Aim 1 investigators will implement changes identified to improve feasibility of the RCT protocol: 1) add additional screening to confirm that participants can complete all measures; 2) assess the feasibility of a play-based PT control condition; 3) test the reduced frequency of 2x/ week with a larger sample, and 4) replace the outcome measures that had low completion rates. Results from Aim 1 will inform the design of a large-scale clinical efficacy trial. In Aim 2, investigators will evaluate preliminary efficacy of the PT intervention using hippotherapy and the equine environment. This will provide the justification for a large-scale efficacy trial, and data for a power analysis. PT using hippotherapy and the equine environment involves interaction between the patient and the equine. Emerging research has shown positive physiological and behavioral impacts of human-animal interactions. This PI's pilot study, which is not yet published, is the first known study to evaluate patient behavioral and physiological responses to HEI during a PT session. In Aim 3, investigators will evaluate these responses longitudinally, compare them to a control intervention, and investigate their potential moderation of patient outcomes. Study Design A pilot, randomized-controlled trial with 24 children with NMD who will be assigned to either the PT intervention using hippotherapy and the equine environment or a standard PT control condition. Participants Investigators will recruit 24 children from rehabilitation clinics, schools, and recreational centers around Western Montana. Screening A research assistant will screen interested participants by phone. Those who meet initial inclusion criteria will attend a screening visit to ensure they can safely mount and dismount the equine, can provide a saliva sample, can follow simple verbal instructions, and can ambulate independently. Sample Size The study design was determined using guidance in the NIH document Pilot Studies: Common Uses and Misuses. Investigators concluded that 80%+ power is not necessary for the current proposal because intervention efficacy is not being tested, which would require a larger sample size and length of time than this funding mechanism accommodates. Rather, the sample size is "based on practical considerations including participant flow, budgetary constraints, and the number of participants needed to reasonably evaluate goals."36 A sample size of 24 will allow sufficient participant variability to assess the feasibility of changes made to the RCT protocol and provide insight into preliminary effects of the intervention (see Preliminary Data). The investigators have assembled a strong network of recruitment partners and treatment sites (see Letters of Support) to ensure that completion of study aims with a sample of 24 subjects is achievable within the 12-month timeline. Setting Participant baseline and post-intervention assessments and the control group treatment sessions will take place at the Pediatric Rehabilitation and Active Lifestyle (PALS) Lab at the University of Montana. The PALS lab is a 1000 ft2 space with two private assessment spaces and a waiting area for caregivers and families. The equine intervention will occur at Trotting Horse Therapeutic Riding (THTR) in Missoula, Hobby Horse Place in Polson, and Two Bear Therapeutic Riding Center in Whitefish (see Letters of Support). The facilities have accessible indoor arenas and trained therapy equines and equine professionals. Therapists Therapists for the equine treatment are licensed physical therapists who have the recommended training to incorporate hippotherapy and the equine environment into treatment (American Hippotherapy Association (AHA) Inc. Hippotherapy Treatment Principles Part I and II). Each therapist will work with an equine professional with PATH Intl. Therapeutic Riding Instructor Certification, to ensure that therapists adhere to industry standards for safety and handling of the equines. The standard PT control intervention will be delivered by Doctor of Physical Therapy (DPT) students under the direct supervision of a licensed physical therapist. Outcomes Assessments Pre- and post-outcomes assessments will be completed by DPT students who are trained on standardized application of all measures and under direct supervision of a licensed PT (study PI). The assessors will be blinded to subject group assignments. Measures The following measures will be applied to all participants: Feasibility Fidelity Delivery of intervention Receipt of intervention Reliability: Coders for fidelity will establish ≥85% reliability by coding the same video, comparing codes, discussing any discrepancies, and reaching a consensus on any item where codes were not in agreement. This process will be repeated until the codes are congruent for ≥85% of items for sufficient reliability. Once reliability is established, the remaining videos will be divided amongst coders. Acceptability: The engagement (PRIME-O) and enjoyment (How Happy Scale) measures described below will be used as measures of participant acceptability of the intervention. The following measures will be collected at baseline (week 0) and post-intervention (week 9) for both groups: GMFM-88 ABILHAND-KIDS GAS PEM-CY The following measures will be collected at weeks 2, 4, 6, and 8 for both groups: PRIME-O HF-HRV Salivary Oxytocin Salivary Cortisol Adaptation of How Happy Scale Behavioral Coding Analysis Aim 1: Descriptive statistics wil be used to calculate rates of enrollment, attendance, attrition, fidelity, and outcome measure completion. Reasons for study withdrawal, missed therapy sessions, or incomplete outcome assessments will be documented. Rates will be compared between the treatment and control groups using independent samples t-tests for attendance, attrition, and measure completion and GLMMRM for fidelity. Aim 2: Changes in outcome scores from pre- to post-intervention will be compared between groups using generalized linear mixed models for repeated measures (GLMMRM). Where available, the percent of participants who meet a minimal clinically important difference (MCID) from pre- to post-intervention will be reported. Aim 3: HF-HRV will be calculated for the pre-, during-, and post-session intervals and compare these means. Pre-post session changes in salivary oxytocin and cortisol will be compared. HF-HRV pre-to during-session mean change and oxytocin and cortisol pre- to post-session mean changes between groups will be compared. For the behavioral data, the percentage of scans where behaviors indicate a positive or negative mood will be calculated and the average frequency of each determined and compare between groups. Longitudinal changes will be compared for all variables throughout the intervention. GLMMRM will be used for all comparisons. |
  | Date | 2025 |
  | Language | English |
  | URL | https://www.embase.com/search/results?subaction=viewrecord&id=LNCT06887647&from=export |
  | Publication | clinicaltrials.gov |
  | Journal Abbr | clinicaltrials.gov |
  | Date Added | 11/07/2025, 12:10:37 |
  | Modified | 11/07/2025, 12:10:37 |

  ### Tags:

  - oxytocin
  - human-animal interaction
  - horse
  - heart rate variability
  - emotion
  - child
  - human
  - hydrocortisone
  - controlled study
  - hippotherapy
  - physiotherapy
  - pilot study
  - randomized controlled trial
  - leisure
  - caregiver
  - cerebral palsy
  - certification
  - cholinergic system
  - clinical trial
  - coregonine
  - goal attainment
  - Gross Motor Function Measure
  - group therapy
  - minimal clinically important difference
  - Montana
  - mood
  - motoneuron
  - motor performance
  - neural tube defect
  - outcome assessment
  - participation
  - pediatric rehabilitation
  - physiotherapist
  - power analysis
  - preliminary data
  - rating scale
  - rehabilitation center
  - reliability
  - rural area
  - rural population
  - spinal dysraphism
  - spinal muscular atrophy
  - treatment outcome
  - upper limb
  - videorecording
  - visual analog scale

  ### Attachments

  - Full Text (HTML)
- ## Evaluating the Efficacy of a Service Dog Training Program for Military Veterans With PTSD

  |  |  |
  | --- | --- |
  | Item Type | Journal Article |
  | Abstract | Brief Summary Post-traumatic stress disorder (PTSD), an invisible wound of war, affects approximately 20%1 of the 18.5 million U.S. veterans and places them at higher risk for impaired biopsychosocial functioning. PTSD symptom severity (PTSDSS) is significantly correlated with stress and psychosocial consequences of inability to regulate emotions, control impulsive behaviors, and function within family and society. Alarming veteran PTSD rates and its insidious effects demand empirically validated treatment programs. More than a million veterans receive new diagnoses of each year. VA PTSD therapy programs reach only 1% of veterans. Nearly 35% of veterans do not respond to widely used psychotherapy and pharmacotherapy treatments. Training a service dog (SD) is a novel rehabilitative animal-assisted intervention that shows promise in other populations. This project evaluates the efficacy of a service dog training program (SDTP) as an alternative and adjunctive treatment and rehabilitative option for veterans with PTSD. Detailed Description Veterans with PTSD attend 8 weekly sessions of either training a service dog or learning about how to train a dog at the Warrior Canine Connection facility in Boyds MD. They complete questionnaires prior to the start of the program and at the midpoint (after 4 sessions) and end of the program (after 8 sessions). Participants also wear a monitor to record heart rate variability during the 1st, 4th, and 8th sessions and provide saliva samples at these same times. |
  | Date | 2018 |
  | Language | English |
  | URL | https://www.embase.com/search/results?subaction=viewrecord&id=LNCT03777020&from=export |
  | Publication | clinicaltrials.gov |
  | Journal Abbr | clinicaltrials.gov |
  | Date Added | 11/07/2025, 12:10:54 |
  | Modified | 11/07/2025, 12:10:54 |

  ### Tags:

  - human-animal interaction
  - veteran
  - heart rate variability
  - dog
  - therapy
  - emotion
  - animal experiment
  - female
  - male
  - physiological stress
  - human
  - adult
  - aged
  - controlled study
  - posttraumatic stress disorder
  - questionnaire
  - clinical trial
  - training
  - service dog
  - army
  - disease severity
  - drug therapy
  - impulsiveness
  - psychotherapy

  ### Attachments

  - Full Text (HTML)
- ## Assessing Mechanisms of Anxiety Reduction in Animal-assisted Interventions for Adolescents With Social aAnxiety

  |  |  |
  | --- | --- |
  | Item Type | Journal Article |
  | Abstract | Brief Summary Adolescence and young adulthood is a critical period for the development of social anxiety, which is often linked to other mental health challenges such as depression, mood disorders, and substance abuse. Initial evidence suggests that interacting with animals can reduce stress and anxiety, but no research has tested whether this benefit extends to adolescents at risk for social anxiety disorder. Additionally, researchers and clinicians do not understand what mechanism is responsible for anxiety reduction in animal-assisted interventions (AAIs). Therefore, the objectives of this study are to explore the specific mechanisms by which interacting with a therapy dog reduces anxiety, and to test whether such an interaction reduces anxiety in adolescents with varying levels of social anxiety. Detailed Description The specific aims of this project are to (1) test the mechanisms by which AAIs reduce anxiety, and (2) determine if the anxiolytic effect of social and physical interaction is moderated by level of pre-existing social anxiety. To achieve these aims, 75 adolescents (age 13-17) will undergo a well-validated laboratory-based social evaluative stressor, the Trier Social Stress Task for Children, and be randomly assigned to one of three conditions: 1) no interaction with a dog (control condition), 2) social interaction only (no physical interaction) with a therapy dog; or 3) social interaction plus physical interaction with a therapy dog. Using a multivariate approach, three levels of outcome data will be collected: a) self-reported experience (anxiety), b) autonomic physiology (heart rate), and c) behavioral performance (error rates on mental math task). In addition, the interactions will be videotaped and behavioral coding will be used to explore the specific social behaviors between the participant and the dog that may predict anxiety reduction (such as frequency or type of social referencing or physical contact). |
  | Date | 2017 |
  | Language | English |
  | URL | https://www.embase.com/search/results?subaction=viewrecord&id=LNCT03249116&from=export |
  | Publication | clinicaltrials.gov |
  | Journal Abbr | clinicaltrials.gov |
  | Date Added | 11/07/2025, 12:10:55 |
  | Modified | 11/07/2025, 12:10:55 |

  ### Tags:

  - human-animal interaction
  - heart rate
  - anxiety
  - therapy
  - social behavior
  - child
  - male
  - physiological stress
  - social interaction
  - human
  - adolescent
  - major clinical study
  - clinical trial
  - mood disorder
  - anxiolytic agent
  - childhood disease
  - depression
  - mental health
  - social anxiety
  - social phobia
  - substance abuse
  - Trier Social Stress Test

  ### Attachments

  - Full Text (HTML)
- ## Physical Therapy for Montana Children With Neuromotor Disorders Using Hippotherapy and the Equine Environment

  |  |  |
  | --- | --- |
  | Item Type | Journal Article |
  | Abstract | Brief Summary The goal of this pilot clinical trial is to determine if physical therapy incorporating horses can improve the motor skills of the arms in children 6-17 years old with neuromotor disorders (such as cerebral palsy, spinal muscular atrophy, or spina bifida) compared to standard play-based physical therapy. The main questions it aims to answer are: 1. Is the study protocol feasible and acceptable for participants, that investigators could apply them to a larger trial? 2. Do participants make improvements toward their goals for motor function, arm use, and participation in life situations following treatment, and is it different between the experimental and comparative intervention groups? 3. What are the physiological, behavioral, and emotional responses of children receiving physical therapy incorporating horses, versus those receiving standard physical therapy? Researchers will compare the experimental group who receive physical therapy incorporating horses to the comparative intervention group who receive standard play-based physical therapy to see if there is a difference in outcomes. Participants will complete a pre- and post-intervention assessment of their motor function and participation in life situations. Participants will receive physical therapy twice a week for 8 weeks for the intervention. In both groups, physiological, behavioral, and emotional responses to the interventions will be measured in 4 total sessions, 1 each at weeks 2, 4, 6, and 8. Detailed Description The long-term goal is to increase the variety of effective PT interventions for children with NMD, to achieve optimum outcomes for all. The investigators have already completed a pilot feasibility randomized-controlled trial (RCT) with waitlist control of an intervention targeting upper extremity function and participation in children with neuromotor disorders. The intervention protocol was feasible, but certain aspects of the RCT protocol needed to be revised. Aim 1 of this proposal evaluates the feasibility of these revisions by: 1) establishing an appropriate control condition, 2) evaluating replacement outcome measures for those that were not feasible; and 3) determining effects of a different treatment dosage on retention and attendance. Aim 2 will explore preliminary effects of the intervention on motor function and participation and Aim 3 will explore behavioral and physiological responses. Aim 1: Evaluate the feasibility of the RCT protocol comparing an 8-week PT intervention using hippotherapy and the equine environment with a play-based PT control group targeting motor function and participation for children with NMD. Inestigators will track recruitment, retention, attendance, outcome measure completion, and reasons for absences and withdrawal. Investigators will assess fidelity and acceptability of the treatments for both groups. This will inform design of a large-scale RCT with full statistical power. Investigators hypothesize that they will recruit and randomize 24 participants with \>80% retention, treatment attendance, and outcome measure completion in both groups; and both groups will meet \>80% intervention fidelity and demonstrate acceptability. Aim 2: Identify preliminary effects of an 8-week PT intervention using hippotherapy and the equine environment on motor function and participation compared to play-based PT for children with NMD. Investigators will measure motor function (BOT-3, ABILHANDKIDS), individual goal attainment (GAS), and participation (PEM-CY) before and after the 8-week treatment period for all participants and compare the average change score between the treatment and control groups. Investigators hypothesize that group average scores will improve from pre- to post-treatment for both groups, with a greater change in the treatment group compared to the control group. Aim 3: Compare physiological, behavioral, and emotional responses in children with NMD during PT sessions incorporating equine interaction versus play-based PT. Investigators will calculate changes in high-frequency heart rate variability (HF-HRV) - the gold standard method of measuring parasympathetic nervous system activity - before, during, and after a treatment session. Investigators will measure levels of salivary oxytocin and cortisol before and after a treatment session. Investigators will assess engagement during treatment using behavioral coding and an observational rating scale. Enjoyment will be measured via patient report on a visual-analog scale following treatment. These measures will be collected during 4 treatment sessions (every 2 weeks) throughout the intervention. Investigators hypothesize that the intervention group will demonstrate an immediate increase in HF-HRV and salivary oxytocin, and a decrease in salivary cortisol after a single 60-minute session and sustained over the course of the 8-week intervention; participants will demonstrate sustained high engagement and enjoyment over the course of the intervention, and ratings of engagement and enjoyment will be greater than in the control condition; and physiological changes and behavioral ratings will be significantly positively correlated with the outcomes measured in Aim 2. Background and Rationale There is a growing number of children with NMD across the United States. In rural areas like Montana, access and adherence to PT is especially challenging. Offering PT services in an equine environment can increase access to rural populations and provide PT in setting that might be more motivating and engaging for children, however there is a need to further evaluate its efficacy for improving important outcomes, like motor function and participation. The investigators began to address this need with a previous feasibility RCT which is linked in References. In Aim 1 investigators will implement changes identified to improve feasibility of the RCT protocol: 1) add additional screening to confirm that participants can complete all measures; 2) assess the feasibility of a play-based PT control condition; 3) test the reduced frequency of 2x/ week with a larger sample, and 4) replace the outcome measures that had low completion rates. Results from Aim 1 will inform the design of a large-scale clinical efficacy trial. In Aim 2, investigators will evaluate preliminary efficacy of the PT intervention using hippotherapy and the equine environment. This will provide the justification for a large-scale efficacy trial, and data for a power analysis. PT using hippotherapy and the equine environment involves interaction between the patient and the equine. Emerging research has shown positive physiological and behavioral impacts of human-animal interactions. This PI's pilot study, which is not yet published, is the first known study to evaluate patient behavioral and physiological responses to HEI during a PT session. In Aim 3, investigators will evaluate these responses longitudinally, compare them to a control intervention, and investigate their potential moderation of patient outcomes. Study Design A pilot, randomized-controlled trial with 24 children with NMD who will be assigned to either the PT intervention using hippotherapy and the equine environment or a standard PT control condition. Participants Investigators will recruit 24 children from rehabilitation clinics, schools, and recreational centers around Western Montana. Screening A research assistant will screen interested participants by phone. Those who meet initial inclusion criteria will attend a screening visit to ensure they can safely mount and dismount the equine, can provide a saliva sample, can follow simple verbal instructions, and can ambulate independently. Sample Size The study design was determined using guidance in the NIH document Pilot Studies: Common Uses and Misuses. Investigators concluded that 80%+ power is not necessary for the current proposal because intervention efficacy is not being tested, which would require a larger sample size and length of time than this funding mechanism accommodates. Rather, the sample size is "based on practical considerations including participant flow, budgetary constraints, and the number of participants needed to reasonably evaluate goals."36 A sample size of 24 will allow sufficient participant variability to assess the feasibility of changes made to the RCT protocol and provide insight into preliminary effects of the intervention (see Preliminary Data). The investigators have assembled a strong network of recruitment partners and treatment sites (see Letters of Support) to ensure that completion of study aims with a sample of 24 subjects is achievable within the 12-month timeline. Setting Participant baseline and post-intervention assessments and the control group treatment sessions will take place at the Pediatric Rehabilitation and Active Lifestyle (PALS) Lab at the University of Montana. The PALS lab is a 1000 ft2 space with two private assessment spaces and a waiting area for caregivers and families. The equine intervention will occur at Trotting Horse Therapeutic Riding (THTR) in Missoula, Hobby Horse Place in Polson, and Two Bear Therapeutic Riding Center in Whitefish (see Letters of Support). The facilities have accessible indoor arenas and trained therapy equines and equine professionals. Therapists Therapists for the equine treatment are licensed physical therapists who have the recommended training to incorporate hippotherapy and the equine environment into treatment (American Hippotherapy Association (AHA) Inc. Hippotherapy Treatment Principles Part I and II). Each therapist will work with an equine professional with PATH Intl. Therapeutic Riding Instructor Certification, to ensure that therapists adhere to industry standards for safety and handling of the equines. The standard PT control intervention will be delivered by Doctor of Physical Therapy (DPT) students under the direct supervision of a licensed physical therapist. Outcomes Assessments Pre- and post-outcomes assessments will be completed by DPT students who are trained on standardized application of all measures and under direct supervision of a licensed PT (study PI). The assessors will be blinded to subject group assignments. Measures The following measures will be applied to all participants: Feasibility Fidelity Delivery of intervention Receipt of intervention Reliability: Coders for fidelity will establish ≥85% reliability by coding the same video, comparing codes, discussing any discrepancies, and reaching a consensus on any item where codes were not in agreement. This process will be repeated until the codes are congruent for ≥85% of items for sufficient reliability. Once reliability is established, the remaining videos will be divided amongst coders. Acceptability: The engagement (PRIME-O) and enjoyment (How Happy Scale) measures described below will be used as measures of participant acceptability of the intervention. The following measures will be collected at baseline (week 0) and post-intervention (week 9) for both groups: GMFM-88 ABILHAND-KIDS GAS PEM-CY The following measures will be collected at weeks 2, 4, 6, and 8 for both groups: PRIME-O HF-HRV Salivary Oxytocin Salivary Cortisol Adaptation of How Happy Scale Behavioral Coding Analysis Aim 1: Descriptive statistics wil be used to calculate rates of enrollment, attendance, attrition, fidelity, and outcome measure completion. Reasons for study withdrawal, missed therapy sessions, or incomplete outcome assessments will be documented. Rates will be compared between the treatment and control groups using independent samples t-tests for attendance, attrition, and measure completion and GLMMRM for fidelity. Aim 2: Changes in outcome scores from pre- to post-intervention will be compared between groups using generalized linear mixed models for repeated measures (GLMMRM). Where available, the percent of participants who meet a minimal clinically important difference (MCID) from pre- to post-intervention will be reported. Aim 3: HF-HRV will be calculated for the pre-, during-, and post-session intervals and compare these means. Pre-post session changes in salivary oxytocin and cortisol will be compared. HF-HRV pre-to during-session mean change and oxytocin and cortisol pre- to post-session mean changes between groups will be compared. For the behavioral data, the percentage of scans where behaviors indicate a positive or negative mood will be calculated and the average frequency of each determined and compare between groups. Longitudinal changes will be compared for all variables throughout the intervention. GLMMRM will be used for all comparisons. |
  | Date | 2025 |
  | Language | English |
  | URL | https://www.embase.com/search/results?subaction=viewrecord&id=LNCT06887647&from=export |
  | Publication | clinicaltrials.gov |
  | Journal Abbr | clinicaltrials.gov |
  | Date Added | 11/07/2025, 12:12:13 |
  | Modified | 11/07/2025, 12:12:13 |

  ### Tags:

  - oxytocin
  - human-animal interaction
  - horse
  - heart rate variability
  - emotion
  - child
  - human
  - hydrocortisone
  - controlled study
  - hippotherapy
  - physiotherapy
  - pilot study
  - randomized controlled trial
  - leisure
  - caregiver
  - cerebral palsy
  - certification
  - cholinergic system
  - clinical trial
  - coregonine
  - goal attainment
  - Gross Motor Function Measure
  - group therapy
  - minimal clinically important difference
  - Montana
  - mood
  - motoneuron
  - motor performance
  - neural tube defect
  - outcome assessment
  - participation
  - pediatric rehabilitation
  - physiotherapist
  - power analysis
  - preliminary data
  - rating scale
  - rehabilitation center
  - reliability
  - rural area
  - rural population
  - spinal dysraphism
  - spinal muscular atrophy
  - treatment outcome
  - upper limb
  - videorecording
  - visual analog scale

  ### Attachments

  - Full Text (HTML)
- ## Effects of Intranasal Oxytocin on Trust Towards Therapists and Dogs: A Randomized Controlled Trial in Healthy Adults

  |  |  |
  | --- | --- |
  | Item Type | Journal Article |
  | Abstract | Brief Summary Oxytocin has been proposed as a neuroendocrine mechanism that may mediate the relationship between dog ownership and positive health outcomes and be linked to human-dog interactions and is thought to be a mechanism of interspecies bonding. While the role of oxytocin in human bonding behaviours and social behaviour, in general, is becoming well-established the role of oxytocin in human-animal interaction and Animal-Assisted Interventions (AAI) remains unclear. This research gap calls for more high-quality research investigating this possible neuroendocrine underlying mechanism to advance knowledge about AAI. If oxytocin indeed might be involved in interspecies bonding, intranasally administered oxytocin should not only enhance trust toward a human but also towards a dog. |
  | Date | 2024 |
  | Language | English |
  | URL | https://www.embase.com/search/results?subaction=viewrecord&id=LNCT06248710&from=export |
  | Publication | clinicaltrials.gov |
  | Journal Abbr | clinicaltrials.gov |
  | Date Added | 11/07/2025, 12:12:16 |
  | Modified | 11/07/2025, 12:12:16 |

  ### Tags:

  - oxytocin
  - human-animal interaction
  - dog
  - social behavior
  - human
  - controlled study
  - randomized controlled trial
  - human experiment
  - normal human
  - clinical trial
  - depression
  - nose spray

  ### Attachments

  - Full Text (HTML)
- ## Animal Assisted Intervention for Hemodialysis Outpatients: A Mixed-method Randomized Controlled Trial for Treatment Adherence and Psychosocial Well-being

  |  |  |
  | --- | --- |
  | Item Type | Journal Article |
  | Abstract | Brief Summary The goal of this clinical trial is to understand if and how an animal-assisted intervention \[AAI\] using therapy dogs can support hemodialysis \[HD\] patients' treatment adherence and enhance their well-being. The main objectives are: \* Objective 1: Determine if the AAI impacts patients' HD treatment adherence (primary outcome is number of unplanned missed treatments no due to hospitalization). \* Objective 2: Evaluate if the AAI impacts patients' psychosocial well-being (secondary outcomes are stress, pain, mood, QOL). \* Objective 3: Examine potential mechanistic biomarkers that underpin human-animal bonding (hormones tied to stress and bonding). (exploratory aim) \* Objective 4: Understand patients' subjective experiences of the AAI. Participants will be asked to engage in several research tasks, including: \* assessments \* therapy dog visits \* monthly blood draws \* focus group Researchers will compare how the treatment group (those who receive 2 dogs visits per week) and the control group (those who receive 0 dog visits per week) to see if the AAI impacts treatment adherence and psychosocial well-being. Detailed Description This study will take place in one outpatient dialysis clinic. The clinical trial will compare a standardized therapy dog interaction delivered twice weekly (based on pilot study outcomes detailed below) to usual care (e.g., no dog exposure) using a 1:1 randomized, 2-arm design. Subjective patient-reported outcomes \[PRO\] and routinely-tracked clinic data (e.g., missed visits) will be used. A total of 30 patients will be recruited then randomized 1:1 into 2 arms: control group with 0 dog visits (n = 15) and intervention group with 2 dog visits per week for 20 weeks (n = 15). The AAI is designed to promote patient comfort, uplift mood, and provide an opportunity for socialization. The nature of the dog interaction involves several different components, including but not limited to: petting the dog, talking to the dog, watching the dog do tricks, conversing with the dog handler, and being prompted to discuss any fond memories/stories of their own personal experiences with human-animal interactions. Regarding duration, the dog visits average 10 minutes but will be allowed to vary, and length of dog visits will be tracked. Each team (dog handler + dog) will have an assigned unique ID so that variation in dog is controlled for. This study will utilize trained certified dog handlers to deliver the intervention from reputable local and national pet therapy organizations. All dogs have gone through extensive training and behavioral assessment, will provide proper documentation, liability insurance, and vaccinations. All study procedures take place in the clinic waiting room. Patient-reported outcomes (PRO)s will be collected as pre-post data (before and after dog visit or lobby-as-usual control condition). All PROs will be collected electronically on iPads using REDcap. Regarding treatment adherence data, the clinic routinely tracks missed appointments and will report these metrics directly to research team. Patients will complete assessments 2 times per week for a total of 20 weeks. A short demographic questionnaire will be administered once at the first study visit. Also, patients will also undergo a monthly blood-draw providing 3mL of blood 1 time a month at the same time as their standard of care blood draw. This will be bio-banked in a repository for later ELISA analyses, which will focus on hormones related to stress and bonding (e.g., oxytocin, cortisol); this may provide insight into mechanistic biomarkers underpinning the human-animal bonding process. Lastly, qualitative focus groups with intervention group participants will be conducted post-trial to learn about their subjective experiences of the AAI. |
  | Date | 2023 |
  | Language | English |
  | URL | https://www.embase.com/search/results?subaction=viewrecord&id=LNCT06030050&from=export |
  | Publication | clinicaltrials.gov |
  | Journal Abbr | clinicaltrials.gov |
  | Date Added | 11/07/2025, 12:12:17 |
  | Modified | 11/07/2025, 12:12:17 |

  ### Tags:

  - oxytocin
  - human-animal interaction
  - dog
  - therapy
  - female
  - male
  - human
  - hydrocortisone
  - adult
  - controlled study
  - ELISA kit
  - pilot study
  - randomized controlled trial
  - tablet computer
  - hospitalization
  - quality of life
  - questionnaire
  - major clinical study
  - patient compliance
  - clinical trial
  - mood
  - pain
  - human-animal bond
  - enzyme linked immunosorbent assay
  - biological marker
  - social well-being
  - pet therapy
  - benchmarking
  - chronic kidney failure
  - chronic pain
  - end stage renal disease
  - hemodialysis
  - liability insurance
  - lobbying
  - outpatient
  - patient comfort
  - patient-reported outcome
  - personal experience
  - socialization
  - vaccination
  - waiting room

  ### Attachments

  - Full Text (HTML)
- ## Physical Therapy for Montana Children With Neuromotor Disorders Using Hippotherapy and the Equine Environment

  |  |  |
  | --- | --- |
  | Item Type | Journal Article |
  | Abstract | Brief Summary The goal of this pilot clinical trial is to determine if physical therapy incorporating horses can improve the motor skills of the arms in children 6-17 years old with neuromotor disorders (such as cerebral palsy, spinal muscular atrophy, or spina bifida) compared to standard play-based physical therapy. The main questions it aims to answer are: 1. Is the study protocol feasible and acceptable for participants, that investigators could apply them to a larger trial? 2. Do participants make improvements toward their goals for motor function, arm use, and participation in life situations following treatment, and is it different between the experimental and comparative intervention groups? 3. What are the physiological, behavioral, and emotional responses of children receiving physical therapy incorporating horses, versus those receiving standard physical therapy? Researchers will compare the experimental group who receive physical therapy incorporating horses to the comparative intervention group who receive standard play-based physical therapy to see if there is a difference in outcomes. Participants will complete a pre- and post-intervention assessment of their motor function and participation in life situations. Participants will receive physical therapy twice a week for 8 weeks for the intervention. In both groups, physiological, behavioral, and emotional responses to the interventions will be measured in 4 total sessions, 1 each at weeks 2, 4, 6, and 8. Detailed Description The long-term goal is to increase the variety of effective PT interventions for children with NMD, to achieve optimum outcomes for all. The investigators have already completed a pilot feasibility randomized-controlled trial (RCT) with waitlist control of an intervention targeting upper extremity function and participation in children with neuromotor disorders. The intervention protocol was feasible, but certain aspects of the RCT protocol needed to be revised. Aim 1 of this proposal evaluates the feasibility of these revisions by: 1) establishing an appropriate control condition, 2) evaluating replacement outcome measures for those that were not feasible; and 3) determining effects of a different treatment dosage on retention and attendance. Aim 2 will explore preliminary effects of the intervention on motor function and participation and Aim 3 will explore behavioral and physiological responses. Aim 1: Evaluate the feasibility of the RCT protocol comparing an 8-week PT intervention using hippotherapy and the equine environment with a play-based PT control group targeting motor function and participation for children with NMD. Inestigators will track recruitment, retention, attendance, outcome measure completion, and reasons for absences and withdrawal. Investigators will assess fidelity and acceptability of the treatments for both groups. This will inform design of a large-scale RCT with full statistical power. Investigators hypothesize that they will recruit and randomize 24 participants with \>80% retention, treatment attendance, and outcome measure completion in both groups; and both groups will meet \>80% intervention fidelity and demonstrate acceptability. Aim 2: Identify preliminary effects of an 8-week PT intervention using hippotherapy and the equine environment on motor function and participation compared to play-based PT for children with NMD. Investigators will measure motor function (BOT-3, ABILHANDKIDS), individual goal attainment (GAS), and participation (PEM-CY) before and after the 8-week treatment period for all participants and compare the average change score between the treatment and control groups. Investigators hypothesize that group average scores will improve from pre- to post-treatment for both groups, with a greater change in the treatment group compared to the control group. Aim 3: Compare physiological, behavioral, and emotional responses in children with NMD during PT sessions incorporating equine interaction versus play-based PT. Investigators will calculate changes in high-frequency heart rate variability (HF-HRV) - the gold standard method of measuring parasympathetic nervous system activity - before, during, and after a treatment session. Investigators will measure levels of salivary oxytocin and cortisol before and after a treatment session. Investigators will assess engagement during treatment using behavioral coding and an observational rating scale. Enjoyment will be measured via patient report on a visual-analog scale following treatment. These measures will be collected during 4 treatment sessions (every 2 weeks) throughout the intervention. Investigators hypothesize that the intervention group will demonstrate an immediate increase in HF-HRV and salivary oxytocin, and a decrease in salivary cortisol after a single 60-minute session and sustained over the course of the 8-week intervention; participants will demonstrate sustained high engagement and enjoyment over the course of the intervention, and ratings of engagement and enjoyment will be greater than in the control condition; and physiological changes and behavioral ratings will be significantly positively correlated with the outcomes measured in Aim 2. Background and Rationale There is a growing number of children with NMD across the United States. In rural areas like Montana, access and adherence to PT is especially challenging. Offering PT services in an equine environment can increase access to rural populations and provide PT in setting that might be more motivating and engaging for children, however there is a need to further evaluate its efficacy for improving important outcomes, like motor function and participation. The investigators began to address this need with a previous feasibility RCT which is linked in References. In Aim 1 investigators will implement changes identified to improve feasibility of the RCT protocol: 1) add additional screening to confirm that participants can complete all measures; 2) assess the feasibility of a play-based PT control condition; 3) test the reduced frequency of 2x/ week with a larger sample, and 4) replace the outcome measures that had low completion rates. Results from Aim 1 will inform the design of a large-scale clinical efficacy trial. In Aim 2, investigators will evaluate preliminary efficacy of the PT intervention using hippotherapy and the equine environment. This will provide the justification for a large-scale efficacy trial, and data for a power analysis. PT using hippotherapy and the equine environment involves interaction between the patient and the equine. Emerging research has shown positive physiological and behavioral impacts of human-animal interactions. This PI's pilot study, which is not yet published, is the first known study to evaluate patient behavioral and physiological responses to HEI during a PT session. In Aim 3, investigators will evaluate these responses longitudinally, compare them to a control intervention, and investigate their potential moderation of patient outcomes. Study Design A pilot, randomized-controlled trial with 24 children with NMD who will be assigned to either the PT intervention using hippotherapy and the equine environment or a standard PT control condition. Participants Investigators will recruit 24 children from rehabilitation clinics, schools, and recreational centers around Western Montana. Screening A research assistant will screen interested participants by phone. Those who meet initial inclusion criteria will attend a screening visit to ensure they can safely mount and dismount the equine, can provide a saliva sample, can follow simple verbal instructions, and can ambulate independently. Sample Size The study design was determined using guidance in the NIH document Pilot Studies: Common Uses and Misuses. Investigators concluded that 80%+ power is not necessary for the current proposal because intervention efficacy is not being tested, which would require a larger sample size and length of time than this funding mechanism accommodates. Rather, the sample size is "based on practical considerations including participant flow, budgetary constraints, and the number of participants needed to reasonably evaluate goals."36 A sample size of 24 will allow sufficient participant variability to assess the feasibility of changes made to the RCT protocol and provide insight into preliminary effects of the intervention (see Preliminary Data). The investigators have assembled a strong network of recruitment partners and treatment sites (see Letters of Support) to ensure that completion of study aims with a sample of 24 subjects is achievable within the 12-month timeline. Setting Participant baseline and post-intervention assessments and the control group treatment sessions will take place at the Pediatric Rehabilitation and Active Lifestyle (PALS) Lab at the University of Montana. The PALS lab is a 1000 ft2 space with two private assessment spaces and a waiting area for caregivers and families. The equine intervention will occur at Trotting Horse Therapeutic Riding (THTR) in Missoula, Hobby Horse Place in Polson, and Two Bear Therapeutic Riding Center in Whitefish (see Letters of Support). The facilities have accessible indoor arenas and trained therapy equines and equine professionals. Therapists Therapists for the equine treatment are licensed physical therapists who have the recommended training to incorporate hippotherapy and the equine environment into treatment (American Hippotherapy Association (AHA) Inc. Hippotherapy Treatment Principles Part I and II). Each therapist will work with an equine professional with PATH Intl. Therapeutic Riding Instructor Certification, to ensure that therapists adhere to industry standards for safety and handling of the equines. The standard PT control intervention will be delivered by Doctor of Physical Therapy (DPT) students under the direct supervision of a licensed physical therapist. Outcomes Assessments Pre- and post-outcomes assessments will be completed by DPT students who are trained on standardized application of all measures and under direct supervision of a licensed PT (study PI). The assessors will be blinded to subject group assignments. Measures The following measures will be applied to all participants: Feasibility Fidelity Delivery of intervention Receipt of intervention Reliability: Coders for fidelity will establish ≥85% reliability by coding the same video, comparing codes, discussing any discrepancies, and reaching a consensus on any item where codes were not in agreement. This process will be repeated until the codes are congruent for ≥85% of items for sufficient reliability. Once reliability is established, the remaining videos will be divided amongst coders. Acceptability: The engagement (PRIME-O) and enjoyment (How Happy Scale) measures described below will be used as measures of participant acceptability of the intervention. The following measures will be collected at baseline (week 0) and post-intervention (week 9) for both groups: GMFM-88 ABILHAND-KIDS GAS PEM-CY The following measures will be collected at weeks 2, 4, 6, and 8 for both groups: PRIME-O HF-HRV Salivary Oxytocin Salivary Cortisol Adaptation of How Happy Scale Behavioral Coding Analysis Aim 1: Descriptive statistics wil be used to calculate rates of enrollment, attendance, attrition, fidelity, and outcome measure completion. Reasons for study withdrawal, missed therapy sessions, or incomplete outcome assessments will be documented. Rates will be compared between the treatment and control groups using independent samples t-tests for attendance, attrition, and measure completion and GLMMRM for fidelity. Aim 2: Changes in outcome scores from pre- to post-intervention will be compared between groups using generalized linear mixed models for repeated measures (GLMMRM). Where available, the percent of participants who meet a minimal clinically important difference (MCID) from pre- to post-intervention will be reported. Aim 3: HF-HRV will be calculated for the pre-, during-, and post-session intervals and compare these means. Pre-post session changes in salivary oxytocin and cortisol will be compared. HF-HRV pre-to during-session mean change and oxytocin and cortisol pre- to post-session mean changes between groups will be compared. For the behavioral data, the percentage of scans where behaviors indicate a positive or negative mood will be calculated and the average frequency of each determined and compare between groups. Longitudinal changes will be compared for all variables throughout the intervention. GLMMRM will be used for all comparisons. |
  | Date | 2025 |
  | Language | English |
  | URL | https://www.embase.com/search/results?subaction=viewrecord&id=LNCT06887647&from=export |
  | Publication | clinicaltrials.gov |
  | Journal Abbr | clinicaltrials.gov |
  | Date Added | 11/07/2025, 12:13:15 |
  | Modified | 11/07/2025, 12:13:15 |

  ### Tags:

  - oxytocin
  - human-animal interaction
  - horse
  - heart rate variability
  - emotion
  - child
  - human
  - hydrocortisone
  - controlled study
  - hippotherapy
  - physiotherapy
  - pilot study
  - randomized controlled trial
  - leisure
  - caregiver
  - cerebral palsy
  - certification
  - cholinergic system
  - clinical trial
  - coregonine
  - goal attainment
  - Gross Motor Function Measure
  - group therapy
  - minimal clinically important difference
  - Montana
  - mood
  - motoneuron
  - motor performance
  - neural tube defect
  - outcome assessment
  - participation
  - pediatric rehabilitation
  - physiotherapist
  - power analysis
  - preliminary data
  - rating scale
  - rehabilitation center
  - reliability
  - rural area
  - rural population
  - spinal dysraphism
  - spinal muscular atrophy
  - treatment outcome
  - upper limb
  - videorecording
  - visual analog scale

  ### Attachments

  - Full Text (HTML)
- ## Effects of Intranasal Oxytocin on Trust Towards Therapists and Dogs: A Randomized Controlled Trial in Healthy Adults

  |  |  |
  | --- | --- |
  | Item Type | Journal Article |
  | Abstract | Brief Summary Oxytocin has been proposed as a neuroendocrine mechanism that may mediate the relationship between dog ownership and positive health outcomes and be linked to human-dog interactions and is thought to be a mechanism of interspecies bonding. While the role of oxytocin in human bonding behaviours and social behaviour, in general, is becoming well-established the role of oxytocin in human-animal interaction and Animal-Assisted Interventions (AAI) remains unclear. This research gap calls for more high-quality research investigating this possible neuroendocrine underlying mechanism to advance knowledge about AAI. If oxytocin indeed might be involved in interspecies bonding, intranasally administered oxytocin should not only enhance trust toward a human but also towards a dog. |
  | Date | 2024 |
  | Language | English |
  | URL | https://www.embase.com/search/results?subaction=viewrecord&id=LNCT06248710&from=export |
  | Publication | clinicaltrials.gov |
  | Journal Abbr | clinicaltrials.gov |
  | Date Added | 11/07/2025, 12:13:17 |
  | Modified | 11/07/2025, 12:13:17 |

  ### Tags:

  - oxytocin
  - human-animal interaction
  - dog
  - social behavior
  - human
  - controlled study
  - randomized controlled trial
  - human experiment
  - normal human
  - clinical trial
  - depression
  - nose spray

  ### Attachments

  - Full Text (HTML)
- ## Animal Assisted Intervention for Hemodialysis Outpatients: A Mixed-method Randomized Controlled Trial for Treatment Adherence and Psychosocial Well-being

  |  |  |
  | --- | --- |
  | Item Type | Journal Article |
  | Abstract | Brief Summary The goal of this clinical trial is to understand if and how an animal-assisted intervention \[AAI\] using therapy dogs can support hemodialysis \[HD\] patients' treatment adherence and enhance their well-being. The main objectives are: \* Objective 1: Determine if the AAI impacts patients' HD treatment adherence (primary outcome is number of unplanned missed treatments no due to hospitalization). \* Objective 2: Evaluate if the AAI impacts patients' psychosocial well-being (secondary outcomes are stress, pain, mood, QOL). \* Objective 3: Examine potential mechanistic biomarkers that underpin human-animal bonding (hormones tied to stress and bonding). (exploratory aim) \* Objective 4: Understand patients' subjective experiences of the AAI. Participants will be asked to engage in several research tasks, including: \* assessments \* therapy dog visits \* monthly blood draws \* focus group Researchers will compare how the treatment group (those who receive 2 dogs visits per week) and the control group (those who receive 0 dog visits per week) to see if the AAI impacts treatment adherence and psychosocial well-being. Detailed Description This study will take place in one outpatient dialysis clinic. The clinical trial will compare a standardized therapy dog interaction delivered twice weekly (based on pilot study outcomes detailed below) to usual care (e.g., no dog exposure) using a 1:1 randomized, 2-arm design. Subjective patient-reported outcomes \[PRO\] and routinely-tracked clinic data (e.g., missed visits) will be used. A total of 30 patients will be recruited then randomized 1:1 into 2 arms: control group with 0 dog visits (n = 15) and intervention group with 2 dog visits per week for 20 weeks (n = 15). The AAI is designed to promote patient comfort, uplift mood, and provide an opportunity for socialization. The nature of the dog interaction involves several different components, including but not limited to: petting the dog, talking to the dog, watching the dog do tricks, conversing with the dog handler, and being prompted to discuss any fond memories/stories of their own personal experiences with human-animal interactions. Regarding duration, the dog visits average 10 minutes but will be allowed to vary, and length of dog visits will be tracked. Each team (dog handler + dog) will have an assigned unique ID so that variation in dog is controlled for. This study will utilize trained certified dog handlers to deliver the intervention from reputable local and national pet therapy organizations. All dogs have gone through extensive training and behavioral assessment, will provide proper documentation, liability insurance, and vaccinations. All study procedures take place in the clinic waiting room. Patient-reported outcomes (PRO)s will be collected as pre-post data (before and after dog visit or lobby-as-usual control condition). All PROs will be collected electronically on iPads using REDcap. Regarding treatment adherence data, the clinic routinely tracks missed appointments and will report these metrics directly to research team. Patients will complete assessments 2 times per week for a total of 20 weeks. A short demographic questionnaire will be administered once at the first study visit. Also, patients will also undergo a monthly blood-draw providing 3mL of blood 1 time a month at the same time as their standard of care blood draw. This will be bio-banked in a repository for later ELISA analyses, which will focus on hormones related to stress and bonding (e.g., oxytocin, cortisol); this may provide insight into mechanistic biomarkers underpinning the human-animal bonding process. Lastly, qualitative focus groups with intervention group participants will be conducted post-trial to learn about their subjective experiences of the AAI. |
  | Date | 2023 |
  | Language | English |
  | URL | https://www.embase.com/search/results?subaction=viewrecord&id=LNCT06030050&from=export |
  | Publication | clinicaltrials.gov |
  | Journal Abbr | clinicaltrials.gov |
  | Date Added | 11/07/2025, 12:13:18 |
  | Modified | 11/07/2025, 12:13:18 |

  ### Tags:

  - oxytocin
  - human-animal interaction
  - dog
  - therapy
  - female
  - male
  - human
  - hydrocortisone
  - adult
  - controlled study
  - ELISA kit
  - pilot study
  - randomized controlled trial
  - tablet computer
  - hospitalization
  - quality of life
  - questionnaire
  - major clinical study
  - patient compliance
  - clinical trial
  - mood
  - pain
  - human-animal bond
  - enzyme linked immunosorbent assay
  - biological marker
  - social well-being
  - pet therapy
  - benchmarking
  - chronic kidney failure
  - chronic pain
  - end stage renal disease
  - hemodialysis
  - liability insurance
  - lobbying
  - outpatient
  - patient comfort
  - patient-reported outcome
  - personal experience
  - socialization
  - vaccination
  - waiting room

  ### Attachments

  - Full Text (HTML)
- ## A Pilot Study of the Effects of Interactions With Therapy Dogs on Child Stress Responsivity

  |  |  |
  | --- | --- |
  | Item Type | Journal Article |
  | Abstract | Brief Summary The objective of this study is to apply a rigorous experimental design to test whether children's interactions with therapy dogs increase immediate prosocial behavior and reduce immediate biological response to stress. Detailed Description The central goal of the study is to determine whether brief interactions with a therapy dog have an immediate impact on children's biological response to stress, prosocial behaviors, and self-reported mood in comparison to interactions with a stuffed toy dog. The study uses a randomized crossover design with two study arms; all children will receive the both interventions during the same session, with the timing of the intervention randomized across subject. All outcomes will be assessed during a single study visit. No follow-up data will be collected. The study uses both between-group and within-subject comparisons. Between groups, the investigators predict that children who interact with a therapy dog prior to a psychosocial stress task (Arm 1) will show attenuated cortisol response to the stress task (primary outcome) and reduced physiological stress (secondary outcomes) compared to children who interact with a stuffed toy dog prior to the psychosocial stress task (Arm 2). As an additional control, children will also watch a 5 minute puppy video prior to the prosocial stress task and will then interact with a stuffed toy dog prior to the psychosocial stress task (Arm 3). Conversely, children who interact with the therapy dog immediately prior to the in-lab behavior tasks (Arm 2) will show higher levels of behavioral carefulness and prosocial behavior (primary outcomes) compared to children who interact with the stuffed toy dog prior to the behavior tasks (Arm 1). Within subjects across both study arms, increases in positive mood and decreases in negative mood (secondary outcomes) will be greatest following interaction with the therapy dog compared to the stuffed toy dog, after controlling for main effects of study arm. Within subjects, physiological markers of stress (secondary outcomes) will be lower during the interaction with the therapy dog than during interaction with the stuffed toy dog. Investigators will seek additional funds to collect and analyze salivary oxytocin data. The hypothesis is that children will show greater increase in oxytocin following interaction with the therapy dog in comparison to interaction with the stuffed toy dog. This study will also investigate the mechanisms through which child-dog interactions influence youth stress responsivity, using coded videotaped data from the subset of children in Arm 1 who interact with the therapy dog prior to the psychosocial stress task. It is hypothesized that child behaviors observed during the interaction, such as duration and frequency of eye gaze, petting and stroking behaviors, and use of positive affect, will be inversely correlated with change in cortisol response to stress. Dog behaviors, such as duration and frequency of eye gaze and approach behaviors, will be inversely correlated with children's change in cortisol response to stress. The study will also investigate whether child characteristics moderate the effects of the child-dog interaction. Investigators hypothesize that the effects of the therapy dog intervention will be stronger among children who currently live with dogs versus non-dog owning children and among children with more positive attitudes towards pets. It is also expected that the effects of the therapy dog interaction will be weaker among children with internalizing problems and for children experiencing higher levels of general stress. Investigators will also test whether the effects of the therapy dog intervention vary across child gender, race/ethnicity, or socioeconomic status, or child personality. |
  | Date | 2019 |
  | Language | English |
  | URL | https://www.embase.com/search/results?subaction=viewrecord&id=LNCT03949569&from=export |
  | Publication | clinicaltrials.gov |
  | Journal Abbr | clinicaltrials.gov |
  | Date Added | 11/07/2025, 12:13:30 |
  | Modified | 11/07/2025, 12:13:30 |

  ### Tags:

  - oxytocin
  - human-animal interaction
  - dog
  - saliva
  - therapy
  - social behavior
  - child
  - female
  - male
  - physiological stress
  - human
  - hydrocortisone
  - controlled study
  - follow up
  - pilot study
  - randomized controlled trial
  - crossover procedure
  - therapy animal
  - major clinical study
  - mental stress
  - clinical trial
  - mood
  - videorecording
  - personality
  - puppy
  - social status
  - child behavior
  - duration
  - ethnicity
  - gaze
  - internalizing disorder
  - prosocial behavior

  ### Attachments

  - Full Text (HTML)
- ## Oxytocin Pathways and the Health Effects of Human-Animal Interaction

  |  |  |
  | --- | --- |
  | Item Type | Journal Article |
  | Abstract | Brief Summary This study investigates the roles of oxytocin pathways in human-animal interaction. Detailed Description This study investigates the roles of oxytocin pathways in human-animal interaction. Children will participate in three conditions involving friendly interactions with dogs, or play with toys at a university laboratory. Child and dog saliva and urine will be assayed for oxytocin concentrations. We will assess variation in oxytocin concentrations in relation to different experimental conditions, and in relation to specific behaviors and psychological constructs. |
  | Date | 2019 |
  | Language | English |
  | URL | https://www.embase.com/search/results?subaction=viewrecord&id=LNCT03852264&from=export |
  | Publication | clinicaltrials.gov |
  | Journal Abbr | clinicaltrials.gov |
  | Date Added | 11/07/2025, 12:13:31 |
  | Modified | 11/07/2025, 12:13:31 |

  ### Tags:

  - oxytocin
  - human-animal interaction
  - dog
  - saliva
  - child
  - male
  - nonhuman
  - hydrocortisone
  - controlled study
  - clinical trial
  - human-animal bond

  ### Attachments

  - Full Text (HTML)
- ## Are Hair Cortisol Levels of Humans, Cats, and Dogs from the Same Household Correlated?

  |  |  |
  | --- | --- |
  | Item Type | Journal Article |
  | Author | J. Wojtaś |
  | Author | A. Garbiec |
  | Author | M. Karpiński |
  | Author | P. Skowronek |
  | Author | A. Strachecka |
  | Abstract | Human–animal interactions and the emotional relationship of the owner with the pet are the subjects of many scientific studies and the constant interest of not only scientists but also pet owners. The aim of this study was to determine and compare the hair cortisol levels of dogs, cats, and their owners living in the same household. The owners were asked to complete a questionnaire concerning the frequency of their interactions with pets and emotional relationship with each of their cats and each of their dogs. The study involved 25 women who owned at least one dog and at least one cat. In total, 45 dogs and 55 cats from 25 households participated in the study. The average level of hair cortisol of the owners was 4.62 ng/mL, of the dogs 0.26 ng/mL, and in the hair of cats 0.45 ng/mL. There was no significant correlation between the hair cortisol level of the owner and dog or the owner and the cat and between dogs and cats living together. A significant positive correlation was observed between the hair cortisol level in the owner and the pet, for dogs in which the owner performs grooming treatments once a week and for cats which are never kissed. Although our study did not find many significant correlations, studies using other stress markers might have yielded different results. |
  | Date | 2022 |
  | Language | English |
  | Archive | Embase |
  | URL | https://www.embase.com/search/results?subaction=viewrecord&id=L2016999160&from=export |
  | Volume | 12 |
  | Publication | Animals |
  | DOI | 10.3390/ani12111472 |
  | Issue | 11 |
  | Journal Abbr | Animals |
  | ISSN | 2076-2615 |
  | Date Added | 11/07/2025, 14:16:48 |
  | Modified | 11/07/2025, 14:16:48 |

  ### Tags:

  - article
  - female
  - male
  - human
  - hydrocortisone
  - adult
  - controlled study
  - questionnaire
  - major clinical study
  - enzyme linked immunosorbent assay
  - hydrocortisone blood level
  - tube
  - cross-sectional study
  - hair
  - methanol
  - incubation time
  - needlestick injury
  - phosphate buffered saline
  - social network
  - specimen container

  ### Attachments

  - Full Text (HTML)
- ## Are Hair Cortisol Levels of Humans, Cats, and Dogs from the Same Household Correlated?

  |  |  |
  | --- | --- |
  | Item Type | Journal Article |
  | Author | J. Wojtaś |
  | Author | A. Garbiec |
  | Author | M. Karpiński |
  | Author | P. Skowronek |
  | Author | A. Strachecka |
  | Abstract | Human–animal interactions and the emotional relationship of the owner with the pet are the subjects of many scientific studies and the constant interest of not only scientists but also pet owners. The aim of this study was to determine and compare the hair cortisol levels of dogs, cats, and their owners living in the same household. The owners were asked to complete a questionnaire concerning the frequency of their interactions with pets and emotional relationship with each of their cats and each of their dogs. The study involved 25 women who owned at least one dog and at least one cat. In total, 45 dogs and 55 cats from 25 households participated in the study. The average level of hair cortisol of the owners was 4.62 ng/mL, of the dogs 0.26 ng/mL, and in the hair of cats 0.45 ng/mL. There was no significant correlation between the hair cortisol level of the owner and dog or the owner and the cat and between dogs and cats living together. A significant positive correlation was observed between the hair cortisol level in the owner and the pet, for dogs in which the owner performs grooming treatments once a week and for cats which are never kissed. Although our study did not find many significant correlations, studies using other stress markers might have yielded different results. |
  | Date | 2022 |
  | Language | English |
  | Archive | Embase |
  | URL | https://www.embase.com/search/results?subaction=viewrecord&id=L2016999160&from=export |
  | Volume | 12 |
  | Publication | Animals |
  | DOI | 10.3390/ani12111472 |
  | Issue | 11 |
  | Journal Abbr | Animals |
  | ISSN | 2076-2615 |
  | Date Added | 11/07/2025, 14:18:21 |
  | Modified | 11/07/2025, 14:18:21 |

  ### Tags:

  - article
  - female
  - male
  - human
  - hydrocortisone
  - adult
  - controlled study
  - questionnaire
  - major clinical study
  - enzyme linked immunosorbent assay
  - hydrocortisone blood level
  - tube
  - cross-sectional study
  - hair
  - methanol
  - incubation time
  - needlestick injury
  - phosphate buffered saline
  - social network
  - specimen container

  ### Attachments

  - Full Text (HTML)
- ## Animals in Animal-Assisted Services: Are They Volunteers or Professionals?

  |  |  |
  | --- | --- |
  | Item Type | Journal Article |
  | Author | B. Wijnen |
  | Author | P. Martens |
  | Abstract | With the increasingly common practice of Animal-Assisted Services (AAS), whether in therapy, coaching, education, or volunteering programs, the concern over animal welfare has also risen. However, no standards have yet been established for good practices to ensure the animal’s mental health. This is largely due to the wide variety of roles played by animals in interventions and the lack of ‘job descriptions’ for the animal in diverse settings. Some professionals call their animal a ‘volunteer’, others mention that some directive guidance is given to the therapy animal, and some assistance animals are highly trained. Misunderstandings could be avoided if the integrated animal were to receive a justifiable label: volunteer or professional. Choosing either one comes with obligations for the owner, handler, or therapist. In this paper, we compare the roles of human volunteers and professionals to the roles of animals involved in therapy. We also demonstrate the obligations that come along with the decision to label animals as such either volunteers or professionals. Furthermore, we make a plea for animal-friendly interventions, whether in a volunteer position or as a professional, in order to stimulate the animal’s cooperation and motivation. Studying dopamine and translating the findings into context-ethograms can provide a way to judge behavior more objectively. |
  | Date | 2022 |
  | Language | English |
  | Archive | Embase |
  | URL | https://www.embase.com/search/results?subaction=viewrecord&id=L2019524800&from=export |
  | Volume | 12 |
  | Publication | Animals |
  | DOI | 10.3390/ani12192564 |
  | Issue | 19 |
  | Journal Abbr | Animals |
  | ISSN | 2076-2615 |
  | Date Added | 11/07/2025, 11:49:33 |
  | Modified | 11/07/2025, 11:49:33 |

  ### Tags:

  - human-animal interaction
  - animal welfare
  - dog
  - emotion
  - nonhuman
  - social interaction
  - electroencephalogram
  - animal well-being
  - compensation
  - dopamine
  - elephant
  - facial expression
  - health care personnel
  - mindfulness-based stress reduction
  - motivation
  - neurotransmitter
  - note
  - quality of life
  - questionnaire
  - therapy animal
  - volunteer

  ### Attachments

  - Full Text (HTML)
- ## Assessing the Visitor and Animal Outcomes of a Zoo Encounter and Guided Tour Program with Ambassador Cheetahs

  |  |  |
  | --- | --- |
  | Item Type | Journal Article |
  | Author | K.M. Whitehouse-Tedd |
  | Author | J. Lozano-Martinez |
  | Author | J. Reeves |
  | Author | M. Page |
  | Author | J.H. Martin |
  | Author | H. Prozesky |
  | Abstract | Research into the effectiveness of zoo Ambassador Animal Programs (AAPs) has typically investigated human or animal factors separately. This study took a multi-dimensional approach and aimed primarily to (1) determine whether change in visitor knowledge was influenced by the type of experience undertaken and (2) assess welfare indicators in ambassador cheetahs (Acinonyx jubatus) during encounter and non-encounter periods at a South African zoological facility. The AAP was evaluated for its visitor impact using a repeated-measures study design, whereby visitor agreement with six statements (3 being correct and 3 being incorrect) were assessed pre- and post-visit; change in responses was then compared according to the type of visit (encounter, guided tour, or combination; n = 182). Behavioral indicators (activity budget, pacing, lying with head down, and behaviors indicative of positive [purring] or negative [tail-flicking] affective states) were measured as well as a physiological (heart rate) indicator of animal welfare for cheetahs (n = 5). The expression of these indicators was compared according to whether the cheetahs were on display (with and without visitor presence at the fence) and involved in an encounter (with and without physical interaction, that is, being stroked). A positive knowledge change was recorded in 62% of visitors, regardless of experience type, whilst 11% and 27% exhibited no change or reduced knowledge score, respectively. Visitors involved in encounters had a significantly lower (but still positive) knowledge change compared with other visit types. No difference in cheetah behavior was detected for interaction and non-interaction periods. However, cheetahs spent greater periods of time lying with their head down whilst being stroked, and the mean heart rate was significantly lower during these interactions. These findings indicate that animal–visitor encounters alone are unlikely to promote conservation-education, but the addition of a guided tour was important in facilitating increased conservation-education. Whilst the welfare of animal ambassadors was not compromised by visitor encounters, negative changes in knowledge scores for some visitors suggest areas for improvement exist. |
  | Date | 2022 |
  | Language | English |
  | Archive | Embase |
  | URL | https://www.embase.com/search/results?subaction=viewrecord&id=L2014057162&from=export |
  | Volume | 35 |
  | Pages | 307-322 |
  | Publication | Anthrozoos |
  | DOI | 10.1080/08927936.2021.1986263 |
  | Issue | 2 |
  | Journal Abbr | Anthrozoos |
  | ISSN | 1753-0377 |
  | Date Added | 11/07/2025, 12:10:48 |
  | Modified | 11/07/2025, 12:10:48 |

  ### Tags:

  - human-animal interaction
  - animal welfare
  - heart rate
  - article
  - nonhuman
  - human
  - controlled study
  - human experiment
  - major clinical study
  - outcome assessment
  - budget
  - cheetah
  - education

  ### Attachments

  - Full Text (HTML)
- ## The blockade of oxytocin receptors in the paraventricular thalamus reduces maternal crouching behavior over pups in lactating mice

  |  |  |
  | --- | --- |
  | Item Type | Journal Article |
  | Author | A. Watarai |
  | Author | S. Tsutaki |
  | Author | K. Nishimori |
  | Author | T. Okuyama |
  | Author | K. Mogi |
  | Author | T. Kikusui |
  | Abstract | Oxytocin (OT) systems contribute to the elicitation of stereotypic maternal behaviors. OT peptide-expressing neurons are predominantly localized in the hypothalamus, whereas OT receptor (OTR)-expressing neurons are widely distributed throughout the brain. Among those OTR-expressing regions, the paraventricular thalamus (PVT) consists of heterogeneous neuropeptide-responsive neurons critical for appetitive motivation, food intake control, and social behaviors; however, the precise distribution of OTR-expressing neurons within the PVT and whether these neurons are involved in maternal behaviors in mice are unknown. The distribution of OTR-expressing neurons was examined in an OTR-Venus transgenic line expressing a fluorescent protein controlled by the OTR promoter. The number of Venus expressing neurons was higher in the posterior PVT (pPVT) than in the anterior PVT (aPVT). When OTR-Venus dams were exposed to pups, the number of double-labelled neurons expressing both OTR-Venus and a marker of neuronal activity (c-Fos) was increased in the pPVT compared to non-exposed dams, while the aPVT remained unchanged. To investigate whether OT signaling in the pPVT is essential for maternal behaviors, an OT antagonist (OTA) was transiently or chronically infused into the pPVT of lactating dams during the postpartum period. Although the transient OTR blockade did not affect maternal behaviors, a chronic OTR blockade specifically reduced the duration of crouching behavior over pups. Taken together, these findings suggest that OTR-expressing neurons in the pPVT are involved in maternal crouching behavior. |
  | Date | 2020 |
  | Language | English |
  | Archive | Embase |
  | URL | https://www.embase.com/search/results?subaction=viewrecord&id=L2004708618&from=export |
  | Volume | 720 |
  | Publication | Neuroscience Letters |
  | DOI | 10.1016/j.neulet.2020.134761 |
  | Issue | (Watarai A.; Tsutaki S.; Mogi K., mogik@azabu-u.ac.jp) Laboratory of Companion Animal Research, School of Veterinary Medicine, Azabu University, Sagamihara, Japan |
  | Journal Abbr | Neurosci. Lett. |
  | ISSN | 1872-7972 |
  | Date Added | 11/07/2025, 12:13:29 |
  | Modified | 11/07/2025, 12:13:29 |

  ### Tags:

  - oxytocin
  - social behavior
  - animal experiment
  - article
  - female
  - nonhuman
  - controlled study
  - nerve cell
  - nerve cell plasticity
  - lactation
  - tactile stimulation
  - animal tissue
  - maternal behavior
  - priority journal
  - oxytocin receptor
  - neuropeptide
  - bregma
  - gluteus maximus muscle
  - histology
  - mouse
  - nucleus accumbens
  - paraventricular thalamic nucleus
  - promoter region
  - protein c fos
  - puerperium
  - total distance traveled

  ### Attachments

  - Full Text (HTML)
- ## Cat Foster Program Outcomes: Behavior, Stress, and Cat–Human Interaction

  |  |  |
  | --- | --- |
  | Item Type | Journal Article |
  | Author | K.R. Vitale |
  | Author | D.H. Frank |
  | Author | J. Conroy |
  | Author | M.A.R. Udell |
  | Abstract | Recent research has demonstrated that cats (Felis catus) have greater social potential and flexibility than was previously assumed. However, many traditional cat care practices have been influenced by the misconception that cats are socially aloof. This can result in less support or guidance for cat-focused programs that may promote improved success or welfare. For example, while dog fostering programs—even overnight programs—are considered highly beneficial, with research to back these claims, relatively little research has been dedicated to understanding the potential risks and benefits of cat fostering programs. Therefore, the aim of this study was to empirically evaluate the social, behavioral, and stress response outcomes associated with placing shelter cats in an overnight or short-term foster environment. While neither overnight nor 1-week fostering lead to a statistically significant improvement in human-directed social behavior or stress levels, foster cats also did not display increased fear or aggression in the foster home and did not have higher cortisol levels. Therefore, cat fostering—even short-term fostering—does not appear to be more stressful or problematic for this species than remaining in a shelter. This information could contribute to life-saving efforts by providing empirical evidence that cats can be safely moved into foster homes, even for short durations, when shelter space is limited. More research is needed to evaluate the potential effects of longer-term fostering in cats, as well as cat fostering practices that could lead to greater welfare benefits. |
  | Date | 2022 |
  | Language | English |
  | Archive | Embase |
  | URL | https://www.embase.com/search/results?subaction=viewrecord&id=L2019003409&from=export |
  | Volume | 12 |
  | Publication | Animals |
  | DOI | 10.3390/ani12172166 |
  | Issue | 17 |
  | Journal Abbr | Animals |
  | ISSN | 2076-2615 |
  | Date Added | 11/07/2025, 14:15:37 |
  | Modified | 11/07/2025, 14:15:37 |

  ### Tags:

  - welfare
  - human-animal interaction
  - behavior
  - physical activity
  - social behavior
  - petting
  - animal experiment
  - article
  - nonhuman
  - physiological stress
  - social interaction
  - hydrocortisone
  - controlled study
  - scoring system
  - foster care
  - chemiluminescence immunoassay
  - creatinine
  - body position
  - caregiver
  - fear
  - domestic cat
  - feeding
  - cat
  - sleep
  - aggression
  - affection
  - body rub
  - chin rub
  - cuddling
  - ear
  - environment
  - experiment
  - experimental social behavior test
  - feline ality test
  - hanging
  - hiding
  - Immulite 1000
  - immunoassay analyzer
  - licking
  - mydriasis
  - picking up
  - playing
  - scratching
  - snuggling
  - talking
  - tooth brushing
  - watching cat

  ### Attachments

  - Full Text (HTML)
- ## Cat Foster Program Outcomes: Behavior, Stress, and Cat–Human Interaction

  |  |  |
  | --- | --- |
  | Item Type | Journal Article |
  | Author | K.R. Vitale |
  | Author | D.H. Frank |
  | Author | J. Conroy |
  | Author | M.A.R. Udell |
  | Abstract | Recent research has demonstrated that cats (Felis catus) have greater social potential and flexibility than was previously assumed. However, many traditional cat care practices have been influenced by the misconception that cats are socially aloof. This can result in less support or guidance for cat-focused programs that may promote improved success or welfare. For example, while dog fostering programs—even overnight programs—are considered highly beneficial, with research to back these claims, relatively little research has been dedicated to understanding the potential risks and benefits of cat fostering programs. Therefore, the aim of this study was to empirically evaluate the social, behavioral, and stress response outcomes associated with placing shelter cats in an overnight or short-term foster environment. While neither overnight nor 1-week fostering lead to a statistically significant improvement in human-directed social behavior or stress levels, foster cats also did not display increased fear or aggression in the foster home and did not have higher cortisol levels. Therefore, cat fostering—even short-term fostering—does not appear to be more stressful or problematic for this species than remaining in a shelter. This information could contribute to life-saving efforts by providing empirical evidence that cats can be safely moved into foster homes, even for short durations, when shelter space is limited. More research is needed to evaluate the potential effects of longer-term fostering in cats, as well as cat fostering practices that could lead to greater welfare benefits. |
  | Date | 2022 |
  | Language | English |
  | Archive | Embase |
  | URL | https://www.embase.com/search/results?subaction=viewrecord&id=L2019003409&from=export |
  | Volume | 12 |
  | Publication | Animals |
  | DOI | 10.3390/ani12172166 |
  | Issue | 17 |
  | Journal Abbr | Animals |
  | ISSN | 2076-2615 |
  | Date Added | 11/07/2025, 14:16:47 |
  | Modified | 11/07/2025, 14:16:47 |

  ### Tags:

  - welfare
  - human-animal interaction
  - behavior
  - physical activity
  - social behavior
  - petting
  - animal experiment
  - article
  - nonhuman
  - physiological stress
  - social interaction
  - hydrocortisone
  - controlled study
  - scoring system
  - foster care
  - chemiluminescence immunoassay
  - creatinine
  - body position
  - caregiver
  - fear
  - domestic cat
  - feeding
  - cat
  - sleep
  - aggression
  - affection
  - body rub
  - chin rub
  - cuddling
  - ear
  - environment
  - experiment
  - experimental social behavior test
  - feline ality test
  - hanging
  - hiding
  - Immulite 1000
  - immunoassay analyzer
  - licking
  - mydriasis
  - picking up
  - playing
  - scratching
  - snuggling
  - talking
  - tooth brushing
  - watching cat

  ### Attachments

  - Full Text (HTML)
- ## Cat Foster Program Outcomes: Behavior, Stress, and Cat–Human Interaction

  |  |  |
  | --- | --- |
  | Item Type | Journal Article |
  | Author | K.R. Vitale |
  | Author | D.H. Frank |
  | Author | J. Conroy |
  | Author | M.A.R. Udell |
  | Abstract | Recent research has demonstrated that cats (Felis catus) have greater social potential and flexibility than was previously assumed. However, many traditional cat care practices have been influenced by the misconception that cats are socially aloof. This can result in less support or guidance for cat-focused programs that may promote improved success or welfare. For example, while dog fostering programs—even overnight programs—are considered highly beneficial, with research to back these claims, relatively little research has been dedicated to understanding the potential risks and benefits of cat fostering programs. Therefore, the aim of this study was to empirically evaluate the social, behavioral, and stress response outcomes associated with placing shelter cats in an overnight or short-term foster environment. While neither overnight nor 1-week fostering lead to a statistically significant improvement in human-directed social behavior or stress levels, foster cats also did not display increased fear or aggression in the foster home and did not have higher cortisol levels. Therefore, cat fostering—even short-term fostering—does not appear to be more stressful or problematic for this species than remaining in a shelter. This information could contribute to life-saving efforts by providing empirical evidence that cats can be safely moved into foster homes, even for short durations, when shelter space is limited. More research is needed to evaluate the potential effects of longer-term fostering in cats, as well as cat fostering practices that could lead to greater welfare benefits. |
  | Date | 2022 |
  | Language | English |
  | Archive | Embase |
  | URL | https://www.embase.com/search/results?subaction=viewrecord&id=L2019003409&from=export |
  | Volume | 12 |
  | Publication | Animals |
  | DOI | 10.3390/ani12172166 |
  | Issue | 17 |
  | Journal Abbr | Animals |
  | ISSN | 2076-2615 |
  | Date Added | 11/07/2025, 14:18:20 |
  | Modified | 11/07/2025, 14:18:20 |

  ### Tags:

  - welfare
  - human-animal interaction
  - behavior
  - physical activity
  - social behavior
  - petting
  - animal experiment
  - article
  - nonhuman
  - physiological stress
  - social interaction
  - hydrocortisone
  - controlled study
  - scoring system
  - foster care
  - chemiluminescence immunoassay
  - creatinine
  - body position
  - caregiver
  - fear
  - domestic cat
  - feeding
  - cat
  - sleep
  - aggression
  - affection
  - body rub
  - chin rub
  - cuddling
  - ear
  - environment
  - experiment
  - experimental social behavior test
  - feline ality test
  - hanging
  - hiding
  - Immulite 1000
  - immunoassay analyzer
  - licking
  - mydriasis
  - picking up
  - playing
  - scratching
  - snuggling
  - talking
  - tooth brushing
  - watching cat

  ### Attachments

  - Full Text (HTML)
- ## Neurobiology of Loneliness, Isolation, and Loss: Integrating Human and Animal Perspectives

  |  |  |
  | --- | --- |
  | Item Type | Journal Article |
  | Author | E.M. Vitale |
  | Author | A.S. Smith |
  | Abstract | In social species such as humans, non-human primates, and even many rodent species, social interaction and the maintenance of social bonds are necessary for mental and physical health and wellbeing. In humans, perceived isolation, or loneliness, is not only characterized by physical isolation from peers or loved ones, but also involves negative perceptions about social interactions and connectedness that reinforce the feelings of isolation and anxiety. As a complex behavioral state, it is no surprise that loneliness and isolation are associated with dysfunction within the ventral striatum and the limbic system – brain regions that regulate motivation and stress responsiveness, respectively. Accompanying these neural changes are physiological symptoms such as increased plasma and urinary cortisol levels and an increase in stress responsivity. Although studies using animal models are not perfectly analogous to the uniquely human state of loneliness, studies on the effects of social isolation in animals have observed similar physiological symptoms such as increased corticosterone, the rodent analog to human cortisol, and also display altered motivation, increased stress responsiveness, and dysregulation of the mesocortical dopamine and limbic systems. This review will discuss behavioral and neuropsychological components of loneliness in humans, social isolation in rodent models, and the neurochemical regulators of these behavioral phenotypes with a neuroanatomical focus on the corticostriatal and limbic systems. We will also discuss social loss as a unique form of social isolation, and the consequences of bond disruption on stress-related behavior and neurophysiology. |
  | Date | 2022 |
  | Language | English |
  | Archive | Embase |
  | URL | https://www.embase.com/search/results?subaction=viewrecord&id=L2015843355&from=export |
  | Volume | 16 |
  | Publication | Frontiers in Behavioral Neuroscience |
  | DOI | 10.3389/fnbeh.2022.846315 |
  | Issue | (Vitale E.M., evitale@ku.edu; Smith A.S., adamsmith@ku.edu) Department of Pharmacology and Toxicology, School of Pharmacy, University of Kansas, Lawrence, KS, United States |
  | Journal Abbr | Front. Behav. Neurosci. |
  | ISSN | 1662-5153 |
  | Date Added | 11/07/2025, 12:12:21 |
  | Modified | 11/07/2025, 12:12:21 |

  ### Tags:

  - human-animal interaction
  - nonhuman
  - physiological stress
  - human
  - social isolation
  - electroencephalogram
  - emotional attachment
  - review
  - reward
  - neurobiology
  - neurophysiology
  - oxytocin receptor
  - antisocial personality disorder
  - anxiety disorder
  - bereavement
  - biological model
  - brain region
  - corticotropin releasing factor
  - dopamine receptor
  - grief
  - limbic system
  - loneliness
  - neuroanatomy
  - neurochemistry
  - neuropsychology
  - neurotransmitter receptor
  - opiate receptor
  - social connectedness
  - social loss
  - striate cortex
  - tachykinin receptor

  ### Attachments

  - Full Text (HTML)
- ## Neurobiology of Loneliness, Isolation, and Loss: Integrating Human and Animal Perspectives

  |  |  |
  | --- | --- |
  | Item Type | Journal Article |
  | Author | E.M. Vitale |
  | Author | A.S. Smith |
  | Abstract | In social species such as humans, non-human primates, and even many rodent species, social interaction and the maintenance of social bonds are necessary for mental and physical health and wellbeing. In humans, perceived isolation, or loneliness, is not only characterized by physical isolation from peers or loved ones, but also involves negative perceptions about social interactions and connectedness that reinforce the feelings of isolation and anxiety. As a complex behavioral state, it is no surprise that loneliness and isolation are associated with dysfunction within the ventral striatum and the limbic system – brain regions that regulate motivation and stress responsiveness, respectively. Accompanying these neural changes are physiological symptoms such as increased plasma and urinary cortisol levels and an increase in stress responsivity. Although studies using animal models are not perfectly analogous to the uniquely human state of loneliness, studies on the effects of social isolation in animals have observed similar physiological symptoms such as increased corticosterone, the rodent analog to human cortisol, and also display altered motivation, increased stress responsiveness, and dysregulation of the mesocortical dopamine and limbic systems. This review will discuss behavioral and neuropsychological components of loneliness in humans, social isolation in rodent models, and the neurochemical regulators of these behavioral phenotypes with a neuroanatomical focus on the corticostriatal and limbic systems. We will also discuss social loss as a unique form of social isolation, and the consequences of bond disruption on stress-related behavior and neurophysiology. |
  | Date | 2022 |
  | Language | English |
  | Archive | Embase |
  | URL | https://www.embase.com/search/results?subaction=viewrecord&id=L2015843355&from=export |
  | Volume | 16 |
  | Publication | Frontiers in Behavioral Neuroscience |
  | DOI | 10.3389/fnbeh.2022.846315 |
  | Issue | (Vitale E.M., evitale@ku.edu; Smith A.S., adamsmith@ku.edu) Department of Pharmacology and Toxicology, School of Pharmacy, University of Kansas, Lawrence, KS, United States |
  | Journal Abbr | Front. Behav. Neurosci. |
  | ISSN | 1662-5153 |
  | Date Added | 11/07/2025, 12:13:23 |
  | Modified | 11/07/2025, 12:13:23 |

  ### Tags:

  - human-animal interaction
  - nonhuman
  - physiological stress
  - human
  - social isolation
  - electroencephalogram
  - emotional attachment
  - review
  - reward
  - neurobiology
  - neurophysiology
  - oxytocin receptor
  - antisocial personality disorder
  - anxiety disorder
  - bereavement
  - biological model
  - brain region
  - corticotropin releasing factor
  - dopamine receptor
  - grief
  - limbic system
  - loneliness
  - neuroanatomy
  - neurochemistry
  - neuropsychology
  - neurotransmitter receptor
  - opiate receptor
  - social connectedness
  - social loss
  - striate cortex
  - tachykinin receptor

  ### Attachments

  - Full Text (HTML)
- ## Effects of Essential Animal Visitation Program (AVP) Components on Students’ Salivary α-Amylase and Amylase-to-Cortisol Ratios

  |  |  |
  | --- | --- |
  | Item Type | Journal Article |
  | Author | J.L. Vandagriff |
  | Author | A.M. Carr |
  | Author | S.M. Roeter Smith |
  | Author | P. Pendry |
  | Abstract | While efficacy trials suggest that Animal Visitation Programs (AVPs) relieve university student stress, their essential components are unknown. Students were randomly assigned to one of four 10-min conditions: AVP touch (n = 73), AVP proximity (n = 62), AVP imagery (n = 57), or AVP waitlist (n = 57). Participants collected salivary cortisol (Cort) and α-amylase (sAA) upon waking and at 15 and 25 min post-condition from which parameters indicating adaptive physiological functioning were calculated. Multiple linear regression analyses showed that students in all three comparison conditions had lower posttest sAA (β proximity = −0.175, p = 0.017; β imagery = −0.214, p = 0.003; β waitlist = −0.138, p = 0.051), lower sAA-to-Cort ratios (AOCs) from pretest to posttest (β proximity = −0.277, p < 0.001; β imagery = −0.307, p < 0.001; β waitlist = −0.172, p = 0.014), lower AOCs from wakeup to posttest (β proximity = −0.135, p = 0.010; β imagery = −0.150, p = 0.004; β waitlist = −0.117, p = 0.021), and a smaller sAA increase from wakeup to posttest (β proximity = −0.216, p = 0.001; β imagery = −0.247, p < 0.001; β waitlist = −0.130, p = 0.033) compared with the AVP touch condition, indicating greater autonomic arousal (sAA) and coordination of stress systems (AOCs) in AVP touch participants. These results suggest that touch is the primary AVP component facilitating adaptive stress-related physiological states among participating university students. |
  | Date | 2022 |
  | Language | English |
  | Archive | Embase |
  | URL | https://www.embase.com/search/results?subaction=viewrecord&id=L2014306471&from=export |
  | Volume | 35 |
  | Pages | 443-461 |
  | Publication | Anthrozoos |
  | DOI | 10.1080/08927936.2021.1996025 |
  | Issue | 3 |
  | Journal Abbr | Anthrozoos |
  | ISSN | 1753-0377 |
  | Date Added | 11/07/2025, 14:16:49 |
  | Modified | 11/07/2025, 14:16:49 |

  ### Tags:

  - human-animal interaction
  - autonomic nervous system
  - anxiety
  - article
  - female
  - male
  - physiological stress
  - human
  - hydrocortisone
  - adult
  - human experiment
  - normal human
  - touch
  - young adult
  - amylase
  - hypothalamus hypophysis adrenal system
  - arousal
  - depression
  - mental health
  - emotionality
  - saliva level
  - saliva analysis
  - adrenal medullary cell
  - animal visitation program
  - animal visitation program imagery
  - animal visitation program proximity
  - animal visitation program waitlist
  - Beck Anxiety Inventory
  - Beck Depression Inventory
  - catecholamine
  - health care organization
  - health survey
  - Penn State Worry Questionnaire
  - Perceived Stress Scale
  - pretest posttest design
  - sympathetic reflex
  - university student

  ### Attachments

  - Full Text (HTML)
- ## Effects of Essential Animal Visitation Program (AVP) Components on Students’ Salivary α-Amylase and Amylase-to-Cortisol Ratios

  |  |  |
  | --- | --- |
  | Item Type | Journal Article |
  | Author | J.L. Vandagriff |
  | Author | A.M. Carr |
  | Author | S.M. Roeter Smith |
  | Author | P. Pendry |
  | Abstract | While efficacy trials suggest that Animal Visitation Programs (AVPs) relieve university student stress, their essential components are unknown. Students were randomly assigned to one of four 10-min conditions: AVP touch (n = 73), AVP proximity (n = 62), AVP imagery (n = 57), or AVP waitlist (n = 57). Participants collected salivary cortisol (Cort) and α-amylase (sAA) upon waking and at 15 and 25 min post-condition from which parameters indicating adaptive physiological functioning were calculated. Multiple linear regression analyses showed that students in all three comparison conditions had lower posttest sAA (β proximity = −0.175, p = 0.017; β imagery = −0.214, p = 0.003; β waitlist = −0.138, p = 0.051), lower sAA-to-Cort ratios (AOCs) from pretest to posttest (β proximity = −0.277, p < 0.001; β imagery = −0.307, p < 0.001; β waitlist = −0.172, p = 0.014), lower AOCs from wakeup to posttest (β proximity = −0.135, p = 0.010; β imagery = −0.150, p = 0.004; β waitlist = −0.117, p = 0.021), and a smaller sAA increase from wakeup to posttest (β proximity = −0.216, p = 0.001; β imagery = −0.247, p < 0.001; β waitlist = −0.130, p = 0.033) compared with the AVP touch condition, indicating greater autonomic arousal (sAA) and coordination of stress systems (AOCs) in AVP touch participants. These results suggest that touch is the primary AVP component facilitating adaptive stress-related physiological states among participating university students. |
  | Date | 2022 |
  | Language | English |
  | Archive | Embase |
  | URL | https://www.embase.com/search/results?subaction=viewrecord&id=L2014306471&from=export |
  | Volume | 35 |
  | Pages | 443-461 |
  | Publication | Anthrozoos |
  | DOI | 10.1080/08927936.2021.1996025 |
  | Issue | 3 |
  | Journal Abbr | Anthrozoos |
  | ISSN | 1753-0377 |
  | Date Added | 11/07/2025, 14:18:23 |
  | Modified | 11/07/2025, 14:18:23 |

  ### Tags:

  - human-animal interaction
  - autonomic nervous system
  - anxiety
  - article
  - female
  - male
  - physiological stress
  - human
  - hydrocortisone
  - adult
  - human experiment
  - normal human
  - touch
  - young adult
  - amylase
  - hypothalamus hypophysis adrenal system
  - arousal
  - depression
  - mental health
  - emotionality
  - saliva level
  - saliva analysis
  - adrenal medullary cell
  - animal visitation program
  - animal visitation program imagery
  - animal visitation program proximity
  - animal visitation program waitlist
  - Beck Anxiety Inventory
  - Beck Depression Inventory
  - catecholamine
  - health care organization
  - health survey
  - Penn State Worry Questionnaire
  - Perceived Stress Scale
  - pretest posttest design
  - sympathetic reflex
  - university student

  ### Attachments

  - Full Text (HTML)
- ## The role of oxytocin in relationships between dogs and humans and potential applications for the treatment of separation anxiety in dogs

  |  |  |
  | --- | --- |
  | Item Type | Journal Article |
  | Author | L.E. Thielke |
  | Author | M.A. Udell |
  | Abstract | The hormone oxytocin plays an important role in attachment formation and bonding between humans and domestic dogs. Recent research has led to increased interest in potential applications for intranasal oxytocin to aid with the treatment of psychological disorders in humans. While a few studies have explored the effects of intranasally administered oxytocin on social cognition and social bonding in dogs, alternative applications have not yet been explored for the treatment of behavioural problems in this species. One potentially important application for intranasal oxytocin in dogs could be the treatment of separation anxiety, a common attachment disorder in dogs. Here we provide an overview of what is known about the role of oxytocin in the human-dog bond and canine separation anxiety, and discuss considerations for future research looking to integrate oxytocin into behavioural treatment based on recent findings from both the human and dog literature. |
  | Date | 2017 |
  | Language | English |
  | Archive | Medline |
  | URL | https://www.embase.com/search/results?subaction=viewrecord&id=L620418131&from=export |
  | Volume | 92 |
  | Pages | 378-388 |
  | Publication | Biological reviews of the Cambridge Philosophical Society |
  | DOI | 10.1111/brv.12235 |
  | Issue | 1 |
  | Journal Abbr | Biol Rev Camb Philos Soc |
  | ISSN | 1469-185X |
  | Date Added | 11/07/2025, 12:13:34 |
  | Modified | 11/07/2025, 12:13:34 |

  ### Tags:

  - oxytocin
  - dog
  - separation anxiety
  - human
  - animal
  - human-animal bond
  - metabolism

  ### Attachments

  - Full Text (HTML)
- ## Repeated handling of pigs during rearing. I. Refusal of contact by the handler and reactivity to familiar and unfamiliar humans

  |  |  |
  | --- | --- |
  | Item Type | Journal Article |
  | Author | E.M.C. Terlouw |
  | Author | J. Porcher |
  | Abstract | Pigs housed in groups received different handling treatments for 40 d until slaughter age. Pigs of the human interaction (HI) and refusal of contact (RC) groups were individually introduced into a pen each day, where they remained for 3 min in the presence of a squatted handler. The handler tried to increase progressively physical reciprocal interactions with the HI pigs using eye and body contact and voice. The handler remained immobile and avoided eye contact and use of voice with RC pigs. These pigs were pushed away when they touched the handler. Control pigs remained in their home pens. Over sessions, HI pigs progressively increased physical interactions with the handler, up to 35% of their time. The RC pigs were motivated to interact with the handler as they tried to establish physical contact with the handler throughout the experiment. They increased levels of locomotion, rubbing, immobility, and snout contact with the wall, suggesting that they were frustrated by the refusal of contact. At the end of the experimental period, all pigs were subjected to three human exposure tests, where pigs were exposed to the handler and two other persons, one of which was unfamiliar, in a Latin square design. During this test, behavior of the humans was the same as for the RC treatment. The HI pigs discriminated between the handler and the other persons as indicated by their increased approach behavior towards the handler. Part of the prior handling experience was generalized to other humans as indicated by higher levels of proximity of HI and RC pigs with the different persons compared with controls. Physical contact with the human was associated with increased heart rates. Two possibilities are that these two characteristics are part of a general behavioral/physiological reactive profile, or that contact with humans provokes an arousal or emotional response. Despite this, behavioral data show that pigs are motivated to be in physical contact with a handler, even when the handler consistently refuses contact. ©2005 American Society of Animal Science. All rights reserved. |
  | Date | 2005 |
  | Language | English |
  | Archive | Medline |
  | URL | https://www.embase.com/search/results?subaction=viewrecord&id=L43599397&from=export |
  | Volume | 83 |
  | Pages | 1653-1663 |
  | Publication | Journal of Animal Science |
  | Issue | 7 |
  | Journal Abbr | J. Anim. Sci. |
  | ISSN | 0021-8812 |
  | Date Added | 11/07/2025, 12:10:58 |
  | Modified | 11/07/2025, 12:10:58 |

  ### Tags:

  - physiology
  - heart rate
  - social behavior
  - animal behavior
  - article
  - male
  - human
  - motivation
  - pig
  - animal
  - animal husbandry
  - statistics
  - classification
  - comparative study
  - meat
  - psychological aspect
  - standard
  - time
- ## Brain and heart activity during interactions with pet dogs: A portable electroencephalogram and heart rate variability study

  |  |  |
  | --- | --- |
  | Item Type | Journal Article |
  | Author | J.T. Teo |
  | Author | S.J. Johnstone |
  | Author | S.J. Thomas |
  | Abstract | Dog ownership has been linked to numerous benefits to human health and wellbeing. However, due to the lack of previous research on changes to brain activity during interactions with pet dogs, the underlying psychophysiological mechanisms are still unclear. The aim of the present study was to examine changes in heart rate (HR), heart rate variability (HRV), and electroencephalogram (EEG) power during interactions between dog owners and their pet dog. Fifty healthy adult dog owners completed baseline psychological measures and pet attachment scales. Subjective units of relaxation (SUR) as well as continuous EEG, HR, and HRV via portable devices were measured during five experimental conditions (baseline resting, relaxation-induction exercise, patting a toy dog, real dog present, and patting a real dog) in participants' homes. SUR was higher in all experimental conditions than at baseline. SUR was also higher during dog interaction than when the dog was present with no interaction. However, SUR during dog interaction was not significantly different from the toy dog and relaxation induction condition. Higher delta, theta, alpha, beta power and HR were found during dog interaction than all other conditions. Higher HRV was found during dog interaction compared to baseline, patting a toy dog, and relaxation-induction exercise, but not significantly different from the real dog present only condition. Lastly, overall HR correlated with psychological measures. Overall, the results show that there are significant changes in brain and heart activity when humans interact with pet dogs, consistent with increases in relaxation and focussed attention. These findings are relevant to understanding the potential mechanisms for health benefits associated with pets. |
  | Date | 2024 |
  | Language | English |
  | Archive | Embase |
  | URL | https://www.embase.com/search/results?subaction=viewrecord&id=L2034075814&from=export |
  | Volume | 204 |
  | Publication | International Journal of Psychophysiology |
  | DOI | 10.1016/j.ijpsycho.2024.112412 |
  | Issue | (Teo J.T., jtt268@uowmail.edu.au; Johnstone S.J., sjohnsto@uow.edu.au) School of Psychology, Faculty of the Arts, Social Sciences and Humanities, University of Wollongong, Wollongong, NSW, Australia |
  | Journal Abbr | Int. J. Psychophysiol. |
  | ISSN | 1872-7697 |
  | Date Added | 11/07/2025, 11:49:32 |
  | Modified | 11/07/2025, 11:49:32 |

  ### Tags:

  - human-animal interaction
  - heart rate variability
  - dog
  - article
  - female
  - male
  - nonhuman
  - human
  - adult
  - computer
  - controlled study
  - data analysis software
  - brain function
  - sensor
  - normal human
  - alpha rhythm
  - beta rhythm
  - continuous electroencephalography
  - delta rhythm
  - electroencephalograph
  - electroencephalograph electrode
  - Elite HRV CorSense
  - emotional attachment
  - exercise
  - lab on a chip
  - Mindwave Mobile II
  - monitor
  - pet animal
  - photoelectric plethysmograph
  - portable equipment
  - psychologic assessment
  - relaxation sensation
  - relaxation training
  - theta rhythm
  - ThinkGear

  ### Attachments

  - Full Text (HTML)
- ## Brain and heart activity during interactions with pet dogs: A portable electroencephalogram and heart rate variability study

  |  |  |
  | --- | --- |
  | Item Type | Journal Article |
  | Author | J.T. Teo |
  | Author | S.J. Johnstone |
  | Author | S.J. Thomas |
  | Abstract | Dog ownership has been linked to numerous benefits to human health and wellbeing. However, due to the lack of previous research on changes to brain activity during interactions with pet dogs, the underlying psychophysiological mechanisms are still unclear. The aim of the present study was to examine changes in heart rate (HR), heart rate variability (HRV), and electroencephalogram (EEG) power during interactions between dog owners and their pet dog. Fifty healthy adult dog owners completed baseline psychological measures and pet attachment scales. Subjective units of relaxation (SUR) as well as continuous EEG, HR, and HRV via portable devices were measured during five experimental conditions (baseline resting, relaxation-induction exercise, patting a toy dog, real dog present, and patting a real dog) in participants' homes. SUR was higher in all experimental conditions than at baseline. SUR was also higher during dog interaction than when the dog was present with no interaction. However, SUR during dog interaction was not significantly different from the toy dog and relaxation induction condition. Higher delta, theta, alpha, beta power and HR were found during dog interaction than all other conditions. Higher HRV was found during dog interaction compared to baseline, patting a toy dog, and relaxation-induction exercise, but not significantly different from the real dog present only condition. Lastly, overall HR correlated with psychological measures. Overall, the results show that there are significant changes in brain and heart activity when humans interact with pet dogs, consistent with increases in relaxation and focussed attention. These findings are relevant to understanding the potential mechanisms for health benefits associated with pets. |
  | Date | 2024 |
  | Language | English |
  | Archive | Embase |
  | URL | https://www.embase.com/search/results?subaction=viewrecord&id=L2034075814&from=export |
  | Volume | 204 |
  | Publication | International Journal of Psychophysiology |
  | DOI | 10.1016/j.ijpsycho.2024.112412 |
  | Issue | (Teo J.T., jtt268@uowmail.edu.au; Johnstone S.J., sjohnsto@uow.edu.au) School of Psychology, Faculty of the Arts, Social Sciences and Humanities, University of Wollongong, Wollongong, NSW, Australia |
  | Journal Abbr | Int. J. Psychophysiol. |
  | ISSN | 1872-7697 |
  | Date Added | 11/07/2025, 11:53:18 |
  | Modified | 11/07/2025, 11:53:18 |

  ### Tags:

  - human-animal interaction
  - heart rate variability
  - dog
  - article
  - female
  - male
  - nonhuman
  - human
  - adult
  - computer
  - controlled study
  - data analysis software
  - brain function
  - sensor
  - normal human
  - alpha rhythm
  - beta rhythm
  - continuous electroencephalography
  - delta rhythm
  - electroencephalograph
  - electroencephalograph electrode
  - Elite HRV CorSense
  - emotional attachment
  - exercise
  - lab on a chip
  - Mindwave Mobile II
  - monitor
  - pet animal
  - photoelectric plethysmograph
  - portable equipment
  - psychologic assessment
  - relaxation sensation
  - relaxation training
  - theta rhythm
  - ThinkGear

  ### Attachments

  - Full Text (HTML)
- ## Brain and heart activity during interactions with pet dogs: A portable electroencephalogram and heart rate variability study

  |  |  |
  | --- | --- |
  | Item Type | Journal Article |
  | Author | J.T. Teo |
  | Author | S.J. Johnstone |
  | Author | S.J. Thomas |
  | Abstract | Dog ownership has been linked to numerous benefits to human health and wellbeing. However, due to the lack of previous research on changes to brain activity during interactions with pet dogs, the underlying psychophysiological mechanisms are still unclear. The aim of the present study was to examine changes in heart rate (HR), heart rate variability (HRV), and electroencephalogram (EEG) power during interactions between dog owners and their pet dog. Fifty healthy adult dog owners completed baseline psychological measures and pet attachment scales. Subjective units of relaxation (SUR) as well as continuous EEG, HR, and HRV via portable devices were measured during five experimental conditions (baseline resting, relaxation-induction exercise, patting a toy dog, real dog present, and patting a real dog) in participants' homes. SUR was higher in all experimental conditions than at baseline. SUR was also higher during dog interaction than when the dog was present with no interaction. However, SUR during dog interaction was not significantly different from the toy dog and relaxation induction condition. Higher delta, theta, alpha, beta power and HR were found during dog interaction than all other conditions. Higher HRV was found during dog interaction compared to baseline, patting a toy dog, and relaxation-induction exercise, but not significantly different from the real dog present only condition. Lastly, overall HR correlated with psychological measures. Overall, the results show that there are significant changes in brain and heart activity when humans interact with pet dogs, consistent with increases in relaxation and focussed attention. These findings are relevant to understanding the potential mechanisms for health benefits associated with pets. |
  | Date | 2024 |
  | Language | English |
  | Archive | Embase |
  | URL | https://www.embase.com/search/results?subaction=viewrecord&id=L2034075814&from=export |
  | Volume | 204 |
  | Publication | International Journal of Psychophysiology |
  | DOI | 10.1016/j.ijpsycho.2024.112412 |
  | Issue | (Teo J.T., jtt268@uowmail.edu.au; Johnstone S.J., sjohnsto@uow.edu.au) School of Psychology, Faculty of the Arts, Social Sciences and Humanities, University of Wollongong, Wollongong, NSW, Australia |
  | Journal Abbr | Int. J. Psychophysiol. |
  | ISSN | 1872-7697 |
  | Date Added | 11/07/2025, 12:10:39 |
  | Modified | 11/07/2025, 12:10:39 |

  ### Tags:

  - human-animal interaction
  - heart rate variability
  - dog
  - article
  - female
  - male
  - nonhuman
  - human
  - adult
  - computer
  - controlled study
  - data analysis software
  - brain function
  - sensor
  - normal human
  - alpha rhythm
  - beta rhythm
  - continuous electroencephalography
  - delta rhythm
  - electroencephalograph
  - electroencephalograph electrode
  - Elite HRV CorSense
  - emotional attachment
  - exercise
  - lab on a chip
  - Mindwave Mobile II
  - monitor
  - pet animal
  - photoelectric plethysmograph
  - portable equipment
  - psychologic assessment
  - relaxation sensation
  - relaxation training
  - theta rhythm
  - ThinkGear

  ### Attachments

  - Full Text (HTML)
- ## Psychophysiological mechanisms underlying the potential health benefits of human-dog interactions: A systematic literature review

  |  |  |
  | --- | --- |
  | Item Type | Journal Article |
  | Author | J.T. Teo |
  | Author | S.J. Johnstone |
  | Author | S.S. Römer |
  | Author | S.J. Thomas |
  | Abstract | While the symbiotic nature of human-dog relationships and perceived benefits to human health have attracted much scientific interest, the mechanisms through which human-dog interactions may confer health benefits to humans are still poorly understood. The aim of this systematic literature review was to synthesize evidence of physiological changes associated with human-dog interactions with relevance to human health. Electronic databases were systematically searched (PubMed, MEDLINE with full text, Scopus, PsycINFO, CINAHL Plus with Full Text, and Web of Science Core Collection) for relevant studies. Of the 13,072 studies identified, 129 met the inclusion criteria, with approximately half being randomized trials (Level 2) based on the Oxford Centre for Evidence Based Medicine level system. Measures employed to study human physiological changes associated with human-dog interactions most commonly involved cardiac parameters and hormones, with negligible research of brain activity. The main positive findings were increases in heart rate variability and oxytocin, and decreases in cortisol with human-dog interactions. These physiological indicators are consistent with activation of the parasympathetic nervous system (PNS) and oxytocinergic system (OTS), and down-regulation of hypothalamic-pituitary-adrenal (HPA) axis activity. These results provide evidence of specific pathways through which human-dog contact may confer health benefits, likely through relaxation, bonding, and stress reduction. However, these findings should be interpreted contextually due to limitations and methodological differences. Previous research using other biological variables was limited in quantity and quality, thus impeding firm conclusions on other possible mechanisms. Further research is needed in some psychophysiological domains, particularly electroencephalography, to better understand central nervous system (CNS) effects. The findings of this review have implications for human-dog interactions to positively affect several stress-sensitive physiological pathways and thus confer health benefits. This supports their incorporation in various clinical, non-clinical, and research settings to develop evidence-based interventions and practices for cost-effective and efficacious ways to improve human health. |
  | Date | 2022 |
  | Language | English |
  | Archive | Embase |
  | URL | https://www.embase.com/search/results?subaction=viewrecord&id=L2019541778&from=export |
  | Volume | 180 |
  | Pages | 27-48 |
  | Publication | International Journal of Psychophysiology |
  | DOI | 10.1016/j.ijpsycho.2022.07.007 |
  | Issue | (Teo J.T., jtt268@uowmail.edu.au) School of Psychology, Faculty of the Arts, Social Sciences and Humanities, University of Wollongong, Wollongong, NSW, Australia |
  | Journal Abbr | Int. J. Psychophysiol. |
  | ISSN | 1872-7697 |
  | Date Added | 11/07/2025, 12:10:46 |
  | Modified | 11/07/2025, 12:10:46 |

  ### Tags:

  - blood pressure
  - oxytocin
  - prolactin
  - human-animal interaction
  - heart rate variability
  - autonomic nervous system
  - heart rate
  - dog
  - child
  - female
  - male
  - nonhuman
  - human
  - hydrocortisone
  - adult
  - aged
  - controlled study
  - epinephrine
  - noradrenalin
  - electroencephalogram
  - oxygen saturation
  - dopamine
  - review
  - leisure
  - adolescent
  - electrodermal response
  - cholinergic system
  - breathing rate
  - psychophysiology
  - young adult
  - human-animal bond
  - Medline
  - Scopus
  - Web of Science
  - alpha amylase saliva isoenzyme
  - amylase
  - animal assisted therapy
  - beta endorphin
  - C reactive protein
  - cardiac index
  - case control study
  - cholesterol
  - chromogranin A
  - Cinahl
  - cytokine
  - down regulation
  - evidence based medicine
  - fibrinogen
  - glycated hemoglobin
  - health
  - heart right atrium pressure
  - high density lipoprotein
  - hypothalamus hypophysis adrenal system
  - immunoglobulin A
  - insulin
  - leukocyte count
  - lymphocyte proliferation
  - maximal oxygen uptake
  - oxygenation
  - oxytocinergic system
  - phenylacetic acid
  - positron emission tomography
  - prefrontal cortex
  - PsycINFO
  - publication
  - pulmonary artery occlusion pressure
  - randomized controlled trial (topic)
  - skin temperature
  - social bonding
  - statistically significant result
  - stress management
  - systematic review
  - systemic vascular resistance
  - total cholesterol level
  - tympanic temperature

  ### Attachments

  - Full Text (HTML)
- ## Psychophysiological mechanisms underlying the potential health benefits of human-dog interactions: A systematic literature review

  |  |  |
  | --- | --- |
  | Item Type | Journal Article |
  | Author | J.T. Teo |
  | Author | S.J. Johnstone |
  | Author | S.S. Römer |
  | Author | S.J. Thomas |
  | Abstract | While the symbiotic nature of human-dog relationships and perceived benefits to human health have attracted much scientific interest, the mechanisms through which human-dog interactions may confer health benefits to humans are still poorly understood. The aim of this systematic literature review was to synthesize evidence of physiological changes associated with human-dog interactions with relevance to human health. Electronic databases were systematically searched (PubMed, MEDLINE with full text, Scopus, PsycINFO, CINAHL Plus with Full Text, and Web of Science Core Collection) for relevant studies. Of the 13,072 studies identified, 129 met the inclusion criteria, with approximately half being randomized trials (Level 2) based on the Oxford Centre for Evidence Based Medicine level system. Measures employed to study human physiological changes associated with human-dog interactions most commonly involved cardiac parameters and hormones, with negligible research of brain activity. The main positive findings were increases in heart rate variability and oxytocin, and decreases in cortisol with human-dog interactions. These physiological indicators are consistent with activation of the parasympathetic nervous system (PNS) and oxytocinergic system (OTS), and down-regulation of hypothalamic-pituitary-adrenal (HPA) axis activity. These results provide evidence of specific pathways through which human-dog contact may confer health benefits, likely through relaxation, bonding, and stress reduction. However, these findings should be interpreted contextually due to limitations and methodological differences. Previous research using other biological variables was limited in quantity and quality, thus impeding firm conclusions on other possible mechanisms. Further research is needed in some psychophysiological domains, particularly electroencephalography, to better understand central nervous system (CNS) effects. The findings of this review have implications for human-dog interactions to positively affect several stress-sensitive physiological pathways and thus confer health benefits. This supports their incorporation in various clinical, non-clinical, and research settings to develop evidence-based interventions and practices for cost-effective and efficacious ways to improve human health. |
  | Date | 2022 |
  | Language | English |
  | Archive | Embase |
  | URL | https://www.embase.com/search/results?subaction=viewrecord&id=L2019541778&from=export |
  | Volume | 180 |
  | Pages | 27-48 |
  | Publication | International Journal of Psychophysiology |
  | DOI | 10.1016/j.ijpsycho.2022.07.007 |
  | Issue | (Teo J.T., jtt268@uowmail.edu.au) School of Psychology, Faculty of the Arts, Social Sciences and Humanities, University of Wollongong, Wollongong, NSW, Australia |
  | Journal Abbr | Int. J. Psychophysiol. |
  | ISSN | 1872-7697 |
  | Date Added | 11/07/2025, 12:12:20 |
  | Modified | 11/07/2025, 12:12:20 |

  ### Tags:

  - blood pressure
  - oxytocin
  - prolactin
  - human-animal interaction
  - heart rate variability
  - autonomic nervous system
  - heart rate
  - dog
  - child
  - female
  - male
  - nonhuman
  - human
  - hydrocortisone
  - adult
  - aged
  - controlled study
  - epinephrine
  - noradrenalin
  - electroencephalogram
  - oxygen saturation
  - dopamine
  - review
  - leisure
  - adolescent
  - electrodermal response
  - cholinergic system
  - breathing rate
  - psychophysiology
  - young adult
  - human-animal bond
  - Medline
  - Scopus
  - Web of Science
  - alpha amylase saliva isoenzyme
  - amylase
  - animal assisted therapy
  - beta endorphin
  - C reactive protein
  - cardiac index
  - case control study
  - cholesterol
  - chromogranin A
  - Cinahl
  - cytokine
  - down regulation
  - evidence based medicine
  - fibrinogen
  - glycated hemoglobin
  - health
  - heart right atrium pressure
  - high density lipoprotein
  - hypothalamus hypophysis adrenal system
  - immunoglobulin A
  - insulin
  - leukocyte count
  - lymphocyte proliferation
  - maximal oxygen uptake
  - oxygenation
  - oxytocinergic system
  - phenylacetic acid
  - positron emission tomography
  - prefrontal cortex
  - PsycINFO
  - publication
  - pulmonary artery occlusion pressure
  - randomized controlled trial (topic)
  - skin temperature
  - social bonding
  - statistically significant result
  - stress management
  - systematic review
  - systemic vascular resistance
  - total cholesterol level
  - tympanic temperature

  ### Attachments

  - Full Text (HTML)
- ## Psychophysiological mechanisms underlying the potential health benefits of human-dog interactions: A systematic literature review

  |  |  |
  | --- | --- |
  | Item Type | Journal Article |
  | Author | J.T. Teo |
  | Author | S.J. Johnstone |
  | Author | S.S. Römer |
  | Author | S.J. Thomas |
  | Abstract | While the symbiotic nature of human-dog relationships and perceived benefits to human health have attracted much scientific interest, the mechanisms through which human-dog interactions may confer health benefits to humans are still poorly understood. The aim of this systematic literature review was to synthesize evidence of physiological changes associated with human-dog interactions with relevance to human health. Electronic databases were systematically searched (PubMed, MEDLINE with full text, Scopus, PsycINFO, CINAHL Plus with Full Text, and Web of Science Core Collection) for relevant studies. Of the 13,072 studies identified, 129 met the inclusion criteria, with approximately half being randomized trials (Level 2) based on the Oxford Centre for Evidence Based Medicine level system. Measures employed to study human physiological changes associated with human-dog interactions most commonly involved cardiac parameters and hormones, with negligible research of brain activity. The main positive findings were increases in heart rate variability and oxytocin, and decreases in cortisol with human-dog interactions. These physiological indicators are consistent with activation of the parasympathetic nervous system (PNS) and oxytocinergic system (OTS), and down-regulation of hypothalamic-pituitary-adrenal (HPA) axis activity. These results provide evidence of specific pathways through which human-dog contact may confer health benefits, likely through relaxation, bonding, and stress reduction. However, these findings should be interpreted contextually due to limitations and methodological differences. Previous research using other biological variables was limited in quantity and quality, thus impeding firm conclusions on other possible mechanisms. Further research is needed in some psychophysiological domains, particularly electroencephalography, to better understand central nervous system (CNS) effects. The findings of this review have implications for human-dog interactions to positively affect several stress-sensitive physiological pathways and thus confer health benefits. This supports their incorporation in various clinical, non-clinical, and research settings to develop evidence-based interventions and practices for cost-effective and efficacious ways to improve human health. |
  | Date | 2022 |
  | Language | English |
  | Archive | Embase |
  | URL | https://www.embase.com/search/results?subaction=viewrecord&id=L2019541778&from=export |
  | Volume | 180 |
  | Pages | 27-48 |
  | Publication | International Journal of Psychophysiology |
  | DOI | 10.1016/j.ijpsycho.2022.07.007 |
  | Issue | (Teo J.T., jtt268@uowmail.edu.au) School of Psychology, Faculty of the Arts, Social Sciences and Humanities, University of Wollongong, Wollongong, NSW, Australia |
  | Journal Abbr | Int. J. Psychophysiol. |
  | ISSN | 1872-7697 |
  | Date Added | 11/07/2025, 12:13:22 |
  | Modified | 11/07/2025, 12:13:22 |

  ### Tags:

  - blood pressure
  - oxytocin
  - prolactin
  - human-animal interaction
  - heart rate variability
  - autonomic nervous system
  - heart rate
  - dog
  - child
  - female
  - male
  - nonhuman
  - human
  - hydrocortisone
  - adult
  - aged
  - controlled study
  - epinephrine
  - noradrenalin
  - electroencephalogram
  - oxygen saturation
  - dopamine
  - review
  - leisure
  - adolescent
  - electrodermal response
  - cholinergic system
  - breathing rate
  - psychophysiology
  - young adult
  - human-animal bond
  - Medline
  - Scopus
  - Web of Science
  - alpha amylase saliva isoenzyme
  - amylase
  - animal assisted therapy
  - beta endorphin
  - C reactive protein
  - cardiac index
  - case control study
  - cholesterol
  - chromogranin A
  - Cinahl
  - cytokine
  - down regulation
  - evidence based medicine
  - fibrinogen
  - glycated hemoglobin
  - health
  - heart right atrium pressure
  - high density lipoprotein
  - hypothalamus hypophysis adrenal system
  - immunoglobulin A
  - insulin
  - leukocyte count
  - lymphocyte proliferation
  - maximal oxygen uptake
  - oxygenation
  - oxytocinergic system
  - phenylacetic acid
  - positron emission tomography
  - prefrontal cortex
  - PsycINFO
  - publication
  - pulmonary artery occlusion pressure
  - randomized controlled trial (topic)
  - skin temperature
  - social bonding
  - statistically significant result
  - stress management
  - systematic review
  - systemic vascular resistance
  - total cholesterol level
  - tympanic temperature

  ### Attachments

  - Full Text (HTML)
- ## Are pets stress busters or cause of allergy

  |  |  |
  | --- | --- |
  | Item Type | Journal Article |
  | Author | M. Tejas |
  | Author | V.M. Anantha Eashwar |
  | Author | S. Gopalakrishnan |
  | Abstract | Background: In brain science, stress is a sentiment of strain and pressure. Stress is a kind of mental torment. Sensitivity to pets are for the most part because of their hide which is truly unimportant .coming up next is the exploration done on the advantages of pets on undergrads and their conceivable unfriendly consequences for them. Method: Philosophy Cross Sectional examination done by convenience testing strategy. Information was gathered with the assistance of a poll. The subjects utilized for this investigation were College understudies from Kanchipuram area. Result: Out of the 150 respondents it was discovered that 66% had pressure and just 20% had hypersensitivity because of different causes. Among the pet proprietors there is a noteworthy decrease in worry after the entry of the pet. Conclusion: There are numerous advantages of pets. One such advantage is the decrease of pressure. The prevalence of sensitivity because of pets is truly immaterial .Thus the advantage of having a pet exceeds its hazard. The significant levels of pets give help and are regular pressure buster. |
  | Date | 2019 |
  | Language | English |
  | Archive | Embase |
  | URL | https://www.embase.com/search/results?subaction=viewrecord&id=L2004086292&from=export |
  | Volume | 10 |
  | Pages | 1619-1622 |
  | Publication | Indian Journal of Public Health Research and Development |
  | DOI | 10.5958/0976-5506.2019.03773.2 |
  | Issue | 11 |
  | Journal Abbr | Indian J. Public Health Res. Dev. |
  | ISSN | 0976-5506 |
  | Date Added | 11/07/2025, 14:16:58 |
  | Modified | 11/07/2025, 14:16:58 |

  ### Tags:

  - article
  - female
  - male
  - nonhuman
  - physiological stress
  - human
  - hydrocortisone
  - pet animal
  - clinical article
  - human-animal bond
  - allergy
  - psychological well-being
  - cross-sectional study
  - allergen
  - food allergy
  - hypersensitivity
  - pressure
  - prevalence

  ### Attachments

  - Full Text (HTML)
- ## Are pets stress busters or cause of allergy

  |  |  |
  | --- | --- |
  | Item Type | Journal Article |
  | Author | M. Tejas |
  | Author | V.M. Anantha Eashwar |
  | Author | S. Gopalakrishnan |
  | Abstract | Background: In brain science, stress is a sentiment of strain and pressure. Stress is a kind of mental torment. Sensitivity to pets are for the most part because of their hide which is truly unimportant .coming up next is the exploration done on the advantages of pets on undergrads and their conceivable unfriendly consequences for them. Method: Philosophy Cross Sectional examination done by convenience testing strategy. Information was gathered with the assistance of a poll. The subjects utilized for this investigation were College understudies from Kanchipuram area. Result: Out of the 150 respondents it was discovered that 66% had pressure and just 20% had hypersensitivity because of different causes. Among the pet proprietors there is a noteworthy decrease in worry after the entry of the pet. Conclusion: There are numerous advantages of pets. One such advantage is the decrease of pressure. The prevalence of sensitivity because of pets is truly immaterial .Thus the advantage of having a pet exceeds its hazard. The significant levels of pets give help and are regular pressure buster. |
  | Date | 2019 |
  | Language | English |
  | Archive | Embase |
  | URL | https://www.embase.com/search/results?subaction=viewrecord&id=L2004086292&from=export |
  | Volume | 10 |
  | Pages | 1619-1622 |
  | Publication | Indian Journal of Public Health Research and Development |
  | DOI | 10.5958/0976-5506.2019.03773.2 |
  | Issue | 11 |
  | Journal Abbr | Indian J. Public Health Res. Dev. |
  | ISSN | 0976-5506 |
  | Date Added | 11/07/2025, 14:18:32 |
  | Modified | 11/07/2025, 14:18:32 |

  ### Tags:

  - article
  - female
  - male
  - nonhuman
  - physiological stress
  - human
  - hydrocortisone
  - pet animal
  - clinical article
  - human-animal bond
  - allergy
  - psychological well-being
  - cross-sectional study
  - allergen
  - food allergy
  - hypersensitivity
  - pressure
  - prevalence

  ### Attachments

  - Full Text (HTML)
- ## Being a Dog: A Review of the Domestication Process

  |  |  |
  | --- | --- |
  | Item Type | Journal Article |
  | Author | D. Tancredi |
  | Author | I. Cardinali |
  | Abstract | The process of canine domestication represents certainly one of the most interesting questions that evolutionary biology aims to address. A “multiphase” view of this process is now accepted, with a first phase during which different groups of wolves were attracted by the anthropogenic niche and a second phase characterized by the gradual establishment of mutual relationships between wolves and humans. Here, we provide a review of dog (Canis familiaris) domestication, highlighting the ecological differences between dogs and wolves, analyzing the molecular mechanisms which seem to have influenced the affiliative behaviors first observed in Belyaev’s foxes, and describing the genetics of ancient European dogs. Then, we focus on three Mediterranean peninsulas (Balkan, Iberian and Italian), which together represent the main geographic area for studying canine domestication dynamics, as it has shaped the current genetic variability of dog populations, and where a well-defined European genetic structure was pinpointed through the analysis of uniparental genetic markers and their phylogeny. |
  | Date | 2023 |
  | Language | English |
  | Archive | Embase |
  | URL | https://www.embase.com/search/results?subaction=viewrecord&id=L2023466129&from=export |
  | Volume | 14 |
  | Publication | Genes |
  | DOI | 10.3390/genes14050992 |
  | Issue | 5 |
  | Journal Abbr | Genes |
  | ISSN | 2073-4425 |
  | Date Added | 11/07/2025, 12:12:17 |
  | Modified | 11/07/2025, 12:12:17 |

  ### Tags:

  - oxytocin
  - human-animal interaction
  - dog
  - animal behavior
  - nonhuman
  - physiological stress
  - review
  - domestication
  - animal genetics
  - fox
  - gene frequency
  - gene mutation
  - gene structure
  - genetic marker
  - genetic trait
  - genetic variability
  - haplogroup
  - haplotype
  - mitochondrial DNA
  - phenotype
  - phylogeny
  - serotonin receptor
  - species difference
  - wolf
  - Y chromosome

  ### Attachments

  - Full Text (HTML)
- ## Being a Dog: A Review of the Domestication Process

  |  |  |
  | --- | --- |
  | Item Type | Journal Article |
  | Author | D. Tancredi |
  | Author | I. Cardinali |
  | Abstract | The process of canine domestication represents certainly one of the most interesting questions that evolutionary biology aims to address. A “multiphase” view of this process is now accepted, with a first phase during which different groups of wolves were attracted by the anthropogenic niche and a second phase characterized by the gradual establishment of mutual relationships between wolves and humans. Here, we provide a review of dog (Canis familiaris) domestication, highlighting the ecological differences between dogs and wolves, analyzing the molecular mechanisms which seem to have influenced the affiliative behaviors first observed in Belyaev’s foxes, and describing the genetics of ancient European dogs. Then, we focus on three Mediterranean peninsulas (Balkan, Iberian and Italian), which together represent the main geographic area for studying canine domestication dynamics, as it has shaped the current genetic variability of dog populations, and where a well-defined European genetic structure was pinpointed through the analysis of uniparental genetic markers and their phylogeny. |
  | Date | 2023 |
  | Language | English |
  | Archive | Embase |
  | URL | https://www.embase.com/search/results?subaction=viewrecord&id=L2023466129&from=export |
  | Volume | 14 |
  | Publication | Genes |
  | DOI | 10.3390/genes14050992 |
  | Issue | 5 |
  | Journal Abbr | Genes |
  | ISSN | 2073-4425 |
  | Date Added | 11/07/2025, 12:13:19 |
  | Modified | 11/07/2025, 12:13:19 |

  ### Tags:

  - oxytocin
  - human-animal interaction
  - dog
  - animal behavior
  - nonhuman
  - physiological stress
  - review
  - domestication
  - animal genetics
  - fox
  - gene frequency
  - gene mutation
  - gene structure
  - genetic marker
  - genetic trait
  - genetic variability
  - haplogroup
  - haplotype
  - mitochondrial DNA
  - phenotype
  - phylogeny
  - serotonin receptor
  - species difference
  - wolf
  - Y chromosome

  ### Attachments

  - Full Text (HTML)
- ## THE IMPACT OF HUMAN-HORSE INTERACTIONS ON PHYSICAL AND EMOTIONAL WELLBEING

  |  |  |
  | --- | --- |
  | Item Type | Journal Article |
  | Author | R. Šveistienė |
  | Author | A. Vainoras |
  | Author | K. Berškienė |
  | Author | M. Landauskas |
  | Abstract | For centuries, people have discussed the special bond between humans and horses, with research indicating that interacting with horses can improve both psychological and physiological well-being [1, 2]. Scientific studies have explored these interactions, revealing that activities such as grooming and riding horses not only foster emotional connection but also lead to measurable health benefits: reductions in systolic blood pressure and improvements in emotional well-being following interactions with horses [3]. The objective of this study was to compare heart rate, blood pressure, and bio-psychosocial satisfaction between non-riders and riders during daily activities and interactions with a horse. The study involved 22 participants, divided into two groups: 15 non-riders with an average age of 27.4 years, and 7 riders with an average age of 26.3 years. Measurements included 24-hour heart rate monitoring, blood pressure, bio-psychosocial satisfaction assessments, and evaluations of physical activities of varying intensities. Interactions with the horse, such as flattering, brushing, feeding, and riding, were also assessed. Data were analyzed using the Pearson correlation, the Mann-Whitney test, and the Wilcoxon test through SPSS and MATLAB. The results showed that non-riders had a higher heart rate compared with riders during horse interactions. For example, the non-riders’ median heart rate was 93.5 beats per minute while flattering, 97.5 beats per minute while brushing, and 100.5 beats per minute while feeding the horse. In contrast, the riders’ median heart rates during these activities were significantly lower, with 77 beats per minute during flattering, 85 beats per minute during brushing, and 84 beats per minute during feeding. In the non-riders’ group, the physical condition after the sessions with the horse increased more than the emotional state (P < 0.05). HR was higher when non-riders were flattering, brushing and feeding the horse compared with the low and medium daily physical activity (P < 0.05). The non-riders’ systolic blood pressure decreased and satisfaction of emotional well-being and social interaction increased (P < 0.05) after sessions with a horse. In conclusion, there were no significant differences in blood pressure or bio-psychosocial satisfaction between the two groups before interacting with the horse. However, non-riders experienced a higher heart rate during flattering, brushing, and feeding compared with riders. Additionally, non-riders showed a greater improvement in the physical condition after the sessions, while both groups reported increased emotional wellbeing and social satisfaction after interacting with the horse. |
  | Date | 2024 |
  | Language | English |
  | Archive | Embase |
  | URL | https://www.embase.com/search/results?subaction=viewrecord&id=L2036571627&from=export |
  | Volume | 82(1) |
  | Pages | 138-138 |
  | Publication | Veterinarija ir Zootechnika |
  | Issue | (Šveistienė R., ruta.sveistiene@lsmu.lt) Animal Science Institute, Lithuanian University of Health Sciences, Lithuania |
  | Journal Abbr | Vet. Zootech. |
  | ISSN | 1392-2130 |
  | Date Added | 11/07/2025, 11:53:21 |
  | Modified | 11/07/2025, 11:53:21 |

  ### Tags:

  - blood pressure
  - human-animal interaction
  - horse
  - heart rate
  - physical activity
  - article
  - social interaction
  - human
  - adult
  - data analysis software
  - biopsychosocial model
  - blood pressure monitoring
  - emotional well-being
  - health insurance
  - MATLAB
  - non-rider
  - physical well-being
  - psychological well-being
  - rider
  - satisfaction
  - SPSS
  - systolic blood pressure
- ## THE IMPACT OF HUMAN-HORSE INTERACTIONS ON PHYSICAL AND EMOTIONAL WELLBEING

  |  |  |
  | --- | --- |
  | Item Type | Journal Article |
  | Author | R. Šveistienė |
  | Author | A. Vainoras |
  | Author | K. Berškienė |
  | Author | M. Landauskas |
  | Abstract | For centuries, people have discussed the special bond between humans and horses, with research indicating that interacting with horses can improve both psychological and physiological well-being [1, 2]. Scientific studies have explored these interactions, revealing that activities such as grooming and riding horses not only foster emotional connection but also lead to measurable health benefits: reductions in systolic blood pressure and improvements in emotional well-being following interactions with horses [3]. The objective of this study was to compare heart rate, blood pressure, and bio-psychosocial satisfaction between non-riders and riders during daily activities and interactions with a horse. The study involved 22 participants, divided into two groups: 15 non-riders with an average age of 27.4 years, and 7 riders with an average age of 26.3 years. Measurements included 24-hour heart rate monitoring, blood pressure, bio-psychosocial satisfaction assessments, and evaluations of physical activities of varying intensities. Interactions with the horse, such as flattering, brushing, feeding, and riding, were also assessed. Data were analyzed using the Pearson correlation, the Mann-Whitney test, and the Wilcoxon test through SPSS and MATLAB. The results showed that non-riders had a higher heart rate compared with riders during horse interactions. For example, the non-riders’ median heart rate was 93.5 beats per minute while flattering, 97.5 beats per minute while brushing, and 100.5 beats per minute while feeding the horse. In contrast, the riders’ median heart rates during these activities were significantly lower, with 77 beats per minute during flattering, 85 beats per minute during brushing, and 84 beats per minute during feeding. In the non-riders’ group, the physical condition after the sessions with the horse increased more than the emotional state (P < 0.05). HR was higher when non-riders were flattering, brushing and feeding the horse compared with the low and medium daily physical activity (P < 0.05). The non-riders’ systolic blood pressure decreased and satisfaction of emotional well-being and social interaction increased (P < 0.05) after sessions with a horse. In conclusion, there were no significant differences in blood pressure or bio-psychosocial satisfaction between the two groups before interacting with the horse. However, non-riders experienced a higher heart rate during flattering, brushing, and feeding compared with riders. Additionally, non-riders showed a greater improvement in the physical condition after the sessions, while both groups reported increased emotional wellbeing and social satisfaction after interacting with the horse. |
  | Date | 2024 |
  | Language | English |
  | Archive | Embase |
  | URL | https://www.embase.com/search/results?subaction=viewrecord&id=L2036571627&from=export |
  | Volume | 82(1) |
  | Pages | 138-138 |
  | Publication | Veterinarija ir Zootechnika |
  | Issue | (Šveistienė R., ruta.sveistiene@lsmu.lt) Animal Science Institute, Lithuanian University of Health Sciences, Lithuania |
  | Journal Abbr | Vet. Zootech. |
  | ISSN | 1392-2130 |
  | Date Added | 11/07/2025, 12:10:44 |
  | Modified | 11/07/2025, 12:10:44 |

  ### Tags:

  - blood pressure
  - human-animal interaction
  - horse
  - heart rate
  - physical activity
  - article
  - social interaction
  - human
  - adult
  - data analysis software
  - biopsychosocial model
  - blood pressure monitoring
  - emotional well-being
  - health insurance
  - MATLAB
  - non-rider
  - physical well-being
  - psychological well-being
  - rider
  - satisfaction
  - SPSS
  - systolic blood pressure
- ## Long-term stress levels are synchronized in dogs and their owners

  |  |  |
  | --- | --- |
  | Item Type | Journal Article |
  | Author | A.-S. Sundman |
  | Author | E. Van Poucke |
  | Author | A.-C. Svensson Holm |
  | Author | Å. Faresjö |
  | Author | E. Theodorsson |
  | Author | P. Jensen |
  | Author | L.S.V. Roth |
  | Abstract | This study reveals, for the first time, an interspecific synchronization in long-term stress levels. Previously, acute stress, has been shown to be highly contagious both among humans and between individuals of other species. Here, long-term stress synchronization in dogs and their owners was investigated. We studied 58 dog-human dyads and analyzed their hair cortisol concentrations (HCC) at two separate occasions, reflecting levels during previous summer and winter months. The personality traits of both dogs and their owners were determined through owner-completed Dog Personality Questionnaire (DPQ) and human Big Five Inventory (BFI) surveys. In addition, the dogs' activity levels were continuously monitored with a remote cloud-based activity collar for one week. Shetland sheepdogs (N = 33) and border collies (N = 25), balanced for sex, participated, and both pet dogs and actively competing dogs (agility and obedience) were included to represent different lifestyles. The results showed significant interspecies correlations in long-term stress where human HCC from both summer and winter samplings correlated strongly with dog HCC (summer: N = 57, χ2 = 23.697, P < 0.001, β = 0.235; winter: N = 55, χ2 = 13.796, P < 0.001, β = 0.027). Interestingly, the dogs' activity levels did not affect HCC, nor did the amount of training sessions per week, showing that the HCC levels were not related to general physical activity. Additionally, there was a seasonal effect in HCC. However, although dogs' personalities had little effects on their HCC, the human personality traits neuroticism, conscientiousness, and openness significantly affected dog HCC. Hence, we suggest that dogs, to a great extent, mirror the stress level of their owners. |
  | Date | 2019 |
  | Language | English |
  | Archive | Medline |
  | URL | https://www.embase.com/search/results?subaction=viewrecord&id=L628167847&from=export |
  | Volume | 9 |
  | Pages | 7391 |
  | Publication | Scientific reports |
  | DOI | 10.1038/s41598-019-43851-x |
  | Issue | 1 |
  | Journal Abbr | Sci Rep |
  | ISSN | 2045-2322 |
  | Date Added | 11/07/2025, 14:18:33 |
  | Modified | 11/07/2025, 14:18:33 |

  ### Tags:

  - physiology
  - dog
  - animal behavior
  - physiological stress
  - human
  - hydrocortisone
  - questionnaire
  - animal
  - human-animal bond
  - personality
  - longitudinal study

  ### Attachments

  - Full Text (HTML)
- ## Dog–Owner Relationship, Owner Interpretations and Dog Personality Are Connected with the Emotional Reactivity of Dogs

  |  |  |
  | --- | --- |
  | Item Type | Journal Article |
  | Author | S. Somppi |
  | Author | H. Törnqvist |
  | Author | A. Koskela |
  | Author | A. Vehkaoja |
  | Author | K. Tiira |
  | Author | H. Väätäjä |
  | Author | V. Surakka |
  | Author | O. Vainio |
  | Author | M.V. Kujala |
  | Abstract | We evaluated the effect of the dog–owner relationship on dogs’ emotional reactivity, quantified with heart rate variability (HRV), behavioral changes, physical activity and dog owner interpretations. Twenty nine adult dogs encountered five different emotional situations (i.e., stroking, a feeding toy, separation from the owner, reunion with the owner, a sudden appearance of a novel object). The results showed that both negative and positive situations provoked signs of heightened arousal in dogs. During negative situations, owners’ ratings about the heightened emotional arousal correlated with lower HRV, higher physical activity and more behaviors that typically index arousal and fear. The three factors of The Monash Dog–Owner Relationship Scale (MDORS) were reflected in the dogs’ heart rate variability and behaviors: the Emotional Closeness factor was related to increased HRV (p = 0.009), suggesting this aspect is associated with the secure base effect, and the Shared Activities factor showed a trend toward lower HRV (p = 0.067) along with more owner-directed behaviors reflecting attachment related arousal. In contrast, the Perceived Costs factor was related to higher HRV (p = 0.009) along with less fear and less owner-directed behaviors, which may reflect the dog’s more independent personality. In conclusion, dogs’ emotional reactivity and the dog–owner relationship modulate each other, depending on the aspect of the relationship and dogs’ individual responsivity. |
  | Date | 2022 |
  | Language | English |
  | Archive | Embase |
  | URL | https://www.embase.com/search/results?subaction=viewrecord&id=L2016914621&from=export |
  | Volume | 12 |
  | Publication | Animals |
  | DOI | 10.3390/ani12111338 |
  | Issue | 11 |
  | Journal Abbr | Animals |
  | ISSN | 2076-2615 |
  | Date Added | 11/07/2025, 12:10:48 |
  | Modified | 11/07/2025, 12:10:48 |

  ### Tags:

  - heart rate variability
  - behavior
  - physical activity
  - dog
  - animal experiment
  - article
  - female
  - male
  - nonhuman
  - emotional attachment
  - questionnaire
  - quantitative analysis
  - cognitive function test
  - feeding
  - intimacy
  - personality
  - shyness
  - task performance

  ### Attachments

  - Full Text (HTML)
- ## Best practices for physiological data collection in youth with autism and co-occurring mental health diagnoses: Implications for human-animal intervention research

  |  |  |
  | --- | --- |
  | Item Type | Journal Article |
  | Author | C.M. Smith |
  | Author | K. Weimann |
  | Author | M. Widick |
  | Author | T. Merritt |
  | Author | H. Christensen |
  | Author | M. Siegel |
  | Author | Z. Pan |
  | Author | R.L. Gabriels |
  | Abstract | The purpose of this paper is to serve as a catalyst for the human-animal interaction research field to improve scientific rigor and accelerate the knowledge of field-based physiological responses during equine-assisted services in youth with autism spectrum disorder. This paper outlines the best practices for collecting and analyzing electrocardiogram and electrodermal activity in youth with autism spectrum disorder, utilized during a 10-week therapeutic horseback riding intervention. • Motivation strategies such as device choice, reward systems, and a visual schedule should be implemented to improve participant compliance. In addition, devices should be secured to the participant following implementation of appropriate desensitization techniques. • Time-domain heart rate variability analyses are more appropriate during therapeutic horseback riding data collection compared to frequency-domain approaches. For electrodermal activity, tonic responses should be assessed as opposed to phasic analyses. • An effective data monitoring team including the Data Collection Research Personnel, Site Principal Investigator, Physiologist, and Therapeutic Riding Center Intervention Lead are key to increasing the quality of usable data in equine-assisted service research environments. |
  | Date | 2025 |
  | Language | English |
  | Archive | Embase |
  | URL | https://www.embase.com/search/results?subaction=viewrecord&id=L2038140847&from=export |
  | Volume | 14 |
  | Publication | MethodsX |
  | DOI | 10.1016/j.mex.2025.103284 |
  | Issue | (Smith C.M., Cory\_M\_Smith@Baylor.edu) Baylor University Waco, TX Robbins College of Health and Human Sciences, United States |
  | Journal Abbr | MethodsX |
  | ISSN | 2215-0161 |
  | Date Added | 11/07/2025, 11:53:15 |
  | Modified | 11/07/2025, 11:53:15 |

  ### Tags:

  - heart rate variability
  - child
  - article
  - female
  - male
  - human
  - controlled study
  - data analysis software
  - hippotherapy
  - horseback riding
  - randomized controlled trial
  - information processing
  - sensor
  - motivation
  - adolescent
  - autism
  - best practice
  - desensitization
  - devices
  - electrocardiograph
  - electrocardiography
  - electrode
  - electrodermal response
  - information processing device
  - juvenile
  - major clinical study
  - mental disease
  - NCT04606966
  - patient compliance
  - personnel
  - reward

  ### Attachments

  - Full Text (HTML)
- ## Best practices for physiological data collection in youth with autism and co-occurring mental health diagnoses: Implications for human-animal intervention research

  |  |  |
  | --- | --- |
  | Item Type | Journal Article |
  | Author | C.M. Smith |
  | Author | K. Weimann |
  | Author | M. Widick |
  | Author | T. Merritt |
  | Author | H. Christensen |
  | Author | M. Siegel |
  | Author | Z. Pan |
  | Author | R.L. Gabriels |
  | Abstract | The purpose of this paper is to serve as a catalyst for the human-animal interaction research field to improve scientific rigor and accelerate the knowledge of field-based physiological responses during equine-assisted services in youth with autism spectrum disorder. This paper outlines the best practices for collecting and analyzing electrocardiogram and electrodermal activity in youth with autism spectrum disorder, utilized during a 10-week therapeutic horseback riding intervention. • Motivation strategies such as device choice, reward systems, and a visual schedule should be implemented to improve participant compliance. In addition, devices should be secured to the participant following implementation of appropriate desensitization techniques. • Time-domain heart rate variability analyses are more appropriate during therapeutic horseback riding data collection compared to frequency-domain approaches. For electrodermal activity, tonic responses should be assessed as opposed to phasic analyses. • An effective data monitoring team including the Data Collection Research Personnel, Site Principal Investigator, Physiologist, and Therapeutic Riding Center Intervention Lead are key to increasing the quality of usable data in equine-assisted service research environments. |
  | Date | 2025 |
  | Language | English |
  | Archive | Embase |
  | URL | https://www.embase.com/search/results?subaction=viewrecord&id=L2038140847&from=export |
  | Volume | 14 |
  | Publication | MethodsX |
  | DOI | 10.1016/j.mex.2025.103284 |
  | Issue | (Smith C.M., Cory\_M\_Smith@Baylor.edu) Baylor University Waco, TX Robbins College of Health and Human Sciences, United States |
  | Journal Abbr | MethodsX |
  | ISSN | 2215-0161 |
  | Date Added | 11/07/2025, 12:10:36 |
  | Modified | 11/07/2025, 12:10:36 |

  ### Tags:

  - heart rate variability
  - child
  - article
  - female
  - male
  - human
  - controlled study
  - data analysis software
  - hippotherapy
  - horseback riding
  - randomized controlled trial
  - information processing
  - sensor
  - motivation
  - adolescent
  - autism
  - best practice
  - desensitization
  - devices
  - electrocardiograph
  - electrocardiography
  - electrode
  - electrodermal response
  - information processing device
  - juvenile
  - major clinical study
  - mental disease
  - NCT04606966
  - patient compliance
  - personnel
  - reward

  ### Attachments

  - Full Text (HTML)
- ## Inhaled oxytocin increases positive social behaviors in newborn macaques

  |  |  |
  | --- | --- |
  | Item Type | Journal Article |
  | Author | E.A. Simpson |
  | Author | V. Sclafani |
  | Author | A. Paukner |
  | Author | A.F. Hamel |
  | Author | M.A. Novak |
  | Author | J.S. Meyer |
  | Author | S.J. Suomi |
  | Author | P.F. Ferrari |
  | Abstract | Early caregiver-infant interactions are critical for infants' socioemotional and cognitive development. Several hormones and neuromodulators, including oxytocin, affect these interactions. Exogenous oxytocin promotes social behaviors in several species, including human and nonhuman primates. Although exogenous oxytocin increases social function in adults-including expression recognition and affiliation-it is unknown whether oxytocin can increase social interactions in infants. We hypothesized that nebulized oxytocin would increase affiliative social behaviors and such effects would be modulated by infants' social skills, measured earlier in development. We also hypothesized that oxytocin's effects on social behaviors may be due to its anxiolytic effects. We tested these hypotheses in a blind study by nebulizing 7-to 14-d-old macaques (n = 28) with oxytocin or saline. Following oxytocin administration, infants' facial gesturing at a human caregiver increased, and infants' salivary oxytocin was positively correlated with the time spent in close proximity to a caregiver. Infants' imitative skill (measured earlier in development: 1-7 d of age) predicted oxytocin-associated increases in affiliative behaviors-lip smacking, visual attention to a caregiver, and time in close proximity to a caregiver-suggesting that infants with higher propensities for positive social interactions are more sensitive to exogenous oxytocin. Oxytocin also decreased salivary cortisol, but not stressrelated behaviors (e.g., scratching), suggesting the possibility of some anxiolytic effects. To our knowledge, this study provides the first evidence that oxytocin increases positive social behaviors in newborns. This information is of critical importance for potential interventions aimed at ameliorating inadequate social behaviors in infants with higher likelihood of developing neurodevelopmental disorder. |
  | Date | 2014 |
  | Language | English |
  | Archive | Embase |
  | URL | https://www.embase.com/search/results?subaction=viewrecord&id=L373081704&from=export |
  | Volume | 111 |
  | Pages | 6922-6927 |
  | Publication | Proceedings of the National Academy of Sciences of the United States of America |
  | DOI | 10.1073/pnas.1402471111 |
  | Issue | 19 |
  | Journal Abbr | Proc. Natl. Acad. Sci. U. S. A. |
  | ISSN | 1091-6490 |
  | Date Added | 11/07/2025, 14:18:42 |
  | Modified | 11/07/2025, 14:18:42 |

  ### Tags:

  - oxytocin
  - social behavior
  - animal behavior
  - animal experiment
  - article
  - female
  - male
  - nonhuman
  - social interaction
  - hydrocortisone
  - controlled study
  - facial expression
  - newborn
  - caregiver
  - human-animal bond
  - priority journal
  - infant
  - saliva level
  - scratching
  - attention
  - cognitive development
  - gesture
  - Macaca
  - nebulization
  - neuromodulation
  - oxytocin test
  - positive feedback
  - skill
  - sodium chloride

  ### Attachments

  - Full Text (HTML)
- ## The value of (research on) animals in children's lives:

  |  |  |
  | --- | --- |
  | Item Type | Journal Article |
  | Author | R.L. Severson |
  | Date | 2014 |
  | Language | English |
  | Archive | Embase |
  | URL | https://www.embase.com/search/results?subaction=viewrecord&id=L372736479&from=export |
  | Volume | 57 |
  | Pages | 26-29 |
  | Publication | Human Development |
  | DOI | 10.1159/000357792 |
  | Issue | 1 |
  | Journal Abbr | Hum. Dev. |
  | ISSN | 1423-0054 |
  | Date Added | 11/07/2025, 12:10:57 |
  | Modified | 11/07/2025, 12:10:57 |

  ### Tags:

  - blood pressure
  - heart rate
  - dog
  - nonhuman
  - social interaction
  - human
  - note
  - autism
  - human-animal bond
  - attention deficit hyperactivity disorder
  - conceptual framework
  - disease severity
  - childhood disease
  - aggression
  - child development
  - conduct disorder
  - domestic animal
  - interpersonal communication
  - pet therapy
  - priority journal
  - systems theory

  ### Attachments

  - Full Text (HTML)
- ## Inside the Interaction: Contact With Familiar Humans Modulates Heart Rate Variability in Horses

  |  |  |
  | --- | --- |
  | Item Type | Journal Article |
  | Author | C. Scopa |
  | Author | A. Greco |
  | Author | L. Contalbrigo |
  | Author | E. Fratini |
  | Author | A. Lanatà |
  | Author | E.P. Scilingo |
  | Author | P. Baragli |
  | Abstract | A human–animal relationship can be developed through subsequent interactions, affected by the positive or negative emotional valence of the proceeding one. Horses implement a process of categorization to classify humans with whom they interact as positive, negative, or neutral stimuli by evaluating the kind of approach and the nature of the contact. In these terms, human–animal interactions are emotionally charged events, eliciting specific emotional states in both subjects involved. Although the human–horse relationship has been mainly investigated through behavioral analysis, physiological indicators are needed for a more objective assessment of the emotional responses. Heart rate variability (HRV) is a commonly used autonomic nervous system (ANS) correlate estimating the sympathovagal balance as a psychophysiological marker of emotion regulation in horses. We have assumed that long-term positive relationships with humans may have a positive and immediate impact on the emotional arousal of the horse, detectable, via ANS activity, during the interaction. We analyzed horses' heartbeat dynamics during their interaction with either familiar or unfamiliar handlers, applying a standardized experimental protocol consisting of three different conditions shifting from the absence of interaction to physical contact. The ANS signals were monitored through an innovative non-invasive wearable system, not interfering with the unconscious emotional response of the animal. We demonstrated that horses appeared to feel more relaxed while physically interacting (e.g., grooming on the right side) with some familiar handlers compared to the same task performed by someone unfamiliar. The shift of the sympathovagal balance toward a vagal predominance suggests that the horses experienced a decrease in stress response as a function not only of the handler's familiarity but also of the type of interaction they are experiencing. These results constitute the objective evidence of horses' capacity to individually recognize a familiar person, adding the crucial role of familiarity with the handler as a paramount component of human–animal interaction. Our rigorous methodological approach may provide a significant contribution to various fields such as animal welfare while further investigating the emotional side of the human–animal relationships. |
  | Date | 2020 |
  | Language | English |
  | Archive | Embase |
  | URL | https://www.embase.com/search/results?subaction=viewrecord&id=L633646243&from=export |
  | Volume | 7 |
  | Publication | Frontiers in Veterinary Science |
  | DOI | 10.3389/fvets.2020.582759 |
  | Issue | (Scopa C., chiara.scopa@gmail.com; Contalbrigo L.) Italian National Reference Centre for Animal Assisted Interventions, Istituto Zooprofilattico Sperimentale delle Venezie, Legnaro, Italy |
  | Journal Abbr | Front. Vet. Sci. |
  | ISSN | 2297-1769 |
  | Date Added | 11/07/2025, 12:10:51 |
  | Modified | 11/07/2025, 12:10:51 |

  ### Tags:

  - animal welfare
  - heart rate variability
  - autonomic nervous system
  - emotion regulation
  - article
  - female
  - male
  - nonhuman
  - physiological stress
  - human
  - adult
  - controlled study
  - grooming
  - human experiment
  - arousal
  - domestic horse
  - personality development

  ### Attachments

  - Full Text (HTML)
- ## Emerging cardiovascular risk research: Impact of pets on cardiovascular risk prevention

  |  |  |
  | --- | --- |
  | Item Type | Journal Article |
  | Author | P.J. Schreiner |
  | Abstract | Animals interact with humans in multiple ways, including as therapy and service animals, commercially as livestock, as wildlife, and in zoos. But the most common interaction is as companion animals in our homes, with an estimated 180 million cats and dogs living in US households. While pet ownership has been reported to have many health benefits, the findings are inconsistent. Cardiovascular risk factors such as lipids, glucose, obesity, and heart rate variability have improved, worsened, or remained the same in the limited number of studies considering companion animals. Physical activity increases have more consistently been linked with dog ownership, although whether this reflects antecedent motivation or direct benefit from the dog is unclear. Allergies and asthma also are variably linked to pet ownership and are confounded by family history of atopy and timing of exposure to pet dander. The benefits of companion animals are most likely to be through reduction in depression, anxiety, and social isolation, but these studies have been largely cross-sectional and may depend on degree of bonding of the owner with the animal. Positive relationships show measurably higher oxytocin with lower cortisol and alpha-amylase levels. Finally, pet ownership is also a marker of better socioeconomic status and family stability, and if companion animals are to provide cardiovascular risk benefit, the route should perhaps be through improved education and opportunity for ownership. |
  | Date | 2016 |
  | Language | English |
  | Archive | Embase |
  | URL | https://www.embase.com/search/results?subaction=viewrecord&id=L613788156&from=export |
  | Volume | 10 |
  | Pages | 1-8 |
  | Publication | Current Cardiovascular Risk Reports |
  | DOI | 10.1007/s12170-016-0489-2 |
  | Issue | 2 |
  | Journal Abbr | Curr. Cardiovasc. Risk Rep. |
  | ISSN | 1932-9563 |
  | Date Added | 11/07/2025, 12:10:56 |
  | Modified | 11/07/2025, 12:10:56 |

  ### Tags:

  - oxytocin
  - heart rate variability
  - anxiety
  - article
  - human
  - social isolation
  - exercise
  - pet animal
  - psychologic assessment
  - blood pressure monitoring
  - hydrocortisone blood level
  - risk factor
  - depression
  - amylase blood level
  - asthma
  - cardiovascular risk
  - cholesterol blood level
  - lifestyle modification
  - lisinopril
  - obesity
  - research
  - socioeconomics

  ### Attachments

  - Full Text (HTML)
- ## Emerging cardiovascular risk research: Impact of pets on cardiovascular risk prevention

  |  |  |
  | --- | --- |
  | Item Type | Journal Article |
  | Author | P.J. Schreiner |
  | Abstract | Animals interact with humans in multiple ways, including as therapy and service animals, commercially as livestock, as wildlife, and in zoos. But the most common interaction is as companion animals in our homes, with an estimated 180 million cats and dogs living in US households. While pet ownership has been reported to have many health benefits, the findings are inconsistent. Cardiovascular risk factors such as lipids, glucose, obesity, and heart rate variability have improved, worsened, or remained the same in the limited number of studies considering companion animals. Physical activity increases have more consistently been linked with dog ownership, although whether this reflects antecedent motivation or direct benefit from the dog is unclear. Allergies and asthma also are variably linked to pet ownership and are confounded by family history of atopy and timing of exposure to pet dander. The benefits of companion animals are most likely to be through reduction in depression, anxiety, and social isolation, but these studies have been largely cross-sectional and may depend on degree of bonding of the owner with the animal. Positive relationships show measurably higher oxytocin with lower cortisol and alpha-amylase levels. Finally, pet ownership is also a marker of better socioeconomic status and family stability, and if companion animals are to provide cardiovascular risk benefit, the route should perhaps be through improved education and opportunity for ownership. |
  | Date | 2016 |
  | Language | English |
  | Archive | Embase |
  | URL | https://www.embase.com/search/results?subaction=viewrecord&id=L613788156&from=export |
  | Volume | 10 |
  | Pages | 1-8 |
  | Publication | Current Cardiovascular Risk Reports |
  | DOI | 10.1007/s12170-016-0489-2 |
  | Issue | 2 |
  | Journal Abbr | Curr. Cardiovasc. Risk Rep. |
  | ISSN | 1932-9563 |
  | Date Added | 11/07/2025, 12:13:34 |
  | Modified | 11/07/2025, 12:13:34 |

  ### Tags:

  - oxytocin
  - heart rate variability
  - anxiety
  - article
  - human
  - social isolation
  - exercise
  - pet animal
  - psychologic assessment
  - blood pressure monitoring
  - hydrocortisone blood level
  - risk factor
  - depression
  - amylase blood level
  - asthma
  - cardiovascular risk
  - cholesterol blood level
  - lifestyle modification
  - lisinopril
  - obesity
  - research
  - socioeconomics

  ### Attachments

  - Full Text (HTML)
- ## Emerging cardiovascular risk research: Impact of pets on cardiovascular risk prevention

  |  |  |
  | --- | --- |
  | Item Type | Journal Article |
  | Author | P.J. Schreiner |
  | Abstract | Animals interact with humans in multiple ways, including as therapy and service animals, commercially as livestock, as wildlife, and in zoos. But the most common interaction is as companion animals in our homes, with an estimated 180 million cats and dogs living in US households. While pet ownership has been reported to have many health benefits, the findings are inconsistent. Cardiovascular risk factors such as lipids, glucose, obesity, and heart rate variability have improved, worsened, or remained the same in the limited number of studies considering companion animals. Physical activity increases have more consistently been linked with dog ownership, although whether this reflects antecedent motivation or direct benefit from the dog is unclear. Allergies and asthma also are variably linked to pet ownership and are confounded by family history of atopy and timing of exposure to pet dander. The benefits of companion animals are most likely to be through reduction in depression, anxiety, and social isolation, but these studies have been largely cross-sectional and may depend on degree of bonding of the owner with the animal. Positive relationships show measurably higher oxytocin with lower cortisol and alpha-amylase levels. Finally, pet ownership is also a marker of better socioeconomic status and family stability, and if companion animals are to provide cardiovascular risk benefit, the route should perhaps be through improved education and opportunity for ownership. |
  | Date | 2016 |
  | Language | English |
  | Archive | Embase |
  | URL | https://www.embase.com/search/results?subaction=viewrecord&id=L613788156&from=export |
  | Volume | 10 |
  | Pages | 1-8 |
  | Publication | Current Cardiovascular Risk Reports |
  | DOI | 10.1007/s12170-016-0489-2 |
  | Issue | 2 |
  | Journal Abbr | Curr. Cardiovasc. Risk Rep. |
  | ISSN | 1932-9563 |
  | Date Added | 11/07/2025, 14:18:39 |
  | Modified | 11/07/2025, 14:18:39 |

  ### Tags:

  - oxytocin
  - heart rate variability
  - anxiety
  - article
  - human
  - social isolation
  - exercise
  - pet animal
  - psychologic assessment
  - blood pressure monitoring
  - hydrocortisone blood level
  - risk factor
  - depression
  - amylase blood level
  - asthma
  - cardiovascular risk
  - cholesterol blood level
  - lifestyle modification
  - lisinopril
  - obesity
  - research
  - socioeconomics

  ### Attachments

  - Full Text (HTML)
- ## Psychobiological Factors Affecting Cortisol Variability in Human-Dog Dyads

  |  |  |
  | --- | --- |
  | Item Type | Journal Article |
  | Author | Iris Schöberl |
  | Author | Manuela Wedl |
  | Author | Andrea Beetz |
  | Author | Kurt Kotrschal |
  | Editor | Urs M. Nater |
  | Date | 2017-2-8 |
  | Language | en |
  | Library Catalogue | DOI.org (Crossref) |
  | URL | https://dx.plos.org/10.1371/journal.pone.0170707 |
  | Accessed | 27/06/2025, 16:34:57 |
  | Volume | 12 |
  | Pages | e0170707 |
  | Publication | PLOS ONE |
  | DOI | 10.1371/journal.pone.0170707 |
  | Issue | 2 |
  | Journal Abbr | PLoS ONE |
  | ISSN | 1932-6203 |
  | Date Added | 27/06/2025, 16:34:57 |
  | Modified | 27/06/2025, 16:34:57 |

  ### Attachments

  - Full Text
- ## Psychobiological factors affecting cortisol variability in human-dog dyads

  |  |  |
  | --- | --- |
  | Item Type | Journal Article |
  | Author | I. Schöberl |
  | Author | M. Wedl |
  | Author | A. Beetz |
  | Author | K. Kotrschal |
  | Abstract | Stress responses within dyads are modulated by interactions such as mutual emotional support and conflict. We investigated dyadic psychobiological factors influencing intra-individual cortisol variability in response to different challenging situations by testing 132 owners and their dogs in a laboratory setting. Salivary cortisol was measured and questionnaires were used to assess owner and dog personality as well as owners' social attitudes towards the dog and towards other humans. We calculated the individual coefficient of variance of cortisol (iCV = sd/mean∗100) over the different test situations as a parameter representing individual variability of cortisol concentration. We hypothesized that high cortisol variability indicates efficient and adaptive coping and a balanced individual and dyadic social performance. Female owners of male dogs had lower iCV than all other owner gender-dog sex combinations (F= 14.194, p<0.001), whereas owner Agreeableness (NEO-FFI) scaled positively with owner iCV (F= 4.981, p = 0.028). Dogs of owners high in Neuroticism (NEO-FFI) and of owners who were insecure-ambivalently attached to their dogs (FERT), had low iCV (F= 4.290, p = 0.041 and F= 5.948, p = 0.016), as had dogs of owners with human-directed separation anxiety (RSQ) or dogs of owners with a strong desire of independence (RSQ) (F= 7.661, p = 0.007 and F= 9.192, p = 0.003). We suggest that both owner and dog social characteristics influence dyadic cortisol variability, with the human partner being more influential than the dog. Our results support systemic approaches (i.e. considering the social context) in science and in counselling. |
  | Date | 2017 |
  | Language | English |
  | Archive | Embase |
  | URL | https://www.embase.com/search/results?subaction=viewrecord&id=L614348170&from=export |
  | Volume | 12 |
  | Publication | PLoS ONE |
  | DOI | 10.1371/journal.pone.0170707 |
  | Issue | 2 |
  | Journal Abbr | PLoS ONE |
  | ISSN | 1932-6203 |
  | Date Added | 11/07/2025, 14:18:38 |
  | Modified | 11/07/2025, 14:18:38 |

  ### Tags:

  - heart rate
  - separation anxiety
  - article
  - female
  - male
  - nonhuman
  - coping
  - human
  - hydrocortisone
  - adaptive behavior
  - adult
  - controlled study
  - emotional attachment
  - psychobiology
  - human-animal bond
  - saliva level
  - sex difference
  - ambivalence
  - aversive behavior
  - character
  - independence
  - insecurity
  - social attitude
  - threat

  ### Attachments

  - Full Text (HTML)
- ## Regular Positive Human Contacts Do Not Improve Pigs' Response to a Lipopolysaccharide Immune Challenge

  |  |  |
  | --- | --- |
  | Item Type | Journal Article |
  | Author | O. Schmitt |
  | Author | C. Knecht |
  | Author | B. Sobczak |
  | Author | H. Volkmann |
  | Author | U. Gimsa |
  | Author | J.-L. Rault |
  | Abstract | Introduction: Little is known about the effects of a positive human-animal relationship on animal health and resilience. This study investigated the effects of regular positive human-animal interactions on pigs response to an immune challenge. Methods: Twenty-four female pigs were recruited at weaning (5 weeks old), and siblings of similar weights were allocated to either the positive contact treatment with positive contacts given by a human to groups of 3 pigs in their home pen or the control treatment only exposed to a human standing immobile and silently in front and outside their home pen. Treatment sessions were applied over 9 consecutive weeks, lasted 10 min per group, and occurred twice daily (morning and afternoon), 3 days a week. At 16 weeks of age, pigs were submitted to an immune challenge, which consisted of a single intravenous administration of lipopolysaccharide (LPS; 2 μg/kg). The sickness behaviours of pigs were observed using scan sampling every 5 min over 6 h post-administration, recording somnolence, vomiting, diarrhoea, cramping, shivering, and panting. Blood samples were taken before the LPS administration, after 1 h and 3 h. Blood plasma was analysed to quantify tumour necrosis factor alpha, interleukins 6 and 10, immunoglobulin A, and cortisol concentrations, and blood serum was analysed to quantify a brain-derived neurotrophic factor. Behavioural and physiological data were statistically analysed using general linear models in R. Results: Both treatments showed signs of sickness behaviour following LPS administration, but the two treatments did not differ in the frequency, severity of sickness behaviours, or length of recovery or in the blood plasma concentration of cytokines and cortisol measured. Conclusion: Therefore, regular exposure to positive contacts with a human over several weeks, although leading to the development of a positive human-animal relationship, did not enhance the pigs response to this immune challenge or the immune parameters measured in this study. |
  | Date | 2025 |
  | Language | English |
  | Archive | Embase |
  | URL | https://www.embase.com/search/results?subaction=viewrecord&id=L2038436696&from=export |
  | Volume | 32 |
  | Pages | 83-93 |
  | Publication | NeuroImmunoModulation |
  | DOI | 10.1159/000544748 |
  | Issue | 1 |
  | Journal Abbr | NeuroImmunomodulation |
  | ISSN | 1423-0216 |
  | Date Added | 11/07/2025, 14:15:26 |
  | Modified | 11/07/2025, 14:15:26 |

  ### Tags:

  - human-animal interaction
  - animal experiment
  - article
  - female
  - nonhuman
  - hydrocortisone
  - controlled study
  - pig
  - immunoglobulin A
  - hydrocortisone blood level
  - disease severity
  - brain derived neurotrophic factor
  - convalescence
  - diarrhea
  - Escherichia coli lipopolysaccharide
  - illness behavior
  - immune response
  - immunocompetence
  - immunoglobulin blood level
  - interleukin 10
  - interleukin 6
  - muscle cramp
  - protein blood level
  - rectal temperature
  - shivering
  - somnolence
  - tumor necrosis factor
  - vomiting

  ### Attachments

  - Full Text (HTML)
- ## Regular Positive Human Contacts Do Not Improve Pigs' Response to a Lipopolysaccharide Immune Challenge

  |  |  |
  | --- | --- |
  | Item Type | Journal Article |
  | Author | O. Schmitt |
  | Author | C. Knecht |
  | Author | B. Sobczak |
  | Author | H. Volkmann |
  | Author | U. Gimsa |
  | Author | J.-L. Rault |
  | Abstract | Introduction: Little is known about the effects of a positive human-animal relationship on animal health and resilience. This study investigated the effects of regular positive human-animal interactions on pigs response to an immune challenge. Methods: Twenty-four female pigs were recruited at weaning (5 weeks old), and siblings of similar weights were allocated to either the positive contact treatment with positive contacts given by a human to groups of 3 pigs in their home pen or the control treatment only exposed to a human standing immobile and silently in front and outside their home pen. Treatment sessions were applied over 9 consecutive weeks, lasted 10 min per group, and occurred twice daily (morning and afternoon), 3 days a week. At 16 weeks of age, pigs were submitted to an immune challenge, which consisted of a single intravenous administration of lipopolysaccharide (LPS; 2 μg/kg). The sickness behaviours of pigs were observed using scan sampling every 5 min over 6 h post-administration, recording somnolence, vomiting, diarrhoea, cramping, shivering, and panting. Blood samples were taken before the LPS administration, after 1 h and 3 h. Blood plasma was analysed to quantify tumour necrosis factor alpha, interleukins 6 and 10, immunoglobulin A, and cortisol concentrations, and blood serum was analysed to quantify a brain-derived neurotrophic factor. Behavioural and physiological data were statistically analysed using general linear models in R. Results: Both treatments showed signs of sickness behaviour following LPS administration, but the two treatments did not differ in the frequency, severity of sickness behaviours, or length of recovery or in the blood plasma concentration of cytokines and cortisol measured. Conclusion: Therefore, regular exposure to positive contacts with a human over several weeks, although leading to the development of a positive human-animal relationship, did not enhance the pigs response to this immune challenge or the immune parameters measured in this study. |
  | Date | 2025 |
  | Language | English |
  | Archive | Embase |
  | URL | https://www.embase.com/search/results?subaction=viewrecord&id=L2038436696&from=export |
  | Volume | 32 |
  | Pages | 83-93 |
  | Publication | NeuroImmunoModulation |
  | DOI | 10.1159/000544748 |
  | Issue | 1 |
  | Journal Abbr | NeuroImmunomodulation |
  | ISSN | 1423-0216 |
  | Date Added | 11/07/2025, 14:16:37 |
  | Modified | 11/07/2025, 14:16:37 |

  ### Tags:

  - human-animal interaction
  - animal experiment
  - article
  - female
  - nonhuman
  - hydrocortisone
  - controlled study
  - pig
  - immunoglobulin A
  - hydrocortisone blood level
  - disease severity
  - brain derived neurotrophic factor
  - convalescence
  - diarrhea
  - Escherichia coli lipopolysaccharide
  - illness behavior
  - immune response
  - immunocompetence
  - immunoglobulin blood level
  - interleukin 10
  - interleukin 6
  - muscle cramp
  - protein blood level
  - rectal temperature
  - shivering
  - somnolence
  - tumor necrosis factor
  - vomiting

  ### Attachments

  - Full Text (HTML)
- ## Regular Positive Human Contacts Do Not Improve Pigs' Response to a Lipopolysaccharide Immune Challenge

  |  |  |
  | --- | --- |
  | Item Type | Journal Article |
  | Author | O. Schmitt |
  | Author | C. Knecht |
  | Author | B. Sobczak |
  | Author | H. Volkmann |
  | Author | U. Gimsa |
  | Author | J.-L. Rault |
  | Abstract | Introduction: Little is known about the effects of a positive human-animal relationship on animal health and resilience. This study investigated the effects of regular positive human-animal interactions on pigs response to an immune challenge. Methods: Twenty-four female pigs were recruited at weaning (5 weeks old), and siblings of similar weights were allocated to either the positive contact treatment with positive contacts given by a human to groups of 3 pigs in their home pen or the control treatment only exposed to a human standing immobile and silently in front and outside their home pen. Treatment sessions were applied over 9 consecutive weeks, lasted 10 min per group, and occurred twice daily (morning and afternoon), 3 days a week. At 16 weeks of age, pigs were submitted to an immune challenge, which consisted of a single intravenous administration of lipopolysaccharide (LPS; 2 μg/kg). The sickness behaviours of pigs were observed using scan sampling every 5 min over 6 h post-administration, recording somnolence, vomiting, diarrhoea, cramping, shivering, and panting. Blood samples were taken before the LPS administration, after 1 h and 3 h. Blood plasma was analysed to quantify tumour necrosis factor alpha, interleukins 6 and 10, immunoglobulin A, and cortisol concentrations, and blood serum was analysed to quantify a brain-derived neurotrophic factor. Behavioural and physiological data were statistically analysed using general linear models in R. Results: Both treatments showed signs of sickness behaviour following LPS administration, but the two treatments did not differ in the frequency, severity of sickness behaviours, or length of recovery or in the blood plasma concentration of cytokines and cortisol measured. Conclusion: Therefore, regular exposure to positive contacts with a human over several weeks, although leading to the development of a positive human-animal relationship, did not enhance the pigs response to this immune challenge or the immune parameters measured in this study. |
  | Date | 2025 |
  | Language | English |
  | Archive | Embase |
  | URL | https://www.embase.com/search/results?subaction=viewrecord&id=L2038436696&from=export |
  | Volume | 32 |
  | Pages | 83-93 |
  | Publication | NeuroImmunoModulation |
  | DOI | 10.1159/000544748 |
  | Issue | 1 |
  | Journal Abbr | NeuroImmunomodulation |
  | ISSN | 1423-0216 |
  | Date Added | 11/07/2025, 14:18:10 |
  | Modified | 11/07/2025, 14:18:10 |

  ### Tags:

  - human-animal interaction
  - animal experiment
  - article
  - female
  - nonhuman
  - hydrocortisone
  - controlled study
  - pig
  - immunoglobulin A
  - hydrocortisone blood level
  - disease severity
  - brain derived neurotrophic factor
  - convalescence
  - diarrhea
  - Escherichia coli lipopolysaccharide
  - illness behavior
  - immune response
  - immunocompetence
  - immunoglobulin blood level
  - interleukin 10
  - interleukin 6
  - muscle cramp
  - protein blood level
  - rectal temperature
  - shivering
  - somnolence
  - tumor necrosis factor
  - vomiting

  ### Attachments

  - Full Text (HTML)
- ## Changes in human health parameters associated with a touch tank experience at a zoological institution

  |  |  |
  | --- | --- |
  | Item Type | Journal Article |
  | Author | J.M. Sahrmann |
  | Author | A. Niedbalski |
  | Author | L. Bradshaw |
  | Author | R. Johnson |
  | Author | S.L. Deem |
  | Abstract | Association of Zoos and Aquariums (AZA) institutions provide a variety of benefits to visitors. However, one area that has received little study is the direct human health benefits from zoo and aquarium visits. With the increase in stress related non-infectious diseases in industrialized countries, understanding the extent of these benefits is important. We studied the effects on visitor stress of an experience at a touch tank exhibit featuring stingrays, sharks, and horseshoe crabs. Stress was measured by physiological and psychological parameters. Heart rate was recorded before, during, and after interacting with the animals, and mood was assessed before and after the experience using a psychological instrument. Multilevel models of heart rate show a quadratic trend, with heart rate elevated (b = -3.01, t = 26.4, P < 0.001) and less variable (b = 3.60, t = 15.9, P < 0.001) while touching the animals compared to before or after. Wilcoxon signed-rank tests on mood data suggest that most visitors felt happier (V = 174.5, P < 0.001), more energized (V = 743.5, P < 0.001), and less tense (V = 5618, P < 0.001) after the experience. This suggests that interacting with animals led to a physiological response during interactions reminiscent of a theme park experience along with a decrease in mental stress. The effects of confounding variables such as crowd size are also discussed. Further studies should be conducted to help deepen our understanding of the health benefits of experiences at AZA institutions. |
  | Date | 2016 |
  | Language | English |
  | Archive | Medline |
  | URL | https://www.embase.com/search/results?subaction=viewrecord&id=L612575093&from=export |
  | Volume | 35 |
  | Pages | 4-13 |
  | Publication | Zoo biology |
  | DOI | 10.1002/zoo.21257 |
  | Issue | 1 |
  | Journal Abbr | Zoo Biol. |
  | ISSN | 1098-2361 |
  | Date Added | 11/07/2025, 12:10:56 |
  | Modified | 11/07/2025, 12:10:56 |

  ### Tags:

  - blood pressure
  - physiology
  - Missouri
  - heart rate
  - female
  - male
  - physiological stress
  - zoo animal
  - human
  - adult
  - aged
  - questionnaire
  - animal
  - young adult
  - middle aged
  - happiness

  ### Attachments

  - Full Text (HTML)
- ## Physiological Indicators of Attachment in Domestic Dogs (Canis familiaris) and Their Owners in the Strange Situation Test

  |  |  |
  | --- | --- |
  | Item Type | Journal Article |
  | Author | M.G. Ryan |
  | Author | A.E. Storey |
  | Author | R.E. Anderson |
  | Author | C.J. Walsh |
  | Abstract | Behaviorally, attachment is demonstrated when one individual maintains close proximity to another individual and shows distress upon separation. For 29 owner-dog dyads, we employed a modified Ainsworth’s Strange Situation Test (SST) to investigate whether both members would show a physiological reaction to separation. Dogs experienced a series of separation from and reuniting events with their owners and were introduced to a stranger. Before and after the SST, saliva samples were taken from each dyad to measure stress-related analytes: cortisol (CORT) and chromogranin A (CgA). Dogs exhibited attachment behaviors toward owners as evidenced by more time spent in close proximity, more contact initiated and less time spent near the door, compared to episodes with the stranger. Dogs that initiated more contact with their owners in re-uniting episodes had lower CgA than dogs that initiated less contact, but their owners had higher CgA levels. Also during re-uniting episodes, dogs and owners spent more time near each other when owner CgA levels were low, owner CORT levels were high, and the dog had owner-reported separation anxiety. During the episodes alone with the stranger, dogs with higher CORT spent more time with the stranger. Finally, dogs’ initial CgA levels were correlated with their owner’s initial CORT levels, and dog final CORT levels were correlated with their owners’ final CORT levels, suggesting some hormonal synchrony within the dyad. As all owner-dog dyads were assessed as securely attached, attachment style differences could not explain variation in hormonal or behavioral results. These results suggest that dogs may respond to owner hormonal state and/or behavior and demonstrate that individual differences in responses to a behavioral challenge reflect the stress physiology of both dogs and their owners. |
  | Date | 2019 |
  | Language | English |
  | Archive | Embase |
  | URL | https://www.embase.com/search/results?subaction=viewrecord&id=L629171845&from=export |
  | Volume | 13 |
  | Publication | Frontiers in Behavioral Neuroscience |
  | DOI | 10.3389/fnbeh.2019.00162 |
  | Issue | (Ryan M.G., morag.g.ryan@mun.ca) Cognitive and Behavioural Ecology Program, Memorial University of Newfoundland, St. John’s, NL, Canada |
  | Journal Abbr | Front. Behav. Neurosci. |
  | ISSN | 1662-5153 |
  | Date Added | 11/07/2025, 14:18:33 |
  | Modified | 11/07/2025, 14:18:33 |

  ### Tags:

  - dog
  - separation anxiety
  - article
  - female
  - male
  - nonhuman
  - physiological stress
  - human
  - hydrocortisone
  - adult
  - aged
  - controlled study
  - human experiment
  - normal human
  - emotional attachment
  - human-animal bond
  - chromogranin A
  - saliva level
  - distress syndrome
  - psychologic test
  - strange situation test

  ### Attachments

  - Full Text (HTML)
- ## A Preliminary Assessment of Equine Affect in Equine-Assisted Services

  |  |  |
  | --- | --- |
  | Item Type | Journal Article |
  | Author | C. Rudd |
  | Author | E. Pasiuk |
  | Author | N. Anderson |
  | Author | N. Hall |
  | Author | R. Foster |
  | Author | K. Schroeder |
  | Abstract | Equine-Assisted Services (EAS) involve health services and adaptive recreation that include equine interactions to promote human wellbeing. While research around the human health outcomes of EAS has grown considerably, there has been limited exploration into the impact of such services on a key service provider, the horse. This study evaluated whether there is variation in how EAS horses experience different types of unmounted interactions with humans. To answer this question, we utilized a mixed method, repeated measures experiment in which 56 human participants and 14 horses, working at a 1:1 ratio, completed a series of three interactions common to EAS: a grooming, a leading, and a maze condition. To measure equine affect, we recorded heart rate variability (HRV) continuously, took eye temperature (ET) immediately after each interaction, and recorded horse behavior. A mixed effects model indicated that horses displayed greater sympathetic activation in the grooming condition than the leading via Low Frequency/High Frequency Ratio (LF:HF: p < 0.001; ET: p = 0.003) and maze conditions (LF:HF: p = 0.005; ET: p < 0.001). Horses also showed significantly more stress-associated behaviors, such as elevated head carriage and pawing, in the grooming condition. These results indicate that there is potential for equine stress during a grooming activity; therefore, more research is needed to determine optimal approaches for addressing horse welfare in unmounted EAS activities involving human interaction. |
  | Date | 2024 |
  | Language | English |
  | Archive | Embase |
  | URL | https://www.embase.com/search/results?subaction=viewrecord&id=L2029331319&from=export |
  | Volume | 37 |
  | Pages | 501-518 |
  | Publication | Anthrozoos |
  | DOI | 10.1080/08927936.2024.2333163 |
  | Issue | 3 |
  | Journal Abbr | Anthrozoos |
  | ISSN | 1753-0377 |
  | Date Added | 11/07/2025, 11:53:21 |
  | Modified | 11/07/2025, 11:53:21 |

  ### Tags:

  - human-animal interaction
  - horse
  - heart rate variability
  - article
  - female
  - male
  - nonhuman
  - physiological stress
  - human
  - grooming
  - human-animal bond
  - temperature
  - Equus
  - recreation

  ### Attachments

  - Full Text (HTML)
- ## A Preliminary Assessment of Equine Affect in Equine-Assisted Services

  |  |  |
  | --- | --- |
  | Item Type | Journal Article |
  | Author | C. Rudd |
  | Author | E. Pasiuk |
  | Author | N. Anderson |
  | Author | N. Hall |
  | Author | R. Foster |
  | Author | K. Schroeder |
  | Abstract | Equine-Assisted Services (EAS) involve health services and adaptive recreation that include equine interactions to promote human wellbeing. While research around the human health outcomes of EAS has grown considerably, there has been limited exploration into the impact of such services on a key service provider, the horse. This study evaluated whether there is variation in how EAS horses experience different types of unmounted interactions with humans. To answer this question, we utilized a mixed method, repeated measures experiment in which 56 human participants and 14 horses, working at a 1:1 ratio, completed a series of three interactions common to EAS: a grooming, a leading, and a maze condition. To measure equine affect, we recorded heart rate variability (HRV) continuously, took eye temperature (ET) immediately after each interaction, and recorded horse behavior. A mixed effects model indicated that horses displayed greater sympathetic activation in the grooming condition than the leading via Low Frequency/High Frequency Ratio (LF:HF: p < 0.001; ET: p = 0.003) and maze conditions (LF:HF: p = 0.005; ET: p < 0.001). Horses also showed significantly more stress-associated behaviors, such as elevated head carriage and pawing, in the grooming condition. These results indicate that there is potential for equine stress during a grooming activity; therefore, more research is needed to determine optimal approaches for addressing horse welfare in unmounted EAS activities involving human interaction. |
  | Date | 2024 |
  | Language | English |
  | Archive | Embase |
  | URL | https://www.embase.com/search/results?subaction=viewrecord&id=L2029331319&from=export |
  | Volume | 37 |
  | Pages | 501-518 |
  | Publication | Anthrozoos |
  | DOI | 10.1080/08927936.2024.2333163 |
  | Issue | 3 |
  | Journal Abbr | Anthrozoos |
  | ISSN | 1753-0377 |
  | Date Added | 11/07/2025, 12:10:43 |
  | Modified | 11/07/2025, 12:10:43 |

  ### Tags:

  - human-animal interaction
  - horse
  - heart rate variability
  - article
  - female
  - male
  - nonhuman
  - physiological stress
  - human
  - grooming
  - human-animal bond
  - temperature
  - Equus
  - recreation

  ### Attachments

  - Full Text (HTML)
- ## Animal-based measurements to assess the welfare of dairy cull cows during pre-slaughter

  |  |  |
  | --- | --- |
  | Item Type | Journal Article |
  | Author | M.H. Romero |
  | Author | M. Rodríguez-Palomares |
  | Author | J.A. Sánchez |
  | Abstract | Culling is the departure of cows from the herd as a result of sale, slaughter, health, national regulations, salvage, or death. Cull cows are removed from farms with poor health, production, behavior, or other problems, and during pre-slaughter they are sometimes kept without food and water, which compromises their well-being. The objective of the present study was to evaluate the welfare state of culled dairy cows during pre-slaughter using some animal-based measurements and to identify possible associations between them. Data were recorded for 62 different dairy production farms referring to 137 cull cows (n = 60 Holstein and n = 77 Normandy crosses) slaughtered in an abattoir in Colombia (South America). In this study, we evaluated and recorded land transport conditions, the health of animals on arrival to the abattoir, human–animal interaction, stress physiological variables and the association of these variables with characteristic bruises on the carcass, the lairage time, the presence of diseases, and the stage of pregnancy. In total, 98.5% of the cows were very thin, 35.7% were pregnant, and 84.7% had bruising on the carcass. In total, 74.5% had clinical conditions; these included skin lesions (32.4%), mastitis (27.5%), lameness (21.6%), vulvar secretions (8.8%), diarrhea (6.8%), and eye carcinoma (2.9%). The total number of cull cows with bruises during pre-slaughter was associated with lot size, transport time, presence of pregnancy, body score condition, and creatine kinase levels. The results suggest that the cows were not fit for transport because their health was severely affected before they left the farms. The animal-based indicators used in this study are useful for evaluating the welfare of cull dairy cows at abattoirs. |
  | Date | 2020 |
  | Language | English |
  | Archive | Embase |
  | URL | https://www.embase.com/search/results?subaction=viewrecord&id=L2005167009&from=export |
  | Volume | 10 |
  | Pages | 1-19 |
  | Publication | Animals |
  | DOI | 10.3390/ani10101802 |
  | Issue | 10 |
  | Journal Abbr | Animals |
  | ISSN | 2076-2615 |
  | Date Added | 11/07/2025, 14:16:55 |
  | Modified | 11/07/2025, 14:16:55 |

  ### Tags:

  - human-animal interaction
  - animal welfare
  - article
  - nonhuman
  - physiological stress
  - hydrocortisone
  - body weight
  - scoring system
  - creatinine
  - hemoglobin
  - dairy cattle
  - slaughtering
  - albumin
  - glucose
  - urea
  - aggression
  - creatine kinase
  - neutrophil lymphocyte ratio
  - skin defect
  - stocking density
  - diarrhea
  - pregnancy
  - 3 hydroxybutyric acid
  - animal culling
  - animal lameness
  - body condition score
  - carcass
  - Colombia
  - delaty time
  - eye cancer
  - hematocrit
  - lairage time
  - lot size
  - mastitis
  - physical parameters
  - slaughterhouse
  - spectrophotometer
  - traffic and transport
  - transport time
  - unloading time

  ### Attachments

  - Full Text (HTML)
- ## Animal-based measurements to assess the welfare of dairy cull cows during pre-slaughter

  |  |  |
  | --- | --- |
  | Item Type | Journal Article |
  | Author | M.H. Romero |
  | Author | M. Rodríguez-Palomares |
  | Author | J.A. Sánchez |
  | Abstract | Culling is the departure of cows from the herd as a result of sale, slaughter, health, national regulations, salvage, or death. Cull cows are removed from farms with poor health, production, behavior, or other problems, and during pre-slaughter they are sometimes kept without food and water, which compromises their well-being. The objective of the present study was to evaluate the welfare state of culled dairy cows during pre-slaughter using some animal-based measurements and to identify possible associations between them. Data were recorded for 62 different dairy production farms referring to 137 cull cows (n = 60 Holstein and n = 77 Normandy crosses) slaughtered in an abattoir in Colombia (South America). In this study, we evaluated and recorded land transport conditions, the health of animals on arrival to the abattoir, human–animal interaction, stress physiological variables and the association of these variables with characteristic bruises on the carcass, the lairage time, the presence of diseases, and the stage of pregnancy. In total, 98.5% of the cows were very thin, 35.7% were pregnant, and 84.7% had bruising on the carcass. In total, 74.5% had clinical conditions; these included skin lesions (32.4%), mastitis (27.5%), lameness (21.6%), vulvar secretions (8.8%), diarrhea (6.8%), and eye carcinoma (2.9%). The total number of cull cows with bruises during pre-slaughter was associated with lot size, transport time, presence of pregnancy, body score condition, and creatine kinase levels. The results suggest that the cows were not fit for transport because their health was severely affected before they left the farms. The animal-based indicators used in this study are useful for evaluating the welfare of cull dairy cows at abattoirs. |
  | Date | 2020 |
  | Language | English |
  | Archive | Embase |
  | URL | https://www.embase.com/search/results?subaction=viewrecord&id=L2005167009&from=export |
  | Volume | 10 |
  | Pages | 1-19 |
  | Publication | Animals |
  | DOI | 10.3390/ani10101802 |
  | Issue | 10 |
  | Journal Abbr | Animals |
  | ISSN | 2076-2615 |
  | Date Added | 11/07/2025, 14:18:30 |
  | Modified | 11/07/2025, 14:18:30 |

  ### Tags:

  - human-animal interaction
  - animal welfare
  - article
  - nonhuman
  - physiological stress
  - hydrocortisone
  - body weight
  - scoring system
  - creatinine
  - hemoglobin
  - dairy cattle
  - slaughtering
  - albumin
  - glucose
  - urea
  - aggression
  - creatine kinase
  - neutrophil lymphocyte ratio
  - skin defect
  - stocking density
  - diarrhea
  - pregnancy
  - 3 hydroxybutyric acid
  - animal culling
  - animal lameness
  - body condition score
  - carcass
  - Colombia
  - delaty time
  - eye cancer
  - hematocrit
  - lairage time
  - lot size
  - mastitis
  - physical parameters
  - slaughterhouse
  - spectrophotometer
  - traffic and transport
  - transport time
  - unloading time

  ### Attachments

  - Full Text (HTML)
- ## The Effect of Mental Activation of One's Pet Dog on Stress Reactivity

  |  |  |
  | --- | --- |
  | Item Type | Journal Article |
  | Author | K.E. Rodriguez |
  | Author | D.J. Graham |
  | Author | R.G. Lucas-Thompson |
  | Abstract | Research suggests that mental activation of human social support may reduce stress reactivity. However, the extent to which social support from pets elicits a similar effect has been less explored. This study aims to determine whether the mental activation of one's pet dog reduces stress reactivity to a subsequent experimental stressor. In a 2 × 2 design, 132 dog-owning participants (Mage = 20.14; 80% female) were randomly assigned to one of two mental activation conditions (pet dog; general) and one of two stressor conditions (social-evaluative; cognitive). Data were analyzed with two-way ANOVAs with self-reported (positive/negative affect, negative self-evaluation) and physiological (blood pressure, heart rate) dependent variables. Results indicated that participants randomized to the pet dog mental activation condition had smaller decreases in positive affect from baseline to post-stressor compared to the general mental activation condition. However, there were no significant interactions between time and mental activation condition on negative affect, negative self-evaluation, heart rate, or blood pressure. Thus, the mental activation of one's pet dog had a minimal effect on stress reactivity to a cognitive or social-evaluative stressor. Results suggest that the physical presence of an animal may be an essential mechanism underlying the benefits of animal-derived social support. |
  | Date | 2023 |
  | Language | English |
  | Archive | Medline |
  | URL | https://www.embase.com/search/results?subaction=viewrecord&id=L642715953&from=export |
  | Volume | 20 |
  | Publication | International journal of environmental research and public health |
  | DOI | 10.3390/ijerph20216995 |
  | Issue | 21 |
  | Journal Abbr | Int J Environ Res Public Health |
  | ISSN | 1660-4601 |
  | Date Added | 11/07/2025, 11:53:21 |
  | Modified | 11/07/2025, 11:53:21 |

  ### Tags:

  - blood pressure
  - social support
  - physiology
  - heart rate
  - dog
  - female
  - male
  - human
  - self report
  - adult
  - controlled study
  - randomized controlled trial
  - questionnaire
  - animal
  - mental stress
  - psychology
  - young adult

  ### Attachments

  - Full Text (HTML)
- ## The Effect of Mental Activation of One's Pet Dog on Stress Reactivity

  |  |  |
  | --- | --- |
  | Item Type | Journal Article |
  | Author | K.E. Rodriguez |
  | Author | D.J. Graham |
  | Author | R.G. Lucas-Thompson |
  | Abstract | Research suggests that mental activation of human social support may reduce stress reactivity. However, the extent to which social support from pets elicits a similar effect has been less explored. This study aims to determine whether the mental activation of one's pet dog reduces stress reactivity to a subsequent experimental stressor. In a 2 × 2 design, 132 dog-owning participants (Mage = 20.14; 80% female) were randomly assigned to one of two mental activation conditions (pet dog; general) and one of two stressor conditions (social-evaluative; cognitive). Data were analyzed with two-way ANOVAs with self-reported (positive/negative affect, negative self-evaluation) and physiological (blood pressure, heart rate) dependent variables. Results indicated that participants randomized to the pet dog mental activation condition had smaller decreases in positive affect from baseline to post-stressor compared to the general mental activation condition. However, there were no significant interactions between time and mental activation condition on negative affect, negative self-evaluation, heart rate, or blood pressure. Thus, the mental activation of one's pet dog had a minimal effect on stress reactivity to a cognitive or social-evaluative stressor. Results suggest that the physical presence of an animal may be an essential mechanism underlying the benefits of animal-derived social support. |
  | Date | 2023 |
  | Language | English |
  | Archive | Medline |
  | URL | https://www.embase.com/search/results?subaction=viewrecord&id=L642715953&from=export |
  | Volume | 20 |
  | Publication | International journal of environmental research and public health |
  | DOI | 10.3390/ijerph20216995 |
  | Issue | 21 |
  | Journal Abbr | Int J Environ Res Public Health |
  | ISSN | 1660-4601 |
  | Date Added | 11/07/2025, 12:10:44 |
  | Modified | 11/07/2025, 12:10:44 |

  ### Tags:

  - blood pressure
  - social support
  - physiology
  - heart rate
  - dog
  - female
  - male
  - human
  - self report
  - adult
  - controlled study
  - randomized controlled trial
  - questionnaire
  - animal
  - mental stress
  - psychology
  - young adult

  ### Attachments

  - Full Text (HTML)
- ## The effect of a service dog on salivary cortisol awakening response in a military population with posttraumatic stress disorder (PTSD)

  |  |  |
  | --- | --- |
  | Item Type | Journal Article |
  | Author | K.E. Rodriguez |
  | Author | C.I. Bryce |
  | Author | D.A. Granger |
  | Author | M.E. O'Haire |
  | Abstract | Recent studies suggest a therapeutic effect of psychiatric service dogs for military veterans with posttraumatic stress disorder (PTSD), but are limited by self-report biases. The current study assessed the effect of PTSD service dogs on the salivary cortisol awakening response (CAR) and arousal-related functioning in a population of military veterans with PTSD. Participants included 73 post-9/11 military veterans with PTSD including 45 with a service dog and 28 on the waitlist to receive one. Saliva samples were collected on two consecutive weekday mornings at awakening and 30 min later to quantify the cortisol awakening response (CAR) and its area under the curve (AUCi) in addition to standardized survey measures of anxiety, anger, sleep quality and disturbance, and alcohol abuse. There was a significant main effect of having a service dog on both the CAR and the AUCi, with individuals with a service dog exhibiting a higher CAR and AUCi compared to those on the waitlist. Results also revealed that those with a service dog reported significantly lower anxiety, anger, and sleep disturbance as well as less alcohol abuse compared to those on the waitlist, with medium to large effect sizes. Although those with a service dog reported significantly less PTSD symptom severity, CAR was not significantly associated with PTSD symptoms within or across group. In conclusion, results indicate that the placement of a PTSD service dog may have a significant positive influence on both physiological and psychosocial indicators of wellbeing in military veterans with PTSD. Although clinical significance cannot be confirmed, a higher CAR/AUCi among those with a service dog may indicate better health and wellbeing in this population. Future within-subject, longitudinal research will be necessary to determine potential clinical significance and impact of individual differences. |
  | Date | 2018 |
  | Language | English |
  | Archive | Embase |
  | URL | https://www.embase.com/search/results?subaction=viewrecord&id=L2001262833&from=export |
  | Volume | 98 |
  | Pages | 202-210 |
  | Publication | Psychoneuroendocrinology |
  | DOI | 10.1016/j.psyneuen.2018.04.026 |
  | Issue | (Rodriguez K.E.; O'Haire M.E., mohaire@purdue.edu) Center for the Human-Animal Bond, Department of Comparative Pathobiology, College of Veterinary Medicine, Purdue University, West Lafayette, United States |
  | Journal Abbr | Psychoneuroendocrinology |
  | ISSN | 1873-3360 |
  | Date Added | 11/07/2025, 14:18:35 |
  | Modified | 11/07/2025, 14:18:35 |

  ### Tags:

  - veteran
  - anxiety
  - article
  - female
  - male
  - human
  - adult
  - controlled study
  - posttraumatic stress disorder
  - major clinical study
  - psychological well-being
  - service dog
  - therapy effect
  - arousal
  - army
  - disease severity
  - priority journal
  - saliva analysis
  - anger
  - cross-sectional study
  - alcohol abuse
  - cortisol awakening response
  - disease association
  - hospital admission
  - hydrocortisone release
  - salivary gland function
  - sleep disorder
  - sleep quality
  - social psychology
  - standardization

  ### Attachments

  - Full Text (HTML)
- ## Physiological Indicators of Acute and Chronic Stress in Securely and Insecurely Attached Dogs Undergoing a Strange Situation Procedure (SSP): Preliminary Results

  |  |  |
  | --- | --- |
  | Item Type | Journal Article |
  | Author | G. Riggio |
  | Author | C. Borrelli |
  | Author | M. Campera |
  | Author | A. Gazzano |
  | Author | C. Mariti |
  | Abstract | The quality of the attachment bond towards the caregiver may affect the dog’s physiological responses to stressful stimuli. This study aimed to measure chronic and acute physiological parameters of stress in ten securely and ten insecurely attached dogs. The twenty experimental subjects were selected from a sample of dogs that participated with their owners in the Strange Situation Procedure. Saliva samples were collected before (T0) and after (T1) the test. Blood pressure, heart rate, respiratory rate, and rectal temperature were measured after the test, only. At this time, a hair sample was also collected. RM ANOVA was used to analyse cortisol concentrations between secure and insecure dogs at T0 and T1. Mann–Whitney U test or T test were used for other physiological parameters. Insecure dogs had significant higher salivary cortisol concentrations than secure dogs at T1 (p = 0.024), but only a non-significant trend towards higher cortisol concentrations at T0 (p = 0.099). Post-test heart rate also tended to be higher in insecure compared to secure dogs (p = 0.077). No significant differences in hair cortisol concentration were found. The quality of attachment may affect the dog’s physiological response to acute stress, at least when related to separation from the caregiver. The effect of attachment on chronic stress requires further investigation. |
  | Date | 2022 |
  | Language | English |
  | Archive | Embase |
  | URL | https://www.embase.com/search/results?subaction=viewrecord&id=L2019762428&from=export |
  | Volume | 9 |
  | Publication | Veterinary Sciences |
  | DOI | 10.3390/vetsci9100519 |
  | Issue | 10 |
  | Journal Abbr | Vet. Sci. |
  | ISSN | 2306-7381 |
  | Date Added | 11/07/2025, 14:15:37 |
  | Modified | 11/07/2025, 14:15:37 |

  ### Tags:

  - dog
  - animal behavior
  - article
  - female
  - male
  - nonhuman
  - human
  - hydrocortisone
  - adult
  - controlled study
  - pilot study
  - emotional attachment
  - behavior assessment
  - caregiver
  - breathing rate
  - young adult
  - human-animal bond
  - middle aged
  - human-animal relation
  - analysis of variance
  - rectal temperature
  - chronic stress
  - acute stress
  - animal hair
  - blood pressure measurement
  - comparative physiology
  - heart rate measurement
  - physiological feedback
  - rank sum test
  - salivary excretion
  - strange situation procedure
  - Student t test

  ### Attachments

  - Full Text (HTML)
- ## Physiological Indicators of Acute and Chronic Stress in Securely and Insecurely Attached Dogs Undergoing a Strange Situation Procedure (SSP): Preliminary Results

  |  |  |
  | --- | --- |
  | Item Type | Journal Article |
  | Author | G. Riggio |
  | Author | C. Borrelli |
  | Author | M. Campera |
  | Author | A. Gazzano |
  | Author | C. Mariti |
  | Abstract | The quality of the attachment bond towards the caregiver may affect the dog’s physiological responses to stressful stimuli. This study aimed to measure chronic and acute physiological parameters of stress in ten securely and ten insecurely attached dogs. The twenty experimental subjects were selected from a sample of dogs that participated with their owners in the Strange Situation Procedure. Saliva samples were collected before (T0) and after (T1) the test. Blood pressure, heart rate, respiratory rate, and rectal temperature were measured after the test, only. At this time, a hair sample was also collected. RM ANOVA was used to analyse cortisol concentrations between secure and insecure dogs at T0 and T1. Mann–Whitney U test or T test were used for other physiological parameters. Insecure dogs had significant higher salivary cortisol concentrations than secure dogs at T1 (p = 0.024), but only a non-significant trend towards higher cortisol concentrations at T0 (p = 0.099). Post-test heart rate also tended to be higher in insecure compared to secure dogs (p = 0.077). No significant differences in hair cortisol concentration were found. The quality of attachment may affect the dog’s physiological response to acute stress, at least when related to separation from the caregiver. The effect of attachment on chronic stress requires further investigation. |
  | Date | 2022 |
  | Language | English |
  | Archive | Embase |
  | URL | https://www.embase.com/search/results?subaction=viewrecord&id=L2019762428&from=export |
  | Volume | 9 |
  | Publication | Veterinary Sciences |
  | DOI | 10.3390/vetsci9100519 |
  | Issue | 10 |
  | Journal Abbr | Vet. Sci. |
  | ISSN | 2306-7381 |
  | Date Added | 11/07/2025, 14:16:47 |
  | Modified | 11/07/2025, 14:16:47 |

  ### Tags:

  - dog
  - animal behavior
  - article
  - female
  - male
  - nonhuman
  - human
  - hydrocortisone
  - adult
  - controlled study
  - pilot study
  - emotional attachment
  - behavior assessment
  - caregiver
  - breathing rate
  - young adult
  - human-animal bond
  - middle aged
  - human-animal relation
  - analysis of variance
  - rectal temperature
  - chronic stress
  - acute stress
  - animal hair
  - blood pressure measurement
  - comparative physiology
  - heart rate measurement
  - physiological feedback
  - rank sum test
  - salivary excretion
  - strange situation procedure
  - Student t test

  ### Attachments

  - Full Text (HTML)
- ## Physiological Indicators of Acute and Chronic Stress in Securely and Insecurely Attached Dogs Undergoing a Strange Situation Procedure (SSP): Preliminary Results

  |  |  |
  | --- | --- |
  | Item Type | Journal Article |
  | Author | G. Riggio |
  | Author | C. Borrelli |
  | Author | M. Campera |
  | Author | A. Gazzano |
  | Author | C. Mariti |
  | Abstract | The quality of the attachment bond towards the caregiver may affect the dog’s physiological responses to stressful stimuli. This study aimed to measure chronic and acute physiological parameters of stress in ten securely and ten insecurely attached dogs. The twenty experimental subjects were selected from a sample of dogs that participated with their owners in the Strange Situation Procedure. Saliva samples were collected before (T0) and after (T1) the test. Blood pressure, heart rate, respiratory rate, and rectal temperature were measured after the test, only. At this time, a hair sample was also collected. RM ANOVA was used to analyse cortisol concentrations between secure and insecure dogs at T0 and T1. Mann–Whitney U test or T test were used for other physiological parameters. Insecure dogs had significant higher salivary cortisol concentrations than secure dogs at T1 (p = 0.024), but only a non-significant trend towards higher cortisol concentrations at T0 (p = 0.099). Post-test heart rate also tended to be higher in insecure compared to secure dogs (p = 0.077). No significant differences in hair cortisol concentration were found. The quality of attachment may affect the dog’s physiological response to acute stress, at least when related to separation from the caregiver. The effect of attachment on chronic stress requires further investigation. |
  | Date | 2022 |
  | Language | English |
  | Archive | Embase |
  | URL | https://www.embase.com/search/results?subaction=viewrecord&id=L2019762428&from=export |
  | Volume | 9 |
  | Publication | Veterinary Sciences |
  | DOI | 10.3390/vetsci9100519 |
  | Issue | 10 |
  | Journal Abbr | Vet. Sci. |
  | ISSN | 2306-7381 |
  | Date Added | 11/07/2025, 14:18:20 |
  | Modified | 11/07/2025, 14:18:20 |

  ### Tags:

  - dog
  - animal behavior
  - article
  - female
  - male
  - nonhuman
  - human
  - hydrocortisone
  - adult
  - controlled study
  - pilot study
  - emotional attachment
  - behavior assessment
  - caregiver
  - breathing rate
  - young adult
  - human-animal bond
  - middle aged
  - human-animal relation
  - analysis of variance
  - rectal temperature
  - chronic stress
  - acute stress
  - animal hair
  - blood pressure measurement
  - comparative physiology
  - heart rate measurement
  - physiological feedback
  - rank sum test
  - salivary excretion
  - strange situation procedure
  - Student t test

  ### Attachments

  - Full Text (HTML)
- ## Characterizing stress during animal interaction: a focus on the human endocrine response during equine-assisted services

  |  |  |
  | --- | --- |
  | Item Type | Journal Article |
  | Author | B.R. Rigby |
  | Abstract | Repeated stresses applied to the rider may contribute to the documented physical and psychosocial outcomes from equine-assisted services. In this brief review, a summary of neuroendocrine markers of stress, including immunoglobulin A, serotonin, cortisol, progesterone, and oxytocin, is presented within the context of the physiology of stress modulation. Results are mixed with regard to the effects of these hormones on rider physiology before, during, and after equine-assisted services. However, some results from existing studies are promising with regard to the attenuation of stress. Future research should include a cross-disciplinary approach when conducting well-controlled studies with proper treatment and experimental fidelity, while also considering exogenous and endogenous factors that influence rider physiology. |
  | Date | 2023 |
  | Language | English |
  | Archive | Embase |
  | URL | https://www.embase.com/search/results?subaction=viewrecord&id=L2027693642&from=export |
  | Volume | 10 |
  | Publication | Frontiers in Veterinary Science |
  | DOI | 10.3389/fvets.2023.1303354 |
  | Issue | (Rigby B.R., brigby@twu.edu) Exercise Physiology Laboratory, School of Health Promotion and Kinesiology, Texas Woman's University, Denton, TX, United States |
  | Journal Abbr | Front. Vet. Sci. |
  | ISSN | 2297-1769 |
  | Date Added | 11/07/2025, 12:12:19 |
  | Modified | 11/07/2025, 12:12:19 |

  ### Tags:

  - oxytocin
  - human-animal interaction
  - horse
  - nonhuman
  - physiological stress
  - human
  - hydrocortisone
  - hippotherapy
  - immunoglobulin A
  - hormone release
  - modulation
  - progesterone
  - serotonin
  - short survey

  ### Attachments

  - Full Text (HTML)
- ## Characterizing stress during animal interaction: a focus on the human endocrine response during equine-assisted services

  |  |  |
  | --- | --- |
  | Item Type | Journal Article |
  | Author | B.R. Rigby |
  | Abstract | Repeated stresses applied to the rider may contribute to the documented physical and psychosocial outcomes from equine-assisted services. In this brief review, a summary of neuroendocrine markers of stress, including immunoglobulin A, serotonin, cortisol, progesterone, and oxytocin, is presented within the context of the physiology of stress modulation. Results are mixed with regard to the effects of these hormones on rider physiology before, during, and after equine-assisted services. However, some results from existing studies are promising with regard to the attenuation of stress. Future research should include a cross-disciplinary approach when conducting well-controlled studies with proper treatment and experimental fidelity, while also considering exogenous and endogenous factors that influence rider physiology. |
  | Date | 2023 |
  | Language | English |
  | Archive | Embase |
  | URL | https://www.embase.com/search/results?subaction=viewrecord&id=L2027693642&from=export |
  | Volume | 10 |
  | Publication | Frontiers in Veterinary Science |
  | DOI | 10.3389/fvets.2023.1303354 |
  | Issue | (Rigby B.R., brigby@twu.edu) Exercise Physiology Laboratory, School of Health Promotion and Kinesiology, Texas Woman's University, Denton, TX, United States |
  | Journal Abbr | Front. Vet. Sci. |
  | ISSN | 2297-1769 |
  | Date Added | 11/07/2025, 12:13:21 |
  | Modified | 11/07/2025, 12:13:21 |

  ### Tags:

  - oxytocin
  - human-animal interaction
  - horse
  - nonhuman
  - physiological stress
  - human
  - hydrocortisone
  - hippotherapy
  - immunoglobulin A
  - hormone release
  - modulation
  - progesterone
  - serotonin
  - short survey

  ### Attachments

  - Full Text (HTML)
- ## Relationship between hyponeophagia and adrenal cortex function in farmed foxes

  |  |  |
  | --- | --- |
  | Item Type | Journal Article |
  | Author | T. Rekilä |
  | Author | M. Harri |
  | Author | L. Jalkanen |
  | Author | J. Mononen |
  | Abstract | The adrenal cortex function of farmed blue (Alopex lagopus) and silver foxes (Vulpes vulpes) differing in their reaction in the feeding test were assessed. The urine cortisol:creatinine ratio was lower for those animals eating in the feeding test in comparison to those not eating in both species. In addition, eater silver foxes had lower baseline serum cortisol concentration and also lower serum cortisol concentration 2 h after ACTH administration than noneaters. There were no differences in any serum cortisol levels between the eater and noneater blue foxes. The weights of body and adrenals did not differ between confident and fearful animals in either species. The present study demonstrates that animals not eating in the feeding test may have higher fearfulness and be more stressed than animals eating. |
  | Date | 1998 |
  | Language | English |
  | Archive | Embase |
  | URL | https://www.embase.com/search/results?subaction=viewrecord&id=L29094715&from=export |
  | Volume | 65 |
  | Pages | 779-783 |
  | Publication | Physiology and Behavior |
  | DOI | 10.1016/S0031-9384(98)00232-7 |
  | Issue | 4-5 |
  | Journal Abbr | Physiol. Behav. |
  | ISSN | 0031-9384 |
  | Date Added | 11/07/2025, 14:18:44 |
  | Modified | 11/07/2025, 14:18:44 |

  ### Tags:

  - animal behavior
  - animal experiment
  - article
  - female
  - male
  - nonhuman
  - hydrocortisone
  - body weight
  - controlled study
  - corticotropin
  - hydrocortisone urine level
  - animal cell
  - animal tissue
  - fear
  - feeding behavior
  - hydrocortisone blood level
  - priority journal
  - fox
  - statistical analysis
  - adrenal cortex function
  - adrenal gland
  - creatine
  - creatinine urine level
  - emotional stress
  - organ weight

  ### Attachments

  - Full Text (HTML)
- ## Validation of the feeding test as an index of fear in farmed blue (Alopex lagopus) and silver foxes (Vulpes vulpes)

  |  |  |
  | --- | --- |
  | Item Type | Journal Article |
  | Author | T. Rekilä |
  | Author | M. Harri |
  | Author | L. Ahola |
  | Abstract | The reliability and validity of the eating behaviour in the presence of man (Feeding test) as an index of fear were assessed in farmed blue (Alopex lagopus) and silver foxes (Vulpes vulpes). Repeatability of the Feeding test was good in both species. No further habituation occurred after the fourth successive test in either species. In addition, the behaviour of both species was independent of the person who performed the test. The normal feeding interval, i.e., 24 h, between feed deliveries, was long enough to provide reliable results. The presence of a cage mate did not influence the blue foxes' response in the Feeding test. A significant relationship between the results of the Feeding test and the Tit-bit test in both species and between the Feeding test and the fearfulness score in silver foxes indicate that all these tests measure similar features, most probably foxes' fear of humans. Those silver foxes that did not eat in the Feeding test had higher base levels of cortisol than the animals that did eat, providing further support for the above conclusion. The present study demonstrates that the Feeding test is a reliable, i.e., repeatable and free of random errors, and fairly valid fear test for blue and silver foxes. The Feeding test seems likely to give good results in measuring fear in farmed blue and silver foxes, but further investigations will be needed to fully validate it, especially for blue foxes. |
  | Date | 1997 |
  | Language | English |
  | Archive | Embase |
  | URL | https://www.embase.com/search/results?subaction=viewrecord&id=L27412638&from=export |
  | Volume | 62 |
  | Pages | 805-810 |
  | Publication | Physiology and Behavior |
  | DOI | 10.1016/S0031-9384(97)00241-2 |
  | Issue | 4 |
  | Journal Abbr | PHYSIOL. BEHAV. |
  | ISSN | 0031-9384 |
  | Date Added | 11/07/2025, 14:18:44 |
  | Modified | 11/07/2025, 14:18:44 |

  ### Tags:

  - behavior
  - animal experiment
  - article
  - nonhuman
  - controlled study
  - reliability
  - fear
  - habituation
  - priority journal
  - fox
  - procedures

  ### Attachments

  - Full Text (HTML)
- ## Mechanisms of Social Attachment Between Children and Pet Dogs

  |  |  |
  | --- | --- |
  | Item Type | Journal Article |
  | Author | O.T. Reilly |
  | Author | L.H. Somerville |
  | Author | E.E. Hecht |
  | Abstract | An increasing body of evidence indicates that owning a pet dog is associated with improvements in child health and well-being. Importantly, the degree of the social bond between child and dog may mediate the beneficial outcomes of dog ownership. The formation of social bonds is an intrinsically dyadic, interactive process where each interactor’s behavior influences the other’s behavior. For this reason, it is critical to evaluate the biological mechanisms of attachment in both children and their pet dogs as a socially bonded pair. Here, we review the physical, mental, and emotional outcomes that are associated with pet dog ownership or interaction in children. We then discuss the evidence that suggests that the strength of a social bond between a child and their pet dog matters for maximizing the beneficial outcomes associated with pet dog ownership, such as possible stress-buffering effects. We review the existing literature on the neural and endocrinological mechanisms of social attachment for inter-species social bonds that form between human children and dogs, situating this emerging knowledge within the context of the mechanisms of intra-species bonds in mammals. Finally, we highlight the remaining open questions and point toward directions for future research. |
  | Date | 2024 |
  | Language | English |
  | Archive | Embase |
  | URL | https://www.embase.com/search/results?subaction=viewrecord&id=L2031978526&from=export |
  | Volume | 14 |
  | Publication | Animals |
  | DOI | 10.3390/ani14203036 |
  | Issue | 20 |
  | Journal Abbr | Animals |
  | ISSN | 2076-2615 |
  | Date Added | 11/07/2025, 12:12:15 |
  | Modified | 11/07/2025, 12:12:15 |

  ### Tags:

  - oxytocin
  - human-animal interaction
  - dog
  - child
  - nonhuman
  - physiological stress
  - human
  - hydrocortisone
  - pet animal
  - review
  - knowledge
  - social bonding
  - child health

  ### Attachments

  - Full Text (HTML)
- ## Mechanisms of Social Attachment Between Children and Pet Dogs

  |  |  |
  | --- | --- |
  | Item Type | Journal Article |
  | Author | O.T. Reilly |
  | Author | L.H. Somerville |
  | Author | E.E. Hecht |
  | Abstract | An increasing body of evidence indicates that owning a pet dog is associated with improvements in child health and well-being. Importantly, the degree of the social bond between child and dog may mediate the beneficial outcomes of dog ownership. The formation of social bonds is an intrinsically dyadic, interactive process where each interactor’s behavior influences the other’s behavior. For this reason, it is critical to evaluate the biological mechanisms of attachment in both children and their pet dogs as a socially bonded pair. Here, we review the physical, mental, and emotional outcomes that are associated with pet dog ownership or interaction in children. We then discuss the evidence that suggests that the strength of a social bond between a child and their pet dog matters for maximizing the beneficial outcomes associated with pet dog ownership, such as possible stress-buffering effects. We review the existing literature on the neural and endocrinological mechanisms of social attachment for inter-species social bonds that form between human children and dogs, situating this emerging knowledge within the context of the mechanisms of intra-species bonds in mammals. Finally, we highlight the remaining open questions and point toward directions for future research. |
  | Date | 2024 |
  | Language | English |
  | Archive | Embase |
  | URL | https://www.embase.com/search/results?subaction=viewrecord&id=L2031978526&from=export |
  | Volume | 14 |
  | Publication | Animals |
  | DOI | 10.3390/ani14203036 |
  | Issue | 20 |
  | Journal Abbr | Animals |
  | ISSN | 2076-2615 |
  | Date Added | 11/07/2025, 12:13:16 |
  | Modified | 11/07/2025, 12:13:16 |

  ### Tags:

  - oxytocin
  - human-animal interaction
  - dog
  - child
  - nonhuman
  - physiological stress
  - human
  - hydrocortisone
  - pet animal
  - review
  - knowledge
  - social bonding
  - child health

  ### Attachments

  - Full Text (HTML)
- ## Grazing intensity and associated frequency of human contact, and horn status, influence activity on pasture, physiological pre-slaughter reactions and meat quality in beef heifers

  |  |  |
  | --- | --- |
  | Item Type | Journal Article |
  | Author | A.-M. Reiche |
  | Author | P. Silacci |
  | Author | F. Dohme-Meier |
  | Author | E.M.C. Terlouw |
  | Abstract | Meat quality is influenced by many factors related to the animal, such as its genetics and health status, farm management, and slaughter and processing conditions. The present study aimed to investigate the effects and interactions of grazing intensity and horn status on behaviour, physiological pre-slaughter stress status and meat characteristics of beef heifers. The study involved 32 horned and 32 disbudded F1 crossbred (Limousin Swiss Dairy breed) heifers during summer grazing on mountain pastures. Half of the heifers of each horn status were assigned to one of two grazing systems, balanced for live weight, dam and behavioural reactivity: grazing at either high (HI) or low (LI) grazing intensity. HI groups grazed in 3 times smaller paddocks and changed the paddock three times more often than LI groups. The effects of horn status and grazing intensity on physical activity on pasture, pre-slaughter stress and meat quality of the m. longissimus thoracis were studied. Compared to HI heifers, LI Heifers walked more when on pasture, showed greater stress levels before stunning, and their meat had greater water losses and greater early troponin levels. The varying pre-slaughter stress levels may be attributed to the differing frequency of human contact resulting from the differing frequency of paddock changes and may explain part of the effects on meat quality. Compared to disbudded heifers, horned heifers had faster heart rates at the abattoir, and their meat had lower cooking loss and was less juicy. Pre-slaughter heart rates showed robust correlations with various meat quality indicators. The study shows that both horn status and grazing management, including human contact, influence meat quality. Part of the effects may be related to different pre-slaughter physiological reactions, which subsequently influence meat quality. |
  | Date | 2024 |
  | Language | English |
  | Archive | Embase |
  | URL | https://www.embase.com/search/results?subaction=viewrecord&id=L2034890241&from=export |
  | Volume | 289 |
  | Publication | Livestock Science |
  | DOI | 10.1016/j.livsci.2024.105578 |
  | Issue | (Reiche A.-M.; Dohme-Meier F.) Ruminant Nutrition and Emissions, Agroscope, Posieux, Switzerland |
  | Journal Abbr | Livest. Sci. |
  | ISSN | 1871-1413 |
  | Date Added | 11/07/2025, 11:53:17 |
  | Modified | 11/07/2025, 11:53:17 |

  ### Tags:

  - human-animal interaction
  - heart rate
  - physical activity
  - animal experiment
  - article
  - female
  - nonhuman
  - physiological stress
  - controlled study
  - animal tissue
  - beef
  - cooking
  - dam (animal)
  - food quality
  - grazing
  - grazing management
  - heifer
  - horn
  - longissimus thoracis
  - pasture
  - slaughtering
  - summer
  - troponin
  - water loss

  ### Attachments

  - Full Text (HTML)
- ## Grazing intensity and associated frequency of human contact, and horn status, influence activity on pasture, physiological pre-slaughter reactions and meat quality in beef heifers

  |  |  |
  | --- | --- |
  | Item Type | Journal Article |
  | Author | A.-M. Reiche |
  | Author | P. Silacci |
  | Author | F. Dohme-Meier |
  | Author | E.M.C. Terlouw |
  | Abstract | Meat quality is influenced by many factors related to the animal, such as its genetics and health status, farm management, and slaughter and processing conditions. The present study aimed to investigate the effects and interactions of grazing intensity and horn status on behaviour, physiological pre-slaughter stress status and meat characteristics of beef heifers. The study involved 32 horned and 32 disbudded F1 crossbred (Limousin Swiss Dairy breed) heifers during summer grazing on mountain pastures. Half of the heifers of each horn status were assigned to one of two grazing systems, balanced for live weight, dam and behavioural reactivity: grazing at either high (HI) or low (LI) grazing intensity. HI groups grazed in 3 times smaller paddocks and changed the paddock three times more often than LI groups. The effects of horn status and grazing intensity on physical activity on pasture, pre-slaughter stress and meat quality of the m. longissimus thoracis were studied. Compared to HI heifers, LI Heifers walked more when on pasture, showed greater stress levels before stunning, and their meat had greater water losses and greater early troponin levels. The varying pre-slaughter stress levels may be attributed to the differing frequency of human contact resulting from the differing frequency of paddock changes and may explain part of the effects on meat quality. Compared to disbudded heifers, horned heifers had faster heart rates at the abattoir, and their meat had lower cooking loss and was less juicy. Pre-slaughter heart rates showed robust correlations with various meat quality indicators. The study shows that both horn status and grazing management, including human contact, influence meat quality. Part of the effects may be related to different pre-slaughter physiological reactions, which subsequently influence meat quality. |
  | Date | 2024 |
  | Language | English |
  | Archive | Embase |
  | URL | https://www.embase.com/search/results?subaction=viewrecord&id=L2034890241&from=export |
  | Volume | 289 |
  | Publication | Livestock Science |
  | DOI | 10.1016/j.livsci.2024.105578 |
  | Issue | (Reiche A.-M.; Dohme-Meier F.) Ruminant Nutrition and Emissions, Agroscope, Posieux, Switzerland |
  | Journal Abbr | Livest. Sci. |
  | ISSN | 1871-1413 |
  | Date Added | 11/07/2025, 12:10:38 |
  | Modified | 11/07/2025, 12:10:38 |

  ### Tags:

  - human-animal interaction
  - heart rate
  - physical activity
  - animal experiment
  - article
  - female
  - nonhuman
  - physiological stress
  - controlled study
  - animal tissue
  - beef
  - cooking
  - dam (animal)
  - food quality
  - grazing
  - grazing management
  - heifer
  - horn
  - longissimus thoracis
  - pasture
  - slaughtering
  - summer
  - troponin
  - water loss

  ### Attachments

  - Full Text (HTML)
- ## Relationships between serum serotonin, plasma cortisol, and behavioral factors in a mixed-breed, -sex, and -age group of pet dogs

  |  |  |
  | --- | --- |
  | Item Type | Journal Article |
  | Author | D.J. Rayment |
  | Author | R.A. Peters |
  | Author | L.C. Marston |
  | Author | B. De Groef |
  | Abstract | Previously identified correlations between circulating cortisol and serotonin levels with aggressive, fearful, or impulsive behavior in dogs have led to the suggestion that these measures be assessed as screening tools to aid in the identification of individual dogs with aggressive or fearful tendencies in applied settings, such as shelters or breeding programs. Previous studies investigating relationships between peripheral serotonin or cortisol and behavioral measures have several limitations, including single-breed sample groups, small sample sizes, and inconsistent methods used to collect behavioral data. This study used previously validated questionnaires to investigate relationships between owner-reported histories of dogs' behavior and peripheral cortisol and serotonin measures collected on presentation to a novel environment, in a mixed-breed and mixed-age sample of dogs. No notable relationships were found between these measures in this group, indicating that circulating cortisol and serotonin are poor candidates for use in applied behavioral assessments for mixed-breed pet dogs of varying ages. Differences between results reported here and previous literature are discussed. |
  | Date | 2020 |
  | Language | English |
  | Archive | Embase |
  | URL | https://www.embase.com/search/results?subaction=viewrecord&id=L2007006937&from=export |
  | Volume | 38 |
  | Pages | 96-102 |
  | Publication | Journal of Veterinary Behavior |
  | DOI | 10.1016/j.jveb.2020.05.007 |
  | Issue | (Rayment D.J.; Marston L.C.; De Groef B., b.degroef@latrobe.edu.au) Department of Physiology, Anatomy and Microbiology, La Trobe University, Bundoora, Victoria, Australia |
  | Journal Abbr | J. Vet. Behav. |
  | ISSN | 1558-7878 |
  | Date Added | 11/07/2025, 14:16:56 |
  | Modified | 11/07/2025, 14:16:56 |

  ### Tags:

  - animal behavior
  - article
  - female
  - male
  - nonhuman
  - human
  - hydrocortisone
  - adult
  - aged
  - controlled study
  - pet animal
  - questionnaire
  - behavior assessment
  - human-animal bond
  - fear
  - hydrocortisone blood level
  - impulsiveness
  - aggression
  - serotonin
  - serotonin blood level
  - sex difference
  - mongrel dog
  - purebred dog

  ### Attachments

  - Full Text (HTML)
- ## Relationships between serum serotonin, plasma cortisol, and behavioral factors in a mixed-breed, -sex, and -age group of pet dogs

  |  |  |
  | --- | --- |
  | Item Type | Journal Article |
  | Author | D.J. Rayment |
  | Author | R.A. Peters |
  | Author | L.C. Marston |
  | Author | B. De Groef |
  | Abstract | Previously identified correlations between circulating cortisol and serotonin levels with aggressive, fearful, or impulsive behavior in dogs have led to the suggestion that these measures be assessed as screening tools to aid in the identification of individual dogs with aggressive or fearful tendencies in applied settings, such as shelters or breeding programs. Previous studies investigating relationships between peripheral serotonin or cortisol and behavioral measures have several limitations, including single-breed sample groups, small sample sizes, and inconsistent methods used to collect behavioral data. This study used previously validated questionnaires to investigate relationships between owner-reported histories of dogs' behavior and peripheral cortisol and serotonin measures collected on presentation to a novel environment, in a mixed-breed and mixed-age sample of dogs. No notable relationships were found between these measures in this group, indicating that circulating cortisol and serotonin are poor candidates for use in applied behavioral assessments for mixed-breed pet dogs of varying ages. Differences between results reported here and previous literature are discussed. |
  | Date | 2020 |
  | Language | English |
  | Archive | Embase |
  | URL | https://www.embase.com/search/results?subaction=viewrecord&id=L2007006937&from=export |
  | Volume | 38 |
  | Pages | 96-102 |
  | Publication | Journal of Veterinary Behavior |
  | DOI | 10.1016/j.jveb.2020.05.007 |
  | Issue | (Rayment D.J.; Marston L.C.; De Groef B., b.degroef@latrobe.edu.au) Department of Physiology, Anatomy and Microbiology, La Trobe University, Bundoora, Victoria, Australia |
  | Journal Abbr | J. Vet. Behav. |
  | ISSN | 1558-7878 |
  | Date Added | 11/07/2025, 14:18:30 |
  | Modified | 11/07/2025, 14:18:30 |

  ### Tags:

  - animal behavior
  - article
  - female
  - male
  - nonhuman
  - human
  - hydrocortisone
  - adult
  - aged
  - controlled study
  - pet animal
  - questionnaire
  - behavior assessment
  - human-animal bond
  - fear
  - hydrocortisone blood level
  - impulsiveness
  - aggression
  - serotonin
  - serotonin blood level
  - sex difference
  - mongrel dog
  - purebred dog

  ### Attachments

  - Full Text (HTML)
- ## Gentle abdominal stroking (‘belly rubbing’) of pigs by a human reduces EEG total power and increases EEG frequencies

  |  |  |
  | --- | --- |
  | Item Type | Journal Article |
  | Author | J.-L. Rault |
  | Author | S. Truong |
  | Author | L. Hemsworth |
  | Author | M. Le Chevoir |
  | Author | S. Bauquier |
  | Author | A. Lai |
  | Abstract | The neurobiological response to gentle touch remains poorly understood, especially in the context of human-animal interaction. A novel approach allowed recording the pig electroencephalogram (EEG) cranially epidurally and wirelessly during positive interactions with a human. Stroking of the pig's abdomen (‘belly rubbing’), applied opportunistically, elicited a distinct behavioral response characterized by lateral recumbency, limb stretching, frequent short-lasting grunts and eye closure. Pigs varied in their responsiveness to belly rubbing but all pigs showed it. Their EEG was compared to EEG during human presence and other positive interactions except belly rubbing; isolation; and in the home pen as a baseline. Total EEG power (‘Ptot’) was lower during belly rubbing, whereas the median frequency (‘F50’, 5.3 ± 0.9 Hz vs. 3.8 ± 0.9 Hz for other contexts) and the 95% spectral edge frequency (‘F95’, 45.2 ± 3.2 Hz vs. 40.0 ± 3.2 Hz for other contexts) were higher during belly rubbing compared to other contexts. Lower EEG total power combined with a shift in spectral power distribution toward higher frequencies were linked to behavioral changes indicative of a positive welfare state during belly rubbing. The effects of belly rubbing on animal psychobiology and well-being warrant further research as a model of positive welfare state induced by touch. |
  | Date | 2019 |
  | Language | English |
  | Archive | Embase |
  | URL | https://www.embase.com/search/results?subaction=viewrecord&id=L2001896926&from=export |
  | Volume | 374 |
  | Publication | Behavioural Brain Research |
  | DOI | 10.1016/j.bbr.2019.04.006 |
  | Issue | (Rault J.-L., jean-loup.rault@vetmeduni.ac.at) Institute of Animal Welfare Science, University of Veterinary Medicine, Vienna, Austria |
  | Journal Abbr | Behav. Brain Res. |
  | ISSN | 1872-7549 |
  | Date Added | 11/07/2025, 11:49:34 |
  | Modified | 11/07/2025, 11:49:34 |

  ### Tags:

  - welfare
  - animal experiment
  - article
  - nonhuman
  - controlled study
  - electroencephalogram
  - abdomen
  - behavior change
  - electrocorticography
  - eyelid closure
  - leisure
  - limb
  - massage
  - pig
  - psychobiology
  - recumbency
  - touch
  - wellbeing

  ### Attachments

  - Full Text (HTML)
- ## Ground-based adaptive horsemanship lessons for veterans with post-traumatic stress disorder: a randomized controlled pilot study

  |  |  |
  | --- | --- |
  | Item Type | Journal Article |
  | Author | E.M. Rankins |
  | Author | A. Quinn |
  | Author | K.H. McKeever |
  | Author | K. Malinowski |
  | Abstract | Introduction: Equine-assisted services (EAS) has received attention as a potential treatment strategy for post-traumatic stress disorder (PTSD), as existing literature indicates that symptoms may decrease following EAS. Relatively little is known about the mechanisms at play during lessons and if physiological measures are impacted. The objectives of this pilot study were to 1) explore the effects of adaptive horsemanship (AH) lessons on symptoms of PTSD, hormone concentrations, and social motor synchrony; 2) determine if physiological changes occur as veterans interact with horses; and 3) explore if the interaction between veteran and horse changes over the 8-week session. Methods: Veterans with PTSD were randomly assigned to control (CON, n = 3) or AH (n = 6) groups for an 8-week period (clinical trial; NCT04850573; clinicaltrials.gov). Veterans completed the PTSD Checklist (PCL-5) and Brief Symptom Inventory (BSI) at pre-, post-, and 2- and 6-month follow-up time points. They also completed a social motor synchrony test (pendulum swinging) and blood draw at pre- and post-time points. In weeks 1, 4, and 8, blood samples were drawn at 0 min, 3 min, 5 min, 25 min, and 30 min during the 30-min AH lessons. Veterans completed the Human-Animal Interaction Scale (HAIS) after each lesson. Blood samples were assayed for plasma cortisol, epinephrine, norepinephrine, and oxytocin. Data were analyzed with repeated measure ANOVAs. Changes in PTSD symptoms from pre- to post-time point were analyzed with paired t-tests. Results: Changes in PCL-5 scores tended to differ (p = 0.0989), and global BSI scores differed (p = 0.0266) between AH (−11.5 ± 5.5, mean ± SE; −0.5 ± 0.2) and CON (5.3 ± 5.4; 0.4 ± 0.2) groups. Social motor synchrony and hormone concentrations did not differ between groups or time points (p > 0.05). Cortisol, norepinephrine, and oxytocin concentrations did not differ across sessions (p > 0.05). Epinephrine concentrations tended (p = 0.0744) to decrease from week 1 to 4 of sessions. HAIS scores increased (p ≥ 0.0437) in week 3 and remained elevated as compared to week 1. Discussion: Participant recruitment was the greatest challenge. These preliminary results agree with the literature suggesting that EAS can reduce symptoms of PTSD. |
  | Date | 2024 |
  | Language | English |
  | Archive | Embase |
  | URL | https://www.embase.com/search/results?subaction=viewrecord&id=L2030160775&from=export |
  | Volume | 15 |
  | Publication | Frontiers in Psychiatry |
  | DOI | 10.3389/fpsyt.2024.1390212 |
  | Issue | (Rankins E.M., ellen.rankins@colostate.edu; McKeever K.H.; Malinowski K.) Equine Science Center, Department of Animal Sciences, Rutgers University, New Brunswick, NJ, United States |
  | Journal Abbr | Front. Psychiatry |
  | ISSN | 1664-0640 |
  | Date Added | 11/07/2025, 11:48:34 |
  | Modified | 11/07/2025, 11:48:34 |

  ### Tags:

  - oxytocin
  - human-animal interaction
  - veteran
  - animal behavior
  - article
  - female
  - male
  - human
  - hydrocortisone
  - 9.4
  - adaptive behavior
  - adaptive horsemanship
  - adult
  - aged
  - Autoguard
  - bioinformatics software
  - biomedical software
  - blood collection tube
  - blood sampling
  - body weight
  - Brief Symptom Inventory
  - checklist
  - competitive ELISA
  - computer
  - controlled study
  - data analysis software
  - electromyograph
  - ELISA kit
  - epinephrine
  - Excel
  - feedback system
  - follow up
  - Fourier transform
  - grooming
  - gyroscope sensor
  - hippotherapy
  - hormone determination
  - horseback riding
  - Human Animal Interaction Scale
  - Insyte
  - intravenous catheter
  - iPad mini 2 OS v12.5.4
  - Life Events Checklist 5
  - MARS v 3.20
  - MATLAB 2022b
  - Mobile Precision 3541
  - muscle contraction
  - myoMUSCLE
  - NCT04850573
  - noradrenalin
  - occupational therapy
  - physiotherapy
  - pilot study
  - post traumatic stress disorder checklist 5
  - posttraumatic stress disorder
  - randomized controlled trial
  - range of vision
  - scoring system
  - silver electrode
  - social motor synchrony
  - social synchronization
  - surface electromyography
  - tablet computer
  - Ultium EMG
  - Vacutainer 23 GA
  - vein puncture
  - visual system parameters

  ### Attachments

  - Full Text (HTML)
- ## Ground-based adaptive horsemanship lessons for veterans with post-traumatic stress disorder: a randomized controlled pilot study

  |  |  |
  | --- | --- |
  | Item Type | Journal Article |
  | Author | E.M. Rankins |
  | Author | A. Quinn |
  | Author | K.H. McKeever |
  | Author | K. Malinowski |
  | Abstract | Introduction: Equine-assisted services (EAS) has received attention as a potential treatment strategy for post-traumatic stress disorder (PTSD), as existing literature indicates that symptoms may decrease following EAS. Relatively little is known about the mechanisms at play during lessons and if physiological measures are impacted. The objectives of this pilot study were to 1) explore the effects of adaptive horsemanship (AH) lessons on symptoms of PTSD, hormone concentrations, and social motor synchrony; 2) determine if physiological changes occur as veterans interact with horses; and 3) explore if the interaction between veteran and horse changes over the 8-week session. Methods: Veterans with PTSD were randomly assigned to control (CON, n = 3) or AH (n = 6) groups for an 8-week period (clinical trial; NCT04850573; clinicaltrials.gov). Veterans completed the PTSD Checklist (PCL-5) and Brief Symptom Inventory (BSI) at pre-, post-, and 2- and 6-month follow-up time points. They also completed a social motor synchrony test (pendulum swinging) and blood draw at pre- and post-time points. In weeks 1, 4, and 8, blood samples were drawn at 0 min, 3 min, 5 min, 25 min, and 30 min during the 30-min AH lessons. Veterans completed the Human-Animal Interaction Scale (HAIS) after each lesson. Blood samples were assayed for plasma cortisol, epinephrine, norepinephrine, and oxytocin. Data were analyzed with repeated measure ANOVAs. Changes in PTSD symptoms from pre- to post-time point were analyzed with paired t-tests. Results: Changes in PCL-5 scores tended to differ (p = 0.0989), and global BSI scores differed (p = 0.0266) between AH (−11.5 ± 5.5, mean ± SE; −0.5 ± 0.2) and CON (5.3 ± 5.4; 0.4 ± 0.2) groups. Social motor synchrony and hormone concentrations did not differ between groups or time points (p > 0.05). Cortisol, norepinephrine, and oxytocin concentrations did not differ across sessions (p > 0.05). Epinephrine concentrations tended (p = 0.0744) to decrease from week 1 to 4 of sessions. HAIS scores increased (p ≥ 0.0437) in week 3 and remained elevated as compared to week 1. Discussion: Participant recruitment was the greatest challenge. These preliminary results agree with the literature suggesting that EAS can reduce symptoms of PTSD. |
  | Date | 2024 |
  | Language | English |
  | Archive | Embase |
  | URL | https://www.embase.com/search/results?subaction=viewrecord&id=L2030160775&from=export |
  | Volume | 15 |
  | Publication | Frontiers in Psychiatry |
  | DOI | 10.3389/fpsyt.2024.1390212 |
  | Issue | (Rankins E.M., ellen.rankins@colostate.edu; McKeever K.H.; Malinowski K.) Equine Science Center, Department of Animal Sciences, Rutgers University, New Brunswick, NJ, United States |
  | Journal Abbr | Front. Psychiatry |
  | ISSN | 1664-0640 |
  | Date Added | 11/07/2025, 12:12:16 |
  | Modified | 11/07/2025, 12:12:16 |

  ### Tags:

  - oxytocin
  - human-animal interaction
  - veteran
  - animal behavior
  - article
  - female
  - male
  - human
  - hydrocortisone
  - 9.4
  - adaptive behavior
  - adaptive horsemanship
  - adult
  - aged
  - Autoguard
  - bioinformatics software
  - biomedical software
  - blood collection tube
  - blood sampling
  - body weight
  - Brief Symptom Inventory
  - checklist
  - competitive ELISA
  - computer
  - controlled study
  - data analysis software
  - electromyograph
  - ELISA kit
  - epinephrine
  - Excel
  - feedback system
  - follow up
  - Fourier transform
  - grooming
  - gyroscope sensor
  - hippotherapy
  - hormone determination
  - horseback riding
  - Human Animal Interaction Scale
  - Insyte
  - intravenous catheter
  - iPad mini 2 OS v12.5.4
  - Life Events Checklist 5
  - MARS v 3.20
  - MATLAB 2022b
  - Mobile Precision 3541
  - muscle contraction
  - myoMUSCLE
  - NCT04850573
  - noradrenalin
  - occupational therapy
  - physiotherapy
  - pilot study
  - post traumatic stress disorder checklist 5
  - posttraumatic stress disorder
  - randomized controlled trial
  - range of vision
  - scoring system
  - silver electrode
  - social motor synchrony
  - social synchronization
  - surface electromyography
  - tablet computer
  - Ultium EMG
  - Vacutainer 23 GA
  - vein puncture
  - visual system parameters

  ### Attachments

  - Full Text (HTML)
- ## Ground-based adaptive horsemanship lessons for veterans with post-traumatic stress disorder: a randomized controlled pilot study

  |  |  |
  | --- | --- |
  | Item Type | Journal Article |
  | Author | E.M. Rankins |
  | Author | A. Quinn |
  | Author | K.H. McKeever |
  | Author | K. Malinowski |
  | Abstract | Introduction: Equine-assisted services (EAS) has received attention as a potential treatment strategy for post-traumatic stress disorder (PTSD), as existing literature indicates that symptoms may decrease following EAS. Relatively little is known about the mechanisms at play during lessons and if physiological measures are impacted. The objectives of this pilot study were to 1) explore the effects of adaptive horsemanship (AH) lessons on symptoms of PTSD, hormone concentrations, and social motor synchrony; 2) determine if physiological changes occur as veterans interact with horses; and 3) explore if the interaction between veteran and horse changes over the 8-week session. Methods: Veterans with PTSD were randomly assigned to control (CON, n = 3) or AH (n = 6) groups for an 8-week period (clinical trial; NCT04850573; clinicaltrials.gov). Veterans completed the PTSD Checklist (PCL-5) and Brief Symptom Inventory (BSI) at pre-, post-, and 2- and 6-month follow-up time points. They also completed a social motor synchrony test (pendulum swinging) and blood draw at pre- and post-time points. In weeks 1, 4, and 8, blood samples were drawn at 0 min, 3 min, 5 min, 25 min, and 30 min during the 30-min AH lessons. Veterans completed the Human-Animal Interaction Scale (HAIS) after each lesson. Blood samples were assayed for plasma cortisol, epinephrine, norepinephrine, and oxytocin. Data were analyzed with repeated measure ANOVAs. Changes in PTSD symptoms from pre- to post-time point were analyzed with paired t-tests. Results: Changes in PCL-5 scores tended to differ (p = 0.0989), and global BSI scores differed (p = 0.0266) between AH (−11.5 ± 5.5, mean ± SE; −0.5 ± 0.2) and CON (5.3 ± 5.4; 0.4 ± 0.2) groups. Social motor synchrony and hormone concentrations did not differ between groups or time points (p > 0.05). Cortisol, norepinephrine, and oxytocin concentrations did not differ across sessions (p > 0.05). Epinephrine concentrations tended (p = 0.0744) to decrease from week 1 to 4 of sessions. HAIS scores increased (p ≥ 0.0437) in week 3 and remained elevated as compared to week 1. Discussion: Participant recruitment was the greatest challenge. These preliminary results agree with the literature suggesting that EAS can reduce symptoms of PTSD. |
  | Date | 2024 |
  | Language | English |
  | Archive | Embase |
  | URL | https://www.embase.com/search/results?subaction=viewrecord&id=L2030160775&from=export |
  | Volume | 15 |
  | Publication | Frontiers in Psychiatry |
  | DOI | 10.3389/fpsyt.2024.1390212 |
  | Issue | (Rankins E.M., ellen.rankins@colostate.edu; McKeever K.H.; Malinowski K.) Equine Science Center, Department of Animal Sciences, Rutgers University, New Brunswick, NJ, United States |
  | Journal Abbr | Front. Psychiatry |
  | ISSN | 1664-0640 |
  | Date Added | 11/07/2025, 12:13:18 |
  | Modified | 11/07/2025, 12:13:18 |

  ### Tags:

  - oxytocin
  - human-animal interaction
  - veteran
  - animal behavior
  - article
  - female
  - male
  - human
  - hydrocortisone
  - 9.4
  - adaptive behavior
  - adaptive horsemanship
  - adult
  - aged
  - Autoguard
  - bioinformatics software
  - biomedical software
  - blood collection tube
  - blood sampling
  - body weight
  - Brief Symptom Inventory
  - checklist
  - competitive ELISA
  - computer
  - controlled study
  - data analysis software
  - electromyograph
  - ELISA kit
  - epinephrine
  - Excel
  - feedback system
  - follow up
  - Fourier transform
  - grooming
  - gyroscope sensor
  - hippotherapy
  - hormone determination
  - horseback riding
  - Human Animal Interaction Scale
  - Insyte
  - intravenous catheter
  - iPad mini 2 OS v12.5.4
  - Life Events Checklist 5
  - MARS v 3.20
  - MATLAB 2022b
  - Mobile Precision 3541
  - muscle contraction
  - myoMUSCLE
  - NCT04850573
  - noradrenalin
  - occupational therapy
  - physiotherapy
  - pilot study
  - post traumatic stress disorder checklist 5
  - posttraumatic stress disorder
  - randomized controlled trial
  - range of vision
  - scoring system
  - silver electrode
  - social motor synchrony
  - social synchronization
  - surface electromyography
  - tablet computer
  - Ultium EMG
  - Vacutainer 23 GA
  - vein puncture
  - visual system parameters

  ### Attachments

  - Full Text (HTML)
- ## Ground-based adaptive horsemanship lessons for veterans with post-traumatic stress disorder: a randomized controlled pilot study

  |  |  |
  | --- | --- |
  | Item Type | Journal Article |
  | Author | E.M. Rankins |
  | Author | A. Quinn |
  | Author | K.H. McKeever |
  | Author | K. Malinowski |
  | Abstract | Introduction: Equine-assisted services (EAS) has received attention as a potential treatment strategy for post-traumatic stress disorder (PTSD), as existing literature indicates that symptoms may decrease following EAS. Relatively little is known about the mechanisms at play during lessons and if physiological measures are impacted. The objectives of this pilot study were to 1) explore the effects of adaptive horsemanship (AH) lessons on symptoms of PTSD, hormone concentrations, and social motor synchrony; 2) determine if physiological changes occur as veterans interact with horses; and 3) explore if the interaction between veteran and horse changes over the 8-week session. Methods: Veterans with PTSD were randomly assigned to control (CON, n = 3) or AH (n = 6) groups for an 8-week period (clinical trial; NCT04850573; clinicaltrials.gov). Veterans completed the PTSD Checklist (PCL-5) and Brief Symptom Inventory (BSI) at pre-, post-, and 2- and 6-month follow-up time points. They also completed a social motor synchrony test (pendulum swinging) and blood draw at pre- and post-time points. In weeks 1, 4, and 8, blood samples were drawn at 0 min, 3 min, 5 min, 25 min, and 30 min during the 30-min AH lessons. Veterans completed the Human-Animal Interaction Scale (HAIS) after each lesson. Blood samples were assayed for plasma cortisol, epinephrine, norepinephrine, and oxytocin. Data were analyzed with repeated measure ANOVAs. Changes in PTSD symptoms from pre- to post-time point were analyzed with paired t-tests. Results: Changes in PCL-5 scores tended to differ (p = 0.0989), and global BSI scores differed (p = 0.0266) between AH (−11.5 ± 5.5, mean ± SE; −0.5 ± 0.2) and CON (5.3 ± 5.4; 0.4 ± 0.2) groups. Social motor synchrony and hormone concentrations did not differ between groups or time points (p > 0.05). Cortisol, norepinephrine, and oxytocin concentrations did not differ across sessions (p > 0.05). Epinephrine concentrations tended (p = 0.0744) to decrease from week 1 to 4 of sessions. HAIS scores increased (p ≥ 0.0437) in week 3 and remained elevated as compared to week 1. Discussion: Participant recruitment was the greatest challenge. These preliminary results agree with the literature suggesting that EAS can reduce symptoms of PTSD. |
  | Date | 2024 |
  | Language | English |
  | Archive | Embase |
  | URL | https://www.embase.com/search/results?subaction=viewrecord&id=L2030160775&from=export |
  | Volume | 15 |
  | Publication | Frontiers in Psychiatry |
  | DOI | 10.3389/fpsyt.2024.1390212 |
  | Issue | (Rankins E.M., ellen.rankins@colostate.edu; McKeever K.H.; Malinowski K.) Equine Science Center, Department of Animal Sciences, Rutgers University, New Brunswick, NJ, United States |
  | Journal Abbr | Front. Psychiatry |
  | ISSN | 1664-0640 |
  | Date Added | 11/07/2025, 14:15:30 |
  | Modified | 11/07/2025, 14:15:30 |

  ### Tags:

  - oxytocin
  - human-animal interaction
  - veteran
  - animal behavior
  - article
  - female
  - male
  - human
  - hydrocortisone
  - 9.4
  - adaptive behavior
  - adaptive horsemanship
  - adult
  - aged
  - Autoguard
  - bioinformatics software
  - biomedical software
  - blood collection tube
  - blood sampling
  - body weight
  - Brief Symptom Inventory
  - checklist
  - competitive ELISA
  - computer
  - controlled study
  - data analysis software
  - electromyograph
  - ELISA kit
  - epinephrine
  - Excel
  - feedback system
  - follow up
  - Fourier transform
  - grooming
  - gyroscope sensor
  - hippotherapy
  - hormone determination
  - horseback riding
  - Human Animal Interaction Scale
  - Insyte
  - intravenous catheter
  - iPad mini 2 OS v12.5.4
  - Life Events Checklist 5
  - MARS v 3.20
  - MATLAB 2022b
  - Mobile Precision 3541
  - muscle contraction
  - myoMUSCLE
  - NCT04850573
  - noradrenalin
  - occupational therapy
  - physiotherapy
  - pilot study
  - post traumatic stress disorder checklist 5
  - posttraumatic stress disorder
  - randomized controlled trial
  - range of vision
  - scoring system
  - silver electrode
  - social motor synchrony
  - social synchronization
  - surface electromyography
  - tablet computer
  - Ultium EMG
  - Vacutainer 23 GA
  - vein puncture
  - visual system parameters

  ### Attachments

  - Full Text (HTML)
- ## Ground-based adaptive horsemanship lessons for veterans with post-traumatic stress disorder: a randomized controlled pilot study

  |  |  |
  | --- | --- |
  | Item Type | Journal Article |
  | Author | E.M. Rankins |
  | Author | A. Quinn |
  | Author | K.H. McKeever |
  | Author | K. Malinowski |
  | Abstract | Introduction: Equine-assisted services (EAS) has received attention as a potential treatment strategy for post-traumatic stress disorder (PTSD), as existing literature indicates that symptoms may decrease following EAS. Relatively little is known about the mechanisms at play during lessons and if physiological measures are impacted. The objectives of this pilot study were to 1) explore the effects of adaptive horsemanship (AH) lessons on symptoms of PTSD, hormone concentrations, and social motor synchrony; 2) determine if physiological changes occur as veterans interact with horses; and 3) explore if the interaction between veteran and horse changes over the 8-week session. Methods: Veterans with PTSD were randomly assigned to control (CON, n = 3) or AH (n = 6) groups for an 8-week period (clinical trial; NCT04850573; clinicaltrials.gov). Veterans completed the PTSD Checklist (PCL-5) and Brief Symptom Inventory (BSI) at pre-, post-, and 2- and 6-month follow-up time points. They also completed a social motor synchrony test (pendulum swinging) and blood draw at pre- and post-time points. In weeks 1, 4, and 8, blood samples were drawn at 0 min, 3 min, 5 min, 25 min, and 30 min during the 30-min AH lessons. Veterans completed the Human-Animal Interaction Scale (HAIS) after each lesson. Blood samples were assayed for plasma cortisol, epinephrine, norepinephrine, and oxytocin. Data were analyzed with repeated measure ANOVAs. Changes in PTSD symptoms from pre- to post-time point were analyzed with paired t-tests. Results: Changes in PCL-5 scores tended to differ (p = 0.0989), and global BSI scores differed (p = 0.0266) between AH (−11.5 ± 5.5, mean ± SE; −0.5 ± 0.2) and CON (5.3 ± 5.4; 0.4 ± 0.2) groups. Social motor synchrony and hormone concentrations did not differ between groups or time points (p > 0.05). Cortisol, norepinephrine, and oxytocin concentrations did not differ across sessions (p > 0.05). Epinephrine concentrations tended (p = 0.0744) to decrease from week 1 to 4 of sessions. HAIS scores increased (p ≥ 0.0437) in week 3 and remained elevated as compared to week 1. Discussion: Participant recruitment was the greatest challenge. These preliminary results agree with the literature suggesting that EAS can reduce symptoms of PTSD. |
  | Date | 2024 |
  | Language | English |
  | Archive | Embase |
  | URL | https://www.embase.com/search/results?subaction=viewrecord&id=L2030160775&from=export |
  | Volume | 15 |
  | Publication | Frontiers in Psychiatry |
  | DOI | 10.3389/fpsyt.2024.1390212 |
  | Issue | (Rankins E.M., ellen.rankins@colostate.edu; McKeever K.H.; Malinowski K.) Equine Science Center, Department of Animal Sciences, Rutgers University, New Brunswick, NJ, United States |
  | Journal Abbr | Front. Psychiatry |
  | ISSN | 1664-0640 |
  | Date Added | 11/07/2025, 14:16:41 |
  | Modified | 11/07/2025, 14:16:41 |

  ### Tags:

  - oxytocin
  - human-animal interaction
  - veteran
  - animal behavior
  - article
  - female
  - male
  - human
  - hydrocortisone
  - 9.4
  - adaptive behavior
  - adaptive horsemanship
  - adult
  - aged
  - Autoguard
  - bioinformatics software
  - biomedical software
  - blood collection tube
  - blood sampling
  - body weight
  - Brief Symptom Inventory
  - checklist
  - competitive ELISA
  - computer
  - controlled study
  - data analysis software
  - electromyograph
  - ELISA kit
  - epinephrine
  - Excel
  - feedback system
  - follow up
  - Fourier transform
  - grooming
  - gyroscope sensor
  - hippotherapy
  - hormone determination
  - horseback riding
  - Human Animal Interaction Scale
  - Insyte
  - intravenous catheter
  - iPad mini 2 OS v12.5.4
  - Life Events Checklist 5
  - MARS v 3.20
  - MATLAB 2022b
  - Mobile Precision 3541
  - muscle contraction
  - myoMUSCLE
  - NCT04850573
  - noradrenalin
  - occupational therapy
  - physiotherapy
  - pilot study
  - post traumatic stress disorder checklist 5
  - posttraumatic stress disorder
  - randomized controlled trial
  - range of vision
  - scoring system
  - silver electrode
  - social motor synchrony
  - social synchronization
  - surface electromyography
  - tablet computer
  - Ultium EMG
  - Vacutainer 23 GA
  - vein puncture
  - visual system parameters

  ### Attachments

  - Full Text (HTML)
- ## Ground-based adaptive horsemanship lessons for veterans with post-traumatic stress disorder: a randomized controlled pilot study

  |  |  |
  | --- | --- |
  | Item Type | Journal Article |
  | Author | E.M. Rankins |
  | Author | A. Quinn |
  | Author | K.H. McKeever |
  | Author | K. Malinowski |
  | Abstract | Introduction: Equine-assisted services (EAS) has received attention as a potential treatment strategy for post-traumatic stress disorder (PTSD), as existing literature indicates that symptoms may decrease following EAS. Relatively little is known about the mechanisms at play during lessons and if physiological measures are impacted. The objectives of this pilot study were to 1) explore the effects of adaptive horsemanship (AH) lessons on symptoms of PTSD, hormone concentrations, and social motor synchrony; 2) determine if physiological changes occur as veterans interact with horses; and 3) explore if the interaction between veteran and horse changes over the 8-week session. Methods: Veterans with PTSD were randomly assigned to control (CON, n = 3) or AH (n = 6) groups for an 8-week period (clinical trial; NCT04850573; clinicaltrials.gov). Veterans completed the PTSD Checklist (PCL-5) and Brief Symptom Inventory (BSI) at pre-, post-, and 2- and 6-month follow-up time points. They also completed a social motor synchrony test (pendulum swinging) and blood draw at pre- and post-time points. In weeks 1, 4, and 8, blood samples were drawn at 0 min, 3 min, 5 min, 25 min, and 30 min during the 30-min AH lessons. Veterans completed the Human-Animal Interaction Scale (HAIS) after each lesson. Blood samples were assayed for plasma cortisol, epinephrine, norepinephrine, and oxytocin. Data were analyzed with repeated measure ANOVAs. Changes in PTSD symptoms from pre- to post-time point were analyzed with paired t-tests. Results: Changes in PCL-5 scores tended to differ (p = 0.0989), and global BSI scores differed (p = 0.0266) between AH (−11.5 ± 5.5, mean ± SE; −0.5 ± 0.2) and CON (5.3 ± 5.4; 0.4 ± 0.2) groups. Social motor synchrony and hormone concentrations did not differ between groups or time points (p > 0.05). Cortisol, norepinephrine, and oxytocin concentrations did not differ across sessions (p > 0.05). Epinephrine concentrations tended (p = 0.0744) to decrease from week 1 to 4 of sessions. HAIS scores increased (p ≥ 0.0437) in week 3 and remained elevated as compared to week 1. Discussion: Participant recruitment was the greatest challenge. These preliminary results agree with the literature suggesting that EAS can reduce symptoms of PTSD. |
  | Date | 2024 |
  | Language | English |
  | Archive | Embase |
  | URL | https://www.embase.com/search/results?subaction=viewrecord&id=L2030160775&from=export |
  | Volume | 15 |
  | Publication | Frontiers in Psychiatry |
  | DOI | 10.3389/fpsyt.2024.1390212 |
  | Issue | (Rankins E.M., ellen.rankins@colostate.edu; McKeever K.H.; Malinowski K.) Equine Science Center, Department of Animal Sciences, Rutgers University, New Brunswick, NJ, United States |
  | Journal Abbr | Front. Psychiatry |
  | ISSN | 1664-0640 |
  | Date Added | 11/07/2025, 14:18:14 |
  | Modified | 11/07/2025, 14:18:14 |

  ### Tags:

  - oxytocin
  - human-animal interaction
  - veteran
  - animal behavior
  - article
  - female
  - male
  - human
  - hydrocortisone
  - 9.4
  - adaptive behavior
  - adaptive horsemanship
  - adult
  - aged
  - Autoguard
  - bioinformatics software
  - biomedical software
  - blood collection tube
  - blood sampling
  - body weight
  - Brief Symptom Inventory
  - checklist
  - competitive ELISA
  - computer
  - controlled study
  - data analysis software
  - electromyograph
  - ELISA kit
  - epinephrine
  - Excel
  - feedback system
  - follow up
  - Fourier transform
  - grooming
  - gyroscope sensor
  - hippotherapy
  - hormone determination
  - horseback riding
  - Human Animal Interaction Scale
  - Insyte
  - intravenous catheter
  - iPad mini 2 OS v12.5.4
  - Life Events Checklist 5
  - MARS v 3.20
  - MATLAB 2022b
  - Mobile Precision 3541
  - muscle contraction
  - myoMUSCLE
  - NCT04850573
  - noradrenalin
  - occupational therapy
  - physiotherapy
  - pilot study
  - post traumatic stress disorder checklist 5
  - posttraumatic stress disorder
  - randomized controlled trial
  - range of vision
  - scoring system
  - silver electrode
  - social motor synchrony
  - social synchronization
  - surface electromyography
  - tablet computer
  - Ultium EMG
  - Vacutainer 23 GA
  - vein puncture
  - visual system parameters

  ### Attachments

  - Full Text (HTML)
- ## Human animal interaction: Bond's bases and mechanisms

  |  |  |
  | --- | --- |
  | Item Type | Journal Article |
  | Author | J. Raimonda |
  | Abstract | Human beings, as a social species, have evolved in relation to nature and especially toward pets, with apparent consequences for both. These relationships include emotional, psychological, and physical interactions among people, animals, and the environment. Knowing the bases and mechanisms of the human-animal bond (HAB) facilitates the understanding of interactions' impact on the well-being of both. In this way, attachment is an important factor, and the oxytocinergic system is the main mechanism involved in setting up of bond. Also, oxytocin has implications in social memory, in sexual and maternal behavior, in reducing stress and anxiety, and in increasing confidence. After eye contact, the hormone is released, particularly during pleasant tactile interactions, in which there is also a decrease in the stress levels of both. After positive interactions, an increase in β-endorphin, prolactin, β-phenylethylamine, and dopamine in both species is observed. The rise of dopamine plasma concentrations in humans and dogs, suggests that they perceive pleasant sensations during the interaction. Recent research reports positive effects on the immune system, with a significant increase in salivary immunoglobulin A in people after petting a dog. Contributions from genetics suggest that oxytocin receptor gene polymorphisms have an impact on the search for proximity to an unknown person or its owner, and how dogs behave friendly with strangers. Functional neuroimaging techniques (PET) could be a useful tool to examine the underlying neuronal mechanisms of wellbeing associated with HAB, which is shown in regional brain responses. Lower stress response was found in people in the presence of their companion animal, correlated with the deactivation of some cortical and para-limbic brain regions. In short, the relationship between humans and animals is dynamic and mutually beneficial, being influenced by the behaviors of both. This bond includes emotional, psychological, and physical interactions between people, animals, and the environment, which can be explained through the study of the biological bases and mechanisms involved at different levels. |
  | Date | 2020 |
  | Language | English |
  | Archive | Embase |
  | URL | https://www.embase.com/search/results?subaction=viewrecord&id=L633494004&from=export |
  | Volume | 44 |
  | Pages | 6-7 |
  | Publication | Biocell |
  | Issue | SUPPL 2 |
  | Journal Abbr | Biocell |
  | ISSN | 1667-5746 |
  | Date Added | 11/07/2025, 12:13:30 |
  | Modified | 11/07/2025, 12:13:30 |

  ### Tags:

  - oxytocin
  - prolactin
  - dog
  - anxiety
  - animal experiment
  - female
  - male
  - nonhuman
  - physiological stress
  - human
  - adult
  - controlled study
  - dopamine
  - wellbeing
  - animal tissue
  - human-animal bond
  - maternal behavior
  - beta endorphin
  - immunoglobulin A
  - endogenous compound
  - oxytocin receptor
  - hormone
  - conference abstract
  - DNA polymorphism
  - functional neuroimaging
  - genetic association
  - immune system
  - limbic cortex
  - memory
  - phenethylamine
  - receptor gene
  - sensation
- ## Neural Basis of Categorical Representations of Animal Body Silhouettes

  |  |  |
  | --- | --- |
  | Item Type | Journal Article |
  | Author | Y. Pu |
  | Author | S. Han |
  | Abstract | Neural activities differentiating bodies versus non-body stimuli have been identified in the occipitotemporal cortex of both humans and nonhuman primates. However, the neural mechanisms of coding the similarity of different individuals’ bodies of the same species to support their categorical representations remain unclear. Using electroencephalography (EEG) and magnetoencephalography (MEG), we investigated the temporal and spatial characteristics of neural processes shared by different individual body silhouettes of the same species by quantifying the repetition suppression of neural responses to human and animal (chimpanzee, dog, and bird) body silhouettes showing different postures. Our EEG results revealed significant repetition suppression of the amplitudes of early frontal/central activity at 180–220 ms (P2) and late occipitoparietal activity at 220–320 ms (P270) in response to animal (but not human) body silhouettes of the same species. Our MEG results further localized the repetition suppression effect related to animal body silhouettes in the left supramarginal gyrus and left frontal cortex at 200–440 ms after stimulus onset. Our findings suggest two neural processes that are involved in spontaneous categorical representations of animal body silhouettes as a cognitive basis of human-animal interactions. |
  | Date | 2025 |
  | Language | English |
  | Archive | Embase |
  | URL | https://www.embase.com/search/results?subaction=viewrecord&id=L2030723845&from=export |
  | Volume | 41 |
  | Pages | 211-223 |
  | Publication | Neuroscience Bulletin |
  | DOI | 10.1007/s12264-024-01268-1 |
  | Issue | 2 |
  | Journal Abbr | Neurosci. Bull. |
  | ISSN | 1995-8218 |
  | Date Added | 11/07/2025, 11:49:32 |
  | Modified | 11/07/2025, 11:49:32 |

  ### Tags:

  - human-animal interaction
  - cognition
  - article
  - female
  - male
  - nonhuman
  - human
  - adult
  - controlled study
  - electroencephalogram
  - body position
  - chimpanzee
  - electroencephalography
  - frontal cortex
  - human experiment
  - intelligence
  - magnetoencephalography
  - nerve cell
  - nerve potential
  - normal human
  - supramarginal gyrus

  ### Attachments

  - Full Text (HTML)
- ## Effects of Human–Dog Interactions on Salivary Oxytocin Concentrations and Heart Rate Variability: A Four-Condition Cross-Over Trial

  |  |  |
  | --- | --- |
  | Item Type | Journal Article |
  | Author | L. Powell |
  | Author | K.M. Edwards |
  | Author | S. Michael |
  | Author | P. McGreevy |
  | Author | A. Bauman |
  | Author | A.J. Guastella |
  | Author | B. Drayton |
  | Author | E. Stamatakis |
  | Abstract | Dog ownership is often advocated for its potential benefits to human health, with changes to oxytocin and autonomic nervous system (ANS) activity proposed as possible underlying mechanisms. The aims of the current study were to a) examine the effect of two common types of human–dog interactions (dog-walking and affiliative interactions) on salivary oxytocin concentrations and heart rate variability (HRV, an indicator of ANS activity), and b) investigate any putative moderating role for the strength of human–dog attachment on such responses. Twenty-nine dog owners completed a four-condition random-order cross-over trial: dog-walking (DW); walking without the dog (W); affiliative human–dog interaction (H-DI); and resting without the dog (C). Each condition was performed for approximately 15 minutes. Saliva and HRV samples were collected before and after each condition. Linear mixed models were used to analyze data, with the participant considered a random effect; condition, order of conditions, and condition duration as fixed effects. Oxytocin concentrations were not significantly different following any of the four conditions. HRV was significantly reduced following DW (mean change HF HRV = –0.37, 95% CI = –0.70, –0.04) and W (mean change HF HRV = –0.49, 95% CI = –0.81, –0.17). Considering moderation by the strength of the human–dog bond, pairwise comparisons revealed that, compared with W, DW elicited an increase in oxytocin concentrations (mean change 9.32 pg/mL, 95% CI = 6.52, 12.12) and HRV (mean change SDRR = 0.41, 95% CI = 0.19, 0.63) in owners with lower levels of attachment. These owners also displayed increased oxytocin concentrations following H-DI (mean change 3.90 pg/mL, 95% CI = 1.48, 6.32), compared with C. Overall, we did not find a consistent pattern for positive oxytocin or HRV responses to human–dog interactions. The strength of owner–dog attachment was found to have a moderating effect, suggesting that human–dog interactions may elicit greater physiological responses in low-attachment individuals. |
  | Date | 2020 |
  | Language | English |
  | Archive | Embase |
  | URL | https://www.embase.com/search/results?subaction=viewrecord&id=L2004097675&from=export |
  | Volume | 33 |
  | Pages | 37-52 |
  | Publication | Anthrozoos |
  | DOI | 10.1080/08927936.2020.1694310 |
  | Issue | 1 |
  | Journal Abbr | Anthrozoos |
  | ISSN | 1753-0377 |
  | Date Added | 11/07/2025, 12:10:53 |
  | Modified | 11/07/2025, 12:10:53 |

  ### Tags:

  - oxytocin
  - heart rate variability
  - physiology
  - dog
  - article
  - female
  - male
  - nonhuman
  - human
  - adult
  - aged
  - ELISA kit
  - hormone determination
  - crossover procedure
  - human experiment
  - normal human
  - emotional attachment
  - questionnaire
  - clinical trial
  - monash dog owner relationship scale
  - enzyme linked immunosorbent assay
  - hormone response
  - ACTRN12617000966392
  - autonomic nervous system function
  - dog walking
  - human dog bond questionnaire
  - organismal interaction

  ### Attachments

  - Full Text (HTML)
- ## Effects of Human–Dog Interactions on Salivary Oxytocin Concentrations and Heart Rate Variability: A Four-Condition Cross-Over Trial

  |  |  |
  | --- | --- |
  | Item Type | Journal Article |
  | Author | L. Powell |
  | Author | K.M. Edwards |
  | Author | S. Michael |
  | Author | P. McGreevy |
  | Author | A. Bauman |
  | Author | A.J. Guastella |
  | Author | B. Drayton |
  | Author | E. Stamatakis |
  | Abstract | Dog ownership is often advocated for its potential benefits to human health, with changes to oxytocin and autonomic nervous system (ANS) activity proposed as possible underlying mechanisms. The aims of the current study were to a) examine the effect of two common types of human–dog interactions (dog-walking and affiliative interactions) on salivary oxytocin concentrations and heart rate variability (HRV, an indicator of ANS activity), and b) investigate any putative moderating role for the strength of human–dog attachment on such responses. Twenty-nine dog owners completed a four-condition random-order cross-over trial: dog-walking (DW); walking without the dog (W); affiliative human–dog interaction (H-DI); and resting without the dog (C). Each condition was performed for approximately 15 minutes. Saliva and HRV samples were collected before and after each condition. Linear mixed models were used to analyze data, with the participant considered a random effect; condition, order of conditions, and condition duration as fixed effects. Oxytocin concentrations were not significantly different following any of the four conditions. HRV was significantly reduced following DW (mean change HF HRV = –0.37, 95% CI = –0.70, –0.04) and W (mean change HF HRV = –0.49, 95% CI = –0.81, –0.17). Considering moderation by the strength of the human–dog bond, pairwise comparisons revealed that, compared with W, DW elicited an increase in oxytocin concentrations (mean change 9.32 pg/mL, 95% CI = 6.52, 12.12) and HRV (mean change SDRR = 0.41, 95% CI = 0.19, 0.63) in owners with lower levels of attachment. These owners also displayed increased oxytocin concentrations following H-DI (mean change 3.90 pg/mL, 95% CI = 1.48, 6.32), compared with C. Overall, we did not find a consistent pattern for positive oxytocin or HRV responses to human–dog interactions. The strength of owner–dog attachment was found to have a moderating effect, suggesting that human–dog interactions may elicit greater physiological responses in low-attachment individuals. |
  | Date | 2020 |
  | Language | English |
  | Archive | Embase |
  | URL | https://www.embase.com/search/results?subaction=viewrecord&id=L2004097675&from=export |
  | Volume | 33 |
  | Pages | 37-52 |
  | Publication | Anthrozoos |
  | DOI | 10.1080/08927936.2020.1694310 |
  | Issue | 1 |
  | Journal Abbr | Anthrozoos |
  | ISSN | 1753-0377 |
  | Date Added | 11/07/2025, 12:13:30 |
  | Modified | 11/07/2025, 12:13:30 |

  ### Tags:

  - oxytocin
  - heart rate variability
  - physiology
  - dog
  - article
  - female
  - male
  - nonhuman
  - human
  - adult
  - aged
  - ELISA kit
  - hormone determination
  - crossover procedure
  - human experiment
  - normal human
  - emotional attachment
  - questionnaire
  - clinical trial
  - monash dog owner relationship scale
  - enzyme linked immunosorbent assay
  - hormone response
  - ACTRN12617000966392
  - autonomic nervous system function
  - dog walking
  - human dog bond questionnaire
  - organismal interaction

  ### Attachments

  - Full Text (HTML)
- ## Canine endogenous oxytocin responses to dog-walking and affiliative human–dog interactions

  |  |  |
  | --- | --- |
  | Item Type | Journal Article |
  | Author | L. Powell |
  | Author | K.M. Edwards |
  | Author | A. Bauman |
  | Author | A.J. Guastella |
  | Author | B. Drayton |
  | Author | E. Stamatakis |
  | Author | P. McGreevy |
  | Abstract | It is widely recognized that humans and dogs share a unique relationship. However, the biological mechanisms that may contribute to this bond between owners and their pet dogs are still unclear. As such, we measured the concentration of oxytocin, a hormone that is important in social bonding, in dogs before and after two different activities: dog-walking and human–dog interactions. We also investigated whether the strength of an owner’s attachment to their dog affected the dog’s oxytocin concentration. Contradicting our suppositions, the experiment showed that the concentration of dog oxytocin was not substantially different following either dog-walking or human–dog interactions. Additionally, the strength of the human–dog bond did not affect oxytocin concentrations. We suggest that more research is needed to fully understand the role of oxytocin in human–dog bonding. Several studies suggest human–dog interactions elicit a positive effect on canine oxytocin concentrations. However, empirical investigations are scant and the joint influence of human–dog interaction and physical activity remains unexplored. The aims of the current study were to (a) examine the canine endogenous oxytocin response to owner-led dog-walking and affiliative human–dog interactions and (b) investigate the moderating effect of the owner-reported strength of the human–dog bond on such responses. Twenty-six dogs took part in a random order cross-over trial, involving dog-walking and human–dog interactions. Urinary samples were collected before and after each condition. The data were analyzed using linear mixed models with condition, order of conditions, condition duration, and latency from initiation of condition to urine sample collection considered as fixed effects, and the participant was considered a random effect. Canine urinary oxytocin concentrations did not differ significantly following dog-walking (mean change: −14.66 pg/mg Cr; 95% CI: −47.22, 17.90) or affiliative human–dog interactions (mean change: 6.94 pg/mg Cr; 95% CI: −26.99, 40.87). The reported strength of the human–dog bond did not significantly moderate the canine oxytocin response to either experimental condition. Contrary to our hypothesis, we did not observe evidence for a positive oxytocin response to dog-walking or human–dog interactions. View Full-Text. |
  | Date | 2019 |
  | Language | English |
  | Archive | Embase |
  | URL | https://www.embase.com/search/results?subaction=viewrecord&id=L2001613644&from=export |
  | Volume | 9 |
  | Publication | Animals |
  | DOI | 10.3390/ani9020051 |
  | Issue | 2 |
  | Journal Abbr | Animals |
  | ISSN | 2076-2615 |
  | Date Added | 11/07/2025, 12:13:31 |
  | Modified | 11/07/2025, 12:13:31 |

  ### Tags:

  - oxytocin
  - physical activity
  - dog
  - animal experiment
  - article
  - female
  - male
  - nonhuman
  - human
  - controlled study
  - hormone determination
  - creatinine
  - crossover procedure
  - pet animal
  - questionnaire
  - clinical trial
  - human-animal bond
  - enzyme linked immunosorbent assay
  - social bonding
  - ACTRN12617000966392
  - dog walking
  - Monash Dog Owners Relationship Scale
  - urinalysis

  ### Attachments

  - Full Text (HTML)
- ## North Atlantic right whales: 2017-present

  |  |  |
  | --- | --- |
  | Item Type | Journal Article |
  | Author | A.L. Powell |
  | Date | 2021 |
  | Language | English |
  | Archive | Embase |
  | URL | https://www.embase.com/search/results?subaction=viewrecord&id=L2017972610&from=export |
  | Volume | 62 |
  | Pages | 765-766 |
  | Publication | Canadian Veterinary Journal |
  | Issue | 7 |
  | Journal Abbr | Can. Vet. J. |
  | ISSN | 0008-5286 |
  | Date Added | 11/07/2025, 14:16:52 |
  | Modified | 11/07/2025, 14:16:52 |

  ### Tags:

  - human-animal interaction
  - article
  - nonhuman
  - hydrocortisone
  - pain
  - veterinary medicine
  - autopsy
  - acoustics
  - arthritis
  - Atlantic Ocean
  - Cetacea
  - chronic wound
  - death
  - endangered species
  - glacier
  - governmental organization
  - joint degeneration
  - mobile phone
  - non-governmental organization
  - prematurity
  - scar formation
  - wasting syndrome
- ## North Atlantic right whales: 2017-present

  |  |  |
  | --- | --- |
  | Item Type | Journal Article |
  | Author | A.L. Powell |
  | Date | 2021 |
  | Language | English |
  | Archive | Embase |
  | URL | https://www.embase.com/search/results?subaction=viewrecord&id=L2017972610&from=export |
  | Volume | 62 |
  | Pages | 765-766 |
  | Publication | Canadian Veterinary Journal |
  | Issue | 7 |
  | Journal Abbr | Can. Vet. J. |
  | ISSN | 0008-5286 |
  | Date Added | 11/07/2025, 14:18:26 |
  | Modified | 11/07/2025, 14:18:26 |

  ### Tags:

  - human-animal interaction
  - article
  - nonhuman
  - hydrocortisone
  - pain
  - veterinary medicine
  - autopsy
  - acoustics
  - arthritis
  - Atlantic Ocean
  - Cetacea
  - chronic wound
  - death
  - endangered species
  - glacier
  - governmental organization
  - joint degeneration
  - mobile phone
  - non-governmental organization
  - prematurity
  - scar formation
  - wasting syndrome
- ## Exploring the Domestication Syndrome Hypothesis in Dogs: Pigmentation Does Not Predict Cortisol Levels

  |  |  |
  | --- | --- |
  | Item Type | Journal Article |
  | Author | J.M. Platzer |
  | Author | L.M. Gunter |
  | Author | E.N. Feuerbacher |
  | Abstract | Previous research has found connections between pigmentation, behavior, and the physiological stress response in both wild and domestic animals; however, to date, no extensive research has been devoted to answering these questions in domestic dogs. Modern dogs are exposed to a variety of stressors; one well-studied stressor is residing in an animal shelter. To explore the possible relationships between dogs’ responses to stress and their pigmentation, we conducted statistical analyses of the cortisol:creatinine ratios of 208 American shelter dogs as a function of their coat color/pattern, eumelanin pigmentation, or white spotting. These dogs had been enrolled in previous welfare studies investigating the effect of interventions during which they left the animal shelter and spent time with humans. In the current investigation, we visually phenotype dogs based on photographs in order to classify their pigmentation and then conduct post hoc analyses to examine whether they differentially experience stress as a function of pigmentation. We found that the dogs did not differ significantly in their urinary cortisol:creatinine ratios based on coat color/pattern, eumelanin pigmentation, or white spotting, either while they were residing in the animal shelter or during the human interaction intervention. These preliminary data suggest that pigmentation alone does not predict the stress responses of shelter dogs; however, due to the small sample size and retrospective nature of the study, more research is needed. |
  | Date | 2023 |
  | Language | English |
  | Archive | Embase |
  | URL | https://www.embase.com/search/results?subaction=viewrecord&id=L2026039034&from=export |
  | Volume | 13 |
  | Publication | Animals |
  | DOI | 10.3390/ani13193095 |
  | Issue | 19 |
  | Journal Abbr | Animals |
  | ISSN | 2076-2615 |
  | Date Added | 11/07/2025, 14:15:32 |
  | Modified | 11/07/2025, 14:15:32 |

  ### Tags:

  - human-animal interaction
  - dog
  - article
  - female
  - male
  - nonhuman
  - physiological stress
  - hydrocortisone
  - creatinine
  - phenotype
  - medical procedures
  - analytical parameters
  - black eumelanin pigmentation
  - blue eumelanin pigmentation
  - brief outing intervention
  - coat color
  - coat pattern
  - domestication syndrome hypothesis
  - eumelanin
  - eumelanin pigmentation
  - hypothesis
  - isabella eumelanin pigmentation
  - liver eumelanin pigmentation
  - morphology
  - pigmentation
  - urinary cortisol creatinine ratio
  - weeklong fostering intervention
  - white spotting

  ### Attachments

  - Full Text (HTML)
- ## Exploring the Domestication Syndrome Hypothesis in Dogs: Pigmentation Does Not Predict Cortisol Levels

  |  |  |
  | --- | --- |
  | Item Type | Journal Article |
  | Author | J.M. Platzer |
  | Author | L.M. Gunter |
  | Author | E.N. Feuerbacher |
  | Abstract | Previous research has found connections between pigmentation, behavior, and the physiological stress response in both wild and domestic animals; however, to date, no extensive research has been devoted to answering these questions in domestic dogs. Modern dogs are exposed to a variety of stressors; one well-studied stressor is residing in an animal shelter. To explore the possible relationships between dogs’ responses to stress and their pigmentation, we conducted statistical analyses of the cortisol:creatinine ratios of 208 American shelter dogs as a function of their coat color/pattern, eumelanin pigmentation, or white spotting. These dogs had been enrolled in previous welfare studies investigating the effect of interventions during which they left the animal shelter and spent time with humans. In the current investigation, we visually phenotype dogs based on photographs in order to classify their pigmentation and then conduct post hoc analyses to examine whether they differentially experience stress as a function of pigmentation. We found that the dogs did not differ significantly in their urinary cortisol:creatinine ratios based on coat color/pattern, eumelanin pigmentation, or white spotting, either while they were residing in the animal shelter or during the human interaction intervention. These preliminary data suggest that pigmentation alone does not predict the stress responses of shelter dogs; however, due to the small sample size and retrospective nature of the study, more research is needed. |
  | Date | 2023 |
  | Language | English |
  | Archive | Embase |
  | URL | https://www.embase.com/search/results?subaction=viewrecord&id=L2026039034&from=export |
  | Volume | 13 |
  | Publication | Animals |
  | DOI | 10.3390/ani13193095 |
  | Issue | 19 |
  | Journal Abbr | Animals |
  | ISSN | 2076-2615 |
  | Date Added | 11/07/2025, 14:16:42 |
  | Modified | 11/07/2025, 14:16:42 |

  ### Tags:

  - human-animal interaction
  - dog
  - article
  - female
  - male
  - nonhuman
  - physiological stress
  - hydrocortisone
  - creatinine
  - phenotype
  - medical procedures
  - analytical parameters
  - black eumelanin pigmentation
  - blue eumelanin pigmentation
  - brief outing intervention
  - coat color
  - coat pattern
  - domestication syndrome hypothesis
  - eumelanin
  - eumelanin pigmentation
  - hypothesis
  - isabella eumelanin pigmentation
  - liver eumelanin pigmentation
  - morphology
  - pigmentation
  - urinary cortisol creatinine ratio
  - weeklong fostering intervention
  - white spotting

  ### Attachments

  - Full Text (HTML)
- ## Exploring the Domestication Syndrome Hypothesis in Dogs: Pigmentation Does Not Predict Cortisol Levels

  |  |  |
  | --- | --- |
  | Item Type | Journal Article |
  | Author | J.M. Platzer |
  | Author | L.M. Gunter |
  | Author | E.N. Feuerbacher |
  | Abstract | Previous research has found connections between pigmentation, behavior, and the physiological stress response in both wild and domestic animals; however, to date, no extensive research has been devoted to answering these questions in domestic dogs. Modern dogs are exposed to a variety of stressors; one well-studied stressor is residing in an animal shelter. To explore the possible relationships between dogs’ responses to stress and their pigmentation, we conducted statistical analyses of the cortisol:creatinine ratios of 208 American shelter dogs as a function of their coat color/pattern, eumelanin pigmentation, or white spotting. These dogs had been enrolled in previous welfare studies investigating the effect of interventions during which they left the animal shelter and spent time with humans. In the current investigation, we visually phenotype dogs based on photographs in order to classify their pigmentation and then conduct post hoc analyses to examine whether they differentially experience stress as a function of pigmentation. We found that the dogs did not differ significantly in their urinary cortisol:creatinine ratios based on coat color/pattern, eumelanin pigmentation, or white spotting, either while they were residing in the animal shelter or during the human interaction intervention. These preliminary data suggest that pigmentation alone does not predict the stress responses of shelter dogs; however, due to the small sample size and retrospective nature of the study, more research is needed. |
  | Date | 2023 |
  | Language | English |
  | Archive | Embase |
  | URL | https://www.embase.com/search/results?subaction=viewrecord&id=L2026039034&from=export |
  | Volume | 13 |
  | Publication | Animals |
  | DOI | 10.3390/ani13193095 |
  | Issue | 19 |
  | Journal Abbr | Animals |
  | ISSN | 2076-2615 |
  | Date Added | 11/07/2025, 14:18:15 |
  | Modified | 11/07/2025, 14:18:15 |

  ### Tags:

  - human-animal interaction
  - dog
  - article
  - female
  - male
  - nonhuman
  - physiological stress
  - hydrocortisone
  - creatinine
  - phenotype
  - medical procedures
  - analytical parameters
  - black eumelanin pigmentation
  - blue eumelanin pigmentation
  - brief outing intervention
  - coat color
  - coat pattern
  - domestication syndrome hypothesis
  - eumelanin
  - eumelanin pigmentation
  - hypothesis
  - isabella eumelanin pigmentation
  - liver eumelanin pigmentation
  - morphology
  - pigmentation
  - urinary cortisol creatinine ratio
  - weeklong fostering intervention
  - white spotting

  ### Attachments

  - Full Text (HTML)
- ## Serum Oxytocin, Cortisol and Social Behavior in Calves: A Study in the Impossible Task Paradigm

  |  |  |
  | --- | --- |
  | Item Type | Journal Article |
  | Author | C. Pinelli |
  | Author | A. Scandurra |
  | Author | V. Mastellone |
  | Author | P. Iommelli |
  | Author | N. Musco |
  | Author | M.E. Pero |
  | Author | A. Di Lucrezia |
  | Author | D. Lotito |
  | Author | R. Tudisco |
  | Author | B. D’Aniello |
  | Author | F. Infascelli |
  | Author | P. Lombardi |
  | Abstract | In this study, we explored the correlations between circulating levels of oxytocin, cortisol, and different social behaviors toward humans in 26 Italian Red Pied calves (all females, with an average age of 174 ± 24 days) using the impossible task paradigm. This paradigm has proved fruitful in highlighting the effect of socialization on the willingness to interact with humans in several domesticated species. The test consists of the violation of an expectation (recovering food from an experimental apparatus) while a caregiver and a stranger are present. Immediately after the end of the test (less than one minute), blood was collected from the coccygeal vein. Statistics were performed by the Spearman’s rank correlation; significant differences were adjusted according to Bonferroni’s correction. Cortisol correlates positively (ρ = 0.565; p < 0.05) with the latency of behaviors directed at the caregiver, and the duration of behaviors directed at the apparatus correlates negatively with both the caregiver (ρ = −0.654; p < 0.05) and a stranger (ρ = −0.644; p < 0.05). Contrary to what is reported in the literature on cows, no correlations were found between oxytocin levels and direct behaviors toward the caregiver. This highlights a different behavioral strategy between calves and cows when placed in front of an impossible task. |
  | Date | 2023 |
  | Language | English |
  | Archive | Embase |
  | URL | https://www.embase.com/search/results?subaction=viewrecord&id=L2021767134&from=export |
  | Volume | 13 |
  | Publication | Animals |
  | DOI | 10.3390/ani13040646 |
  | Issue | 4 |
  | Journal Abbr | Animals |
  | ISSN | 2076-2615 |
  | Date Added | 11/07/2025, 12:12:18 |
  | Modified | 11/07/2025, 12:12:18 |

  ### Tags:

  - oxytocin
  - human-animal interaction
  - social behavior
  - animal experiment
  - article
  - female
  - nonhuman
  - human
  - hydrocortisone
  - controlled study
  - ELISA kit
  - dairy cattle
  - caregiver
  - reliability
  - animal tissue
  - enzyme linked immunosorbent assay
  - training
  - hydrocortisone blood level
  - gene expression
  - protein expression
  - socialization
  - calf (bovine)
  - DNA isolation
  - expectation
  - gene flow
  - interneuron
  - introgression
  - oxytocin blood level
  - sequence homology
  - vein

  ### Attachments

  - Full Text (HTML)
- ## Serum Oxytocin, Cortisol and Social Behavior in Calves: A Study in the Impossible Task Paradigm

  |  |  |
  | --- | --- |
  | Item Type | Journal Article |
  | Author | C. Pinelli |
  | Author | A. Scandurra |
  | Author | V. Mastellone |
  | Author | P. Iommelli |
  | Author | N. Musco |
  | Author | M.E. Pero |
  | Author | A. Di Lucrezia |
  | Author | D. Lotito |
  | Author | R. Tudisco |
  | Author | B. D’Aniello |
  | Author | F. Infascelli |
  | Author | P. Lombardi |
  | Abstract | In this study, we explored the correlations between circulating levels of oxytocin, cortisol, and different social behaviors toward humans in 26 Italian Red Pied calves (all females, with an average age of 174 ± 24 days) using the impossible task paradigm. This paradigm has proved fruitful in highlighting the effect of socialization on the willingness to interact with humans in several domesticated species. The test consists of the violation of an expectation (recovering food from an experimental apparatus) while a caregiver and a stranger are present. Immediately after the end of the test (less than one minute), blood was collected from the coccygeal vein. Statistics were performed by the Spearman’s rank correlation; significant differences were adjusted according to Bonferroni’s correction. Cortisol correlates positively (ρ = 0.565; p < 0.05) with the latency of behaviors directed at the caregiver, and the duration of behaviors directed at the apparatus correlates negatively with both the caregiver (ρ = −0.654; p < 0.05) and a stranger (ρ = −0.644; p < 0.05). Contrary to what is reported in the literature on cows, no correlations were found between oxytocin levels and direct behaviors toward the caregiver. This highlights a different behavioral strategy between calves and cows when placed in front of an impossible task. |
  | Date | 2023 |
  | Language | English |
  | Archive | Embase |
  | URL | https://www.embase.com/search/results?subaction=viewrecord&id=L2021767134&from=export |
  | Volume | 13 |
  | Publication | Animals |
  | DOI | 10.3390/ani13040646 |
  | Issue | 4 |
  | Journal Abbr | Animals |
  | ISSN | 2076-2615 |
  | Date Added | 11/07/2025, 12:13:19 |
  | Modified | 11/07/2025, 12:13:19 |

  ### Tags:

  - oxytocin
  - human-animal interaction
  - social behavior
  - animal experiment
  - article
  - female
  - nonhuman
  - human
  - hydrocortisone
  - controlled study
  - ELISA kit
  - dairy cattle
  - caregiver
  - reliability
  - animal tissue
  - enzyme linked immunosorbent assay
  - training
  - hydrocortisone blood level
  - gene expression
  - protein expression
  - socialization
  - calf (bovine)
  - DNA isolation
  - expectation
  - gene flow
  - interneuron
  - introgression
  - oxytocin blood level
  - sequence homology
  - vein

  ### Attachments

  - Full Text (HTML)
- ## Serum Oxytocin, Cortisol and Social Behavior in Calves: A Study in the Impossible Task Paradigm

  |  |  |
  | --- | --- |
  | Item Type | Journal Article |
  | Author | C. Pinelli |
  | Author | A. Scandurra |
  | Author | V. Mastellone |
  | Author | P. Iommelli |
  | Author | N. Musco |
  | Author | M.E. Pero |
  | Author | A. Di Lucrezia |
  | Author | D. Lotito |
  | Author | R. Tudisco |
  | Author | B. D’Aniello |
  | Author | F. Infascelli |
  | Author | P. Lombardi |
  | Abstract | In this study, we explored the correlations between circulating levels of oxytocin, cortisol, and different social behaviors toward humans in 26 Italian Red Pied calves (all females, with an average age of 174 ± 24 days) using the impossible task paradigm. This paradigm has proved fruitful in highlighting the effect of socialization on the willingness to interact with humans in several domesticated species. The test consists of the violation of an expectation (recovering food from an experimental apparatus) while a caregiver and a stranger are present. Immediately after the end of the test (less than one minute), blood was collected from the coccygeal vein. Statistics were performed by the Spearman’s rank correlation; significant differences were adjusted according to Bonferroni’s correction. Cortisol correlates positively (ρ = 0.565; p < 0.05) with the latency of behaviors directed at the caregiver, and the duration of behaviors directed at the apparatus correlates negatively with both the caregiver (ρ = −0.654; p < 0.05) and a stranger (ρ = −0.644; p < 0.05). Contrary to what is reported in the literature on cows, no correlations were found between oxytocin levels and direct behaviors toward the caregiver. This highlights a different behavioral strategy between calves and cows when placed in front of an impossible task. |
  | Date | 2023 |
  | Language | English |
  | Archive | Embase |
  | URL | https://www.embase.com/search/results?subaction=viewrecord&id=L2021767134&from=export |
  | Volume | 13 |
  | Publication | Animals |
  | DOI | 10.3390/ani13040646 |
  | Issue | 4 |
  | Journal Abbr | Animals |
  | ISSN | 2076-2615 |
  | Date Added | 11/07/2025, 14:15:35 |
  | Modified | 11/07/2025, 14:15:35 |

  ### Tags:

  - oxytocin
  - human-animal interaction
  - social behavior
  - animal experiment
  - article
  - female
  - nonhuman
  - human
  - hydrocortisone
  - controlled study
  - ELISA kit
  - dairy cattle
  - caregiver
  - reliability
  - animal tissue
  - enzyme linked immunosorbent assay
  - training
  - hydrocortisone blood level
  - gene expression
  - protein expression
  - socialization
  - calf (bovine)
  - DNA isolation
  - expectation
  - gene flow
  - interneuron
  - introgression
  - oxytocin blood level
  - sequence homology
  - vein

  ### Attachments

  - Full Text (HTML)
- ## Serum Oxytocin, Cortisol and Social Behavior in Calves: A Study in the Impossible Task Paradigm

  |  |  |
  | --- | --- |
  | Item Type | Journal Article |
  | Author | C. Pinelli |
  | Author | A. Scandurra |
  | Author | V. Mastellone |
  | Author | P. Iommelli |
  | Author | N. Musco |
  | Author | M.E. Pero |
  | Author | A. Di Lucrezia |
  | Author | D. Lotito |
  | Author | R. Tudisco |
  | Author | B. D’Aniello |
  | Author | F. Infascelli |
  | Author | P. Lombardi |
  | Abstract | In this study, we explored the correlations between circulating levels of oxytocin, cortisol, and different social behaviors toward humans in 26 Italian Red Pied calves (all females, with an average age of 174 ± 24 days) using the impossible task paradigm. This paradigm has proved fruitful in highlighting the effect of socialization on the willingness to interact with humans in several domesticated species. The test consists of the violation of an expectation (recovering food from an experimental apparatus) while a caregiver and a stranger are present. Immediately after the end of the test (less than one minute), blood was collected from the coccygeal vein. Statistics were performed by the Spearman’s rank correlation; significant differences were adjusted according to Bonferroni’s correction. Cortisol correlates positively (ρ = 0.565; p < 0.05) with the latency of behaviors directed at the caregiver, and the duration of behaviors directed at the apparatus correlates negatively with both the caregiver (ρ = −0.654; p < 0.05) and a stranger (ρ = −0.644; p < 0.05). Contrary to what is reported in the literature on cows, no correlations were found between oxytocin levels and direct behaviors toward the caregiver. This highlights a different behavioral strategy between calves and cows when placed in front of an impossible task. |
  | Date | 2023 |
  | Language | English |
  | Archive | Embase |
  | URL | https://www.embase.com/search/results?subaction=viewrecord&id=L2021767134&from=export |
  | Volume | 13 |
  | Publication | Animals |
  | DOI | 10.3390/ani13040646 |
  | Issue | 4 |
  | Journal Abbr | Animals |
  | ISSN | 2076-2615 |
  | Date Added | 11/07/2025, 14:16:45 |
  | Modified | 11/07/2025, 14:16:45 |

  ### Tags:

  - oxytocin
  - human-animal interaction
  - social behavior
  - animal experiment
  - article
  - female
  - nonhuman
  - human
  - hydrocortisone
  - controlled study
  - ELISA kit
  - dairy cattle
  - caregiver
  - reliability
  - animal tissue
  - enzyme linked immunosorbent assay
  - training
  - hydrocortisone blood level
  - gene expression
  - protein expression
  - socialization
  - calf (bovine)
  - DNA isolation
  - expectation
  - gene flow
  - interneuron
  - introgression
  - oxytocin blood level
  - sequence homology
  - vein

  ### Attachments

  - Full Text (HTML)
- ## Serum Oxytocin, Cortisol and Social Behavior in Calves: A Study in the Impossible Task Paradigm

  |  |  |
  | --- | --- |
  | Item Type | Journal Article |
  | Author | C. Pinelli |
  | Author | A. Scandurra |
  | Author | V. Mastellone |
  | Author | P. Iommelli |
  | Author | N. Musco |
  | Author | M.E. Pero |
  | Author | A. Di Lucrezia |
  | Author | D. Lotito |
  | Author | R. Tudisco |
  | Author | B. D’Aniello |
  | Author | F. Infascelli |
  | Author | P. Lombardi |
  | Abstract | In this study, we explored the correlations between circulating levels of oxytocin, cortisol, and different social behaviors toward humans in 26 Italian Red Pied calves (all females, with an average age of 174 ± 24 days) using the impossible task paradigm. This paradigm has proved fruitful in highlighting the effect of socialization on the willingness to interact with humans in several domesticated species. The test consists of the violation of an expectation (recovering food from an experimental apparatus) while a caregiver and a stranger are present. Immediately after the end of the test (less than one minute), blood was collected from the coccygeal vein. Statistics were performed by the Spearman’s rank correlation; significant differences were adjusted according to Bonferroni’s correction. Cortisol correlates positively (ρ = 0.565; p < 0.05) with the latency of behaviors directed at the caregiver, and the duration of behaviors directed at the apparatus correlates negatively with both the caregiver (ρ = −0.654; p < 0.05) and a stranger (ρ = −0.644; p < 0.05). Contrary to what is reported in the literature on cows, no correlations were found between oxytocin levels and direct behaviors toward the caregiver. This highlights a different behavioral strategy between calves and cows when placed in front of an impossible task. |
  | Date | 2023 |
  | Language | English |
  | Archive | Embase |
  | URL | https://www.embase.com/search/results?subaction=viewrecord&id=L2021767134&from=export |
  | Volume | 13 |
  | Publication | Animals |
  | DOI | 10.3390/ani13040646 |
  | Issue | 4 |
  | Journal Abbr | Animals |
  | ISSN | 2076-2615 |
  | Date Added | 11/07/2025, 14:18:19 |
  | Modified | 11/07/2025, 14:18:19 |

  ### Tags:

  - oxytocin
  - human-animal interaction
  - social behavior
  - animal experiment
  - article
  - female
  - nonhuman
  - human
  - hydrocortisone
  - controlled study
  - ELISA kit
  - dairy cattle
  - caregiver
  - reliability
  - animal tissue
  - enzyme linked immunosorbent assay
  - training
  - hydrocortisone blood level
  - gene expression
  - protein expression
  - socialization
  - calf (bovine)
  - DNA isolation
  - expectation
  - gene flow
  - interneuron
  - introgression
  - oxytocin blood level
  - sequence homology
  - vein

  ### Attachments

  - Full Text (HTML)
- ## Dog–Owner Relationship and Its Association with Social Cognition in French Bulldogs

  |  |  |
  | --- | --- |
  | Item Type | Journal Article |
  | Author | L. Peterca |
  | Author | E. Gobbo |
  | Author | M. Zupan Šemrov |
  | Abstract | Our understanding of social cognition in brachycephalic dog breeds is limited. This study focused specifically on French Bulldogs and hypothesized that a closer relationship between dog and owner would improve the dogs’ understanding of nonverbal cues, particularly pointing gestures. To investigate this, we tested twenty-six dogs and their owners in a two-way object choice test in which the familiar person pointed to the bowl. As understanding of pointing gestures is influenced by human–animal interaction, we used the Monash Dog Owner Relationship Scale (MDORS) to assess various aspects of the dog–owner relationship, including dog–owner interaction, emotional closeness, and negative aspects of dog ownership and measured salivary oxytocin levels. This study examined the dogs’ correct choices and their straight approach patterns to a reward bowl. Several factors influenced the two measures of social cognition, such as the age and sex of the dog, dog–owner interaction, emotional closeness, and the choice time (time it took the dog to reach the reward bowl), but also that neutered dogs and those with no training history showed better cognitive performance. We found a very high number of correct choices, which could be attributed to the owners’ high scores on the dog–owner interaction scale. Oxytocin showed no direct effect on these measures. To increase the generalizability of the results, we suggest future studies focus on more than one breed to capture the temporal development of the owner–dog relationship and social cognitive abilities. |
  | Date | 2025 |
  | Language | English |
  | Archive | Embase |
  | URL | https://www.embase.com/search/results?subaction=viewrecord&id=L2032908486&from=export |
  | Volume | 15 |
  | Publication | Animals |
  | DOI | 10.3390/ani15010017 |
  | Issue | 1 |
  | Journal Abbr | Animals |
  | ISSN | 2076-2615 |
  | Date Added | 11/07/2025, 12:12:14 |
  | Modified | 11/07/2025, 12:12:14 |

  ### Tags:

  - oxytocin
  - article
  - female
  - male
  - nonhuman
  - human
  - data analysis software
  - ELISA kit
  - scoring system
  - questionnaire
  - human-animal relation
  - bulldog
  - camera
  - cotton swab
  - monash dog owner Rrelationship scale
  - Nikon D5300 digital camera
  - saliva collector
  - Salivette
  - social cognition
  - STAT software
  - version 9.4 of the SAS System for Windows

  ### Attachments

  - Full Text (HTML)
- ## Dog–Owner Relationship and Its Association with Social Cognition in French Bulldogs

  |  |  |
  | --- | --- |
  | Item Type | Journal Article |
  | Author | L. Peterca |
  | Author | E. Gobbo |
  | Author | M. Zupan Šemrov |
  | Abstract | Our understanding of social cognition in brachycephalic dog breeds is limited. This study focused specifically on French Bulldogs and hypothesized that a closer relationship between dog and owner would improve the dogs’ understanding of nonverbal cues, particularly pointing gestures. To investigate this, we tested twenty-six dogs and their owners in a two-way object choice test in which the familiar person pointed to the bowl. As understanding of pointing gestures is influenced by human–animal interaction, we used the Monash Dog Owner Relationship Scale (MDORS) to assess various aspects of the dog–owner relationship, including dog–owner interaction, emotional closeness, and negative aspects of dog ownership and measured salivary oxytocin levels. This study examined the dogs’ correct choices and their straight approach patterns to a reward bowl. Several factors influenced the two measures of social cognition, such as the age and sex of the dog, dog–owner interaction, emotional closeness, and the choice time (time it took the dog to reach the reward bowl), but also that neutered dogs and those with no training history showed better cognitive performance. We found a very high number of correct choices, which could be attributed to the owners’ high scores on the dog–owner interaction scale. Oxytocin showed no direct effect on these measures. To increase the generalizability of the results, we suggest future studies focus on more than one breed to capture the temporal development of the owner–dog relationship and social cognitive abilities. |
  | Date | 2025 |
  | Language | English |
  | Archive | Embase |
  | URL | https://www.embase.com/search/results?subaction=viewrecord&id=L2032908486&from=export |
  | Volume | 15 |
  | Publication | Animals |
  | DOI | 10.3390/ani15010017 |
  | Issue | 1 |
  | Journal Abbr | Animals |
  | ISSN | 2076-2615 |
  | Date Added | 11/07/2025, 12:13:15 |
  | Modified | 11/07/2025, 12:13:15 |

  ### Tags:

  - oxytocin
  - article
  - female
  - male
  - nonhuman
  - human
  - data analysis software
  - ELISA kit
  - scoring system
  - questionnaire
  - human-animal relation
  - bulldog
  - camera
  - cotton swab
  - monash dog owner Rrelationship scale
  - Nikon D5300 digital camera
  - saliva collector
  - Salivette
  - social cognition
  - STAT software
  - version 9.4 of the SAS System for Windows

  ### Attachments

  - Full Text (HTML)
- ## Effect of Postweaning Handling Strategies on Welfare and Productive Traits in Lambs

  |  |  |
  | --- | --- |
  | Item Type | Journal Article |
  | Author | M. Pascual-Alonso |
  | Author | G.C. Miranda-de la Lama |
  | Author | L. Aguayo-Ulloa |
  | Author | L. Ezquerro |
  | Author | M. Villarroel |
  | Author | R.H. Marín |
  | Author | G.A. Maria |
  | Abstract | Postweaning management strategies that include an element of social enrichment may reduce weaning stress and improve welfare and productive performance. We analyzed the effect of postweaning handling strategies on welfare and production traits in lambs. After weaning, 36 lambs were assigned to 3 experimental groups with 12 lambs each (control [C], fattening with gentle human female contact [H], and fattening with 2 adult ewes [E]). The average daily gain (ADG) was estimated. Blood samples were taken, and infrared thermography was used to estimate stress variables. There were significant differences among treatments (in favor of alternative strategies) regarding production and stress variables (cortisol, glucose, and creatine kinase). The results suggest that the lambs handled gently during the fattening were less reactive and better able to modulate their physiological stress. The E group adapted better to acute stress than the C group but was less efficient in modulating chronic stress. Both treatments showed higher slaughter live weights and better ADGs compared with the control. The use of social enrichment at weaning, especially to establish a positive human–nonhuman animal bond, alleviates lamb weaning stress and improves welfare and performance. |
  | Date | 2015 |
  | Language | English |
  | Archive | Embase |
  | URL | https://www.embase.com/search/results?subaction=viewrecord&id=L53280155&from=export |
  | Volume | 18 |
  | Pages | 42-56 |
  | Publication | Journal of Applied Animal Welfare Science |
  | DOI | 10.1080/10888705.2014.941107 |
  | Issue | 1 |
  | Journal Abbr | J. Appl. Anim. Welf. Sci. |
  | ISSN | 1532-7604 |
  | Date Added | 11/07/2025, 14:18:39 |
  | Modified | 11/07/2025, 14:18:39 |

  ### Tags:

  - animal welfare
  - lamb
  - animal experiment
  - article
  - female
  - male
  - nonhuman
  - physiological stress
  - human
  - hydrocortisone
  - controlled study
  - weaning
  - human-animal bond
  - glucose
  - feeding behavior
  - creatine kinase
  - enzyme immunoassay
  - blood cell count
  - environmental enrichment
  - fatty acid
  - infrared photography

  ### Attachments

  - Full Text (HTML)
- ## Limited Short-Term Effects of Tactile Stimulation on the Welfare of Newborn Nellore Calves

  |  |  |
  | --- | --- |
  | Item Type | Journal Article |
  | Author | M. Parra Cerezo |
  | Author | V. Brusin |
  | Author | P.H. Esteves Trindade |
  | Author | A. Hernández |
  | Author | J. Jung |
  | Author | C. Berg |
  | Author | M.J.R. Paranhos da Costa |
  | Abstract | Previous results indicate that tactile stimulation enhances animal welfare and human well-being when applied at a young age. This study aimed to evaluate the effects of tactile stimulation on the welfare of Nellore calves. A total of 54 Nellore calves were observed, 28 of which received tactile stimulation (WTS) for ~60 s, while 26 did not (NTS). The calves’ behavior was assessed through body movements and facial expressions scoring. Heart rates (HRs) were recorded in three situations: when the calves were placed in lateral recumbency (HR1), during identification procedures (HR2), and after completion of identification procedures (HR3). Average daily gain and weaning weight adjusted to 240 days were calculated. Behavioral analyses revealed that most WTS calves exhibited positive emotional states and high excitability, whereas NTS calves displayed the opposite. WTS calves also had lower HR3 values and higher absolute difference between HR3 and HR1 (p < 0.06), as well as between HR3 and HR2 (p < 0.05) compared to NTS calves. However, long-term performance indicators did not differ between WTS and NTS calves (p > 0.05). We conclude that tactile stimulation during the initial handling of newborn Nellore calves likely promotes their short-term welfare, but only to a limited extent. |
  | Date | 2025 |
  | Language | English |
  | Archive | Embase |
  | URL | https://www.embase.com/search/results?subaction=viewrecord&id=L2034395708&from=export |
  | Volume | 12 |
  | Publication | Veterinary Sciences |
  | DOI | 10.3390/vetsci12040393 |
  | Issue | 4 |
  | Journal Abbr | Vet. Sci. |
  | ISSN | 2306-7381 |
  | Date Added | 11/07/2025, 11:53:15 |
  | Modified | 11/07/2025, 11:53:15 |

  ### Tags:

  - human-animal interaction
  - animal welfare
  - average daily gain
  - heart rate
  - cow
  - emotion
  - animal experiment
  - article
  - nonhuman
  - human
  - facial expression
  - recumbency
  - behavior assessment
  - body movement
  - bovine
  - excitability
  - newborn
  - subcutaneous drug administration
  - tactile stimulation
  - weaning

  ### Attachments

  - Full Text (HTML)
- ## Limited Short-Term Effects of Tactile Stimulation on the Welfare of Newborn Nellore Calves

  |  |  |
  | --- | --- |
  | Item Type | Journal Article |
  | Author | M. Parra Cerezo |
  | Author | V. Brusin |
  | Author | P.H. Esteves Trindade |
  | Author | A. Hernández |
  | Author | J. Jung |
  | Author | C. Berg |
  | Author | M.J.R. Paranhos da Costa |
  | Abstract | Previous results indicate that tactile stimulation enhances animal welfare and human well-being when applied at a young age. This study aimed to evaluate the effects of tactile stimulation on the welfare of Nellore calves. A total of 54 Nellore calves were observed, 28 of which received tactile stimulation (WTS) for ~60 s, while 26 did not (NTS). The calves’ behavior was assessed through body movements and facial expressions scoring. Heart rates (HRs) were recorded in three situations: when the calves were placed in lateral recumbency (HR1), during identification procedures (HR2), and after completion of identification procedures (HR3). Average daily gain and weaning weight adjusted to 240 days were calculated. Behavioral analyses revealed that most WTS calves exhibited positive emotional states and high excitability, whereas NTS calves displayed the opposite. WTS calves also had lower HR3 values and higher absolute difference between HR3 and HR1 (p < 0.06), as well as between HR3 and HR2 (p < 0.05) compared to NTS calves. However, long-term performance indicators did not differ between WTS and NTS calves (p > 0.05). We conclude that tactile stimulation during the initial handling of newborn Nellore calves likely promotes their short-term welfare, but only to a limited extent. |
  | Date | 2025 |
  | Language | English |
  | Archive | Embase |
  | URL | https://www.embase.com/search/results?subaction=viewrecord&id=L2034395708&from=export |
  | Volume | 12 |
  | Publication | Veterinary Sciences |
  | DOI | 10.3390/vetsci12040393 |
  | Issue | 4 |
  | Journal Abbr | Vet. Sci. |
  | ISSN | 2306-7381 |
  | Date Added | 11/07/2025, 12:10:36 |
  | Modified | 11/07/2025, 12:10:36 |

  ### Tags:

  - human-animal interaction
  - animal welfare
  - average daily gain
  - heart rate
  - cow
  - emotion
  - animal experiment
  - article
  - nonhuman
  - human
  - facial expression
  - recumbency
  - behavior assessment
  - body movement
  - bovine
  - excitability
  - newborn
  - subcutaneous drug administration
  - tactile stimulation
  - weaning

  ### Attachments

  - Full Text (HTML)
- ## Replication pilot trial of therapeutic horseback riding and cortisol collection with children on the autism spectrum

  |  |  |
  | --- | --- |
  | Item Type | Journal Article |
  | Author | Z. Pan |
  | Author | D.A. Granger |
  | Author | N.A. Guérin |
  | Author | A. Shoffner |
  | Author | R.L. Gabriels |
  | Abstract | We aimed to determine whether results of our prior randomized control trial [RCT; NCT02301195, (1)] of Therapeutic Horseback Riding (THR) for children and adolescents with autism spectrum disorder (ASD) could be replicated at a different riding center and if treatment effects also included differences in the expression of associations between problem behavior and the activity of the hypothalamic-pituitary-adrenal (HPA) axis. Participants with ASD (N = 16) ages 6-16 years were randomized by nonverbal intelligence quotient to either a 10-week THR group (n = 8) or no horse interaction barn activity (BA) control group (n = 8). Outcome measures were a standard speech-language sample and caregiver-report of aberrant and social behaviors. Participants' saliva was sampled weekly at a consistent afternoon time immediately pre- and 20 min' post-condition (later assayed for cortisol). Intent-to-treat analysis revealed that compared to controls, THR participants had significant improvements in hyperactivity, and social awareness, and significant improvements at the 0.1 significance level in irritability and social communication behaviors. There were no significant improvements in number of words or new words spoken during the standard language sample. Linear mixed effects model analysis indicated that greater weekly pre-lesson irritability levels were associated with smaller post-lesson reduction in salivary cortisol levels, and greater weekly pre-lesson hyperactivity levels were associated with smaller cortisol reduction in the THR group, but not in the BA control group. The findings represent a partial replication of prior results (1), extend prior observations to include THR effects on biobehavioral relationships and suggest that cortisol could be a target mediator for THR effects on irritability and hyperactivity behaviors in youth with ASD. |
  | Date | 2019 |
  | Language | English |
  | Archive | Embase |
  | URL | https://www.embase.com/search/results?subaction=viewrecord&id=L625982322&from=export |
  | Volume | 5 |
  | Publication | Frontiers in Veterinary Science |
  | DOI | 10.3389/fvets.2018.00312 |
  | Issue | JAN |
  | Journal Abbr | Front. Vet. Sci. |
  | ISSN | 2297-1769 |
  | Date Added | 11/07/2025, 14:18:34 |
  | Modified | 11/07/2025, 14:18:34 |

  ### Tags:

  - child
  - article
  - female
  - male
  - social interaction
  - human
  - hydrocortisone
  - controlled study
  - horseback riding
  - randomized controlled trial
  - adolescent
  - autism
  - clinical article
  - human-animal bond
  - hypothalamus hypophysis adrenal system
  - saliva level
  - social adaptation
  - agoraphobia
  - clinical outcome
  - hyperactivity
  - intelligence quotient
  - irritability
  - NCT02301195
  - panic
  - problem behavior
  - replication study
  - therapeutic horseback riding

  ### Attachments

  - Full Text (HTML)
- ## Influence of riders' skill on plasma cortisol levels of horses walking on forest and field trekking courses

  |  |  |
  | --- | --- |
  | Item Type | Journal Article |
  | Author | A. Ono |
  | Author | A. Matsuura |
  | Author | Y. Yamazaki |
  | Author | W. Sakai |
  | Author | K. Watanabe |
  | Author | T. Nakanowatari |
  | Author | H. Kobayashi |
  | Author | M. Irimajiri |
  | Author | K. Hodate |
  | Abstract | The aim of this study was to evaluate the influence of rider's skill on the plasma cortisol levels of trekking horses on two courses, walking on field and forest courses (about 4.5 to 5.1 km each). Three riders of different skills did horse trekking (HT) in a tandem line under a fixed order: advanced-leading, beginner-second and intermediate-last. A total of six horses were used and they experienced all positions in both courses; a total of 12 experiments were done. Blood samples were obtained before HT, immediately after and 2 h after HT. As a control, additional blood samples were obtained from the same horses on non-riding days. Irrespective of the course and the rider's skill, the cortisol level before HT was higher than that of control (P < 0.05). In both courses, the cortisol levels immediately after HT ridden by the advanced rider were higher than that of control (P < 0.05). However, in every case, the cortisol level 2 h after HT was closely similar to the level of the control. Thus, we concluded the stress of trekking horse was not sufficient to disturb the circadian rhythm of the cortisol level, irrespective of the course and the rider's skill. |
  | Date | 2017 |
  | Language | English |
  | Archive | Medline |
  | URL | https://www.embase.com/search/results?subaction=viewrecord&id=L619629538&from=export |
  | Volume | 88 |
  | Pages | 1629-1635 |
  | Publication | Animal science journal = Nihon chikusan Gakkaiho |
  | DOI | 10.1111/asj.12801 |
  | Issue | 10 |
  | Journal Abbr | Anim. Sci. J. |
  | ISSN | 1740-0929 |
  | Date Added | 11/07/2025, 14:18:37 |
  | Modified | 11/07/2025, 14:18:37 |

  ### Tags:

  - horse
  - physiology
  - human
  - hydrocortisone
  - animal
  - mental stress
  - psychology
  - motor performance
  - blood
  - human-animal bond
  - forest
  - pathophysiology
  - circadian rhythm
  - gait
  - sport
  - time factor
  - track and field

  ### Attachments

  - Full Text (HTML)
- ## No More Evasion: Redefining Conflict Behaviour in Human–Horse Interactions

  |  |  |
  | --- | --- |
  | Item Type | Journal Article |
  | Author | E. O’Connell |
  | Author | S. Dyson |
  | Author | A. McLean |
  | Author | P. McGreevy |
  | Abstract | Euphemisms, anthropomorphisms, and equivocation are established characteristics of traditional equestrian language. ‘Evasion’, ‘resistance’, and ‘disobedience’ are common labels assigned to unwelcome equine behaviours, implying that the horse is at fault for not complying with the human’s cues and expectations. These terms appear to overlook multiple motivations that may directly result in the horse offering unwelcome responses, which may then inadvertently be reinforced. This article revisits some of the anthropocentric inferences in these terms and explores the harmful consequences of such convenient but incorrect labels before proposing a redefinition of ‘conflict behaviour’ in human–horse interactions: Responses reflective of competing motivations for the horse that may exist on a continuum from subtle to overt, with frequencies that range from a singular momentary behavioural response to repetitive displays when motivational conflict is prolonged. Addressing how inadequate terms may mask pain, obscure the horse’s motivation, and deflect human culpability, this commentary highlights the merits of a multidisciplinary approach to terminology across equine research. Acknowledging that variables contributing to behaviour can be biological, environmental and anthropogenic, it emphasises the need for more investigation into the relationships between equicentric motivations reflecting equine telos and problematic horse behaviours. |
  | Date | 2025 |
  | Language | English |
  | Archive | Embase |
  | URL | https://www.embase.com/search/results?subaction=viewrecord&id=L2033181853&from=export |
  | Volume | 15 |
  | Publication | Animals |
  | DOI | 10.3390/ani15030399 |
  | Issue | 3 |
  | Journal Abbr | Animals |
  | ISSN | 2076-2615 |
  | Date Added | 11/07/2025, 11:53:16 |
  | Modified | 11/07/2025, 11:53:16 |

  ### Tags:

  - human-animal interaction
  - horse
  - behavior
  - heart rate
  - cognition
  - nonhuman
  - human
  - note
  - adaptive immunity
  - anthropocentrism
  - anthropomorphism
  - biochemistry
  - breathing rate
  - conflict
  - deep learning
  - human horse interaction
  - immune evasion
  - learning
  - learning theory
  - pain
  - veterinary medicine

  ### Attachments

  - Full Text (HTML)
- ## No More Evasion: Redefining Conflict Behaviour in Human–Horse Interactions

  |  |  |
  | --- | --- |
  | Item Type | Journal Article |
  | Author | E. O’Connell |
  | Author | S. Dyson |
  | Author | A. McLean |
  | Author | P. McGreevy |
  | Abstract | Euphemisms, anthropomorphisms, and equivocation are established characteristics of traditional equestrian language. ‘Evasion’, ‘resistance’, and ‘disobedience’ are common labels assigned to unwelcome equine behaviours, implying that the horse is at fault for not complying with the human’s cues and expectations. These terms appear to overlook multiple motivations that may directly result in the horse offering unwelcome responses, which may then inadvertently be reinforced. This article revisits some of the anthropocentric inferences in these terms and explores the harmful consequences of such convenient but incorrect labels before proposing a redefinition of ‘conflict behaviour’ in human–horse interactions: Responses reflective of competing motivations for the horse that may exist on a continuum from subtle to overt, with frequencies that range from a singular momentary behavioural response to repetitive displays when motivational conflict is prolonged. Addressing how inadequate terms may mask pain, obscure the horse’s motivation, and deflect human culpability, this commentary highlights the merits of a multidisciplinary approach to terminology across equine research. Acknowledging that variables contributing to behaviour can be biological, environmental and anthropogenic, it emphasises the need for more investigation into the relationships between equicentric motivations reflecting equine telos and problematic horse behaviours. |
  | Date | 2025 |
  | Language | English |
  | Archive | Embase |
  | URL | https://www.embase.com/search/results?subaction=viewrecord&id=L2033181853&from=export |
  | Volume | 15 |
  | Publication | Animals |
  | DOI | 10.3390/ani15030399 |
  | Issue | 3 |
  | Journal Abbr | Animals |
  | ISSN | 2076-2615 |
  | Date Added | 11/07/2025, 12:10:37 |
  | Modified | 11/07/2025, 12:10:37 |

  ### Tags:

  - human-animal interaction
  - horse
  - behavior
  - heart rate
  - cognition
  - nonhuman
  - human
  - note
  - adaptive immunity
  - anthropocentrism
  - anthropomorphism
  - biochemistry
  - breathing rate
  - conflict
  - deep learning
  - human horse interaction
  - immune evasion
  - learning
  - learning theory
  - pain
  - veterinary medicine

  ### Attachments

  - Full Text (HTML)
- ## Influence of Interactive Behaviors Induced by a Therapy Dog and Her Handler on the Physiology of Residents in Nursing Homes: An Exploratory Study

  |  |  |
  | --- | --- |
  | Item Type | Journal Article |
  | Author | A. Nilsson |
  | Author | L. Lidfors |
  | Author | A. Wichman |
  | Author | L. Handlin |
  | Author | M. Petersson |
  | Author | K. Uvnäs-Moberg |
  | Abstract | The aim of this exploratory study was to investigate interactive behaviors performed between residents at nursing homes and a therapy dog and her handler and explore if they influenced residents’ physiological variables such as fingertip temperature, heart rate, and systolic and diastolic blood pressure. The therapy dog–handler team visited 12 older people at three nursing homes for 60 min twice a week during a four-week period. The visits were videotaped, and the duration of interactive behaviors was recorded. The physiological variables were measured before (0 min) and after (60 min) the interaction between the residents and the dog–handler team, and the delta value was calculated. The interactive behaviors during the first two and the last two weeks were as follows: the resident looking at the dog (799 and 697 s/h), the resident in physical contact with the dog (183 and 109 s/h, p < 0.001, Wilcoxon signed-rank test), the resident playing with the dog (123 and 126 s/h), the resident talking with others (559 and 511 s/h), and the dog handler having physical contact with the resident (822 and 764 s/h). The mean values for fingertip temperature, heart rate, and systolic and diastolic blood pressure did not differ significantly between the first and two last weeks (paired t-test). However, the delta values varied largely between the different residents. The more physical contact the residents had with the dog handler, the more the fingertip temperature increased (p < 0.05, mixed linear model). The duration of physical contact between the residents and the dog tended to be associated with an increased fingertip temperature (p < 0.1). Furthermore, the more the residents were in verbal contact with the dog handler, the more their heart rate decreased (p < 0.05). These results demonstrate some associations between specific interactive behaviors and physiological changes in residents in connection with visits by a dog–handler team. |
  | Date | 2024 |
  | Language | English |
  | Archive | Embase |
  | URL | https://www.embase.com/search/results?subaction=viewrecord&id=L2026875211&from=export |
  | Volume | 37 |
  | Pages | 323-342 |
  | Publication | Anthrozoos |
  | DOI | 10.1080/08927936.2023.2280374 |
  | Issue | 2 |
  | Journal Abbr | Anthrozoos |
  | ISSN | 1753-0377 |
  | Date Added | 11/07/2025, 11:53:20 |
  | Modified | 11/07/2025, 11:53:20 |

  ### Tags:

  - human-animal interaction
  - behavior
  - heart rate
  - dog
  - therapy
  - article
  - female
  - male
  - human
  - adult
  - aged
  - adrenergic system
  - diastolic blood pressure
  - nursing home
  - temperature
  - Wilcoxon signed ranks test

  ### Attachments

  - Full Text (HTML)
- ## Influence of Interactive Behaviors Induced by a Therapy Dog and Her Handler on the Physiology of Residents in Nursing Homes: An Exploratory Study

  |  |  |
  | --- | --- |
  | Item Type | Journal Article |
  | Author | A. Nilsson |
  | Author | L. Lidfors |
  | Author | A. Wichman |
  | Author | L. Handlin |
  | Author | M. Petersson |
  | Author | K. Uvnäs-Moberg |
  | Abstract | The aim of this exploratory study was to investigate interactive behaviors performed between residents at nursing homes and a therapy dog and her handler and explore if they influenced residents’ physiological variables such as fingertip temperature, heart rate, and systolic and diastolic blood pressure. The therapy dog–handler team visited 12 older people at three nursing homes for 60 min twice a week during a four-week period. The visits were videotaped, and the duration of interactive behaviors was recorded. The physiological variables were measured before (0 min) and after (60 min) the interaction between the residents and the dog–handler team, and the delta value was calculated. The interactive behaviors during the first two and the last two weeks were as follows: the resident looking at the dog (799 and 697 s/h), the resident in physical contact with the dog (183 and 109 s/h, p < 0.001, Wilcoxon signed-rank test), the resident playing with the dog (123 and 126 s/h), the resident talking with others (559 and 511 s/h), and the dog handler having physical contact with the resident (822 and 764 s/h). The mean values for fingertip temperature, heart rate, and systolic and diastolic blood pressure did not differ significantly between the first and two last weeks (paired t-test). However, the delta values varied largely between the different residents. The more physical contact the residents had with the dog handler, the more the fingertip temperature increased (p < 0.05, mixed linear model). The duration of physical contact between the residents and the dog tended to be associated with an increased fingertip temperature (p < 0.1). Furthermore, the more the residents were in verbal contact with the dog handler, the more their heart rate decreased (p < 0.05). These results demonstrate some associations between specific interactive behaviors and physiological changes in residents in connection with visits by a dog–handler team. |
  | Date | 2024 |
  | Language | English |
  | Archive | Embase |
  | URL | https://www.embase.com/search/results?subaction=viewrecord&id=L2026875211&from=export |
  | Volume | 37 |
  | Pages | 323-342 |
  | Publication | Anthrozoos |
  | DOI | 10.1080/08927936.2023.2280374 |
  | Issue | 2 |
  | Journal Abbr | Anthrozoos |
  | ISSN | 1753-0377 |
  | Date Added | 11/07/2025, 12:10:42 |
  | Modified | 11/07/2025, 12:10:42 |

  ### Tags:

  - human-animal interaction
  - behavior
  - heart rate
  - dog
  - therapy
  - article
  - female
  - male
  - human
  - adult
  - aged
  - adrenergic system
  - diastolic blood pressure
  - nursing home
  - temperature
  - Wilcoxon signed ranks test

  ### Attachments

  - Full Text (HTML)
- ## Attachment-like behavioral expressions to humans in puppies are related to oxytocin and cortisol: A comparative study of Akitas and Labrador Retrievers

  |  |  |
  | --- | --- |
  | Item Type | Journal Article |
  | Author | M. Nagasawa |
  | Author | S. Tomori |
  | Author | K. Mogi |
  | Author | T. Kikusui |
  | Abstract | This study investigated the relationship between urinary hormone concentrations and attachment-related behaviors in two dog breeds, the Akitas and Labrador Retrievers, to elucidate the hormonal and behavioral mechanisms underlying domestication and interspecies attachment to humans. By measuring cortisol and oxytocin concentrations, and conducting the Strange Situation Test (SST), we aimed to investigate breed differences in endocrine secretions associated with domestication and how these differences influence dog behavior toward humans. Our results showed significant breed differences in urinary cortisol concentrations, with Akitas exhibiting higher levels than Labrador Retrievers. This suggests a breed-specific stress response related to genetic proximity to wolves. However, oxytocin concentrations did not differ significantly, which suggests a complex interplay between factors influencing the domestication process and the formation of attachment behaviors. Behavioral observations during the SST revealed breed-specific patterns, with Labrador Retrievers showing more playful and attachment-like behaviors and Akitas showing more exploratory and passive behaviors. The study found correlations between hormones and behaviors within breeds, particularly in Labrador Retrievers, where oxytocin concentrations were associated with attachment-like behaviors, and cortisol concentrations reflected individual differences in physical activity rather than stress responses to social situations. These findings contribute to the understanding of the evolutionary and adaptive processes underlying the ability of domestic dogs to form close relationships with humans while highlighting the role of hormonal mechanisms in mediating attachment behaviors and the influence of breed-specific genetic backgrounds on these processes. |
  | Date | 2024 |
  | Language | English |
  | Archive | Embase |
  | URL | https://www.embase.com/search/results?subaction=viewrecord&id=L2031888249&from=export |
  | Volume | 177 |
  | Publication | Peptides |
  | DOI | 10.1016/j.peptides.2024.171224 |
  | Issue | (Nagasawa M.; Tomori S.; Mogi K.; Kikusui T., kikusui@azabu-u.ac.jp) Department of Animal Science and Biotechnology, Azabu University, Japan |
  | Journal Abbr | Peptides |
  | ISSN | 1873-5169 |
  | Date Added | 11/07/2025, 12:12:15 |
  | Modified | 11/07/2025, 12:12:15 |

  ### Tags:

  - oxytocin
  - human-animal interaction
  - physical activity
  - animal behavior
  - animal experiment
  - article
  - female
  - male
  - nonhuman
  - physiological stress
  - hydrocortisone
  - adult
  - controlled study
  - hormone determination
  - hydrocortisone urine level
  - emotional attachment
  - hormone urine level
  - akita dog
  - breed difference
  - dog breed
  - domestication
  - exploratory behavior
  - genetic background
  - Labrador retriever
  - puppy

  ### Attachments

  - Full Text (HTML)
- ## Attachment-like behavioral expressions to humans in puppies are related to oxytocin and cortisol: A comparative study of Akitas and Labrador Retrievers

  |  |  |
  | --- | --- |
  | Item Type | Journal Article |
  | Author | M. Nagasawa |
  | Author | S. Tomori |
  | Author | K. Mogi |
  | Author | T. Kikusui |
  | Abstract | This study investigated the relationship between urinary hormone concentrations and attachment-related behaviors in two dog breeds, the Akitas and Labrador Retrievers, to elucidate the hormonal and behavioral mechanisms underlying domestication and interspecies attachment to humans. By measuring cortisol and oxytocin concentrations, and conducting the Strange Situation Test (SST), we aimed to investigate breed differences in endocrine secretions associated with domestication and how these differences influence dog behavior toward humans. Our results showed significant breed differences in urinary cortisol concentrations, with Akitas exhibiting higher levels than Labrador Retrievers. This suggests a breed-specific stress response related to genetic proximity to wolves. However, oxytocin concentrations did not differ significantly, which suggests a complex interplay between factors influencing the domestication process and the formation of attachment behaviors. Behavioral observations during the SST revealed breed-specific patterns, with Labrador Retrievers showing more playful and attachment-like behaviors and Akitas showing more exploratory and passive behaviors. The study found correlations between hormones and behaviors within breeds, particularly in Labrador Retrievers, where oxytocin concentrations were associated with attachment-like behaviors, and cortisol concentrations reflected individual differences in physical activity rather than stress responses to social situations. These findings contribute to the understanding of the evolutionary and adaptive processes underlying the ability of domestic dogs to form close relationships with humans while highlighting the role of hormonal mechanisms in mediating attachment behaviors and the influence of breed-specific genetic backgrounds on these processes. |
  | Date | 2024 |
  | Language | English |
  | Archive | Embase |
  | URL | https://www.embase.com/search/results?subaction=viewrecord&id=L2031888249&from=export |
  | Volume | 177 |
  | Publication | Peptides |
  | DOI | 10.1016/j.peptides.2024.171224 |
  | Issue | (Nagasawa M.; Tomori S.; Mogi K.; Kikusui T., kikusui@azabu-u.ac.jp) Department of Animal Science and Biotechnology, Azabu University, Japan |
  | Journal Abbr | Peptides |
  | ISSN | 1873-5169 |
  | Date Added | 11/07/2025, 12:13:17 |
  | Modified | 11/07/2025, 12:13:17 |

  ### Tags:

  - oxytocin
  - human-animal interaction
  - physical activity
  - animal behavior
  - animal experiment
  - article
  - female
  - male
  - nonhuman
  - physiological stress
  - hydrocortisone
  - adult
  - controlled study
  - hormone determination
  - hydrocortisone urine level
  - emotional attachment
  - hormone urine level
  - akita dog
  - breed difference
  - dog breed
  - domestication
  - exploratory behavior
  - genetic background
  - Labrador retriever
  - puppy

  ### Attachments

  - Full Text (HTML)
- ## Attachment-like behavioral expressions to humans in puppies are related to oxytocin and cortisol: A comparative study of Akitas and Labrador Retrievers

  |  |  |
  | --- | --- |
  | Item Type | Journal Article |
  | Author | M. Nagasawa |
  | Author | S. Tomori |
  | Author | K. Mogi |
  | Author | T. Kikusui |
  | Abstract | This study investigated the relationship between urinary hormone concentrations and attachment-related behaviors in two dog breeds, the Akitas and Labrador Retrievers, to elucidate the hormonal and behavioral mechanisms underlying domestication and interspecies attachment to humans. By measuring cortisol and oxytocin concentrations, and conducting the Strange Situation Test (SST), we aimed to investigate breed differences in endocrine secretions associated with domestication and how these differences influence dog behavior toward humans. Our results showed significant breed differences in urinary cortisol concentrations, with Akitas exhibiting higher levels than Labrador Retrievers. This suggests a breed-specific stress response related to genetic proximity to wolves. However, oxytocin concentrations did not differ significantly, which suggests a complex interplay between factors influencing the domestication process and the formation of attachment behaviors. Behavioral observations during the SST revealed breed-specific patterns, with Labrador Retrievers showing more playful and attachment-like behaviors and Akitas showing more exploratory and passive behaviors. The study found correlations between hormones and behaviors within breeds, particularly in Labrador Retrievers, where oxytocin concentrations were associated with attachment-like behaviors, and cortisol concentrations reflected individual differences in physical activity rather than stress responses to social situations. These findings contribute to the understanding of the evolutionary and adaptive processes underlying the ability of domestic dogs to form close relationships with humans while highlighting the role of hormonal mechanisms in mediating attachment behaviors and the influence of breed-specific genetic backgrounds on these processes. |
  | Date | 2024 |
  | Language | English |
  | Archive | Embase |
  | URL | https://www.embase.com/search/results?subaction=viewrecord&id=L2031888249&from=export |
  | Volume | 177 |
  | Publication | Peptides |
  | DOI | 10.1016/j.peptides.2024.171224 |
  | Issue | (Nagasawa M.; Tomori S.; Mogi K.; Kikusui T., kikusui@azabu-u.ac.jp) Department of Animal Science and Biotechnology, Azabu University, Japan |
  | Journal Abbr | Peptides |
  | ISSN | 1873-5169 |
  | Date Added | 11/07/2025, 14:15:28 |
  | Modified | 11/07/2025, 14:15:28 |

  ### Tags:

  - oxytocin
  - human-animal interaction
  - physical activity
  - animal behavior
  - animal experiment
  - article
  - female
  - male
  - nonhuman
  - physiological stress
  - hydrocortisone
  - adult
  - controlled study
  - hormone determination
  - hydrocortisone urine level
  - emotional attachment
  - hormone urine level
  - akita dog
  - breed difference
  - dog breed
  - domestication
  - exploratory behavior
  - genetic background
  - Labrador retriever
  - puppy

  ### Attachments

  - Full Text (HTML)
- ## Attachment-like behavioral expressions to humans in puppies are related to oxytocin and cortisol: A comparative study of Akitas and Labrador Retrievers

  |  |  |
  | --- | --- |
  | Item Type | Journal Article |
  | Author | M. Nagasawa |
  | Author | S. Tomori |
  | Author | K. Mogi |
  | Author | T. Kikusui |
  | Abstract | This study investigated the relationship between urinary hormone concentrations and attachment-related behaviors in two dog breeds, the Akitas and Labrador Retrievers, to elucidate the hormonal and behavioral mechanisms underlying domestication and interspecies attachment to humans. By measuring cortisol and oxytocin concentrations, and conducting the Strange Situation Test (SST), we aimed to investigate breed differences in endocrine secretions associated with domestication and how these differences influence dog behavior toward humans. Our results showed significant breed differences in urinary cortisol concentrations, with Akitas exhibiting higher levels than Labrador Retrievers. This suggests a breed-specific stress response related to genetic proximity to wolves. However, oxytocin concentrations did not differ significantly, which suggests a complex interplay between factors influencing the domestication process and the formation of attachment behaviors. Behavioral observations during the SST revealed breed-specific patterns, with Labrador Retrievers showing more playful and attachment-like behaviors and Akitas showing more exploratory and passive behaviors. The study found correlations between hormones and behaviors within breeds, particularly in Labrador Retrievers, where oxytocin concentrations were associated with attachment-like behaviors, and cortisol concentrations reflected individual differences in physical activity rather than stress responses to social situations. These findings contribute to the understanding of the evolutionary and adaptive processes underlying the ability of domestic dogs to form close relationships with humans while highlighting the role of hormonal mechanisms in mediating attachment behaviors and the influence of breed-specific genetic backgrounds on these processes. |
  | Date | 2024 |
  | Language | English |
  | Archive | Embase |
  | URL | https://www.embase.com/search/results?subaction=viewrecord&id=L2031888249&from=export |
  | Volume | 177 |
  | Publication | Peptides |
  | DOI | 10.1016/j.peptides.2024.171224 |
  | Issue | (Nagasawa M.; Tomori S.; Mogi K.; Kikusui T., kikusui@azabu-u.ac.jp) Department of Animal Science and Biotechnology, Azabu University, Japan |
  | Journal Abbr | Peptides |
  | ISSN | 1873-5169 |
  | Date Added | 11/07/2025, 14:16:38 |
  | Modified | 11/07/2025, 14:16:38 |

  ### Tags:

  - oxytocin
  - human-animal interaction
  - physical activity
  - animal behavior
  - animal experiment
  - article
  - female
  - male
  - nonhuman
  - physiological stress
  - hydrocortisone
  - adult
  - controlled study
  - hormone determination
  - hydrocortisone urine level
  - emotional attachment
  - hormone urine level
  - akita dog
  - breed difference
  - dog breed
  - domestication
  - exploratory behavior
  - genetic background
  - Labrador retriever
  - puppy

  ### Attachments

  - Full Text (HTML)
- ## The Urinary Hormonal State of Cats Associated With Social Interaction With Humans

  |  |  |
  | --- | --- |
  | Item Type | Journal Article |
  | Author | T. Nagasawa |
  | Author | M. Ohta |
  | Author | H. Uchiyama |
  | Abstract | Research to assess the relationship between cats and humans is in a nascent stage. Some studies have assessed the stress status in cats using physiological indicators, such as the cortisol hormone, but have not focused on the social interaction with humans. Moreover, the role of oxytocin secretion in the relationship between cats and humans remains unclear. In this study, we determined the possibility of quantifying the urinary concentration of oxytocin in cats and assessed the effects of social contact with humans on the levels of urinary oxytocin and cortisol metabolite. Four cats were subjected to two conditions, namely, social (control), and non-social (no social contact with humans) conditions. The levels of cortisol and oxytocin metabolite in urine samples from the cats in both conditions were determined using enzyme-linked immunosorbent assays. The urinary concentrations of cortisol and oxytocin under the non-social condition were significantly higher than those under the social condition. In addition, the concentration of oxytocin significantly correlated with that of cortisol in cats under the non-social condition. In this study, it was possible to quantify the concentration of oxytocin in the urine of cats, and the obtained results suggest that cats recognize the social interaction with humans as important. This information might contribute to the establishment of an assessment method for the welfare of cats and might help in clarifying the relationship between cats and humans. |
  | Date | 2021 |
  | Language | English |
  | Archive | Embase |
  | URL | https://www.embase.com/search/results?subaction=viewrecord&id=L635658582&from=export |
  | Volume | 8 |
  | Publication | Frontiers in Veterinary Science |
  | DOI | 10.3389/fvets.2021.680843 |
  | Issue | (Nagasawa T.; Ohta M.) Department of Human and Animal-Plant Relationships, Graduate School of Agriculture, Tokyo University of Agriculture, Atsugi, Japan |
  | Journal Abbr | Front. Vet. Sci. |
  | ISSN | 2297-1769 |
  | Date Added | 11/07/2025, 14:16:52 |
  | Modified | 11/07/2025, 14:16:52 |

  ### Tags:

  - oxytocin
  - animal welfare
  - animal experiment
  - article
  - female
  - male
  - nonhuman
  - social interaction
  - human
  - hydrocortisone
  - controlled study
  - ELISA kit
  - hydrocortisone urine level
  - urine sampling
  - human-animal bond
  - enzyme linked immunosorbent assay
  - quantitative analysis
  - cat
  - hormone urine level
  - oxytocin release
  - social status

  ### Attachments

  - Full Text (HTML)
- ## Attachment-like behavioral expressions to humans in puppies are related to oxytocin and cortisol: A comparative study of Akitas and Labrador Retrievers

  |  |  |
  | --- | --- |
  | Item Type | Journal Article |
  | Author | M. Nagasawa |
  | Author | S. Tomori |
  | Author | K. Mogi |
  | Author | T. Kikusui |
  | Abstract | This study investigated the relationship between urinary hormone concentrations and attachment-related behaviors in two dog breeds, the Akitas and Labrador Retrievers, to elucidate the hormonal and behavioral mechanisms underlying domestication and interspecies attachment to humans. By measuring cortisol and oxytocin concentrations, and conducting the Strange Situation Test (SST), we aimed to investigate breed differences in endocrine secretions associated with domestication and how these differences influence dog behavior toward humans. Our results showed significant breed differences in urinary cortisol concentrations, with Akitas exhibiting higher levels than Labrador Retrievers. This suggests a breed-specific stress response related to genetic proximity to wolves. However, oxytocin concentrations did not differ significantly, which suggests a complex interplay between factors influencing the domestication process and the formation of attachment behaviors. Behavioral observations during the SST revealed breed-specific patterns, with Labrador Retrievers showing more playful and attachment-like behaviors and Akitas showing more exploratory and passive behaviors. The study found correlations between hormones and behaviors within breeds, particularly in Labrador Retrievers, where oxytocin concentrations were associated with attachment-like behaviors, and cortisol concentrations reflected individual differences in physical activity rather than stress responses to social situations. These findings contribute to the understanding of the evolutionary and adaptive processes underlying the ability of domestic dogs to form close relationships with humans while highlighting the role of hormonal mechanisms in mediating attachment behaviors and the influence of breed-specific genetic backgrounds on these processes. |
  | Date | 2024 |
  | Language | English |
  | Archive | Embase |
  | URL | https://www.embase.com/search/results?subaction=viewrecord&id=L2031888249&from=export |
  | Volume | 177 |
  | Publication | Peptides |
  | DOI | 10.1016/j.peptides.2024.171224 |
  | Issue | (Nagasawa M.; Tomori S.; Mogi K.; Kikusui T., kikusui@azabu-u.ac.jp) Department of Animal Science and Biotechnology, Azabu University, Japan |
  | Journal Abbr | Peptides |
  | ISSN | 1873-5169 |
  | Date Added | 11/07/2025, 14:18:11 |
  | Modified | 11/07/2025, 14:18:11 |

  ### Tags:

  - oxytocin
  - human-animal interaction
  - physical activity
  - animal behavior
  - animal experiment
  - article
  - female
  - male
  - nonhuman
  - physiological stress
  - hydrocortisone
  - adult
  - controlled study
  - hormone determination
  - hydrocortisone urine level
  - emotional attachment
  - hormone urine level
  - akita dog
  - breed difference
  - dog breed
  - domestication
  - exploratory behavior
  - genetic background
  - Labrador retriever
  - puppy

  ### Attachments

  - Full Text (HTML)
- ## The Urinary Hormonal State of Cats Associated With Social Interaction With Humans

  |  |  |
  | --- | --- |
  | Item Type | Journal Article |
  | Author | T. Nagasawa |
  | Author | M. Ohta |
  | Author | H. Uchiyama |
  | Abstract | Research to assess the relationship between cats and humans is in a nascent stage. Some studies have assessed the stress status in cats using physiological indicators, such as the cortisol hormone, but have not focused on the social interaction with humans. Moreover, the role of oxytocin secretion in the relationship between cats and humans remains unclear. In this study, we determined the possibility of quantifying the urinary concentration of oxytocin in cats and assessed the effects of social contact with humans on the levels of urinary oxytocin and cortisol metabolite. Four cats were subjected to two conditions, namely, social (control), and non-social (no social contact with humans) conditions. The levels of cortisol and oxytocin metabolite in urine samples from the cats in both conditions were determined using enzyme-linked immunosorbent assays. The urinary concentrations of cortisol and oxytocin under the non-social condition were significantly higher than those under the social condition. In addition, the concentration of oxytocin significantly correlated with that of cortisol in cats under the non-social condition. In this study, it was possible to quantify the concentration of oxytocin in the urine of cats, and the obtained results suggest that cats recognize the social interaction with humans as important. This information might contribute to the establishment of an assessment method for the welfare of cats and might help in clarifying the relationship between cats and humans. |
  | Date | 2021 |
  | Language | English |
  | Archive | Embase |
  | URL | https://www.embase.com/search/results?subaction=viewrecord&id=L635658582&from=export |
  | Volume | 8 |
  | Publication | Frontiers in Veterinary Science |
  | DOI | 10.3389/fvets.2021.680843 |
  | Issue | (Nagasawa T.; Ohta M.) Department of Human and Animal-Plant Relationships, Graduate School of Agriculture, Tokyo University of Agriculture, Atsugi, Japan |
  | Journal Abbr | Front. Vet. Sci. |
  | ISSN | 2297-1769 |
  | Date Added | 11/07/2025, 14:18:25 |
  | Modified | 11/07/2025, 14:18:25 |

  ### Tags:

  - oxytocin
  - animal welfare
  - animal experiment
  - article
  - female
  - male
  - nonhuman
  - social interaction
  - human
  - hydrocortisone
  - controlled study
  - ELISA kit
  - hydrocortisone urine level
  - urine sampling
  - human-animal bond
  - enzyme linked immunosorbent assay
  - quantitative analysis
  - cat
  - hormone urine level
  - oxytocin release
  - social status

  ### Attachments

  - Full Text (HTML)
- ## Positive human contact on the first day of life alters the piglet's behavioural response to humans and husbandry practices

  |  |  |
  | --- | --- |
  | Item Type | Journal Article |
  | Author | R. Muns |
  | Author | J.-L. Rault |
  | Author | P. Hemsworth |
  | Abstract | This experiment examined the effects of positive human contact at suckling on the first day of life on the behavioural and physiological responses of piglets to both humans and routine husbandry procedures. Forty litters from multiparous sows were randomly allocated to one of two treatments: Control (CC, minimal human interaction with day-old piglets) or Positive Contact (PC, human talking and caressing piglets during 6 suckling bouts on their first day of life, day 1). In each litter, 2 males and 2 females were randomly selected and their behavioural responses to tail docking (day 2), and to an experimenter (day 35) were studied. Escape behaviour at tail docking was assessed according to intensity (on a scale from 0 to 4 representing no movement to high intensity movement) and duration (on a scale from 0 to 3 representing no movement to continuous movement). At day 15 of age, a human approach and avoidance test was performed on focal piglets and at day 15, escape behaviour to capture before and after testing was recorded again. Blood samples for cortisol analysis were obtained from the focal piglets 30. min after tail docking and 1. h after weaning. Escape behaviour to tail docking of the PC piglets was of shorter duration than that of the CC piglets (P=0.05). There was a tendency for the escape behaviour both before and after testing at day 15 to be of a lower intensity (P=0.11 and P=0.06, respectively) and a shorter duration (P=0.06 and P=. 0.08, respectively) in the PC piglets. There was a tendency for PC piglets to have higher cortisol concentrations after tail docking than the CC piglets (P=0.07). Male piglets had higher cortisol concentrations after tail docking and after weaning than female piglets (P=0.02 and P=0.03). The results indicate that Positive Contact treatment reduced the duration of escape behaviour of piglets to tail docking. The role of classical conditioning, habituation and developmental changes in the observed effects of the Positive Contact treatment is unclear. Nonetheless, this experiment demonstrated that brief positive human contacts early in life can alter the behavioural responses of piglets to subsequent stressful events. |
  | Date | 2015 |
  | Language | English |
  | Archive | Embase |
  | URL | https://www.embase.com/search/results?subaction=viewrecord&id=L605287338&from=export |
  | Volume | 151 |
  | Pages | 162-167 |
  | Publication | Physiology and Behavior |
  | DOI | 10.1016/j.physbeh.2015.06.030 |
  | Issue | (Muns R., rmunsvila@gmail.com) Servei de Nutrició i Benestar Animal (SNiBA), Departament de Ciència Animal i dels Aliments, Facultat de Veterinària, Universitat Autònoma de Barcelona, Bellaterra, Spain |
  | Journal Abbr | Physiol. Behav. |
  | ISSN | 1873-507X |
  | Date Added | 11/07/2025, 14:18:40 |
  | Modified | 11/07/2025, 14:18:40 |

  ### Tags:

  - animal behavior
  - animal experiment
  - article
  - female
  - male
  - nonhuman
  - physiological stress
  - human
  - hydrocortisone
  - blood sampling
  - controlled study
  - weaning
  - human-animal bond
  - habituation
  - animal husbandry
  - hydrocortisone blood level
  - priority journal
  - personal experience
  - vocalization
  - avoidance behavior
  - piglet
  - escape behavior
  - suckling animal

  ### Attachments

  - Full Text (HTML)
- ## Tactile, Auditory, and Visual Stimulation as Sensory Enrichment for Dairy Cattle

  |  |  |
  | --- | --- |
  | Item Type | Journal Article |
  | Author | D. Mota-Rojas |
  | Author | A.L. Whittaker |
  | Author | A. Domínguez-Oliva |
  | Author | A.C. Strappini |
  | Author | A. Álvarez-Macías |
  | Author | P. Mora-Medina |
  | Author | M. Ghezzi |
  | Author | P. Lendez |
  | Author | K. Lezama-García |
  | Author | T. Grandin |
  | Abstract | Several types of enrichment can be used to improve animal welfare. This review summarizes the literature on the use of mechanical brushes, tactile udder stimulation, music, and visual stimuli as enrichment methods for dairy cows. Mechanical brushes and tactile stimulation of the udder have been shown to have a positive effect on milk yield and overall behavioral repertoire, enhancing natural behavior. Classical music reduces stress levels and has similarly been associated with increased milk yield. A slow or moderate tempo (70 to 100 bpm) at frequencies below 70 dB is recommended to have this positive effect. Evidence on the impacts of other types of enrichment, such as visual stimulation through mirrors, pictures, and color lights, or the use of olfactory stimuli, is equivocal and requires further study. |
  | Date | 2024 |
  | Language | English |
  | Archive | Embase |
  | URL | https://www.embase.com/search/results?subaction=viewrecord&id=L2029813120&from=export |
  | Volume | 14 |
  | Publication | Animals |
  | DOI | 10.3390/ani14091265 |
  | Issue | 9 |
  | Journal Abbr | Animals |
  | ISSN | 2076-2615 |
  | Date Added | 11/07/2025, 11:53:19 |
  | Modified | 11/07/2025, 11:53:19 |

  ### Tags:

  - heart rate
  - nonhuman
  - review
  - wellbeing
  - dairy cattle
  - milk yield
  - tactile stimulation
  - auditory cortex
  - auditory stimulation
  - Holstein cattle
  - Medline
  - ruminant
  - Scopus
  - visual stimulation
  - Web of Science

  ### Attachments

  - Full Text (HTML)
- ## Tactile, Auditory, and Visual Stimulation as Sensory Enrichment for Dairy Cattle

  |  |  |
  | --- | --- |
  | Item Type | Journal Article |
  | Author | D. Mota-Rojas |
  | Author | A.L. Whittaker |
  | Author | A. Domínguez-Oliva |
  | Author | A.C. Strappini |
  | Author | A. Álvarez-Macías |
  | Author | P. Mora-Medina |
  | Author | M. Ghezzi |
  | Author | P. Lendez |
  | Author | K. Lezama-García |
  | Author | T. Grandin |
  | Abstract | Several types of enrichment can be used to improve animal welfare. This review summarizes the literature on the use of mechanical brushes, tactile udder stimulation, music, and visual stimuli as enrichment methods for dairy cows. Mechanical brushes and tactile stimulation of the udder have been shown to have a positive effect on milk yield and overall behavioral repertoire, enhancing natural behavior. Classical music reduces stress levels and has similarly been associated with increased milk yield. A slow or moderate tempo (70 to 100 bpm) at frequencies below 70 dB is recommended to have this positive effect. Evidence on the impacts of other types of enrichment, such as visual stimulation through mirrors, pictures, and color lights, or the use of olfactory stimuli, is equivocal and requires further study. |
  | Date | 2024 |
  | Language | English |
  | Archive | Embase |
  | URL | https://www.embase.com/search/results?subaction=viewrecord&id=L2029813120&from=export |
  | Volume | 14 |
  | Publication | Animals |
  | DOI | 10.3390/ani14091265 |
  | Issue | 9 |
  | Journal Abbr | Animals |
  | ISSN | 2076-2615 |
  | Date Added | 11/07/2025, 12:10:41 |
  | Modified | 11/07/2025, 12:10:41 |

  ### Tags:

  - heart rate
  - nonhuman
  - review
  - wellbeing
  - dairy cattle
  - milk yield
  - tactile stimulation
  - auditory cortex
  - auditory stimulation
  - Holstein cattle
  - Medline
  - ruminant
  - Scopus
  - visual stimulation
  - Web of Science

  ### Attachments

  - Full Text (HTML)
- ## Can dogs reduce stress levels in school children? effects of dog-assisted interventions on salivary cortisol in children with and without special educational needs using randomized controlled trials

  |  |  |
  | --- | --- |
  | Item Type | Journal Article |
  | Author | K. Meints |
  | Author | V.L. Brelsford |
  | Author | M. Dimolareva |
  | Author | L. Maréchal |
  | Author | K. Pennington |
  | Author | E. Rowan |
  | Author | N.R. Gee |
  | Abstract | Prolonged or excessive stress negatively affects learning, behavior and health across the lifespan. To alleviate adverse effects of stress in school children, stressors should be reduced, and support and effective interventions provided. Animal-assisted interventions (AAI) have shown beneficial effects on health and wellbeing, however, robust knowledge on stress mediation in children is lacking. Despite this, AAIs are increasingly employed in settings world-wide, including schools, to reduce stress and support learning and wellbeing. This study is the first randomized controlled trial to investigate dog-assisted interventions as a mediator of stress in school children with and without special educational needs (SEN) over the school term. Interventions were carried out individually and in small groups twice a week for 20 minutes over the course of 4 weeks. We compared physiological changes in salivary cortisol in a dog intervention group with a relaxation intervention group and a no treatment control group. We compared cortisol level means before and after the 4 weeks of interventions in all children as well as acute cortisol in mainstream school children. Dog interventions lead to significantly lower stress in children with and without special educational needs compared to their peers in relaxation or no treatment control groups. In neurotypical children, those in the dog interventions showed no baseline stress level increases over the school term. In addition, acute cortisol levels evidenced significant stress reduction following the interventions. In contrast, the no treatment control group showed significant rises in baseline cortisol levels from beginning to end of school term. Increases also occurred in the relaxation intervention group. Children with SEN showed significantly decreased cortisol levels after dog group interventions. No changes occurred in the relaxation or no treatment control groups. These findings provide crucial evidence that dog interventions can successfully attenuate stress levels in school children with important implications for AAI implementation, learning and wellbeing. |
  | Date | 2022 |
  | Language | English |
  | Archive | Embase |
  | URL | https://www.embase.com/search/results?subaction=viewrecord&id=L2018840683&from=export |
  | Volume | 17 |
  | Publication | PLoS ONE |
  | DOI | 10.1371/journal.pone.0269333 |
  | Issue | 6 June |
  | Journal Abbr | PLoS ONE |
  | ISSN | 1932-6203 |
  | Date Added | 11/07/2025, 14:16:48 |
  | Modified | 11/07/2025, 14:16:48 |

  ### Tags:

  - animal welfare
  - dog
  - anxiety
  - child
  - article
  - female
  - male
  - nonhuman
  - human
  - hydrocortisone
  - controlled study
  - randomized controlled trial
  - questionnaire
  - wellbeing
  - autism
  - outcome assessment
  - learning
  - veterinary medicine
  - enzyme linked immunosorbent assay
  - training
  - attention deficit hyperactivity disorder
  - feces analysis
  - developmental delay
  - hypersalivation
  - yoga

  ### Attachments

  - Full Text (HTML)
- ## Can dogs reduce stress levels in school children? effects of dog-assisted interventions on salivary cortisol in children with and without special educational needs using randomized controlled trials

  |  |  |
  | --- | --- |
  | Item Type | Journal Article |
  | Author | K. Meints |
  | Author | V.L. Brelsford |
  | Author | M. Dimolareva |
  | Author | L. Maréchal |
  | Author | K. Pennington |
  | Author | E. Rowan |
  | Author | N.R. Gee |
  | Abstract | Prolonged or excessive stress negatively affects learning, behavior and health across the lifespan. To alleviate adverse effects of stress in school children, stressors should be reduced, and support and effective interventions provided. Animal-assisted interventions (AAI) have shown beneficial effects on health and wellbeing, however, robust knowledge on stress mediation in children is lacking. Despite this, AAIs are increasingly employed in settings world-wide, including schools, to reduce stress and support learning and wellbeing. This study is the first randomized controlled trial to investigate dog-assisted interventions as a mediator of stress in school children with and without special educational needs (SEN) over the school term. Interventions were carried out individually and in small groups twice a week for 20 minutes over the course of 4 weeks. We compared physiological changes in salivary cortisol in a dog intervention group with a relaxation intervention group and a no treatment control group. We compared cortisol level means before and after the 4 weeks of interventions in all children as well as acute cortisol in mainstream school children. Dog interventions lead to significantly lower stress in children with and without special educational needs compared to their peers in relaxation or no treatment control groups. In neurotypical children, those in the dog interventions showed no baseline stress level increases over the school term. In addition, acute cortisol levels evidenced significant stress reduction following the interventions. In contrast, the no treatment control group showed significant rises in baseline cortisol levels from beginning to end of school term. Increases also occurred in the relaxation intervention group. Children with SEN showed significantly decreased cortisol levels after dog group interventions. No changes occurred in the relaxation or no treatment control groups. These findings provide crucial evidence that dog interventions can successfully attenuate stress levels in school children with important implications for AAI implementation, learning and wellbeing. |
  | Date | 2022 |
  | Language | English |
  | Archive | Embase |
  | URL | https://www.embase.com/search/results?subaction=viewrecord&id=L2018840683&from=export |
  | Volume | 17 |
  | Publication | PLoS ONE |
  | DOI | 10.1371/journal.pone.0269333 |
  | Issue | 6 June |
  | Journal Abbr | PLoS ONE |
  | ISSN | 1932-6203 |
  | Date Added | 11/07/2025, 14:18:22 |
  | Modified | 11/07/2025, 14:18:22 |

  ### Tags:

  - animal welfare
  - dog
  - anxiety
  - child
  - article
  - female
  - male
  - nonhuman
  - human
  - hydrocortisone
  - controlled study
  - randomized controlled trial
  - questionnaire
  - wellbeing
  - autism
  - outcome assessment
  - learning
  - veterinary medicine
  - enzyme linked immunosorbent assay
  - training
  - attention deficit hyperactivity disorder
  - feces analysis
  - developmental delay
  - hypersalivation
  - yoga

  ### Attachments

  - Full Text (HTML)
- ## Pet ownership and physical health

  |  |  |
  | --- | --- |
  | Item Type | Journal Article |
  | Author | R.L. Matchock |
  | Abstract | Purpose of review. Pet ownership and brief human-animal interactions can serve as a form of social support and convey a host of beneficial psychological and physiological health benefits. This article critically examines recent relevant literature on the pet-health connection. Recent findings. Cross-sectional studies indicate correlations between pet ownership and numerous aspects of positive health outcomes, including improvements on cardiovascular measures and decreases in loneliness. Quasiexperimental studies and better controlled experimental studies corroborate these associations and suggest that owning and/or interacting with a pet may be causally related to some positive health outcomes. Summary. The value of pet ownership and animal-assisted therapy (AAT), as a nonpharmacological treatment modality, augmentation to traditional treatment, and healthy preventive behavior (in the case of pet ownership), is starting to be realized. However, more investigations that employ randomized controlled trials with larger sample sizes and investigations that more closely examine the underlying mechanism of the pet-health effect, such as oxytocin, are needed. |
  | Date | 2015 |
  | Language | English |
  | Archive | Embase |
  | URL | https://www.embase.com/search/results?subaction=viewrecord&id=L606208611&from=export |
  | Volume | 28 |
  | Pages | 386-392 |
  | Publication | Current Opinion in Psychiatry |
  | DOI | 10.1097/YCO.0000000000000183 |
  | Issue | 5 |
  | Journal Abbr | Curr. Opin. Psychiatry |
  | ISSN | 1473-6578 |
  | Date Added | 11/07/2025, 12:13:35 |
  | Modified | 11/07/2025, 12:13:35 |

  ### Tags:

  - oxytocin
  - pet ownership
  - social support
  - physical activity
  - human
  - posttraumatic stress disorder
  - pet animal
  - quality of life
  - review
  - autism
  - animal assisted therapy
  - health
  - task performance
  - attention deficit hyperactivity disorder
  - experimental study
  - sample size
  - psychotherapy
  - mental health
  - loneliness
  - child sexual abuse
  - cross-sectional study
  - dementia
  - fibromyalgia
  - quasi experimental study

  ### Attachments

  - Full Text (HTML)
- ## INTERACTION OF EMOTIONAL CONNECTION BETWEEN HORSE AND HUMAN

  |  |  |
  | --- | --- |
  | Item Type | Journal Article |
  | Author | O. Mataitė |
  | Author | V. Vilienė |
  | Abstract | As people become more emotionally involved with horses, they develop sophisticated communication skills, enabling horses to respond to human emotions [1]. An animal's reaction to stress can be influenced by the rider's or handler's emotional state, not just physical exertion, or environmental stimuli [2]. Cooperation and a strong emotional connection between the rider and the horse are crucial for good sporting results, reducing the risk of injuries, accidents, and stress for both [3]. To optimise the horse-human relationship, it is necessary to promote positive experiences through verbal or tactile communication, which strengthens the mutual emotional connection, decreases stress levels, and improves riding results. The purpose of this study was to analyse the interaction between the riding horses and the people riding them. To determine the mutual emotional connection between the horse and the human, the body language of the horse, the rider's touch to the horse and his voice were evaluated. Riding tests were conducted to determine the emotional connection between a horse and a person. The riding test consisted of two exercises: riding commands of 4 and a complete performance of 8-tuck. Both exercises were performed 4 times with an interval of 30 s. To determine the emotional level of the horse and the human, the heart rates of the horses and subjects were analysed per minute. A 5-question questionnaire for the subjects and a 3-question questionnaire for the parents of the subjects were used to determine the subjects’ emotional level before and after riding. The results of the study showed that the strength of the emotional connection between the horses in the stable and the people who ride them depends on the body language of the horses, the rider's touch to the horse and the result of his voice assessment scores. The execution time of the command also affected the efficiency of further executed commands. However, a study of heart rates between horses and humans showed that the heart rates of horses in a stable and the people who ride them are independent of each other. From the results of the survey of the subjects and the parents of the subjects, we can see that all children experience joy and happiness while riding and become more self-confident. Surveys also revealed to us that most subjects had fallen off their horses. Most developed a fear of falling again, while only a minority of subjects did not develop any psychological fear. |
  | Date | 2024 |
  | Language | English |
  | Archive | Embase |
  | URL | https://www.embase.com/search/results?subaction=viewrecord&id=L2036571723&from=export |
  | Volume | 82(1) |
  | Pages | 155-155 |
  | Publication | Veterinarija ir Zootechnika |
  | Issue | (Vilienė V.) Institute of Animal Rearing Technologies, Veterinary Academy, Lithuanian University of Health Sciences, Lithuania |
  | Journal Abbr | Vet. Zootech. |
  | ISSN | 1392-2130 |
  | Date Added | 11/07/2025, 11:53:21 |
  | Modified | 11/07/2025, 11:53:21 |

  ### Tags:

  - human-animal interaction
  - horse
  - heart rate
  - emotion
  - article
  - nonhuman
  - human
  - horseback riding
  - questionnaire
  - fear
  - accidental injury
  - human relation
  - knowledge
  - training
  - voice
- ## INTERACTION OF EMOTIONAL CONNECTION BETWEEN HORSE AND HUMAN

  |  |  |
  | --- | --- |
  | Item Type | Journal Article |
  | Author | O. Mataitė |
  | Author | V. Vilienė |
  | Abstract | As people become more emotionally involved with horses, they develop sophisticated communication skills, enabling horses to respond to human emotions [1]. An animal's reaction to stress can be influenced by the rider's or handler's emotional state, not just physical exertion, or environmental stimuli [2]. Cooperation and a strong emotional connection between the rider and the horse are crucial for good sporting results, reducing the risk of injuries, accidents, and stress for both [3]. To optimise the horse-human relationship, it is necessary to promote positive experiences through verbal or tactile communication, which strengthens the mutual emotional connection, decreases stress levels, and improves riding results. The purpose of this study was to analyse the interaction between the riding horses and the people riding them. To determine the mutual emotional connection between the horse and the human, the body language of the horse, the rider's touch to the horse and his voice were evaluated. Riding tests were conducted to determine the emotional connection between a horse and a person. The riding test consisted of two exercises: riding commands of 4 and a complete performance of 8-tuck. Both exercises were performed 4 times with an interval of 30 s. To determine the emotional level of the horse and the human, the heart rates of the horses and subjects were analysed per minute. A 5-question questionnaire for the subjects and a 3-question questionnaire for the parents of the subjects were used to determine the subjects’ emotional level before and after riding. The results of the study showed that the strength of the emotional connection between the horses in the stable and the people who ride them depends on the body language of the horses, the rider's touch to the horse and the result of his voice assessment scores. The execution time of the command also affected the efficiency of further executed commands. However, a study of heart rates between horses and humans showed that the heart rates of horses in a stable and the people who ride them are independent of each other. From the results of the survey of the subjects and the parents of the subjects, we can see that all children experience joy and happiness while riding and become more self-confident. Surveys also revealed to us that most subjects had fallen off their horses. Most developed a fear of falling again, while only a minority of subjects did not develop any psychological fear. |
  | Date | 2024 |
  | Language | English |
  | Archive | Embase |
  | URL | https://www.embase.com/search/results?subaction=viewrecord&id=L2036571723&from=export |
  | Volume | 82(1) |
  | Pages | 155-155 |
  | Publication | Veterinarija ir Zootechnika |
  | Issue | (Vilienė V.) Institute of Animal Rearing Technologies, Veterinary Academy, Lithuanian University of Health Sciences, Lithuania |
  | Journal Abbr | Vet. Zootech. |
  | ISSN | 1392-2130 |
  | Date Added | 11/07/2025, 12:10:44 |
  | Modified | 11/07/2025, 12:10:44 |

  ### Tags:

  - human-animal interaction
  - horse
  - heart rate
  - emotion
  - article
  - nonhuman
  - human
  - horseback riding
  - questionnaire
  - fear
  - accidental injury
  - human relation
  - knowledge
  - training
  - voice
- ## Bringing human-animal interaction to sport: Potential impacts on athletic performance

  |  |  |
  | --- | --- |
  | Item Type | Journal Article |
  | Author | S. Marvin |
  | Author | K. Sorenson |
  | Author | J.R. Stevens |
  | Abstract | To gain an edge in performance, athletes, coaches, trainers, and sport psychologists worldwide leverage findings from psychological research to develop training and performance strategies. The field of sport psychology draws upon research on stress, anxiety, mindfulness, and team building to develop these strategies. Here, we introduce human-animal interaction as a potential area of research that may apply to athletic performance. Structured interactions with animals-particularly therapy dogs-can provide physiological benefits associated with stress and the oxytocin system, psychological benefits for anxiety and motivation, and social benefits through social support. Yet these effects have not yet been systematically investigated in athletes. Integration of human-animal interactions into athletics can occur through animal visitation programmes and resident therapy animal programmes. Integrating human-animal interactions into athletics presents some unique challenges and limitations that must be considered before implementing these programmes, and these interactions are not a panacea that will work in every situation. But, given the amount of human-animal interaction research suggesting benefits in medicine, mental health, and education contexts, it is worthwhile exploring potential benefits not just for athletic performance, but also for injury prevention and recovery. HighlightsHuman-animal interaction is a potential area of research that may apply to athletic performance.Structured interactions with animals can provide physiological, psychological, and social benefits to athletes, through it is not a panacea that will work in every situation.Integrating human-animal interactions into athletics presents some unique challenges and limitations that must be considered before implementing these programs. |
  | Date | 2022 |
  | Language | English |
  | Archive | Medline |
  | URL | https://www.embase.com/search/results?subaction=viewrecord&id=L634791146&from=export |
  | Volume | 22 |
  | Pages | 955-963 |
  | Publication | European journal of sport science |
  | DOI | 10.1080/17461391.2021.1916084 |
  | Issue | 7 |
  | Journal Abbr | Eur J Sport Sci |
  | ISSN | 1536-7290 |
  | Date Added | 11/07/2025, 12:12:21 |
  | Modified | 11/07/2025, 12:12:21 |

  ### Tags:

  - dog
  - human
  - animal
  - psychology
  - mindfulness
  - athlete
  - athletic performance
  - sport injury
  - sports medicine

  ### Attachments

  - Full Text (HTML)
- ## Bringing human-animal interaction to sport: Potential impacts on athletic performance

  |  |  |
  | --- | --- |
  | Item Type | Journal Article |
  | Author | S. Marvin |
  | Author | K. Sorenson |
  | Author | J.R. Stevens |
  | Abstract | To gain an edge in performance, athletes, coaches, trainers, and sport psychologists worldwide leverage findings from psychological research to develop training and performance strategies. The field of sport psychology draws upon research on stress, anxiety, mindfulness, and team building to develop these strategies. Here, we introduce human-animal interaction as a potential area of research that may apply to athletic performance. Structured interactions with animals-particularly therapy dogs-can provide physiological benefits associated with stress and the oxytocin system, psychological benefits for anxiety and motivation, and social benefits through social support. Yet these effects have not yet been systematically investigated in athletes. Integration of human-animal interactions into athletics can occur through animal visitation programmes and resident therapy animal programmes. Integrating human-animal interactions into athletics presents some unique challenges and limitations that must be considered before implementing these programmes, and these interactions are not a panacea that will work in every situation. But, given the amount of human-animal interaction research suggesting benefits in medicine, mental health, and education contexts, it is worthwhile exploring potential benefits not just for athletic performance, but also for injury prevention and recovery. HighlightsHuman-animal interaction is a potential area of research that may apply to athletic performance.Structured interactions with animals can provide physiological, psychological, and social benefits to athletes, through it is not a panacea that will work in every situation.Integrating human-animal interactions into athletics presents some unique challenges and limitations that must be considered before implementing these programs. |
  | Date | 2022 |
  | Language | English |
  | Archive | Medline |
  | URL | https://www.embase.com/search/results?subaction=viewrecord&id=L634791146&from=export |
  | Volume | 22 |
  | Pages | 955-963 |
  | Publication | European journal of sport science |
  | DOI | 10.1080/17461391.2021.1916084 |
  | Issue | 7 |
  | Journal Abbr | Eur J Sport Sci |
  | ISSN | 1536-7290 |
  | Date Added | 11/07/2025, 12:13:23 |
  | Modified | 11/07/2025, 12:13:23 |

  ### Tags:

  - dog
  - human
  - animal
  - psychology
  - mindfulness
  - athlete
  - athletic performance
  - sport injury
  - sports medicine

  ### Attachments

  - Full Text (HTML)
- ## Effects of contact with a dog on prefrontal brain activation in patients in a minimally conscious state: A controlled crossover trial

  |  |  |
  | --- | --- |
  | Item Type | Journal Article |
  | Author | R. Marti |
  | Author | M. Petignat |
  | Author | V.L. Marcar |
  | Author | J. Hattendorf |
  | Author | M. Wolf |
  | Author | M. Hund-Georgiadis |
  | Author | K. Hediger |
  | Abstract | The first studies have indicated that animal-assisted therapy benefits patients in a minimally conscious state (MCS), but the evidence is scarce. It is thus crucial to understand how these patients react to animal contact. This study aimed to measure the prefrontal brain activation in MCS patients during contact with a dog compared with a plush animal using functional near-infrared spectroscopy (fNIRS). We conducted a controlled crossover trial with 22 MCS patients, who each participated in six sessions. Patients interacted with a dog in three sessions and with a plush animal in three control sessions. Each session consisted of five 2-minute phases with a neutral phase at the start and the end. The contact intensity with the dog or the plush animal increased from the second to the fourth phase. The fNIRS parameters did not differ between the conditions. The mean heart rate was significantly higher in the dog condition than in the control. In both conditions, prefrontal brain activation, mean heart rate, and one heart rate variability parameter increased with the increased intensity of contact with the dog and plush animal. The results show that MCS patients react with the same prefrontal brain activation during contact with a dog and a plush animal but have increased heart rate in contact with the dog, indicating physiological arousal. These findings suggest that the incorporation of animals into MCS therapy has the potential to stimulate patients, thereby facilitating greater participation. However, more research is needed to understand the effects of animals on brain activation. |
  | Date | 2025 |
  | Language | English |
  | Archive | Embase |
  | URL | https://www.embase.com/search/results?subaction=viewrecord&id=L2038827911&from=export |
  | Volume | 577 |
  | Pages | 175-189 |
  | Publication | Neuroscience |
  | DOI | 10.1016/j.neuroscience.2025.05.014 |
  | Issue | (Marti R.; Petignat M.; Hediger K., karin.hediger@unilu.ch) Faculty of Psychology, University of Basel, Basel, Switzerland |
  | Journal Abbr | Neuroscience |
  | ISSN | 1873-7544 |
  | Date Added | 11/07/2025, 11:49:32 |
  | Modified | 11/07/2025, 11:49:32 |

  ### Tags:

  - human-animal interaction
  - physiology
  - heart rate
  - dog
  - animal experiment
  - article
  - female
  - male
  - nonhuman
  - controlled study
  - data analysis software
  - assessment of humans
  - basler vegetative state assessment
  - body temperature
  - brain function
  - brain injury
  - clinical trial (topic)
  - coma remission scale revised
  - crossover procedure
  - deoxygenated hemoglobin
  - electroencephalogram
  - Erprec-EEG/ERP recording Software version 2.0.7.1
  - functional near infrared spectrometer
  - functional near-infrared spectroscopy
  - Glasgow coma scale
  - golden retriever
  - hemoglobin
  - hemoglobin determination
  - imaging software
  - Kubios HRV Standard version 3.1.0–3.5.0
  - minimally conscious state
  - Model 8004CA Sensors Adhesive
  - NCT03341325
  - neurorehabilitation
  - oxygen saturation
  - oxygenated hemoglobin
  - prefrontal brain activation
  - R 4.1.0
  - R package lme4
  - sensitivity analysis
  - SenSmart Model X- 100
  - SenSmart software version 1.0.1.0
  - sensor
  - spectrometer
  - unclassified drug

  ### Attachments

  - Full Text (HTML)
- ## Effects of contact with a dog on prefrontal brain activation in patients in a minimally conscious state: A controlled crossover trial

  |  |  |
  | --- | --- |
  | Item Type | Journal Article |
  | Author | R. Marti |
  | Author | M. Petignat |
  | Author | V.L. Marcar |
  | Author | J. Hattendorf |
  | Author | M. Wolf |
  | Author | M. Hund-Georgiadis |
  | Author | K. Hediger |
  | Abstract | The first studies have indicated that animal-assisted therapy benefits patients in a minimally conscious state (MCS), but the evidence is scarce. It is thus crucial to understand how these patients react to animal contact. This study aimed to measure the prefrontal brain activation in MCS patients during contact with a dog compared with a plush animal using functional near-infrared spectroscopy (fNIRS). We conducted a controlled crossover trial with 22 MCS patients, who each participated in six sessions. Patients interacted with a dog in three sessions and with a plush animal in three control sessions. Each session consisted of five 2-minute phases with a neutral phase at the start and the end. The contact intensity with the dog or the plush animal increased from the second to the fourth phase. The fNIRS parameters did not differ between the conditions. The mean heart rate was significantly higher in the dog condition than in the control. In both conditions, prefrontal brain activation, mean heart rate, and one heart rate variability parameter increased with the increased intensity of contact with the dog and plush animal. The results show that MCS patients react with the same prefrontal brain activation during contact with a dog and a plush animal but have increased heart rate in contact with the dog, indicating physiological arousal. These findings suggest that the incorporation of animals into MCS therapy has the potential to stimulate patients, thereby facilitating greater participation. However, more research is needed to understand the effects of animals on brain activation. |
  | Date | 2025 |
  | Language | English |
  | Archive | Embase |
  | URL | https://www.embase.com/search/results?subaction=viewrecord&id=L2038827911&from=export |
  | Volume | 577 |
  | Pages | 175-189 |
  | Publication | Neuroscience |
  | DOI | 10.1016/j.neuroscience.2025.05.014 |
  | Issue | (Marti R.; Petignat M.; Hediger K., karin.hediger@unilu.ch) Faculty of Psychology, University of Basel, Basel, Switzerland |
  | Journal Abbr | Neuroscience |
  | ISSN | 1873-7544 |
  | Date Added | 11/07/2025, 11:50:52 |
  | Modified | 11/07/2025, 11:50:52 |

  ### Tags:

  - human-animal interaction
  - physiology
  - heart rate
  - dog
  - animal experiment
  - article
  - female
  - male
  - nonhuman
  - controlled study
  - data analysis software
  - assessment of humans
  - basler vegetative state assessment
  - body temperature
  - brain function
  - brain injury
  - clinical trial (topic)
  - coma remission scale revised
  - crossover procedure
  - deoxygenated hemoglobin
  - electroencephalogram
  - Erprec-EEG/ERP recording Software version 2.0.7.1
  - functional near infrared spectrometer
  - functional near-infrared spectroscopy
  - Glasgow coma scale
  - golden retriever
  - hemoglobin
  - hemoglobin determination
  - imaging software
  - Kubios HRV Standard version 3.1.0–3.5.0
  - minimally conscious state
  - Model 8004CA Sensors Adhesive
  - NCT03341325
  - neurorehabilitation
  - oxygen saturation
  - oxygenated hemoglobin
  - prefrontal brain activation
  - R 4.1.0
  - R package lme4
  - sensitivity analysis
  - SenSmart Model X- 100
  - SenSmart software version 1.0.1.0
  - sensor
  - spectrometer
  - unclassified drug

  ### Attachments

  - Full Text (HTML)
- ## Effects of contact with a dog on prefrontal brain activation in patients in a minimally conscious state: A controlled crossover trial

  |  |  |
  | --- | --- |
  | Item Type | Journal Article |
  | Author | R. Marti |
  | Author | M. Petignat |
  | Author | V.L. Marcar |
  | Author | J. Hattendorf |
  | Author | M. Wolf |
  | Author | M. Hund-Georgiadis |
  | Author | K. Hediger |
  | Abstract | The first studies have indicated that animal-assisted therapy benefits patients in a minimally conscious state (MCS), but the evidence is scarce. It is thus crucial to understand how these patients react to animal contact. This study aimed to measure the prefrontal brain activation in MCS patients during contact with a dog compared with a plush animal using functional near-infrared spectroscopy (fNIRS). We conducted a controlled crossover trial with 22 MCS patients, who each participated in six sessions. Patients interacted with a dog in three sessions and with a plush animal in three control sessions. Each session consisted of five 2-minute phases with a neutral phase at the start and the end. The contact intensity with the dog or the plush animal increased from the second to the fourth phase. The fNIRS parameters did not differ between the conditions. The mean heart rate was significantly higher in the dog condition than in the control. In both conditions, prefrontal brain activation, mean heart rate, and one heart rate variability parameter increased with the increased intensity of contact with the dog and plush animal. The results show that MCS patients react with the same prefrontal brain activation during contact with a dog and a plush animal but have increased heart rate in contact with the dog, indicating physiological arousal. These findings suggest that the incorporation of animals into MCS therapy has the potential to stimulate patients, thereby facilitating greater participation. However, more research is needed to understand the effects of animals on brain activation. |
  | Date | 2025 |
  | Language | English |
  | Archive | Embase |
  | URL | https://www.embase.com/search/results?subaction=viewrecord&id=L2038827911&from=export |
  | Volume | 577 |
  | Pages | 175-189 |
  | Publication | Neuroscience |
  | DOI | 10.1016/j.neuroscience.2025.05.014 |
  | Issue | (Marti R.; Petignat M.; Hediger K., karin.hediger@unilu.ch) Faculty of Psychology, University of Basel, Basel, Switzerland |
  | Journal Abbr | Neuroscience |
  | ISSN | 1873-7544 |
  | Date Added | 11/07/2025, 11:53:14 |
  | Modified | 11/07/2025, 11:53:14 |

  ### Tags:

  - human-animal interaction
  - physiology
  - heart rate
  - dog
  - animal experiment
  - article
  - female
  - male
  - nonhuman
  - controlled study
  - data analysis software
  - assessment of humans
  - basler vegetative state assessment
  - body temperature
  - brain function
  - brain injury
  - clinical trial (topic)
  - coma remission scale revised
  - crossover procedure
  - deoxygenated hemoglobin
  - electroencephalogram
  - Erprec-EEG/ERP recording Software version 2.0.7.1
  - functional near infrared spectrometer
  - functional near-infrared spectroscopy
  - Glasgow coma scale
  - golden retriever
  - hemoglobin
  - hemoglobin determination
  - imaging software
  - Kubios HRV Standard version 3.1.0–3.5.0
  - minimally conscious state
  - Model 8004CA Sensors Adhesive
  - NCT03341325
  - neurorehabilitation
  - oxygen saturation
  - oxygenated hemoglobin
  - prefrontal brain activation
  - R 4.1.0
  - R package lme4
  - sensitivity analysis
  - SenSmart Model X- 100
  - SenSmart software version 1.0.1.0
  - sensor
  - spectrometer
  - unclassified drug

  ### Attachments

  - Full Text (HTML)
- ## Effects of contact with a dog on prefrontal brain activation in patients in a minimally conscious state: A controlled crossover trial

  |  |  |
  | --- | --- |
  | Item Type | Journal Article |
  | Author | R. Marti |
  | Author | M. Petignat |
  | Author | V.L. Marcar |
  | Author | J. Hattendorf |
  | Author | M. Wolf |
  | Author | M. Hund-Georgiadis |
  | Author | K. Hediger |
  | Abstract | The first studies have indicated that animal-assisted therapy benefits patients in a minimally conscious state (MCS), but the evidence is scarce. It is thus crucial to understand how these patients react to animal contact. This study aimed to measure the prefrontal brain activation in MCS patients during contact with a dog compared with a plush animal using functional near-infrared spectroscopy (fNIRS). We conducted a controlled crossover trial with 22 MCS patients, who each participated in six sessions. Patients interacted with a dog in three sessions and with a plush animal in three control sessions. Each session consisted of five 2-minute phases with a neutral phase at the start and the end. The contact intensity with the dog or the plush animal increased from the second to the fourth phase. The fNIRS parameters did not differ between the conditions. The mean heart rate was significantly higher in the dog condition than in the control. In both conditions, prefrontal brain activation, mean heart rate, and one heart rate variability parameter increased with the increased intensity of contact with the dog and plush animal. The results show that MCS patients react with the same prefrontal brain activation during contact with a dog and a plush animal but have increased heart rate in contact with the dog, indicating physiological arousal. These findings suggest that the incorporation of animals into MCS therapy has the potential to stimulate patients, thereby facilitating greater participation. However, more research is needed to understand the effects of animals on brain activation. |
  | Date | 2025 |
  | Language | English |
  | Archive | Embase |
  | URL | https://www.embase.com/search/results?subaction=viewrecord&id=L2038827911&from=export |
  | Volume | 577 |
  | Pages | 175-189 |
  | Publication | Neuroscience |
  | DOI | 10.1016/j.neuroscience.2025.05.014 |
  | Issue | (Marti R.; Petignat M.; Hediger K., karin.hediger@unilu.ch) Faculty of Psychology, University of Basel, Basel, Switzerland |
  | Journal Abbr | Neuroscience |
  | ISSN | 1873-7544 |
  | Date Added | 11/07/2025, 12:10:35 |
  | Modified | 11/07/2025, 12:10:35 |

  ### Tags:

  - human-animal interaction
  - physiology
  - heart rate
  - dog
  - animal experiment
  - article
  - female
  - male
  - nonhuman
  - controlled study
  - data analysis software
  - assessment of humans
  - basler vegetative state assessment
  - body temperature
  - brain function
  - brain injury
  - clinical trial (topic)
  - coma remission scale revised
  - crossover procedure
  - deoxygenated hemoglobin
  - electroencephalogram
  - Erprec-EEG/ERP recording Software version 2.0.7.1
  - functional near infrared spectrometer
  - functional near-infrared spectroscopy
  - Glasgow coma scale
  - golden retriever
  - hemoglobin
  - hemoglobin determination
  - imaging software
  - Kubios HRV Standard version 3.1.0–3.5.0
  - minimally conscious state
  - Model 8004CA Sensors Adhesive
  - NCT03341325
  - neurorehabilitation
  - oxygen saturation
  - oxygenated hemoglobin
  - prefrontal brain activation
  - R 4.1.0
  - R package lme4
  - sensitivity analysis
  - SenSmart Model X- 100
  - SenSmart software version 1.0.1.0
  - sensor
  - spectrometer
  - unclassified drug

  ### Attachments

  - Full Text (HTML)
- ## Comparative analysis of stress responses in dogs and cats during the covid-19 pandemic: a focus on cortisol, total leukocytes, eosinophils, and behavioral changes

  |  |  |
  | --- | --- |
  | Item Type | Journal Article |
  | Author | R.N. Malancus |
  | Author | V.N. Arsenoaia |
  | Author | M. Ghita |
  | Abstract | The COVID-19 pandemic has ushered in unprecedented changes in the lives of humans and their companion animals, impacting their daily routines, social interactions, and stress levels. This study investigates the effect of pandemic-related restrictions, including limited time outside and changes in owner habits, on the stress indicators of 34 dogs and 29 cats. Cortisol levels, total leukocyte counts, eosinophil counts, and behavioral changes have been analyzed to assess how these species are differentially affected by the altered environment and human interactions during the pandemic. The study revealed important behavioral changes in cats while the biochemical and hematological parameters were considerably affected in dogs. Our findings shed light on the unique stress experiences of dogs and cats and underscore the importance of considering individualized strategies for managing their well-being during extraordinary circumstances. |
  | Date | 2024 |
  | Language | English |
  | Archive | Embase |
  | URL | https://www.embase.com/search/results?subaction=viewrecord&id=L2033122647&from=export |
  | Volume | 76 |
  | Publication | Arquivo Brasileiro de Medicina Veterinaria e Zootecnia |
  | DOI | 10.1590/1678-4162-13153 |
  | Issue | 3 |
  | Journal Abbr | Arq. Bras. Med. Vet. Zootec. |
  | ISSN | 1678-4162 |
  | Date Added | 11/07/2025, 14:15:31 |
  | Modified | 11/07/2025, 14:15:31 |

  ### Tags:

  - human-animal interaction
  - dog
  - animal behavior
  - article
  - coronavirus disease 2019
  - lockdown
  - nonhuman
  - pandemic
  - physiological stress
  - social interaction
  - hydrocortisone
  - grooming
  - pet animal
  - behavior change
  - emotional well-being
  - leukocyte count
  - cat
  - comparative study
  - species difference
  - environmental factor
  - daily life activity
  - eosinophil count
  - hematological parameters
  - individualization
  - restlessness
  - social distancing
  - territoriality
  - travel restriction
  - vocalization

  ### Attachments

  - Full Text (HTML)
- ## Comparative analysis of stress responses in dogs and cats during the covid-19 pandemic: a focus on cortisol, total leukocytes, eosinophils, and behavioral changes

  |  |  |
  | --- | --- |
  | Item Type | Journal Article |
  | Author | R.N. Malancus |
  | Author | V.N. Arsenoaia |
  | Author | M. Ghita |
  | Abstract | The COVID-19 pandemic has ushered in unprecedented changes in the lives of humans and their companion animals, impacting their daily routines, social interactions, and stress levels. This study investigates the effect of pandemic-related restrictions, including limited time outside and changes in owner habits, on the stress indicators of 34 dogs and 29 cats. Cortisol levels, total leukocyte counts, eosinophil counts, and behavioral changes have been analyzed to assess how these species are differentially affected by the altered environment and human interactions during the pandemic. The study revealed important behavioral changes in cats while the biochemical and hematological parameters were considerably affected in dogs. Our findings shed light on the unique stress experiences of dogs and cats and underscore the importance of considering individualized strategies for managing their well-being during extraordinary circumstances. |
  | Date | 2024 |
  | Language | English |
  | Archive | Embase |
  | URL | https://www.embase.com/search/results?subaction=viewrecord&id=L2033122647&from=export |
  | Volume | 76 |
  | Publication | Arquivo Brasileiro de Medicina Veterinaria e Zootecnia |
  | DOI | 10.1590/1678-4162-13153 |
  | Issue | 3 |
  | Journal Abbr | Arq. Bras. Med. Vet. Zootec. |
  | ISSN | 1678-4162 |
  | Date Added | 11/07/2025, 14:16:42 |
  | Modified | 11/07/2025, 14:16:42 |

  ### Tags:

  - human-animal interaction
  - dog
  - animal behavior
  - article
  - coronavirus disease 2019
  - lockdown
  - nonhuman
  - pandemic
  - physiological stress
  - social interaction
  - hydrocortisone
  - grooming
  - pet animal
  - behavior change
  - emotional well-being
  - leukocyte count
  - cat
  - comparative study
  - species difference
  - environmental factor
  - daily life activity
  - eosinophil count
  - hematological parameters
  - individualization
  - restlessness
  - social distancing
  - territoriality
  - travel restriction
  - vocalization

  ### Attachments

  - Full Text (HTML)
- ## Comparative analysis of stress responses in dogs and cats during the covid-19 pandemic: a focus on cortisol, total leukocytes, eosinophils, and behavioral changes

  |  |  |
  | --- | --- |
  | Item Type | Journal Article |
  | Author | R.N. Malancus |
  | Author | V.N. Arsenoaia |
  | Author | M. Ghita |
  | Abstract | The COVID-19 pandemic has ushered in unprecedented changes in the lives of humans and their companion animals, impacting their daily routines, social interactions, and stress levels. This study investigates the effect of pandemic-related restrictions, including limited time outside and changes in owner habits, on the stress indicators of 34 dogs and 29 cats. Cortisol levels, total leukocyte counts, eosinophil counts, and behavioral changes have been analyzed to assess how these species are differentially affected by the altered environment and human interactions during the pandemic. The study revealed important behavioral changes in cats while the biochemical and hematological parameters were considerably affected in dogs. Our findings shed light on the unique stress experiences of dogs and cats and underscore the importance of considering individualized strategies for managing their well-being during extraordinary circumstances. |
  | Date | 2024 |
  | Language | English |
  | Archive | Embase |
  | URL | https://www.embase.com/search/results?subaction=viewrecord&id=L2033122647&from=export |
  | Volume | 76 |
  | Publication | Arquivo Brasileiro de Medicina Veterinaria e Zootecnia |
  | DOI | 10.1590/1678-4162-13153 |
  | Issue | 3 |
  | Journal Abbr | Arq. Bras. Med. Vet. Zootec. |
  | ISSN | 1678-4162 |
  | Date Added | 11/07/2025, 14:18:14 |
  | Modified | 11/07/2025, 14:18:14 |

  ### Tags:

  - human-animal interaction
  - dog
  - animal behavior
  - article
  - coronavirus disease 2019
  - lockdown
  - nonhuman
  - pandemic
  - physiological stress
  - social interaction
  - hydrocortisone
  - grooming
  - pet animal
  - behavior change
  - emotional well-being
  - leukocyte count
  - cat
  - comparative study
  - species difference
  - environmental factor
  - daily life activity
  - eosinophil count
  - hematological parameters
  - individualization
  - restlessness
  - social distancing
  - territoriality
  - travel restriction
  - vocalization

  ### Attachments

  - Full Text (HTML)
- ## Validation of salivary oxytocin and vasopressin as biomarkers in domestic dogs

  |  |  |
  | --- | --- |
  | Item Type | Journal Article |
  | Author | E.L. MacLean |
  | Author | L.R. Gesquiere |
  | Author | N. Gee |
  | Author | K. Levy |
  | Author | W.L. Martin |
  | Author | C.S. Carter |
  | Abstract | Background Oxytocin (OT) and Vasopressin (AVP) are phylogenetically conserved neuropeptides with effects on social behavior, cognition and stress responses. Although OT and AVP are most commonly measured in blood, urine and cerebrospinal fluid (CSF), these approaches present an array of challenges including concerns related to the invasiveness of sample collection, the potential for matrix interference in immunoassays, and whether samples can be collected at precise time points to assess event-linked endocrine responses. New method We validated enzyme-linked immunosorbent assays (ELISAs) for the measurement of salivary OT and AVP in domestic dogs. Results Both OT and AVP were present in dog saliva and detectable by ELISA and high performance liquid chromatography – mass spectrometry (HPLC–MS). OT concentrations in dog saliva were much higher than those typically detected in humans. OT concentrations in the same samples analyzed with and without sample extraction were highly correlated, but this was not true for AVP. ELISA validation studies revealed good accuracy and parallelism, both with and without solid phase extraction. Collection of salivary samples with different synthetic swabs, or following salivary stimulation or the consumption of food led to variance in results. However, samples collected from the same dogs using different techniques tended to be positively correlated. We detected concurrent elevations in salivary and plasma OT during nursing. Comparison with existing methods There are currently no other validated methods for measuring OT/AVP in dog saliva. Conclusions OT and AVP are present in dog saliva, and ELISAs for their detection are methodologically valid. |
  | Date | 2018 |
  | Language | English |
  | Archive | Embase |
  | URL | https://www.embase.com/search/results?subaction=viewrecord&id=L618372841&from=export |
  | Volume | 293 |
  | Pages | 67-76 |
  | Publication | Journal of Neuroscience Methods |
  | DOI | 10.1016/j.jneumeth.2017.08.033 |
  | Issue | (MacLean E.L., evanmaclean@arizona.edu) School of Anthropology, University of Arizona, Tucson, AZ, United States |
  | Journal Abbr | J. Neurosci. Methods |
  | ISSN | 1872-678X |
  | Date Added | 11/07/2025, 12:13:32 |
  | Modified | 11/07/2025, 12:13:32 |

  ### Tags:

  - oxytocin
  - dog
  - saliva
  - vasopressin
  - article
  - female
  - male
  - nonhuman
  - controlled study
  - enzyme linked immunosorbent assay
  - priority journal
  - oxytocin blood level
  - limit of detection
  - validation study
  - food intake
  - high performance liquid chromatography
  - liquid chromatography-mass spectrometry
  - mass spectrometry
  - solid phase extraction

  ### Attachments

  - Full Text (HTML)
- ## Human-animal interaction, stress, and embryo production in Bos indicus embryo donors under tropical conditions

  |  |  |
  | --- | --- |
  | Item Type | Journal Article |
  | Author | G.G. Macedo |
  | Author | C.E.S.N. Zúccari |
  | Author | U.G.P. de Abreu |
  | Author | J.A. Negrão |
  | Author | E.V. da Costa e Silva |
  | Abstract | This study investigated the effect of human-animal interaction (HAI) and the stress response on the quality of embryo production in superovulated Nelore (Bos indicus) cattle, under tropical conditions. Thirty-two females underwent a superovulation protocol for 5 days. Cortisol concentrations were determined in blood plasma collected on days 0, 4, and 5. Artificial insemination was performed on days 4 and 5, and nonsurgical embryo flushing on day 11. Embryo production and viability were determined. Human stimulation, animal behaviors, accidents, and handling time were recorded to assess HAI. Cattle age was negatively correlated with accidents, frequency of aversive behaviors, and negative stimuli by stockperson during transit through corral compartments to receive superovulation treatments. The factor analysis revealed two distinct groups. The first group was called stressed and had higher cortisol concentration than the nonstressed group, 16.0 ± 2.1 and 12.5 ± 1.0 ng/mL, respectively. Comparisons between these groups showed that the frequency of voice emissions by the stockperson and the number of accidents were higher in the stressed group, and also, the mean handling time was longer in the stressed group than for the nonstressed. As a result, viability rate of the embryos was 19% lower in the stressed group (P < 0.05). This indicates that intensive negative HAI is likely related to stress, which affects embryo production in a superovulation program. © 2011 Springer Science+Business Media B.V. |
  | Date | 2011 |
  | Language | English |
  | Archive | Medline |
  | URL | https://www.embase.com/search/results?subaction=viewrecord&id=L51344667&from=export |
  | Volume | 43 |
  | Pages | 1175-1182 |
  | Publication | Tropical Animal Health and Production |
  | DOI | 10.1007/s11250-011-9820-6 |
  | Issue | 6 |
  | Journal Abbr | Trop. Anim. Health Prod. |
  | ISSN | 0049-4747 |
  | Date Added | 11/07/2025, 14:18:43 |
  | Modified | 11/07/2025, 14:18:43 |

  ### Tags:

  - animal welfare
  - animal behavior
  - article
  - female
  - physiological stress
  - hydrocortisone
  - bovine
  - animal
  - blood
  - animal disease
  - artificial insemination
  - Brazil
  - embryo transfer
  - human activities
  - superovulation
  - tropic climate

  ### Attachments

  - Full Text (HTML)
- ## Salivary oxytocin in pigs, cattle, and goats during positive human-animal interactions

  |  |  |
  | --- | --- |
  | Item Type | Journal Article |
  | Author | S. Lürzel |
  | Author | L. Bückendorf |
  | Author | S. Waiblinger |
  | Author | J.-L. Rault |
  | Abstract | Research on oxytocin (OT) has provided crucial insight into the neuroendocrine mechanisms of social behavior and human-animal interactions. Nonetheless, this peptide hormone remains challenging to sample, usually requiring invasive measures in the brain or blood, which limits its use. We conducted an analytical validation of the measurement of OT in the saliva of three common farm animal species: pigs, cattle and goats. We then investigated the biological significance of salivary OT concentrations by using a positive human-animal interaction paradigm. The paradigm varied in terms of human familiarity and included a control condition where the animal was alone in the testing pen. A total of 18 pigs, 18 cattle and 9 goats were subjected to each condition using a within-individual counterbalanced design. Animals were free to choose to interact with the human. Saliva samples were collected immediately before and after the 10-min test, and behavior during the test was analyzed. Oxytocin could be reliably measured by ELISA in the saliva of goats (without extraction), and in the saliva of pigs and cattle after extraction. Salivary OT concentration did not change consistently according to testing condition. However, salivary OT concentration was significantly associated with some behaviors, with different relationships depending on the species. In conclusion, OT can be reliably measured in the saliva of goats and, after extraction, in the saliva of pigs and cattle. The relationships of OT with human-animal interactions and animal behavior were complex and to some extent species-dependent, possibly reflecting the perception of the interaction by the animal. |
  | Date | 2020 |
  | Language | English |
  | Archive | Embase |
  | URL | https://www.embase.com/search/results?subaction=viewrecord&id=L2005154015&from=export |
  | Volume | 115 |
  | Publication | Psychoneuroendocrinology |
  | DOI | 10.1016/j.psyneuen.2020.104636 |
  | Issue | (Lürzel S.; Bückendorf L.; Waiblinger S.; Rault J.-L., jean-loup.rault@vetmeduni.ac.at) Institute of Animal Welfare Science, University of Veterinary Medicine, Vienna, Veterinärplatz 1, Vienna, Austria |
  | Journal Abbr | Psychoneuroendocrinology |
  | ISSN | 1873-3360 |
  | Date Added | 11/07/2025, 12:13:29 |
  | Modified | 11/07/2025, 12:13:29 |

  ### Tags:

  - oxytocin
  - human-animal interaction
  - saliva
  - animal behavior
  - article
  - female
  - nonhuman
  - human
  - controlled study
  - pig
  - bovine
  - human-animal bond
  - enzyme linked immunosorbent assay
  - priority journal
  - infant
  - species difference
  - saliva analysis
  - extraction
  - goat

  ### Attachments

  - Full Text (HTML)
- ## Effects of gentle interactions on the relationship with humans and on stress-related parameters in group-housed calves

  |  |  |
  | --- | --- |
  | Item Type | Journal Article |
  | Author | S. Lürzel |
  | Author | I. Windschnurer |
  | Author | A. Futschik |
  | Author | R. Palme |
  | Author | S. Waiblinger |
  | Abstract | Although the relationship between farm animals and humans has strong implications for animal welfare and productivity, there have been few experimental studies on the influence of gentle interactions in group-housed calves. In the present study, Austrian Simmental calves were housed in groups of four under standard management conditions. Fourteen calves experienced 40 min of additional gentle interactions in the form of stroking and gentle talking during the first four weeks of life, whereas the remaining eleven calves did not. The animals' fear of humans was measured by avoidance distance tests on 33 and 76 days of age and by an arena test that comprised three phases - isolation, presence of a human, isolation - at 34 days of age. The very low avoidance distances did not differ significantly between the groups. In the arena test, there was less behaviour indicative of stress in the presence of the experimenter compared with the isolation phases. Heart-rate measurements showed a corresponding pattern. Control calves showed more tail-flicking than stroked calves and had higher concentrations of salivary cortisol before and after the test. There were no other significant differences between the groups. The minor number of behavioural differences may result from the control animals' good relationship with humans, ie there is a ceiling effect. If the general contact between stockpeople and calves is gentle and negative experiences are minimised, it is possible to achieve a good calf-human relationship without additional efforts. |
  | Date | 2015 |
  | Language | English |
  | Archive | Embase |
  | URL | https://www.embase.com/search/results?subaction=viewrecord&id=L606867788&from=export |
  | Volume | 24 |
  | Pages | 475-484 |
  | Publication | Animal Welfare |
  | DOI | 10.7120/09627286.24.4.475 |
  | Issue | 4 |
  | Journal Abbr | Anim. Welf. |
  | ISSN | 0962-7286 |
  | Date Added | 11/07/2025, 14:18:41 |
  | Modified | 11/07/2025, 14:18:41 |

  ### Tags:

  - heart rate
  - animal behavior
  - animal experiment
  - article
  - female
  - locomotion
  - male
  - nonhuman
  - physiological stress
  - human
  - hydrocortisone
  - controlled study
  - human-animal bond
  - human relation
  - calf (bovine)
  - vocalization
  - avoidance behavior

  ### Attachments

  - Full Text (HTML)
- ## Why should human-animal interactions be included in research of working equids’ welfare?

  |  |  |
  | --- | --- |
  | Item Type | Journal Article |
  | Author | Daniela Luna |
  | Author | Tamara A. Tadich |
  | Date | 2019 |
  | Library Catalogue | Google Scholar |
  | URL | https://www.mdpi.com/2076-2615/9/2/42 |
  | Accessed | 11/07/2025, 09:27:29 |
  | Volume | 9 |
  | Publisher | MDPI |
  | Pages | 42 |
  | Publication | Animals |
  | Issue | 2 |
  | Date Added | 11/07/2025, 09:27:42 |
  | Modified | 04/01/2026, 11:25:33 |

  ### Tags:

  - horse
  - animal welfare
  - heart rate
  - emotion
  - empathy
  - animal behavior
  - nonhuman
  - physiological stress
  - social interaction
  - human
  - questionnaire
  - review
  - pain
  - veterinary medicine
  - human-animal bond
  - animal care
  - animal health
  - attitude
  - Equidae
  - experience
  - livestock
  - locus of control
  - nociception
  - perception
  - risk factor
  - thinking
  - veterinarian

  ### Attachments

  - Available Version (via Google Scholar)
  - Full Text (HTML)
- ## Early human contact and housing for pigs - part 1: responses to humans, novelty and isolation

  |  |  |
  | --- | --- |
  | Item Type | Journal Article |
  | Author | M.E. Lucas |
  | Author | L.M. Hemsworth |
  | Author | K.L. Butler |
  | Author | R.S. Morrison |
  | Author | A.J. Tilbrook |
  | Author | J.N. Marchant |
  | Author | J.-L. Rault |
  | Author | R.Y. Galea |
  | Author | P.H. Hemsworth |
  | Abstract | The development of fear and stress responses in animals can be influenced by early life experiences, including interactions with humans, maternal care, and the physical surroundings. This paper is the first of three reporting on a large experiment examining the effects of the early housing environment and early positive human contact on stress resilience in pigs. This first paper reports on the responses of pigs to humans, novelty, and social isolation. Using a 2 × 2 factorial design, 48 litters of pigs were reared in either a conventional farrowing crate (FC) where the sow was confined or a loose farrowing pen (LP; PigSAFE pen) which was larger, more physically complex and allowed the sow to move freely throughout the farrowing and lactation period. Piglets were provided with either routine contact from stockpeople (C), or routine contact plus regular opportunities for positive human contact (+HC) involving 5 min of scratching, patting and stroking imposed to the litter 5 days/week from 0-4 weeks of age. The positive handling treatment was highly effective in reducing piglets' fear of humans, based on +HC piglets showing greater approach and less avoidance of an unfamiliar person at 3 weeks of age. There was evidence that this reduction in fear of humans lasted well beyond when the treatment was applied (lactation), with +HC pigs showing greater approach and less avoidance of humans in tests at 6, 9 and 14 weeks of age. The +HC treatment also reduced piglets' fear of a novel object at 3 weeks of age, and for pigs in FC, the cortisol response after social isolation at 7 weeks of age. Rearing in FC compared to LP reduced piglets' fear of novelty at 3 weeks of age, as well as their vocalisations and cortisol response to isolation at 7 weeks of age. The FC pigs showed greater approach and less avoidance of humans compared to LP pigs at 3, 4 and 6 weeks of age, but not at 9 and 14 weeks of age. These results show that positive handling early in life can reduce pigs' fear of humans, fear of novelty and physiological stress response to social isolation. The LP pigs were reared in a more isolated environment with less overall contact with stockpeople and other pigs, which may have increased their fear responses to humans and novel situations, suggesting that different housing systems can modulate these pigs' responses. |
  | Date | 2024 |
  | Language | English |
  | Archive | Medline |
  | URL | https://www.embase.com/search/results?subaction=viewrecord&id=L644303098&from=export |
  | Volume | 18 |
  | Pages | 101164 |
  | Publication | Animal : an international journal of animal bioscience |
  | DOI | 10.1016/j.animal.2024.101164 |
  | Issue | 6 |
  | Journal Abbr | Animal |
  | ISSN | 1751-732X |
  | Date Added | 11/07/2025, 14:15:29 |
  | Modified | 11/07/2025, 14:15:29 |

  ### Tags:

  - human-animal interaction
  - physiology
  - animal behavior
  - female
  - male
  - human
  - hydrocortisone
  - social isolation
  - pig
  - animal
  - mental stress
  - psychology
  - fear
  - animal husbandry
  - animal housing
  - procedures

  ### Attachments

  - Full Text (HTML)
- ## Early human contact and housing for pigs - part 1: responses to humans, novelty and isolation

  |  |  |
  | --- | --- |
  | Item Type | Journal Article |
  | Author | M.E. Lucas |
  | Author | L.M. Hemsworth |
  | Author | K.L. Butler |
  | Author | R.S. Morrison |
  | Author | A.J. Tilbrook |
  | Author | J.N. Marchant |
  | Author | J.-L. Rault |
  | Author | R.Y. Galea |
  | Author | P.H. Hemsworth |
  | Abstract | The development of fear and stress responses in animals can be influenced by early life experiences, including interactions with humans, maternal care, and the physical surroundings. This paper is the first of three reporting on a large experiment examining the effects of the early housing environment and early positive human contact on stress resilience in pigs. This first paper reports on the responses of pigs to humans, novelty, and social isolation. Using a 2 × 2 factorial design, 48 litters of pigs were reared in either a conventional farrowing crate (FC) where the sow was confined or a loose farrowing pen (LP; PigSAFE pen) which was larger, more physically complex and allowed the sow to move freely throughout the farrowing and lactation period. Piglets were provided with either routine contact from stockpeople (C), or routine contact plus regular opportunities for positive human contact (+HC) involving 5 min of scratching, patting and stroking imposed to the litter 5 days/week from 0-4 weeks of age. The positive handling treatment was highly effective in reducing piglets' fear of humans, based on +HC piglets showing greater approach and less avoidance of an unfamiliar person at 3 weeks of age. There was evidence that this reduction in fear of humans lasted well beyond when the treatment was applied (lactation), with +HC pigs showing greater approach and less avoidance of humans in tests at 6, 9 and 14 weeks of age. The +HC treatment also reduced piglets' fear of a novel object at 3 weeks of age, and for pigs in FC, the cortisol response after social isolation at 7 weeks of age. Rearing in FC compared to LP reduced piglets' fear of novelty at 3 weeks of age, as well as their vocalisations and cortisol response to isolation at 7 weeks of age. The FC pigs showed greater approach and less avoidance of humans compared to LP pigs at 3, 4 and 6 weeks of age, but not at 9 and 14 weeks of age. These results show that positive handling early in life can reduce pigs' fear of humans, fear of novelty and physiological stress response to social isolation. The LP pigs were reared in a more isolated environment with less overall contact with stockpeople and other pigs, which may have increased their fear responses to humans and novel situations, suggesting that different housing systems can modulate these pigs' responses. |
  | Date | 2024 |
  | Language | English |
  | Archive | Medline |
  | URL | https://www.embase.com/search/results?subaction=viewrecord&id=L644303098&from=export |
  | Volume | 18 |
  | Pages | 101164 |
  | Publication | Animal : an international journal of animal bioscience |
  | DOI | 10.1016/j.animal.2024.101164 |
  | Issue | 6 |
  | Journal Abbr | Animal |
  | ISSN | 1751-732X |
  | Date Added | 11/07/2025, 14:16:39 |
  | Modified | 11/07/2025, 14:16:39 |

  ### Tags:

  - human-animal interaction
  - physiology
  - animal behavior
  - female
  - male
  - human
  - hydrocortisone
  - social isolation
  - pig
  - animal
  - mental stress
  - psychology
  - fear
  - animal husbandry
  - animal housing
  - procedures

  ### Attachments

  - Full Text (HTML)
- ## Early human contact and housing for pigs - part 1: responses to humans, novelty and isolation

  |  |  |
  | --- | --- |
  | Item Type | Journal Article |
  | Author | M.E. Lucas |
  | Author | L.M. Hemsworth |
  | Author | K.L. Butler |
  | Author | R.S. Morrison |
  | Author | A.J. Tilbrook |
  | Author | J.N. Marchant |
  | Author | J.-L. Rault |
  | Author | R.Y. Galea |
  | Author | P.H. Hemsworth |
  | Abstract | The development of fear and stress responses in animals can be influenced by early life experiences, including interactions with humans, maternal care, and the physical surroundings. This paper is the first of three reporting on a large experiment examining the effects of the early housing environment and early positive human contact on stress resilience in pigs. This first paper reports on the responses of pigs to humans, novelty, and social isolation. Using a 2 × 2 factorial design, 48 litters of pigs were reared in either a conventional farrowing crate (FC) where the sow was confined or a loose farrowing pen (LP; PigSAFE pen) which was larger, more physically complex and allowed the sow to move freely throughout the farrowing and lactation period. Piglets were provided with either routine contact from stockpeople (C), or routine contact plus regular opportunities for positive human contact (+HC) involving 5 min of scratching, patting and stroking imposed to the litter 5 days/week from 0-4 weeks of age. The positive handling treatment was highly effective in reducing piglets' fear of humans, based on +HC piglets showing greater approach and less avoidance of an unfamiliar person at 3 weeks of age. There was evidence that this reduction in fear of humans lasted well beyond when the treatment was applied (lactation), with +HC pigs showing greater approach and less avoidance of humans in tests at 6, 9 and 14 weeks of age. The +HC treatment also reduced piglets' fear of a novel object at 3 weeks of age, and for pigs in FC, the cortisol response after social isolation at 7 weeks of age. Rearing in FC compared to LP reduced piglets' fear of novelty at 3 weeks of age, as well as their vocalisations and cortisol response to isolation at 7 weeks of age. The FC pigs showed greater approach and less avoidance of humans compared to LP pigs at 3, 4 and 6 weeks of age, but not at 9 and 14 weeks of age. These results show that positive handling early in life can reduce pigs' fear of humans, fear of novelty and physiological stress response to social isolation. The LP pigs were reared in a more isolated environment with less overall contact with stockpeople and other pigs, which may have increased their fear responses to humans and novel situations, suggesting that different housing systems can modulate these pigs' responses. |
  | Date | 2024 |
  | Language | English |
  | Archive | Medline |
  | URL | https://www.embase.com/search/results?subaction=viewrecord&id=L644303098&from=export |
  | Volume | 18 |
  | Pages | 101164 |
  | Publication | Animal : an international journal of animal bioscience |
  | DOI | 10.1016/j.animal.2024.101164 |
  | Issue | 6 |
  | Journal Abbr | Animal |
  | ISSN | 1751-732X |
  | Date Added | 11/07/2025, 14:18:12 |
  | Modified | 11/07/2025, 14:18:12 |

  ### Tags:

  - human-animal interaction
  - physiology
  - animal behavior
  - female
  - male
  - human
  - hydrocortisone
  - social isolation
  - pig
  - animal
  - mental stress
  - psychology
  - fear
  - animal husbandry
  - animal housing
  - procedures

  ### Attachments

  - Full Text (HTML)
- ## Changes in salivary oxytocin after stroking in dogs: Validation of two assays for its assessment

  |  |  |
  | --- | --- |
  | Item Type | Journal Article |
  | Author | M. López-Arjona |
  | Author | S.V. Mateo |
  | Author | J.J. Cerón |
  | Author | S. Martínez-Subiela |
  | Abstract | Oxytocin is currently of high interest as a biomarker of welfare and stress in humans and animals. The purpose of this study was to validate two new assays (one using a monoclonal antibody and the other using a polyclonal antibody) for the oxytocin measurement in the saliva of dogs. For this purpose, an analytical validation was performed, and these assays were applied in an experimental trial in which dogs were stroked by their owners. In the experimental trial, saliva samples of 17 dogs were collected by the owners at three different times: a basal sample, at the end of 10 min of an affiliative interaction with their owners consisting of stroking and 15 min after the end of the affiliative interaction. The dogs were separated into two groups (group 1, n = 8 and group 2, n = 9) according to the acceptance of the sponge and the response to the stroking. Significant differences in the response of salivary oxytocin after stroking in the two groups were found when the assay with the monoclonal antibody was used. This assay showed a significant increase just after the end of affiliative interaction (P < 0.01) and 15 min after (P < 0.01) in those dogs that had a good acceptance of the sponge and the stroking induced a positive response on them (based in a Likert-type scale from 1 to 10). These data reflect that the assays used in this study can lead to different results when quantifying oxytocin in the saliva of dogs after stroking. |
  | Date | 2021 |
  | Language | English |
  | Archive | Embase |
  | URL | https://www.embase.com/search/results?subaction=viewrecord&id=L2011707054&from=export |
  | Volume | 136 |
  | Pages | 527-534 |
  | Publication | Research in Veterinary Science |
  | DOI | 10.1016/j.rvsc.2021.04.007 |
  | Issue | (López-Arjona M.; Mateo S.V.; Cerón J.J., jjceron@um.es; Martínez-Subiela S.) Interdisciplinary Laboratory of Clinical Analysis of the University of Murcia (Interlab-UMU), Regional Campus of International Excellence 'Campus Mare Nostrum', University of Murcia, Campus de Espinardo s/n, Espinardo, Murcia, Spain |
  | Journal Abbr | Res. Vet. Sci. |
  | ISSN | 1532-2661 |
  | Date Added | 11/07/2025, 12:12:25 |
  | Modified | 11/07/2025, 12:12:25 |

  ### Tags:

  - oxytocin
  - human-animal interaction
  - stroking
  - dog
  - article
  - female
  - male
  - nonhuman
  - controlled study
  - hormone determination
  - Likert scale
  - saliva level
  - dog breed
  - centrifugation
  - limit of detection
  - limit of quantitation
  - monoclonal antibody
  - saliva analysis
  - statistical analysis
  - supernatant
  - validation study

  ### Attachments

  - Full Text (HTML)
- ## Changes in salivary oxytocin after stroking in dogs: Validation of two assays for its assessment

  |  |  |
  | --- | --- |
  | Item Type | Journal Article |
  | Author | M. López-Arjona |
  | Author | S.V. Mateo |
  | Author | J.J. Cerón |
  | Author | S. Martínez-Subiela |
  | Abstract | Oxytocin is currently of high interest as a biomarker of welfare and stress in humans and animals. The purpose of this study was to validate two new assays (one using a monoclonal antibody and the other using a polyclonal antibody) for the oxytocin measurement in the saliva of dogs. For this purpose, an analytical validation was performed, and these assays were applied in an experimental trial in which dogs were stroked by their owners. In the experimental trial, saliva samples of 17 dogs were collected by the owners at three different times: a basal sample, at the end of 10 min of an affiliative interaction with their owners consisting of stroking and 15 min after the end of the affiliative interaction. The dogs were separated into two groups (group 1, n = 8 and group 2, n = 9) according to the acceptance of the sponge and the response to the stroking. Significant differences in the response of salivary oxytocin after stroking in the two groups were found when the assay with the monoclonal antibody was used. This assay showed a significant increase just after the end of affiliative interaction (P < 0.01) and 15 min after (P < 0.01) in those dogs that had a good acceptance of the sponge and the stroking induced a positive response on them (based in a Likert-type scale from 1 to 10). These data reflect that the assays used in this study can lead to different results when quantifying oxytocin in the saliva of dogs after stroking. |
  | Date | 2021 |
  | Language | English |
  | Archive | Embase |
  | URL | https://www.embase.com/search/results?subaction=viewrecord&id=L2011707054&from=export |
  | Volume | 136 |
  | Pages | 527-534 |
  | Publication | Research in Veterinary Science |
  | DOI | 10.1016/j.rvsc.2021.04.007 |
  | Issue | (López-Arjona M.; Mateo S.V.; Cerón J.J., jjceron@um.es; Martínez-Subiela S.) Interdisciplinary Laboratory of Clinical Analysis of the University of Murcia (Interlab-UMU), Regional Campus of International Excellence 'Campus Mare Nostrum', University of Murcia, Campus de Espinardo s/n, Espinardo, Murcia, Spain |
  | Journal Abbr | Res. Vet. Sci. |
  | ISSN | 1532-2661 |
  | Date Added | 11/07/2025, 12:13:27 |
  | Modified | 11/07/2025, 12:13:27 |

  ### Tags:

  - oxytocin
  - human-animal interaction
  - stroking
  - dog
  - article
  - female
  - male
  - nonhuman
  - controlled study
  - hormone determination
  - Likert scale
  - saliva level
  - dog breed
  - centrifugation
  - limit of detection
  - limit of quantitation
  - monoclonal antibody
  - saliva analysis
  - statistical analysis
  - supernatant
  - validation study

  ### Attachments

  - Full Text (HTML)
- ## Current approaches to modeling the virtual reality in rodents for the assessment of brain plasticity and behavior

  |  |  |
  | --- | --- |
  | Item Type | Journal Article |
  | Author | O.L. Lopatina |
  | Author | A.V. Morgun |
  | Author | Y.V. Gorina |
  | Author | V.V. Salmin |
  | Author | A.B. Salmina |
  | Abstract | Virtual reality (VR) and augmented reality (AR) have become valuable tools to study brains and behaviors resulting in development of new methods of diagnostics and treatment. Neurodegenerаtion is one of the best examples demonstrating efficacy of VR/АR technologies in modern neurology. Development of novel VR systems for rodents and combination of VR tools with up-to-date imaging techniques (i.e. MRI, imaging of neural networks etc.), brain electrophysiology (EEG, patch-clamp), precise analytics (microdialysis) allowed implementing of VR protocols into the animal neurobiology to study brain plasticity, sensorimotor integration, spatial navigation, memory, and decision-making. VR/AR for rodents is а young field of experimental neuroscience and has already provided more consistent testing conditions, less human-animal interaction, opportunities to use a wider variety of experimental parameters. Here we discuss present and future perspectives of using VR/AR to assess brain plasticity, neurogenesis and complex behavior in rodent and human study, and their advantages for translational neuroscience. |
  | Date | 2020 |
  | Language | English |
  | Archive | Embase |
  | URL | https://www.embase.com/search/results?subaction=viewrecord&id=L2004943544&from=export |
  | Volume | 335 |
  | Publication | Journal of Neuroscience Methods |
  | DOI | 10.1016/j.jneumeth.2020.108616 |
  | Issue | (Lopatina O.L., ol.lopatina@gmail.com; Morgun A.V.; Gorina Y.V.; Salmin V.V.; Salmina A.B.) Department of Biochemistry, Medical, Pharmaceutical, and Toxicological Chemistry, Krasnoyarsk State Medical University Named after Prof. V.F. Voino-Yasenetsky, Krasnoyarsk, Russian Federation |
  | Journal Abbr | J. Neurosci. Methods |
  | ISSN | 1872-678X |
  | Date Added | 11/07/2025, 11:49:33 |
  | Modified | 11/07/2025, 11:49:33 |

  ### Tags:

  - cognition
  - animal experiment
  - nonhuman
  - animal cell
  - animal model
  - augmented reality
  - nerve cell plasticity
  - nervous system development
  - neuroscience
  - review
  - rodent
  - virtual reality

  ### Attachments

  - Full Text (HTML)
- ## The paws that refresh us: Can animal-assisted therapy have an effect on exam performance in medical students?

  |  |  |
  | --- | --- |
  | Item Type | Journal Article |
  | Author | S.P. Lephart |
  | Author | C. Jennings |
  | Author | A.-N.C. Hamilton |
  | Author | A.R. Hoellein |
  | Abstract | Overview: Pet Therapy, also referred to as animal-assisted intervention (AAI) and animal-assisted therapy (AAT), is defined as any practice that involves animals as a part of a therapeutic or ameliorative process with the goal of positively affecting human health by utilizing animals as adjuncts to therapy. Human-animal interaction has been shown to provide numerous psychological benefits. Some of these include decreased levels of anxiety, sadness, and increased social functioning, and the secretion of the neuropeptide oxytocin during human-animal contact has been suggested to be a modulating factor. Additionally, interaction with a friendly dog can increase short-term tolerance of aversive acoustic stimulation. Based on this information, how then do first and second year medical students respond to interactions with AAT at various times around their scheduled exams? This innovative intervention is being offered at different intervals for the first and second year medical students at the University of Kentucky. These interactions include contact with AAT before or after their exams, with an occasional option for interactions both before and after, or for no interaction around the exam at all. Brief surveys are administered electronically to the medical students after each interaction for feedback specific to their reactions and perceptions of how this intervention may or may not have effected their performance or state of mind during and/or around the exam period. Rationale: A 2016 study by Rotenstein et al., found that more than 27% of medical students suffer from depression or symptoms of it, and that 11% reported suicidal thoughts. These future leaders of our medical world are at the beginning of their careers when the chance is ripe for them to learn novel, integrative management techniques for stress and anxiety. In doing so, they will not only acquire the knowledge of how AAT can help modulate their own psychological state, but also help them realize the value of implementing this modality in the lives of their future patients. Objectives: To gain an objective understanding of the value of AAT as a modality in medical student training, to obtain innovative ideas for further utilization of AAT and to acquire a model for replication and future study. |
  | Date | 2018 |
  | Language | English |
  | Archive | Embase |
  | URL | https://www.embase.com/search/results?subaction=viewrecord&id=L625880127&from=export |
  | Volume | 7 |
  | Pages | 203-204 |
  | Publication | Global Advances in Health and Medicine |
  | DOI | 10.1177/2164956118773837 |
  | Issue | (Lephart S.P.; Jennings C.; Hamilton A.-N.C.; Hoellein A.R.) University of Kentucky, Lexington, KY, United States |
  | ISSN | 2164-9561 |
  | Date Added | 11/07/2025, 12:13:33 |
  | Modified | 11/07/2025, 12:13:33 |

  ### Tags:

  - oxytocin
  - dog
  - anxiety
  - animal experiment
  - female
  - male
  - nonhuman
  - physiological stress
  - social interaction
  - adult
  - animal model
  - auditory stimulation
  - endogenous compound
  - perception
  - pet therapy
  - paw
  - neuropeptide
  - conference abstract
  - career
  - Kentucky
  - leadership
  - medical student
  - sadness
  - suicidal ideation

  ### Attachments

  - Full Text (HTML)
- ## Quantitative heartbeat coupling measures in human-horse interaction

  |  |  |
  | --- | --- |
  | Item Type | Journal Article |
  | Author | A. Lanata |
  | Author | A. Guidi |
  | Author | G. Valenza |
  | Author | P. Baragli |
  | Author | E.P. Scilingo |
  | Abstract | We present a study focused on a quantitative estimation of a human-horse dynamic interaction. A set of measures based on magnitude and phase coupling between heartbeat dynamics of both humans and horses in three different conditions is reported: no interaction, visual/olfactory interaction and grooming. Specifically, Magnitude Squared Coherence (MSC), Mean Phase Coherence (MPC) and Dynamic Time Warping (DTW) have been used as estimators of the amount of coupling between human and horse through the analysis of their heart rate variability (HRV) time series in a group of eleven human subjects, and one horse. The rationale behind this study is that the interaction of two complex biological systems go towards a coupling process whose dynamical evolution is modulated by the kind and time duration of the interaction itself. We achieved a congruent and consistent statistical significant difference for all of the three indices. Moreover, a Nearest Mean Classifier was able to recognize the three classes of interaction with an accuracy greater than 70%. Although preliminary, these encouraging results allow a discrimination of three distinct phases in a real human-animal interaction opening to the characterization of the empirically proven relationship between human and horse. |
  | Date | 2016 |
  | Language | English |
  | Archive | Medline |
  | URL | https://www.embase.com/search/results?subaction=viewrecord&id=L617664121&from=export |
  | Volume | 2016 |
  | Pages | 2696-2699 |
  | Publication | Conference proceedings : ... Annual International Conference of the IEEE Engineering in Medicine and Biology Society. IEEE Engineering in Medicine and Biology Society. Annual Conference |
  | DOI | 10.1109/EMBC.2016.7591286 |
  | Issue | (Lanata A.; Guidi A.; Valenza G.; Baragli P.; Scilingo E.P.) |
  | Journal Abbr | Conf Proc IEEE Eng Med Biol Soc |
  | ISSN | 1557-170X |
  | Date Added | 11/07/2025, 12:10:55 |
  | Modified | 11/07/2025, 12:10:55 |

  ### Tags:

  - horse
  - heart rate
  - human
  - animal
  - animal husbandry

  ### Attachments

  - Full Text (HTML)
- ## Veterans and Shelter Dogs: Examining the Impact of a Dog-Walking Intervention on Physiological and Post-Traumatic Stress Symptoms

  |  |  |
  | --- | --- |
  | Item Type | Journal Article |
  | Author | Cheryl A. Krause-Parello |
  | Author | Erika Friedmann |
  | Author | Kelly Blanchard |
  | Author | Megan Payton |
  | Author | Nancy R. Gee |
  | Date | 2020 |
  | URL | https://www.scopus.com/inward/record.uri?eid=2-s2.0-85081891890&doi=10.1080%2f08927936.2020.1719763&partnerID=40&md5=0da00f53aa0bd1b2b6b949f0cb60934b |
  | Extra | Type: Article |
  | Volume | 33 |
  | Pages | 225 – 241 |
  | Publication | Anthrozoos |
  | DOI | 10.1080/08927936.2020.1719763 |
  | Issue | 2 |
  | Date Added | 19/06/2025, 15:40:12 |
  | Modified | 04/01/2026, 11:26:09 |

  ### Tags:

  - veteran
  - heart rate variability
  - dog
  - animal experiment
  - article
  - female
  - male
  - nonhuman
  - physiological stress
  - human
  - hydrocortisone
  - adult
  - controlled study
  - posttraumatic stress disorder
  - crossover procedure
  - animal model
  - mental stress
  - outcome assessment
  - amylase
  - endogenous compound
  - gene expression
  - protein expression
  - sample size
  - walking

  ### Notes:

  - Cited by: 10
  - Cited by: 10

  ### Attachments

  - Full Text (HTML)
  - Full Text (HTML)
  - Full Text (HTML)
- ## Examining the Effects of a Service-Trained Facility Dog on Stress in Children Undergoing Forensic Interview for Allegations of Child Sexual Abuse

  |  |  |
  | --- | --- |
  | Item Type | Journal Article |
  | Author | C.A. Krause-Parello |
  | Author | M. Thames |
  | Author | C.M. Ray |
  | Author | J. Kolassa |
  | Abstract | Disclosure of child sexual abuse can be a stressful experience for the child. Gaining a better understanding of how best to serve the child, while preserving the quality of their disclosure, is an ever-evolving process. The data to answer this question come from 51 children aged 4-16 (M = 9.1, SD = 3.5), who were referred to a child advocacy center in Virginia for a forensic interview (FI) following allegations of sexual abuse. A repeated measures design was conducted to examine how the presence of a service-trained facility dog (e.g. animal-assisted intervention (AAI) may serve as a mode of lowering stress levels in children during their FIs. Children were randomized to one of the two FI conditions: experimental condition (service-trained facility dog present-AAI) or control condition (service-trained facility dog not present- standard forensic interview). Stress biomarkers salivary cortisol, alpha-amylase, immunoglobulin A (IgA), heart rate, and blood pressure, and Immunoglobulin A were collected before and after the FI. Self-report data were also collected. Results supported a significant decrease in heart rate for those in the experimental condition (p = .0086) vs the control condition (p = .4986). Regression models revealed a significant decrease in systolic and diastolic blood pressure in the experimental condition (p = .03285) and (p = .04381), respectively. Statistically significant changes in alpha-amylase and IgA were also found in relation to disclosure and type of offense. The results of this study support the stress reducing effects of a service-trained facility dog for children undergoing FI for allegations of child sexual abuse. |
  | Date | 2018 |
  | Language | English |
  | Archive | Medline |
  | URL | https://www.embase.com/search/results?subaction=viewrecord&id=L624043229&from=export |
  | Volume | 27 |
  | Pages | 305-320 |
  | Publication | Journal of child sexual abuse |
  | DOI | 10.1080/10538712.2018.1443303 |
  | Issue | 3 |
  | Journal Abbr | J Child Sex Abus |
  | ISSN | 1547-0679 |
  | Date Added | 11/07/2025, 14:18:36 |
  | Modified | 11/07/2025, 14:18:36 |

  ### Tags:

  - blood pressure
  - physiology
  - heart rate
  - dog
  - saliva
  - therapy
  - child
  - female
  - male
  - human
  - hydrocortisone
  - adolescent
  - animal
  - mental stress
  - psychology
  - chemistry
  - human-animal bond
  - alpha amylase saliva isoenzyme
  - interpersonal communication
  - child sexual abuse
  - child advocacy
  - diagnosis
  - forensic psychiatry
  - preschool child
  - psychological interview

  ### Attachments

  - Full Text (HTML)
- ## Sociality of Cats toward Humans Can Be Influenced by Hormonal and Socio-Environmental Factors: Pilot Study

  |  |  |
  | --- | --- |
  | Item Type | Journal Article |
  | Author | H. Koyasu |
  | Author | H. Takahashi |
  | Author | I. Sasao |
  | Author | S. Takagi |
  | Author | M. Nagasawa |
  | Author | T. Kikusui |
  | Abstract | Individual differences in the sociality of cats are influenced by inherited and environmental factors. We recently revealed that hormones can make a difference in intraspecies social behavior. It remains unclear whether cat behavior toward humans is modulated by hormones. Therefore, we analyzed the relationship between cat behavior and their basal hormone concentrations after spending time together with human experimenters. In addition, we analyzed the relationship between cat behavior and the timing of when the individual cats began living with a human because the sociality of cats could be dependent on their developmental experiences. The results showed that male cats that began living with humans earlier had more contact with an experimenter. In addition, individual male cats with low testosterone levels were more likely to interact with an experimenter. These findings of this pilot study suggest that the sociality of male cats toward humans is affected by testosterone and the age at which they begin to live with humans. |
  | Date | 2023 |
  | Language | English |
  | Archive | Embase |
  | URL | https://www.embase.com/search/results?subaction=viewrecord&id=L2020954725&from=export |
  | Volume | 13 |
  | Publication | Animals |
  | DOI | 10.3390/ani13010146 |
  | Issue | 1 |
  | Journal Abbr | Animals |
  | ISSN | 2076-2615 |
  | Date Added | 11/07/2025, 12:12:18 |
  | Modified | 11/07/2025, 12:12:18 |

  ### Tags:

  - oxytocin
  - social behavior
  - testosterone
  - animal experiment
  - article
  - female
  - male
  - nonhuman
  - human
  - hydrocortisone
  - computer
  - controlled study
  - ELISA kit
  - pilot study
  - scoring system
  - creatinine
  - human experiment
  - enzyme linked immunosorbent assay
  - cat
  - camera
  - socialization
  - ADI 901 153A 0001
  - ELISA washer
  - environmental factor
  - HDR-AS50R
  - hormone
  - microplate reader
  - Model 680XR

  ### Attachments

  - Full Text (HTML)
- ## Correlations between behavior and hormone concentrations or gut microbiome imply that domestic cats (Felis silvestris catus) living in a group are not like ‘groupmates’

  |  |  |
  | --- | --- |
  | Item Type | Journal Article |
  | Author | H. Koyasu |
  | Author | H. Takahashi |
  | Author | M. Yoneda |
  | Author | S. Naba |
  | Author | N. Sakawa |
  | Author | I. Sasao |
  | Author | M. Nagasawa |
  | Author | T. Kikusui |
  | Abstract | Domestic cats (Felis silvestris catus) can live in high densities, although most feline species are solitary and exclusively territorial animals; it is possible that certain behavioral strategies enable this phenomenon. These behaviors are regulated by hormones and the gut microbiome, which, in turn, is influenced by domestication. Therefore, we investigated the relationships between the sociality, hormone concentrations, and gut microbiome of domestic cats by conducting three sets of experiments for each group of five cats and analyzing their behavior, hormone concentrations (cortisol, oxytocin, and testosterone), and their gut microbiomes. We observed that individuals with high cortisol and testosterone concentrations established less contact with others, and individuals with high oxytocin concentrations did not exhibit affiliative behaviors as much as expected. Additionally, the higher the frequency of contact among the individuals, the greater the similarity in gut microbiome; gut microbial composition was also related to behavioral patterns and cortisol secretion. Notably, individuals with low cortisol and testosterone concentrations were highly tolerant, making high-density living easy. Oxytocin usually functions in an affiliative manner within groups, but our results suggest that even if typically solitary and territorial animals live in high densities, their oxytocin functions are opposite to those of typically group-living animals. |
  | Date | 2022 |
  | Language | English |
  | Archive | Embase |
  | URL | https://www.embase.com/search/results?subaction=viewrecord&id=L2019496855&from=export |
  | Volume | 17 |
  | Publication | PLoS ONE |
  | DOI | 10.1371/journal.pone.0269589 |
  | Issue | 7 July |
  | Journal Abbr | PLoS ONE |
  | ISSN | 1932-6203 |
  | Date Added | 11/07/2025, 12:12:21 |
  | Modified | 11/07/2025, 12:12:21 |

  ### Tags:

  - oxytocin
  - behavior
  - testosterone
  - article
  - human
  - hydrocortisone
  - ELISA kit
  - enzyme linked immunosorbent assay
  - domestic cat
  - camera
  - hormone urine level
  - hormone
  - microplate reader
  - DNA extraction
  - food
  - gastrointestinal tract
  - HX A1H
  - intestine flora
  - MODEL 680XR
  - nucleic acid isolation kit
  - polymerase chain reaction

  ### Attachments

  - Full Text (HTML)
- ## Sociality of Cats toward Humans Can Be Influenced by Hormonal and Socio-Environmental Factors: Pilot Study

  |  |  |
  | --- | --- |
  | Item Type | Journal Article |
  | Author | H. Koyasu |
  | Author | H. Takahashi |
  | Author | I. Sasao |
  | Author | S. Takagi |
  | Author | M. Nagasawa |
  | Author | T. Kikusui |
  | Abstract | Individual differences in the sociality of cats are influenced by inherited and environmental factors. We recently revealed that hormones can make a difference in intraspecies social behavior. It remains unclear whether cat behavior toward humans is modulated by hormones. Therefore, we analyzed the relationship between cat behavior and their basal hormone concentrations after spending time together with human experimenters. In addition, we analyzed the relationship between cat behavior and the timing of when the individual cats began living with a human because the sociality of cats could be dependent on their developmental experiences. The results showed that male cats that began living with humans earlier had more contact with an experimenter. In addition, individual male cats with low testosterone levels were more likely to interact with an experimenter. These findings of this pilot study suggest that the sociality of male cats toward humans is affected by testosterone and the age at which they begin to live with humans. |
  | Date | 2023 |
  | Language | English |
  | Archive | Embase |
  | URL | https://www.embase.com/search/results?subaction=viewrecord&id=L2020954725&from=export |
  | Volume | 13 |
  | Publication | Animals |
  | DOI | 10.3390/ani13010146 |
  | Issue | 1 |
  | Journal Abbr | Animals |
  | ISSN | 2076-2615 |
  | Date Added | 11/07/2025, 12:13:20 |
  | Modified | 11/07/2025, 12:13:20 |

  ### Tags:

  - oxytocin
  - social behavior
  - testosterone
  - animal experiment
  - article
  - female
  - male
  - nonhuman
  - human
  - hydrocortisone
  - computer
  - controlled study
  - ELISA kit
  - pilot study
  - scoring system
  - creatinine
  - human experiment
  - enzyme linked immunosorbent assay
  - cat
  - camera
  - socialization
  - ADI 901 153A 0001
  - ELISA washer
  - environmental factor
  - HDR-AS50R
  - hormone
  - microplate reader
  - Model 680XR

  ### Attachments

  - Full Text (HTML)
- ## Correlations between behavior and hormone concentrations or gut microbiome imply that domestic cats (Felis silvestris catus) living in a group are not like ‘groupmates’

  |  |  |
  | --- | --- |
  | Item Type | Journal Article |
  | Author | H. Koyasu |
  | Author | H. Takahashi |
  | Author | M. Yoneda |
  | Author | S. Naba |
  | Author | N. Sakawa |
  | Author | I. Sasao |
  | Author | M. Nagasawa |
  | Author | T. Kikusui |
  | Abstract | Domestic cats (Felis silvestris catus) can live in high densities, although most feline species are solitary and exclusively territorial animals; it is possible that certain behavioral strategies enable this phenomenon. These behaviors are regulated by hormones and the gut microbiome, which, in turn, is influenced by domestication. Therefore, we investigated the relationships between the sociality, hormone concentrations, and gut microbiome of domestic cats by conducting three sets of experiments for each group of five cats and analyzing their behavior, hormone concentrations (cortisol, oxytocin, and testosterone), and their gut microbiomes. We observed that individuals with high cortisol and testosterone concentrations established less contact with others, and individuals with high oxytocin concentrations did not exhibit affiliative behaviors as much as expected. Additionally, the higher the frequency of contact among the individuals, the greater the similarity in gut microbiome; gut microbial composition was also related to behavioral patterns and cortisol secretion. Notably, individuals with low cortisol and testosterone concentrations were highly tolerant, making high-density living easy. Oxytocin usually functions in an affiliative manner within groups, but our results suggest that even if typically solitary and territorial animals live in high densities, their oxytocin functions are opposite to those of typically group-living animals. |
  | Date | 2022 |
  | Language | English |
  | Archive | Embase |
  | URL | https://www.embase.com/search/results?subaction=viewrecord&id=L2019496855&from=export |
  | Volume | 17 |
  | Publication | PLoS ONE |
  | DOI | 10.1371/journal.pone.0269589 |
  | Issue | 7 July |
  | Journal Abbr | PLoS ONE |
  | ISSN | 1932-6203 |
  | Date Added | 11/07/2025, 12:13:22 |
  | Modified | 11/07/2025, 12:13:22 |

  ### Tags:

  - oxytocin
  - behavior
  - testosterone
  - article
  - human
  - hydrocortisone
  - ELISA kit
  - enzyme linked immunosorbent assay
  - domestic cat
  - camera
  - hormone urine level
  - hormone
  - microplate reader
  - DNA extraction
  - food
  - gastrointestinal tract
  - HX A1H
  - intestine flora
  - MODEL 680XR
  - nucleic acid isolation kit
  - polymerase chain reaction

  ### Attachments

  - Full Text (HTML)
- ## Sociality of Cats toward Humans Can Be Influenced by Hormonal and Socio-Environmental Factors: Pilot Study

  |  |  |
  | --- | --- |
  | Item Type | Journal Article |
  | Author | H. Koyasu |
  | Author | H. Takahashi |
  | Author | I. Sasao |
  | Author | S. Takagi |
  | Author | M. Nagasawa |
  | Author | T. Kikusui |
  | Abstract | Individual differences in the sociality of cats are influenced by inherited and environmental factors. We recently revealed that hormones can make a difference in intraspecies social behavior. It remains unclear whether cat behavior toward humans is modulated by hormones. Therefore, we analyzed the relationship between cat behavior and their basal hormone concentrations after spending time together with human experimenters. In addition, we analyzed the relationship between cat behavior and the timing of when the individual cats began living with a human because the sociality of cats could be dependent on their developmental experiences. The results showed that male cats that began living with humans earlier had more contact with an experimenter. In addition, individual male cats with low testosterone levels were more likely to interact with an experimenter. These findings of this pilot study suggest that the sociality of male cats toward humans is affected by testosterone and the age at which they begin to live with humans. |
  | Date | 2023 |
  | Language | English |
  | Archive | Embase |
  | URL | https://www.embase.com/search/results?subaction=viewrecord&id=L2020954725&from=export |
  | Volume | 13 |
  | Publication | Animals |
  | DOI | 10.3390/ani13010146 |
  | Issue | 1 |
  | Journal Abbr | Animals |
  | ISSN | 2076-2615 |
  | Date Added | 11/07/2025, 14:15:36 |
  | Modified | 11/07/2025, 14:15:36 |

  ### Tags:

  - oxytocin
  - social behavior
  - testosterone
  - animal experiment
  - article
  - female
  - male
  - nonhuman
  - human
  - hydrocortisone
  - computer
  - controlled study
  - ELISA kit
  - pilot study
  - scoring system
  - creatinine
  - human experiment
  - enzyme linked immunosorbent assay
  - cat
  - camera
  - socialization
  - ADI 901 153A 0001
  - ELISA washer
  - environmental factor
  - HDR-AS50R
  - hormone
  - microplate reader
  - Model 680XR

  ### Attachments

  - Full Text (HTML)
- ## Sociality of Cats toward Humans Can Be Influenced by Hormonal and Socio-Environmental Factors: Pilot Study

  |  |  |
  | --- | --- |
  | Item Type | Journal Article |
  | Author | H. Koyasu |
  | Author | H. Takahashi |
  | Author | I. Sasao |
  | Author | S. Takagi |
  | Author | M. Nagasawa |
  | Author | T. Kikusui |
  | Abstract | Individual differences in the sociality of cats are influenced by inherited and environmental factors. We recently revealed that hormones can make a difference in intraspecies social behavior. It remains unclear whether cat behavior toward humans is modulated by hormones. Therefore, we analyzed the relationship between cat behavior and their basal hormone concentrations after spending time together with human experimenters. In addition, we analyzed the relationship between cat behavior and the timing of when the individual cats began living with a human because the sociality of cats could be dependent on their developmental experiences. The results showed that male cats that began living with humans earlier had more contact with an experimenter. In addition, individual male cats with low testosterone levels were more likely to interact with an experimenter. These findings of this pilot study suggest that the sociality of male cats toward humans is affected by testosterone and the age at which they begin to live with humans. |
  | Date | 2023 |
  | Language | English |
  | Archive | Embase |
  | URL | https://www.embase.com/search/results?subaction=viewrecord&id=L2020954725&from=export |
  | Volume | 13 |
  | Publication | Animals |
  | DOI | 10.3390/ani13010146 |
  | Issue | 1 |
  | Journal Abbr | Animals |
  | ISSN | 2076-2615 |
  | Date Added | 11/07/2025, 14:16:46 |
  | Modified | 11/07/2025, 14:16:46 |

  ### Tags:

  - oxytocin
  - social behavior
  - testosterone
  - animal experiment
  - article
  - female
  - male
  - nonhuman
  - human
  - hydrocortisone
  - computer
  - controlled study
  - ELISA kit
  - pilot study
  - scoring system
  - creatinine
  - human experiment
  - enzyme linked immunosorbent assay
  - cat
  - camera
  - socialization
  - ADI 901 153A 0001
  - ELISA washer
  - environmental factor
  - HDR-AS50R
  - hormone
  - microplate reader
  - Model 680XR

  ### Attachments

  - Full Text (HTML)
- ## Correlations between behavior and hormone concentrations or gut microbiome imply that domestic cats (Felis silvestris catus) living in a group are not like ‘groupmates’

  |  |  |
  | --- | --- |
  | Item Type | Journal Article |
  | Author | H. Koyasu |
  | Author | H. Takahashi |
  | Author | M. Yoneda |
  | Author | S. Naba |
  | Author | N. Sakawa |
  | Author | I. Sasao |
  | Author | M. Nagasawa |
  | Author | T. Kikusui |
  | Abstract | Domestic cats (Felis silvestris catus) can live in high densities, although most feline species are solitary and exclusively territorial animals; it is possible that certain behavioral strategies enable this phenomenon. These behaviors are regulated by hormones and the gut microbiome, which, in turn, is influenced by domestication. Therefore, we investigated the relationships between the sociality, hormone concentrations, and gut microbiome of domestic cats by conducting three sets of experiments for each group of five cats and analyzing their behavior, hormone concentrations (cortisol, oxytocin, and testosterone), and their gut microbiomes. We observed that individuals with high cortisol and testosterone concentrations established less contact with others, and individuals with high oxytocin concentrations did not exhibit affiliative behaviors as much as expected. Additionally, the higher the frequency of contact among the individuals, the greater the similarity in gut microbiome; gut microbial composition was also related to behavioral patterns and cortisol secretion. Notably, individuals with low cortisol and testosterone concentrations were highly tolerant, making high-density living easy. Oxytocin usually functions in an affiliative manner within groups, but our results suggest that even if typically solitary and territorial animals live in high densities, their oxytocin functions are opposite to those of typically group-living animals. |
  | Date | 2022 |
  | Language | English |
  | Archive | Embase |
  | URL | https://www.embase.com/search/results?subaction=viewrecord&id=L2019496855&from=export |
  | Volume | 17 |
  | Publication | PLoS ONE |
  | DOI | 10.1371/journal.pone.0269589 |
  | Issue | 7 July |
  | Journal Abbr | PLoS ONE |
  | ISSN | 1932-6203 |
  | Date Added | 11/07/2025, 14:16:47 |
  | Modified | 11/07/2025, 14:16:47 |

  ### Tags:

  - oxytocin
  - behavior
  - testosterone
  - article
  - human
  - hydrocortisone
  - ELISA kit
  - enzyme linked immunosorbent assay
  - domestic cat
  - camera
  - hormone urine level
  - hormone
  - microplate reader
  - DNA extraction
  - food
  - gastrointestinal tract
  - HX A1H
  - intestine flora
  - MODEL 680XR
  - nucleic acid isolation kit
  - polymerase chain reaction

  ### Attachments

  - Full Text (HTML)
- ## Sociality of Cats toward Humans Can Be Influenced by Hormonal and Socio-Environmental Factors: Pilot Study

  |  |  |
  | --- | --- |
  | Item Type | Journal Article |
  | Author | H. Koyasu |
  | Author | H. Takahashi |
  | Author | I. Sasao |
  | Author | S. Takagi |
  | Author | M. Nagasawa |
  | Author | T. Kikusui |
  | Abstract | Individual differences in the sociality of cats are influenced by inherited and environmental factors. We recently revealed that hormones can make a difference in intraspecies social behavior. It remains unclear whether cat behavior toward humans is modulated by hormones. Therefore, we analyzed the relationship between cat behavior and their basal hormone concentrations after spending time together with human experimenters. In addition, we analyzed the relationship between cat behavior and the timing of when the individual cats began living with a human because the sociality of cats could be dependent on their developmental experiences. The results showed that male cats that began living with humans earlier had more contact with an experimenter. In addition, individual male cats with low testosterone levels were more likely to interact with an experimenter. These findings of this pilot study suggest that the sociality of male cats toward humans is affected by testosterone and the age at which they begin to live with humans. |
  | Date | 2023 |
  | Language | English |
  | Archive | Embase |
  | URL | https://www.embase.com/search/results?subaction=viewrecord&id=L2020954725&from=export |
  | Volume | 13 |
  | Publication | Animals |
  | DOI | 10.3390/ani13010146 |
  | Issue | 1 |
  | Journal Abbr | Animals |
  | ISSN | 2076-2615 |
  | Date Added | 11/07/2025, 14:18:19 |
  | Modified | 11/07/2025, 14:18:19 |

  ### Tags:

  - oxytocin
  - social behavior
  - testosterone
  - animal experiment
  - article
  - female
  - male
  - nonhuman
  - human
  - hydrocortisone
  - computer
  - controlled study
  - ELISA kit
  - pilot study
  - scoring system
  - creatinine
  - human experiment
  - enzyme linked immunosorbent assay
  - cat
  - camera
  - socialization
  - ADI 901 153A 0001
  - ELISA washer
  - environmental factor
  - HDR-AS50R
  - hormone
  - microplate reader
  - Model 680XR

  ### Attachments

  - Full Text (HTML)
- ## Correlations between behavior and hormone concentrations or gut microbiome imply that domestic cats (Felis silvestris catus) living in a group are not like ‘groupmates’

  |  |  |
  | --- | --- |
  | Item Type | Journal Article |
  | Author | H. Koyasu |
  | Author | H. Takahashi |
  | Author | M. Yoneda |
  | Author | S. Naba |
  | Author | N. Sakawa |
  | Author | I. Sasao |
  | Author | M. Nagasawa |
  | Author | T. Kikusui |
  | Abstract | Domestic cats (Felis silvestris catus) can live in high densities, although most feline species are solitary and exclusively territorial animals; it is possible that certain behavioral strategies enable this phenomenon. These behaviors are regulated by hormones and the gut microbiome, which, in turn, is influenced by domestication. Therefore, we investigated the relationships between the sociality, hormone concentrations, and gut microbiome of domestic cats by conducting three sets of experiments for each group of five cats and analyzing their behavior, hormone concentrations (cortisol, oxytocin, and testosterone), and their gut microbiomes. We observed that individuals with high cortisol and testosterone concentrations established less contact with others, and individuals with high oxytocin concentrations did not exhibit affiliative behaviors as much as expected. Additionally, the higher the frequency of contact among the individuals, the greater the similarity in gut microbiome; gut microbial composition was also related to behavioral patterns and cortisol secretion. Notably, individuals with low cortisol and testosterone concentrations were highly tolerant, making high-density living easy. Oxytocin usually functions in an affiliative manner within groups, but our results suggest that even if typically solitary and territorial animals live in high densities, their oxytocin functions are opposite to those of typically group-living animals. |
  | Date | 2022 |
  | Language | English |
  | Archive | Embase |
  | URL | https://www.embase.com/search/results?subaction=viewrecord&id=L2019496855&from=export |
  | Volume | 17 |
  | Publication | PLoS ONE |
  | DOI | 10.1371/journal.pone.0269589 |
  | Issue | 7 July |
  | Journal Abbr | PLoS ONE |
  | ISSN | 1932-6203 |
  | Date Added | 11/07/2025, 14:18:21 |
  | Modified | 11/07/2025, 14:18:21 |

  ### Tags:

  - oxytocin
  - behavior
  - testosterone
  - article
  - human
  - hydrocortisone
  - ELISA kit
  - enzyme linked immunosorbent assay
  - domestic cat
  - camera
  - hormone urine level
  - hormone
  - microplate reader
  - DNA extraction
  - food
  - gastrointestinal tract
  - HX A1H
  - intestine flora
  - MODEL 680XR
  - nucleic acid isolation kit
  - polymerase chain reaction

  ### Attachments

  - Full Text (HTML)
- ## Behavioral and emotional co-modulation during dog-owner interaction measured by heart rate variability and activity

  |  |  |
  | --- | --- |
  | Item Type | Journal Article |
  | Author | A. Koskela |
  | Author | H. Törnqvist |
  | Author | S. Somppi |
  | Author | K. Tiira |
  | Author | V.-L. Kykyri |
  | Author | L. Hänninen |
  | Author | J. Kujala |
  | Author | M. Nagasawa |
  | Author | T. Kikusui |
  | Author | M.V. Kujala |
  | Abstract | Behavioral and physiological synchrony facilitate emotional closeness in attachment relationships. The aim of this pseudorandomized cross-over study was to investigate the emotional and physiological link, designated as co-modulation, between dogs and their owners. We measured the heart rate variability (HRV) and physical activity of dogs belonging to co-operative breeds (n = 29) and their owners during resting baselines and positive interaction tasks (Stroking, Training, Sniffing, Playing) and collected survey data on owner temperament and dog-owner relationship. Although overall HRV and activity correlated between dogs and their owners across tasks, task-specific analyses showed that HRV of dogs and owners correlated during free behaving (Pre- and Post-Baseline), whereas the activity of dogs and owners correlated during predefined interaction tasks (Stroking and Playing). Dog overall HRV was the only predictive factor for owner overall HRV, while dog height, ownership duration, owner negative affectivity, and dog-owner interaction scale predicted dog overall HRV. Thus, the characteristics of dog, owner, and the relationship modified the HRV responses in dog-owner dyads. The physiology and behavior of dogs belonging to co-operative breeds and their owners were therefore co-modulated, demonstrating physiological and emotional connection comparable to those found in attachment relationships between humans. |
  | Date | 2024 |
  | Language | English |
  | Archive | Medline |
  | URL | https://www.embase.com/search/results?subaction=viewrecord&id=L645616175&from=export |
  | Volume | 14 |
  | Pages | 25201 |
  | Publication | Scientific reports |
  | DOI | 10.1038/s41598-024-76831-x |
  | Issue | 1 |
  | Journal Abbr | Sci Rep |
  | ISSN | 2045-2322 |
  | Date Added | 11/07/2025, 11:53:18 |
  | Modified | 11/07/2025, 11:53:18 |

  ### Tags:

  - human-animal interaction
  - physiology
  - heart rate
  - dog
  - emotion
  - animal behavior
  - female
  - male
  - human
  - adult
  - crossover procedure
  - animal
  - human-animal bond
  - middle aged

  ### Attachments

  - Full Text (HTML)
- ## Behavioral and emotional co-modulation during dog-owner interaction measured by heart rate variability and activity

  |  |  |
  | --- | --- |
  | Item Type | Journal Article |
  | Author | A. Koskela |
  | Author | H. Törnqvist |
  | Author | S. Somppi |
  | Author | K. Tiira |
  | Author | V.-L. Kykyri |
  | Author | L. Hänninen |
  | Author | J. Kujala |
  | Author | M. Nagasawa |
  | Author | T. Kikusui |
  | Author | M.V. Kujala |
  | Abstract | Behavioral and physiological synchrony facilitate emotional closeness in attachment relationships. The aim of this pseudorandomized cross-over study was to investigate the emotional and physiological link, designated as co-modulation, between dogs and their owners. We measured the heart rate variability (HRV) and physical activity of dogs belonging to co-operative breeds (n = 29) and their owners during resting baselines and positive interaction tasks (Stroking, Training, Sniffing, Playing) and collected survey data on owner temperament and dog-owner relationship. Although overall HRV and activity correlated between dogs and their owners across tasks, task-specific analyses showed that HRV of dogs and owners correlated during free behaving (Pre- and Post-Baseline), whereas the activity of dogs and owners correlated during predefined interaction tasks (Stroking and Playing). Dog overall HRV was the only predictive factor for owner overall HRV, while dog height, ownership duration, owner negative affectivity, and dog-owner interaction scale predicted dog overall HRV. Thus, the characteristics of dog, owner, and the relationship modified the HRV responses in dog-owner dyads. The physiology and behavior of dogs belonging to co-operative breeds and their owners were therefore co-modulated, demonstrating physiological and emotional connection comparable to those found in attachment relationships between humans. |
  | Date | 2024 |
  | Language | English |
  | Archive | Medline |
  | URL | https://www.embase.com/search/results?subaction=viewrecord&id=L645616175&from=export |
  | Volume | 14 |
  | Pages | 25201 |
  | Publication | Scientific reports |
  | DOI | 10.1038/s41598-024-76831-x |
  | Issue | 1 |
  | Journal Abbr | Sci Rep |
  | ISSN | 2045-2322 |
  | Date Added | 11/07/2025, 12:10:39 |
  | Modified | 11/07/2025, 12:10:39 |

  ### Tags:

  - human-animal interaction
  - physiology
  - heart rate
  - dog
  - emotion
  - animal behavior
  - female
  - male
  - human
  - adult
  - crossover procedure
  - animal
  - human-animal bond
  - middle aged

  ### Attachments

  - Full Text (HTML)
- ## Equine behaviour and heart rate in temperament tests with or without rider or handler

  |  |  |
  | --- | --- |
  | Item Type | Journal Article |
  | Author | U. König von Borstel |
  | Author | S. Euent |
  | Author | P. Graf |
  | Author | S. König |
  | Author | M. Gauly |
  | Abstract | The aim of the present study was to compare horses' heart rate (HR), heart rate variability (RMSSD, pNN50) and behaviour in the same temperament test when being ridden, led, and released free. Behavioural measurements included scores and linear measurements for reactivity (R), activity (A), time to calm down (T) and emotionality (E), recorded during the approach (1) and/or during confrontation with the stimulus (2). Sixty-five horses were each confronted 3 times (1 ridden, 1 led, 1 free running in balanced order) with 3 novel and/or sudden stimuli. Mixed model analysis indicated that leading resulted in the lowest (P < 0.05 throughout) reactions as measured by A1, A2, E1, E2, R2, and pNN50 while riding produced the strongest (A1, T2, HR, RMSSD, pNN50) or medium (E1, E2, R2) reactions. Free running resulted either in the strongest (A2, E1, E2, R2) or in the lowest (A1, T2, HR, RMSSD, pNN50) reactions. The repeatability across tests for HR (0.57), but not for RMSSD (0.23) or pNN50 (0.25) was higher than for any behavioural measurement: the latter ranged from values below 0.10 (A1, A2, T2) to values between 0.30 and 0.45 (E1, E2, R2). Overall, the results show that a rider or handler influences, but not completely masks, the horses' intrinsic behaviour in a temperament test, and this influence appeared to be stronger on behavioural variables and heart rate variability than on the horses' heart rates. Taking both practical considerations and repeatabilities into account, reactivity appears to be the most valuable parameter. Emotionality and heart rate can also yield valid results reflecting additional dimensions of temperament although their practical relevance may be less obvious. If a combination of observed variables is chosen with care, a valid assessment of a horse's temperament may be possible in all types of tests. However, in practice, tests that resemble the practical circumstances most closely, i.e. testing riding horses under a rider, should be chosen. © 2011 Elsevier B.V. |
  | Date | 2011 |
  | Language | English |
  | Archive | Embase |
  | URL | https://www.embase.com/search/results?subaction=viewrecord&id=L51448492&from=export |
  | Volume | 104 |
  | Pages | 454-463 |
  | Publication | Physiology and Behavior |
  | DOI | 10.1016/j.physbeh.2011.05.010 |
  | Issue | 3 |
  | Journal Abbr | Physiol. Behav. |
  | ISSN | 0031-9384 |
  | Date Added | 11/07/2025, 12:10:58 |
  | Modified | 11/07/2025, 12:10:58 |

  ### Tags:

  - horse
  - heart rate variability
  - animal behavior
  - animal experiment
  - article
  - female
  - male
  - nonhuman
  - horseback riding
  - exercise
  - tactile stimulation
  - auditory stimulation
  - visual stimulation
  - priority journal
  - emotionality

  ### Attachments

  - Full Text (HTML)
- ## Stress levels in dogs, and its recognition by their handlers, during animal-assisted therapy in a prison

  |  |  |
  | --- | --- |
  | Item Type | Journal Article |
  | Author | N. Koda |
  | Author | G. Watanabe |
  | Author | Y. Miyaji |
  | Author | A. Ishida |
  | Author | C. Miyaji |
  | Abstract | The stress on dogs and their handlers during animal-assisted therapy in a prison programme was evaluated using questionnaires and measurement of the dogs' saliva cortisol concentrations before and after the sessions. Their handlers were volunteers who underwent training classes with their pet dogs. Overall, the dogs did not show serious signs of stress in the programme, which was also the impression of their handlers. In most cases, the dogs' saliva cortisol values decreased following their participation in the sessions. There was an association between the dogs' stress levels and the handlers' self-reported stress. In 11% of cases, the dogs were evaluated as stressed during the session, but their saliva cortisol values did not change significantly from before to after the session. Some handlers might have misconstrued their dogs' behavioural states. Improvement of the dogs' welfare may be achievable through giving feedback to the handlers to more accurately evaluate their dogs' behaviours, by strengthening the selection of appropriate units and classes prior the programme, by developing a programme and handling methods less burdensome to the animals, and by enhancing the aftercare of animals when they are stressed in a session. Achievement of these goals would also strengthen the bonds between dogs and handlers, contributing to a more effective programme for clients. |
  | Date | 2015 |
  | Language | English |
  | Archive | Embase |
  | URL | https://www.embase.com/search/results?subaction=viewrecord&id=L604234038&from=export |
  | Volume | 24 |
  | Pages | 203-209 |
  | Publication | Animal Welfare |
  | DOI | 10.7120/09627286.24.2.203 |
  | Issue | 2 |
  | Journal Abbr | Anim. Welf. |
  | ISSN | 0962-7286 |
  | Date Added | 11/07/2025, 14:18:40 |
  | Modified | 11/07/2025, 14:18:40 |

  ### Tags:

  - animal welfare
  - dog
  - animal behavior
  - article
  - female
  - male
  - nonhuman
  - physiological stress
  - human
  - hydrocortisone
  - adult
  - golden retriever
  - pet animal
  - human-animal bond
  - animal assisted therapy
  - Labrador retriever
  - saliva analysis
  - mongrel dog
  - beagle
  - correctional facility
  - greyhound
  - prisoner

  ### Attachments

  - Full Text (HTML)
- ## Social bonding between humans, animals, and robots: Dogs outperform AIBOs, their robotic replicas, as social companions

  |  |  |
  | --- | --- |
  | Item Type | Journal Article |
  | Author | S. Klumpe |
  | Author | K.C. Mitchell |
  | Author | E. Cox |
  | Author | J.S. Katz |
  | Author | L. Lazarowski |
  | Author | G. Deshpande |
  | Author | J. Gratch |
  | Author | E.J. de Visser |
  | Author | H. Ayaz |
  | Author | X. Li |
  | Author | A.A. Franke |
  | Author | F. Krueger |
  | Abstract | In the evolving landscape of technology, robots have emerged as social companions, prompting an investigation into social bonding between humans and robots. While human-animal interactions are well-studied, human-robot interactions (HRI) remain comparatively underexplored. Ethorobotics, a field of social robotic engineering based on ecology and ethology, suggests designing companion robots modeled on animal companions, which are simpler to emulate than humans. However, it is unclear whether these robots can match the social companionship provided by their original models. This study examined social bonding between humans and AIBOs, dog-inspired companion robots, compared to real dogs. Nineteen female participants engaged in 12 affiliative interactions with dogs and AIBOs across two counterbalanced, one-month bonding phases. Social bonding was assessed through urinary oxytocin (OXT) level change over an interaction, self-reported attachment using an adapted version of the Lexington Attachment to Pets Scale, and social companionship evaluations administering the Robot-Dog Questionnaire. To examine OXT level changes and self-reported attachment by comparing the two social companions, we conducted mixed-effects model analyses and planned follow-up comparisons. Frequency comparison, binary logistic regression, and thematic analysis were performed to analyze social companionship evaluations. Results revealed significant differences between dogs and AIBOs in fostering social bonds. OXT level change increased during interactions with dogs but decreased with AIBOs. Participants reported stronger attachment to dogs and rated them as better social companions. These findings highlight the current limitations of AIBOs in fostering social bonding immediately compared to dogs. Our study contributes to the growing HRI research by demonstrating an existing gap between AIBOs and dogs as social companions. It highlights the need for further investigation to understand the complexities of social bonding with companion robots, which is essential to implement successful applications for social robots in diverse domains such as the elderly and health care, education, and entertainment. |
  | Date | 2025 |
  | Language | English |
  | Archive | Embase |
  | URL | https://www.embase.com/search/results?subaction=viewrecord&id=L2039000813&from=export |
  | Volume | 20 |
  | Publication | PLOS ONE |
  | DOI | 10.1371/journal.pone.0324312 |
  | Issue | 6 June |
  | Journal Abbr | Plos One. |
  | ISSN | 1932-6203 |
  | Date Added | 11/07/2025, 12:12:13 |
  | Modified | 11/07/2025, 12:12:13 |

  ### Tags:

  - human-animal interaction
  - cognition
  - article
  - female
  - nonhuman
  - human
  - data analysis software
  - ELISA kit
  - follow up
  - questionnaire
  - social bonding
  - chromatin accessibility
  - functional magnetic resonance imaging
  - informed consent
  - landscape
  - Likert scale
  - logistic regression analysis
  - neurosis
  - room temperature
  - self-reported questionnaire
  - SPSS Version 23
  - thematic analysis

  ### Attachments

  - Full Text (HTML)
- ## Social bonding between humans, animals, and robots: Dogs outperform AIBOs, their robotic replicas, as social companions

  |  |  |
  | --- | --- |
  | Item Type | Journal Article |
  | Author | S. Klumpe |
  | Author | K.C. Mitchell |
  | Author | E. Cox |
  | Author | J.S. Katz |
  | Author | L. Lazarowski |
  | Author | G. Deshpande |
  | Author | J. Gratch |
  | Author | E.J. de Visser |
  | Author | H. Ayaz |
  | Author | X. Li |
  | Author | A.A. Franke |
  | Author | F. Krueger |
  | Abstract | In the evolving landscape of technology, robots have emerged as social companions, prompting an investigation into social bonding between humans and robots. While human-animal interactions are well-studied, human-robot interactions (HRI) remain comparatively underexplored. Ethorobotics, a field of social robotic engineering based on ecology and ethology, suggests designing companion robots modeled on animal companions, which are simpler to emulate than humans. However, it is unclear whether these robots can match the social companionship provided by their original models. This study examined social bonding between humans and AIBOs, dog-inspired companion robots, compared to real dogs. Nineteen female participants engaged in 12 affiliative interactions with dogs and AIBOs across two counterbalanced, one-month bonding phases. Social bonding was assessed through urinary oxytocin (OXT) level change over an interaction, self-reported attachment using an adapted version of the Lexington Attachment to Pets Scale, and social companionship evaluations administering the Robot-Dog Questionnaire. To examine OXT level changes and self-reported attachment by comparing the two social companions, we conducted mixed-effects model analyses and planned follow-up comparisons. Frequency comparison, binary logistic regression, and thematic analysis were performed to analyze social companionship evaluations. Results revealed significant differences between dogs and AIBOs in fostering social bonds. OXT level change increased during interactions with dogs but decreased with AIBOs. Participants reported stronger attachment to dogs and rated them as better social companions. These findings highlight the current limitations of AIBOs in fostering social bonding immediately compared to dogs. Our study contributes to the growing HRI research by demonstrating an existing gap between AIBOs and dogs as social companions. It highlights the need for further investigation to understand the complexities of social bonding with companion robots, which is essential to implement successful applications for social robots in diverse domains such as the elderly and health care, education, and entertainment. |
  | Date | 2025 |
  | Language | English |
  | Archive | Embase |
  | URL | https://www.embase.com/search/results?subaction=viewrecord&id=L2039000813&from=export |
  | Volume | 20 |
  | Publication | PLOS ONE |
  | DOI | 10.1371/journal.pone.0324312 |
  | Issue | 6 June |
  | Journal Abbr | Plos One. |
  | ISSN | 1932-6203 |
  | Date Added | 11/07/2025, 12:13:14 |
  | Modified | 11/07/2025, 12:13:14 |

  ### Tags:

  - human-animal interaction
  - cognition
  - article
  - female
  - nonhuman
  - human
  - data analysis software
  - ELISA kit
  - follow up
  - questionnaire
  - social bonding
  - chromatin accessibility
  - functional magnetic resonance imaging
  - informed consent
  - landscape
  - Likert scale
  - logistic regression analysis
  - neurosis
  - room temperature
  - self-reported questionnaire
  - SPSS Version 23
  - thematic analysis

  ### Attachments

  - Full Text (HTML)
- ## Soybean oil supplement induces increased approaching behavior to humans and alters serotonin concentrations in horses

  |  |  |
  | --- | --- |
  | Item Type | Journal Article |
  | Author | S. Kim |
  | Author | Y. Choi |
  | Author | J. Kim |
  | Author | C.L. Wickens |
  | Author | M. Yoon |
  | Abstract | Enhancement of human-horse interaction is crucial for safety in equine management, as poor relationships between humans and horses can lead to accidents. Serotonin is a neurotransmitter that is highly related to social affinity in animals and several studies have been documented that supplementation of tryptophan, which is a precursor of serotonin, can increase calmness of horses. This study aimed to assess the effect of tryptophan through soybean oil supplementation on serotonin concentrations and the behavior of horses. Six horses participated and the horses were treated with or without soybean oil diets in a cross-over design. Daily feed intake was 2 % of the body weight of horses, with a timothy hay to grain ratio of 8:2. Behavioral observations and blood collections were conducted and serotonin and cortisol concentrations were quantified using enzyme-linked immunosorbent assays. Data was analyzed using two-way repeated measures analysis of variance to explore the effects of diet, time, and their interaction. The difference was considered statistically significant when p-value was less than 0.05. The results demonstrated that soybean oil supplementation significantly increased approaching behaviors towards humans (5.57 ± 1.12) compared to baseline scores (1.2 ± 0.57). Moreover, serotonin concentrations were elevated following the soybean oil diet (150.09 ± 21.32 ng/mL) compared to concentrations on day 1 (115.89 ± 22.3 ng/mL). Cortisol concentrations remained stable throughout the experimental period when horses were treated with the soybean oil diet (405.29 ± 35.29 ng/mL vs. 427.64 ± 87.34 ng/mL). In contrast, cortisol concentrations increased significantly in horses on the non-oil diet (405.29 ± 35.29 ng/mL vs. 427.64 ± 87.34 ng/mL). These findings suggest that soybean oil supplementation positively influences horse behavior towards humans, indicating that dietary modification may enhance human-horse interactions. |
  | Date | 2025 |
  | Language | English |
  | Archive | Embase |
  | URL | https://www.embase.com/search/results?subaction=viewrecord&id=L2037171194&from=export |
  | Volume | 146 |
  | Publication | Journal of Equine Veterinary Science |
  | DOI | 10.1016/j.jevs.2025.105361 |
  | Issue | (Kim S.; Choi Y.; Kim J.) Department of Animal Science and Biotechnology, The Graduate school, Kyungpook National University, Buksangju-ro 2559, Sangju, Gyeongsangbuk-do, South Korea |
  | Journal Abbr | J. Equine Vet. Sci. |
  | ISSN | 0737-0806 |
  | Date Added | 11/07/2025, 14:15:26 |
  | Modified | 11/07/2025, 14:15:26 |

  ### Tags:

  - human-animal interaction
  - horse
  - animal behavior
  - animal experiment
  - article
  - female
  - male
  - nonhuman
  - human
  - hydrocortisone
  - controlled study
  - crossover procedure
  - neurotransmitter
  - behavioral observation
  - enzyme linked immunosorbent assay
  - biological marker
  - quantitative analysis
  - hydrocortisone blood level
  - serotonin
  - serotonin blood level
  - analysis of variance
  - concentration response
  - diet supplementation
  - dietary intake
  - fat intake
  - soybean oil
  - statistical significance
  - tryptophan

  ### Attachments

  - Full Text (HTML)
- ## Soybean oil supplement induces increased approaching behavior to humans and alters serotonin concentrations in horses

  |  |  |
  | --- | --- |
  | Item Type | Journal Article |
  | Author | S. Kim |
  | Author | Y. Choi |
  | Author | J. Kim |
  | Author | C.L. Wickens |
  | Author | M. Yoon |
  | Abstract | Enhancement of human-horse interaction is crucial for safety in equine management, as poor relationships between humans and horses can lead to accidents. Serotonin is a neurotransmitter that is highly related to social affinity in animals and several studies have been documented that supplementation of tryptophan, which is a precursor of serotonin, can increase calmness of horses. This study aimed to assess the effect of tryptophan through soybean oil supplementation on serotonin concentrations and the behavior of horses. Six horses participated and the horses were treated with or without soybean oil diets in a cross-over design. Daily feed intake was 2 % of the body weight of horses, with a timothy hay to grain ratio of 8:2. Behavioral observations and blood collections were conducted and serotonin and cortisol concentrations were quantified using enzyme-linked immunosorbent assays. Data was analyzed using two-way repeated measures analysis of variance to explore the effects of diet, time, and their interaction. The difference was considered statistically significant when p-value was less than 0.05. The results demonstrated that soybean oil supplementation significantly increased approaching behaviors towards humans (5.57 ± 1.12) compared to baseline scores (1.2 ± 0.57). Moreover, serotonin concentrations were elevated following the soybean oil diet (150.09 ± 21.32 ng/mL) compared to concentrations on day 1 (115.89 ± 22.3 ng/mL). Cortisol concentrations remained stable throughout the experimental period when horses were treated with the soybean oil diet (405.29 ± 35.29 ng/mL vs. 427.64 ± 87.34 ng/mL). In contrast, cortisol concentrations increased significantly in horses on the non-oil diet (405.29 ± 35.29 ng/mL vs. 427.64 ± 87.34 ng/mL). These findings suggest that soybean oil supplementation positively influences horse behavior towards humans, indicating that dietary modification may enhance human-horse interactions. |
  | Date | 2025 |
  | Language | English |
  | Archive | Embase |
  | URL | https://www.embase.com/search/results?subaction=viewrecord&id=L2037171194&from=export |
  | Volume | 146 |
  | Publication | Journal of Equine Veterinary Science |
  | DOI | 10.1016/j.jevs.2025.105361 |
  | Issue | (Kim S.; Choi Y.; Kim J.) Department of Animal Science and Biotechnology, The Graduate school, Kyungpook National University, Buksangju-ro 2559, Sangju, Gyeongsangbuk-do, South Korea |
  | Journal Abbr | J. Equine Vet. Sci. |
  | ISSN | 0737-0806 |
  | Date Added | 11/07/2025, 14:16:36 |
  | Modified | 11/07/2025, 14:16:36 |

  ### Tags:

  - human-animal interaction
  - horse
  - animal behavior
  - animal experiment
  - article
  - female
  - male
  - nonhuman
  - human
  - hydrocortisone
  - controlled study
  - crossover procedure
  - neurotransmitter
  - behavioral observation
  - enzyme linked immunosorbent assay
  - biological marker
  - quantitative analysis
  - hydrocortisone blood level
  - serotonin
  - serotonin blood level
  - analysis of variance
  - concentration response
  - diet supplementation
  - dietary intake
  - fat intake
  - soybean oil
  - statistical significance
  - tryptophan

  ### Attachments

  - Full Text (HTML)
- ## Soybean oil supplement induces increased approaching behavior to humans and alters serotonin concentrations in horses

  |  |  |
  | --- | --- |
  | Item Type | Journal Article |
  | Author | S. Kim |
  | Author | Y. Choi |
  | Author | J. Kim |
  | Author | C.L. Wickens |
  | Author | M. Yoon |
  | Abstract | Enhancement of human-horse interaction is crucial for safety in equine management, as poor relationships between humans and horses can lead to accidents. Serotonin is a neurotransmitter that is highly related to social affinity in animals and several studies have been documented that supplementation of tryptophan, which is a precursor of serotonin, can increase calmness of horses. This study aimed to assess the effect of tryptophan through soybean oil supplementation on serotonin concentrations and the behavior of horses. Six horses participated and the horses were treated with or without soybean oil diets in a cross-over design. Daily feed intake was 2 % of the body weight of horses, with a timothy hay to grain ratio of 8:2. Behavioral observations and blood collections were conducted and serotonin and cortisol concentrations were quantified using enzyme-linked immunosorbent assays. Data was analyzed using two-way repeated measures analysis of variance to explore the effects of diet, time, and their interaction. The difference was considered statistically significant when p-value was less than 0.05. The results demonstrated that soybean oil supplementation significantly increased approaching behaviors towards humans (5.57 ± 1.12) compared to baseline scores (1.2 ± 0.57). Moreover, serotonin concentrations were elevated following the soybean oil diet (150.09 ± 21.32 ng/mL) compared to concentrations on day 1 (115.89 ± 22.3 ng/mL). Cortisol concentrations remained stable throughout the experimental period when horses were treated with the soybean oil diet (405.29 ± 35.29 ng/mL vs. 427.64 ± 87.34 ng/mL). In contrast, cortisol concentrations increased significantly in horses on the non-oil diet (405.29 ± 35.29 ng/mL vs. 427.64 ± 87.34 ng/mL). These findings suggest that soybean oil supplementation positively influences horse behavior towards humans, indicating that dietary modification may enhance human-horse interactions. |
  | Date | 2025 |
  | Language | English |
  | Archive | Embase |
  | URL | https://www.embase.com/search/results?subaction=viewrecord&id=L2037171194&from=export |
  | Volume | 146 |
  | Publication | Journal of Equine Veterinary Science |
  | DOI | 10.1016/j.jevs.2025.105361 |
  | Issue | (Kim S.; Choi Y.; Kim J.) Department of Animal Science and Biotechnology, The Graduate school, Kyungpook National University, Buksangju-ro 2559, Sangju, Gyeongsangbuk-do, South Korea |
  | Journal Abbr | J. Equine Vet. Sci. |
  | ISSN | 0737-0806 |
  | Date Added | 11/07/2025, 14:18:09 |
  | Modified | 11/07/2025, 14:18:09 |

  ### Tags:

  - human-animal interaction
  - horse
  - animal behavior
  - animal experiment
  - article
  - female
  - male
  - nonhuman
  - human
  - hydrocortisone
  - controlled study
  - crossover procedure
  - neurotransmitter
  - behavioral observation
  - enzyme linked immunosorbent assay
  - biological marker
  - quantitative analysis
  - hydrocortisone blood level
  - serotonin
  - serotonin blood level
  - analysis of variance
  - concentration response
  - diet supplementation
  - dietary intake
  - fat intake
  - soybean oil
  - statistical significance
  - tryptophan

  ### Attachments

  - Full Text (HTML)
- ## The effect of serotonin and oxytocin on equine docility and friendliness to humans

  |  |  |
  | --- | --- |
  | Item Type | Journal Article |
  | Author | J. Kim |
  | Author | M. Yoon |
  | Abstract | Neurotransmitters play important roles in animal behaviors. Serotonin (5-HT) and oxytocin (OXT) are well-known neurotransmitters affecting animals' social behavior. Recently, we demonstrated the effect of OXT on horse docility and friendliness to humans. However, the correlation of 5-HT and OXT with horse docility and friendliness to humans remains to be elucidated. This study aims to (1) evaluate the effect of breed, age, and sex on 5-HT and OXT concentrations in horses, (2) examine the correlation between 5-HT and OXT, and (3) to test the effect of 5-HT on the docility and friendliness toward humans. Blood samples were collected from 29 horses at the Horse Industry Complex Center of Jeonju Kijeon College. The horse breeds comprised Thoroughbreds, ponies, Warmbloods, and Haflingers. There were 20 mares and 9 geldings. Based on age, the horses were grouped into postpubertal, adult, and aged horses. The plasma concentration of 5-HT or OXT was measured by ELISA. The temperament of each horse was evaluated by 3 professors who were familiar with all the horses. The mixed model through least significant difference posthoc analysis was conducted to analyze the effect of fixed factors (breed, age, and sex) and interaction factor (breed × age × sex) on random effect (neurotransmitter levels). The Student T-test was performed to analyze the effect of sex (geldings and mares) on 5-HT or OXT concentrations. The linear regression analysis was performed to test the correlation between (1) the plasma concentration of 5-HT and OXT and (2) the plasma concentration of 5-HT or OXT with the score of equine docility and friendliness toward humans. The 5-HT concentration of Warmbloods and OXT concentration of Warmbloods and Thoroughbreds were significantly higher than those of ponies. The concentration of OXT in the aged horse was considerably higher than that of postpubertal horses. Interestingly, there is a positive correlation between the concentrations of plasma 5-HT and OXT. Also, 5-HT showed a positive correlation with horses’ docility and friendliness toward humans. In conclusion, the plasma concentration of 5-HT in combination with OXT can be used as a parameter to identify a horse's docility and friendliness to humans. |
  | Date | 2022 |
  | Language | English |
  | Archive | Embase |
  | URL | https://www.embase.com/search/results?subaction=viewrecord&id=L2017087568&from=export |
  | Volume | 50 |
  | Pages | 18-22 |
  | Publication | Journal of Veterinary Behavior |
  | DOI | 10.1016/j.jveb.2022.01.004 |
  | Issue | (Kim J.; Yoon M., mjyoonemail@gmail.com) Department of Animal Science and Biotechnology, Kyungpook National University, Sangju, South Korea |
  | Journal Abbr | J. Vet. Behav. |
  | ISSN | 1558-7878 |
  | Date Added | 11/07/2025, 12:12:22 |
  | Modified | 11/07/2025, 12:12:22 |

  ### Tags:

  - oxytocin
  - human-animal interaction
  - animal behavior
  - article
  - female
  - male
  - nonhuman
  - human
  - adult
  - blood sampling
  - controlled study
  - neurotransmitter
  - enzyme linked immunosorbent assay
  - Equus
  - oxytocin blood level
  - serotonin
  - breeding
  - concentration (parameter)
  - gelding
  - groups by age
  - haflinger
  - horse breed
  - mare
  - ponies
  - serotonin blood level
  - sex difference
  - thoroughbred
  - warmblood

  ### Attachments

  - Full Text (HTML)
- ## The effect of serotonin and oxytocin on equine docility and friendliness to humans

  |  |  |
  | --- | --- |
  | Item Type | Journal Article |
  | Author | J. Kim |
  | Author | M. Yoon |
  | Abstract | Neurotransmitters play important roles in animal behaviors. Serotonin (5-HT) and oxytocin (OXT) are well-known neurotransmitters affecting animals' social behavior. Recently, we demonstrated the effect of OXT on horse docility and friendliness to humans. However, the correlation of 5-HT and OXT with horse docility and friendliness to humans remains to be elucidated. This study aims to (1) evaluate the effect of breed, age, and sex on 5-HT and OXT concentrations in horses, (2) examine the correlation between 5-HT and OXT, and (3) to test the effect of 5-HT on the docility and friendliness toward humans. Blood samples were collected from 29 horses at the Horse Industry Complex Center of Jeonju Kijeon College. The horse breeds comprised Thoroughbreds, ponies, Warmbloods, and Haflingers. There were 20 mares and 9 geldings. Based on age, the horses were grouped into postpubertal, adult, and aged horses. The plasma concentration of 5-HT or OXT was measured by ELISA. The temperament of each horse was evaluated by 3 professors who were familiar with all the horses. The mixed model through least significant difference posthoc analysis was conducted to analyze the effect of fixed factors (breed, age, and sex) and interaction factor (breed × age × sex) on random effect (neurotransmitter levels). The Student T-test was performed to analyze the effect of sex (geldings and mares) on 5-HT or OXT concentrations. The linear regression analysis was performed to test the correlation between (1) the plasma concentration of 5-HT and OXT and (2) the plasma concentration of 5-HT or OXT with the score of equine docility and friendliness toward humans. The 5-HT concentration of Warmbloods and OXT concentration of Warmbloods and Thoroughbreds were significantly higher than those of ponies. The concentration of OXT in the aged horse was considerably higher than that of postpubertal horses. Interestingly, there is a positive correlation between the concentrations of plasma 5-HT and OXT. Also, 5-HT showed a positive correlation with horses’ docility and friendliness toward humans. In conclusion, the plasma concentration of 5-HT in combination with OXT can be used as a parameter to identify a horse's docility and friendliness to humans. |
  | Date | 2022 |
  | Language | English |
  | Archive | Embase |
  | URL | https://www.embase.com/search/results?subaction=viewrecord&id=L2017087568&from=export |
  | Volume | 50 |
  | Pages | 18-22 |
  | Publication | Journal of Veterinary Behavior |
  | DOI | 10.1016/j.jveb.2022.01.004 |
  | Issue | (Kim J.; Yoon M., mjyoonemail@gmail.com) Department of Animal Science and Biotechnology, Kyungpook National University, Sangju, South Korea |
  | Journal Abbr | J. Vet. Behav. |
  | ISSN | 1558-7878 |
  | Date Added | 11/07/2025, 12:13:24 |
  | Modified | 11/07/2025, 12:13:24 |

  ### Tags:

  - oxytocin
  - human-animal interaction
  - animal behavior
  - article
  - female
  - male
  - nonhuman
  - human
  - adult
  - blood sampling
  - controlled study
  - neurotransmitter
  - enzyme linked immunosorbent assay
  - Equus
  - oxytocin blood level
  - serotonin
  - breeding
  - concentration (parameter)
  - gelding
  - groups by age
  - haflinger
  - horse breed
  - mare
  - ponies
  - serotonin blood level
  - sex difference
  - thoroughbred
  - warmblood

  ### Attachments

  - Full Text (HTML)
- ## Flight distance at pasture and stress response during shearing in alpacas

  |  |  |
  | --- | --- |
  | Item Type | Journal Article |
  | Author | J. Kapustka |
  | Author | M. Budzyńska |
  | Author | O. Witkowska – Piłaszewicz |
  | Author | J. Fabjanowska |
  | Abstract | Potential influence of management and handling procedures on alpacas’ behavioral and physiological responses has received little research attention so far and these are the first combined observations of alpacas' behavior with physiological stress assessment in Poland. Human influence on a farm animal can be measured by direct contact in neutral conditions and by an animal's reactions during husbandry procedures. Quantifying the strength of the flight response toward approaching persons can help assess an individual's suitability for use in various human–animal contact activities. Shearing is one of the potentially stressful husbandry procedures that can affect alpacas’ behavior and welfare. This study aimed to assess flight distance on the pasture and behavioral and physiological reactions during shearing in 34 alpacas. Two related questions have been raised: (1) do reactions to humans affect reactions to the shearing procedure? and (2) is shearing a stressful practice to alpacas based on both salivary cortisol and stress-related behavior? The flight distance toward humans (familiar and unfamiliar person) was measured. The stress reaction during shearing was assessed by behavioral observation and saliva sampling (to determine cortisol level). The animals that had a short flight distance to a familiar person also had a shorter flight distance to an unfamiliar one (P < 0.001). This study did not show that shorter flight distance to humans was connected with less intensive stress response during shearing. There was a difference (P < 0.001) between salivary cortisol levels before and after shearing, indicating that shearing is stressful for alpacas. It was observed that the later in order the animal was shorn, the fewer attempts to break free through abrupt movements were shown (P < 0.05). It was also noticed that the number of agonistic behaviors reduced as the shearing time increased (P < 0.05). Alpacas can adapt to stressful situations, and the animals most sensitive to stress should be shorn last. |
  | Date | 2023 |
  | Language | English |
  | Archive | Embase |
  | URL | https://www.embase.com/search/results?subaction=viewrecord&id=L2023860912&from=export |
  | Volume | 63 |
  | Pages | 1-9 |
  | Publication | Journal of Veterinary Behavior |
  | DOI | 10.1016/j.jveb.2023.03.002 |
  | Issue | (Kapustka J., joanna.kapustka@up.lublin.pl; Budzyńska M., monika.budzynska@up.lublin.pl) Department of Animal Ethology and Wildlife Management, University of Life Sciences in Lublin, Lublin, Poland |
  | Journal Abbr | J. Vet. Behav. |
  | ISSN | 1558-7878 |
  | Date Added | 11/07/2025, 14:15:34 |
  | Modified | 11/07/2025, 14:15:34 |

  ### Tags:

  - human-animal interaction
  - animal behavior
  - article
  - female
  - male
  - nonhuman
  - physiological stress
  - hydrocortisone
  - controlled study
  - pasture
  - animal husbandry
  - saliva level
  - agonistic behavior
  - alpaca
  - flight
  - flight distance
  - shearing

  ### Attachments

  - Full Text (HTML)
- ## Flight distance at pasture and stress response during shearing in alpacas

  |  |  |
  | --- | --- |
  | Item Type | Journal Article |
  | Author | J. Kapustka |
  | Author | M. Budzyńska |
  | Author | O. Witkowska – Piłaszewicz |
  | Author | J. Fabjanowska |
  | Abstract | Potential influence of management and handling procedures on alpacas’ behavioral and physiological responses has received little research attention so far and these are the first combined observations of alpacas' behavior with physiological stress assessment in Poland. Human influence on a farm animal can be measured by direct contact in neutral conditions and by an animal's reactions during husbandry procedures. Quantifying the strength of the flight response toward approaching persons can help assess an individual's suitability for use in various human–animal contact activities. Shearing is one of the potentially stressful husbandry procedures that can affect alpacas’ behavior and welfare. This study aimed to assess flight distance on the pasture and behavioral and physiological reactions during shearing in 34 alpacas. Two related questions have been raised: (1) do reactions to humans affect reactions to the shearing procedure? and (2) is shearing a stressful practice to alpacas based on both salivary cortisol and stress-related behavior? The flight distance toward humans (familiar and unfamiliar person) was measured. The stress reaction during shearing was assessed by behavioral observation and saliva sampling (to determine cortisol level). The animals that had a short flight distance to a familiar person also had a shorter flight distance to an unfamiliar one (P < 0.001). This study did not show that shorter flight distance to humans was connected with less intensive stress response during shearing. There was a difference (P < 0.001) between salivary cortisol levels before and after shearing, indicating that shearing is stressful for alpacas. It was observed that the later in order the animal was shorn, the fewer attempts to break free through abrupt movements were shown (P < 0.05). It was also noticed that the number of agonistic behaviors reduced as the shearing time increased (P < 0.05). Alpacas can adapt to stressful situations, and the animals most sensitive to stress should be shorn last. |
  | Date | 2023 |
  | Language | English |
  | Archive | Embase |
  | URL | https://www.embase.com/search/results?subaction=viewrecord&id=L2023860912&from=export |
  | Volume | 63 |
  | Pages | 1-9 |
  | Publication | Journal of Veterinary Behavior |
  | DOI | 10.1016/j.jveb.2023.03.002 |
  | Issue | (Kapustka J., joanna.kapustka@up.lublin.pl; Budzyńska M., monika.budzynska@up.lublin.pl) Department of Animal Ethology and Wildlife Management, University of Life Sciences in Lublin, Lublin, Poland |
  | Journal Abbr | J. Vet. Behav. |
  | ISSN | 1558-7878 |
  | Date Added | 11/07/2025, 14:16:44 |
  | Modified | 11/07/2025, 14:16:44 |

  ### Tags:

  - human-animal interaction
  - animal behavior
  - article
  - female
  - male
  - nonhuman
  - physiological stress
  - hydrocortisone
  - controlled study
  - pasture
  - animal husbandry
  - saliva level
  - agonistic behavior
  - alpaca
  - flight
  - flight distance
  - shearing

  ### Attachments

  - Full Text (HTML)
- ## Flight distance at pasture and stress response during shearing in alpacas

  |  |  |
  | --- | --- |
  | Item Type | Journal Article |
  | Author | J. Kapustka |
  | Author | M. Budzyńska |
  | Author | O. Witkowska – Piłaszewicz |
  | Author | J. Fabjanowska |
  | Abstract | Potential influence of management and handling procedures on alpacas’ behavioral and physiological responses has received little research attention so far and these are the first combined observations of alpacas' behavior with physiological stress assessment in Poland. Human influence on a farm animal can be measured by direct contact in neutral conditions and by an animal's reactions during husbandry procedures. Quantifying the strength of the flight response toward approaching persons can help assess an individual's suitability for use in various human–animal contact activities. Shearing is one of the potentially stressful husbandry procedures that can affect alpacas’ behavior and welfare. This study aimed to assess flight distance on the pasture and behavioral and physiological reactions during shearing in 34 alpacas. Two related questions have been raised: (1) do reactions to humans affect reactions to the shearing procedure? and (2) is shearing a stressful practice to alpacas based on both salivary cortisol and stress-related behavior? The flight distance toward humans (familiar and unfamiliar person) was measured. The stress reaction during shearing was assessed by behavioral observation and saliva sampling (to determine cortisol level). The animals that had a short flight distance to a familiar person also had a shorter flight distance to an unfamiliar one (P < 0.001). This study did not show that shorter flight distance to humans was connected with less intensive stress response during shearing. There was a difference (P < 0.001) between salivary cortisol levels before and after shearing, indicating that shearing is stressful for alpacas. It was observed that the later in order the animal was shorn, the fewer attempts to break free through abrupt movements were shown (P < 0.05). It was also noticed that the number of agonistic behaviors reduced as the shearing time increased (P < 0.05). Alpacas can adapt to stressful situations, and the animals most sensitive to stress should be shorn last. |
  | Date | 2023 |
  | Language | English |
  | Archive | Embase |
  | URL | https://www.embase.com/search/results?subaction=viewrecord&id=L2023860912&from=export |
  | Volume | 63 |
  | Pages | 1-9 |
  | Publication | Journal of Veterinary Behavior |
  | DOI | 10.1016/j.jveb.2023.03.002 |
  | Issue | (Kapustka J., joanna.kapustka@up.lublin.pl; Budzyńska M., monika.budzynska@up.lublin.pl) Department of Animal Ethology and Wildlife Management, University of Life Sciences in Lublin, Lublin, Poland |
  | Journal Abbr | J. Vet. Behav. |
  | ISSN | 1558-7878 |
  | Date Added | 11/07/2025, 14:18:16 |
  | Modified | 11/07/2025, 14:18:16 |

  ### Tags:

  - human-animal interaction
  - animal behavior
  - article
  - female
  - male
  - nonhuman
  - physiological stress
  - hydrocortisone
  - controlled study
  - pasture
  - animal husbandry
  - saliva level
  - agonistic behavior
  - alpaca
  - flight
  - flight distance
  - shearing

  ### Attachments

  - Full Text (HTML)
- ## Positive Reinforcement Training for Blood Collection in Grizzly Bears (Ursus arctos horribilis) Results in Undetectable Elevations in Serum Cortisol Levels: A Preliminary Investigation

  |  |  |
  | --- | --- |
  | Item Type | Journal Article |
  | Author | N.M. Joyce-Zuniga |
  | Author | R.C. Newberry |
  | Author | C.T. Robbins |
  | Author | J.V. Ware |
  | Author | H.T. Jansen |
  | Author | O.L. Nelson |
  | Abstract | Training nonhuman animals in captivity for participation in routine husbandry procedures is believed to produce a lower stress environment compared with undergoing a general anesthetic event for the same procedure. This hypothesis rests largely on anecdotal evidence that the captive subjects appear more relaxed with the trained event. Blood markers of physiological stress responses were evaluated in 4 captive grizzly bears (Ursus arctos horribilis) who were clicker-trained for blood collection versus 4 bears who were chemically immobilized for blood collection. Serum cortisol and immunoglobulin A (IgA) and plasma β-endorphin were measured as indicators of responses to stress. Plasma β-endorphin was not different between the groups. Serum IgA was undetectable in all bears. Serum cortisol was undetectable in all trained bears, whereas chemically immobilized bears had marked cortisol elevations (p < .05). The highest cortisol elevations were found in 2 bears with extensive recent immobilization experience. These findings support the use of positive reinforcement training for routine health procedures to minimize anxiety. |
  | Date | 2016 |
  | Language | English |
  | Archive | Embase |
  | URL | https://www.embase.com/search/results?subaction=viewrecord&id=L608511688&from=export |
  | Volume | 19 |
  | Pages | 210-215 |
  | Publication | Journal of Applied Animal Welfare Science |
  | DOI | 10.1080/10888705.2015.1126523 |
  | Issue | 2 |
  | Journal Abbr | J. Appl. Anim. Welf. Sci. |
  | ISSN | 1532-7604 |
  | Date Added | 11/07/2025, 14:18:38 |
  | Modified | 11/07/2025, 14:18:38 |

  ### Tags:

  - animal behavior
  - animal experiment
  - article
  - female
  - male
  - nonhuman
  - physiological stress
  - hydrocortisone
  - adult
  - blood sampling
  - vein puncture
  - enzyme linked immunosorbent assay
  - training
  - beta endorphin
  - immunoglobulin A
  - hydrocortisone blood level
  - radioimmunoassay
  - reinforcement (psychology)
  - bear
  - immunodiffusion

  ### Attachments

  - Full Text (HTML)
- ## Interaction with Caged Budgerigars (Melopsittacus Undulatus) Enhances Human Affect

  |  |  |
  | --- | --- |
  | Item Type | Journal Article |
  | Author | A.G. Jones |
  | Author | A.J. Skolnick |
  | Author | M.J. Anderson |
  | Abstract | Research interest in the benefits of human–animal interaction (HAI) has grown within the scientific community. One current limitation in the HAI literature however, is that the majority of research focuses on the beneficial effects of dogs and cats, paying less attention to other species commonly kept as pets, such as birds. In the present study, 88 participants were asked to read aloud to a live bird, a toy bird with sound and motion, a toy bird without sound and motion, or an empty cage (n = 22 for each group). Affect scores, heart rate and blood pressure were taken before and after the session and scores from a questionnaire regarding pet ownership and bird experience were collected. Results indicated that interactions with a live bird maintain positive affect while significantly decreasing negative affect. Overall, this study demonstrated a clear affective benefit to interactions with birds, but more research is warranted onto the effects of interaction with under-studied pet animals beyond dogs and cats. |
  | Date | 2021 |
  | Language | English |
  | Archive | Embase |
  | URL | https://www.embase.com/search/results?subaction=viewrecord&id=L2010441530&from=export |
  | Volume | 34 |
  | Pages | 127-138 |
  | Publication | Anthrozoos |
  | DOI | 10.1080/08927936.2021.1874113 |
  | Issue | 1 |
  | Journal Abbr | Anthrozoos |
  | ISSN | 1753-0377 |
  | Date Added | 11/07/2025, 12:10:50 |
  | Modified | 11/07/2025, 12:10:50 |

  ### Tags:

  - blood pressure
  - human-animal interaction
  - heart rate
  - dog
  - article
  - nonhuman
  - human
  - adult
  - controlled study
  - human experiment
  - pet animal
  - questionnaire
  - major clinical study
  - Positive and Negative Affect Schedule
  - budgerigar
  - cat
  - motion
  - sound

  ### Attachments

  - Full Text (HTML)
- ## Exploring women’s oxytocin responses to interactions with their pet cats

  |  |  |
  | --- | --- |
  | Item Type | Journal Article |
  | Author | E.A. Johnson |
  | Author | A. Portillo |
  | Author | N.E. Bennett |
  | Author | P.B. Gray |
  | Abstract | Background. Extensive research has evaluated the involvement of the neuropeptide oxytocin (OT) in human social behaviors, including parent-infant relationships. Studies have investigated OT’s connection to human attachment to nonhuman animals, with the majority of the literature focusing on domestic dogs (Canis lupis familiaris). Utilizing what is known about OT and its role in maternal-infant and human-dog bonding, we apply these frameworks to the study of human-domestic cat (Felis catus) interactions. Methods. We investigated changes in salivary OT levels in 30 U.S. women of reproductive age before and after two conditions: reading a book (control) and interacting with their pet cat. Participant and cat behavioral patterns during the cat interaction condition were also quantified to determine if differences in women’s OT concentrations were associated with specific human and cat behaviors. Results. Our results revealed no changes in women’s OT levels during the cat interaction, relative to the control condition, and pre-cat interaction OT levels. However, differences in women’s OT concentrations were correlated with some human-cat interactions (e.g., positively with petting cat and cat approach initiation, negatively with cat agonistic behavior) but not all observed behaviors (e.g., use of gentle or baby voice) coded during human-cat interactions. Discussion. This study is the first to explore women’s OT in response to interactions with their pet cat and has identified distinct human and cat behaviors that influence OT release in humans. |
  | Date | 2021 |
  | Language | English |
  | Archive | Embase |
  | URL | https://www.embase.com/search/results?subaction=viewrecord&id=L2015610800&from=export |
  | Volume | 9 |
  | Publication | PeerJ |
  | DOI | 10.7717/peerj.12393 |
  | Issue | (Johnson E.A., Elizabeth.johnson@unlv.edu; Portillo A.; Bennett N.E.; Gray P.B.) Department of Anthropology, University of Nevada, Las Vegas, Las Vegas, NV, United States |
  | Journal Abbr | PeerJ |
  | ISSN | 2167-8359 |
  | Date Added | 11/07/2025, 12:12:23 |
  | Modified | 11/07/2025, 12:12:23 |

  ### Tags:

  - oxytocin
  - human-animal interaction
  - animal behavior
  - article
  - female
  - nonhuman
  - human
  - adult
  - controlled study
  - pet animal
  - questionnaire
  - clinical article
  - young adult
  - human-animal bond
  - enzyme linked immunosorbent assay
  - quantitative analysis
  - domestic cat
  - cat
  - gene expression
  - protein expression
  - Likert scale
  - oxytocin release
  - autopsy
  - birth control
  - distress syndrome
  - Expanded Disability Status Scale
  - household
  - neuropeptide
  - protein function

  ### Attachments

  - Full Text (HTML)
- ## Exploring women’s oxytocin responses to interactions with their pet cats

  |  |  |
  | --- | --- |
  | Item Type | Journal Article |
  | Author | E.A. Johnson |
  | Author | A. Portillo |
  | Author | N.E. Bennett |
  | Author | P.B. Gray |
  | Abstract | Background. Extensive research has evaluated the involvement of the neuropeptide oxytocin (OT) in human social behaviors, including parent-infant relationships. Studies have investigated OT’s connection to human attachment to nonhuman animals, with the majority of the literature focusing on domestic dogs (Canis lupis familiaris). Utilizing what is known about OT and its role in maternal-infant and human-dog bonding, we apply these frameworks to the study of human-domestic cat (Felis catus) interactions. Methods. We investigated changes in salivary OT levels in 30 U.S. women of reproductive age before and after two conditions: reading a book (control) and interacting with their pet cat. Participant and cat behavioral patterns during the cat interaction condition were also quantified to determine if differences in women’s OT concentrations were associated with specific human and cat behaviors. Results. Our results revealed no changes in women’s OT levels during the cat interaction, relative to the control condition, and pre-cat interaction OT levels. However, differences in women’s OT concentrations were correlated with some human-cat interactions (e.g., positively with petting cat and cat approach initiation, negatively with cat agonistic behavior) but not all observed behaviors (e.g., use of gentle or baby voice) coded during human-cat interactions. Discussion. This study is the first to explore women’s OT in response to interactions with their pet cat and has identified distinct human and cat behaviors that influence OT release in humans. |
  | Date | 2021 |
  | Language | English |
  | Archive | Embase |
  | URL | https://www.embase.com/search/results?subaction=viewrecord&id=L2015610800&from=export |
  | Volume | 9 |
  | Publication | PeerJ |
  | DOI | 10.7717/peerj.12393 |
  | Issue | (Johnson E.A., Elizabeth.johnson@unlv.edu; Portillo A.; Bennett N.E.; Gray P.B.) Department of Anthropology, University of Nevada, Las Vegas, Las Vegas, NV, United States |
  | Journal Abbr | PeerJ |
  | ISSN | 2167-8359 |
  | Date Added | 11/07/2025, 12:13:25 |
  | Modified | 11/07/2025, 12:13:25 |

  ### Tags:

  - oxytocin
  - human-animal interaction
  - animal behavior
  - article
  - female
  - nonhuman
  - human
  - adult
  - controlled study
  - pet animal
  - questionnaire
  - clinical article
  - young adult
  - human-animal bond
  - enzyme linked immunosorbent assay
  - quantitative analysis
  - domestic cat
  - cat
  - gene expression
  - protein expression
  - Likert scale
  - oxytocin release
  - autopsy
  - birth control
  - distress syndrome
  - Expanded Disability Status Scale
  - household
  - neuropeptide
  - protein function

  ### Attachments

  - Full Text (HTML)
- ## Horses Working in Therapeutic Riding Programs: Cortisol, Adrenocorticotropic Hormone, Glucose, and Behavior Stress Indicators

  |  |  |
  | --- | --- |
  | Item Type | Journal Article |
  | Author | R.A. Johnson |
  | Author | P.J. Johnson |
  | Author | D.V. Megarani |
  | Author | S.D. Patel |
  | Author | H.D. Yaglom |
  | Author | S. Osterlind |
  | Author | K. Grindler |
  | Author | C.M. Vogelweid |
  | Author | T.M. Parker |
  | Author | C.K. Pascua |
  | Author | S.M. Crowder |
  | Abstract | In order to determine horses’ stress and ensure health and welfare of horses working with military veterans with posttraumatic stress disorder (PTSD) in a therapeutic horseback riding program, we measured the stress levels of the horses. Five horses worked in two 6-week therapeutic riding sessions: one 6-week session ridden by military veterans with PTSD (session 1) and one 6-week session ridden by experienced riders (session 2). Plasma adrenocorticotropic hormone (ACTH), glucose, and cortisol levels were determined on a resting day (baseline [BL]) and in weeks 1, 3, and 6 before each riding class (T1), after tacking (T2), and after each riding class (T3). Behavioral patterns for stress were determined before venipuncture at the same intervals. In session 1, cortisol level at BL was significantly lower than at T1 and T3, whereas in session 2, it was significantly higher than at T1 and T3. Cortisol level in session 1 was significantly higher than session 2 at T1 and T3. No significant differences were found between two sessions or over time for ACTH. Glucose level at BL was significantly higher than at T3 in session 1 and higher than at T1 in session 2. Glucose level in session 1 was significantly higher than session 2 at T1, T2, and T3. Stress behavior scores were significantly lower in session 1 than session 2. Although physiological and behavioral patterns for stress varied across experimental conditions, they remained within normal reference ranges. There was no evidence that horses carrying riders with a disability were unduly stressed. |
  | Date | 2017 |
  | Language | English |
  | Archive | Embase |
  | URL | https://www.embase.com/search/results?subaction=viewrecord&id=L617586909&from=export |
  | Volume | 57 |
  | Pages | 77-85 |
  | Publication | Journal of Equine Veterinary Science |
  | DOI | 10.1016/j.jevs.2017.05.006 |
  | Issue | (Johnson R.A., RAJohnson@missouri.edu; Megarani D.V.; Patel S.D.; Yaglom H.D.; Parker T.M.; Pascua C.K.; Crowder S.M.) Department of Veterinary Medicine & Surgery, Research Center for Human-Animal Interaction, College of Veterinary Medicine, University of Missouri, Columbia, MO, United States |
  | Journal Abbr | J. Equine Vet. Sci. |
  | ISSN | 0737-0806 |
  | Date Added | 11/07/2025, 14:18:37 |
  | Modified | 11/07/2025, 14:18:37 |

  ### Tags:

  - veteran
  - animal welfare
  - animal experiment
  - article
  - female
  - male
  - nonhuman
  - human
  - hydrocortisone
  - adult
  - horseback riding
  - posttraumatic stress disorder
  - vein puncture
  - corticotropin
  - clinical article
  - glucose
  - quantitative analysis
  - hydrocortisone blood level
  - army
  - behavioral stress
  - glucose blood level
  - corticotropin blood level
  - prospective study
  - traumatic brain injury

  ### Attachments

  - Full Text (HTML)
- ## Effects of Automated Short-Term Incomplete Milking During Peak to Mid Lactation on Udder Firmness, Heart-Rate Variability, and Behavior Recordings in Dairy Cows

  |  |  |
  | --- | --- |
  | Item Type | Journal Article |
  | Author | R. Joest |
  | Author | J. Stracke |
  | Author | U. Müller |
  | Abstract | Incomplete Milking (IM) can strategically be used to induce short-term milk yield reduction, benefiting dairy cows’ metabolism without affecting long-term productivity. However, while intermitted milking has been associated with elevated udder pressure and discomfort in dairy cows, IM´s welfare effects remain less well studied. The aim of this study was to evaluate the effects of automated short-term IM on udder filling and udder firmness, as well as on the well-being of dairy cows, by assessing heart rate variability (HRV) and behavior recordings. Udder healthy, multiparous dairy cows in peak- to mid-lactation were randomly assigned to the complete milking treatment (COMP; n = 23, 95 ± 49 days in milk, lactation number 3.0 ± 1.3 (mean ± SD)) or the incomplete milking treatment (INCL; n = 23, 93 ± 46 days in milk, lactation number 2.6 ± 0.8 (mean ± SD)). The IM consisted of two phases: In the 1st IM phase, milk withdrawal was gradually reduced by 9%/day over 5.5 days, resulting in app. 40% reduction and was followed by 3 complete milkings. Subsequently, in the 2nd IM phase, the 40% reduction was maintained consistently for further 5.5 days. Milk yield did not differ between treatments before IM (COMP: 39.1 ± 1.42 kg/day; INCL: 40.2 ± 1.66 kg/day (mean ± SEM) and a 43% reduced milk withdrawal was effectively achieved at the end of the 1st IM phase and throughout the 2nd IM phase. In INCL cows, udder filling increased, and udder firmness before milking was elevated on average by 18% during the 1st and by 22% during the 2nd IM phase, compared to COMP cows. After milking, udder firmness was on average 33% higher in INCL cows throughout both IM phases. Udder health was not affected by IM and the IM had no effect on HRV indices, which remained consistent between groups and over time (P > 0.05). No defense behavior was observed in the milking parlor, and behavioral observations in the barn showed no differences between treatments. This study demonstrates that, even though automated short-term IM resulted in increased udder filling and firmness – suggesting elevated residual milk and internal udder pressure –, IM does not indicate adverse effects on the well-being of dairy cows. These findings support IM as a viable management strategy for dairy cows. |
  | Date | 2025 |
  | Language | English |
  | URL | https://www.embase.com/search/results?subaction=viewrecord&id=L2039175151&from=export |
  | Publication | SSRN |
  | Issue | (Joest R.; Stracke J.; Müller U., ute-mueller@uni-bonn.de) Institute of Animal Science, University of Bonn, Endenicher Allee 15, Bonn, Germany |
  | ISSN | 1556-5068 |
  | Date Added | 11/07/2025, 11:53:14 |
  | Modified | 11/07/2025, 11:53:14 |

  ### Tags:

  - heart rate variability
  - cow
  - animal experiment
  - female
  - nonhuman
  - behavioral observation
  - dairy cattle
  - defensive behavior
  - lactation
  - milk yield
  - preprint
  - udder
- ## Effects of Automated Short-Term Incomplete Milking During Peak to Mid Lactation on Udder Firmness, Heart-Rate Variability, and Behavior Recordings in Dairy Cows

  |  |  |
  | --- | --- |
  | Item Type | Journal Article |
  | Author | R. Joest |
  | Author | J. Stracke |
  | Author | U. Müller |
  | Abstract | Incomplete Milking (IM) can strategically be used to induce short-term milk yield reduction, benefiting dairy cows’ metabolism without affecting long-term productivity. However, while intermitted milking has been associated with elevated udder pressure and discomfort in dairy cows, IM´s welfare effects remain less well studied. The aim of this study was to evaluate the effects of automated short-term IM on udder filling and udder firmness, as well as on the well-being of dairy cows, by assessing heart rate variability (HRV) and behavior recordings. Udder healthy, multiparous dairy cows in peak- to mid-lactation were randomly assigned to the complete milking treatment (COMP; n = 23, 95 ± 49 days in milk, lactation number 3.0 ± 1.3 (mean ± SD)) or the incomplete milking treatment (INCL; n = 23, 93 ± 46 days in milk, lactation number 2.6 ± 0.8 (mean ± SD)). The IM consisted of two phases: In the 1st IM phase, milk withdrawal was gradually reduced by 9%/day over 5.5 days, resulting in app. 40% reduction and was followed by 3 complete milkings. Subsequently, in the 2nd IM phase, the 40% reduction was maintained consistently for further 5.5 days. Milk yield did not differ between treatments before IM (COMP: 39.1 ± 1.42 kg/day; INCL: 40.2 ± 1.66 kg/day (mean ± SEM) and a 43% reduced milk withdrawal was effectively achieved at the end of the 1st IM phase and throughout the 2nd IM phase. In INCL cows, udder filling increased, and udder firmness before milking was elevated on average by 18% during the 1st and by 22% during the 2nd IM phase, compared to COMP cows. After milking, udder firmness was on average 33% higher in INCL cows throughout both IM phases. Udder health was not affected by IM and the IM had no effect on HRV indices, which remained consistent between groups and over time (P > 0.05). No defense behavior was observed in the milking parlor, and behavioral observations in the barn showed no differences between treatments. This study demonstrates that, even though automated short-term IM resulted in increased udder filling and firmness – suggesting elevated residual milk and internal udder pressure –, IM does not indicate adverse effects on the well-being of dairy cows. These findings support IM as a viable management strategy for dairy cows. |
  | Date | 2025 |
  | Language | English |
  | URL | https://www.embase.com/search/results?subaction=viewrecord&id=L2039175151&from=export |
  | Publication | SSRN |
  | Issue | (Joest R.; Stracke J.; Müller U., ute-mueller@uni-bonn.de) Institute of Animal Science, University of Bonn, Endenicher Allee 15, Bonn, Germany |
  | ISSN | 1556-5068 |
  | Date Added | 11/07/2025, 12:10:36 |
  | Modified | 11/07/2025, 12:10:36 |

  ### Tags:

  - heart rate variability
  - cow
  - animal experiment
  - female
  - nonhuman
  - behavioral observation
  - dairy cattle
  - defensive behavior
  - lactation
  - milk yield
  - preprint
  - udder
- ## Can dogs serve as stress mediators to decrease salivary cortisol levels in a population of liberal arts college undergraduate students?

  |  |  |
  | --- | --- |
  | Item Type | Journal Article |
  | Author | A.G. Jimenez |
  | Author | L. Calderaro |
  | Author | S. Clark |
  | Author | D. Elacqua |
  | Author | E. Hazen |
  | Author | V. Lam |
  | Author | G.S. Leightheiser |
  | Abstract | The steroid hormone cortisol can be used to measure physiological stress in humans. The hypothalamic-pituitary-adrenal (HPA) axis synthesizes cortisol, and a negative feedback cycle regulates cortisol depending on an individual's stress level and/or circadian rhythm. Chronic stress of college undergraduate students is associated with various adverse health effects, including anxiety and depression. Reports suggest that stress levels have risen dramatically in recent years, particularly among university students dealing with intense academic loads in addition to COVID-19 pandemic-related uncertainty. The increasing rate of mental illness on college campuses necessitates the study of mediators potentially capable of lowering stress, and thus cortisol levels. Research on mediation techniques and coping mechanisms have gained traction to address the concerning levels of stress, including the employment of human-animal interaction sessions on college campuses. In this study, human-canine interaction as a stress mediation strategy for undergraduate students was investigated. We measured salivary cortisol levels in 73 college undergraduate students during a 60-min interaction period with a dog to determine whether human-canine interactions are effective in lowering cortisol levels and potentially reducing chronic stress typical of undergraduate students. Our results indicate that a human-canine interaction for 60 min is an effective method for significantly reducing salivary cortisol and stress levels among undergraduate college students. These findings support the expansion of animal visitation programs on college campuses to help students manage stress. |
  | Date | 2023 |
  | Language | English |
  | Archive | Embase |
  | URL | https://www.embase.com/search/results?subaction=viewrecord&id=L2019846292&from=export |
  | Volume | 19 |
  | Pages | 283-289 |
  | Publication | Explore |
  | DOI | 10.1016/j.explore.2022.08.007 |
  | Issue | 3 |
  | Journal Abbr | Explor. |
  | ISSN | 1878-7541 |
  | Date Added | 11/07/2025, 14:15:33 |
  | Modified | 11/07/2025, 14:15:33 |

  ### Tags:

  - human-animal interaction
  - dog
  - article
  - female
  - male
  - nonhuman
  - physiological stress
  - college student
  - human
  - hydrocortisone
  - adult
  - controlled study
  - ELISA kit
  - pet animal
  - young adult
  - saliva level
  - chronic stress
  - undergraduate student
  - university

  ### Attachments

  - Full Text (HTML)
- ## Can dogs serve as stress mediators to decrease salivary cortisol levels in a population of liberal arts college undergraduate students?

  |  |  |
  | --- | --- |
  | Item Type | Journal Article |
  | Author | A.G. Jimenez |
  | Author | L. Calderaro |
  | Author | S. Clark |
  | Author | D. Elacqua |
  | Author | E. Hazen |
  | Author | V. Lam |
  | Author | G.S. Leightheiser |
  | Abstract | The steroid hormone cortisol can be used to measure physiological stress in humans. The hypothalamic-pituitary-adrenal (HPA) axis synthesizes cortisol, and a negative feedback cycle regulates cortisol depending on an individual's stress level and/or circadian rhythm. Chronic stress of college undergraduate students is associated with various adverse health effects, including anxiety and depression. Reports suggest that stress levels have risen dramatically in recent years, particularly among university students dealing with intense academic loads in addition to COVID-19 pandemic-related uncertainty. The increasing rate of mental illness on college campuses necessitates the study of mediators potentially capable of lowering stress, and thus cortisol levels. Research on mediation techniques and coping mechanisms have gained traction to address the concerning levels of stress, including the employment of human-animal interaction sessions on college campuses. In this study, human-canine interaction as a stress mediation strategy for undergraduate students was investigated. We measured salivary cortisol levels in 73 college undergraduate students during a 60-min interaction period with a dog to determine whether human-canine interactions are effective in lowering cortisol levels and potentially reducing chronic stress typical of undergraduate students. Our results indicate that a human-canine interaction for 60 min is an effective method for significantly reducing salivary cortisol and stress levels among undergraduate college students. These findings support the expansion of animal visitation programs on college campuses to help students manage stress. |
  | Date | 2023 |
  | Language | English |
  | Archive | Embase |
  | URL | https://www.embase.com/search/results?subaction=viewrecord&id=L2019846292&from=export |
  | Volume | 19 |
  | Pages | 283-289 |
  | Publication | Explore |
  | DOI | 10.1016/j.explore.2022.08.007 |
  | Issue | 3 |
  | Journal Abbr | Explor. |
  | ISSN | 1878-7541 |
  | Date Added | 11/07/2025, 14:16:43 |
  | Modified | 11/07/2025, 14:16:43 |

  ### Tags:

  - human-animal interaction
  - dog
  - article
  - female
  - male
  - nonhuman
  - physiological stress
  - college student
  - human
  - hydrocortisone
  - adult
  - controlled study
  - ELISA kit
  - pet animal
  - young adult
  - saliva level
  - chronic stress
  - undergraduate student
  - university

  ### Attachments

  - Full Text (HTML)
- ## Can dogs serve as stress mediators to decrease salivary cortisol levels in a population of liberal arts college undergraduate students?

  |  |  |
  | --- | --- |
  | Item Type | Journal Article |
  | Author | A.G. Jimenez |
  | Author | L. Calderaro |
  | Author | S. Clark |
  | Author | D. Elacqua |
  | Author | E. Hazen |
  | Author | V. Lam |
  | Author | G.S. Leightheiser |
  | Abstract | The steroid hormone cortisol can be used to measure physiological stress in humans. The hypothalamic-pituitary-adrenal (HPA) axis synthesizes cortisol, and a negative feedback cycle regulates cortisol depending on an individual's stress level and/or circadian rhythm. Chronic stress of college undergraduate students is associated with various adverse health effects, including anxiety and depression. Reports suggest that stress levels have risen dramatically in recent years, particularly among university students dealing with intense academic loads in addition to COVID-19 pandemic-related uncertainty. The increasing rate of mental illness on college campuses necessitates the study of mediators potentially capable of lowering stress, and thus cortisol levels. Research on mediation techniques and coping mechanisms have gained traction to address the concerning levels of stress, including the employment of human-animal interaction sessions on college campuses. In this study, human-canine interaction as a stress mediation strategy for undergraduate students was investigated. We measured salivary cortisol levels in 73 college undergraduate students during a 60-min interaction period with a dog to determine whether human-canine interactions are effective in lowering cortisol levels and potentially reducing chronic stress typical of undergraduate students. Our results indicate that a human-canine interaction for 60 min is an effective method for significantly reducing salivary cortisol and stress levels among undergraduate college students. These findings support the expansion of animal visitation programs on college campuses to help students manage stress. |
  | Date | 2023 |
  | Language | English |
  | Archive | Embase |
  | URL | https://www.embase.com/search/results?subaction=viewrecord&id=L2019846292&from=export |
  | Volume | 19 |
  | Pages | 283-289 |
  | Publication | Explore |
  | DOI | 10.1016/j.explore.2022.08.007 |
  | Issue | 3 |
  | Journal Abbr | Explor. |
  | ISSN | 1878-7541 |
  | Date Added | 11/07/2025, 14:18:16 |
  | Modified | 11/07/2025, 14:18:16 |

  ### Tags:

  - human-animal interaction
  - dog
  - article
  - female
  - male
  - nonhuman
  - physiological stress
  - college student
  - human
  - hydrocortisone
  - adult
  - controlled study
  - ELISA kit
  - pet animal
  - young adult
  - saliva level
  - chronic stress
  - undergraduate student
  - university

  ### Attachments

  - Full Text (HTML)
- ## Companion animals as buffer against the impact of stress on affect: An experience sampling study

  |  |  |
  | --- | --- |
  | Item Type | Journal Article |
  | Author | M. Janssens |
  | Author | E. Janssens |
  | Author | J. Eshuis |
  | Author | J. Lataster |
  | Author | M. Simons |
  | Author | J. Reijnders |
  | Author | N. Jacobs |
  | Abstract | Companion animals have been identified as a unique source of social support and as contributors to mental wellbeing. This study uses the Experience Sampling Method to test whether this effect is due to stress-buffering. A total of 159 dog and cat owners responded to a series of randomly scheduled questionnaires on their smartphones. At each measurement moment, they reported in whether a pet is present at that moment and to what extent they have interacted with the pet. They also reported on stressful activities and events and on their current positive (PA) and negative (NA) affect. Multilevel regression analyses showed that when a companion animal was present (vs. absent) the negative association between stress and PA is less pronounced (event stress: B = 0.13, p = 0.002, 95%CI = 0.05; 0.21 activity stress: B = 0.08, p < 0.001, 95%CI = 0.04; 0.12). No additional main effect was revealed when tested in a subsample of records that reported low or no stress. Main effects were found for the presence of a companion animal on negative affect (B = 0.08, p < 0.001; 95%CI = 0.12; 0.05) and for interacting with a companion animal on positive affect (B = 0.06, p < 0.001; 95%CI = 0.04; 0.08). This shows that the presence of a companion animal buffers against the negative consequences of stress on positive affect, indicating stress-buffering as a mechanism behind the peteffect. It is, however, not the only mechanism and more research is required to further elucidate how companion animals contribute to human wellbeing. |
  | Date | 2021 |
  | Language | English |
  | Archive | Embase |
  | URL | https://www.embase.com/search/results?subaction=viewrecord&id=L2007872649&from=export |
  | Volume | 11 |
  | Publication | Animals |
  | DOI | 10.3390/ani11082171 |
  | Issue | 8 |
  | Journal Abbr | Animals |
  | ISSN | 2076-2615 |
  | Date Added | 11/07/2025, 12:12:25 |
  | Modified | 11/07/2025, 12:12:25 |

  ### Tags:

  - oxytocin
  - social support
  - dog
  - article
  - female
  - male
  - physiological stress
  - college student
  - human
  - adult
  - aged
  - normal human
  - pet animal
  - questionnaire
  - wellbeing
  - human-animal bond
  - smartphone
  - education
  - cat
  - mental health
  - buffer

  ### Attachments

  - Full Text (HTML)
- ## Companion animals as buffer against the impact of stress on affect: An experience sampling study

  |  |  |
  | --- | --- |
  | Item Type | Journal Article |
  | Author | M. Janssens |
  | Author | E. Janssens |
  | Author | J. Eshuis |
  | Author | J. Lataster |
  | Author | M. Simons |
  | Author | J. Reijnders |
  | Author | N. Jacobs |
  | Abstract | Companion animals have been identified as a unique source of social support and as contributors to mental wellbeing. This study uses the Experience Sampling Method to test whether this effect is due to stress-buffering. A total of 159 dog and cat owners responded to a series of randomly scheduled questionnaires on their smartphones. At each measurement moment, they reported in whether a pet is present at that moment and to what extent they have interacted with the pet. They also reported on stressful activities and events and on their current positive (PA) and negative (NA) affect. Multilevel regression analyses showed that when a companion animal was present (vs. absent) the negative association between stress and PA is less pronounced (event stress: B = 0.13, p = 0.002, 95%CI = 0.05; 0.21 activity stress: B = 0.08, p < 0.001, 95%CI = 0.04; 0.12). No additional main effect was revealed when tested in a subsample of records that reported low or no stress. Main effects were found for the presence of a companion animal on negative affect (B = 0.08, p < 0.001; 95%CI = 0.12; 0.05) and for interacting with a companion animal on positive affect (B = 0.06, p < 0.001; 95%CI = 0.04; 0.08). This shows that the presence of a companion animal buffers against the negative consequences of stress on positive affect, indicating stress-buffering as a mechanism behind the peteffect. It is, however, not the only mechanism and more research is required to further elucidate how companion animals contribute to human wellbeing. |
  | Date | 2021 |
  | Language | English |
  | Archive | Embase |
  | URL | https://www.embase.com/search/results?subaction=viewrecord&id=L2007872649&from=export |
  | Volume | 11 |
  | Publication | Animals |
  | DOI | 10.3390/ani11082171 |
  | Issue | 8 |
  | Journal Abbr | Animals |
  | ISSN | 2076-2615 |
  | Date Added | 11/07/2025, 12:13:26 |
  | Modified | 11/07/2025, 12:13:26 |

  ### Tags:

  - oxytocin
  - social support
  - dog
  - article
  - female
  - male
  - physiological stress
  - college student
  - human
  - adult
  - aged
  - normal human
  - pet animal
  - questionnaire
  - wellbeing
  - human-animal bond
  - smartphone
  - education
  - cat
  - mental health
  - buffer

  ### Attachments

  - Full Text (HTML)
- ## Physiological Effect of Gentle Stroking in Lambs

  |  |  |
  | --- | --- |
  | Item Type | Journal Article |
  | Author | K. Janicka |
  | Author | P. Masier |
  | Author | P. Nazar |
  | Author | P. Staniszewska |
  | Author | G. Zięba |
  | Author | A. Strachecka |
  | Author | I. Rozempolska-Rucińska |
  | Abstract | The aim of the study was to determine changes in physiological indicators in lambs subjected to gentle stroking. The study included 40 three-week-old lambs (20 females of the synthetic prolific-meat (BCP) line and 20 females of the Świniarka (SW) breed). The animals were assigned to two control groups (n = 20) and two experimental groups (n = 20). Lambs from the experimental groups received treatment. Analyses consisted of determining the following parameters: heart rate (HR), saturation (S) and biochemical analysis. In the groups of sheep subjected to gentle massage, the analyses revealed a significant increase in the levels of cortisol, CAT, GST, GPx, among others, and a significant decrease in the levels of total protein, SOD, TAC, uric acid and Na+. At the same time, indicators of reduced stress levels were revealed, i.e., a significant increase in glycogen levels and a significant decrease in glucose and HR values. These results suggest that the introduction of gentle touch can induce positive states in lambs, and that the stress response may be transient and adaptive. Nevertheless, it is important to note that these interactions can be a source of stress, even if the behavioral response does not necessarily indicate stress. |
  | Date | 2024 |
  | Language | English |
  | Archive | Embase |
  | URL | https://www.embase.com/search/results?subaction=viewrecord&id=L2029149365&from=export |
  | Volume | 14 |
  | Publication | Animals |
  | DOI | 10.3390/ani14060887 |
  | Issue | 6 |
  | Journal Abbr | Animals |
  | ISSN | 2076-2615 |
  | Date Added | 11/07/2025, 11:53:19 |
  | Modified | 11/07/2025, 11:53:19 |

  ### Tags:

  - human-animal interaction
  - lamb
  - oxidative stress
  - physiology
  - stroking
  - heart rate
  - animal experiment
  - article
  - female
  - nonhuman
  - physiological stress
  - hydrocortisone
  - controlled study
  - ELISA kit
  - creatinine
  - oxygen saturation
  - sensor
  - tactile stimulation
  - adenosine triphosphatase
  - alanine aminotransferase
  - albumin
  - alkaline phosphatase
  - antioxidant activity
  - aspartate aminotransferase
  - biochemical analysis
  - calcium
  - calcium blood level
  - catalase
  - centrifuge
  - colorimetry
  - enzyme linked immunosorbent assay
  - experimental behavioral test
  - gamma glutamyltransferase
  - glucose
  - glutathione
  - glutathione peroxidase
  - glutathione peroxidase assay kit
  - glutathione reductase
  - glutathione transferase
  - glycogen
  - glycogen assay kit K646-100
  - lactate dehydrogenase
  - lactate dehydrogenase assay kit
  - lactate dehydrogenase test kit
  - LDH Assay Kit
  - magnesium
  - malonaldehyde
  - MPW 350R
  - multivariate analysis of variance
  - muscle excitation
  - protein
  - pulse oximeter
  - SOD Determination Kit
  - sodium
  - superoxide dismutase
  - thoracic limb
  - total antioxidant capacity
  - triacylglycerol
  - urea
  - uric acid
  - water availability

  ### Attachments

  - Full Text (HTML)
- ## Physiological Effect of Gentle Stroking in Lambs

  |  |  |
  | --- | --- |
  | Item Type | Journal Article |
  | Author | K. Janicka |
  | Author | P. Masier |
  | Author | P. Nazar |
  | Author | P. Staniszewska |
  | Author | G. Zięba |
  | Author | A. Strachecka |
  | Author | I. Rozempolska-Rucińska |
  | Abstract | The aim of the study was to determine changes in physiological indicators in lambs subjected to gentle stroking. The study included 40 three-week-old lambs (20 females of the synthetic prolific-meat (BCP) line and 20 females of the Świniarka (SW) breed). The animals were assigned to two control groups (n = 20) and two experimental groups (n = 20). Lambs from the experimental groups received treatment. Analyses consisted of determining the following parameters: heart rate (HR), saturation (S) and biochemical analysis. In the groups of sheep subjected to gentle massage, the analyses revealed a significant increase in the levels of cortisol, CAT, GST, GPx, among others, and a significant decrease in the levels of total protein, SOD, TAC, uric acid and Na+. At the same time, indicators of reduced stress levels were revealed, i.e., a significant increase in glycogen levels and a significant decrease in glucose and HR values. These results suggest that the introduction of gentle touch can induce positive states in lambs, and that the stress response may be transient and adaptive. Nevertheless, it is important to note that these interactions can be a source of stress, even if the behavioral response does not necessarily indicate stress. |
  | Date | 2024 |
  | Language | English |
  | Archive | Embase |
  | URL | https://www.embase.com/search/results?subaction=viewrecord&id=L2029149365&from=export |
  | Volume | 14 |
  | Publication | Animals |
  | DOI | 10.3390/ani14060887 |
  | Issue | 6 |
  | Journal Abbr | Animals |
  | ISSN | 2076-2615 |
  | Date Added | 11/07/2025, 12:10:42 |
  | Modified | 11/07/2025, 12:10:42 |

  ### Tags:

  - human-animal interaction
  - lamb
  - oxidative stress
  - physiology
  - stroking
  - heart rate
  - animal experiment
  - article
  - female
  - nonhuman
  - physiological stress
  - hydrocortisone
  - controlled study
  - ELISA kit
  - creatinine
  - oxygen saturation
  - sensor
  - tactile stimulation
  - adenosine triphosphatase
  - alanine aminotransferase
  - albumin
  - alkaline phosphatase
  - antioxidant activity
  - aspartate aminotransferase
  - biochemical analysis
  - calcium
  - calcium blood level
  - catalase
  - centrifuge
  - colorimetry
  - enzyme linked immunosorbent assay
  - experimental behavioral test
  - gamma glutamyltransferase
  - glucose
  - glutathione
  - glutathione peroxidase
  - glutathione peroxidase assay kit
  - glutathione reductase
  - glutathione transferase
  - glycogen
  - glycogen assay kit K646-100
  - lactate dehydrogenase
  - lactate dehydrogenase assay kit
  - lactate dehydrogenase test kit
  - LDH Assay Kit
  - magnesium
  - malonaldehyde
  - MPW 350R
  - multivariate analysis of variance
  - muscle excitation
  - protein
  - pulse oximeter
  - SOD Determination Kit
  - sodium
  - superoxide dismutase
  - thoracic limb
  - total antioxidant capacity
  - triacylglycerol
  - urea
  - uric acid
  - water availability

  ### Attachments

  - Full Text (HTML)
- ## Physiological Effect of Gentle Stroking in Lambs

  |  |  |
  | --- | --- |
  | Item Type | Journal Article |
  | Author | K. Janicka |
  | Author | P. Masier |
  | Author | P. Nazar |
  | Author | P. Staniszewska |
  | Author | G. Zięba |
  | Author | A. Strachecka |
  | Author | I. Rozempolska-Rucińska |
  | Abstract | The aim of the study was to determine changes in physiological indicators in lambs subjected to gentle stroking. The study included 40 three-week-old lambs (20 females of the synthetic prolific-meat (BCP) line and 20 females of the Świniarka (SW) breed). The animals were assigned to two control groups (n = 20) and two experimental groups (n = 20). Lambs from the experimental groups received treatment. Analyses consisted of determining the following parameters: heart rate (HR), saturation (S) and biochemical analysis. In the groups of sheep subjected to gentle massage, the analyses revealed a significant increase in the levels of cortisol, CAT, GST, GPx, among others, and a significant decrease in the levels of total protein, SOD, TAC, uric acid and Na+. At the same time, indicators of reduced stress levels were revealed, i.e., a significant increase in glycogen levels and a significant decrease in glucose and HR values. These results suggest that the introduction of gentle touch can induce positive states in lambs, and that the stress response may be transient and adaptive. Nevertheless, it is important to note that these interactions can be a source of stress, even if the behavioral response does not necessarily indicate stress. |
  | Date | 2024 |
  | Language | English |
  | Archive | Embase |
  | URL | https://www.embase.com/search/results?subaction=viewrecord&id=L2029149365&from=export |
  | Volume | 14 |
  | Publication | Animals |
  | DOI | 10.3390/ani14060887 |
  | Issue | 6 |
  | Journal Abbr | Animals |
  | ISSN | 2076-2615 |
  | Date Added | 11/07/2025, 14:15:30 |
  | Modified | 11/07/2025, 14:15:30 |

  ### Tags:

  - human-animal interaction
  - lamb
  - oxidative stress
  - physiology
  - stroking
  - heart rate
  - animal experiment
  - article
  - female
  - nonhuman
  - physiological stress
  - hydrocortisone
  - controlled study
  - ELISA kit
  - creatinine
  - oxygen saturation
  - sensor
  - tactile stimulation
  - adenosine triphosphatase
  - alanine aminotransferase
  - albumin
  - alkaline phosphatase
  - antioxidant activity
  - aspartate aminotransferase
  - biochemical analysis
  - calcium
  - calcium blood level
  - catalase
  - centrifuge
  - colorimetry
  - enzyme linked immunosorbent assay
  - experimental behavioral test
  - gamma glutamyltransferase
  - glucose
  - glutathione
  - glutathione peroxidase
  - glutathione peroxidase assay kit
  - glutathione reductase
  - glutathione transferase
  - glycogen
  - glycogen assay kit K646-100
  - lactate dehydrogenase
  - lactate dehydrogenase assay kit
  - lactate dehydrogenase test kit
  - LDH Assay Kit
  - magnesium
  - malonaldehyde
  - MPW 350R
  - multivariate analysis of variance
  - muscle excitation
  - protein
  - pulse oximeter
  - SOD Determination Kit
  - sodium
  - superoxide dismutase
  - thoracic limb
  - total antioxidant capacity
  - triacylglycerol
  - urea
  - uric acid
  - water availability

  ### Attachments

  - Full Text (HTML)
- ## Physiological Effect of Gentle Stroking in Lambs

  |  |  |
  | --- | --- |
  | Item Type | Journal Article |
  | Author | K. Janicka |
  | Author | P. Masier |
  | Author | P. Nazar |
  | Author | P. Staniszewska |
  | Author | G. Zięba |
  | Author | A. Strachecka |
  | Author | I. Rozempolska-Rucińska |
  | Abstract | The aim of the study was to determine changes in physiological indicators in lambs subjected to gentle stroking. The study included 40 three-week-old lambs (20 females of the synthetic prolific-meat (BCP) line and 20 females of the Świniarka (SW) breed). The animals were assigned to two control groups (n = 20) and two experimental groups (n = 20). Lambs from the experimental groups received treatment. Analyses consisted of determining the following parameters: heart rate (HR), saturation (S) and biochemical analysis. In the groups of sheep subjected to gentle massage, the analyses revealed a significant increase in the levels of cortisol, CAT, GST, GPx, among others, and a significant decrease in the levels of total protein, SOD, TAC, uric acid and Na+. At the same time, indicators of reduced stress levels were revealed, i.e., a significant increase in glycogen levels and a significant decrease in glucose and HR values. These results suggest that the introduction of gentle touch can induce positive states in lambs, and that the stress response may be transient and adaptive. Nevertheless, it is important to note that these interactions can be a source of stress, even if the behavioral response does not necessarily indicate stress. |
  | Date | 2024 |
  | Language | English |
  | Archive | Embase |
  | URL | https://www.embase.com/search/results?subaction=viewrecord&id=L2029149365&from=export |
  | Volume | 14 |
  | Publication | Animals |
  | DOI | 10.3390/ani14060887 |
  | Issue | 6 |
  | Journal Abbr | Animals |
  | ISSN | 2076-2615 |
  | Date Added | 11/07/2025, 14:16:40 |
  | Modified | 11/07/2025, 14:16:40 |

  ### Tags:

  - human-animal interaction
  - lamb
  - oxidative stress
  - physiology
  - stroking
  - heart rate
  - animal experiment
  - article
  - female
  - nonhuman
  - physiological stress
  - hydrocortisone
  - controlled study
  - ELISA kit
  - creatinine
  - oxygen saturation
  - sensor
  - tactile stimulation
  - adenosine triphosphatase
  - alanine aminotransferase
  - albumin
  - alkaline phosphatase
  - antioxidant activity
  - aspartate aminotransferase
  - biochemical analysis
  - calcium
  - calcium blood level
  - catalase
  - centrifuge
  - colorimetry
  - enzyme linked immunosorbent assay
  - experimental behavioral test
  - gamma glutamyltransferase
  - glucose
  - glutathione
  - glutathione peroxidase
  - glutathione peroxidase assay kit
  - glutathione reductase
  - glutathione transferase
  - glycogen
  - glycogen assay kit K646-100
  - lactate dehydrogenase
  - lactate dehydrogenase assay kit
  - lactate dehydrogenase test kit
  - LDH Assay Kit
  - magnesium
  - malonaldehyde
  - MPW 350R
  - multivariate analysis of variance
  - muscle excitation
  - protein
  - pulse oximeter
  - SOD Determination Kit
  - sodium
  - superoxide dismutase
  - thoracic limb
  - total antioxidant capacity
  - triacylglycerol
  - urea
  - uric acid
  - water availability

  ### Attachments

  - Full Text (HTML)
- ## Physiological Effect of Gentle Stroking in Lambs

  |  |  |
  | --- | --- |
  | Item Type | Journal Article |
  | Author | K. Janicka |
  | Author | P. Masier |
  | Author | P. Nazar |
  | Author | P. Staniszewska |
  | Author | G. Zięba |
  | Author | A. Strachecka |
  | Author | I. Rozempolska-Rucińska |
  | Abstract | The aim of the study was to determine changes in physiological indicators in lambs subjected to gentle stroking. The study included 40 three-week-old lambs (20 females of the synthetic prolific-meat (BCP) line and 20 females of the Świniarka (SW) breed). The animals were assigned to two control groups (n = 20) and two experimental groups (n = 20). Lambs from the experimental groups received treatment. Analyses consisted of determining the following parameters: heart rate (HR), saturation (S) and biochemical analysis. In the groups of sheep subjected to gentle massage, the analyses revealed a significant increase in the levels of cortisol, CAT, GST, GPx, among others, and a significant decrease in the levels of total protein, SOD, TAC, uric acid and Na+. At the same time, indicators of reduced stress levels were revealed, i.e., a significant increase in glycogen levels and a significant decrease in glucose and HR values. These results suggest that the introduction of gentle touch can induce positive states in lambs, and that the stress response may be transient and adaptive. Nevertheless, it is important to note that these interactions can be a source of stress, even if the behavioral response does not necessarily indicate stress. |
  | Date | 2024 |
  | Language | English |
  | Archive | Embase |
  | URL | https://www.embase.com/search/results?subaction=viewrecord&id=L2029149365&from=export |
  | Volume | 14 |
  | Publication | Animals |
  | DOI | 10.3390/ani14060887 |
  | Issue | 6 |
  | Journal Abbr | Animals |
  | ISSN | 2076-2615 |
  | Date Added | 11/07/2025, 14:18:13 |
  | Modified | 11/07/2025, 14:18:13 |

  ### Tags:

  - human-animal interaction
  - lamb
  - oxidative stress
  - physiology
  - stroking
  - heart rate
  - animal experiment
  - article
  - female
  - nonhuman
  - physiological stress
  - hydrocortisone
  - controlled study
  - ELISA kit
  - creatinine
  - oxygen saturation
  - sensor
  - tactile stimulation
  - adenosine triphosphatase
  - alanine aminotransferase
  - albumin
  - alkaline phosphatase
  - antioxidant activity
  - aspartate aminotransferase
  - biochemical analysis
  - calcium
  - calcium blood level
  - catalase
  - centrifuge
  - colorimetry
  - enzyme linked immunosorbent assay
  - experimental behavioral test
  - gamma glutamyltransferase
  - glucose
  - glutathione
  - glutathione peroxidase
  - glutathione peroxidase assay kit
  - glutathione reductase
  - glutathione transferase
  - glycogen
  - glycogen assay kit K646-100
  - lactate dehydrogenase
  - lactate dehydrogenase assay kit
  - lactate dehydrogenase test kit
  - LDH Assay Kit
  - magnesium
  - malonaldehyde
  - MPW 350R
  - multivariate analysis of variance
  - muscle excitation
  - protein
  - pulse oximeter
  - SOD Determination Kit
  - sodium
  - superoxide dismutase
  - thoracic limb
  - total antioxidant capacity
  - triacylglycerol
  - urea
  - uric acid
  - water availability

  ### Attachments

  - Full Text (HTML)
- ## Herd-level associations between human-animal relationship, management, fecal cortisol metabolites, and udder health of organic dairy cows

  |  |  |
  | --- | --- |
  | Item Type | Journal Article |
  | Author | S. Ivemeyer |
  | Author | C. Simantke |
  | Author | A. Ebinghaus |
  | Author | P.H. Poulsen |
  | Author | J.T. Sorensen |
  | Author | T. Rousing |
  | Author | R. Palme |
  | Author | U. Knierim |
  | Abstract | Impact patterns of human-animal relationship (HAR) and herd stress level on udder health were investigated in a cross-sectional study on 30 German and Danish organic dairy herds also taking into account influencing factors regarding housing and management. Cow behavior (avoidance distance, tolerance to tactile interaction, release behavior) was assessed in tests, milkers' behavior recorded during milking, and information about contacts with animals during routine work gathered by interview. Additionally, stockpersons' attitudes were recorded via questionnaires. Fecal cortisol metabolites were measured in approximately 30 focal cows on each farm and used as a proxy to determine the level of distress within the herd. Management and housing were assessed on-farm. The following herd udder health indicators were calculated: the prevalence of mastitis quarters (≥100,000 cells/mL), and, from milk recording data over 1 yr retrospectively, the average somatic cell score and the self-cure rates during lactation per herd. Multivariable regression models with stepwise selection were calculated at herd level. The following HAR-related factors were associated with better udder health (in at least 1 of the final models): stockpersons' higher agreement on patience being important when moving the cows and on necessary contact to cows being pleasant, higher amount of positive interactions with cows during milking, more docile cows in the release behavior test, no routine change of milkers, more contact time during routine work, no active heifer habituation to milking, and performance of barn controls beyond routine work. Lower fecal cortisol metabolite levels were related to higher self-cure rates during lactation. Concerning housing, management, and herd characteristics, the following known factors were related to impaired udder health for at least 1 of the indicators: straw yards, automatic milking system, higher average lactation number, and less antibiotic udder treatments. The results confirm earlier findings that HAR is associated with udder health and should therefore be considered in future research and mastitis control programs. First indications of negative associations between herd stress level and mastitis curing capacity should be followed up in future studies. |
  | Date | 2018 |
  | Language | English |
  | Archive | Medline |
  | URL | https://www.embase.com/search/results?subaction=viewrecord&id=L626591019&from=export |
  | Volume | 101 |
  | Pages | 7361-7374 |
  | Publication | Journal of dairy science |
  | DOI | 10.3168/jds.2017-13912 |
  | Issue | 8 |
  | Journal Abbr | J. Dairy Sci. |
  | ISSN | 1525-3198 |
  | Date Added | 11/07/2025, 14:18:36 |
  | Modified | 11/07/2025, 14:18:36 |

  ### Tags:

  - physiology
  - female
  - human
  - hydrocortisone
  - udder
  - bovine
  - animal
  - blood
  - human-animal bond
  - metabolism
  - cross-sectional study
  - procedures
  - dairying
  - milk
  - retrospective study

  ### Attachments

  - Full Text (HTML)
- ## Impact of Procedures and Human-Animal Interactions during Transport and Slaughter on Animal Welfare of Pigs: A Systematic Literature Review

  |  |  |
  | --- | --- |
  | Item Type | Journal Article |
  | Author | R. Isbrandt |
  | Author | M. Wiegard |
  | Author | D. Meemken |
  | Author | N. Langkabel |
  | Abstract | Animal welfare is a high social and political priority and is enshrined in European and national legislation. This systematic literature review (funded by the German Ministry of Food and Agriculture; grant no. 2817806A18) was conducted to find animal and management-based indicators that could be influenced by changes in pig handling and management procedures on the day of slaughter and, therefore, will directly result in improved animal welfare. For this systematic literature review, following the PRISMA guidelines, we conducted a structured literature search using the databases PubMed®, Web of ScienceTM, and LIVIVO with set search terms and their combinations in German and English. Only peer-reviewed original articles from European countries from 2009 until 2022 that dealt with welfare during transport and/or slaughter of fattening pigs and offered potential measurable indicators on the basis of the animal or management, and either directly or indirectly recommended handling practices were included. We used the literature management system EndNoteTM, and after duplicate removal, a total of 1099 records were found for further analysis. After analysis and discussion in the review team, which consisted of three experts in total, 105 articles underwent full-text screening. Finally, 39 articles were included in the results for this systematic literature review. According to this systematic literature review, the following procedures have a positive impact on the animal welfare of pigs on the day of slaughter. Calm—rather than rough—handling of pigs was the most influential factor. This can be achieved by using animal-friendly driving aids. Loud noise should be avoided in general or reduced as far as possible. Transport time is not always influenceable, but shorter transport duration with stocking density as stated by the European regulations as a maximum will reduce stress in pigs. Our review showed that there are differing scientific results about lairage duration, although this should be set in relation to prior stress and transport time. Knowledge of good stunning parameters, such as gas concentration, exposure time to the gas, recommended amperage, and current flow duration is essential. If electrical stunning is performed, the correct attachment of electrodes is crucial. The control of stunning effectiveness is extremely important to avoid suffering and pain, with the proviso that it is assessed on the basis of multiple parameters. The corneal reflex, regular gasping, and the up-righting reflex could be assessed together. Certificates of competence and continuing educational programmes for the personnel form the basis of animal-friendly handling and a stressless slaughter routine. It can be assumed that the results of this systematic literature review reflect the state of current research of pig welfare during transport and slaughter in the European Union, even though it must be assumed that not all relevant points were covered by the review, which can be seen as a limitation at the same time because important non-European or older publications could not be included. |
  | Date | 2022 |
  | Language | English |
  | Archive | Embase |
  | URL | https://www.embase.com/search/results?subaction=viewrecord&id=L2020506030&from=export |
  | Volume | 12 |
  | Publication | Animals |
  | DOI | 10.3390/ani12233391 |
  | Issue | 23 |
  | Journal Abbr | Animals |
  | ISSN | 2076-2615 |
  | Date Added | 11/07/2025, 12:12:19 |
  | Modified | 11/07/2025, 12:12:19 |

  ### Tags:

  - oxytocin
  - human-animal interaction
  - animal welfare
  - animal behavior
  - nonhuman
  - human
  - review
  - pig
  - electrode
  - pain
  - slaughtering
  - lactate dehydrogenase
  - amylase
  - C reactive protein
  - systematic review
  - hydrocortisone blood level
  - risk factor
  - meat
  - bleeding time
  - carbon dioxide stunning
  - cholinesterase
  - cornea reflex
  - creatine kinase
  - driver
  - electroanesthesia
  - esterase
  - gasping
  - haptoglobin
  - lactate blood level
  - medical procedures
  - neutrophil lymphocyte ratio
  - noise
  - pathologic nystagmus
  - righting reflex
  - skin defect
  - stocking density

  ### Attachments

  - Full Text (HTML)
- ## Impact of Procedures and Human-Animal Interactions during Transport and Slaughter on Animal Welfare of Pigs: A Systematic Literature Review

  |  |  |
  | --- | --- |
  | Item Type | Journal Article |
  | Author | R. Isbrandt |
  | Author | M. Wiegard |
  | Author | D. Meemken |
  | Author | N. Langkabel |
  | Abstract | Animal welfare is a high social and political priority and is enshrined in European and national legislation. This systematic literature review (funded by the German Ministry of Food and Agriculture; grant no. 2817806A18) was conducted to find animal and management-based indicators that could be influenced by changes in pig handling and management procedures on the day of slaughter and, therefore, will directly result in improved animal welfare. For this systematic literature review, following the PRISMA guidelines, we conducted a structured literature search using the databases PubMed®, Web of ScienceTM, and LIVIVO with set search terms and their combinations in German and English. Only peer-reviewed original articles from European countries from 2009 until 2022 that dealt with welfare during transport and/or slaughter of fattening pigs and offered potential measurable indicators on the basis of the animal or management, and either directly or indirectly recommended handling practices were included. We used the literature management system EndNoteTM, and after duplicate removal, a total of 1099 records were found for further analysis. After analysis and discussion in the review team, which consisted of three experts in total, 105 articles underwent full-text screening. Finally, 39 articles were included in the results for this systematic literature review. According to this systematic literature review, the following procedures have a positive impact on the animal welfare of pigs on the day of slaughter. Calm—rather than rough—handling of pigs was the most influential factor. This can be achieved by using animal-friendly driving aids. Loud noise should be avoided in general or reduced as far as possible. Transport time is not always influenceable, but shorter transport duration with stocking density as stated by the European regulations as a maximum will reduce stress in pigs. Our review showed that there are differing scientific results about lairage duration, although this should be set in relation to prior stress and transport time. Knowledge of good stunning parameters, such as gas concentration, exposure time to the gas, recommended amperage, and current flow duration is essential. If electrical stunning is performed, the correct attachment of electrodes is crucial. The control of stunning effectiveness is extremely important to avoid suffering and pain, with the proviso that it is assessed on the basis of multiple parameters. The corneal reflex, regular gasping, and the up-righting reflex could be assessed together. Certificates of competence and continuing educational programmes for the personnel form the basis of animal-friendly handling and a stressless slaughter routine. It can be assumed that the results of this systematic literature review reflect the state of current research of pig welfare during transport and slaughter in the European Union, even though it must be assumed that not all relevant points were covered by the review, which can be seen as a limitation at the same time because important non-European or older publications could not be included. |
  | Date | 2022 |
  | Language | English |
  | Archive | Embase |
  | URL | https://www.embase.com/search/results?subaction=viewrecord&id=L2020506030&from=export |
  | Volume | 12 |
  | Publication | Animals |
  | DOI | 10.3390/ani12233391 |
  | Issue | 23 |
  | Journal Abbr | Animals |
  | ISSN | 2076-2615 |
  | Date Added | 11/07/2025, 12:13:21 |
  | Modified | 11/07/2025, 12:13:21 |

  ### Tags:

  - oxytocin
  - human-animal interaction
  - animal welfare
  - animal behavior
  - nonhuman
  - human
  - review
  - pig
  - electrode
  - pain
  - slaughtering
  - lactate dehydrogenase
  - amylase
  - C reactive protein
  - systematic review
  - hydrocortisone blood level
  - risk factor
  - meat
  - bleeding time
  - carbon dioxide stunning
  - cholinesterase
  - cornea reflex
  - creatine kinase
  - driver
  - electroanesthesia
  - esterase
  - gasping
  - haptoglobin
  - lactate blood level
  - medical procedures
  - neutrophil lymphocyte ratio
  - noise
  - pathologic nystagmus
  - righting reflex
  - skin defect
  - stocking density

  ### Attachments

  - Full Text (HTML)
- ## Effect of long-term management of hypoadrenocorticism on the quality of life of affected dogs and their owners

  |  |  |
  | --- | --- |
  | Item Type | Journal Article |
  | Author | J. Hupfeld |
  | Author | M. Dölle |
  | Author | H. Volk |
  | Author | J. Rieder |
  | Abstract | Background: The treatment of canine adrenal insufficiency consists of hormone substitution and requires high owner compliance and intense human–dog interaction. This might affect the quality of life (QoL) of owners and their pets. The aim of the study was to evaluate the impact of hypoadrenocorticism and its treatment on the QoL of dogs and their owners. Methods: Owners completed a web-based survey that contained items concerning signalment, owner QoL, dog QoL and long-term therapy. Results: Three hundred and twenty-two owners participated. Most owners feared an adrenal crisis. Approximately half of the participants reported that the bond between them and their pet increased after diagnosis. Although many participants felt that their own QoL was not affected by their dog's disease, worries about costs and leaving their dog unsupervised were frequently reported. Half of the study participants increased their dog's glucocorticoid doses when a stressful situation was foreseeable (‘boosting’ of therapy). Some administered hydrocortisone, mostly switched from prednisolone, resulting in a reduction in side effects. Conclusions: Special attention should be given to glucocorticoid therapy and owner's QoL. The overall worry of an adrenal crisis might increase caregivers’ burden, reducing their overall QoL. Hydrocortisone might be a safe alternative to prednisolone, but further research is necessary to evaluate its long-term efficacy and safety in dogs. |
  | Date | 2022 |
  | Language | English |
  | Archive | Embase |
  | URL | https://www.embase.com/search/results?subaction=viewrecord&id=L2018456780&from=export |
  | Volume | 191 |
  | Pages | no |
  | Publication | Veterinary Record |
  | DOI | 10.1002/vetr.1977 |
  | Issue | 10 |
  | Journal Abbr | Vet. Rec. |
  | ISSN | 2042-7670 |
  | Date Added | 11/07/2025, 14:15:36 |
  | Modified | 11/07/2025, 14:15:36 |

  ### Tags:

  - dog
  - article
  - female
  - male
  - nonhuman
  - human
  - hydrocortisone
  - quality of life
  - human-animal bond
  - fear
  - observational study
  - long term care
  - cross-sectional study
  - adrenal cortex insufficiency
  - corticosteroid therapy
  - dog disease
  - drug safety
  - health care cost
  - hormone substitution
  - hypoadrenocorticism
  - mineralocorticoid
  - prednisolone

  ### Attachments

  - Full Text (HTML)
- ## Effect of long-term management of hypoadrenocorticism on the quality of life of affected dogs and their owners

  |  |  |
  | --- | --- |
  | Item Type | Journal Article |
  | Author | J. Hupfeld |
  | Author | M. Dölle |
  | Author | H. Volk |
  | Author | J. Rieder |
  | Abstract | Background: The treatment of canine adrenal insufficiency consists of hormone substitution and requires high owner compliance and intense human–dog interaction. This might affect the quality of life (QoL) of owners and their pets. The aim of the study was to evaluate the impact of hypoadrenocorticism and its treatment on the QoL of dogs and their owners. Methods: Owners completed a web-based survey that contained items concerning signalment, owner QoL, dog QoL and long-term therapy. Results: Three hundred and twenty-two owners participated. Most owners feared an adrenal crisis. Approximately half of the participants reported that the bond between them and their pet increased after diagnosis. Although many participants felt that their own QoL was not affected by their dog's disease, worries about costs and leaving their dog unsupervised were frequently reported. Half of the study participants increased their dog's glucocorticoid doses when a stressful situation was foreseeable (‘boosting’ of therapy). Some administered hydrocortisone, mostly switched from prednisolone, resulting in a reduction in side effects. Conclusions: Special attention should be given to glucocorticoid therapy and owner's QoL. The overall worry of an adrenal crisis might increase caregivers’ burden, reducing their overall QoL. Hydrocortisone might be a safe alternative to prednisolone, but further research is necessary to evaluate its long-term efficacy and safety in dogs. |
  | Date | 2022 |
  | Language | English |
  | Archive | Embase |
  | URL | https://www.embase.com/search/results?subaction=viewrecord&id=L2018456780&from=export |
  | Volume | 191 |
  | Pages | no |
  | Publication | Veterinary Record |
  | DOI | 10.1002/vetr.1977 |
  | Issue | 10 |
  | Journal Abbr | Vet. Rec. |
  | ISSN | 2042-7670 |
  | Date Added | 11/07/2025, 14:16:46 |
  | Modified | 11/07/2025, 14:16:46 |

  ### Tags:

  - dog
  - article
  - female
  - male
  - nonhuman
  - human
  - hydrocortisone
  - quality of life
  - human-animal bond
  - fear
  - observational study
  - long term care
  - cross-sectional study
  - adrenal cortex insufficiency
  - corticosteroid therapy
  - dog disease
  - drug safety
  - health care cost
  - hormone substitution
  - hypoadrenocorticism
  - mineralocorticoid
  - prednisolone

  ### Attachments

  - Full Text (HTML)
- ## Effect of long-term management of hypoadrenocorticism on the quality of life of affected dogs and their owners

  |  |  |
  | --- | --- |
  | Item Type | Journal Article |
  | Author | J. Hupfeld |
  | Author | M. Dölle |
  | Author | H. Volk |
  | Author | J. Rieder |
  | Abstract | Background: The treatment of canine adrenal insufficiency consists of hormone substitution and requires high owner compliance and intense human–dog interaction. This might affect the quality of life (QoL) of owners and their pets. The aim of the study was to evaluate the impact of hypoadrenocorticism and its treatment on the QoL of dogs and their owners. Methods: Owners completed a web-based survey that contained items concerning signalment, owner QoL, dog QoL and long-term therapy. Results: Three hundred and twenty-two owners participated. Most owners feared an adrenal crisis. Approximately half of the participants reported that the bond between them and their pet increased after diagnosis. Although many participants felt that their own QoL was not affected by their dog's disease, worries about costs and leaving their dog unsupervised were frequently reported. Half of the study participants increased their dog's glucocorticoid doses when a stressful situation was foreseeable (‘boosting’ of therapy). Some administered hydrocortisone, mostly switched from prednisolone, resulting in a reduction in side effects. Conclusions: Special attention should be given to glucocorticoid therapy and owner's QoL. The overall worry of an adrenal crisis might increase caregivers’ burden, reducing their overall QoL. Hydrocortisone might be a safe alternative to prednisolone, but further research is necessary to evaluate its long-term efficacy and safety in dogs. |
  | Date | 2022 |
  | Language | English |
  | Archive | Embase |
  | URL | https://www.embase.com/search/results?subaction=viewrecord&id=L2018456780&from=export |
  | Volume | 191 |
  | Pages | no |
  | Publication | Veterinary Record |
  | DOI | 10.1002/vetr.1977 |
  | Issue | 10 |
  | Journal Abbr | Vet. Rec. |
  | ISSN | 2042-7670 |
  | Date Added | 11/07/2025, 14:18:20 |
  | Modified | 11/07/2025, 14:18:20 |

  ### Tags:

  - dog
  - article
  - female
  - male
  - nonhuman
  - human
  - hydrocortisone
  - quality of life
  - human-animal bond
  - fear
  - observational study
  - long term care
  - cross-sectional study
  - adrenal cortex insufficiency
  - corticosteroid therapy
  - dog disease
  - drug safety
  - health care cost
  - hormone substitution
  - hypoadrenocorticism
  - mineralocorticoid
  - prednisolone

  ### Attachments

  - Full Text (HTML)
- ## Exploring the Dynamics of Canine-Assisted Interactions: A Wearable Approach to Understanding Interspecies Well-Being

  |  |  |
  | --- | --- |
  | Item Type | Journal Article |
  | Author | T.R.N. Holder |
  | Author | C. Nichols |
  | Author | E. Summers |
  | Author | D.L. Roberts |
  | Author | A. Bozkurt |
  | Abstract | Canine-assisted interactions (CAIs) have been explored to offer therapeutic benefits to human participants in various contexts, from addressing cancer-related fatigue to treating post-traumatic stress disorder. Despite their widespread adoption, there are still unresolved questions regarding the outcomes for both humans and animals involved in these interactions. Previous attempts to address these questions have suffered from core methodological weaknesses, especially due to absence of tools for an efficient objective evaluation and lack of focus on the canine perspective. In this article, we present a first-of-its-kind system and study to collect simultaneous and continuous physiological data from both of the CAI interactants. Motivated by our extensive field reviews and stakeholder feedback, this comprehensive wearable system is composed of custom-designed and commercially available sensor devices. We performed a repeated-measures pilot study, to combine data collected via this system with a novel dyadic behavioral coding method and short- and long-term surveys. We evaluated these multimodal data streams independently, and we further correlated the psychological, physiological, and behavioral metrics to better elucidate the outcomes and dynamics of CAIs. Confirming previous field results, human electrodermal activity is the measure most strongly distinguished between the dyads’ non-interaction and interaction periods. Valence, arousal, and the positive affect of the human participant significantly increased during interaction with the canine participant. Also, we observed in our pilot study that (a) the canine heart rate was more dynamic than the human’s during interactions, (b) the surveys proved to be the best indicator of the subjects’ affective state, and (c) the behavior coding approaches best tracked the bond quality between the interacting dyads. Notably, we found that most of the interaction sessions were characterized by extended neutral periods with some positive and negative peaks, where the bonded pairs might display decreased behavioral synchrony. We also present three new representations of the internal and overall dynamics of CAIs for adoption by the broader field. Lastly, this paper discusses ongoing options for further dyadic analysis, interspecies emotion prediction, integration of contextually relevant environmental data, and standardization of human–animal interaction equipment and analytical approaches. Altogether, this work takes a significant step forward on a promising path to our better understanding of how CAIs improve well-being and how interspecies psychophysiological states can be appropriately measured. |
  | Date | 2024 |
  | Language | English |
  | Archive | Embase |
  | URL | https://www.embase.com/search/results?subaction=viewrecord&id=L2032752914&from=export |
  | Volume | 14 |
  | Publication | Animals |
  | DOI | 10.3390/ani14243628 |
  | Issue | 24 |
  | Journal Abbr | Animals |
  | ISSN | 2076-2615 |
  | Date Added | 11/07/2025, 11:53:17 |
  | Modified | 11/07/2025, 11:53:17 |

  ### Tags:

  - heart rate variability
  - heart rate
  - physical activity
  - dog
  - emotion
  - animal experiment
  - article
  - female
  - male
  - nonhuman
  - human
  - adult
  - data analysis software
  - pilot study
  - data analysis
  - animal model
  - wellbeing
  - electrodermal response
  - analytic method
  - behavioral synchrony
  - canine behavioral assessment and research questionnaire
  - clinical article
  - disease assessment
  - dynamics
  - electrocardiogram
  - general medical device
  - Microsoft Excel software
  - monash dog owner relationship scale
  - Positive and Negative Affect Schedule
  - prediction
  - psychophysiology
  - Python 3.7
  - self assessment mannikin
  - stakeholder participation
  - SySy harness device
  - wearable device
  - wearable technology
  - young adult

  ### Attachments

  - Full Text (HTML)
- ## Exploring the Dynamics of Canine-Assisted Interactions: A Wearable Approach to Understanding Interspecies Well-Being

  |  |  |
  | --- | --- |
  | Item Type | Journal Article |
  | Author | T.R.N. Holder |
  | Author | C. Nichols |
  | Author | E. Summers |
  | Author | D.L. Roberts |
  | Author | A. Bozkurt |
  | Abstract | Canine-assisted interactions (CAIs) have been explored to offer therapeutic benefits to human participants in various contexts, from addressing cancer-related fatigue to treating post-traumatic stress disorder. Despite their widespread adoption, there are still unresolved questions regarding the outcomes for both humans and animals involved in these interactions. Previous attempts to address these questions have suffered from core methodological weaknesses, especially due to absence of tools for an efficient objective evaluation and lack of focus on the canine perspective. In this article, we present a first-of-its-kind system and study to collect simultaneous and continuous physiological data from both of the CAI interactants. Motivated by our extensive field reviews and stakeholder feedback, this comprehensive wearable system is composed of custom-designed and commercially available sensor devices. We performed a repeated-measures pilot study, to combine data collected via this system with a novel dyadic behavioral coding method and short- and long-term surveys. We evaluated these multimodal data streams independently, and we further correlated the psychological, physiological, and behavioral metrics to better elucidate the outcomes and dynamics of CAIs. Confirming previous field results, human electrodermal activity is the measure most strongly distinguished between the dyads’ non-interaction and interaction periods. Valence, arousal, and the positive affect of the human participant significantly increased during interaction with the canine participant. Also, we observed in our pilot study that (a) the canine heart rate was more dynamic than the human’s during interactions, (b) the surveys proved to be the best indicator of the subjects’ affective state, and (c) the behavior coding approaches best tracked the bond quality between the interacting dyads. Notably, we found that most of the interaction sessions were characterized by extended neutral periods with some positive and negative peaks, where the bonded pairs might display decreased behavioral synchrony. We also present three new representations of the internal and overall dynamics of CAIs for adoption by the broader field. Lastly, this paper discusses ongoing options for further dyadic analysis, interspecies emotion prediction, integration of contextually relevant environmental data, and standardization of human–animal interaction equipment and analytical approaches. Altogether, this work takes a significant step forward on a promising path to our better understanding of how CAIs improve well-being and how interspecies psychophysiological states can be appropriately measured. |
  | Date | 2024 |
  | Language | English |
  | Archive | Embase |
  | URL | https://www.embase.com/search/results?subaction=viewrecord&id=L2032752914&from=export |
  | Volume | 14 |
  | Publication | Animals |
  | DOI | 10.3390/ani14243628 |
  | Issue | 24 |
  | Journal Abbr | Animals |
  | ISSN | 2076-2615 |
  | Date Added | 11/07/2025, 12:10:38 |
  | Modified | 11/07/2025, 12:10:38 |

  ### Tags:

  - heart rate variability
  - heart rate
  - physical activity
  - dog
  - emotion
  - animal experiment
  - article
  - female
  - male
  - nonhuman
  - human
  - adult
  - data analysis software
  - pilot study
  - data analysis
  - animal model
  - wellbeing
  - electrodermal response
  - analytic method
  - behavioral synchrony
  - canine behavioral assessment and research questionnaire
  - clinical article
  - disease assessment
  - dynamics
  - electrocardiogram
  - general medical device
  - Microsoft Excel software
  - monash dog owner relationship scale
  - Positive and Negative Affect Schedule
  - prediction
  - psychophysiology
  - Python 3.7
  - self assessment mannikin
  - stakeholder participation
  - SySy harness device
  - wearable device
  - wearable technology
  - young adult

  ### Attachments

  - Full Text (HTML)
- ## Long-term stress in dogs is related to the human-dog relationship and personality traits

  |  |  |
  | --- | --- |
  | Item Type | Journal Article |
  | Author | A. Höglin |
  | Author | E. Van Poucke |
  | Author | R. Katajamaa |
  | Author | P. Jensen |
  | Author | E. Theodorsson |
  | Author | L.S.V. Roth |
  | Abstract | Previously, we found that dogs belonging to the herding breed group, selected for human cooperation, synchronise their long-term stress levels with their owners. The aim of the current study was to investigate features that could influence long-term stress levels in ancient dog breeds, genetically closer to wolves, and dogs specifically selected to work independently of their owner. Twenty-four ancient breed dogs and 18 solitary hunting dogs were recruited and hair samples were obtained from both dogs and owners from which hair cortisol concentration (HCC) was analysed. Additionally, the owners completed lifestyle surveys, the Monash Dog Owner Relationship Scale (MDORS) on human-dog relationship, and both dog and owner personality questionnaires (Dog Personality questionnaire and Big Five Inventory survey). The results from the MDORS indicate that the subscale Perceived cost correlated to the dog HCC of tested breed groups: solitary hunting breeds (χ2 = 4.95, P = 0.026, β = 0.055), ancient breeds (χ2 = 2.74, P = 0.098, β = 0.027), and herding dogs included from a previous study (χ2 = 6.82, P = 0.009, β = - 0.061). The HCC of the solitary hunting dogs was also related to the owner personality traits Agreeableness (χ2 = 12.30, P < 0.001, β = - 0.060) and Openness (χ2 = 9.56, P = 0.002, β = 0.048) suggesting a more substantial influence of the owner on the solitary hunting dog's HCC compared to the ancient breeds. No effect of owner HCC on dog HCC was found in either ancient or in solitary hunting breeds. Hence, the long-term stress synchronisation is likely to be a trait in breeds selected for human cooperation. In conclusion, dog HCC is often related to the owners' personality, but is primarily influenced by the owner-dog relationship. |
  | Date | 2021 |
  | Language | English |
  | Archive | Medline |
  | URL | https://www.embase.com/search/results?subaction=viewrecord&id=L634864137&from=export |
  | Volume | 11 |
  | Pages | 8612 |
  | Publication | Scientific reports |
  | DOI | 10.1038/s41598-021-88201-y |
  | Issue | 1 |
  | Journal Abbr | Sci Rep |
  | ISSN | 2045-2322 |
  | Date Added | 11/07/2025, 14:16:53 |
  | Modified | 11/07/2025, 14:16:53 |

  ### Tags:

  - physiology
  - dog
  - animal behavior
  - female
  - male
  - human
  - hydrocortisone
  - questionnaire
  - animal
  - mental stress
  - psychology
  - human-animal bond
  - personality
  - breeding
  - procedures
  - pathophysiology
  - personality disorder

  ### Attachments

  - Full Text (HTML)
- ## Long-term stress in dogs is related to the human-dog relationship and personality traits

  |  |  |
  | --- | --- |
  | Item Type | Journal Article |
  | Author | A. Höglin |
  | Author | E. Van Poucke |
  | Author | R. Katajamaa |
  | Author | P. Jensen |
  | Author | E. Theodorsson |
  | Author | L.S.V. Roth |
  | Abstract | Previously, we found that dogs belonging to the herding breed group, selected for human cooperation, synchronise their long-term stress levels with their owners. The aim of the current study was to investigate features that could influence long-term stress levels in ancient dog breeds, genetically closer to wolves, and dogs specifically selected to work independently of their owner. Twenty-four ancient breed dogs and 18 solitary hunting dogs were recruited and hair samples were obtained from both dogs and owners from which hair cortisol concentration (HCC) was analysed. Additionally, the owners completed lifestyle surveys, the Monash Dog Owner Relationship Scale (MDORS) on human-dog relationship, and both dog and owner personality questionnaires (Dog Personality questionnaire and Big Five Inventory survey). The results from the MDORS indicate that the subscale Perceived cost correlated to the dog HCC of tested breed groups: solitary hunting breeds (χ2 = 4.95, P = 0.026, β = 0.055), ancient breeds (χ2 = 2.74, P = 0.098, β = 0.027), and herding dogs included from a previous study (χ2 = 6.82, P = 0.009, β = - 0.061). The HCC of the solitary hunting dogs was also related to the owner personality traits Agreeableness (χ2 = 12.30, P < 0.001, β = - 0.060) and Openness (χ2 = 9.56, P = 0.002, β = 0.048) suggesting a more substantial influence of the owner on the solitary hunting dog's HCC compared to the ancient breeds. No effect of owner HCC on dog HCC was found in either ancient or in solitary hunting breeds. Hence, the long-term stress synchronisation is likely to be a trait in breeds selected for human cooperation. In conclusion, dog HCC is often related to the owners' personality, but is primarily influenced by the owner-dog relationship. |
  | Date | 2021 |
  | Language | English |
  | Archive | Medline |
  | URL | https://www.embase.com/search/results?subaction=viewrecord&id=L634864137&from=export |
  | Volume | 11 |
  | Pages | 8612 |
  | Publication | Scientific reports |
  | DOI | 10.1038/s41598-021-88201-y |
  | Issue | 1 |
  | Journal Abbr | Sci Rep |
  | ISSN | 2045-2322 |
  | Date Added | 11/07/2025, 14:18:26 |
  | Modified | 11/07/2025, 14:18:26 |

  ### Tags:

  - physiology
  - dog
  - animal behavior
  - female
  - male
  - human
  - hydrocortisone
  - questionnaire
  - animal
  - mental stress
  - psychology
  - human-animal bond
  - personality
  - breeding
  - procedures
  - pathophysiology
  - personality disorder

  ### Attachments

  - Full Text (HTML)
- ## Investigating Dog Welfare When Interacting with Autistic Children within Canine-Assisted Occupational Therapy Sessions: A Single Case Study

  |  |  |
  | --- | --- |
  | Item Type | Journal Article |
  | Author | J. Hill |
  | Author | C. Driscoll |
  | Author | J. Cawdell-Smith |
  | Author | S. Anderson |
  | Author | J. Ziviani |
  | Abstract | Human-animal bond is defined as the mutually beneficial relationship between humans and animals. Recent years have seen increasing research regarding the benefits of interaction with animals for autistic children. However, there continue to be limited studies exploring the impact of this interaction on the welfare of therapy dogs. As part of a pilot randomised control trial assessing the efficacy of canine-assisted occupational therapy with autistic children, this project assessed welfare markers of the therapy dog involved. A total of twenty-one saliva samples were taken from the therapy dog to assess cortisol, alpha amylase, and oxytocin concentrations at home and throughout the treatment days. Additionally, six hours of therapy session videos were analysed for stress indicators of canine behaviour. No significant differences were found between days spent at home and treatment days for any of the biomarkers or stress indicators. Results suggest that the therapy dog involved did not experience increased stress resulting from interaction with the autistic children throughout the therapy sessions. This study supports the need for further research regarding therapy dog welfare when interacting with autistic children including an increased sample size of therapy dogs and therapists. |
  | Date | 2023 |
  | Language | English |
  | Archive | Embase |
  | URL | https://www.embase.com/search/results?subaction=viewrecord&id=L2024174492&from=export |
  | Volume | 13 |
  | Publication | Animals |
  | DOI | 10.3390/ani13121965 |
  | Issue | 12 |
  | Journal Abbr | Animals |
  | ISSN | 2076-2615 |
  | Date Added | 11/07/2025, 14:15:33 |
  | Modified | 11/07/2025, 14:15:33 |

  ### Tags:

  - oxytocin
  - animal welfare
  - saliva
  - child
  - animal behavior
  - animal experiment
  - article
  - female
  - male
  - nonhuman
  - human
  - hydrocortisone
  - data analysis software
  - ELISA kit
  - occupational therapy
  - autism
  - outcome assessment
  - videorecording
  - clinical article
  - human-animal bond
  - enzyme linked immunosorbent assay
  - SPSS
  - feeding behavior
  - amylase
  - sample size
  - comparative effectiveness
  - occupational therapist
  - single-case study
  - treatment response

  ### Attachments

  - Full Text (HTML)
- ## Investigating Dog Welfare When Interacting with Autistic Children within Canine-Assisted Occupational Therapy Sessions: A Single Case Study

  |  |  |
  | --- | --- |
  | Item Type | Journal Article |
  | Author | J. Hill |
  | Author | C. Driscoll |
  | Author | J. Cawdell-Smith |
  | Author | S. Anderson |
  | Author | J. Ziviani |
  | Abstract | Human-animal bond is defined as the mutually beneficial relationship between humans and animals. Recent years have seen increasing research regarding the benefits of interaction with animals for autistic children. However, there continue to be limited studies exploring the impact of this interaction on the welfare of therapy dogs. As part of a pilot randomised control trial assessing the efficacy of canine-assisted occupational therapy with autistic children, this project assessed welfare markers of the therapy dog involved. A total of twenty-one saliva samples were taken from the therapy dog to assess cortisol, alpha amylase, and oxytocin concentrations at home and throughout the treatment days. Additionally, six hours of therapy session videos were analysed for stress indicators of canine behaviour. No significant differences were found between days spent at home and treatment days for any of the biomarkers or stress indicators. Results suggest that the therapy dog involved did not experience increased stress resulting from interaction with the autistic children throughout the therapy sessions. This study supports the need for further research regarding therapy dog welfare when interacting with autistic children including an increased sample size of therapy dogs and therapists. |
  | Date | 2023 |
  | Language | English |
  | Archive | Embase |
  | URL | https://www.embase.com/search/results?subaction=viewrecord&id=L2024174492&from=export |
  | Volume | 13 |
  | Publication | Animals |
  | DOI | 10.3390/ani13121965 |
  | Issue | 12 |
  | Journal Abbr | Animals |
  | ISSN | 2076-2615 |
  | Date Added | 11/07/2025, 14:16:43 |
  | Modified | 11/07/2025, 14:16:43 |

  ### Tags:

  - oxytocin
  - animal welfare
  - saliva
  - child
  - animal behavior
  - animal experiment
  - article
  - female
  - male
  - nonhuman
  - human
  - hydrocortisone
  - data analysis software
  - ELISA kit
  - occupational therapy
  - autism
  - outcome assessment
  - videorecording
  - clinical article
  - human-animal bond
  - enzyme linked immunosorbent assay
  - SPSS
  - feeding behavior
  - amylase
  - sample size
  - comparative effectiveness
  - occupational therapist
  - single-case study
  - treatment response

  ### Attachments

  - Full Text (HTML)
- ## Investigating Dog Welfare When Interacting with Autistic Children within Canine-Assisted Occupational Therapy Sessions: A Single Case Study

  |  |  |
  | --- | --- |
  | Item Type | Journal Article |
  | Author | J. Hill |
  | Author | C. Driscoll |
  | Author | J. Cawdell-Smith |
  | Author | S. Anderson |
  | Author | J. Ziviani |
  | Abstract | Human-animal bond is defined as the mutually beneficial relationship between humans and animals. Recent years have seen increasing research regarding the benefits of interaction with animals for autistic children. However, there continue to be limited studies exploring the impact of this interaction on the welfare of therapy dogs. As part of a pilot randomised control trial assessing the efficacy of canine-assisted occupational therapy with autistic children, this project assessed welfare markers of the therapy dog involved. A total of twenty-one saliva samples were taken from the therapy dog to assess cortisol, alpha amylase, and oxytocin concentrations at home and throughout the treatment days. Additionally, six hours of therapy session videos were analysed for stress indicators of canine behaviour. No significant differences were found between days spent at home and treatment days for any of the biomarkers or stress indicators. Results suggest that the therapy dog involved did not experience increased stress resulting from interaction with the autistic children throughout the therapy sessions. This study supports the need for further research regarding therapy dog welfare when interacting with autistic children including an increased sample size of therapy dogs and therapists. |
  | Date | 2023 |
  | Language | English |
  | Archive | Embase |
  | URL | https://www.embase.com/search/results?subaction=viewrecord&id=L2024174492&from=export |
  | Volume | 13 |
  | Publication | Animals |
  | DOI | 10.3390/ani13121965 |
  | Issue | 12 |
  | Journal Abbr | Animals |
  | ISSN | 2076-2615 |
  | Date Added | 11/07/2025, 14:18:16 |
  | Modified | 11/07/2025, 14:18:16 |

  ### Tags:

  - oxytocin
  - animal welfare
  - saliva
  - child
  - animal behavior
  - animal experiment
  - article
  - female
  - male
  - nonhuman
  - human
  - hydrocortisone
  - data analysis software
  - ELISA kit
  - occupational therapy
  - autism
  - outcome assessment
  - videorecording
  - clinical article
  - human-animal bond
  - enzyme linked immunosorbent assay
  - SPSS
  - feeding behavior
  - amylase
  - sample size
  - comparative effectiveness
  - occupational therapist
  - single-case study
  - treatment response

  ### Attachments

  - Full Text (HTML)
- ## Oxytocin effects on the behavior in historical and experimental domesticated animals

  |  |  |
  | --- | --- |
  | Item Type | Journal Article |
  | Author | Y.E. Herbeck |
  | Author | A.A. Mаlyavko |
  | Author | D.V. Shepeleva |
  | Abstract | Domestication is one of the important evolutionary and genetic problems of biology. It addresses fundamental evolution aspects as well as allows us to study mechanisms of aggression, pro-social behavior and anthropogenesis. One of the potential key mechanisms of domestication may be associated with the central oxytocin (OT) system. OT has been repeatedly shown to exhibit pro-social and anxiolytic effects rodents, dogs, humans and other species [1]. In our study, we used intranasal OT administration on Siberian tame foxes and non-selected one. It is well-known experimental domestication model of. The OT effect on behavior had been investigated in intermale interactions (triple cage after adaptation) and human contact (new enclosure). OT administration increased patterns of playful (crouches on paws), affiliative (friendly) behavior, motor (fast move) and mixed activity in male-to-male interactions in tame foxes, whereas in case of unselected foxes, OT administration increased behavior patterns associated with anxiety (latent time for home cage exit, freezing, total time in home cage). In fox-human interactions, OT administration had the greatest effect on increasing the time tamed fox stayed at less than a meter away from a human. It is this parameter that is the defining one in similar tests on dogs. The results allow to suggest that OT has a context-dependent effect rather than explicitly pro-social effect in the tests, therefore amplifying the perception of social conditions according to the social salience hypothesis of OT. OT effect is associated with emotional positive and negative animal behaviors toward human that could be related to the genotype. Such OT effect might separate animals into more or less tolerant to humans under the same early domestication conditions. |
  | Date | 2022 |
  | Language | English |
  | Archive | Embase |
  | URL | https://www.embase.com/search/results?subaction=viewrecord&id=L2016787066&from=export |
  | Volume | 2022 |
  | Pages | 110 |
  | Publication | Siberian Medical Review |
  | DOI | 10.20333/25000136-2022-2-110 |
  | Issue | 2 |
  | Journal Abbr | Sib. Med. Rev. |
  | ISSN | 2500-0136 |
  | Date Added | 11/07/2025, 12:12:23 |
  | Modified | 11/07/2025, 12:12:23 |

  ### Tags:

  - oxytocin
  - human-animal interaction
  - behavior
  - dog
  - anxiety
  - social behavior
  - animal behavior
  - article
  - nonhuman
  - human
  - domestic animal
  - domestication
  - fox
  - cage
  - experimental animal
  - freezing
  - friendly behavior
  - genotype
  - historical period
  - intranasal drug administration
  - motor activity
  - negative valence
  - paw
  - playful behavior
  - positive valence
  - Siberian tame fox
  - social adaptation
  - social status
  - valence (emotion)

  ### Attachments

  - Full Text (HTML)
- ## Oxytocin effects on the behavior in historical and experimental domesticated animals

  |  |  |
  | --- | --- |
  | Item Type | Journal Article |
  | Author | Y.E. Herbeck |
  | Author | A.A. Mаlyavko |
  | Author | D.V. Shepeleva |
  | Abstract | Domestication is one of the important evolutionary and genetic problems of biology. It addresses fundamental evolution aspects as well as allows us to study mechanisms of aggression, pro-social behavior and anthropogenesis. One of the potential key mechanisms of domestication may be associated with the central oxytocin (OT) system. OT has been repeatedly shown to exhibit pro-social and anxiolytic effects rodents, dogs, humans and other species [1]. In our study, we used intranasal OT administration on Siberian tame foxes and non-selected one. It is well-known experimental domestication model of. The OT effect on behavior had been investigated in intermale interactions (triple cage after adaptation) and human contact (new enclosure). OT administration increased patterns of playful (crouches on paws), affiliative (friendly) behavior, motor (fast move) and mixed activity in male-to-male interactions in tame foxes, whereas in case of unselected foxes, OT administration increased behavior patterns associated with anxiety (latent time for home cage exit, freezing, total time in home cage). In fox-human interactions, OT administration had the greatest effect on increasing the time tamed fox stayed at less than a meter away from a human. It is this parameter that is the defining one in similar tests on dogs. The results allow to suggest that OT has a context-dependent effect rather than explicitly pro-social effect in the tests, therefore amplifying the perception of social conditions according to the social salience hypothesis of OT. OT effect is associated with emotional positive and negative animal behaviors toward human that could be related to the genotype. Such OT effect might separate animals into more or less tolerant to humans under the same early domestication conditions. |
  | Date | 2022 |
  | Language | English |
  | Archive | Embase |
  | URL | https://www.embase.com/search/results?subaction=viewrecord&id=L2016787066&from=export |
  | Volume | 2022 |
  | Pages | 110 |
  | Publication | Siberian Medical Review |
  | DOI | 10.20333/25000136-2022-2-110 |
  | Issue | 2 |
  | Journal Abbr | Sib. Med. Rev. |
  | ISSN | 2500-0136 |
  | Date Added | 11/07/2025, 12:13:25 |
  | Modified | 11/07/2025, 12:13:25 |

  ### Tags:

  - oxytocin
  - human-animal interaction
  - behavior
  - dog
  - anxiety
  - social behavior
  - animal behavior
  - article
  - nonhuman
  - human
  - domestic animal
  - domestication
  - fox
  - cage
  - experimental animal
  - freezing
  - friendly behavior
  - genotype
  - historical period
  - intranasal drug administration
  - motor activity
  - negative valence
  - paw
  - playful behavior
  - positive valence
  - Siberian tame fox
  - social adaptation
  - social status
  - valence (emotion)

  ### Attachments

  - Full Text (HTML)
- ## Relationships between handling, behaviour and stress in lambs at abattoirs

  |  |  |
  | --- | --- |
  | Item Type | Journal Article |
  | Author | P.H. Hemsworth |
  | Author | M. Rice |
  | Author | S. Borg |
  | Author | L.E. Edwards |
  | Author | E.N. Ponnampalam |
  | Author | G.J. Coleman |
  | Abstract | There is community concern about the treatment of farm animals post-farm gate, particularly animal transport and slaughter. Relationships between lamb behavioural and physiological variables on farm, stockperson, dog and lamb behavioural variables pre-slaughter and plasma cortisol, glucose and lactate in lambs post-slaughter were studied in 400 lambs. The lambs were observed in three behavioural tests, novel arena, flight distance to a human and temperament tests, before transport for slaughter. Closed-circuit television video footage was used to record stockperson, dog and lamb behaviour immediately before slaughter. Blood samples for cortisol, glucose and lactate analyses were collected on farm following the three behavioural tests and immediately post-slaughter. The regression models that best predicted plasma cortisol, glucose and lactate concentrations post-slaughter included a mixture of stockperson and dog behavioural variables as well as lamb variables both on-farm and pre-slaughter. These regression models accounted for 33%, 34% and 44% of the variance in plasma cortisol, glucose and lactate concentrations post-slaughter, respectively. Some of the stockperson and dog behaviours pre-slaughter that were predictive of the stress and metabolic variables post-slaughter included the duration of negative stockperson behaviours such as fast locomotion and lifting/pulling lambs, and the duration of dog behaviours such as lunging and barking at the lamb, while some of the predictive lamb behaviour variables included the durations of jumping and fleeing. Some of the physiological and behavioural responses to the behavioural tests on farm were also predictive of the stress and metabolic variables post-slaughter. These relationships support the well-demonstrated effect of handling on fear and stress responses in livestock, and although not direct evidence of causal relationships, highlight the potential benefits of training stockpeople to reduce fear and stress in sheep at abattoirs. |
  | Date | 2019 |
  | Language | English |
  | Archive | Medline |
  | URL | https://www.embase.com/search/results?subaction=viewrecord&id=L627911337&from=export |
  | Volume | 13 |
  | Pages | 1287-1296 |
  | Publication | Animal : an international journal of animal bioscience |
  | DOI | 10.1017/S1751731118002744 |
  | Issue | 6 |
  | Journal Abbr | Animal |
  | ISSN | 1751-732X |
  | Date Added | 11/07/2025, 14:18:33 |
  | Modified | 11/07/2025, 14:18:33 |

  ### Tags:

  - physiology
  - animal behavior
  - physiological stress
  - hydrocortisone
  - animal
  - blood
  - animal husbandry
  - slaughterhouse
  - analysis
  - domestic sheep
  - glucose blood level
  - lactic acid
  - Victoria

  ### Attachments

  - Full Text (HTML)
- ## Identification of QTLs for behavioral reactivity to social separation and humans in sheep using the OvineSNP50 BeadChip

  |  |  |
  | --- | --- |
  | Item Type | Journal Article |
  | Author | D. Hazard |
  | Author | C. Moreno |
  | Author | D. Foulquie´ |
  | Author | E. Delval |
  | Author | D. Franc¸ois |
  | Author | J. Bouix |
  | Author | G. Salle´ |
  | Author | A. Boissy |
  | Abstract | Background: Current trends in sheep farming practices rely on animals with a greater level of behavioral autonomy than before, a phenotype that actively contributes to the sustainability of animal production. Social reactivity and reactivity to humans are relevant behavioral traits in sheep, known for their strong gregariousness and weak tolerance to handling, which have previously been reported with moderate to high heritabilities. To identify loci underlying such behaviors, we performed a genome study in Romane lambs.Results: The experiment was carried out on 934 male and female lambs allocated into 9 half-sib families (average of 103 lambs per family) and reared outside. After weaning, all the lambs were individually exposed to 4 standardized behavioral tests combining social isolation, exposure to humans or handling, confinement and novelty (i.e. arena test, corridor test, isolation box test, shearing test). A broad range of behaviors including vocalizations, locomotion, vigilance and flight distance, as well as the cortisol response to handling, were collected. All lambs were genotyped using the Illumina OvineSNP50 BeadChip. QTL detection was performed by linkage, association and joint linkage and association analyses using the QTLmap software. Five main QTL regions were identified on sheep chromosomes (Ovis Aries Region, OAR) 12, 16, 19, 21 and 23 among many other QTLs with small to moderate effects. The QTLs on OAR12, 16 and 21 showed significant associations with social reactivity. The QTLs on OAR19 and 23 were found to be associated with reactivity to humans. No overlapping QTLs were identified for the different traits measured in the behavioral tests, supporting the hypothesis that different genetic factors influence social reactivity and tolerance to humans.Conclusion: The results of this study using ovine SNP data suggest that in domestic sheep the behavioral responses to social separation and exposure to humans are under polygenic influence. The most relevant QTLs reported in the present study contain interesting candidate genes previously described to be associated with various emotional and social behaviors in mammals. |
  | Date | 2014 |
  | Language | English |
  | Archive | Embase |
  | URL | https://www.embase.com/search/results?subaction=viewrecord&id=L600035937&from=export |
  | Volume | 15 |
  | Publication | BMC Genomics |
  | DOI | 10.1186/1471-2164-15-778 |
  | Issue | 1 |
  | Journal Abbr | BMC Genomics |
  | ISSN | 1471-2164 |
  | Date Added | 11/07/2025, 14:18:42 |
  | Modified | 11/07/2025, 14:18:42 |

  ### Tags:

  - emotion
  - social behavior
  - animal behavior
  - animal experiment
  - article
  - female
  - locomotion
  - male
  - nonhuman
  - controlled study
  - social isolation
  - human-animal bond
  - experimental behavioral test
  - genetic variability
  - genetic association
  - vocalization
  - domestic sheep
  - alertness
  - arena test
  - behavior genetics
  - behavioral reactivity
  - chromosome
  - chromosome 12
  - chromosome 16
  - chromosome 19
  - chromosome 21
  - chromosome 23
  - chromosome analysis
  - corridor test
  - environmental exposure
  - gene identification
  - genetic linkage
  - genome analysis
  - isolation box test
  - mammalian genetics
  - quantitative trait locus
  - quantitative trait locus mapping
  - shearing test
  - sheep farming
  - single nucleotide polymorphism

  ### Attachments

  - Full Text (HTML)
- ## Positive human contact and housing systems impact the responses of piglets to various stressors

  |  |  |
  | --- | --- |
  | Item Type | Journal Article |
  | Author | M.E. Hayes |
  | Author | L.M. Hemsworth |
  | Author | R.S. Morrison |
  | Author | A.J. Tilbrook |
  | Author | P.H. Hemsworth |
  | Abstract | This experiment studied the effects of lactation housing systems and human interaction on piglets’ responses to routine stressors. Forty litters of piglets were reared in either a standard farrowing crate (FC) or a loose farrowing and lactation pen (LP; PigSAFE pen) and received either routine contact with humans (C) or regular opportunities for positive human contact (+HC; 3 min of patting, stroking and scratching 5 times/week). Behavioural and physiological responses to routine husbandry procedures, weaning, novelty and humans were studied in addition to effects on piglet growth, injuries and survival. Compared to C piglets, +HC piglets vocalised for shorter durations (p = 0.018) during husbandry procedures and showed a lower intensity of escape behaviour during iron injection (p = 0.042) and oral vaccination (p = 0.026) at 3 d of age, capture at 2 wk of age (p < 0.001), and intramuscular vaccination (p = 0.005) at 3 wk of age. +HC piglets at 2 wk of age were faster than C piglets to approach (p = 0.048) and interact (p = 0.042) with a stationary unfamiliar human. Compared to LP piglets, FC piglets showed a lower intensity of escape behaviour during capture and iron administration by a stockperson at 3 d of age (p = 0.043). FC piglets at 2 wk of age were faster than LP piglets to approach (p = 0.005) and interact (p = 0.027) with a novel object and approach (p = 0.009) and interact (p = 0.008) with an unfamiliar human. FC piglets had fewer injuries than LP piglets at 2 wk of age (p = 0.004). +HC pigs had fewer injuries than C pigs after weaning (p = 0.003). After weaning there were more pigs from LP than FC observed to be upright (both stationary, p = 0.002 and walking, p = 0.024), vocalizing (p = 0.004), nosing another pig (p = 0.035) and nosing the pen floor (p = 0.038). There were no significant effects on neutrophil:lymphocyte ratios or plasma cortisol concentrations 1.5 h after weaning. However, 25 h after weaning +HC pigs had higher haptoglobin concentrations than C pigs (p = 0.002), and C/LP pigs had higher cortisol concentrations than +HC/LP and C/FC pigs (p = 0.012). There were no significant effects on piglet growth, the number of piglets born alive or the number stillborn, however there were more piglets weaned from FC than LP (p = 0.035). The results from this experiment raise questions that require further research on the ability of pigs reared in loose pens to cope with stressors such as exposure to humans, novelty, husbandry procedures and weaning. This experiment also provides evidence that regular positive human interaction reduces pigs’ fear of humans and husbandry procedures imposed by stockpeople. More research is required to determine if any of these effects are sustained long-term. |
  | Date | 2021 |
  | Language | English |
  | Archive | Embase |
  | URL | https://www.embase.com/search/results?subaction=viewrecord&id=L2007360312&from=export |
  | Volume | 11 |
  | Publication | Animals |
  | DOI | 10.3390/ani11061619 |
  | Issue | 6 |
  | Journal Abbr | Animals |
  | ISSN | 2076-2615 |
  | Date Added | 11/07/2025, 14:16:53 |
  | Modified | 11/07/2025, 14:16:53 |

  ### Tags:

  - human-animal interaction
  - animal behavior
  - animal experiment
  - article
  - female
  - male
  - nonhuman
  - physiological stress
  - human
  - human experiment
  - lactation
  - weaning
  - fear
  - enzyme linked immunosorbent assay
  - animal husbandry
  - animal housing
  - hydrocortisone blood level
  - walking
  - vaccination
  - haptoglobin
  - neutrophil lymphocyte ratio
  - scratching
  - lymphocyte count
  - reproductive success
  - sow (swine)
  - stillbirth
  - survival

  ### Attachments

  - Full Text (HTML)
- ## Effects of positive human contact during gestation on the behaviour, physiology and reproductive performance of sows

  |  |  |
  | --- | --- |
  | Item Type | Journal Article |
  | Author | M.E. Hayes |
  | Author | L.M. Hemsworth |
  | Author | R.S. Morrison |
  | Author | K.L. Butler |
  | Author | M. Rice |
  | Author | J.-L. Rault |
  | Author | P.H. Hemsworth |
  | Abstract | Previous positive interactions with humans may ameliorate the stress response of farm animals to aversive routine practices such as painful or stressful procedures, particularly those associated with stockpeople. We studied the effects of positive handling by providing younger (parity 1–2) and older (parity 3–8) sows housed in pens of fifteen (n = 24 pens in total) with either positive human contact (+HC) or routine human contact (control) during gestation. The +HC treatment involved a familiar stockperson patting and scratching sows and was imposed at a pen-level for 2 min daily. Measurements studied included behavioural, physiological and productivity variables. The +HC sows showed reduced avoidance of the stockperson conducting pregnancy testing and vaccination in the home pens, however the behavioural and cortisol responses of sows in a standard unfamiliar human approach test did not differ. There were no effects of +HC on aggression between sows, serum cortisol or serum brain-derived neurotrophic factor concentrations during gestation, or on the behavioural and cortisol response to being moved to farrowing crates. There were also no effects of +HC on the maternal responsiveness of sows, farrowing rate or the number of piglets born alive, stillborn or weaned. Sows in the +HC pens reduced their physical interaction with the stockpeople imposing the treatment after 2 weeks, which suggests the sows may have habituated to the novel or possible rewarding elements of the handling treatment. This experiment shows that regular positive interaction with stockpeople does reduce sows’ fear of stockpeople, but does not always confer stress resilence. |
  | Date | 2021 |
  | Language | English |
  | Archive | Embase |
  | URL | https://www.embase.com/search/results?subaction=viewrecord&id=L2005826171&from=export |
  | Volume | 11 |
  | Pages | 1-16 |
  | Publication | Animals |
  | DOI | 10.3390/ani11010214 |
  | Issue | 1 |
  | Journal Abbr | Animals |
  | ISSN | 2076-2615 |
  | Date Added | 11/07/2025, 14:16:54 |
  | Modified | 11/07/2025, 14:16:54 |

  ### Tags:

  - human-animal interaction
  - animal welfare
  - physiology
  - animal experiment
  - article
  - female
  - male
  - nonhuman
  - physiological stress
  - human
  - hydrocortisone
  - adult
  - controlled study
  - human experiment
  - lactation
  - fear
  - enzyme linked immunosorbent assay
  - maternal behavior
  - animal husbandry
  - wildlife
  - feeding
  - hydrocortisone blood level
  - aggression
  - vaccination
  - histology
  - brain derived neurotrophic factor
  - feces analysis
  - scratching
  - reproductive success
  - sow (swine)
  - avoidance behavior
  - insemination
  - parity
  - piglet
  - pregnancy
  - productivity
  - psychological resilience
  - telecommunication
  - third trimester pregnancy
  - valine
  - vomitoxin

  ### Attachments

  - Full Text (HTML)
- ## Positive human contact and housing systems impact the responses of piglets to various stressors

  |  |  |
  | --- | --- |
  | Item Type | Journal Article |
  | Author | M.E. Hayes |
  | Author | L.M. Hemsworth |
  | Author | R.S. Morrison |
  | Author | A.J. Tilbrook |
  | Author | P.H. Hemsworth |
  | Abstract | This experiment studied the effects of lactation housing systems and human interaction on piglets’ responses to routine stressors. Forty litters of piglets were reared in either a standard farrowing crate (FC) or a loose farrowing and lactation pen (LP; PigSAFE pen) and received either routine contact with humans (C) or regular opportunities for positive human contact (+HC; 3 min of patting, stroking and scratching 5 times/week). Behavioural and physiological responses to routine husbandry procedures, weaning, novelty and humans were studied in addition to effects on piglet growth, injuries and survival. Compared to C piglets, +HC piglets vocalised for shorter durations (p = 0.018) during husbandry procedures and showed a lower intensity of escape behaviour during iron injection (p = 0.042) and oral vaccination (p = 0.026) at 3 d of age, capture at 2 wk of age (p < 0.001), and intramuscular vaccination (p = 0.005) at 3 wk of age. +HC piglets at 2 wk of age were faster than C piglets to approach (p = 0.048) and interact (p = 0.042) with a stationary unfamiliar human. Compared to LP piglets, FC piglets showed a lower intensity of escape behaviour during capture and iron administration by a stockperson at 3 d of age (p = 0.043). FC piglets at 2 wk of age were faster than LP piglets to approach (p = 0.005) and interact (p = 0.027) with a novel object and approach (p = 0.009) and interact (p = 0.008) with an unfamiliar human. FC piglets had fewer injuries than LP piglets at 2 wk of age (p = 0.004). +HC pigs had fewer injuries than C pigs after weaning (p = 0.003). After weaning there were more pigs from LP than FC observed to be upright (both stationary, p = 0.002 and walking, p = 0.024), vocalizing (p = 0.004), nosing another pig (p = 0.035) and nosing the pen floor (p = 0.038). There were no significant effects on neutrophil:lymphocyte ratios or plasma cortisol concentrations 1.5 h after weaning. However, 25 h after weaning +HC pigs had higher haptoglobin concentrations than C pigs (p = 0.002), and C/LP pigs had higher cortisol concentrations than +HC/LP and C/FC pigs (p = 0.012). There were no significant effects on piglet growth, the number of piglets born alive or the number stillborn, however there were more piglets weaned from FC than LP (p = 0.035). The results from this experiment raise questions that require further research on the ability of pigs reared in loose pens to cope with stressors such as exposure to humans, novelty, husbandry procedures and weaning. This experiment also provides evidence that regular positive human interaction reduces pigs’ fear of humans and husbandry procedures imposed by stockpeople. More research is required to determine if any of these effects are sustained long-term. |
  | Date | 2021 |
  | Language | English |
  | Archive | Embase |
  | URL | https://www.embase.com/search/results?subaction=viewrecord&id=L2007360312&from=export |
  | Volume | 11 |
  | Publication | Animals |
  | DOI | 10.3390/ani11061619 |
  | Issue | 6 |
  | Journal Abbr | Animals |
  | ISSN | 2076-2615 |
  | Date Added | 11/07/2025, 14:18:26 |
  | Modified | 11/07/2025, 14:18:26 |

  ### Tags:

  - human-animal interaction
  - animal behavior
  - animal experiment
  - article
  - female
  - male
  - nonhuman
  - physiological stress
  - human
  - human experiment
  - lactation
  - weaning
  - fear
  - enzyme linked immunosorbent assay
  - animal husbandry
  - animal housing
  - hydrocortisone blood level
  - walking
  - vaccination
  - haptoglobin
  - neutrophil lymphocyte ratio
  - scratching
  - lymphocyte count
  - reproductive success
  - sow (swine)
  - stillbirth
  - survival

  ### Attachments

  - Full Text (HTML)
- ## Effects of positive human contact during gestation on the behaviour, physiology and reproductive performance of sows

  |  |  |
  | --- | --- |
  | Item Type | Journal Article |
  | Author | M.E. Hayes |
  | Author | L.M. Hemsworth |
  | Author | R.S. Morrison |
  | Author | K.L. Butler |
  | Author | M. Rice |
  | Author | J.-L. Rault |
  | Author | P.H. Hemsworth |
  | Abstract | Previous positive interactions with humans may ameliorate the stress response of farm animals to aversive routine practices such as painful or stressful procedures, particularly those associated with stockpeople. We studied the effects of positive handling by providing younger (parity 1–2) and older (parity 3–8) sows housed in pens of fifteen (n = 24 pens in total) with either positive human contact (+HC) or routine human contact (control) during gestation. The +HC treatment involved a familiar stockperson patting and scratching sows and was imposed at a pen-level for 2 min daily. Measurements studied included behavioural, physiological and productivity variables. The +HC sows showed reduced avoidance of the stockperson conducting pregnancy testing and vaccination in the home pens, however the behavioural and cortisol responses of sows in a standard unfamiliar human approach test did not differ. There were no effects of +HC on aggression between sows, serum cortisol or serum brain-derived neurotrophic factor concentrations during gestation, or on the behavioural and cortisol response to being moved to farrowing crates. There were also no effects of +HC on the maternal responsiveness of sows, farrowing rate or the number of piglets born alive, stillborn or weaned. Sows in the +HC pens reduced their physical interaction with the stockpeople imposing the treatment after 2 weeks, which suggests the sows may have habituated to the novel or possible rewarding elements of the handling treatment. This experiment shows that regular positive interaction with stockpeople does reduce sows’ fear of stockpeople, but does not always confer stress resilence. |
  | Date | 2021 |
  | Language | English |
  | Archive | Embase |
  | URL | https://www.embase.com/search/results?subaction=viewrecord&id=L2005826171&from=export |
  | Volume | 11 |
  | Pages | 1-16 |
  | Publication | Animals |
  | DOI | 10.3390/ani11010214 |
  | Issue | 1 |
  | Journal Abbr | Animals |
  | ISSN | 2076-2615 |
  | Date Added | 11/07/2025, 14:18:28 |
  | Modified | 11/07/2025, 14:18:28 |

  ### Tags:

  - human-animal interaction
  - animal welfare
  - physiology
  - animal experiment
  - article
  - female
  - male
  - nonhuman
  - physiological stress
  - human
  - hydrocortisone
  - adult
  - controlled study
  - human experiment
  - lactation
  - fear
  - enzyme linked immunosorbent assay
  - maternal behavior
  - animal husbandry
  - wildlife
  - feeding
  - hydrocortisone blood level
  - aggression
  - vaccination
  - histology
  - brain derived neurotrophic factor
  - feces analysis
  - scratching
  - reproductive success
  - sow (swine)
  - avoidance behavior
  - insemination
  - parity
  - piglet
  - pregnancy
  - productivity
  - psychological resilience
  - telecommunication
  - third trimester pregnancy
  - valine
  - vomitoxin

  ### Attachments

  - Full Text (HTML)
- ## Does stress run through the leash? An examination of stress transmission between owners and dogs during a walk

  |  |  |
  | --- | --- |
  | Item Type | Journal Article |
  | Author | Helen Harvie |
  | Author | Alejandro Rodrigo |
  | Author | Candace Briggs |
  | Author | Shane Thiessen |
  | Author | Debbie M. Kelly |
  | Date | 03/2021 |
  | Language | en |
  | Short Title | Does stress run through the leash? |
  | Library Catalogue | DOI.org (Crossref) |
  | URL | https://link.springer.com/10.1007/s10071-020-01460-6 |
  | Accessed | 27/06/2025, 16:32:56 |
  | Volume | 24 |
  | Pages | 239-250 |
  | Publication | Animal Cognition |
  | DOI | 10.1007/s10071-020-01460-6 |
  | Issue | 2 |
  | Journal Abbr | Anim Cogn |
  | ISSN | 1435-9448, 1435-9456 |
  | Date Added | 27/06/2025, 16:32:56 |
  | Modified | 27/06/2025, 16:32:56 |

  ### Attachments

  - PDF
- ## Does stress run through the leash? An examination of stress transmission between owners and dogs during a walk

  |  |  |
  | --- | --- |
  | Item Type | Journal Article |
  | Author | H. Harvie |
  | Author | A. Rodrigo |
  | Author | C. Briggs |
  | Author | S. Thiessen |
  | Author | D.M. Kelly |
  | Abstract | Humans and dogs have co-evolved for over 10,000 years. Recent research suggests that, through the domestication process, dogs have become proficient at responding to human commands, attention and emotional states. However, the extent to which a companion dog responds to human emotions, such as stress, remains to be understood. This study examines whether a companion dog's stress, as measured by cortisol levels and heart rate, increases during a familiar outdoor walk in response to its owner's experience of stress. Sixty-eight owner/dog dyads participated in this study. The dyads were randomly assigned to an Experimental or Control group. Owners in the Experimental group were informed the walk would be digitally recorded for subsequent evaluation of their handling skills, whereas those in the Control group were informed the walk would be digitally recorded for archival purposes (no evaluation). This manipulation was implemented to induce a mild stress response in the owners. Salivary cortisol samples were collected from the owner and their dog before and after the walk. The dyad was also fitted with monitoring devices to record heart rate throughout the walk. Finally, personality information regarding the owner and their dog was collected. We found that cortisol production within the dyad showed a marginal inverse correlation. We also found that owners' Openness to Experience and dogs' Fearfulness influenced the heart rate of the other during the first minute of a walk. These results support that although stress may be detected within a dyad, this does not result in an associated significant change in cortisol or heart rate. |
  | Date | 2021 |
  | Language | English |
  | Archive | Medline |
  | URL | https://www.embase.com/search/results?subaction=viewrecord&id=L633921862&from=export |
  | Volume | 24 |
  | Pages | 239-250 |
  | Publication | Animal cognition |
  | DOI | 10.1007/s10071-020-01460-6 |
  | Issue | 2 |
  | Journal Abbr | Anim Cogn |
  | ISSN | 1435-9456 |
  | Date Added | 11/07/2025, 14:16:53 |
  | Modified | 11/07/2025, 14:16:53 |

  ### Tags:

  - dog
  - emotion
  - pet animal
  - animal
  - human-animal bond
  - personality
  - attention

  ### Attachments

  - Full Text (HTML)
- ## Does stress run through the leash? An examination of stress transmission between owners and dogs during a walk

  |  |  |
  | --- | --- |
  | Item Type | Journal Article |
  | Author | H. Harvie |
  | Author | A. Rodrigo |
  | Author | C. Briggs |
  | Author | S. Thiessen |
  | Author | D.M. Kelly |
  | Abstract | Humans and dogs have co-evolved for over 10,000 years. Recent research suggests that, through the domestication process, dogs have become proficient at responding to human commands, attention and emotional states. However, the extent to which a companion dog responds to human emotions, such as stress, remains to be understood. This study examines whether a companion dog's stress, as measured by cortisol levels and heart rate, increases during a familiar outdoor walk in response to its owner's experience of stress. Sixty-eight owner/dog dyads participated in this study. The dyads were randomly assigned to an Experimental or Control group. Owners in the Experimental group were informed the walk would be digitally recorded for subsequent evaluation of their handling skills, whereas those in the Control group were informed the walk would be digitally recorded for archival purposes (no evaluation). This manipulation was implemented to induce a mild stress response in the owners. Salivary cortisol samples were collected from the owner and their dog before and after the walk. The dyad was also fitted with monitoring devices to record heart rate throughout the walk. Finally, personality information regarding the owner and their dog was collected. We found that cortisol production within the dyad showed a marginal inverse correlation. We also found that owners' Openness to Experience and dogs' Fearfulness influenced the heart rate of the other during the first minute of a walk. These results support that although stress may be detected within a dyad, this does not result in an associated significant change in cortisol or heart rate. |
  | Date | 2021 |
  | Language | English |
  | Archive | Medline |
  | URL | https://www.embase.com/search/results?subaction=viewrecord&id=L633921862&from=export |
  | Volume | 24 |
  | Pages | 239-250 |
  | Publication | Animal cognition |
  | DOI | 10.1007/s10071-020-01460-6 |
  | Issue | 2 |
  | Journal Abbr | Anim Cogn |
  | ISSN | 1435-9456 |
  | Date Added | 11/07/2025, 14:18:27 |
  | Modified | 11/07/2025, 14:18:27 |

  ### Tags:

  - dog
  - emotion
  - pet animal
  - animal
  - human-animal bond
  - personality
  - attention

  ### Attachments

  - Full Text (HTML)
- ## The Influence of Brief Outing and Temporary Fostering Programs on Shelter Dog Welfare

  |  |  |
  | --- | --- |
  | Item Type | Journal Article |
  | Author | L.M. Gunter |
  | Author | E.M. Blade |
  | Author | R.J. Gilchrist |
  | Author | B.J. Nixon |
  | Author | J.L. Reed |
  | Author | J.M. Platzer |
  | Author | I.C. Wurpts |
  | Author | E.N. Feuerbacher |
  | Author | C.D.L. Wynne |
  | Abstract | Human interaction is one of the most consistently effective interventions that can improve the welfare of shelter-living dogs. Time out of the kennel with a person has been shown to reduce physiological measures of stress as can leaving the shelter for a night or more in a foster home. In this study, we assessed the effects of brief outings and temporary fostering stays on dogs’ length of stay and outcomes. In total, we analyzed data of 1955 dogs from 51 animal shelters that received these interventions as well as 25,946 dogs residing at these shelters that served as our controls. We found that brief outings and temporary fostering stays increased dogs’ likelihood of adoption by 5.0 and 14.3 times, respectively. While their lengths of stay were longer in comparison to control dogs, this difference was present prior to the intervention. Additionally, we found that these programs were more successful when greater percentages of community members (as compared to volunteers and staff) were involved in caregiving as well as when programs were implemented by better-resourced shelters. As such, animal welfare organizations should consider implementing these fostering programs as evidence-based best practices that can positively impact the outcomes of shelter dogs. |
  | Date | 2023 |
  | Language | English |
  | Archive | Embase |
  | URL | https://www.embase.com/search/results?subaction=viewrecord&id=L2026784029&from=export |
  | Volume | 13 |
  | Publication | Animals |
  | DOI | 10.3390/ani13223528 |
  | Issue | 22 |
  | Journal Abbr | Animals |
  | ISSN | 2076-2615 |
  | Date Added | 11/07/2025, 11:48:34 |
  | Modified | 11/07/2025, 11:48:34 |

  ### Tags:

  - human-animal interaction
  - animal welfare
  - dog
  - article
  - male
  - nonhuman
  - physiological stress
  - human
  - controlled study
  - care behavior
  - evidence based practice
  - foster care
  - length of stay

  ### Attachments

  - Full Text (HTML)
- ## Investigating the impact of brief outings on the welfare of dogs living in us shelters

  |  |  |
  | --- | --- |
  | Item Type | Journal Article |
  | Author | L.M. Gunter |
  | Author | R.J. Gilchrist |
  | Author | E.M. Blade |
  | Author | R.T. Barber |
  | Author | E.N. Feuerbacher |
  | Author | J.M. Platzer |
  | Author | C.D.L. Wynne |
  | Abstract | Social isolation likely contributes to reduced welfare for shelter‐living dogs. Several studies have established that time out of the kennel with a person can improve dogs’ behavior and reduce physiological measures of stress. This study assessed the effects of two‐and‐a‐half‐hour outings on the urinary cortisol levels and activity of dogs as they awaited adoption at four animal shelters. Dogs’ urine was collected before and after outings for cortisol:creatinine analysis, and accelerometer devices were used to measure dogs’ physical activity. In total, 164 dogs participated in this study, with 793 cortisol values and 3750 activity measures used in the statistical analyses. We found that dogs’ cortisol:creatinine ratios were significantly higher during the afternoon of the intervention but returned to pre‐field trip levels the following day. Dogs’ minutes of low activity were significantly reduced, and high activity significantly increased during the outing. Although dogs’ cortisol and activity returned to baseline after the intervention, our findings suggest that short‐term outings do not confer the same stress reduction benefits as previously shown with temporary fostering. Nevertheless, it is possible that these types of outing programs are beneficial to adoptions by increasing the visibility of dogs and should be further investigated to elucidate these effects. |
  | Date | 2021 |
  | Language | English |
  | Archive | Embase |
  | URL | https://www.embase.com/search/results?subaction=viewrecord&id=L2006063987&from=export |
  | Volume | 11 |
  | Pages | 1-15 |
  | Publication | Animals |
  | DOI | 10.3390/ani11020548 |
  | Issue | 2 |
  | Journal Abbr | Animals |
  | ISSN | 2076-2615 |
  | Date Added | 11/07/2025, 11:48:35 |
  | Modified | 11/07/2025, 11:48:35 |

  ### Tags:

  - human-animal interaction
  - animal welfare
  - accelerometer
  - dog
  - animal experiment
  - article
  - nonhuman
  - physiological stress
  - hydrocortisone
  - controlled study
  - length of stay
  - animal food
  - autoanalyzer
  - bacterium isolation
  - chemiluminescence immunoassay
  - corticotropin
  - creatinine
  - data analysis
  - geographic distribution
  - hospitalization
  - hydrocortisone urine level
  - information processing
  - social isolation
  - United States
  - urine sampling
  - water

  ### Attachments

  - Full Text (HTML)
- ## Evaluating the effects of a temporary fostering program on shelter dog welfare

  |  |  |
  | --- | --- |
  | Item Type | Journal Article |
  | Author | L.M. Gunter |
  | Author | E.N. Feuerbacher |
  | Author | R.J. Gilchrist |
  | Author | C.D.L. Wynne |
  | Abstract | One of the greatest stressors for dogs living in animal shelters is social isolation. Many studies have demonstrated that human interaction reduces cortisol in shelter dogs, with the possibility that longer periods of interaction may yield greater effects. These types of interventions are contingent upon removing the dog from the kennel and any such reductions in cortisol are often lost when the dog returns to the kennel. More recently, animal shelters are utilizing short-term fostering programs to provide relief from the perceived stresses of kennel life; however the effects of these programs are not well understood. This study assessed the impacts of one- and two-night fostering programs on the urinary cortisol levels, resting pulse rates, longest bout of uninterrupted rest, and proportion of time spent resting of dogs awaiting adoption. Five animal shelters, open and limited-admission facilities, from across the United States participated in the study. During the study, dogs' urine was collected in the morning before, during, and after fostering stays for cortisol: Creatinine analysis. Non-invasive health monitors were worn by the dogs, which collected heart rates and activity levels, in the shelter and in foster homes. In total, 207 dogs participated in the study, and 1,076 cortisol values were used in our analysis. Across all shelters, we found that dogs' cortisol: Creatinine ratios dropped significantly during their fostering stay, but returned to baseline levels after return to the shelter. However, the observed reduction in cortisol varied in magnitude across shelters. We found that dogs of greater weight, age, and average resting pulse rate had higher cortisol levels; and dogs with longer bouts of uninterrupted rest had lower cortisol levels. Dogs had their longest bouts of rest during sleepovers, followed by in the shelter after their sleepovers. Lastly, significant differences were found when comparing in-shelter cortisol values at our five shelters, differences that were in some cases greater than the impact of the fostering intervention itself. Considering the diversity of facilities that participated in this study, it is possible that as yet unstudied, shelter-specific, environmental factors could be contributing to the overall welfare of shelter dogs. Thus while a reprieve from the shelter is impactful for dogs awaiting adoption, mitigating the stressors present in kenneling conditions should also be addressed to improve the lives of shelter dogs. |
  | Date | 2019 |
  | Language | English |
  | Archive | Embase |
  | URL | https://www.embase.com/search/results?subaction=viewrecord&id=L626944929&from=export |
  | Volume | 2019 |
  | Publication | PeerJ |
  | DOI | 10.7717/peerj.6620 |
  | Issue | 3 |
  | Journal Abbr | PeerJ |
  | ISSN | 2167-8359 |
  | Date Added | 11/07/2025, 12:10:54 |
  | Modified | 11/07/2025, 12:10:54 |

  ### Tags:

  - animal welfare
  - heart rate
  - physical activity
  - dog
  - article
  - nonhuman
  - hydrocortisone
  - body weight
  - length of stay
  - autoanalyzer
  - creatinine
  - social isolation
  - urine sampling
  - body temperature monitoring
  - breathing
  - community care
  - pulse rate
  - sleep

  ### Attachments

  - Full Text (HTML)
- ## Investigating the impact of brief outings on the welfare of dogs living in us shelters

  |  |  |
  | --- | --- |
  | Item Type | Journal Article |
  | Author | L.M. Gunter |
  | Author | R.J. Gilchrist |
  | Author | E.M. Blade |
  | Author | R.T. Barber |
  | Author | E.N. Feuerbacher |
  | Author | J.M. Platzer |
  | Author | C.D.L. Wynne |
  | Abstract | Social isolation likely contributes to reduced welfare for shelter‐living dogs. Several studies have established that time out of the kennel with a person can improve dogs’ behavior and reduce physiological measures of stress. This study assessed the effects of two‐and‐a‐half‐hour outings on the urinary cortisol levels and activity of dogs as they awaited adoption at four animal shelters. Dogs’ urine was collected before and after outings for cortisol:creatinine analysis, and accelerometer devices were used to measure dogs’ physical activity. In total, 164 dogs participated in this study, with 793 cortisol values and 3750 activity measures used in the statistical analyses. We found that dogs’ cortisol:creatinine ratios were significantly higher during the afternoon of the intervention but returned to pre‐field trip levels the following day. Dogs’ minutes of low activity were significantly reduced, and high activity significantly increased during the outing. Although dogs’ cortisol and activity returned to baseline after the intervention, our findings suggest that short‐term outings do not confer the same stress reduction benefits as previously shown with temporary fostering. Nevertheless, it is possible that these types of outing programs are beneficial to adoptions by increasing the visibility of dogs and should be further investigated to elucidate these effects. |
  | Date | 2021 |
  | Language | English |
  | Archive | Embase |
  | URL | https://www.embase.com/search/results?subaction=viewrecord&id=L2006063987&from=export |
  | Volume | 11 |
  | Pages | 1-15 |
  | Publication | Animals |
  | DOI | 10.3390/ani11020548 |
  | Issue | 2 |
  | Journal Abbr | Animals |
  | ISSN | 2076-2615 |
  | Date Added | 11/07/2025, 14:16:54 |
  | Modified | 11/07/2025, 14:16:54 |

  ### Tags:

  - human-animal interaction
  - animal welfare
  - accelerometer
  - dog
  - animal experiment
  - article
  - nonhuman
  - physiological stress
  - hydrocortisone
  - controlled study
  - length of stay
  - animal food
  - autoanalyzer
  - bacterium isolation
  - chemiluminescence immunoassay
  - corticotropin
  - creatinine
  - data analysis
  - geographic distribution
  - hospitalization
  - hydrocortisone urine level
  - information processing
  - social isolation
  - United States
  - urine sampling
  - water

  ### Attachments

  - Full Text (HTML)
- ## Investigating the impact of brief outings on the welfare of dogs living in us shelters

  |  |  |
  | --- | --- |
  | Item Type | Journal Article |
  | Author | L.M. Gunter |
  | Author | R.J. Gilchrist |
  | Author | E.M. Blade |
  | Author | R.T. Barber |
  | Author | E.N. Feuerbacher |
  | Author | J.M. Platzer |
  | Author | C.D.L. Wynne |
  | Abstract | Social isolation likely contributes to reduced welfare for shelter‐living dogs. Several studies have established that time out of the kennel with a person can improve dogs’ behavior and reduce physiological measures of stress. This study assessed the effects of two‐and‐a‐half‐hour outings on the urinary cortisol levels and activity of dogs as they awaited adoption at four animal shelters. Dogs’ urine was collected before and after outings for cortisol:creatinine analysis, and accelerometer devices were used to measure dogs’ physical activity. In total, 164 dogs participated in this study, with 793 cortisol values and 3750 activity measures used in the statistical analyses. We found that dogs’ cortisol:creatinine ratios were significantly higher during the afternoon of the intervention but returned to pre‐field trip levels the following day. Dogs’ minutes of low activity were significantly reduced, and high activity significantly increased during the outing. Although dogs’ cortisol and activity returned to baseline after the intervention, our findings suggest that short‐term outings do not confer the same stress reduction benefits as previously shown with temporary fostering. Nevertheless, it is possible that these types of outing programs are beneficial to adoptions by increasing the visibility of dogs and should be further investigated to elucidate these effects. |
  | Date | 2021 |
  | Language | English |
  | Archive | Embase |
  | URL | https://www.embase.com/search/results?subaction=viewrecord&id=L2006063987&from=export |
  | Volume | 11 |
  | Pages | 1-15 |
  | Publication | Animals |
  | DOI | 10.3390/ani11020548 |
  | Issue | 2 |
  | Journal Abbr | Animals |
  | ISSN | 2076-2615 |
  | Date Added | 11/07/2025, 14:18:27 |
  | Modified | 11/07/2025, 14:18:27 |

  ### Tags:

  - human-animal interaction
  - animal welfare
  - accelerometer
  - dog
  - animal experiment
  - article
  - nonhuman
  - physiological stress
  - hydrocortisone
  - controlled study
  - length of stay
  - animal food
  - autoanalyzer
  - bacterium isolation
  - chemiluminescence immunoassay
  - corticotropin
  - creatinine
  - data analysis
  - geographic distribution
  - hospitalization
  - hydrocortisone urine level
  - information processing
  - social isolation
  - United States
  - urine sampling
  - water

  ### Attachments

  - Full Text (HTML)
- ## Evaluating the effects of a temporary fostering program on shelter dog welfare

  |  |  |
  | --- | --- |
  | Item Type | Journal Article |
  | Author | L.M. Gunter |
  | Author | E.N. Feuerbacher |
  | Author | R.J. Gilchrist |
  | Author | C.D.L. Wynne |
  | Abstract | One of the greatest stressors for dogs living in animal shelters is social isolation. Many studies have demonstrated that human interaction reduces cortisol in shelter dogs, with the possibility that longer periods of interaction may yield greater effects. These types of interventions are contingent upon removing the dog from the kennel and any such reductions in cortisol are often lost when the dog returns to the kennel. More recently, animal shelters are utilizing short-term fostering programs to provide relief from the perceived stresses of kennel life; however the effects of these programs are not well understood. This study assessed the impacts of one- and two-night fostering programs on the urinary cortisol levels, resting pulse rates, longest bout of uninterrupted rest, and proportion of time spent resting of dogs awaiting adoption. Five animal shelters, open and limited-admission facilities, from across the United States participated in the study. During the study, dogs' urine was collected in the morning before, during, and after fostering stays for cortisol: Creatinine analysis. Non-invasive health monitors were worn by the dogs, which collected heart rates and activity levels, in the shelter and in foster homes. In total, 207 dogs participated in the study, and 1,076 cortisol values were used in our analysis. Across all shelters, we found that dogs' cortisol: Creatinine ratios dropped significantly during their fostering stay, but returned to baseline levels after return to the shelter. However, the observed reduction in cortisol varied in magnitude across shelters. We found that dogs of greater weight, age, and average resting pulse rate had higher cortisol levels; and dogs with longer bouts of uninterrupted rest had lower cortisol levels. Dogs had their longest bouts of rest during sleepovers, followed by in the shelter after their sleepovers. Lastly, significant differences were found when comparing in-shelter cortisol values at our five shelters, differences that were in some cases greater than the impact of the fostering intervention itself. Considering the diversity of facilities that participated in this study, it is possible that as yet unstudied, shelter-specific, environmental factors could be contributing to the overall welfare of shelter dogs. Thus while a reprieve from the shelter is impactful for dogs awaiting adoption, mitigating the stressors present in kenneling conditions should also be addressed to improve the lives of shelter dogs. |
  | Date | 2019 |
  | Language | English |
  | Archive | Embase |
  | URL | https://www.embase.com/search/results?subaction=viewrecord&id=L626944929&from=export |
  | Volume | 2019 |
  | Publication | PeerJ |
  | DOI | 10.7717/peerj.6620 |
  | Issue | 3 |
  | Journal Abbr | PeerJ |
  | ISSN | 2167-8359 |
  | Date Added | 11/07/2025, 14:18:34 |
  | Modified | 11/07/2025, 14:18:34 |

  ### Tags:

  - animal welfare
  - heart rate
  - physical activity
  - dog
  - article
  - nonhuman
  - hydrocortisone
  - body weight
  - length of stay
  - autoanalyzer
  - creatinine
  - social isolation
  - urine sampling
  - body temperature monitoring
  - breathing
  - community care
  - pulse rate
  - sleep

  ### Attachments

  - Full Text (HTML)
- ## Editorial: Exploring cats: their behaviors and human-cat interactions

  |  |  |
  | --- | --- |
  | Item Type | Journal Article |
  | Author | E.K. Grigg |
  | Author | D.C. Turner |
  | Author | L.A. Lyons |
  | Author | B.L. Hart |
  | Author | L.A. Hart |
  | Date | 2023 |
  | Language | English |
  | Archive | Embase |
  | URL | https://www.embase.com/search/results?subaction=viewrecord&id=L2027197995&from=export |
  | Volume | 10 |
  | Publication | Frontiers in Veterinary Science |
  | DOI | 10.3389/fvets.2023.1329398 |
  | Issue | (Grigg E.K., ekgrigg@ucdavis.edu; Hart L.A.) Department of Population Health and Reproduction, School of Veterinary Medicine, University of California, Davis, Davis, CA, United States |
  | Journal Abbr | Front. Vet. Sci. |
  | ISSN | 2297-1769 |
  | Date Added | 11/07/2025, 12:10:46 |
  | Modified | 11/07/2025, 12:10:46 |

  ### Tags:

  - human-animal interaction
  - animal welfare
  - heart rate variability
  - emotion
  - animal behavior
  - nonhuman
  - controlled study
  - editorial
  - wildlife
  - domestic cat
  - predation

  ### Attachments

  - Full Text (HTML)
- ## Assessing the Relationship Between Emotional States of Dogs and Their Human Handlers, Using Simultaneous Behavioral and Cardiac Measures

  |  |  |
  | --- | --- |
  | Item Type | Journal Article |
  | Author | E.K. Grigg |
  | Author | S. Liu |
  | Author | D.G. Dempsey |
  | Author | K. Wong |
  | Author | M. Bain |
  | Author | J.J. Sollers |
  | Author | R. Haddock |
  | Author | L.R. Kogan |
  | Author | J.A. Barnhard |
  | Author | A.A. Tringali |
  | Author | A.P. Thigpen |
  | Author | L.A. Hart |
  | Abstract | Negative stress due to human handling has been reported for a number of domestic animals, including dogs. Many companion dogs display significant stress during routine care in the veterinary clinic, risking injury to staff and potentially compromising the quality of care that these dogs receive. On the other hand, positive interactions with humans can have a beneficial effect on dogs, particularly in stressful situations such as animal shelters. Research has shown that dogs can detect human emotions through visual, auditory, and chemical channels, and that dogs will exhibit emotional contagion, particularly with familiar humans. This study investigated relationships between emotional states of dogs and unfamiliar human handlers, using simultaneous measures of cardiac activity and behavior, during two sessions of three consecutive routine handling sets. Measures of cardiac activity included mean heart rate (HRmean), and two measures of heart rate variability (HRV): the root mean square of successive differences between normal heartbeats (RMSSD); and the high frequency absolute power component of HRV, log transformed (HFlog). We also assessed human handlers' emotional state during handling sessions following an intervention designed to reduce stress, compared with sessions conducted on a different day and following a control activity. Polar H10 cardiac sensors were used to simultaneously record cardiac activity for both canine and human participants, and behavioral data were collected via digital video. The strongest influence on the dogs' stress levels in our study was found to be increasing familiarity with the setting and the handler; HRmean and SI decreased, and HRV (as RMSSD) increased, significantly from the first to the third handling set. Canine HRV (as HFlog) was also highest in set 3, although the difference was not statistically significant. There were no strong patterns found in the human cardiac data across handling set, session, or by pre-handling activity. We did not find consistent support for emotional contagion between the dogs and their handlers in this study, perhaps due to the brief time that the dogs spent with the handlers. Recommendations for application to dog handling, and limitations of our methods, are described. |
  | Date | 2022 |
  | Language | English |
  | Archive | Embase |
  | URL | https://www.embase.com/search/results?subaction=viewrecord&id=L2018307753&from=export |
  | Volume | 9 |
  | Publication | Frontiers in Veterinary Science |
  | DOI | 10.3389/fvets.2022.897287 |
  | Issue | (Grigg E.K., ekgrigg@ucdavis.edu; Tringali A.A.; Thigpen A.P.; Hart L.A.) Department of Population Health and Reproduction, School of Veterinary Medicine, University of California, Davis, Davis, CA, United States |
  | Journal Abbr | Front. Vet. Sci. |
  | ISSN | 2297-1769 |
  | Date Added | 11/07/2025, 12:10:47 |
  | Modified | 11/07/2025, 12:10:47 |

  ### Tags:

  - human-animal interaction
  - heart rate variability
  - heart rate
  - dog
  - emotion
  - animal behavior
  - animal experiment
  - article
  - female
  - male
  - nonhuman
  - physiological stress
  - human
  - adult
  - controlled study
  - information processing
  - sensor
  - electrode
  - power analysis
  - videorecording
  - heart contraction
  - mindfulness
  - mindfulness meditation
  - smartphone
  - statistics

  ### Attachments

  - Full Text (HTML)
- ## Effects of human-animal interaction on salivary and urinary oxytocin in children and dogs

  |  |  |
  | --- | --- |
  | Item Type | Journal Article |
  | Author | G.E. Gnanadesikan |
  | Author | K.M. King |
  | Author | E. Carranza |
  | Author | A.C. Flyer |
  | Author | G. Ossello |
  | Author | P.G. Smith |
  | Author | N.G. Steklis |
  | Author | H.D. Steklis |
  | Author | C.S. Carter |
  | Author | J.J. Connelly |
  | Author | M. Barnett |
  | Author | N. Gee |
  | Author | S.R. Tecot |
  | Author | E.L. MacLean |
  | Abstract | Oxytocin pathways are hypothesized to play important roles in human-animal interactions and may contribute to some benefits of these interspecific social relationships. We explored the effects of naturalistic interactions between children and dogs on oxytocin release in both species, as well as associations between methylation of the oxytocin receptor gene (OXTRm), social behavior, and oxytocin response in this context. Children (N = 55) participated in a within-subjects design involving a) interaction with their pet dog, b) interaction with an unfamiliar dog, and c) a nonsocial control condition (solitary play). We used immunoassays to measure salivary and urinary oxytocin in both the children and dogs, behavioral coding to characterize dog-child interactions, and bisulfite sequencing to quantify methylation of the oxytocin receptor gene (N = 32 children). Child salivary oxytocin decreased moderately across time in all conditions, but the extent of this effect varied between conditions, with greater oxytocin output during interactions with dogs than the control condition. In the pet dog condition, children's salivary oxytocin response was positively associated with the duration of visual co-orientation between the child and dog. Child urinary oxytocin did not deviate substantially from baseline in any condition. Children with higher levels of OXTRm had greater oxytocin output during interactions with their pet dogs, but lower oxytocin output in the control condition, and engaged in lower levels of affectionate interaction with dogs across conditions. Children's pet dogs exhibited increases in salivary oxytocin, but we observed the opposite pattern in the unfamiliar dog, who exhibited decreases in both urinary and salivary oxytocin on average. Collectively, our results support the hypothesis that oxytocin pathways may shape and respond to social interactions between children and dogs, highlighting an important role for companion animals in child development. |
  | Date | 2024 |
  | Language | English |
  | Archive | Embase |
  | URL | https://www.embase.com/search/results?subaction=viewrecord&id=L2033629127&from=export |
  | Volume | 169 |
  | Publication | Psychoneuroendocrinology |
  | DOI | 10.1016/j.psyneuen.2024.107147 |
  | Issue | (Gnanadesikan G.E., gnanadesikan@emory.edu; King K.M., kingk@arizona.edu; Flyer A.C.; Ossello G.; Smith P.G.; Tecot S.R.) School of Anthropology, University of Arizona, Tucson, AZ, United States |
  | Journal Abbr | Psychoneuroendocrinology |
  | ISSN | 1873-3360 |
  | Date Added | 11/07/2025, 12:12:14 |
  | Modified | 11/07/2025, 12:12:14 |

  ### Tags:

  - oxytocin
  - human-animal interaction
  - dog
  - social behavior
  - child
  - animal experiment
  - article
  - female
  - male
  - nonhuman
  - human
  - controlled study
  - human experiment
  - normal human
  - pet animal
  - adolescent
  - hormone response
  - affect
  - bisulfite sequencing
  - DNA methylation
  - gene
  - hormone urine level
  - immunoassay
  - infant
  - NCT03852264
  - OXTR gene
  - oxytocin receptor
  - oxytocin release
  - saliva level
  - school child
  - visual orientation

  ### Attachments

  - Full Text (HTML)
- ## Effects of human-animal interaction on salivary and urinary oxytocin in children and dogs

  |  |  |
  | --- | --- |
  | Item Type | Journal Article |
  | Author | G.E. Gnanadesikan |
  | Author | K.M. King |
  | Author | E. Carranza |
  | Author | A.C. Flyer |
  | Author | G. Ossello |
  | Author | P.G. Smith |
  | Author | N.G. Steklis |
  | Author | H.D. Steklis |
  | Author | C.S. Carter |
  | Author | J.J. Connelly |
  | Author | M. Barnett |
  | Author | N. Gee |
  | Author | S.R. Tecot |
  | Author | E.L. MacLean |
  | Abstract | Oxytocin pathways are hypothesized to play important roles in human-animal interactions and may contribute to some benefits of these interspecific social relationships. We explored the effects of naturalistic interactions between children and dogs on oxytocin release in both species, as well as associations between methylation of the oxytocin receptor gene (OXTRm), social behavior, and oxytocin response in this context. Children (N = 55) participated in a within-subjects design involving a) interaction with their pet dog, b) interaction with an unfamiliar dog, and c) a nonsocial control condition (solitary play). We used immunoassays to measure salivary and urinary oxytocin in both the children and dogs, behavioral coding to characterize dog-child interactions, and bisulfite sequencing to quantify methylation of the oxytocin receptor gene (N = 32 children). Child salivary oxytocin decreased moderately across time in all conditions, but the extent of this effect varied between conditions, with greater oxytocin output during interactions with dogs than the control condition. In the pet dog condition, children's salivary oxytocin response was positively associated with the duration of visual co-orientation between the child and dog. Child urinary oxytocin did not deviate substantially from baseline in any condition. Children with higher levels of OXTRm had greater oxytocin output during interactions with their pet dogs, but lower oxytocin output in the control condition, and engaged in lower levels of affectionate interaction with dogs across conditions. Children's pet dogs exhibited increases in salivary oxytocin, but we observed the opposite pattern in the unfamiliar dog, who exhibited decreases in both urinary and salivary oxytocin on average. Collectively, our results support the hypothesis that oxytocin pathways may shape and respond to social interactions between children and dogs, highlighting an important role for companion animals in child development. |
  | Date | 2024 |
  | Language | English |
  | Archive | Embase |
  | URL | https://www.embase.com/search/results?subaction=viewrecord&id=L2033629127&from=export |
  | Volume | 169 |
  | Publication | Psychoneuroendocrinology |
  | DOI | 10.1016/j.psyneuen.2024.107147 |
  | Issue | (Gnanadesikan G.E., gnanadesikan@emory.edu; King K.M., kingk@arizona.edu; Flyer A.C.; Ossello G.; Smith P.G.; Tecot S.R.) School of Anthropology, University of Arizona, Tucson, AZ, United States |
  | Journal Abbr | Psychoneuroendocrinology |
  | ISSN | 1873-3360 |
  | Date Added | 11/07/2025, 12:13:16 |
  | Modified | 11/07/2025, 12:13:16 |

  ### Tags:

  - oxytocin
  - human-animal interaction
  - dog
  - social behavior
  - child
  - animal experiment
  - article
  - female
  - male
  - nonhuman
  - human
  - controlled study
  - human experiment
  - normal human
  - pet animal
  - adolescent
  - hormone response
  - affect
  - bisulfite sequencing
  - DNA methylation
  - gene
  - hormone urine level
  - immunoassay
  - infant
  - NCT03852264
  - OXTR gene
  - oxytocin receptor
  - oxytocin release
  - saliva level
  - school child
  - visual orientation

  ### Attachments

  - Full Text (HTML)
- ## Glucocorticoid response to naturalistic interactions between children and dogs

  |  |  |
  | --- | --- |
  | Item Type | Journal Article |
  | Author | G.E. Gnanadesikan |
  | Author | E. Carranza |
  | Author | K.M. King |
  | Author | A.C. Flyer |
  | Author | G. Ossello |
  | Author | P.G. Smith |
  | Author | N.G. Steklis |
  | Author | H.D. Steklis |
  | Author | J.J. Connelly |
  | Author | M. Barnett |
  | Author | N. Gee |
  | Author | S. Tecot |
  | Author | E.L. MacLean |
  | Abstract | Although research has shown that pets appear to provide certain types of social support to children, little is known about the physiological bases of these effects, especially in naturalistic contexts. In this study, we investigated the effect of free-form interactions between children (ages 8–10 years) and dogs on salivary cortisol concentrations in both species. We further investigated the role of the child-dog relationship by comparing interactions with the child's pet dog to interactions with an unfamiliar dog or a nonsocial control condition, and modeled associations between survey measures of the human-animal bond and children's physiological responses. In both children and dogs, salivary cortisol decreased from pre- to post-interaction; the effect was strongest for children interacting with an unfamiliar dog (compared to their pet dog) and for the pet dogs (compared to the unfamiliar dog). We found minimal evidence for associations between cortisol output and behaviors coded from video, but children scoring higher on survey measures of the human-animal bond exhibited the greatest reductions in cortisol when interacting with dogs. Self-reported loneliness was not related to cortisol or the human-animal bond, but measures of both loneliness and the human-animal bond were higher among children who participated after the onset of the COVID-19 pandemic, relative to those who participated before the pandemic. This study builds on previous work that investigated potential stress-buffering effects of human-animal interaction during explicit stressors and demonstrates important physiological correlates of naturalistic interactions between children and dogs, similar to those that occur in daily life. |
  | Date | 2024 |
  | Language | English |
  | Archive | Embase |
  | URL | https://www.embase.com/search/results?subaction=viewrecord&id=L2031006555&from=export |
  | Volume | 161 |
  | Publication | Hormones and Behavior |
  | DOI | 10.1016/j.yhbeh.2024.105523 |
  | Issue | (Gnanadesikan G.E.; King K.M.; Flyer A.C.; Ossello G.; Smith P.G.; Tecot S.) School of Anthropology, University of Arizona, Tucson, AZ, United States |
  | Journal Abbr | Horm. Behav. |
  | ISSN | 1095-6867 |
  | Date Added | 11/07/2025, 14:15:29 |
  | Modified | 11/07/2025, 14:15:29 |

  ### Tags:

  - human-animal interaction
  - social support
  - dog
  - child
  - animal experiment
  - article
  - coronavirus disease 2019
  - female
  - male
  - nonhuman
  - pandemic
  - physiological stress
  - human
  - hydrocortisone
  - controlled study
  - scoring system
  - human experiment
  - pet animal
  - social bonding
  - saliva level
  - school child
  - loneliness

  ### Attachments

  - Full Text (HTML)
- ## Glucocorticoid response to naturalistic interactions between children and dogs

  |  |  |
  | --- | --- |
  | Item Type | Journal Article |
  | Author | G.E. Gnanadesikan |
  | Author | E. Carranza |
  | Author | K.M. King |
  | Author | A.C. Flyer |
  | Author | G. Ossello |
  | Author | P.G. Smith |
  | Author | N.G. Steklis |
  | Author | H.D. Steklis |
  | Author | J.J. Connelly |
  | Author | M. Barnett |
  | Author | N. Gee |
  | Author | S. Tecot |
  | Author | E.L. MacLean |
  | Abstract | Although research has shown that pets appear to provide certain types of social support to children, little is known about the physiological bases of these effects, especially in naturalistic contexts. In this study, we investigated the effect of free-form interactions between children (ages 8–10 years) and dogs on salivary cortisol concentrations in both species. We further investigated the role of the child-dog relationship by comparing interactions with the child's pet dog to interactions with an unfamiliar dog or a nonsocial control condition, and modeled associations between survey measures of the human-animal bond and children's physiological responses. In both children and dogs, salivary cortisol decreased from pre- to post-interaction; the effect was strongest for children interacting with an unfamiliar dog (compared to their pet dog) and for the pet dogs (compared to the unfamiliar dog). We found minimal evidence for associations between cortisol output and behaviors coded from video, but children scoring higher on survey measures of the human-animal bond exhibited the greatest reductions in cortisol when interacting with dogs. Self-reported loneliness was not related to cortisol or the human-animal bond, but measures of both loneliness and the human-animal bond were higher among children who participated after the onset of the COVID-19 pandemic, relative to those who participated before the pandemic. This study builds on previous work that investigated potential stress-buffering effects of human-animal interaction during explicit stressors and demonstrates important physiological correlates of naturalistic interactions between children and dogs, similar to those that occur in daily life. |
  | Date | 2024 |
  | Language | English |
  | Archive | Embase |
  | URL | https://www.embase.com/search/results?subaction=viewrecord&id=L2031006555&from=export |
  | Volume | 161 |
  | Publication | Hormones and Behavior |
  | DOI | 10.1016/j.yhbeh.2024.105523 |
  | Issue | (Gnanadesikan G.E.; King K.M.; Flyer A.C.; Ossello G.; Smith P.G.; Tecot S.) School of Anthropology, University of Arizona, Tucson, AZ, United States |
  | Journal Abbr | Horm. Behav. |
  | ISSN | 1095-6867 |
  | Date Added | 11/07/2025, 14:16:40 |
  | Modified | 11/07/2025, 14:16:40 |

  ### Tags:

  - human-animal interaction
  - social support
  - dog
  - child
  - animal experiment
  - article
  - coronavirus disease 2019
  - female
  - male
  - nonhuman
  - pandemic
  - physiological stress
  - human
  - hydrocortisone
  - controlled study
  - scoring system
  - human experiment
  - pet animal
  - social bonding
  - saliva level
  - school child
  - loneliness

  ### Attachments

  - Full Text (HTML)
- ## Glucocorticoid response to naturalistic interactions between children and dogs

  |  |  |
  | --- | --- |
  | Item Type | Journal Article |
  | Author | G.E. Gnanadesikan |
  | Author | E. Carranza |
  | Author | K.M. King |
  | Author | A.C. Flyer |
  | Author | G. Ossello |
  | Author | P.G. Smith |
  | Author | N.G. Steklis |
  | Author | H.D. Steklis |
  | Author | J.J. Connelly |
  | Author | M. Barnett |
  | Author | N. Gee |
  | Author | S. Tecot |
  | Author | E.L. MacLean |
  | Abstract | Although research has shown that pets appear to provide certain types of social support to children, little is known about the physiological bases of these effects, especially in naturalistic contexts. In this study, we investigated the effect of free-form interactions between children (ages 8–10 years) and dogs on salivary cortisol concentrations in both species. We further investigated the role of the child-dog relationship by comparing interactions with the child's pet dog to interactions with an unfamiliar dog or a nonsocial control condition, and modeled associations between survey measures of the human-animal bond and children's physiological responses. In both children and dogs, salivary cortisol decreased from pre- to post-interaction; the effect was strongest for children interacting with an unfamiliar dog (compared to their pet dog) and for the pet dogs (compared to the unfamiliar dog). We found minimal evidence for associations between cortisol output and behaviors coded from video, but children scoring higher on survey measures of the human-animal bond exhibited the greatest reductions in cortisol when interacting with dogs. Self-reported loneliness was not related to cortisol or the human-animal bond, but measures of both loneliness and the human-animal bond were higher among children who participated after the onset of the COVID-19 pandemic, relative to those who participated before the pandemic. This study builds on previous work that investigated potential stress-buffering effects of human-animal interaction during explicit stressors and demonstrates important physiological correlates of naturalistic interactions between children and dogs, similar to those that occur in daily life. |
  | Date | 2024 |
  | Language | English |
  | Archive | Embase |
  | URL | https://www.embase.com/search/results?subaction=viewrecord&id=L2031006555&from=export |
  | Volume | 161 |
  | Publication | Hormones and Behavior |
  | DOI | 10.1016/j.yhbeh.2024.105523 |
  | Issue | (Gnanadesikan G.E.; King K.M.; Flyer A.C.; Ossello G.; Smith P.G.; Tecot S.) School of Anthropology, University of Arizona, Tucson, AZ, United States |
  | Journal Abbr | Horm. Behav. |
  | ISSN | 1095-6867 |
  | Date Added | 11/07/2025, 14:18:13 |
  | Modified | 11/07/2025, 14:18:13 |

  ### Tags:

  - human-animal interaction
  - social support
  - dog
  - child
  - animal experiment
  - article
  - coronavirus disease 2019
  - female
  - male
  - nonhuman
  - pandemic
  - physiological stress
  - human
  - hydrocortisone
  - controlled study
  - scoring system
  - human experiment
  - pet animal
  - social bonding
  - saliva level
  - school child
  - loneliness

  ### Attachments

  - Full Text (HTML)
- ## Therapy dog welfare revisited: A review of the literature

  |  |  |
  | --- | --- |
  | Item Type | Journal Article |
  | Author | L.M. Glenk |
  | Author | S. Foltin |
  | Abstract | During the past decade, the field of human–animal interaction(s) research has been characterized by a significant increase in scientific findings. These data have contributed to our current understanding of how humans may benefit from contact with animals. However, the animal experience of these interactions is still an under-researched area. This paper addresses the welfare of dogs who participate in animal-assisted interventions (AAIs) to improve health in human recipients. This paper builds on previous work by Glenk (2017) and provides an updated review of the literature on therapy dog welfare published from 2017–2021. New advances in scientific methodology, such as the determination of salivary oxytocin, breath rate and tympanic membrane temperature, are analyzed regarding their value and limitations for research in AAIs. Moreover, welfare-related social and environmental factors (e.g., freedom of choice, exploration of novel environments, inequity aversion, individual development, working experience, relationship with handler and handler skills) that profoundly influence dog perception and well-being are reviewed and discussed. Accounting for the globally increasing interest and the number of dogs utilized in AAIs, safeguarding therapy dog well-being, and identifying situations, circumstances and protocols that may challenge animal welfare remains an emerging and crucial area of scientific effort. |
  | Date | 2021 |
  | Language | English |
  | Archive | Embase |
  | URL | https://www.embase.com/search/results?subaction=viewrecord&id=L2014202517&from=export |
  | Volume | 8 |
  | Publication | Veterinary Sciences |
  | DOI | 10.3390/vetsci8100226 |
  | Issue | 10 |
  | Journal Abbr | Vet. Sci. |
  | ISSN | 2306-7381 |
  | Date Added | 11/07/2025, 12:12:24 |
  | Modified | 11/07/2025, 12:12:24 |

  ### Tags:

  - oxytocin
  - human-animal interaction
  - animal welfare
  - dog
  - saliva
  - nonhuman
  - animal well-being
  - review
  - wellbeing
  - animal assisted therapy
  - perception
  - breathing
  - environmental factor
  - environmental temperature
  - social aspect
  - work experience

  ### Attachments

  - Full Text (HTML)
- ## Therapy dog welfare revisited: A review of the literature

  |  |  |
  | --- | --- |
  | Item Type | Journal Article |
  | Author | L.M. Glenk |
  | Author | S. Foltin |
  | Abstract | During the past decade, the field of human–animal interaction(s) research has been characterized by a significant increase in scientific findings. These data have contributed to our current understanding of how humans may benefit from contact with animals. However, the animal experience of these interactions is still an under-researched area. This paper addresses the welfare of dogs who participate in animal-assisted interventions (AAIs) to improve health in human recipients. This paper builds on previous work by Glenk (2017) and provides an updated review of the literature on therapy dog welfare published from 2017–2021. New advances in scientific methodology, such as the determination of salivary oxytocin, breath rate and tympanic membrane temperature, are analyzed regarding their value and limitations for research in AAIs. Moreover, welfare-related social and environmental factors (e.g., freedom of choice, exploration of novel environments, inequity aversion, individual development, working experience, relationship with handler and handler skills) that profoundly influence dog perception and well-being are reviewed and discussed. Accounting for the globally increasing interest and the number of dogs utilized in AAIs, safeguarding therapy dog well-being, and identifying situations, circumstances and protocols that may challenge animal welfare remains an emerging and crucial area of scientific effort. |
  | Date | 2021 |
  | Language | English |
  | Archive | Embase |
  | URL | https://www.embase.com/search/results?subaction=viewrecord&id=L2014202517&from=export |
  | Volume | 8 |
  | Publication | Veterinary Sciences |
  | DOI | 10.3390/vetsci8100226 |
  | Issue | 10 |
  | Journal Abbr | Vet. Sci. |
  | ISSN | 2306-7381 |
  | Date Added | 11/07/2025, 12:13:26 |
  | Modified | 11/07/2025, 12:13:26 |

  ### Tags:

  - oxytocin
  - human-animal interaction
  - animal welfare
  - dog
  - saliva
  - nonhuman
  - animal well-being
  - review
  - wellbeing
  - animal assisted therapy
  - perception
  - breathing
  - environmental factor
  - environmental temperature
  - social aspect
  - work experience

  ### Attachments

  - Full Text (HTML)
- ## Therapy dog welfare revisited: A review of the literature

  |  |  |
  | --- | --- |
  | Item Type | Journal Article |
  | Author | L.M. Glenk |
  | Author | S. Foltin |
  | Abstract | During the past decade, the field of human–animal interaction(s) research has been characterized by a significant increase in scientific findings. These data have contributed to our current understanding of how humans may benefit from contact with animals. However, the animal experience of these interactions is still an under-researched area. This paper addresses the welfare of dogs who participate in animal-assisted interventions (AAIs) to improve health in human recipients. This paper builds on previous work by Glenk (2017) and provides an updated review of the literature on therapy dog welfare published from 2017–2021. New advances in scientific methodology, such as the determination of salivary oxytocin, breath rate and tympanic membrane temperature, are analyzed regarding their value and limitations for research in AAIs. Moreover, welfare-related social and environmental factors (e.g., freedom of choice, exploration of novel environments, inequity aversion, individual development, working experience, relationship with handler and handler skills) that profoundly influence dog perception and well-being are reviewed and discussed. Accounting for the globally increasing interest and the number of dogs utilized in AAIs, safeguarding therapy dog well-being, and identifying situations, circumstances and protocols that may challenge animal welfare remains an emerging and crucial area of scientific effort. |
  | Date | 2021 |
  | Language | English |
  | Archive | Embase |
  | URL | https://www.embase.com/search/results?subaction=viewrecord&id=L2014202517&from=export |
  | Volume | 8 |
  | Publication | Veterinary Sciences |
  | DOI | 10.3390/vetsci8100226 |
  | Issue | 10 |
  | Journal Abbr | Vet. Sci. |
  | ISSN | 2306-7381 |
  | Date Added | 11/07/2025, 14:20:20 |
  | Modified | 11/07/2025, 14:20:20 |

  ### Tags:

  - oxytocin
  - human-animal interaction
  - animal welfare
  - dog
  - saliva
  - nonhuman
  - animal well-being
  - review
  - wellbeing
  - animal assisted therapy
  - perception
  - breathing
  - environmental factor
  - environmental temperature
  - social aspect
  - work experience

  ### Attachments

  - Full Text (HTML)
- ## Visitor effects on the welfare of captive Sumatran orangutans (Pongo abelii) during the pandemic lockdowns

  |  |  |
  | --- | --- |
  | Item Type | Journal Article |
  | Author | Ezekiel F. Gading |
  | Author | Valerie Am Schoof |
  | Author | Maria Franke |
  | Author | Suzanne E. MacDonald |
  | Abstract | The COVID-19 pandemic led to unprecedented lockdowns with rippling impacts on the lives of humans and animals alike. Since zoos were among the first institutions to close during the pandemic, the lockdowns presented the opportunity to conduct a natural experiment examining the relationship between visitor presence and the welfare of zoo-housed animals. In this study, we assessed the welfare of six Sumatran orangutans (Pongo abelii) at Toronto Zoo both during and following the pandemic lockdowns. We compared behavioural and physiological indicators of welfare during a lockdown and after visitors were reintroduced. Specifically, if the orangutans' welfare was affected by the visitor re-introduction phase we predicted there would be an increase in the following measures: (1) use of exhibit areas away from visitors; (2) behavioural measures (hiding, self-directed behaviours, agonistic behaviours, agitated movement, and idiosyncratic object-directed behaviours [head slamming, and fabric tearing]); and (3) physiological measures (faecal consistency and glucocorticoid metabolites) when compared to the lockdown. We also measured changes in activity levels such as foraging and inactivity. We found that orangutan exhibit space use did not change when visitors were reintroduced. In fact, the orangutans hid less when visitors were introduced than during the lockdown. Foraging, inactivity, and other behavioural indicators of stress did not change when visitors were introduced. Similarly, neither faecal consistency nor glucocorticoid metabolites changed across the study phases. Our data show that visitor re-introduction did not negatively affect the welfare of the Toronto Zoo orangutans. However, the presence of keepers was found to affect the behaviour of the orangutans and warrants further study. |
  | Date | 2025 |
  | Language | eng |
  | Library Catalogue | PubMed |
  | Volume | 34 |
  | Pages | e15 |
  | Publication | Animal Welfare (South Mimms, England) |
  | DOI | 10.1017/awf.2025.9 |
  | Journal Abbr | Anim Welf |
  | ISSN | 2054-1538 |
  | PMID | 40071106 |
  | PMCID | PMC11894407 |
  | Date Added | 11/07/2025, 11:33:21 |
  | Modified | 04/01/2026, 11:26:06 |

  ### Tags:

  - human-animal interaction
  - stress
  - animal welfare
  - behaviour
  - COVID-19
  - primates
  - agitation
  - animal behavior
  - animal experiment
  - article
  - Canada
  - coronavirus disease 2019
  - defecation
  - female
  - foraging
  - glucocorticoid
  - lockdown
  - locomotion
  - male
  - metabolite
  - nonhuman
  - orangutan
  - pandemic
  - physiological stress
  - play
  - Pongo abelii
  - primate
  - social interaction
  - zoo animal

  ### Notes:

  - Cited by: 0; All Open Access, Gold Open Access

  ### Attachments

  - Full Text (HTML)
  - PubMed entry
- ## Human physiological responses to different types of human-dog interactions: A randomised crossover study

  |  |  |
  | --- | --- |
  | Item Type | Journal Article |
  | Author | L.H. Fuglsang-Damgaard |
  | Author | S.J. Lunde |
  | Author | J.W. Christensen |
  | Author | L. Vase |
  | Author | P.B. Videbech |
  | Author | K. Thodberg |
  | Abstract | Previous studies of human-dog interventions vary in terms of type of interaction, which is rarely quantified, leading to contradictory findings and limited comparability. To uncover the influence of different types of interactions, the present study investigated if it was possible to detect differences in immediate physiological measurements of healthy humans during different standardised types of interaction with a dog. Thirty-three healthy participants (women = 25, men = 8, >18 years) were exposed to four different test situations with standardised types of interaction intensity with a dog in random order: no dog present (CONTROL), looking at a dog (VISUAL), petting a dog (TACTILE) or performing tricks with a dog (ACTIVE). Each test situation lasted 10 min with a 30-min break between each. Heart rate (HR), heart rate variability (HRV) and skin conductance (tonic level (SCL) and peak counts (SCR)) were continuously recorded. Blood pressure (BP) and salivary cortisol (s-cortisol) were measured before and after each test situation. Linear Mixed Models were applied. HR, HRV, BP, SCL and SCR increased with increased interaction with the dog (for all: p < 0.001). HRV increased with decreased HR (p = 0.002), increased SCL (p = 0.027), and SCR (p < 0.001) depending on the type of interaction. Generally, s-cortisol increased with increased HR (p = 0.042), SCL increased with increased SCR (p < 0.001), and SCR increased with increased HRV (p = 0.013), depending on type of interaction. The physiological measurements HR, HRV, BP, SCL and SCR are influenced by different types of dog interaction, and thus it is important to quantify and report the type of interaction in human-dog interaction studies. (ClinicalTrials.gov ID:NCT04696419). |
  | Date | 2024 |
  | Language | English |
  | Archive | Medline |
  | URL | https://www.embase.com/search/results?subaction=viewrecord&id=L645153993&from=export |
  | Volume | 57 |
  | Pages | 101899 |
  | Publication | Complementary therapies in clinical practice |
  | DOI | 10.1016/j.ctcp.2024.101899 |
  | Issue | (Fuglsang-Damgaard L.H.) Department of Animal and Veterinary Sciences, Aarhus University, Denmark |
  | Journal Abbr | Complement Ther Clin Pract |
  | ISSN | 1873-6947 |
  | Date Added | 11/07/2025, 11:53:17 |
  | Modified | 11/07/2025, 11:53:17 |

  ### Tags:

  - blood pressure
  - human-animal interaction
  - physiology
  - heart rate
  - dog
  - saliva
  - female
  - male
  - human
  - hydrocortisone
  - adult
  - controlled study
  - randomized controlled trial
  - crossover procedure
  - animal
  - young adult
  - blood
  - chemistry
  - NCT04696419

  ### Attachments

  - Full Text (HTML)
- ## Human physiological responses to different types of human-dog interactions: A randomised crossover study

  |  |  |
  | --- | --- |
  | Item Type | Journal Article |
  | Author | L.H. Fuglsang-Damgaard |
  | Author | S.J. Lunde |
  | Author | J.W. Christensen |
  | Author | L. Vase |
  | Author | P.B. Videbech |
  | Author | K. Thodberg |
  | Abstract | Previous studies of human-dog interventions vary in terms of type of interaction, which is rarely quantified, leading to contradictory findings and limited comparability. To uncover the influence of different types of interactions, the present study investigated if it was possible to detect differences in immediate physiological measurements of healthy humans during different standardised types of interaction with a dog. Thirty-three healthy participants (women = 25, men = 8, >18 years) were exposed to four different test situations with standardised types of interaction intensity with a dog in random order: no dog present (CONTROL), looking at a dog (VISUAL), petting a dog (TACTILE) or performing tricks with a dog (ACTIVE). Each test situation lasted 10 min with a 30-min break between each. Heart rate (HR), heart rate variability (HRV) and skin conductance (tonic level (SCL) and peak counts (SCR)) were continuously recorded. Blood pressure (BP) and salivary cortisol (s-cortisol) were measured before and after each test situation. Linear Mixed Models were applied. HR, HRV, BP, SCL and SCR increased with increased interaction with the dog (for all: p < 0.001). HRV increased with decreased HR (p = 0.002), increased SCL (p = 0.027), and SCR (p < 0.001) depending on the type of interaction. Generally, s-cortisol increased with increased HR (p = 0.042), SCL increased with increased SCR (p < 0.001), and SCR increased with increased HRV (p = 0.013), depending on type of interaction. The physiological measurements HR, HRV, BP, SCL and SCR are influenced by different types of dog interaction, and thus it is important to quantify and report the type of interaction in human-dog interaction studies. (ClinicalTrials.gov ID:NCT04696419). |
  | Date | 2024 |
  | Language | English |
  | Archive | Medline |
  | URL | https://www.embase.com/search/results?subaction=viewrecord&id=L645153993&from=export |
  | Volume | 57 |
  | Pages | 101899 |
  | Publication | Complementary therapies in clinical practice |
  | DOI | 10.1016/j.ctcp.2024.101899 |
  | Issue | (Fuglsang-Damgaard L.H.) Department of Animal and Veterinary Sciences, Aarhus University, Denmark |
  | Journal Abbr | Complement Ther Clin Pract |
  | ISSN | 1873-6947 |
  | Date Added | 11/07/2025, 12:10:38 |
  | Modified | 11/07/2025, 12:10:38 |

  ### Tags:

  - blood pressure
  - human-animal interaction
  - physiology
  - heart rate
  - dog
  - saliva
  - female
  - male
  - human
  - hydrocortisone
  - adult
  - controlled study
  - randomized controlled trial
  - crossover procedure
  - animal
  - young adult
  - blood
  - chemistry
  - NCT04696419

  ### Attachments

  - Full Text (HTML)
- ## Human physiological responses to different types of human-dog interactions: A randomised crossover study

  |  |  |
  | --- | --- |
  | Item Type | Journal Article |
  | Author | L.H. Fuglsang-Damgaard |
  | Author | S.J. Lunde |
  | Author | J.W. Christensen |
  | Author | L. Vase |
  | Author | P.B. Videbech |
  | Author | K. Thodberg |
  | Abstract | Previous studies of human-dog interventions vary in terms of type of interaction, which is rarely quantified, leading to contradictory findings and limited comparability. To uncover the influence of different types of interactions, the present study investigated if it was possible to detect differences in immediate physiological measurements of healthy humans during different standardised types of interaction with a dog. Thirty-three healthy participants (women = 25, men = 8, >18 years) were exposed to four different test situations with standardised types of interaction intensity with a dog in random order: no dog present (CONTROL), looking at a dog (VISUAL), petting a dog (TACTILE) or performing tricks with a dog (ACTIVE). Each test situation lasted 10 min with a 30-min break between each. Heart rate (HR), heart rate variability (HRV) and skin conductance (tonic level (SCL) and peak counts (SCR)) were continuously recorded. Blood pressure (BP) and salivary cortisol (s-cortisol) were measured before and after each test situation. Linear Mixed Models were applied. HR, HRV, BP, SCL and SCR increased with increased interaction with the dog (for all: p < 0.001). HRV increased with decreased HR (p = 0.002), increased SCL (p = 0.027), and SCR (p < 0.001) depending on the type of interaction. Generally, s-cortisol increased with increased HR (p = 0.042), SCL increased with increased SCR (p < 0.001), and SCR increased with increased HRV (p = 0.013), depending on type of interaction. The physiological measurements HR, HRV, BP, SCL and SCR are influenced by different types of dog interaction, and thus it is important to quantify and report the type of interaction in human-dog interaction studies. (ClinicalTrials.gov ID:NCT04696419). |
  | Date | 2024 |
  | Language | English |
  | Archive | Medline |
  | URL | https://www.embase.com/search/results?subaction=viewrecord&id=L645153993&from=export |
  | Volume | 57 |
  | Pages | 101899 |
  | Publication | Complementary therapies in clinical practice |
  | DOI | 10.1016/j.ctcp.2024.101899 |
  | Issue | (Fuglsang-Damgaard L.H.) Department of Animal and Veterinary Sciences, Aarhus University, Denmark |
  | Journal Abbr | Complement Ther Clin Pract |
  | ISSN | 1873-6947 |
  | Date Added | 11/07/2025, 14:15:27 |
  | Modified | 11/07/2025, 14:15:27 |

  ### Tags:

  - blood pressure
  - human-animal interaction
  - physiology
  - heart rate
  - dog
  - saliva
  - female
  - male
  - human
  - hydrocortisone
  - adult
  - controlled study
  - randomized controlled trial
  - crossover procedure
  - animal
  - young adult
  - blood
  - chemistry
  - NCT04696419

  ### Attachments

  - Full Text (HTML)
- ## Human physiological responses to different types of human-dog interactions: A randomised crossover study

  |  |  |
  | --- | --- |
  | Item Type | Journal Article |
  | Author | L.H. Fuglsang-Damgaard |
  | Author | S.J. Lunde |
  | Author | J.W. Christensen |
  | Author | L. Vase |
  | Author | P.B. Videbech |
  | Author | K. Thodberg |
  | Abstract | Previous studies of human-dog interventions vary in terms of type of interaction, which is rarely quantified, leading to contradictory findings and limited comparability. To uncover the influence of different types of interactions, the present study investigated if it was possible to detect differences in immediate physiological measurements of healthy humans during different standardised types of interaction with a dog. Thirty-three healthy participants (women = 25, men = 8, >18 years) were exposed to four different test situations with standardised types of interaction intensity with a dog in random order: no dog present (CONTROL), looking at a dog (VISUAL), petting a dog (TACTILE) or performing tricks with a dog (ACTIVE). Each test situation lasted 10 min with a 30-min break between each. Heart rate (HR), heart rate variability (HRV) and skin conductance (tonic level (SCL) and peak counts (SCR)) were continuously recorded. Blood pressure (BP) and salivary cortisol (s-cortisol) were measured before and after each test situation. Linear Mixed Models were applied. HR, HRV, BP, SCL and SCR increased with increased interaction with the dog (for all: p < 0.001). HRV increased with decreased HR (p = 0.002), increased SCL (p = 0.027), and SCR (p < 0.001) depending on the type of interaction. Generally, s-cortisol increased with increased HR (p = 0.042), SCL increased with increased SCR (p < 0.001), and SCR increased with increased HRV (p = 0.013), depending on type of interaction. The physiological measurements HR, HRV, BP, SCL and SCR are influenced by different types of dog interaction, and thus it is important to quantify and report the type of interaction in human-dog interaction studies. (ClinicalTrials.gov ID:NCT04696419). |
  | Date | 2024 |
  | Language | English |
  | Archive | Medline |
  | URL | https://www.embase.com/search/results?subaction=viewrecord&id=L645153993&from=export |
  | Volume | 57 |
  | Pages | 101899 |
  | Publication | Complementary therapies in clinical practice |
  | DOI | 10.1016/j.ctcp.2024.101899 |
  | Issue | (Fuglsang-Damgaard L.H.) Department of Animal and Veterinary Sciences, Aarhus University, Denmark |
  | Journal Abbr | Complement Ther Clin Pract |
  | ISSN | 1873-6947 |
  | Date Added | 11/07/2025, 14:16:37 |
  | Modified | 11/07/2025, 14:16:37 |

  ### Tags:

  - blood pressure
  - human-animal interaction
  - physiology
  - heart rate
  - dog
  - saliva
  - female
  - male
  - human
  - hydrocortisone
  - adult
  - controlled study
  - randomized controlled trial
  - crossover procedure
  - animal
  - young adult
  - blood
  - chemistry
  - NCT04696419

  ### Attachments

  - Full Text (HTML)
- ## Human physiological responses to different types of human-dog interactions: A randomised crossover study

  |  |  |
  | --- | --- |
  | Item Type | Journal Article |
  | Author | L.H. Fuglsang-Damgaard |
  | Author | S.J. Lunde |
  | Author | J.W. Christensen |
  | Author | L. Vase |
  | Author | P.B. Videbech |
  | Author | K. Thodberg |
  | Abstract | Previous studies of human-dog interventions vary in terms of type of interaction, which is rarely quantified, leading to contradictory findings and limited comparability. To uncover the influence of different types of interactions, the present study investigated if it was possible to detect differences in immediate physiological measurements of healthy humans during different standardised types of interaction with a dog. Thirty-three healthy participants (women = 25, men = 8, >18 years) were exposed to four different test situations with standardised types of interaction intensity with a dog in random order: no dog present (CONTROL), looking at a dog (VISUAL), petting a dog (TACTILE) or performing tricks with a dog (ACTIVE). Each test situation lasted 10 min with a 30-min break between each. Heart rate (HR), heart rate variability (HRV) and skin conductance (tonic level (SCL) and peak counts (SCR)) were continuously recorded. Blood pressure (BP) and salivary cortisol (s-cortisol) were measured before and after each test situation. Linear Mixed Models were applied. HR, HRV, BP, SCL and SCR increased with increased interaction with the dog (for all: p < 0.001). HRV increased with decreased HR (p = 0.002), increased SCL (p = 0.027), and SCR (p < 0.001) depending on the type of interaction. Generally, s-cortisol increased with increased HR (p = 0.042), SCL increased with increased SCR (p < 0.001), and SCR increased with increased HRV (p = 0.013), depending on type of interaction. The physiological measurements HR, HRV, BP, SCL and SCR are influenced by different types of dog interaction, and thus it is important to quantify and report the type of interaction in human-dog interaction studies. (ClinicalTrials.gov ID:NCT04696419). |
  | Date | 2024 |
  | Language | English |
  | Archive | Medline |
  | URL | https://www.embase.com/search/results?subaction=viewrecord&id=L645153993&from=export |
  | Volume | 57 |
  | Pages | 101899 |
  | Publication | Complementary therapies in clinical practice |
  | DOI | 10.1016/j.ctcp.2024.101899 |
  | Issue | (Fuglsang-Damgaard L.H.) Department of Animal and Veterinary Sciences, Aarhus University, Denmark |
  | Journal Abbr | Complement Ther Clin Pract |
  | ISSN | 1873-6947 |
  | Date Added | 11/07/2025, 14:18:10 |
  | Modified | 11/07/2025, 14:18:10 |

  ### Tags:

  - blood pressure
  - human-animal interaction
  - physiology
  - heart rate
  - dog
  - saliva
  - female
  - male
  - human
  - hydrocortisone
  - adult
  - controlled study
  - randomized controlled trial
  - crossover procedure
  - animal
  - young adult
  - blood
  - chemistry
  - NCT04696419

  ### Attachments

  - Full Text (HTML)
- ## A Leash on Life: An Exploratory Study on the Effects of a Shelter-Dog Walking Program for Veterans on Dogs’ Stress

  |  |  |
  | --- | --- |
  | Item Type | Journal Article |
  | Author | E. Friedmann |
  | Author | C.A. Krause-Parello |
  | Author | M. Payton |
  | Author | K. Blanchard |
  | Author | A. Storm |
  | Author | E. Barr |
  | Author | N.R. Gee |
  | Abstract | Human–animal interaction (HAI) such a dog walking is used as an intervention to decrease stress in individuals with Post-traumatic Stress Disorder (PTSD). However, it is important to know whether PTSD symptoms of a dog walker and the walk itself stress the shelter dogs. This exploratory project evaluated the impact of a shelter-dog walking program for veterans on stress experienced by the dogs. It also examined whether the PTSD status of the veteran influenced the impact of the walk on the dog. Prior to data collection university, IRB/IACUC approvals were obtained. Shelter dogs (n = 25) were walked by veterans who were participating in an 8-week walking program. Seventeen of the walks (45%) were by veterans who reported PTSD. Dog stress was assessed with heart rate variability (HRV) measured for 24 h before, during, and for one hour after the veteran’s first and last walks. The walks were designed to take 30 min. The study included 38 walks by 25 dogs and their 28 veteran walkers. The dogs were largely male (60%) and mixed breeds (80%). Sizes of dogs were approximately equally distributed between small, medium, and large sizes. Dog stress was not significantly higher during dog walks when compared with similar times of day in the previous day or with the period immediately prior to the walk. Post-walk, dog stress was significantly lower after walks with veterans with PTSD (HRV M = 11.64, SD = 0.15, p = 0.01, d = 1.09) than for walks with veterans without PTSD (HRV M= 11.42, SD = 0.28). This study provided no evidence that being walked by veterans with PTSD was stressful for shelter dogs despite the unfamiliarity of the veteran dog walkers. Future studies with larger sample sizes in multiple shelters should be conducted to extend the current results. |
  | Date | 2022 |
  | Language | English |
  | Archive | Embase |
  | URL | https://www.embase.com/search/results?subaction=viewrecord&id=L2013146879&from=export |
  | Volume | 35 |
  | Pages | 23-36 |
  | Publication | Anthrozoos |
  | DOI | 10.1080/08927936.2021.1944559 |
  | Issue | 1 |
  | Journal Abbr | Anthrozoos |
  | ISSN | 1753-0377 |
  | Date Added | 11/07/2025, 12:10:48 |
  | Modified | 11/07/2025, 12:10:48 |

  ### Tags:

  - human-animal interaction
  - veteran
  - heart rate variability
  - accelerometer
  - physical activity
  - dog
  - animal experiment
  - article
  - female
  - male
  - nonhuman
  - human
  - adult
  - aged
  - controlled study
  - posttraumatic stress disorder
  - questionnaire
  - animal model
  - exploratory research
  - observational study
  - sinus rhythm
  - walking speed

  ### Attachments

  - Full Text (HTML)
- ## The Power of Discourse: Associations between Trainers’ Speech and the Responses of Socialized Wolves and Dogs to Training

  |  |  |
  | --- | --- |
  | Item Type | Journal Article |
  | Author | M.G.B. Fonseca |
  | Author | H.O. Hilário |
  | Author | K. Kotrschal |
  | Author | F. Range |
  | Author | Z. Virányi |
  | Author | M.H.L. Duarte |
  | Author | L.C.G. Pereira |
  | Author | A.D.S. Vasconcellos |
  | Abstract | In a previous study, we found that Positive Reinforcement Training reduced cortisol of wolves and dogs; however, this effect varied across trainer–animal dyads. Here we investigate whether and how the trainers’ use of speech may contribute to this effect. Dogs’ great interest in high-pitched, intense speech (also known as Dog Directed Speech) has already been reported, but whether and how wolves respond similarly/differently to voice characteristics has never been studied before. We analyzed 270 training sessions, conducted by five trainers, with nine mixed-breed dogs and nine wolves, all human-socialized. Through Generalized Linear Mixed Models, we analyzed the effects of (a) three speech categories (nice, neutral, reprehensive) and laugh; and (b) acoustic characteristics of trainers’ voices on animals’ responses (correct responses, latency, orientation, time at less than 1 m, non-training behaviors, tail position/movements, cortisol variation). In both subspecies, tail wagging occurred more often in sessions with longer durations of nice speech, and less often in sessions with reprehensive speech. For dogs, the duration of reprehensive speech within a session was also negatively related to correct responses. For wolves, retreat time was associated with more reprehensive speech, whereas duration of nice speech was positively associated with time spent within one meter from the trainer. In addition, most dog behavioral responses were associated with higher average intonations within sessions, while wolf responses were correlated with lower intonations within sessions. We did not find any effects of the variables considered on cortisol variation. Our study highlights the relevance of voice tone and speech in a training context on animals’ performances and emotional reactions. |
  | Date | 2023 |
  | Language | English |
  | Archive | Embase |
  | URL | https://www.embase.com/search/results?subaction=viewrecord&id=L2022294333&from=export |
  | Volume | 13 |
  | Publication | Animals |
  | DOI | 10.3390/ani13061071 |
  | Issue | 6 |
  | Journal Abbr | Animals |
  | ISSN | 2076-2615 |
  | Date Added | 11/07/2025, 14:15:35 |
  | Modified | 11/07/2025, 14:15:35 |

  ### Tags:

  - human-animal interaction
  - animal welfare
  - dog
  - saliva
  - animal behavior
  - animal experiment
  - article
  - female
  - male
  - nonhuman
  - human
  - hydrocortisone
  - adult
  - controlled study
  - data analysis software
  - training
  - voice
  - latent period
  - emotionality
  - saliva collector
  - Salivette
  - visual orientation
  - socialization
  - wolf
  - vocalization
  - acoustics
  - analytical equipment
  - Canis lupus
  - data visualization
  - frequency analysis
  - hydrocellulose sponge
  - plastic tube
  - positive reinforcement training
  - Raven Pro 1.5
  - reinforcement (psychology)
  - spectroscopy
  - speech
  - subspecies
  - surgical sponge
  - tail
  - time perception
  - trainer

  ### Attachments

  - Full Text (HTML)
- ## The Power of Discourse: Associations between Trainers’ Speech and the Responses of Socialized Wolves and Dogs to Training

  |  |  |
  | --- | --- |
  | Item Type | Journal Article |
  | Author | M.G.B. Fonseca |
  | Author | H.O. Hilário |
  | Author | K. Kotrschal |
  | Author | F. Range |
  | Author | Z. Virányi |
  | Author | M.H.L. Duarte |
  | Author | L.C.G. Pereira |
  | Author | A.D.S. Vasconcellos |
  | Abstract | In a previous study, we found that Positive Reinforcement Training reduced cortisol of wolves and dogs; however, this effect varied across trainer–animal dyads. Here we investigate whether and how the trainers’ use of speech may contribute to this effect. Dogs’ great interest in high-pitched, intense speech (also known as Dog Directed Speech) has already been reported, but whether and how wolves respond similarly/differently to voice characteristics has never been studied before. We analyzed 270 training sessions, conducted by five trainers, with nine mixed-breed dogs and nine wolves, all human-socialized. Through Generalized Linear Mixed Models, we analyzed the effects of (a) three speech categories (nice, neutral, reprehensive) and laugh; and (b) acoustic characteristics of trainers’ voices on animals’ responses (correct responses, latency, orientation, time at less than 1 m, non-training behaviors, tail position/movements, cortisol variation). In both subspecies, tail wagging occurred more often in sessions with longer durations of nice speech, and less often in sessions with reprehensive speech. For dogs, the duration of reprehensive speech within a session was also negatively related to correct responses. For wolves, retreat time was associated with more reprehensive speech, whereas duration of nice speech was positively associated with time spent within one meter from the trainer. In addition, most dog behavioral responses were associated with higher average intonations within sessions, while wolf responses were correlated with lower intonations within sessions. We did not find any effects of the variables considered on cortisol variation. Our study highlights the relevance of voice tone and speech in a training context on animals’ performances and emotional reactions. |
  | Date | 2023 |
  | Language | English |
  | Archive | Embase |
  | URL | https://www.embase.com/search/results?subaction=viewrecord&id=L2022294333&from=export |
  | Volume | 13 |
  | Publication | Animals |
  | DOI | 10.3390/ani13061071 |
  | Issue | 6 |
  | Journal Abbr | Animals |
  | ISSN | 2076-2615 |
  | Date Added | 11/07/2025, 14:16:45 |
  | Modified | 11/07/2025, 14:16:45 |

  ### Tags:

  - human-animal interaction
  - animal welfare
  - dog
  - saliva
  - animal behavior
  - animal experiment
  - article
  - female
  - male
  - nonhuman
  - human
  - hydrocortisone
  - adult
  - controlled study
  - data analysis software
  - training
  - voice
  - latent period
  - emotionality
  - saliva collector
  - Salivette
  - visual orientation
  - socialization
  - wolf
  - vocalization
  - acoustics
  - analytical equipment
  - Canis lupus
  - data visualization
  - frequency analysis
  - hydrocellulose sponge
  - plastic tube
  - positive reinforcement training
  - Raven Pro 1.5
  - reinforcement (psychology)
  - spectroscopy
  - speech
  - subspecies
  - surgical sponge
  - tail
  - time perception
  - trainer

  ### Attachments

  - Full Text (HTML)
- ## The Power of Discourse: Associations between Trainers’ Speech and the Responses of Socialized Wolves and Dogs to Training

  |  |  |
  | --- | --- |
  | Item Type | Journal Article |
  | Author | M.G.B. Fonseca |
  | Author | H.O. Hilário |
  | Author | K. Kotrschal |
  | Author | F. Range |
  | Author | Z. Virányi |
  | Author | M.H.L. Duarte |
  | Author | L.C.G. Pereira |
  | Author | A.D.S. Vasconcellos |
  | Abstract | In a previous study, we found that Positive Reinforcement Training reduced cortisol of wolves and dogs; however, this effect varied across trainer–animal dyads. Here we investigate whether and how the trainers’ use of speech may contribute to this effect. Dogs’ great interest in high-pitched, intense speech (also known as Dog Directed Speech) has already been reported, but whether and how wolves respond similarly/differently to voice characteristics has never been studied before. We analyzed 270 training sessions, conducted by five trainers, with nine mixed-breed dogs and nine wolves, all human-socialized. Through Generalized Linear Mixed Models, we analyzed the effects of (a) three speech categories (nice, neutral, reprehensive) and laugh; and (b) acoustic characteristics of trainers’ voices on animals’ responses (correct responses, latency, orientation, time at less than 1 m, non-training behaviors, tail position/movements, cortisol variation). In both subspecies, tail wagging occurred more often in sessions with longer durations of nice speech, and less often in sessions with reprehensive speech. For dogs, the duration of reprehensive speech within a session was also negatively related to correct responses. For wolves, retreat time was associated with more reprehensive speech, whereas duration of nice speech was positively associated with time spent within one meter from the trainer. In addition, most dog behavioral responses were associated with higher average intonations within sessions, while wolf responses were correlated with lower intonations within sessions. We did not find any effects of the variables considered on cortisol variation. Our study highlights the relevance of voice tone and speech in a training context on animals’ performances and emotional reactions. |
  | Date | 2023 |
  | Language | English |
  | Archive | Embase |
  | URL | https://www.embase.com/search/results?subaction=viewrecord&id=L2022294333&from=export |
  | Volume | 13 |
  | Publication | Animals |
  | DOI | 10.3390/ani13061071 |
  | Issue | 6 |
  | Journal Abbr | Animals |
  | ISSN | 2076-2615 |
  | Date Added | 11/07/2025, 14:18:18 |
  | Modified | 11/07/2025, 14:18:18 |

  ### Tags:

  - human-animal interaction
  - animal welfare
  - dog
  - saliva
  - animal behavior
  - animal experiment
  - article
  - female
  - male
  - nonhuman
  - human
  - hydrocortisone
  - adult
  - controlled study
  - data analysis software
  - training
  - voice
  - latent period
  - emotionality
  - saliva collector
  - Salivette
  - visual orientation
  - socialization
  - wolf
  - vocalization
  - acoustics
  - analytical equipment
  - Canis lupus
  - data visualization
  - frequency analysis
  - hydrocellulose sponge
  - plastic tube
  - positive reinforcement training
  - Raven Pro 1.5
  - reinforcement (psychology)
  - spectroscopy
  - speech
  - subspecies
  - surgical sponge
  - tail
  - time perception
  - trainer

  ### Attachments

  - Full Text (HTML)
- ## The Effect of Therapy Dogs on Preoperative Anxiety

  |  |  |
  | --- | --- |
  | Item Type | Journal Article |
  | Author | P. Foerder |
  | Author | M. Royer |
  | Abstract | Animal-assisted interventions have been used to improve human psychological and physiological wellbeing. Research studies on the effects of therapy dogs have found that the companionship of a dog significantly decreases stress levels in many different situations, including health care facilities. We investigated the effects of therapy dogs on adults’ anxiety before going into day surgery. Participants were patients from a Surgical Ambulatory Care Unit. Four conditions were compared to examine the effects of dogs on the patients’ stress: a therapy dog and handler team, a stuffed dog and handler, a person without a dog, or no visitor. Before and after these visits, the heart rate and blood pressure of the patient was taken. Patients also completed the State-Trait Anxiety Inventory short form (STAI) and a brief demographics survey. No change was found in blood pressure or heart rate in any of the conditions. Based on the STAI scores, we found a statistically significant reduction in anxiety for any intervention over no visit at all. We also found that, although there was no statistical difference between the effects of the real dog and the stuffed toy dog, the stuffed dog condition lowered anxiety enough to show a statistically significant reduction compared with no visitor. Our research is one of the few studies to compare a live dog to a similar size and breed stuffed toy dog. Our results indicate that, in the absence of a real dog, a stuffed toy dog may have equivalent therapeutic value. Our study provides further knowledge on the effects of dog-assisted interventions on medical patients and indicates avenues for future research. |
  | Date | 2021 |
  | Language | English |
  | Archive | Embase |
  | URL | https://www.embase.com/search/results?subaction=viewrecord&id=L2011401725&from=export |
  | Volume | 34 |
  | Pages | 659-670 |
  | Publication | Anthrozoos |
  | DOI | 10.1080/08927936.2021.1914440 |
  | Issue | 5 |
  | Journal Abbr | Anthrozoos |
  | ISSN | 1753-0377 |
  | Date Added | 11/07/2025, 12:10:50 |
  | Modified | 11/07/2025, 12:10:50 |

  ### Tags:

  - blood pressure
  - human-animal interaction
  - heart rate
  - dog
  - animal experiment
  - article
  - female
  - male
  - nonhuman
  - physiological stress
  - human
  - adult
  - controlled study
  - animal model
  - wellbeing
  - health care facility
  - ambulatory care
  - ambulatory surgery
  - breed
  - demography
  - State Trait Anxiety Inventory
  - surgery
  - therapy effect

  ### Attachments

  - Full Text (HTML)
- ## Longitudinal fecal hormone monitoring of adrenocortical function in zoo housed fishing cats (Prionailurus viverrinus) during institutional transfers and breeding introductions

  |  |  |
  | --- | --- |
  | Item Type | Journal Article |
  | Author | J.M. Fazio |
  | Author | E.W. Freeman |
  | Author | E. Bauer |
  | Author | L. Rockwood |
  | Author | J.L. Brown |
  | Author | K. Hope |
  | Author | J. Siegal-Willott |
  | Author | E.C.M. Parsons |
  | Abstract | The ex situ population of fishing cats (Prionailurus viverrinus) has become increasingly important for the conservation of this species. Unfortunately, captivity-induced stress is a concern and potential factor for lack of breeding success in this small felid, resulting in an unsustainable population. The objectives of this study were to: 1) validate an enzyme immunoassay for monitoring of fecal glucocorticoid metabolite (FGM) concentrations in the fishing cat; 2) identify potential exogenous stressors in the captive environment; 3) pinpoint management techniques that may lower FGM concentrations; and 4) determine if FGM concentrations are related to breeding success. Through a successful adrenocorticotrophic hormone challenge and additional laboratory methods, a cortisol enzyme immunoassay was validated as an effective tool for detecting FGM in this species. Between 2010 and 2013, longitudinal FGM monitoring was conducted in 26 fishing cats in the North American Species Survival Plan®. Exogenous stressors that elevated FGM concentrations included: chemical immobilizations; permanent transfers between facilities; construction; facility events; and fights/aggression among breeding pairs. Management factors that lowered FGM concentrations included: increased animal-keeper interaction through formal training; and providing indoor, off-exhibit, holding areas. In addition, social housing of individuals (either established breeding pairs or same sex pairs) decreased FGM concentrations. Individuals with breeding success (defined as observed copulations during the study period) also had lower FGM concentrations than unsuccessful individuals. Findings indicate that management factors play a role in lowering glucocorticoid (stress) levels in fishing cats, which may ultimately affect breeding success in the ex situ population. |
  | Date | 2020 |
  | Language | English |
  | Archive | Embase |
  | URL | https://www.embase.com/search/results?subaction=viewrecord&id=L2005259904&from=export |
  | Volume | 15 |
  | Publication | PLoS ONE |
  | DOI | 10.1371/journal.pone.0230239 |
  | Issue | 3 |
  | Journal Abbr | PLoS ONE |
  | ISSN | 1932-6203 |
  | Date Added | 11/07/2025, 14:16:58 |
  | Modified | 11/07/2025, 14:16:58 |

  ### Tags:

  - animal experiment
  - article
  - female
  - glucocorticoid
  - male
  - nonhuman
  - hydrocortisone
  - adult
  - hormone determination
  - corticotropin
  - unclassified drug
  - animal model
  - human-animal bond
  - animal housing
  - cat
  - aggression
  - breeding
  - enzyme immunoassay
  - feces analysis
  - adrenal cortex function
  - copulation
  - corticosteroid metabolism
  - environmental stress
  - fecal glucocorticoid metabolite
  - fighting
  - immobilization
  - Prionailurus viverrinus

  ### Attachments

  - Full Text (HTML)
- ## Longitudinal fecal hormone monitoring of adrenocortical function in zoo housed fishing cats (Prionailurus viverrinus) during institutional transfers and breeding introductions

  |  |  |
  | --- | --- |
  | Item Type | Journal Article |
  | Author | J.M. Fazio |
  | Author | E.W. Freeman |
  | Author | E. Bauer |
  | Author | L. Rockwood |
  | Author | J.L. Brown |
  | Author | K. Hope |
  | Author | J. Siegal-Willott |
  | Author | E.C.M. Parsons |
  | Abstract | The ex situ population of fishing cats (Prionailurus viverrinus) has become increasingly important for the conservation of this species. Unfortunately, captivity-induced stress is a concern and potential factor for lack of breeding success in this small felid, resulting in an unsustainable population. The objectives of this study were to: 1) validate an enzyme immunoassay for monitoring of fecal glucocorticoid metabolite (FGM) concentrations in the fishing cat; 2) identify potential exogenous stressors in the captive environment; 3) pinpoint management techniques that may lower FGM concentrations; and 4) determine if FGM concentrations are related to breeding success. Through a successful adrenocorticotrophic hormone challenge and additional laboratory methods, a cortisol enzyme immunoassay was validated as an effective tool for detecting FGM in this species. Between 2010 and 2013, longitudinal FGM monitoring was conducted in 26 fishing cats in the North American Species Survival Plan®. Exogenous stressors that elevated FGM concentrations included: chemical immobilizations; permanent transfers between facilities; construction; facility events; and fights/aggression among breeding pairs. Management factors that lowered FGM concentrations included: increased animal-keeper interaction through formal training; and providing indoor, off-exhibit, holding areas. In addition, social housing of individuals (either established breeding pairs or same sex pairs) decreased FGM concentrations. Individuals with breeding success (defined as observed copulations during the study period) also had lower FGM concentrations than unsuccessful individuals. Findings indicate that management factors play a role in lowering glucocorticoid (stress) levels in fishing cats, which may ultimately affect breeding success in the ex situ population. |
  | Date | 2020 |
  | Language | English |
  | Archive | Embase |
  | URL | https://www.embase.com/search/results?subaction=viewrecord&id=L2005259904&from=export |
  | Volume | 15 |
  | Publication | PLoS ONE |
  | DOI | 10.1371/journal.pone.0230239 |
  | Issue | 3 |
  | Journal Abbr | PLoS ONE |
  | ISSN | 1932-6203 |
  | Date Added | 11/07/2025, 14:18:32 |
  | Modified | 11/07/2025, 14:18:32 |

  ### Tags:

  - animal experiment
  - article
  - female
  - glucocorticoid
  - male
  - nonhuman
  - hydrocortisone
  - adult
  - hormone determination
  - corticotropin
  - unclassified drug
  - animal model
  - human-animal bond
  - animal housing
  - cat
  - aggression
  - breeding
  - enzyme immunoassay
  - feces analysis
  - adrenal cortex function
  - copulation
  - corticosteroid metabolism
  - environmental stress
  - fecal glucocorticoid metabolite
  - fighting
  - immobilization
  - Prionailurus viverrinus

  ### Attachments

  - Full Text (HTML)
- ## Short-term equine interaction for reducing test anxiety and facilitating coping skill development in college students during examination periods: A preliminary study

  |  |  |
  | --- | --- |
  | Item Type | Journal Article |
  | Author | K. Everett |
  | Author | M.M. Friend |
  | Author | E. Farnlacher |
  | Author | A. Hilliard |
  | Author | M.C. Nicodemus |
  | Author | C.A. Cavinder |
  | Author | K. Holtcamp |
  | Author | D. Jousan |
  | Abstract | Mental health concerns are common among college students and more prevalent during examination periods when stress and anxiety are heightened. While the benefits of equine interaction have been reported, the impact of short-term equine interaction on coping skills valuable for managing test anxiety have yet to be studied. Therefore, the purpose of this preliminary study was to evaluate the impact of short-term equine interaction during the college examination period on development of coping skills and anxiety levels associated with testing, both self-reported and those reflected in physiological measures. Full-time college students engaged in a one-hour equine interaction session held on campus at the onset of final exam week. Participants completed a survey targeting perception of anxiety levels and coping skills. Heart rate and salivary cortisol measurements were collected pre- and post-session. Paired t-tests were performed between pre- and post- session measures and correlations between survey answers and physiological measures determined. Significant reductions were found in all areas of anxiety (Overall Academics P = 0.0003, Final Exam Preparation P = 0.0003, Taking Final Exam P = 0.0003). The reduction in exam-related anxiety was positively correlated to cortisol concentrations (r = 0.69, P = 0.03) with lower concentrations (P = 0.05) and increased heart rate (P = 0.003) post-session. Regarding coping skills, improvements were observed post-session in assertiveness (P = 0.008). Skill development coupled with reduced anxiety supports the potential benefits short-term equine interaction can offer students during the high stress periods, but these findings should be confirmed with more in-depth study. |
  | Date | 2024 |
  | Language | English |
  | Archive | Embase |
  | URL | https://www.embase.com/search/results?subaction=viewrecord&id=L2032143649&from=export |
  | Volume | 137 |
  | Publication | Journal of Equine Veterinary Science |
  | DOI | 10.1016/j.jevs.2024.105091 |
  | Issue | (Everett K.; Farnlacher E.; Hilliard A.; Nicodemus M.C., mnicodemus@ads.msstate.edu; Cavinder C.A.; Jousan D.) Department of Animal and Dairy Sciences, Mississippi State University, Box 9815, Mississippi State, MS, United States |
  | Journal Abbr | J. Equine Vet. Sci. |
  | ISSN | 0737-0806 |
  | Date Added | 11/07/2025, 11:48:34 |
  | Modified | 11/07/2025, 11:48:34 |

  ### Tags:

  - human-animal interaction
  - heart rate
  - article
  - assertiveness
  - college student
  - coping
  - curriculum development
  - human
  - hydrocortisone
  - self concept
  - self report
  - short term equine interaction
  - test anxiety
  - test retest reliability

  ### Attachments

  - Full Text (HTML)
- ## Short-term equine interaction for reducing test anxiety and facilitating coping skill development in college students during examination periods: A preliminary study

  |  |  |
  | --- | --- |
  | Item Type | Journal Article |
  | Author | K. Everett |
  | Author | M.M. Friend |
  | Author | E. Farnlacher |
  | Author | A. Hilliard |
  | Author | M.C. Nicodemus |
  | Author | C.A. Cavinder |
  | Author | K. Holtcamp |
  | Author | D. Jousan |
  | Abstract | Mental health concerns are common among college students and more prevalent during examination periods when stress and anxiety are heightened. While the benefits of equine interaction have been reported, the impact of short-term equine interaction on coping skills valuable for managing test anxiety have yet to be studied. Therefore, the purpose of this preliminary study was to evaluate the impact of short-term equine interaction during the college examination period on development of coping skills and anxiety levels associated with testing, both self-reported and those reflected in physiological measures. Full-time college students engaged in a one-hour equine interaction session held on campus at the onset of final exam week. Participants completed a survey targeting perception of anxiety levels and coping skills. Heart rate and salivary cortisol measurements were collected pre- and post-session. Paired t-tests were performed between pre- and post- session measures and correlations between survey answers and physiological measures determined. Significant reductions were found in all areas of anxiety (Overall Academics P = 0.0003, Final Exam Preparation P = 0.0003, Taking Final Exam P = 0.0003). The reduction in exam-related anxiety was positively correlated to cortisol concentrations (r = 0.69, P = 0.03) with lower concentrations (P = 0.05) and increased heart rate (P = 0.003) post-session. Regarding coping skills, improvements were observed post-session in assertiveness (P = 0.008). Skill development coupled with reduced anxiety supports the potential benefits short-term equine interaction can offer students during the high stress periods, but these findings should be confirmed with more in-depth study. |
  | Date | 2024 |
  | Language | English |
  | Archive | Embase |
  | URL | https://www.embase.com/search/results?subaction=viewrecord&id=L2032143649&from=export |
  | Volume | 137 |
  | Publication | Journal of Equine Veterinary Science |
  | DOI | 10.1016/j.jevs.2024.105091 |
  | Issue | (Everett K.; Farnlacher E.; Hilliard A.; Nicodemus M.C., mnicodemus@ads.msstate.edu; Cavinder C.A.; Jousan D.) Department of Animal and Dairy Sciences, Mississippi State University, Box 9815, Mississippi State, MS, United States |
  | Journal Abbr | J. Equine Vet. Sci. |
  | ISSN | 0737-0806 |
  | Date Added | 11/07/2025, 11:53:19 |
  | Modified | 11/07/2025, 11:53:19 |

  ### Tags:

  - human-animal interaction
  - heart rate
  - article
  - assertiveness
  - college student
  - coping
  - curriculum development
  - human
  - hydrocortisone
  - self concept
  - self report
  - short term equine interaction
  - test anxiety
  - test retest reliability

  ### Attachments

  - Full Text (HTML)
- ## Short-term equine interaction for reducing test anxiety and facilitating coping skill development in college students during examination periods: A preliminary study

  |  |  |
  | --- | --- |
  | Item Type | Journal Article |
  | Author | K. Everett |
  | Author | M.M. Friend |
  | Author | E. Farnlacher |
  | Author | A. Hilliard |
  | Author | M.C. Nicodemus |
  | Author | C.A. Cavinder |
  | Author | K. Holtcamp |
  | Author | D. Jousan |
  | Abstract | Mental health concerns are common among college students and more prevalent during examination periods when stress and anxiety are heightened. While the benefits of equine interaction have been reported, the impact of short-term equine interaction on coping skills valuable for managing test anxiety have yet to be studied. Therefore, the purpose of this preliminary study was to evaluate the impact of short-term equine interaction during the college examination period on development of coping skills and anxiety levels associated with testing, both self-reported and those reflected in physiological measures. Full-time college students engaged in a one-hour equine interaction session held on campus at the onset of final exam week. Participants completed a survey targeting perception of anxiety levels and coping skills. Heart rate and salivary cortisol measurements were collected pre- and post-session. Paired t-tests were performed between pre- and post- session measures and correlations between survey answers and physiological measures determined. Significant reductions were found in all areas of anxiety (Overall Academics P = 0.0003, Final Exam Preparation P = 0.0003, Taking Final Exam P = 0.0003). The reduction in exam-related anxiety was positively correlated to cortisol concentrations (r = 0.69, P = 0.03) with lower concentrations (P = 0.05) and increased heart rate (P = 0.003) post-session. Regarding coping skills, improvements were observed post-session in assertiveness (P = 0.008). Skill development coupled with reduced anxiety supports the potential benefits short-term equine interaction can offer students during the high stress periods, but these findings should be confirmed with more in-depth study. |
  | Date | 2024 |
  | Language | English |
  | Archive | Embase |
  | URL | https://www.embase.com/search/results?subaction=viewrecord&id=L2032143649&from=export |
  | Volume | 137 |
  | Publication | Journal of Equine Veterinary Science |
  | DOI | 10.1016/j.jevs.2024.105091 |
  | Issue | (Everett K.; Farnlacher E.; Hilliard A.; Nicodemus M.C., mnicodemus@ads.msstate.edu; Cavinder C.A.; Jousan D.) Department of Animal and Dairy Sciences, Mississippi State University, Box 9815, Mississippi State, MS, United States |
  | Journal Abbr | J. Equine Vet. Sci. |
  | ISSN | 0737-0806 |
  | Date Added | 11/07/2025, 12:10:40 |
  | Modified | 11/07/2025, 12:10:40 |

  ### Tags:

  - human-animal interaction
  - heart rate
  - article
  - assertiveness
  - college student
  - coping
  - curriculum development
  - human
  - hydrocortisone
  - self concept
  - self report
  - short term equine interaction
  - test anxiety
  - test retest reliability

  ### Attachments

  - Full Text (HTML)
- ## Short-term equine interaction for reducing test anxiety and facilitating coping skill development in college students during examination periods: A preliminary study

  |  |  |
  | --- | --- |
  | Item Type | Journal Article |
  | Author | K. Everett |
  | Author | M.M. Friend |
  | Author | E. Farnlacher |
  | Author | A. Hilliard |
  | Author | M.C. Nicodemus |
  | Author | C.A. Cavinder |
  | Author | K. Holtcamp |
  | Author | D. Jousan |
  | Abstract | Mental health concerns are common among college students and more prevalent during examination periods when stress and anxiety are heightened. While the benefits of equine interaction have been reported, the impact of short-term equine interaction on coping skills valuable for managing test anxiety have yet to be studied. Therefore, the purpose of this preliminary study was to evaluate the impact of short-term equine interaction during the college examination period on development of coping skills and anxiety levels associated with testing, both self-reported and those reflected in physiological measures. Full-time college students engaged in a one-hour equine interaction session held on campus at the onset of final exam week. Participants completed a survey targeting perception of anxiety levels and coping skills. Heart rate and salivary cortisol measurements were collected pre- and post-session. Paired t-tests were performed between pre- and post- session measures and correlations between survey answers and physiological measures determined. Significant reductions were found in all areas of anxiety (Overall Academics P = 0.0003, Final Exam Preparation P = 0.0003, Taking Final Exam P = 0.0003). The reduction in exam-related anxiety was positively correlated to cortisol concentrations (r = 0.69, P = 0.03) with lower concentrations (P = 0.05) and increased heart rate (P = 0.003) post-session. Regarding coping skills, improvements were observed post-session in assertiveness (P = 0.008). Skill development coupled with reduced anxiety supports the potential benefits short-term equine interaction can offer students during the high stress periods, but these findings should be confirmed with more in-depth study. |
  | Date | 2024 |
  | Language | English |
  | Archive | Embase |
  | URL | https://www.embase.com/search/results?subaction=viewrecord&id=L2032143649&from=export |
  | Volume | 137 |
  | Publication | Journal of Equine Veterinary Science |
  | DOI | 10.1016/j.jevs.2024.105091 |
  | Issue | (Everett K.; Farnlacher E.; Hilliard A.; Nicodemus M.C., mnicodemus@ads.msstate.edu; Cavinder C.A.; Jousan D.) Department of Animal and Dairy Sciences, Mississippi State University, Box 9815, Mississippi State, MS, United States |
  | Journal Abbr | J. Equine Vet. Sci. |
  | ISSN | 0737-0806 |
  | Date Added | 11/07/2025, 14:15:28 |
  | Modified | 11/07/2025, 14:15:28 |

  ### Tags:

  - human-animal interaction
  - heart rate
  - article
  - assertiveness
  - college student
  - coping
  - curriculum development
  - human
  - hydrocortisone
  - self concept
  - self report
  - short term equine interaction
  - test anxiety
  - test retest reliability

  ### Attachments

  - Full Text (HTML)
- ## Short-term equine interaction for reducing test anxiety and facilitating coping skill development in college students during examination periods: A preliminary study

  |  |  |
  | --- | --- |
  | Item Type | Journal Article |
  | Author | K. Everett |
  | Author | M.M. Friend |
  | Author | E. Farnlacher |
  | Author | A. Hilliard |
  | Author | M.C. Nicodemus |
  | Author | C.A. Cavinder |
  | Author | K. Holtcamp |
  | Author | D. Jousan |
  | Abstract | Mental health concerns are common among college students and more prevalent during examination periods when stress and anxiety are heightened. While the benefits of equine interaction have been reported, the impact of short-term equine interaction on coping skills valuable for managing test anxiety have yet to be studied. Therefore, the purpose of this preliminary study was to evaluate the impact of short-term equine interaction during the college examination period on development of coping skills and anxiety levels associated with testing, both self-reported and those reflected in physiological measures. Full-time college students engaged in a one-hour equine interaction session held on campus at the onset of final exam week. Participants completed a survey targeting perception of anxiety levels and coping skills. Heart rate and salivary cortisol measurements were collected pre- and post-session. Paired t-tests were performed between pre- and post- session measures and correlations between survey answers and physiological measures determined. Significant reductions were found in all areas of anxiety (Overall Academics P = 0.0003, Final Exam Preparation P = 0.0003, Taking Final Exam P = 0.0003). The reduction in exam-related anxiety was positively correlated to cortisol concentrations (r = 0.69, P = 0.03) with lower concentrations (P = 0.05) and increased heart rate (P = 0.003) post-session. Regarding coping skills, improvements were observed post-session in assertiveness (P = 0.008). Skill development coupled with reduced anxiety supports the potential benefits short-term equine interaction can offer students during the high stress periods, but these findings should be confirmed with more in-depth study. |
  | Date | 2024 |
  | Language | English |
  | Archive | Embase |
  | URL | https://www.embase.com/search/results?subaction=viewrecord&id=L2032143649&from=export |
  | Volume | 137 |
  | Publication | Journal of Equine Veterinary Science |
  | DOI | 10.1016/j.jevs.2024.105091 |
  | Issue | (Everett K.; Farnlacher E.; Hilliard A.; Nicodemus M.C., mnicodemus@ads.msstate.edu; Cavinder C.A.; Jousan D.) Department of Animal and Dairy Sciences, Mississippi State University, Box 9815, Mississippi State, MS, United States |
  | Journal Abbr | J. Equine Vet. Sci. |
  | ISSN | 0737-0806 |
  | Date Added | 11/07/2025, 14:16:38 |
  | Modified | 11/07/2025, 14:16:38 |

  ### Tags:

  - human-animal interaction
  - heart rate
  - article
  - assertiveness
  - college student
  - coping
  - curriculum development
  - human
  - hydrocortisone
  - self concept
  - self report
  - short term equine interaction
  - test anxiety
  - test retest reliability

  ### Attachments

  - Full Text (HTML)
- ## Short-term equine interaction for reducing test anxiety and facilitating coping skill development in college students during examination periods: A preliminary study

  |  |  |
  | --- | --- |
  | Item Type | Journal Article |
  | Author | K. Everett |
  | Author | M.M. Friend |
  | Author | E. Farnlacher |
  | Author | A. Hilliard |
  | Author | M.C. Nicodemus |
  | Author | C.A. Cavinder |
  | Author | K. Holtcamp |
  | Author | D. Jousan |
  | Abstract | Mental health concerns are common among college students and more prevalent during examination periods when stress and anxiety are heightened. While the benefits of equine interaction have been reported, the impact of short-term equine interaction on coping skills valuable for managing test anxiety have yet to be studied. Therefore, the purpose of this preliminary study was to evaluate the impact of short-term equine interaction during the college examination period on development of coping skills and anxiety levels associated with testing, both self-reported and those reflected in physiological measures. Full-time college students engaged in a one-hour equine interaction session held on campus at the onset of final exam week. Participants completed a survey targeting perception of anxiety levels and coping skills. Heart rate and salivary cortisol measurements were collected pre- and post-session. Paired t-tests were performed between pre- and post- session measures and correlations between survey answers and physiological measures determined. Significant reductions were found in all areas of anxiety (Overall Academics P = 0.0003, Final Exam Preparation P = 0.0003, Taking Final Exam P = 0.0003). The reduction in exam-related anxiety was positively correlated to cortisol concentrations (r = 0.69, P = 0.03) with lower concentrations (P = 0.05) and increased heart rate (P = 0.003) post-session. Regarding coping skills, improvements were observed post-session in assertiveness (P = 0.008). Skill development coupled with reduced anxiety supports the potential benefits short-term equine interaction can offer students during the high stress periods, but these findings should be confirmed with more in-depth study. |
  | Date | 2024 |
  | Language | English |
  | Archive | Embase |
  | URL | https://www.embase.com/search/results?subaction=viewrecord&id=L2032143649&from=export |
  | Volume | 137 |
  | Publication | Journal of Equine Veterinary Science |
  | DOI | 10.1016/j.jevs.2024.105091 |
  | Issue | (Everett K.; Farnlacher E.; Hilliard A.; Nicodemus M.C., mnicodemus@ads.msstate.edu; Cavinder C.A.; Jousan D.) Department of Animal and Dairy Sciences, Mississippi State University, Box 9815, Mississippi State, MS, United States |
  | Journal Abbr | J. Equine Vet. Sci. |
  | ISSN | 0737-0806 |
  | Date Added | 11/07/2025, 14:18:11 |
  | Modified | 11/07/2025, 14:18:11 |

  ### Tags:

  - human-animal interaction
  - heart rate
  - article
  - assertiveness
  - college student
  - coping
  - curriculum development
  - human
  - hydrocortisone
  - self concept
  - self report
  - short term equine interaction
  - test anxiety
  - test retest reliability

  ### Attachments

  - Full Text (HTML)
- ## Effects on Wellbeing of Exposure to Dog Videos Before a Stressor

  |  |  |
  | --- | --- |
  | Item Type | Journal Article |
  | Author | N. Ein |
  | Author | J. Gervasio |
  | Author | M.J. Reed |
  | Author | K. Vickers |
  | Abstract | Animal-assisted intervention (AAI) has been used as a means of stress relief in clinical and general settings; however, animals are not always allowed in certain spaces. Adapting AAI to video or virtual mediums could improve accessibility and is temporally relevant given the recent shift to online interventions. The current study explored: (1) whether an active video (dog or nature) watched before a stressor would improve wellbeing more than tranquil videos; (2) whether exposure to a dog video improves wellbeing more than a nature video; and (3) whether exposure to either a dog or nature video improves outcomes more than exposure to a control video. One hundred and seven undergraduates were randomly assigned to watch one of five videos (active dog, tranquil dog, active nature, tranquil nature, and control) for 3 minutes and then complete a 3-minute stress task. Subjective (anxiety, stress, happiness, relaxation, positive affect, and negative affect) and physiological (blood pressure and heart rate) outcomes were collected at baseline, video, stressor, and recovery time points. Results showed that the activity level of the dog in the video did not influence outcomes. However, relative to the control group, the dog-video condition showed decreases in stress from baseline to video and a smaller decrease in stress from stressor to recovery. Additionally, relative to the nature-video condition, the dog-video condition showed a slightly higher increase in happiness scores from baseline to video. Lastly, relative to the control group, the nature-video condition showed increased relaxation scores from baseline to video and a larger decrease in relaxation scores from video to stressor. This research may inform the development of alternate modes of AAIs. |
  | Date | 2023 |
  | Language | English |
  | Archive | Embase |
  | URL | https://www.embase.com/search/results?subaction=viewrecord&id=L2020468718&from=export |
  | Volume | 36 |
  | Pages | 349-367 |
  | Publication | Anthrozoos |
  | DOI | 10.1080/08927936.2022.2149925 |
  | Issue | 3 |
  | Journal Abbr | Anthrozoos |
  | ISSN | 1753-0377 |
  | Date Added | 11/07/2025, 11:53:23 |
  | Modified | 11/07/2025, 11:53:23 |

  ### Tags:

  - blood pressure
  - human-animal interaction
  - heart rate
  - dog
  - anxiety
  - animal experiment
  - article
  - nonhuman
  - physiological stress
  - human
  - controlled study
  - leisure
  - wellbeing
  - videorecording
  - happiness
  - web-based intervention

  ### Attachments

  - Full Text (HTML)
- ## Effects on Wellbeing of Exposure to Dog Videos Before a Stressor

  |  |  |
  | --- | --- |
  | Item Type | Journal Article |
  | Author | N. Ein |
  | Author | J. Gervasio |
  | Author | M.J. Reed |
  | Author | K. Vickers |
  | Abstract | Animal-assisted intervention (AAI) has been used as a means of stress relief in clinical and general settings; however, animals are not always allowed in certain spaces. Adapting AAI to video or virtual mediums could improve accessibility and is temporally relevant given the recent shift to online interventions. The current study explored: (1) whether an active video (dog or nature) watched before a stressor would improve wellbeing more than tranquil videos; (2) whether exposure to a dog video improves wellbeing more than a nature video; and (3) whether exposure to either a dog or nature video improves outcomes more than exposure to a control video. One hundred and seven undergraduates were randomly assigned to watch one of five videos (active dog, tranquil dog, active nature, tranquil nature, and control) for 3 minutes and then complete a 3-minute stress task. Subjective (anxiety, stress, happiness, relaxation, positive affect, and negative affect) and physiological (blood pressure and heart rate) outcomes were collected at baseline, video, stressor, and recovery time points. Results showed that the activity level of the dog in the video did not influence outcomes. However, relative to the control group, the dog-video condition showed decreases in stress from baseline to video and a smaller decrease in stress from stressor to recovery. Additionally, relative to the nature-video condition, the dog-video condition showed a slightly higher increase in happiness scores from baseline to video. Lastly, relative to the control group, the nature-video condition showed increased relaxation scores from baseline to video and a larger decrease in relaxation scores from video to stressor. This research may inform the development of alternate modes of AAIs. |
  | Date | 2023 |
  | Language | English |
  | Archive | Embase |
  | URL | https://www.embase.com/search/results?subaction=viewrecord&id=L2020468718&from=export |
  | Volume | 36 |
  | Pages | 349-367 |
  | Publication | Anthrozoos |
  | DOI | 10.1080/08927936.2022.2149925 |
  | Issue | 3 |
  | Journal Abbr | Anthrozoos |
  | ISSN | 1753-0377 |
  | Date Added | 11/07/2025, 12:10:45 |
  | Modified | 11/07/2025, 12:10:45 |

  ### Tags:

  - blood pressure
  - human-animal interaction
  - heart rate
  - dog
  - anxiety
  - animal experiment
  - article
  - nonhuman
  - physiological stress
  - human
  - controlled study
  - leisure
  - wellbeing
  - videorecording
  - happiness
  - web-based intervention

  ### Attachments

  - Full Text (HTML)
- ## The Effect of Dog Videos on Subjective and Physiological Responses to Stress

  |  |  |
  | --- | --- |
  | Item Type | Journal Article |
  | Author | N. Ein |
  | Author | M.J. Reed |
  | Author | K. Vickers |
  | Abstract | Research indicates that animal-assisted therapy programs can reduce stress responses. However, animals are not always permitted in public settings. Thus, alternative forms to the physical presence of an animal could be beneficial. The objective of this study was to determine (1) whether exposure to an active-dog video can help improve subjective and physiological responses to stress more than a tranquil-dog video, (2) whether exposure to dog videos can improve subjective and physiological stress responses more than nature videos, and (3) whether exposure to dog (and nature) videos can improve subjective and physiological stress responses more than a control video. Participants (n = 103; female = 78, male = 25) completed a stressful task and were randomly assigned to watch one of five videos: active dog (dog playing with a toy), tranquil dog (dog lying down quietly), active nature (fast-paced waterfall in a forest), tranquil nature (slow-moving stream in a forest), or blank screen (control video; a video of a black screen). Improvements in subjective (i.e., decrease in stress, anxiety, negative affect, and/or increase in happiness, relaxation, positive affect) and physiological (decrease in heart rate and blood pressure) responses to the stressor were examined. This study found no evidence that the active-dog video improved subjective or physiological responses more than the tranquil-dog video. However, this study found evidence that dog videos can decrease subjective anxiety and increase positive affect more than nature videos. Similarly, this study also found that dog videos can decrease subjective anxiety and increase happiness and positive affect more than can the control video. The effects of the dog videos and nature videos (and control video) on the remaining subjective measures and all physiological responses did not differ. Together, the results show some evidence that dog videos may be better at improving subjective anxiety, happiness, and positive affect responses than nature and/or control videos. However, the results did not show evidence that dog videos could alleviate any physiological responses more than the other videos. Practical applications of these findings include how to improve subjective anxiety and affect responses in public settings (e.g., universities) when animals are not allowed. |
  | Date | 2022 |
  | Language | English |
  | Archive | Embase |
  | URL | https://www.embase.com/search/results?subaction=viewrecord&id=L2014244928&from=export |
  | Volume | 35 |
  | Pages | 463-482 |
  | Publication | Anthrozoos |
  | DOI | 10.1080/08927936.2021.1999606 |
  | Issue | 3 |
  | Journal Abbr | Anthrozoos |
  | ISSN | 1753-0377 |
  | Date Added | 11/07/2025, 12:10:49 |
  | Modified | 11/07/2025, 12:10:49 |

  ### Tags:

  - blood pressure
  - human-animal interaction
  - heart rate
  - dog
  - anxiety
  - animal experiment
  - article
  - female
  - male
  - nonhuman
  - physiological stress
  - human
  - adult
  - controlled study
  - animal model
  - leisure
  - videorecording
  - happiness
  - forest

  ### Attachments

  - Full Text (HTML)
- ## Calming Effects of Touch in Human, Animal, and Robotic Interaction—Scientific State-of-the-Art and Technical Advances

  |  |  |
  | --- | --- |
  | Item Type | Journal Article |
  | Author | M. Eckstein |
  | Author | I. Mamaev |
  | Author | B. Ditzen |
  | Author | U. Sailer |
  | Abstract | Small everyday gestures such as a tap on the shoulder can affect the way humans feel and act. Touch can have a calming effect and alter the way stress is handled, thereby promoting mental and physical health. Due to current technical advances and the growing role of intelligent robots in households and healthcare, recent research also addressed the potential of robotic touch for stress reduction. In addition, touch by non-human agents such as animals or inanimate objects may have a calming effect. This conceptual article will review a selection of the most relevant studies reporting the physiological, hormonal, neural, and subjective effects of touch on stress, arousal, and negative affect. Robotic systems capable of non-social touch will be assessed together with control strategies and sensor technologies. Parallels and differences of human-to-human touch and human-to-non-human touch will be discussed. We propose that, under appropriate conditions, touch can act as (social) signal for safety, even when the interaction partner is an animal or a machine. We will also outline potential directions for future research and clinical relevance. Thereby, this review can provide a foundation for further investigations into the beneficial contribution of touch by different agents to regulate negative affect and arousal in humans. |
  | Date | 2020 |
  | Language | English |
  | Archive | Embase |
  | URL | https://www.embase.com/search/results?subaction=viewrecord&id=L633401496&from=export |
  | Volume | 11 |
  | Publication | Frontiers in Psychiatry |
  | DOI | 10.3389/fpsyt.2020.555058 |
  | Issue | (Eckstein M., monika.eckstein@med.uni-heidelberg.de; Ditzen B.) Institute of Medical Psychology, University Hospital Heidelberg, and Ruprecht-Karls University Heidelberg, Heidelberg, Germany |
  | Journal Abbr | Front. Psychiatry |
  | ISSN | 1664-0640 |
  | Date Added | 11/07/2025, 12:10:51 |
  | Modified | 11/07/2025, 12:10:51 |

  ### Tags:

  - oxytocin
  - human-animal interaction
  - heart rate variability
  - social behavior
  - nonhuman
  - human
  - hydrocortisone
  - review
  - massage
  - tactile stimulation
  - mental stress
  - fear
  - therapy effect
  - arousal
  - biosafety
  - biosensor
  - conceptual framework
  - experimental study
  - hormone response
  - human machine interface
  - mechanic pressure device
  - medical device
  - medical technology
  - mood disorder
  - nerve conduction
  - neurobiology
  - neuroendocrine system
  - neurophysiology
  - pressure sensation
  - professionalism
  - regulatory mechanism
  - robotics
  - Romanticism
  - therapeutic touch

  ### Attachments

  - Full Text (HTML)
- ## Calming Effects of Touch in Human, Animal, and Robotic Interaction—Scientific State-of-the-Art and Technical Advances

  |  |  |
  | --- | --- |
  | Item Type | Journal Article |
  | Author | M. Eckstein |
  | Author | I. Mamaev |
  | Author | B. Ditzen |
  | Author | U. Sailer |
  | Abstract | Small everyday gestures such as a tap on the shoulder can affect the way humans feel and act. Touch can have a calming effect and alter the way stress is handled, thereby promoting mental and physical health. Due to current technical advances and the growing role of intelligent robots in households and healthcare, recent research also addressed the potential of robotic touch for stress reduction. In addition, touch by non-human agents such as animals or inanimate objects may have a calming effect. This conceptual article will review a selection of the most relevant studies reporting the physiological, hormonal, neural, and subjective effects of touch on stress, arousal, and negative affect. Robotic systems capable of non-social touch will be assessed together with control strategies and sensor technologies. Parallels and differences of human-to-human touch and human-to-non-human touch will be discussed. We propose that, under appropriate conditions, touch can act as (social) signal for safety, even when the interaction partner is an animal or a machine. We will also outline potential directions for future research and clinical relevance. Thereby, this review can provide a foundation for further investigations into the beneficial contribution of touch by different agents to regulate negative affect and arousal in humans. |
  | Date | 2020 |
  | Language | English |
  | Archive | Embase |
  | URL | https://www.embase.com/search/results?subaction=viewrecord&id=L633401496&from=export |
  | Volume | 11 |
  | Publication | Frontiers in Psychiatry |
  | DOI | 10.3389/fpsyt.2020.555058 |
  | Issue | (Eckstein M., monika.eckstein@med.uni-heidelberg.de; Ditzen B.) Institute of Medical Psychology, University Hospital Heidelberg, and Ruprecht-Karls University Heidelberg, Heidelberg, Germany |
  | Journal Abbr | Front. Psychiatry |
  | ISSN | 1664-0640 |
  | Date Added | 11/07/2025, 12:13:28 |
  | Modified | 11/07/2025, 12:13:28 |

  ### Tags:

  - oxytocin
  - human-animal interaction
  - heart rate variability
  - social behavior
  - nonhuman
  - human
  - hydrocortisone
  - review
  - massage
  - tactile stimulation
  - mental stress
  - fear
  - therapy effect
  - arousal
  - biosafety
  - biosensor
  - conceptual framework
  - experimental study
  - hormone response
  - human machine interface
  - mechanic pressure device
  - medical device
  - medical technology
  - mood disorder
  - nerve conduction
  - neurobiology
  - neuroendocrine system
  - neurophysiology
  - pressure sensation
  - professionalism
  - regulatory mechanism
  - robotics
  - Romanticism
  - therapeutic touch

  ### Attachments

  - Full Text (HTML)
- ## Fecal cortisol metabolites in dairy cows: A cross-sectional exploration of associations with animal, stockperson, and farm characteristics

  |  |  |
  | --- | --- |
  | Item Type | Journal Article |
  | Author | A. Ebinghaus |
  | Author | U. Knierim |
  | Author | C. Simantke |
  | Author | R. Palme |
  | Author | S. Ivemeyer |
  | Abstract | To date, little is known about influences on cows’ physiological stress levels on farms. The present study explored associations of fecal cortisol metabolite concentrations (FCM) with (1) farm factors including human–animal contact, (2) cows’ fear behaviors towards humans, and (3) milk production and udder health, involving 25 dairy farms and repeated fecal samples (n = 2625) from 674 focal cows. Farm factors via interviews and observations, avoidance distance (AD) and qualitative behavior assessment (QBA) during a human–animal interaction were recorded. Milk yield and somatic cell scores (SCS) were calculated from milk recordings. Levels of FCMs were in general relatively low. No associations with AD and milk yield could be detected. Correlations between FCMs and QBA and SCS were significant, but on a low level. Against expectations, FCMs were higher, when the farm provided concentrates by hand and habituated heifers to milking, in part possibly due to reversed cause–effect relations. Decreased FCM levels were found on farms that did not separate diseased cows, possibly due to the avoidance of social stress following changes in group structure. Additionally, straw yards compared to raised cubicles and generous compared to suboptimal lying space were associated with decreased levels, underlining the importance of comfort around resting. Moreover, FCMs were decreased with increased human contact time per cow. The different associations detected in this study provide a basis for further experimental investigations that moreover might provide insights into causal relationships. |
  | Date | 2020 |
  | Language | English |
  | Archive | Embase |
  | URL | https://www.embase.com/search/results?subaction=viewrecord&id=L2005159081&from=export |
  | Volume | 10 |
  | Pages | 1-17 |
  | Publication | Animals |
  | DOI | 10.3390/ani10101787 |
  | Issue | 10 |
  | Journal Abbr | Animals |
  | ISSN | 2076-2615 |
  | Date Added | 11/07/2025, 14:16:55 |
  | Modified | 11/07/2025, 14:16:55 |

  ### Tags:

  - human-animal interaction
  - animal welfare
  - article
  - metabolite
  - nonhuman
  - physiological stress
  - hydrocortisone
  - dairy cattle
  - milk yield
  - behavior assessment
  - fear
  - milk production
  - animal husbandry
  - qualitative analysis
  - social stress
  - cross-sectional study
  - feces analysis
  - energy balance
  - farm animal
  - herd
  - housing
  - somatic cell

  ### Attachments

  - Full Text (HTML)
- ## Fecal cortisol metabolites in dairy cows: A cross-sectional exploration of associations with animal, stockperson, and farm characteristics

  |  |  |
  | --- | --- |
  | Item Type | Journal Article |
  | Author | A. Ebinghaus |
  | Author | U. Knierim |
  | Author | C. Simantke |
  | Author | R. Palme |
  | Author | S. Ivemeyer |
  | Abstract | To date, little is known about influences on cows’ physiological stress levels on farms. The present study explored associations of fecal cortisol metabolite concentrations (FCM) with (1) farm factors including human–animal contact, (2) cows’ fear behaviors towards humans, and (3) milk production and udder health, involving 25 dairy farms and repeated fecal samples (n = 2625) from 674 focal cows. Farm factors via interviews and observations, avoidance distance (AD) and qualitative behavior assessment (QBA) during a human–animal interaction were recorded. Milk yield and somatic cell scores (SCS) were calculated from milk recordings. Levels of FCMs were in general relatively low. No associations with AD and milk yield could be detected. Correlations between FCMs and QBA and SCS were significant, but on a low level. Against expectations, FCMs were higher, when the farm provided concentrates by hand and habituated heifers to milking, in part possibly due to reversed cause–effect relations. Decreased FCM levels were found on farms that did not separate diseased cows, possibly due to the avoidance of social stress following changes in group structure. Additionally, straw yards compared to raised cubicles and generous compared to suboptimal lying space were associated with decreased levels, underlining the importance of comfort around resting. Moreover, FCMs were decreased with increased human contact time per cow. The different associations detected in this study provide a basis for further experimental investigations that moreover might provide insights into causal relationships. |
  | Date | 2020 |
  | Language | English |
  | Archive | Embase |
  | URL | https://www.embase.com/search/results?subaction=viewrecord&id=L2005159081&from=export |
  | Volume | 10 |
  | Pages | 1-17 |
  | Publication | Animals |
  | DOI | 10.3390/ani10101787 |
  | Issue | 10 |
  | Journal Abbr | Animals |
  | ISSN | 2076-2615 |
  | Date Added | 11/07/2025, 14:18:29 |
  | Modified | 11/07/2025, 14:18:29 |

  ### Tags:

  - human-animal interaction
  - animal welfare
  - article
  - metabolite
  - nonhuman
  - physiological stress
  - hydrocortisone
  - dairy cattle
  - milk yield
  - behavior assessment
  - fear
  - milk production
  - animal husbandry
  - qualitative analysis
  - social stress
  - cross-sectional study
  - feces analysis
  - energy balance
  - farm animal
  - herd
  - housing
  - somatic cell

  ### Attachments

  - Full Text (HTML)
- ## Clippers are superior to scissors in the collection of hair for chemical analysis in companion dogs: a Dog Aging Project preliminary study

  |  |  |
  | --- | --- |
  | Item Type | Journal Article |
  | Author | B. Ditzler |
  | Author | J.B. Evans |
  | Author | K. Illing |
  | Author | M. Kaeberlein |
  | Author | D.E.L. Promislow |
  | Author | E. Brindle |
  | Author | J.M. Hoffman |
  | Author | K.E. Creevy |
  | Abstract | OBJECTIVE To identify the safest, most efficient method for hair sample collection from companion dogs among clippers, scissors, and razors and to validate obtained samples with cortisol concentration analysis. ANIMALS 25 healthy, privately owned dogs. METHODS 2 hair samples were collected from each dog’s ischiatic region with different implements (scissors, razors, or clippers). The collecting clinician completed a Hair Collection Questionnaire (HCQ) for each sample that compared subjective sample quality, time of collection, restraint needed, and patient experience. Each sample was evaluated by cortisol enzyme immunoassay. RESULTS Clippers had higher overall HCQ scores than scissors, and scissors had higher HCQ scores than razors. Collection was faster for clippers than scissors, and scissors were faster than razors. There were no differences in sample quality between scissors and clippers, and sample quality was lower with razors. There was no difference in restraint needed or patient experience. Collection of long hair had higher HCQ scores than collection of medium and short hair. Collection of hair from dogs with an undercoat had higher HCQ scores than collection of hair from dogs without an undercoat. Dog size had no effect on HCQ score. Hair cortisol concentration did not vary between scissors or clippers (P = .111). Hair color and age did not affect hair cortisol concentration (P = .966 and P = .676, respectively). CLINICAL RELEVANCE Clippers are recommended for hair sample collection from companion dogs. Scissors are an adequate alternative. |
  | Date | 2024 |
  | Language | English |
  | Archive | Embase |
  | URL | https://www.embase.com/search/results?subaction=viewrecord&id=L2029282382&from=export |
  | Volume | 85 |
  | Pages | 1-7 |
  | Publication | American Journal of Veterinary Research |
  | DOI | 10.2460/ajvr.23.12.0293 |
  | Issue | 5 |
  | Journal Abbr | Am. J. Vet. Res. |
  | ISSN | 1943-5681 |
  | Date Added | 11/07/2025, 14:15:29 |
  | Modified | 11/07/2025, 14:15:29 |

  ### Tags:

  - dog
  - article
  - nonhuman
  - hydrocortisone
  - golden retriever
  - pet animal
  - questionnaire
  - volunteer
  - preliminary data
  - human-animal bond
  - Labrador retriever
  - personal experience
  - aging
  - chemical analysis
  - clinical significance
  - clipper
  - color
  - dachshund (dog)
  - enzyme immunoassay
  - German shepherd
  - great dane
  - hair
  - hair collection questionnaire
  - hypothyroidism
  - Jack Russell
  - methanol
  - Pomeranian dog
  - sciatic nerve
  - scissors
  - self help device

  ### Attachments

  - Full Text (HTML)
- ## Clippers are superior to scissors in the collection of hair for chemical analysis in companion dogs: a Dog Aging Project preliminary study

  |  |  |
  | --- | --- |
  | Item Type | Journal Article |
  | Author | B. Ditzler |
  | Author | J.B. Evans |
  | Author | K. Illing |
  | Author | M. Kaeberlein |
  | Author | D.E.L. Promislow |
  | Author | E. Brindle |
  | Author | J.M. Hoffman |
  | Author | K.E. Creevy |
  | Abstract | OBJECTIVE To identify the safest, most efficient method for hair sample collection from companion dogs among clippers, scissors, and razors and to validate obtained samples with cortisol concentration analysis. ANIMALS 25 healthy, privately owned dogs. METHODS 2 hair samples were collected from each dog’s ischiatic region with different implements (scissors, razors, or clippers). The collecting clinician completed a Hair Collection Questionnaire (HCQ) for each sample that compared subjective sample quality, time of collection, restraint needed, and patient experience. Each sample was evaluated by cortisol enzyme immunoassay. RESULTS Clippers had higher overall HCQ scores than scissors, and scissors had higher HCQ scores than razors. Collection was faster for clippers than scissors, and scissors were faster than razors. There were no differences in sample quality between scissors and clippers, and sample quality was lower with razors. There was no difference in restraint needed or patient experience. Collection of long hair had higher HCQ scores than collection of medium and short hair. Collection of hair from dogs with an undercoat had higher HCQ scores than collection of hair from dogs without an undercoat. Dog size had no effect on HCQ score. Hair cortisol concentration did not vary between scissors or clippers (P = .111). Hair color and age did not affect hair cortisol concentration (P = .966 and P = .676, respectively). CLINICAL RELEVANCE Clippers are recommended for hair sample collection from companion dogs. Scissors are an adequate alternative. |
  | Date | 2024 |
  | Language | English |
  | Archive | Embase |
  | URL | https://www.embase.com/search/results?subaction=viewrecord&id=L2029282382&from=export |
  | Volume | 85 |
  | Pages | 1-7 |
  | Publication | American Journal of Veterinary Research |
  | DOI | 10.2460/ajvr.23.12.0293 |
  | Issue | 5 |
  | Journal Abbr | Am. J. Vet. Res. |
  | ISSN | 1943-5681 |
  | Date Added | 11/07/2025, 14:16:39 |
  | Modified | 11/07/2025, 14:16:39 |

  ### Tags:

  - dog
  - article
  - nonhuman
  - hydrocortisone
  - golden retriever
  - pet animal
  - questionnaire
  - volunteer
  - preliminary data
  - human-animal bond
  - Labrador retriever
  - personal experience
  - aging
  - chemical analysis
  - clinical significance
  - clipper
  - color
  - dachshund (dog)
  - enzyme immunoassay
  - German shepherd
  - great dane
  - hair
  - hair collection questionnaire
  - hypothyroidism
  - Jack Russell
  - methanol
  - Pomeranian dog
  - sciatic nerve
  - scissors
  - self help device

  ### Attachments

  - Full Text (HTML)
- ## Clippers are superior to scissors in the collection of hair for chemical analysis in companion dogs: a Dog Aging Project preliminary study

  |  |  |
  | --- | --- |
  | Item Type | Journal Article |
  | Author | B. Ditzler |
  | Author | J.B. Evans |
  | Author | K. Illing |
  | Author | M. Kaeberlein |
  | Author | D.E.L. Promislow |
  | Author | E. Brindle |
  | Author | J.M. Hoffman |
  | Author | K.E. Creevy |
  | Abstract | OBJECTIVE To identify the safest, most efficient method for hair sample collection from companion dogs among clippers, scissors, and razors and to validate obtained samples with cortisol concentration analysis. ANIMALS 25 healthy, privately owned dogs. METHODS 2 hair samples were collected from each dog’s ischiatic region with different implements (scissors, razors, or clippers). The collecting clinician completed a Hair Collection Questionnaire (HCQ) for each sample that compared subjective sample quality, time of collection, restraint needed, and patient experience. Each sample was evaluated by cortisol enzyme immunoassay. RESULTS Clippers had higher overall HCQ scores than scissors, and scissors had higher HCQ scores than razors. Collection was faster for clippers than scissors, and scissors were faster than razors. There were no differences in sample quality between scissors and clippers, and sample quality was lower with razors. There was no difference in restraint needed or patient experience. Collection of long hair had higher HCQ scores than collection of medium and short hair. Collection of hair from dogs with an undercoat had higher HCQ scores than collection of hair from dogs without an undercoat. Dog size had no effect on HCQ score. Hair cortisol concentration did not vary between scissors or clippers (P = .111). Hair color and age did not affect hair cortisol concentration (P = .966 and P = .676, respectively). CLINICAL RELEVANCE Clippers are recommended for hair sample collection from companion dogs. Scissors are an adequate alternative. |
  | Date | 2024 |
  | Language | English |
  | Archive | Embase |
  | URL | https://www.embase.com/search/results?subaction=viewrecord&id=L2029282382&from=export |
  | Volume | 85 |
  | Pages | 1-7 |
  | Publication | American Journal of Veterinary Research |
  | DOI | 10.2460/ajvr.23.12.0293 |
  | Issue | 5 |
  | Journal Abbr | Am. J. Vet. Res. |
  | ISSN | 1943-5681 |
  | Date Added | 11/07/2025, 14:18:12 |
  | Modified | 11/07/2025, 14:18:12 |

  ### Tags:

  - dog
  - article
  - nonhuman
  - hydrocortisone
  - golden retriever
  - pet animal
  - questionnaire
  - volunteer
  - preliminary data
  - human-animal bond
  - Labrador retriever
  - personal experience
  - aging
  - chemical analysis
  - clinical significance
  - clipper
  - color
  - dachshund (dog)
  - enzyme immunoassay
  - German shepherd
  - great dane
  - hair
  - hair collection questionnaire
  - hypothyroidism
  - Jack Russell
  - methanol
  - Pomeranian dog
  - sciatic nerve
  - scissors
  - self help device

  ### Attachments

  - Full Text (HTML)
- ## The power of interspecific sociality: how humans provide social buffering for horses

  |  |  |
  | --- | --- |
  | Item Type | Journal Article |
  | Author | A. Di Lucrezia |
  | Author | A. Scandurra |
  | Author | D. Lotito |
  | Author | V. Iervolino |
  | Author | B. D'Aniello |
  | Author | V. Mastellone |
  | Author | P. Lombardi |
  | Author | C. Pinelli |
  | Abstract | In this study, we assessed the interspecific "social buffering effect" of humans on horses, exploring how human presence influences stress responses in horses in an unfamiliar environment using the "isolation paradigm." We examined nine Haflinger horses under two counterbalanced conditions: with a passive human stranger (social condition) or alone (isolation condition). Stress responses were assessed through cortisol measurements, heart rate monitoring, and behavioral observations. While cortisol levels significantly increased in both conditions, with no notable differences before and after the tests, heart rate data revealed a different pattern. Results indicated that stress generally decreased in both scenarios, impacting heart rate. Initially, during the first five minutes, heart rate was significantly higher in the social condition compared to isolation, but this trend reversed in the following intervals, with heart rate significantly decreasing as interaction with the stranger increased. Positive interaction between time and stranger-directed behaviors suggested the stranger's influence on heart rate strengthened over time. Overall, these finding suggest that while cortisol data did not reflect a social buffering effect, other metrics indicated that human presence effectively reduced stress in horses after a brief adjustment period, supporting the hypothesis that horses can benefit from human presence during stress, after a short adaptation time. This study highlights the complex nature of stress responses in horses and the potential role of humans as social buffers in interspecific contexts. |
  | Date | 2025 |
  | Language | English |
  | Archive | Medline |
  | URL | https://www.embase.com/search/results?subaction=viewrecord&id=L646792917&from=export |
  | Volume | 28 |
  | Pages | 20 |
  | Publication | Animal cognition |
  | DOI | 10.1007/s10071-025-01942-5 |
  | Issue | 1 |
  | Journal Abbr | Anim Cogn |
  | ISSN | 1435-9456 |
  | Date Added | 11/07/2025, 11:53:15 |
  | Modified | 11/07/2025, 11:53:15 |

  ### Tags:

  - horse
  - physiology
  - heart rate
  - social behavior
  - animal behavior
  - female
  - male
  - human
  - hydrocortisone
  - social isolation
  - animal
  - mental stress
  - psychology

  ### Attachments

  - Full Text (HTML)
- ## The power of interspecific sociality: how humans provide social buffering for horses

  |  |  |
  | --- | --- |
  | Item Type | Journal Article |
  | Author | A. Di Lucrezia |
  | Author | A. Scandurra |
  | Author | D. Lotito |
  | Author | V. Iervolino |
  | Author | B. D'Aniello |
  | Author | V. Mastellone |
  | Author | P. Lombardi |
  | Author | C. Pinelli |
  | Abstract | In this study, we assessed the interspecific "social buffering effect" of humans on horses, exploring how human presence influences stress responses in horses in an unfamiliar environment using the "isolation paradigm." We examined nine Haflinger horses under two counterbalanced conditions: with a passive human stranger (social condition) or alone (isolation condition). Stress responses were assessed through cortisol measurements, heart rate monitoring, and behavioral observations. While cortisol levels significantly increased in both conditions, with no notable differences before and after the tests, heart rate data revealed a different pattern. Results indicated that stress generally decreased in both scenarios, impacting heart rate. Initially, during the first five minutes, heart rate was significantly higher in the social condition compared to isolation, but this trend reversed in the following intervals, with heart rate significantly decreasing as interaction with the stranger increased. Positive interaction between time and stranger-directed behaviors suggested the stranger's influence on heart rate strengthened over time. Overall, these finding suggest that while cortisol data did not reflect a social buffering effect, other metrics indicated that human presence effectively reduced stress in horses after a brief adjustment period, supporting the hypothesis that horses can benefit from human presence during stress, after a short adaptation time. This study highlights the complex nature of stress responses in horses and the potential role of humans as social buffers in interspecific contexts. |
  | Date | 2025 |
  | Language | English |
  | Archive | Medline |
  | URL | https://www.embase.com/search/results?subaction=viewrecord&id=L646792917&from=export |
  | Volume | 28 |
  | Pages | 20 |
  | Publication | Animal cognition |
  | DOI | 10.1007/s10071-025-01942-5 |
  | Issue | 1 |
  | Journal Abbr | Anim Cogn |
  | ISSN | 1435-9456 |
  | Date Added | 11/07/2025, 12:10:37 |
  | Modified | 11/07/2025, 12:10:37 |

  ### Tags:

  - horse
  - physiology
  - heart rate
  - social behavior
  - animal behavior
  - female
  - male
  - human
  - hydrocortisone
  - social isolation
  - animal
  - mental stress
  - psychology

  ### Attachments

  - Full Text (HTML)
- ## The power of interspecific sociality: how humans provide social buffering for horses

  |  |  |
  | --- | --- |
  | Item Type | Journal Article |
  | Author | A. Di Lucrezia |
  | Author | A. Scandurra |
  | Author | D. Lotito |
  | Author | V. Iervolino |
  | Author | B. D'Aniello |
  | Author | V. Mastellone |
  | Author | P. Lombardi |
  | Author | C. Pinelli |
  | Abstract | In this study, we assessed the interspecific "social buffering effect" of humans on horses, exploring how human presence influences stress responses in horses in an unfamiliar environment using the "isolation paradigm." We examined nine Haflinger horses under two counterbalanced conditions: with a passive human stranger (social condition) or alone (isolation condition). Stress responses were assessed through cortisol measurements, heart rate monitoring, and behavioral observations. While cortisol levels significantly increased in both conditions, with no notable differences before and after the tests, heart rate data revealed a different pattern. Results indicated that stress generally decreased in both scenarios, impacting heart rate. Initially, during the first five minutes, heart rate was significantly higher in the social condition compared to isolation, but this trend reversed in the following intervals, with heart rate significantly decreasing as interaction with the stranger increased. Positive interaction between time and stranger-directed behaviors suggested the stranger's influence on heart rate strengthened over time. Overall, these finding suggest that while cortisol data did not reflect a social buffering effect, other metrics indicated that human presence effectively reduced stress in horses after a brief adjustment period, supporting the hypothesis that horses can benefit from human presence during stress, after a short adaptation time. This study highlights the complex nature of stress responses in horses and the potential role of humans as social buffers in interspecific contexts. |
  | Date | 2025 |
  | Language | English |
  | Archive | Medline |
  | URL | https://www.embase.com/search/results?subaction=viewrecord&id=L646792917&from=export |
  | Volume | 28 |
  | Pages | 20 |
  | Publication | Animal cognition |
  | DOI | 10.1007/s10071-025-01942-5 |
  | Issue | 1 |
  | Journal Abbr | Anim Cogn |
  | ISSN | 1435-9456 |
  | Date Added | 11/07/2025, 14:15:25 |
  | Modified | 11/07/2025, 14:15:25 |

  ### Tags:

  - horse
  - physiology
  - heart rate
  - social behavior
  - animal behavior
  - female
  - male
  - human
  - hydrocortisone
  - social isolation
  - animal
  - mental stress
  - psychology

  ### Attachments

  - Full Text (HTML)
- ## The power of interspecific sociality: how humans provide social buffering for horses

  |  |  |
  | --- | --- |
  | Item Type | Journal Article |
  | Author | A. Di Lucrezia |
  | Author | A. Scandurra |
  | Author | D. Lotito |
  | Author | V. Iervolino |
  | Author | B. D'Aniello |
  | Author | V. Mastellone |
  | Author | P. Lombardi |
  | Author | C. Pinelli |
  | Abstract | In this study, we assessed the interspecific "social buffering effect" of humans on horses, exploring how human presence influences stress responses in horses in an unfamiliar environment using the "isolation paradigm." We examined nine Haflinger horses under two counterbalanced conditions: with a passive human stranger (social condition) or alone (isolation condition). Stress responses were assessed through cortisol measurements, heart rate monitoring, and behavioral observations. While cortisol levels significantly increased in both conditions, with no notable differences before and after the tests, heart rate data revealed a different pattern. Results indicated that stress generally decreased in both scenarios, impacting heart rate. Initially, during the first five minutes, heart rate was significantly higher in the social condition compared to isolation, but this trend reversed in the following intervals, with heart rate significantly decreasing as interaction with the stranger increased. Positive interaction between time and stranger-directed behaviors suggested the stranger's influence on heart rate strengthened over time. Overall, these finding suggest that while cortisol data did not reflect a social buffering effect, other metrics indicated that human presence effectively reduced stress in horses after a brief adjustment period, supporting the hypothesis that horses can benefit from human presence during stress, after a short adaptation time. This study highlights the complex nature of stress responses in horses and the potential role of humans as social buffers in interspecific contexts. |
  | Date | 2025 |
  | Language | English |
  | Archive | Medline |
  | URL | https://www.embase.com/search/results?subaction=viewrecord&id=L646792917&from=export |
  | Volume | 28 |
  | Pages | 20 |
  | Publication | Animal cognition |
  | DOI | 10.1007/s10071-025-01942-5 |
  | Issue | 1 |
  | Journal Abbr | Anim Cogn |
  | ISSN | 1435-9456 |
  | Date Added | 11/07/2025, 14:16:36 |
  | Modified | 11/07/2025, 14:16:36 |

  ### Tags:

  - horse
  - physiology
  - heart rate
  - social behavior
  - animal behavior
  - female
  - male
  - human
  - hydrocortisone
  - social isolation
  - animal
  - mental stress
  - psychology

  ### Attachments

  - Full Text (HTML)
- ## The power of interspecific sociality: how humans provide social buffering for horses

  |  |  |
  | --- | --- |
  | Item Type | Journal Article |
  | Author | A. Di Lucrezia |
  | Author | A. Scandurra |
  | Author | D. Lotito |
  | Author | V. Iervolino |
  | Author | B. D'Aniello |
  | Author | V. Mastellone |
  | Author | P. Lombardi |
  | Author | C. Pinelli |
  | Abstract | In this study, we assessed the interspecific "social buffering effect" of humans on horses, exploring how human presence influences stress responses in horses in an unfamiliar environment using the "isolation paradigm." We examined nine Haflinger horses under two counterbalanced conditions: with a passive human stranger (social condition) or alone (isolation condition). Stress responses were assessed through cortisol measurements, heart rate monitoring, and behavioral observations. While cortisol levels significantly increased in both conditions, with no notable differences before and after the tests, heart rate data revealed a different pattern. Results indicated that stress generally decreased in both scenarios, impacting heart rate. Initially, during the first five minutes, heart rate was significantly higher in the social condition compared to isolation, but this trend reversed in the following intervals, with heart rate significantly decreasing as interaction with the stranger increased. Positive interaction between time and stranger-directed behaviors suggested the stranger's influence on heart rate strengthened over time. Overall, these finding suggest that while cortisol data did not reflect a social buffering effect, other metrics indicated that human presence effectively reduced stress in horses after a brief adjustment period, supporting the hypothesis that horses can benefit from human presence during stress, after a short adaptation time. This study highlights the complex nature of stress responses in horses and the potential role of humans as social buffers in interspecific contexts. |
  | Date | 2025 |
  | Language | English |
  | Archive | Medline |
  | URL | https://www.embase.com/search/results?subaction=viewrecord&id=L646792917&from=export |
  | Volume | 28 |
  | Pages | 20 |
  | Publication | Animal cognition |
  | DOI | 10.1007/s10071-025-01942-5 |
  | Issue | 1 |
  | Journal Abbr | Anim Cogn |
  | ISSN | 1435-9456 |
  | Date Added | 11/07/2025, 14:18:09 |
  | Modified | 11/07/2025, 14:18:09 |

  ### Tags:

  - horse
  - physiology
  - heart rate
  - social behavior
  - animal behavior
  - female
  - male
  - human
  - hydrocortisone
  - social isolation
  - animal
  - mental stress
  - psychology

  ### Attachments

  - Full Text (HTML)
- ## Serum Oxytocin in Cows Is Positively Correlated with Caregiver Interactions in the Impossible Task Paradigm

  |  |  |
  | --- | --- |
  | Item Type | Journal Article |
  | Author | B. D’aniello |
  | Author | V. Mastellone |
  | Author | C. Pinelli |
  | Author | A. Scandurra |
  | Author | N. Musco |
  | Author | R. Tudisco |
  | Author | M.E. Pero |
  | Author | F. Infascelli |
  | Author | A. Di Lucrezia |
  | Author | P. Lombardi |
  | Abstract | This study explored a possible relationship between the circulating oxytocin, cortisol, and the willingness of dairy cows to engage in social behaviors with humans in an experimental context. The behaviors of twenty-nine cows were recorded during the impossible task paradigm, a procedure aimed at creating a violation of expectancy, in the presence of the caregiver and a stranger. The results showed that serum oxytocin levels were positively correlated with duration and negatively correlated with the latency of the cows’ social interactions with the caregiver. This research provides a clear correlation between circulating oxytocin and a willingness to engage in social contact with the caregiver, excluding the possible effect of different cortisol levels on such behavior. |
  | Date | 2022 |
  | Language | English |
  | Archive | Embase |
  | URL | https://www.embase.com/search/results?subaction=viewrecord&id=L2015387871&from=export |
  | Volume | 12 |
  | Publication | Animals |
  | DOI | 10.3390/ani12030276 |
  | Issue | 3 |
  | Journal Abbr | Animals |
  | ISSN | 2076-2615 |
  | Date Added | 11/07/2025, 12:12:22 |
  | Modified | 11/07/2025, 12:12:22 |

  ### Tags:

  - oxytocin
  - human-animal interaction
  - cow
  - social behavior
  - animal behavior
  - article
  - nonhuman
  - social interaction
  - human
  - hydrocortisone
  - competitive ELISA
  - ELISA kit
  - dairy cattle
  - caregiver
  - enzyme linked immunosorbent assay
  - training
  - task performance

  ### Attachments

  - Full Text (HTML)
- ## Serum Oxytocin in Cows Is Positively Correlated with Caregiver Interactions in the Impossible Task Paradigm

  |  |  |
  | --- | --- |
  | Item Type | Journal Article |
  | Author | B. D’aniello |
  | Author | V. Mastellone |
  | Author | C. Pinelli |
  | Author | A. Scandurra |
  | Author | N. Musco |
  | Author | R. Tudisco |
  | Author | M.E. Pero |
  | Author | F. Infascelli |
  | Author | A. Di Lucrezia |
  | Author | P. Lombardi |
  | Abstract | This study explored a possible relationship between the circulating oxytocin, cortisol, and the willingness of dairy cows to engage in social behaviors with humans in an experimental context. The behaviors of twenty-nine cows were recorded during the impossible task paradigm, a procedure aimed at creating a violation of expectancy, in the presence of the caregiver and a stranger. The results showed that serum oxytocin levels were positively correlated with duration and negatively correlated with the latency of the cows’ social interactions with the caregiver. This research provides a clear correlation between circulating oxytocin and a willingness to engage in social contact with the caregiver, excluding the possible effect of different cortisol levels on such behavior. |
  | Date | 2022 |
  | Language | English |
  | Archive | Embase |
  | URL | https://www.embase.com/search/results?subaction=viewrecord&id=L2015387871&from=export |
  | Volume | 12 |
  | Publication | Animals |
  | DOI | 10.3390/ani12030276 |
  | Issue | 3 |
  | Journal Abbr | Animals |
  | ISSN | 2076-2615 |
  | Date Added | 11/07/2025, 12:13:24 |
  | Modified | 11/07/2025, 12:13:24 |

  ### Tags:

  - oxytocin
  - human-animal interaction
  - cow
  - social behavior
  - animal behavior
  - article
  - nonhuman
  - social interaction
  - human
  - hydrocortisone
  - competitive ELISA
  - ELISA kit
  - dairy cattle
  - caregiver
  - enzyme linked immunosorbent assay
  - training
  - task performance

  ### Attachments

  - Full Text (HTML)
- ## Serum Oxytocin in Cows Is Positively Correlated with Caregiver Interactions in the Impossible Task Paradigm

  |  |  |
  | --- | --- |
  | Item Type | Journal Article |
  | Author | B. D’aniello |
  | Author | V. Mastellone |
  | Author | C. Pinelli |
  | Author | A. Scandurra |
  | Author | N. Musco |
  | Author | R. Tudisco |
  | Author | M.E. Pero |
  | Author | F. Infascelli |
  | Author | A. Di Lucrezia |
  | Author | P. Lombardi |
  | Abstract | This study explored a possible relationship between the circulating oxytocin, cortisol, and the willingness of dairy cows to engage in social behaviors with humans in an experimental context. The behaviors of twenty-nine cows were recorded during the impossible task paradigm, a procedure aimed at creating a violation of expectancy, in the presence of the caregiver and a stranger. The results showed that serum oxytocin levels were positively correlated with duration and negatively correlated with the latency of the cows’ social interactions with the caregiver. This research provides a clear correlation between circulating oxytocin and a willingness to engage in social contact with the caregiver, excluding the possible effect of different cortisol levels on such behavior. |
  | Date | 2022 |
  | Language | English |
  | Archive | Embase |
  | URL | https://www.embase.com/search/results?subaction=viewrecord&id=L2015387871&from=export |
  | Volume | 12 |
  | Publication | Animals |
  | DOI | 10.3390/ani12030276 |
  | Issue | 3 |
  | Journal Abbr | Animals |
  | ISSN | 2076-2615 |
  | Date Added | 11/07/2025, 14:16:49 |
  | Modified | 11/07/2025, 14:16:49 |

  ### Tags:

  - oxytocin
  - human-animal interaction
  - cow
  - social behavior
  - animal behavior
  - article
  - nonhuman
  - social interaction
  - human
  - hydrocortisone
  - competitive ELISA
  - ELISA kit
  - dairy cattle
  - caregiver
  - enzyme linked immunosorbent assay
  - training
  - task performance

  ### Attachments

  - Full Text (HTML)
- ## Serum Oxytocin in Cows Is Positively Correlated with Caregiver Interactions in the Impossible Task Paradigm

  |  |  |
  | --- | --- |
  | Item Type | Journal Article |
  | Author | B. D’aniello |
  | Author | V. Mastellone |
  | Author | C. Pinelli |
  | Author | A. Scandurra |
  | Author | N. Musco |
  | Author | R. Tudisco |
  | Author | M.E. Pero |
  | Author | F. Infascelli |
  | Author | A. Di Lucrezia |
  | Author | P. Lombardi |
  | Abstract | This study explored a possible relationship between the circulating oxytocin, cortisol, and the willingness of dairy cows to engage in social behaviors with humans in an experimental context. The behaviors of twenty-nine cows were recorded during the impossible task paradigm, a procedure aimed at creating a violation of expectancy, in the presence of the caregiver and a stranger. The results showed that serum oxytocin levels were positively correlated with duration and negatively correlated with the latency of the cows’ social interactions with the caregiver. This research provides a clear correlation between circulating oxytocin and a willingness to engage in social contact with the caregiver, excluding the possible effect of different cortisol levels on such behavior. |
  | Date | 2022 |
  | Language | English |
  | Archive | Embase |
  | URL | https://www.embase.com/search/results?subaction=viewrecord&id=L2015387871&from=export |
  | Volume | 12 |
  | Publication | Animals |
  | DOI | 10.3390/ani12030276 |
  | Issue | 3 |
  | Journal Abbr | Animals |
  | ISSN | 2076-2615 |
  | Date Added | 11/07/2025, 14:18:23 |
  | Modified | 11/07/2025, 14:18:23 |

  ### Tags:

  - oxytocin
  - human-animal interaction
  - cow
  - social behavior
  - animal behavior
  - article
  - nonhuman
  - social interaction
  - human
  - hydrocortisone
  - competitive ELISA
  - ELISA kit
  - dairy cattle
  - caregiver
  - enzyme linked immunosorbent assay
  - training
  - task performance

  ### Attachments

  - Full Text (HTML)
- ## Do Workplace Fish Tanks Influence Employee Wellbeing and Cognitive Performance? An Embedded Mixed-Methods Study

  |  |  |
  | --- | --- |
  | Item Type | Journal Article |
  | Author | H. Clements |
  | Author | S. Valentin |
  | Author | N. Jenkins |
  | Author | J. Rankin |
  | Author | N.R. Gee |
  | Author | D. Snellgrove |
  | Author | K.A. Sloman |
  | Abstract | Evidence from “pet-friendly” workplaces highlights potential benefits associated with taking companion animals to work, including reduced stress among employees. Ornamental fishes carry a much lower risk than other companion animals and may be a suitable alternative in situations where other animals would introduce too great a risk (e.g., allergy, accidental injury). The aim of this study was to investigate whether watching an aquarium during the working day influenced employee wellbeing through the reduction of stress and improvements in stress-related outcomes. An embedded mixed-methods study was conducted, comprising two within-subjects trials (Trials A and B) and a qualitative follow-up. Participants were university employees and research students who participated during their working day. In Trial A (n = 30), the immediate effects of watching live fishes on mood, physiological stress, and cognitive performance were compared with the effects of watching a fish video or resting quietly. Although some outcomes improved from pre- to post-activity, there was no evidence that watching fishes (live or video) had greater effects than resting quietly. In Trial B (n = 27), the effects of repeatedly engaging in the same three activities over several weeks were examined. Watching fish videos was associated with improvements in “high pleasure-low arousal” and overall job-related affective wellbeing, but no further effects of condition were found. Qualitative follow-up data collected from a subset of participants from the experimental trials (n = 13) indicated that all three activities may be beneficial as leaving their desks provided detachment from work for a short period. Qualitative data suggested that live fishes were perceived as more engaging, but this did not translate to quantitative findings. Locating fish aquaria within offices (rather than a separate workplace location) may promote wellbeing by encouraging “microbreaks”; further research is needed to investigate this hypothesis. |
  | Date | 2024 |
  | Language | English |
  | Archive | Embase |
  | URL | https://www.embase.com/search/results?subaction=viewrecord&id=L2028178135&from=export |
  | Volume | 37 |
  | Pages | 459-478 |
  | Publication | Anthrozoos |
  | DOI | 10.1080/08927936.2024.2303227 |
  | Issue | 3 |
  | Journal Abbr | Anthrozoos |
  | ISSN | 1753-0377 |
  | Date Added | 11/07/2025, 11:53:20 |
  | Modified | 11/07/2025, 11:53:20 |

  ### Tags:

  - blood pressure
  - heart rate
  - article
  - physiological stress
  - human
  - follow up
  - data analysis
  - human experiment
  - accidental injury
  - allergy
  - confidence interval
  - demographics
  - job satisfaction
  - job stress
  - mental performance
  - ornamental fish
  - Stroop test

  ### Attachments

  - Full Text (HTML)
- ## Do Workplace Fish Tanks Influence Employee Wellbeing and Cognitive Performance? An Embedded Mixed-Methods Study

  |  |  |
  | --- | --- |
  | Item Type | Journal Article |
  | Author | H. Clements |
  | Author | S. Valentin |
  | Author | N. Jenkins |
  | Author | J. Rankin |
  | Author | N.R. Gee |
  | Author | D. Snellgrove |
  | Author | K.A. Sloman |
  | Abstract | Evidence from “pet-friendly” workplaces highlights potential benefits associated with taking companion animals to work, including reduced stress among employees. Ornamental fishes carry a much lower risk than other companion animals and may be a suitable alternative in situations where other animals would introduce too great a risk (e.g., allergy, accidental injury). The aim of this study was to investigate whether watching an aquarium during the working day influenced employee wellbeing through the reduction of stress and improvements in stress-related outcomes. An embedded mixed-methods study was conducted, comprising two within-subjects trials (Trials A and B) and a qualitative follow-up. Participants were university employees and research students who participated during their working day. In Trial A (n = 30), the immediate effects of watching live fishes on mood, physiological stress, and cognitive performance were compared with the effects of watching a fish video or resting quietly. Although some outcomes improved from pre- to post-activity, there was no evidence that watching fishes (live or video) had greater effects than resting quietly. In Trial B (n = 27), the effects of repeatedly engaging in the same three activities over several weeks were examined. Watching fish videos was associated with improvements in “high pleasure-low arousal” and overall job-related affective wellbeing, but no further effects of condition were found. Qualitative follow-up data collected from a subset of participants from the experimental trials (n = 13) indicated that all three activities may be beneficial as leaving their desks provided detachment from work for a short period. Qualitative data suggested that live fishes were perceived as more engaging, but this did not translate to quantitative findings. Locating fish aquaria within offices (rather than a separate workplace location) may promote wellbeing by encouraging “microbreaks”; further research is needed to investigate this hypothesis. |
  | Date | 2024 |
  | Language | English |
  | Archive | Embase |
  | URL | https://www.embase.com/search/results?subaction=viewrecord&id=L2028178135&from=export |
  | Volume | 37 |
  | Pages | 459-478 |
  | Publication | Anthrozoos |
  | DOI | 10.1080/08927936.2024.2303227 |
  | Issue | 3 |
  | Journal Abbr | Anthrozoos |
  | ISSN | 1753-0377 |
  | Date Added | 11/07/2025, 12:10:43 |
  | Modified | 11/07/2025, 12:10:43 |

  ### Tags:

  - blood pressure
  - heart rate
  - article
  - physiological stress
  - human
  - follow up
  - data analysis
  - human experiment
  - accidental injury
  - allergy
  - confidence interval
  - demographics
  - job satisfaction
  - job stress
  - mental performance
  - ornamental fish
  - Stroop test

  ### Attachments

  - Full Text (HTML)
- ## Exploratory Study of Fecal Cortisol, Weight, and Behavior as Measures of Stress and Welfare in Shelter Cats During Assimilation Into Families of Children With Autism Spectrum Disorder

  |  |  |
  | --- | --- |
  | Item Type | Journal Article |
  | Author | G.K. Carlisle |
  | Author | R.A. Johnson |
  | Author | C.S. Koch |
  | Author | L.A. Lyons |
  | Author | Z. Wang |
  | Author | J. Bibbo |
  | Author | N. Cheak-Zamora |
  | Abstract | Background: Cats are a common companion animal (CA) in US households, and many live in families of children with autism spectrum disorder (ASD). The prevalence of ASD is one in 54, and many children have behavior challenges as well as their diagnostic communication disorders. Objective: Benefits of CAs for children with ASD have been identified, but little is known about the welfare of CAs in these homes. This study explored the welfare of cats (N = 10) screened for ideal social and calm temperament using the Feline Temperament Profile (FTP) and adopted by families of children with ASD. Methods: Cat stress was measured using fecal cortisol, weight, and a behavior stress measure (cat stress score). Measures were taken at baseline in the shelter, 2–3 days after adoption, and at weeks 6, 12, and 18. Result: Outcome measures suggested the adopted cats' stress levels did not increase postadoption; however, the small sample size limited analytical power and generalizability. Conclusion: This study provides preliminary evidence for the success of cat adoption by families of children with ASD, when cats have been temperament screened and cat behavior educational information is provided. Further research is warranted to confirm these findings. |
  | Date | 2021 |
  | Language | English |
  | Archive | Embase |
  | URL | https://www.embase.com/search/results?subaction=viewrecord&id=L636009693&from=export |
  | Volume | 8 |
  | Publication | Frontiers in Veterinary Science |
  | DOI | 10.3389/fvets.2021.643803 |
  | Issue | (Carlisle G.K., carlislegk@missouri.edu; Johnson R.A.) University of Missouri College of Veterinary Medicine Research Center for Human-Animal Interaction, Columbia, MO, United States |
  | Journal Abbr | Front. Vet. Sci. |
  | ISSN | 2297-1769 |
  | Date Added | 11/07/2025, 14:16:50 |
  | Modified | 11/07/2025, 14:16:50 |

  ### Tags:

  - adoption
  - animal welfare
  - child
  - animal behavior
  - article
  - nonhuman
  - physiological stress
  - human
  - hydrocortisone
  - body weight
  - pet animal
  - adolescent
  - autism
  - outcome assessment
  - cat
  - stress assessment
  - feces analysis
  - body weight loss
  - cat stress score
  - cat weight
  - temperament
  - weight

  ### Attachments

  - Full Text (HTML)
- ## Exploratory Study of Fecal Cortisol, Weight, and Behavior as Measures of Stress and Welfare in Shelter Cats During Assimilation Into Families of Children With Autism Spectrum Disorder

  |  |  |
  | --- | --- |
  | Item Type | Journal Article |
  | Author | G.K. Carlisle |
  | Author | R.A. Johnson |
  | Author | C.S. Koch |
  | Author | L.A. Lyons |
  | Author | Z. Wang |
  | Author | J. Bibbo |
  | Author | N. Cheak-Zamora |
  | Abstract | Background: Cats are a common companion animal (CA) in US households, and many live in families of children with autism spectrum disorder (ASD). The prevalence of ASD is one in 54, and many children have behavior challenges as well as their diagnostic communication disorders. Objective: Benefits of CAs for children with ASD have been identified, but little is known about the welfare of CAs in these homes. This study explored the welfare of cats (N = 10) screened for ideal social and calm temperament using the Feline Temperament Profile (FTP) and adopted by families of children with ASD. Methods: Cat stress was measured using fecal cortisol, weight, and a behavior stress measure (cat stress score). Measures were taken at baseline in the shelter, 2–3 days after adoption, and at weeks 6, 12, and 18. Result: Outcome measures suggested the adopted cats' stress levels did not increase postadoption; however, the small sample size limited analytical power and generalizability. Conclusion: This study provides preliminary evidence for the success of cat adoption by families of children with ASD, when cats have been temperament screened and cat behavior educational information is provided. Further research is warranted to confirm these findings. |
  | Date | 2021 |
  | Language | English |
  | Archive | Embase |
  | URL | https://www.embase.com/search/results?subaction=viewrecord&id=L636009693&from=export |
  | Volume | 8 |
  | Publication | Frontiers in Veterinary Science |
  | DOI | 10.3389/fvets.2021.643803 |
  | Issue | (Carlisle G.K., carlislegk@missouri.edu; Johnson R.A.) University of Missouri College of Veterinary Medicine Research Center for Human-Animal Interaction, Columbia, MO, United States |
  | Journal Abbr | Front. Vet. Sci. |
  | ISSN | 2297-1769 |
  | Date Added | 11/07/2025, 14:18:24 |
  | Modified | 11/07/2025, 14:18:24 |

  ### Tags:

  - adoption
  - animal welfare
  - child
  - animal behavior
  - article
  - nonhuman
  - physiological stress
  - human
  - hydrocortisone
  - body weight
  - pet animal
  - adolescent
  - autism
  - outcome assessment
  - cat
  - stress assessment
  - feces analysis
  - body weight loss
  - cat stress score
  - cat weight
  - temperament
  - weight

  ### Attachments

  - Full Text (HTML)
- ## Do dogs rescue their owners from a stressful situation? A behavioral and physiological assessment

  |  |  |
  | --- | --- |
  | Item Type | Journal Article |
  | Author | F. Carballo |
  | Author | V. Dzik |
  | Author | E. Freidin |
  | Author | J.P. Damián |
  | Author | E.B. Casanave |
  | Author | M. Bentosela |
  | Abstract | Rescue behavior is considered a type of pro-social response, defined as a voluntary action directed to benefit another individual who is in a stressful or dangerous situation. In two experiments, we investigated whether dogs would rescue their owners when the person was trapped inside a wooden box and emitted clear signs of stress. The performance of these dogs was compared against that of a control group in which the owners remained calm while trapped. In addition, to assess if training modulated this behavior, we tested a group of dogs from the military trained in search and rescue tasks (Experiment 1). Results showed that dogs opened the box more frequently when the owner pretended to be stressed than when calm. Training shortened latencies to open the door but not the frequency of the behavior. In Experiment 2, we investigated if emotional contagion could be a possible mechanism underlying dogs' rescue responses by measuring dogs' behavior, heart rate, and saliva cortisol level in the stressed and calm conditions, and also controlled for obedience by having the calm owners call their pets while trapped. We replicated the findings of Experiment 1 as more dogs opened the door in the stressed owner condition than in the calm condition. In addition, we observed an increase in heart rate across trials in the stressed condition and a decrease across trials in the calm condition, but no differences in cortisol levels or stress-related behaviors between conditions. In brief, we found evidence that approximately half of the dogs without previous training showed spontaneous rescue behaviors directed to their owners. Neither was this behavior motivated by obedience nor by the motivation to re-establish social contact with the owner. We conclude that emotional contagion is a plausible mechanism underlying dogs' rescue behavior in the present protocol. |
  | Date | 2020 |
  | Language | English |
  | Archive | Medline |
  | URL | https://www.embase.com/search/results?subaction=viewrecord&id=L630548825&from=export |
  | Volume | 23 |
  | Pages | 389-403 |
  | Publication | Animal cognition |
  | DOI | 10.1007/s10071-019-01343-5 |
  | Issue | 2 |
  | Journal Abbr | Anim Cogn |
  | ISSN | 1435-9456 |
  | Date Added | 11/07/2025, 14:16:58 |
  | Modified | 11/07/2025, 14:16:58 |

  ### Tags:

  - physiology
  - heart rate
  - dog
  - saliva
  - emotion
  - animal behavior
  - female
  - male
  - human
  - motivation
  - animal
  - mental stress
  - human-animal bond

  ### Attachments

  - Full Text (HTML)
- ## Do dogs rescue their owners from a stressful situation? A behavioral and physiological assessment

  |  |  |
  | --- | --- |
  | Item Type | Journal Article |
  | Author | F. Carballo |
  | Author | V. Dzik |
  | Author | E. Freidin |
  | Author | J.P. Damián |
  | Author | E.B. Casanave |
  | Author | M. Bentosela |
  | Abstract | Rescue behavior is considered a type of pro-social response, defined as a voluntary action directed to benefit another individual who is in a stressful or dangerous situation. In two experiments, we investigated whether dogs would rescue their owners when the person was trapped inside a wooden box and emitted clear signs of stress. The performance of these dogs was compared against that of a control group in which the owners remained calm while trapped. In addition, to assess if training modulated this behavior, we tested a group of dogs from the military trained in search and rescue tasks (Experiment 1). Results showed that dogs opened the box more frequently when the owner pretended to be stressed than when calm. Training shortened latencies to open the door but not the frequency of the behavior. In Experiment 2, we investigated if emotional contagion could be a possible mechanism underlying dogs' rescue responses by measuring dogs' behavior, heart rate, and saliva cortisol level in the stressed and calm conditions, and also controlled for obedience by having the calm owners call their pets while trapped. We replicated the findings of Experiment 1 as more dogs opened the door in the stressed owner condition than in the calm condition. In addition, we observed an increase in heart rate across trials in the stressed condition and a decrease across trials in the calm condition, but no differences in cortisol levels or stress-related behaviors between conditions. In brief, we found evidence that approximately half of the dogs without previous training showed spontaneous rescue behaviors directed to their owners. Neither was this behavior motivated by obedience nor by the motivation to re-establish social contact with the owner. We conclude that emotional contagion is a plausible mechanism underlying dogs' rescue behavior in the present protocol. |
  | Date | 2020 |
  | Language | English |
  | Archive | Medline |
  | URL | https://www.embase.com/search/results?subaction=viewrecord&id=L630548825&from=export |
  | Volume | 23 |
  | Pages | 389-403 |
  | Publication | Animal cognition |
  | DOI | 10.1007/s10071-019-01343-5 |
  | Issue | 2 |
  | Journal Abbr | Anim Cogn |
  | ISSN | 1435-9456 |
  | Date Added | 11/07/2025, 14:18:31 |
  | Modified | 11/07/2025, 14:18:31 |

  ### Tags:

  - physiology
  - heart rate
  - dog
  - saliva
  - emotion
  - animal behavior
  - female
  - male
  - human
  - motivation
  - animal
  - mental stress
  - human-animal bond

  ### Attachments

  - Full Text (HTML)
- ## Concomitant Campylobacteriosis in a Puppy and in Its Caregiver: A One Health Perspective Paradigm in Human-Pet Relationship

  |  |  |
  | --- | --- |
  | Item Type | Journal Article |
  | Author | A. Candellone |
  | Author | P. Badino |
  | Author | F. Girolami |
  | Author | M. Cerquetella |
  | Author | L. Aresu |
  | Author | S. Zoppi |
  | Author | D. Bergero |
  | Author | R. Odore |
  | Author | P. Nebbia |
  | Abstract | Simple Summary: Dogs are considered family members and a growing amount of attention has been dedicated to their nutrition by pet parents during last decades. The administration of unbalanced, poultry-based raw diets (PRD) has represented a rising trend, with some nutritional and microbiological implications on both pets and human wellbeing. Here, we report a case of a laboratory-confirmed Campylobacter (C). jejuni and C. upsaliensis symptomatic infection in a puppy, a French Bouledogue, female, 6 months of age, and fed a PRD, and in its owner. Both recovered after appropriate diag-nostic investigations and treatments: however, hospitalization and a long course of antimicrobials were necessary to fight the multi-drug-resistant infection. This case underlines the potential risk of Campylobacter spp. exposure related to current pet food fads, highlighting the importance of the One Health approach, where veterinarians, physicians and caregivers need to develop effective strategies for the prevention of zoonoses spread. Abstract: We report a case of laboratory-confirmed Campylobacter (C). jejuni and C. upsaliensis symptomatic infection in a puppy, a French Bouledogue, female, 6 months of age, fed a raw, unbalanced, poultry-based diet (RPD), (48.1 CP, 33% EE, 0.3% Ca, 0.5% Phos, 0.5 Ca/P, on a dry-matter basis), and in its owner. Soon after adoption, the pet and the caregiver showed severe gastrointestinal signs and needed hospitalization. Fecal PCR assays, selective cultures, and antibiotic susceptibility testing were performed, and multi-drug resistant C. jejuni and C. upsaliensis were isolated from the feces of both. The same bacteria were also identified by FISH in the dog colonic biopsies collected during endoscopy. The puppy was prescribed a complete commercial diet for growing dogs, (30.00% CP, 21.00% EE, 1.2% Ca; 1% Phos; as fed) and treated with ciprofloxacin. The dog and the man healed uneventfully and tested negative for further fecal PCR analyses. This report focuses on dog nutritional management and explores the potential routes of exposure, with emphasis on emerging outbreaks related to current pet food fads. Our data support the One Health approach, where veterinarians, physicians, and owners are challenged to build effective stewardship to prevent the spread of zoonoses. |
  | Date | 2023 |
  | Language | English |
  | Archive | Embase |
  | URL | https://www.embase.com/search/results?subaction=viewrecord&id=L2023497002&from=export |
  | Volume | 10 |
  | Publication | Veterinary Sciences |
  | DOI | 10.3390/vetsci10040244 |
  | Issue | 4 |
  | Journal Abbr | Vet. Sci. |
  | ISSN | 2306-7381 |
  | Date Added | 11/07/2025, 14:15:34 |
  | Modified | 11/07/2025, 14:15:34 |

  ### Tags:

  - One Health
  - article
  - female
  - nonhuman
  - human
  - hydrocortisone
  - creatinine
  - hospitalization
  - wellbeing
  - caregiver
  - biochemistry
  - animal tissue
  - human-animal bond
  - alanine aminotransferase
  - albumin
  - alkaline phosphatase
  - aspartate aminotransferase
  - calcium
  - glucose
  - sodium
  - leukocyte count
  - veterinarian
  - puppy
  - polymerase chain reaction
  - diet supplementation
  - Addison disease
  - antibiotic sensitivity
  - apple
  - beetroot
  - biochemical equipment
  - blackberry
  - bloody diarrhea
  - Campylobacter jejuni
  - Campylobacter upsaliensis
  - campylobacteriosis
  - caraway
  - carrot
  - ciprofloxacin
  - ciproxin
  - colon biopsy
  - cytology
  - endoscopy
  - enterocolitis
  - erythrocyte
  - feces analysis
  - fennel
  - fish oil
  - flotation
  - fluorescence in situ hybridization
  - gastroenteritis
  - gastrointestinal discomfort
  - ileocolic intussusception
  - laboratory test
  - Lactobacillus fermentum
  - Lactobacillus plantarum
  - Lactobacillus rhamnosus
  - linseed
  - marigold
  - mass spectrometer
  - matrix-assisted laser desorption-ionization mass spectrometry
  - microbiology
  - multidrug resistant infection
  - nutrition
  - nutritional assessment
  - pea
  - phosphorus
  - potassium
  - potato starch
  - poultry
  - probiotic agent
  - spinach
  - sunflower oil
  - urea nitrogen blood level
  - zoonosis

  ### Attachments

  - Full Text (HTML)
- ## Concomitant Campylobacteriosis in a Puppy and in Its Caregiver: A One Health Perspective Paradigm in Human-Pet Relationship

  |  |  |
  | --- | --- |
  | Item Type | Journal Article |
  | Author | A. Candellone |
  | Author | P. Badino |
  | Author | F. Girolami |
  | Author | M. Cerquetella |
  | Author | L. Aresu |
  | Author | S. Zoppi |
  | Author | D. Bergero |
  | Author | R. Odore |
  | Author | P. Nebbia |
  | Abstract | Simple Summary: Dogs are considered family members and a growing amount of attention has been dedicated to their nutrition by pet parents during last decades. The administration of unbalanced, poultry-based raw diets (PRD) has represented a rising trend, with some nutritional and microbiological implications on both pets and human wellbeing. Here, we report a case of a laboratory-confirmed Campylobacter (C). jejuni and C. upsaliensis symptomatic infection in a puppy, a French Bouledogue, female, 6 months of age, and fed a PRD, and in its owner. Both recovered after appropriate diag-nostic investigations and treatments: however, hospitalization and a long course of antimicrobials were necessary to fight the multi-drug-resistant infection. This case underlines the potential risk of Campylobacter spp. exposure related to current pet food fads, highlighting the importance of the One Health approach, where veterinarians, physicians and caregivers need to develop effective strategies for the prevention of zoonoses spread. Abstract: We report a case of laboratory-confirmed Campylobacter (C). jejuni and C. upsaliensis symptomatic infection in a puppy, a French Bouledogue, female, 6 months of age, fed a raw, unbalanced, poultry-based diet (RPD), (48.1 CP, 33% EE, 0.3% Ca, 0.5% Phos, 0.5 Ca/P, on a dry-matter basis), and in its owner. Soon after adoption, the pet and the caregiver showed severe gastrointestinal signs and needed hospitalization. Fecal PCR assays, selective cultures, and antibiotic susceptibility testing were performed, and multi-drug resistant C. jejuni and C. upsaliensis were isolated from the feces of both. The same bacteria were also identified by FISH in the dog colonic biopsies collected during endoscopy. The puppy was prescribed a complete commercial diet for growing dogs, (30.00% CP, 21.00% EE, 1.2% Ca; 1% Phos; as fed) and treated with ciprofloxacin. The dog and the man healed uneventfully and tested negative for further fecal PCR analyses. This report focuses on dog nutritional management and explores the potential routes of exposure, with emphasis on emerging outbreaks related to current pet food fads. Our data support the One Health approach, where veterinarians, physicians, and owners are challenged to build effective stewardship to prevent the spread of zoonoses. |
  | Date | 2023 |
  | Language | English |
  | Archive | Embase |
  | URL | https://www.embase.com/search/results?subaction=viewrecord&id=L2023497002&from=export |
  | Volume | 10 |
  | Publication | Veterinary Sciences |
  | DOI | 10.3390/vetsci10040244 |
  | Issue | 4 |
  | Journal Abbr | Vet. Sci. |
  | ISSN | 2306-7381 |
  | Date Added | 11/07/2025, 14:16:44 |
  | Modified | 11/07/2025, 14:16:44 |

  ### Tags:

  - One Health
  - article
  - female
  - nonhuman
  - human
  - hydrocortisone
  - creatinine
  - hospitalization
  - wellbeing
  - caregiver
  - biochemistry
  - animal tissue
  - human-animal bond
  - alanine aminotransferase
  - albumin
  - alkaline phosphatase
  - aspartate aminotransferase
  - calcium
  - glucose
  - sodium
  - leukocyte count
  - veterinarian
  - puppy
  - polymerase chain reaction
  - diet supplementation
  - Addison disease
  - antibiotic sensitivity
  - apple
  - beetroot
  - biochemical equipment
  - blackberry
  - bloody diarrhea
  - Campylobacter jejuni
  - Campylobacter upsaliensis
  - campylobacteriosis
  - caraway
  - carrot
  - ciprofloxacin
  - ciproxin
  - colon biopsy
  - cytology
  - endoscopy
  - enterocolitis
  - erythrocyte
  - feces analysis
  - fennel
  - fish oil
  - flotation
  - fluorescence in situ hybridization
  - gastroenteritis
  - gastrointestinal discomfort
  - ileocolic intussusception
  - laboratory test
  - Lactobacillus fermentum
  - Lactobacillus plantarum
  - Lactobacillus rhamnosus
  - linseed
  - marigold
  - mass spectrometer
  - matrix-assisted laser desorption-ionization mass spectrometry
  - microbiology
  - multidrug resistant infection
  - nutrition
  - nutritional assessment
  - pea
  - phosphorus
  - potassium
  - potato starch
  - poultry
  - probiotic agent
  - spinach
  - sunflower oil
  - urea nitrogen blood level
  - zoonosis

  ### Attachments

  - Full Text (HTML)
- ## Concomitant Campylobacteriosis in a Puppy and in Its Caregiver: A One Health Perspective Paradigm in Human-Pet Relationship

  |  |  |
  | --- | --- |
  | Item Type | Journal Article |
  | Author | A. Candellone |
  | Author | P. Badino |
  | Author | F. Girolami |
  | Author | M. Cerquetella |
  | Author | L. Aresu |
  | Author | S. Zoppi |
  | Author | D. Bergero |
  | Author | R. Odore |
  | Author | P. Nebbia |
  | Abstract | Simple Summary: Dogs are considered family members and a growing amount of attention has been dedicated to their nutrition by pet parents during last decades. The administration of unbalanced, poultry-based raw diets (PRD) has represented a rising trend, with some nutritional and microbiological implications on both pets and human wellbeing. Here, we report a case of a laboratory-confirmed Campylobacter (C). jejuni and C. upsaliensis symptomatic infection in a puppy, a French Bouledogue, female, 6 months of age, and fed a PRD, and in its owner. Both recovered after appropriate diag-nostic investigations and treatments: however, hospitalization and a long course of antimicrobials were necessary to fight the multi-drug-resistant infection. This case underlines the potential risk of Campylobacter spp. exposure related to current pet food fads, highlighting the importance of the One Health approach, where veterinarians, physicians and caregivers need to develop effective strategies for the prevention of zoonoses spread. Abstract: We report a case of laboratory-confirmed Campylobacter (C). jejuni and C. upsaliensis symptomatic infection in a puppy, a French Bouledogue, female, 6 months of age, fed a raw, unbalanced, poultry-based diet (RPD), (48.1 CP, 33% EE, 0.3% Ca, 0.5% Phos, 0.5 Ca/P, on a dry-matter basis), and in its owner. Soon after adoption, the pet and the caregiver showed severe gastrointestinal signs and needed hospitalization. Fecal PCR assays, selective cultures, and antibiotic susceptibility testing were performed, and multi-drug resistant C. jejuni and C. upsaliensis were isolated from the feces of both. The same bacteria were also identified by FISH in the dog colonic biopsies collected during endoscopy. The puppy was prescribed a complete commercial diet for growing dogs, (30.00% CP, 21.00% EE, 1.2% Ca; 1% Phos; as fed) and treated with ciprofloxacin. The dog and the man healed uneventfully and tested negative for further fecal PCR analyses. This report focuses on dog nutritional management and explores the potential routes of exposure, with emphasis on emerging outbreaks related to current pet food fads. Our data support the One Health approach, where veterinarians, physicians, and owners are challenged to build effective stewardship to prevent the spread of zoonoses. |
  | Date | 2023 |
  | Language | English |
  | Archive | Embase |
  | URL | https://www.embase.com/search/results?subaction=viewrecord&id=L2023497002&from=export |
  | Volume | 10 |
  | Publication | Veterinary Sciences |
  | DOI | 10.3390/vetsci10040244 |
  | Issue | 4 |
  | Journal Abbr | Vet. Sci. |
  | ISSN | 2306-7381 |
  | Date Added | 11/07/2025, 14:18:17 |
  | Modified | 11/07/2025, 14:18:17 |

  ### Tags:

  - One Health
  - article
  - female
  - nonhuman
  - human
  - hydrocortisone
  - creatinine
  - hospitalization
  - wellbeing
  - caregiver
  - biochemistry
  - animal tissue
  - human-animal bond
  - alanine aminotransferase
  - albumin
  - alkaline phosphatase
  - aspartate aminotransferase
  - calcium
  - glucose
  - sodium
  - leukocyte count
  - veterinarian
  - puppy
  - polymerase chain reaction
  - diet supplementation
  - Addison disease
  - antibiotic sensitivity
  - apple
  - beetroot
  - biochemical equipment
  - blackberry
  - bloody diarrhea
  - Campylobacter jejuni
  - Campylobacter upsaliensis
  - campylobacteriosis
  - caraway
  - carrot
  - ciprofloxacin
  - ciproxin
  - colon biopsy
  - cytology
  - endoscopy
  - enterocolitis
  - erythrocyte
  - feces analysis
  - fennel
  - fish oil
  - flotation
  - fluorescence in situ hybridization
  - gastroenteritis
  - gastrointestinal discomfort
  - ileocolic intussusception
  - laboratory test
  - Lactobacillus fermentum
  - Lactobacillus plantarum
  - Lactobacillus rhamnosus
  - linseed
  - marigold
  - mass spectrometer
  - matrix-assisted laser desorption-ionization mass spectrometry
  - microbiology
  - multidrug resistant infection
  - nutrition
  - nutritional assessment
  - pea
  - phosphorus
  - potassium
  - potato starch
  - poultry
  - probiotic agent
  - spinach
  - sunflower oil
  - urea nitrogen blood level
  - zoonosis

  ### Attachments

  - Full Text (HTML)
- ## Affective Implications of Human–Animal Relationship on Pig Welfare: Integrating Non-Linear Heart Rate Variability Measures

  |  |  |
  | --- | --- |
  | Item Type | Journal Article |
  | Author | J. Calderón-Amor |
  | Author | B. Zuleta |
  | Author | M.C. Ceballos |
  | Author | D. Cartes |
  | Author | C.J. Byrd |
  | Author | B. Lecorps |
  | Author | R. Palomo |
  | Author | S.A. Guzmán-Pino |
  | Author | D. Siel |
  | Author | D. Luna |
  | Abstract | The human–animal relationship is crucial for animal welfare. Gentle handling enhances pigs’ comfort while rough handling causes fear and stress. This study examined how different human–animal relationship qualities affect the behavior and heart rate variability (linear and non-linear parameters) of 36 nursery pigs. Over six weeks, pigs experienced positive (n = 12), minimal (n = 12), or negative (n = 12) human handling. Their responses to handlers were then assessed in an experimental arena with four phases: habituation, exposure to the handler standing and sitting, and forced interaction. Pigs subjected to negative handling exhibited increased fear-related behaviors, spending less time in contact with the handler. They also exhibited heightened stress responses, with greater LF/HF ratio and Lmean values compared with positively handled pigs. Conversely, gently handled pigs displayed affiliative behaviors, accepting more strokes, and higher parasympathetic activation, indicated by greater RMSSD/SDNN and SampEn values, suggesting a more positive affective state. Minimally handled pigs exhibited some behavioral similarities to gently handled pigs, although physiological data indicated that the interaction was likely more rewarding for the gently handled pigs. These results emphasize the impact of human–animal relationships on pig welfare and highlight the value of incorporating non-linear heart rate variability parameters in such evaluations. |
  | Date | 2024 |
  | Language | English |
  | Archive | Embase |
  | URL | https://www.embase.com/search/results?subaction=viewrecord&id=L2030904400&from=export |
  | Volume | 14 |
  | Publication | Animals |
  | DOI | 10.3390/ani14152217 |
  | Issue | 15 |
  | Journal Abbr | Animals |
  | ISSN | 2076-2615 |
  | Date Added | 11/07/2025, 11:53:18 |
  | Modified | 11/07/2025, 11:53:18 |

  ### Tags:

  - human-animal interaction
  - animal welfare
  - heart rate variability
  - heart rate
  - animal experiment
  - article
  - nonhuman
  - physiological stress
  - human
  - pig
  - fear
  - habituation
  - human-animal relation
  - minipig
  - sitting
  - standing

  ### Attachments

  - Full Text (HTML)
- ## Affective Implications of Human–Animal Relationship on Pig Welfare: Integrating Non-Linear Heart Rate Variability Measures

  |  |  |
  | --- | --- |
  | Item Type | Journal Article |
  | Author | J. Calderón-Amor |
  | Author | B. Zuleta |
  | Author | M.C. Ceballos |
  | Author | D. Cartes |
  | Author | C.J. Byrd |
  | Author | B. Lecorps |
  | Author | R. Palomo |
  | Author | S.A. Guzmán-Pino |
  | Author | D. Siel |
  | Author | D. Luna |
  | Abstract | The human–animal relationship is crucial for animal welfare. Gentle handling enhances pigs’ comfort while rough handling causes fear and stress. This study examined how different human–animal relationship qualities affect the behavior and heart rate variability (linear and non-linear parameters) of 36 nursery pigs. Over six weeks, pigs experienced positive (n = 12), minimal (n = 12), or negative (n = 12) human handling. Their responses to handlers were then assessed in an experimental arena with four phases: habituation, exposure to the handler standing and sitting, and forced interaction. Pigs subjected to negative handling exhibited increased fear-related behaviors, spending less time in contact with the handler. They also exhibited heightened stress responses, with greater LF/HF ratio and Lmean values compared with positively handled pigs. Conversely, gently handled pigs displayed affiliative behaviors, accepting more strokes, and higher parasympathetic activation, indicated by greater RMSSD/SDNN and SampEn values, suggesting a more positive affective state. Minimally handled pigs exhibited some behavioral similarities to gently handled pigs, although physiological data indicated that the interaction was likely more rewarding for the gently handled pigs. These results emphasize the impact of human–animal relationships on pig welfare and highlight the value of incorporating non-linear heart rate variability parameters in such evaluations. |
  | Date | 2024 |
  | Language | English |
  | Archive | Embase |
  | URL | https://www.embase.com/search/results?subaction=viewrecord&id=L2030904400&from=export |
  | Volume | 14 |
  | Publication | Animals |
  | DOI | 10.3390/ani14152217 |
  | Issue | 15 |
  | Journal Abbr | Animals |
  | ISSN | 2076-2615 |
  | Date Added | 11/07/2025, 12:10:40 |
  | Modified | 11/07/2025, 12:10:40 |

  ### Tags:

  - human-animal interaction
  - animal welfare
  - heart rate variability
  - heart rate
  - animal experiment
  - article
  - nonhuman
  - physiological stress
  - human
  - pig
  - fear
  - habituation
  - human-animal relation
  - minipig
  - sitting
  - standing

  ### Attachments

  - Full Text (HTML)
- ## Early life adversity in dogs produces altered physiological and behavioral responses during a social stress-buffering paradigm

  |  |  |
  | --- | --- |
  | Item Type | Journal Article |
  | Author | A.P. Buttner |
  | Author | S.L. Awalt |
  | Author | R. Strasser |
  | Abstract | Although owners can act as stress buffers for their dogs, whether dogs with poor early life histories with humans will respond similarly is unknown. We tested 45 dogs, 23 of which were rescued from adverse conditions, in a social paradigm in which a threatening stranger confronted them with either their owner or an unfamiliar human present. Salivary cortisol levels were assessed at three points, and the dogs' behavior and owners' responses to questionnaires were evaluated. Dogs from adverse backgrounds engaged in greater contact and exhibited more relaxed behaviors and social referencing when their owners were present. Dogs from the comparison group explored more when accompanied by their owners. Dogs from adverse backgrounds experienced greater decreases in cortisol levels from the first to third samples relative to dogs in the comparison group. Dogs from adverse backgrounds were also more likely to respond fearfully to a threatening stranger. Their owners rated them as having higher levels of stranger-directed fear, nonsocial fear, separation-related problems, attention seeking, and lower levels of chasing and trainability. These findings from this study suggest that early adverse environments may have lasting effects on dogs' social behavior. |
  | Date | 2023 |
  | Language | English |
  | Archive | Medline |
  | URL | https://www.embase.com/search/results?subaction=viewrecord&id=L641373835&from=export |
  | Volume | 120 |
  | Pages | 6-20 |
  | Publication | Journal of the experimental analysis of behavior |
  | DOI | 10.1002/jeab.856 |
  | Issue | 1 |
  | Journal Abbr | J Exp Anal Behav |
  | ISSN | 1938-3711 |
  | Date Added | 11/07/2025, 14:15:32 |
  | Modified | 11/07/2025, 14:15:32 |

  ### Tags:

  - physiology
  - dog
  - animal behavior
  - human
  - hydrocortisone
  - animal
  - human-animal bond
  - fear
  - childhood adversity

  ### Attachments

  - Full Text (HTML)
- ## Early life adversity in dogs produces altered physiological and behavioral responses during a social stress-buffering paradigm

  |  |  |
  | --- | --- |
  | Item Type | Journal Article |
  | Author | A.P. Buttner |
  | Author | S.L. Awalt |
  | Author | R. Strasser |
  | Abstract | Although owners can act as stress buffers for their dogs, whether dogs with poor early life histories with humans will respond similarly is unknown. We tested 45 dogs, 23 of which were rescued from adverse conditions, in a social paradigm in which a threatening stranger confronted them with either their owner or an unfamiliar human present. Salivary cortisol levels were assessed at three points, and the dogs' behavior and owners' responses to questionnaires were evaluated. Dogs from adverse backgrounds engaged in greater contact and exhibited more relaxed behaviors and social referencing when their owners were present. Dogs from the comparison group explored more when accompanied by their owners. Dogs from adverse backgrounds experienced greater decreases in cortisol levels from the first to third samples relative to dogs in the comparison group. Dogs from adverse backgrounds were also more likely to respond fearfully to a threatening stranger. Their owners rated them as having higher levels of stranger-directed fear, nonsocial fear, separation-related problems, attention seeking, and lower levels of chasing and trainability. These findings from this study suggest that early adverse environments may have lasting effects on dogs' social behavior. |
  | Date | 2023 |
  | Language | English |
  | Archive | Medline |
  | URL | https://www.embase.com/search/results?subaction=viewrecord&id=L641373835&from=export |
  | Volume | 120 |
  | Pages | 6-20 |
  | Publication | Journal of the experimental analysis of behavior |
  | DOI | 10.1002/jeab.856 |
  | Issue | 1 |
  | Journal Abbr | J Exp Anal Behav |
  | ISSN | 1938-3711 |
  | Date Added | 11/07/2025, 14:16:42 |
  | Modified | 11/07/2025, 14:16:42 |

  ### Tags:

  - physiology
  - dog
  - animal behavior
  - human
  - hydrocortisone
  - animal
  - human-animal bond
  - fear
  - childhood adversity

  ### Attachments

  - Full Text (HTML)
- ## Early life adversity in dogs produces altered physiological and behavioral responses during a social stress-buffering paradigm

  |  |  |
  | --- | --- |
  | Item Type | Journal Article |
  | Author | A.P. Buttner |
  | Author | S.L. Awalt |
  | Author | R. Strasser |
  | Abstract | Although owners can act as stress buffers for their dogs, whether dogs with poor early life histories with humans will respond similarly is unknown. We tested 45 dogs, 23 of which were rescued from adverse conditions, in a social paradigm in which a threatening stranger confronted them with either their owner or an unfamiliar human present. Salivary cortisol levels were assessed at three points, and the dogs' behavior and owners' responses to questionnaires were evaluated. Dogs from adverse backgrounds engaged in greater contact and exhibited more relaxed behaviors and social referencing when their owners were present. Dogs from the comparison group explored more when accompanied by their owners. Dogs from adverse backgrounds experienced greater decreases in cortisol levels from the first to third samples relative to dogs in the comparison group. Dogs from adverse backgrounds were also more likely to respond fearfully to a threatening stranger. Their owners rated them as having higher levels of stranger-directed fear, nonsocial fear, separation-related problems, attention seeking, and lower levels of chasing and trainability. These findings from this study suggest that early adverse environments may have lasting effects on dogs' social behavior. |
  | Date | 2023 |
  | Language | English |
  | Archive | Medline |
  | URL | https://www.embase.com/search/results?subaction=viewrecord&id=L641373835&from=export |
  | Volume | 120 |
  | Pages | 6-20 |
  | Publication | Journal of the experimental analysis of behavior |
  | DOI | 10.1002/jeab.856 |
  | Issue | 1 |
  | Journal Abbr | J Exp Anal Behav |
  | ISSN | 1938-3711 |
  | Date Added | 11/07/2025, 14:18:15 |
  | Modified | 11/07/2025, 14:18:15 |

  ### Tags:

  - physiology
  - dog
  - animal behavior
  - human
  - hydrocortisone
  - animal
  - human-animal bond
  - fear
  - childhood adversity

  ### Attachments

  - Full Text (HTML)
- ## Neurobiological underpinnings of dogs’ human-like social competence: How interactions between stress response systems and oxytocin mediate dogs’ social skills

  |  |  |
  | --- | --- |
  | Item Type | Journal Article |
  | Author | A.P. Buttner |
  | Abstract | Domestic dogs (Canis familiaris) have been suggested as a natural model for human social cognition, possessing social skills that are in many ways functionally analogous to those of young humans. Researchers have debated the origins of dogs’ human-like social competence and the underlying cognitive mechanisms, but only recently have researchers begun to explore their neurobiological underpinnings. In this review, findings from behavioral studies are integrated with what is known about the biological basis of dogs’ human-directed social competence, with an emphasis on how stress-mediating systems, particularly the hypothalamic-pituitary-adrenal (HPA) axis, interact with oxytocin and underlying neural systems to facilitate dogs’ interspecific social-cognitive abilities. The working model presented in this paper offers a biological explanation for many of the inconsistent findings from past work on social cognition in dogs and generates questions for future research in the field of canine social competence. |
  | Date | 2016 |
  | Language | English |
  | Archive | Embase |
  | URL | https://www.embase.com/search/results?subaction=viewrecord&id=L612005916&from=export |
  | Volume | 71 |
  | Pages | 198-214 |
  | Publication | Neuroscience and Biobehavioral Reviews |
  | DOI | 10.1016/j.neubiorev.2016.08.029 |
  | Issue | (Buttner A.P., akphillips@unomaha.edu) Department of Psychology, Neuroscience and Behavior, University of Nebraska at Omaha, Allwine Hall, Room 419, 6001 Dodge St., Omaha, NE, United States |
  | Journal Abbr | Neurosci. Biobehav. Rev. |
  | ISSN | 1873-7528 |
  | Date Added | 11/07/2025, 12:13:34 |
  | Modified | 11/07/2025, 12:13:34 |

  ### Tags:

  - dog
  - social behavior
  - nonhuman
  - social interaction
  - human
  - emotional attachment
  - review
  - hypothalamus hypophysis adrenal system
  - prefrontal cortex
  - neurobiology
  - domestic animal
  - priority journal
  - social cognition
  - domestication
  - socialization
  - social adaptation
  - social competence
  - social stress

  ### Attachments

  - Full Text (HTML)
- ## PSYCHOLOGICAL MANIFESTATIONS OF HYPERSENSITIVITY PNEUMONITIS

  |  |  |
  | --- | --- |
  | Item Type | Journal Article |
  | Author | J. Blanco Jimenez |
  | Author | A. Madhavan |
  | Abstract | SESSION TITLE: Occupational and Environmental Lung Diseases Case Report Posters 1 SESSION TYPE: Case Report Posters PRESENTED ON: 10/10/2023 09:40 am - 10:25 am INTRODUCTION: Hypersensitivity pneumonitis is a group of immunologically mediated lung diseases caused by inhalation of environmental agents in susceptible individuals. Most patients are non-smokers and are exposed to animal antigens. We present a case of a young female with lung disease from exposure to her parrots, in which emotional attachment impacted management. CASE PRESENTATION: A 37-year-old female with no past medical history came to our outpatient pulmonary clinic for evaluation of dry cough for 14 weeks along with shortness of breath on exertion. She was initially treated with a course of azithromycin and prednisone by her primary medical provider with only partial improvement. However, symptoms returned upon discontinuation of therapy. She was not a smoker. She had bought two parrots 8 months before the appearance of symptoms. She cleaned their cage and was in direct contact with the birds during most of the day. Physical exam was significant for crackles in bilateral lung fields. Pulmonary Function Tests demonstrated decreased Total Lung Capacity (66%) and diffusing capacity for carbon monoxide (50%), consistent with restrictive lung disease. Chest High Resolution Computed Tomography showed diffuse ground glass and nodular opacities as well as mosaic changes. Bird fancier profile was positive IgG for parrot, pigeon, parakeet and canary droppings. Hypersensitivity pneumonitis from bird antigen exposure was suspected and the patient was recommended to give away her birds. She went to visit her family abroad for 1 month and noticed a decrease in symptoms, however, they returned soon after she got back home. When educated about her diagnosis and that management involved removal of exposure, the patient refused to be separated from her birds. She deeply loved her birds and considered them as her children. She would never get rid of them. DISCUSSION: Hypersensitivity pneumonitis involves a group of lung diseases usually caused by inhalation of environmental agents, commonly animal products. The association between human disease from animal exposure and emotional attachment to pets is underexplored. Moreover, human-animal bond interaction in patients with hypersensitivity pneumonitis needs further attention as these behaviors directly impact patients' health. Antropomorphism is the attribution of human cognitive processes and emotional states to animals, such as feelings of happiness, love, or guilt. Patients with pets tend to trust less in human beings and more in their pets. People tend to view their animals as a consistent source of social support, which makes the loss difficult to bear. A break in the human-animal bond can occur with separation or death. Moreover, pet ownership has been shown to be a significant predictor of the likelihood of ever having experienced depression. In our case, we are actively following the patient and have recommended psychiatric evaluation as she is at risk of developing pulmonary fibrosis. CONCLUSIONS: Human-animal bond is an underrecognized factor influencing management in certain diseases that are directly related to animal exposures. Hypersensitivity pneumonitis is an example of such. Understanding the psychosocial determinants of human-animal interactions is of paramount importance for the successful management of patients suffering from this entity. REFERENCE #1: Mueller, M.K., Gee, N.R. & Bures, R.M. Human-animal interaction as a social determinant of health: descriptive findings from the health and retirement study. BMC Public Health 18, 305 (2018). https://doi.org/10.1186/s12889-018-5188-0 REFERENCE #2: Yi ES. Hypersensitivity pneumonitis. Crit Rev Clin Lab Sci. 2002;39(6):581-629. doi:10.1080/10408360290795583 REFERENCE #3: Overgaauw PAM, Vinke CM, Hagen MAEV, Lipman LJA. A One Health Perspective on the Human-Companion Animal Relationship with Emphasis on Zoonotic Aspects. Int J Environ Res Public Health. 2020 May 27;17(11):3789. doi: 10.3390/ijerph17113789. PMID: 32471058; PMCID: PMC7312520. DISCLOSURES: No relevant relationships by JAVIER BLANCO JIMENEZ No disclosure on file for Arjun Madhavan |
  | Date | 2023 |
  | Language | English |
  | Archive | Embase |
  | URL | https://www.embase.com/search/results?subaction=viewrecord&id=L2027231107&from=export |
  | Volume | 164 |
  | Pages | A5132 |
  | Publication | Chest |
  | DOI | 10.1016/j.chest.2023.07.3324 |
  | Issue | 4 |
  | Journal Abbr | Chest |
  | ISSN | 1931-3543 |
  | Date Added | 11/07/2025, 14:20:20 |
  | Modified | 11/07/2025, 14:20:20 |

  ### Tags:

  - social support
  - One Health
  - emotion
  - cognition
  - child
  - female
  - nonhuman
  - human
  - adult
  - emotional attachment
  - pet animal
  - clinical article
  - human-animal bond
  - happiness
  - endogenous compound
  - depression
  - outpatient
  - conference abstract
  - attention
  - adverse drug reaction
  - allergic pneumonitis
  - animal product
  - antigen
  - azithromycin
  - case report
  - Columbidae
  - crackle
  - diffusing capacity for carbon monoxide
  - drug combination
  - dry cough
  - exertional dyspnea
  - glass
  - guilt
  - high resolution computer tomography
  - immunoglobulin G
  - inhalation
  - lung disease
  - lung fibrosis
  - lung function test
  - medical history
  - non-smoker
  - occupational lung disease
  - parakeet
  - parrot
  - physical examination
  - prednisone
  - public health
  - retirement
  - Serinus
  - side effect
  - smoking
  - social determinants of health
  - total lung capacity
  - trust

  ### Attachments

  - Full Text (HTML)
- ## Call for vets to participate in microchip study

  |  |  |
  | --- | --- |
  | Item Type | Journal Article |
  | Author | L. Blake |
  | Date | 2022 |
  | Language | English |
  | Archive | Embase |
  | URL | https://www.embase.com/search/results?subaction=viewrecord&id=L2014938383&from=export |
  | Volume | 190 |
  | Pages | 128-129 |
  | Publication | Veterinary Record |
  | DOI | 10.1002/vetr.1460 |
  | Issue | 3 |
  | Journal Abbr | Vet. Rec. |
  | ISSN | 2042-7670 |
  | Date Added | 11/07/2025, 14:16:49 |
  | Modified | 11/07/2025, 14:16:49 |

  ### Tags:

  - article
  - nonhuman
  - human
  - hydrocortisone
  - information processing
  - certification
  - veterinary medicine
  - human-animal bond
  - glucose
  - biosensor
  - animal health
  - veterinarian
  - animal identification
  - computer interface
  - early intervention
  - general device
  - machine learning
  - pet collar
  - pet microchip
  - telehealth
  - wearable sensor

  ### Attachments

  - Full Text (HTML)
- ## Call for vets to participate in microchip study

  |  |  |
  | --- | --- |
  | Item Type | Journal Article |
  | Author | L. Blake |
  | Date | 2022 |
  | Language | English |
  | Archive | Embase |
  | URL | https://www.embase.com/search/results?subaction=viewrecord&id=L2014938383&from=export |
  | Volume | 190 |
  | Pages | 128-129 |
  | Publication | Veterinary Record |
  | DOI | 10.1002/vetr.1460 |
  | Issue | 3 |
  | Journal Abbr | Vet. Rec. |
  | ISSN | 2042-7670 |
  | Date Added | 11/07/2025, 14:18:22 |
  | Modified | 11/07/2025, 14:18:22 |

  ### Tags:

  - article
  - nonhuman
  - human
  - hydrocortisone
  - information processing
  - certification
  - veterinary medicine
  - human-animal bond
  - glucose
  - biosensor
  - animal health
  - veterinarian
  - animal identification
  - computer interface
  - early intervention
  - general device
  - machine learning
  - pet collar
  - pet microchip
  - telehealth
  - wearable sensor

  ### Attachments

  - Full Text (HTML)
- ## Editor’s Note: Announcing the 25th anniversary of the publication of JAAWS

  |  |  |
  | --- | --- |
  | Item Type | Journal Article |
  | Author | C. Bennett |
  | Author | K. Shapiro |
  | Date | 2023 |
  | Language | English |
  | Archive | Embase |
  | URL | https://www.embase.com/search/results?subaction=viewrecord&id=L2019464456&from=export |
  | Volume | 26 |
  | Pages | 1-3 |
  | Publication | Journal of Applied Animal Welfare Science |
  | DOI | 10.1080/10888705.2022.2131061 |
  | Issue | 1 |
  | Journal Abbr | J. Appl. Anim. Welf. Sci. |
  | ISSN | 1532-7604 |
  | Date Added | 11/07/2025, 11:53:22 |
  | Modified | 11/07/2025, 11:53:22 |

  ### Tags:

  - human-animal interaction
  - animal welfare
  - heart rate
  - cognition
  - glucocorticoid
  - nonhuman
  - zoo animal
  - physical well-being
  - psychological well-being
  - animal husbandry
  - awareness
  - editorial
  - qualitative analysis
  - quantitative analysis
  - scientific literature
  - social well-being
  - wildlife
  - wildlife conservation

  ### Attachments

  - Full Text (HTML)
- ## Editor’s Note: Announcing the 25th anniversary of the publication of JAAWS

  |  |  |
  | --- | --- |
  | Item Type | Journal Article |
  | Author | C. Bennett |
  | Author | K. Shapiro |
  | Date | 2023 |
  | Language | English |
  | Archive | Embase |
  | URL | https://www.embase.com/search/results?subaction=viewrecord&id=L2019464456&from=export |
  | Volume | 26 |
  | Pages | 1-3 |
  | Publication | Journal of Applied Animal Welfare Science |
  | DOI | 10.1080/10888705.2022.2131061 |
  | Issue | 1 |
  | Journal Abbr | J. Appl. Anim. Welf. Sci. |
  | ISSN | 1532-7604 |
  | Date Added | 11/07/2025, 12:10:45 |
  | Modified | 11/07/2025, 12:10:45 |

  ### Tags:

  - human-animal interaction
  - animal welfare
  - heart rate
  - cognition
  - glucocorticoid
  - nonhuman
  - zoo animal
  - physical well-being
  - psychological well-being
  - animal husbandry
  - awareness
  - editorial
  - qualitative analysis
  - quantitative analysis
  - scientific literature
  - social well-being
  - wildlife
  - wildlife conservation

  ### Attachments

  - Full Text (HTML)
- ## Animal Farm in healthcare: definitions, policies, laws and implications for health professionals

  |  |  |
  | --- | --- |
  | Item Type | Journal Article |
  | Author | E. Ben-Sefer |
  | Author | L. Shields |
  | Date | 2021 |
  | Language | English |
  | Archive | Embase |
  | URL | https://www.embase.com/search/results?subaction=viewrecord&id=L2010676422&from=export |
  | Volume | 114 |
  | Pages | 171-177 |
  | Publication | Journal of the Royal Society of Medicine |
  | DOI | 10.1177/0141076821996003 |
  | Issue | 4 |
  | Journal Abbr | J. R. Soc. Med. |
  | ISSN | 1758-1095 |
  | Date Added | 11/07/2025, 12:10:49 |
  | Modified | 11/07/2025, 12:10:49 |

  ### Tags:

  - human-animal interaction
  - veteran
  - heart rate
  - dog
  - animal behavior
  - Canada
  - nonhuman
  - social interaction
  - human
  - hydrocortisone
  - epinephrine
  - noradrenalin
  - posttraumatic stress disorder
  - animal food
  - hospitalization
  - United States
  - animal well-being
  - note
  - quality of life
  - autism
  - psychology
  - treatment outcome
  - veterinary medicine
  - training
  - animal assisted therapy
  - adrenalin blood level
  - Americans with disabilities act
  - animal allergy
  - animal care
  - animal housing
  - Asia
  - attention deficit hyperactivity disorder
  - Australia
  - aviation
  - behavior therapy
  - blood pressure regulation
  - child care
  - criminal justice
  - danger, risk, safety and related phenomena
  - diabetes mellitus
  - emotional disorder
  - epilepsy
  - European Union
  - family
  - food safety
  - Germany
  - government
  - health care access
  - health care facility
  - health care policy
  - health care system
  - health practitioner
  - health service
  - health status
  - Hong Kong
  - hormone release
  - human rights
  - hydrocortisone blood level
  - infection risk
  - Japan
  - legal aspect
  - long term care
  - medical documentation
  - mental deficiency
  - New Zealand
  - noradrenalin blood level
  - patient care
  - pediatric hospital
  - personal hygiene
  - physical disease
  - practice guideline
  - Queensland
  - service animal
  - service dog
  - sociology
  - travel
  - United Kingdom
  - war

  ### Attachments

  - Full Text (HTML)
- ## Genomic Selection for Dairy Cattle Behaviour Considering Novel Traits in a Changing Technical Production Environment

  |  |  |
  | --- | --- |
  | Item Type | Journal Article |
  | Author | L.E. Behren |
  | Author | S. König |
  | Author | K. May |
  | Abstract | Cow behaviour is a major factor influencing dairy herd profitability and is an indicator of animal welfare and disease. Behaviour is a complex network of behavioural patterns in response to environmental and social stimuli and human handling. Advances in agricultural technology have led to changes in dairy cow husbandry systems worldwide. Increasing herd sizes, less time availability to take care of the animals and modern technology such as automatic milking systems (AMSs) imply limited human–cow interactions. On the other hand, cow behaviour responses to the technical environment (cow–AMS interactions) simultaneously improve production efficiency and welfare and contribute to simplified “cow handling” and reduced labour time. Automatic milking systems generate objective behaviour traits linked to workability, milkability and health, which can be implemented into genomic selection tools. However, there is insufficient understanding of the genetic mechanisms influencing cow learning and social behaviour, in turn affecting herd management, productivity and welfare. Moreover, physiological and molecular biomarkers such as heart rate, neurotransmitters and hormones might be useful indicators and predictors of cow behaviour. This review gives an overview of published behaviour studies in dairy cows in the context of genetics and genomics and discusses possibilities for breeding approaches to achieve desired behaviour in a technical production environment. |
  | Date | 2023 |
  | Language | English |
  | Archive | Embase |
  | URL | https://www.embase.com/search/results?subaction=viewrecord&id=L2026352960&from=export |
  | Volume | 14 |
  | Publication | Genes |
  | DOI | 10.3390/genes14101933 |
  | Issue | 10 |
  | Journal Abbr | Genes |
  | ISSN | 2073-4425 |
  | Date Added | 11/07/2025, 11:53:22 |
  | Modified | 11/07/2025, 11:53:22 |

  ### Tags:

  - human-animal interaction
  - behavior
  - cow
  - social behavior
  - nonhuman
  - review
  - dairy cattle
  - biological marker
  - feeding behavior
  - genetic correlation
  - genetic selection
  - genetics
  - genomic selection
  - genomics
  - heritability
  - maternal behavior
  - milk production
  - reproduction
  - reproductive behavior
  - rumination

  ### Attachments

  - Full Text (HTML)
- ## Genomic Selection for Dairy Cattle Behaviour Considering Novel Traits in a Changing Technical Production Environment

  |  |  |
  | --- | --- |
  | Item Type | Journal Article |
  | Author | L.E. Behren |
  | Author | S. König |
  | Author | K. May |
  | Abstract | Cow behaviour is a major factor influencing dairy herd profitability and is an indicator of animal welfare and disease. Behaviour is a complex network of behavioural patterns in response to environmental and social stimuli and human handling. Advances in agricultural technology have led to changes in dairy cow husbandry systems worldwide. Increasing herd sizes, less time availability to take care of the animals and modern technology such as automatic milking systems (AMSs) imply limited human–cow interactions. On the other hand, cow behaviour responses to the technical environment (cow–AMS interactions) simultaneously improve production efficiency and welfare and contribute to simplified “cow handling” and reduced labour time. Automatic milking systems generate objective behaviour traits linked to workability, milkability and health, which can be implemented into genomic selection tools. However, there is insufficient understanding of the genetic mechanisms influencing cow learning and social behaviour, in turn affecting herd management, productivity and welfare. Moreover, physiological and molecular biomarkers such as heart rate, neurotransmitters and hormones might be useful indicators and predictors of cow behaviour. This review gives an overview of published behaviour studies in dairy cows in the context of genetics and genomics and discusses possibilities for breeding approaches to achieve desired behaviour in a technical production environment. |
  | Date | 2023 |
  | Language | English |
  | Archive | Embase |
  | URL | https://www.embase.com/search/results?subaction=viewrecord&id=L2026352960&from=export |
  | Volume | 14 |
  | Publication | Genes |
  | DOI | 10.3390/genes14101933 |
  | Issue | 10 |
  | Journal Abbr | Genes |
  | ISSN | 2073-4425 |
  | Date Added | 11/07/2025, 12:10:45 |
  | Modified | 11/07/2025, 12:10:45 |

  ### Tags:

  - human-animal interaction
  - behavior
  - cow
  - social behavior
  - nonhuman
  - review
  - dairy cattle
  - biological marker
  - feeding behavior
  - genetic correlation
  - genetic selection
  - genetics
  - genomic selection
  - genomics
  - heritability
  - maternal behavior
  - milk production
  - reproduction
  - reproductive behavior
  - rumination

  ### Attachments

  - Full Text (HTML)
- ## Judgement bias in goats (Capra hircus): Investigating the effects of human grooming

  |  |  |
  | --- | --- |
  | Item Type | Journal Article |
  | Author | L. Baciadonna |
  | Author | C. Nawroth |
  | Author | A.G. McElligott |
  | Abstract | Animal emotional states can be investigated by evaluating their impact on cognitive processes. In this study, we used a judgement bias paradigm to determine if shortterm positive human-animal interaction (grooming) induced a positive affective state in goats. We tested two groups of goats and trained them to discriminate between a rewarded and a non-rewarded location over nine training days. During training, the experimental group (nD9) was gently groomed by brushing their heads and backs for five min over 11 days (nine training days, plus two testing days, total time 55 min). During training, the control group (nD10) did not experience any direct interaction with the experimenter, but was kept unconstrained next to him for the same period of time. After successful completion of the training, the responses (latency time) of the two groups to reach ambiguous locations situated between the two reference locations (i.e., rewarded/non-rewarded) were compared over two days of testing. There was not a positive bias effect after the animals had been groomed. In a second experiment, 10 goats were tested to investigate whether grooming induced changes in physiological activation (i.e., heart rate and heart rate variability). Heart rate increased when goats were groomed compared to the baseline condition, when the same goats did not receive any contact with the experimenter. Also, subjects did not move away from the experimenter, suggesting that the grooming was positively accepted. The very good care and the regular positive contacts that goats received from humans at the study site could potentially account for the results obtained. Good husbandry outcomes are influenced by animals' perception of the events and this is based on current circumstances, past experiences and individual variables. Taking into account animals' individual characteristics and identifying effective strategies to induce positive emotions could increase the understanding and reliability of using cognitive biases paradigms to investigate and promote animal welfare. Copyright 2016 Baciadonna et al. |
  | Date | 2016 |
  | Language | English |
  | Archive | Embase |
  | URL | https://www.embase.com/search/results?subaction=viewrecord&id=L612902538&from=export |
  | Volume | 2016 |
  | Publication | PeerJ |
  | DOI | 10.7717/peerj.2485 |
  | Issue | 10 |
  | Journal Abbr | PeerJ |
  | ISSN | 2167-8359 |
  | Date Added | 11/07/2025, 12:10:56 |
  | Modified | 11/07/2025, 12:10:56 |

  ### Tags:

  - animal welfare
  - heart rate variability
  - animal experiment
  - male
  - nonhuman
  - controlled study
  - grooming
  - reliability
  - perception
  - agriculture
  - cognitive bias
  - control group
  - domestic goat
  - experimental model
  - head
  - human versus animal comparison
  - latent period

  ### Attachments

  - Full Text (HTML)
- ## Infrared thermography as a tool in welfare assessment of equines handled in paddock and stall

  |  |  |
  | --- | --- |
  | Item Type | Journal Article |
  | Author | D.R.O.E. Azevedo |
  | Author | B.S.L. Dallago |
  | Author | A.C. Silva |
  | Author | J.R.M. Pereira |
  | Author | R.F. Ferreira |
  | Author | T.S. Borges |
  | Author | T.B. Bisol |
  | Author | L.V. Sobrinho |
  | Author | T.O. Fernandes |
  | Author | F.E.M. Bernal |
  | Abstract | This study aims to describe the effects of stabling on thermographic characteristics of adult equine limbs, to compare the level of animal welfare between horses kept loose and those under continuous stabling and to investigate whether the Infrared Thermography (IRT) can be an effective tool to evaluate the welfare of equines. 24 gelding horses of Brazilian Sport Horse breed, aged between 5 and 9 years and weighing 500 ± 50kg were used in a 6-week trial. They were divided into 2 groups: 1) Paddock - equines kept in paddocks and ridden exclusively on urban patrol; 2) Stall - animals that ridden exclusively on urban patrol, but were kept in continuous stabling, housed in 12m2 with concrete and bedless stables. Every animal was evaluated once a week over six weeks. Animal behavior and temperature of the eyes and distal extremities of the limbs as well as blood samples were collected. The confined horses showed stereotyped behavior and higher serum cortisol, indicating lower animal welfare when compared to those managed in a pasture. Limb IRT showed predictive potential for identifying chronic stress as the discriminating analysis showed 74.5% hits while eye temperature was not efficient for this purpose. |
  | Date | 2024 |
  | Language | English |
  | Archive | Embase |
  | URL | https://www.embase.com/search/results?subaction=viewrecord&id=L2033122645&from=export |
  | Volume | 76 |
  | Publication | Arquivo Brasileiro de Medicina Veterinaria e Zootecnia |
  | DOI | 10.1590/1678-4162-13037 |
  | Issue | 4 |
  | Journal Abbr | Arq. Bras. Med. Vet. Zootec. |
  | ISSN | 1678-4162 |
  | Date Added | 11/07/2025, 14:15:31 |
  | Modified | 11/07/2025, 14:15:31 |

  ### Tags:

  - human-animal interaction
  - animal welfare
  - animal behavior
  - animal experiment
  - article
  - male
  - nonhuman
  - hydrocortisone
  - adult
  - body temperature
  - limb
  - prediction
  - Equus
  - animal housing
  - hydrocortisone blood level
  - horse breed
  - negative valence
  - chronic stress
  - correlation analysis
  - eye temperature
  - stress assessment
  - thermography

  ### Attachments

  - Full Text (HTML)
- ## Infrared thermography as a tool in welfare assessment of equines handled in paddock and stall

  |  |  |
  | --- | --- |
  | Item Type | Journal Article |
  | Author | D.R.O.E. Azevedo |
  | Author | B.S.L. Dallago |
  | Author | A.C. Silva |
  | Author | J.R.M. Pereira |
  | Author | R.F. Ferreira |
  | Author | T.S. Borges |
  | Author | T.B. Bisol |
  | Author | L.V. Sobrinho |
  | Author | T.O. Fernandes |
  | Author | F.E.M. Bernal |
  | Abstract | This study aims to describe the effects of stabling on thermographic characteristics of adult equine limbs, to compare the level of animal welfare between horses kept loose and those under continuous stabling and to investigate whether the Infrared Thermography (IRT) can be an effective tool to evaluate the welfare of equines. 24 gelding horses of Brazilian Sport Horse breed, aged between 5 and 9 years and weighing 500 ± 50kg were used in a 6-week trial. They were divided into 2 groups: 1) Paddock - equines kept in paddocks and ridden exclusively on urban patrol; 2) Stall - animals that ridden exclusively on urban patrol, but were kept in continuous stabling, housed in 12m2 with concrete and bedless stables. Every animal was evaluated once a week over six weeks. Animal behavior and temperature of the eyes and distal extremities of the limbs as well as blood samples were collected. The confined horses showed stereotyped behavior and higher serum cortisol, indicating lower animal welfare when compared to those managed in a pasture. Limb IRT showed predictive potential for identifying chronic stress as the discriminating analysis showed 74.5% hits while eye temperature was not efficient for this purpose. |
  | Date | 2024 |
  | Language | English |
  | Archive | Embase |
  | URL | https://www.embase.com/search/results?subaction=viewrecord&id=L2033122645&from=export |
  | Volume | 76 |
  | Publication | Arquivo Brasileiro de Medicina Veterinaria e Zootecnia |
  | DOI | 10.1590/1678-4162-13037 |
  | Issue | 4 |
  | Journal Abbr | Arq. Bras. Med. Vet. Zootec. |
  | ISSN | 1678-4162 |
  | Date Added | 11/07/2025, 14:16:41 |
  | Modified | 11/07/2025, 14:16:41 |

  ### Tags:

  - human-animal interaction
  - animal welfare
  - animal behavior
  - animal experiment
  - article
  - male
  - nonhuman
  - hydrocortisone
  - adult
  - body temperature
  - limb
  - prediction
  - Equus
  - animal housing
  - hydrocortisone blood level
  - horse breed
  - negative valence
  - chronic stress
  - correlation analysis
  - eye temperature
  - stress assessment
  - thermography

  ### Attachments

  - Full Text (HTML)
- ## Infrared thermography as a tool in welfare assessment of equines handled in paddock and stall

  |  |  |
  | --- | --- |
  | Item Type | Journal Article |
  | Author | D.R.O.E. Azevedo |
  | Author | B.S.L. Dallago |
  | Author | A.C. Silva |
  | Author | J.R.M. Pereira |
  | Author | R.F. Ferreira |
  | Author | T.S. Borges |
  | Author | T.B. Bisol |
  | Author | L.V. Sobrinho |
  | Author | T.O. Fernandes |
  | Author | F.E.M. Bernal |
  | Abstract | This study aims to describe the effects of stabling on thermographic characteristics of adult equine limbs, to compare the level of animal welfare between horses kept loose and those under continuous stabling and to investigate whether the Infrared Thermography (IRT) can be an effective tool to evaluate the welfare of equines. 24 gelding horses of Brazilian Sport Horse breed, aged between 5 and 9 years and weighing 500 ± 50kg were used in a 6-week trial. They were divided into 2 groups: 1) Paddock - equines kept in paddocks and ridden exclusively on urban patrol; 2) Stall - animals that ridden exclusively on urban patrol, but were kept in continuous stabling, housed in 12m2 with concrete and bedless stables. Every animal was evaluated once a week over six weeks. Animal behavior and temperature of the eyes and distal extremities of the limbs as well as blood samples were collected. The confined horses showed stereotyped behavior and higher serum cortisol, indicating lower animal welfare when compared to those managed in a pasture. Limb IRT showed predictive potential for identifying chronic stress as the discriminating analysis showed 74.5% hits while eye temperature was not efficient for this purpose. |
  | Date | 2024 |
  | Language | English |
  | Archive | Embase |
  | URL | https://www.embase.com/search/results?subaction=viewrecord&id=L2033122645&from=export |
  | Volume | 76 |
  | Publication | Arquivo Brasileiro de Medicina Veterinaria e Zootecnia |
  | DOI | 10.1590/1678-4162-13037 |
  | Issue | 4 |
  | Journal Abbr | Arq. Bras. Med. Vet. Zootec. |
  | ISSN | 1678-4162 |
  | Date Added | 11/07/2025, 14:18:14 |
  | Modified | 11/07/2025, 14:18:14 |

  ### Tags:

  - human-animal interaction
  - animal welfare
  - animal behavior
  - animal experiment
  - article
  - male
  - nonhuman
  - hydrocortisone
  - adult
  - body temperature
  - limb
  - prediction
  - Equus
  - animal housing
  - hydrocortisone blood level
  - horse breed
  - negative valence
  - chronic stress
  - correlation analysis
  - eye temperature
  - stress assessment
  - thermography

  ### Attachments

  - Full Text (HTML)
- ## Impact of Human–Animal Interactions on Psychological and Physiological Factors Associated With Veterinary School Students and Donkeys

  |  |  |
  | --- | --- |
  | Item Type | Journal Article |
  | Author | E. Artemiou |
  | Author | P. Hutchison |
  | Author | M. Machado |
  | Author | D. Ellis |
  | Author | J. Bradtke |
  | Author | M.M. Pereira |
  | Author | J. Carter |
  | Author | D. Bergfelt |
  | Abstract | There has been an increased interest in evaluating human–animal interactions and assessing the mutual health and wellbeing. In this study, first-year female and male veterinary school students not paired (n = 58) or paired (n = 25) with immature (≤9 mo) donkeys (n = 13) were engaged in three different types of interactions (1st, hands-off remote learning, 2nd, hands-on passive learning, and 3rd, hands-on active learning) for 30 min each during Week 2 (Time 1), Weeks 5–8 (Time 2), and Week 12 (Time 3) over three, 15-week periods. Student psychological data involved the Penn State Worry Questionnaire (PSWQ) scores collected from the interactive (student-donkey pairs) and non-interactive (no student-donkey pairs) groups and modified Comfort from Companion Animals Scale (CCAS) scores collected from the interactive group during Times 1, 2, and 3. Donkey physiological data involved collection of saliva within 10 min pre- and post-interaction during Times 1, 2, and 3 in association with the different types of interactions for immunoanalysis of cortisol. There were no significant effects of the various times and types of interactions on CCAS scores. While there were no significant effects of group and types of interactions on PSWQ scores, there was an effect (P = 0.01) of time. Overall mean PSWQ scores were significantly lower during Week 12 versus Week 2. Correspondingly, while there were no effects pre- vs. post-interaction within or among times on saliva cortisol concentrations in donkeys, there was an effect (P = 0.02) of the type of interaction. Mean concentrations were significantly lower with the hands-on passive and hands-on active learning versus the hands-off remote learning. In conclusion, while this study provides preliminary evidence surrounding student donkey interactions, future studies are required with more comprehensive designs to clarify these benefits and better understand the advantages and challenges surrounding student-donkey interactions. |
  | Date | 2021 |
  | Language | English |
  | Archive | Embase |
  | URL | https://www.embase.com/search/results?subaction=viewrecord&id=L635900541&from=export |
  | Volume | 8 |
  | Publication | Frontiers in Veterinary Science |
  | DOI | 10.3389/fvets.2021.701302 |
  | Issue | (Artemiou E., eartemiou@rossvet.edu.kn; Pereira M.M.) Department of Clinical Sciences, Ross University School of Veterinary Medicine, Basseterre, Saint Kitts and Nevis |
  | Journal Abbr | Front. Vet. Sci. |
  | ISSN | 2297-1769 |
  | Date Added | 11/07/2025, 14:16:52 |
  | Modified | 11/07/2025, 14:16:52 |

  ### Tags:

  - human-animal interaction
  - saliva
  - animal experiment
  - article
  - female
  - male
  - nonhuman
  - human
  - adult
  - human experiment
  - rating scale
  - learning
  - psychological aspect
  - immunoassay
  - Penn State Worry Questionnaire
  - comfort
  - concentration ratio
  - donkey
  - physiological process
  - veterinary student

  ### Attachments

  - Full Text (HTML)
- ## Impact of Human–Animal Interactions on Psychological and Physiological Factors Associated With Veterinary School Students and Donkeys

  |  |  |
  | --- | --- |
  | Item Type | Journal Article |
  | Author | E. Artemiou |
  | Author | P. Hutchison |
  | Author | M. Machado |
  | Author | D. Ellis |
  | Author | J. Bradtke |
  | Author | M.M. Pereira |
  | Author | J. Carter |
  | Author | D. Bergfelt |
  | Abstract | There has been an increased interest in evaluating human–animal interactions and assessing the mutual health and wellbeing. In this study, first-year female and male veterinary school students not paired (n = 58) or paired (n = 25) with immature (≤9 mo) donkeys (n = 13) were engaged in three different types of interactions (1st, hands-off remote learning, 2nd, hands-on passive learning, and 3rd, hands-on active learning) for 30 min each during Week 2 (Time 1), Weeks 5–8 (Time 2), and Week 12 (Time 3) over three, 15-week periods. Student psychological data involved the Penn State Worry Questionnaire (PSWQ) scores collected from the interactive (student-donkey pairs) and non-interactive (no student-donkey pairs) groups and modified Comfort from Companion Animals Scale (CCAS) scores collected from the interactive group during Times 1, 2, and 3. Donkey physiological data involved collection of saliva within 10 min pre- and post-interaction during Times 1, 2, and 3 in association with the different types of interactions for immunoanalysis of cortisol. There were no significant effects of the various times and types of interactions on CCAS scores. While there were no significant effects of group and types of interactions on PSWQ scores, there was an effect (P = 0.01) of time. Overall mean PSWQ scores were significantly lower during Week 12 versus Week 2. Correspondingly, while there were no effects pre- vs. post-interaction within or among times on saliva cortisol concentrations in donkeys, there was an effect (P = 0.02) of the type of interaction. Mean concentrations were significantly lower with the hands-on passive and hands-on active learning versus the hands-off remote learning. In conclusion, while this study provides preliminary evidence surrounding student donkey interactions, future studies are required with more comprehensive designs to clarify these benefits and better understand the advantages and challenges surrounding student-donkey interactions. |
  | Date | 2021 |
  | Language | English |
  | Archive | Embase |
  | URL | https://www.embase.com/search/results?subaction=viewrecord&id=L635900541&from=export |
  | Volume | 8 |
  | Publication | Frontiers in Veterinary Science |
  | DOI | 10.3389/fvets.2021.701302 |
  | Issue | (Artemiou E., eartemiou@rossvet.edu.kn; Pereira M.M.) Department of Clinical Sciences, Ross University School of Veterinary Medicine, Basseterre, Saint Kitts and Nevis |
  | Journal Abbr | Front. Vet. Sci. |
  | ISSN | 2297-1769 |
  | Date Added | 11/07/2025, 14:18:25 |
  | Modified | 11/07/2025, 14:18:25 |

  ### Tags:

  - human-animal interaction
  - saliva
  - animal experiment
  - article
  - female
  - male
  - nonhuman
  - human
  - adult
  - human experiment
  - rating scale
  - learning
  - psychological aspect
  - immunoassay
  - Penn State Worry Questionnaire
  - comfort
  - concentration ratio
  - donkey
  - physiological process
  - veterinary student

  ### Attachments

  - Full Text (HTML)
- ## The Health Benefits of Visiting a Zoo, Park, and Aquarium for Older Japanese

  |  |  |
  | --- | --- |
  | Item Type | Journal Article |
  | Author | J. Akiyama |
  | Author | T. Sakagami |
  | Author | H. Uchiyama |
  | Author | M. Ohta |
  | Abstract | In Japan, older people are expected to comprise 30% of the population as early as 2025. We hypothesized that visiting a zoo or aquarium might be good for their health, compared with visiting a nearby park. We conducted two studies with older Japanese participants to compare the psychological and physical effects of visiting a zoo or aquarium with those of visiting a park. In these studies, we administered the Profile of Mood States-Brief questionnaire and measured blood pressure, pulse, and salivary hormones before and after visits. In the first study, 30 participants visited a zoo, a park, and an aquarium on separate days. They also participated in a video-watching experiment as a control. The visitors’ cortisol and/or blood pressure fell significantly (p < 0.05) after visiting the zoo and park. Also, mood improved on a few scales during the zoo visit. Visiting the park had almost the same health benefits as visiting the zoo. To clarify the difference between the two, in the second study, we recruited new older volunteers and assessed changes in salivary oxytocin and cortisol during zoo and park visits. Oxytocin increased significantly (p < 0.05) and cortisol decreased during visits to the zoo, and post-visit oxytocin levels were lower for park visits than zoo visits. |
  | Date | 2021 |
  | Language | English |
  | Archive | Embase |
  | URL | https://www.embase.com/search/results?subaction=viewrecord&id=L2010853203&from=export |
  | Volume | 34 |
  | Pages | 463-473 |
  | Publication | Anthrozoos |
  | DOI | 10.1080/08927936.2021.1898211 |
  | Issue | 3 |
  | Journal Abbr | Anthrozoos |
  | ISSN | 1753-0377 |
  | Date Added | 11/07/2025, 12:13:27 |
  | Modified | 11/07/2025, 12:13:27 |

  ### Tags:

  - blood pressure
  - oxytocin
  - human-animal interaction
  - article
  - female
  - male
  - zoo animal
  - human
  - hydrocortisone
  - aged
  - ELISA kit
  - human experiment
  - questionnaire
  - mood
  - videorecording
  - clinical article
  - enzyme linked immunosorbent assay
  - blood pressure monitoring
  - pulse rate
  - cotton swab
  - hormone
  - anger
  - bath
  - confusion
  - fatigue
  - MURON HJ325
  - OMURON HEM-650
  - pedometer
  - polypropylene
  - Profile of Mood States
  - radioimmunoassay
  - sphygmomanometer
  - tension
  - tube

  ### Attachments

  - Full Text (HTML)
- ## The Health Benefits of Visiting a Zoo, Park, and Aquarium for Older Japanese

  |  |  |
  | --- | --- |
  | Item Type | Journal Article |
  | Author | J. Akiyama |
  | Author | T. Sakagami |
  | Author | H. Uchiyama |
  | Author | M. Ohta |
  | Abstract | In Japan, older people are expected to comprise 30% of the population as early as 2025. We hypothesized that visiting a zoo or aquarium might be good for their health, compared with visiting a nearby park. We conducted two studies with older Japanese participants to compare the psychological and physical effects of visiting a zoo or aquarium with those of visiting a park. In these studies, we administered the Profile of Mood States-Brief questionnaire and measured blood pressure, pulse, and salivary hormones before and after visits. In the first study, 30 participants visited a zoo, a park, and an aquarium on separate days. They also participated in a video-watching experiment as a control. The visitors’ cortisol and/or blood pressure fell significantly (p < 0.05) after visiting the zoo and park. Also, mood improved on a few scales during the zoo visit. Visiting the park had almost the same health benefits as visiting the zoo. To clarify the difference between the two, in the second study, we recruited new older volunteers and assessed changes in salivary oxytocin and cortisol during zoo and park visits. Oxytocin increased significantly (p < 0.05) and cortisol decreased during visits to the zoo, and post-visit oxytocin levels were lower for park visits than zoo visits. |
  | Date | 2021 |
  | Language | English |
  | Archive | Embase |
  | URL | https://www.embase.com/search/results?subaction=viewrecord&id=L2010853203&from=export |
  | Volume | 34 |
  | Pages | 463-473 |
  | Publication | Anthrozoos |
  | DOI | 10.1080/08927936.2021.1898211 |
  | Issue | 3 |
  | Journal Abbr | Anthrozoos |
  | ISSN | 1753-0377 |
  | Date Added | 11/07/2025, 14:16:55 |
  | Modified | 11/07/2025, 14:16:55 |

  ### Tags:

  - blood pressure
  - oxytocin
  - human-animal interaction
  - article
  - female
  - male
  - zoo animal
  - human
  - hydrocortisone
  - aged
  - ELISA kit
  - human experiment
  - questionnaire
  - mood
  - videorecording
  - clinical article
  - enzyme linked immunosorbent assay
  - blood pressure monitoring
  - pulse rate
  - cotton swab
  - hormone
  - anger
  - bath
  - confusion
  - fatigue
  - MURON HJ325
  - OMURON HEM-650
  - pedometer
  - polypropylene
  - Profile of Mood States
  - radioimmunoassay
  - sphygmomanometer
  - tension
  - tube

  ### Attachments

  - Full Text (HTML)
- ## The Health Benefits of Visiting a Zoo, Park, and Aquarium for Older Japanese

  |  |  |
  | --- | --- |
  | Item Type | Journal Article |
  | Author | J. Akiyama |
  | Author | T. Sakagami |
  | Author | H. Uchiyama |
  | Author | M. Ohta |
  | Abstract | In Japan, older people are expected to comprise 30% of the population as early as 2025. We hypothesized that visiting a zoo or aquarium might be good for their health, compared with visiting a nearby park. We conducted two studies with older Japanese participants to compare the psychological and physical effects of visiting a zoo or aquarium with those of visiting a park. In these studies, we administered the Profile of Mood States-Brief questionnaire and measured blood pressure, pulse, and salivary hormones before and after visits. In the first study, 30 participants visited a zoo, a park, and an aquarium on separate days. They also participated in a video-watching experiment as a control. The visitors’ cortisol and/or blood pressure fell significantly (p < 0.05) after visiting the zoo and park. Also, mood improved on a few scales during the zoo visit. Visiting the park had almost the same health benefits as visiting the zoo. To clarify the difference between the two, in the second study, we recruited new older volunteers and assessed changes in salivary oxytocin and cortisol during zoo and park visits. Oxytocin increased significantly (p < 0.05) and cortisol decreased during visits to the zoo, and post-visit oxytocin levels were lower for park visits than zoo visits. |
  | Date | 2021 |
  | Language | English |
  | Archive | Embase |
  | URL | https://www.embase.com/search/results?subaction=viewrecord&id=L2010853203&from=export |
  | Volume | 34 |
  | Pages | 463-473 |
  | Publication | Anthrozoos |
  | DOI | 10.1080/08927936.2021.1898211 |
  | Issue | 3 |
  | Journal Abbr | Anthrozoos |
  | ISSN | 1753-0377 |
  | Date Added | 11/07/2025, 14:18:28 |
  | Modified | 11/07/2025, 14:18:28 |

  ### Tags:

  - blood pressure
  - oxytocin
  - human-animal interaction
  - article
  - female
  - male
  - zoo animal
  - human
  - hydrocortisone
  - aged
  - ELISA kit
  - human experiment
  - questionnaire
  - mood
  - videorecording
  - clinical article
  - enzyme linked immunosorbent assay
  - blood pressure monitoring
  - pulse rate
  - cotton swab
  - hormone
  - anger
  - bath
  - confusion
  - fatigue
  - MURON HJ325
  - OMURON HEM-650
  - pedometer
  - polypropylene
  - Profile of Mood States
  - radioimmunoassay
  - sphygmomanometer
  - tension
  - tube

  ### Attachments

  - Full Text (HTML)
- ## Hormonal and neurological aspects of dog walking for dog owners and pet dogs

  |  |  |
  | --- | --- |
  | Item Type | Journal Article |
  | Author | J. Akiyama |
  | Author | M. Ohta |
  | Abstract | The hormone oxytocin is involved in various aspects of the relationship between humans and animals. Dog walking is a common activity for dog owners and their dogs. The walk, of course, should be good for the health of the dog as well as its owner. In Experiment I, we assessed whether salivary oxytocin and cortisol in dog owners changed because of walking their dogs. Ten owners walked with their dogs and walked alone. Similar to other previous research, walking with a dog did not significantly change oxytocin and cortisol. Therefore, in Experiment II, we investigated the effect of dog walking on brain noradrenergic and GABAergic neural activity, as indicated by salivary MHPG and GABA, in 14 dog owners. Walking with a dog reduced salivary MHPG compared to walking alone, and MHPG was correlated negatively with GABA. Thus, dog walking activated GABAergic nerves in the brain and suppressed noradrenergic nerves, effectively relieving stress. |
  | Date | 2021 |
  | Language | English |
  | Archive | Embase |
  | URL | https://www.embase.com/search/results?subaction=viewrecord&id=L2013789973&from=export |
  | Volume | 11 |
  | Publication | Animals |
  | DOI | 10.3390/ani11092732 |
  | Issue | 9 |
  | Journal Abbr | Animals |
  | ISSN | 2076-2615 |
  | Date Added | 11/07/2025, 14:16:51 |
  | Modified | 11/07/2025, 14:16:51 |

  ### Tags:

  - oxytocin
  - dog
  - saliva
  - article
  - female
  - male
  - nonhuman
  - physiological stress
  - human
  - hydrocortisone
  - adult
  - aged
  - ELISA kit
  - epinephrine
  - noradrenalin
  - golden retriever
  - unclassified drug
  - human experiment
  - pet animal
  - dopamine
  - human-animal bond
  - middle aged
  - enzyme linked immunosorbent assay
  - walking
  - cotton swab
  - dog breed
  - Labrador retriever
  - hormone
  - serotonin
  - high performance liquid chromatography
  - methanol
  - 3,4 dihydroxyphenylacetic acid
  - 4 aminobutyric acid
  - 4 hydroxy 3 methoxyphenylethylene glycol
  - acetic acid
  - area under the curve
  - brain
  - citrate buffer
  - edetic acid
  - electrochemical detector
  - filter
  - GABAergic signaling
  - high performance liquid chromatography column
  - laboratory device
  - Mentip
  - metabolic fingerprinting
  - miniature dachshund
  - monoamine
  - neurology
  - noradrenergic nerve
  - phthalaldehyde
  - Shiba Inu
  - silver chloride
  - toy poodle

  ### Attachments

  - Full Text (HTML)
- ## Hormonal and neurological aspects of dog walking for dog owners and pet dogs

  |  |  |
  | --- | --- |
  | Item Type | Journal Article |
  | Author | J. Akiyama |
  | Author | M. Ohta |
  | Abstract | The hormone oxytocin is involved in various aspects of the relationship between humans and animals. Dog walking is a common activity for dog owners and their dogs. The walk, of course, should be good for the health of the dog as well as its owner. In Experiment I, we assessed whether salivary oxytocin and cortisol in dog owners changed because of walking their dogs. Ten owners walked with their dogs and walked alone. Similar to other previous research, walking with a dog did not significantly change oxytocin and cortisol. Therefore, in Experiment II, we investigated the effect of dog walking on brain noradrenergic and GABAergic neural activity, as indicated by salivary MHPG and GABA, in 14 dog owners. Walking with a dog reduced salivary MHPG compared to walking alone, and MHPG was correlated negatively with GABA. Thus, dog walking activated GABAergic nerves in the brain and suppressed noradrenergic nerves, effectively relieving stress. |
  | Date | 2021 |
  | Language | English |
  | Archive | Embase |
  | URL | https://www.embase.com/search/results?subaction=viewrecord&id=L2013789973&from=export |
  | Volume | 11 |
  | Publication | Animals |
  | DOI | 10.3390/ani11092732 |
  | Issue | 9 |
  | Journal Abbr | Animals |
  | ISSN | 2076-2615 |
  | Date Added | 11/07/2025, 14:18:24 |
  | Modified | 11/07/2025, 14:18:24 |

  ### Tags:

  - oxytocin
  - dog
  - saliva
  - article
  - female
  - male
  - nonhuman
  - physiological stress
  - human
  - hydrocortisone
  - adult
  - aged
  - ELISA kit
  - epinephrine
  - noradrenalin
  - golden retriever
  - unclassified drug
  - human experiment
  - pet animal
  - dopamine
  - human-animal bond
  - middle aged
  - enzyme linked immunosorbent assay
  - walking
  - cotton swab
  - dog breed
  - Labrador retriever
  - hormone
  - serotonin
  - high performance liquid chromatography
  - methanol
  - 3,4 dihydroxyphenylacetic acid
  - 4 aminobutyric acid
  - 4 hydroxy 3 methoxyphenylethylene glycol
  - acetic acid
  - area under the curve
  - brain
  - citrate buffer
  - edetic acid
  - electrochemical detector
  - filter
  - GABAergic signaling
  - high performance liquid chromatography column
  - laboratory device
  - Mentip
  - metabolic fingerprinting
  - miniature dachshund
  - monoamine
  - neurology
  - noradrenergic nerve
  - phthalaldehyde
  - Shiba Inu
  - silver chloride
  - toy poodle

  ### Attachments

  - Full Text (HTML)
- ## The effect of behind-the-scenes encounters and interactive presentations on the welfare of captive servals (Leptailurus serval)

  |  |  |
  | --- | --- |
  | Item Type | Journal Article |
  | Author | L.K. Acaralp-Rehnberg |
  | Author | G.J. Coleman |
  | Author | M.J.L. Magrath |
  | Author | V. Melfi |
  | Author | K.V. Fanson |
  | Author | I.M. Bland |
  | Abstract | The serval (Leptailurus serval) is a small African felid that is well represented in zoos and often serves as an animal ambassador in encounter programs with zoo visitors. The impact on serval welfare in relation to such programs has not been investigated to date, and the aim of this study was to assess short-term welfare effects of varying levels of visitor interaction in two captive servals. Weekly blocks of four different treatments were imposed three times on each animal over 12 weeks, and the treatments involved (1) Presentations (serval undertaking a routine training session in a designated presentation space, typically attracting high visitor numbers), (2) Behind-the-scenes (BTS, a close encounter allowing a small group of visitors to interact closely with the cat in its enclosure), (3) Presentations and BTS combined, and (4) No visitor interaction. Serval activity budgets as well as behavioural diversity were created from behaviours observed from Close Circuit Television (CCTV) footage during four daily recording sessions per animal over three consecutive days per treatment, using instantaneous scan sampling every 60 s. Individual faecal samples were collected daily to monitor changes in faecal glucocorticoid metabolite (FGM) concentration. Results indicate that the mean number of scans with stereotypic pacing was significantly reduced (p = 0.01) during Treatments 1 and 3, when cats participated in presentations only, or the two activities combined. Conversely, a significant reduction in behavioural diversity (p < 0.001) was observed when cats participated in Treatment 3, i.e., cats expressed fewer behaviours when interaction with visitors was more frequent. FGM concentrations did not vary significantly with treatment (p > 0.05). Given the reduction in stereotypic pacing, these findings suggest that involvement in an encounter program appears to exert an overall positive short-term welfare effect on the individual servals in this study. Although a reduction in behavioural diversity was not considered a negative welfare effect in the short term, potential long-term negative welfare effects resulting from a more frequent encounter program could not be ruled out in the present study. |
  | Date | 2020 |
  | Language | English |
  | Archive | Embase |
  | URL | https://www.embase.com/search/results?subaction=viewrecord&id=L2004233437&from=export |
  | Volume | 10 |
  | Publication | Animals |
  | DOI | 10.3390/ani10040743 |
  | Issue | 4 |
  | Journal Abbr | Animals |
  | ISSN | 2076-2615 |
  | Date Added | 11/07/2025, 14:16:56 |
  | Modified | 11/07/2025, 14:16:56 |

  ### Tags:

  - animal welfare
  - animal behavior
  - article
  - glucocorticoid
  - nonhuman
  - physiological stress
  - hydrocortisone
  - adult
  - behavior assessment
  - human-animal bond
  - training
  - adrenal cortex
  - behavioral stress
  - compulsion
  - corticosterone
  - encounter group
  - Felidae
  - foraging behavior
  - Leptailurus serval
  - physical stimulation

  ### Attachments

  - Full Text (HTML)
- ## The effect of behind-the-scenes encounters and interactive presentations on the welfare of captive servals (Leptailurus serval)

  |  |  |
  | --- | --- |
  | Item Type | Journal Article |
  | Author | L.K. Acaralp-Rehnberg |
  | Author | G.J. Coleman |
  | Author | M.J.L. Magrath |
  | Author | V. Melfi |
  | Author | K.V. Fanson |
  | Author | I.M. Bland |
  | Abstract | The serval (Leptailurus serval) is a small African felid that is well represented in zoos and often serves as an animal ambassador in encounter programs with zoo visitors. The impact on serval welfare in relation to such programs has not been investigated to date, and the aim of this study was to assess short-term welfare effects of varying levels of visitor interaction in two captive servals. Weekly blocks of four different treatments were imposed three times on each animal over 12 weeks, and the treatments involved (1) Presentations (serval undertaking a routine training session in a designated presentation space, typically attracting high visitor numbers), (2) Behind-the-scenes (BTS, a close encounter allowing a small group of visitors to interact closely with the cat in its enclosure), (3) Presentations and BTS combined, and (4) No visitor interaction. Serval activity budgets as well as behavioural diversity were created from behaviours observed from Close Circuit Television (CCTV) footage during four daily recording sessions per animal over three consecutive days per treatment, using instantaneous scan sampling every 60 s. Individual faecal samples were collected daily to monitor changes in faecal glucocorticoid metabolite (FGM) concentration. Results indicate that the mean number of scans with stereotypic pacing was significantly reduced (p = 0.01) during Treatments 1 and 3, when cats participated in presentations only, or the two activities combined. Conversely, a significant reduction in behavioural diversity (p < 0.001) was observed when cats participated in Treatment 3, i.e., cats expressed fewer behaviours when interaction with visitors was more frequent. FGM concentrations did not vary significantly with treatment (p > 0.05). Given the reduction in stereotypic pacing, these findings suggest that involvement in an encounter program appears to exert an overall positive short-term welfare effect on the individual servals in this study. Although a reduction in behavioural diversity was not considered a negative welfare effect in the short term, potential long-term negative welfare effects resulting from a more frequent encounter program could not be ruled out in the present study. |
  | Date | 2020 |
  | Language | English |
  | Archive | Embase |
  | URL | https://www.embase.com/search/results?subaction=viewrecord&id=L2004233437&from=export |
  | Volume | 10 |
  | Publication | Animals |
  | DOI | 10.3390/ani10040743 |
  | Issue | 4 |
  | Journal Abbr | Animals |
  | ISSN | 2076-2615 |
  | Date Added | 11/07/2025, 14:18:30 |
  | Modified | 11/07/2025, 14:18:30 |

  ### Tags:

  - animal welfare
  - animal behavior
  - article
  - glucocorticoid
  - nonhuman
  - physiological stress
  - hydrocortisone
  - adult
  - behavior assessment
  - human-animal bond
  - training
  - adrenal cortex
  - behavioral stress
  - compulsion
  - corticosterone
  - encounter group
  - Felidae
  - foraging behavior
  - Leptailurus serval
  - physical stimulation

  ### Attachments

  - Full Text (HTML)
