# Supplementary material for: Evidence of Physiological Comodulation During Human–Animal Interaction: A Systematic Review
Source: Ann N Y Acad Sci. 2026 Jun 4;1560(1):e70299. doi: 10.1111/nyas.70299 (PMC13238372; doi:10.1111/nyas.70299)
Supplement: Supplementary file 2 — Supplementary Materials: Supp2‐Zotero‐Collection.zip [file NYAS-1560-0-s002.zip › Supp2_Zotero_Collection/title screened/Google Scholar.htm]

Zotero Report


- ## Human-Animal Interaction and Child Health and Development

  |  |  |
  | --- | --- |
  | Item Type | Book Section |
  | Book Author | Megan K. Mueller |
  | Date | 2021 |
  | Language | en |
  | Library Catalogue | Crossref |
  | URL | http://link.springer.com/10.1007/978-3-030-64085-9\_5 |
  | Accessed | 11/07/2025, 09:27:21 |
  | Rights | http://www.springer.com/tdm |
  | Place | Cham |
  | Publisher | Springer International Publishing |
  | ISBN | 978-3-030-64084-2 978-3-030-64085-9 |
  | Pages | 53-67 |
  | Book Title | SpringerBriefs in Well-Being and Quality of Life Research |
  | DOI | 10.1007/978-3-030-64085-9\_5 |
  | ISSN | 2211-7644, 2211-7652 |
  | Date Added | 11/07/2025, 09:27:42 |
  | Modified | 11/07/2025, 09:27:42 |
- ## Research Challenges and Gaps on the Recent Advances in Animal Emotion Recognition

  |  |  |
  | --- | --- |
  | Item Type | Book Section |
  | Book Author | Vaisnavi |
  | Book Author | Vaishali GaneshKumar |
  | Book Author | K. Anirudh Chakravarty |
  | Book Author | Arti Arya |
  | Book Author | R. Prema |
  | Date | 2025 |
  | Language | en |
  | Library Catalogue | Crossref |
  | URL | https://link.springer.com/10.1007/978-981-96-1758-6\_35 |
  | Accessed | 11/07/2025, 09:27:38 |
  | Rights | https://www.springernature.com/gp/researchers/text-and-data-mining |
  | Place | Singapore |
  | Publisher | Springer Nature Singapore |
  | ISBN | 978-981-96-1757-9 978-981-96-1758-6 |
  | Pages | 423-434 |
  | Book Title | Lecture Notes in Networks and Systems |
  | DOI | 10.1007/978-981-96-1758-6\_35 |
  | ISSN | 2367-3370, 2367-3389 |
  | Date Added | 11/07/2025, 09:27:42 |
  | Modified | 11/07/2025, 09:27:42 |
- ## Human-Animal Interaction and Child Health and Development

  |  |  |
  | --- | --- |
  | Item Type | Book Section |
  | Book Author | Megan K. Mueller |
  | Date | 2021 |
  | Language | en |
  | Library Catalogue | Crossref |
  | URL | http://link.springer.com/10.1007/978-3-030-64085-9\_5 |
  | Accessed | 11/07/2025, 10:07:17 |
  | Rights | http://www.springer.com/tdm |
  | Place | Cham |
  | Publisher | Springer International Publishing |
  | ISBN | 978-3-030-64084-2 978-3-030-64085-9 |
  | Pages | 53-67 |
  | Book Title | SpringerBriefs in Well-Being and Quality of Life Research |
  | DOI | 10.1007/978-3-030-64085-9\_5 |
  | ISSN | 2211-7644, 2211-7652 |
  | Date Added | 11/07/2025, 10:07:52 |
  | Modified | 11/07/2025, 10:07:52 |
- ## Pet Ownership and Health

  |  |  |
  | --- | --- |
  | Item Type | Book Section |
  | Book Author | Judith M. Siegel |
  | Date | 2011 |
  | Language | en |
  | Library Catalogue | Crossref |
  | URL | http://link.springer.com/10.1007/978-1-4419-9761-6\_10 |
  | Accessed | 11/07/2025, 10:07:52 |
  | Place | New York, NY |
  | Publisher | Springer New York |
  | ISBN | 978-1-4419-9760-9 978-1-4419-9761-6 |
  | Pages | 167-177 |
  | Book Title | The Psychology of the Human-Animal Bond |
  | DOI | 10.1007/978-1-4419-9761-6\_10 |
  | Date Added | 11/07/2025, 10:07:52 |
  | Modified | 11/07/2025, 10:07:52 |

  ### Attachments

  - Available Version (via Google Scholar)
- ## An Overview of the History, Rationale, and Clinical Application of the Use of EAS in Veterans with Psychiatric Disorders

  |  |  |
  | --- | --- |
  | Item Type | Book Section |
  | Book Author | William R. Marchand |
  | Date | 2025 |
  | Language | en |
  | Library Catalogue | Crossref |
  | URL | https://link.springer.com/10.1007/978-3-031-74426-6\_2 |
  | Accessed | 11/07/2025, 10:20:23 |
  | Rights | https://www.springernature.com/gp/researchers/text-and-data-mining |
  | Place | Cham |
  | Publisher | Springer Nature Switzerland |
  | ISBN | 978-3-031-74425-9 978-3-031-74426-6 |
  | Pages | 25-51 |
  | Book Title | Guide to Equine Assisted Therapy |
  | DOI | 10.1007/978-3-031-74426-6\_2 |
  | Date Added | 11/07/2025, 10:20:29 |
  | Modified | 11/07/2025, 10:20:29 |
- ## Saliva Collection, Handling, Transport, and Storage: Special Considerations and Best Practices for Interdisciplinary Salivary Bioscience Research

  |  |  |
  | --- | --- |
  | Item Type | Book Section |
  | Book Author | Genieleah A. Padilla |
  | Book Author | Jessica L. Calvi |
  | Book Author | Marcus K. Taylor |
  | Book Author | Douglas A. Granger |
  | Date | 2020 |
  | Language | en |
  | Short Title | Saliva Collection, Handling, Transport, and Storage |
  | Library Catalogue | Crossref |
  | URL | http://link.springer.com/10.1007/978-3-030-35784-9\_3 |
  | Accessed | 11/07/2025, 11:15:40 |
  | Rights | http://www.springer.com/tdm |
  | Place | Cham |
  | Publisher | Springer International Publishing |
  | ISBN | 978-3-030-35783-2 978-3-030-35784-9 |
  | Pages | 21-47 |
  | Book Title | Salivary Bioscience |
  | DOI | 10.1007/978-3-030-35784-9\_3 |
  | Date Added | 11/07/2025, 11:15:52 |
  | Modified | 11/07/2025, 11:15:52 |
- ## Antarctic tourism research: the first half-century.

  |  |  |
  | --- | --- |
  | Item Type | Book Section |
  | Book Author | B. Stonehouse |
  | Book Author | K. Crosbie |
  | Date | 01/2007 |
  | Language | en |
  | Short Title | Antarctic tourism research |
  | Library Catalogue | Crossref |
  | URL | http://www.cabidigitallibrary.org/doi/10.1079/9781845932473.0210 |
  | Accessed | 11/07/2025, 11:15:47 |
  | Place | UK |
  | Publisher | CABI |
  | ISBN | 978-1-84593-247-3 978-1-84593-248-0 |
  | Pages | 210-228 |
  | Edition | 1 |
  | Book Title | Prospects for polar tourism |
  | DOI | 10.1079/9781845932473.0210 |
  | Date Added | 11/07/2025, 11:15:52 |
  | Modified | 11/07/2025, 11:15:52 |
- ## An Overview of the History, Rationale, and Clinical Application of the Use of EAS in Veterans with Psychiatric Disorders

  |  |  |
  | --- | --- |
  | Item Type | Book Section |
  | Book Author | William R. Marchand |
  | Date | 2025 |
  | Language | en |
  | Library Catalogue | Crossref |
  | URL | https://link.springer.com/10.1007/978-3-031-74426-6\_2 |
  | Accessed | 11/07/2025, 13:44:51 |
  | Rights | https://www.springernature.com/gp/researchers/text-and-data-mining |
  | Place | Cham |
  | Publisher | Springer Nature Switzerland |
  | ISBN | 978-3-031-74425-9 978-3-031-74426-6 |
  | Pages | 25-51 |
  | Book Title | Guide to Equine Assisted Therapy |
  | DOI | 10.1007/978-3-031-74426-6\_2 |
  | Date Added | 11/07/2025, 13:45:12 |
  | Modified | 11/07/2025, 13:45:12 |
- ## Effect of Stress on Reproduction and Reproductive Technologies in Male and Female, Beef and Dairy Cattle

  |  |  |
  | --- | --- |
  | Item Type | Book Section |
  | Book Author | Sonia S. Pérez-Garnelo |
  | Book Author | María José Utrilla |
  | Book Author | Aitor Fernández-Novo |
  | Book Author | Ángel Revilla-Ruiz |
  | Book Author | Arantxa Villagrá |
  | Book Author | Susana Astiz |
  | Date | 2024 |
  | Language | en |
  | Library Catalogue | Crossref |
  | URL | https://link.springer.com/10.1007/978-3-031-73079-5\_6 |
  | Accessed | 11/07/2025, 13:45:07 |
  | Rights | https://www.springernature.com/gp/researchers/text-and-data-mining |
  | Place | Cham |
  | Publisher | Springer Nature Switzerland |
  | ISBN | 978-3-031-73078-8 978-3-031-73079-5 |
  | Pages | 127-193 |
  | Book Title | Assisted Reproductive Technologies in Animals Volume 1 |
  | DOI | 10.1007/978-3-031-73079-5\_6 |
  | Date Added | 11/07/2025, 13:45:12 |
  | Modified | 11/07/2025, 13:45:12 |
- ## Systematic Review and Meta-Analyses: Safety and Efficacy of Complementary and Alternative Treatments for Pediatric Attention-Deficit/Hyperactivity Disorder

  |  |  |
  | --- | --- |
  | Item Type | Journal Article |
  | Author | Courtney A. Zulauf-McCurdy |
  | Author | Patrick A. LaCount |
  | Author | Christopher R. Shelton |
  | Author | Anne S. Morrow |
  | Author | Xin A. Zhao |
  | Author | Douglas Russell |
  | Author | Margaret H. Sibley |
  | Author | L. Eugene Arnold |
  | Date | 2023 |
  | Short Title | Systematic Review and Meta-Analyses |
  | Library Catalogue | Google Scholar |
  | URL | https://journals.lww.com/jrnldbp/fulltext/2023/05000/systematic\_review\_and\_meta\_analyses\_\_safety\_and.11.aspx |
  | Accessed | 11/07/2025, 09:57:54 |
  | Volume | 44 |
  | Publisher | LWW |
  | Pages | e322–e332 |
  | Publication | Journal of Developmental & Behavioral Pediatrics |
  | Issue | 4 |
  | Date Added | 11/07/2025, 09:58:25 |
  | Modified | 11/07/2025, 09:58:25 |
- ## A Scoping Review of the Risks Posed by Companion Animals to Older Adults

  |  |  |
  | --- | --- |
  | Item Type | Journal Article |
  | Author | Joshua Zoanetti |
  | Author | Janette Young |
  | Author | Torben Dahl Nielsen |
  | Date | 2024-09-02 |
  | Language | en |
  | Library Catalogue | Crossref |
  | URL | https://www.tandfonline.com/doi/full/10.1080/08927936.2024.2351278 |
  | Accessed | 11/07/2025, 10:16:47 |
  | Rights | http://creativecommons.org/licenses/by-nc-nd/4.0/ |
  | Volume | 37 |
  | Publisher | Informa UK Limited |
  | Pages | 1015-1031 |
  | Publication | Anthrozoös |
  | DOI | 10.1080/08927936.2024.2351278 |
  | Issue | 5 |
  | ISSN | 0892-7936, 1753-0377 |
  | Date Added | 11/07/2025, 10:16:47 |
  | Modified | 11/07/2025, 10:16:47 |
- ## A Systematic Review of the Positive Effects of Pets on Child Development

  |  |  |
  | --- | --- |
  | Item Type | Journal Article |
  | Author | Caoziru Yu |
  | Date | 2024 |
  | Library Catalogue | Google Scholar |
  | URL | https://www.ewadirect.com/proceedings/chr/article/view/15021 |
  | Accessed | 11/07/2025, 10:20:26 |
  | Volume | 39 |
  | Pages | 82–87 |
  | Publication | Communications in Humanities Research |
  | Date Added | 11/07/2025, 10:20:29 |
  | Modified | 11/07/2025, 10:20:29 |
- ## A modest protective association between pet ownership and cardiovascular diseases: A systematic review and meta-analysis

  |  |  |
  | --- | --- |
  | Item Type | Journal Article |
  | Author | Tzu-Lin Yeh |
  | Author | Wei-Te Lei |
  | Author | Shu-Jung Liu |
  | Author | Kuo-Liong Chien |
  | Date | 2019 |
  | Short Title | A modest protective association between pet ownership and cardiovascular diseases |
  | Library Catalogue | Google Scholar |
  | URL | https://journals.plos.org/plosone/article?id=10.1371/journal.pone.0216231 |
  | Accessed | 11/07/2025, 10:16:13 |
  | Volume | 14 |
  | Publisher | Public Library of Science San Francisco, CA USA |
  | Pages | e0216231 |
  | Publication | PLoS One |
  | Issue | 5 |
  | Date Added | 11/07/2025, 10:16:47 |
  | Modified | 11/07/2025, 10:16:47 |

  ### Attachments

  - Available Version (via Google Scholar)
- ## The effects of hippotherapy in children and adolescents with autism: a systematic review

  |  |  |
  | --- | --- |
  | Item Type | Journal Article |
  | Author | Emily Layne Ybarbo |
  | Date | 2017 |
  | Short Title | The effects of hippotherapy in children and adolescents with autism |
  | Library Catalogue | Google Scholar |
  | URL | https://repositories.lib.utexas.edu/items/37fd8ca6-ed53-4441-abe9-e8fefb67cfc8 |
  | Accessed | 11/07/2025, 11:01:31 |
  | Date Added | 11/07/2025, 11:01:44 |
  | Modified | 11/07/2025, 11:01:44 |

  ### Attachments

  - Available Version (via Google Scholar)
- ## Effects of equine-assisted activities and therapies for individuals with autism spectrum disorder: systematic review and meta-analysis

  |  |  |
  | --- | --- |
  | Item Type | Journal Article |
  | Author | Ningkun Xiao |
  | Author | Khyber Shinwari |
  | Author | Sergey Kiselev |
  | Author | Xinlin Huang |
  | Author | Baoheng Li |
  | Author | Jingjing Qi |
  | Date | 2023 |
  | Short Title | Effects of equine-assisted activities and therapies for individuals with autism spectrum disorder |
  | Library Catalogue | Google Scholar |
  | URL | https://www.mdpi.com/1660-4601/20/3/2630 |
  | Accessed | 11/07/2025, 09:36:06 |
  | Volume | 20 |
  | Publisher | MDPI |
  | Pages | 2630 |
  | Publication | International journal of environmental research and public health |
  | Issue | 3 |
  | Date Added | 11/07/2025, 09:36:11 |
  | Modified | 11/07/2025, 09:36:11 |

  ### Attachments

  - Available Version (via Google Scholar)
- ## Effectiveness of animal-assisted activities and therapies for autism spectrum disorder: A systematic review and meta-analysis

  |  |  |
  | --- | --- |
  | Item Type | Journal Article |
  | Author | Ningkun Xiao |
  | Author | Vaishnavi Bagayi |
  | Author | Dandan Yang |
  | Author | Xinlin Huang |
  | Author | Lei Zhong |
  | Author | Sergey Kiselev |
  | Author | Mikhail A. Bolkov |
  | Author | Irina A. Tuzankina |
  | Author | Valery A. Chereshnev |
  | Date | 2024 |
  | Short Title | Effectiveness of animal-assisted activities and therapies for autism spectrum disorder |
  | Library Catalogue | Google Scholar |
  | URL | https://www.frontiersin.org/articles/10.3389/fvets.2024.1403527/full |
  | Accessed | 11/07/2025, 13:46:25 |
  | Volume | 11 |
  | Publisher | Frontiers Media SA |
  | Pages | 1403527 |
  | Publication | Frontiers in Veterinary Science |
  | Date Added | 11/07/2025, 13:46:33 |
  | Modified | 11/07/2025, 13:46:33 |

  ### Attachments

  - Available Version (via Google Scholar)
- ## Guidance on Conducting a Systematic Literature Review

  |  |  |
  | --- | --- |
  | Item Type | Journal Article |
  | Author | Yu Xiao |
  | Author | Maria Watson |
  | Abstract | Literature reviews establish the foundation of academic inquires. However, in the planning field, we lack rigorous systematic reviews. In this article, through a systematic search on the methodology of literature review, we categorize a typology of literature reviews, discuss steps in conducting a systematic literature review, and provide suggestions on how to enhance rigor in literature reviews in planning education and research. |
  | Date | 03/2019 |
  | Language | en |
  | Library Catalogue | Crossref |
  | URL | https://journals.sagepub.com/doi/10.1177/0739456X17723971 |
  | Accessed | 11/07/2025, 09:38:03 |
  | Rights | https://journals.sagepub.com/page/policies/text-and-data-mining-license |
  | Volume | 39 |
  | Publisher | SAGE Publications |
  | Pages | 93-112 |
  | Publication | Journal of Planning Education and Research |
  | DOI | 10.1177/0739456x17723971 |
  | Issue | 1 |
  | ISSN | 0739-456X, 1552-6577 |
  | Date Added | 11/07/2025, 09:38:03 |
  | Modified | 11/07/2025, 09:38:03 |

  ### Attachments

  - Available Version (via Google Scholar)
- ## Bird welfare in zoos and aquariums: general insights across industries

  |  |  |
  | --- | --- |
  | Item Type | Journal Article |
  | Author | Jocelyn M. Woods |
  | Author | Adrienne Eyer |
  | Author | Lance J. Miller |
  | Date | 2022 |
  | Short Title | Bird welfare in zoos and aquariums |
  | Library Catalogue | Google Scholar |
  | URL | https://www.mdpi.com/2673-5636/3/2/17 |
  | Accessed | 11/07/2025, 09:31:55 |
  | Volume | 3 |
  | Publisher | MDPI |
  | Pages | 198–222 |
  | Publication | Journal of Zoological and Botanical Gardens |
  | Issue | 2 |
  | Date Added | 11/07/2025, 09:32:09 |
  | Modified | 11/07/2025, 09:32:09 |
- ## Animal-assisted therapy in cardiovascular disease

  |  |  |
  | --- | --- |
  | Item Type | Conference Paper |
  | Author | Andrew I. Wolff |
  | Author | William H. Frishman |
  | Date | 2004 |
  | Library Catalogue | Google Scholar |
  | URL | https://www.sciencedirect.com/science/article/pii/S1543115005000050 |
  | Accessed | 11/07/2025, 10:07:32 |
  | Volume | 2 |
  | Publisher | Elsevier |
  | Pages | 131–134 |
  | Proceedings Title | Seminars in Integrative Medicine |
  | Issue | 4 |
  | Date Added | 11/07/2025, 10:07:52 |
  | Modified | 11/07/2025, 10:07:52 |
- ## Social and emotional therapy dog-assisted interventions in mainstream school settings: a systematic review

  |  |  |
  | --- | --- |
  | Item Type | Journal Article |
  | Author | Lena Wintermantel |
  | Author | Christine Grove |
  | Author | Linda Henderson |
  | Author | Stella Laletas |
  | Date | 2024-01-02 |
  | Language | en |
  | Short Title | Social and emotional therapy dog-assisted interventions in mainstream school settings |
  | Library Catalogue | Crossref |
  | URL | https://www.tandfonline.com/doi/full/10.1080/20590776.2023.2256444 |
  | Accessed | 11/07/2025, 13:46:00 |
  | Volume | 41 |
  | Publisher | Informa UK Limited |
  | Pages | 74-90 |
  | Publication | Educational and Developmental Psychologist |
  | DOI | 10.1080/20590776.2023.2256444 |
  | Issue | 1 |
  | ISSN | 2059-0776, 2059-0784 |
  | Date Added | 11/07/2025, 13:46:33 |
  | Modified | 11/07/2025, 13:46:33 |
- ## The impact of visitors on non-primate species in zoos: a quantitative review

  |  |  |
  | --- | --- |
  | Item Type | Journal Article |
  | Author | Ellen Williams |
  | Author | Violet Hunton |
  | Author | Geoff Hosey |
  | Author | Samantha J. Ward |
  | Date | 2023 |
  | Short Title | The impact of visitors on non-primate species in zoos |
  | Library Catalogue | Google Scholar |
  | URL | https://www.mdpi.com/2076-2615/13/7/1178 |
  | Accessed | 11/07/2025, 09:29:00 |
  | Volume | 13 |
  | Publisher | MDPI |
  | Pages | 1178 |
  | Publication | Animals |
  | Issue | 7 |
  | Date Added | 11/07/2025, 09:29:28 |
  | Modified | 11/07/2025, 09:29:28 |

  ### Attachments

  - Available Version (via Google Scholar)
- ## The impact of visitors on non-primate species in zoos: a quantitative review

  |  |  |
  | --- | --- |
  | Item Type | Journal Article |
  | Author | Ellen Williams |
  | Author | Violet Hunton |
  | Author | Geoff Hosey |
  | Author | Samantha J. Ward |
  | Date | 2023 |
  | Short Title | The impact of visitors on non-primate species in zoos |
  | Library Catalogue | Google Scholar |
  | URL | https://www.mdpi.com/2076-2615/13/7/1178 |
  | Accessed | 11/07/2025, 10:16:32 |
  | Volume | 13 |
  | Publisher | MDPI |
  | Pages | 1178 |
  | Publication | Animals |
  | Issue | 7 |
  | Date Added | 11/07/2025, 10:16:47 |
  | Modified | 11/07/2025, 10:16:47 |
- ## Assessing cattle welfare at slaughter–Why is it important and what challenges are faced?

  |  |  |
  | --- | --- |
  | Item Type | Journal Article |
  | Author | Eleanor E. Wigham |
  | Author | Andy Butterworth |
  | Author | Steve Wotton |
  | Date | 2018 |
  | Library Catalogue | Google Scholar |
  | URL | https://www.sciencedirect.com/science/article/pii/S0309174018301219 |
  | Accessed | 11/07/2025, 10:16:21 |
  | Volume | 145 |
  | Publisher | Elsevier |
  | Pages | 171–177 |
  | Publication | Meat Science |
  | Date Added | 11/07/2025, 10:16:47 |
  | Modified | 11/07/2025, 10:16:47 |
- ## The State of Research on Human–Animal Relations: Implications for Human Health

  |  |  |
  | --- | --- |
  | Item Type | Journal Article |
  | Author | Deborah L. Wells |
  | Date | 2019-03-04 |
  | Language | en |
  | Short Title | The State of Research on Human–Animal Relations |
  | Library Catalogue | Crossref |
  | URL | https://www.tandfonline.com/doi/full/10.1080/08927936.2019.1569902 |
  | Accessed | 11/07/2025, 10:05:00 |
  | Rights | http://creativecommons.org/licenses/by-nc-nd/4.0/ |
  | Volume | 32 |
  | Publisher | Informa UK Limited |
  | Pages | 169-181 |
  | Publication | Anthrozoös |
  | DOI | 10.1080/08927936.2019.1569902 |
  | Issue | 2 |
  | ISSN | 0892-7936, 1753-0377 |
  | Date Added | 11/07/2025, 10:05:27 |
  | Modified | 11/07/2025, 10:05:27 |

  ### Attachments

  - Available Version (via Google Scholar)
- ## What we can measure, we can manage: The importance of using robust welfare indicators in Equitation Science

  |  |  |
  | --- | --- |
  | Item Type | Journal Article |
  | Author | Natalie Waran |
  | Author | Hayley Randle |
  | Date | 2017 |
  | Short Title | What we can measure, we can manage |
  | Library Catalogue | Google Scholar |
  | URL | https://www.sciencedirect.com/science/article/pii/S0168159117300679 |
  | Accessed | 11/07/2025, 09:27:42 |
  | Volume | 190 |
  | Publisher | Elsevier |
  | Pages | 74–81 |
  | Publication | Applied Animal Behaviour Science |
  | Date Added | 11/07/2025, 09:27:42 |
  | Modified | 04/01/2026, 11:25:42 |
- ## <p>Spotlight on the psychological basis of childhood pet attachment and its implications</p>

  |  |  |
  | --- | --- |
  | Item Type | Journal Article |
  | Author | Shelby H Wanser |
  | Author | Kristyn R Vitale |
  | Author | Lauren E Thielke |
  | Author | Lauren Brubaker |
  | Author | Monique A.R Udell |
  | Date | 06/2019 |
  | Language | en |
  | Library Catalogue | Crossref |
  | URL | https://www.dovepress.com/spotlight-on-the-psychological-basis-of-childhood-pet-attachment-and-i-peer-reviewed-article-PRBM |
  | Accessed | 11/07/2025, 10:09:28 |
  | Rights | http://creativecommons.org/licenses/by-nc/3.0/ |
  | Volume | Volume 12 |
  | Publisher | Informa UK Limited |
  | Pages | 469-479 |
  | Publication | Psychology Research and Behavior Management |
  | DOI | 10.2147/prbm.s158998 |
  | Journal Abbr | PRBM |
  | ISSN | 1179-1578 |
  | Date Added | 11/07/2025, 10:09:48 |
  | Modified | 11/07/2025, 10:09:48 |

  ### Attachments

  - Available Version (via Google Scholar)
- ## Human‐Animal Bonds I: The Relational Significance of Companion Animals

  |  |  |
  | --- | --- |
  | Item Type | Journal Article |
  | Author | Froma Walsh |
  | Abstract | The importance of human‐animal bonds has been documented throughout history, across cultures, and in recent research. However, attachments with companion animals have been undervalued and even pathologized in the field of mental health. This article briefly surveys the evolution of human‐animal bonds, reviews research on their health and mental health benefits, and examines their profound relational significance across the life course. Finally, the emerging field of animal‐assisted interventions is described, noting applications in hospital and eldercare settings, and in innovative school, prison, farm, and community programs. The aim of this overview paper is to stimulate more attention to these vital bonds in systems‐oriented theory, practice, and research. A companion paper in this issue focuses on the role of pets and relational dynamics in family systems and family therapy (Walsh, 2009a). |
  | Date | 12/2009 |
  | Language | en |
  | Short Title | Human‐Animal Bonds I |
  | Library Catalogue | Crossref |
  | URL | https://onlinelibrary.wiley.com/doi/10.1111/j.1545-5300.2009.01296.x |
  | Accessed | 11/07/2025, 09:27:24 |
  | Rights | http://onlinelibrary.wiley.com/termsAndConditions#vor |
  | Volume | 48 |
  | Publisher | Wiley |
  | Pages | 462-480 |
  | Publication | Family Process |
  | DOI | 10.1111/j.1545-5300.2009.01296.x |
  | Issue | 4 |
  | ISSN | 0014-7370, 1545-5300 |
  | Date Added | 11/07/2025, 09:27:42 |
  | Modified | 11/07/2025, 09:27:42 |
- ## 40 More than

  |  |  |
  | --- | --- |
  | Item Type | Journal Article |
  | Author | Jennifer Vonk |
  | Author | Lauri Torgerson-White |
  | Author | Jared Edge |
  | Author | Bridget Benton |
  | Library Catalogue | Google Scholar |
  | URL | https://academic.oup.com/book/56886/book-pdf/58061764/oxfordhb-9780197544754.pdf#page=785 |
  | Accessed | 11/07/2025, 09:37:27 |
  | Pages | 763 |
  | Publication | The Oxford Handbook of Evolution and the Emotions |
  | Date Added | 11/07/2025, 09:38:03 |
  | Modified | 11/07/2025, 09:38:03 |

  ### Attachments

  - Available Version (via Google Scholar)
- ## 40 More than

  |  |  |
  | --- | --- |
  | Item Type | Journal Article |
  | Author | Jennifer Vonk |
  | Author | Lauri Torgerson-White |
  | Author | Jared Edge |
  | Author | Bridget Benton |
  | Library Catalogue | Google Scholar |
  | URL | https://academic.oup.com/book/56886/book-pdf/58061764/oxfordhb-9780197544754.pdf#page=785 |
  | Accessed | 11/07/2025, 11:09:26 |
  | Pages | 763 |
  | Publication | The Oxford Handbook of Evolution and the Emotions |
  | Date Added | 11/07/2025, 11:09:44 |
  | Modified | 11/07/2025, 11:09:44 |
- ## 40 More than

  |  |  |
  | --- | --- |
  | Item Type | Journal Article |
  | Author | Jennifer Vonk |
  | Author | Lauri Torgerson-White |
  | Author | Jared Edge |
  | Author | Bridget Benton |
  | Library Catalogue | Google Scholar |
  | URL | https://academic.oup.com/book/56886/book-pdf/58061764/oxfordhb-9780197544754.pdf#page=785 |
  | Accessed | 11/07/2025, 13:45:06 |
  | Pages | 763 |
  | Publication | The Oxford Handbook of Evolution and the Emotions |
  | Date Added | 11/07/2025, 13:45:12 |
  | Modified | 11/07/2025, 13:45:12 |
- ## Effect of animal-assisted therapy on the psychological and functional status of elderly populations and patients with psychiatric disorders: a meta-analysis

  |  |  |
  | --- | --- |
  | Item Type | Journal Article |
  | Author | Javier Virués-Ortega |
  | Author | Roberto Pastor-Barriuso |
  | Author | Juan M. Castellote |
  | Author | Adolfo Población |
  | Author | Jesús De Pedro-Cuesta |
  | Date | 2012-09-01 |
  | Language | en |
  | Short Title | Effect of animal-assisted therapy on the psychological and functional status of elderly populations and patients with psychiatric disorders |
  | Library Catalogue | Crossref |
  | URL | https://www.tandfonline.com/doi/full/10.1080/17437199.2010.534965 |
  | Accessed | 11/07/2025, 10:13:21 |
  | Volume | 6 |
  | Publisher | Informa UK Limited |
  | Pages | 197-221 |
  | Publication | Health Psychology Review |
  | DOI | 10.1080/17437199.2010.534965 |
  | Issue | 2 |
  | ISSN | 1743-7199, 1743-7202 |
  | Date Added | 11/07/2025, 10:13:55 |
  | Modified | 11/07/2025, 10:13:55 |
- ## Psychophysiological effects of human-animal interaction: Theoretical issues and long-term interaction effects

  |  |  |
  | --- | --- |
  | Item Type | Journal Article |
  | Author | Javier Virués-Ortega |
  | Author | Gualberto Buela-Casal |
  | Date | 2006 |
  | Short Title | Psychophysiological effects of human-animal interaction |
  | Library Catalogue | Google Scholar |
  | URL | https://journals.lww.com/jonmd/fulltext/2006/01000/psychophysiological\_effects\_of\_human\_animal.10.aspx |
  | Accessed | 11/07/2025, 09:27:04 |
  | Volume | 194 |
  | Publisher | LWW |
  | Pages | 52–57 |
  | Publication | The Journal of nervous and mental disease |
  | Issue | 1 |
  | Date Added | 11/07/2025, 09:27:42 |
  | Modified | 11/07/2025, 09:27:42 |

  ### Attachments

  - Available Version (via Google Scholar)
- ## Psychophysiological effects of human-animal interaction: Theoretical issues and long-term interaction effects

  |  |  |
  | --- | --- |
  | Item Type | Journal Article |
  | Author | Javier Virués-Ortega |
  | Author | Gualberto Buela-Casal |
  | Date | 2006 |
  | Short Title | Psychophysiological effects of human-animal interaction |
  | Library Catalogue | Google Scholar |
  | URL | https://journals.lww.com/jonmd/fulltext/2006/01000/psychophysiological\_effects\_of\_human\_animal.10.aspx |
  | Accessed | 11/07/2025, 10:04:50 |
  | Volume | 194 |
  | Publisher | LWW |
  | Pages | 52–57 |
  | Publication | The Journal of nervous and mental disease |
  | Issue | 1 |
  | Date Added | 11/07/2025, 10:05:27 |
  | Modified | 11/07/2025, 10:05:27 |

  ### Attachments

  - Available Version (via Google Scholar)
- ## A review of factors affecting the welfare of weaned replacement heifers in pasture-based dairy production systems

  |  |  |
  | --- | --- |
  | Item Type | Journal Article |
  | Author | Megan Verdon |
  | Date | 2023 |
  | Library Catalogue | Google Scholar |
  | URL | https://www.publish.csiro.au/an/AN22248 |
  | Accessed | 11/07/2025, 11:14:32 |
  | Volume | 63 |
  | Publisher | CSIRO Publishing |
  | Pages | 723–741 |
  | Publication | Animal Production Science |
  | Issue | 8 |
  | Date Added | 11/07/2025, 11:14:45 |
  | Modified | 11/07/2025, 11:14:45 |
- ## Distress Among Hospitalized Pediatric Cancer Patients Modified By Pet-Therapy Intervention to Improve Quality of Life

  |  |  |
  | --- | --- |
  | Item Type | Journal Article |
  | Author | Beth L. Urbanski |
  | Author | Mark Lazenby |
  | Abstract | This state of the science, integrative literature review focuses on animal-facilitated therapy (AFT) and the benefits provided to quality of life in hospitalized pediatric oncology patients. Results showed physiological and psychological benefits in pediatric inpatients settings. AFT has been shown to decrease pain, change vital signs, provide distraction, decrease fear, increase socialization, increase pleasure and decrease emotional distress in hospitalized pediatric patients. AFT needs to be implemented with appropriate medical discretion, but for the appropriate high-risk patients, AFT can improve quality of life. Pain, adjustment difficulties, mood changes and symptom management can be improved in inpatient pediatric cancer patients receiving AFT, thus improving overall quality of life. |
  | Date | 09/2012 |
  | Language | en |
  | Library Catalogue | Crossref |
  | URL | https://journals.sagepub.com/doi/10.1177/1043454212455697 |
  | Accessed | 11/07/2025, 10:09:43 |
  | Rights | https://journals.sagepub.com/page/policies/text-and-data-mining-license |
  | Volume | 29 |
  | Publisher | SAGE Publications |
  | Pages | 272-282 |
  | Publication | Journal of Pediatric Oncology Nursing |
  | DOI | 10.1177/1043454212455697 |
  | Issue | 5 |
  | Journal Abbr | J Pediatr Oncol Nurs |
  | ISSN | 1043-4542, 1532-8457 |
  | Date Added | 11/07/2025, 10:09:48 |
  | Modified | 11/07/2025, 10:09:48 |

  ### Attachments

  - Available Version (via Google Scholar)
- ## Gruppstorlek och sociala behov hos katter och hundar

  |  |  |
  | --- | --- |
  | Item Type | Journal Article |
  | Author | Katarina Tsingos |
  | Date | 2018 |
  | Library Catalogue | Google Scholar |
  | URL | https://stud.epsilon.slu.se/13850/ |
  | Accessed | 11/07/2025, 11:14:18 |
  | Publisher | SLU, Dept. of Animal Environment and Health |
  | Date Added | 11/07/2025, 11:14:45 |
  | Modified | 11/07/2025, 11:14:45 |
- ## Choosing an AASC Animal Partner

  |  |  |
  | --- | --- |
  | Item Type | Journal Article |
  | Author | Melissa E. Trevathan-Minnis |
  | Author | Kimberly Scott |
  | Date | 2024 |
  | Library Catalogue | Google Scholar |
  | URL | https://www.taylorfrancis.com/chapters/edit/10.4324/9781003392415-3/choosing-aasc-animal-partner-melissa-trevathan-minnis-kimberly-scott |
  | Accessed | 11/07/2025, 13:44:46 |
  | Publisher | Routledge |
  | Pages | 26–46 |
  | Publication | Animal-Assisted School Counseling |
  | Date Added | 11/07/2025, 13:45:12 |
  | Modified | 11/07/2025, 13:45:12 |
- ## How do children and adolescents with ASD look at animals? A scoping review

  |  |  |
  | --- | --- |
  | Item Type | Journal Article |
  | Author | Manon Toutain |
  | Author | Nicolas Dollion |
  | Author | Laurence Henry |
  | Author | Marine Grandgeorge |
  | Date | 2024 |
  | Short Title | How do children and adolescents with ASD look at animals? |
  | Library Catalogue | Google Scholar |
  | URL | https://www.mdpi.com/2227-9067/11/2/211 |
  | Accessed | 11/07/2025, 13:44:33 |
  | Volume | 11 |
  | Publisher | MDPI |
  | Pages | 211 |
  | Publication | Children |
  | Issue | 2 |
  | Date Added | 11/07/2025, 13:45:12 |
  | Modified | 11/07/2025, 13:45:12 |

  ### Attachments

  - Available Version (via Google Scholar)
  - Available Version (via Google Scholar)
- ## A Comprehensive Review on COVID-19 Pandemic: Causes, Effects, and Concerns From Environmental Perspective

  |  |  |
  | --- | --- |
  | Item Type | Journal Article |
  | Author | Shyam Thapa |
  | Date | 2021 |
  | Short Title | A Comprehensive Review on COVID-19 Pandemic |
  | Library Catalogue | Google Scholar |
  | URL | https://www.researchgate.net/profile/Shyam-Thapa-6/publication/353037668\_A\_Comprehensive\_Review\_on\_COVID-19\_Pandemic\_Causes\_Effects\_and\_Concerns\_from\_Environmental\_Perspective/links/60e54cec4585156c95e814b9/A-Comprehensive-Review-on-COVID-19-Pandemic-Causes-Effects-and-Concerns-from-Environmental-Perspective.pdf |
  | Accessed | 11/07/2025, 11:15:34 |
  | Volume | 17 |
  | Pages | 196 |
  | Publication | J. Environ. Sci |
  | Date Added | 11/07/2025, 11:15:52 |
  | Modified | 11/07/2025, 11:15:52 |
- ## Can Interacting with Animals Improve Executive Functions? A Systematic Review

  |  |  |
  | --- | --- |
  | Item Type | Journal Article |
  | Author | Deanna Tepper |
  | Author | Joanna Shnookal |
  | Author | Tiffani Howell |
  | Author | Pauleen Bennett |
  | Date | 2023 |
  | Short Title | Can Interacting with Animals Improve Executive Functions? |
  | Library Catalogue | Google Scholar |
  | URL | https://www.mdpi.com/2076-2615/13/13/2080 |
  | Accessed | 11/07/2025, 10:00:23 |
  | Volume | 13 |
  | Publisher | MDPI |
  | Pages | 2080 |
  | Publication | Animals |
  | Issue | 13 |
  | Date Added | 11/07/2025, 10:00:41 |
  | Modified | 11/07/2025, 10:00:41 |

  ### Attachments

  - Available Version (via Google Scholar)
  - Available Version (via Google Scholar)
- ## Psychophysiological mechanisms underlying the potential health benefits of human-dog interactions: A systematic literature review

  |  |  |
  | --- | --- |
  | Item Type | Journal Article |
  | Author | Jillian T. Teo |
  | Author | Stuart J. Johnstone |
  | Author | Stephanie S. Römer |
  | Author | Susan J. Thomas |
  | Date | 2022 |
  | Short Title | Psychophysiological mechanisms underlying the potential health benefits of human-dog interactions |
  | Library Catalogue | Google Scholar |
  | URL | https://www.sciencedirect.com/science/article/pii/S0167876022001787 |
  | Accessed | 11/07/2025, 09:27:07 |
  | Volume | 180 |
  | Publisher | Elsevier |
  | Pages | 27–48 |
  | Publication | International Journal of Psychophysiology |
  | Date Added | 11/07/2025, 09:27:42 |
  | Modified | 11/07/2025, 09:27:42 |
- ## Psychophysiological mechanisms underlying the potential health benefits of human-dog interactions: A systematic literature review

  |  |  |
  | --- | --- |
  | Item Type | Journal Article |
  | Author | Jillian T. Teo |
  | Author | Stuart J. Johnstone |
  | Author | Stephanie S. Römer |
  | Author | Susan J. Thomas |
  | Date | 2022 |
  | Short Title | Psychophysiological mechanisms underlying the potential health benefits of human-dog interactions |
  | Library Catalogue | Google Scholar |
  | URL | https://www.sciencedirect.com/science/article/pii/S0167876022001787 |
  | Accessed | 11/07/2025, 10:04:55 |
  | Volume | 180 |
  | Publisher | Elsevier |
  | Pages | 27–48 |
  | Publication | International Journal of Psychophysiology |
  | Date Added | 11/07/2025, 10:05:27 |
  | Modified | 11/07/2025, 10:05:27 |
- ## Nature-based interventions for psychological wellbeing in long-term conditions: a systematic review

  |  |  |
  | --- | --- |
  | Item Type | Journal Article |
  | Author | Eleanor M. Taylor |
  | Author | Noelle Robertson |
  | Author | Courtney J. Lightfoot |
  | Author | Alice C. Smith |
  | Author | Ceri R. Jones |
  | Date | 2022 |
  | Short Title | Nature-based interventions for psychological wellbeing in long-term conditions |
  | Library Catalogue | Google Scholar |
  | URL | https://www.mdpi.com/1660-4601/19/6/3214 |
  | Accessed | 11/07/2025, 09:31:49 |
  | Volume | 19 |
  | Publisher | MDPI |
  | Pages | 3214 |
  | Publication | International journal of environmental research and public health |
  | Issue | 6 |
  | Date Added | 11/07/2025, 09:32:09 |
  | Modified | 11/07/2025, 09:32:09 |
- ## Being a dog: A review of the domestication process

  |  |  |
  | --- | --- |
  | Item Type | Journal Article |
  | Author | Domenico Tancredi |
  | Author | Irene Cardinali |
  | Date | 2023 |
  | Short Title | Being a dog |
  | Library Catalogue | Google Scholar |
  | URL | https://www.mdpi.com/2073-4425/14/5/992 |
  | Accessed | 11/07/2025, 11:12:01 |
  | Volume | 14 |
  | Publisher | MDPI |
  | Pages | 992 |
  | Publication | Genes |
  | Issue | 5 |
  | Date Added | 11/07/2025, 11:12:01 |
  | Modified | 11/07/2025, 11:12:01 |

  ### Attachments

  - Available Version (via Google Scholar)
- ## Therapeutic role of animals: A comprehensive literature review on the prevalent forms and species in animal-assisted interventions

  |  |  |
  | --- | --- |
  | Item Type | Journal Article |
  | Author | Dorota Szewczyk |
  | Author | Jakub Fiega |
  | Author | Milena Michalska |
  | Author | Urszula Żurek |
  | Author | Zuzanna Lubaszka |
  | Author | Ewa Sikorska |
  | Date | 2023 |
  | Short Title | Therapeutic role of animals |
  | Library Catalogue | Google Scholar |
  | URL | https://apcz.umk.pl/JEHS/article/view/45312 |
  | Accessed | 11/07/2025, 10:16:17 |
  | Volume | 45 |
  | Pages | 215–235 |
  | Publication | Journal of Education, Health and Sport |
  | Issue | 1 |
  | Date Added | 11/07/2025, 10:16:47 |
  | Modified | 11/07/2025, 10:16:47 |

  ### Attachments

  - Available Version (via Google Scholar)
- ## The role of physical activity in animal-assisted interventions for autism: A systematic review

  |  |  |
  | --- | --- |
  | Item Type | Journal Article |
  | Author | Ann-Marie Sylvia |
  | Author | Sofiya Alhassan |
  | Author | Katie Potter |
  | Abstract | Purpose: Animal-assisted interventions (AAIs) may have therapeutic effects for autism. Physical activity (PA) has many established benefits for autism. AAIs appear to involve PA, which may drive some of their beneficial effects. The purpose of this review was to quantify the extent to which AAIs involve PA and to describe the PA dose. Methods: A systematic search was conducted for relevant articles published between January 2015 and May 2022. A total of 1,949 articles were identified with 38 articles eligible for inclusion. PA involvement and dose were determined by matching the intervention description to PA values in the Compendium of Physical Activities. Results: PA was involved in 31 of the studies. Animal species included horses ( n = 17), dogs ( n = 17), dolphins ( n = 3), and cats ( n = 1). All horse, 70% of dog and 67% of dolphin studies included PA. PA dose varied; however, horse and dolphin studies involved moderate-to-vigorous intensity PA and dog studies involved light-intensity PA. PA was assessed in only one study. Conclusion: AAIs for autism involve PA but are not designed as PA interventions nor is PA measured. AAIs could be designed as animal-assisted PA interventions to capitalize on the benefits of PA for both overall health and characteristics of autism. |
  | Date | 01/2024 |
  | Language | en |
  | Short Title | The role of physical activity in animal-assisted interventions for autism |
  | Library Catalogue | Crossref |
  | URL | https://journals.sagepub.com/doi/10.1177/27546330241249880 |
  | Accessed | 11/07/2025, 13:46:18 |
  | Rights | https://creativecommons.org/licenses/by-nc/4.0/ |
  | Volume | 2 |
  | Publisher | SAGE Publications |
  | Publication | Neurodiversity |
  | DOI | 10.1177/27546330241249880 |
  | ISSN | 2754-6330, 2754-6330 |
  | Date Added | 11/07/2025, 13:46:33 |
  | Modified | 11/07/2025, 13:46:33 |

  ### Attachments

  - Available Version (via Google Scholar)
- ## The Use of Neuroplasticity in the Treatment of Altered Sensory Processing in ASD: A Narrative Review

  |  |  |
  | --- | --- |
  | Item Type | Journal Article |
  | Author | Maria Suprunowicz |
  | Author | Julia Bogucka |
  | Author | Natalia Szczerbińska |
  | Author | Beata Konarzewska |
  | Author | Napoleon Waszkiewicz |
  | Date | 2025 |
  | Short Title | The Use of Neuroplasticity in the Treatment of Altered Sensory Processing in ASD |
  | Library Catalogue | Google Scholar |
  | URL | https://www.preprints.org/manuscript/202503.1218 |
  | Accessed | 11/07/2025, 09:57:56 |
  | Publisher | Preprints |
  | Date Added | 11/07/2025, 09:58:25 |
  | Modified | 11/07/2025, 09:58:25 |

  ### Attachments

  - Available Version (via Google Scholar)
- ## Saúde mental e relação humano-animal: uma revisão integrativa de ensaios clínicos randomizados

  |  |  |
  | --- | --- |
  | Item Type | Journal Article |
  | Author | Alberto Sumiya |
  | Author | Joordana Fiorese de Faria |
  | Author | Léa Beatriz Vogel Oravec |
  | Author | Miriam Izabel Dobler |
  | Author | Claudia Mayumi Uekubo |
  | Author | Cíntia Faquin |
  | Author | Ana Paula Dondoerfer Teixeira |
  | Author | Marcy Lancia Pereira |
  | Date | 2024 |
  | Short Title | Saúde mental e relação humano-animal |
  | Library Catalogue | Google Scholar |
  | URL | https://ojs.studiespublicacoes.com.br/ojs/index.php/cadped/article/view/3900 |
  | Accessed | 11/07/2025, 13:45:57 |
  | Volume | 21 |
  | Pages | e3900–e3900 |
  | Publication | Caderno Pedagógico |
  | Issue | 4 |
  | Date Added | 11/07/2025, 13:46:33 |
  | Modified | 11/07/2025, 13:46:33 |

  ### Attachments

  - Available Version (via Google Scholar)
- ## Relação humano-animal e saúde mental na pandemia de Covid-19: uma revisão integrativa.

  |  |  |
  | --- | --- |
  | Item Type | Journal Article |
  | Author | Alberto Sumiya |
  | Date | 2024 |
  | Short Title | Relação humano-animal e saúde mental na pandemia de Covid-19 |
  | Library Catalogue | Google Scholar |
  | URL | https://search.ebscohost.com/login.aspx?direct=true&profile=ehost&scope=site&authtype=crawler&jrnl=16797361&AN=179402586&h=YV4c8xvS0tPpyb5sDBSUXmyK%2FEEtG7gamskwVds3JmeY0oJ4bas32ybUQnYrBxX%2BnlKsSeYRTy7nlx%2FzTAs8Rw%3D%3D&crl=c |
  | Accessed | 11/07/2025, 13:46:31 |
  | Volume | 46 |
  | Publication | Acta Scientiarum: Human & Social Sciences |
  | Issue | 2 |
  | Date Added | 11/07/2025, 13:46:33 |
  | Modified | 11/07/2025, 13:46:33 |
- ## The effects of Canine-Assisted Interventions (CAIs) on the health and social care of older people residing in long term care: A systematic review

  |  |  |
  | --- | --- |
  | Item Type | Journal Article |
  | Author | Cindy Stern |
  | Author | Rie Konno |
  | Date | 2011 |
  | Short Title | The effects of Canine-Assisted Interventions (CAIs) on the health and social care of older people residing in long term care |
  | Library Catalogue | Google Scholar |
  | URL | https://journals.lww.com/jbisrir/fulltext/2011/09060/The\_effects\_of\_Canine\_Assisted\_Interventions.1.aspx |
  | Accessed | 11/07/2025, 09:37:39 |
  | Volume | 9 |
  | Publisher | LWW |
  | Pages | 146–206 |
  | Publication | JBI Evidence Synthesis |
  | Issue | 6 |
  | Date Added | 11/07/2025, 09:38:03 |
  | Modified | 11/07/2025, 09:38:03 |
- ## The effects of Canine-Assisted Interventions (CAIs) on the health and social care of older people residing in long term care: A systematic review

  |  |  |
  | --- | --- |
  | Item Type | Journal Article |
  | Author | Cindy Stern |
  | Author | Rie Konno |
  | Date | 2011 |
  | Short Title | The effects of Canine-Assisted Interventions (CAIs) on the health and social care of older people residing in long term care |
  | Library Catalogue | Google Scholar |
  | URL | https://journals.lww.com/jbisrir/fulltext/2011/09060/The\_effects\_of\_Canine\_Assisted\_Interventions.1.aspx |
  | Accessed | 11/07/2025, 11:11:48 |
  | Volume | 9 |
  | Publisher | LWW |
  | Pages | 146–206 |
  | Publication | JBI Evidence Synthesis |
  | Issue | 6 |
  | Date Added | 11/07/2025, 11:12:01 |
  | Modified | 11/07/2025, 11:12:01 |
- ## Conservation education: Are zoo animals effective ambassadors and is there any cost to their welfare?

  |  |  |
  | --- | --- |
  | Item Type | Journal Article |
  | Author | Sarah L. Spooner |
  | Author | Mark J. Farnworth |
  | Author | Samantha J. Ward |
  | Author | Katherine M. Whitehouse-Tedd |
  | Date | 2021 |
  | Short Title | Conservation education |
  | Library Catalogue | Google Scholar |
  | URL | https://www.mdpi.com/2673-5636/2/1/4 |
  | Accessed | 11/07/2025, 09:29:06 |
  | Volume | 2 |
  | Publisher | MDPI |
  | Pages | 41–65 |
  | Publication | Journal of Zoological and Botanical Gardens |
  | Issue | 1 |
  | Date Added | 11/07/2025, 09:29:28 |
  | Modified | 11/07/2025, 09:29:28 |

  ### Attachments

  - Available Version (via Google Scholar)
- ## Effects of Canine-Assisted Intervention on Stress and Depression in Humans

  |  |  |
  | --- | --- |
  | Item Type | Journal Article |
  | Author | Yujin Song |
  | Author | Youngwook Jung |
  | Author | Yeonju Choi |
  | Author | Minjung Yoon |
  | Date | 2025 |
  | Library Catalogue | Google Scholar |
  | URL | http://www.ejast.org/archive/view\_article?pid=jast-2025-e5 |
  | Accessed | 11/07/2025, 09:31:45 |
  | Publisher | Korean Society of Animal Science and Technology |
  | Publication | Journal of Animal Science and Technology |
  | Date Added | 11/07/2025, 09:32:09 |
  | Modified | 11/07/2025, 09:32:09 |

  ### Attachments

  - Available Version (via Google Scholar)
- ## Effects of Canine-Assisted Intervention on Stress and Depression in Humans

  |  |  |
  | --- | --- |
  | Item Type | Journal Article |
  | Author | Yujin Song |
  | Author | Youngwook Jung |
  | Author | Yeonju Choi |
  | Author | Minjung Yoon |
  | Date | 2025 |
  | Library Catalogue | Google Scholar |
  | URL | http://www.ejast.org/archive/view\_article?pid=jast-2025-e5 |
  | Accessed | 11/07/2025, 10:16:25 |
  | Publisher | Korean Society of Animal Science and Technology |
  | Publication | Journal of Animal Science and Technology |
  | Date Added | 11/07/2025, 10:16:47 |
  | Modified | 11/07/2025, 10:16:47 |

  ### Attachments

  - Available Version (via Google Scholar)
- ## Methods of assessing cattle temperament and factors affecting it: a review

  |  |  |
  | --- | --- |
  | Item Type | Journal Article |
  | Author | Jože Smolinger |
  | Author | Dejan Škorjanc |
  | Date | 2021 |
  | Short Title | Methods of assessing cattle temperament and factors affecting it |
  | Library Catalogue | Google Scholar |
  | URL | https://journals.um.si/index.php/agricultura/article/view/1645 |
  | Accessed | 11/07/2025, 11:01:43 |
  | Volume | 18 |
  | Pages | 23–37 |
  | Publication | Agricultura Scientia |
  | Issue | 1-2 |
  | Date Added | 11/07/2025, 11:01:44 |
  | Modified | 11/07/2025, 11:01:44 |

  ### Attachments

  - Available Version (via Google Scholar)
- ## <i>Calm with horses?</i> A systematic review of animal-assisted interventions for improving social functioning in children with autism

  |  |  |
  | --- | --- |
  | Item Type | Journal Article |
  | Author | Jon H Sissons |
  | Author | Elise Blakemore |
  | Author | Hannah Shafi |
  | Author | Naomi Skotny |
  | Author | Donna M Lloyd |
  | Abstract | The aim of this systematic review was to evaluate the effect of animal-assisted interventions on social functioning in children with autism spectrum disorder, based on evidence from randomized control trials. Included studies were articles published in English, with school aged children from 4 to 18 years with autism spectrum disorder. Databases searched were MEDLINE, PsycINFO, EMBASE, Web of Science, CINAHL and Zoological Record. Data extraction from included studies included demographics and sample features, interventions and controls descriptions, outcome measures, study funding and descriptive statistics. Risk of bias was assessed, considering randomization, allocation concealment, blinding, attrition, selective reporting and other sources of bias. Studies were synthesized narratively based on the animal approach taken and the use of waitlist versus active controls. Nine studies were included reporting across eight trials. Studies overall reported improvements in social functioning following equine-assisted services, with preliminary evidence suggesting improvements are sustained in the short and medium term. Insufficient evidence was available to draw conclusions on the efficacy of other animal-assisted interventions. Future research should aim to address the limitations common to included designs. Lay abstract Children with autism typically experience difficulties interacting socially with others when compared to their non-autistic peers. Establishing how effective interventions are for improving social functioning is important to help inform what should be offered to children with autism. This study reviewed how effective interventions that involved interaction with a live animal, known as animal-assisted interventions, are in improving social functioning in children with autism. A systematic search of the evidence on this topic found nine studies, which were explored for the effectiveness of animal-assisted interventions and the quality of methods used. Overall, these studies showed improvements in social functioning following equine-assisted or therapeutic horse-riding interventions, with initial evidence showing improvements are sustained in the short and medium term. However, several issues were identified, which limit the strength of any conclusions that can be drawn from this evidence. For example, in many studies people assessing the children were aware that they received the intervention or were in a control group. There was also not enough evidence available to draw conclusions on the effectiveness of other animal-assisted interventions. Future research should address the limitations that were common in the designs of these studies and investigate the potential benefit of other animal populations, such as dogs and cats. |
  | Date | 08/2022 |
  | Language | en |
  | Short Title | <i>Calm with horses?</i> |
  | Library Catalogue | Crossref |
  | URL | https://journals.sagepub.com/doi/10.1177/13623613221085338 |
  | Accessed | 11/07/2025, 11:01:37 |
  | Rights | https://creativecommons.org/licenses/by/4.0/ |
  | Volume | 26 |
  | Publisher | SAGE Publications |
  | Pages | 1320-1340 |
  | Publication | Autism |
  | DOI | 10.1177/13623613221085338 |
  | Issue | 6 |
  | ISSN | 1362-3613, 1461-7005 |
  | Date Added | 11/07/2025, 11:01:44 |
  | Modified | 11/07/2025, 11:01:44 |

  ### Attachments

  - Available Version (via Google Scholar)
- ## Contribution of precision livestock farming systems to the improvement of welfare status and productivity of dairy animals

  |  |  |
  | --- | --- |
  | Item Type | Journal Article |
  | Author | Panagiotis Simitzis |
  | Author | Christos Tzanidakis |
  | Author | Ouranios Tzamaloukas |
  | Author | Evangelia Sossidou |
  | Date | 2021 |
  | Library Catalogue | Google Scholar |
  | URL | https://www.mdpi.com/2624-862X/3/1/2 |
  | Accessed | 11/07/2025, 10:18:03 |
  | Volume | 3 |
  | Publisher | MDPI |
  | Pages | 12–28 |
  | Publication | Dairy |
  | Issue | 1 |
  | Date Added | 11/07/2025, 10:18:17 |
  | Modified | 11/07/2025, 10:18:17 |
- ## Literature Review on Technological Applications to Monitor and Evaluate Calves’ Health and Welfare

  |  |  |
  | --- | --- |
  | Item Type | Journal Article |
  | Author | Flávio G. Silva |
  | Author | Cristina Conceição |
  | Author | Alfredo MF Pereira |
  | Author | Joaquim L. Cerqueira |
  | Author | Severiano R. Silva |
  | Date | 2023 |
  | Library Catalogue | Google Scholar |
  | URL | https://www.mdpi.com/2076-2615/13/7/1148 |
  | Accessed | 11/07/2025, 09:36:05 |
  | Volume | 13 |
  | Publisher | MDPI |
  | Pages | 1148 |
  | Publication | Animals |
  | Issue | 7 |
  | Date Added | 11/07/2025, 09:36:11 |
  | Modified | 11/07/2025, 09:36:11 |

  ### Attachments

  - Available Version (via Google Scholar)
- ## Dog-assisted interventions for children and adults with mental health or neurodevelopmental conditions: systematic review

  |  |  |
  | --- | --- |
  | Item Type | Journal Article |
  | Author | Emily Shoesmith |
  | Author | Sophie Hall |
  | Author | Amanda Sowden |
  | Author | Heidi Stevens |
  | Author | Jodi Pervin |
  | Author | Jenny Riga |
  | Author | Dean McMillan |
  | Author | Daniel Mills |
  | Author | Chris Clarke |
  | Author | Qi Wu |
  | Date | 2025 |
  | Short Title | Dog-assisted interventions for children and adults with mental health or neurodevelopmental conditions |
  | Library Catalogue | Google Scholar |
  | URL | https://www.cambridge.org/core/journals/the-british-journal-of-psychiatry/article/dogassisted-interventions-for-children-and-adults-with-mental-health-or-neurodevelopmental-conditions-systematic-review/72599C9A5BDBA836E61D043FE8F097E7 |
  | Accessed | 11/07/2025, 09:35:47 |
  | Publisher | Cambridge University Press |
  | Pages | 1–14 |
  | Publication | The British Journal of Psychiatry |
  | Date Added | 11/07/2025, 09:36:11 |
  | Modified | 11/07/2025, 09:36:11 |

  ### Attachments

  - Available Version (via Google Scholar)
- ## Dog-assisted interventions for children and adults with mental health or neurodevelopmental conditions: systematic review

  |  |  |
  | --- | --- |
  | Item Type | Journal Article |
  | Author | Emily Shoesmith |
  | Author | Sophie Hall |
  | Author | Amanda Sowden |
  | Author | Heidi Stevens |
  | Author | Jodi Pervin |
  | Author | Jenny Riga |
  | Author | Dean McMillan |
  | Author | Daniel Mills |
  | Author | Chris Clarke |
  | Author | Qi Wu |
  | Date | 2025 |
  | Short Title | Dog-assisted interventions for children and adults with mental health or neurodevelopmental conditions |
  | Library Catalogue | Google Scholar |
  | URL | https://www.cambridge.org/core/journals/the-british-journal-of-psychiatry/article/dogassisted-interventions-for-children-and-adults-with-mental-health-or-neurodevelopmental-conditions-systematic-review/72599C9A5BDBA836E61D043FE8F097E7 |
  | Accessed | 11/07/2025, 13:44:35 |
  | Publisher | Cambridge University Press |
  | Pages | 1–14 |
  | Publication | The British Journal of Psychiatry |
  | Date Added | 11/07/2025, 13:45:12 |
  | Modified | 11/07/2025, 13:45:12 |

  ### Attachments

  - Available Version (via Google Scholar)
- ## Köpek Destekli Terapi

  |  |  |
  | --- | --- |
  | Item Type | Journal Article |
  | Author | Melisa Ayhan Sevinç |
  | Author | Gülhan Yılmaz Gökmen |
  | Date | 2025 |
  | Library Catalogue | Google Scholar |
  | URL | https://dergipark.org.tr/en/pub/ktokusbd/issue/91460/1586234 |
  | Accessed | 11/07/2025, 13:45:12 |
  | Volume | 6 |
  | Publisher | KTO Karatay Üniversitesi |
  | Pages | 130–141 |
  | Publication | KTO Karatay Üniversitesi Sağlık Bilimleri Dergisi |
  | Issue | 1 |
  | Date Added | 11/07/2025, 13:45:12 |
  | Modified | 11/07/2025, 13:45:12 |

  ### Attachments

  - Available Version (via Google Scholar)
- ## Heat stress and goat welfare: Adaptation and production considerations

  |  |  |
  | --- | --- |
  | Item Type | Journal Article |
  | Author | Veerasamy Sejian |
  | Author | Mullakkalparambil V. Silpa |
  | Author | Mini R. Reshma Nair |
  | Author | Chinnasamy Devaraj |
  | Author | Govindan Krishnan |
  | Author | Madiajagan Bagath |
  | Author | Surinder S. Chauhan |
  | Author | Rajendran U. Suganthi |
  | Author | Vinicius FC Fonseca |
  | Author | Sven König |
  | Date | 2021 |
  | Short Title | Heat stress and goat welfare |
  | Library Catalogue | Google Scholar |
  | URL | https://www.mdpi.com/2076-2615/11/4/1021 |
  | Accessed | 11/07/2025, 10:16:35 |
  | Volume | 11 |
  | Publisher | MDPI |
  | Pages | 1021 |
  | Publication | Animals |
  | Issue | 4 |
  | Date Added | 11/07/2025, 10:16:47 |
  | Modified | 11/07/2025, 10:16:47 |
- ## Equine-assisted services for people living with dementia: a systematic review

  |  |  |
  | --- | --- |
  | Item Type | Journal Article |
  | Author | Menka Sebalj |
  | Author | Ali Lakhani |
  | Author | Andrea Grindrod |
  | Author | Rwth Stuckey |
  | Abstract | Abstract Background Dementia has a significant impact on the social, physical, and psychological wellbeing of people living with dementia, their families and society. Animal-assisted interventions can have positive effects on the health and wellbeing of people living with dementia. Equine-assisted services are animal-assisted non-pharmacological interventions which have improved the health and wellbeing of diverse populations. The impact of participating in equine-assisted services on the health and wellbeing of people with dementia is unclear. A systematic review was conducted to synthesise evidence investigating the effects of participating in equine-assisted services on the health and wellbeing of people living with dementia. Design Systematic review following the Preferred Reporting Items for Systematic Reviews and Meta-Analyses (PRISMA) guidelines. Methods The databases CINAHL, EMBASE, MEDLINE, and Web of Science were searched for any research published prior to 14 June 2023. Peer-reviewed publications in the English language utilizing methods deriving quantitative and/or qualitative data were eligible. Methodological quality of included studies was assessed using the Mixed Methods Appraisal Tool. Findings from studies were synthesised using a deductive approach. Results Of the 223 articles screened, six met the inclusion criteria: four quantitative and two qualitative studies. The six studies represent four separate equine interventions. Studies were of moderate to strong quality. Participants were people living with dementia (n = 44, mean age range 70–83 years), dementia care partners (n = 5, mean age 58), and equine-assisted services providers (n = 5). Interventions varied in duration, activities conducted, outcomes measured, and measurement tools used. Studies found a favourable impact of participating in equine-assisted services on the neuropsychiatric symptoms and quality of life of people living with dementia. Participating in equine-assisted services improved well-being, functional abilities, social participation, and communication, while also having a positive effect on social, emotional, and behavioural outcomes, and physical health. Conclusions The limited but high-quality literature investigating the impact of equine-assisted services among people living with dementia suggests that equine-assisted services can have a positive impact on the health and wellbeing of people living with dementia. Additional robust studies contributing to the evidence base are warranted; such studies can support the development of programs and further elucidate the impact of participation. |
  | Date | 2024-04-09 |
  | Language | en |
  | Short Title | Equine-assisted services for people living with dementia |
  | Library Catalogue | Crossref |
  | URL | https://alzres.biomedcentral.com/articles/10.1186/s13195-024-01453-4 |
  | Accessed | 11/07/2025, 13:45:03 |
  | Rights | https://creativecommons.org/licenses/by/4.0 |
  | Volume | 16 |
  | Publisher | Springer Science and Business Media LLC |
  | Publication | Alzheimer's Research & Therapy |
  | DOI | 10.1186/s13195-024-01453-4 |
  | Issue | 1 |
  | Journal Abbr | Alz Res Therapy |
  | ISSN | 1758-9193 |
  | Date Added | 11/07/2025, 13:45:12 |
  | Modified | 11/07/2025, 13:45:12 |

  ### Attachments

  - Full Text
- ## Emotional transfer in human–horse interaction: new perspectives on equine assisted interventions

  |  |  |
  | --- | --- |
  | Item Type | Journal Article |
  | Author | Chiara Scopa |
  | Author | Laura Contalbrigo |
  | Author | Alberto Greco |
  | Author | Antonio Lanatà |
  | Author | Enzo Pasquale Scilingo |
  | Author | Paolo Baragli |
  | Date | 2019 |
  | Short Title | Emotional transfer in human–horse interaction |
  | Library Catalogue | Google Scholar |
  | URL | https://www.mdpi.com/2076-2615/9/12/1030 |
  | Accessed | 11/07/2025, 10:07:26 |
  | Volume | 9 |
  | Publisher | MDPI |
  | Pages | 1030 |
  | Publication | Animals |
  | Issue | 12 |
  | Date Added | 11/07/2025, 10:07:52 |
  | Modified | 11/07/2025, 10:07:52 |

  ### Attachments

  - Available Version (via Google Scholar)
- ## Recent advances on wearable electronics and embedded computing systems for biomedical applications

  |  |  |
  | --- | --- |
  | Item Type | Journal Article |
  | Author | Enzo Pasquale Scilingo |
  | Author | Gaetano Valenza |
  | Date | 2017 |
  | Library Catalogue | Google Scholar |
  | URL | https://www.mdpi.com/2079-9292/6/1/12 |
  | Accessed | 11/07/2025, 11:09:36 |
  | Volume | 6 |
  | Publisher | MDPI |
  | Pages | 12 |
  | Publication | Electronics |
  | Issue | 1 |
  | Date Added | 11/07/2025, 11:09:44 |
  | Modified | 11/07/2025, 11:09:44 |

  ### Attachments

  - Available Version (via Google Scholar)
- ## Emerging Cardiovascular Risk Research: Impact of Pets on Cardiovascular Risk Prevention

  |  |  |
  | --- | --- |
  | Item Type | Journal Article |
  | Author | Pamela J. Schreiner |
  | Date | 02/2016 |
  | Language | en |
  | Short Title | Emerging Cardiovascular Risk Research |
  | Library Catalogue | Crossref |
  | URL | http://link.springer.com/10.1007/s12170-016-0489-2 |
  | Accessed | 11/07/2025, 10:07:27 |
  | Rights | http://www.springer.com/tdm |
  | Volume | 10 |
  | Publisher | Springer Science and Business Media LLC |
  | Publication | Current Cardiovascular Risk Reports |
  | DOI | 10.1007/s12170-016-0489-2 |
  | Issue | 2 |
  | Journal Abbr | Curr Cardiovasc Risk Rep |
  | ISSN | 1932-9520, 1932-9563 |
  | Date Added | 11/07/2025, 10:07:52 |
  | Modified | 11/07/2025, 10:07:52 |

  ### Attachments

  - Available Version (via Google Scholar)
- ## Exploring the differences between pet and non-pet owners: Implications for human-animal interaction research and policy

  |  |  |
  | --- | --- |
  | Item Type | Journal Article |
  | Author | Jessica Saunders |
  | Author | Layla Parast |
  | Author | Susan H. Babey |
  | Author | Jeremy V. Miles |
  | Date | 2017 |
  | Short Title | Exploring the differences between pet and non-pet owners |
  | Library Catalogue | Google Scholar |
  | URL | https://journals.plos.org/plosone/article?id=10.1371/journal.pone.0179494 |
  | Accessed | 11/07/2025, 10:05:23 |
  | Volume | 12 |
  | Publisher | Public Library of Science San Francisco, CA USA |
  | Pages | e0179494 |
  | Publication | PLoS one |
  | Issue | 6 |
  | Date Added | 11/07/2025, 10:05:27 |
  | Modified | 11/07/2025, 10:05:27 |

  ### Attachments

  - Available Version (via Google Scholar)
- ## The research of standardized protocols for dog involvement in animal-assisted therapy: A systematic review

  |  |  |
  | --- | --- |
  | Item Type | Journal Article |
  | Author | Antonio Santaniello |
  | Author | Susanne Garzillo |
  | Author | Serena Cristiano |
  | Author | Alessandro Fioretti |
  | Author | Lucia Francesca Menna |
  | Date | 2021 |
  | Short Title | The research of standardized protocols for dog involvement in animal-assisted therapy |
  | Library Catalogue | Google Scholar |
  | URL | https://www.mdpi.com/2076-2615/11/9/2576 |
  | Accessed | 11/07/2025, 10:17:59 |
  | Volume | 11 |
  | Publisher | MDPI |
  | Pages | 2576 |
  | Publication | Animals |
  | Issue | 9 |
  | Date Added | 11/07/2025, 10:18:17 |
  | Modified | 11/07/2025, 10:18:17 |
- ## The importance of good stockmanship and its benefits to animals.

  |  |  |
  | --- | --- |
  | Item Type | Journal Article |
  | Author | Jeffrey Rushen |
  | Author | ANNE MARIE de Passillé |
  | Date | 2020 |
  | Library Catalogue | Google Scholar |
  | URL | https://books.google.com/books?hl=it&lr=&id=wXcREAAAQBAJ&oi=fnd&pg=PA145&dq=%22human+animal+interaction%22+AND+%22heart+rate%22+&ots=TDNrD6mZ8O&sig=FOfdF5c1Z8kysQHgFADASeh4hyM |
  | Accessed | 11/07/2025, 11:09:44 |
  | Publisher | CABI |
  | Pages | 145–159 |
  | Publication | Improving Animal Welfare: A Practical Approach |
  | Date Added | 11/07/2025, 11:09:44 |
  | Modified | 11/07/2025, 11:09:44 |
- ## Reflections on Recent Research Into Animal-Assisted Interventions in the Military and Beyond

  |  |  |
  | --- | --- |
  | Item Type | Journal Article |
  | Author | Christina B. Rumayor |
  | Author | Amy M. Thrasher |
  | Date | 12/2017 |
  | Language | en |
  | Library Catalogue | Crossref |
  | URL | http://link.springer.com/10.1007/s11920-017-0861-z |
  | Accessed | 11/07/2025, 10:09:24 |
  | Rights | http://www.springer.com/tdm |
  | Volume | 19 |
  | Publisher | Springer Science and Business Media LLC |
  | Publication | Current Psychiatry Reports |
  | DOI | 10.1007/s11920-017-0861-z |
  | Issue | 12 |
  | Journal Abbr | Curr Psychiatry Rep |
  | ISSN | 1523-3812, 1535-1645 |
  | Date Added | 11/07/2025, 10:09:48 |
  | Modified | 11/07/2025, 10:09:48 |

  ### Attachments

  - Available Version (via Google Scholar)
- ## Land Use Change and Infectious Disease Emergence

  |  |  |
  | --- | --- |
  | Item Type | Journal Article |
  | Author | M. Cristina Rulli |
  | Author | Paolo D’Odorico |
  | Author | Nikolas Galli |
  | Author | Reju S. John |
  | Author | Renata L. Muylaert |
  | Author | Monia Santini |
  | Author | David T. S. Hayman |
  | Abstract | AbstractMajor infectious diseases threatening human health are transmitted to people from animals or by arthropod vectors such as insects. In recent decades, disease outbreaks have become more common, especially in tropical regions, including new and emerging infections that were previously undetected or unknown. Even though there is growing awareness that altering natural habitats can lead to disease outbreaks, the link between land use change and emerging diseases is still often overlooked and poorly understood. Land use change typically destroys natural habitat and alters landscape composition and configuration, thus altering wildlife population dynamics, including those of pathogen hosts, domesticated (often intermediary) hosts, infectious agents, and their vectors. Moreover, land use changes provide opportunities for human exposure to direct contact with wildlife, livestock, and disease‐carrying vectors, thereby increasing pathogen spillover from animals to humans. Here we explore the nexus between human health and land use change, highlighting multiple pathways linking emerging disease outbreaks and deforestation, forest fragmentation, urbanization, agricultural expansion, intensified farming systems, and concentrated livestock production. We connect direct and underlying drivers of land use change to human health outcomes related to infectious disease emergence. Despite growing evidence of land‐use induced spillover, strategies to reduce the risks of emerging diseases are often absent from discussions on sustainable food systems and land management. A “One Health” perspective—integrating human, animal, and environmental health—provides a critical yet often‐overlooked dimension for understanding the health impacts of land use change. |
  | Date | 06/2025 |
  | Language | en |
  | Library Catalogue | Crossref |
  | URL | https://agupubs.onlinelibrary.wiley.com/doi/10.1029/2022RG000785 |
  | Accessed | 11/07/2025, 13:43:22 |
  | Rights | http://creativecommons.org/licenses/by/4.0/ |
  | Volume | 63 |
  | Publisher | American Geophysical Union (AGU) |
  | Publication | Reviews of Geophysics |
  | DOI | 10.1029/2022rg000785 |
  | Issue | 2 |
  | ISSN | 8755-1209, 1944-9208 |
  | Date Added | 11/07/2025, 13:43:25 |
  | Modified | 11/07/2025, 13:43:25 |
- ## A Transdisciplinary Perspective on Dog-Handler-Client Interactions in Animal Assisted Activities for Children, Youth and Young Adults

  |  |  |
  | --- | --- |
  | Item Type | Journal Article |
  | Author | Renata P. S. Roma |
  | Author | Christine Yvette Tardif-Williams |
  | Author | Shannon A. Moore |
  | Author | Sandra L. Bosacki |
  | Abstract | Abstract A growing body of research has linked the inclusion of dogs in Animal-Assisted Activities (AAA) for children and young adults to a diverse range of positive social emotional and cognitive outcomes. However, many studies have focused exclusively on aspects directly related to dog-client interactions. There is a need to gain a better understanding of how dog-handler teams have been described, conceptualized and incorporated into the analysis in previous research. In addition, few studies have investigated the mutual adjustments inherent to dog-handler-client triadic relationships. This paper explores if and how the unique characteristics of dog-handler teams have been conceptualized and measured in previous studies. First, this paper undertakes a scoping review to map what, if any, characteristics of dogs, handlers, and dog-handler teams have been described and incorporated into the assessment of AAAs from 2004 to 2019 including: demographic characteristics, formal training and certification, handlers’ or dogs’ behavioral and physiological responses to AAAs, handlers’ roles during activities, and configuration of AAA teams. This scoping review also highlights key features of AAA teams requiring further investigation. In addition, this paper proposes the incorporation of a transdisciplinary framework to the analysis of AAAs. Such a holistic framework can inform the field of human-animal interactions by prioritizing a relational and contextual focus to the study of AAAs. |
  | Date | 12/2021 |
  | Language | en |
  | Library Catalogue | Crossref |
  | URL | http://www.cabidigitallibrary.org/doi/10.1079/hai.2021.0026 |
  | Accessed | 11/07/2025, 09:27:22 |
  | Publisher | CABI Publishing |
  | Publication | Human-animal interaction bulletin |
  | DOI | 10.1079/hai.2021.0026 |
  | ISSN | 2333-522X |
  | Date Added | 11/07/2025, 09:27:42 |
  | Modified | 11/07/2025, 09:27:42 |

  ### Attachments

  - Available Version (via Google Scholar)
- ## Recommendations for the Housing of Cats in the Home, in Catteries and Animal Shelters, in Laboratories and in Veterinary Surgeries

  |  |  |
  | --- | --- |
  | Item Type | Journal Article |
  | Author | I Rochlitz |
  | Abstract | In recent years, there has been an increase in interest in applied ethology and animal welfare, and an increase in the popularity of the domestic cat. This has stimulated research on the behaviour and welfare of cats kept in different environments. This article presents a review of the recent research and makes recommendations for the housing of domestic cats in the home, in catteries and animal shelters, in laboratories and in veterinary surgeries. |
  | Date | 09/1999 |
  | Language | en |
  | Library Catalogue | Crossref |
  | URL | https://journals.sagepub.com/doi/10.1016/S1098-612X%2899%2990207-3 |
  | Accessed | 11/07/2025, 09:37:47 |
  | Rights | https://journals.sagepub.com/page/policies/text-and-data-mining-license |
  | Volume | 1 |
  | Publisher | SAGE Publications |
  | Pages | 181-191 |
  | Publication | Journal of Feline Medicine and Surgery |
  | DOI | 10.1016/s1098-612x(99)90207-3 |
  | Issue | 3 |
  | ISSN | 1098-612X, 1532-2750 |
  | Date Added | 11/07/2025, 09:38:03 |
  | Modified | 11/07/2025, 09:38:03 |

  ### Attachments

  - Available Version (via Google Scholar)
- ## Characterizing stress during animal interaction: a focus on the human endocrine response during equine-assisted services

  |  |  |
  | --- | --- |
  | Item Type | Journal Article |
  | Author | Brandon R. Rigby |
  | Date | 2023 |
  | Short Title | Characterizing stress during animal interaction |
  | Library Catalogue | Google Scholar |
  | URL | https://www.frontiersin.org/articles/10.3389/fvets.2023.1303354/full |
  | Accessed | 11/07/2025, 10:05:00 |
  | Volume | 10 |
  | Publisher | Frontiers Media SA |
  | Pages | 1303354 |
  | Publication | Frontiers in Veterinary Science |
  | Date Added | 11/07/2025, 10:05:27 |
  | Modified | 11/07/2025, 10:05:27 |

  ### Attachments

  - Available Version (via Google Scholar)
- ## Animal welfare monitoring in pig research

  |  |  |
  | --- | --- |
  | Item Type | Journal Article |
  | Author | Maxine Rice |
  | Author | Paul Hemsworth |
  | Author | Jean-Loup Rault |
  | Author | Jeremy Skuse |
  | Author | Ellen Jongman |
  | Author | Lauren Hemsworth |
  | Author | Christina Ng |
  | Date | 2013 |
  | Library Catalogue | Google Scholar |
  | URL | https://porkcrc.com.au/wp-content/uploads/2014/05/1C-109-Research-Report.pdf |
  | Accessed | 11/07/2025, 09:35:34 |
  | Date Added | 11/07/2025, 09:36:11 |
  | Modified | 11/07/2025, 09:36:11 |

  ### Attachments

  - Available Version (via Google Scholar)
- ## Animal welfare monitoring in pig research

  |  |  |
  | --- | --- |
  | Item Type | Journal Article |
  | Author | Maxine Rice |
  | Author | Paul Hemsworth |
  | Author | Jean-Loup Rault |
  | Author | Jeremy Skuse |
  | Author | Ellen Jongman |
  | Author | Lauren Hemsworth |
  | Author | Christina Ng |
  | Date | 2013 |
  | Library Catalogue | Google Scholar |
  | URL | https://porkcrc.com.au/wp-content/uploads/2014/05/1C-109-Research-Report.pdf |
  | Accessed | 11/07/2025, 11:11:53 |
  | Date Added | 11/07/2025, 11:12:01 |
  | Modified | 11/07/2025, 11:12:01 |

  ### Attachments

  - Available Version (via Google Scholar)
- ## Mechanisms of Social Attachment Between Children and Pet Dogs

  |  |  |
  | --- | --- |
  | Item Type | Journal Article |
  | Author | Olivia T. Reilly |
  | Author | Leah H. Somerville |
  | Author | Erin E. Hecht |
  | Date | 2024 |
  | Library Catalogue | Google Scholar |
  | URL | https://www.mdpi.com/2076-2615/14/20/3036 |
  | Accessed | 11/07/2025, 11:01:41 |
  | Volume | 14 |
  | Publisher | MDPI |
  | Pages | 3036 |
  | Publication | Animals |
  | Issue | 20 |
  | Date Added | 11/07/2025, 11:01:44 |
  | Modified | 11/07/2025, 11:01:44 |
- ## Measuring dog-owner relationships: Crossing boundaries between animal behaviour and human psychology

  |  |  |
  | --- | --- |
  | Item Type | Journal Article |
  | Author | Therese Rehn |
  | Author | Linda J. Keeling |
  | Date | 2016 |
  | Short Title | Measuring dog-owner relationships |
  | Library Catalogue | Google Scholar |
  | URL | https://www.sciencedirect.com/science/article/pii/S0168159116302088 |
  | Accessed | 11/07/2025, 09:27:24 |
  | Volume | 183 |
  | Publisher | Elsevier |
  | Pages | 1–9 |
  | Publication | Applied Animal Behaviour Science |
  | Date Added | 11/07/2025, 09:27:42 |
  | Modified | 11/07/2025, 09:27:42 |
- ## Applied personality assessment in domestic dogs: Limitations and caveats

  |  |  |
  | --- | --- |
  | Item Type | Journal Article |
  | Author | Diana J. Rayment |
  | Author | Bert De Groef |
  | Author | Richard A. Peters |
  | Author | Linda C. Marston |
  | Date | 2015 |
  | Short Title | Applied personality assessment in domestic dogs |
  | Library Catalogue | Google Scholar |
  | URL | https://www.sciencedirect.com/science/article/pii/S0168159114003074 |
  | Accessed | 11/07/2025, 09:29:20 |
  | Volume | 163 |
  | Publisher | Elsevier |
  | Pages | 1–18 |
  | Publication | Applied Animal Behaviour Science |
  | Date Added | 11/07/2025, 09:29:28 |
  | Modified | 11/07/2025, 09:29:28 |
- ## Applied personality assessment in domestic dogs: Limitations and caveats

  |  |  |
  | --- | --- |
  | Item Type | Journal Article |
  | Author | Diana J. Rayment |
  | Author | Bert De Groef |
  | Author | Richard A. Peters |
  | Author | Linda C. Marston |
  | Date | 2015 |
  | Short Title | Applied personality assessment in domestic dogs |
  | Library Catalogue | Google Scholar |
  | URL | https://www.sciencedirect.com/science/article/pii/S0168159114003074 |
  | Accessed | 11/07/2025, 10:16:19 |
  | Volume | 163 |
  | Publisher | Elsevier |
  | Pages | 1–18 |
  | Publication | Applied Animal Behaviour Science |
  | Date Added | 11/07/2025, 10:16:47 |
  | Modified | 11/07/2025, 10:16:47 |
- ## The power of a positive human–animal relationship for animal welfare

  |  |  |
  | --- | --- |
  | Item Type | Journal Article |
  | Author | Jean-Loup Rault |
  | Author | Susanne Waiblinger |
  | Author | Xavier Boivin |
  | Author | Paul Hemsworth |
  | Date | 2020 |
  | Library Catalogue | Google Scholar |
  | URL | https://www.frontiersin.org/articles/10.3389/fvets.2020.590867/full |
  | Accessed | 11/07/2025, 10:00:28 |
  | Volume | 7 |
  | Publisher | Frontiers Media SA |
  | Pages | 590867 |
  | Publication | Frontiers in veterinary science |
  | Date Added | 11/07/2025, 10:00:41 |
  | Modified | 11/07/2025, 10:00:41 |

  ### Attachments

  - Available Version (via Google Scholar)
- ## The power of a positive human–animal relationship for animal welfare

  |  |  |
  | --- | --- |
  | Item Type | Journal Article |
  | Author | Jean-Loup Rault |
  | Author | Susanne Waiblinger |
  | Author | Xavier Boivin |
  | Author | Paul Hemsworth |
  | Date | 2020 |
  | Library Catalogue | Google Scholar |
  | URL | https://www.frontiersin.org/articles/10.3389/fvets.2020.590867/full |
  | Accessed | 11/07/2025, 10:05:07 |
  | Volume | 7 |
  | Publisher | Frontiers Media SA |
  | Pages | 590867 |
  | Publication | Frontiers in veterinary science |
  | Date Added | 11/07/2025, 10:05:27 |
  | Modified | 11/07/2025, 10:05:27 |

  ### Attachments

  - Available Version (via Google Scholar)
- ## Oxytocin as an indicator of psychological and social well-being in domesticated animals: a critical review

  |  |  |
  | --- | --- |
  | Item Type | Journal Article |
  | Author | Jean-Loup Rault |
  | Author | Marleen van den Munkhof |
  | Author | Femke TA Buisman-Pijlman |
  | Date | 2017 |
  | Short Title | Oxytocin as an indicator of psychological and social well-being in domesticated animals |
  | Library Catalogue | Google Scholar |
  | URL | https://www.frontiersin.org/articles/10.3389/fpsyg.2017.01521/full |
  | Accessed | 11/07/2025, 10:19:57 |
  | Volume | 8 |
  | Publisher | Frontiers Media SA |
  | Pages | 1521 |
  | Publication | Frontiers in psychology |
  | Date Added | 11/07/2025, 10:20:29 |
  | Modified | 11/07/2025, 10:20:29 |

  ### Attachments

  - Available Version (via Google Scholar)
- ## How to reduce synthetic oxytocin administration and stimulate the production of endogenous oxytocin in childbirth

  |  |  |
  | --- | --- |
  | Item Type | Journal Article |
  | Author | Antonio Ragusa |
  | Date | 2015 |
  | Library Catalogue | Google Scholar |
  | URL | https://jpnim.com/index.php/jpnim/article/view/320 |
  | Accessed | 11/07/2025, 11:15:38 |
  | Volume | 4 |
  | Pages | e040228–e040228 |
  | Publication | Journal of Pediatric and Neonatal Individualized Medicine (JPNIM) |
  | Issue | 2 |
  | Date Added | 11/07/2025, 11:15:52 |
  | Modified | 11/07/2025, 11:15:52 |

  ### Attachments

  - Available Version (via Google Scholar)
- ## Adoption and relinquishment interventions at the animal shelter: A review

  |  |  |
  | --- | --- |
  | Item Type | Journal Article |
  | Author | Alexandra Protopopova |
  | Author | Lisa M. Gunter |
  | Date | 2017 |
  | Short Title | Adoption and relinquishment interventions at the animal shelter |
  | Library Catalogue | Google Scholar |
  | URL | https://www.cambridge.org/core/journals/animal-welfare/article/adoption-and-relinquishment-interventions-at-the-animal-shelter-a-review/7603511EB262628BF49AE8D624367A5E |
  | Accessed | 11/07/2025, 11:09:06 |
  | Volume | 26 |
  | Publisher | Cambridge University Press |
  | Pages | 35–48 |
  | Publication | Animal Welfare |
  | Issue | 1 |
  | Date Added | 11/07/2025, 11:09:44 |
  | Modified | 11/07/2025, 11:09:44 |

  ### Attachments

  - Available Version (via Google Scholar)
  - Available Version (via Google Scholar)
- ## Effects of sheltering on physiology, immune function, behavior, and the welfare of dogs

  |  |  |
  | --- | --- |
  | Item Type | Journal Article |
  | Author | Alexandra Protopopova |
  | Date | 2016 |
  | Library Catalogue | Google Scholar |
  | URL | https://www.sciencedirect.com/science/article/pii/S0031938416301068 |
  | Accessed | 11/07/2025, 09:29:18 |
  | Volume | 159 |
  | Publisher | Elsevier |
  | Pages | 95–103 |
  | Publication | Physiology & behavior |
  | Date Added | 11/07/2025, 09:29:28 |
  | Modified | 11/07/2025, 09:29:28 |
- ## Effects of sheltering on physiology, immune function, behavior, and the welfare of dogs

  |  |  |
  | --- | --- |
  | Item Type | Journal Article |
  | Author | Alexandra Protopopova |
  | Date | 2016 |
  | Library Catalogue | Google Scholar |
  | URL | https://www.sciencedirect.com/science/article/pii/S0031938416301068 |
  | Accessed | 11/07/2025, 11:09:32 |
  | Volume | 159 |
  | Publisher | Elsevier |
  | Pages | 95–103 |
  | Publication | Physiology & behavior |
  | Date Added | 11/07/2025, 11:09:44 |
  | Modified | 11/07/2025, 11:09:44 |
- ## The complexity of the human–animal bond: Empathy, attachment and anthropomorphism in human–animal relationships and animal hoarding

  |  |  |
  | --- | --- |
  | Item Type | Journal Article |
  | Author | Emanuela Prato-Previde |
  | Author | Elisa Basso Ricci |
  | Author | Elisa Silvia Colombo |
  | Date | 2022 |
  | Short Title | The complexity of the human–animal bond |
  | Library Catalogue | Google Scholar |
  | URL | https://www.mdpi.com/2076-2615/12/20/2835 |
  | Accessed | 11/07/2025, 10:13:49 |
  | Volume | 12 |
  | Publisher | MDPI |
  | Pages | 2835 |
  | Publication | Animals |
  | Issue | 20 |
  | Date Added | 11/07/2025, 10:13:55 |
  | Modified | 11/07/2025, 10:13:55 |
- ## The physiological function of oxytocin in humans and its acute response to human-dog interactions: A review of the literature

  |  |  |
  | --- | --- |
  | Item Type | Journal Article |
  | Author | Lauren Powell |
  | Author | Adam J. Guastella |
  | Author | Paul McGreevy |
  | Author | Adrian Bauman |
  | Author | Kate M. Edwards |
  | Author | Emmanuel Stamatakis |
  | Date | 2019 |
  | Short Title | The physiological function of oxytocin in humans and its acute response to human-dog interactions |
  | Library Catalogue | Google Scholar |
  | URL | https://www.sciencedirect.com/science/article/pii/S1558787818301709 |
  | Accessed | 11/07/2025, 10:07:28 |
  | Volume | 30 |
  | Publisher | Elsevier |
  | Pages | 25–32 |
  | Publication | Journal of Veterinary Behavior |
  | Date Added | 11/07/2025, 10:07:52 |
  | Modified | 11/07/2025, 10:07:52 |
- ## Physiological Effects of Human-Animal Positive Interaction in Dogs-Review of the Literature.

  |  |  |
  | --- | --- |
  | Item Type | Journal Article |
  | Author | Denisa POP |
  | Author | Vlad POP-VANCIA |
  | Author | Radu CONSTANTINESCU |
  | Author | Vioara MIRESAN |
  | Author | Alina S. RUSU |
  | Author | Ionel PAPUC |
  | Date | 2014 |
  | Library Catalogue | Google Scholar |
  | URL | https://ftp.cramagiurgea.ro/index.php/zootehnie/article/view/10398 |
  | Accessed | 11/07/2025, 10:04:51 |
  | Volume | 71 |
  | Publication | Bulletin of the University of Agricultural Sciences & Veterinary Medicine Cluj-Napoca. Animal Science & Biotechnologies |
  | Issue | 2 |
  | Date Added | 11/07/2025, 10:05:27 |
  | Modified | 11/07/2025, 10:05:27 |

  ### Attachments

  - Available Version (via Google Scholar)
- ## Behavioural differences and similarities between dog breeds: proposing an ecologically valid approach for canine behavioural research

  |  |  |
  | --- | --- |
  | Item Type | Journal Article |
  | Author | Péter Pongrácz |
  | Author | Petra Dobos |
  | Abstract | ABSTRACTThe behaviour of dogs holds great relevance for not only scientists from fundamental and applied research areas, but also due to the widespread roles of dogs in our societies as companions and working animals; their behaviour is also an important factor in animal and human welfare. A large proportion of dogs currently under human supervision belong to one of roughly 400 recognised breeds. Dog breeds can be characterised by distinctive, predictable and reproducible features, including some of their behavioural traits. To the scientist, the comparative analysis of the behaviour of dog breeds provides an opportunity for investigating an array of intriguing phenomena within an easily accessible model organism created from natural and human‐driven evolutionary processes. There are many ways to design and conduct breed‐related behavioural investigations, but such endeavours should always be based around biologically relevant research questions and lead to ecologically valid conclusions. In this review, we surveyed recent research efforts that included dog behaviour‐related comparisons and applied a critical evaluation according to their methods of breed choice and the subsequent research design. Our aim was to assess whether these two fundamentally important components of experimental design provide a solid basis to reach valid conclusions. Based on 97 publications that fulfilled our selection criteria, we identified three primary methods used by researchers to select breeds for their investigations: (i) convenience sampling; (ii) hypothesis‐driven, ancestry‐based sampling; and (iii) hypothesis‐driven, functional sampling. By using the SWOT (Strengths, Weaknesses, Opportunities, Threats) evaluation system, we highlight each of these techniques' merits and shortcomings. We identify when particular methods may be inherently unable to produce biologically meaningful results due to a mismatch between breed choice and the initial research goals. We hope that our evaluation will help researchers adopt best practices in experimental design regarding future dog breed comparisons. |
  | Date | 02/2025 |
  | Language | en |
  | Short Title | Behavioural differences and similarities between dog breeds |
  | Library Catalogue | Crossref |
  | URL | https://onlinelibrary.wiley.com/doi/10.1111/brv.13128 |
  | Accessed | 11/07/2025, 13:45:07 |
  | Rights | http://creativecommons.org/licenses/by-nc-nd/4.0/ |
  | Volume | 100 |
  | Publisher | Wiley |
  | Pages | 68-84 |
  | Publication | Biological Reviews |
  | DOI | 10.1111/brv.13128 |
  | Issue | 1 |
  | ISSN | 1464-7931, 1469-185X |
  | Date Added | 11/07/2025, 13:45:12 |
  | Modified | 11/07/2025, 13:45:12 |

  ### Attachments

  - Available Version (via Google Scholar)
- ## Doggone Good? Potential Benefits of Assistance Animals for Students on College Campuses.

  |  |  |
  | --- | --- |
  | Item Type | Journal Article |
  | Author | Amanda K. Polking |
  | Author | Jeffrey HD Cornelius-White |
  | Author | Tracy L. Stout |
  | Date | 2017 |
  | Short Title | Doggone Good? |
  | Library Catalogue | Google Scholar |
  | URL | https://eric.ed.gov/?id=EJ1163999 |
  | Accessed | 11/07/2025, 11:09:28 |
  | Volume | 30 |
  | Publisher | ERIC |
  | Pages | 237–250 |
  | Publication | Journal of Postsecondary Education and Disability |
  | Issue | 3 |
  | Date Added | 11/07/2025, 11:09:44 |
  | Modified | 11/07/2025, 11:09:44 |

  ### Attachments

  - Available Version (via Google Scholar)
- ## What we (don’t) know about parrot welfare: Finding welfare indicators through a systematic literature review

  |  |  |
  | --- | --- |
  | Item Type | Journal Article |
  | Author | Andrea Piseddu |
  | Author | Yvonne RA van Zeeland |
  | Author | Jean-Loup Rault |
  | Date | 2024 |
  | Short Title | What we (don’t) know about parrot welfare |
  | Library Catalogue | Google Scholar |
  | URL | https://www.cambridge.org/core/journals/animal-welfare/article/what-we-dont-know-about-parrot-welfare-finding-welfare-indicators-through-a-systematic-literature-review/6779997704C0A850AA20F78C50106179 |
  | Accessed | 11/07/2025, 09:31:57 |
  | Volume | 33 |
  | Publisher | Cambridge University Press |
  | Pages | e57 |
  | Publication | Animal Welfare |
  | Date Added | 11/07/2025, 09:32:09 |
  | Modified | 11/07/2025, 09:32:09 |

  ### Attachments

  - Available Version (via Google Scholar)
- ## What We (Don’t) Know about Parrot Welfare: A Systematic Literature Review

  |  |  |
  | --- | --- |
  | Item Type | Journal Article |
  | Author | Andrea Piseddu |
  | Author | Yvonne RA van Zeeland |
  | Author | Jean-Loup Rault |
  | Date | 2024 |
  | Short Title | What We (Don’t) Know about Parrot Welfare |
  | Library Catalogue | Google Scholar |
  | URL | https://www.biorxiv.org/content/10.1101/2024.03.27.586789.abstract |
  | Accessed | 11/07/2025, 13:43:25 |
  | Publisher | Cold Spring Harbor Laboratory |
  | Pages | 2024–03 |
  | Publication | bioRxiv |
  | Date Added | 11/07/2025, 13:43:25 |
  | Modified | 11/07/2025, 13:43:25 |

  ### Attachments

  - Available Version (via Google Scholar)
- ## Alternative practices in organic dairy production and effects on animal behavior, health, and welfare

  |  |  |
  | --- | --- |
  | Item Type | Journal Article |
  | Author | Hannah N. Phillips |
  | Author | Bradley J. Heins |
  | Date | 2022 |
  | Library Catalogue | Google Scholar |
  | URL | https://www.mdpi.com/2076-2615/12/14/1785 |
  | Accessed | 11/07/2025, 09:37:41 |
  | Volume | 12 |
  | Publisher | MDPI |
  | Pages | 1785 |
  | Publication | Animals |
  | Issue | 14 |
  | Date Added | 11/07/2025, 09:38:03 |
  | Modified | 11/07/2025, 09:38:03 |
- ## Companion animal foster caregiving: a scoping review exploring animal and caregiver welfare, barriers to caregiver recruitment and retention, and best practices for foster care programs in animal shelters

  |  |  |
  | --- | --- |
  | Item Type | Journal Article |
  | Author | Grace E. Phillips |
  | Author | Lisa M. Gunter |
  | Date | 2024 |
  | Short Title | Companion animal foster caregiving |
  | Library Catalogue | Google Scholar |
  | URL | https://peerj.com/articles/18623/ |
  | Accessed | 11/07/2025, 10:12:20 |
  | Volume | 12 |
  | Publisher | PeerJ Inc. |
  | Pages | e18623 |
  | Publication | PeerJ |
  | Date Added | 11/07/2025, 10:12:39 |
  | Modified | 11/07/2025, 10:12:39 |
- ## Interactions of Oxytocin and Dopamine—Effects on Behavior in Health and Disease

  |  |  |
  | --- | --- |
  | Item Type | Journal Article |
  | Author | Maria Petersson |
  | Author | Kerstin Uvnäs-Moberg |
  | Date | 2024 |
  | Library Catalogue | Google Scholar |
  | URL | https://www.mdpi.com/2227-9059/12/11/2440 |
  | Accessed | 11/07/2025, 13:44:58 |
  | Volume | 12 |
  | Publisher | MDPI |
  | Pages | 2440 |
  | Publication | Biomedicines |
  | Issue | 11 |
  | Date Added | 11/07/2025, 13:45:12 |
  | Modified | 11/07/2025, 13:45:12 |
- ## Application of intervention strategies for behavior management in autism spectrum disorder in childhood and adolescence. a systematic review.

  |  |  |
  | --- | --- |
  | Item Type | Journal Article |
  | Author | Paola Teresa Penagos Gómez |
  | Author | Cyndi Meneses Castaño |
  | Author | Lina Johanna Álvarez Toro |
  | Date | 2023 |
  | Library Catalogue | Google Scholar |
  | URL | https://search.ebscohost.com/login.aspx?direct=true&profile=ehost&scope=site&authtype=crawler&jrnl=15791726&AN=161426794&h=7XhEyEDoaeC%2Bj4%2FvQvPV1c0Wgaonoy87dk7fArtb%2FpSXi5aVGdhwlTApFYGSGy%2BqxUOQMOQH5alZdrfIqClNag%3D%3D&crl=c |
  | Accessed | 11/07/2025, 11:14:34 |
  | Volume | 47 |
  | Publication | Retos: Nuevas Perspectivas de Educación Física, Deporte y Recreación |
  | Date Added | 11/07/2025, 11:14:45 |
  | Modified | 11/07/2025, 11:14:45 |
- ## Application of intervention strategies for behavior management in autism spectrum disorder in child-hood and adolescence: a systematic review

  |  |  |
  | --- | --- |
  | Item Type | Journal Article |
  | Author | Paola Teresa Penagos |
  | Author | Cyndi Meneses Castaño |
  | Author | Lina Johanna Álvarez Toro |
  | Date | 2023 |
  | Short Title | Application of intervention strategies for behavior management in autism spectrum disorder in child-hood and adolescence |
  | Library Catalogue | Google Scholar |
  | URL | https://dialnet.unirioja.es/servlet/articulo?codigo=8612096 |
  | Accessed | 11/07/2025, 11:14:22 |
  | Publisher | Federación Española de Asociaciones de Docentes de Educación Física (FEADEF) |
  | Pages | 201–214 |
  | Publication | Retos: nuevas tendencias en Educación Física, deporte y recreación |
  | Issue | 47 |
  | Date Added | 11/07/2025, 11:14:45 |
  | Modified | 11/07/2025, 11:14:45 |
- ## Aplicación de estrategias en intervención para el manejo de la conducta en el trastorno del espectro autista en infancia y adolescencia. una revisión sistemática

  |  |  |
  | --- | --- |
  | Item Type | Journal Article |
  | Author | Paola Teresa Penagos |
  | Author | Cyndi Yacira Meneses Castaño |
  | Author | Lina Johana Álvarez Toro |
  | Date | 2023 |
  | Library Catalogue | Google Scholar |
  | URL | https://search.proquest.com/openview/177164a002b462b735ff12f794f45f27/1?pq-origsite=gscholar&cbl=5517081 |
  | Accessed | 11/07/2025, 11:15:44 |
  | Volume | 47 |
  | Publisher | Federación Española de Docentes de Educación Física (FEADEF) |
  | Pages | 201 |
  | Publication | Retos |
  | Date Added | 11/07/2025, 11:15:52 |
  | Modified | 11/07/2025, 11:15:52 |
- ## A scoping review of flow research

  |  |  |
  | --- | --- |
  | Item Type | Journal Article |
  | Author | Corinna Peifer |
  | Author | Gina Wolters |
  | Author | László Harmat |
  | Author | Jean Heutte |
  | Author | Jasmine Tan |
  | Author | Teresa Freire |
  | Author | Dionísia Tavares |
  | Author | Carla Fonte |
  | Author | Frans Orsted Andersen |
  | Author | Jef van den Hout |
  | Date | 2022 |
  | Library Catalogue | Google Scholar |
  | URL | https://www.frontiersin.org/articles/10.3389/fpsyg.2022.815665/full |
  | Accessed | 11/07/2025, 09:58:13 |
  | Volume | 13 |
  | Publisher | Frontiers Media SA |
  | Pages | 815665 |
  | Publication | Frontiers in Psychology |
  | Date Added | 11/07/2025, 09:58:25 |
  | Modified | 11/07/2025, 09:58:25 |

  ### Attachments

  - Available Version (via Google Scholar)
- ## A scoping review of flow research

  |  |  |
  | --- | --- |
  | Item Type | Journal Article |
  | Author | Corinna Peifer |
  | Author | Gina Wolters |
  | Author | László Harmat |
  | Author | Jean Heutte |
  | Author | Jasmine Tan |
  | Author | Teresa Freire |
  | Author | Dionísia Tavares |
  | Author | Carla Fonte |
  | Author | Frans Orsted Andersen |
  | Author | Jef van den Hout |
  | Date | 2022 |
  | Library Catalogue | Google Scholar |
  | URL | https://www.frontiersin.org/articles/10.3389/fpsyg.2022.815665/full |
  | Accessed | 11/07/2025, 11:14:40 |
  | Volume | 13 |
  | Publisher | Frontiers Media SA |
  | Pages | 815665 |
  | Publication | Frontiers in Psychology |
  | Date Added | 11/07/2025, 11:14:45 |
  | Modified | 11/07/2025, 11:14:45 |
- ## Evidence of horsemanship and dogmanship and their application in veterinary contexts

  |  |  |
  | --- | --- |
  | Item Type | Journal Article |
  | Author | Elyssa Payne |
  | Author | M. Boot |
  | Author | M. Starling |
  | Author | C. Henshall |
  | Author | A. McLean |
  | Author | P. Bennett |
  | Author | P. McGreevy |
  | Date | 2015 |
  | Library Catalogue | Google Scholar |
  | URL | https://www.sciencedirect.com/science/article/pii/S109002331500146X |
  | Accessed | 11/07/2025, 10:13:19 |
  | Volume | 204 |
  | Publisher | Elsevier |
  | Pages | 247–254 |
  | Publication | The Veterinary Journal |
  | Issue | 3 |
  | Date Added | 11/07/2025, 10:13:55 |
  | Modified | 11/07/2025, 10:13:55 |
- ## CAMEL-ASSISTED SERVICES (CAS): TREATMENT, EDUCATION AND SUPPORT PROGRAMMES

  |  |  |
  | --- | --- |
  | Item Type | Journal Article |
  | Author | Carlos Iglesias Pastrana |
  | Author | Francisco Javier Navas González |
  | Author | Juan Vicente Delgado Bermejo |
  | Author | Douglas Baum |
  | Author | Valeri Crenshaw |
  | Author | Malin Larsson |
  | Author | Jenny Brachmann |
  | Author | Christina Adams |
  | Author | Samantha Arevalo |
  | Author | Michael Kaufmann |
  | Date | 2024 |
  | Short Title | CAMEL-ASSISTED SERVICES (CAS) |
  | Library Catalogue | Google Scholar |
  | URL | https://www.camelsandcamelids.com/uploads/journal-manuscript/camel-assisted-services-cas-treatment-education-and-support-programmes.pdf |
  | Accessed | 11/07/2025, 13:46:33 |
  | Pages | 257 |
  | Publication | Journal of Camel Practice and Research |
  | Date Added | 11/07/2025, 13:46:33 |
  | Modified | 11/07/2025, 13:46:33 |

  ### Attachments

  - Available Version (via Google Scholar)
- ## Animal-assisted interventions for the improvement of mental health outcomes in higher education students: A systematic review of randomised controlled trials

  |  |  |
  | --- | --- |
  | Item Type | Journal Article |
  | Author | Charlotte Parbery-Clark |
  | Author | Marvellas Lubamba |
  | Author | Louise Tanner |
  | Author | Elaine McColl |
  | Date | 2021 |
  | Short Title | Animal-assisted interventions for the improvement of mental health outcomes in higher education students |
  | Library Catalogue | Google Scholar |
  | URL | https://www.mdpi.com/1660-4601/18/20/10768 |
  | Accessed | 11/07/2025, 09:32:03 |
  | Volume | 18 |
  | Publisher | MDPI |
  | Pages | 10768 |
  | Publication | International journal of environmental research and public health |
  | Issue | 20 |
  | Date Added | 11/07/2025, 09:32:09 |
  | Modified | 11/07/2025, 09:32:09 |

  ### Attachments

  - Available Version (via Google Scholar)
- ## Group housing of sows in small pens: advantages, disadvantages and recent research

  |  |  |
  | --- | --- |
  | Item Type | Conference Paper |
  | Author | E. A. Pajor |
  | Date | 2002 |
  | Short Title | Group housing of sows in small pens |
  | Library Catalogue | Google Scholar |
  | URL | https://porkgateway.org/wp-content/uploads/2015/07/group-housing-of-sows-in-small-pens-advantages-disadvantages-and-recent-research1.pdf |
  | Accessed | 11/07/2025, 09:37:53 |
  | Pages | 37–44 |
  | Proceedings Title | Proceedings: Symposium on Swine Housing and Well-being. US Departments of Agriculture, Agricultural Research Service, National Agricultural Library, Animal Welfare Information Centre |
  | Date Added | 11/07/2025, 09:38:03 |
  | Modified | 11/07/2025, 09:38:03 |

  ### Attachments

  - Available Version (via Google Scholar)
- ## Group housing of sows in small pens: advantages, disadvantages and recent research

  |  |  |
  | --- | --- |
  | Item Type | Conference Paper |
  | Author | E. A. Pajor |
  | Date | 2002 |
  | Short Title | Group housing of sows in small pens |
  | Library Catalogue | Google Scholar |
  | URL | https://porkgateway.org/wp-content/uploads/2015/07/group-housing-of-sows-in-small-pens-advantages-disadvantages-and-recent-research1.pdf |
  | Accessed | 11/07/2025, 11:14:20 |
  | Pages | 37–44 |
  | Proceedings Title | Proceedings: Symposium on Swine Housing and Well-being. US Departments of Agriculture, Agricultural Research Service, National Agricultural Library, Animal Welfare Information Centre |
  | Date Added | 11/07/2025, 11:14:45 |
  | Modified | 11/07/2025, 11:14:45 |
- ## Mental Health and the Correlation with Professional Athlete’s Performance, a Rapid Literature Review

  |  |  |
  | --- | --- |
  | Item Type | Journal Article |
  | Author | Nathalia Pachón-Blanco |
  | Author | María Clara Peña-Ciro |
  | Author | Juliana Pineda-Ortega |
  | Author | Alejandra Restrepo-Martínez |
  | Author | Francisco Palencia-Sánchez |
  | Date | 2022 |
  | Library Catalogue | Google Scholar |
  | URL | https://www.researchgate.net/profile/Maria-Pena-Ciro/publication/359650501\_Mental\_Health\_and\_the\_Correlation\_with\_Professional\_Athlete's\_Performance\_a\_Rapid\_Literature\_Review-Working\_Paper/links/6247975021077329f2e9d447/Mental-Health-and-the-Correlation-with-Professional-Athletes-Performance-a-Rapid-Literature-Review-Working-Paper.pdf |
  | Accessed | 11/07/2025, 09:37:57 |
  | Publication | Available at SSRN 4037506 |
  | Date Added | 11/07/2025, 09:38:03 |
  | Modified | 11/07/2025, 09:38:03 |
- ## Robotics for poultry farming: Challenges and opportunities

  |  |  |
  | --- | --- |
  | Item Type | Journal Article |
  | Author | Uğur Özentürk |
  | Author | Zhengqi Chen |
  | Author | Lorenzo Jamone |
  | Author | Elisabetta Versace |
  | Date | 2024 |
  | Short Title | Robotics for poultry farming |
  | Library Catalogue | Google Scholar |
  | URL | https://www.sciencedirect.com/science/article/pii/S0168169924008020 |
  | Accessed | 11/07/2025, 09:54:53 |
  | Volume | 226 |
  | Publisher | Elsevier |
  | Pages | 109411 |
  | Publication | Computers and Electronics in Agriculture |
  | Date Added | 11/07/2025, 09:55:10 |
  | Modified | 11/07/2025, 09:55:11 |
- ## Management of livestock behavior to improve welfare and production

  |  |  |
  | --- | --- |
  | Item Type | Journal Article |
  | Author | A. Orihuela |
  | Date | 2021 |
  | Library Catalogue | Google Scholar |
  | URL | https://www.sciencedirect.com/science/article/pii/S1751731121001336 |
  | Accessed | 11/07/2025, 10:13:39 |
  | Volume | 15 |
  | Publisher | Elsevier |
  | Pages | 100290 |
  | Publication | Animal |
  | Date Added | 11/07/2025, 10:13:55 |
  | Modified | 11/07/2025, 10:13:55 |
- ## Neurophysiological Correlates of Affiliative Behaviour between Humans and Dogs

  |  |  |
  | --- | --- |
  | Item Type | Journal Article |
  | Author | J.S.J Odendaal |
  | Author | R.A Meintjes |
  | Abstract | Few physiological parameters for positive human–companion animal contact have been identiﬁed and those that are established have all been in humans. The implication is that if the physiological reactions are mutual, dogs would experience the same psychological beneﬁts from these neurophysiological changes as humans. Therefore, we have determined the role of certain neurochemicals during afﬁliation behaviour on an interspecies basis. Our results indicate that concentrations of b-endorphin, oxytocin, prolactin, b-phenylethylamine, and dopamine increased in both species after positive interspecies interaction, while that of cortisol decreased in the humans only. Indicators of mutual physiological changes during positive interaction between dog lovers and dogs may contribute to a better understanding of the human–animal bond in veterinary practice. |
  | Date | 5/2003 |
  | Language | en |
  | Library Catalogue | DOI.org (Crossref) |
  | URL | https://linkinghub.elsevier.com/retrieve/pii/S109002330200237X |
  | Accessed | 17/06/2025, 17:10:02 |
  | Rights | https://www.elsevier.com/tdm/userlicense/1.0/ |
  | Volume | 165 |
  | Pages | 296-301 |
  | Publication | The Veterinary Journal |
  | DOI | 10.1016/S1090-0233(02)00237-X |
  | Issue | 3 |
  | Journal Abbr | The Veterinary Journal |
  | ISSN | 10900233 |
  | Date Added | 17/06/2025, 17:10:02 |
  | Modified | 17/06/2025, 17:10:02 |

  ### Attachments

  - PDF
- ## Animal-assisted therapy — magic or medicine?

  |  |  |
  | --- | --- |
  | Item Type | Journal Article |
  | Author | J.S.J Odendaal |
  | Date | 10/2000 |
  | Language | en |
  | Library Catalogue | DOI.org (Crossref) |
  | URL | https://linkinghub.elsevier.com/retrieve/pii/S0022399900001835 |
  | Accessed | 17/06/2025, 17:08:24 |
  | Rights | https://www.elsevier.com/tdm/userlicense/1.0/ |
  | Volume | 49 |
  | Pages | 275-280 |
  | Publication | Journal of Psychosomatic Research |
  | DOI | 10.1016/S0022-3999(00)00183-5 |
  | Issue | 4 |
  | Journal Abbr | Journal of Psychosomatic Research |
  | ISSN | 00223999 |
  | Date Added | 17/06/2025, 17:08:24 |
  | Modified | 17/06/2025, 17:08:24 |

  ### Attachments

  - PDF
- ## Animal‐assisted interventions in adult hospital rehabilitation settings: A scoping review

  |  |  |
  | --- | --- |
  | Item Type | Journal Article |
  | Author | Mary O'Loughlin |
  | Author | Rachael Edwards |
  | Author | Em Bould |
  | Author | Sue Devine |
  | Author | Sandra Downing |
  | Abstract | AbstractAnimal‐assisted interventions (AAIs) have the potential to enhance people's well‐being and function and are increasingly being implemented across a range of settings. This scoping review explored how AAIs have been used in adult hospital rehabilitative care. Using JBI and PRISMA‐ScR guidelines, a systematic search of four databases was undertaken. Inclusion criteria involved adults, aged >18 years, who had received AAIs in the hospital rehabilitation setting. Twenty‐two articles met the inclusion criteria. Results identified two intervention types: visitation activities (n = 8 studies) and structured therapeutic interventions (n = 14 studies). Dogs were the most common animal species. Improvements in social and emotional well‐being were reported across both types of interventions, with improvements in ambulation, motor skills, and verbal communication reported by those engaged in structured therapeutic interventions. Implementation challenges included a dependency on volunteer dog‐handlers; the need for better recording of interventions in medical records to enable evaluation; and cost, safety, infection control, and animal welfare considerations. Strengthening the planning of AAIs is fundamental for the realization of potential outcomes from human–animal interactions in hospital rehabilitative care. |
  | Date | 09/2024 |
  | Language | en |
  | Short Title | Animal‐assisted interventions in adult hospital rehabilitation settings |
  | Library Catalogue | Crossref |
  | URL | https://onlinelibrary.wiley.com/doi/10.1111/nhs.13138 |
  | Accessed | 11/07/2025, 13:45:59 |
  | Rights | http://creativecommons.org/licenses/by-nc-nd/4.0/ |
  | Volume | 26 |
  | Publisher | Wiley |
  | Publication | Nursing & Health Sciences |
  | DOI | 10.1111/nhs.13138 |
  | Issue | 3 |
  | Journal Abbr | Nursing &amp; Health Sciences |
  | ISSN | 1441-0745, 1442-2018 |
  | Date Added | 11/07/2025, 13:46:33 |
  | Modified | 11/07/2025, 13:46:33 |

  ### Attachments

  - Full Text PDF
- ## Animal-assisted intervention for trauma: A systematic literature review

  |  |  |
  | --- | --- |
  | Item Type | Journal Article |
  | Author | Marguerite E. O'haire |
  | Author | Noémie A. Guérin |
  | Author | Alison C. Kirkham |
  | Date | 2015 |
  | Short Title | Animal-assisted intervention for trauma |
  | Library Catalogue | Google Scholar |
  | URL | https://www.frontiersin.org/articles/10.3389/fpsyg.2015.01121/full |
  | Accessed | 11/07/2025, 09:28:58 |
  | Volume | 6 |
  | Publisher | Frontiers Media SA |
  | Pages | 1121 |
  | Publication | Frontiers in psychology |
  | Date Added | 11/07/2025, 09:29:28 |
  | Modified | 11/07/2025, 09:29:28 |

  ### Attachments

  - Available Version (via Google Scholar)
- ## Animal-assisted intervention for trauma: A systematic literature review

  |  |  |
  | --- | --- |
  | Item Type | Journal Article |
  | Author | Marguerite E. O'haire |
  | Author | Noémie A. Guérin |
  | Author | Alison C. Kirkham |
  | Date | 2015 |
  | Short Title | Animal-assisted intervention for trauma |
  | Library Catalogue | Google Scholar |
  | URL | https://www.frontiersin.org/articles/10.3389/fpsyg.2015.01121/full |
  | Accessed | 11/07/2025, 10:12:30 |
  | Volume | 6 |
  | Publisher | Frontiers Media SA |
  | Pages | 1121 |
  | Publication | Frontiers in psychology |
  | Date Added | 11/07/2025, 10:12:39 |
  | Modified | 11/07/2025, 10:12:39 |
- ## Companion animals and human health: Benefits, challenges, and the road ahead

  |  |  |
  | --- | --- |
  | Item Type | Journal Article |
  | Author | Marguerite O'Haire |
  | Date | 2010 |
  | Short Title | Companion animals and human health |
  | Library Catalogue | Google Scholar |
  | URL | https://www.sciencedirect.com/science/article/pii/S155878781000016X |
  | Accessed | 11/07/2025, 10:09:16 |
  | Volume | 5 |
  | Publisher | Elsevier |
  | Pages | 226–234 |
  | Publication | Journal of Veterinary Behavior |
  | Issue | 5 |
  | Date Added | 11/07/2025, 10:09:48 |
  | Modified | 11/07/2025, 10:09:48 |
- ## The benefits of companion animals for human mental and physical health.

  |  |  |
  | --- | --- |
  | Item Type | Journal Article |
  | Author | M. O'Haire |
  | Date | 2009 |
  | Library Catalogue | Google Scholar |
  | URL | https://www.cabidigitallibrary.org/doi/full/10.5555/20123352964 |
  | Accessed | 11/07/2025, 10:12:26 |
  | Date Added | 11/07/2025, 10:12:39 |
  | Modified | 11/07/2025, 10:12:39 |
- ## Animal-Assisted Intervention for Autism Spectrum Disorder: A Systematic Literature Review

  |  |  |
  | --- | --- |
  | Item Type | Journal Article |
  | Author | Marguerite E. O’Haire |
  | Date | 07/2013 |
  | Language | en |
  | Short Title | Animal-Assisted Intervention for Autism Spectrum Disorder |
  | Library Catalogue | Crossref |
  | URL | http://link.springer.com/10.1007/s10803-012-1707-5 |
  | Accessed | 11/07/2025, 10:13:25 |
  | Rights | http://www.springer.com/tdm |
  | Volume | 43 |
  | Publisher | Springer Science and Business Media LLC |
  | Pages | 1606-1622 |
  | Publication | Journal of Autism and Developmental Disorders |
  | DOI | 10.1007/s10803-012-1707-5 |
  | Issue | 7 |
  | Journal Abbr | J Autism Dev Disord |
  | ISSN | 0162-3257, 1573-3432 |
  | Date Added | 11/07/2025, 10:13:55 |
  | Modified | 11/07/2025, 10:13:55 |

  ### Attachments

  - Available Version (via Google Scholar)
- ## Review of current evidence and future directions in animal-assisted intervention for children with autism

  |  |  |
  | --- | --- |
  | Item Type | Journal Article |
  | Author | M. E. O’Haire |
  | Date | 2013 |
  | Library Catalogue | Google Scholar |
  | URL | https://www.researchgate.net/profile/Marguerite-Ohaire/publication/258021625\_Review\_of\_current\_evidence\_and\_future\_directions\_in\_animal-assisted\_intervention\_for\_children\_with\_autism/links/02e7e52744abb75c4e000000/Review-of-current-evidence-and-future-directions-in-animal-assisted-intervention-for-children-with-autism.pdf |
  | Accessed | 11/07/2025, 10:13:33 |
  | Volume | 1 |
  | Publisher | OA Publishing London |
  | Pages | 6–10 |
  | Publication | OA autism |
  | Issue | 1 |
  | Date Added | 11/07/2025, 10:13:55 |
  | Modified | 11/07/2025, 10:13:55 |
- ## Identification of potential welfare indicators for commercially farmed King salmon (Hāmana, Oncorhynchus tshawytscha): a scoping review to inform the development of a national code of welfare: a thesis presented in partial fulfilment of the requirements for the degree of Master of Science in Physiology at Massey University, Manawatu, New Zealand

  |  |  |
  | --- | --- |
  | Item Type | Journal Article |
  | Author | Izabella Maree Norris |
  | Date | 2022 |
  | Short Title | Identification of potential welfare indicators for commercially farmed King salmon (Hāmana, Oncorhynchus tshawytscha) |
  | Library Catalogue | Google Scholar |
  | URL | https://mro.massey.ac.nz/handle/10179/17671 |
  | Accessed | 11/07/2025, 09:57:58 |
  | Publisher | Massey University |
  | Date Added | 11/07/2025, 09:58:25 |
  | Modified | 11/07/2025, 09:58:25 |

  ### Attachments

  - Available Version (via Google Scholar)
- ## Animal-Assisted Interventions for Autism Spectrum Disorder: A Systematic Review of the Literature from 2016 to 2020

  |  |  |
  | --- | --- |
  | Item Type | Journal Article |
  | Author | Leanne O. Nieforth |
  | Author | A. J. Schwichtenberg |
  | Author | Marguerite E. O’Haire |
  | Date | 06/2023 |
  | Language | en |
  | Short Title | Animal-Assisted Interventions for Autism Spectrum Disorder |
  | Library Catalogue | Crossref |
  | URL | https://link.springer.com/10.1007/s40489-021-00291-6 |
  | Accessed | 11/07/2025, 09:29:12 |
  | Rights | https://www.springernature.com/gp/researchers/text-and-data-mining |
  | Volume | 10 |
  | Publisher | Springer Science and Business Media LLC |
  | Pages | 255-280 |
  | Publication | Review Journal of Autism and Developmental Disorders |
  | DOI | 10.1007/s40489-021-00291-6 |
  | Issue | 2 |
  | Journal Abbr | Rev J Autism Dev Disord |
  | ISSN | 2195-7177, 2195-7185 |
  | Date Added | 11/07/2025, 09:29:28 |
  | Modified | 11/07/2025, 09:29:28 |

  ### Attachments

  - Available Version (via Google Scholar)
- ## Cute and cuddly and a whole lot more? A call for empirical investigation into the therapeutic benefits of human–animal interaction for children

  |  |  |
  | --- | --- |
  | Item Type | Journal Article |
  | Author | R. Fawcett Nicholas |
  | Author | Eleonora Gullone |
  | Date | 2001 |
  | Short Title | Cute and cuddly and a whole lot more? |
  | Library Catalogue | Google Scholar |
  | URL | https://www.cambridge.org/core/journals/behaviour-change/article/cute-and-cuddly-and-a-whole-lot-more-a-call-for-empirical-investigation-into-the-therapeutic-benefits-of-humananimal-interaction-for-children/798747FD2527E6D6E13C5B4DD4CEF311 |
  | Accessed | 11/07/2025, 10:07:08 |
  | Volume | 18 |
  | Publisher | Cambridge University Press |
  | Pages | 124–133 |
  | Publication | Behaviour Change |
  | Issue | 2 |
  | Date Added | 11/07/2025, 10:07:52 |
  | Modified | 11/07/2025, 10:07:52 |

  ### Attachments

  - Available Version (via Google Scholar)
- ## Agency in Livestock Farming-A Perspective on Human–Animal–Computer Interactions

  |  |  |
  | --- | --- |
  | Item Type | Journal Article |
  | Author | Suresh Neethirajan |
  | Date | 2025 |
  | Library Catalogue | Google Scholar |
  | URL | https://www.preprints.org/manuscript/202506.0694/download/final\_file |
  | Accessed | 11/07/2025, 10:13:10 |
  | Date Added | 11/07/2025, 10:13:55 |
  | Modified | 11/07/2025, 10:13:55 |

  ### Attachments

  - Available Version (via Google Scholar)
  - Available Version (via Google Scholar)
- ## Invited review: Socio-cognitive capacities of goats and their impact on human–animal interactions

  |  |  |
  | --- | --- |
  | Item Type | Journal Article |
  | Author | Christian Nawroth |
  | Date | 2017 |
  | Short Title | Invited review |
  | Library Catalogue | Google Scholar |
  | URL | https://www.sciencedirect.com/science/article/pii/S0921448817300639 |
  | Accessed | 11/07/2025, 10:09:14 |
  | Volume | 150 |
  | Publisher | Elsevier |
  | Pages | 70–75 |
  | Publication | Small Ruminant Research |
  | Date Added | 11/07/2025, 10:09:48 |
  | Modified | 11/07/2025, 10:09:48 |
- ## Does Oxytocin Increase Trust in Humans? A Critical Review of Research

  |  |  |
  | --- | --- |
  | Item Type | Journal Article |
  | Author | Gideon Nave |
  | Author | Colin Camerer |
  | Author | Michael McCullough |
  | Abstract | Behavioral neuroscientists have shown that the neuropeptide oxytocin (OT) plays a key role in social attachment and affiliation in nonhuman mammals. Inspired by this initial research, many social scientists proceeded to examine the associations of OT with trust in humans over the past decade. To conduct this work, they have (a) examined the effects of exogenous OT increase caused by intranasal administration on trusting behavior, (b) correlated individual difference measures of OT plasma levels with measures of trust, and (c) searched for genetic polymorphisms of the OT receptor gene that might be associated with trust. We discuss the different methods used by OT behavioral researchers and review evidence that links OT to trust in humans. Unfortunately, the simplest promising finding associating intranasal OT with higher trust has not replicated well. Moreover, the plasma OT evidence is flawed by how OT is measured in peripheral bodily fluids. Finally, in recent large-sample studies, researchers failed to find consistent associations of specific OT-related genetic polymorphisms and trust. We conclude that the cumulative evidence does not provide robust convergent evidence that human trust is reliably associated with OT (or caused by it). We end with constructive ideas for improving the robustness and rigor of OT research. |
  | Date | 11/2015 |
  | Language | en |
  | Short Title | Does Oxytocin Increase Trust in Humans? |
  | Library Catalogue | Crossref |
  | URL | https://journals.sagepub.com/doi/10.1177/1745691615600138 |
  | Accessed | 11/07/2025, 09:58:12 |
  | Rights | https://journals.sagepub.com/page/policies/text-and-data-mining-license |
  | Volume | 10 |
  | Publisher | SAGE Publications |
  | Pages | 772-789 |
  | Publication | Perspectives on Psychological Science |
  | DOI | 10.1177/1745691615600138 |
  | Issue | 6 |
  | Journal Abbr | Perspect Psychol Sci |
  | ISSN | 1745-6916, 1745-6924 |
  | Date Added | 11/07/2025, 09:58:25 |
  | Modified | 11/07/2025, 09:58:25 |

  ### Attachments

  - Available Version (via Google Scholar)
- ## Terapias não farmacológicas no tratamento de transtorno do estresse pós-traumático em emergencistas: revisão de escopo

  |  |  |
  | --- | --- |
  | Item Type | Journal Article |
  | Author | Jessica Cristhyane Peixoto Nascimento |
  | Author | Kauanny Vitoria Gurgel dos Santos |
  | Author | Joyce Karolayne dos Santos Dantas |
  | Author | Daniele Vieira Dantas |
  | Author | Rodrigo Assis Neves Dantas |
  | Date | 2021 |
  | Short Title | Terapias não farmacológicas no tratamento de transtorno do estresse pós-traumático em emergencistas |
  | Library Catalogue | Google Scholar |
  | URL | https://www.scielo.br/j/reeusp/a/txtjH75CtHXqJbXk6vrkKLf/?lang=pt |
  | Accessed | 11/07/2025, 11:15:32 |
  | Volume | 55 |
  | Publisher | SciELO Brasil |
  | Pages | e03724 |
  | Publication | Revista da Escola de Enfermagem da USP |
  | Date Added | 11/07/2025, 11:15:52 |
  | Modified | 11/07/2025, 11:15:52 |

  ### Attachments

  - Available Version (via Google Scholar)
- ## The human- animal relationship in dairy animals

  |  |  |
  | --- | --- |
  | Item Type | Journal Article |
  | Author | Fabio Napolitano |
  | Author | Andrea Bragaglio |
  | Author | Emilio Sabia |
  | Author | Francesco Serrapica |
  | Author | Ada Braghieri |
  | Author | Giuseppe De Rosa |
  | Date | 2020 |
  | Library Catalogue | Google Scholar |
  | URL | https://www.cambridge.org/core/journals/journal-of-dairy-research/article/humananimal-relationship-in-dairy-animals/3C65E8C6A66BDFCBB43CEEA41022CAAA |
  | Accessed | 11/07/2025, 10:07:42 |
  | Volume | 87 |
  | Publisher | Cambridge University Press |
  | Pages | 47–52 |
  | Publication | Journal of Dairy Research |
  | Issue | S1 |
  | Date Added | 11/07/2025, 10:07:52 |
  | Modified | 11/07/2025, 10:07:52 |

  ### Attachments

  - Available Version (via Google Scholar)
- ## Oxytocin-gaze positive loop and the coevolution of human-dog bonds

  |  |  |
  | --- | --- |
  | Item Type | Journal Article |
  | Author | Miho Nagasawa |
  | Author | Shouhei Mitsui |
  | Author | Shiori En |
  | Author | Nobuyo Ohtani |
  | Author | Mitsuaki Ohta |
  | Author | Yasuo Sakuma |
  | Author | Tatsushi Onaka |
  | Author | Kazutaka Mogi |
  | Author | Takefumi Kikusui |
  | Date | 2015 |
  | Language | en |
  | Library Catalogue | Zotero |
  | Date Added | 17/06/2025, 17:34:17 |
  | Modified | 15/07/2025, 09:33:15 |

  ### Attachments

  - 1261022-nagasawa-sm
  - PDF
- ## Animal-Assisted Therapy in Dentistry: A Review

  |  |  |
  | --- | --- |
  | Item Type | Journal Article |
  | Author | Begüm Mutlu |
  | Author | Mine Keskin |
  | Author | Yelda Kasımoğlu |
  | Date | 2025 |
  | Short Title | Animal-Assisted Therapy in Dentistry |
  | Library Catalogue | Google Scholar |
  | URL | https://essentdent.org/Content/files/sayilar/2/ED\_20240022\_nlm\_new\_indd.pdf |
  | Accessed | 11/07/2025, 09:31:41 |
  | Volume | 4 |
  | Publication | Essentials of Dentistry |
  | Date Added | 11/07/2025, 09:32:09 |
  | Modified | 11/07/2025, 09:32:09 |

  ### Attachments

  - Available Version (via Google Scholar)
- ## Animal-Assisted Therapy in Dentistry: A Review

  |  |  |
  | --- | --- |
  | Item Type | Journal Article |
  | Author | Begüm Mutlu |
  | Author | Mine Keskin |
  | Author | Yelda Kasımoğlu |
  | Date | 2025 |
  | Short Title | Animal-Assisted Therapy in Dentistry |
  | Library Catalogue | Google Scholar |
  | URL | https://essentdent.org/Content/files/sayilar/2/ED\_20240022\_nlm\_new\_indd.pdf |
  | Accessed | 11/07/2025, 10:16:15 |
  | Volume | 4 |
  | Publication | Essentials of Dentistry |
  | Date Added | 11/07/2025, 10:16:47 |
  | Modified | 11/07/2025, 10:16:47 |

  ### Attachments

  - Available Version (via Google Scholar)
- ## Assessing emotions in pigs: determining negative and positive mental states

  |  |  |
  | --- | --- |
  | Item Type | Journal Article |
  | Author | Eimear Murphy |
  | Author | Luca Melotti |
  | Author | Michael Mendl |
  | Date | 2021 |
  | Short Title | Assessing emotions in pigs |
  | Library Catalogue | Google Scholar |
  | URL | https://www.taylorfrancis.com/chapters/edit/10.1201/9781003048220-17/assessing-emotions-pigs-determining-negative-positive-mental-states-eimear-murphy-luca-melotti-michael-mendl |
  | Accessed | 11/07/2025, 09:32:00 |
  | Publisher | Burleigh Dodds Science Publishing |
  | Pages | 455–496 |
  | Publication | Understanding the behaviour and improving the welfare of pigs |
  | Date Added | 11/07/2025, 09:32:09 |
  | Modified | 11/07/2025, 09:32:09 |
- ## Assessing emotions in pigs: determining negative and positive mental states

  |  |  |
  | --- | --- |
  | Item Type | Journal Article |
  | Author | Eimear Murphy |
  | Author | Luca Melotti |
  | Author | Michael Mendl |
  | Date | 2021 |
  | Short Title | Assessing emotions in pigs |
  | Library Catalogue | Google Scholar |
  | URL | https://www.taylorfrancis.com/chapters/edit/10.1201/9781003048220-17/assessing-emotions-pigs-determining-negative-positive-mental-states-eimear-murphy-luca-melotti-michael-mendl |
  | Accessed | 11/07/2025, 11:01:37 |
  | Publisher | Burleigh Dodds Science Publishing |
  | Pages | 455–496 |
  | Publication | Understanding the behaviour and improving the welfare of pigs |
  | Date Added | 11/07/2025, 11:01:44 |
  | Modified | 11/07/2025, 11:01:44 |
- ## Positive aspects of welfare in sheep: current debates and future opportunities

  |  |  |
  | --- | --- |
  | Item Type | Journal Article |
  | Author | Mukhtar Muhammad |
  | Author | Jessica E. Stokes |
  | Author | Louise Manning |
  | Date | 2022 |
  | Short Title | Positive aspects of welfare in sheep |
  | Library Catalogue | Google Scholar |
  | URL | https://www.mdpi.com/2076-2615/12/23/3265 |
  | Accessed | 11/07/2025, 10:00:33 |
  | Volume | 12 |
  | Publisher | MDPI |
  | Pages | 3265 |
  | Publication | Animals |
  | Issue | 23 |
  | Date Added | 11/07/2025, 10:00:41 |
  | Modified | 11/07/2025, 10:00:41 |
- ## Human animal relationships in Bos indicus cattle breeds addressed from a Five Domains welfare framework

  |  |  |
  | --- | --- |
  | Item Type | Journal Article |
  | Author | Daniel Mota-Rojas |
  | Author | Alexandra L. Whittaker |
  | Author | Ana C. Strappini |
  | Author | Agustín Orihuela |
  | Author | Adriana Domínguez-Oliva |
  | Author | Patricia Mora-Medina |
  | Author | Adolfo Álvarez-Macías |
  | Author | Ismael Hernández-Avalos |
  | Author | Adriana Olmos-Hernández |
  | Author | Brenda Reyes-Sotelo |
  | Date | 2024 |
  | Library Catalogue | Google Scholar |
  | URL | https://www.frontiersin.org/journals/veterinary-science/articles/10.3389/fvets.2024.1456120/full |
  | Accessed | 11/07/2025, 10:09:38 |
  | Volume | 11 |
  | Publisher | Frontiers Media SA |
  | Pages | 1456120 |
  | Publication | Frontiers in Veterinary Science |
  | Date Added | 11/07/2025, 10:09:48 |
  | Modified | 11/07/2025, 10:09:48 |

  ### Attachments

  - Available Version (via Google Scholar)
- ## Tactile, auditory, and visual stimulation as sensory enrichment for dairy cattle

  |  |  |
  | --- | --- |
  | Item Type | Journal Article |
  | Author | Daniel Mota-Rojas |
  | Author | Alexandra L. Whittaker |
  | Author | Adriana Domínguez-Oliva |
  | Author | Ana C. Strappini |
  | Author | Adolfo Álvarez-Macías |
  | Author | Patricia Mora-Medina |
  | Author | Marcelo Ghezzi |
  | Author | Pamela Lendez |
  | Author | Karina Lezama-García |
  | Author | Temple Grandin |
  | Date | 2024 |
  | Library Catalogue | Google Scholar |
  | URL | https://www.mdpi.com/2076-2615/14/9/1265 |
  | Accessed | 11/07/2025, 10:16:05 |
  | Volume | 14 |
  | Publisher | MDPI |
  | Pages | 1265 |
  | Publication | Animals |
  | Issue | 9 |
  | Date Added | 11/07/2025, 10:16:47 |
  | Modified | 11/07/2025, 10:16:47 |

  ### Attachments

  - Available Version (via Google Scholar)
- ## The use of draught animals in rural labour

  |  |  |
  | --- | --- |
  | Item Type | Journal Article |
  | Author | Daniel Mota-Rojas |
  | Author | Ada Braghieri |
  | Author | Adolfo Álvarez-Macías |
  | Author | Francesco Serrapica |
  | Author | Efrén Ramírez-Bribiesca |
  | Author | Rosy Cruz-Monterrosa |
  | Author | Felicia Masucci |
  | Author | Patricia Mora-Medina |
  | Author | Fabio Napolitano |
  | Date | 2021 |
  | Library Catalogue | Google Scholar |
  | URL | https://www.mdpi.com/2076-2615/11/9/2683 |
  | Accessed | 11/07/2025, 10:20:02 |
  | Volume | 11 |
  | Publisher | MDPI |
  | Pages | 2683 |
  | Publication | Animals |
  | Issue | 9 |
  | Date Added | 11/07/2025, 10:20:29 |
  | Modified | 11/07/2025, 10:20:29 |
- ## Current advances in assessment of dog’s emotions, facial expressions, and their use for clinical recognition of pain

  |  |  |
  | --- | --- |
  | Item Type | Journal Article |
  | Author | Daniel Mota-Rojas |
  | Author | Míriam Marcet-Rius |
  | Author | Asahi Ogi |
  | Author | Ismael Hernández-Ávalos |
  | Author | Chiara Mariti |
  | Author | Julio Martínez-Burnes |
  | Author | Patricia Mora-Medina |
  | Author | Alejandro Casas |
  | Author | Adriana Domínguez |
  | Author | Brenda Reyes |
  | Date | 2021 |
  | Library Catalogue | Google Scholar |
  | URL | https://www.mdpi.com/2076-2615/11/11/3334 |
  | Accessed | 11/07/2025, 10:20:14 |
  | Volume | 11 |
  | Publisher | MDPI |
  | Pages | 3334 |
  | Publication | Animals |
  | Issue | 11 |
  | Date Added | 11/07/2025, 10:20:29 |
  | Modified | 11/07/2025, 10:20:29 |

  ### Attachments

  - Available Version (via Google Scholar)
- ## Tactile, auditory, and visual stimulation as sensory enrichment for dairy cattle

  |  |  |
  | --- | --- |
  | Item Type | Journal Article |
  | Author | Daniel Mota-Rojas |
  | Author | Alexandra L. Whittaker |
  | Author | Adriana Domínguez-Oliva |
  | Author | Ana C. Strappini |
  | Author | Adolfo Álvarez-Macías |
  | Author | Patricia Mora-Medina |
  | Author | Marcelo Ghezzi |
  | Author | Pamela Lendez |
  | Author | Karina Lezama-García |
  | Author | Temple Grandin |
  | Date | 2024 |
  | Library Catalogue | Google Scholar |
  | URL | https://www.mdpi.com/2076-2615/14/9/1265 |
  | Accessed | 11/07/2025, 13:46:13 |
  | Volume | 14 |
  | Publisher | MDPI |
  | Pages | 1265 |
  | Publication | Animals |
  | Issue | 9 |
  | Date Added | 11/07/2025, 13:46:33 |
  | Modified | 11/07/2025, 13:46:33 |
- ## Methods of behavioral testing in dogs: a scoping review and analysis of test stimuli

  |  |  |
  | --- | --- |
  | Item Type | Journal Article |
  | Author | Ariella Y. Moser |
  | Author | Mitchell Welch |
  | Author | Wendy Y. Brown |
  | Author | Paul McGreevy |
  | Author | Pauleen C. Bennett |
  | Date | 2024 |
  | Short Title | Methods of behavioral testing in dogs |
  | Library Catalogue | Google Scholar |
  | URL | https://www.frontiersin.org/journals/veterinary-science/articles/10.3389/fvets.2024.1455574/full |
  | Accessed | 11/07/2025, 09:35:43 |
  | Volume | 11 |
  | Publisher | Frontiers Media SA |
  | Pages | 1455574 |
  | Publication | Frontiers in Veterinary Science |
  | Date Added | 11/07/2025, 09:36:11 |
  | Modified | 11/07/2025, 09:36:11 |

  ### Attachments

  - Available Version (via Google Scholar)
- ## Methods of behavioral testing in dogs: a scoping review and analysis of test stimuli

  |  |  |
  | --- | --- |
  | Item Type | Journal Article |
  | Author | Ariella Y. Moser |
  | Author | Mitchell Welch |
  | Author | Wendy Y. Brown |
  | Author | Paul McGreevy |
  | Author | Pauleen C. Bennett |
  | Date | 2024 |
  | Short Title | Methods of behavioral testing in dogs |
  | Library Catalogue | Google Scholar |
  | URL | https://www.frontiersin.org/journals/veterinary-science/articles/10.3389/fvets.2024.1455574/full |
  | Accessed | 11/07/2025, 11:09:24 |
  | Volume | 11 |
  | Publisher | Frontiers Media SA |
  | Pages | 1455574 |
  | Publication | Frontiers in Veterinary Science |
  | Date Added | 11/07/2025, 11:09:44 |
  | Modified | 11/07/2025, 11:09:44 |

  ### Attachments

  - Available Version (via Google Scholar)
- ## Methods of behavioral testing in dogs: a scoping review and analysis of test stimuli

  |  |  |
  | --- | --- |
  | Item Type | Journal Article |
  | Author | Ariella Y. Moser |
  | Author | Mitchell Welch |
  | Author | Wendy Y. Brown |
  | Author | Paul McGreevy |
  | Author | Pauleen C. Bennett |
  | Date | 2024 |
  | Short Title | Methods of behavioral testing in dogs |
  | Library Catalogue | Google Scholar |
  | URL | https://www.frontiersin.org/journals/veterinary-science/articles/10.3389/fvets.2024.1455574/full |
  | Accessed | 11/07/2025, 13:44:52 |
  | Volume | 11 |
  | Publisher | Frontiers Media SA |
  | Pages | 1455574 |
  | Publication | Frontiers in Veterinary Science |
  | Date Added | 11/07/2025, 13:45:12 |
  | Modified | 11/07/2025, 13:45:12 |

  ### Attachments

  - Available Version (via Google Scholar)
- ## The Role of Nutrition Across Production Stages to Improve Sow Longevity

  |  |  |
  | --- | --- |
  | Item Type | Journal Article |
  | Author | Matheus Saliba Monteiro |
  | Author | Rafaella Fernandes Carnevale |
  | Author | Bruno Bracco Donatelli Muro |
  | Author | Ana Lígia Braga Mezzina |
  | Author | Bruno Braga Carnino |
  | Author | André Pegoraro Poor |
  | Author | Carlos Emilio Cabrera Matajira |
  | Author | Cesar Augusto Pospissil Garbossa |
  | Date | 2025 |
  | Library Catalogue | Google Scholar |
  | URL | https://www.mdpi.com/2076-2615/15/2/189 |
  | Accessed | 11/07/2025, 11:14:44 |
  | Volume | 15 |
  | Publisher | MDPI |
  | Pages | 189 |
  | Publication | Animals |
  | Issue | 2 |
  | Date Added | 11/07/2025, 11:14:45 |
  | Modified | 11/07/2025, 11:14:45 |
- ## The Role of Nutrition Across Production Stages to Improve Sow Longevity

  |  |  |
  | --- | --- |
  | Item Type | Journal Article |
  | Author | Matheus Saliba Monteiro |
  | Author | Rafaella Fernandes Carnevale |
  | Author | Bruno Bracco Donatelli Muro |
  | Author | Ana Lígia Braga Mezzina |
  | Author | Bruno Braga Carnino |
  | Author | André Pegoraro Poor |
  | Author | Carlos Emilio Cabrera Matajira |
  | Author | Cesar Augusto Pospissil Garbossa |
  | Date | 2025 |
  | Library Catalogue | Google Scholar |
  | URL | https://www.mdpi.com/2076-2615/15/2/189 |
  | Accessed | 11/07/2025, 13:43:59 |
  | Volume | 15 |
  | Publisher | MDPI |
  | Pages | 189 |
  | Publication | Animals |
  | Issue | 2 |
  | Date Added | 11/07/2025, 13:44:05 |
  | Modified | 11/07/2025, 13:44:05 |
- ## A review on Japanese Encephalitis virus emergence, pathogenesis and detection: From conventional diagnostics to emerging rapid detection techniques

  |  |  |
  | --- | --- |
  | Item Type | Journal Article |
  | Author | Fatima Mohsin |
  | Author | Shariq Suleman |
  | Author | Nigar Anzar |
  | Author | Jagriti Narang |
  | Author | Shikha Wadhwa |
  | Date | 2022 |
  | Short Title | A review on Japanese Encephalitis virus emergence, pathogenesis and detection |
  | Library Catalogue | Google Scholar |
  | URL | https://www.sciencedirect.com/science/article/pii/S0141813022014507 |
  | Accessed | 11/07/2025, 09:58:00 |
  | Volume | 217 |
  | Publisher | Elsevier |
  | Pages | 435–448 |
  | Publication | International Journal of Biological Macromolecules |
  | Date Added | 11/07/2025, 09:58:25 |
  | Modified | 11/07/2025, 09:58:25 |
- ## Service dogs as interventions: State of the science

  |  |  |
  | --- | --- |
  | Item Type | Journal Article |
  | Author | Susan J. Modlin |
  | Date | 2000 |
  | Short Title | Service dogs as interventions |
  | Library Catalogue | Google Scholar |
  | URL | https://journals.lww.com/rehabnursingjournal/abstract/2000/11000/service\_dogs\_as\_interventions\_\_state\_of\_the.4.aspx |
  | Accessed | 11/07/2025, 10:12:00 |
  | Volume | 25 |
  | Publisher | LWW |
  | Pages | 212–219 |
  | Publication | Rehabilitation Nursing Journal |
  | Issue | 6 |
  | Date Added | 11/07/2025, 10:12:39 |
  | Modified | 11/07/2025, 10:12:39 |
- ## Exploring Emotional Connections: A Systematic Literature Review of Attachment in Human-Robot Interaction

  |  |  |
  | --- | --- |
  | Item Type | Journal Article |
  | Author | Jennifer J. Mitchell |
  | Author | Myounghoon Jeon |
  | Date | 2025-01-23 |
  | Language | en |
  | Short Title | Exploring Emotional Connections |
  | Library Catalogue | Crossref |
  | URL | https://www.tandfonline.com/doi/full/10.1080/10447318.2024.2445100 |
  | Accessed | 11/07/2025, 13:44:53 |
  | Publisher | Informa UK Limited |
  | Pages | 1-22 |
  | Publication | International Journal of Human–Computer Interaction |
  | DOI | 10.1080/10447318.2024.2445100 |
  | ISSN | 1044-7318, 1532-7590 |
  | Date Added | 11/07/2025, 13:45:12 |
  | Modified | 11/07/2025, 13:45:12 |
- ## The importance of evaluating positive welfare characteristics and temperament in working therapy dogs

  |  |  |
  | --- | --- |
  | Item Type | Journal Article |
  | Author | Sharmaine L. Miller |
  | Author | James A. Serpell |
  | Author | Kathryn R. Dalton |
  | Author | Kaitlin B. Waite |
  | Author | Daniel O. Morris |
  | Author | Laurel E. Redding |
  | Author | Nancy A. Dreschel |
  | Author | Meghan F. Davis |
  | Date | 2022 |
  | Library Catalogue | Google Scholar |
  | URL | https://www.frontiersin.org/articles/10.3389/fvets.2022.844252/full |
  | Accessed | 11/07/2025, 10:20:11 |
  | Volume | 9 |
  | Publisher | Frontiers Media SA |
  | Pages | 844252 |
  | Publication | Frontiers in veterinary science |
  | Date Added | 11/07/2025, 10:20:29 |
  | Modified | 11/07/2025, 10:20:29 |

  ### Attachments

  - Available Version (via Google Scholar)
- ## The Smartphone Psychology Manifesto

  |  |  |
  | --- | --- |
  | Item Type | Journal Article |
  | Author | Geoffrey Miller |
  | Abstract | By 2025, when most of today’s psychology undergraduates will be in their mid-30s, more than 5 billion people on our planet will be using ultra-broadband, sensor-rich smartphones far beyond the abilities of today’s iPhones, Androids, and Blackberries. Although smartphones were not designed for psychological research, they can collect vast amounts of ecologically valid data, easily and quickly, from large global samples. If participants download the right “psych apps,” smartphones can record where they are, what they are doing, and what they can see and hear and can run interactive surveys, tests, and experiments through touch screens and wireless connections to nearby screens, headsets, biosensors, and other peripherals. This article reviews previous behavioral research using mobile electronic devices, outlines what smartphones can do now and will be able to do in the near future, explains how a smartphone study could work practically given current technology (e.g., in studying ovulatory cycle effects on women’s sexuality), discusses some limitations and challenges of smartphone research, and compares smartphones to other research methods. Smartphone research will require new skills in app development and data analysis and will raise tough new ethical issues, but smartphones could transform psychology even more profoundly than PCs and brain imaging did. |
  | Date | 05/2012 |
  | Language | en |
  | Library Catalogue | Crossref |
  | URL | https://journals.sagepub.com/doi/10.1177/1745691612441215 |
  | Accessed | 11/07/2025, 11:14:45 |
  | Rights | https://journals.sagepub.com/page/policies/text-and-data-mining-license |
  | Volume | 7 |
  | Publisher | SAGE Publications |
  | Pages | 221-237 |
  | Publication | Perspectives on Psychological Science |
  | DOI | 10.1177/1745691612441215 |
  | Issue | 3 |
  | Journal Abbr | Perspect Psychol Sci |
  | ISSN | 1745-6916, 1745-6924 |
  | Date Added | 11/07/2025, 11:14:45 |
  | Modified | 11/07/2025, 11:14:45 |
- ## Searching where the treasure is: on the emergence of human companion animal partnership (HCAP)

  |  |  |
  | --- | --- |
  | Item Type | Journal Article |
  | Author | Ádám Miklósi |
  | Author | Judit Abdai |
  | Author | Andrea Temesi |
  | Abstract | AbstractIn our view, the discipline, often referred to as human–animal interaction (HAI), lacks a well-defined conceptual framework. It is too narrow both with respect to the animal species investigated and the nature of human–animal interactions studied. So instead, we introduce the term human–companion animal partnership (HCAP) that is not only a better descriptor for most research efforts within HAI but also helps to direct research efforts on an ethological basis. In our approach, ‘companion’ is a function and not a feature of some species. This means that many species had and could have a potential to form mixed social groups with humans if they evolve some capacity of social competence. This view may initiate new comparative research involving a range of species to find out how complex social engagement could be maintained in such hetero-specific social groups based on evolutionary heritage, recent selection and individual experience (socialisation). Our approach emphasises the role of human caring behaviour and social competence in the emergence of a partnership with several species, and thus could also help in setting expectations for welfare and aid in designing artificial companions for specific purposes. |
  | Date | 03/2021 |
  | Language | en |
  | Short Title | Searching where the treasure is |
  | Library Catalogue | Crossref |
  | URL | https://link.springer.com/10.1007/s10071-020-01467-z |
  | Accessed | 11/07/2025, 09:54:51 |
  | Rights | https://creativecommons.org/licenses/by/4.0 |
  | Volume | 24 |
  | Publisher | Springer Science and Business Media LLC |
  | Pages | 387-394 |
  | Publication | Animal Cognition |
  | DOI | 10.1007/s10071-020-01467-z |
  | Issue | 2 |
  | Journal Abbr | Anim Cogn |
  | ISSN | 1435-9448, 1435-9456 |
  | Date Added | 11/07/2025, 09:55:10 |
  | Modified | 11/07/2025, 09:55:10 |

  ### Attachments

  - Available Version (via Google Scholar)
  - Available Version (via Google Scholar)
- ## Searching where the treasure is: on the emergence of human companion animal partnership (HCAP)

  |  |  |
  | --- | --- |
  | Item Type | Journal Article |
  | Author | Ádám Miklósi |
  | Author | Judit Abdai |
  | Author | Andrea Temesi |
  | Abstract | AbstractIn our view, the discipline, often referred to as human–animal interaction (HAI), lacks a well-defined conceptual framework. It is too narrow both with respect to the animal species investigated and the nature of human–animal interactions studied. So instead, we introduce the term human–companion animal partnership (HCAP) that is not only a better descriptor for most research efforts within HAI but also helps to direct research efforts on an ethological basis. In our approach, ‘companion’ is a function and not a feature of some species. This means that many species had and could have a potential to form mixed social groups with humans if they evolve some capacity of social competence. This view may initiate new comparative research involving a range of species to find out how complex social engagement could be maintained in such hetero-specific social groups based on evolutionary heritage, recent selection and individual experience (socialisation). Our approach emphasises the role of human caring behaviour and social competence in the emergence of a partnership with several species, and thus could also help in setting expectations for welfare and aid in designing artificial companions for specific purposes. |
  | Date | 03/2021 |
  | Language | en |
  | Short Title | Searching where the treasure is |
  | Library Catalogue | Crossref |
  | URL | https://link.springer.com/10.1007/s10071-020-01467-z |
  | Accessed | 11/07/2025, 10:18:17 |
  | Rights | https://creativecommons.org/licenses/by/4.0 |
  | Volume | 24 |
  | Publisher | Springer Science and Business Media LLC |
  | Pages | 387-394 |
  | Publication | Animal Cognition |
  | DOI | 10.1007/s10071-020-01467-z |
  | Issue | 2 |
  | Journal Abbr | Anim Cogn |
  | ISSN | 1435-9448, 1435-9456 |
  | Date Added | 11/07/2025, 10:18:17 |
  | Modified | 11/07/2025, 10:18:17 |

  ### Attachments

  - Available Version (via Google Scholar)
- ## Pain Relief Interventions in Australian Livestock Husbandry: A Review of Animal Welfare and Pain Duration

  |  |  |
  | --- | --- |
  | Item Type | Journal Article |
  | Author | Lee Metcalf |
  | Author | Sabrina Lomax |
  | Author | Dominique Van der Saag |
  | Author | Sanjay Garg |
  | Author | Peter J. White |
  | Date | 2024 |
  | Short Title | Pain Relief Interventions in Australian Livestock Husbandry |
  | Library Catalogue | Google Scholar |
  | URL | https://www.mdpi.com/2076-2615/14/13/1901 |
  | Accessed | 11/07/2025, 09:37:37 |
  | Volume | 14 |
  | Publisher | MDPI |
  | Pages | 1901 |
  | Publication | Animals |
  | Issue | 13 |
  | Date Added | 11/07/2025, 09:38:03 |
  | Modified | 11/07/2025, 09:38:03 |

  ### Attachments

  - Available Version (via Google Scholar)
- ## Pain Relief Interventions in Australian Livestock Husbandry: A Review of Animal Welfare and Pain Duration

  |  |  |
  | --- | --- |
  | Item Type | Journal Article |
  | Author | Lee Metcalf |
  | Author | Sabrina Lomax |
  | Author | Dominique Van der Saag |
  | Author | Sanjay Garg |
  | Author | Peter J. White |
  | Date | 2024 |
  | Short Title | Pain Relief Interventions in Australian Livestock Husbandry |
  | Library Catalogue | Google Scholar |
  | URL | https://www.mdpi.com/2076-2615/14/13/1901 |
  | Accessed | 11/07/2025, 13:44:37 |
  | Volume | 14 |
  | Publisher | MDPI |
  | Pages | 1901 |
  | Publication | Animals |
  | Issue | 13 |
  | Date Added | 11/07/2025, 13:45:12 |
  | Modified | 11/07/2025, 13:45:12 |

  ### Attachments

  - Available Version (via Google Scholar)
- ## Human-Dog-Relationship and its positive effects on dogs and their humans with special needs

  |  |  |
  | --- | --- |
  | Item Type | Journal Article |
  | Author | David Menor-Campos |
  | Author | Angelo Gazzano |
  | Author | Karina Lezama-García |
  | Author | Adriana Domínguez-Oliva |
  | Author | Asahi Ogi |
  | Author | Daniel Mota-Rojas |
  | Date | 2023 |
  | Library Catalogue | Google Scholar |
  | URL | https://www.malque.pub/ojs/index.php/jabb/article/view/1213 |
  | Accessed | 11/07/2025, 09:57:52 |
  | Volume | 11 |
  | Publication | Journal of Animal Behaviour and Biometeorology |
  | Date Added | 11/07/2025, 09:58:25 |
  | Modified | 11/07/2025, 09:58:25 |

  ### Attachments

  - Available Version (via Google Scholar)
  - Available Version (via Google Scholar)
- ## Human-Dog-Relationship and its positive effects on dogs and their humans with special needs

  |  |  |
  | --- | --- |
  | Item Type | Journal Article |
  | Author | David Menor-Campos |
  | Author | Angelo Gazzano |
  | Author | Karina Lezama-García |
  | Author | Adriana Domínguez-Oliva |
  | Author | Asahi Ogi |
  | Author | Daniel Mota-Rojas |
  | Date | 2023 |
  | Library Catalogue | Google Scholar |
  | URL | https://www.malque.pub/ojs/index.php/jabb/article/view/1213 |
  | Accessed | 11/07/2025, 11:14:28 |
  | Volume | 11 |
  | Publication | Journal of Animal Behaviour and Biometeorology |
  | Date Added | 11/07/2025, 11:14:45 |
  | Modified | 11/07/2025, 11:14:45 |
- ## Effectiveness of Animal-assisted Interventions (AAIs) in Treatment of Adults with Depressive Symptoms: A Systematic Review

  |  |  |
  | --- | --- |
  | Item Type | Journal Article |
  | Author | Chris McFalls-Steger |
  | Author | David Patterson |
  | Author | Phyllis Thompson |
  | Abstract | Abstract Animal-assisted interventions (AAIs) have become widespread, with programs targeting various populations and mental health conditions. Despite its popularity, AAI’s operational definition and its efficacy are unclear. This systematic review aims to assess the utility of AAIs in decreasing depressive symptoms in adults based upon results of empirically validated depression assessment scales used by researchers. A systematic published literature search was conducted using Web of Science, PsychInfo, PubMed, ProQuest, SCOPUS, CINAHL, Social Work Abstracts, Web of Science, and Google Scholar. Peer-reviewed research articles on the effectiveness of AAIs on depressive symptoms in adults using empirically validated depression scales published from 2010 through October 2020 were chosen for this systematic review. Search results were filtered to include only quantitative, peer-reviewed articles for adults 18 and over; those were reviewed, and only journal articles using an empirically established depression evaluation tool were chosen. A total of 10 quantitative articles met these inclusion criteria. Overall, research design quality was low, but AAI had a statistically significant effect on outcomes in most studies. Results are moderately favorable but more thorough, standardized, and controlled research is needed. |
  | Date | 12/2021 |
  | Language | en |
  | Short Title | Effectiveness of Animal-assisted Interventions (AAIs) in Treatment of Adults with Depressive Symptoms |
  | Library Catalogue | Crossref |
  | URL | http://www.cabidigitallibrary.org/doi/10.1079/hai.2021.0007 |
  | Accessed | 11/07/2025, 10:12:06 |
  | Publisher | CABI Publishing |
  | Publication | Human-animal interaction bulletin |
  | DOI | 10.1079/hai.2021.0007 |
  | ISSN | 2333-522X |
  | Date Added | 11/07/2025, 10:12:39 |
  | Modified | 11/07/2025, 10:12:39 |
- ## Understanding the role of therapy dogs in human health promotion

  |  |  |
  | --- | --- |
  | Item Type | Journal Article |
  | Author | Sonya McDowall |
  | Author | Susan J. Hazel |
  | Author | Mia Cobb |
  | Author | Anne Hamilton-Bruce |
  | Date | 2023 |
  | Library Catalogue | Google Scholar |
  | URL | https://www.mdpi.com/1660-4601/20/10/5801 |
  | Accessed | 11/07/2025, 09:29:12 |
  | Volume | 20 |
  | Publisher | MDPI |
  | Pages | 5801 |
  | Publication | International Journal of Environmental Research and Public Health |
  | Issue | 10 |
  | Date Added | 11/07/2025, 09:29:28 |
  | Modified | 11/07/2025, 09:29:28 |

  ### Attachments

  - Available Version (via Google Scholar)
- ## Human–animal interaction and perinatal mental health: A narrative review of selected literature and call for research

  |  |  |
  | --- | --- |
  | Item Type | Journal Article |
  | Author | Shelby E. McDonald |
  | Author | Camie A. Tomlinson |
  | Author | Jennifer W. Applebaum |
  | Author | Sara W. Moyer |
  | Author | Samantha M. Brown |
  | Author | Sue Carter |
  | Author | Patricia A. Kinser |
  | Date | 2021 |
  | Short Title | Human–animal interaction and perinatal mental health |
  | Library Catalogue | Google Scholar |
  | URL | https://www.mdpi.com/1660-4601/18/19/10114 |
  | Accessed | 11/07/2025, 10:05:21 |
  | Volume | 18 |
  | Publisher | MDPI |
  | Pages | 10114 |
  | Publication | International Journal of Environmental Research and Public Health |
  | Issue | 19 |
  | Date Added | 11/07/2025, 10:05:27 |
  | Modified | 11/07/2025, 10:05:27 |
- ## Heart Rate Variability: New Perspectives on Physiological Mechanisms, Assessment of Self-regulatory Capacity, and Health Risk

  |  |  |
  | --- | --- |
  | Item Type | Journal Article |
  | Author | Rollin Mccraty |
  | Author | Fred Shaffer |
  | Abstract | Heart rate variability, the change in the time intervals between adjacent heartbeats, is an emergent property of interdependent regulatory systems that operates on different time scales to adapt to environmental and psychological challenges. This article briefly reviews neural regulation of the heart and offers some new perspectives on mechanisms underlying the very low frequency rhythm of heart rate variability. Interpretation of heart rate variability rhythms in the context of health risk and physiological and psychological self-regulatory capacity assessment is discussed. The cardiovascular regulatory centers in the spinal cord and medulla integrate inputs from higher brain centers with afferent cardiovascular system inputs to adjust heart rate and blood pressure via sympathetic and parasympathetic efferent pathways. We also discuss the intrinsic cardiac nervous system and the heart-brain connection pathways, through which afferent information can influence activity in the subcortical, frontocortical, and motor cortex areas. In addition, the use of realtime HRV feedback to increase self-regulatory capacity is reviewed. We conclude that the heart's rhythms are characterized by both complexity and stability over longer time scales that reflect both physiological and psychological functional status of these internal self-regulatory systems. |
  | Date | 01/2015 |
  | Language | en |
  | Short Title | Heart Rate Variability |
  | Library Catalogue | Crossref |
  | URL | https://journals.sagepub.com/doi/10.7453/gahmj.2014.073 |
  | Accessed | 11/07/2025, 09:37:45 |
  | Rights | https://journals.sagepub.com/page/policies/text-and-data-mining-license |
  | Volume | 4 |
  | Publisher | SAGE Publications |
  | Pages | 46-61 |
  | Publication | Global Advances in Health and Medicine |
  | DOI | 10.7453/gahmj.2014.073 |
  | Issue | 1 |
  | Journal Abbr | Glob Adv Health Med |
  | ISSN | 2164-957X, 2164-9561 |
  | Date Added | 11/07/2025, 09:38:03 |
  | Modified | 11/07/2025, 09:38:03 |

  ### Attachments

  - Available Version (via Google Scholar)
- ## Heart Rate Variability: New Perspectives on Physiological Mechanisms, Assessment of Self-regulatory Capacity, and Health Risk

  |  |  |
  | --- | --- |
  | Item Type | Journal Article |
  | Author | Rollin Mccraty |
  | Author | Fred Shaffer |
  | Abstract | Heart rate variability, the change in the time intervals between adjacent heartbeats, is an emergent property of interdependent regulatory systems that operates on different time scales to adapt to environmental and psychological challenges. This article briefly reviews neural regulation of the heart and offers some new perspectives on mechanisms underlying the very low frequency rhythm of heart rate variability. Interpretation of heart rate variability rhythms in the context of health risk and physiological and psychological self-regulatory capacity assessment is discussed. The cardiovascular regulatory centers in the spinal cord and medulla integrate inputs from higher brain centers with afferent cardiovascular system inputs to adjust heart rate and blood pressure via sympathetic and parasympathetic efferent pathways. We also discuss the intrinsic cardiac nervous system and the heart-brain connection pathways, through which afferent information can influence activity in the subcortical, frontocortical, and motor cortex areas. In addition, the use of realtime HRV feedback to increase self-regulatory capacity is reviewed. We conclude that the heart's rhythms are characterized by both complexity and stability over longer time scales that reflect both physiological and psychological functional status of these internal self-regulatory systems. |
  | Date | 01/2015 |
  | Language | en |
  | Short Title | Heart Rate Variability |
  | Library Catalogue | Crossref |
  | URL | https://journals.sagepub.com/doi/10.7453/gahmj.2014.073 |
  | Accessed | 11/07/2025, 09:55:10 |
  | Rights | https://journals.sagepub.com/page/policies/text-and-data-mining-license |
  | Volume | 4 |
  | Publisher | SAGE Publications |
  | Pages | 46-61 |
  | Publication | Global Advances in Health and Medicine |
  | DOI | 10.7453/gahmj.2014.073 |
  | Issue | 1 |
  | Journal Abbr | Glob Adv Health Med |
  | ISSN | 2164-957X, 2164-9561 |
  | Date Added | 11/07/2025, 09:55:10 |
  | Modified | 11/07/2025, 09:55:12 |

  ### Attachments

  - Available Version (via Google Scholar)
- ## Heart Rate Variability: New Perspectives on Physiological Mechanisms, Assessment of Self-regulatory Capacity, and Health Risk

  |  |  |
  | --- | --- |
  | Item Type | Journal Article |
  | Author | Rollin Mccraty |
  | Author | Fred Shaffer |
  | Abstract | Heart rate variability, the change in the time intervals between adjacent heartbeats, is an emergent property of interdependent regulatory systems that operates on different time scales to adapt to environmental and psychological challenges. This article briefly reviews neural regulation of the heart and offers some new perspectives on mechanisms underlying the very low frequency rhythm of heart rate variability. Interpretation of heart rate variability rhythms in the context of health risk and physiological and psychological self-regulatory capacity assessment is discussed. The cardiovascular regulatory centers in the spinal cord and medulla integrate inputs from higher brain centers with afferent cardiovascular system inputs to adjust heart rate and blood pressure via sympathetic and parasympathetic efferent pathways. We also discuss the intrinsic cardiac nervous system and the heart-brain connection pathways, through which afferent information can influence activity in the subcortical, frontocortical, and motor cortex areas. In addition, the use of realtime HRV feedback to increase self-regulatory capacity is reviewed. We conclude that the heart's rhythms are characterized by both complexity and stability over longer time scales that reflect both physiological and psychological functional status of these internal self-regulatory systems. |
  | Date | 01/2015 |
  | Language | en |
  | Short Title | Heart Rate Variability |
  | Library Catalogue | Crossref |
  | URL | https://journals.sagepub.com/doi/10.7453/gahmj.2014.073 |
  | Accessed | 11/07/2025, 10:00:41 |
  | Rights | https://journals.sagepub.com/page/policies/text-and-data-mining-license |
  | Volume | 4 |
  | Publisher | SAGE Publications |
  | Pages | 46-61 |
  | Publication | Global Advances in Health and Medicine |
  | DOI | 10.7453/gahmj.2014.073 |
  | Issue | 1 |
  | Journal Abbr | Glob Adv Health Med |
  | ISSN | 2164-957X, 2164-9561 |
  | Date Added | 11/07/2025, 10:00:41 |
  | Modified | 11/07/2025, 10:00:41 |

  ### Attachments

  - Available Version (via Google Scholar)
- ## How can we assess positive welfare in ruminants?

  |  |  |
  | --- | --- |
  | Item Type | Journal Article |
  | Author | Silvana Mattiello |
  | Author | Monica Battini |
  | Author | Giuseppe De Rosa |
  | Author | Fabio Napolitano |
  | Author | Cathy Dwyer |
  | Date | 2019 |
  | Library Catalogue | Google Scholar |
  | URL | https://www.mdpi.com/2076-2615/9/10/758 |
  | Accessed | 11/07/2025, 09:35:38 |
  | Volume | 9 |
  | Publisher | MDPI |
  | Pages | 758 |
  | Publication | Animals |
  | Issue | 10 |
  | Date Added | 11/07/2025, 09:36:11 |
  | Modified | 11/07/2025, 09:36:11 |
- ## animals MDPI

  |  |  |
  | --- | --- |
  | Item Type | Journal Article |
  | Author | Silvana Mattiello |
  | Author | Monica Battini |
  | Author | Giuseppe De Rosa |
  | Author | Fabio Napolitano |
  | Author | Cathy Dwyer |
  | Date | 2020 |
  | Library Catalogue | Google Scholar |
  | URL | https://books.google.com/books?hl=it&lr=&id=HuncDwAAQBAJ&oi=fnd&pg=PA123&dq=%22human+animal+interaction%22+AND+%22physiological+measures%22&ots=u2HPXefXlZ&sig=cPiDeB1Z4ipO-U1QksQWebqCpGM |
  | Accessed | 11/07/2025, 09:35:56 |
  | Publisher | MDPI |
  | Pages | 123 |
  | Publication | Positive Aspects of Animal Welfare |
  | Date Added | 11/07/2025, 09:36:11 |
  | Modified | 11/07/2025, 09:36:11 |
- ## animals MDPI

  |  |  |
  | --- | --- |
  | Item Type | Journal Article |
  | Author | Silvana Mattiello |
  | Author | Monica Battini |
  | Author | Giuseppe De Rosa |
  | Author | Fabio Napolitano |
  | Author | Cathy Dwyer |
  | Date | 2020 |
  | Library Catalogue | Google Scholar |
  | URL | https://books.google.com/books?hl=it&lr=&id=HuncDwAAQBAJ&oi=fnd&pg=PA123&dq=%22human+animal+interaction%22+AND+%22heart+rate%22+&ots=u2HPXfbTnU&sig=ilyQjMkbgN7R1w84lUL6IjWJLSQ |
  | Accessed | 11/07/2025, 11:09:34 |
  | Publisher | MDPI |
  | Pages | 123 |
  | Publication | Positive Aspects of Animal Welfare |
  | Date Added | 11/07/2025, 11:09:44 |
  | Modified | 11/07/2025, 11:09:44 |
- ## The General and Systemic Consequences of Obesity in Cats and Dogs: https://doi. org/10.12982/VIS. 2024.020

  |  |  |
  | --- | --- |
  | Item Type | Journal Article |
  | Author | Wajahat Masood |
  | Date | 2024 |
  | Short Title | The General and Systemic Consequences of Obesity in Cats and Dogs |
  | Library Catalogue | Google Scholar |
  | URL | https://he02.tci-thaijo.org/index.php/vis/article/view/264066 |
  | Accessed | 11/07/2025, 11:15:48 |
  | Volume | 22 |
  | Pages | 265–250 |
  | Publication | Veterinary Integrative Sciences |
  | Issue | 1 |
  | Date Added | 11/07/2025, 11:15:52 |
  | Modified | 11/07/2025, 11:15:52 |

  ### Attachments

  - Available Version (via Google Scholar)
- ## Bringing human‐animal interaction to sport: Potential impacts on athletic performance

  |  |  |
  | --- | --- |
  | Item Type | Journal Article |
  | Author | Sutton Marvin |
  | Author | Kennet Sorenson |
  | Author | Jeffrey R. Stevens |
  | Abstract | ABSTRACTTo gain an edge in performance, athletes, coaches, trainers, and sport psychologists worldwide leverage findings from psychological research to develop training and performance strategies. The field of sport psychology draws upon research on stress, anxiety, mindfulness, and team building to develop these strategies. Here, we introduce human‐animal interaction as a potential area of research that may apply to athletic performance. Structured interactions with animals—particularly therapy dogs—can provide physiological benefits associated with stress and the oxytocin system, psychological benefits for anxiety and motivation, and social benefits through social support. Yet these effects have not yet been systematically investigated in athletes. Integration of human‐animal interactions into athletics can occur through animal visitation programmes and resident therapy animal programmes. Integrating human‐animal interactions into athletics presents some unique challenges and limitations that must be considered before implementing these programmes, and these interactions are not a panacea that will work in every situation. But, given the amount of human‐animal interaction research suggesting benefits in medicine, mental health, and education contexts, it is worthwhile exploring potential benefits not just for athletic performance, but also for injury prevention and recovery.HighlightsHuman–animal interaction is a potential area of research that may apply to athletic performance.Structured interactions with animals can provide physiological, psychological, and social benefits to athletes, through it is not a panacea that will work in every situation.Integrating human–animal interactions into athletics presents some unique challenges and limitations that must be considered before implementing these programs. |
  | Date | 07/2022 |
  | Language | en |
  | Short Title | Bringing human‐animal interaction to sport |
  | Library Catalogue | Crossref |
  | URL | https://onlinelibrary.wiley.com/doi/10.1080/17461391.2021.1916084 |
  | Accessed | 11/07/2025, 09:27:12 |
  | Rights | http://onlinelibrary.wiley.com/termsAndConditions#vor |
  | Volume | 22 |
  | Publisher | Wiley |
  | Pages | 955-963 |
  | Publication | European Journal of Sport Science |
  | DOI | 10.1080/17461391.2021.1916084 |
  | Issue | 7 |
  | ISSN | 1746-1391, 1536-7290 |
  | Date Added | 11/07/2025, 09:27:42 |
  | Modified | 11/07/2025, 09:27:42 |

  ### Attachments

  - Submitted Version
- ## Bringing human‐animal interaction to sport: Potential impacts on athletic performance

  |  |  |
  | --- | --- |
  | Item Type | Journal Article |
  | Author | Sutton Marvin |
  | Author | Kennet Sorenson |
  | Author | Jeffrey R. Stevens |
  | Abstract | ABSTRACTTo gain an edge in performance, athletes, coaches, trainers, and sport psychologists worldwide leverage findings from psychological research to develop training and performance strategies. The field of sport psychology draws upon research on stress, anxiety, mindfulness, and team building to develop these strategies. Here, we introduce human‐animal interaction as a potential area of research that may apply to athletic performance. Structured interactions with animals—particularly therapy dogs—can provide physiological benefits associated with stress and the oxytocin system, psychological benefits for anxiety and motivation, and social benefits through social support. Yet these effects have not yet been systematically investigated in athletes. Integration of human‐animal interactions into athletics can occur through animal visitation programmes and resident therapy animal programmes. Integrating human‐animal interactions into athletics presents some unique challenges and limitations that must be considered before implementing these programmes, and these interactions are not a panacea that will work in every situation. But, given the amount of human‐animal interaction research suggesting benefits in medicine, mental health, and education contexts, it is worthwhile exploring potential benefits not just for athletic performance, but also for injury prevention and recovery.HighlightsHuman–animal interaction is a potential area of research that may apply to athletic performance.Structured interactions with animals can provide physiological, psychological, and social benefits to athletes, through it is not a panacea that will work in every situation.Integrating human–animal interactions into athletics presents some unique challenges and limitations that must be considered before implementing these programs. |
  | Date | 07/2022 |
  | Language | en |
  | Short Title | Bringing human‐animal interaction to sport |
  | Library Catalogue | Crossref |
  | URL | https://onlinelibrary.wiley.com/doi/10.1080/17461391.2021.1916084 |
  | Accessed | 11/07/2025, 10:04:59 |
  | Rights | http://onlinelibrary.wiley.com/termsAndConditions#vor |
  | Volume | 22 |
  | Publisher | Wiley |
  | Pages | 955-963 |
  | Publication | European Journal of Sport Science |
  | DOI | 10.1080/17461391.2021.1916084 |
  | Issue | 7 |
  | ISSN | 1746-1391, 1536-7290 |
  | Date Added | 11/07/2025, 10:05:27 |
  | Modified | 11/07/2025, 10:05:27 |

  ### Attachments

  - Submitted Version
- ## Influence of the human-animal relationship on productivity and animal welfare in dairy farms.

  |  |  |
  | --- | --- |
  | Item Type | Journal Article |
  | Author | G. M. Martínez |
  | Author | V. H. Suárez |
  | Author | M. D. Ghezzi |
  | Date | 2016 |
  | Library Catalogue | Google Scholar |
  | URL | https://www.cabidigitallibrary.org/doi/full/10.5555/20173099612 |
  | Accessed | 11/07/2025, 10:13:25 |
  | Date Added | 11/07/2025, 10:13:55 |
  | Modified | 11/07/2025, 10:13:55 |
- ## The Science Behind Animal-Assisted Therapy

  |  |  |
  | --- | --- |
  | Item Type | Journal Article |
  | Author | Dawn A. Marcus |
  | Date | 04/2013 |
  | Language | en |
  | Library Catalogue | Crossref |
  | URL | http://link.springer.com/10.1007/s11916-013-0322-2 |
  | Accessed | 11/07/2025, 10:20:25 |
  | Rights | http://www.springer.com/tdm |
  | Volume | 17 |
  | Publisher | Springer Science and Business Media LLC |
  | Publication | Current Pain and Headache Reports |
  | DOI | 10.1007/s11916-013-0322-2 |
  | Issue | 4 |
  | Journal Abbr | Curr Pain Headache Rep |
  | ISSN | 1531-3433, 1534-3081 |
  | Date Added | 11/07/2025, 10:20:29 |
  | Modified | 11/07/2025, 10:20:29 |

  ### Attachments

  - Available Version (via Google Scholar)
- ## Potential mechanisms of action and outcomes of equine-assisted services for veterans with a history of trauma: A narrative review of the literature

  |  |  |
  | --- | --- |
  | Item Type | Journal Article |
  | Author | William R. Marchand |
  | Date | 2023 |
  | Short Title | Potential mechanisms of action and outcomes of equine-assisted services for veterans with a history of trauma |
  | Library Catalogue | Google Scholar |
  | URL | https://www.mdpi.com/1660-4601/20/14/6377 |
  | Accessed | 11/07/2025, 09:35:36 |
  | Volume | 20 |
  | Publisher | MDPI |
  | Pages | 6377 |
  | Publication | International Journal of Environmental Research and Public Health |
  | Issue | 14 |
  | Date Added | 11/07/2025, 09:36:11 |
  | Modified | 11/07/2025, 09:36:11 |
- ## An Overview of the History, Rationale, and Clinical Application of the Use of EAS

  |  |  |
  | --- | --- |
  | Item Type | Journal Article |
  | Author | William R. Marchand |
  | Library Catalogue | Google Scholar |
  | URL | https://books.google.com/books?hl=it&lr=&id=-yhGEQAAQBAJ&oi=fnd&pg=PA25&dq=%22human+animal+interaction%22+AND+%22heart+rate%22+&ots=CW44xoQtyk&sig=ZhhsP\_pejk71f38N0IvrRBqS5gU |
  | Accessed | 11/07/2025, 11:09:10 |
  | Publisher | Springer Nature |
  | Pages | 25 |
  | Publication | Guide to Equine Assisted Therapy |
  | Date Added | 11/07/2025, 11:09:44 |
  | Modified | 11/07/2025, 11:09:44 |
- ## Effects of dairy cow temperament on milk yield: a systematic review and meta-analysis

  |  |  |
  | --- | --- |
  | Item Type | Journal Article |
  | Author | Maria G. Marçal-Pedroza |
  | Author | Maria Eugênia Andrighetto Canozzi |
  | Author | Mariana M. Campos |
  | Author | Aline C. Sant’Anna |
  | Date | 2023 |
  | Short Title | Effects of dairy cow temperament on milk yield |
  | Library Catalogue | Google Scholar |
  | URL | https://academic.oup.com/jas/article-abstract/doi/10.1093/jas/skad099/7099629 |
  | Accessed | 11/07/2025, 09:37:34 |
  | Volume | 101 |
  | Publisher | Oxford University Press US |
  | Pages | skad099 |
  | Publication | Journal of Animal Science |
  | Date Added | 11/07/2025, 09:38:03 |
  | Modified | 11/07/2025, 09:38:03 |

  ### Attachments

  - Available Version (via Google Scholar)
- ## Effects of Canine-Assisted Intervention on the Mental Health of Higher Education students: a systematic review.

  |  |  |
  | --- | --- |
  | Item Type | Journal Article |
  | Author | Karen Manville |
  | Author | Mark Coulson |
  | Author | Michele A. Mulqueen |
  | Author | Kirsty Neller |
  | Author | Caroline Searing |
  | Author | Sarah Welland |
  | Author | Gemma Reynolds |
  | Date | 2022 |
  | Short Title | Effects of Canine-Assisted Intervention on the Mental Health of Higher Education students |
  | Library Catalogue | Google Scholar |
  | URL | https://www.cabidigitallibrary.org/doi/abs/10.1079/hai.2022.0012 |
  | Accessed | 11/07/2025, 09:27:26 |
  | Publisher | CABI UK |
  | Publication | Human-Animal Interaction Bulletin |
  | Issue | 2022 |
  | Date Added | 11/07/2025, 09:27:42 |
  | Modified | 11/07/2025, 09:27:42 |

  ### Attachments

  - Available Version (via Google Scholar)
- ## Effects of Canine-Assisted Intervention on the Mental Health of Higher Education students: a systematic review.

  |  |  |
  | --- | --- |
  | Item Type | Journal Article |
  | Author | Karen Manville |
  | Author | Mark Coulson |
  | Author | Michele A. Mulqueen |
  | Author | Kirsty Neller |
  | Author | Caroline Searing |
  | Author | Sarah Welland |
  | Author | Gemma Reynolds |
  | Date | 2022 |
  | Short Title | Effects of Canine-Assisted Intervention on the Mental Health of Higher Education students |
  | Library Catalogue | Google Scholar |
  | URL | https://www.cabidigitallibrary.org/doi/abs/10.1079/hai.2022.0012 |
  | Accessed | 11/07/2025, 10:09:40 |
  | Publisher | CABI UK |
  | Publication | Human-Animal Interaction Bulletin |
  | Issue | 2022 |
  | Date Added | 11/07/2025, 10:09:48 |
  | Modified | 11/07/2025, 10:09:48 |

  ### Attachments

  - Available Version (via Google Scholar)
- ## Individual differences in temperament of domestic animals: a review of methodology

  |  |  |
  | --- | --- |
  | Item Type | Journal Article |
  | Author | X. Manteca |
  | Author | J. M. Deag |
  | Date | 1993 |
  | Short Title | Individual differences in temperament of domestic animals |
  | Library Catalogue | Google Scholar |
  | URL | https://www.cambridge.org/core/journals/animal-welfare/article/individual-differences-in-temperament-of-domestic-animals-a-review-of-methodology/5789D8C1FB304029EF2112847B41D08F |
  | Accessed | 11/07/2025, 09:29:04 |
  | Volume | 2 |
  | Publisher | Cambridge University Press |
  | Pages | 247–268 |
  | Publication | Animal Welfare |
  | Issue | 3 |
  | Date Added | 11/07/2025, 09:29:28 |
  | Modified | 11/07/2025, 09:29:28 |
- ## Mental Health Promotion in University Students: A Systematic Review of Mental Health Interventions Strategies and Implementations

  |  |  |
  | --- | --- |
  | Item Type | Journal Article |
  | Author | Lamees Mansour |
  | Date | 2024 |
  | Short Title | Mental Health Promotion in University Students |
  | Library Catalogue | Google Scholar |
  | URL | https://search.proquest.com/openview/e9c6dcbc1bafb8ae4463b9f45c8acd5d/1?pq-origsite=gscholar&cbl=2026366&diss=y |
  | Accessed | 11/07/2025, 09:38:01 |
  | Publisher | Universidade do Porto (Portugal) |
  | Publication | PQDT-Global |
  | Date Added | 11/07/2025, 09:38:03 |
  | Modified | 11/07/2025, 09:38:03 |
- ## Development of prosociality and the effects of adversity

  |  |  |
  | --- | --- |
  | Item Type | Journal Article |
  | Author | Tina Malti |
  | Author | Ruth Speidel |
  | Date | 2024 |
  | Library Catalogue | Google Scholar |
  | URL | https://www.nature.com/articles/s44159-024-00328-7 |
  | Accessed | 11/07/2025, 11:15:36 |
  | Volume | 3 |
  | Publisher | Nature Publishing Group US New York |
  | Pages | 524–535 |
  | Publication | Nature Reviews Psychology |
  | Issue | 8 |
  | Date Added | 11/07/2025, 11:15:52 |
  | Modified | 11/07/2025, 11:15:52 |
- ## Healthy nature healthy people:‘contact with nature’as an upstream health promotion intervention for populations

  |  |  |
  | --- | --- |
  | Item Type | Journal Article |
  | Author | Cecily Maller |
  | Author | Mardie Townsend |
  | Author | Anita Pryor |
  | Author | Peter Brown |
  | Author | Lawrence St Leger |
  | Date | 2006 |
  | Short Title | Healthy nature healthy people |
  | Library Catalogue | Google Scholar |
  | URL | https://academic.oup.com/heapro/article-abstract/21/1/45/646436 |
  | Accessed | 11/07/2025, 09:37:31 |
  | Volume | 21 |
  | Publisher | Oxford University Press |
  | Pages | 45–54 |
  | Publication | Health promotion international |
  | Issue | 1 |
  | Date Added | 11/07/2025, 09:38:03 |
  | Modified | 11/07/2025, 09:38:03 |

  ### Attachments

  - Available Version (via Google Scholar)
- ## Animal-assisted interventions in intensive care delirium: a literature review

  |  |  |
  | --- | --- |
  | Item Type | Journal Article |
  | Author | Jahanzeb Malik |
  | Date | 2021 |
  | Short Title | Animal-assisted interventions in intensive care delirium |
  | Library Catalogue | Google Scholar |
  | URL | https://aacnjournals.org/aacnacconline/article-abstract/32/4/391/31636 |
  | Accessed | 11/07/2025, 10:12:04 |
  | Volume | 32 |
  | Publisher | American Association of Critical Care Nurses |
  | Pages | 391–397 |
  | Publication | AACN Advanced Critical Care |
  | Issue | 4 |
  | Date Added | 11/07/2025, 10:12:39 |
  | Modified | 11/07/2025, 10:12:39 |
- ## Patient benefit of dog-assisted interventions in health care: a systematic review

  |  |  |
  | --- | --- |
  | Item Type | Journal Article |
  | Author | Martina Lundqvist |
  | Author | Per Carlsson |
  | Author | Rune Sjödahl |
  | Author | Elvar Theodorsson |
  | Author | Lars-Åke Levin |
  | Date | 2017 |
  | Short Title | Patient benefit of dog-assisted interventions in health care |
  | Library Catalogue | Google Scholar |
  | URL | https://link.springer.com/article/10.1186/s12906-017-1844-7 |
  | Accessed | 11/07/2025, 10:12:33 |
  | Volume | 17 |
  | Publisher | Springer |
  | Pages | 1–12 |
  | Publication | BMC complementary and alternative medicine |
  | Date Added | 11/07/2025, 10:12:39 |
  | Modified | 11/07/2025, 10:12:39 |
- ## Nature-Based Therapy: Its Potential as a Complementary Approach to Treating Communication Disorders

  |  |  |
  | --- | --- |
  | Item Type | Journal Article |
  | Author | Kristine Lundgren |
  | Date | 2004-05-07 |
  | Language | en |
  | Short Title | Nature-Based Therapy |
  | Library Catalogue | Crossref |
  | URL | http://www.thieme-connect.de/DOI/DOI?10.1055/s-2004-825650 |
  | Accessed | 11/07/2025, 09:32:01 |
  | Volume | 25 |
  | Publisher | Georg Thieme Verlag KG |
  | Pages | 121-131 |
  | Publication | Seminars in Speech and Language |
  | DOI | 10.1055/s-2004-825650 |
  | Issue | 02 |
  | Journal Abbr | Semin Speech Lang |
  | ISSN | 0734-0478, 1098-9056 |
  | Date Added | 11/07/2025, 09:32:09 |
  | Modified | 11/07/2025, 09:32:09 |

  ### Attachments

  - Available Version (via Google Scholar)
- ## Nature-Based Therapy: Its Potential as a Complementary Approach to Treating Communication Disorders

  |  |  |
  | --- | --- |
  | Item Type | Journal Article |
  | Author | Kristine Lundgren |
  | Date | 2004-05-07 |
  | Language | en |
  | Short Title | Nature-Based Therapy |
  | Library Catalogue | Crossref |
  | URL | http://www.thieme-connect.de/DOI/DOI?10.1055/s-2004-825650 |
  | Accessed | 11/07/2025, 10:19:55 |
  | Volume | 25 |
  | Publisher | Georg Thieme Verlag KG |
  | Pages | 121-131 |
  | Publication | Seminars in Speech and Language |
  | DOI | 10.1055/s-2004-825650 |
  | Issue | 02 |
  | Journal Abbr | Semin Speech Lang |
  | ISSN | 0734-0478, 1098-9056 |
  | Date Added | 11/07/2025, 10:20:29 |
  | Modified | 11/07/2025, 10:20:29 |

  ### Attachments

  - Available Version (via Google Scholar)
- ## Why should human-animal interactions be included in research of working equids’ welfare?

  |  |  |
  | --- | --- |
  | Item Type | Journal Article |
  | Author | Daniela Luna |
  | Author | Tamara A. Tadich |
  | Date | 2019 |
  | Library Catalogue | Google Scholar |
  | URL | https://www.mdpi.com/2076-2615/9/2/42 |
  | Accessed | 11/07/2025, 09:27:29 |
  | Volume | 9 |
  | Publisher | MDPI |
  | Pages | 42 |
  | Publication | Animals |
  | Issue | 2 |
  | Date Added | 11/07/2025, 09:27:42 |
  | Modified | 04/01/2026, 11:25:33 |

  ### Tags:

  - horse
  - animal welfare
  - heart rate
  - emotion
  - empathy
  - animal behavior
  - nonhuman
  - physiological stress
  - social interaction
  - human
  - questionnaire
  - review
  - pain
  - veterinary medicine
  - human-animal bond
  - animal care
  - animal health
  - attitude
  - Equidae
  - experience
  - livestock
  - locus of control
  - nociception
  - perception
  - risk factor
  - thinking
  - veterinarian

  ### Attachments

  - Available Version (via Google Scholar)
  - Full Text (HTML)
- ## The Heart Rate as indicator in Relation to Stress in Dairy Cows

  |  |  |
  | --- | --- |
  | Item Type | Journal Article |
  | Author | Kathryn Lovegrove |
  | Date | 2017 |
  | Library Catalogue | Google Scholar |
  | URL | https://huveta.hu/bitstream/handle/10832/2030/lovegrove\_kathryn\_2018.pdf?sequence=1 |
  | Accessed | 11/07/2025, 10:07:18 |
  | Date Added | 11/07/2025, 10:07:52 |
  | Modified | 11/07/2025, 10:07:52 |

  ### Attachments

  - Available Version (via Google Scholar)
- ## Cognitive mechanisms and neurological foundations of companion animals’ role in enhancing human psychological well-being

  |  |  |
  | --- | --- |
  | Item Type | Journal Article |
  | Author | Heng Liu |
  | Author | Jingyuan Lin |
  | Author | Wuji Lin |
  | Date | 2024 |
  | Library Catalogue | Google Scholar |
  | URL | https://www.frontiersin.org/articles/10.3389/fpsyg.2024.1354220/full |
  | Accessed | 11/07/2025, 10:00:32 |
  | Volume | 15 |
  | Publisher | Frontiers Media SA |
  | Pages | 1354220 |
  | Publication | Frontiers in Psychology |
  | Date Added | 11/07/2025, 10:00:41 |
  | Modified | 11/07/2025, 10:00:41 |

  ### Attachments

  - Available Version (via Google Scholar)
- ## Animal-based welfare indicators for dairy cows and their validity and practicality: a systematic review of the existing literature

  |  |  |
  | --- | --- |
  | Item Type | Journal Article |
  | Author | Jenny Linstädt |
  | Author | Christa Thöne-Reineke |
  | Author | Roswitha Merle |
  | Date | 2024 |
  | Short Title | Animal-based welfare indicators for dairy cows and their validity and practicality |
  | Library Catalogue | Google Scholar |
  | URL | https://www.frontiersin.org/journals/veterinary-science/articles/10.3389/fvets.2024.1429097/full |
  | Accessed | 11/07/2025, 10:09:32 |
  | Volume | 11 |
  | Publisher | Frontiers Media SA |
  | Pages | 1429097 |
  | Publication | Frontiers in Veterinary Science |
  | Date Added | 11/07/2025, 10:09:48 |
  | Modified | 11/07/2025, 10:09:48 |

  ### Attachments

  - Available Version (via Google Scholar)
- ## for dairy cows and their validity and practicality: a systematic review of the existing literature

  |  |  |
  | --- | --- |
  | Item Type | Journal Article |
  | Author | Jenny Linstädt |
  | Author | Christa Thöne-Reineke^1 |
  | Author | Roswitha Merle |
  | Date | 2024 |
  | Short Title | for dairy cows and their validity and practicality |
  | Library Catalogue | Google Scholar |
  | URL | https://books.google.com/books?hl=it&lr=&id=1yMnEQAAQBAJ&oi=fnd&pg=PA105&dq=%22human+animal+interaction%22+AND+%22cortisol%22+&ots=-vbqkcbDNk&sig=AA9MHQ7SD2fY9Z3sRs7DUScALjE |
  | Accessed | 11/07/2025, 13:45:02 |
  | Publisher | Frontiers Media SA |
  | Publication | Reviews in Animal Welfare |
  | Date Added | 11/07/2025, 13:45:12 |
  | Modified | 11/07/2025, 13:45:12 |
- ## Animal–Visitor Interactions in Zoos and Aquariums: A Systematic Review

  |  |  |
  | --- | --- |
  | Item Type | Journal Article |
  | Author | Ga-Yi Lin |
  | Author | Keith Chi Hui Ng |
  | Author | Eduardo J. Fernandez |
  | Date | 2025 |
  | Short Title | Animal–Visitor Interactions in Zoos and Aquariums |
  | Library Catalogue | Google Scholar |
  | URL | https://www.mdpi.com/2076-2615/15/13/1924 |
  | Accessed | 11/07/2025, 09:28:53 |
  | Volume | 15 |
  | Publisher | MDPI |
  | Pages | 1924 |
  | Publication | Animals |
  | Issue | 13 |
  | Date Added | 11/07/2025, 09:29:28 |
  | Modified | 11/07/2025, 09:29:28 |
- ## Equine-assisted interventions for veterans with posttraumatic stress disorder: a systematic review

  |  |  |
  | --- | --- |
  | Item Type | Journal Article |
  | Author | Jiaxin Li |
  | Author | Raúl Sánchez-García |
  | Date | 2023 |
  | Short Title | Equine-assisted interventions for veterans with posttraumatic stress disorder |
  | Library Catalogue | Google Scholar |
  | URL | https://www.frontiersin.org/articles/10.3389/fpsyt.2023.1277338/full |
  | Accessed | 11/07/2025, 10:20:25 |
  | Volume | 14 |
  | Publisher | Frontiers Media SA |
  | Pages | 1277338 |
  | Publication | Frontiers in psychiatry |
  | Date Added | 11/07/2025, 10:20:29 |
  | Modified | 11/07/2025, 10:20:29 |

  ### Attachments

  - Available Version (via Google Scholar)
- ## A non‐adaptationist hypothesis of play behaviour

  |  |  |
  | --- | --- |
  | Item Type | Journal Article |
  | Author | Amelia Lewis |
  | Abstract | AbstractPlay is a suite of apparently non‐functional, pleasurable behaviours observed in human and non‐human animals. Although the phenomenon has been studied extensively, no adaptationist behavioural theory of how play evolved can be supported by the available evidence. However, the advancement of the extended evolutionary synthesis and developments in systems biology offer alternative avenues for non‐adaptationist physiological hypotheses. I therefore propose a hypothesis of play, based upon a complex ACh activity that is under agential control of the organism, whereby play initiates ACh‐mediated feedforward and feedback processes which act to: (i) regulate metabolic processes; (ii) form new ACh receptors via ACh mRNA activity; (iii) mediate attention, memory consolidation and learning; and (iv) mediate social behaviours, reproduction and embryonic development. However, play occurs across taxa, but does not occur across all taxonomic groups or within all species of a taxonomic group. Thus, to support the validity of the proposed hypothesis, I further propose potential explanations for this anomaly, which include sampling and observer biases, altricial versus precocial juvenile development, and the influence of habitat niche and environmental conditions on behaviour. The proposed hypothesis thus offers new avenues for study in both the biological and social sciences, in addition to having potential applications in applied sciences, such as animal welfare and biomedical research. Crucially, it is hoped that this hypothesis will promote further study of a valid and behaviourally significant, yet currently enigmatic, biological phenomenon.image |
  | Date | 06/2024 |
  | Language | en |
  | Library Catalogue | Crossref |
  | URL | https://physoc.onlinelibrary.wiley.com/doi/10.1113/JP284413 |
  | Accessed | 11/07/2025, 10:16:43 |
  | Rights | http://onlinelibrary.wiley.com/termsAndConditions#vor |
  | Volume | 602 |
  | Publisher | Wiley |
  | Pages | 2433-2453 |
  | Publication | The Journal of Physiology |
  | DOI | 10.1113/jp284413 |
  | Issue | 11 |
  | ISSN | 0022-3751, 1469-7793 |
  | Date Added | 11/07/2025, 10:16:47 |
  | Modified | 11/07/2025, 10:16:47 |
- ## Equine-Facilitated Psychotherapy With Children and Adolescents: An Update and Literature Review

  |  |  |
  | --- | --- |
  | Item Type | Journal Article |
  | Author | Jennifer A. Lentini |
  | Author | Michele S. Knox |
  | Date | 2015-07-03 |
  | Language | en |
  | Short Title | Equine-Facilitated Psychotherapy With Children and Adolescents |
  | Library Catalogue | Crossref |
  | URL | http://www.tandfonline.com/doi/full/10.1080/15401383.2015.1023916 |
  | Accessed | 11/07/2025, 09:31:53 |
  | Volume | 10 |
  | Publisher | Informa UK Limited |
  | Pages | 278-305 |
  | Publication | Journal of Creativity in Mental Health |
  | DOI | 10.1080/15401383.2015.1023916 |
  | Issue | 3 |
  | ISSN | 1540-1383, 1540-1391 |
  | Date Added | 11/07/2025, 09:32:09 |
  | Modified | 11/07/2025, 09:32:09 |
- ## A text-mining analysis of research trends in animal-assisted therapy

  |  |  |
  | --- | --- |
  | Item Type | Journal Article |
  | Author | Shin-Ja Lee |
  | Author | Geun-Hyeon Kim |
  | Author | Yea-Hwang Moon |
  | Author | Sung-Sill Lee |
  | Date | 2023 |
  | Library Catalogue | Google Scholar |
  | URL | https://www.mdpi.com/2076-2615/13/19/3133 |
  | Accessed | 11/07/2025, 10:09:48 |
  | Volume | 13 |
  | Publisher | MDPI |
  | Pages | 3133 |
  | Publication | Animals |
  | Issue | 19 |
  | Date Added | 11/07/2025, 10:09:48 |
  | Modified | 11/07/2025, 10:09:48 |
- ## Improving cattle welfare: an evidence-based approach.

  |  |  |
  | --- | --- |
  | Item Type | Journal Article |
  | Author | T. L. Lee |
  | Author | D. U. Thomson |
  | Abstract | Abstract Animal welfare is a growing scientific field that is constantly changing and improving. The importance of this field is becoming more evident in all production agriculture settings, including cattle and dairy production units. In 2007, Fraser defined animal welfare as a 'mandatory science', one in which science has been used to guide actions, decisions and policy. While values-based ideas are an integral part of the idea of animal welfare, science-based ideas and the scientific method must be used to assess and improve animal welfare in our production units. Animal welfare practices must advance from the laboratory to the field. In this light, animal welfare outcome measures must be defined to allow people to measure and assess animal welfare in beef and dairy production units daily. This review focuses on defining the outcome-based measures that can be used in the cattle industry to increase our understanding of animal health and well-being. This paper will give farmers, ranchers and veterinarians a framework to determine if changes in management or other husbandry practices improve or compromise cattle health or welfare. |
  | Date | 2015-10-14 |
  | Language | en |
  | Short Title | Improving cattle welfare |
  | Library Catalogue | Crossref |
  | URL | http://www.cabidigitallibrary.org/doi/10.1079/PAVSNNR201510022 |
  | Accessed | 11/07/2025, 11:14:21 |
  | Publisher | CABI Publishing |
  | Pages | 1-17 |
  | Publication | CABI Reviews |
  | DOI | 10.1079/pavsnnr201510022 |
  | ISSN | 1749-8848 |
  | Date Added | 11/07/2025, 11:14:45 |
  | Modified | 11/07/2025, 11:14:45 |
- ## Behavioural Synchronisation between Dogs and Humans: Unveiling Interspecific Motor Resonance?

  |  |  |
  | --- | --- |
  | Item Type | Journal Article |
  | Author | Angélique Lamontagne |
  | Author | Florence Gaunet |
  | Date | 2024 |
  | Short Title | Behavioural Synchronisation between Dogs and Humans |
  | Library Catalogue | Google Scholar |
  | URL | https://www.mdpi.com/2076-2615/14/4/548 |
  | Accessed | 11/07/2025, 10:00:25 |
  | Volume | 14 |
  | Publisher | MDPI |
  | Pages | 548 |
  | Publication | Animals |
  | Issue | 4 |
  | Date Added | 11/07/2025, 10:00:41 |
  | Modified | 11/07/2025, 10:00:41 |

  ### Attachments

  - Available Version (via Google Scholar)
- ## Dairy cattle welfare assessment models for dairy farms: A review

  |  |  |
  | --- | --- |
  | Item Type | Journal Article |
  | Author | Rupendra Kumar |
  | Author | C. P. Ghosh |
  | Author | Sanjoy Datta |
  | Author | Anand Kumar Yadav |
  | Date | 2024 |
  | Short Title | Dairy cattle welfare assessment models for dairy farms |
  | Library Catalogue | Google Scholar |
  | URL | https://www.researchgate.net/profile/Chittapriya-Ghosh/publication/382829863\_Dairy\_cattle\_welfare\_assessment\_models\_for\_dairy\_farms\_A\_review/links/66addcf4299c327096a7a466/Dairy-cattle-welfare-assessment-models-for-dairy-farms-A-review.pdf |
  | Accessed | 11/07/2025, 09:35:55 |
  | Date Added | 11/07/2025, 09:36:11 |
  | Modified | 11/07/2025, 09:36:11 |
- ## Emotional studies in dogs and cats and their estimation techniques: an engineering perspective

  |  |  |
  | --- | --- |
  | Item Type | Journal Article |
  | Author | Hikari Koyasu |
  | Author | Nanako Miyai |
  | Author | Takatomi Kubo |
  | Author | Saho Takagi |
  | Author | Yurina Wada |
  | Author | Yuki Maruno |
  | Author | Miho Nagasawa |
  | Author | Kazunori Ohno |
  | Date | 2024-07-17 |
  | Language | en |
  | Short Title | Emotional studies in dogs and cats and their estimation techniques |
  | Library Catalogue | Crossref |
  | URL | https://www.tandfonline.com/doi/full/10.1080/01691864.2024.2358439 |
  | Accessed | 11/07/2025, 13:43:20 |
  | Rights | http://creativecommons.org/licenses/by-nc-nd/4.0/ |
  | Volume | 38 |
  | Publisher | Informa UK Limited |
  | Pages | 908-925 |
  | Publication | Advanced Robotics |
  | DOI | 10.1080/01691864.2024.2358439 |
  | Issue | 14 |
  | ISSN | 0169-1864, 1568-5535 |
  | Date Added | 11/07/2025, 13:43:25 |
  | Modified | 11/07/2025, 13:43:25 |

  ### Attachments

  - Available Version (via Google Scholar)
- ## Emotional studies in dogs and cats and their estimation techniques: an engineering perspective

  |  |  |
  | --- | --- |
  | Item Type | Journal Article |
  | Author | Hikari Koyasu |
  | Author | Nanako Miyai |
  | Author | Takatomi Kubo |
  | Author | Saho Takagi |
  | Author | Yurina Wada |
  | Author | Yuki Maruno |
  | Author | Miho Nagasawa |
  | Author | Kazunori Ohno |
  | Date | 2024-07-17 |
  | Language | en |
  | Short Title | Emotional studies in dogs and cats and their estimation techniques |
  | Library Catalogue | Crossref |
  | URL | https://www.tandfonline.com/doi/full/10.1080/01691864.2024.2358439 |
  | Accessed | 11/07/2025, 13:46:10 |
  | Rights | http://creativecommons.org/licenses/by-nc-nd/4.0/ |
  | Volume | 38 |
  | Publisher | Informa UK Limited |
  | Pages | 908-925 |
  | Publication | Advanced Robotics |
  | DOI | 10.1080/01691864.2024.2358439 |
  | Issue | 14 |
  | ISSN | 0169-1864, 1568-5535 |
  | Date Added | 11/07/2025, 13:46:33 |
  | Modified | 11/07/2025, 13:46:33 |

  ### Attachments

  - Available Version (via Google Scholar)
- ## Welfare implication of measuring heart rate and heart rate variability in dairy cattle: literature review and conclusions for future research

  |  |  |
  | --- | --- |
  | Item Type | Journal Article |
  | Author | L. Kovács |
  | Author | V. Jurkovich |
  | Author | M. Bakony |
  | Author | O. Szenci |
  | Author | P. Póti |
  | Author | J. T\Hozsér |
  | Date | 2014 |
  | Short Title | Welfare implication of measuring heart rate and heart rate variability in dairy cattle |
  | Library Catalogue | Google Scholar |
  | URL | https://www.cambridge.org/core/journals/animal/article/welfare-implication-of-measuring-heart-rate-and-heart-rate-variability-in-dairy-cattle-literature-review-and-conclusions-for-future-research/E5B958B7C4C6F4C26ADC00E82AEB3270 |
  | Accessed | 11/07/2025, 09:31:27 |
  | Volume | 8 |
  | Publisher | Cambridge University Press |
  | Pages | 316–330 |
  | Publication | animal |
  | Issue | 2 |
  | Date Added | 11/07/2025, 09:32:09 |
  | Modified | 04/01/2026, 11:25:45 |

  ### Attachments

  - Available Version (via Google Scholar)
  - Available Version (via Google Scholar)
- ## Psychodynamic based equine—assisted psychotherapy in adults with intertwined personality problems and traumatization: A systematic review

  |  |  |
  | --- | --- |
  | Item Type | Journal Article |
  | Author | Géza Kovács |
  | Author | Annemiek Van Dijke |
  | Author | Marie-Jose Enders-Slegers |
  | Date | 2020 |
  | Short Title | Psychodynamic based equine—assisted psychotherapy in adults with intertwined personality problems and traumatization |
  | Library Catalogue | Google Scholar |
  | URL | https://www.mdpi.com/1660-4601/17/16/5661 |
  | Accessed | 11/07/2025, 11:01:30 |
  | Volume | 17 |
  | Publisher | MDPI |
  | Pages | 5661 |
  | Publication | International journal of environmental research and public health |
  | Issue | 16 |
  | Date Added | 11/07/2025, 11:01:44 |
  | Modified | 11/07/2025, 11:01:44 |
- ## Wolf–Dog–Human: Companionship Based on Common Social Tools

  |  |  |
  | --- | --- |
  | Item Type | Journal Article |
  | Author | Kurt Kotrschal |
  | Date | 2023 |
  | Short Title | Wolf–Dog–Human |
  | Library Catalogue | Google Scholar |
  | URL | https://www.mdpi.com/2076-2615/13/17/2729 |
  | Accessed | 11/07/2025, 10:17:53 |
  | Volume | 13 |
  | Publisher | MDPI |
  | Pages | 2729 |
  | Publication | Animals |
  | Issue | 17 |
  | Date Added | 11/07/2025, 10:18:17 |
  | Modified | 11/07/2025, 10:18:17 |

  ### Attachments

  - Available Version (via Google Scholar)
- ## Assessing and influencing personality for improvement of animal welfare: a review of equine studies.

  |  |  |
  | --- | --- |
  | Item Type | Journal Article |
  | Author | U. König V. Borstel |
  | Abstract | Abstract Personality and its various sub-traits such as temperament can be defined as behavioural attitudes that remain relatively stable across time and situations. However, various personality models exist. Contrasting their strengths and weaknesses leads to the conclusion that there is presently no model that is truly superior to others in depicting animal personality. Furthermore, the present review highlights aspects in which animal, and in particular equine personality potentially relate to animal welfare. Three approaches are examined which may be taken to improve welfare: (1) genetically selecting for personality traits that make the horses better adapted to their designated work and/or husbandry conditions, (2) assessing personality in individual horses to optimize matching with owners and type of work and (3) influencing the ontogeny of personality traits, e.g. by specific training and husbandry regimes. Each of these strategies has its merit, but as a prerequisite, valid personality assessment methods are required. Although publication bias is likely present, most testing procedures yield acceptable inter-observer reliabilities and repeatabilities across time, even if repeatability across situations tends to be comparably low. Heritability estimates ranging mostly between h 2 =0.15 and h 2 =0.40 for traits assessed in personality tests are likewise promising. Research has paid less attention to intra-observer reliability, construct validity or discriminant ability, but based on pleiotropy it is argued that the latter is not essential for a trait to be valid. Therefore, some valid methods are available for use in assessment and subsequent selection of horses or personality-influencing strategies, ultimate resulting in reduction of stress in horses' everyday life. |
  | Date | 2013-02-06 |
  | Language | en |
  | Short Title | Assessing and influencing personality for improvement of animal welfare |
  | Library Catalogue | Crossref |
  | URL | http://www.cabidigitallibrary.org/doi/10.1079/PAVSNNR20138006 |
  | Accessed | 11/07/2025, 09:55:04 |
  | Publisher | CABI Publishing |
  | Pages | 1-27 |
  | Publication | CABI Reviews |
  | DOI | 10.1079/pavsnnr20138006 |
  | ISSN | 1749-8848 |
  | Date Added | 11/07/2025, 09:55:10 |
  | Modified | 11/07/2025, 09:55:12 |
- ## Assessing and influencing personality for improvement of animal welfare: a review of equine studies.

  |  |  |
  | --- | --- |
  | Item Type | Journal Article |
  | Author | U. König V. Borstel |
  | Abstract | Abstract Personality and its various sub-traits such as temperament can be defined as behavioural attitudes that remain relatively stable across time and situations. However, various personality models exist. Contrasting their strengths and weaknesses leads to the conclusion that there is presently no model that is truly superior to others in depicting animal personality. Furthermore, the present review highlights aspects in which animal, and in particular equine personality potentially relate to animal welfare. Three approaches are examined which may be taken to improve welfare: (1) genetically selecting for personality traits that make the horses better adapted to their designated work and/or husbandry conditions, (2) assessing personality in individual horses to optimize matching with owners and type of work and (3) influencing the ontogeny of personality traits, e.g. by specific training and husbandry regimes. Each of these strategies has its merit, but as a prerequisite, valid personality assessment methods are required. Although publication bias is likely present, most testing procedures yield acceptable inter-observer reliabilities and repeatabilities across time, even if repeatability across situations tends to be comparably low. Heritability estimates ranging mostly between h 2 =0.15 and h 2 =0.40 for traits assessed in personality tests are likewise promising. Research has paid less attention to intra-observer reliability, construct validity or discriminant ability, but based on pleiotropy it is argued that the latter is not essential for a trait to be valid. Therefore, some valid methods are available for use in assessment and subsequent selection of horses or personality-influencing strategies, ultimate resulting in reduction of stress in horses' everyday life. |
  | Date | 2013-02-06 |
  | Language | en |
  | Short Title | Assessing and influencing personality for improvement of animal welfare |
  | Library Catalogue | Crossref |
  | URL | http://www.cabidigitallibrary.org/doi/10.1079/PAVSNNR20138006 |
  | Accessed | 11/07/2025, 11:08:18 |
  | Publisher | CABI Publishing |
  | Pages | 1-27 |
  | Publication | CABI Reviews |
  | DOI | 10.1079/pavsnnr20138006 |
  | ISSN | 1749-8848 |
  | Date Added | 11/07/2025, 11:08:27 |
  | Modified | 11/07/2025, 11:08:27 |
- ## READING IN EFL IN THE COMPANY OF THERAPY DOGS: LITERATURE REVIEW-ČITANJE NA ENGLESKOM KAO STRANOM JEZIKU U DRUŠTVU TERAPIJSKIH PASA: PREGLED LITERATURE

  |  |  |
  | --- | --- |
  | Item Type | Journal Article |
  | Author | Anja Kolarić |
  | Short Title | READING IN EFL IN THE COMPANY OF THERAPY DOGS |
  | Library Catalogue | Google Scholar |
  | URL | https://repozitorij.ufzg.unizg.hr/islandora/object/ufzg:5174/datastream/PDF/view |
  | Accessed | 11/07/2025, 13:44:50 |
  | Date Added | 11/07/2025, 13:45:12 |
  | Modified | 11/07/2025, 13:45:12 |
- ## Reading in the EFL in the company of therapy dogs: literature review

  |  |  |
  | --- | --- |
  | Item Type | Journal Article |
  | Author | Anja Kolarić |
  | Date | 2024 |
  | Short Title | Reading in the EFL in the company of therapy dogs |
  | Library Catalogue | Google Scholar |
  | URL | https://zir.nsk.hr/islandora/object/ufzg:5174 |
  | Accessed | 11/07/2025, 13:46:27 |
  | Publisher | University of Zagreb. Faculty of Teacher Education |
  | Date Added | 11/07/2025, 13:46:33 |
  | Modified | 11/07/2025, 13:46:33 |
- ## Social buffering: relief from stress and anxiety

  |  |  |
  | --- | --- |
  | Item Type | Journal Article |
  | Author | Takefumi Kikusui |
  | Author | James T Winslow |
  | Author | Yuji Mori |
  | Abstract | Communication is essential to members of a society not only for the expression of personal information, but also for the protection from environmental threats. Highly social mammals have a distinct characteristic: when conspecific animals are together, they show a better recovery from experiences of distress. This phenomenon, termed ‘social buffering’, has been found in rodents, birds, non-human primates and also in humans. This paper reviews classical findings on social buffering and focuses, in particular, on social buffering effects in relation to neuroendocrine stress responses. The social cues that transmit social buffering signals, the neural mechanisms of social buffering and a partner's efficacy with respect to social buffering are also detailed. Social contact appears to have a very positive influence on the psychological and the physiological aspects of social animals, including human beings. Research leading towards further understanding of the mechanisms of social buffering could provide alternative medical treatments based on the natural, individual characteristics of social animals, which could improve the quality of life. |
  | Date | 2006-12-29 |
  | Language | en |
  | Short Title | Social buffering |
  | Library Catalogue | Crossref |
  | URL | https://royalsocietypublishing.org/doi/10.1098/rstb.2006.1941 |
  | Accessed | 11/07/2025, 11:09:24 |
  | Rights | https://royalsociety.org/journals/ethics-policies/data-sharing-mining/ |
  | Volume | 361 |
  | Publisher | The Royal Society |
  | Pages | 2215-2228 |
  | Publication | Philosophical Transactions of the Royal Society B: Biological Sciences |
  | DOI | 10.1098/rstb.2006.1941 |
  | Issue | 1476 |
  | Journal Abbr | Phil. Trans. R. Soc. B |
  | ISSN | 0962-8436, 1471-2970 |
  | Date Added | 11/07/2025, 11:09:44 |
  | Modified | 11/07/2025, 11:09:44 |

  ### Attachments

  - Available Version (via Google Scholar)
- ## Interspecies Relational Theory: A Framework for Compassionate Interspecies Interactions

  |  |  |
  | --- | --- |
  | Item Type | Journal Article |
  | Author | Emily Kieson |
  | Date | 2025 |
  | Short Title | Interspecies Relational Theory |
  | Library Catalogue | Google Scholar |
  | URL | https://www.mdpi.com/2306-7381/12/6/586 |
  | Accessed | 11/07/2025, 09:29:26 |
  | Volume | 12 |
  | Publisher | MDPI |
  | Pages | 586 |
  | Publication | Veterinary Sciences |
  | Issue | 6 |
  | Date Added | 11/07/2025, 09:29:28 |
  | Modified | 11/07/2025, 09:29:28 |
- ## The relationship between canine behavioral disorders and gut microbiome and future therapeutic perspectives

  |  |  |
  | --- | --- |
  | Item Type | Journal Article |
  | Author | Paula Kiełbik |
  | Author | Olga Witkowska-Piłaszewicz |
  | Date | 2024 |
  | Library Catalogue | Google Scholar |
  | URL | https://www.mdpi.com/2076-2615/14/14/2048 |
  | Accessed | 11/07/2025, 11:09:14 |
  | Volume | 14 |
  | Publisher | MDPI |
  | Pages | 2048 |
  | Publication | Animals |
  | Issue | 14 |
  | Date Added | 11/07/2025, 11:09:44 |
  | Modified | 11/07/2025, 11:09:44 |
- ## The relationship between canine behavioral disorders and gut microbiome and future therapeutic perspectives

  |  |  |
  | --- | --- |
  | Item Type | Journal Article |
  | Author | Paula Kiełbik |
  | Author | Olga Witkowska-Piłaszewicz |
  | Date | 2024 |
  | Library Catalogue | Google Scholar |
  | URL | https://www.mdpi.com/2076-2615/14/14/2048 |
  | Accessed | 11/07/2025, 13:46:29 |
  | Volume | 14 |
  | Publisher | MDPI |
  | Pages | 2048 |
  | Publication | Animals |
  | Issue | 14 |
  | Date Added | 11/07/2025, 13:46:33 |
  | Modified | 11/07/2025, 13:46:33 |
- ## Evolution of research on human–animal interaction: A review

  |  |  |
  | --- | --- |
  | Item Type | Journal Article |
  | Author | Vivekswar Khandai |
  | Author | Pragya Shrivastava |
  | Date | 2023 |
  | Short Title | Evolution of research on human–animal interaction |
  | Library Catalogue | Google Scholar |
  | URL | https://www.academia.edu/download/100214445/11\_2\_9\_806.pdf |
  | Accessed | 11/07/2025, 10:05:27 |
  | Date Added | 11/07/2025, 10:05:27 |
  | Modified | 11/07/2025, 10:05:27 |

  ### Attachments

  - Available Version (via Google Scholar)
- ## The effect of human–horse interactions on equine behaviour, physiology, and welfare: A scoping review

  |  |  |
  | --- | --- |
  | Item Type | Journal Article |
  | Author | Katherine Jennifer Kelly |
  | Author | Laurie Anne McDuffee |
  | Author | Kimberly Mears |
  | Date | 2021 |
  | Short Title | The effect of human–horse interactions on equine behaviour, physiology, and welfare |
  | Library Catalogue | Google Scholar |
  | URL | https://www.mdpi.com/2076-2615/11/10/2782?trk=public\_post\_share-update\_update-text |
  | Accessed | 11/07/2025, 09:27:12 |
  | Volume | 11 |
  | Publisher | Multidisciplinary Digital Publishing Institute |
  | Pages | 2782 |
  | Publication | Animals |
  | Issue | 10 |
  | Date Added | 11/07/2025, 09:27:42 |
  | Modified | 11/07/2025, 09:27:42 |
- ## The effect of human–horse interactions on equine behaviour, physiology, and welfare: A scoping review

  |  |  |
  | --- | --- |
  | Item Type | Journal Article |
  | Author | Katherine Jennifer Kelly |
  | Author | Laurie Anne McDuffee |
  | Author | Kimberly Mears |
  | Date | 2021 |
  | Short Title | The effect of human–horse interactions on equine behaviour, physiology, and welfare |
  | Library Catalogue | Google Scholar |
  | URL | https://www.mdpi.com/2076-2615/11/10/2782?trk=public\_post\_share-update\_update-text |
  | Accessed | 11/07/2025, 10:05:13 |
  | Volume | 11 |
  | Publisher | Multidisciplinary Digital Publishing Institute |
  | Pages | 2782 |
  | Publication | Animals |
  | Issue | 10 |
  | Date Added | 11/07/2025, 10:05:27 |
  | Modified | 11/07/2025, 10:05:27 |
- ## Effects and Nursing Considerations for Equine-Assisted Activities and Therapies for Children with Autism Spectrum Disorders: A Literature Review

  |  |  |
  | --- | --- |
  | Item Type | Journal Article |
  | Author | Namiko Kawamura |
  | Author | Mayu Sakamoto |
  | Author | Kayoko Machida |
  | Date | 2024 |
  | Short Title | Effects and Nursing Considerations for Equine-Assisted Activities and Therapies for Children with Autism Spectrum Disorders |
  | Library Catalogue | Google Scholar |
  | URL | https://docs.lib.purdue.edu/paij/vol7/iss1/9/ |
  | Accessed | 11/07/2025, 13:43:55 |
  | Volume | 7 |
  | Pages | 9 |
  | Publication | People and Animals: The International Journal of Research and Practice |
  | Issue | 1 |
  | Date Added | 11/07/2025, 13:44:05 |
  | Modified | 11/07/2025, 13:44:05 |

  ### Attachments

  - Available Version (via Google Scholar)
  - Available Version (via Google Scholar)
- ## An Insight into the Challenges to Welfare of Small Ruminants in India and their Mitigation Strategies

  |  |  |
  | --- | --- |
  | Item Type | Journal Article |
  | Author | Garima Kansal |
  | Author | Arun Kumar Misra |
  | Library Catalogue | Google Scholar |
  | URL | https://epubs.icar.org.in/index.php/IJAPM/issue/download/4199/1338#page=104 |
  | Accessed | 11/07/2025, 11:14:24 |
  | Volume | 100 |
  | Pages | 95 |
  | Publication | Behaviour and Welfare of Native Livestock Species |
  | Issue | 3000.00 |
  | Date Added | 11/07/2025, 11:14:45 |
  | Modified | 11/07/2025, 11:14:45 |
- ## An Insight into the Challenges to Welfare of Small Ruminants in India and their Mitigation Strategies

  |  |  |
  | --- | --- |
  | Item Type | Journal Article |
  | Author | Garima Kansal |
  | Author | Arun Kumar Misra |
  | Library Catalogue | Google Scholar |
  | URL | https://epubs.icar.org.in/index.php/IJAPM/issue/download/4199/1338#page=104 |
  | Accessed | 11/07/2025, 13:44:05 |
  | Volume | 100 |
  | Pages | 95 |
  | Publication | Behaviour and Welfare of Native Livestock Species |
  | Issue | 3000.00 |
  | Date Added | 11/07/2025, 13:44:05 |
  | Modified | 11/07/2025, 13:44:05 |
- ## Understanding the Influence of Companion Animal on Child Development: a Literature Review

  |  |  |
  | --- | --- |
  | Item Type | Journal Article |
  | Author | Risa Juliadilla |
  | Author | Nia Anggri Noveni |
  | Date | 2021 |
  | Short Title | Understanding the Influence of Companion Animal on Child Development |
  | Library Catalogue | Google Scholar |
  | URL | https://www.academia.edu/download/106849700/1350-8278-4-PB.pdf |
  | Accessed | 11/07/2025, 10:09:30 |
  | Volume | 6 |
  | Pages | 1493–1500 |
  | Publication | Jurnal Obsesi: Jurnal Pendidikan Anak Usia Dini |
  | Issue | 3 |
  | Date Added | 11/07/2025, 10:09:48 |
  | Modified | 11/07/2025, 10:09:48 |

  ### Attachments

  - Available Version (via Google Scholar)
- ## Smart computing and sensing technologies for animal welfare: A systematic review

  |  |  |
  | --- | --- |
  | Item Type | Journal Article |
  | Author | Admela Jukan |
  | Author | Xavi Masip-Bruin |
  | Author | Nina Amla |
  | Date | 2017 |
  | Short Title | Smart computing and sensing technologies for animal welfare |
  | Library Catalogue | Google Scholar |
  | URL | https://dl.acm.org/doi/abs/10.1145/3041960 |
  | Accessed | 11/07/2025, 09:54:55 |
  | Volume | 50 |
  | Publisher | ACM New York, NY, USA |
  | Pages | 1–27 |
  | Publication | ACM Computing Surveys (CSUR) |
  | Issue | 1 |
  | Date Added | 11/07/2025, 09:55:10 |
  | Modified | 11/07/2025, 09:55:11 |

  ### Attachments

  - Available Version (via Google Scholar)
- ## Smart computing and sensing technologies for animal welfare: A systematic review

  |  |  |
  | --- | --- |
  | Item Type | Journal Article |
  | Author | Admela Jukan |
  | Author | Xavi Masip-Bruin |
  | Author | Nina Amla |
  | Date | 2017 |
  | Short Title | Smart computing and sensing technologies for animal welfare |
  | Library Catalogue | Google Scholar |
  | URL | https://dl.acm.org/doi/abs/10.1145/3041960 |
  | Accessed | 11/07/2025, 10:18:11 |
  | Volume | 50 |
  | Publisher | ACM New York, NY, USA |
  | Pages | 1–27 |
  | Publication | ACM Computing Surveys (CSUR) |
  | Issue | 1 |
  | Date Added | 11/07/2025, 10:18:17 |
  | Modified | 11/07/2025, 10:18:17 |

  ### Attachments

  - Available Version (via Google Scholar)
- ## 10 Impacts of climate change on

  |  |  |
  | --- | --- |
  | Item Type | Journal Article |
  | Author | Joe Joseph |
  | Author | Renae Charalambous |
  | Author | Harsh Pahuja |
  | Author | Dylan Fox |
  | Author | Jiwoo Jeon |
  | Author | Ning-Yuan Ko |
  | Author | Nishit Rao |
  | Author | Zhiheng Wang |
  | Author | Sneh Kashinath Nerurkar |
  | Author | Sharvari Avinash Sherekar |
  | Date | 2024 |
  | Library Catalogue | Google Scholar |
  | URL | https://books.google.com/books?hl=it&lr=&id=VelDEQAAQBAJ&oi=fnd&pg=PA169&dq=%22human+animal+interaction%22+AND+%22cortisol%22+&ots=db6gseUF-K&sig=7cx4LJGrS7515WVoNpgRJlc5W0c |
  | Accessed | 11/07/2025, 13:46:07 |
  | Publisher | CABI |
  | Pages | 169 |
  | Publication | Climate Change Reviews: 2022-2024 |
  | Date Added | 11/07/2025, 13:46:33 |
  | Modified | 11/07/2025, 13:46:33 |
- ## Fear and adaptability in poultry: insights, implications and imperatives

  |  |  |
  | --- | --- |
  | Item Type | Journal Article |
  | Author | R. Bryan Jones |
  | Date | 1996 |
  | Short Title | Fear and adaptability in poultry |
  | Library Catalogue | Google Scholar |
  | URL | https://www.cambridge.org/core/journals/world-s-poultry-science-journal/article/fear-and-adaptability-in-poultry-insights-implications-and-imperatives/83930B9CA98066660057580547508705 |
  | Accessed | 11/07/2025, 09:54:52 |
  | Volume | 52 |
  | Publisher | Cambridge University Press on behalf of World's Poultry Science Association |
  | Pages | 131–174 |
  | Publication | World's Poultry Science Journal |
  | Issue | 2 |
  | Date Added | 11/07/2025, 09:55:10 |
  | Modified | 11/07/2025, 09:55:11 |
- ## Fear and adaptability in poultry: insights, implications and imperatives

  |  |  |
  | --- | --- |
  | Item Type | Journal Article |
  | Author | R. Bryan Jones |
  | Date | 1996 |
  | Short Title | Fear and adaptability in poultry |
  | Library Catalogue | Google Scholar |
  | URL | https://www.cambridge.org/core/journals/world-s-poultry-science-journal/article/fear-and-adaptability-in-poultry-insights-implications-and-imperatives/83930B9CA98066660057580547508705 |
  | Accessed | 11/07/2025, 11:01:39 |
  | Volume | 52 |
  | Publisher | Cambridge University Press on behalf of World's Poultry Science Association |
  | Pages | 131–174 |
  | Publication | World's Poultry Science Journal |
  | Issue | 2 |
  | Date Added | 11/07/2025, 11:01:44 |
  | Modified | 11/07/2025, 11:01:44 |
- ## Canines and childhood cancer. Examining the effects of therapy dogs with childhood cancer patients and their families: literature review.

  |  |  |
  | --- | --- |
  | Item Type | Journal Article |
  | Author | M. Jenkins |
  | Author | A. Ruchrdanz |
  | Author | A. McCullough |
  | Author | K. Casillas |
  | Author | J. D. Fluke |
  | Date | 2012 |
  | Short Title | Canines and childhood cancer. Examining the effects of therapy dogs with childhood cancer patients and their families |
  | Library Catalogue | Google Scholar |
  | URL | https://www.cabidigitallibrary.org/doi/full/10.5555/20123107901 |
  | Accessed | 11/07/2025, 09:36:01 |
  | Date Added | 11/07/2025, 09:36:11 |
  | Modified | 11/07/2025, 09:36:11 |
- ## Canines and childhood cancer. Examining the effects of therapy dogs with childhood cancer patients and their families: literature review.

  |  |  |
  | --- | --- |
  | Item Type | Journal Article |
  | Author | M. Jenkins |
  | Author | A. Ruchrdanz |
  | Author | A. McCullough |
  | Author | K. Casillas |
  | Author | J. D. Fluke |
  | Date | 2012 |
  | Short Title | Canines and childhood cancer. Examining the effects of therapy dogs with childhood cancer patients and their families |
  | Library Catalogue | Google Scholar |
  | URL | https://www.cabidigitallibrary.org/doi/full/10.5555/20123107901 |
  | Accessed | 11/07/2025, 10:19:53 |
  | Date Added | 11/07/2025, 10:20:29 |
  | Modified | 11/07/2025, 10:20:29 |

  ### Attachments

  - Available Version (via Google Scholar)
- ## The effectiveness of therapy dogs in a South African clinical context: a critical review

  |  |  |
  | --- | --- |
  | Item Type | Journal Article |
  | Author | Francis E. Jansen van Rensburg |
  | Date | 2021 |
  | Short Title | The effectiveness of therapy dogs in a South African clinical context |
  | Library Catalogue | Google Scholar |
  | Publisher | North-West University (South-Africa) |
  | Date Added | 11/07/2025, 11:12:01 |
  | Modified | 11/07/2025, 11:12:01 |
- ## Cat and dog companionship and well-being: A systematic review

  |  |  |
  | --- | --- |
  | Item Type | Journal Article |
  | Author | Azharul Islam |
  | Author | Tony Towell |
  | Date | 2013 |
  | Short Title | Cat and dog companionship and well-being |
  | Library Catalogue | Google Scholar |
  | URL | https://www.academia.edu/download/96641497/showpaperpdf.pdf |
  | Accessed | 11/07/2025, 10:12:16 |
  | Volume | 3 |
  | Pages | 149–155 |
  | Publication | Int. J. Appl. Psychol |
  | Date Added | 11/07/2025, 10:12:39 |
  | Modified | 11/07/2025, 10:12:39 |
- ## Impact of procedures and human-animal interactions during transport and slaughter on animal welfare of pigs: a systematic literature review

  |  |  |
  | --- | --- |
  | Item Type | Journal Article |
  | Author | Rudi Isbrandt |
  | Author | Mechthild Wiegard |
  | Author | Diana Meemken |
  | Author | Nina Langkabel |
  | Date | 2022 |
  | Short Title | Impact of procedures and human-animal interactions during transport and slaughter on animal welfare of pigs |
  | Library Catalogue | Google Scholar |
  | URL | https://www.mdpi.com/2076-2615/12/23/3391 |
  | Accessed | 11/07/2025, 10:07:30 |
  | Volume | 12 |
  | Publisher | MDPI |
  | Pages | 3391 |
  | Publication | Animals |
  | Issue | 23 |
  | Date Added | 11/07/2025, 10:07:52 |
  | Modified | 11/07/2025, 10:07:52 |
- ## A Systematic Review of Dog-Assisted Therapy in Children with Behavioural and Developmental Disorders

  |  |  |
  | --- | --- |
  | Item Type | Journal Article |
  | Author | Candela J. Hüsgen |
  | Author | Nienke C. Peters-Scheffer |
  | Author | Robert Didden |
  | Abstract | Abstract Objectives Animal-assisted therapy with dogs is regularly used in children with behavioural and developmental disorders. Aims of this systematic review were threefold: to analyse the methodological quality of studies on dog-assisted therapy (DAT) for children with behavioural and developmental disorders, to determine to which extent the studies on DAT adhere to the quality criteria developed by the International Association of Human Animal Interaction Organisation (IAHAIO) and to describe the characteristics of the participants, the intervention and the outcomes. Method Three databases (i.e. PsycInfo, MedLine and Eric) were searched, and 14 studies on DAT were included. The Joanna Briggs Institute checklist (JBIC) and the quality criteria developed by the IAHAIO were used during data extraction. Characteristics of the participants, the intervention, the therapy dogs and the outcomes of the studies were summarised. Results Six of the 14 included studies reported significant outcomes of DAT, whereof six in the social domain and two in the psychological domain. However, scores on the JBIC indicated low to moderate methodological quality and only three of the included studies adhered to the IAHAIO quality criteria. Conclusions DAT is a promising intervention for children with behavioural and developmental disorders, especially for children with autism spectrum disorder. A clear description of the therapy’s components, the role of the therapy dog and analysis of the treatment integrity and procedural fidelity would improve the methodological quality of the studies and the field of dog-assisted interventions. |
  | Date | 03/2022 |
  | Language | en |
  | Library Catalogue | Crossref |
  | URL | https://link.springer.com/10.1007/s41252-022-00239-9 |
  | Accessed | 11/07/2025, 10:18:01 |
  | Rights | https://creativecommons.org/licenses/by/4.0 |
  | Volume | 6 |
  | Publisher | Springer Science and Business Media LLC |
  | Pages | 1-10 |
  | Publication | Advances in Neurodevelopmental Disorders |
  | DOI | 10.1007/s41252-022-00239-9 |
  | Issue | 1 |
  | Journal Abbr | Adv Neurodev Disord |
  | ISSN | 2366-7532, 2366-7540 |
  | Date Added | 11/07/2025, 10:18:17 |
  | Modified | 11/07/2025, 10:18:17 |

  ### Attachments

  - Available Version (via Google Scholar)
- ## Animals in higher education settings: Do animal-assisted interventions improve mental and cognitive health outcomes of students? A systematic review and meta-analysis

  |  |  |
  | --- | --- |
  | Item Type | Journal Article |
  | Author | Annalena Huber |
  | Author | Stefanie J. Klug |
  | Author | Annette Abraham |
  | Author | Erica Westenberg |
  | Author | Veronika Schmidt |
  | Author | Andrea S. Winkler |
  | Date | 2022 |
  | Short Title | Animals in higher education settings |
  | Library Catalogue | Google Scholar |
  | URL | https://www.medrxiv.org/content/10.1101/2022.04.11.22273607.abstract |
  | Accessed | 11/07/2025, 10:12:06 |
  | Publisher | Cold Spring Harbor Laboratory Press |
  | Pages | 2022–04 |
  | Publication | medRxiv |
  | Date Added | 11/07/2025, 10:12:39 |
  | Modified | 11/07/2025, 10:12:39 |
- ## Animal-Assisted Interventions Improve Mental, But Not Cognitive or Physiological Health Outcomes of Higher Education Students: a Systematic Review and Meta-analysis

  |  |  |
  | --- | --- |
  | Item Type | Journal Article |
  | Author | Annalena Huber |
  | Author | Stefanie J. Klug |
  | Author | Annette Abraham |
  | Author | Erica Westenberg |
  | Author | Veronika Schmidt |
  | Author | Andrea S. Winkler |
  | Abstract | AbstractDue to the high burden of mental health issues among students at higher education institutions world-wide, animal-assisted interventions (AAIs) are being used to relieve student stress. The objective of this study was to systematically review of the effects of AAIs on the mental, physiological, and cognitive outcomes of higher education students. Randomized controlled trials using any unfamiliar animal as the sole intervention tool were included in this review. Study quality was assessed using the Cochrane Risk-of-Bias tool. Where possible, effect sizes (Hedges’ g) were pooled for individual outcomes using random-effects meta-analyses. Albatross plots were used to supplement the data synthesis. Of 2.494 identified studies, 35 were included. Almost all studies used dogs as the intervention animal. The quality of most included studies was rated as moderate. Studies showed an overall reduction of acute anxiety and stress. For other mental outcomes, studies showed smaller, but nonetheless beneficial effects. Studies showed no clear effect on physiological or cognitive outcomes. Strong methodological heterogeneity between studies limited the ability to draw clear conclusions. |
  | Date | 06/2024 |
  | Language | en |
  | Short Title | Animal-Assisted Interventions Improve Mental, But Not Cognitive or Physiological Health Outcomes of Higher Education Students |
  | Library Catalogue | Crossref |
  | URL | https://link.springer.com/10.1007/s11469-022-00945-4 |
  | Accessed | 11/07/2025, 13:46:26 |
  | Rights | https://creativecommons.org/licenses/by/4.0 |
  | Volume | 22 |
  | Publisher | Springer Science and Business Media LLC |
  | Pages | 1597-1628 |
  | Publication | International Journal of Mental Health and Addiction |
  | DOI | 10.1007/s11469-022-00945-4 |
  | Issue | 3 |
  | Journal Abbr | Int J Ment Health Addiction |
  | ISSN | 1557-1874, 1557-1882 |
  | Date Added | 11/07/2025, 13:46:33 |
  | Modified | 11/07/2025, 13:46:33 |

  ### Attachments

  - Available Version (via Google Scholar)
- ## Health Benefits of Human-Companion Animal Interaction: A Review

  |  |  |
  | --- | --- |
  | Item Type | Journal Article |
  | Author | Shreyansh Hosure |
  | Author | T. S. Rajeev |
  | Date | 2020 |
  | Short Title | Health Benefits of Human-Companion Animal Interaction |
  | Library Catalogue | Google Scholar |
  | URL | https://pdfs.semanticscholar.org/9d5e/68490416bf18e6920e211c6ae8f32f880f71.pdf |
  | Accessed | 11/07/2025, 10:05:05 |
  | Volume | 8 |
  | Pages | 658–62 |
  | Publication | Indian Journal of Pure & Applied Biosciences |
  | Date Added | 11/07/2025, 10:05:27 |
  | Modified | 11/07/2025, 10:05:27 |

  ### Attachments

  - Available Version (via Google Scholar)
- ## Human-animal interactions, relationships and bonds: A review and analysis of the literature

  |  |  |
  | --- | --- |
  | Item Type | Journal Article |
  | Author | Geoff Hosey |
  | Author | Vicky Melfi |
  | Date | 2014 |
  | Short Title | Human-animal interactions, relationships and bonds |
  | Library Catalogue | Google Scholar |
  | URL | https://escholarship.org/uc/item/6955n8kd |
  | Accessed | 11/07/2025, 10:05:01 |
  | Volume | 27 |
  | Publication | International Journal of Comparative Psychology |
  | Issue | 1 |
  | Date Added | 11/07/2025, 10:05:27 |
  | Modified | 11/07/2025, 10:05:27 |

  ### Attachments

  - Available Version (via Google Scholar)
- ## A preliminary model of human–animal relationships in the zoo

  |  |  |
  | --- | --- |
  | Item Type | Journal Article |
  | Author | Geoff Hosey |
  | Date | 2008 |
  | Library Catalogue | Google Scholar |
  | URL | https://www.sciencedirect.com/science/article/pii/S0168159107001426 |
  | Accessed | 11/07/2025, 09:27:34 |
  | Volume | 109 |
  | Publisher | Elsevier |
  | Pages | 105–127 |
  | Publication | Applied Animal Behaviour Science |
  | Issue | 2-4 |
  | Date Added | 11/07/2025, 09:27:42 |
  | Modified | 11/07/2025, 09:27:42 |
- ## Influence of crude fibre in piglets’ rations and the animal-to-feeding-place ratio on tail-biting in weaning pigs

  |  |  |
  | --- | --- |
  | Item Type | Journal Article |
  | Author | Anja Honeck |
  | Date | 2019 |
  | Library Catalogue | Google Scholar |
  | URL | https://elib.tiho-hannover.de/servlets/MCRFileNodeServlet/tiho\_derivate\_00000069/honecka-ss19.pdf |
  | Accessed | 11/07/2025, 09:37:55 |
  | Publisher | Dissertation, Hannover, Tierärztliche Hochschule Hannover, 2019 |
  | Date Added | 11/07/2025, 09:38:03 |
  | Modified | 11/07/2025, 09:38:03 |

  ### Attachments

  - Available Version (via Google Scholar)
- ## Impact of stress on the body of service dogs and alleviation methods: Literature overview

  |  |  |
  | --- | --- |
  | Item Type | Journal Article |
  | Author | Serhii Holopura |
  | Author | Nataliia Boiko |
  | Short Title | Impact of stress on the body of service dogs and alleviation methods |
  | Library Catalogue | Google Scholar |
  | URL | https://scireports.com.ua/web/uploads/pdf/Scientific%20Reports\_20\_6\_2024\_138-150.pdf |
  | Accessed | 11/07/2025, 10:18:11 |
  | Date Added | 11/07/2025, 10:18:17 |
  | Modified | 11/07/2025, 10:18:17 |

  ### Attachments

  - Available Version (via Google Scholar)
- ## Champing at the bit for improvements: A review of equine welfare in equestrian sports in the United Kingdom

  |  |  |
  | --- | --- |
  | Item Type | Journal Article |
  | Author | Tim Q. Holmes |
  | Author | Ashleigh F. Brown |
  | Date | 2022 |
  | Short Title | Champing at the bit for improvements |
  | Library Catalogue | Google Scholar |
  | URL | https://www.mdpi.com/2076-2615/12/9/1186 |
  | Accessed | 11/07/2025, 11:09:42 |
  | Volume | 12 |
  | Publisher | MDPI |
  | Pages | 1186 |
  | Publication | Animals |
  | Issue | 9 |
  | Date Added | 11/07/2025, 11:09:44 |
  | Modified | 11/07/2025, 11:09:44 |
- ## A Systematic Literature Review of Animal-Assisted Interventions in Oncology (Part II): Theoretical Mechanisms and Frameworks

  |  |  |
  | --- | --- |
  | Item Type | Journal Article |
  | Author | Timothy R. N. Holder |
  | Author | Margaret E. Gruen |
  | Author | David L. Roberts |
  | Author | Tamara Somers |
  | Author | Alper Bozkurt |
  | Abstract | Animal-assisted interventions (AAIs) can improve patients’ quality of life as complementary medical treatments. Part I of this 2-paper systematic review focused on the methods and results of cancer-related AAIs; Part II discusses the theories of the field’s investigators. Researchers cite animal personality, physical touch, physical movement, distraction, and increased human interaction as sources of observed positive outcomes. These mechanisms then group under theoretical frameworks such as the social support hypothesis or the human-animal bond concept to fully explain AAI in oncology. The cognitive activation theory of stress, the science of unitary human beings, and the self-object hypothesis are additional frameworks mentioned by some researchers. We also discuss concepts of neurobiological transduction connecting mechanisms to AAI benefits. Future researchers should base study design on theories with testable hypotheses and use consistent terminology to report results. This review aids progress toward a unified theoretical framework and toward more holistic cancer treatments. |
  | Date | 01/2020 |
  | Language | en |
  | Short Title | A Systematic Literature Review of Animal-Assisted Interventions in Oncology (Part II) |
  | Library Catalogue | Crossref |
  | URL | https://journals.sagepub.com/doi/10.1177/1534735420943269 |
  | Accessed | 11/07/2025, 10:11:55 |
  | Rights | https://creativecommons.org/licenses/by-nc/4.0/ |
  | Volume | 19 |
  | Publisher | SAGE Publications |
  | Publication | Integrative Cancer Therapies |
  | DOI | 10.1177/1534735420943269 |
  | Journal Abbr | Integr Cancer Ther |
  | ISSN | 1534-7354, 1552-695X |
  | Date Added | 11/07/2025, 10:12:39 |
  | Modified | 11/07/2025, 10:12:39 |

  ### Attachments

  - Available Version (via Google Scholar)
- ## Exploring Synchronicity in the Heart Rates of Familiar and Unfamiliar Pairs of Horses and Humans Undertaking an In-Hand Task

  |  |  |
  | --- | --- |
  | Item Type | Journal Article |
  | Author | Jo Hockenhull |
  | Author | Tamsin J. Young |
  | Author | Sarah E. Redgate |
  | Author | Lynda Birke |
  | Date | 2015-09-02 |
  | Language | en |
  | Library Catalogue | DOI.org (Crossref) |
  | URL | https://www.tandfonline.com/doi/full/10.1080/08927936.2015.1052284 |
  | Accessed | 17/06/2025, 18:06:24 |
  | Volume | 28 |
  | Pages | 501-511 |
  | Publication | Anthrozoös |
  | DOI | 10.1080/08927936.2015.1052284 |
  | Issue | 3 |
  | Journal Abbr | Anthrozoös |
  | ISSN | 0892-7936, 1753-0377 |
  | Date Added | 17/06/2025, 18:06:24 |
  | Modified | 17/06/2025, 18:06:24 |

  ### Attachments

  - Accepted Version
- ## An independent animal welfare assessment of mass destruction methods for poultry on-farm

  |  |  |
  | --- | --- |
  | Item Type | Journal Article |
  | Author | Leisha Hewitt |
  | Date | 2023 |
  | Library Catalogue | Google Scholar |
  | URL | https://www.agriculture.gov.au/sites/default/files/documents/independent-animal-welfare-assessment-mass-destruction-methods-poultry-on-farm.pdf |
  | Accessed | 11/07/2025, 09:58:06 |
  | Date Added | 11/07/2025, 09:58:25 |
  | Modified | 11/07/2025, 09:58:25 |

  ### Attachments

  - Available Version (via Google Scholar)
- ## Compilation of Animal-Assisted Therapy Studies: A Narrative Review of Clinical Evidence.

  |  |  |
  | --- | --- |
  | Item Type | Journal Article |
  | Author | V. Herrera |
  | Author | L. Gutierrez-Rojas |
  | Date | 2025 |
  | Short Title | Compilation of Animal-Assisted Therapy Studies |
  | Library Catalogue | Google Scholar |
  | URL | https://www.sciencedirect.com/science/article/pii/S1134593425000405 |
  | Accessed | 11/07/2025, 10:20:22 |
  | Publisher | Elsevier |
  | Pages | 100738 |
  | Publication | Psiquiatría Biológica |
  | Date Added | 11/07/2025, 10:20:29 |
  | Modified | 11/07/2025, 10:20:29 |
- ## Science alone is not always enough: The importance of ethical assessment for a more comprehensive view of equine welfare

  |  |  |
  | --- | --- |
  | Item Type | Journal Article |
  | Author | Camie R. Heleski |
  | Author | Raymond Anthony |
  | Date | 2012 |
  | Short Title | Science alone is not always enough |
  | Library Catalogue | Google Scholar |
  | URL | https://www.sciencedirect.com/science/article/pii/S1558787811001456 |
  | Accessed | 11/07/2025, 11:09:40 |
  | Volume | 7 |
  | Publisher | Elsevier |
  | Pages | 169–178 |
  | Publication | Journal of Veterinary Behavior |
  | Issue | 3 |
  | Date Added | 11/07/2025, 11:09:44 |
  | Modified | 11/07/2025, 11:09:44 |
- ## Behavioral seizure correlates in animal models of epilepsy: a road map for assay selection, data interpretation, and the search for causal mechanisms

  |  |  |
  | --- | --- |
  | Item Type | Journal Article |
  | Author | Stephen C. Heinrichs |
  | Author | Thomas N. Seyfried |
  | Date | 2006 |
  | Short Title | Behavioral seizure correlates in animal models of epilepsy |
  | Library Catalogue | Google Scholar |
  | URL | https://www.sciencedirect.com/science/article/pii/S1525505005003318 |
  | Accessed | 11/07/2025, 09:35:32 |
  | Volume | 8 |
  | Publisher | Elsevier |
  | Pages | 5–38 |
  | Publication | Epilepsy & Behavior |
  | Issue | 1 |
  | Date Added | 11/07/2025, 09:36:11 |
  | Modified | 11/07/2025, 09:36:11 |

  ### Attachments

  - Available Version (via Google Scholar)
- ## Behavioral seizure correlates in animal models of epilepsy: a road map for assay selection, data interpretation, and the search for causal mechanisms

  |  |  |
  | --- | --- |
  | Item Type | Journal Article |
  | Author | Stephen C. Heinrichs |
  | Author | Thomas N. Seyfried |
  | Date | 2006 |
  | Short Title | Behavioral seizure correlates in animal models of epilepsy |
  | Library Catalogue | Google Scholar |
  | URL | https://www.sciencedirect.com/science/article/pii/S1525505005003318 |
  | Accessed | 11/07/2025, 11:09:12 |
  | Volume | 8 |
  | Publisher | Elsevier |
  | Pages | 5–38 |
  | Publication | Epilepsy & Behavior |
  | Issue | 1 |
  | Date Added | 11/07/2025, 11:09:44 |
  | Modified | 11/07/2025, 11:09:44 |
- ## Effectiveness of animal-assisted interventions for children and adults with post-traumatic stress disorder symptoms: A systematic review and meta-analysis

  |  |  |
  | --- | --- |
  | Item Type | Journal Article |
  | Author | Karin Hediger |
  | Author | Julia Wagner |
  | Author | Pascale Künzi |
  | Author | Anna Haefeli |
  | Author | Felicitas Theis |
  | Author | Carmina Grob |
  | Author | Elena Pauli |
  | Author | Heike Gerger |
  | Date | 2021 |
  | Short Title | Effectiveness of animal-assisted interventions for children and adults with post-traumatic stress disorder symptoms |
  | Library Catalogue | Google Scholar |
  | URL | https://www.tandfonline.com/doi/abs/10.1080/20008198.2021.1879713 |
  | Accessed | 11/07/2025, 10:13:38 |
  | Volume | 12 |
  | Publisher | Taylor & Francis |
  | Pages | 1879713 |
  | Publication | European journal of psychotraumatology |
  | Issue | 1 |
  | Date Added | 11/07/2025, 10:13:55 |
  | Modified | 11/07/2025, 10:13:55 |

  ### Attachments

  - Available Version (via Google Scholar)
- ## Animal-assisted therapy for schizophrenia and related disorders: A systematic review

  |  |  |
  | --- | --- |
  | Item Type | Journal Article |
  | Author | Emma L. Hawkins |
  | Author | Roxanne D. Hawkins |
  | Author | Martin Dennis |
  | Author | Joanne M. Williams |
  | Author | Stephen M. Lawrie |
  | Date | 2019 |
  | Short Title | Animal-assisted therapy for schizophrenia and related disorders |
  | Library Catalogue | Google Scholar |
  | URL | https://www.sciencedirect.com/science/article/pii/S0022395619300937 |
  | Accessed | 11/07/2025, 09:29:16 |
  | Volume | 115 |
  | Publisher | Elsevier |
  | Pages | 51–60 |
  | Publication | Journal of psychiatric research |
  | Date Added | 11/07/2025, 09:29:28 |
  | Modified | 11/07/2025, 09:29:28 |
- ## La terapia asistida con perros: una revisión de la literatura

  |  |  |
  | --- | --- |
  | Item Type | Journal Article |
  | Author | Helaina Louise Harvey |
  | Short Title | La terapia asistida con perros |
  | Library Catalogue | Google Scholar |
  | URL | http://dspace.umh.es/handle/11000/6138 |
  | Accessed | 11/07/2025, 11:14:26 |
  | Date Added | 11/07/2025, 11:14:45 |
  | Modified | 11/07/2025, 11:14:45 |
- ## Future Directions in AASC

  |  |  |
  | --- | --- |
  | Item Type | Journal Article |
  | Author | Elizabeth Kjellstrand Hartwig |
  | Date | 2024 |
  | Library Catalogue | Google Scholar |
  | URL | https://www.taylorfrancis.com/chapters/edit/10.4324/9781003392415-12/future-directions-aasc-elizabeth-kjellstrand-hartwig |
  | Accessed | 11/07/2025, 13:45:00 |
  | Publisher | Routledge |
  | Pages | 172–182 |
  | Publication | Animal-Assisted School Counseling |
  | Date Added | 11/07/2025, 13:45:12 |
  | Modified | 11/07/2025, 13:45:12 |
- ## The role of animal-assisted interventions (AAI) in healthcare waiting rooms: A scoping review on enhancing patients’ well-being and experience

  |  |  |
  | --- | --- |
  | Item Type | Journal Article |
  | Author | Irene Hartigan |
  | Author | Yvonne Pennisi |
  | Author | Claire Harman |
  | Author | Claire Keating |
  | Author | Kate Fitzgerald |
  | Author | My Linh Truong |
  | Abstract | Abstract This scoping review investigates the role of snimal-sssisted interventions (AAI) in enhancing patient well-being and experience in healthcare waiting rooms. From an initial pool of 1689 articles across CINAHL, PUBMED, and ASSIA, 8 studies met the inclusion criteria. The review evaluates the psychological and physical impacts of AAI, considerations for its implementation, and its potential to complement patient-centered care. Findings revealed that AAI consistently improved psychological outcomes, such as reducing anxiety and depression, though effects on physical parameters like pain and blood pressure remain mixed. While the term “therapy dog” may be misleading, these animals play a vital role in providing emotional support and enhancing patient well-being in healthcare settings. Future research should further clarify the distinct functions of therapy versus service dogs and explore the long-term and population-specific impacts of AAI in patient-centered care. |
  | Date | 2025-06-19 |
  | Language | en |
  | Short Title | The role of animal-assisted interventions (AAI) in healthcare waiting rooms |
  | Library Catalogue | Crossref |
  | URL | http://www.cabidigitallibrary.org/doi/10.1079/hai.2025.0030 |
  | Accessed | 11/07/2025, 09:31:38 |
  | Publisher | CABI Publishing |
  | Publication | Human-Animal Interactions |
  | DOI | 10.1079/hai.2025.0030 |
  | ISSN | 2957-9538 |
  | Date Added | 11/07/2025, 09:32:09 |
  | Modified | 11/07/2025, 09:32:09 |

  ### Attachments

  - Available Version (via Google Scholar)
- ## Equine Assisted Psychotherapy: Implications for Treating Trauma

  |  |  |
  | --- | --- |
  | Item Type | Journal Article |
  | Author | Kara L. Harrison |
  | Date | 2022 |
  | Short Title | Equine Assisted Psychotherapy |
  | Library Catalogue | Google Scholar |
  | URL | https://encompass.eku.edu/psych\_doctorals/26/ |
  | Accessed | 11/07/2025, 09:37:43 |
  | Date Added | 11/07/2025, 09:38:03 |
  | Modified | 11/07/2025, 09:38:03 |

  ### Attachments

  - Available Version (via Google Scholar)
- ## Equine Assisted Psychotherapy: Implications for Treating Trauma

  |  |  |
  | --- | --- |
  | Item Type | Journal Article |
  | Author | Kara L. Harrison |
  | Date | 2022 |
  | Short Title | Equine Assisted Psychotherapy |
  | Library Catalogue | Google Scholar |
  | URL | https://encompass.eku.edu/psych\_doctorals/26/ |
  | Accessed | 11/07/2025, 11:11:54 |
  | Date Added | 11/07/2025, 11:12:01 |
  | Modified | 11/07/2025, 11:12:01 |

  ### Attachments

  - Available Version (via Google Scholar)
- ## Short-Term Interaction between Dogs and Their Owners: Effects on Oxytocin, Cortisol, Insulin and Heart Rate—An Exploratory Study

  |  |  |
  | --- | --- |
  | Item Type | Journal Article |
  | Author | Linda Handlin |
  | Author | Eva Hydbring-Sandberg |
  | Author | Anne Nilsson |
  | Author | Mikael Ejdebäck |
  | Author | Anna Jansson |
  | Author | Kerstin Uvnäs-Moberg |
  | Date | 09/2011 |
  | Language | en |
  | Short Title | Short-Term Interaction between Dogs and Their Owners |
  | Library Catalogue | DOI.org (Crossref) |
  | URL | https://www.tandfonline.com/doi/full/10.2752/175303711X13045914865385 |
  | Accessed | 17/06/2025, 17:35:50 |
  | Volume | 24 |
  | Pages | 301-315 |
  | Publication | Anthrozoös |
  | DOI | 10.2752/175303711X13045914865385 |
  | Issue | 3 |
  | Journal Abbr | Anthrozoös |
  | ISSN | 0892-7936, 1753-0377 |
  | Date Added | 17/06/2025, 17:35:50 |
  | Modified | 17/06/2025, 17:35:50 |

  ### Attachments

  - PDF
- ## Human-Human and Human-Animal Interaction : Some Common Physiological and Psychological Effects

  |  |  |
  | --- | --- |
  | Item Type | Thesis |
  | Author | Linda Handlin |
  | Abstract | The aim of the present thesis was to investigate hormonal and physiological effects in mothers during a breastfeeding session and in dogs and their owners in response to short-term interaction. In study one, sixty-six mothers receiving either exogenous oxytocin infusion and/or epidural analgesia (EDA) during labor or intramuscular oxytocin injection post partum were studied. Oxytocin, prolactin, adrenocorticotrophic hormone (ACTH) and cortisol levels, as well as blood pressure were measured during a breastfeeding session two days after birth. In response to breastfeeding two days after birth, the mothers displayed a pulsatile release of oxytocin and increasing prolactin levels. In addition, the activity in the HPA-axis was reduced and maternal blood pressure decreased. The results also show that EDA administration in combination with oxytocin during labor resulted insignificantly lower oxytocin levels and higher cortisol levels, as well as higher bloodpressure in response to breastfeeding two days after birth, compared to EDA administration alone. In addition, oxytocin infusions dose-dependently lowered the mothers’ endogenous oxytocin levels two days after birth. In study two, ten female dog owners and their male Labrador dogs participated, together with ten controls. Their levels of oxytocin, cortisol and insulin, as well as their heart rate, were measured. The connection between the quality of the dogowner relationship and hormone levels was also explored. Short-term interaction between dogs and their owners resulted in oxytocin release in both species and their cortisol levels and heart rate were also affected. Oxytocin levels and positive attitudes regarding the dog-owner relationship were positively correlated. In conclusion, both human-human and human-animal interactions induce oxytocin release and promote oxytocin mediated effects, such as decreasing cortisol levels and blood pressure. In addition, social interaction and oxytocin levels arepositively related. |
  | Date | 2010 |
  | URL | https://www.mynewsdesk.com/se/sveriges\_lantbruksuniversitet\_\_slu/pressreleases/maenniska-och-djur-har-gemensamt-reaktionsmoenster-562390 |
  | Extra | Backup Publisher: Swedish University of Agricultural Sciences, Department of Animal Environment and Health, Skara, Sweden Issue: 2010:98 |
  | ISBN | 978-91-576-7543-9 |
  | Series | Acta Universitatis agriculturae Sueciae |
  | # of Pages | 88 |
  | Type | PhD Thesis |
  | University | Swedish University of Agricultural Sciences, Department of Animal Environment and Health, Skara, Sweden |
  | Date Added | 17/06/2025, 16:42:03 |
  | Modified | 17/06/2025, 16:42:03 |

  ### Tags:

  - ACTH
  - blood pressure
  - cortisol
  - interaction
  - medical interventions
  - oxytocin
  - prolactin
  - sensory stimulation

  ### Notes:

  - Doctoral thesis

  ### Attachments

  - PDF
- ## Effects of stroking horses on both humans' and horses' heart rate responses<sup>1</sup>

  |  |  |
  | --- | --- |
  | Item Type | Journal Article |
  | Author | Haruyo Hama |
  | Author | Masao Yogo |
  | Author | Yoshinori Matsuyama |
  | Abstract | The present study examined both human and horse heart rates (HRs) when humans stroked horses for 90 seconds; the subjective arousal levels of the humans were measured by the Tohoku Activation Deactivation Adjective Check List before and after stroking horses. Six male sublects with a positive attitude toward companion animals and 6 male subjects with a negative attitude were selected by their scores on the Pet Attitude Scale, and these two groups, together with a third group, of 6 subjects who were male members of the Doshisha University horse-riding club, participated in this experiment. The HRs of the human subjects during the first 10 seconds immediately after the stroking began were significantly higher than those obtained after that period, but these higher levels gradually returned to baseline levels. This tendency appears more clearly in the negative attitude group. The HRs of the horses increased during the first 20 seconds immediately after the human subjects of the NA group started stroking them, but gradually reduced as the stroking continued. The results of subjective arousal levels suggest a decrease in tension by stroking horses. These results suggest that a certain affectional interaction may exist between humans and companion animals. |
  | Date | 05/1996 |
  | Language | en |
  | Library Catalogue | DOI.org (Crossref) |
  | URL | https://onlinelibrary.wiley.com/doi/10.1111/j.1468-5884.1996.tb00009.x |
  | Accessed | 17/06/2025, 16:48:18 |
  | Rights | http://onlinelibrary.wiley.com/termsAndConditions#vor |
  | Volume | 38 |
  | Pages | 66-73 |
  | Publication | Japanese Psychological Research |
  | DOI | 10.1111/j.1468-5884.1996.tb00009.x |
  | Issue | 2 |
  | Journal Abbr | Jpn Psychol Res |
  | ISSN | 0021-5368, 1468-5884 |
  | Date Added | 17/06/2025, 16:48:18 |
  | Modified | 17/06/2025, 16:48:18 |

  ### Attachments

  - PDF
- ## Education and Licensing of Horse Owners: Addressing Poor Horse Welfare in the UK

  |  |  |
  | --- | --- |
  | Item Type | Journal Article |
  | Author | Aurelia Hall-Bromley |
  | Author | Laura Dixon |
  | Date | 2025 |
  | Short Title | Education and Licensing of Horse Owners |
  | Library Catalogue | Google Scholar |
  | URL | https://www.mdpi.com/2076-2615/15/7/1037 |
  | Accessed | 11/07/2025, 09:37:59 |
  | Volume | 15 |
  | Publisher | MDPI |
  | Pages | 1037 |
  | Publication | Animals |
  | Issue | 7 |
  | Date Added | 11/07/2025, 09:38:03 |
  | Modified | 11/07/2025, 09:38:03 |
- ## Education and Licensing of Horse Owners: Addressing Poor Horse Welfare in the UK

  |  |  |
  | --- | --- |
  | Item Type | Journal Article |
  | Author | Aurelia Hall-Bromley |
  | Author | Laura Dixon |
  | Date | 2025 |
  | Short Title | Education and Licensing of Horse Owners |
  | Library Catalogue | Google Scholar |
  | URL | https://www.mdpi.com/2076-2615/15/7/1037 |
  | Accessed | 11/07/2025, 11:14:10 |
  | Volume | 15 |
  | Publisher | MDPI |
  | Pages | 1037 |
  | Publication | Animals |
  | Issue | 7 |
  | Date Added | 11/07/2025, 11:14:45 |
  | Modified | 11/07/2025, 11:14:45 |
- ## Education and Licensing of Horse Owners: Addressing Poor Horse Welfare in the UK

  |  |  |
  | --- | --- |
  | Item Type | Journal Article |
  | Author | Aurelia Hall-Bromley |
  | Author | Laura Dixon |
  | Date | 2025 |
  | Short Title | Education and Licensing of Horse Owners |
  | Library Catalogue | Google Scholar |
  | URL | https://www.mdpi.com/2076-2615/15/7/1037 |
  | Accessed | 11/07/2025, 13:44:03 |
  | Volume | 15 |
  | Publisher | MDPI |
  | Pages | 1037 |
  | Publication | Animals |
  | Issue | 7 |
  | Date Added | 11/07/2025, 13:44:05 |
  | Modified | 11/07/2025, 13:44:05 |
- ## Organic milk production and dairy farming constraints and prospects under the laws of the European Union

  |  |  |
  | --- | --- |
  | Item Type | Journal Article |
  | Author | Grzegorz Grodkowski |
  | Author | Marcin Gołębiewski |
  | Author | Jan Slósarz |
  | Author | Kinga Grodkowska |
  | Author | Piotr Kostusiak |
  | Author | Tomasz Sakowski |
  | Author | Kamila Puppel |
  | Date | 2023 |
  | Library Catalogue | Google Scholar |
  | URL | https://www.mdpi.com/2076-2615/13/9/1457 |
  | Accessed | 11/07/2025, 11:14:06 |
  | Volume | 13 |
  | Publisher | MDPI |
  | Pages | 1457 |
  | Publication | Animals |
  | Issue | 9 |
  | Date Added | 11/07/2025, 11:14:45 |
  | Modified | 11/07/2025, 11:14:45 |

  ### Attachments

  - Available Version (via Google Scholar)
- ## The impact of dog therapy on nursing students’ heart rates and ability to pay attention in class

  |  |  |
  | --- | --- |
  | Item Type | Journal Article |
  | Author | Odette Griscti |
  | Author | Liberato Camilleri |
  | Date | 2020 |
  | Library Catalogue | Google Scholar |
  | URL | https://www.sciencedirect.com/science/article/pii/S0883035519316295 |
  | Accessed | 11/07/2025, 09:31:33 |
  | Volume | 99 |
  | Publisher | Elsevier |
  | Pages | 101498 |
  | Publication | International Journal of Educational Research |
  | Date Added | 11/07/2025, 09:32:09 |
  | Modified | 11/07/2025, 09:32:09 |
- ## The impact of dog therapy on nursing students’ heart rates and ability to pay attention in class

  |  |  |
  | --- | --- |
  | Item Type | Journal Article |
  | Author | Odette Griscti |
  | Author | Liberato Camilleri |
  | Date | 2020 |
  | Library Catalogue | Google Scholar |
  | URL | https://www.sciencedirect.com/science/article/pii/S0883035519316295 |
  | Accessed | 11/07/2025, 10:05:25 |
  | Volume | 99 |
  | Publisher | Elsevier |
  | Pages | 101498 |
  | Publication | International Journal of Educational Research |
  | Date Added | 11/07/2025, 10:05:27 |
  | Modified | 11/07/2025, 10:05:27 |
- ## Head, Eye and Comb temperature changes in Chooks during handling: the use of infrared thermal imaging in observing stress in chooks.

  |  |  |
  | --- | --- |
  | Item Type | Journal Article |
  | Author | Courtney Estelle Good |
  | Date | 2016 |
  | Short Title | Head, Eye and Comb temperature changes in Chooks during handling |
  | Library Catalogue | Google Scholar |
  | URL | https://researchcommons.waikato.ac.nz/bitstream/10289/11079/3/thesis.pdf |
  | Accessed | 11/07/2025, 09:37:35 |
  | Publisher | University of Waikato |
  | Date Added | 11/07/2025, 09:38:03 |
  | Modified | 11/07/2025, 09:38:03 |
- ## Nature-Oriented Activities In Kindergarten: Literature Review Of The Effects Of Children-Animal Interaction

  |  |  |
  | --- | --- |
  | Item Type | Conference Paper |
  | Author | Terry Goldstein\* |
  | Author | Alina S. Rusu |
  | Date | 2018-06-28 |
  | Short Title | Nature-Oriented Activities In Kindergarten |
  | Library Catalogue | Crossref |
  | URL | https://www.europeanproceedings.com/article/10.15405/epsbs.2018.06.81 |
  | Accessed | 11/07/2025, 10:07:21 |
  | Publisher | Cognitive-Crcs |
  | Pages | 678-684 |
  | Proceedings Title | The European Proceedings of Social and Behavioural Sciences |
  | Conference Name | ERD 2017 - Education, Reflection, Development, Fourth Edition |
  | DOI | 10.15405/epsbs.2018.06.81 |
  | ISSN | 2357-1330 |
  | Date Added | 11/07/2025, 10:07:52 |
  | Modified | 11/07/2025, 10:07:52 |

  ### Attachments

  - Available Version (via Google Scholar)
- ## Influence of the Human-Animal Relationship on Productivity and Animal Welfare in Dairy Farms

  |  |  |
  | --- | --- |
  | Item Type | Journal Article |
  | Author | Martínez GM |
  | Author | V. H. Suárez |
  | Author | M. D. Ghezzi |
  | Library Catalogue | Google Scholar |
  | URL | https://www.researchgate.net/profile/Gabriela-Martinez-13/publication/333293249\_Influence\_of\_the\_Human-Animal\_Relationship\_on\_Productivity\_and\_Animal\_Welfare\_in\_Dairy\_Farms/links/5ce56de1a6fdccc9ddc6dbd6/Influence-of-the-Human-Animal-Relationship-on-Productivity-and-Animal-Welfare-in-Dairy-Farms.pdf |
  | Accessed | 11/07/2025, 10:16:23 |
  | Date Added | 11/07/2025, 10:16:47 |
  | Modified | 11/07/2025, 10:16:47 |
- ## Therapy dog welfare revisited: a review of the literature

  |  |  |
  | --- | --- |
  | Item Type | Journal Article |
  | Author | Lisa Maria Glenk |
  | Author | Sandra Foltin |
  | Date | 2021 |
  | Short Title | Therapy dog welfare revisited |
  | Library Catalogue | Google Scholar |
  | URL | https://www.mdpi.com/2306-7381/8/10/226 |
  | Accessed | 11/07/2025, 10:07:24 |
  | Volume | 8 |
  | Publisher | MDPI |
  | Pages | 226 |
  | Publication | Veterinary Sciences |
  | Issue | 10 |
  | Date Added | 11/07/2025, 10:07:52 |
  | Modified | 11/07/2025, 10:07:52 |

  ### Attachments

  - Available Version (via Google Scholar)
- ## Current perspectives on therapy dog welfare in animal-assisted interventions

  |  |  |
  | --- | --- |
  | Item Type | Journal Article |
  | Author | Lisa Maria Glenk |
  | Date | 2017 |
  | Library Catalogue | Google Scholar |
  | URL | https://www.mdpi.com/2076-2615/7/2/7 |
  | Accessed | 11/07/2025, 09:27:28 |
  | Volume | 7 |
  | Publisher | MDPI |
  | Pages | 7 |
  | Publication | Animals |
  | Issue | 2 |
  | Date Added | 11/07/2025, 09:27:42 |
  | Modified | 11/07/2025, 09:27:42 |

  ### Attachments

  - Available Version (via Google Scholar)
- ## Current perspectives on therapy dog welfare in animal-assisted interventions

  |  |  |
  | --- | --- |
  | Item Type | Journal Article |
  | Author | Lisa Maria Glenk |
  | Date | 2017 |
  | Library Catalogue | Google Scholar |
  | URL | https://www.mdpi.com/2076-2615/7/2/7 |
  | Accessed | 11/07/2025, 10:07:34 |
  | Volume | 7 |
  | Publisher | MDPI |
  | Pages | 7 |
  | Publication | Animals |
  | Issue | 2 |
  | Date Added | 11/07/2025, 10:07:52 |
  | Modified | 11/07/2025, 10:07:52 |

  ### Attachments

  - Available Version (via Google Scholar)
- ## Coping style in farm animals: behavioural trait or production index?

  |  |  |
  | --- | --- |
  | Item Type | Journal Article |
  | Author | L. R. Giles |
  | Author | R. J. Kilgour |
  | Date | 1999 |
  | Short Title | Coping style in farm animals |
  | Library Catalogue | Google Scholar |
  | URL | http://www.livestocklibrary.com/bitstream/handle/1234/19871/99\_187.pdf?sequence=1 |
  | Accessed | 11/07/2025, 10:19:58 |
  | Publisher | RAAN |
  | Date Added | 11/07/2025, 10:20:29 |
  | Modified | 11/07/2025, 10:20:29 |

  ### Attachments

  - Available Version (via Google Scholar)
- ## Dogs supporting human health and well-being: A biopsychosocial approach

  |  |  |
  | --- | --- |
  | Item Type | Journal Article |
  | Author | Nancy R. Gee |
  | Author | Kerri E. Rodriguez |
  | Author | Aubrey H. Fine |
  | Author | Janet P. Trammell |
  | Date | 2021 |
  | Short Title | Dogs supporting human health and well-being |
  | Library Catalogue | Google Scholar |
  | URL | https://www.frontiersin.org/articles/10.3389/fvets.2021.630465/full |
  | Accessed | 11/07/2025, 09:27:40 |
  | Volume | 8 |
  | Publisher | Frontiers Media SA |
  | Pages | 630465 |
  | Publication | Frontiers in Veterinary Science |
  | Date Added | 11/07/2025, 09:27:42 |
  | Modified | 11/07/2025, 09:27:42 |

  ### Attachments

  - Available Version (via Google Scholar)
- ## Human–animal interaction and older adults: An overview

  |  |  |
  | --- | --- |
  | Item Type | Journal Article |
  | Author | Nancy R. Gee |
  | Author | Megan K. Mueller |
  | Author | Angela L. Curl |
  | Date | 2017 |
  | Short Title | Human–animal interaction and older adults |
  | Library Catalogue | Google Scholar |
  | URL | https://www.frontiersin.org/articles/10.3389/fpsyg.2017.01416/full |
  | Accessed | 11/07/2025, 10:05:09 |
  | Volume | 8 |
  | Publisher | Frontiers Media SA |
  | Pages | 1416 |
  | Publication | Frontiers in psychology |
  | Date Added | 11/07/2025, 10:05:27 |
  | Modified | 11/07/2025, 10:05:27 |

  ### Attachments

  - Available Version (via Google Scholar)
- ## Human–Animal Interaction Research in School Settings: Current Knowledge and Future Directions

  |  |  |
  | --- | --- |
  | Item Type | Journal Article |
  | Author | Nancy R. Gee |
  | Author | James A. Griffin |
  | Author | Peggy McCardle |
  | Abstract | The involvement of animals is almost commonplace in many schools, although actual data documenting the extent and nature of human–animal interaction (HAI) in these settings are sparse. We provide an overview of the existing research and argue that the inclusion of animals in classroom settings can have an indirect effect on learning by directly affecting motivation, engagement, self-regulation, and human social interaction through those activities in which the interaction with animals is embedded. We support this theory with examples from the growing body of work indicating that, under specific conditions, with proper safeguards, HAI activities can benefit both typically developing children and those with developmental disabilities by reducing stress and anxiety and improving social interactions and by enhancing motivation, engagement, and learning. Nonetheless, a more comprehensive evidence base is needed to support this theory and to inform policies and practices for HAI in education settings, activities, and interventions. |
  | Date | 07/2017 |
  | Language | en |
  | Short Title | Human–Animal Interaction Research in School Settings |
  | Library Catalogue | Crossref |
  | URL | https://journals.sagepub.com/doi/10.1177/2332858417724346 |
  | Accessed | 11/07/2025, 10:05:20 |
  | Rights | https://journals.sagepub.com/page/policies/text-and-data-mining-license |
  | Volume | 3 |
  | Publisher | SAGE Publications |
  | Publication | AERA Open |
  | DOI | 10.1177/2332858417724346 |
  | Issue | 3 |
  | ISSN | 2332-8584, 2332-8584 |
  | Date Added | 11/07/2025, 10:05:27 |
  | Modified | 11/07/2025, 10:05:27 |

  ### Attachments

  - Available Version (via Google Scholar)
- ## Dogs supporting human health and well-being: A biopsychosocial approach

  |  |  |
  | --- | --- |
  | Item Type | Journal Article |
  | Author | Nancy R. Gee |
  | Author | Kerri E. Rodriguez |
  | Author | Aubrey H. Fine |
  | Author | Janet P. Trammell |
  | Date | 2021 |
  | Short Title | Dogs supporting human health and well-being |
  | Library Catalogue | Google Scholar |
  | URL | https://www.frontiersin.org/articles/10.3389/fvets.2021.630465/full |
  | Accessed | 11/07/2025, 10:09:42 |
  | Volume | 8 |
  | Publisher | Frontiers Media SA |
  | Pages | 630465 |
  | Publication | Frontiers in Veterinary Science |
  | Date Added | 11/07/2025, 10:09:48 |
  | Modified | 11/07/2025, 10:09:48 |

  ### Attachments

  - Available Version (via Google Scholar)
- ## A Systematic Review of Research on Pet Ownership and Animal Interactions among Older Adults

  |  |  |
  | --- | --- |
  | Item Type | Journal Article |
  | Author | Nancy R. Gee |
  | Author | Megan K. Mueller |
  | Date | 2019-03-04 |
  | Language | en |
  | Library Catalogue | Crossref |
  | URL | https://www.tandfonline.com/doi/full/10.1080/08927936.2019.1569903 |
  | Accessed | 11/07/2025, 10:09:29 |
  | Rights | http://creativecommons.org/licenses/by-nc-nd/4.0/ |
  | Volume | 32 |
  | Publisher | Informa UK Limited |
  | Pages | 183-207 |
  | Publication | Anthrozoös |
  | DOI | 10.1080/08927936.2019.1569903 |
  | Issue | 2 |
  | ISSN | 0892-7936, 1753-0377 |
  | Date Added | 11/07/2025, 10:09:48 |
  | Modified | 11/07/2025, 10:09:48 |

  ### Attachments

  - Available Version (via Google Scholar)
- ## A One Health Approach to Public Safety: A Review of Police Canines in the United States

  |  |  |
  | --- | --- |
  | Item Type | Journal Article |
  | Author | Meera Gatlin |
  | Date | 2024 |
  | Short Title | A One Health Approach to Public Safety |
  | Library Catalogue | Google Scholar |
  | URL | https://pmc.ncbi.nlm.nih.gov/articles/PMC11432170/ |
  | Accessed | 11/07/2025, 09:31:43 |
  | Volume | 21 |
  | Pages | 1235 |
  | Publication | International Journal of Environmental Research and Public Health |
  | Issue | 9 |
  | Date Added | 11/07/2025, 09:32:09 |
  | Modified | 11/07/2025, 09:32:09 |
- ## Pets in the workplace: a scoping review

  |  |  |
  | --- | --- |
  | Item Type | Journal Article |
  | Author | Dh Gardner |
  | Date | 2024-08-14 |
  | Language | en |
  | Short Title | Pets in the workplace |
  | Library Catalogue | Crossref |
  | URL | https://www.tandfonline.com/doi/full/10.1080/00480169.2024.2387562 |
  | Accessed | 11/07/2025, 09:32:09 |
  | Rights | http://creativecommons.org/licenses/by-nc-nd/4.0/ |
  | Publisher | Informa UK Limited |
  | Pages | 1-10 |
  | Publication | New Zealand Veterinary Journal |
  | DOI | 10.1080/00480169.2024.2387562 |
  | ISSN | 0048-0169, 1176-0710 |
  | Date Added | 11/07/2025, 09:32:09 |
  | Modified | 11/07/2025, 09:32:09 |

  ### Attachments

  - Available Version (via Google Scholar)
- ## Pets in the workplace: a scoping review

  |  |  |
  | --- | --- |
  | Item Type | Journal Article |
  | Author | Dh Gardner |
  | Date | 2024-08-14 |
  | Language | en |
  | Short Title | Pets in the workplace |
  | Library Catalogue | Crossref |
  | URL | https://www.tandfonline.com/doi/full/10.1080/00480169.2024.2387562 |
  | Accessed | 11/07/2025, 11:09:31 |
  | Rights | http://creativecommons.org/licenses/by-nc-nd/4.0/ |
  | Publisher | Informa UK Limited |
  | Pages | 1-10 |
  | Publication | New Zealand Veterinary Journal |
  | DOI | 10.1080/00480169.2024.2387562 |
  | ISSN | 0048-0169, 1176-0710 |
  | Date Added | 11/07/2025, 11:09:44 |
  | Modified | 11/07/2025, 11:09:44 |

  ### Attachments

  - Available Version (via Google Scholar)
- ## Pets in the workplace: a scoping review

  |  |  |
  | --- | --- |
  | Item Type | Journal Article |
  | Author | Dh Gardner |
  | Date | 2024-08-14 |
  | Language | en |
  | Short Title | Pets in the workplace |
  | Library Catalogue | Crossref |
  | URL | https://www.tandfonline.com/doi/full/10.1080/00480169.2024.2387562 |
  | Accessed | 11/07/2025, 13:44:45 |
  | Rights | http://creativecommons.org/licenses/by-nc-nd/4.0/ |
  | Publisher | Informa UK Limited |
  | Pages | 1-10 |
  | Publication | New Zealand Veterinary Journal |
  | DOI | 10.1080/00480169.2024.2387562 |
  | ISSN | 0048-0169, 1176-0710 |
  | Date Added | 11/07/2025, 13:45:12 |
  | Modified | 11/07/2025, 13:45:12 |

  ### Attachments

  - Available Version (via Google Scholar)
- ## Efectividad de la terapia asistida con perros como técnica de apoyo conductual en pacientes pediátricos durante la atención odontológica convencional. revisión sistemática

  |  |  |
  | --- | --- |
  | Item Type | Journal Article |
  | Author | Fernanda Beatriz García Hormazábal |
  | Date | 2024 |
  | Library Catalogue | Google Scholar |
  | URL | https://repositorio.uchile.cl/handle/2250/200460 |
  | Accessed | 11/07/2025, 11:14:30 |
  | Publisher | Universidad de Chile |
  | Date Added | 11/07/2025, 11:14:45 |
  | Modified | 11/07/2025, 11:14:45 |
- ## Efectividad de la terapia asistida con perros como técnica de apoyo conductual en pacientes pediátricos durante la atención odontológica convencional. revisión sistemática

  |  |  |
  | --- | --- |
  | Item Type | Journal Article |
  | Author | Fernanda Beatriz García Hormazábal |
  | Date | 2024 |
  | Library Catalogue | Google Scholar |
  | URL | https://repositorio.uchile.cl/handle/2250/200460 |
  | Accessed | 11/07/2025, 13:43:57 |
  | Publisher | Universidad de Chile |
  | Date Added | 11/07/2025, 13:44:05 |
  | Modified | 11/07/2025, 13:44:05 |

  ### Attachments

  - Available Version (via Google Scholar)
- ## Molecular biomarkers of adult human and dog stress during canine-assisted interventions: a systematic scoping review

  |  |  |
  | --- | --- |
  | Item Type | Journal Article |
  | Author | Jaci Gandenberger |
  | Author | Erin Flynn |
  | Author | Em Moratto |
  | Author | Ashley Wendt |
  | Author | Kevin N. Morris |
  | Date | 2022 |
  | Short Title | Molecular biomarkers of adult human and dog stress during canine-assisted interventions |
  | Library Catalogue | Google Scholar |
  | URL | https://www.mdpi.com/2076-2615/12/5/651 |
  | Accessed | 11/07/2025, 09:27:14 |
  | Volume | 12 |
  | Publisher | MDPI |
  | Pages | 651 |
  | Publication | Animals |
  | Issue | 5 |
  | Date Added | 11/07/2025, 09:27:42 |
  | Modified | 11/07/2025, 09:27:42 |

  ### Attachments

  - Available Version (via Google Scholar)
- ## Molecular biomarkers of adult human and dog stress during canine-assisted interventions: a systematic scoping review

  |  |  |
  | --- | --- |
  | Item Type | Journal Article |
  | Author | Jaci Gandenberger |
  | Author | Erin Flynn |
  | Author | Em Moratto |
  | Author | Ashley Wendt |
  | Author | Kevin N. Morris |
  | Date | 2022 |
  | Short Title | Molecular biomarkers of adult human and dog stress during canine-assisted interventions |
  | Library Catalogue | Google Scholar |
  | URL | https://www.mdpi.com/2076-2615/12/5/651 |
  | Accessed | 11/07/2025, 10:09:28 |
  | Volume | 12 |
  | Publisher | MDPI |
  | Pages | 651 |
  | Publication | Animals |
  | Issue | 5 |
  | Date Added | 11/07/2025, 10:09:48 |
  | Modified | 11/07/2025, 10:09:48 |

  ### Attachments

  - Available Version (via Google Scholar)
- ## The impact of dog-assisted therapy among children and adolescents with autism spectrum disorder: A systematic review

  |  |  |
  | --- | --- |
  | Item Type | Journal Article |
  | Author | Paula Galvany-López |
  | Author | Manuel Martí-Vilar |
  | Author | Sergio Hidalgo-Fuentes |
  | Author | Javier Cabedo-Peris |
  | Date | 2024 |
  | Short Title | The impact of dog-assisted therapy among children and adolescents with autism spectrum disorder |
  | Library Catalogue | Google Scholar |
  | URL | https://www.mdpi.com/2227-9067/11/12/1499 |
  | Accessed | 11/07/2025, 09:35:50 |
  | Volume | 11 |
  | Publisher | MDPI |
  | Pages | 1499 |
  | Publication | Children |
  | Issue | 12 |
  | Date Added | 11/07/2025, 09:36:11 |
  | Modified | 11/07/2025, 09:36:11 |
- ## The impact of dog-assisted therapy among children and adolescents with autism spectrum disorder: A systematic review

  |  |  |
  | --- | --- |
  | Item Type | Journal Article |
  | Author | Paula Galvany-López |
  | Author | Manuel Martí-Vilar |
  | Author | Sergio Hidalgo-Fuentes |
  | Author | Javier Cabedo-Peris |
  | Date | 2024 |
  | Short Title | The impact of dog-assisted therapy among children and adolescents with autism spectrum disorder |
  | Library Catalogue | Google Scholar |
  | URL | https://www.mdpi.com/2227-9067/11/12/1499 |
  | Accessed | 11/07/2025, 13:46:19 |
  | Volume | 11 |
  | Publisher | MDPI |
  | Pages | 1499 |
  | Publication | Children |
  | Issue | 12 |
  | Date Added | 11/07/2025, 13:46:33 |
  | Modified | 11/07/2025, 13:46:33 |
- ## Benefícios da relação entre o cão e seu tutor com sintomas de estresse, ansiedade e depressão: revisão de literatura

  |  |  |
  | --- | --- |
  | Item Type | Journal Article |
  | Author | Matheus Assis Furtado |
  | Author | Elen Tassiane Bauer Ramos |
  | Author | Bruna Samara Alves-Ribeiro |
  | Author | Zara Mariana de Assis-Silva |
  | Author | Alice Caroline da Silva Rocha |
  | Author | Guilherme Oliveira Maia |
  | Author | Ana Vitória Alves-Sobrinho |
  | Author | Lizandra Fernandes da Silva |
  | Author | Nicoly Ferreira de Urzedo |
  | Author | Iago de Sá Moraes |
  | Date | 2024 |
  | Short Title | Benefícios da relação entre o cão e seu tutor com sintomas de estresse, ansiedade e depressão |
  | Library Catalogue | Google Scholar |
  | URL | https://cuadernoseducacion.com/ojs/index.php/ced/article/view/3477 |
  | Accessed | 11/07/2025, 13:44:44 |
  | Volume | 16 |
  | Pages | e3477–e3477 |
  | Publication | Cuadernos de Educación y Desarrollo |
  | Issue | 2 |
  | Date Added | 11/07/2025, 13:45:12 |
  | Modified | 11/07/2025, 13:45:12 |

  ### Attachments

  - Available Version (via Google Scholar)
- ## Animal-Assisted Therapy for PTSD: A Literature Review

  |  |  |
  | --- | --- |
  | Item Type | Journal Article |
  | Author | Kristie M. Fuller |
  | Short Title | Animal-Assisted Therapy for PTSD |
  | Library Catalogue | Google Scholar |
  | URL | https://www.kristiemariecounseling.com/uploads/1/2/2/5/122533278/k\_fuller\_-\_lit\_review-ptsd\_and\_aat.pdf |
  | Accessed | 11/07/2025, 09:29:14 |
  | Date Added | 11/07/2025, 09:29:28 |
  | Modified | 11/07/2025, 09:29:28 |

  ### Attachments

  - Available Version (via Google Scholar)
- ## Exploring Animal-Assisted Programs with Children in School and Therapeutic Contexts

  |  |  |
  | --- | --- |
  | Item Type | Journal Article |
  | Author | Lori Friesen |
  | Date | 01/2010 |
  | Language | en |
  | Library Catalogue | Crossref |
  | URL | http://link.springer.com/10.1007/s10643-009-0349-5 |
  | Accessed | 11/07/2025, 10:07:37 |
  | Rights | http://www.springer.com/tdm |
  | Volume | 37 |
  | Publisher | Springer Science and Business Media LLC |
  | Pages | 261-267 |
  | Publication | Early Childhood Education Journal |
  | DOI | 10.1007/s10643-009-0349-5 |
  | Issue | 4 |
  | Journal Abbr | Early Childhood Educ J |
  | ISSN | 1082-3301, 1573-1707 |
  | Date Added | 11/07/2025, 10:07:52 |
  | Modified | 11/07/2025, 10:07:52 |

  ### Attachments

  - Available Version (via Google Scholar)
- ## Current perspectives on the health effects (mental and physical) of human-animal interaction (HAI)

  |  |  |
  | --- | --- |
  | Item Type | Journal Article |
  | Author | Erika Friedmann |
  | Author | Lincy Koodaly |
  | Date | 2023 |
  | Library Catalogue | Google Scholar |
  | URL | https://www.taylorfrancis.com/chapters/edit/10.4324/9781032153346-5/current-perspectives-health-effects-mental-physical-human-animal-interaction-hai-erika-friedmann-lincy-koodaly |
  | Accessed | 11/07/2025, 10:05:11 |
  | Publisher | Routledge |
  | Pages | 65–78 |
  | Publication | The Routledge International Handbook of Human-Animal Interactions and Anthrozoology |
  | Date Added | 11/07/2025, 10:05:27 |
  | Modified | 11/07/2025, 10:05:27 |
- ## Critical review of research methods used to consider the impact of human–animal interaction on older adults’ health

  |  |  |
  | --- | --- |
  | Item Type | Journal Article |
  | Author | Erika Friedmann |
  | Author | Nancy R. Gee |
  | Date | 2019 |
  | Library Catalogue | Google Scholar |
  | URL | https://academic.oup.com/gerontologist/article-abstract/59/5/964/4969199 |
  | Accessed | 11/07/2025, 10:05:17 |
  | Volume | 59 |
  | Publisher | Oxford University Press US |
  | Pages | 964–972 |
  | Publication | The Gerontologist |
  | Issue | 5 |
  | Date Added | 11/07/2025, 10:05:27 |
  | Modified | 11/07/2025, 10:05:27 |

  ### Attachments

  - Available Version (via Google Scholar)
- ## A critical review of fear tests used on cattle, pigs, sheep, poultry and horses

  |  |  |
  | --- | --- |
  | Item Type | Journal Article |
  | Author | Björn Forkman |
  | Author | Alain Boissy |
  | Author | M.-C. Meunier-Salaün |
  | Author | Elisabetta Canali |
  | Author | Robert Bryan Jones |
  | Date | 2007 |
  | Library Catalogue | Google Scholar |
  | URL | https://www.sciencedirect.com/science/article/pii/S0031938407001096 |
  | Accessed | 11/07/2025, 10:20:00 |
  | Volume | 92 |
  | Publisher | Elsevier |
  | Pages | 340–374 |
  | Publication | Physiology & behavior |
  | Issue | 3 |
  | Date Added | 11/07/2025, 10:20:29 |
  | Modified | 11/07/2025, 10:20:29 |
- ## The effects of animal-assisted therapy on the health and well-being of military veterans: A systematic scoping review and recommendations for future research

  |  |  |
  | --- | --- |
  | Item Type | Journal Article |
  | Author | Brooke Fonseka |
  | Author | Fiona Marshall |
  | Author | Laura J. Edwards |
  | Date | 2022 |
  | Short Title | The effects of animal-assisted therapy on the health and well-being of military veterans |
  | Library Catalogue | Google Scholar |
  | URL | https://docs.lib.purdue.edu/paij/vol5/iss1/12/ |
  | Accessed | 11/07/2025, 10:16:39 |
  | Volume | 5 |
  | Pages | 12 |
  | Publication | People and Animals: The International Journal of Research and Practice |
  | Issue | 1 |
  | Date Added | 11/07/2025, 10:16:47 |
  | Modified | 11/07/2025, 10:16:47 |
- ## Risks and benefits of animal-assisted interventions for critically ill patients admitted to intensive care units

  |  |  |
  | --- | --- |
  | Item Type | Journal Article |
  | Author | Marco Fiore |
  | Author | Andrea Cortegiani |
  | Author | Giansaverio Friolo |
  | Author | Francesca Frigieri Covani |
  | Author | Luigi Cardia |
  | Author | Fausto Ferraro |
  | Author | Daniela Alampi |
  | Date | 2023 |
  | Library Catalogue | Google Scholar |
  | URL | https://link.springer.com/article/10.1186/s44158-023-00100-y |
  | Accessed | 11/07/2025, 10:13:46 |
  | Volume | 3 |
  | Publisher | Springer |
  | Pages | 15 |
  | Publication | Journal of Anesthesia, Analgesia and Critical Care |
  | Issue | 1 |
  | Date Added | 11/07/2025, 10:13:55 |
  | Modified | 11/07/2025, 10:13:55 |

  ### Attachments

  - Available Version (via Google Scholar)
- ## Animal-assisted therapy in patients affected by schizophrenia and schizophrenic-related disorders: A scoping review

  |  |  |
  | --- | --- |
  | Item Type | Journal Article |
  | Author | Martina Finistrella |
  | Author | Paolo Flores |
  | Author | Gianpaolo Frediani |
  | Date | 2024 |
  | Short Title | Animal-assisted therapy in patients affected by schizophrenia and schizophrenic-related disorders |
  | Library Catalogue | Google Scholar |
  | URL | https://hal.science/hal-04567974/ |
  | Accessed | 11/07/2025, 13:46:03 |
  | Volume | 1 |
  | Pages | 53–61 |
  | Publication | Advances in Medicine, Psychology, and Public Health |
  | Issue | 2 |
  | Date Added | 11/07/2025, 13:46:33 |
  | Modified | 11/07/2025, 13:46:33 |

  ### Attachments

  - Available Version (via Google Scholar)
- ## A commentary on the contemporary issues confronting animal assisted and equine assisted interactions

  |  |  |
  | --- | --- |
  | Item Type | Journal Article |
  | Author | Aubrey H. Fine |
  | Author | Sarah J. Andersen |
  | Date | 2021 |
  | Library Catalogue | Google Scholar |
  | URL | https://www.sciencedirect.com/science/article/pii/S0737080621000666 |
  | Accessed | 11/07/2025, 09:27:38 |
  | Volume | 100 |
  | Publisher | Elsevier |
  | Pages | 103436 |
  | Publication | Journal of Equine Veterinary Science |
  | Date Added | 11/07/2025, 09:27:42 |
  | Modified | 11/07/2025, 09:27:42 |
- ## A commentary on the contemporary issues confronting animal assisted and equine assisted interactions

  |  |  |
  | --- | --- |
  | Item Type | Journal Article |
  | Author | Aubrey H. Fine |
  | Author | Sarah J. Andersen |
  | Date | 2021 |
  | Library Catalogue | Google Scholar |
  | URL | https://www.sciencedirect.com/science/article/pii/S0737080621000666 |
  | Accessed | 11/07/2025, 10:09:26 |
  | Volume | 100 |
  | Publisher | Elsevier |
  | Pages | 103436 |
  | Publication | Journal of Equine Veterinary Science |
  | Date Added | 11/07/2025, 10:09:48 |
  | Modified | 11/07/2025, 10:09:48 |
- ## Animal-assisted therapy for dementia: a review of the literature

  |  |  |
  | --- | --- |
  | Item Type | Journal Article |
  | Author | Susan L. Filan |
  | Author | Robert H. Llewellyn-Jones |
  | Date | 2006 |
  | Short Title | Animal-assisted therapy for dementia |
  | Library Catalogue | Google Scholar |
  | URL | https://www.cambridge.org/core/journals/international-psychogeriatrics/article/animalassisted-therapy-for-dementia-a-review-of-the-literature/CA29DC68F280C4A7EFBE67C9BDD0FDD0 |
  | Accessed | 11/07/2025, 10:11:52 |
  | Volume | 18 |
  | Publisher | Cambridge University Press |
  | Pages | 597–611 |
  | Publication | International psychogeriatrics |
  | Issue | 4 |
  | Date Added | 11/07/2025, 10:12:39 |
  | Modified | 11/07/2025, 10:12:39 |

  ### Attachments

  - Available Version (via Google Scholar)
- ## Canine-assisted occupational therapy: a scoping review of the Brazilian literature

  |  |  |
  | --- | --- |
  | Item Type | Journal Article |
  | Author | Mirela de Oliveira Figueiredo |
  | Author | Ana Luiza Alegretti |
  | Author | Lilian Magalhães |
  | Date | 2021 |
  | Short Title | Canine-assisted occupational therapy |
  | Library Catalogue | Google Scholar |
  | URL | https://www.scielo.br/j/cadbto/a/nGz8ch7fyMwvWCGB4rK9GYf/?lang=en |
  | Accessed | 11/07/2025, 11:09:08 |
  | Volume | 29 |
  | Publisher | SciELO Brasil |
  | Pages | e2087 |
  | Publication | Cadernos Brasileiros de Terapia Ocupacional |
  | Date Added | 11/07/2025, 11:09:44 |
  | Modified | 11/07/2025, 11:09:44 |

  ### Attachments

  - Available Version (via Google Scholar)
- ## and Female, Beef and Dairy Cattle

  |  |  |
  | --- | --- |
  | Item Type | Journal Article |
  | Author | Aitor Fernández-Novo |
  | Author | Ángel Revilla-Ruiz |
  | Date | 2024 |
  | Library Catalogue | Google Scholar |
  | URL | https://books.google.com/books?hl=it&lr=&id=5CI7EQAAQBAJ&oi=fnd&pg=PA127&dq=%22human+animal+interaction%22+AND+%22heart+rate%22+&ots=2BMsUyOJGn&sig=cy2wdPAu-y1TXGT9wue\_Ns740aU |
  | Accessed | 11/07/2025, 11:14:38 |
  | Publisher | Springer Nature |
  | Pages | 127 |
  | Publication | Assisted Reproductive Technologies in Animals Volume 1 |
  | Date Added | 11/07/2025, 11:14:45 |
  | Modified | 11/07/2025, 11:14:45 |
- ## and Female, Beef and Dairy Cattle

  |  |  |
  | --- | --- |
  | Item Type | Journal Article |
  | Author | Aitor Fernández-Novo |
  | Author | Ángel Revilla-Ruiz |
  | Date | 2024 |
  | Library Catalogue | Google Scholar |
  | URL | https://books.google.com/books?hl=it&lr=&id=5CI7EQAAQBAJ&oi=fnd&pg=PA127&dq=%22human+animal+interaction%22+AND+%22cortisol%22+&ots=2BMsUzNIKl&sig=8c6njelTEolqY4bP79zTTuuF\_As |
  | Accessed | 11/07/2025, 13:44:01 |
  | Publisher | Springer Nature |
  | Pages | 127 |
  | Publication | Assisted Reproductive Technologies in Animals Volume 1 |
  | Date Added | 11/07/2025, 13:44:05 |
  | Modified | 11/07/2025, 13:44:05 |
- ## Costs and benefits of improving farm animal welfare

  |  |  |
  | --- | --- |
  | Item Type | Journal Article |
  | Author | Jill N. Fernandes |
  | Author | Paul H. Hemsworth |
  | Author | Grahame J. Coleman |
  | Author | Alan J. Tilbrook |
  | Date | 2021 |
  | Library Catalogue | Google Scholar |
  | URL | https://www.mdpi.com/2077-0472/11/2/104 |
  | Accessed | 11/07/2025, 11:08:21 |
  | Volume | 11 |
  | Publisher | MDPI |
  | Pages | 104 |
  | Publication | Agriculture |
  | Issue | 2 |
  | Date Added | 11/07/2025, 11:08:27 |
  | Modified | 11/07/2025, 11:08:27 |
- ## The role of hormones during Equine-Assisted Activity and Therapy: a literature review

  |  |  |
  | --- | --- |
  | Item Type | Journal Article |
  | Author | Adriana Ferlazzo |
  | Author | Esterina Fazio |
  | Author | Cristina Cravana |
  | Author | Pietro Medica |
  | Date | 2021 |
  | Short Title | The role of hormones during Equine-Assisted Activity and Therapy |
  | Library Catalogue | Google Scholar |
  | URL | https://cab.unime.it/journals/index.php/APMB/article/view/3268 |
  | Accessed | 11/07/2025, 10:12:12 |
  | Volume | 109 |
  | Pages | 1–8 |
  | Publication | Atti della Accademia Peloritana dei Pericolanti-Classe di Scienze Medico-Biologiche |
  | Issue | 2 |
  | Date Added | 11/07/2025, 10:12:39 |
  | Modified | 11/07/2025, 10:12:39 |
- ## Effects of animal-assisted therapy on hospitalized children and teenagers: A systematic review and meta-analysis

  |  |  |
  | --- | --- |
  | Item Type | Journal Article |
  | Author | Yongshen Feng |
  | Author | Yeqing Lin |
  | Author | Ningning Zhang |
  | Author | Xiaohan Jiang |
  | Author | Lifeng Zhang |
  | Date | 2021 |
  | Short Title | Effects of animal-assisted therapy on hospitalized children and teenagers |
  | Library Catalogue | Google Scholar |
  | URL | https://www.sciencedirect.com/science/article/pii/S0882596321000233 |
  | Accessed | 11/07/2025, 10:07:38 |
  | Volume | 60 |
  | Publisher | Elsevier |
  | Pages | 11–23 |
  | Publication | Journal of Pediatric Nursing |
  | Date Added | 11/07/2025, 10:07:52 |
  | Modified | 11/07/2025, 10:07:52 |
- ## A bibliometric study for global hotspots and trends in animal-assisted interventions (1983–2023)

  |  |  |
  | --- | --- |
  | Item Type | Journal Article |
  | Author | Xiaowei Feng |
  | Author | Shanguang Zhao |
  | Author | Dong Zhang |
  | Author | Qing Yi |
  | Author | Yanlan Chen |
  | Author | Xinding Zhang |
  | Date | 2025 |
  | Library Catalogue | Google Scholar |
  | URL | https://www.frontiersin.org/journals/psychiatry/articles/10.3389/fpsyt.2025.1490122/full |
  | Accessed | 11/07/2025, 13:46:05 |
  | Volume | 16 |
  | Publisher | Frontiers Media SA |
  | Pages | 1490122 |
  | Publication | Frontiers in Psychiatry |
  | Date Added | 11/07/2025, 13:46:33 |
  | Modified | 11/07/2025, 13:46:33 |

  ### Attachments

  - Available Version (via Google Scholar)
- ## Systematic Review of the Effects of Human-Animal Interactions on Individuals with Developmental Disabilities

  |  |  |
  | --- | --- |
  | Item Type | Journal Article |
  | Author | Jennie Dapice Feinstein |
  | Author | Shelly J. Lane |
  | Author | Sandra Barker |
  | Author | Jennifer McDaniel |
  | Library Catalogue | Google Scholar |
  | URL | https://core.ac.uk/download/pdf/51290502.pdf#page=21 |
  | Accessed | 11/07/2025, 10:09:34 |
  | Pages | 11 |
  | Publication | Examining the Effects of Human-Animal Interaction on Individuals with Developmental Disabilities |
  | Date Added | 11/07/2025, 10:09:48 |
  | Modified | 11/07/2025, 10:09:48 |

  ### Attachments

  - Available Version (via Google Scholar)
- ## Effect of Brief Dog-Assisted Interventions on Stress Biomarkers: A Systematic Review

  |  |  |
  | --- | --- |
  | Item Type | Journal Article |
  | Author | Caroline Faucher |
  | Author | Anna Behler |
  | Author | Megan Campbell |
  | Author | Renate Thienel |
  | Date | 2023 |
  | Short Title | Effect of Brief Dog-Assisted Interventions on Stress Biomarkers |
  | Library Catalogue | Google Scholar |
  | URL | https://www.medrxiv.org/content/10.1101/2023.12.10.23299796.abstract |
  | Accessed | 11/07/2025, 10:09:47 |
  | Publisher | Cold Spring Harbor Laboratory Press |
  | Pages | 2023–12 |
  | Publication | medRxiv |
  | Date Added | 11/07/2025, 10:09:48 |
  | Modified | 11/07/2025, 10:09:48 |

  ### Attachments

  - Available Version (via Google Scholar)
- ## Effect of brief dog-assisted interventions on psychobiological indicators of stress: a systematic review

  |  |  |
  | --- | --- |
  | Item Type | Journal Article |
  | Author | Caroline Faucher |
  | Author | Anna Behler |
  | Author | Megan E. J. Campbell |
  | Author | Renate Thienel |
  | Date | 2025-05-16 |
  | Language | en |
  | Short Title | Effect of brief dog-assisted interventions on psychobiological indicators of stress |
  | Library Catalogue | Crossref |
  | URL | https://www.tandfonline.com/doi/full/10.1080/10615806.2025.2505902 |
  | Accessed | 11/07/2025, 10:16:12 |
  | Rights | http://creativecommons.org/licenses/by-nc-nd/4.0/ |
  | Publisher | Informa UK Limited |
  | Pages | 1-17 |
  | Publication | Anxiety, Stress, & Coping |
  | DOI | 10.1080/10615806.2025.2505902 |
  | ISSN | 1061-5806, 1477-2205 |
  | Date Added | 11/07/2025, 10:16:47 |
  | Modified | 11/07/2025, 10:16:47 |

  ### Attachments

  - Available Version (via Google Scholar)
- ## Dietary strategies for relieving stress in pet dogs and cats

  |  |  |
  | --- | --- |
  | Item Type | Journal Article |
  | Author | Zhicong Fan |
  | Author | Zhaowei Bian |
  | Author | Hongcan Huang |
  | Author | Tingting Liu |
  | Author | Ruiti Ren |
  | Author | Xiaomin Chen |
  | Author | Xiaohe Zhang |
  | Author | Yingjia Wang |
  | Author | Baichuan Deng |
  | Author | Lingna Zhang |
  | Date | 2023 |
  | Library Catalogue | Google Scholar |
  | URL | https://www.mdpi.com/2076-3921/12/3/545 |
  | Accessed | 11/07/2025, 11:08:19 |
  | Volume | 12 |
  | Publisher | MDPI |
  | Pages | 545 |
  | Publication | Antioxidants |
  | Issue | 3 |
  | Date Added | 11/07/2025, 11:08:27 |
  | Modified | 11/07/2025, 11:08:27 |

  ### Attachments

  - Available Version (via Google Scholar)
- ## Animal-Assisted Therapy for the Management of Anxiety in the Hospital Setting: A Systematic Review

  |  |  |
  | --- | --- |
  | Item Type | Journal Article |
  | Author | Leticia Antolín Esteve |
  | Author | Patricia López-Mases |
  | Author | Leticia E. Bartolomé Del Pino |
  | Author | Esther Lázaro |
  | Short Title | Animal-Assisted Therapy for the Management of Anxiety in the Hospital Setting |
  | Library Catalogue | Google Scholar |
  | URL | https://journals.lww.com/hnpjournal/fulltext/9900/animal\_assisted\_therapy\_for\_the\_management\_of.89.aspx |
  | Accessed | 11/07/2025, 10:13:27 |
  | Publisher | LWW |
  | Pages | 10–1097 |
  | Publication | Holistic Nursing Practice |
  | Date Added | 11/07/2025, 10:13:55 |
  | Modified | 11/07/2025, 10:13:55 |
- ## Animal-Assisted Therapy for the Management of Anxiety in the Hospital Setting: A Systematic Review

  |  |  |
  | --- | --- |
  | Item Type | Journal Article |
  | Author | Leticia Antolín Esteve |
  | Author | Patricia López-Mases |
  | Author | Leticia E. Bartolomé Del Pino |
  | Author | Esther Lázaro |
  | Short Title | Animal-Assisted Therapy for the Management of Anxiety in the Hospital Setting |
  | Library Catalogue | Google Scholar |
  | URL | https://journals.lww.com/hnpjournal/fulltext/9900/animal\_assisted\_therapy\_for\_the\_management\_of.89.aspx |
  | Accessed | 11/07/2025, 13:45:55 |
  | Publisher | LWW |
  | Pages | 10–1097 |
  | Publication | Holistic Nursing Practice |
  | Date Added | 11/07/2025, 13:46:33 |
  | Modified | 11/07/2025, 13:46:33 |

  ### Attachments

  - Available Version (via Google Scholar)
- ## Directions in Human-Animal Interaction Research: Child Development, Health, and Therapeutic Interventions

  |  |  |
  | --- | --- |
  | Item Type | Journal Article |
  | Author | Layla Esposito |
  | Author | Sandra McCune |
  | Author | James A. Griffin |
  | Author | Valerie Maholmes |
  | Date | 09/2011 |
  | Language | en |
  | Short Title | Directions in Human-Animal Interaction Research |
  | Library Catalogue | Crossref |
  | URL | https://onlinelibrary.wiley.com/doi/10.1111/j.1750-8606.2011.00175.x |
  | Accessed | 11/07/2025, 10:07:12 |
  | Rights | http://doi.wiley.com/10.1002/tdm\_license\_1.1 |
  | Volume | 5 |
  | Publisher | Wiley |
  | Pages | 205-211 |
  | Publication | Child Development Perspectives |
  | DOI | 10.1111/j.1750-8606.2011.00175.x |
  | Issue | 3 |
  | ISSN | 1750-8592 |
  | Date Added | 11/07/2025, 10:07:52 |
  | Modified | 11/07/2025, 10:07:52 |
- ## The effect of loneliness on depression: A meta-analysis

  |  |  |
  | --- | --- |
  | Item Type | Journal Article |
  | Author | Evren Erzen |
  | Author | Özkan Çikrikci |
  | Abstract | Background: Negative emotions, which have a common, chronic and recurrent structure, play a vital role in the development and maintenance of psychopathology. In this study, loneliness as a negative emotion was considered to be a predisposing factor in depression. Aim: The aim of this meta-analysis is to determine the effect of loneliness on depression. Method: Initially, a literature scan was performed and all related literature was pooled together ( n = 531). Based on scales determined by the researchers, it was decided to include 88 studies in the analysis. This study obtained a sampling group of 40,068 individuals. Results: The results of using a random effects model for analysis showed that loneliness had a moderately significant effect on depression. None of the variables of study sampling group, type of publication and publication year were found to be moderator variables. Conclusion: According to the results of the research, loneliness may be said to be a significant variable affecting depression. The findings obtained are discussed in light of the literature. |
  | Date | 08/2018 |
  | Language | en |
  | Short Title | The effect of loneliness on depression |
  | Library Catalogue | Crossref |
  | URL | https://journals.sagepub.com/doi/10.1177/0020764018776349 |
  | Accessed | 11/07/2025, 11:15:52 |
  | Rights | https://journals.sagepub.com/page/policies/text-and-data-mining-license |
  | Volume | 64 |
  | Publisher | SAGE Publications |
  | Pages | 427-435 |
  | Publication | International Journal of Social Psychiatry |
  | DOI | 10.1177/0020764018776349 |
  | Issue | 5 |
  | Journal Abbr | Int J Soc Psychiatry |
  | ISSN | 0020-7640, 1741-2854 |
  | Date Added | 11/07/2025, 11:15:52 |
  | Modified | 11/07/2025, 11:15:52 |

  ### Attachments

  - Available Version (via Google Scholar)
- ## Human–animal interactions: Effects, challenges, and progress

  |  |  |
  | --- | --- |
  | Item Type | Journal Article |
  | Author | Lily N. Edwards-Callaway |
  | Date | 2018 |
  | Short Title | Human–animal interactions |
  | Library Catalogue | Google Scholar |
  | URL | https://www.sciencedirect.com/science/article/pii/B9780081009383000048 |
  | Accessed | 11/07/2025, 10:04:57 |
  | Publisher | Elsevier |
  | Pages | 71–92 |
  | Publication | Advances in cattle welfare |
  | Date Added | 11/07/2025, 10:05:27 |
  | Modified | 11/07/2025, 10:05:27 |
- ## A Review of Animal-Assisted Interventions in Long-Term Care Facilities

  |  |  |
  | --- | --- |
  | Item Type | Journal Article |
  | Author | Jenna Ebener |
  | Author | Hunhui Oh |
  | Date | 2017-04-03 |
  | Language | en |
  | Library Catalogue | Crossref |
  | URL | https://www.tandfonline.com/doi/full/10.1080/01924788.2017.1306380 |
  | Accessed | 11/07/2025, 10:20:18 |
  | Volume | 41 |
  | Publisher | Informa UK Limited |
  | Pages | 107-128 |
  | Publication | Activities, Adaptation & Aging |
  | DOI | 10.1080/01924788.2017.1306380 |
  | Issue | 2 |
  | ISSN | 0192-4788, 1544-4368 |
  | Date Added | 11/07/2025, 10:20:29 |
  | Modified | 11/07/2025, 10:20:29 |

  ### Attachments

  - Available Version (via Google Scholar)
- ## ANIMAL-ASSISTED THERAPY: A SUBJECT FIELD REVIEW

  |  |  |
  | --- | --- |
  | Item Type | Journal Article |
  | Author | Z. Dyachenko |
  | Date | 2022 |
  | Short Title | ANIMAL-ASSISTED THERAPY |
  | Library Catalogue | Google Scholar |
  | URL | https://elibrary.ru/item.asp?id=50318335 |
  | Accessed | 11/07/2025, 09:31:31 |
  | Date Added | 11/07/2025, 09:32:09 |
  | Modified | 11/07/2025, 09:32:09 |
- ## Robotic psychology: a PRISMA systematic review on social-robot-based interventions in psychological domains

  |  |  |
  | --- | --- |
  | Item Type | Journal Article |
  | Author | Mirko Duradoni |
  | Author | Giulia Colombini |
  | Author | Paola Andrea Russo |
  | Author | Andrea Guazzini |
  | Date | 2021 |
  | Short Title | Robotic psychology |
  | Library Catalogue | Google Scholar |
  | URL | https://www.mdpi.com/2571-8800/4/4/48 |
  | Accessed | 11/07/2025, 09:37:29 |
  | Volume | 4 |
  | Publisher | MDPI |
  | Pages | 664–697 |
  | Publication | J |
  | Issue | 4 |
  | Date Added | 11/07/2025, 09:38:03 |
  | Modified | 11/07/2025, 09:38:03 |
- ## Epidemiology and techno-economical aspect of haemorrhagic septicaemia in India: a review

  |  |  |
  | --- | --- |
  | Item Type | Journal Article |
  | Author | Shreya Dubey |
  | Author | M. N. Brahambhatta |
  | Author | R. V. Singh |
  | Author | Bhavana Gupta |
  | Author | Nidhi Sharma |
  | Author | Renuka Patel |
  | Author | Boreddy Manoj Reddy |
  | Date | 2021 |
  | Short Title | Epidemiology and techno-economical aspect of haemorrhagic septicaemia in India |
  | Library Catalogue | Google Scholar |
  | URL | https://www.researchgate.net/profile/Pugazharasi-Chidambaram/publication/356891784\_Bargur\_Cattle\_An\_Overview/links/61b186074d7ff64f0536968c/Bargur-Cattle-An-Overview.pdf#page=6 |
  | Accessed | 11/07/2025, 11:14:08 |
  | Publication | Reader’s Desk |
  | Date Added | 11/07/2025, 11:14:45 |
  | Modified | 11/07/2025, 11:14:45 |
- ## The human-animal relationship in zoo-housed mammals: Behavioral and physiological responses to visitor and keeper interactions

  |  |  |
  | --- | --- |
  | Item Type | Journal Article |
  | Author | Adriana Domínguez-Oliva |
  | Author | Míriam Marcet-Rius |
  | Author | Carlos Gómez-Medina |
  | Author | Adriana Olmos-Hernández |
  | Author | Quetzalli Gutiérrez |
  | Author | Daniel Mota-Rojas |
  | Date | 2023 |
  | Short Title | The human-animal relationship in zoo-housed mammals |
  | Library Catalogue | Google Scholar |
  | URL | https://malque.pub/ojs/index.php/jabb/article/view/1963 |
  | Accessed | 11/07/2025, 09:37:51 |
  | Volume | 11 |
  | Publication | Journal of Animal Behaviour and Biometeorology |
  | Date Added | 11/07/2025, 09:38:03 |
  | Modified | 11/07/2025, 09:38:03 |

  ### Attachments

  - Available Version (via Google Scholar)
- ## Stress and Disease in Domestic Animals

  |  |  |
  | --- | --- |
  | Item Type | Journal Article |
  | Author | Laszlo DeROTH |
  | Date | 10/1993 |
  | Language | en |
  | Library Catalogue | Crossref |
  | URL | https://nyaspubs.onlinelibrary.wiley.com/doi/10.1111/j.1749-6632.1993.tb49942.x |
  | Accessed | 11/07/2025, 10:13:25 |
  | Rights | http://onlinelibrary.wiley.com/termsAndConditions#vor |
  | Volume | 697 |
  | Publisher | Wiley |
  | Pages | 285-292 |
  | Publication | Annals of the New York Academy of Sciences |
  | DOI | 10.1111/j.1749-6632.1993.tb49942.x |
  | Issue | 1 |
  | ISSN | 0077-8923, 1749-6632 |
  | Date Added | 11/07/2025, 10:13:55 |
  | Modified | 11/07/2025, 10:13:55 |
- ## The role of the human-canine bond in recovery from substance use disorder: A scoping review and narrative synthesis protocol

  |  |  |
  | --- | --- |
  | Item Type | Journal Article |
  | Author | Colleen Dell |
  | Author | Brynn Kosteniuk |
  | Author | Carolyn Doi |
  | Author | Darlene Chalmers |
  | Author | Peter Butt |
  | Date | 2023 |
  | Short Title | The role of the human-canine bond in recovery from substance use disorder |
  | Library Catalogue | Google Scholar |
  | URL | https://www.cabidigitallibrary.org/doi/abs/10.1079/hai.2023.0029 |
  | Accessed | 11/07/2025, 10:12:25 |
  | Publisher | CABI Wallingford UK |
  | Publication | Human-Animal Interactions |
  | Issue | 2023 |
  | Date Added | 11/07/2025, 10:12:39 |
  | Modified | 11/07/2025, 10:12:39 |
- ## The role of the human-canine bond in recovery from substance use disorder: A scoping review and narrative synthesis

  |  |  |
  | --- | --- |
  | Item Type | Journal Article |
  | Author | Colleen Dell |
  | Author | Brynn Kosteniuk |
  | Author | Carolyn Doi |
  | Author | Courtney Townsend |
  | Author | Alexis Cook |
  | Author | Darlene Chalmers |
  | Author | Peter Butt |
  | Abstract | Abstract Recovery from substance use disorder (SUD) is a personal journey that includes connection with self and others, including animals – known as the human-animal bond (HAB). Research shows that canines are the most common type of animal integrated into animal-assisted interventions to support people with SUD. Yet, to our knowledge, there has been no review of the evidence on the role of canines in SUD recovery. The scoping review’s objective was to examine the literature on the human-canine bond’s role in recovery from SUD among adolescents and adults, including how the bond may help or hinder recovery. The review considered records that described the human-canine bond with respect to recovery in any recovery- or therapy-related setting globally. Eleven databases were searched, and 32 sources met inclusion criteria that involved companion dogs, therapy dogs, service/assistance, dogs and others. The thematic analysis across records identified three key benefits of the human-canine bond in SUD recovery: (1) a source of social connection and a conduit for human-to-human social connection, (2) a calming and comforting effect on individuals with SUD that can reduce stress and anxiety, and (3) the human-canine bond as a motivating factor for positive change. Through these themes, the bond may help divert substance use-related thoughts and reduce cravings, bolster engagement in treatment and recovery, and help to decrease and prevent substance use. However, a few articles found no role or a limited role of the human-canine bond in recovery, and challenges and considerations were reported, particularly for marginalized populations (e.g., related to obtaining and maintaining housing, employment, and SUD treatment). Most of the records discussed canine welfare in some capacity. Calls were also made for improved policy, public awareness, and animal welfare. |
  | Date | 2024-10-09 |
  | Language | en |
  | Short Title | The role of the human-canine bond in recovery from substance use disorder |
  | Library Catalogue | Crossref |
  | URL | http://www.cabidigitallibrary.org/doi/10.1079/hai.2024.0038 |
  | Accessed | 11/07/2025, 10:20:11 |
  | Publisher | CABI Publishing |
  | Publication | Human-Animal Interactions |
  | DOI | 10.1079/hai.2024.0038 |
  | ISSN | 2957-9538 |
  | Date Added | 11/07/2025, 10:20:29 |
  | Modified | 11/07/2025, 10:20:29 |
- ## Animal-assisted therapy: Evaluation and implementation of a complementary therapy to improve the psychological and physiological health of critically ill patients

  |  |  |
  | --- | --- |
  | Item Type | Journal Article |
  | Author | Mary DeCourcey |
  | Author | Anne C. Russell |
  | Author | Kathy J. Keister |
  | Date | 2010 |
  | Short Title | Animal-assisted therapy |
  | Library Catalogue | Google Scholar |
  | URL | https://journals.lww.com/dccnjournal/FullText/2010/09000/Animal\_Assisted\_Therapy\_\_Evaluation\_and.4.aspx |
  | Accessed | 11/07/2025, 10:07:44 |
  | Volume | 29 |
  | Publisher | LWW |
  | Pages | 211–214 |
  | Publication | Dimensions of critical care nursing |
  | Issue | 5 |
  | Date Added | 11/07/2025, 10:07:52 |
  | Modified | 11/07/2025, 10:07:52 |
- ## Invited review: Associations between variables of routine herd data and dairy cattle welfare indicators

  |  |  |
  | --- | --- |
  | Item Type | Journal Article |
  | Author | M. De Vries |
  | Author | E. A. M. Bokkers |
  | Author | T. Dijkstra |
  | Author | G. Van Schaik |
  | Author | I. J. M. De Boer |
  | Date | 2011 |
  | Short Title | Invited review |
  | Library Catalogue | Google Scholar |
  | URL | https://www.sciencedirect.com/science/article/pii/S0022030211003146 |
  | Accessed | 11/07/2025, 09:36:03 |
  | Volume | 94 |
  | Publisher | Elsevier |
  | Pages | 3213–3228 |
  | Publication | Journal of Dairy Science |
  | Issue | 7 |
  | Date Added | 11/07/2025, 09:36:11 |
  | Modified | 11/07/2025, 09:36:11 |
- ## How to Measure Human-Dog Interaction in Dog Assisted Interventions? A Scoping Review

  |  |  |
  | --- | --- |
  | Item Type | Journal Article |
  | Author | Marta De Santis |
  | Author | Lorena Filugelli |
  | Author | Alberto Mair |
  | Author | Simona Normando |
  | Author | Franco Mutinelli |
  | Author | Laura Contalbrigo |
  | Date | 2024 |
  | Short Title | How to Measure Human-Dog Interaction in Dog Assisted Interventions? |
  | Library Catalogue | Google Scholar |
  | URL | https://www.mdpi.com/2076-2615/14/3/410 |
  | Accessed | 11/07/2025, 09:27:05 |
  | Volume | 14 |
  | Publisher | MDPI |
  | Pages | 410 |
  | Publication | Animals |
  | Issue | 3 |
  | Date Added | 11/07/2025, 09:27:42 |
  | Modified | 11/07/2025, 09:27:42 |
- ## Equine Assisted Interventions (EAIs): Methodological considerations for stress assessment in horses

  |  |  |
  | --- | --- |
  | Item Type | Journal Article |
  | Author | Marta De Santis |
  | Author | Laura Contalbrigo |
  | Author | Marta Borgi |
  | Author | Francesca Cirulli |
  | Author | Fabio Luzi |
  | Author | Veronica Redaelli |
  | Author | Annalisa Stefani |
  | Author | Marica Toson |
  | Author | Rosangela Odore |
  | Author | Cristina Vercelli |
  | Date | 2017 |
  | Short Title | Equine Assisted Interventions (EAIs) |
  | Library Catalogue | Google Scholar |
  | URL | https://www.mdpi.com/2306-7381/4/3/44 |
  | Accessed | 11/07/2025, 09:28:50 |
  | Volume | 4 |
  | Publisher | MDPI |
  | Pages | 44 |
  | Publication | Veterinary sciences |
  | Issue | 3 |
  | Date Added | 11/07/2025, 09:29:28 |
  | Modified | 11/07/2025, 09:29:28 |

  ### Attachments

  - Available Version (via Google Scholar)
  - Available Version (via Google Scholar)
- ## Equine Assisted Interventions (EAIs): Methodological considerations for stress assessment in horses

  |  |  |
  | --- | --- |
  | Item Type | Journal Article |
  | Author | Marta De Santis |
  | Author | Laura Contalbrigo |
  | Author | Marta Borgi |
  | Author | Francesca Cirulli |
  | Author | Fabio Luzi |
  | Author | Veronica Redaelli |
  | Author | Annalisa Stefani |
  | Author | Marica Toson |
  | Author | Rosangela Odore |
  | Author | Cristina Vercelli |
  | Date | 2017 |
  | Short Title | Equine Assisted Interventions (EAIs) |
  | Library Catalogue | Google Scholar |
  | URL | https://www.mdpi.com/2306-7381/4/3/44 |
  | Accessed | 11/07/2025, 10:12:08 |
  | Volume | 4 |
  | Publisher | MDPI |
  | Pages | 44 |
  | Publication | Veterinary sciences |
  | Issue | 3 |
  | Date Added | 11/07/2025, 10:12:39 |
  | Modified | 11/07/2025, 10:12:39 |
- ## Review-Relational enrichment in ruminants and equines

  |  |  |
  | --- | --- |
  | Item Type | Journal Article |
  | Author | D. de Oliveira |
  | Author | X. Boivin |
  | Date | 2024 |
  | Library Catalogue | Google Scholar |
  | URL | https://www.eurcaw-ruminants-equines.eu/wp-content/uploads/2024/07/REV-Ruminants-Equines-2023-04-EN.pdf |
  | Accessed | 11/07/2025, 13:46:15 |
  | Publisher | EURCAW |
  | Date Added | 11/07/2025, 13:46:33 |
  | Modified | 11/07/2025, 13:46:33 |

  ### Attachments

  - Available Version (via Google Scholar)
- ## INTERAÇÃO HOMEM-ANIMAL: UMA REVISÃO DE LITERATURA SOBRE OS BENEFÍCIOS TERAPÊUTICOS DA ZOOTERAPIA NA PSICOLOGIA

  |  |  |
  | --- | --- |
  | Item Type | Journal Article |
  | Author | Daniela Barbosa de Macedo |
  | Author | Clauber Rosanova |
  | Author | Geovanne Ferreira Rebouças |
  | Author | Raquel Marcon Gomes |
  | Date | 2024 |
  | Short Title | INTERAÇÃO HOMEM-ANIMAL |
  | Library Catalogue | Google Scholar |
  | URL | http://revista.unipacto.com.br/index.php/multidisciplinar/article/view/2607 |
  | Accessed | 11/07/2025, 13:46:23 |
  | Volume | 7 |
  | Publication | Revista Multidisciplinar do Nordeste Mineiro |
  | Issue | 1 |
  | Date Added | 11/07/2025, 13:46:33 |
  | Modified | 11/07/2025, 13:46:33 |

  ### Attachments

  - Available Version (via Google Scholar)
- ## Animal assisted interventions for children with autism spectrum disorder: A systematic review

  |  |  |
  | --- | --- |
  | Item Type | Journal Article |
  | Author | Tonya N. Davis |
  | Author | Rachel Scalzo |
  | Author | Erin Butler |
  | Author | Megan Stauffer |
  | Author | Yara N. Farah |
  | Author | Scott Perez |
  | Author | Kristen Mainor |
  | Author | Cathryn Clark |
  | Author | Stacy Miller |
  | Author | Alicia Kobylecky |
  | Date | 2015 |
  | Short Title | Animal assisted interventions for children with autism spectrum disorder |
  | Library Catalogue | Google Scholar |
  | URL | https://www.jstor.org/stable/24827513 |
  | Accessed | 11/07/2025, 09:31:35 |
  | Publisher | JSTOR |
  | Pages | 316–329 |
  | Publication | Education and Training in Autism and Developmental Disabilities |
  | Date Added | 11/07/2025, 09:32:09 |
  | Modified | 11/07/2025, 09:32:09 |

  ### Attachments

  - Available Version (via Google Scholar)
- ## Investigating the impact of pre-slaughter management factors on indicators of fed beef cattle welfare–a scoping review

  |  |  |
  | --- | --- |
  | Item Type | Journal Article |
  | Author | Melissa Davis |
  | Author | Paxton Sullivan |
  | Author | Jimena Bretón |
  | Author | Lauren Dean |
  | Author | Lily Edwards-Callaway |
  | Date | 2022 |
  | Library Catalogue | Google Scholar |
  | URL | https://www.frontiersin.org/articles/10.3389/fanim.2022.1073849/full |
  | Accessed | 11/07/2025, 09:36:09 |
  | Volume | 3 |
  | Publisher | Frontiers Media SA |
  | Pages | 1073849 |
  | Publication | Frontiers in Animal Science |
  | Date Added | 11/07/2025, 09:36:11 |
  | Modified | 11/07/2025, 09:36:11 |

  ### Attachments

  - Available Version (via Google Scholar)
- ## Investigating the impact of pre-slaughter management factors on indicators of fed beef cattle welfare–a scoping review

  |  |  |
  | --- | --- |
  | Item Type | Journal Article |
  | Author | Melissa Davis |
  | Author | Paxton Sullivan |
  | Author | Jimena Bretón |
  | Author | Lauren Dean |
  | Author | Lily Edwards-Callaway |
  | Date | 2022 |
  | Library Catalogue | Google Scholar |
  | URL | https://www.frontiersin.org/articles/10.3389/fanim.2022.1073849/full |
  | Accessed | 11/07/2025, 09:58:08 |
  | Volume | 3 |
  | Publisher | Frontiers Media SA |
  | Pages | 1073849 |
  | Publication | Frontiers in Animal Science |
  | Date Added | 11/07/2025, 09:58:25 |
  | Modified | 11/07/2025, 09:58:25 |

  ### Attachments

  - Available Version (via Google Scholar)
- ## Animal assisted interventions for children with autism spectrum disorder: A systematic review

  |  |  |
  | --- | --- |
  | Item Type | Journal Article |
  | Author | Tonya N. Davis |
  | Author | Rachel Scalzo |
  | Author | Erin Butler |
  | Author | Megan Stauffer |
  | Author | Yara N. Farah |
  | Author | Scott Perez |
  | Author | Kristen Mainor |
  | Author | Cathryn Clark |
  | Author | Stacy Miller |
  | Author | Alicia Kobylecky |
  | Date | 2015 |
  | Short Title | Animal assisted interventions for children with autism spectrum disorder |
  | Library Catalogue | Google Scholar |
  | URL | https://www.jstor.org/stable/24827513 |
  | Accessed | 11/07/2025, 10:17:57 |
  | Publisher | JSTOR |
  | Pages | 316–329 |
  | Publication | Education and Training in Autism and Developmental Disabilities |
  | Date Added | 11/07/2025, 10:18:17 |
  | Modified | 11/07/2025, 10:18:17 |

  ### Attachments

  - Available Version (via Google Scholar)
- ## Where do we stand in the domestic dog (Canis familiaris) positive-emotion assessment: A state-of-the-art review and future directions

  |  |  |
  | --- | --- |
  | Item Type | Journal Article |
  | Author | Erika Csoltova |
  | Author | Emira Mehinagic |
  | Date | 2020 |
  | Short Title | Where do we stand in the domestic dog (Canis familiaris) positive-emotion assessment |
  | Library Catalogue | Google Scholar |
  | URL | https://www.frontiersin.org/articles/10.3389/fpsyg.2020.02131/full |
  | Accessed | 11/07/2025, 10:00:35 |
  | Volume | 11 |
  | Publisher | Frontiers Media SA |
  | Pages | 2131 |
  | Publication | Frontiers in psychology |
  | Date Added | 11/07/2025, 10:00:41 |
  | Modified | 11/07/2025, 10:00:41 |

  ### Attachments

  - Available Version (via Google Scholar)
- ## Pediatric oncology and animal-assisted interventions: A systematic review

  |  |  |
  | --- | --- |
  | Item Type | Journal Article |
  | Author | Crina Cotoc |
  | Author | Ruopeng An |
  | Author | Hillary Klonoff-Cohen |
  | Date | 2019 |
  | Short Title | Pediatric oncology and animal-assisted interventions |
  | Library Catalogue | Google Scholar |
  | URL | https://journals.lww.com/hnpjournal/fulltext/2019/03000/pediatric\_oncology\_and\_animal\_assisted.6.aspx |
  | Accessed | 11/07/2025, 10:20:20 |
  | Volume | 33 |
  | Publisher | LWW |
  | Pages | 101–110 |
  | Publication | Holistic nursing practice |
  | Issue | 2 |
  | Date Added | 11/07/2025, 10:20:29 |
  | Modified | 11/07/2025, 10:20:29 |
- ## Race, zoonoses and animal assisted interventions in pediatric cancer

  |  |  |
  | --- | --- |
  | Item Type | Journal Article |
  | Author | Crina Cotoc |
  | Author | Stephen Notaro |
  | Date | 2022 |
  | Library Catalogue | Google Scholar |
  | URL | https://www.mdpi.com/1660-4601/19/13/7772 |
  | Accessed | 11/07/2025, 10:13:37 |
  | Volume | 19 |
  | Publisher | MDPI |
  | Pages | 7772 |
  | Publication | International Journal of Environmental Research and Public Health |
  | Issue | 13 |
  | Date Added | 11/07/2025, 10:13:55 |
  | Modified | 11/07/2025, 10:13:55 |
- ## Improving the emotional distress and the experience of hospitalization in children and adolescent patients through animal assisted interventions: a systematic review

  |  |  |
  | --- | --- |
  | Item Type | Journal Article |
  | Author | Cinzia Correale |
  | Author | Marta Borgi |
  | Author | Barbara Collacchi |
  | Author | Chiara Falamesca |
  | Author | Simonetta Gentile |
  | Author | Federico Vigevano |
  | Author | Simona Cappelletti |
  | Author | Francesca Cirulli |
  | Date | 2022 |
  | Short Title | Improving the emotional distress and the experience of hospitalization in children and adolescent patients through animal assisted interventions |
  | Library Catalogue | Google Scholar |
  | URL | https://www.frontiersin.org/articles/10.3389/fpsyg.2022.840107/full |
  | Accessed | 11/07/2025, 09:28:52 |
  | Volume | 13 |
  | Publisher | Frontiers Media SA |
  | Pages | 840107 |
  | Publication | Frontiers in psychology |
  | Date Added | 11/07/2025, 09:29:28 |
  | Modified | 11/07/2025, 09:29:28 |

  ### Attachments

  - Available Version (via Google Scholar)
- ## Improving the emotional distress and the experience of hospitalization in children and adolescent patients through animal assisted interventions: a systematic review

  |  |  |
  | --- | --- |
  | Item Type | Journal Article |
  | Author | Cinzia Correale |
  | Author | Marta Borgi |
  | Author | Barbara Collacchi |
  | Author | Chiara Falamesca |
  | Author | Simonetta Gentile |
  | Author | Federico Vigevano |
  | Author | Simona Cappelletti |
  | Author | Francesca Cirulli |
  | Date | 2022 |
  | Short Title | Improving the emotional distress and the experience of hospitalization in children and adolescent patients through animal assisted interventions |
  | Library Catalogue | Google Scholar |
  | URL | https://www.frontiersin.org/articles/10.3389/fpsyg.2022.840107/full |
  | Accessed | 11/07/2025, 10:16:30 |
  | Volume | 13 |
  | Publisher | Frontiers Media SA |
  | Pages | 840107 |
  | Publication | Frontiers in psychology |
  | Date Added | 11/07/2025, 10:16:47 |
  | Modified | 11/07/2025, 10:16:47 |
- ## The neurobiology of behavior and its applicability for animal welfare: A review

  |  |  |
  | --- | --- |
  | Item Type | Journal Article |
  | Author | Genaro A. Coria-Avila |
  | Author | James G. Pfaus |
  | Author | Agustín Orihuela |
  | Author | Adriana Domínguez-Oliva |
  | Author | Nancy José-Pérez |
  | Author | Laura Astrid Hernández |
  | Author | Daniel Mota-Rojas |
  | Date | 2022 |
  | Short Title | The neurobiology of behavior and its applicability for animal welfare |
  | Library Catalogue | Google Scholar |
  | URL | https://www.mdpi.com/2076-2615/12/7/928 |
  | Accessed | 11/07/2025, 10:20:12 |
  | Volume | 12 |
  | Publisher | MDPI |
  | Pages | 928 |
  | Publication | Animals |
  | Issue | 7 |
  | Date Added | 11/07/2025, 10:20:29 |
  | Modified | 11/07/2025, 10:20:29 |

  ### Attachments

  - Available Version (via Google Scholar)
- ## Terapia assistida por animais combinada à terapia cognitivo comportamental: revisão sistemática de literatura

  |  |  |
  | --- | --- |
  | Item Type | Journal Article |
  | Author | Vitória Ermel Córdova |
  | Date | 2021 |
  | Short Title | Terapia assistida por animais combinada à terapia cognitivo comportamental |
  | Library Catalogue | Google Scholar |
  | URL | https://lume.ufrgs.br/handle/10183/229629 |
  | Accessed | 11/07/2025, 11:14:16 |
  | Date Added | 11/07/2025, 11:14:45 |
  | Modified | 11/07/2025, 11:14:45 |
- ## Canine-assisted Therapy as an Adjunct Tool in the Care of the Surgical Patient: A Literature Review and Opportunity for Research.

  |  |  |
  | --- | --- |
  | Item Type | Journal Article |
  | Author | Lauren Folgosa Cooley |
  | Author | Sandra B. Barker |
  | Date | 2018 |
  | Short Title | Canine-assisted Therapy as an Adjunct Tool in the Care of the Surgical Patient |
  | Library Catalogue | Google Scholar |
  | URL | https://search.ebscohost.com/login.aspx?direct=true&profile=ehost&scope=site&authtype=crawler&jrnl=10786791&AN=130886599&h=gaaNFsCzeWxtWovY%2BjNInD7aMMyciq2YC6tJ4J6MNkUIay%2BNvW739slMVPzyaz1vvJPF42YJ%2BitgTeK2ceoOCw%3D%3D&crl=c |
  | Accessed | 11/07/2025, 10:09:24 |
  | Volume | 24 |
  | Publication | Alternative Therapies in Health & Medicine |
  | Issue | 3 |
  | Date Added | 11/07/2025, 10:09:48 |
  | Modified | 11/07/2025, 10:09:48 |
- ## Animal-assisted interventions in universities: A scoping review of implementation and associated outcomes

  |  |  |
  | --- | --- |
  | Item Type | Journal Article |
  | Author | Emily Cooke |
  | Author | Claire Henderson-Wilson |
  | Author | Elyse Warner |
  | Author | Anthony LaMontagne |
  | Date | 2023 |
  | Short Title | Animal-assisted interventions in universities |
  | Library Catalogue | Google Scholar |
  | URL | https://academic.oup.com/heapro/article-abstract/38/3/daac001/6517178 |
  | Accessed | 11/07/2025, 09:35:52 |
  | Volume | 38 |
  | Publisher | Oxford University Press |
  | Pages | daac001 |
  | Publication | Health promotion international |
  | Issue | 3 |
  | Date Added | 11/07/2025, 09:36:11 |
  | Modified | 11/07/2025, 09:36:11 |

  ### Attachments

  - Available Version (via Google Scholar)
- ## EMPATHY TOWARDS NON-HUMAN ANIMALS: ITS ROLE IN EMOTION RECOGNITION, VETERINARY MEDICINE AND ANIMAL HOARDING DISORDER.

  |  |  |
  | --- | --- |
  | Item Type | Journal Article |
  | Author | ELISA SILVIA Colombo |
  | Date | 2015 |
  | Short Title | EMPATHY TOWARDS NON-HUMAN ANIMALS |
  | Library Catalogue | Google Scholar |
  | URL | https://tesidottorato.depositolegale.it/handle/20.500.14242/174321 |
  | Accessed | 11/07/2025, 09:27:32 |
  | Publisher | Università degli Studi di Milano |
  | Date Added | 11/07/2025, 09:27:42 |
  | Modified | 11/07/2025, 09:27:42 |
- ## EMPATHY TOWARDS NON-HUMAN ANIMALS: ITS ROLE IN EMOTION RECOGNITION, VETERINARY MEDICINE AND ANIMAL HOARDING DISORDER.

  |  |  |
  | --- | --- |
  | Item Type | Journal Article |
  | Author | ELISA SILVIA Colombo |
  | Date | 2015 |
  | Short Title | EMPATHY TOWARDS NON-HUMAN ANIMALS |
  | Library Catalogue | Google Scholar |
  | URL | https://tesidottorato.depositolegale.it/handle/20.500.14242/174321 |
  | Accessed | 11/07/2025, 10:07:40 |
  | Publisher | Università degli Studi di Milano |
  | Date Added | 11/07/2025, 10:07:52 |
  | Modified | 11/07/2025, 10:07:52 |
- ## The animal welfare science of working dogs: current perspectives on recent advances and future directions

  |  |  |
  | --- | --- |
  | Item Type | Journal Article |
  | Author | Mia L. Cobb |
  | Author | Cynthia M. Otto |
  | Author | Aubrey H. Fine |
  | Date | 2021 |
  | Short Title | The animal welfare science of working dogs |
  | Library Catalogue | Google Scholar |
  | URL | https://www.frontiersin.org/articles/10.3389/fvets.2021.666898/full |
  | Accessed | 11/07/2025, 09:29:28 |
  | Volume | 8 |
  | Publisher | Frontiers Media SA |
  | Pages | 666898 |
  | Publication | Frontiers in veterinary science |
  | Date Added | 11/07/2025, 09:29:28 |
  | Modified | 11/07/2025, 09:29:28 |

  ### Attachments

  - Available Version (via Google Scholar)
- ## A systematic review and meta-analysis of salivary cortisol measurement in domestic canines

  |  |  |
  | --- | --- |
  | Item Type | Journal Article |
  | Author | M. L. Cobb |
  | Author | K. Iskandarani |
  | Author | V. M. Chinchilli |
  | Author | N. A. Dreschel |
  | Date | 2016 |
  | Library Catalogue | Google Scholar |
  | URL | https://www.sciencedirect.com/science/article/pii/S0739724016300418 |
  | Accessed | 11/07/2025, 09:31:53 |
  | Volume | 57 |
  | Publisher | Elsevier |
  | Pages | 31–42 |
  | Publication | Domestic Animal Endocrinology |
  | Date Added | 11/07/2025, 09:32:09 |
  | Modified | 11/07/2025, 09:32:09 |
- ## The Animal Welfare Science of Working Dogs: Current Perspectives on Recent Advances and Future Directions. Front. Vet. Sci. 8: 666898. doi: 10.3389/fvets. 2021.666898

  |  |  |
  | --- | --- |
  | Item Type | Journal Article |
  | Author | M. L. Cobb |
  | Author | C. M. Otto |
  | Author | A. H. Fine |
  | Date | 2021 |
  | Short Title | The Animal Welfare Science of Working Dogs |
  | Library Catalogue | Google Scholar |
  | URL | https://minerva-access.unimelb.edu.au/bitstream/handle/11343/289466/fvets-08-666898.pdf?sequence=3&isAllowed=y |
  | Accessed | 11/07/2025, 09:35:58 |
  | Extra | Publisher: The Animal Welfare Science of Working Dogs: Current Perspectives on Recent … |
  | Date Added | 11/07/2025, 09:36:11 |
  | Modified | 11/07/2025, 09:36:11 |
- ## The Animal Welfare Science of Working Dogs: Current Perspectives on Recent Advances and Future Directions. Front. Vet. Sci. 8: 666898. doi: 10.3389/fvets. 2021.666898

  |  |  |
  | --- | --- |
  | Item Type | Journal Article |
  | Author | M. L. Cobb |
  | Author | C. M. Otto |
  | Author | A. H. Fine |
  | Date | 2021 |
  | Short Title | The Animal Welfare Science of Working Dogs |
  | Library Catalogue | Google Scholar |
  | URL | https://minerva-access.unimelb.edu.au/bitstream/handle/11343/289466/fvets-08-666898.pdf?sequence=3&isAllowed=y |
  | Accessed | 11/07/2025, 11:09:22 |
  | Extra | Publisher: The Animal Welfare Science of Working Dogs: Current Perspectives on Recent … |
  | Date Added | 11/07/2025, 11:09:44 |
  | Modified | 11/07/2025, 11:09:44 |
- ## Obesity in the Dog and Cat

  |  |  |
  | --- | --- |
  | Item Type | Journal Article |
  | Author | Martha G. Cline |
  | Author | Maryanne Murphy |
  | Date | 2019 |
  | Library Catalogue | Google Scholar |
  | URL | https://books.google.com/books?hl=it&lr=&id=VceWDwAAQBAJ&oi=fnd&pg=PT6&dq=%22human+animal+interaction%22+AND+%22heart+rate%22+&ots=C1CcrZL6bB&sig=DhMENkSGsK6wuc-4PrYVGj8LELw |
  | Accessed | 11/07/2025, 11:11:46 |
  | Publisher | CRC Press |
  | Date Added | 11/07/2025, 11:12:01 |
  | Modified | 11/07/2025, 11:12:01 |
- ## The effects of interacting with fish in aquariums on human health and well-being: A systematic review

  |  |  |
  | --- | --- |
  | Item Type | Journal Article |
  | Author | Heather Clements |
  | Author | Stephanie Valentin |
  | Author | Nicholas Jenkins |
  | Author | Jean Rankin |
  | Author | Julien S. Baker |
  | Author | Nancy Gee |
  | Author | Donna Snellgrove |
  | Author | Katherine Sloman |
  | Date | 2019 |
  | Short Title | The effects of interacting with fish in aquariums on human health and well-being |
  | Library Catalogue | Google Scholar |
  | URL | https://journals.plos.org/plosone/article?id=10.1371/journal.pone.0220524 |
  | Accessed | 11/07/2025, 10:09:12 |
  | Volume | 14 |
  | Publisher | Public Library of Science San Francisco, CA USA |
  | Pages | e0220524 |
  | Publication | PloS one |
  | Issue | 7 |
  | Date Added | 11/07/2025, 10:09:48 |
  | Modified | 11/07/2025, 10:09:48 |

  ### Attachments

  - Available Version (via Google Scholar)
- ## Gaps in the evidence about companion animals and human health: Some suggestions for progress

  |  |  |
  | --- | --- |
  | Item Type | Journal Article |
  | Author | Anna Chur-Hansen |
  | Author | Cindy Stern |
  | Author | Helen Winefield |
  | Date | 2010 |
  | Short Title | Gaps in the evidence about companion animals and human health |
  | Library Catalogue | Google Scholar |
  | URL | https://journals.lww.com/ijebh/fulltext/2010/09000/gaps\_in\_the\_evidence\_about\_companion\_animals\_and.5.aspx |
  | Accessed | 11/07/2025, 10:12:22 |
  | Volume | 8 |
  | Publisher | LWW |
  | Pages | 140–146 |
  | Publication | JBI Evidence Implementation |
  | Issue | 3 |
  | Date Added | 11/07/2025, 10:12:39 |
  | Modified | 11/07/2025, 10:12:39 |
- ## Animal‐Assisted Therapy as an Intervention for Older Adults: A Systematic Review and Meta‐Analysis to Guide Evidence‐Based Practice

  |  |  |
  | --- | --- |
  | Item Type | Journal Article |
  | Author | Sun Ju Chang |
  | Author | Jongeun Lee |
  | Author | Hyeran An |
  | Author | Woi‐Hyun Hong |
  | Author | Joo Yun Lee |
  | Abstract | AbstractBackgroundAnimal‐assisted therapy (AAT) can ameliorate diverse health problems in older adults. However, applications of AAT have been limited because of the lack of intervention guidelines for older adults.AimsThis study aimed to explore applications of AAT to older adults, analyze its health effects, and provide evidence for future interventions.MethodsA systematic review and meta‐analysis were conducted based on the Preferred Reporting Items for Systematic Reviews and Meta‐Analyses checklist. Data were analyzed based on both a narrative synthesis and a meta‐analysis specifically for depression.ResultsA total of 47 studies were selected for analysis. About 45% focused on older adults with diseases such as dementia, and 57.4% selected dog(s) as an intervention animal. About 34.0% delivered interventions once a week, and the behavioral outcome domain was the most frequently investigated. The meta‐analysis showed that the effect sizes of the AAT group were −1.310 (95% CI [−1.900, −.721]).Linking Evidence to ActionThis review provides evidence for AAT as an intervention in the physiological, psychosocial, cognitive, and behavioral domains of older adults. When planning interventions for older adults, nurses should consider intended health outcomes, appropriate therapeutic animals, and the consequent intervention contents. |
  | Date | 02/2021 |
  | Language | en |
  | Short Title | Animal‐Assisted Therapy as an Intervention for Older Adults |
  | Library Catalogue | Crossref |
  | URL | https://sigmapubs.onlinelibrary.wiley.com/doi/10.1111/wvn.12484 |
  | Accessed | 11/07/2025, 10:16:17 |
  | Rights | http://onlinelibrary.wiley.com/termsAndConditions#vor |
  | Volume | 18 |
  | Publisher | Wiley |
  | Pages | 60-67 |
  | Publication | Worldviews on Evidence-Based Nursing |
  | DOI | 10.1111/wvn.12484 |
  | Issue | 1 |
  | Journal Abbr | Worldviews Ev Based Nurs |
  | ISSN | 1545-102X, 1741-6787 |
  | Date Added | 11/07/2025, 10:16:47 |
  | Modified | 11/07/2025, 10:16:47 |

  ### Attachments

  - Available Version (via Google Scholar)
- ## Incorporating temperament traits in dairy cattle breeding programs: challenges and opportunities in the phenomics era

  |  |  |
  | --- | --- |
  | Item Type | Journal Article |
  | Author | Yao Chang |
  | Author | Luiz F. Brito |
  | Author | Amanda B. Alvarenga |
  | Author | Yachun Wang |
  | Date | 2020 |
  | Short Title | Incorporating temperament traits in dairy cattle breeding programs |
  | Library Catalogue | Google Scholar |
  | URL | https://academic.oup.com/af/article-abstract/10/2/29/5814667 |
  | Accessed | 11/07/2025, 10:18:09 |
  | Volume | 10 |
  | Publisher | Oxford University Press US |
  | Pages | 29–36 |
  | Publication | Animal Frontiers |
  | Issue | 2 |
  | Date Added | 11/07/2025, 10:18:17 |
  | Modified | 11/07/2025, 10:18:17 |
- ## A review of studies conducted with animal assisted interventions for children with autism spectrum disorder

  |  |  |
  | --- | --- |
  | Item Type | Journal Article |
  | Author | Damla Çetin |
  | Author | Selmin Çuhadar |
  | Date | 2021 |
  | Library Catalogue | Google Scholar |
  | URL | http://www.cappsy.org/archives/vol13/no3/cap\_13\_03\_15\_en.pdf |
  | Accessed | 11/07/2025, 10:20:04 |
  | Extra | Publisher: Psikiyatride Guncel Yaklasimlar: Current Approaches in Psychiatry |
  | Volume | 13 |
  | Pages | 619–639 |
  | Publication | Psikiyatride Guncel Yaklasimlar |
  | Issue | 3 |
  | Date Added | 11/07/2025, 10:20:29 |
  | Modified | 11/07/2025, 10:20:29 |

  ### Attachments

  - Available Version (via Google Scholar)
- ## Profile of studies on the welfare of dogs: a systematic review of the literature from 1999 to 2020.

  |  |  |
  | --- | --- |
  | Item Type | Journal Article |
  | Author | Luciana Velasques Cervo |
  | Author | Emily Edgren Adams |
  | Author | Pedro Lucas Vogt |
  | Author | Dalila Moter Benvegnú |
  | Date | 2024 |
  | Short Title | Profile of studies on the welfare of dogs |
  | Library Catalogue | Google Scholar |
  | URL | https://revistas.ufpr.br/veterinary/article/download/92228/51668 |
  | Accessed | 11/07/2025, 13:46:11 |
  | Volume | 29 |
  | Publication | Archives of Veterinary Science |
  | Issue | 1 |
  | Date Added | 11/07/2025, 13:46:33 |
  | Modified | 11/07/2025, 13:46:33 |

  ### Attachments

  - Available Version (via Google Scholar)
- ## I benefici e l'interazione armoniosa tra i pazienti pediatrici oncologici e gli animali da compagnia: una revisione della letteratura

  |  |  |
  | --- | --- |
  | Item Type | Journal Article |
  | Author | VALENTINA CERVILLIO |
  | Date | 2019 |
  | Short Title | I benefici e l'interazione armoniosa tra i pazienti pediatrici oncologici e gli animali da compagnia |
  | Library Catalogue | Google Scholar |
  | URL | https://tesi.univpm.it/handle/20.500.12075/3345 |
  | Accessed | 11/07/2025, 09:58:04 |
  | Date Added | 11/07/2025, 09:58:25 |
  | Modified | 11/07/2025, 09:58:25 |

  ### Attachments

  - Available Version (via Google Scholar)
- ## Alguns indicadores de avaliação de bem-estar em vacas leiteiras–revisão Some indicators for the assessment of welfare in dairy cows–a review

  |  |  |
  | --- | --- |
  | Item Type | Journal Article |
  | Author | Joaquim L. Cerqueira |
  | Author | José P. Araújo |
  | Author | Jan T. Sorensen |
  | Author | João Niza-Ribeiro |
  | Date | 2012 |
  | Library Catalogue | Google Scholar |
  | Pages | 5–19 |
  | Publication | Rev Port Cienc Vet |
  | Date Added | 11/07/2025, 11:15:52 |
  | Modified | 11/07/2025, 11:15:52 |

  ### Attachments

  - Available Version (via Google Scholar)
- ## Dog alerting and/or responding to epileptic seizures: A scoping review

  |  |  |
  | --- | --- |
  | Item Type | Journal Article |
  | Author | Amelie Catala |
  | Author | Hugo Cousillas |
  | Author | Martine Hausberger |
  | Author | Marine Grandgeorge |
  | Date | 2018 |
  | Short Title | Dog alerting and/or responding to epileptic seizures |
  | Library Catalogue | Google Scholar |
  | URL | https://journals.plos.org/plosone/article?id=10.1371/journal.pone.0208280 |
  | Accessed | 11/07/2025, 09:35:40 |
  | Volume | 13 |
  | Publisher | Public Library of Science San Francisco, CA USA |
  | Pages | e0208280 |
  | Publication | PloS one |
  | Issue | 12 |
  | Date Added | 11/07/2025, 09:36:11 |
  | Modified | 11/07/2025, 09:36:11 |

  ### Attachments

  - Available Version (via Google Scholar)
- ## Welfare implications on management strategies for rearing dairy calves: A systematic review. Part 2–Social management

  |  |  |
  | --- | --- |
  | Item Type | Journal Article |
  | Author | Patricia Carulla |
  | Author | Arantxa Villagrá |
  | Author | Fernando Estellés |
  | Author | Isabel Blanco-Penedo |
  | Date | 2023 |
  | Short Title | Welfare implications on management strategies for rearing dairy calves |
  | Library Catalogue | Google Scholar |
  | URL | https://www.frontiersin.org/articles/10.3389/fvets.2023.1154555/full |
  | Accessed | 11/07/2025, 11:08:17 |
  | Volume | 10 |
  | Publisher | Frontiers Media SA |
  | Pages | 1154555 |
  | Publication | Frontiers in Veterinary Science |
  | Date Added | 11/07/2025, 11:08:27 |
  | Modified | 11/07/2025, 11:08:27 |

  ### Attachments

  - Available Version (via Google Scholar)
  - Available Version (via Google Scholar)
- ## Moving toward fear-free husbandry and veterinary care for horses

  |  |  |
  | --- | --- |
  | Item Type | Journal Article |
  | Author | Sharon L. Carroll |
  | Author | Benjamin W. Sykes |
  | Author | Paul C. Mills |
  | Date | 2022 |
  | Library Catalogue | Google Scholar |
  | URL | https://www.mdpi.com/2076-2615/12/21/2907 |
  | Accessed | 11/07/2025, 09:36:11 |
  | Volume | 12 |
  | Publisher | Mdpi |
  | Pages | 2907 |
  | Publication | Animals |
  | Issue | 21 |
  | Date Added | 11/07/2025, 09:36:11 |
  | Modified | 11/07/2025, 09:36:11 |
- ## Equine-Facilitated Counselling and Anxiety: An Exploration of Possibilities

  |  |  |
  | --- | --- |
  | Item Type | Journal Article |
  | Author | Theresa Cardiff |
  | Date | 2021 |
  | Short Title | Equine-Facilitated Counselling and Anxiety |
  | Library Catalogue | Google Scholar |
  | Publisher | City University of Seattle |
  | Date Added | 11/07/2025, 11:12:01 |
  | Modified | 11/07/2025, 11:12:01 |
- ## Animal assisted therapy

  |  |  |
  | --- | --- |
  | Item Type | Journal Article |
  | Author | Arzu Çakıcı |
  | Author | Mehmet Kök |
  | Date | 2020 |
  | Library Catalogue | Google Scholar |
  | URL | https://search.proquest.com/openview/62476d9e5892b075d19e0c01aef2b62a/1?pq-origsite=gscholar&cbl=166138 |
  | Accessed | 11/07/2025, 10:13:35 |
  | Extra | Publisher: Psikiyatride Guncel Yaklasimlar: Current Approaches in Psychiatry |
  | Volume | 12 |
  | Pages | 117–130 |
  | Publication | Psikiyatride Guncel Yaklasimlar |
  | Issue | 1 |
  | Date Added | 11/07/2025, 10:13:55 |
  | Modified | 11/07/2025, 10:13:55 |
- ## Neurobiological underpinnings of dogs’ human-like social competence: How interactions between stress response systems and oxytocin mediate dogs’ social skills

  |  |  |
  | --- | --- |
  | Item Type | Journal Article |
  | Author | Alicia Phillips Buttner |
  | Date | 2016 |
  | Short Title | Neurobiological underpinnings of dogs’ human-like social competence |
  | Library Catalogue | Google Scholar |
  | URL | https://www.sciencedirect.com/science/article/pii/S0149763416302652 |
  | Accessed | 11/07/2025, 09:29:22 |
  | Volume | 71 |
  | Publisher | Elsevier |
  | Pages | 198–214 |
  | Publication | Neuroscience & Biobehavioral Reviews |
  | Date Added | 11/07/2025, 09:29:28 |
  | Modified | 11/07/2025, 09:29:28 |
- ## Neurobiological underpinnings of dogs’ human-like social competence: How interactions between stress response systems and oxytocin mediate dogs’ social skills

  |  |  |
  | --- | --- |
  | Item Type | Journal Article |
  | Author | Alicia Phillips Buttner |
  | Date | 2016 |
  | Short Title | Neurobiological underpinnings of dogs’ human-like social competence |
  | Library Catalogue | Google Scholar |
  | URL | https://www.sciencedirect.com/science/article/pii/S0149763416302652 |
  | Accessed | 11/07/2025, 10:18:01 |
  | Volume | 71 |
  | Publisher | Elsevier |
  | Pages | 198–214 |
  | Publication | Neuroscience & Biobehavioral Reviews |
  | Date Added | 11/07/2025, 10:18:17 |
  | Modified | 11/07/2025, 10:18:17 |
- ## Animal-Assisted Interventions for Children with Attention Deficit/Hyperactivity Disorder: A Theoretical Review and Consideration of Future Research Directions

  |  |  |
  | --- | --- |
  | Item Type | Journal Article |
  | Author | Caroline Busch |
  | Author | Lara Tucha |
  | Author | Alzbeta Talarovicova |
  | Author | Anselm B. M. Fuermaier |
  | Author | Ben Lewis-Evans |
  | Author | Oliver Tucha |
  | Abstract | Attention Deficit Hyperactivity Disorder (ADHD) is one of the most prevalent neurodevelopmental disorders and is characterized by core symptoms of inattention, impulsivity, and hyperactivity. Given the limitations of the existing treatment strategies, it seems necessary to consider the further exploration of alternative treatment approaches. In this review, the application and complementary use of animal-assisted interventions to the treatment of ADHD were discussed. Several mechanisms including calming, socializing, motivating, and cognitive effects of animal-assisted interventions were explored. Since studies directly investigating these effects on ADHD are scarce, so each of them were examined in terms of how it could benefit the treatment of ADHD. These effects can have a positive effect on several core symptoms of ADHD. |
  | Date | 02/2016 |
  | Language | en |
  | Short Title | Animal-Assisted Interventions for Children with Attention Deficit/Hyperactivity Disorder |
  | Library Catalogue | Crossref |
  | URL | https://journals.sagepub.com/doi/10.1177/0033294115626633 |
  | Accessed | 11/07/2025, 09:29:25 |
  | Rights | https://journals.sagepub.com/page/policies/text-and-data-mining-license |
  | Volume | 118 |
  | Publisher | SAGE Publications |
  | Pages | 292-331 |
  | Publication | Psychological Reports |
  | DOI | 10.1177/0033294115626633 |
  | Issue | 1 |
  | Journal Abbr | Psychol Rep |
  | ISSN | 0033-2941, 1558-691X |
  | Date Added | 11/07/2025, 09:29:28 |
  | Modified | 11/07/2025, 09:29:28 |

  ### Attachments

  - Available Version (via Google Scholar)
- ## Animal-Assisted Interventions for Children with Attention Deficit/Hyperactivity Disorder: A Theoretical Review and Consideration of Future Research Directions

  |  |  |
  | --- | --- |
  | Item Type | Journal Article |
  | Author | Caroline Busch |
  | Author | Lara Tucha |
  | Author | Alzbeta Talarovicova |
  | Author | Anselm B. M. Fuermaier |
  | Author | Ben Lewis-Evans |
  | Author | Oliver Tucha |
  | Abstract | Attention Deficit Hyperactivity Disorder (ADHD) is one of the most prevalent neurodevelopmental disorders and is characterized by core symptoms of inattention, impulsivity, and hyperactivity. Given the limitations of the existing treatment strategies, it seems necessary to consider the further exploration of alternative treatment approaches. In this review, the application and complementary use of animal-assisted interventions to the treatment of ADHD were discussed. Several mechanisms including calming, socializing, motivating, and cognitive effects of animal-assisted interventions were explored. Since studies directly investigating these effects on ADHD are scarce, so each of them were examined in terms of how it could benefit the treatment of ADHD. These effects can have a positive effect on several core symptoms of ADHD. |
  | Date | 02/2016 |
  | Language | en |
  | Short Title | Animal-Assisted Interventions for Children with Attention Deficit/Hyperactivity Disorder |
  | Library Catalogue | Crossref |
  | URL | https://journals.sagepub.com/doi/10.1177/0033294115626633 |
  | Accessed | 11/07/2025, 09:54:56 |
  | Rights | https://journals.sagepub.com/page/policies/text-and-data-mining-license |
  | Volume | 118 |
  | Publisher | SAGE Publications |
  | Pages | 292-331 |
  | Publication | Psychological Reports |
  | DOI | 10.1177/0033294115626633 |
  | Issue | 1 |
  | Journal Abbr | Psychol Rep |
  | ISSN | 0033-2941, 1558-691X |
  | Date Added | 11/07/2025, 09:55:10 |
  | Modified | 11/07/2025, 09:55:12 |

  ### Attachments

  - Available Version (via Google Scholar)
- ## Animal-Assisted Interventions for Children with Attention Deficit/Hyperactivity Disorder: A Theoretical Review and Consideration of Future Research Directions

  |  |  |
  | --- | --- |
  | Item Type | Journal Article |
  | Author | Caroline Busch |
  | Author | Lara Tucha |
  | Author | Alzbeta Talarovicova |
  | Author | Anselm B. M. Fuermaier |
  | Author | Ben Lewis-Evans |
  | Author | Oliver Tucha |
  | Abstract | Attention Deficit Hyperactivity Disorder (ADHD) is one of the most prevalent neurodevelopmental disorders and is characterized by core symptoms of inattention, impulsivity, and hyperactivity. Given the limitations of the existing treatment strategies, it seems necessary to consider the further exploration of alternative treatment approaches. In this review, the application and complementary use of animal-assisted interventions to the treatment of ADHD were discussed. Several mechanisms including calming, socializing, motivating, and cognitive effects of animal-assisted interventions were explored. Since studies directly investigating these effects on ADHD are scarce, so each of them were examined in terms of how it could benefit the treatment of ADHD. These effects can have a positive effect on several core symptoms of ADHD. |
  | Date | 02/2016 |
  | Language | en |
  | Short Title | Animal-Assisted Interventions for Children with Attention Deficit/Hyperactivity Disorder |
  | Library Catalogue | Crossref |
  | URL | https://journals.sagepub.com/doi/10.1177/0033294115626633 |
  | Accessed | 11/07/2025, 10:13:44 |
  | Rights | https://journals.sagepub.com/page/policies/text-and-data-mining-license |
  | Volume | 118 |
  | Publisher | SAGE Publications |
  | Pages | 292-331 |
  | Publication | Psychological Reports |
  | DOI | 10.1177/0033294115626633 |
  | Issue | 1 |
  | Journal Abbr | Psychol Rep |
  | ISSN | 0033-2941, 1558-691X |
  | Date Added | 11/07/2025, 10:13:55 |
  | Modified | 11/07/2025, 10:13:55 |

  ### Attachments

  - Available Version (via Google Scholar)
- ## Practical importance of behaviour assessment with regard to welfare and productivity of cattle.

  |  |  |
  | --- | --- |
  | Item Type | Journal Article |
  | Author | MONIKA Budzyńska |
  | Author | JAROSŁAW Kamieniak |
  | Author | Dorota Marko |
  | Date | 2019 |
  | Library Catalogue | Google Scholar |
  | URL | https://www.cabidigitallibrary.org/doi/full/10.5555/20193419227 |
  | Accessed | 11/07/2025, 11:09:38 |
  | Date Added | 11/07/2025, 11:09:44 |
  | Modified | 11/07/2025, 11:09:44 |
- ## An exploration of the potential benefits of pet-facilitated therapy.

  |  |  |
  | --- | --- |
  | Item Type | Journal Article |
  | Author | Sarah J. Brodie |
  | Author | Francis C. Biley |
  | Date | 1999 |
  | Library Catalogue | Google Scholar |
  | URL | https://search.ebscohost.com/login.aspx?direct=true&profile=ehost&scope=site&authtype=crawler&jrnl=09621067&AN=107093590&h=fru3vONuXidRlH%2FHPrExD2%2BUFBLz0hRAu98aMOesc4Q4ldIYkw2VKLSB0G6PZS0%2FI9%2FCSRP84SPf9JMcK44OrQ%3D%3D&crl=c |
  | Accessed | 11/07/2025, 10:09:10 |
  | Volume | 8 |
  | Publication | Journal of Clinical Nursing (Wiley-Blackwell) |
  | Issue | 4 |
  | Date Added | 11/07/2025, 10:09:48 |
  | Modified | 11/07/2025, 10:09:48 |

  ### Attachments

  - Available Version (via Google Scholar)
- ## Animal-assisted interventions in the classroom—A systematic review

  |  |  |
  | --- | --- |
  | Item Type | Journal Article |
  | Author | Victoria L. Brelsford |
  | Author | Kerstin Meints |
  | Author | Nancy R. Gee |
  | Author | Karen Pfeffer |
  | Date | 2017 |
  | Library Catalogue | Google Scholar |
  | URL | https://www.mdpi.com/1660-4601/14/7/669 |
  | Accessed | 11/07/2025, 09:28:55 |
  | Volume | 14 |
  | Publisher | MDPI |
  | Pages | 669 |
  | Publication | International journal of environmental research and public health |
  | Issue | 7 |
  | Date Added | 11/07/2025, 09:29:28 |
  | Modified | 11/07/2025, 09:29:28 |

  ### Attachments

  - Available Version (via Google Scholar)
- ## Optimal marine mammal welfare under human care: Current efforts and future directions

  |  |  |
  | --- | --- |
  | Item Type | Journal Article |
  | Author | Sabrina Brando |
  | Author | Donald M. Broom |
  | Author | Cristina Acasuso-Rivero |
  | Author | Fay Clark |
  | Date | 2018 |
  | Short Title | Optimal marine mammal welfare under human care |
  | Library Catalogue | Google Scholar |
  | URL | https://www.sciencedirect.com/science/article/pii/S0376635717304291 |
  | Accessed | 11/07/2025, 09:31:39 |
  | Volume | 156 |
  | Publisher | Elsevier |
  | Pages | 16–36 |
  | Publication | Behavioural Processes |
  | Date Added | 11/07/2025, 09:32:09 |
  | Modified | 11/07/2025, 09:32:09 |
- ## Optimal marine mammal welfare under human care: Current efforts and future directions

  |  |  |
  | --- | --- |
  | Item Type | Journal Article |
  | Author | Sabrina Brando |
  | Author | Donald M. Broom |
  | Author | Cristina Acasuso-Rivero |
  | Author | Fay Clark |
  | Date | 2018 |
  | Short Title | Optimal marine mammal welfare under human care |
  | Library Catalogue | Google Scholar |
  | URL | https://www.sciencedirect.com/science/article/pii/S0376635717304291 |
  | Accessed | 11/07/2025, 11:08:25 |
  | Volume | 156 |
  | Publisher | Elsevier |
  | Pages | 16–36 |
  | Publication | Behavioural Processes |
  | Date Added | 11/07/2025, 11:08:27 |
  | Modified | 11/07/2025, 11:08:27 |
- ## A Multidimensional Meta-Analysis of Psychotherapy for PTSD

  |  |  |
  | --- | --- |
  | Item Type | Journal Article |
  | Author | Rebekah Bradley |
  | Author | Jamelle Greene |
  | Author | Eric Russ |
  | Author | Lissa Dutra |
  | Author | Drew Westen |
  | Date | 2005-02-01 |
  | Language | en |
  | Library Catalogue | Crossref |
  | URL | https://psychiatryonline.org/doi/10.1176/appi.ajp.162.2.214 |
  | Accessed | 11/07/2025, 09:58:25 |
  | Volume | 162 |
  | Publisher | American Psychiatric Association Publishing |
  | Pages | 214-227 |
  | Publication | American Journal of Psychiatry |
  | DOI | 10.1176/appi.ajp.162.2.214 |
  | Issue | 2 |
  | Journal Abbr | AJP |
  | ISSN | 0002-953X, 1535-7228 |
  | Date Added | 11/07/2025, 09:58:25 |
  | Modified | 11/07/2025, 09:58:25 |

  ### Attachments

  - Available Version (via Google Scholar)
- ## A Systematic Review of Equine-Assisted Interventions in Military Veterans Diagnosed with PTSD

  |  |  |
  | --- | --- |
  | Item Type | Journal Article |
  | Author | Lisa Boss |
  | Author | Sandy Branson |
  | Author | Heather Hagan |
  | Author | Cheryl Krause-Parello |
  | Date | 2019-12-04 |
  | Library Catalogue | Crossref |
  | URL | https://journal-veterans-studies.org/article/10.21061/jvs.v5i1.134/ |
  | Accessed | 11/07/2025, 09:35:44 |
  | Volume | 5 |
  | Publisher | Virginia Tech Libraries |
  | Pages | 23 |
  | Publication | Journal of Veterans Studies |
  | DOI | 10.21061/jvs.v5i1.134 |
  | Issue | 1 |
  | Journal Abbr | JVS |
  | ISSN | 2470-4768 |
  | Date Added | 11/07/2025, 09:36:11 |
  | Modified | 11/07/2025, 09:36:11 |

  ### Attachments

  - Full Text PDF
- ## A Systematic Review of Equine-Assisted Interventions in Military Veterans Diagnosed with PTSD

  |  |  |
  | --- | --- |
  | Item Type | Journal Article |
  | Author | Lisa Boss |
  | Author | Sandy Branson |
  | Author | Heather Hagan |
  | Author | Cheryl Krause-Parello |
  | Date | 2019-12-04 |
  | Library Catalogue | Crossref |
  | URL | https://journal-veterans-studies.org/article/10.21061/jvs.v5i1.134/ |
  | Accessed | 11/07/2025, 10:16:36 |
  | Volume | 5 |
  | Publisher | Virginia Tech Libraries |
  | Pages | 23 |
  | Publication | Journal of Veterans Studies |
  | DOI | 10.21061/jvs.v5i1.134 |
  | Issue | 1 |
  | Journal Abbr | JVS |
  | ISSN | 2470-4768 |
  | Date Added | 11/07/2025, 10:16:47 |
  | Modified | 11/07/2025, 10:16:47 |
- ## Pet face: Mechanisms underlying human-animal relationships

  |  |  |
  | --- | --- |
  | Item Type | Journal Article |
  | Author | Marta Borgi |
  | Author | Francesca Cirulli |
  | Date | 2016 |
  | Short Title | Pet face |
  | Library Catalogue | Google Scholar |
  | URL | https://www.frontiersin.org/articles/10.3389/fpsyg.2016.00298/full |
  | Accessed | 11/07/2025, 10:09:36 |
  | Volume | 7 |
  | Publisher | Frontiers Media SA |
  | Pages | 298 |
  | Publication | Frontiers in psychology |
  | Date Added | 11/07/2025, 10:09:48 |
  | Modified | 11/07/2025, 10:09:48 |

  ### Attachments

  - Available Version (via Google Scholar)
- ## How Assistance Dogs Impact the Academic and Social/Emotional Development of Students With Disabilities

  |  |  |
  | --- | --- |
  | Item Type | Journal Article |
  | Author | Elizabeth A. Bloom |
  | Date | 2025 |
  | Library Catalogue | Google Scholar |
  | URL | https://spark.bethel.edu/etd/1177/ |
  | Accessed | 11/07/2025, 13:44:41 |
  | Date Added | 11/07/2025, 13:45:12 |
  | Modified | 11/07/2025, 13:45:12 |

  ### Attachments

  - Available Version (via Google Scholar)
- ## Mother–young bonding: Neurobiological aspects and maternal biochemical signaling in altricial domesticated mammals

  |  |  |
  | --- | --- |
  | Item Type | Journal Article |
  | Author | Cécile Bienboire-Frosini |
  | Author | Míriam Marcet-Rius |
  | Author | Agustín Orihuela |
  | Author | Adriana Domínguez-Oliva |
  | Author | Patricia Mora-Medina |
  | Author | Adriana Olmos-Hernández |
  | Author | Alejandro Casas-Alvarado |
  | Author | Daniel Mota-Rojas |
  | Date | 2023 |
  | Short Title | Mother–young bonding |
  | Library Catalogue | Google Scholar |
  | URL | https://www.mdpi.com/2076-2615/13/3/532 |
  | Accessed | 11/07/2025, 11:11:44 |
  | Volume | 13 |
  | Publisher | MDPI |
  | Pages | 532 |
  | Publication | Animals |
  | Issue | 3 |
  | Date Added | 11/07/2025, 11:12:01 |
  | Modified | 11/07/2025, 11:12:01 |

  ### Attachments

  - Available Version (via Google Scholar)
- ## A Review on a Therapy with bonds between Human and Animals

  |  |  |
  | --- | --- |
  | Item Type | Journal Article |
  | Author | Surajit Bhattacharyya |
  | Author | Goutam Mukhopadhyay |
  | Date | 2014 |
  | Library Catalogue | Google Scholar |
  | URL | http://www.abhipublications.org/journal/G\_112\_I.pdf |
  | Accessed | 11/07/2025, 10:19:54 |
  | Volume | 2 |
  | Pages | 519–531 |
  | Publication | International Journal of Pharmacy and Engineering |
  | Issue | 4 |
  | Date Added | 11/07/2025, 10:20:29 |
  | Modified | 11/07/2025, 10:20:29 |

  ### Attachments

  - Available Version (via Google Scholar)
- ## Precision livestock farming in swine welfare: a review for swine practitioners

  |  |  |
  | --- | --- |
  | Item Type | Journal Article |
  | Author | Madonna Benjamin |
  | Author | Steven Yik |
  | Date | 2019 |
  | Short Title | Precision livestock farming in swine welfare |
  | Library Catalogue | Google Scholar |
  | URL | https://www.mdpi.com/2076-2615/9/4/133 |
  | Accessed | 11/07/2025, 11:09:18 |
  | Volume | 9 |
  | Publisher | MDPI |
  | Pages | 133 |
  | Publication | Animals |
  | Issue | 4 |
  | Date Added | 11/07/2025, 11:09:44 |
  | Modified | 11/07/2025, 11:09:44 |
- ## Theories and possible processes of action in animal assisted interventions

  |  |  |
  | --- | --- |
  | Item Type | Journal Article |
  | Author | Andrea M. Beetz |
  | Date | 2017-04-03 |
  | Language | en |
  | Library Catalogue | Crossref |
  | URL | https://www.tandfonline.com/doi/full/10.1080/10888691.2016.1262263 |
  | Accessed | 11/07/2025, 10:11:57 |
  | Volume | 21 |
  | Publisher | Informa UK Limited |
  | Pages | 139-149 |
  | Publication | Applied Developmental Science |
  | DOI | 10.1080/10888691.2016.1262263 |
  | Issue | 2 |
  | ISSN | 1088-8691, 1532-480X |
  | Date Added | 11/07/2025, 10:12:39 |
  | Modified | 11/07/2025, 10:12:39 |
- ## From human–animal relation practice research to the development of the livestock farmer's activity: an ergonomics–applied ethology interaction

  |  |  |
  | --- | --- |
  | Item Type | Journal Article |
  | Author | Joffrey Beaujouan |
  | Author | Damien Cromer |
  | Author | Xavier Boivin |
  | Date | 2021 |
  | Short Title | From human–animal relation practice research to the development of the livestock farmer's activity |
  | Library Catalogue | Google Scholar |
  | URL | https://www.sciencedirect.com/science/article/pii/S175173112100238X |
  | Accessed | 11/07/2025, 10:13:29 |
  | Volume | 15 |
  | Publisher | Elsevier |
  | Pages | 100395 |
  | Publication | Animal |
  | Issue | 12 |
  | Date Added | 11/07/2025, 10:13:55 |
  | Modified | 11/07/2025, 10:13:55 |
- ## Understanding canine aggression: Neurobiological insights for a complex behavior

  |  |  |
  | --- | --- |
  | Item Type | Journal Article |
  | Author | Gökçen Güvenç Bayram |
  | Author | Zeynep Semen |
  | Short Title | Understanding canine aggression |
  | Library Catalogue | Google Scholar |
  | URL | https://dergipark.org.tr/en/pub/http-www-jivs-net/article/1272983 |
  | Accessed | 11/07/2025, 10:20:28 |
  | Volume | 7 |
  | Publisher | The Chamber of Veterinary Surgeons, Istanbul |
  | Pages | 68–79 |
  | Publication | Journal of Istanbul Veterinary Sciences |
  | Issue | 2 |
  | Date Added | 11/07/2025, 10:20:29 |
  | Modified | 11/07/2025, 10:20:29 |

  ### Attachments

  - Available Version (via Google Scholar)
- ## The welfare implications of large litter size in the domestic pig II: management factors

  |  |  |
  | --- | --- |
  | Item Type | Journal Article |
  | Author | E. M. Baxter |
  | Author | K. M. D. Rutherford |
  | Author | R. B. D’eath |
  | Author | G. Arnott |
  | Author | S. P. Turner |
  | Author | P. Sandøe |
  | Author | V. A. Moustsen |
  | Author | F. Thorup |
  | Author | S. A. Edwards |
  | Author | A. B. Lawrence |
  | Date | 2013 |
  | Short Title | The welfare implications of large litter size in the domestic pig II |
  | Library Catalogue | Google Scholar |
  | URL | https://www.cambridge.org/core/journals/animal-welfare/article/welfare-implications-of-large-litter-size-in-the-domestic-pig-ii-management-factors/9CC2025572848F31EAAFC9D6F3F7DBB4 |
  | Accessed | 11/07/2025, 09:35:49 |
  | Volume | 22 |
  | Publisher | Cambridge University Press |
  | Pages | 219–238 |
  | Publication | Animal Welfare |
  | Issue | 2 |
  | Date Added | 11/07/2025, 09:36:11 |
  | Modified | 11/07/2025, 09:36:11 |

  ### Attachments

  - Available Version (via Google Scholar)
- ## The Benefits of Human–Companion Animal Interaction: A Review

  |  |  |
  | --- | --- |
  | Item Type | Journal Article |
  | Author | Sandra B. Barker |
  | Author | Aaron R. Wolen |
  | Abstract | This article provides a review of research published since 1980 on the benefits of human–companion animal interaction. Studies focusing on the benefits of pet ownership are presented first, followed by research on the benefits of interacting with companion animals that are not owned by the subject (animal-assisted activities). While most of the published studies are descriptive and have been conducted with convenience samples, a promising number of controlled studies support the health benefits of interacting with companion animals. Future research employing more rigorous designs and systematically building upon a clearly defined line of inquiry is needed to advance our knowledge of the benefits of human–companion animal interaction. |
  | Date | 12/2008 |
  | Language | en |
  | Short Title | The Benefits of Human–Companion Animal Interaction |
  | Library Catalogue | Crossref |
  | URL | https://utppublishing.com/doi/10.3138/jvme.35.4.487 |
  | Accessed | 11/07/2025, 09:27:20 |
  | Volume | 35 |
  | Publisher | University of Toronto Press Inc. (UTPress) |
  | Pages | 487-495 |
  | Publication | Journal of Veterinary Medical Education |
  | DOI | 10.3138/jvme.35.4.487 |
  | Issue | 4 |
  | ISSN | 0748-321X, 1943-7218 |
  | Date Added | 11/07/2025, 09:27:42 |
  | Modified | 11/07/2025, 09:27:42 |

  ### Attachments

  - Available Version (via Google Scholar)
- ## The Benefits of Human–Companion Animal Interaction: A Review

  |  |  |
  | --- | --- |
  | Item Type | Journal Article |
  | Author | Sandra B. Barker |
  | Author | Aaron R. Wolen |
  | Abstract | This article provides a review of research published since 1980 on the benefits of human–companion animal interaction. Studies focusing on the benefits of pet ownership are presented first, followed by research on the benefits of interacting with companion animals that are not owned by the subject (animal-assisted activities). While most of the published studies are descriptive and have been conducted with convenience samples, a promising number of controlled studies support the health benefits of interacting with companion animals. Future research employing more rigorous designs and systematically building upon a clearly defined line of inquiry is needed to advance our knowledge of the benefits of human–companion animal interaction. |
  | Date | 12/2008 |
  | Language | en |
  | Short Title | The Benefits of Human–Companion Animal Interaction |
  | Library Catalogue | Crossref |
  | URL | https://utppublishing.com/doi/10.3138/jvme.35.4.487 |
  | Accessed | 11/07/2025, 10:05:19 |
  | Volume | 35 |
  | Publisher | University of Toronto Press Inc. (UTPress) |
  | Pages | 487-495 |
  | Publication | Journal of Veterinary Medical Education |
  | DOI | 10.3138/jvme.35.4.487 |
  | Issue | 4 |
  | ISSN | 0748-321X, 1943-7218 |
  | Date Added | 11/07/2025, 10:05:27 |
  | Modified | 11/07/2025, 10:05:27 |

  ### Attachments

  - Available Version (via Google Scholar)
- ## Canine-assisted interventions in hospitals: best practices for maximizing human and canine safety

  |  |  |
  | --- | --- |
  | Item Type | Journal Article |
  | Author | Sandra B. Barker |
  | Author | Nancy R. Gee |
  | Date | 2021 |
  | Short Title | Canine-assisted interventions in hospitals |
  | Library Catalogue | Google Scholar |
  | URL | https://www.frontiersin.org/articles/10.3389/fvets.2021.615730/full |
  | Accessed | 11/07/2025, 10:13:21 |
  | Volume | 8 |
  | Publisher | Frontiers Media SA |
  | Pages | 615730 |
  | Publication | Frontiers in Veterinary Science |
  | Date Added | 11/07/2025, 10:13:55 |
  | Modified | 11/07/2025, 10:13:55 |

  ### Attachments

  - Available Version (via Google Scholar)
- ## The best medicine: personal pets and therapy animals in the hospital setting

  |  |  |
  | --- | --- |
  | Item Type | Journal Article |
  | Author | Denise Barchas |
  | Author | Melissa Melaragni |
  | Author | Heather Abrahim |
  | Author | Eric Barchas |
  | Date | 2020 |
  | Short Title | The best medicine |
  | Library Catalogue | Google Scholar |
  | URL | https://www.ccnursing.theclinics.com/article/S0899-5885(20)30012-5/abstract |
  | Accessed | 11/07/2025, 10:18:13 |
  | Volume | 32 |
  | Publisher | Elsevier |
  | Pages | 167–190 |
  | Publication | Critical Care Nursing Clinics |
  | Issue | 2 |
  | Date Added | 11/07/2025, 10:18:17 |
  | Modified | 11/07/2025, 10:18:17 |
- ## A Scoping Review of Campus-Based Animal-Assisted Interactions Programs for College Student Mental Health

  |  |  |
  | --- | --- |
  | Item Type | Journal Article |
  | Author | Tanya K. Bailey |
  | Date | 2023 |
  | Library Catalogue | Google Scholar |
  | URL | https://docs.lib.purdue.edu/paij/vol6/iss1/1/ |
  | Accessed | 11/07/2025, 10:18:05 |
  | Volume | 6 |
  | Pages | 1 |
  | Publication | People and Animals: The International Journal of Research and Practice |
  | Issue | 1 |
  | Date Added | 11/07/2025, 10:18:17 |
  | Modified | 11/07/2025, 10:18:17 |

  ### Attachments

  - Available Version (via Google Scholar)
- ## Effects of equine-assisted interventions on older adults’ health: A systematic review

  |  |  |
  | --- | --- |
  | Item Type | Journal Article |
  | Author | Léa Badin |
  | Author | Émilie Alibran |
  | Author | Kristell Pothier |
  | Author | Nathalie Bailly |
  | Date | 2022 |
  | Short Title | Effects of equine-assisted interventions on older adults’ health |
  | Library Catalogue | Google Scholar |
  | URL | https://www.sciencedirect.com/science/article/pii/S2352013222000746 |
  | Accessed | 11/07/2025, 09:29:08 |
  | Volume | 9 |
  | Publisher | Elsevier |
  | Pages | 542–552 |
  | Publication | International journal of nursing sciences |
  | Issue | 4 |
  | Date Added | 11/07/2025, 09:29:28 |
  | Modified | 11/07/2025, 09:29:28 |
- ## Equine-Facilitated Prison-Based Programs Within the Context of Prison-Based Animal Programs: State of the Science Review

  |  |  |
  | --- | --- |
  | Item Type | Journal Article |
  | Author | Keren Bachi |
  | Date | 01/2013 |
  | Language | en |
  | Short Title | Equine-Facilitated Prison-Based Programs Within the Context of Prison-Based Animal Programs |
  | Library Catalogue | Crossref |
  | URL | http://www.tandfonline.com/doi/abs/10.1080/10509674.2012.734371 |
  | Accessed | 11/07/2025, 09:31:47 |
  | Volume | 52 |
  | Publisher | Informa UK Limited |
  | Pages | 46-74 |
  | Publication | Journal of Offender Rehabilitation |
  | DOI | 10.1080/10509674.2012.734371 |
  | Issue | 1 |
  | ISSN | 1050-9674, 1540-8558 |
  | Date Added | 11/07/2025, 09:32:09 |
  | Modified | 11/07/2025, 09:32:09 |
- ## Addressing the Psychosocial Needs of Individuals with Communication Disorders: The Integration of Animal-Assisted Therapy within Counseling

  |  |  |
  | --- | --- |
  | Item Type | Journal Article |
  | Author | W. Leigh Atherton |
  | Author | Daniel Hudock |
  | Abstract | AbstractIndividuals with speech, language, and communication disorders often present with psychosocial concerns that span the physiological, intrapersonal, and interpersonal domains of functioning. Despite this fact, the provision of counseling service by speech-language pathologist (SLP) that directly addresses clients' psychosocial needs is sparse. Research shows the primary counseling strategy used by therapists is psychoeducation, failing to effectively address the psychosocial concerns. Integrating complementary approaches to traditional counseling in SLP can enhance both the quality of therapeutic intervention and client outcomes. The purpose of this article is to demonstrate the potential of animal-assisted therapy (AAT) as an adjunctive or complementary approach for counseling within SLP. A review of literature demonstrates a need for improved counseling service provision within SLP treatment, as well as the benefits of integrating AAT. A framework for how AAT intentions and techniques fit within SLP Scope of Practice counseling activities is presented, along with case examples to demonstrate how AAT can be integrated within SLP treatment. It is concluded that the integration of AAT as a complementary approach to traditional SLP counseling can enhance both the frequency of counseling services provided and clients' psychosocial outcomes. |
  | Date | 01/2022 |
  | Language | en |
  | Short Title | Addressing the Psychosocial Needs of Individuals with Communication Disorders |
  | Library Catalogue | Crossref |
  | URL | http://www.thieme-connect.de/DOI/DOI?10.1055/s-0041-1741556 |
  | Accessed | 11/07/2025, 09:31:36 |
  | Volume | 43 |
  | Publisher | Georg Thieme Verlag KG |
  | Pages | 024-034 |
  | Publication | Seminars in Speech and Language |
  | DOI | 10.1055/s-0041-1741556 |
  | Issue | 01 |
  | Journal Abbr | Semin Speech Lang |
  | ISSN | 0734-0478, 1098-9056 |
  | Date Added | 11/07/2025, 09:32:09 |
  | Modified | 11/07/2025, 09:32:09 |
- ## Addressing the Psychosocial Needs of Individuals with Communication Disorders: The Integration of Animal-Assisted Therapy within Counseling

  |  |  |
  | --- | --- |
  | Item Type | Journal Article |
  | Author | W. Leigh Atherton |
  | Author | Daniel Hudock |
  | Abstract | AbstractIndividuals with speech, language, and communication disorders often present with psychosocial concerns that span the physiological, intrapersonal, and interpersonal domains of functioning. Despite this fact, the provision of counseling service by speech-language pathologist (SLP) that directly addresses clients' psychosocial needs is sparse. Research shows the primary counseling strategy used by therapists is psychoeducation, failing to effectively address the psychosocial concerns. Integrating complementary approaches to traditional counseling in SLP can enhance both the quality of therapeutic intervention and client outcomes. The purpose of this article is to demonstrate the potential of animal-assisted therapy (AAT) as an adjunctive or complementary approach for counseling within SLP. A review of literature demonstrates a need for improved counseling service provision within SLP treatment, as well as the benefits of integrating AAT. A framework for how AAT intentions and techniques fit within SLP Scope of Practice counseling activities is presented, along with case examples to demonstrate how AAT can be integrated within SLP treatment. It is concluded that the integration of AAT as a complementary approach to traditional SLP counseling can enhance both the frequency of counseling services provided and clients' psychosocial outcomes. |
  | Date | 01/2022 |
  | Language | en |
  | Short Title | Addressing the Psychosocial Needs of Individuals with Communication Disorders |
  | Library Catalogue | Crossref |
  | URL | http://www.thieme-connect.de/DOI/DOI?10.1055/s-0041-1741556 |
  | Accessed | 11/07/2025, 10:16:42 |
  | Volume | 43 |
  | Publisher | Georg Thieme Verlag KG |
  | Pages | 024-034 |
  | Publication | Seminars in Speech and Language |
  | DOI | 10.1055/s-0041-1741556 |
  | Issue | 01 |
  | Journal Abbr | Semin Speech Lang |
  | ISSN | 0734-0478, 1098-9056 |
  | Date Added | 11/07/2025, 10:16:47 |
  | Modified | 11/07/2025, 10:16:47 |
- ## The Role of Animal Assisted Therapy in the Rehabilitation of Mental Health Disorders: A Systematic Literature Review

  |  |  |
  | --- | --- |
  | Item Type | Journal Article |
  | Author | Denis Arsovski |
  | Date | 2024 |
  | Short Title | The Role of Animal Assisted Therapy in the Rehabilitation of Mental Health Disorders |
  | Library Catalogue | Google Scholar |
  | URL | https://www.integrmed.org/journal/view.php?number=55 |
  | Accessed | 11/07/2025, 10:16:43 |
  | Volume | 3 |
  | Publisher | Jaseng Medical Foundation |
  | Pages | 142–151 |
  | Publication | Perspectives on Integrative Medicine |
  | Issue | 3 |
  | Date Added | 11/07/2025, 10:16:47 |
  | Modified | 11/07/2025, 10:16:47 |
- ## Healing Smiles: Enhancing Pediatric Dental Care through Animal-assisted Therapy–A Narrative Review

  |  |  |
  | --- | --- |
  | Item Type | Journal Article |
  | Author | Aishwarya Vineshkumar Antala |
  | Author | Pratik B. Kariya |
  | Date | 2023 |
  | Short Title | Healing Smiles |
  | Library Catalogue | Google Scholar |
  | URL | https://journals.lww.com/armh/fulltext/2023/11020/healing\_smiles\_\_enhancing\_pediatric\_dental\_care.19.aspx?context=latestarticles |
  | Accessed | 11/07/2025, 10:16:11 |
  | Volume | 11 |
  | Publisher | Medknow |
  | Pages | 258–262 |
  | Publication | Archives of Medicine and Health Sciences |
  | Issue | 2 |
  | Date Added | 11/07/2025, 10:16:47 |
  | Modified | 11/07/2025, 10:16:47 |
- ## Temperamento de fêmeas bovinas de corte submetidas à inseminação e relações com indicadores de produção: revisão sistemática e meta-análise

  |  |  |
  | --- | --- |
  | Item Type | Journal Article |
  | Author | Naiane Teixeira de Andrade |
  | Date | 2021 |
  | Short Title | Temperamento de fêmeas bovinas de corte submetidas à inseminação e relações com indicadores de produção |
  | Library Catalogue | Google Scholar |
  | URL | https://lume.ufrgs.br/handle/10183/233178 |
  | Accessed | 11/07/2025, 11:14:36 |
  | Date Added | 11/07/2025, 11:14:45 |
  | Modified | 11/07/2025, 11:14:45 |
- ## Toward a psychology of human–animal relations.

  |  |  |
  | --- | --- |
  | Item Type | Journal Article |
  | Author | Catherine E. Amiot |
  | Author | Brock Bastian |
  | Date | 2015 |
  | Library Catalogue | Google Scholar |
  | URL | https://psycnet.apa.org/journals/bul/141/1/6/ |
  | Accessed | 11/07/2025, 09:29:02 |
  | Volume | 141 |
  | Publisher | American Psychological Association |
  | Pages | 6 |
  | Publication | Psychological bulletin |
  | Issue | 1 |
  | Date Added | 11/07/2025, 09:29:28 |
  | Modified | 11/07/2025, 09:29:28 |

  ### Attachments

  - Available Version (via Google Scholar)
- ## Toward a psychology of human–animal relations.

  |  |  |
  | --- | --- |
  | Item Type | Journal Article |
  | Author | Catherine E. Amiot |
  | Author | Brock Bastian |
  | Date | 2015 |
  | Library Catalogue | Google Scholar |
  | URL | https://psycnet.apa.org/journals/bul/141/1/6/ |
  | Accessed | 11/07/2025, 10:12:14 |
  | Volume | 141 |
  | Publisher | American Psychological Association |
  | Pages | 6 |
  | Publication | Psychological bulletin |
  | Issue | 1 |
  | Date Added | 11/07/2025, 10:12:39 |
  | Modified | 11/07/2025, 10:12:39 |
- ## An exploration of how biophilic attributes on campuses might support student connectedness to nature, others, and self

  |  |  |
  | --- | --- |
  | Item Type | Journal Article |
  | Author | Susana Alves |
  | Author | Gowri Betrabet Gulwadi |
  | Author | Pia Nilsson |
  | Date | 2022 |
  | Library Catalogue | Google Scholar |
  | URL | https://www.frontiersin.org/articles/10.3389/fpsyg.2021.793175/full |
  | Accessed | 11/07/2025, 09:37:50 |
  | Volume | 12 |
  | Publisher | Frontiers Media SA |
  | Pages | 793175 |
  | Publication | Frontiers in psychology |
  | Date Added | 11/07/2025, 09:38:03 |
  | Modified | 11/07/2025, 09:38:03 |

  ### Attachments

  - Available Version (via Google Scholar)
- ## An exploration of how biophilic attributes on campuses might support student connectedness to nature, others, and self

  |  |  |
  | --- | --- |
  | Item Type | Journal Article |
  | Author | Susana Alves |
  | Author | Gowri Betrabet Gulwadi |
  | Author | Pia Nilsson |
  | Date | 2022 |
  | Library Catalogue | Google Scholar |
  | URL | https://www.frontiersin.org/articles/10.3389/fpsyg.2021.793175/full |
  | Accessed | 11/07/2025, 11:14:12 |
  | Volume | 12 |
  | Publisher | Frontiers Media SA |
  | Pages | 793175 |
  | Publication | Frontiers in psychology |
  | Date Added | 11/07/2025, 11:14:45 |
  | Modified | 11/07/2025, 11:14:45 |

  ### Attachments

  - Available Version (via Google Scholar)
- ## Pet food palatability evaluation: a review of standard assay techniques and interpretation of results with a primary focus on limitations

  |  |  |
  | --- | --- |
  | Item Type | Journal Article |
  | Author | Gregory C. Aldrich |
  | Author | Kadri Koppel |
  | Date | 2015 |
  | Short Title | Pet food palatability evaluation |
  | Library Catalogue | Google Scholar |
  | URL | https://www.mdpi.com/2076-2615/5/1/43 |
  | Accessed | 11/07/2025, 10:18:15 |
  | Volume | 5 |
  | Publisher | MDPI |
  | Pages | 43–55 |
  | Publication | Animals |
  | Issue | 1 |
  | Date Added | 11/07/2025, 10:18:17 |
  | Modified | 11/07/2025, 10:18:17 |

  ### Attachments

  - Available Version (via Google Scholar)
- ## Animal-Assisted Therapy in Pediatric Care: A qualitative descriptive literature review

  |  |  |
  | --- | --- |
  | Item Type | Journal Article |
  | Author | Romesha Abayanayake |
  | Author | Dilini Weerasooriya |
  | Date | 2024 |
  | Short Title | Animal-Assisted Therapy in Pediatric Care |
  | Library Catalogue | Google Scholar |
  | URL | https://www.theseus.fi/handle/10024/870592 |
  | Accessed | 11/07/2025, 11:09:20 |
  | Date Added | 11/07/2025, 11:09:44 |
  | Modified | 11/07/2025, 11:09:44 |

  ### Attachments

  - Available Version (via Google Scholar)
- ## Animal-Assisted Therapy in Pediatric Care: A qualitative descriptive literature review

  |  |  |
  | --- | --- |
  | Item Type | Journal Article |
  | Author | Romesha Abayanayake |
  | Author | Dilini Weerasooriya |
  | Date | 2024 |
  | Short Title | Animal-Assisted Therapy in Pediatric Care |
  | Library Catalogue | Google Scholar |
  | URL | https://www.theseus.fi/handle/10024/870592 |
  | Accessed | 11/07/2025, 13:44:39 |
  | Date Added | 11/07/2025, 13:45:12 |
  | Modified | 11/07/2025, 13:45:12 |

  ### Attachments

  - Available Version (via Google Scholar)
- ## Research, Policy, and Practice Regarding Rehabilitation of Children with Developmental Disorder in Africa: A Scoping Review

  |  |  |
  | --- | --- |
  | Item Type | Journal Article |
  | Author | Ukachukwu O. Abaraogu |
  | Author | Chidera P. Eleke |
  | Date | 2024 |
  | Short Title | Research, Policy, and Practice Regarding Rehabilitation of Children with Developmental Disorder in Africa |
  | Library Catalogue | Google Scholar |
  | URL | https://www.academia.edu/download/118720658/latest.pdf |
  | Accessed | 11/07/2025, 09:58:10 |
  | Date Added | 11/07/2025, 09:58:25 |
  | Modified | 11/07/2025, 09:58:25 |

  ### Attachments

  - Available Version (via Google Scholar)
