# Supplementary material for: Evidence of Physiological Comodulation During Human–Animal Interaction: A Systematic Review
Source: Ann N Y Acad Sci. 2026 Jun 4;1560(1):e70299. doi: 10.1111/nyas.70299 (PMC13238372; doi:10.1111/nyas.70299)
Supplement: Supplementary file 2 — Supplementary Materials: Supp2‐Zotero‐Collection.zip [file NYAS-1560-0-s002.zip › Supp2_Zotero_Collection/title screened/PubMed.htm]

Zotero Report


- ## Are Hair Cortisol Levels of Humans, Cats, and Dogs from the Same Household Correlated?

  |  |  |
  | --- | --- |
  | Item Type | Journal Article |
  | Author | Justyna Wojtaś |
  | Author | Aleksandra Garbiec |
  | Author | Mirosław Karpiński |
  | Author | Patrycja Skowronek |
  | Author | Aneta Strachecka |
  | Abstract | Human-animal interactions and the emotional relationship of the owner with the pet are the subjects of many scientific studies and the constant interest of not only scientists but also pet owners. The aim of this study was to determine and compare the hair cortisol levels of dogs, cats, and their owners living in the same household. The owners were asked to complete a questionnaire concerning the frequency of their interactions with pets and emotional relationship with each of their cats and each of their dogs. The study involved 25 women who owned at least one dog and at least one cat. In total, 45 dogs and 55 cats from 25 households participated in the study. The average level of hair cortisol of the owners was 4.62 ng/mL, of the dogs 0.26 ng/mL, and in the hair of cats 0.45 ng/mL. There was no significant correlation between the hair cortisol level of the owner and dog or the owner and the cat and between dogs and cats living together. A significant positive correlation was observed between the hair cortisol level in the owner and the pet, for dogs in which the owner performs grooming treatments once a week and for cats which are never kissed. Although our study did not find many significant correlations, studies using other stress markers might have yielded different results. |
  | Date | 2022-06-06 |
  | Language | eng |
  | Library Catalogue | PubMed |
  | Volume | 12 |
  | Pages | 1472 |
  | Publication | Animals: an open access journal from MDPI |
  | DOI | 10.3390/ani12111472 |
  | Issue | 11 |
  | Journal Abbr | Animals (Basel) |
  | ISSN | 2076-2615 |
  | PMID | 35681936 |
  | PMCID | PMC9179579 |
  | Date Added | 11/07/2025, 14:21:52 |
  | Modified | 11/07/2025, 14:21:52 |

  ### Tags:

  - human-animal interaction
  - dog
  - cat
  - hair cortisol level

  ### Attachments

  - Full Text
  - PubMed entry
- ## The blockade of oxytocin receptors in the paraventricular thalamus reduces maternal crouching behavior over pups in lactating mice

  |  |  |
  | --- | --- |
  | Item Type | Journal Article |
  | Author | Akiyuki Watarai |
  | Author | Satoko Tsutaki |
  | Author | Katsuhiko Nishimori |
  | Author | Teruhiro Okuyama |
  | Author | Kazutaka Mogi |
  | Author | Takefumi Kikusui |
  | Abstract | Oxytocin (OT) systems contribute to the elicitation of stereotypic maternal behaviors. OT peptide-expressing neurons are predominantly localized in the hypothalamus, whereas OT receptor (OTR)-expressing neurons are widely distributed throughout the brain. Among those OTR-expressing regions, the paraventricular thalamus (PVT) consists of heterogeneous neuropeptide-responsive neurons critical for appetitive motivation, food intake control, and social behaviors; however, the precise distribution of OTR-expressing neurons within the PVT and whether these neurons are involved in maternal behaviors in mice are unknown. The distribution of OTR-expressing neurons was examined in an OTR-Venus transgenic line expressing a fluorescent protein controlled by the OTR promoter. The number of Venus expressing neurons was higher in the posterior PVT (pPVT) than in the anterior PVT (aPVT). When OTR-Venus dams were exposed to pups, the number of double-labelled neurons expressing both OTR-Venus and a marker of neuronal activity (c-Fos) was increased in the pPVT compared to non-exposed dams, while the aPVT remained unchanged. To investigate whether OT signaling in the pPVT is essential for maternal behaviors, an OT antagonist (OTA) was transiently or chronically infused into the pPVT of lactating dams during the postpartum period. Although the transient OTR blockade did not affect maternal behaviors, a chronic OTR blockade specifically reduced the duration of crouching behavior over pups. Taken together, these findings suggest that OTR-expressing neurons in the pPVT are involved in maternal crouching behavior. |
  | Date | 2020-02-16 |
  | Language | eng |
  | Library Catalogue | PubMed |
  | Volume | 720 |
  | Pages | 134761 |
  | Publication | Neuroscience Letters |
  | DOI | 10.1016/j.neulet.2020.134761 |
  | Journal Abbr | Neurosci Lett |
  | ISSN | 1872-7972 |
  | PMID | 31952987 |
  | Date Added | 11/07/2025, 11:45:53 |
  | Modified | 11/07/2025, 11:45:59 |

  ### Tags:

  - Oxytocin
  - Animals
  - Female
  - Receptors, Oxytocin
  - Lactation
  - Crouching behavior
  - Maternal behavior
  - Maternal Behavior
  - Mice, Inbred C57BL
  - Mice, Transgenic
  - Midline Thalamic Nuclei
  - Neurons
  - Paraventricular thalamus

  ### Attachments

  - PubMed entry
- ## Psychophysiological effects of human-animal interaction: theoretical issues and long-term interaction effects

  |  |  |
  | --- | --- |
  | Item Type | Journal Article |
  | Author | Javier Virués-Ortega |
  | Author | Gualberto Buela-Casal |
  | Abstract | This paper reviews literature published on the psychophysiological effects of long-term human-animal interaction (i.e., pet ownership, pet adoption). A literature search was conducted using PsycInfo and Medline databases. Although the available evidence is far from being consistent, it can be concluded that, in some cases, long-term relationships with animals may moderate baseline physiological variables, particularly blood pressure. Results proved more coherent in studies where animals were adopted by owners as part of the procedure. This paper examines existing hypotheses seeking to account for these effects and the supporting evidence. Two major hypotheses have been suggested to explain the psychophysiological effects of long-term interaction, namely (1) stress-buffering effects of noncritical social support provided by pets; and (2) classical conditioning of relaxation. These mechanisms may partially account for the long-term health outcomes observed in a number of human-animal interaction studies. |
  | Date | 2006-01 |
  | Language | eng |
  | Short Title | Psychophysiological effects of human-animal interaction |
  | Library Catalogue | PubMed |
  | Volume | 194 |
  | Pages | 52-57 |
  | Publication | The Journal of Nervous and Mental Disease |
  | DOI | 10.1097/01.nmd.0000195354.03653.63 |
  | Issue | 1 |
  | Journal Abbr | J Nerv Ment Dis |
  | ISSN | 0022-3018 |
  | PMID | 16462556 |
  | Date Added | 11/07/2025, 11:37:24 |
  | Modified | 11/07/2025, 11:37:24 |

  ### Tags:

  - Relaxation
  - Animals
  - Heart Rate
  - Human-Animal Bond
  - Humans
  - Adaptation, Psychological
  - Animals, Domestic
  - Blood Pressure
  - Cardiovascular Physiological Phenomena
  - Conditioning, Classical
  - Models, Biological
  - Psychophysiology
  - Social Support
  - Stress Disorders, Traumatic, Acute
  - Stress, Psychological

  ### Attachments

  - PubMed entry
- ## Identification of genes associated with human-canine communication in canine evolution

  |  |  |
  | --- | --- |
  | Item Type | Journal Article |
  | Author | Akiko Tonoike |
  | Author | Ken-Ichi Otaki |
  | Author | Go Terauchi |
  | Author | Misato Ogawa |
  | Author | Maki Katayama |
  | Author | Hikari Sakata |
  | Author | Fumina Miyasako |
  | Author | Kazutaka Mogi |
  | Author | Takefumi Kikusui |
  | Author | Miho Nagasawa |
  | Abstract | The dog (Canis familiaris) was the first domesticated animal and hundreds of breeds exist today. During domestication, dogs experienced strong selection for temperament, behaviour, and cognitive ability. However, the genetic basis of these abilities is not well-understood. We focused on ancient dog breeds to investigate breed-related differences in social cognitive abilities. In a problem-solving task, ancient breeds showed a lower tendency to look back at humans than other European breeds. In a two-way object choice task, they showed no differences in correct response rate or ability to read human communicative gestures. We examined gene polymorphisms in oxytocin, oxytocin receptor, melanocortin 2 receptor, and a Williams-Beuren syndrome-related gene (WBSCR17), as candidate genes of dog domestication. The single-nucleotide polymorphisms on melanocortin 2 receptor were related to both tasks, while other polymorphisms were associated with the unsolvable task. This indicates that glucocorticoid functions are involved in the cognitive skills acquired during dog domestication. |
  | Date | 2022-06-09 |
  | Language | eng |
  | Library Catalogue | PubMed |
  | Volume | 12 |
  | Pages | 6950 |
  | Publication | Scientific Reports |
  | DOI | 10.1038/s41598-022-11130-x |
  | Issue | 1 |
  | Journal Abbr | Sci Rep |
  | ISSN | 2045-2322 |
  | PMID | 35680934 |
  | PMCID | PMC9184530 |
  | Date Added | 11/07/2025, 11:45:53 |
  | Modified | 11/07/2025, 11:45:58 |

  ### Tags:

  - Oxytocin
  - Dogs
  - Animals
  - Behavior, Animal
  - Human-Animal Interaction
  - Humans
  - Animals, Domestic
  - Receptors, Oxytocin
  - Domestication
  - Communication
  - Gestures
  - N-Acetylgalactosaminyltransferases
  - Polymorphism, Single Nucleotide
  - Polypeptide N-acetylgalactosaminyltransferase
  - Receptor, Melanocortin, Type 2

  ### Attachments

  - Full Text
  - PubMed entry
- ## Nasally-Administered Oxytocin Has Limited Effects on Owner-Directed Attachment Behavior in Pet Dogs (Canis lupus familiaris)

  |  |  |
  | --- | --- |
  | Item Type | Journal Article |
  | Author | Lauren E. Thielke |
  | Author | Giovanna Rosenlicht |
  | Author | Sarina R. Saturn |
  | Author | Monique A. R. Udell |
  | Abstract | The present study explored the effects of intranasal oxytocin, a naturally occurring hormone, on the behavior of pet dogs during an attachment test. Each dog participated in two testing sessions. On one visit saline was administered nasally, and on another, oxytocin was administered nasally. For half of the dogs (n = 20), solutions were administered with a Mucosal Atomization Device (MAD) and for half of the dogs (n = 20), solutions were administered using a nasal spray bottle. Condition order was counterbalanced and a double-blind methodology was employed. Following a 30-min wait period after administration of solutions, dog-owner pairs participated in the Secure Base Test, a short attachment test consisting of three 2-min phases: (1) Baseline- the owner was present, dogs were able to freely explore the testing room (2) Alone- dogs were left alone in the testing room (3) Return- owners re-entered the room and were reunited with their dog. In each phase the dog was evaluated for contact seeking, exploration, and avoidance behaviors. Although, oxytocin administration was expected to increase owner-directed proximity and contact seeking behavior, this effect was not observed. In fact, in the baseline phase, dogs spent significantly more time seeking the proximity of their owners when they received saline than when they received OT (p < 0.05). Sex differences were also assessed for the behavioral variables of interest in the Secure Base Test, and results indicated that OT did not affect dogs' behavior in the alone phase, but when saline was administered, females spent significantly more time in contact with the door than males in the alone phase (p < 0.05). Overall, the effects of nasally administered oxytocin on attachment related behavior appeared to be limited or inconsistent for this pet dog population. |
  | Date | 2017 |
  | Language | eng |
  | Library Catalogue | PubMed |
  | Volume | 8 |
  | Pages | 1699 |
  | Publication | Frontiers in Psychology |
  | DOI | 10.3389/fpsyg.2017.01699 |
  | Journal Abbr | Front Psychol |
  | ISSN | 1664-1078 |
  | PMID | 29033879 |
  | PMCID | PMC5626864 |
  | Date Added | 11/07/2025, 11:45:53 |
  | Modified | 11/07/2025, 11:45:59 |

  ### Tags:

  - oxytocin
  - dogs
  - social behavior
  - attachment style
  - human-animal interactions
  - secure base

  ### Attachments

  - Full Text
  - PubMed entry
- ## The role of oxytocin in relationships between dogs and humans and potential applications for the treatment of separation anxiety in dogs

  |  |  |
  | --- | --- |
  | Item Type | Journal Article |
  | Author | Lauren E. Thielke |
  | Author | Monique A. R. Udell |
  | Abstract | The hormone oxytocin plays an important role in attachment formation and bonding between humans and domestic dogs. Recent research has led to increased interest in potential applications for intranasal oxytocin to aid with the treatment of psychological disorders in humans. While a few studies have explored the effects of intranasally administered oxytocin on social cognition and social bonding in dogs, alternative applications have not yet been explored for the treatment of behavioural problems in this species. One potentially important application for intranasal oxytocin in dogs could be the treatment of separation anxiety, a common attachment disorder in dogs. Here we provide an overview of what is known about the role of oxytocin in the human-dog bond and canine separation anxiety, and discuss considerations for future research looking to integrate oxytocin into behavioural treatment based on recent findings from both the human and dog literature. |
  | Date | 2017-02 |
  | Language | eng |
  | Library Catalogue | PubMed |
  | Volume | 92 |
  | Pages | 378-388 |
  | Publication | Biological Reviews of the Cambridge Philosophical Society |
  | DOI | 10.1111/brv.12235 |
  | Issue | 1 |
  | Journal Abbr | Biol Rev Camb Philos Soc |
  | ISSN | 1469-185X |
  | PMID | 26548910 |
  | Date Added | 11/07/2025, 11:45:53 |
  | Modified | 11/07/2025, 11:45:57 |

  ### Tags:

  - oxytocin
  - Oxytocin
  - Dogs
  - human-animal interaction
  - Animals
  - Human-Animal Bond
  - Humans
  - dog
  - Anxiety, Separation
  - Canis lupus familiaris
  - separation anxiety

  ### Attachments

  - PubMed entry
- ## Brain and heart activity during interactions with pet dogs: A portable electroencephalogram and heart rate variability study

  |  |  |
  | --- | --- |
  | Item Type | Journal Article |
  | Author | Jillian T. Teo |
  | Author | Stuart J. Johnstone |
  | Author | Susan J. Thomas |
  | Abstract | Dog ownership has been linked to numerous benefits to human health and wellbeing. However, due to the lack of previous research on changes to brain activity during interactions with pet dogs, the underlying psychophysiological mechanisms are still unclear. The aim of the present study was to examine changes in heart rate (HR), heart rate variability (HRV), and electroencephalogram (EEG) power during interactions between dog owners and their pet dog. Fifty healthy adult dog owners completed baseline psychological measures and pet attachment scales. Subjective units of relaxation (SUR) as well as continuous EEG, HR, and HRV via portable devices were measured during five experimental conditions (baseline resting, relaxation-induction exercise, patting a toy dog, real dog present, and patting a real dog) in participants' homes. SUR was higher in all experimental conditions than at baseline. SUR was also higher during dog interaction than when the dog was present with no interaction. However, SUR during dog interaction was not significantly different from the toy dog and relaxation induction condition. Higher delta, theta, alpha, beta power and HR were found during dog interaction than all other conditions. Higher HRV was found during dog interaction compared to baseline, patting a toy dog, and relaxation-induction exercise, but not significantly different from the real dog present only condition. Lastly, overall HR correlated with psychological measures. Overall, the results show that there are significant changes in brain and heart activity when humans interact with pet dogs, consistent with increases in relaxation and focussed attention. These findings are relevant to understanding the potential mechanisms for health benefits associated with pets. |
  | Date | 2024-10 |
  | Language | eng |
  | Short Title | Brain and heart activity during interactions with pet dogs |
  | Library Catalogue | PubMed |
  | Volume | 204 |
  | Pages | 112412 |
  | Publication | International Journal of Psychophysiology: Official Journal of the International Organization of Psychophysiology |
  | DOI | 10.1016/j.ijpsycho.2024.112412 |
  | Journal Abbr | Int J Psychophysiol |
  | ISSN | 1872-7697 |
  | PMID | 39111638 |
  | Date Added | 11/07/2025, 11:33:54 |
  | Modified | 11/07/2025, 11:33:54 |

  ### Tags:

  - Dogs
  - Electroencephalography
  - Heart rate variability
  - Pets
  - Portable device
  - Relaxation
  - Animals
  - Brain
  - Female
  - Adult
  - Heart Rate
  - Human-Animal Bond
  - Human-Animal Interaction
  - Humans
  - Male
  - Middle Aged
  - Young Adult

  ### Attachments

  - PubMed entry
- ## Brain and heart activity during interactions with pet dogs: A portable electroencephalogram and heart rate variability study

  |  |  |
  | --- | --- |
  | Item Type | Journal Article |
  | Author | Jillian T. Teo |
  | Author | Stuart J. Johnstone |
  | Author | Susan J. Thomas |
  | Abstract | Dog ownership has been linked to numerous benefits to human health and wellbeing. However, due to the lack of previous research on changes to brain activity during interactions with pet dogs, the underlying psychophysiological mechanisms are still unclear. The aim of the present study was to examine changes in heart rate (HR), heart rate variability (HRV), and electroencephalogram (EEG) power during interactions between dog owners and their pet dog. Fifty healthy adult dog owners completed baseline psychological measures and pet attachment scales. Subjective units of relaxation (SUR) as well as continuous EEG, HR, and HRV via portable devices were measured during five experimental conditions (baseline resting, relaxation-induction exercise, patting a toy dog, real dog present, and patting a real dog) in participants' homes. SUR was higher in all experimental conditions than at baseline. SUR was also higher during dog interaction than when the dog was present with no interaction. However, SUR during dog interaction was not significantly different from the toy dog and relaxation induction condition. Higher delta, theta, alpha, beta power and HR were found during dog interaction than all other conditions. Higher HRV was found during dog interaction compared to baseline, patting a toy dog, and relaxation-induction exercise, but not significantly different from the real dog present only condition. Lastly, overall HR correlated with psychological measures. Overall, the results show that there are significant changes in brain and heart activity when humans interact with pet dogs, consistent with increases in relaxation and focussed attention. These findings are relevant to understanding the potential mechanisms for health benefits associated with pets. |
  | Date | 2024-10 |
  | Language | eng |
  | Short Title | Brain and heart activity during interactions with pet dogs |
  | Library Catalogue | PubMed |
  | Volume | 204 |
  | Pages | 112412 |
  | Publication | International Journal of Psychophysiology: Official Journal of the International Organization of Psychophysiology |
  | DOI | 10.1016/j.ijpsycho.2024.112412 |
  | Journal Abbr | Int J Psychophysiol |
  | ISSN | 1872-7697 |
  | PMID | 39111638 |
  | Date Added | 11/07/2025, 11:37:24 |
  | Modified | 11/07/2025, 11:37:24 |

  ### Tags:

  - Dogs
  - Electroencephalography
  - Heart rate variability
  - Pets
  - Portable device
  - Relaxation
  - Animals
  - Brain
  - Female
  - Adult
  - Heart Rate
  - Human-Animal Bond
  - Human-Animal Interaction
  - Humans
  - Male
  - Middle Aged
  - Young Adult

  ### Attachments

  - PubMed entry
- ## Psychophysiological mechanisms underlying the potential health benefits of human-dog interactions: A systematic literature review

  |  |  |
  | --- | --- |
  | Item Type | Journal Article |
  | Author | Jillian T. Teo |
  | Author | Stuart J. Johnstone |
  | Author | Stephanie S. Römer |
  | Author | Susan J. Thomas |
  | Abstract | While the symbiotic nature of human-dog relationships and perceived benefits to human health have attracted much scientific interest, the mechanisms through which human-dog interactions may confer health benefits to humans are still poorly understood. The aim of this systematic literature review was to synthesize evidence of physiological changes associated with human-dog interactions with relevance to human health. Electronic databases were systematically searched (PubMed, MEDLINE with full text, Scopus, PsycINFO, CINAHL Plus with Full Text, and Web of Science Core Collection) for relevant studies. Of the 13,072 studies identified, 129 met the inclusion criteria, with approximately half being randomized trials (Level 2) based on the Oxford Centre for Evidence Based Medicine level system. Measures employed to study human physiological changes associated with human-dog interactions most commonly involved cardiac parameters and hormones, with negligible research of brain activity. The main positive findings were increases in heart rate variability and oxytocin, and decreases in cortisol with human-dog interactions. These physiological indicators are consistent with activation of the parasympathetic nervous system (PNS) and oxytocinergic system (OTS), and down-regulation of hypothalamic-pituitary-adrenal (HPA) axis activity. These results provide evidence of specific pathways through which human-dog contact may confer health benefits, likely through relaxation, bonding, and stress reduction. However, these findings should be interpreted contextually due to limitations and methodological differences. Previous research using other biological variables was limited in quantity and quality, thus impeding firm conclusions on other possible mechanisms. Further research is needed in some psychophysiological domains, particularly electroencephalography, to better understand central nervous system (CNS) effects. The findings of this review have implications for human-dog interactions to positively affect several stress-sensitive physiological pathways and thus confer health benefits. This supports their incorporation in various clinical, non-clinical, and research settings to develop evidence-based interventions and practices for cost-effective and efficacious ways to improve human health. |
  | Date | 2022-10 |
  | Language | eng |
  | Short Title | Psychophysiological mechanisms underlying the potential health benefits of human-dog interactions |
  | Library Catalogue | PubMed |
  | Volume | 180 |
  | Pages | 27-48 |
  | Publication | International Journal of Psychophysiology: Official Journal of the International Organization of Psychophysiology |
  | DOI | 10.1016/j.ijpsycho.2022.07.007 |
  | Journal Abbr | Int J Psychophysiol |
  | ISSN | 1872-7697 |
  | PMID | 35901904 |
  | Date Added | 11/07/2025, 11:37:24 |
  | Modified | 11/07/2025, 11:37:24 |

  ### Tags:

  - Dogs
  - Animals
  - Humans
  - Psychophysiology
  - Animal-assisted intervention
  - Human-animal interaction
  - Systematic review

  ### Attachments

  - PubMed entry
- ## Psychophysiological mechanisms underlying the potential health benefits of human-dog interactions: A systematic literature review

  |  |  |
  | --- | --- |
  | Item Type | Journal Article |
  | Author | Jillian T. Teo |
  | Author | Stuart J. Johnstone |
  | Author | Stephanie S. Römer |
  | Author | Susan J. Thomas |
  | Abstract | While the symbiotic nature of human-dog relationships and perceived benefits to human health have attracted much scientific interest, the mechanisms through which human-dog interactions may confer health benefits to humans are still poorly understood. The aim of this systematic literature review was to synthesize evidence of physiological changes associated with human-dog interactions with relevance to human health. Electronic databases were systematically searched (PubMed, MEDLINE with full text, Scopus, PsycINFO, CINAHL Plus with Full Text, and Web of Science Core Collection) for relevant studies. Of the 13,072 studies identified, 129 met the inclusion criteria, with approximately half being randomized trials (Level 2) based on the Oxford Centre for Evidence Based Medicine level system. Measures employed to study human physiological changes associated with human-dog interactions most commonly involved cardiac parameters and hormones, with negligible research of brain activity. The main positive findings were increases in heart rate variability and oxytocin, and decreases in cortisol with human-dog interactions. These physiological indicators are consistent with activation of the parasympathetic nervous system (PNS) and oxytocinergic system (OTS), and down-regulation of hypothalamic-pituitary-adrenal (HPA) axis activity. These results provide evidence of specific pathways through which human-dog contact may confer health benefits, likely through relaxation, bonding, and stress reduction. However, these findings should be interpreted contextually due to limitations and methodological differences. Previous research using other biological variables was limited in quantity and quality, thus impeding firm conclusions on other possible mechanisms. Further research is needed in some psychophysiological domains, particularly electroencephalography, to better understand central nervous system (CNS) effects. The findings of this review have implications for human-dog interactions to positively affect several stress-sensitive physiological pathways and thus confer health benefits. This supports their incorporation in various clinical, non-clinical, and research settings to develop evidence-based interventions and practices for cost-effective and efficacious ways to improve human health. |
  | Date | 2022-10 |
  | Language | eng |
  | Short Title | Psychophysiological mechanisms underlying the potential health benefits of human-dog interactions |
  | Library Catalogue | PubMed |
  | Volume | 180 |
  | Pages | 27-48 |
  | Publication | International Journal of Psychophysiology: Official Journal of the International Organization of Psychophysiology |
  | DOI | 10.1016/j.ijpsycho.2022.07.007 |
  | Journal Abbr | Int J Psychophysiol |
  | ISSN | 1872-7697 |
  | PMID | 35901904 |
  | Date Added | 11/07/2025, 11:45:53 |
  | Modified | 11/07/2025, 11:45:58 |

  ### Tags:

  - Dogs
  - Animals
  - Humans
  - Psychophysiology
  - Animal-assisted intervention
  - Human-animal interaction
  - Systematic review

  ### Attachments

  - PubMed entry
- ## Psychophysiological mechanisms underlying the potential health benefits of human-dog interactions: A systematic literature review

  |  |  |
  | --- | --- |
  | Item Type | Journal Article |
  | Author | Jillian T. Teo |
  | Author | Stuart J. Johnstone |
  | Author | Stephanie S. Römer |
  | Author | Susan J. Thomas |
  | Abstract | While the symbiotic nature of human-dog relationships and perceived benefits to human health have attracted much scientific interest, the mechanisms through which human-dog interactions may confer health benefits to humans are still poorly understood. The aim of this systematic literature review was to synthesize evidence of physiological changes associated with human-dog interactions with relevance to human health. Electronic databases were systematically searched (PubMed, MEDLINE with full text, Scopus, PsycINFO, CINAHL Plus with Full Text, and Web of Science Core Collection) for relevant studies. Of the 13,072 studies identified, 129 met the inclusion criteria, with approximately half being randomized trials (Level 2) based on the Oxford Centre for Evidence Based Medicine level system. Measures employed to study human physiological changes associated with human-dog interactions most commonly involved cardiac parameters and hormones, with negligible research of brain activity. The main positive findings were increases in heart rate variability and oxytocin, and decreases in cortisol with human-dog interactions. These physiological indicators are consistent with activation of the parasympathetic nervous system (PNS) and oxytocinergic system (OTS), and down-regulation of hypothalamic-pituitary-adrenal (HPA) axis activity. These results provide evidence of specific pathways through which human-dog contact may confer health benefits, likely through relaxation, bonding, and stress reduction. However, these findings should be interpreted contextually due to limitations and methodological differences. Previous research using other biological variables was limited in quantity and quality, thus impeding firm conclusions on other possible mechanisms. Further research is needed in some psychophysiological domains, particularly electroencephalography, to better understand central nervous system (CNS) effects. The findings of this review have implications for human-dog interactions to positively affect several stress-sensitive physiological pathways and thus confer health benefits. This supports their incorporation in various clinical, non-clinical, and research settings to develop evidence-based interventions and practices for cost-effective and efficacious ways to improve human health. |
  | Date | 2022-10 |
  | Language | eng |
  | Short Title | Psychophysiological mechanisms underlying the potential health benefits of human-dog interactions |
  | Library Catalogue | PubMed |
  | Volume | 180 |
  | Pages | 27-48 |
  | Publication | International Journal of Psychophysiology: Official Journal of the International Organization of Psychophysiology |
  | DOI | 10.1016/j.ijpsycho.2022.07.007 |
  | Journal Abbr | Int J Psychophysiol |
  | ISSN | 1872-7697 |
  | PMID | 35901904 |
  | Date Added | 11/07/2025, 14:21:52 |
  | Modified | 11/07/2025, 14:21:52 |

  ### Tags:

  - Dogs
  - Animals
  - Humans
  - Psychophysiology
  - Animal-assisted intervention
  - Human-animal interaction
  - Systematic review

  ### Attachments

  - PubMed entry
- ## Dog-Owner Relationship, Owner Interpretations and Dog Personality Are Connected with the Emotional Reactivity of Dogs

  |  |  |
  | --- | --- |
  | Item Type | Journal Article |
  | Author | Sanni Somppi |
  | Author | Heini Törnqvist |
  | Author | Aija Koskela |
  | Author | Antti Vehkaoja |
  | Author | Katriina Tiira |
  | Author | Heli Väätäjä |
  | Author | Veikko Surakka |
  | Author | Outi Vainio |
  | Author | Miiamaaria V. Kujala |
  | Abstract | We evaluated the effect of the dog-owner relationship on dogs' emotional reactivity, quantified with heart rate variability (HRV), behavioral changes, physical activity and dog owner interpretations. Twenty nine adult dogs encountered five different emotional situations (i.e., stroking, a feeding toy, separation from the owner, reunion with the owner, a sudden appearance of a novel object). The results showed that both negative and positive situations provoked signs of heightened arousal in dogs. During negative situations, owners' ratings about the heightened emotional arousal correlated with lower HRV, higher physical activity and more behaviors that typically index arousal and fear. The three factors of The Monash Dog-Owner Relationship Scale (MDORS) were reflected in the dogs' heart rate variability and behaviors: the Emotional Closeness factor was related to increased HRV (p = 0.009), suggesting this aspect is associated with the secure base effect, and the Shared Activities factor showed a trend toward lower HRV (p = 0.067) along with more owner-directed behaviors reflecting attachment related arousal. In contrast, the Perceived Costs factor was related to higher HRV (p = 0.009) along with less fear and less owner-directed behaviors, which may reflect the dog's more independent personality. In conclusion, dogs' emotional reactivity and the dog-owner relationship modulate each other, depending on the aspect of the relationship and dogs' individual responsivity. |
  | Date | 2022-05-24 |
  | Language | eng |
  | Library Catalogue | PubMed |
  | Volume | 12 |
  | Pages | 1338 |
  | Publication | Animals: an open access journal from MDPI |
  | DOI | 10.3390/ani12111338 |
  | Issue | 11 |
  | Journal Abbr | Animals (Basel) |
  | ISSN | 2076-2615 |
  | PMID | 35681804 |
  | PMCID | PMC9179432 |
  | Date Added | 11/07/2025, 11:37:24 |
  | Modified | 11/07/2025, 11:37:24 |

  ### Tags:

  - human–animal interaction
  - heart rate variability
  - autonomic nervous system
  - behavior
  - Canis familiaris
  - emotions

  ### Attachments

  - Full Text
  - PubMed entry
- ## Best practices for physiological data collection in youth with autism and co-occurring mental health diagnoses: Implications for human-animal intervention research

  |  |  |
  | --- | --- |
  | Item Type | Journal Article |
  | Author | Cory M. Smith |
  | Author | Katharine Weimann |
  | Author | Madison Widick |
  | Author | Tamara Merritt |
  | Author | Hannah Christensen |
  | Author | Matthew Siegel |
  | Author | Zhaoxing Pan |
  | Author | Robin L. Gabriels |
  | Abstract | The purpose of this paper is to serve as a catalyst for the human-animal interaction research field to improve scientific rigor and accelerate the knowledge of field-based physiological responses during equine-assisted services in youth with autism spectrum disorder. This paper outlines the best practices for collecting and analyzing electrocardiogram and electrodermal activity in youth with autism spectrum disorder, utilized during a 10-week therapeutic horseback riding intervention.•Motivation strategies such as device choice, reward systems, and a visual schedule should be implemented to improve participant compliance. In addition, devices should be secured to the participant following implementation of appropriate desensitization techniques.•Time-domain heart rate variability analyses are more appropriate during therapeutic horseback riding data collection compared to frequency-domain approaches. For electrodermal activity, tonic responses should be assessed as opposed to phasic analyses.•An effective data monitoring team including the Data Collection Research Personnel, Site Principal Investigator, Physiologist, and Therapeutic Riding Center Intervention Lead are key to increasing the quality of usable data in equine-assisted service research environments. |
  | Date | 2025-06 |
  | Language | eng |
  | Short Title | Best practices for physiological data collection in youth with autism and co-occurring mental health diagnoses |
  | Library Catalogue | PubMed |
  | Volume | 14 |
  | Pages | 103284 |
  | Publication | MethodsX |
  | DOI | 10.1016/j.mex.2025.103284 |
  | Journal Abbr | MethodsX |
  | ISSN | 2215-0161 |
  | PMID | 40236803 |
  | PMCID | PMC11999311 |
  | Date Added | 11/07/2025, 11:37:24 |
  | Modified | 11/07/2025, 11:37:24 |

  ### Tags:

  - Heart rate variability
  - ASD
  - Electrocardiogram
  - Electrodermal activity
  - Galvanic skin response
  - Human animal interventions
  - Stress response
  - Team dynamics
  - THR, Horseback riding
  - Wearable Physiological Monitoring during Equine-Assisted Services.

  ### Attachments

  - PubMed entry
- ## Inside the Interaction: Contact With Familiar Humans Modulates Heart Rate Variability in Horses

  |  |  |
  | --- | --- |
  | Item Type | Journal Article |
  | Author | Chiara Scopa |
  | Author | Alberto Greco |
  | Author | Laura Contalbrigo |
  | Author | Elisabetta Fratini |
  | Author | Antonio Lanatà |
  | Author | Enzo Pasquale Scilingo |
  | Author | Paolo Baragli |
  | Abstract | A human-animal relationship can be developed through subsequent interactions, affected by the positive or negative emotional valence of the proceeding one. Horses implement a process of categorization to classify humans with whom they interact as positive, negative, or neutral stimuli by evaluating the kind of approach and the nature of the contact. In these terms, human-animal interactions are emotionally charged events, eliciting specific emotional states in both subjects involved. Although the human-horse relationship has been mainly investigated through behavioral analysis, physiological indicators are needed for a more objective assessment of the emotional responses. Heart rate variability (HRV) is a commonly used autonomic nervous system (ANS) correlate estimating the sympathovagal balance as a psychophysiological marker of emotion regulation in horses. We have assumed that long-term positive relationships with humans may have a positive and immediate impact on the emotional arousal of the horse, detectable, via ANS activity, during the interaction. We analyzed horses' heartbeat dynamics during their interaction with either familiar or unfamiliar handlers, applying a standardized experimental protocol consisting of three different conditions shifting from the absence of interaction to physical contact. The ANS signals were monitored through an innovative non-invasive wearable system, not interfering with the unconscious emotional response of the animal. We demonstrated that horses appeared to feel more relaxed while physically interacting (e.g., grooming on the right side) with some familiar handlers compared to the same task performed by someone unfamiliar. The shift of the sympathovagal balance toward a vagal predominance suggests that the horses experienced a decrease in stress response as a function not only of the handler's familiarity but also of the type of interaction they are experiencing. These results constitute the objective evidence of horses' capacity to individually recognize a familiar person, adding the crucial role of familiarity with the handler as a paramount component of human-animal interaction. Our rigorous methodological approach may provide a significant contribution to various fields such as animal welfare while further investigating the emotional side of the human-animal relationships. |
  | Date | 2020 |
  | Language | eng |
  | Short Title | Inside the Interaction |
  | Library Catalogue | PubMed |
  | Volume | 7 |
  | Pages | 582759 |
  | Publication | Frontiers in Veterinary Science |
  | DOI | 10.3389/fvets.2020.582759 |
  | Journal Abbr | Front Vet Sci |
  | ISSN | 2297-1769 |
  | PMID | 33330706 |
  | PMCID | PMC7734029 |
  | Date Added | 11/07/2025, 11:37:24 |
  | Modified | 11/07/2025, 11:37:24 |

  ### Tags:

  - autonomic nervous system
  - emotional valence
  - Equus caballus
  - heartbeat dynamics
  - human–animal relationship
  - inter-specific interaction

  ### Attachments

  - Full Text
  - PubMed entry
- ## Minor Immediate Effects of a Dog on Children's Reading Performance and Physiology

  |  |  |
  | --- | --- |
  | Item Type | Journal Article |
  | Author | Lisa Schretzmayer |
  | Author | Kurt Kotrschal |
  | Author | Andrea Beetz |
  | Abstract | Literacy is a key factor in occupational success and social integration. However, an increasing number of children lack appropriate reading skills. There is growing evidence that dogs have positive effects on reading performance. We investigated the short-term effects of dogs on reading performance in 36 third-graders and monitored physiological parameters [heart rate (HR), heart rate variability (HRV), and salivary cortisol] as well as behavioral variables. Each child took part in two test sessions at the presence of a tutor, in one of which a dog and its handler were present. To assess reading performance two reading tests were used: two subtests of the standardized "Ein Leseverständnistest für Erst- bis Sechstklässler", where the children have to carry out time-limited reading tasks, to assess sentence and text comprehension, and repeated reading (RR), where the children have to read the same text twice, to assess reading speed and short-term improvement. Although the dog had no effect on reading performance scores, within the first test session the children improved from the first to the second run of RR when a dog was present but not without dog. The behavior of the children indicated a calming effect of the dog in the first test session with less nervous movements and the children being less talkative. We found no impact of the dog on HR and HRV. However, the excitement about the dog in combination with the unknown situation in the first test session was reflected in a higher difference in the mean HR difference between the two test sessions for the children, who in the first test session had a dog present, compared to the children, who had the dog in the second test session. In the second test session, the children were more aroused with a dog present than with no dog present, as indicated by the area under the curve increase (AUCi) of salivary cortisol values. We conclude that the presence of a dog had a minor short-term positive effect on the children's motivation and reading performance. More substantial effects could probably be achieved with repeated sessions. |
  | Date | 2017 |
  | Language | eng |
  | Library Catalogue | PubMed |
  | Volume | 4 |
  | Pages | 90 |
  | Publication | Frontiers in Veterinary Science |
  | DOI | 10.3389/fvets.2017.00090 |
  | Journal Abbr | Front Vet Sci |
  | ISSN | 2297-1769 |
  | PMID | 28674695 |
  | PMCID | PMC5475382 |
  | Date Added | 11/07/2025, 11:37:24 |
  | Modified | 11/07/2025, 11:37:24 |

  ### Tags:

  - dogs
  - human–animal interaction
  - animal-assisted interventions
  - behavior
  - children
  - physiological effects
  - reading

  ### Attachments

  - Full Text
  - PubMed entry
- ## Minor Immediate Effects of a Dog on Children's Reading Performance and Physiology

  |  |  |
  | --- | --- |
  | Item Type | Journal Article |
  | Author | Lisa Schretzmayer |
  | Author | Kurt Kotrschal |
  | Author | Andrea Beetz |
  | Abstract | Literacy is a key factor in occupational success and social integration. However, an increasing number of children lack appropriate reading skills. There is growing evidence that dogs have positive effects on reading performance. We investigated the short-term effects of dogs on reading performance in 36 third-graders and monitored physiological parameters [heart rate (HR), heart rate variability (HRV), and salivary cortisol] as well as behavioral variables. Each child took part in two test sessions at the presence of a tutor, in one of which a dog and its handler were present. To assess reading performance two reading tests were used: two subtests of the standardized "Ein Leseverständnistest für Erst- bis Sechstklässler", where the children have to carry out time-limited reading tasks, to assess sentence and text comprehension, and repeated reading (RR), where the children have to read the same text twice, to assess reading speed and short-term improvement. Although the dog had no effect on reading performance scores, within the first test session the children improved from the first to the second run of RR when a dog was present but not without dog. The behavior of the children indicated a calming effect of the dog in the first test session with less nervous movements and the children being less talkative. We found no impact of the dog on HR and HRV. However, the excitement about the dog in combination with the unknown situation in the first test session was reflected in a higher difference in the mean HR difference between the two test sessions for the children, who in the first test session had a dog present, compared to the children, who had the dog in the second test session. In the second test session, the children were more aroused with a dog present than with no dog present, as indicated by the area under the curve increase (AUCi) of salivary cortisol values. We conclude that the presence of a dog had a minor short-term positive effect on the children's motivation and reading performance. More substantial effects could probably be achieved with repeated sessions. |
  | Date | 2017 |
  | Language | eng |
  | Library Catalogue | PubMed |
  | Volume | 4 |
  | Pages | 90 |
  | Publication | Frontiers in Veterinary Science |
  | DOI | 10.3389/fvets.2017.00090 |
  | Journal Abbr | Front Vet Sci |
  | ISSN | 2297-1769 |
  | PMID | 28674695 |
  | PMCID | PMC5475382 |
  | Date Added | 11/07/2025, 14:21:52 |
  | Modified | 11/07/2025, 14:21:52 |

  ### Tags:

  - dogs
  - human–animal interaction
  - animal-assisted interventions
  - behavior
  - children
  - physiological effects
  - reading

  ### Attachments

  - Full Text
  - PubMed entry
- ## Emerging Cardiovascular Risk Research: Impact of Pets on Cardiovascular Risk Prevention

  |  |  |
  | --- | --- |
  | Item Type | Journal Article |
  | Author | Pamela J. Schreiner |
  | Abstract | Animals interact with humans in multiple ways, including as therapy and service animals, commercially as livestock, as wildlife, and in zoos. But the most common interaction is as companion animals in our homes, with an estimated 180 million cats and dogs living in US households. While pet ownership has been reported to have many health benefits, the findings are inconsistent. Cardiovascular risk factors such as lipids, glucose, obesity, and heart rate variability have improved, worsened, or remained the same in the limited number of studies considering companion animals. Physical activity increases have more consistently been linked with dog ownership, although whether this reflects antecedent motivation or direct benefit from the dog is unclear. Allergies and asthma also are variably linked to pet ownership and are confounded by family history of atopy and timing of exposure to pet dander. The benefits of companion animals are most likely to be through reduction in depression, anxiety, and social isolation, but these studies have been largely cross-sectional and may depend on degree of bonding of the owner with the animal. Positive relationships show measurably higher oxytocin with lower cortisol and alpha-amylase levels. Finally, pet ownership is also a marker of better socioeconomic status and family stability, and if companion animals are to provide cardiovascular risk benefit, the route should perhaps be through improved education and opportunity for ownership. |
  | Date | 2016-02 |
  | Language | eng |
  | Short Title | Emerging Cardiovascular Risk Research |
  | Library Catalogue | PubMed |
  | Volume | 10 |
  | Pages | 8 |
  | Publication | Current Cardiovascular Risk Reports |
  | DOI | 10.1007/s12170-016-0489-2 |
  | Issue | 2 |
  | Journal Abbr | Curr Cardiovasc Risk Rep |
  | ISSN | 1932-9520 |
  | PMID | 27547289 |
  | PMCID | PMC4991891 |
  | Date Added | 11/07/2025, 11:37:24 |
  | Modified | 11/07/2025, 11:37:24 |

  ### Tags:

  - Oxytocin
  - Pets
  - Human-animal interaction
  - Asthma
  - Depression
  - Physical activity

  ### Attachments

  - Accepted Version
  - PubMed entry
- ## Emerging Cardiovascular Risk Research: Impact of Pets on Cardiovascular Risk Prevention

  |  |  |
  | --- | --- |
  | Item Type | Journal Article |
  | Author | Pamela J. Schreiner |
  | Abstract | Animals interact with humans in multiple ways, including as therapy and service animals, commercially as livestock, as wildlife, and in zoos. But the most common interaction is as companion animals in our homes, with an estimated 180 million cats and dogs living in US households. While pet ownership has been reported to have many health benefits, the findings are inconsistent. Cardiovascular risk factors such as lipids, glucose, obesity, and heart rate variability have improved, worsened, or remained the same in the limited number of studies considering companion animals. Physical activity increases have more consistently been linked with dog ownership, although whether this reflects antecedent motivation or direct benefit from the dog is unclear. Allergies and asthma also are variably linked to pet ownership and are confounded by family history of atopy and timing of exposure to pet dander. The benefits of companion animals are most likely to be through reduction in depression, anxiety, and social isolation, but these studies have been largely cross-sectional and may depend on degree of bonding of the owner with the animal. Positive relationships show measurably higher oxytocin with lower cortisol and alpha-amylase levels. Finally, pet ownership is also a marker of better socioeconomic status and family stability, and if companion animals are to provide cardiovascular risk benefit, the route should perhaps be through improved education and opportunity for ownership. |
  | Date | 2016-02 |
  | Language | eng |
  | Short Title | Emerging Cardiovascular Risk Research |
  | Library Catalogue | PubMed |
  | Volume | 10 |
  | Pages | 8 |
  | Publication | Current Cardiovascular Risk Reports |
  | DOI | 10.1007/s12170-016-0489-2 |
  | Issue | 2 |
  | Journal Abbr | Curr Cardiovasc Risk Rep |
  | ISSN | 1932-9520 |
  | PMID | 27547289 |
  | PMCID | PMC4991891 |
  | Date Added | 11/07/2025, 11:45:53 |
  | Modified | 11/07/2025, 11:45:59 |

  ### Tags:

  - Oxytocin
  - Pets
  - Human-animal interaction
  - Asthma
  - Depression
  - Physical activity

  ### Attachments

  - Accepted Version
  - PubMed entry
- ## Emerging Cardiovascular Risk Research: Impact of Pets on Cardiovascular Risk Prevention

  |  |  |
  | --- | --- |
  | Item Type | Journal Article |
  | Author | Pamela J. Schreiner |
  | Abstract | Animals interact with humans in multiple ways, including as therapy and service animals, commercially as livestock, as wildlife, and in zoos. But the most common interaction is as companion animals in our homes, with an estimated 180 million cats and dogs living in US households. While pet ownership has been reported to have many health benefits, the findings are inconsistent. Cardiovascular risk factors such as lipids, glucose, obesity, and heart rate variability have improved, worsened, or remained the same in the limited number of studies considering companion animals. Physical activity increases have more consistently been linked with dog ownership, although whether this reflects antecedent motivation or direct benefit from the dog is unclear. Allergies and asthma also are variably linked to pet ownership and are confounded by family history of atopy and timing of exposure to pet dander. The benefits of companion animals are most likely to be through reduction in depression, anxiety, and social isolation, but these studies have been largely cross-sectional and may depend on degree of bonding of the owner with the animal. Positive relationships show measurably higher oxytocin with lower cortisol and alpha-amylase levels. Finally, pet ownership is also a marker of better socioeconomic status and family stability, and if companion animals are to provide cardiovascular risk benefit, the route should perhaps be through improved education and opportunity for ownership. |
  | Date | 2016-02 |
  | Language | eng |
  | Short Title | Emerging Cardiovascular Risk Research |
  | Library Catalogue | PubMed |
  | Volume | 10 |
  | Pages | 8 |
  | Publication | Current Cardiovascular Risk Reports |
  | DOI | 10.1007/s12170-016-0489-2 |
  | Issue | 2 |
  | Journal Abbr | Curr Cardiovasc Risk Rep |
  | ISSN | 1932-9520 |
  | PMID | 27547289 |
  | PMCID | PMC4991891 |
  | Date Added | 11/07/2025, 14:21:52 |
  | Modified | 11/07/2025, 14:21:52 |

  ### Tags:

  - Oxytocin
  - Pets
  - Human-animal interaction
  - Asthma
  - Depression
  - Physical activity

  ### Attachments

  - Accepted Version
  - PubMed entry
- ## Changes in human health parameters associated with a touch tank experience at a zoological institution

  |  |  |
  | --- | --- |
  | Item Type | Journal Article |
  | Author | John M. Sahrmann |
  | Author | Amy Niedbalski |
  | Author | Louise Bradshaw |
  | Author | Rebecca Johnson |
  | Author | Sharon L. Deem |
  | Abstract | Association of Zoos and Aquariums (AZA) institutions provide a variety of benefits to visitors. However, one area that has received little study is the direct human health benefits from zoo and aquarium visits. With the increase in stress related non-infectious diseases in industrialized countries, understanding the extent of these benefits is important. We studied the effects on visitor stress of an experience at a touch tank exhibit featuring stingrays, sharks, and horseshoe crabs. Stress was measured by physiological and psychological parameters. Heart rate was recorded before, during, and after interacting with the animals, and mood was assessed before and after the experience using a psychological instrument. Multilevel models of heart rate show a quadratic trend, with heart rate elevated (b = -3.01, t = 26.4, P < 0.001) and less variable (b = 3.60, t = 15.9, P < 0.001) while touching the animals compared to before or after. Wilcoxon signed-rank tests on mood data suggest that most visitors felt happier (V = 174.5, P < 0.001), more energized (V = 743.5, P < 0.001), and less tense (V = 5618, P < 0.001) after the experience. This suggests that interacting with animals led to a physiological response during interactions reminiscent of a theme park experience along with a decrease in mental stress. The effects of confounding variables such as crowd size are also discussed. Further studies should be conducted to help deepen our understanding of the health benefits of experiences at AZA institutions. |
  | Date | 2016 |
  | Language | eng |
  | Library Catalogue | PubMed |
  | Volume | 35 |
  | Pages | 4-13 |
  | Publication | Zoo Biology |
  | DOI | 10.1002/zoo.21257 |
  | Issue | 1 |
  | Journal Abbr | Zoo Biol |
  | ISSN | 1098-2361 |
  | PMID | 26662049 |
  | Date Added | 11/07/2025, 11:37:24 |
  | Modified | 11/07/2025, 11:37:24 |

  ### Tags:

  - stress
  - Animals
  - Female
  - Adult
  - Heart Rate
  - Humans
  - Male
  - Middle Aged
  - Young Adult
  - Aged
  - Blood Pressure
  - Surveys and Questionnaires
  - Animals, Zoo
  - Happiness
  - Missouri
  - nature deficit disorder
  - One Health
  - physiological
  - psychological
  - Stress, Physiological

  ### Attachments

  - PubMed entry
- ## The Effect of Mental Activation of One's Pet Dog on Stress Reactivity

  |  |  |
  | --- | --- |
  | Item Type | Journal Article |
  | Author | Kerri E. Rodriguez |
  | Author | Dan J. Graham |
  | Author | Rachel G. Lucas-Thompson |
  | Abstract | Research suggests that mental activation of human social support may reduce stress reactivity. However, the extent to which social support from pets elicits a similar effect has been less explored. This study aims to determine whether the mental activation of one's pet dog reduces stress reactivity to a subsequent experimental stressor. In a 2 × 2 design, 132 dog-owning participants (Mage = 20.14; 80% female) were randomly assigned to one of two mental activation conditions (pet dog; general) and one of two stressor conditions (social-evaluative; cognitive). Data were analyzed with two-way ANOVAs with self-reported (positive/negative affect, negative self-evaluation) and physiological (blood pressure, heart rate) dependent variables. Results indicated that participants randomized to the pet dog mental activation condition had smaller decreases in positive affect from baseline to post-stressor compared to the general mental activation condition. However, there were no significant interactions between time and mental activation condition on negative affect, negative self-evaluation, heart rate, or blood pressure. Thus, the mental activation of one's pet dog had a minimal effect on stress reactivity to a cognitive or social-evaluative stressor. Results suggest that the physical presence of an animal may be an essential mechanism underlying the benefits of animal-derived social support. |
  | Date | 2023-10-30 |
  | Language | eng |
  | Library Catalogue | PubMed |
  | Volume | 20 |
  | Pages | 6995 |
  | Publication | International Journal of Environmental Research and Public Health |
  | DOI | 10.3390/ijerph20216995 |
  | Issue | 21 |
  | Journal Abbr | Int J Environ Res Public Health |
  | ISSN | 1660-4601 |
  | PMID | 37947553 |
  | PMCID | PMC10648142 |
  | Date Added | 11/07/2025, 11:37:24 |
  | Modified | 11/07/2025, 11:37:24 |

  ### Tags:

  - Dogs
  - dogs
  - human–animal interaction
  - stress
  - Animals
  - Female
  - Adult
  - Heart Rate
  - Humans
  - Male
  - Young Adult
  - Blood Pressure
  - Social Support
  - Stress, Psychological
  - Self Report
  - social support
  - Surveys and Questionnaires

  ### Attachments

  - Full Text
  - PubMed entry
- ## The effect of a service dog on salivary cortisol awakening response in a military population with posttraumatic stress disorder (PTSD)

  |  |  |
  | --- | --- |
  | Item Type | Journal Article |
  | Author | Kerri E. Rodriguez |
  | Author | Crystal I. Bryce |
  | Author | Douglas A. Granger |
  | Author | Marguerite E. O'Haire |
  | Abstract | Recent studies suggest a therapeutic effect of psychiatric service dogs for military veterans with posttraumatic stress disorder (PTSD), but are limited by self-report biases. The current study assessed the effect of PTSD service dogs on the salivary cortisol awakening response (CAR) and arousal-related functioning in a population of military veterans with PTSD. Participants included 73 post-9/11 military veterans with PTSD including 45 with a service dog and 28 on the waitlist to receive one. Saliva samples were collected on two consecutive weekday mornings at awakening and 30 min later to quantify the cortisol awakening response (CAR) and its area under the curve (AUCi) in addition to standardized survey measures of anxiety, anger, sleep quality and disturbance, and alcohol abuse. There was a significant main effect of having a service dog on both the CAR and the AUCi, with individuals with a service dog exhibiting a higher CAR and AUCi compared to those on the waitlist. Results also revealed that those with a service dog reported significantly lower anxiety, anger, and sleep disturbance as well as less alcohol abuse compared to those on the waitlist, with medium to large effect sizes. Although those with a service dog reported significantly less PTSD symptom severity, CAR was not significantly associated with PTSD symptoms within or across group. In conclusion, results indicate that the placement of a PTSD service dog may have a significant positive influence on both physiological and psychosocial indicators of wellbeing in military veterans with PTSD. Although clinical significance cannot be confirmed, a higher CAR/AUCi among those with a service dog may indicate better health and wellbeing in this population. Future within-subject, longitudinal research will be necessary to determine potential clinical significance and impact of individual differences. |
  | Date | 2018-12 |
  | Language | eng |
  | Library Catalogue | PubMed |
  | Volume | 98 |
  | Pages | 202-210 |
  | Publication | Psychoneuroendocrinology |
  | DOI | 10.1016/j.psyneuen.2018.04.026 |
  | Journal Abbr | Psychoneuroendocrinology |
  | ISSN | 1873-3360 |
  | PMID | 29907299 |
  | PMCID | PMC8454180 |
  | Date Added | 11/07/2025, 14:21:52 |
  | Modified | 11/07/2025, 14:21:52 |

  ### Tags:

  - Hydrocortisone
  - Dogs
  - PTSD
  - Animals
  - Female
  - Adult
  - Humans
  - Male
  - Middle Aged
  - Animal Assisted Therapy
  - Stress, Psychological
  - Human-animal interaction
  - Surveys and Questionnaires
  - Saliva
  - Cortisol awakening response
  - Military Personnel
  - Military veterans
  - Posttraumatic stress disorder
  - Service dogs
  - Stress Disorders, Post-Traumatic
  - Veterans

  ### Attachments

  - Accepted Version
  - PubMed entry
- ## Mechanisms of Social Attachment Between Children and Pet Dogs

  |  |  |
  | --- | --- |
  | Item Type | Journal Article |
  | Author | Olivia T. Reilly |
  | Author | Leah H. Somerville |
  | Author | Erin E. Hecht |
  | Abstract | An increasing body of evidence indicates that owning a pet dog is associated with improvements in child health and well-being. Importantly, the degree of the social bond between child and dog may mediate the beneficial outcomes of dog ownership. The formation of social bonds is an intrinsically dyadic, interactive process where each interactor's behavior influences the other's behavior. For this reason, it is critical to evaluate the biological mechanisms of attachment in both children and their pet dogs as a socially bonded pair. Here, we review the physical, mental, and emotional outcomes that are associated with pet dog ownership or interaction in children. We then discuss the evidence that suggests that the strength of a social bond between a child and their pet dog matters for maximizing the beneficial outcomes associated with pet dog ownership, such as possible stress-buffering effects. We review the existing literature on the neural and endocrinological mechanisms of social attachment for inter-species social bonds that form between human children and dogs, situating this emerging knowledge within the context of the mechanisms of intra-species bonds in mammals. Finally, we highlight the remaining open questions and point toward directions for future research. |
  | Date | 2024-10-20 |
  | Language | eng |
  | Library Catalogue | PubMed |
  | Volume | 14 |
  | Pages | 3036 |
  | Publication | Animals: an open access journal from MDPI |
  | DOI | 10.3390/ani14203036 |
  | Issue | 20 |
  | Journal Abbr | Animals (Basel) |
  | ISSN | 2076-2615 |
  | PMID | 39457966 |
  | PMCID | PMC11505475 |
  | Date Added | 11/07/2025, 11:45:53 |
  | Modified | 11/07/2025, 11:45:58 |

  ### Tags:

  - cortisol
  - oxytocin
  - dogs
  - human-animal interaction
  - children
  - social attachment

  ### Attachments

  - Full Text PDF
  - PubMed entry
- ## Mechanisms of Social Attachment Between Children and Pet Dogs

  |  |  |
  | --- | --- |
  | Item Type | Journal Article |
  | Author | Olivia T. Reilly |
  | Author | Leah H. Somerville |
  | Author | Erin E. Hecht |
  | Abstract | An increasing body of evidence indicates that owning a pet dog is associated with improvements in child health and well-being. Importantly, the degree of the social bond between child and dog may mediate the beneficial outcomes of dog ownership. The formation of social bonds is an intrinsically dyadic, interactive process where each interactor's behavior influences the other's behavior. For this reason, it is critical to evaluate the biological mechanisms of attachment in both children and their pet dogs as a socially bonded pair. Here, we review the physical, mental, and emotional outcomes that are associated with pet dog ownership or interaction in children. We then discuss the evidence that suggests that the strength of a social bond between a child and their pet dog matters for maximizing the beneficial outcomes associated with pet dog ownership, such as possible stress-buffering effects. We review the existing literature on the neural and endocrinological mechanisms of social attachment for inter-species social bonds that form between human children and dogs, situating this emerging knowledge within the context of the mechanisms of intra-species bonds in mammals. Finally, we highlight the remaining open questions and point toward directions for future research. |
  | Date | 2024-10-20 |
  | Language | eng |
  | Library Catalogue | PubMed |
  | Volume | 14 |
  | Pages | 3036 |
  | Publication | Animals: an open access journal from MDPI |
  | DOI | 10.3390/ani14203036 |
  | Issue | 20 |
  | Journal Abbr | Animals (Basel) |
  | ISSN | 2076-2615 |
  | PMID | 39457966 |
  | PMCID | PMC11505475 |
  | Date Added | 11/07/2025, 14:21:52 |
  | Modified | 11/07/2025, 14:21:52 |

  ### Tags:

  - cortisol
  - oxytocin
  - dogs
  - human-animal interaction
  - children
  - social attachment

  ### Attachments

  - Full Text PDF
  - PubMed entry
- ## Gentle abdominal stroking ('belly rubbing') of pigs by a human reduces EEG total power and increases EEG frequencies

  |  |  |
  | --- | --- |
  | Item Type | Journal Article |
  | Author | Jean-Loup Rault |
  | Author | Suzanne Truong |
  | Author | Lauren Hemsworth |
  | Author | Matthias Le Chevoir |
  | Author | Sebastien Bauquier |
  | Author | Alan Lai |
  | Abstract | The neurobiological response to gentle touch remains poorly understood, especially in the context of human-animal interaction. A novel approach allowed recording the pig electroencephalogram (EEG) cranially epidurally and wirelessly during positive interactions with a human. Stroking of the pig's abdomen ('belly rubbing'), applied opportunistically, elicited a distinct behavioral response characterized by lateral recumbency, limb stretching, frequent short-lasting grunts and eye closure. Pigs varied in their responsiveness to belly rubbing but all pigs showed it. Their EEG was compared to EEG during human presence and other positive interactions except belly rubbing; isolation; and in the home pen as a baseline. Total EEG power ('Ptot') was lower during belly rubbing, whereas the median frequency ('F50', 5.3 ± 0.9 Hz vs. 3.8 ± 0.9 Hz for other contexts) and the 95% spectral edge frequency ('F95', 45.2 ± 3.2 Hz vs. 40.0 ± 3.2 Hz for other contexts) were higher during belly rubbing compared to other contexts. Lower EEG total power combined with a shift in spectral power distribution toward higher frequencies were linked to behavioral changes indicative of a positive welfare state during belly rubbing. The effects of belly rubbing on animal psychobiology and well-being warrant further research as a model of positive welfare state induced by touch. |
  | Date | 2019-11-18 |
  | Language | eng |
  | Library Catalogue | PubMed |
  | Volume | 374 |
  | Pages | 111892 |
  | Publication | Behavioural Brain Research |
  | DOI | 10.1016/j.bbr.2019.04.006 |
  | Journal Abbr | Behav Brain Res |
  | ISSN | 1872-7549 |
  | PMID | 30959126 |
  | Date Added | 11/07/2025, 11:33:54 |
  | Modified | 11/07/2025, 11:33:54 |

  ### Tags:

  - Electroencephalography
  - Relaxation
  - Electrocorticogram
  - Human animal
  - Massage
  - Positive welfare
  - Touch
  - Abdomen
  - Animals
  - Behavior, Animal
  - Brain
  - Female
  - Swine

  ### Attachments

  - PubMed entry
- ## Oxytocin as an Indicator of Psychological and Social Well-Being in Domesticated Animals: A Critical Review

  |  |  |
  | --- | --- |
  | Item Type | Journal Article |
  | Author | Jean-Loup Rault |
  | Author | Marleen van den Munkhof |
  | Author | Femke T. A. Buisman-Pijlman |
  | Abstract | Oxytocin is often portrayed as a hormone specific to social behavior, reflective of positive welfare states, and linked to mental states. Research on oxytocin in domesticated animal species has been few to date but is rapidly increasing (in dog, pig, cattle, sheep), with direct implications for animal welfare. This review evaluates the evidence for the specificity of oxytocin as an indicator of: 1. Social, 2. Positive, and 3. Psychological well-being. Oxytocin has most often been studied in socially relevant paradigms, with a lack of non-social control paradigms. Oxytocin research appears biased toward investigating positive valence, with a lack of control in valence or arousal. Oxytocin actions are modulated by the environmental and social contexts, which are important factors to consider. Limited evidence supports that oxytocin's actions are linked to psychological states; nevertheless whether this is a direct effect of oxytocin per se remains to be demonstrated. Overall, it is premature to judge oxytocin's potential as an animal welfare indicator given the few and discrepant findings and a lack of standardization in methodology. We cover potential causes for discrepancies and suggest solutions through appropriate methodological design, oxytocin sampling or delivery, analysis and reporting. Of particular interest, the oxytocinergic system as a whole remains poorly understood. Appreciation for the differences that social contact and group living pose in domesticated species and the way they interact with humans should be key considerations in using oxytocin as a psychosocial indicator of well-being. |
  | Date | 2017 |
  | Language | eng |
  | Short Title | Oxytocin as an Indicator of Psychological and Social Well-Being in Domesticated Animals |
  | Library Catalogue | PubMed |
  | Volume | 8 |
  | Pages | 1521 |
  | Publication | Frontiers in Psychology |
  | DOI | 10.3389/fpsyg.2017.01521 |
  | Journal Abbr | Front Psychol |
  | ISSN | 1664-1078 |
  | PMID | 28955264 |
  | PMCID | PMC5601408 |
  | Date Added | 11/07/2025, 11:45:53 |
  | Modified | 11/07/2025, 11:45:57 |

  ### Tags:

  - oxytocin
  - human-animal interaction
  - animal welfare
  - affiliation
  - emotion
  - intranasal administration
  - positive
  - social behavior

  ### Attachments

  - Full Text
  - PubMed entry
- ## Ground-based adaptive horsemanship lessons for veterans with post-traumatic stress disorder: a randomized controlled pilot study

  |  |  |
  | --- | --- |
  | Item Type | Journal Article |
  | Author | Ellen M. Rankins |
  | Author | Andrea Quinn |
  | Author | Kenneth H. McKeever |
  | Author | Karyn Malinowski |
  | Abstract | INTRODUCTION: Equine-assisted services (EAS) has received attention as a potential treatment strategy for post-traumatic stress disorder (PTSD), as existing literature indicates that symptoms may decrease following EAS. Relatively little is known about the mechanisms at play during lessons and if physiological measures are impacted. The objectives of this pilot study were to 1) explore the effects of adaptive horsemanship (AH) lessons on symptoms of PTSD, hormone concentrations, and social motor synchrony; 2) determine if physiological changes occur as veterans interact with horses; and 3) explore if the interaction between veteran and horse changes over the 8-week session. METHODS: Veterans with PTSD were randomly assigned to control (CON, n = 3) or AH (n = 6) groups for an 8-week period (clinical trial; NCT04850573; clinicaltrials.gov). Veterans completed the PTSD Checklist (PCL-5) and Brief Symptom Inventory (BSI) at pre-, post-, and 2- and 6-month follow-up time points. They also completed a social motor synchrony test (pendulum swinging) and blood draw at pre- and post-time points. In weeks 1, 4, and 8, blood samples were drawn at 0 min, 3 min, 5 min, 25 min, and 30 min during the 30-min AH lessons. Veterans completed the Human-Animal Interaction Scale (HAIS) after each lesson. Blood samples were assayed for plasma cortisol, epinephrine, norepinephrine, and oxytocin. Data were analyzed with repeated measure ANOVAs. Changes in PTSD symptoms from pre- to post-time point were analyzed with paired t-tests. RESULTS: Changes in PCL-5 scores tended to differ (p = 0.0989), and global BSI scores differed (p = 0.0266) between AH (-11.5 ± 5.5, mean ± SE; -0.5 ± 0.2) and CON (5.3 ± 5.4; 0.4 ± 0.2) groups. Social motor synchrony and hormone concentrations did not differ between groups or time points (p > 0.05). Cortisol, norepinephrine, and oxytocin concentrations did not differ across sessions (p > 0.05). Epinephrine concentrations tended (p = 0.0744) to decrease from week 1 to 4 of sessions. HAIS scores increased (p ≥ 0.0437) in week 3 and remained elevated as compared to week 1. DISCUSSION: Participant recruitment was the greatest challenge. These preliminary results agree with the literature suggesting that EAS can reduce symptoms of PTSD. |
  | Date | 2024 |
  | Language | eng |
  | Short Title | Ground-based adaptive horsemanship lessons for veterans with post-traumatic stress disorder |
  | Library Catalogue | PubMed |
  | Volume | 15 |
  | Pages | 1390212 |
  | Publication | Frontiers in Psychiatry |
  | DOI | 10.3389/fpsyt.2024.1390212 |
  | Journal Abbr | Front Psychiatry |
  | ISSN | 1664-0640 |
  | PMID | 38863605 |
  | PMCID | PMC11165701 |
  | Date Added | 11/07/2025, 11:33:21 |
  | Modified | 11/07/2025, 11:33:21 |

  ### Tags:

  - cortisol
  - oxytocin
  - equine-assisted services
  - horse
  - nervous system
  - PTSD
  - trauma
  - veteran

  ### Attachments

  - Full Text
  - PubMed entry
- ## Ground-based adaptive horsemanship lessons for veterans with post-traumatic stress disorder: a randomized controlled pilot study

  |  |  |
  | --- | --- |
  | Item Type | Journal Article |
  | Author | Ellen M. Rankins |
  | Author | Andrea Quinn |
  | Author | Kenneth H. McKeever |
  | Author | Karyn Malinowski |
  | Abstract | INTRODUCTION: Equine-assisted services (EAS) has received attention as a potential treatment strategy for post-traumatic stress disorder (PTSD), as existing literature indicates that symptoms may decrease following EAS. Relatively little is known about the mechanisms at play during lessons and if physiological measures are impacted. The objectives of this pilot study were to 1) explore the effects of adaptive horsemanship (AH) lessons on symptoms of PTSD, hormone concentrations, and social motor synchrony; 2) determine if physiological changes occur as veterans interact with horses; and 3) explore if the interaction between veteran and horse changes over the 8-week session. METHODS: Veterans with PTSD were randomly assigned to control (CON, n = 3) or AH (n = 6) groups for an 8-week period (clinical trial; NCT04850573; clinicaltrials.gov). Veterans completed the PTSD Checklist (PCL-5) and Brief Symptom Inventory (BSI) at pre-, post-, and 2- and 6-month follow-up time points. They also completed a social motor synchrony test (pendulum swinging) and blood draw at pre- and post-time points. In weeks 1, 4, and 8, blood samples were drawn at 0 min, 3 min, 5 min, 25 min, and 30 min during the 30-min AH lessons. Veterans completed the Human-Animal Interaction Scale (HAIS) after each lesson. Blood samples were assayed for plasma cortisol, epinephrine, norepinephrine, and oxytocin. Data were analyzed with repeated measure ANOVAs. Changes in PTSD symptoms from pre- to post-time point were analyzed with paired t-tests. RESULTS: Changes in PCL-5 scores tended to differ (p = 0.0989), and global BSI scores differed (p = 0.0266) between AH (-11.5 ± 5.5, mean ± SE; -0.5 ± 0.2) and CON (5.3 ± 5.4; 0.4 ± 0.2) groups. Social motor synchrony and hormone concentrations did not differ between groups or time points (p > 0.05). Cortisol, norepinephrine, and oxytocin concentrations did not differ across sessions (p > 0.05). Epinephrine concentrations tended (p = 0.0744) to decrease from week 1 to 4 of sessions. HAIS scores increased (p ≥ 0.0437) in week 3 and remained elevated as compared to week 1. DISCUSSION: Participant recruitment was the greatest challenge. These preliminary results agree with the literature suggesting that EAS can reduce symptoms of PTSD. |
  | Date | 2024 |
  | Language | eng |
  | Short Title | Ground-based adaptive horsemanship lessons for veterans with post-traumatic stress disorder |
  | Library Catalogue | PubMed |
  | Volume | 15 |
  | Pages | 1390212 |
  | Publication | Frontiers in Psychiatry |
  | DOI | 10.3389/fpsyt.2024.1390212 |
  | Journal Abbr | Front Psychiatry |
  | ISSN | 1664-0640 |
  | PMID | 38863605 |
  | PMCID | PMC11165701 |
  | Date Added | 11/07/2025, 11:45:53 |
  | Modified | 11/07/2025, 11:45:59 |

  ### Tags:

  - cortisol
  - oxytocin
  - equine-assisted services
  - horse
  - nervous system
  - PTSD
  - trauma
  - veteran

  ### Attachments

  - Full Text
  - PubMed entry
- ## Ground-based adaptive horsemanship lessons for veterans with post-traumatic stress disorder: a randomized controlled pilot study

  |  |  |
  | --- | --- |
  | Item Type | Journal Article |
  | Author | Ellen M. Rankins |
  | Author | Andrea Quinn |
  | Author | Kenneth H. McKeever |
  | Author | Karyn Malinowski |
  | Abstract | INTRODUCTION: Equine-assisted services (EAS) has received attention as a potential treatment strategy for post-traumatic stress disorder (PTSD), as existing literature indicates that symptoms may decrease following EAS. Relatively little is known about the mechanisms at play during lessons and if physiological measures are impacted. The objectives of this pilot study were to 1) explore the effects of adaptive horsemanship (AH) lessons on symptoms of PTSD, hormone concentrations, and social motor synchrony; 2) determine if physiological changes occur as veterans interact with horses; and 3) explore if the interaction between veteran and horse changes over the 8-week session. METHODS: Veterans with PTSD were randomly assigned to control (CON, n = 3) or AH (n = 6) groups for an 8-week period (clinical trial; NCT04850573; clinicaltrials.gov). Veterans completed the PTSD Checklist (PCL-5) and Brief Symptom Inventory (BSI) at pre-, post-, and 2- and 6-month follow-up time points. They also completed a social motor synchrony test (pendulum swinging) and blood draw at pre- and post-time points. In weeks 1, 4, and 8, blood samples were drawn at 0 min, 3 min, 5 min, 25 min, and 30 min during the 30-min AH lessons. Veterans completed the Human-Animal Interaction Scale (HAIS) after each lesson. Blood samples were assayed for plasma cortisol, epinephrine, norepinephrine, and oxytocin. Data were analyzed with repeated measure ANOVAs. Changes in PTSD symptoms from pre- to post-time point were analyzed with paired t-tests. RESULTS: Changes in PCL-5 scores tended to differ (p = 0.0989), and global BSI scores differed (p = 0.0266) between AH (-11.5 ± 5.5, mean ± SE; -0.5 ± 0.2) and CON (5.3 ± 5.4; 0.4 ± 0.2) groups. Social motor synchrony and hormone concentrations did not differ between groups or time points (p > 0.05). Cortisol, norepinephrine, and oxytocin concentrations did not differ across sessions (p > 0.05). Epinephrine concentrations tended (p = 0.0744) to decrease from week 1 to 4 of sessions. HAIS scores increased (p ≥ 0.0437) in week 3 and remained elevated as compared to week 1. DISCUSSION: Participant recruitment was the greatest challenge. These preliminary results agree with the literature suggesting that EAS can reduce symptoms of PTSD. |
  | Date | 2024 |
  | Language | eng |
  | Short Title | Ground-based adaptive horsemanship lessons for veterans with post-traumatic stress disorder |
  | Library Catalogue | PubMed |
  | Volume | 15 |
  | Pages | 1390212 |
  | Publication | Frontiers in Psychiatry |
  | DOI | 10.3389/fpsyt.2024.1390212 |
  | Journal Abbr | Front Psychiatry |
  | ISSN | 1664-0640 |
  | PMID | 38863605 |
  | PMCID | PMC11165701 |
  | Date Added | 11/07/2025, 14:21:52 |
  | Modified | 11/07/2025, 14:21:52 |

  ### Tags:

  - cortisol
  - oxytocin
  - equine-assisted services
  - horse
  - nervous system
  - PTSD
  - trauma
  - veteran

  ### Attachments

  - Full Text
  - PubMed entry
- ## Does dog acquisition improve physical activity, sedentary behaviour and biological markers of cardiometabolic health? Results from a three-arm controlled study

  |  |  |
  | --- | --- |
  | Item Type | Journal Article |
  | Author | Lauren Powell |
  | Author | Kate M. Edwards |
  | Author | Adrian Bauman |
  | Author | Paul McGreevy |
  | Author | Anthony Podberscek |
  | Author | Brendon Neilly |
  | Author | Catherine Sherrington |
  | Author | Emmanuel Stamatakis |
  | Abstract | OBJECTIVES: Dog ownership has been associated with improved cardiometabolic risk factors, including physical activity. Most of the evidence originates from cross-sectional studies or populations with established disease. This study investigated changes in physical activity and other cardiometabolic risk factors following dog acquisition in a sample of 71 community-dwelling adults. METHODS: Participants self-allocated to three groups: 17 individuals acquired a dog within 1 month of baseline (dog acquisition), 29 delayed dog acquisition until study completion (lagged control) and 25 had no interest in dog acquisition (community control). Self-reported and thigh-worn accelerometer-based physical activity patterns, systolic and diastolic blood pressures, resting heart rate and VO2max were measured three times: baseline, 3 months and 8 months. Data were analysed using repeated measures analysis of covariance with owner age, season, sex and education included as covariates. Post hoc between-group tests were performed where there were significant overall effects (p<0.05). RESULTS: We found significant effects in mean daily steps (F(4,64)=3.02, p=0.02) and sit-to-stand transitions (F(4,66)=3.49, p=0.01). The dog acquisition group performed an additional 2589 steps (p=0.004) and 8.2 sit-to-stand transitions (p=0.03) per day at 3 months, although these effects were not maintained at 8 months. We found a significant effect in self-reported weekly walking duration (F(4,130)=2.84, p=0.03) among the lagged control group with an 80 min increase between 3 and 8 months (p=0.04). Other cardiometabolic risk factors were unchanged following dog acquisition. CONCLUSION: Our study provides encouraging results that suggest a positive influence of dog acquisition on physical activity in the short term but larger and more generalisable controlled studies are needed. TRIAL REGISTRATION NUMBER: ACTRN12617000967381. |
  | Date | 2020 |
  | Language | eng |
  | Short Title | Does dog acquisition improve physical activity, sedentary behaviour and biological markers of cardiometabolic health? |
  | Library Catalogue | PubMed |
  | Volume | 6 |
  | Pages | e000703 |
  | Publication | BMJ open sport & exercise medicine |
  | DOI | 10.1136/bmjsem-2019-000703 |
  | Issue | 1 |
  | Journal Abbr | BMJ Open Sport Exerc Med |
  | ISSN | 2055-7647 |
  | PMID | 32518673 |
  | PMCID | PMC7254141 |
  | Date Added | 11/07/2025, 11:37:24 |
  | Modified | 11/07/2025, 11:37:24 |

  ### Tags:

  - accelerometer
  - aerobic fitness
  - cardiovascular
  - physical activity
  - sedentary

  ### Attachments

  - Full Text
  - PubMed entry
- ## Serum Oxytocin, Cortisol and Social Behavior in Calves: A Study in the Impossible Task Paradigm

  |  |  |
  | --- | --- |
  | Item Type | Journal Article |
  | Author | Claudia Pinelli |
  | Author | Anna Scandurra |
  | Author | Vincenzo Mastellone |
  | Author | Piera Iommelli |
  | Author | Nadia Musco |
  | Author | Maria Elena Pero |
  | Author | Alfredo Di Lucrezia |
  | Author | Daria Lotito |
  | Author | Raffaella Tudisco |
  | Author | Biagio D'Aniello |
  | Author | Federico Infascelli |
  | Author | Pietro Lombardi |
  | Abstract | In this study, we explored the correlations between circulating levels of oxytocin, cortisol, and different social behaviors toward humans in 26 Italian Red Pied calves (all females, with an average age of 174 ± 24 days) using the impossible task paradigm. This paradigm has proved fruitful in highlighting the effect of socialization on the willingness to interact with humans in several domesticated species. The test consists of the violation of an expectation (recovering food from an experimental apparatus) while a caregiver and a stranger are present. Immediately after the end of the test (less than one minute), blood was collected from the coccygeal vein. Statistics were performed by the Spearman's rank correlation; significant differences were adjusted according to Bonferroni's correction. Cortisol correlates positively (ρ = 0.565; p < 0.05) with the latency of behaviors directed at the caregiver, and the duration of behaviors directed at the apparatus correlates negatively with both the caregiver (ρ = -0.654; p < 0.05) and a stranger (ρ = -0.644; p < 0.05). Contrary to what is reported in the literature on cows, no correlations were found between oxytocin levels and direct behaviors toward the caregiver. This highlights a different behavioral strategy between calves and cows when placed in front of an impossible task. |
  | Date | 2023-02-13 |
  | Language | eng |
  | Short Title | Serum Oxytocin, Cortisol and Social Behavior in Calves |
  | Library Catalogue | PubMed |
  | Volume | 13 |
  | Pages | 646 |
  | Publication | Animals: an open access journal from MDPI |
  | DOI | 10.3390/ani13040646 |
  | Issue | 4 |
  | Journal Abbr | Animals (Basel) |
  | ISSN | 2076-2615 |
  | PMID | 36830435 |
  | PMCID | PMC9951757 |
  | Date Added | 11/07/2025, 11:45:53 |
  | Modified | 11/07/2025, 11:45:58 |

  ### Tags:

  - cortisol
  - oxytocin
  - human–animal interaction
  - behavior
  - calves
  - dairy cows
  - impossible task

  ### Attachments

  - Full Text
  - PubMed entry
- ## Serum Oxytocin, Cortisol and Social Behavior in Calves: A Study in the Impossible Task Paradigm

  |  |  |
  | --- | --- |
  | Item Type | Journal Article |
  | Author | Claudia Pinelli |
  | Author | Anna Scandurra |
  | Author | Vincenzo Mastellone |
  | Author | Piera Iommelli |
  | Author | Nadia Musco |
  | Author | Maria Elena Pero |
  | Author | Alfredo Di Lucrezia |
  | Author | Daria Lotito |
  | Author | Raffaella Tudisco |
  | Author | Biagio D'Aniello |
  | Author | Federico Infascelli |
  | Author | Pietro Lombardi |
  | Abstract | In this study, we explored the correlations between circulating levels of oxytocin, cortisol, and different social behaviors toward humans in 26 Italian Red Pied calves (all females, with an average age of 174 ± 24 days) using the impossible task paradigm. This paradigm has proved fruitful in highlighting the effect of socialization on the willingness to interact with humans in several domesticated species. The test consists of the violation of an expectation (recovering food from an experimental apparatus) while a caregiver and a stranger are present. Immediately after the end of the test (less than one minute), blood was collected from the coccygeal vein. Statistics were performed by the Spearman's rank correlation; significant differences were adjusted according to Bonferroni's correction. Cortisol correlates positively (ρ = 0.565; p < 0.05) with the latency of behaviors directed at the caregiver, and the duration of behaviors directed at the apparatus correlates negatively with both the caregiver (ρ = -0.654; p < 0.05) and a stranger (ρ = -0.644; p < 0.05). Contrary to what is reported in the literature on cows, no correlations were found between oxytocin levels and direct behaviors toward the caregiver. This highlights a different behavioral strategy between calves and cows when placed in front of an impossible task. |
  | Date | 2023-02-13 |
  | Language | eng |
  | Short Title | Serum Oxytocin, Cortisol and Social Behavior in Calves |
  | Library Catalogue | PubMed |
  | Volume | 13 |
  | Pages | 646 |
  | Publication | Animals: an open access journal from MDPI |
  | DOI | 10.3390/ani13040646 |
  | Issue | 4 |
  | Journal Abbr | Animals (Basel) |
  | ISSN | 2076-2615 |
  | PMID | 36830435 |
  | PMCID | PMC9951757 |
  | Date Added | 11/07/2025, 14:21:52 |
  | Modified | 11/07/2025, 14:21:52 |

  ### Tags:

  - cortisol
  - oxytocin
  - human–animal interaction
  - behavior
  - calves
  - dairy cows
  - impossible task

  ### Attachments

  - Full Text
  - PubMed entry
- ## Dog-Owner Relationship and Its Association with Social Cognition in French Bulldogs

  |  |  |
  | --- | --- |
  | Item Type | Journal Article |
  | Author | Lara Peterca |
  | Author | Elena Gobbo |
  | Author | Manja Zupan Šemrov |
  | Abstract | Our understanding of social cognition in brachycephalic dog breeds is limited. This study focused specifically on French Bulldogs and hypothesized that a closer relationship between dog and owner would improve the dogs' understanding of nonverbal cues, particularly pointing gestures. To investigate this, we tested twenty-six dogs and their owners in a two-way object choice test in which the familiar person pointed to the bowl. As understanding of pointing gestures is influenced by human-animal interaction, we used the Monash Dog Owner Relationship Scale (MDORS) to assess various aspects of the dog-owner relationship, including dog-owner interaction, emotional closeness, and negative aspects of dog ownership and measured salivary oxytocin levels. This study examined the dogs' correct choices and their straight approach patterns to a reward bowl. Several factors influenced the two measures of social cognition, such as the age and sex of the dog, dog-owner interaction, emotional closeness, and the choice time (time it took the dog to reach the reward bowl), but also that neutered dogs and those with no training history showed better cognitive performance. We found a very high number of correct choices, which could be attributed to the owners' high scores on the dog-owner interaction scale. Oxytocin showed no direct effect on these measures. To increase the generalizability of the results, we suggest future studies focus on more than one breed to capture the temporal development of the owner-dog relationship and social cognitive abilities. |
  | Date | 2024-12-25 |
  | Language | eng |
  | Library Catalogue | PubMed |
  | Volume | 15 |
  | Pages | 17 |
  | Publication | Animals: an open access journal from MDPI |
  | DOI | 10.3390/ani15010017 |
  | Issue | 1 |
  | Journal Abbr | Animals (Basel) |
  | ISSN | 2076-2615 |
  | PMID | 39794959 |
  | PMCID | PMC11718994 |
  | Date Added | 11/07/2025, 11:45:53 |
  | Modified | 11/07/2025, 11:45:58 |

  ### Tags:

  - brachycephalic dogs
  - cognition
  - French Bulldogs
  - MDORS
  - pointing gestures
  - two-way object choice test

  ### Attachments

  - Full Text PDF
  - PubMed entry
- ## Limited Short-Term Effects of Tactile Stimulation on the Welfare of Newborn Nellore Calves

  |  |  |
  | --- | --- |
  | Item Type | Journal Article |
  | Author | Mariana Parra Cerezo |
  | Author | Victor Brusin |
  | Author | Pedro Henrique Esteves Trindade |
  | Author | Adalinda Hernández |
  | Author | Jens Jung |
  | Author | Charlotte Berg |
  | Author | Mateus José Rodrigues Paranhos da Costa |
  | Abstract | This study aimed to evaluate the effects of tactile stimulation on calf welfare. A total of 54 Nellore calves were assessed, with 28 of them receiving tactile stimulation (WTS) for ~60 s and 26 serving as a control. Five body movements and seven facial expressions were scored. Heart rates (HRs) were recorded in three situations: when the calves were placed in lateral recumbency (HR1), during identification procedures (HR2), and after completion of identification procedures (HR3). The differences between HR3 and HR1, as well as HR3 and HR2 were calculated. Initial and weaning weights were recorded, and ADG and weaning weights adjusted to 240 days were determined. Tactile stimulation significantly influenced "head movements", "third eyelid" exposure, "eye-opening", and "strained nostrils". Except for "strained nostrils", WTS calves exhibited higher scores in these behavioral categories. Treatment also influenced the difference between HR3 and HR2 (p < 0.05) and showed a trend for HR3 and the difference between HR3 and HR1 (p < 0.06). A qualitative behavior assessment (QBA) was applied using facial expressions. Two main principal components were identified, PC1 explaining 63.01% of the data variance and reflecting the calves' emotionality, and PC2 explaining 19.88% and reflecting excitability. Most WTS calves displayed positive emotional states and high excitability, whereas most NTS calves exhibited the opposite. Treatment did not significantly impact PC1 and PC2 indexes and long-term performance indicators (p > 0.05). We conclude that tactile stimulation of newborn Nellore calves during their initial handling has the potential to enhance their short-term welfare, but only to a limited extent. |
  | Date | 2025-04-21 |
  | Language | eng |
  | Library Catalogue | PubMed |
  | Volume | 12 |
  | Pages | 393 |
  | Publication | Veterinary Sciences |
  | DOI | 10.3390/vetsci12040393 |
  | Issue | 4 |
  | Journal Abbr | Vet Sci |
  | ISSN | 2306-7381 |
  | PMID | 40284895 |
  | PMCID | PMC12031576 |
  | Date Added | 11/07/2025, 11:37:24 |
  | Modified | 11/07/2025, 11:37:24 |

  ### Tags:

  - human–animal interaction
  - average daily gain
  - cow–calf operation
  - heart rate
  - qualitative behavior assessment

  ### Attachments

  - Full Text PDF
  - PubMed entry
- ## Replication Pilot Trial of Therapeutic Horseback Riding and Cortisol Collection With Children on the Autism Spectrum

  |  |  |
  | --- | --- |
  | Item Type | Journal Article |
  | Author | Zhaoxing Pan |
  | Author | Douglas A. Granger |
  | Author | Noémie A. Guérin |
  | Author | Amy Shoffner |
  | Author | Robin L. Gabriels |
  | Abstract | We aimed to determine whether results of our prior randomized control trial [RCT; NCT02301195, (1)] of Therapeutic Horseback Riding (THR) for children and adolescents with autism spectrum disorder (ASD) could be replicated at a different riding center and if treatment effects also included differences in the expression of associations between problem behavior and the activity of the hypothalamic-pituitary-adrenal (HPA) axis. Participants with ASD (N = 16) ages 6-16 years were randomized by nonverbal intelligence quotient to either a 10-week THR group (n = 8) or no horse interaction barn activity (BA) control group (n = 8). Outcome measures were a standard speech-language sample and caregiver-report of aberrant and social behaviors. Participants' saliva was sampled weekly at a consistent afternoon time immediately pre- and 20 min' post-condition (later assayed for cortisol). Intent-to-treat analysis revealed that compared to controls, THR participants had significant improvements in hyperactivity, and social awareness, and significant improvements at the 0.1 significance level in irritability and social communication behaviors. There were no significant improvements in number of words or new words spoken during the standard language sample. Linear mixed effects model analysis indicated that greater weekly pre-lesson irritability levels were associated with smaller post-lesson reduction in salivary cortisol levels, and greater weekly pre-lesson hyperactivity levels were associated with smaller cortisol reduction in the THR group, but not in the BA control group. The findings represent a partial replication of prior results (1), extend prior observations to include THR effects on biobehavioral relationships and suggest that cortisol could be a target mediator for THR effects on irritability and hyperactivity behaviors in youth with ASD. Clinical Trial Registration: Trial of Therapeutic Horseback Riding in Children and Adolescents with Autism Spectrum Disorder; http://clinicaltrials.gov, identifier: NCT02301195. |
  | Date | 2018 |
  | Language | eng |
  | Library Catalogue | PubMed |
  | Volume | 5 |
  | Pages | 312 |
  | Publication | Frontiers in Veterinary Science |
  | DOI | 10.3389/fvets.2018.00312 |
  | Journal Abbr | Front Vet Sci |
  | ISSN | 2297-1769 |
  | PMID | 30693284 |
  | PMCID | PMC6339889 |
  | Date Added | 11/07/2025, 14:21:52 |
  | Modified | 11/07/2025, 14:21:52 |

  ### Tags:

  - human-animal interaction
  - therapeutic horseback riding
  - autism spectrum disorder
  - equine-assisted activities and therapies
  - salivary cortisol

  ### Attachments

  - Full Text
  - PubMed entry
- ## Effects of Stroking on Salivary Oxytocin and Cortisol in Guide Dogs: Preliminary Results

  |  |  |
  | --- | --- |
  | Item Type | Journal Article |
  | Author | Asahi Ogi |
  | Author | Chiara Mariti |
  | Author | Paolo Baragli |
  | Author | Valeria Sergi |
  | Author | Angelo Gazzano |
  | Abstract | This pilot study aimed at investigating how salivary oxytocin levels are affected by human interaction and isolation in eight guide dogs (six Labrador retrievers and two golden retrievers; four males and four females, 21.87 ± 1.36 months old) just before assignment to the blind person. Each dog engaged, at one-week intervals, in a positive (5 min of affiliative interaction with their trainer) and a negative (5 min of isolation) condition. Saliva samples used for Enzyme Immunoassay (EIA) quantification of salivary oxytocin were collected before and immediately after both experimental conditions. In order to assess potential hypothalamic pituitary adrenal (HPA) axis activation that could have affected oxytocin levels, saliva samples were collected 15 min after both experimental conditions for EIA quantification of salivary cortisol and a behavioral assessment was performed during the negative condition. The results were compared using the Wilcoxon test (p < 0.05). Oxytocin concentrations showed a statistically significant increase after the positive interaction (p = 0.036) and no difference after the negative one (p = 0.779). Moreover, no difference (p = 0.263) was found between the cortisol concentrations after each experimental condition and no signs of distress were observed during the isolation phase. These preliminary findings support the hypothesis that stroking dogs has positive effects on their emotional state independently of hypothalamic pituitary adrenal axis activation. |
  | Date | 2020-04-18 |
  | Language | eng |
  | Short Title | Effects of Stroking on Salivary Oxytocin and Cortisol in Guide Dogs |
  | Library Catalogue | PubMed |
  | Volume | 10 |
  | Pages | 708 |
  | Publication | Animals: an open access journal from MDPI |
  | DOI | 10.3390/ani10040708 |
  | Issue | 4 |
  | Journal Abbr | Animals (Basel) |
  | ISSN | 2076-2615 |
  | PMID | 32325673 |
  | PMCID | PMC7222818 |
  | Date Added | 11/07/2025, 11:45:53 |
  | Modified | 11/07/2025, 11:45:58 |

  ### Tags:

  - cortisol
  - oxytocin
  - human–animal interaction
  - dog
  - saliva
  - isolation

  ### Attachments

  - Full Text
  - PubMed entry
- ## Effects of Stroking on Salivary Oxytocin and Cortisol in Guide Dogs: Preliminary Results

  |  |  |
  | --- | --- |
  | Item Type | Journal Article |
  | Author | Asahi Ogi |
  | Author | Chiara Mariti |
  | Author | Paolo Baragli |
  | Author | Valeria Sergi |
  | Author | Angelo Gazzano |
  | Abstract | This pilot study aimed at investigating how salivary oxytocin levels are affected by human interaction and isolation in eight guide dogs (six Labrador retrievers and two golden retrievers; four males and four females, 21.87 ± 1.36 months old) just before assignment to the blind person. Each dog engaged, at one-week intervals, in a positive (5 min of affiliative interaction with their trainer) and a negative (5 min of isolation) condition. Saliva samples used for Enzyme Immunoassay (EIA) quantification of salivary oxytocin were collected before and immediately after both experimental conditions. In order to assess potential hypothalamic pituitary adrenal (HPA) axis activation that could have affected oxytocin levels, saliva samples were collected 15 min after both experimental conditions for EIA quantification of salivary cortisol and a behavioral assessment was performed during the negative condition. The results were compared using the Wilcoxon test (p < 0.05). Oxytocin concentrations showed a statistically significant increase after the positive interaction (p = 0.036) and no difference after the negative one (p = 0.779). Moreover, no difference (p = 0.263) was found between the cortisol concentrations after each experimental condition and no signs of distress were observed during the isolation phase. These preliminary findings support the hypothesis that stroking dogs has positive effects on their emotional state independently of hypothalamic pituitary adrenal axis activation. |
  | Date | 2020-04-18 |
  | Language | eng |
  | Short Title | Effects of Stroking on Salivary Oxytocin and Cortisol in Guide Dogs |
  | Library Catalogue | PubMed |
  | Volume | 10 |
  | Pages | 708 |
  | Publication | Animals: an open access journal from MDPI |
  | DOI | 10.3390/ani10040708 |
  | Issue | 4 |
  | Journal Abbr | Animals (Basel) |
  | ISSN | 2076-2615 |
  | PMID | 32325673 |
  | PMCID | PMC7222818 |
  | Date Added | 11/07/2025, 14:21:52 |
  | Modified | 11/07/2025, 14:21:52 |

  ### Tags:

  - cortisol
  - oxytocin
  - human–animal interaction
  - dog
  - saliva
  - isolation

  ### Attachments

  - Full Text
  - PubMed entry
- ## A shoulder to cry on: Heart rate variability and empathetic behavioral responses to crying and laughing in dogs

  |  |  |
  | --- | --- |
  | Item Type | Journal Article |
  | Author | Julia E. Meyers-Manor |
  | Author | Marijo L. Botten |
  | Abstract | Dogs have a reputation for empathy toward their owners, which is also supported by some research (e.g., Carballo et al., 2020; Sanford, Burt, & Meyers-Manor, 2018). Many dog owners anecdotally report that dogs comfort them by making visual and/or physical contact when they cry or help them when they are sick. These behaviours provide a good way to assess the capacity for empathy and its physiological correlates in dogs. This study is a replication and extension of Custance and Mayer (2012). We examined whether using laughing as an alternative stimulus to humming produced similar responses to crying. Dogs were tested in their homes while a stranger and owner pretended to cry, laugh, and while the owner and stranger were talking. During each counterbalanced condition, the dog was observed for person-oriented behaviours and simultaneously had their heart rate variability measured. Like Custance and Mayer, dogs showed more behaviours directed toward the person crying, whether the owner or the stranger, than during baseline or laughing conditions. We did not find an effect of laughing on person-oriented behaviours, suggesting that dogs respond to the crying uniquely and not as a novel stimulus. In the condition when the stranger was crying, dogs that showed higher stress responses, as indicated by lower heart rate variability, were most likely to show person-oriented behaviours toward the stranger. This suggests that dogs that experience more distress, through emotional contagion, are more likely to show person-oriented behaviours toward the distressed stranger, indicating a possible mechanism for empathy-like behaviours. (PsycInfo Database Record (c) 2020 APA, all rights reserved). |
  | Date | 2020-09 |
  | Language | eng |
  | Short Title | A shoulder to cry on |
  | Library Catalogue | PubMed |
  | Volume | 74 |
  | Pages | 235-243 |
  | Publication | Canadian Journal of Experimental Psychology = Revue Canadienne De Psychologie Experimentale |
  | DOI | 10.1037/cep0000225 |
  | Issue | 3 |
  | Journal Abbr | Can J Exp Psychol |
  | ISSN | 1878-7290 |
  | PMID | 33090854 |
  | Date Added | 11/07/2025, 11:37:24 |
  | Modified | 11/07/2025, 11:37:24 |

  ### Tags:

  - Dogs
  - Animals
  - Behavior, Animal
  - Female
  - Heart Rate
  - Human-Animal Interaction
  - Male
  - Crying
  - Emotional Regulation
  - Empathy
  - Laughter
  - Psychological Distress
  - Social Perception

  ### Attachments

  - PubMed entry
- ## Animal-Assisted Interventions With Dogs in Special Education-A Systematic Review

  |  |  |
  | --- | --- |
  | Item Type | Journal Article |
  | Author | Jana Meixner |
  | Author | Kurt Kotrschal |
  | Abstract | Dogs are becoming increasingly popular in pedagogical settings. Particularly children with special educational needs are believed to benefit from dog-assisted interventions. However, reliable evidence for supporting such claims is still scarce and reports on the effectiveness of this approach are often anecdotal. With our review we aim at evaluating the literature to answer the question, whether dog-assisted interventions in an educational setting can help children with special educational needs to improve and to develop their emotional, social and cognitive skills. Following the PRISMA Guidelines, the literature was systematically searched for experimental studies until February 2021. Eighteen studies were finally included, which varied greatly in type of intervention, outcomes measured, sample sizes, and scientific quality, which precluded a formal meta-analysis. Hence, we resorted to a narrative synthesis. Overall, the studies report mixed results in the different functional domains of stress reduction, motivation, social skills, cognitive abilities, reading abilities, social conduct, and mental wellbeing. No study reported any negative effects of the intervention. The most unequivocal evidence comes from studies on dogs' effects on physiological stress response in challenging situations and on motivation and adherence to instructions, reporting significantly lower levels of cortisol in both children and pedagogues in the presence of dogs, as well as increased motivation to learn and participate. Findings for other outcomes, academic or social, however, remain inconclusive. Data on long-term effects are lacking altogether. Still, this review indicates the potentials of dog-assisted interventions in special pedagogy, particularly towards supporting a calm and trustful social atmosphere. |
  | Date | 2022 |
  | Language | eng |
  | Library Catalogue | PubMed |
  | Volume | 13 |
  | Pages | 876290 |
  | Publication | Frontiers in Psychology |
  | DOI | 10.3389/fpsyg.2022.876290 |
  | Journal Abbr | Front Psychol |
  | ISSN | 1664-1078 |
  | PMID | 35712211 |
  | PMCID | PMC9197485 |
  | Date Added | 11/07/2025, 14:21:52 |
  | Modified | 11/07/2025, 14:21:52 |

  ### Tags:

  - dogs
  - human-animal interaction
  - animal-assisted interventions
  - canine (dog)
  - dog-assisted interventions
  - special education
  - special pedagogy

  ### Attachments

  - Full Text
  - PubMed entry
- ## Can dogs reduce stress levels in school children? effects of dog-assisted interventions on salivary cortisol in children with and without special educational needs using randomized controlled trials

  |  |  |
  | --- | --- |
  | Item Type | Journal Article |
  | Author | Kerstin Meints |
  | Author | Victoria L. Brelsford |
  | Author | Mirena Dimolareva |
  | Author | Laëtitia Maréchal |
  | Author | Kyla Pennington |
  | Author | Elise Rowan |
  | Author | Nancy R. Gee |
  | Abstract | Prolonged or excessive stress negatively affects learning, behavior and health across the lifespan. To alleviate adverse effects of stress in school children, stressors should be reduced, and support and effective interventions provided. Animal-assisted interventions (AAI) have shown beneficial effects on health and wellbeing, however, robust knowledge on stress mediation in children is lacking. Despite this, AAIs are increasingly employed in settings world-wide, including schools, to reduce stress and support learning and wellbeing. This study is the first randomized controlled trial to investigate dog-assisted interventions as a mediator of stress in school children with and without special educational needs (SEN) over the school term. Interventions were carried out individually and in small groups twice a week for 20 minutes over the course of 4 weeks. We compared physiological changes in salivary cortisol in a dog intervention group with a relaxation intervention group and a no treatment control group. We compared cortisol level means before and after the 4 weeks of interventions in all children as well as acute cortisol in mainstream school children. Dog interventions lead to significantly lower stress in children with and without special educational needs compared to their peers in relaxation or no treatment control groups. In neurotypical children, those in the dog interventions showed no baseline stress level increases over the school term. In addition, acute cortisol levels evidenced significant stress reduction following the interventions. In contrast, the no treatment control group showed significant rises in baseline cortisol levels from beginning to end of school term. Increases also occurred in the relaxation intervention group. Children with SEN showed significantly decreased cortisol levels after dog group interventions. No changes occurred in the relaxation or no treatment control groups. These findings provide crucial evidence that dog interventions can successfully attenuate stress levels in school children with important implications for AAI implementation, learning and wellbeing. |
  | Date | 2022 |
  | Language | eng |
  | Short Title | Can dogs reduce stress levels in school children? |
  | Library Catalogue | PubMed |
  | Volume | 17 |
  | Pages | e0269333 |
  | Publication | PloS One |
  | DOI | 10.1371/journal.pone.0269333 |
  | Issue | 6 |
  | Journal Abbr | PLoS One |
  | ISSN | 1932-6203 |
  | PMID | 35704561 |
  | PMCID | PMC9200172 |
  | Date Added | 11/07/2025, 14:21:52 |
  | Modified | 11/07/2025, 14:21:52 |

  ### Tags:

  - Hydrocortisone
  - Dogs
  - Animals
  - Humans
  - Child
  - Mainstreaming, Education
  - Peer Group
  - Schools

  ### Attachments

  - Full Text
  - PubMed entry
- ## Bringing human-animal interaction to sport: Potential impacts on athletic performance

  |  |  |
  | --- | --- |
  | Item Type | Journal Article |
  | Author | Sutton Marvin |
  | Author | Kennet Sorenson |
  | Author | Jeffrey R. Stevens |
  | Abstract | To gain an edge in performance, athletes, coaches, trainers, and sport psychologists worldwide leverage findings from psychological research to develop training and performance strategies. The field of sport psychology draws upon research on stress, anxiety, mindfulness, and team building to develop these strategies. Here, we introduce human-animal interaction as a potential area of research that may apply to athletic performance. Structured interactions with animals-particularly therapy dogs-can provide physiological benefits associated with stress and the oxytocin system, psychological benefits for anxiety and motivation, and social benefits through social support. Yet these effects have not yet been systematically investigated in athletes. Integration of human-animal interactions into athletics can occur through animal visitation programmes and resident therapy animal programmes. Integrating human-animal interactions into athletics presents some unique challenges and limitations that must be considered before implementing these programmes, and these interactions are not a panacea that will work in every situation. But, given the amount of human-animal interaction research suggesting benefits in medicine, mental health, and education contexts, it is worthwhile exploring potential benefits not just for athletic performance, but also for injury prevention and recovery. HighlightsHuman-animal interaction is a potential area of research that may apply to athletic performance.Structured interactions with animals can provide physiological, psychological, and social benefits to athletes, through it is not a panacea that will work in every situation.Integrating human-animal interactions into athletics presents some unique challenges and limitations that must be considered before implementing these programs. |
  | Date | 2022-07 |
  | Language | eng |
  | Short Title | Bringing human-animal interaction to sport |
  | Library Catalogue | PubMed |
  | Volume | 22 |
  | Pages | 955-963 |
  | Publication | European Journal of Sport Science |
  | DOI | 10.1080/17461391.2021.1916084 |
  | Issue | 7 |
  | Journal Abbr | Eur J Sport Sci |
  | ISSN | 1536-7290 |
  | PMID | 33840355 |
  | Date Added | 11/07/2025, 11:45:53 |
  | Modified | 11/07/2025, 11:45:56 |

  ### Tags:

  - oxytocin
  - Dogs
  - human-animal interaction
  - stress
  - Animals
  - Human-Animal Interaction
  - Humans
  - Athletes
  - Athletic Injuries
  - Athletic performance
  - Athletic Performance
  - Mindfulness
  - performance anxiety
  - sport psychology
  - Sports Medicine

  ### Attachments

  - PubMed entry
  - Submitted Version
- ## Effects of contact with a dog on prefrontal brain activation in patients in a minimally conscious state: A controlled crossover trial

  |  |  |
  | --- | --- |
  | Item Type | Journal Article |
  | Author | Rahel Marti |
  | Author | Milena Petignat |
  | Author | Valentine L. Marcar |
  | Author | Jan Hattendorf |
  | Author | Martin Wolf |
  | Author | Margret Hund-Georgiadis |
  | Author | Karin Hediger |
  | Abstract | The first studies have indicated that animal-assisted therapy benefits patients in a minimally conscious state (MCS), but the evidence is scarce. It is thus crucial to understand how these patients react to animal contact. This study aimed to measure the prefrontal brain activation in MCS patients during contact with a dog compared with a plush animal using functional near-infrared spectroscopy (fNIRS). We conducted a controlled crossover trial with 22 MCS patients, who each participated in six sessions. Patients interacted with a dog in three sessions and with a plush animal in three control sessions. Each session consisted of five 2-minute phases with a neutral phase at the start and the end. The contact intensity with the dog or the plush animal increased from the second to the fourth phase. The fNIRS parameters did not differ between the conditions. The mean heart rate was significantly higher in the dog condition than in the control. In both conditions, prefrontal brain activation, mean heart rate, and one heart rate variability parameter increased with the increased intensity of contact with the dog and plush animal. The results show that MCS patients react with the same prefrontal brain activation during contact with a dog and a plush animal but have increased heart rate in contact with the dog, indicating physiological arousal. These findings suggest that the incorporation of animals into MCS therapy has the potential to stimulate patients, thereby facilitating greater participation. However, more research is needed to understand the effects of animals on brain activation. |
  | Date | 2025-06-21 |
  | Language | eng |
  | Short Title | Effects of contact with a dog on prefrontal brain activation in patients in a minimally conscious state |
  | Library Catalogue | PubMed |
  | Volume | 577 |
  | Pages | 175-189 |
  | Publication | Neuroscience |
  | DOI | 10.1016/j.neuroscience.2025.05.014 |
  | Journal Abbr | Neuroscience |
  | ISSN | 1873-7544 |
  | PMID | 40360130 |
  | Date Added | 11/07/2025, 11:34:20 |
  | Modified | 11/07/2025, 11:34:20 |

  ### Tags:

  - Brain activation
  - Functional near-infrared spectroscopy
  - Heart rate
  - Human–animal interaction
  - Minimally conscious state
  - Neurorehabilitation
  - Dogs
  - Animals
  - Female
  - Adult
  - Heart Rate
  - Humans
  - Male
  - Middle Aged
  - Aged
  - Animal Assisted Therapy
  - Cross-Over Studies
  - Persistent Vegetative State
  - Prefrontal Cortex
  - Spectroscopy, Near-Infrared

  ### Attachments

  - PubMed entry
- ## Effects of contact with a dog on prefrontal brain activation in patients in a minimally conscious state: A controlled crossover trial

  |  |  |
  | --- | --- |
  | Item Type | Journal Article |
  | Author | Rahel Marti |
  | Author | Milena Petignat |
  | Author | Valentine L. Marcar |
  | Author | Jan Hattendorf |
  | Author | Martin Wolf |
  | Author | Margret Hund-Georgiadis |
  | Author | Karin Hediger |
  | Abstract | The first studies have indicated that animal-assisted therapy benefits patients in a minimally conscious state (MCS), but the evidence is scarce. It is thus crucial to understand how these patients react to animal contact. This study aimed to measure the prefrontal brain activation in MCS patients during contact with a dog compared with a plush animal using functional near-infrared spectroscopy (fNIRS). We conducted a controlled crossover trial with 22 MCS patients, who each participated in six sessions. Patients interacted with a dog in three sessions and with a plush animal in three control sessions. Each session consisted of five 2-minute phases with a neutral phase at the start and the end. The contact intensity with the dog or the plush animal increased from the second to the fourth phase. The fNIRS parameters did not differ between the conditions. The mean heart rate was significantly higher in the dog condition than in the control. In both conditions, prefrontal brain activation, mean heart rate, and one heart rate variability parameter increased with the increased intensity of contact with the dog and plush animal. The results show that MCS patients react with the same prefrontal brain activation during contact with a dog and a plush animal but have increased heart rate in contact with the dog, indicating physiological arousal. These findings suggest that the incorporation of animals into MCS therapy has the potential to stimulate patients, thereby facilitating greater participation. However, more research is needed to understand the effects of animals on brain activation. |
  | Date | 2025-06-21 |
  | Language | eng |
  | Short Title | Effects of contact with a dog on prefrontal brain activation in patients in a minimally conscious state |
  | Library Catalogue | PubMed |
  | Volume | 577 |
  | Pages | 175-189 |
  | Publication | Neuroscience |
  | DOI | 10.1016/j.neuroscience.2025.05.014 |
  | Journal Abbr | Neuroscience |
  | ISSN | 1873-7544 |
  | PMID | 40360130 |
  | Date Added | 11/07/2025, 11:37:24 |
  | Modified | 11/07/2025, 11:37:24 |

  ### Tags:

  - Brain activation
  - Functional near-infrared spectroscopy
  - Heart rate
  - Human–animal interaction
  - Minimally conscious state
  - Neurorehabilitation
  - Dogs
  - Animals
  - Female
  - Adult
  - Heart Rate
  - Humans
  - Male
  - Middle Aged
  - Aged
  - Animal Assisted Therapy
  - Cross-Over Studies
  - Persistent Vegetative State
  - Prefrontal Cortex
  - Spectroscopy, Near-Infrared

  ### Attachments

  - PubMed entry
- ## Effects of Affiliative Human-Animal Interaction on Dog Salivary and Plasma Oxytocin and Vasopressin

  |  |  |
  | --- | --- |
  | Item Type | Journal Article |
  | Author | Evan L. MacLean |
  | Author | Laurence R. Gesquiere |
  | Author | Nancy R. Gee |
  | Author | Kerinne Levy |
  | Author | W. Lance Martin |
  | Author | C. Sue Carter |
  | Abstract | Oxytocin (OT) and vasopressin (AVP) are neuropeptides with diverse effects on social behavior, cognition and stress responses. Recent studies suggest that OT facilitates and responds to affiliative forms of human-animal interaction (HAI). However, previous studies measuring OT and AVP in dogs have been limited to measures from blood or urine, which present concerns related to the invasiveness of sample collection, the potential for matrix interference in immunoassays, and whether samples can be collected at precise time points to assess event-linked endocrine responses. Previous studies from our laboratory validated salivary measures of OT and AVP in dogs, however, it is currently unknown whether these measures respond dynamically to aspects of HAI. Here, we investigated the effects of affiliative forms of HAI on both plasma and salivary OT and AVP in dogs. We employed a within- and between-subjects design with a group of Labrador retrievers and Labrador retriever × golden retriever crosses (23 females, 15 males). Half of the dogs engaged in 10 min of free-form friendly interaction with a human experimenter (HAI condition), and the other half rested quietly in the same environment, without human interaction (control condition). We collected blood and saliva samples before, and immediately following both experimental conditions, and all samples were analyzed using enzyme-linked immunosorbent assays (ELISAs) following previously validated protocols. Dogs participating in HAI exhibited a significant increase in both salivary OT (+39%) and plasma OT (+5.7%) whereas dogs in the control group did not. Salivary AVP showed no change in the HAI group but increased significantly (+33%) in the control group. Plasma AVP decreased significantly following HAI (-13%) but did not change across time in the control condition. Within the dogs exposed to HAI, increases in salivary OT, and decreases in plasma AVP, were predicted by the extent of affiliative behavior between the dog and human (indexed by scores from a principal components analysis of social behaviors between the dog and human). Collectively our results suggest that measures of salivary OT and AVP provide useful biomarkers in studies of HAI, and afford a flexible and non-invasive toolkit than can be employed in diverse research contexts. |
  | Date | 2017 |
  | Language | eng |
  | Library Catalogue | PubMed |
  | Volume | 8 |
  | Pages | 1606 |
  | Publication | Frontiers in Psychology |
  | DOI | 10.3389/fpsyg.2017.01606 |
  | Journal Abbr | Front Psychol |
  | ISSN | 1664-1078 |
  | PMID | 28979224 |
  | PMCID | PMC5611686 |
  | Date Added | 11/07/2025, 11:45:53 |
  | Modified | 11/07/2025, 11:45:56 |

  ### Tags:

  - oxytocin
  - human–animal interaction
  - dog
  - ELISA
  - plasma
  - saliva
  - vasopressin

  ### Attachments

  - Full Text
  - PubMed entry
- ## Validation of salivary oxytocin and vasopressin as biomarkers in domestic dogs

  |  |  |
  | --- | --- |
  | Item Type | Journal Article |
  | Author | Evan L. MacLean |
  | Author | Laurence R. Gesquiere |
  | Author | Nancy Gee |
  | Author | Kerinne Levy |
  | Author | W. Lance Martin |
  | Author | C. Sue Carter |
  | Abstract | BACKGROUND: Oxytocin (OT) and Vasopressin (AVP) are phylogenetically conserved neuropeptides with effects on social behavior, cognition and stress responses. Although OT and AVP are most commonly measured in blood, urine and cerebrospinal fluid (CSF), these approaches present an array of challenges including concerns related to the invasiveness of sample collection, the potential for matrix interference in immunoassays, and whether samples can be collected at precise time points to assess event-linked endocrine responses. NEW METHOD: We validated enzyme-linked immunosorbent assays (ELISAs) for the measurement of salivary OT and AVP in domestic dogs. RESULTS: Both OT and AVP were present in dog saliva and detectable by ELISA and high performance liquid chromatography - mass spectrometry (HPLC-MS). OT concentrations in dog saliva were much higher than those typically detected in humans. OT concentrations in the same samples analyzed with and without sample extraction were highly correlated, but this was not true for AVP. ELISA validation studies revealed good accuracy and parallelism, both with and without solid phase extraction. Collection of salivary samples with different synthetic swabs, or following salivary stimulation or the consumption of food led to variance in results. However, samples collected from the same dogs using different techniques tended to be positively correlated. We detected concurrent elevations in salivary and plasma OT during nursing. COMPARISON WITH EXISTING METHODS: There are currently no other validated methods for measuring OT/AVP in dog saliva. CONCLUSIONS: OT and AVP are present in dog saliva, and ELISAs for their detection are methodologically valid. |
  | Date | 2018-01-01 |
  | Language | eng |
  | Library Catalogue | PubMed |
  | Volume | 293 |
  | Pages | 67-76 |
  | Publication | Journal of Neuroscience Methods |
  | DOI | 10.1016/j.jneumeth.2017.08.033 |
  | Journal Abbr | J Neurosci Methods |
  | ISSN | 1872-678X |
  | PMID | 28865986 |
  | Date Added | 11/07/2025, 11:45:53 |
  | Modified | 11/07/2025, 11:45:58 |

  ### Tags:

  - Oxytocin
  - Dogs
  - Animals
  - Female
  - Male
  - Human animal interaction
  - Saliva
  - Enzyme-Linked Immunosorbent Assay
  - Validation
  - Dog
  - Biomarkers
  - Chromatography, High Pressure Liquid
  - Immunoassay
  - Lactation
  - Mass Spectrometry
  - Vasopressin
  - Vasopressins

  ### Attachments

  - Full Text
  - PubMed entry
- ## Human-animal interaction, stress, and embryo production in Bos indicus embryo donors under tropical conditions

  |  |  |
  | --- | --- |
  | Item Type | Journal Article |
  | Author | Gustavo Guerino Macedo |
  | Author | Carmem Estefânia Serra Neto Zúccari |
  | Author | Urbano Gomes Pinto de Abreu |
  | Author | João Alberto Negrão |
  | Author | Eliane Vianna da Costa e Silva |
  | Abstract | This study investigated the effect of human-animal interaction (HAI) and the stress response on the quality of embryo production in superovulated Nelore (Bos indicus) cattle, under tropical conditions. Thirty-two females underwent a superovulation protocol for 5 days. Cortisol concentrations were determined in blood plasma collected on days 0, 4, and 5. Artificial insemination was performed on days 4 and 5, and nonsurgical embryo flushing on day 11. Embryo production and viability were determined. Human stimulation, animal behaviors, accidents, and handling time were recorded to assess HAI. Cattle age was negatively correlated with accidents, frequency of aversive behaviors, and negative stimuli by stockperson during transit through corral compartments to receive superovulation treatments. The factor analysis revealed two distinct groups. The first group was called stressed and had higher cortisol concentration than the nonstressed group, 16.0 ± 2.1 and 12.5 ± 1.0 ng/mL, respectively. Comparisons between these groups showed that the frequency of voice emissions by the stockperson and the number of accidents were higher in the stressed group, and also, the mean handling time was longer in the stressed group than for the nonstressed. As a result, viability rate of the embryos was 19% lower in the stressed group (P < 0.05). This indicates that intensive negative HAI is likely related to stress, which affects embryo production in a superovulation program. |
  | Date | 2011-08 |
  | Language | eng |
  | Library Catalogue | PubMed |
  | Volume | 43 |
  | Pages | 1175-1182 |
  | Publication | Tropical Animal Health and Production |
  | DOI | 10.1007/s11250-011-9820-6 |
  | Issue | 6 |
  | Journal Abbr | Trop Anim Health Prod |
  | ISSN | 1573-7438 |
  | PMID | 21455696 |
  | Date Added | 11/07/2025, 14:21:52 |
  | Modified | 11/07/2025, 14:21:52 |

  ### Tags:

  - Hydrocortisone
  - Animals
  - Behavior, Animal
  - Female
  - Stress, Physiological
  - Cattle
  - Brazil
  - Animal Welfare
  - Embryo Transfer
  - Human Activities
  - Insemination, Artificial
  - Superovulation
  - Tropical Climate

  ### Attachments

  - PubMed entry
- ## Salivary oxytocin in pigs, cattle, and goats during positive human-animal interactions

  |  |  |
  | --- | --- |
  | Item Type | Journal Article |
  | Author | Stephanie Lürzel |
  | Author | Laura Bückendorf |
  | Author | Susanne Waiblinger |
  | Author | Jean-Loup Rault |
  | Abstract | Research on oxytocin (OT) has provided crucial insight into the neuroendocrine mechanisms of social behavior and human-animal interactions. Nonetheless, this peptide hormone remains challenging to sample, usually requiring invasive measures in the brain or blood, which limits its use. We conducted an analytical validation of the measurement of OT in the saliva of three common farm animal species: pigs, cattle and goats. We then investigated the biological significance of salivary OT concentrations by using a positive human-animal interaction paradigm. The paradigm varied in terms of human familiarity and included a control condition where the animal was alone in the testing pen. A total of 18 pigs, 18 cattle and 9 goats were subjected to each condition using a within-individual counterbalanced design. Animals were free to choose to interact with the human. Saliva samples were collected immediately before and after the 10-min test, and behavior during the test was analyzed. Oxytocin could be reliably measured by ELISA in the saliva of goats (without extraction), and in the saliva of pigs and cattle after extraction. Salivary OT concentration did not change consistently according to testing condition. However, salivary OT concentration was significantly associated with some behaviors, with different relationships depending on the species. In conclusion, OT can be reliably measured in the saliva of goats and, after extraction, in the saliva of pigs and cattle. The relationships of OT with human-animal interactions and animal behavior were complex and to some extent species-dependent, possibly reflecting the perception of the interaction by the animal. |
  | Date | 2020-05 |
  | Language | eng |
  | Library Catalogue | PubMed |
  | Volume | 115 |
  | Pages | 104636 |
  | Publication | Psychoneuroendocrinology |
  | DOI | 10.1016/j.psyneuen.2020.104636 |
  | Journal Abbr | Psychoneuroendocrinology |
  | ISSN | 1873-3360 |
  | PMID | 32160578 |
  | Date Added | 11/07/2025, 11:45:53 |
  | Modified | 11/07/2025, 11:45:57 |

  ### Tags:

  - Oxytocin
  - Animals
  - Behavior, Animal
  - Female
  - Swine
  - Adult
  - Human-Animal Interaction
  - Humans
  - Saliva
  - Social Behavior
  - Affiliative
  - Cattle
  - Enzyme-Linked Immunosorbent Assay
  - Familiar
  - Goats
  - Human-animal relationship
  - Recognition, Psychology
  - Reproducibility of Results
  - Validation

  ### Attachments

  - PubMed entry
- ## Early human contact and housing for pigs - part 1: responses to humans, novelty and isolation

  |  |  |
  | --- | --- |
  | Item Type | Journal Article |
  | Author | M. E. Lucas |
  | Author | L. M. Hemsworth |
  | Author | K. L. Butler |
  | Author | R. S. Morrison |
  | Author | A. J. Tilbrook |
  | Author | J. N. Marchant |
  | Author | J.-L. Rault |
  | Author | R. Y. Galea |
  | Author | P. H. Hemsworth |
  | Abstract | The development of fear and stress responses in animals can be influenced by early life experiences, including interactions with humans, maternal care, and the physical surroundings. This paper is the first of three reporting on a large experiment examining the effects of the early housing environment and early positive human contact on stress resilience in pigs. This first paper reports on the responses of pigs to humans, novelty, and social isolation. Using a 2 × 2 factorial design, 48 litters of pigs were reared in either a conventional farrowing crate (FC) where the sow was confined or a loose farrowing pen (LP; PigSAFE pen) which was larger, more physically complex and allowed the sow to move freely throughout the farrowing and lactation period. Piglets were provided with either routine contact from stockpeople (C), or routine contact plus regular opportunities for positive human contact (+HC) involving 5 min of scratching, patting and stroking imposed to the litter 5 days/week from 0-4 weeks of age. The positive handling treatment was highly effective in reducing piglets' fear of humans, based on +HC piglets showing greater approach and less avoidance of an unfamiliar person at 3 weeks of age. There was evidence that this reduction in fear of humans lasted well beyond when the treatment was applied (lactation), with +HC pigs showing greater approach and less avoidance of humans in tests at 6, 9 and 14 weeks of age. The +HC treatment also reduced piglets' fear of a novel object at 3 weeks of age, and for pigs in FC, the cortisol response after social isolation at 7 weeks of age. Rearing in FC compared to LP reduced piglets' fear of novelty at 3 weeks of age, as well as their vocalisations and cortisol response to isolation at 7 weeks of age. The FC pigs showed greater approach and less avoidance of humans compared to LP pigs at 3, 4 and 6 weeks of age, but not at 9 and 14 weeks of age. These results show that positive handling early in life can reduce pigs' fear of humans, fear of novelty and physiological stress response to social isolation. The LP pigs were reared in a more isolated environment with less overall contact with stockpeople and other pigs, which may have increased their fear responses to humans and novel situations, suggesting that different housing systems can modulate these pigs' responses. |
  | Date | 2024-06 |
  | Language | eng |
  | Short Title | Early human contact and housing for pigs - part 1 |
  | Library Catalogue | PubMed |
  | Volume | 18 |
  | Pages | 101164 |
  | Publication | Animal: An International Journal of Animal Bioscience |
  | DOI | 10.1016/j.animal.2024.101164 |
  | Issue | 6 |
  | Journal Abbr | Animal |
  | ISSN | 1751-732X |
  | PMID | 38761440 |
  | Date Added | 11/07/2025, 14:21:52 |
  | Modified | 11/07/2025, 14:21:52 |

  ### Tags:

  - Hydrocortisone
  - Animals
  - Behavior, Animal
  - Female
  - Swine
  - Human-Animal Interaction
  - Humans
  - Male
  - Stress, Psychological
  - Animal Husbandry
  - Animal welfare
  - Early experience
  - Fear
  - Handling
  - Housing, Animal
  - Human-animal interactions
  - Social Isolation
  - Stress

  ### Attachments

  - PubMed entry
- ## Current approaches to modeling the virtual reality in rodents for the assessment of brain plasticity and behavior

  |  |  |
  | --- | --- |
  | Item Type | Journal Article |
  | Author | Olga L. Lopatina |
  | Author | Andrey V. Morgun |
  | Author | Yana V. Gorina |
  | Author | Vladimir V. Salmin |
  | Author | Alla B. Salmina |
  | Abstract | Virtual reality (VR) and augmented reality (AR) have become valuable tools to study brains and behaviors resulting in development of new methods of diagnostics and treatment. Neurodegenerаtion is one of the best examples demonstrating efficacy of VR/АR technologies in modern neurology. Development of novel VR systems for rodents and combination of VR tools with up-to-date imaging techniques (i.e. MRI, imaging of neural networks etc.), brain electrophysiology (EEG, patch-clamp), precise analytics (microdialysis) allowed implementing of VR protocols into the animal neurobiology to study brain plasticity, sensorimotor integration, spatial navigation, memory, and decision-making. VR/AR for rodents is а young field of experimental neuroscience and has already provided more consistent testing conditions, less human-animal interaction, opportunities to use a wider variety of experimental parameters. Here we discuss present and future perspectives of using VR/AR to assess brain plasticity, neurogenesis and complex behavior in rodent and human study, and their advantages for translational neuroscience. |
  | Date | 2020-04-01 |
  | Language | eng |
  | Library Catalogue | PubMed |
  | Volume | 335 |
  | Pages | 108616 |
  | Publication | Journal of Neuroscience Methods |
  | DOI | 10.1016/j.jneumeth.2020.108616 |
  | Journal Abbr | J Neurosci Methods |
  | ISSN | 1872-678X |
  | PMID | 32007483 |
  | Date Added | 11/07/2025, 11:33:54 |
  | Modified | 11/07/2025, 11:33:54 |

  ### Tags:

  - Animals
  - Augmented reality
  - Cognition
  - Enriched environment
  - Memory
  - Neuronal Plasticity
  - Neuroplastisity
  - Rodentia
  - User-Computer Interface
  - Virtual reality
  - Virtual Reality

  ### Attachments

  - PubMed entry
- ## Cognitive mechanisms and neurological foundations of companion animals' role in enhancing human psychological well-being

  |  |  |
  | --- | --- |
  | Item Type | Journal Article |
  | Author | Heng Liu |
  | Author | Jingyuan Lin |
  | Author | Wuji Lin |
  | Abstract | The impact of companion animals on human psychological health has garnered widespread attention. Research demonstrates that companion animals contribute positively in various ways, including reducing depression, anxiety, stress, and fostering positive emotions in humans. Recent studies have revealed significant changes in the activity levels of human emotion-related cortical areas (such as the frontal cortex and amygdala) and neurotransmitter (e.g., oxytocin, cortisol) secretion due to interaction with companion animals. However, research in this domain is still in a nascent stage, with many unknowns in the cognitive neural mechanisms involved. This paper proposes that to understand the cognitive mechanisms through which companion animals affect human psychological health, we need to examine changes in emotional cognitive processing. It aims to uncover the neurological underpinnings of how companion animals enhance human psychological well-being from the perspective of brain connectivity. This approach is expected to provide theoretical support and direction for future research and practical applications in this field. |
  | Date | 2024 |
  | Language | eng |
  | Library Catalogue | PubMed |
  | Volume | 15 |
  | Pages | 1354220 |
  | Publication | Frontiers in Psychology |
  | DOI | 10.3389/fpsyg.2024.1354220 |
  | Journal Abbr | Front Psychol |
  | ISSN | 1664-1078 |
  | PMID | 38721326 |
  | PMCID | PMC11076790 |
  | Date Added | 11/07/2025, 11:45:53 |
  | Modified | 11/07/2025, 11:45:57 |

  ### Tags:

  - human-animal interaction
  - animal-assisted therapy
  - cognitive neural mechanisms
  - companion animals
  - emotion regulation

  ### Attachments

  - Full Text
  - PubMed entry
- ## Cognitive mechanisms and neurological foundations of companion animals' role in enhancing human psychological well-being

  |  |  |
  | --- | --- |
  | Item Type | Journal Article |
  | Author | Heng Liu |
  | Author | Jingyuan Lin |
  | Author | Wuji Lin |
  | Abstract | The impact of companion animals on human psychological health has garnered widespread attention. Research demonstrates that companion animals contribute positively in various ways, including reducing depression, anxiety, stress, and fostering positive emotions in humans. Recent studies have revealed significant changes in the activity levels of human emotion-related cortical areas (such as the frontal cortex and amygdala) and neurotransmitter (e.g., oxytocin, cortisol) secretion due to interaction with companion animals. However, research in this domain is still in a nascent stage, with many unknowns in the cognitive neural mechanisms involved. This paper proposes that to understand the cognitive mechanisms through which companion animals affect human psychological health, we need to examine changes in emotional cognitive processing. It aims to uncover the neurological underpinnings of how companion animals enhance human psychological well-being from the perspective of brain connectivity. This approach is expected to provide theoretical support and direction for future research and practical applications in this field. |
  | Date | 2024 |
  | Language | eng |
  | Library Catalogue | PubMed |
  | Volume | 15 |
  | Pages | 1354220 |
  | Publication | Frontiers in Psychology |
  | DOI | 10.3389/fpsyg.2024.1354220 |
  | Journal Abbr | Front Psychol |
  | ISSN | 1664-1078 |
  | PMID | 38721326 |
  | PMCID | PMC11076790 |
  | Date Added | 11/07/2025, 14:21:52 |
  | Modified | 11/07/2025, 14:21:52 |

  ### Tags:

  - human-animal interaction
  - animal-assisted therapy
  - cognitive neural mechanisms
  - companion animals
  - emotion regulation

  ### Attachments

  - Full Text
  - PubMed entry
- ## Quantitative heartbeat coupling measures in human-horse interaction

  |  |  |
  | --- | --- |
  | Item Type | Journal Article |
  | Author | Antonio Lanata |
  | Author | Andrea Guidi |
  | Author | Gaetano Valenza |
  | Author | Paolo Baragli |
  | Author | Enzo Pasquale Scilingo |
  | Abstract | We present a study focused on a quantitative estimation of a human-horse dynamic interaction. A set of measures based on magnitude and phase coupling between heartbeat dynamics of both humans and horses in three different conditions is reported: no interaction, visual/olfactory interaction and grooming. Specifically, Magnitude Squared Coherence (MSC), Mean Phase Coherence (MPC) and Dynamic Time Warping (DTW) have been used as estimators of the amount of coupling between human and horse through the analysis of their heart rate variability (HRV) time series in a group of eleven human subjects, and one horse. The rationale behind this study is that the interaction of two complex biological systems go towards a coupling process whose dynamical evolution is modulated by the kind and time duration of the interaction itself. We achieved a congruent and consistent statistical significant difference for all of the three indices. Moreover, a Nearest Mean Classifier was able to recognize the three classes of interaction with an accuracy greater than 70%. Although preliminary, these encouraging results allow a discrimination of three distinct phases in a real human-animal interaction opening to the characterization of the empirically proven relationship between human and horse. |
  | Date | 2016-08 |
  | Language | eng |
  | Library Catalogue | PubMed |
  | Volume | 2016 |
  | Pages | 2696-2699 |
  | Publication | Annual International Conference of the IEEE Engineering in Medicine and Biology Society. IEEE Engineering in Medicine and Biology Society. Annual International Conference |
  | DOI | 10.1109/EMBC.2016.7591286 |
  | Journal Abbr | Annu Int Conf IEEE Eng Med Biol Soc |
  | ISSN | 2694-0604 |
  | PMID | 28268877 |
  | Date Added | 11/07/2025, 11:37:24 |
  | Modified | 11/07/2025, 11:37:24 |

  ### Tags:

  - Animals
  - Heart Rate
  - Humans
  - Animal Husbandry
  - Horses

  ### Attachments

  - PubMed entry
- ## Correlations between behavior and hormone concentrations or gut microbiome imply that domestic cats (Felis silvestris catus) living in a group are not like 'groupmates'

  |  |  |
  | --- | --- |
  | Item Type | Journal Article |
  | Author | Hikari Koyasu |
  | Author | Hironobu Takahashi |
  | Author | Moeka Yoneda |
  | Author | Syunpei Naba |
  | Author | Natsumi Sakawa |
  | Author | Ikuto Sasao |
  | Author | Miho Nagasawa |
  | Author | Takefumi Kikusui |
  | Abstract | Domestic cats (Felis silvestris catus) can live in high densities, although most feline species are solitary and exclusively territorial animals; it is possible that certain behavioral strategies enable this phenomenon. These behaviors are regulated by hormones and the gut microbiome, which, in turn, is influenced by domestication. Therefore, we investigated the relationships between the sociality, hormone concentrations, and gut microbiome of domestic cats by conducting three sets of experiments for each group of five cats and analyzing their behavior, hormone concentrations (cortisol, oxytocin, and testosterone), and their gut microbiomes. We observed that individuals with high cortisol and testosterone concentrations established less contact with others, and individuals with high oxytocin concentrations did not exhibit affiliative behaviors as much as expected. Additionally, the higher the frequency of contact among the individuals, the greater the similarity in gut microbiome; gut microbial composition was also related to behavioral patterns and cortisol secretion. Notably, individuals with low cortisol and testosterone concentrations were highly tolerant, making high-density living easy. Oxytocin usually functions in an affiliative manner within groups, but our results suggest that even if typically solitary and territorial animals live in high densities, their oxytocin functions are opposite to those of typically group-living animals. |
  | Date | 2022 |
  | Language | eng |
  | Library Catalogue | PubMed |
  | Volume | 17 |
  | Pages | e0269589 |
  | Publication | PloS One |
  | DOI | 10.1371/journal.pone.0269589 |
  | Issue | 7 |
  | Journal Abbr | PLoS One |
  | ISSN | 1932-6203 |
  | PMID | 35895662 |
  | PMCID | PMC9328509 |
  | Date Added | 11/07/2025, 11:45:53 |
  | Modified | 11/07/2025, 11:45:59 |

  ### Tags:

  - Hydrocortisone
  - Oxytocin
  - Animals
  - Cats
  - Felis
  - Gastrointestinal Microbiome
  - Testosterone

  ### Attachments

  - Full Text
  - PubMed entry
- ## Sociality of Cats toward Humans Can Be Influenced by Hormonal and Socio-Environmental Factors: Pilot Study

  |  |  |
  | --- | --- |
  | Item Type | Journal Article |
  | Author | Hikari Koyasu |
  | Author | Hironobu Takahashi |
  | Author | Ikuto Sasao |
  | Author | Saho Takagi |
  | Author | Miho Nagasawa |
  | Author | Takefumi Kikusui |
  | Abstract | Individual differences in the sociality of cats are influenced by inherited and environmental factors. We recently revealed that hormones can make a difference in intraspecies social behavior. It remains unclear whether cat behavior toward humans is modulated by hormones. Therefore, we analyzed the relationship between cat behavior and their basal hormone concentrations after spending time together with human experimenters. In addition, we analyzed the relationship between cat behavior and the timing of when the individual cats began living with a human because the sociality of cats could be dependent on their developmental experiences. The results showed that male cats that began living with humans earlier had more contact with an experimenter. In addition, individual male cats with low testosterone levels were more likely to interact with an experimenter. These findings of this pilot study suggest that the sociality of male cats toward humans is affected by testosterone and the age at which they begin to live with humans. |
  | Date | 2022-12-30 |
  | Language | eng |
  | Short Title | Sociality of Cats toward Humans Can Be Influenced by Hormonal and Socio-Environmental Factors |
  | Library Catalogue | PubMed |
  | Volume | 13 |
  | Pages | 146 |
  | Publication | Animals: an open access journal from MDPI |
  | DOI | 10.3390/ani13010146 |
  | Issue | 1 |
  | Journal Abbr | Animals (Basel) |
  | ISSN | 2076-2615 |
  | PMID | 36611754 |
  | PMCID | PMC9817699 |
  | Date Added | 11/07/2025, 11:45:53 |
  | Modified | 11/07/2025, 11:45:59 |

  ### Tags:

  - cortisol
  - oxytocin
  - animal socialization
  - cat behavior
  - cat-human interaction
  - testosterone

  ### Attachments

  - Full Text
  - PubMed entry
- ## Correlations between behavior and hormone concentrations or gut microbiome imply that domestic cats (Felis silvestris catus) living in a group are not like 'groupmates'

  |  |  |
  | --- | --- |
  | Item Type | Journal Article |
  | Author | Hikari Koyasu |
  | Author | Hironobu Takahashi |
  | Author | Moeka Yoneda |
  | Author | Syunpei Naba |
  | Author | Natsumi Sakawa |
  | Author | Ikuto Sasao |
  | Author | Miho Nagasawa |
  | Author | Takefumi Kikusui |
  | Abstract | Domestic cats (Felis silvestris catus) can live in high densities, although most feline species are solitary and exclusively territorial animals; it is possible that certain behavioral strategies enable this phenomenon. These behaviors are regulated by hormones and the gut microbiome, which, in turn, is influenced by domestication. Therefore, we investigated the relationships between the sociality, hormone concentrations, and gut microbiome of domestic cats by conducting three sets of experiments for each group of five cats and analyzing their behavior, hormone concentrations (cortisol, oxytocin, and testosterone), and their gut microbiomes. We observed that individuals with high cortisol and testosterone concentrations established less contact with others, and individuals with high oxytocin concentrations did not exhibit affiliative behaviors as much as expected. Additionally, the higher the frequency of contact among the individuals, the greater the similarity in gut microbiome; gut microbial composition was also related to behavioral patterns and cortisol secretion. Notably, individuals with low cortisol and testosterone concentrations were highly tolerant, making high-density living easy. Oxytocin usually functions in an affiliative manner within groups, but our results suggest that even if typically solitary and territorial animals live in high densities, their oxytocin functions are opposite to those of typically group-living animals. |
  | Date | 2022 |
  | Language | eng |
  | Library Catalogue | PubMed |
  | Volume | 17 |
  | Pages | e0269589 |
  | Publication | PloS One |
  | DOI | 10.1371/journal.pone.0269589 |
  | Issue | 7 |
  | Journal Abbr | PLoS One |
  | ISSN | 1932-6203 |
  | PMID | 35895662 |
  | PMCID | PMC9328509 |
  | Date Added | 11/07/2025, 14:21:52 |
  | Modified | 11/07/2025, 14:21:52 |

  ### Tags:

  - Hydrocortisone
  - Oxytocin
  - Animals
  - Cats
  - Felis
  - Gastrointestinal Microbiome
  - Testosterone

  ### Attachments

  - Full Text
  - PubMed entry
- ## Sociality of Cats toward Humans Can Be Influenced by Hormonal and Socio-Environmental Factors: Pilot Study

  |  |  |
  | --- | --- |
  | Item Type | Journal Article |
  | Author | Hikari Koyasu |
  | Author | Hironobu Takahashi |
  | Author | Ikuto Sasao |
  | Author | Saho Takagi |
  | Author | Miho Nagasawa |
  | Author | Takefumi Kikusui |
  | Abstract | Individual differences in the sociality of cats are influenced by inherited and environmental factors. We recently revealed that hormones can make a difference in intraspecies social behavior. It remains unclear whether cat behavior toward humans is modulated by hormones. Therefore, we analyzed the relationship between cat behavior and their basal hormone concentrations after spending time together with human experimenters. In addition, we analyzed the relationship between cat behavior and the timing of when the individual cats began living with a human because the sociality of cats could be dependent on their developmental experiences. The results showed that male cats that began living with humans earlier had more contact with an experimenter. In addition, individual male cats with low testosterone levels were more likely to interact with an experimenter. These findings of this pilot study suggest that the sociality of male cats toward humans is affected by testosterone and the age at which they begin to live with humans. |
  | Date | 2022-12-30 |
  | Language | eng |
  | Short Title | Sociality of Cats toward Humans Can Be Influenced by Hormonal and Socio-Environmental Factors |
  | Library Catalogue | PubMed |
  | Volume | 13 |
  | Pages | 146 |
  | Publication | Animals: an open access journal from MDPI |
  | DOI | 10.3390/ani13010146 |
  | Issue | 1 |
  | Journal Abbr | Animals (Basel) |
  | ISSN | 2076-2615 |
  | PMID | 36611754 |
  | PMCID | PMC9817699 |
  | Date Added | 11/07/2025, 14:21:52 |
  | Modified | 11/07/2025, 14:21:52 |

  ### Tags:

  - cortisol
  - oxytocin
  - animal socialization
  - cat behavior
  - cat-human interaction
  - testosterone

  ### Attachments

  - Full Text
  - PubMed entry
- ## Behavioral and emotional co-modulation during dog-owner interaction measured by heart rate variability and activity

  |  |  |
  | --- | --- |
  | Item Type | Journal Article |
  | Author | Aija Koskela |
  | Author | Heini Törnqvist |
  | Author | Sanni Somppi |
  | Author | Katriina Tiira |
  | Author | Virpi-Liisa Kykyri |
  | Author | Laura Hänninen |
  | Author | Jan Kujala |
  | Author | Miho Nagasawa |
  | Author | Takefumi Kikusui |
  | Author | Miiamaaria V. Kujala |
  | Abstract | Behavioral and physiological synchrony facilitate emotional closeness in attachment relationships. The aim of this pseudorandomized cross-over study was to investigate the emotional and physiological link, designated as co-modulation, between dogs and their owners. We measured the heart rate variability (HRV) and physical activity of dogs belonging to co-operative breeds (n = 29) and their owners during resting baselines and positive interaction tasks (Stroking, Training, Sniffing, Playing) and collected survey data on owner temperament and dog-owner relationship. Although overall HRV and activity correlated between dogs and their owners across tasks, task-specific analyses showed that HRV of dogs and owners correlated during free behaving (Pre- and Post-Baseline), whereas the activity of dogs and owners correlated during predefined interaction tasks (Stroking and Playing). Dog overall HRV was the only predictive factor for owner overall HRV, while dog height, ownership duration, owner negative affectivity, and dog-owner interaction scale predicted dog overall HRV. Thus, the characteristics of dog, owner, and the relationship modified the HRV responses in dog-owner dyads. The physiology and behavior of dogs belonging to co-operative breeds and their owners were therefore co-modulated, demonstrating physiological and emotional connection comparable to those found in attachment relationships between humans. |
  | Date | 2024-10-24 |
  | Language | eng |
  | Library Catalogue | PubMed |
  | Volume | 14 |
  | Pages | 25201 |
  | Publication | Scientific Reports |
  | DOI | 10.1038/s41598-024-76831-x |
  | Issue | 1 |
  | Journal Abbr | Sci Rep |
  | ISSN | 2045-2322 |
  | PMID | 39448721 |
  | PMCID | PMC11502769 |
  | Date Added | 11/07/2025, 11:37:24 |
  | Modified | 11/07/2025, 11:37:24 |

  ### Tags:

  - Dogs
  - Animals
  - Behavior, Animal
  - Female
  - Adult
  - Heart Rate
  - Human-Animal Bond
  - Human-Animal Interaction
  - Humans
  - Male
  - Middle Aged
  - Cross-Over Studies
  - Activity
  - Canine
  - Dog–human interaction
  - Electrocardiography
  - Emotion
  - Emotions
  - HRV
  - Synchrony

  ### Attachments

  - Full Text PDF
  - PubMed entry
- ## Social bonding between humans, animals, and robots: Dogs outperform AIBOs, their robotic replicas, as social companions

  |  |  |
  | --- | --- |
  | Item Type | Journal Article |
  | Author | Stella Klumpe |
  | Author | Kelsey C. Mitchell |
  | Author | Emma Cox |
  | Author | Jeffrey S. Katz |
  | Author | Lucia Lazarowski |
  | Author | Gopikrishna Deshpande |
  | Author | Jonathan Gratch |
  | Author | Ewart J. de Visser |
  | Author | Hasan Ayaz |
  | Author | Xingnan Li |
  | Author | Adrian A. Franke |
  | Author | Frank Krueger |
  | Abstract | In the evolving landscape of technology, robots have emerged as social companions, prompting an investigation into social bonding between humans and robots. While human-animal interactions are well-studied, human-robot interactions (HRI) remain comparatively underexplored. Ethorobotics, a field of social robotic engineering based on ecology and ethology, suggests designing companion robots modeled on animal companions, which are simpler to emulate than humans. However, it is unclear whether these robots can match the social companionship provided by their original models. This study examined social bonding between humans and AIBOs, dog-inspired companion robots, compared to real dogs. Nineteen female participants engaged in 12 affiliative interactions with dogs and AIBOs across two counter-balanced, one-month bonding phases. Social bonding was assessed through urinary oxytocin (OXT) level change over an interaction, self-reported attachment using an adapted version of the Lexington Attachment to Pets Scale, and social companionship evaluations administering the Robot-Dog Questionnaire. To examine OXT level changes and self-reported attachment by comparing the two social companions, we conducted mixed-effects model analyses and planned follow-up comparisons. Frequency comparison, binary logistic regression, and thematic analysis were performed to analyze social companionship evaluations. Results revealed significant differences between dogs and AIBOs in fostering social bonds. OXT level change increased during interactions with dogs but decreased with AIBOs. Participants reported stronger attachment to dogs and rated them as better social companions. These findings highlight the current limitations of AIBOs in fostering social bonding immediately compared to dogs. Our study contributes to the growing HRI research by demonstrating an existing gap between AIBOs and dogs as social companions. It highlights the need for further investigation to understand the complexities of social bonding with companion robots, which is essential to implement successful applications for social robots in diverse domains such as the elderly and health care, education, and entertainment. |
  | Date | 2025 |
  | Language | eng |
  | Short Title | Social bonding between humans, animals, and robots |
  | Library Catalogue | PubMed |
  | Volume | 20 |
  | Pages | e0324312 |
  | Publication | PloS One |
  | DOI | 10.1371/journal.pone.0324312 |
  | Issue | 6 |
  | Journal Abbr | PLoS One |
  | ISSN | 1932-6203 |
  | PMID | 40460066 |
  | PMCID | PMC12132934 |
  | Date Added | 11/07/2025, 11:45:53 |
  | Modified | 11/07/2025, 11:45:59 |

  ### Tags:

  - Dogs
  - Animals
  - Female
  - Adult
  - Human-Animal Bond
  - Human-Animal Interaction
  - Humans
  - Young Adult
  - Social Behavior
  - Robotics

  ### Attachments

  - PubMed entry
- ## Children's Relationship With Their Pet Dogs and OXTR Genotype Predict Child-Pet Interaction in an Experimental Setting

  |  |  |
  | --- | --- |
  | Item Type | Journal Article |
  | Author | Darlene A. Kertes |
  | Author | Nathan Hall |
  | Author | Samarth S. Bhatt |
  | Abstract | Human-animal interaction (HAI) research has increasingly documented the important role of pet dogs in children's lives. The quality of interaction between children and their pet dogs, however, is likely influenced by individual differences among children as well as their perceived relationship with their pet dog. Ninety-seven children aged 7-12 years and their pet dogs participated in a laboratory protocol during which the child solicited interaction with their dog, from which time petting and gazing were recorded. Children reported on their perceived relationship with the pet dog via interview. Children provided saliva samples, from which a polymorphism in the oxytocin receptor, OXTR rs53576, which has long been implicated in social behavior, was genotyped. The results showed that OXTR genotype and children's perceived antagonism with the pet dog predicted the amount of petting, but not gazing, between children and their pet dogs. This research adds to the growing body of HAI research by documenting individual differences that may influence children's interactions with animals, which is key to research related to pet ownership and understanding factors that may impact therapeutic interventions involving HAI. |
  | Date | 2018 |
  | Language | eng |
  | Library Catalogue | PubMed |
  | Volume | 9 |
  | Pages | 1472 |
  | Publication | Frontiers in Psychology |
  | DOI | 10.3389/fpsyg.2018.01472 |
  | Journal Abbr | Front Psychol |
  | ISSN | 1664-1078 |
  | PMID | 30233440 |
  | PMCID | PMC6134068 |
  | Date Added | 11/07/2025, 11:45:53 |
  | Modified | 11/07/2025, 11:45:59 |

  ### Tags:

  - oxytocin
  - dogs
  - human–animal interaction
  - OXTR
  - child
  - oxytocin receptor gene
  - petting
  - relationships

  ### Attachments

  - Full Text
  - PubMed entry
- ## Pet Dogs: Does their presence influence preadolescents' emotional responses to a social stressor?

  |  |  |
  | --- | --- |
  | Item Type | Journal Article |
  | Author | Kathryn A. Kerns |
  | Author | Kaela L. Stuart-Parrigon |
  | Author | Karin G. Coifman |
  | Author | Manfred H. M. van Dulmen |
  | Author | Amanda Koehn |
  | Abstract | Despite interest in human-animal interaction, few studies have tested whether the presence of a dog facilitates children's emotional responding. Preadolescents (n = 99) were randomly assigned to complete the Trier Social Stress Test either with or without their pet dog. Children rated their positive and negative affect, and high frequency heart rate variability (HF-HRV) was assessed throughout the session. Children reported higher positive affect when they completed the task with their pet dog, although there were no differences for negative affect or HF-HRV. Children who had more physical contact with their dog at baseline reported higher positive affect. The findings suggest contact with pets is associated with enhanced positive affect. |
  | Date | 2018-02 |
  | Language | eng |
  | Short Title | Pet Dogs |
  | Library Catalogue | PubMed |
  | Volume | 27 |
  | Pages | 34-44 |
  | Publication | Social Development (Oxford, England) |
  | DOI | 10.1111/sode.12246 |
  | Issue | 1 |
  | Journal Abbr | Soc Dev |
  | ISSN | 0961-205X |
  | PMID | 29379226 |
  | PMCID | PMC5785939 |
  | Date Added | 11/07/2025, 11:37:24 |
  | Modified | 11/07/2025, 11:37:24 |

  ### Attachments

  - Accepted Version
  - PubMed entry
- ## Positive Reinforcement Training for Blood Collection in Grizzly Bears (Ursus arctos horribilis) Results in Undetectable Elevations in Serum Cortisol Levels: A Preliminary Investigation

  |  |  |
  | --- | --- |
  | Item Type | Journal Article |
  | Author | Nicole M. Joyce-Zuniga |
  | Author | Ruth C. Newberry |
  | Author | Charles T. Robbins |
  | Author | Jasmine V. Ware |
  | Author | Heiko T. Jansen |
  | Author | O. Lynne Nelson |
  | Abstract | Training nonhuman animals in captivity for participation in routine husbandry procedures is believed to produce a lower stress environment compared with undergoing a general anesthetic event for the same procedure. This hypothesis rests largely on anecdotal evidence that the captive subjects appear more relaxed with the trained event. Blood markers of physiological stress responses were evaluated in 4 captive grizzly bears (Ursus arctos horribilis) who were clicker-trained for blood collection versus 4 bears who were chemically immobilized for blood collection. Serum cortisol and immunoglobulin A (IgA) and plasma β-endorphin were measured as indicators of responses to stress. Plasma β-endorphin was not different between the groups. Serum IgA was undetectable in all bears. Serum cortisol was undetectable in all trained bears, whereas chemically immobilized bears had marked cortisol elevations (p < .05). The highest cortisol elevations were found in 2 bears with extensive recent immobilization experience. These findings support the use of positive reinforcement training for routine health procedures to minimize anxiety. |
  | Date | 2016 |
  | Language | eng |
  | Short Title | Positive Reinforcement Training for Blood Collection in Grizzly Bears (Ursus arctos horribilis) Results in Undetectable Elevations in Serum Cortisol Levels |
  | Library Catalogue | PubMed |
  | Volume | 19 |
  | Pages | 210-215 |
  | Publication | Journal of applied animal welfare science: JAAWS |
  | DOI | 10.1080/10888705.2015.1126523 |
  | Issue | 2 |
  | Journal Abbr | J Appl Anim Welf Sci |
  | ISSN | 1532-7604 |
  | PMID | 26847149 |
  | Date Added | 11/07/2025, 14:21:52 |
  | Modified | 11/07/2025, 14:21:52 |

  ### Tags:

  - Hydrocortisone
  - human–animal interaction
  - stress
  - Animals
  - Female
  - Male
  - Stress, Psychological
  - anxiety
  - blood sampling
  - Animal welfare
  - beta-Endorphin
  - Blood Specimen Collection
  - chemical immobilization
  - Immobilization
  - Immunoglobulin A
  - Reinforcement, Psychology
  - Ursidae

  ### Attachments

  - PubMed entry
- ## Can dogs serve as stress mediators to decrease salivary cortisol levels in a population of liberal arts college undergraduate students?

  |  |  |
  | --- | --- |
  | Item Type | Journal Article |
  | Author | Ana Gabriela Jimenez |
  | Author | Luke Calderaro |
  | Author | Sophia Clark |
  | Author | David Elacqua |
  | Author | Emily Hazen |
  | Author | Vanessa Lam |
  | Author | Grace S. Leightheiser |
  | Abstract | The steroid hormone cortisol can be used to measure physiological stress in humans. The hypothalamic-pituitary-adrenal (HPA) axis synthesizes cortisol, and a negative feedback cycle regulates cortisol depending on an individual's stress level and/or circadian rhythm. Chronic stress of college undergraduate students is associated with various adverse health effects, including anxiety and depression. Reports suggest that stress levels have risen dramatically in recent years, particularly among university students dealing with intense academic loads in addition to COVID-19 pandemic-related uncertainty. The increasing rate of mental illness on college campuses necessitates the study of mediators potentially capable of lowering stress, and thus cortisol levels. Research on mediation techniques and coping mechanisms have gained traction to address the concerning levels of stress, including the employment of human-animal interaction sessions on college campuses. In this study, human-canine interaction as a stress mediation strategy for undergraduate students was investigated. We measured salivary cortisol levels in 73 college undergraduate students during a 60-min interaction period with a dog to determine whether human-canine interactions are effective in lowering cortisol levels and potentially reducing chronic stress typical of undergraduate students. Our results indicate that a human-canine interaction for 60 min is an effective method for significantly reducing salivary cortisol and stress levels among undergraduate college students. These findings support the expansion of animal visitation programs on college campuses to help students manage stress. |
  | Date | 2023 |
  | Language | eng |
  | Library Catalogue | PubMed |
  | Volume | 19 |
  | Pages | 283-289 |
  | Publication | Explore (New York, N.Y.) |
  | DOI | 10.1016/j.explore.2022.08.007 |
  | Issue | 3 |
  | Journal Abbr | Explore (NY) |
  | ISSN | 1878-7541 |
  | PMID | 35989236 |
  | Date Added | 11/07/2025, 14:21:52 |
  | Modified | 11/07/2025, 14:21:52 |

  ### Tags:

  - Hydrocortisone
  - Dogs
  - COVID-19
  - Animals
  - Humans
  - Stress, Psychological
  - Saliva
  - Pandemics
  - Students

  ### Attachments

  - PubMed entry
- ## Physiological Effect of Gentle Stroking in Lambs

  |  |  |
  | --- | --- |
  | Item Type | Journal Article |
  | Author | Kamila Janicka |
  | Author | Patrycja Masier |
  | Author | Paulina Nazar |
  | Author | Patrycja Staniszewska |
  | Author | Grzegorz Zięba |
  | Author | Aneta Strachecka |
  | Author | Iwona Rozempolska-Rucińska |
  | Abstract | The aim of the study was to determine changes in physiological indicators in lambs subjected to gentle stroking. The study included 40 three-week-old lambs (20 females of the synthetic prolific-meat (BCP) line and 20 females of the Świniarka (SW) breed). The animals were assigned to two control groups (n = 20) and two experimental groups (n = 20). Lambs from the experimental groups received treatment. Analyses consisted of determining the following parameters: heart rate (HR), saturation (S) and biochemical analysis. In the groups of sheep subjected to gentle massage, the analyses revealed a significant increase in the levels of cortisol, CAT, GST, GPx, among others, and a significant decrease in the levels of total protein, SOD, TAC, uric acid and Na+. At the same time, indicators of reduced stress levels were revealed, i.e., a significant increase in glycogen levels and a significant decrease in glucose and HR values. These results suggest that the introduction of gentle touch can induce positive states in lambs, and that the stress response may be transient and adaptive. Nevertheless, it is important to note that these interactions can be a source of stress, even if the behavioral response does not necessarily indicate stress. |
  | Date | 2024-03-13 |
  | Language | eng |
  | Library Catalogue | PubMed |
  | Volume | 14 |
  | Pages | 887 |
  | Publication | Animals: an open access journal from MDPI |
  | DOI | 10.3390/ani14060887 |
  | Issue | 6 |
  | Journal Abbr | Animals (Basel) |
  | ISSN | 2076-2615 |
  | PMID | 38539984 |
  | PMCID | PMC10967445 |
  | Date Added | 11/07/2025, 11:37:24 |
  | Modified | 11/07/2025, 11:37:24 |

  ### Tags:

  - human-animal interaction
  - lamb
  - oxidative stress
  - physiology
  - stroking

  ### Attachments

  - Full Text
  - PubMed entry
- ## Physiological Effect of Gentle Stroking in Lambs

  |  |  |
  | --- | --- |
  | Item Type | Journal Article |
  | Author | Kamila Janicka |
  | Author | Patrycja Masier |
  | Author | Paulina Nazar |
  | Author | Patrycja Staniszewska |
  | Author | Grzegorz Zięba |
  | Author | Aneta Strachecka |
  | Author | Iwona Rozempolska-Rucińska |
  | Abstract | The aim of the study was to determine changes in physiological indicators in lambs subjected to gentle stroking. The study included 40 three-week-old lambs (20 females of the synthetic prolific-meat (BCP) line and 20 females of the Świniarka (SW) breed). The animals were assigned to two control groups (n = 20) and two experimental groups (n = 20). Lambs from the experimental groups received treatment. Analyses consisted of determining the following parameters: heart rate (HR), saturation (S) and biochemical analysis. In the groups of sheep subjected to gentle massage, the analyses revealed a significant increase in the levels of cortisol, CAT, GST, GPx, among others, and a significant decrease in the levels of total protein, SOD, TAC, uric acid and Na+. At the same time, indicators of reduced stress levels were revealed, i.e., a significant increase in glycogen levels and a significant decrease in glucose and HR values. These results suggest that the introduction of gentle touch can induce positive states in lambs, and that the stress response may be transient and adaptive. Nevertheless, it is important to note that these interactions can be a source of stress, even if the behavioral response does not necessarily indicate stress. |
  | Date | 2024-03-13 |
  | Language | eng |
  | Library Catalogue | PubMed |
  | Volume | 14 |
  | Pages | 887 |
  | Publication | Animals: an open access journal from MDPI |
  | DOI | 10.3390/ani14060887 |
  | Issue | 6 |
  | Journal Abbr | Animals (Basel) |
  | ISSN | 2076-2615 |
  | PMID | 38539984 |
  | PMCID | PMC10967445 |
  | Date Added | 11/07/2025, 14:21:52 |
  | Modified | 11/07/2025, 14:21:52 |

  ### Tags:

  - human-animal interaction
  - lamb
  - oxidative stress
  - physiology
  - stroking

  ### Attachments

  - Full Text
  - PubMed entry
- ## Atypical experiences of captive chimpanzees (Pan troglodytes) are associated with higher hair cortisol concentrations as adults

  |  |  |
  | --- | --- |
  | Item Type | Journal Article |
  | Author | S. L. Jacobson |
  | Author | H. D. Freeman |
  | Author | R. M. Santymire |
  | Author | S. R. Ross |
  | Abstract | Experiences during early development are influential on the lives of human and non-human primates into adulthood. The population of captive chimpanzees in the USA can provide insight into this relationship, as collectively they have experienced a wide range of exposure to both conspecifics (those raised in natal groups) and humans (those raised as personal pets or performers). Our study investigated chimpanzee exposure to humans using a continuous measure of categorization, the chimpanzee-human interaction index, and the relationship between this experience and cortisol concentrations in adulthood. Historical records and hair samples were collected from 60 chimpanzees which were socially housed in 13 zoos and sanctuaries. We found that more human exposure throughout the life of a chimpanzee was associated with higher hair cortisol concentrations in adulthood. Sex was also a significant factor affecting cortisol concentration, with male chimpanzees having higher cortisol concentrations than female chimpanzees. These results build upon the extensive literature about aversive effects of atypical social histories for chimpanzees and emphasize to managers the importance of monitoring potential negative health consequences and social deficits these individuals may exhibit. |
  | Date | 2017-12 |
  | Language | eng |
  | Library Catalogue | PubMed |
  | Volume | 4 |
  | Pages | 170932 |
  | Publication | Royal Society Open Science |
  | DOI | 10.1098/rsos.170932 |
  | Issue | 12 |
  | Journal Abbr | R Soc Open Sci |
  | ISSN | 2054-5703 |
  | PMID | 29308234 |
  | PMCID | PMC5750001 |
  | Date Added | 11/07/2025, 14:21:52 |
  | Modified | 11/07/2025, 14:21:52 |

  ### Tags:

  - cortisol
  - human–animal interaction
  - animal welfare
  - chimpanzee
  - atypical rearing
  - development

  ### Attachments

  - Full Text PDF
  - PubMed entry
- ## Exploring the Dynamics of Canine-Assisted Interactions: A Wearable Approach to Understanding Interspecies Well-Being

  |  |  |
  | --- | --- |
  | Item Type | Journal Article |
  | Author | Timothy R. N. Holder |
  | Author | Colt Nichols |
  | Author | Emily Summers |
  | Author | David L. Roberts |
  | Author | Alper Bozkurt |
  | Abstract | Canine-assisted interactions (CAIs) have been explored to offer therapeutic benefits to human participants in various contexts, from addressing cancer-related fatigue to treating post-traumatic stress disorder. Despite their widespread adoption, there are still unresolved questions regarding the outcomes for both humans and animals involved in these interactions. Previous attempts to address these questions have suffered from core methodological weaknesses, especially due to absence of tools for an efficient objective evaluation and lack of focus on the canine perspective. In this article, we present a first-of-its-kind system and study to collect simultaneous and continuous physiological data from both of the CAI interactants. Motivated by our extensive field reviews and stakeholder feedback, this comprehensive wearable system is composed of custom-designed and commercially available sensor devices. We performed a repeated-measures pilot study, to combine data collected via this system with a novel dyadic behavioral coding method and short- and long-term surveys. We evaluated these multimodal data streams independently, and we further correlated the psychological, physiological, and behavioral metrics to better elucidate the outcomes and dynamics of CAIs. Confirming previous field results, human electrodermal activity is the measure most strongly distinguished between the dyads' non-interaction and interaction periods. Valence, arousal, and the positive affect of the human participant significantly increased during interaction with the canine participant. Also, we observed in our pilot study that (a) the canine heart rate was more dynamic than the human's during interactions, (b) the surveys proved to be the best indicator of the subjects' affective state, and (c) the behavior coding approaches best tracked the bond quality between the interacting dyads. Notably, we found that most of the interaction sessions were characterized by extended neutral periods with some positive and negative peaks, where the bonded pairs might display decreased behavioral synchrony. We also present three new representations of the internal and overall dynamics of CAIs for adoption by the broader field. Lastly, this paper discusses ongoing options for further dyadic analysis, interspecies emotion prediction, integration of contextually relevant environmental data, and standardization of human-animal interaction equipment and analytical approaches. Altogether, this work takes a significant step forward on a promising path to our better understanding of how CAIs improve well-being and how interspecies psychophysiological states can be appropriately measured. |
  | Date | 2024-12-16 |
  | Language | eng |
  | Short Title | Exploring the Dynamics of Canine-Assisted Interactions |
  | Library Catalogue | PubMed |
  | Volume | 14 |
  | Pages | 3628 |
  | Publication | Animals: an open access journal from MDPI |
  | DOI | 10.3390/ani14243628 |
  | Issue | 24 |
  | Journal Abbr | Animals (Basel) |
  | ISSN | 2076-2615 |
  | PMID | 39765532 |
  | PMCID | PMC11672835 |
  | Date Added | 11/07/2025, 11:37:24 |
  | Modified | 11/07/2025, 11:37:24 |

  ### Tags:

  - canine-assisted interactions
  - psychophysiology sensors
  - wearable systems

  ### Attachments

  - Full Text PDF
  - PubMed entry
- ## Relationships between handling, behaviour and stress in lambs at abattoirs

  |  |  |
  | --- | --- |
  | Item Type | Journal Article |
  | Author | P. H. Hemsworth |
  | Author | M. Rice |
  | Author | S. Borg |
  | Author | L. E. Edwards |
  | Author | E. N. Ponnampalam |
  | Author | G. J. Coleman |
  | Abstract | There is community concern about the treatment of farm animals post-farm gate, particularly animal transport and slaughter. Relationships between lamb behavioural and physiological variables on farm, stockperson, dog and lamb behavioural variables pre-slaughter and plasma cortisol, glucose and lactate in lambs post-slaughter were studied in 400 lambs. The lambs were observed in three behavioural tests, novel arena, flight distance to a human and temperament tests, before transport for slaughter. Closed-circuit television video footage was used to record stockperson, dog and lamb behaviour immediately before slaughter. Blood samples for cortisol, glucose and lactate analyses were collected on farm following the three behavioural tests and immediately post-slaughter. The regression models that best predicted plasma cortisol, glucose and lactate concentrations post-slaughter included a mixture of stockperson and dog behavioural variables as well as lamb variables both on-farm and pre-slaughter. These regression models accounted for 33%, 34% and 44% of the variance in plasma cortisol, glucose and lactate concentrations post-slaughter, respectively. Some of the stockperson and dog behaviours pre-slaughter that were predictive of the stress and metabolic variables post-slaughter included the duration of negative stockperson behaviours such as fast locomotion and lifting/pulling lambs, and the duration of dog behaviours such as lunging and barking at the lamb, while some of the predictive lamb behaviour variables included the durations of jumping and fleeing. Some of the physiological and behavioural responses to the behavioural tests on farm were also predictive of the stress and metabolic variables post-slaughter. These relationships support the well-demonstrated effect of handling on fear and stress responses in livestock, and although not direct evidence of causal relationships, highlight the potential benefits of training stockpeople to reduce fear and stress in sheep at abattoirs. |
  | Date | 2019-06 |
  | Language | eng |
  | Library Catalogue | PubMed |
  | Volume | 13 |
  | Pages | 1287-1296 |
  | Publication | Animal: An International Journal of Animal Bioscience |
  | DOI | 10.1017/S1751731118002744 |
  | Issue | 6 |
  | Journal Abbr | Animal |
  | ISSN | 1751-732X |
  | PMID | 30345951 |
  | Date Added | 11/07/2025, 14:21:52 |
  | Modified | 11/07/2025, 14:21:52 |

  ### Tags:

  - Hydrocortisone
  - human–animal interaction
  - Animals
  - Behavior, Animal
  - Animal Husbandry
  - physiology
  - Stress, Physiological
  - Victoria
  - Abattoirs
  - Blood Glucose
  - Lactic Acid
  - sheep
  - Sheep, Domestic
  - slaughter
  - stockperson

  ### Attachments

  - PubMed entry
- ## The Influence of Brief Outing and Temporary Fostering Programs on Shelter Dog Welfare

  |  |  |
  | --- | --- |
  | Item Type | Journal Article |
  | Author | Lisa M. Gunter |
  | Author | Emily M. Blade |
  | Author | Rachel J. Gilchrist |
  | Author | Betsy J. Nixon |
  | Author | Jenifer L. Reed |
  | Author | Joanna M. Platzer |
  | Author | Ingrid C. Wurpts |
  | Author | Erica N. Feuerbacher |
  | Author | Clive D. L. Wynne |
  | Abstract | Human interaction is one of the most consistently effective interventions that can improve the welfare of shelter-living dogs. Time out of the kennel with a person has been shown to reduce physiological measures of stress as can leaving the shelter for a night or more in a foster home. In this study, we assessed the effects of brief outings and temporary fostering stays on dogs' length of stay and outcomes. In total, we analyzed data of 1955 dogs from 51 animal shelters that received these interventions as well as 25,946 dogs residing at these shelters that served as our controls. We found that brief outings and temporary fostering stays increased dogs' likelihood of adoption by 5.0 and 14.3 times, respectively. While their lengths of stay were longer in comparison to control dogs, this difference was present prior to the intervention. Additionally, we found that these programs were more successful when greater percentages of community members (as compared to volunteers and staff) were involved in caregiving as well as when programs were implemented by better-resourced shelters. As such, animal welfare organizations should consider implementing these fostering programs as evidence-based best practices that can positively impact the outcomes of shelter dogs. |
  | Date | 2023-11-15 |
  | Language | eng |
  | Library Catalogue | PubMed |
  | Volume | 13 |
  | Pages | 3528 |
  | Publication | Animals: an open access journal from MDPI |
  | DOI | 10.3390/ani13223528 |
  | Issue | 22 |
  | Journal Abbr | Animals (Basel) |
  | ISSN | 2076-2615 |
  | PMID | 38003145 |
  | PMCID | PMC10668817 |
  | Date Added | 11/07/2025, 11:33:21 |
  | Modified | 11/07/2025, 11:33:21 |

  ### Tags:

  - adoption
  - animal shelter
  - dogs
  - human–animal interaction
  - welfare

  ### Attachments

  - Full Text
  - PubMed entry
- ## Investigating the Impact of Brief Outings on the Welfare of Dogs Living in US Shelters

  |  |  |
  | --- | --- |
  | Item Type | Journal Article |
  | Author | Lisa M. Gunter |
  | Author | Rachel J. Gilchrist |
  | Author | Emily M. Blade |
  | Author | Rebecca T. Barber |
  | Author | Erica N. Feuerbacher |
  | Author | JoAnna M. Platzer |
  | Author | Clive D. L. Wynne |
  | Abstract | Social isolation likely contributes to reduced welfare for shelter-living dogs. Several studies have established that time out of the kennel with a person can improve dogs' behavior and reduce physiological measures of stress. This study assessed the effects of two-and-a-half-hour outings on the urinary cortisol levels and activity of dogs as they awaited adoption at four animal shelters. Dogs' urine was collected before and after outings for cortisol:creatinine analysis, and accelerometer devices were used to measure dogs' physical activity. In total, 164 dogs participated in this study, with 793 cortisol values and 3750 activity measures used in the statistical analyses. We found that dogs' cortisol:creatinine ratios were significantly higher during the afternoon of the intervention but returned to pre-field trip levels the following day. Dogs' minutes of low activity were significantly reduced, and high activity significantly increased during the outing. Although dogs' cortisol and activity returned to baseline after the intervention, our findings suggest that short-term outings do not confer the same stress reduction benefits as previously shown with temporary fostering. Nevertheless, it is possible that these types of outing programs are beneficial to adoptions by increasing the visibility of dogs and should be further investigated to elucidate these effects. |
  | Date | 2021-02-19 |
  | Language | eng |
  | Library Catalogue | PubMed |
  | Volume | 11 |
  | Pages | 548 |
  | Publication | Animals: an open access journal from MDPI |
  | DOI | 10.3390/ani11020548 |
  | Issue | 2 |
  | Journal Abbr | Animals (Basel) |
  | ISSN | 2076-2615 |
  | PMID | 33669874 |
  | PMCID | PMC7923296 |
  | Date Added | 11/07/2025, 11:33:21 |
  | Modified | 11/07/2025, 11:33:21 |

  ### Tags:

  - cortisol
  - animal shelter
  - dogs
  - welfare
  - activity
  - human-animal interaction
  - stress

  ### Attachments

  - Full Text
  - PubMed entry
- ## Investigating the Impact of Brief Outings on the Welfare of Dogs Living in US Shelters

  |  |  |
  | --- | --- |
  | Item Type | Journal Article |
  | Author | Lisa M. Gunter |
  | Author | Rachel J. Gilchrist |
  | Author | Emily M. Blade |
  | Author | Rebecca T. Barber |
  | Author | Erica N. Feuerbacher |
  | Author | JoAnna M. Platzer |
  | Author | Clive D. L. Wynne |
  | Abstract | Social isolation likely contributes to reduced welfare for shelter-living dogs. Several studies have established that time out of the kennel with a person can improve dogs' behavior and reduce physiological measures of stress. This study assessed the effects of two-and-a-half-hour outings on the urinary cortisol levels and activity of dogs as they awaited adoption at four animal shelters. Dogs' urine was collected before and after outings for cortisol:creatinine analysis, and accelerometer devices were used to measure dogs' physical activity. In total, 164 dogs participated in this study, with 793 cortisol values and 3750 activity measures used in the statistical analyses. We found that dogs' cortisol:creatinine ratios were significantly higher during the afternoon of the intervention but returned to pre-field trip levels the following day. Dogs' minutes of low activity were significantly reduced, and high activity significantly increased during the outing. Although dogs' cortisol and activity returned to baseline after the intervention, our findings suggest that short-term outings do not confer the same stress reduction benefits as previously shown with temporary fostering. Nevertheless, it is possible that these types of outing programs are beneficial to adoptions by increasing the visibility of dogs and should be further investigated to elucidate these effects. |
  | Date | 2021-02-19 |
  | Language | eng |
  | Library Catalogue | PubMed |
  | Volume | 11 |
  | Pages | 548 |
  | Publication | Animals: an open access journal from MDPI |
  | DOI | 10.3390/ani11020548 |
  | Issue | 2 |
  | Journal Abbr | Animals (Basel) |
  | ISSN | 2076-2615 |
  | PMID | 33669874 |
  | PMCID | PMC7923296 |
  | Date Added | 11/07/2025, 14:21:52 |
  | Modified | 11/07/2025, 14:21:52 |

  ### Tags:

  - cortisol
  - animal shelter
  - dogs
  - welfare
  - activity
  - human-animal interaction
  - stress

  ### Attachments

  - Full Text
  - PubMed entry
- ## Evaluating the effects of a temporary fostering program on shelter dog welfare

  |  |  |
  | --- | --- |
  | Item Type | Journal Article |
  | Author | Lisa M. Gunter |
  | Author | Erica N. Feuerbacher |
  | Author | Rachel J. Gilchrist |
  | Author | Clive D. L. Wynne |
  | Abstract | One of the greatest stressors for dogs living in animal shelters is social isolation. Many studies have demonstrated that human interaction reduces cortisol in shelter dogs, with the possibility that longer periods of interaction may yield greater effects. These types of interventions are contingent upon removing the dog from the kennel and any such reductions in cortisol are often lost when the dog returns to the kennel. More recently, animal shelters are utilizing short-term fostering programs to provide relief from the perceived stresses of kennel life; however the effects of these programs are not well understood. This study assessed the impacts of one- and two-night fostering programs on the urinary cortisol levels, resting pulse rates, longest bout of uninterrupted rest, and proportion of time spent resting of dogs awaiting adoption. Five animal shelters, open and limited-admission facilities, from across the United States participated in the study. During the study, dogs' urine was collected in the morning before, during, and after fostering stays for cortisol: creatinine analysis. Non-invasive health monitors were worn by the dogs, which collected heart rates and activity levels, in the shelter and in foster homes. In total, 207 dogs participated in the study, and 1,076 cortisol values were used in our analysis. Across all shelters, we found that dogs' cortisol: creatinine ratios dropped significantly during their fostering stay, but returned to baseline levels after return to the shelter. However, the observed reduction in cortisol varied in magnitude across shelters. We found that dogs of greater weight, age, and average resting pulse rate had higher cortisol levels; and dogs with longer bouts of uninterrupted rest had lower cortisol levels. Dogs had their longest bouts of rest during sleepovers, followed by in the shelter after their sleepovers. Lastly, significant differences were found when comparing in-shelter cortisol values at our five shelters, differences that were in some cases greater than the impact of the fostering intervention itself. Considering the diversity of facilities that participated in this study, it is possible that as yet unstudied, shelter-specific, environmental factors could be contributing to the overall welfare of shelter dogs. Thus while a reprieve from the shelter is impactful for dogs awaiting adoption, mitigating the stressors present in kenneling conditions should also be addressed to improve the lives of shelter dogs. |
  | Date | 2019 |
  | Language | eng |
  | Library Catalogue | PubMed |
  | Volume | 7 |
  | Pages | e6620 |
  | Publication | PeerJ |
  | DOI | 10.7717/peerj.6620 |
  | Journal Abbr | PeerJ |
  | ISSN | 2167-8359 |
  | PMID | 30944778 |
  | PMCID | PMC6441318 |
  | Date Added | 11/07/2025, 14:21:52 |
  | Modified | 11/07/2025, 14:21:52 |

  ### Tags:

  - Dogs
  - Human-animal interaction
  - Cortisol
  - Stress
  - Animal shelter
  - Enrichment
  - Welfare

  ### Attachments

  - Full Text
  - PubMed entry
- ## Effects of human-animal interaction on salivary and urinary oxytocin in children and dogs

  |  |  |
  | --- | --- |
  | Item Type | Journal Article |
  | Author | Gitanjali E. Gnanadesikan |
  | Author | Katherine M. King |
  | Author | Elizabeth Carranza |
  | Author | Abigail C. Flyer |
  | Author | Gianna Ossello |
  | Author | Paige G. Smith |
  | Author | Netzin G. Steklis |
  | Author | H. Dieter Steklis |
  | Author | C. Sue Carter |
  | Author | Jessica J. Connelly |
  | Author | Melissa Barnett |
  | Author | Nancy Gee |
  | Author | Stacey R. Tecot |
  | Author | Evan L. MacLean |
  | Abstract | Oxytocin pathways are hypothesized to play important roles in human-animal interactions and may contribute to some benefits of these interspecific social relationships. We explored the effects of naturalistic interactions between children and dogs on oxytocin release in both species, as well as associations between methylation of the oxytocin receptor gene (OXTRm), social behavior, and oxytocin response in this context. Children (N = 55) participated in a within-subjects design involving a) interaction with their pet dog, b) interaction with an unfamiliar dog, and c) a nonsocial control condition (solitary play). We used immunoassays to measure salivary and urinary oxytocin in both the children and dogs, behavioral coding to characterize dog-child interactions, and bisulfite sequencing to quantify methylation of the oxytocin receptor gene (N = 32 children). Child salivary oxytocin decreased moderately across time in all conditions, but the extent of this effect varied between conditions, with greater oxytocin output during interactions with dogs than the control condition. In the pet dog condition, children's salivary oxytocin response was positively associated with the duration of visual co-orientation between the child and dog. Child urinary oxytocin did not deviate substantially from baseline in any condition. Children with higher levels of OXTRm had greater oxytocin output during interactions with their pet dogs, but lower oxytocin output in the control condition, and engaged in lower levels of affectionate interaction with dogs across conditions. Children's pet dogs exhibited increases in salivary oxytocin, but we observed the opposite pattern in the unfamiliar dog, who exhibited decreases in both urinary and salivary oxytocin on average. Collectively, our results support the hypothesis that oxytocin pathways may shape and respond to social interactions between children and dogs, highlighting an important role for companion animals in child development. |
  | Date | 2024-11 |
  | Language | eng |
  | Library Catalogue | PubMed |
  | Volume | 169 |
  | Pages | 107147 |
  | Publication | Psychoneuroendocrinology |
  | DOI | 10.1016/j.psyneuen.2024.107147 |
  | Journal Abbr | Psychoneuroendocrinology |
  | ISSN | 1873-3360 |
  | PMID | 39094516 |
  | PMCID | PMC11381145 |
  | Date Added | 11/07/2025, 11:45:53 |
  | Modified | 11/07/2025, 11:45:56 |

  ### Tags:

  - Oxytocin
  - Dogs
  - Animals
  - Female
  - Human-Animal Bond
  - Human-Animal Interaction
  - Humans
  - Male
  - Saliva
  - Social Behavior
  - Child
  - Child, Preschool
  - Receptors, Oxytocin

  ### Attachments

  - PubMed entry
- ## Glucocorticoid response to naturalistic interactions between children and dogs

  |  |  |
  | --- | --- |
  | Item Type | Journal Article |
  | Author | Gitanjali E. Gnanadesikan |
  | Author | Elizabeth Carranza |
  | Author | Katherine M. King |
  | Author | Abigail C. Flyer |
  | Author | Gianna Ossello |
  | Author | Paige G. Smith |
  | Author | Netzin G. Steklis |
  | Author | H. Dieter Steklis |
  | Author | Jessica J. Connelly |
  | Author | Melissa Barnett |
  | Author | Nancy Gee |
  | Author | Stacey Tecot |
  | Author | Evan L. MacLean |
  | Abstract | Although research has shown that pets appear to provide certain types of social support to children, little is known about the physiological bases of these effects, especially in naturalistic contexts. In this study, we investigated the effect of free-form interactions between children (ages 8-10 years) and dogs on salivary cortisol concentrations in both species. We further investigated the role of the child-dog relationship by comparing interactions with the child's pet dog to interactions with an unfamiliar dog or a nonsocial control condition, and modeled associations between survey measures of the human-animal bond and children's physiological responses. In both children and dogs, salivary cortisol decreased from pre- to post-interaction; the effect was strongest for children interacting with an unfamiliar dog (compared to their pet dog) and for the pet dogs (compared to the unfamiliar dog). We found minimal evidence for associations between cortisol output and behaviors coded from video, but children scoring higher on survey measures of the human-animal bond exhibited the greatest reductions in cortisol when interacting with dogs. Self-reported loneliness was not related to cortisol or the human-animal bond, but measures of both loneliness and the human-animal bond were higher among children who participated after the onset of the COVID-19 pandemic, relative to those who participated before the pandemic. This study builds on previous work that investigated potential stress-buffering effects of human-animal interaction during explicit stressors and demonstrates important physiological correlates of naturalistic interactions between children and dogs, similar to those that occur in daily life. |
  | Date | 2024-05 |
  | Language | eng |
  | Library Catalogue | PubMed |
  | Volume | 161 |
  | Pages | 105523 |
  | Publication | Hormones and Behavior |
  | DOI | 10.1016/j.yhbeh.2024.105523 |
  | Journal Abbr | Horm Behav |
  | ISSN | 1095-6867 |
  | PMID | 38484567 |
  | PMCID | PMC11065563 |
  | Date Added | 11/07/2025, 14:21:52 |
  | Modified | 11/07/2025, 14:21:52 |

  ### Tags:

  - Hydrocortisone
  - Dogs
  - Pets
  - COVID-19
  - Animals
  - Female
  - Human-Animal Bond
  - Human-Animal Interaction
  - Humans
  - Male
  - Human-animal interaction
  - Saliva
  - Cortisol
  - Child
  - Dog
  - Stress
  - Glucocorticoids
  - Human-animal bond
  - Loneliness
  - Social support

  ### Attachments

  - PubMed entry
- ## Therapy Dog Welfare Revisited: A Review of the Literature

  |  |  |
  | --- | --- |
  | Item Type | Journal Article |
  | Author | Lisa Maria Glenk |
  | Author | Sandra Foltin |
  | Abstract | During the past decade, the field of human-animal interaction(s) research has been characterized by a significant increase in scientific findings. These data have contributed to our current understanding of how humans may benefit from contact with animals. However, the animal experience of these interactions is still an under-researched area. This paper addresses the welfare of dogs who participate in animal-assisted interventions (AAIs) to improve health in human recipients. This paper builds on previous work by Glenk (2017) and provides an updated review of the literature on therapy dog welfare published from 2017-2021. New advances in scientific methodology, such as the determination of salivary oxytocin, breath rate and tympanic membrane temperature, are analyzed regarding their value and limitations for research in AAIs. Moreover, welfare-related social and environmental factors (e.g., freedom of choice, exploration of novel environments, inequity aversion, individual development, working experience, relationship with handler and handler skills) that profoundly influence dog perception and well-being are reviewed and discussed. Accounting for the globally increasing interest and the number of dogs utilized in AAIs, safeguarding therapy dog well-being, and identifying situations, circumstances and protocols that may challenge animal welfare remains an emerging and crucial area of scientific effort. |
  | Date | 2021-10-12 |
  | Language | eng |
  | Short Title | Therapy Dog Welfare Revisited |
  | Library Catalogue | PubMed |
  | Volume | 8 |
  | Pages | 226 |
  | Publication | Veterinary Sciences |
  | DOI | 10.3390/vetsci8100226 |
  | Issue | 10 |
  | Journal Abbr | Vet Sci |
  | ISSN | 2306-7381 |
  | PMID | 34679056 |
  | PMCID | PMC8538106 |
  | Date Added | 11/07/2025, 11:45:53 |
  | Modified | 11/07/2025, 11:45:57 |

  ### Tags:

  - welfare
  - stress
  - dog
  - animal-assisted intervention
  - canine
  - therapy

  ### Attachments

  - Full Text
  - PubMed entry
- ## Therapy Dog Welfare Revisited: A Review of the Literature

  |  |  |
  | --- | --- |
  | Item Type | Journal Article |
  | Author | Lisa Maria Glenk |
  | Author | Sandra Foltin |
  | Abstract | During the past decade, the field of human-animal interaction(s) research has been characterized by a significant increase in scientific findings. These data have contributed to our current understanding of how humans may benefit from contact with animals. However, the animal experience of these interactions is still an under-researched area. This paper addresses the welfare of dogs who participate in animal-assisted interventions (AAIs) to improve health in human recipients. This paper builds on previous work by Glenk (2017) and provides an updated review of the literature on therapy dog welfare published from 2017-2021. New advances in scientific methodology, such as the determination of salivary oxytocin, breath rate and tympanic membrane temperature, are analyzed regarding their value and limitations for research in AAIs. Moreover, welfare-related social and environmental factors (e.g., freedom of choice, exploration of novel environments, inequity aversion, individual development, working experience, relationship with handler and handler skills) that profoundly influence dog perception and well-being are reviewed and discussed. Accounting for the globally increasing interest and the number of dogs utilized in AAIs, safeguarding therapy dog well-being, and identifying situations, circumstances and protocols that may challenge animal welfare remains an emerging and crucial area of scientific effort. |
  | Date | 2021-10-12 |
  | Language | eng |
  | Short Title | Therapy Dog Welfare Revisited |
  | Library Catalogue | PubMed |
  | Volume | 8 |
  | Pages | 226 |
  | Publication | Veterinary Sciences |
  | DOI | 10.3390/vetsci8100226 |
  | Issue | 10 |
  | Journal Abbr | Vet Sci |
  | ISSN | 2306-7381 |
  | PMID | 34679056 |
  | PMCID | PMC8538106 |
  | Date Added | 11/07/2025, 14:23:27 |
  | Modified | 11/07/2025, 14:23:27 |

  ### Tags:

  - welfare
  - stress
  - dog
  - animal-assisted intervention
  - canine
  - therapy

  ### Attachments

  - Full Text
  - PubMed entry
- ## Visitor effects on the welfare of captive Sumatran orangutans (Pongo abelii) during the pandemic lockdowns

  |  |  |
  | --- | --- |
  | Item Type | Journal Article |
  | Author | Ezekiel F. Gading |
  | Author | Valerie Am Schoof |
  | Author | Maria Franke |
  | Author | Suzanne E. MacDonald |
  | Abstract | The COVID-19 pandemic led to unprecedented lockdowns with rippling impacts on the lives of humans and animals alike. Since zoos were among the first institutions to close during the pandemic, the lockdowns presented the opportunity to conduct a natural experiment examining the relationship between visitor presence and the welfare of zoo-housed animals. In this study, we assessed the welfare of six Sumatran orangutans (Pongo abelii) at Toronto Zoo both during and following the pandemic lockdowns. We compared behavioural and physiological indicators of welfare during a lockdown and after visitors were reintroduced. Specifically, if the orangutans' welfare was affected by the visitor re-introduction phase we predicted there would be an increase in the following measures: (1) use of exhibit areas away from visitors; (2) behavioural measures (hiding, self-directed behaviours, agonistic behaviours, agitated movement, and idiosyncratic object-directed behaviours [head slamming, and fabric tearing]); and (3) physiological measures (faecal consistency and glucocorticoid metabolites) when compared to the lockdown. We also measured changes in activity levels such as foraging and inactivity. We found that orangutan exhibit space use did not change when visitors were reintroduced. In fact, the orangutans hid less when visitors were introduced than during the lockdown. Foraging, inactivity, and other behavioural indicators of stress did not change when visitors were introduced. Similarly, neither faecal consistency nor glucocorticoid metabolites changed across the study phases. Our data show that visitor re-introduction did not negatively affect the welfare of the Toronto Zoo orangutans. However, the presence of keepers was found to affect the behaviour of the orangutans and warrants further study. |
  | Date | 2025 |
  | Language | eng |
  | Library Catalogue | PubMed |
  | Volume | 34 |
  | Pages | e15 |
  | Publication | Animal Welfare (South Mimms, England) |
  | DOI | 10.1017/awf.2025.9 |
  | Journal Abbr | Anim Welf |
  | ISSN | 2054-1538 |
  | PMID | 40071106 |
  | PMCID | PMC11894407 |
  | Date Added | 11/07/2025, 11:33:21 |
  | Modified | 04/01/2026, 11:26:06 |

  ### Tags:

  - human-animal interaction
  - stress
  - animal welfare
  - behaviour
  - COVID-19
  - primates
  - agitation
  - animal behavior
  - animal experiment
  - article
  - Canada
  - coronavirus disease 2019
  - defecation
  - female
  - foraging
  - glucocorticoid
  - lockdown
  - locomotion
  - male
  - metabolite
  - nonhuman
  - orangutan
  - pandemic
  - physiological stress
  - play
  - Pongo abelii
  - primate
  - social interaction
  - zoo animal

  ### Notes:

  - Cited by: 0; All Open Access, Gold Open Access

  ### Attachments

  - Full Text (HTML)
  - PubMed entry
- ## Human physiological responses to different types of human-dog interactions: A randomised crossover study

  |  |  |
  | --- | --- |
  | Item Type | Journal Article |
  | Author | Lene Høeg Fuglsang-Damgaard |
  | Author | Sigrid Juhl Lunde |
  | Author | Janne Winther Christensen |
  | Author | Lene Vase |
  | Author | Poul B. Videbech |
  | Author | Karen Thodberg |
  | Abstract | Previous studies of human-dog interventions vary in terms of type of interaction, which is rarely quantified, leading to contradictory findings and limited comparability. To uncover the influence of different types of interactions, the present study investigated if it was possible to detect differences in immediate physiological measurements of healthy humans during different standardised types of interaction with a dog. Thirty-three healthy participants (women = 25, men = 8, >18 years) were exposed to four different test situations with standardised types of interaction intensity with a dog in random order: no dog present (CONTROL), looking at a dog (VISUAL), petting a dog (TACTILE) or performing tricks with a dog (ACTIVE). Each test situation lasted 10 min with a 30-min break between each. Heart rate (HR), heart rate variability (HRV) and skin conductance (tonic level (SCL) and peak counts (SCR)) were continuously recorded. Blood pressure (BP) and salivary cortisol (s-cortisol) were measured before and after each test situation. Linear Mixed Models were applied. HR, HRV, BP, SCL and SCR increased with increased interaction with the dog (for all: p < 0.001). HRV increased with decreased HR (p = 0.002), increased SCL (p = 0.027), and SCR (p < 0.001) depending on the type of interaction. Generally, s-cortisol increased with increased HR (p = 0.042), SCL increased with increased SCR (p < 0.001), and SCR increased with increased HRV (p = 0.013), depending on type of interaction. The physiological measurements HR, HRV, BP, SCL and SCR are influenced by different types of dog interaction, and thus it is important to quantify and report the type of interaction in human-dog interaction studies. (ClinicalTrials.gov ID:NCT04696419). |
  | Date | 2024-11 |
  | Language | eng |
  | Short Title | Human physiological responses to different types of human-dog interactions |
  | Library Catalogue | PubMed |
  | Volume | 57 |
  | Pages | 101899 |
  | Publication | Complementary Therapies in Clinical Practice |
  | DOI | 10.1016/j.ctcp.2024.101899 |
  | Journal Abbr | Complement Ther Clin Pract |
  | ISSN | 1873-6947 |
  | PMID | 39217835 |
  | Date Added | 11/07/2025, 11:37:24 |
  | Modified | 11/07/2025, 11:37:24 |

  ### Tags:

  - Hydrocortisone
  - Dogs
  - Heart rate variability
  - Animals
  - Female
  - Adult
  - Heart Rate
  - Human-Animal Interaction
  - Humans
  - Male
  - Young Adult
  - Cross-Over Studies
  - Blood Pressure
  - Blood pressure
  - Human animal interaction
  - Saliva
  - Salivary cortisol
  - Skin conductance

  ### Attachments

  - PubMed entry
- ## Human physiological responses to different types of human-dog interactions: A randomised crossover study

  |  |  |
  | --- | --- |
  | Item Type | Journal Article |
  | Author | Lene Høeg Fuglsang-Damgaard |
  | Author | Sigrid Juhl Lunde |
  | Author | Janne Winther Christensen |
  | Author | Lene Vase |
  | Author | Poul B. Videbech |
  | Author | Karen Thodberg |
  | Abstract | Previous studies of human-dog interventions vary in terms of type of interaction, which is rarely quantified, leading to contradictory findings and limited comparability. To uncover the influence of different types of interactions, the present study investigated if it was possible to detect differences in immediate physiological measurements of healthy humans during different standardised types of interaction with a dog. Thirty-three healthy participants (women = 25, men = 8, >18 years) were exposed to four different test situations with standardised types of interaction intensity with a dog in random order: no dog present (CONTROL), looking at a dog (VISUAL), petting a dog (TACTILE) or performing tricks with a dog (ACTIVE). Each test situation lasted 10 min with a 30-min break between each. Heart rate (HR), heart rate variability (HRV) and skin conductance (tonic level (SCL) and peak counts (SCR)) were continuously recorded. Blood pressure (BP) and salivary cortisol (s-cortisol) were measured before and after each test situation. Linear Mixed Models were applied. HR, HRV, BP, SCL and SCR increased with increased interaction with the dog (for all: p < 0.001). HRV increased with decreased HR (p = 0.002), increased SCL (p = 0.027), and SCR (p < 0.001) depending on the type of interaction. Generally, s-cortisol increased with increased HR (p = 0.042), SCL increased with increased SCR (p < 0.001), and SCR increased with increased HRV (p = 0.013), depending on type of interaction. The physiological measurements HR, HRV, BP, SCL and SCR are influenced by different types of dog interaction, and thus it is important to quantify and report the type of interaction in human-dog interaction studies. (ClinicalTrials.gov ID:NCT04696419). |
  | Date | 2024-11 |
  | Language | eng |
  | Short Title | Human physiological responses to different types of human-dog interactions |
  | Library Catalogue | PubMed |
  | Volume | 57 |
  | Pages | 101899 |
  | Publication | Complementary Therapies in Clinical Practice |
  | DOI | 10.1016/j.ctcp.2024.101899 |
  | Journal Abbr | Complement Ther Clin Pract |
  | ISSN | 1873-6947 |
  | PMID | 39217835 |
  | Date Added | 11/07/2025, 14:21:52 |
  | Modified | 11/07/2025, 14:21:52 |

  ### Tags:

  - Hydrocortisone
  - Dogs
  - Heart rate variability
  - Animals
  - Female
  - Adult
  - Heart Rate
  - Human-Animal Interaction
  - Humans
  - Male
  - Young Adult
  - Cross-Over Studies
  - Blood Pressure
  - Blood pressure
  - Human animal interaction
  - Saliva
  - Salivary cortisol
  - Skin conductance

  ### Attachments

  - PubMed entry
- ## Fecal Cortisol Metabolites in Dairy Cows: A Cross-Sectional Exploration of Associations with Animal, Stockperson, and Farm Characteristics

  |  |  |
  | --- | --- |
  | Item Type | Journal Article |
  | Author | Asja Ebinghaus |
  | Author | Ute Knierim |
  | Author | Christel Simantke |
  | Author | Rupert Palme |
  | Author | Silvia Ivemeyer |
  | Abstract | To date, little is known about influences on cows' physiological stress levels on farms. The present study explored associations of fecal cortisol metabolite concentrations (FCM) with (1) farm factors including human-animal contact, (2) cows' fear behaviors towards humans, and (3) milk production and udder health, involving 25 dairy farms and repeated fecal samples (n = 2625) from 674 focal cows. Farm factors via interviews and observations, avoidance distance (AD) and qualitative behavior assessment (QBA) during a human-animal interaction were recorded. Milk yield and somatic cell scores (SCS) were calculated from milk recordings. Levels of FCMs were in general relatively low. No associations with AD and milk yield could be detected. Correlations between FCMs and QBA and SCS were significant, but on a low level. Against expectations, FCMs were higher, when the farm provided concentrates by hand and habituated heifers to milking, in part possibly due to reversed cause-effect relations. Decreased FCM levels were found on farms that did not separate diseased cows, possibly due to the avoidance of social stress following changes in group structure. Additionally, straw yards compared to raised cubicles and generous compared to suboptimal lying space were associated with decreased levels, underlining the importance of comfort around resting. Moreover, FCMs were decreased with increased human contact time per cow. The different associations detected in this study provide a basis for further experimental investigations that moreover might provide insights into causal relationships. |
  | Date | 2020-10-01 |
  | Language | eng |
  | Short Title | Fecal Cortisol Metabolites in Dairy Cows |
  | Library Catalogue | PubMed |
  | Volume | 10 |
  | Pages | 1787 |
  | Publication | Animals: an open access journal from MDPI |
  | DOI | 10.3390/ani10101787 |
  | Issue | 10 |
  | Journal Abbr | Animals (Basel) |
  | ISSN | 2076-2615 |
  | PMID | 33019694 |
  | PMCID | PMC7600853 |
  | Date Added | 11/07/2025, 14:21:52 |
  | Modified | 11/07/2025, 14:21:52 |

  ### Tags:

  - stress
  - human–animal relationship
  - dairy cows
  - cortisol metabolites

  ### Attachments

  - Full Text
  - PubMed entry
- ## The power of interspecific sociality: how humans provide social buffering for horses

  |  |  |
  | --- | --- |
  | Item Type | Journal Article |
  | Author | Alfredo Di Lucrezia |
  | Author | Anna Scandurra |
  | Author | Daria Lotito |
  | Author | Valeria Iervolino |
  | Author | Biagio D'Aniello |
  | Author | Vincenzo Mastellone |
  | Author | Pietro Lombardi |
  | Author | Claudia Pinelli |
  | Abstract | In this study, we assessed the interspecific "social buffering effect" of humans on horses, exploring how human presence influences stress responses in horses in an unfamiliar environment using the "isolation paradigm." We examined nine Haflinger horses under two counterbalanced conditions: with a passive human stranger (social condition) or alone (isolation condition). Stress responses were assessed through cortisol measurements, heart rate monitoring, and behavioral observations. While cortisol levels significantly increased in both conditions, with no notable differences before and after the tests, heart rate data revealed a different pattern. Results indicated that stress generally decreased in both scenarios, impacting heart rate. Initially, during the first five minutes, heart rate was significantly higher in the social condition compared to isolation, but this trend reversed in the following intervals, with heart rate significantly decreasing as interaction with the stranger increased. Positive interaction between time and stranger-directed behaviors suggested the stranger's influence on heart rate strengthened over time. Overall, these finding suggest that while cortisol data did not reflect a social buffering effect, other metrics indicated that human presence effectively reduced stress in horses after a brief adjustment period, supporting the hypothesis that horses can benefit from human presence during stress, after a short adaptation time. This study highlights the complex nature of stress responses in horses and the potential role of humans as social buffers in interspecific contexts. |
  | Date | 2025-03-12 |
  | Language | eng |
  | Short Title | The power of interspecific sociality |
  | Library Catalogue | PubMed |
  | Volume | 28 |
  | Pages | 20 |
  | Publication | Animal Cognition |
  | DOI | 10.1007/s10071-025-01942-5 |
  | Issue | 1 |
  | Journal Abbr | Anim Cogn |
  | ISSN | 1435-9456 |
  | PMID | 40072705 |
  | PMCID | PMC11903558 |
  | Date Added | 11/07/2025, 11:37:24 |
  | Modified | 11/07/2025, 11:37:24 |

  ### Tags:

  - Hydrocortisone
  - Heart rate
  - Animals
  - Behavior, Animal
  - Female
  - Heart Rate
  - Humans
  - Male
  - Stress, Psychological
  - Human-animal interaction
  - Saliva
  - Horses
  - Cortisol
  - Isolation paradigm
  - Social Behavior
  - Social buffer
  - Sociality

  ### Attachments

  - PubMed entry
- ## The power of interspecific sociality: how humans provide social buffering for horses

  |  |  |
  | --- | --- |
  | Item Type | Journal Article |
  | Author | Alfredo Di Lucrezia |
  | Author | Anna Scandurra |
  | Author | Daria Lotito |
  | Author | Valeria Iervolino |
  | Author | Biagio D'Aniello |
  | Author | Vincenzo Mastellone |
  | Author | Pietro Lombardi |
  | Author | Claudia Pinelli |
  | Abstract | In this study, we assessed the interspecific "social buffering effect" of humans on horses, exploring how human presence influences stress responses in horses in an unfamiliar environment using the "isolation paradigm." We examined nine Haflinger horses under two counterbalanced conditions: with a passive human stranger (social condition) or alone (isolation condition). Stress responses were assessed through cortisol measurements, heart rate monitoring, and behavioral observations. While cortisol levels significantly increased in both conditions, with no notable differences before and after the tests, heart rate data revealed a different pattern. Results indicated that stress generally decreased in both scenarios, impacting heart rate. Initially, during the first five minutes, heart rate was significantly higher in the social condition compared to isolation, but this trend reversed in the following intervals, with heart rate significantly decreasing as interaction with the stranger increased. Positive interaction between time and stranger-directed behaviors suggested the stranger's influence on heart rate strengthened over time. Overall, these finding suggest that while cortisol data did not reflect a social buffering effect, other metrics indicated that human presence effectively reduced stress in horses after a brief adjustment period, supporting the hypothesis that horses can benefit from human presence during stress, after a short adaptation time. This study highlights the complex nature of stress responses in horses and the potential role of humans as social buffers in interspecific contexts. |
  | Date | 2025-03-12 |
  | Language | eng |
  | Short Title | The power of interspecific sociality |
  | Library Catalogue | PubMed |
  | Volume | 28 |
  | Pages | 20 |
  | Publication | Animal Cognition |
  | DOI | 10.1007/s10071-025-01942-5 |
  | Issue | 1 |
  | Journal Abbr | Anim Cogn |
  | ISSN | 1435-9456 |
  | PMID | 40072705 |
  | PMCID | PMC11903558 |
  | Date Added | 11/07/2025, 14:21:52 |
  | Modified | 11/07/2025, 14:21:52 |

  ### Tags:

  - Hydrocortisone
  - Heart rate
  - Animals
  - Behavior, Animal
  - Female
  - Heart Rate
  - Humans
  - Male
  - Stress, Psychological
  - Human-animal interaction
  - Saliva
  - Horses
  - Cortisol
  - Isolation paradigm
  - Social Behavior
  - Social buffer
  - Sociality

  ### Attachments

  - PubMed entry
- ## Serum Oxytocin in Cows Is Positively Correlated with Caregiver Interactions in the Impossible Task Paradigm

  |  |  |
  | --- | --- |
  | Item Type | Journal Article |
  | Author | Biagio D'Aniello |
  | Author | Vincenzo Mastellone |
  | Author | Claudia Pinelli |
  | Author | Anna Scandurra |
  | Author | Nadia Musco |
  | Author | Raffaella Tudisco |
  | Author | Maria Elena Pero |
  | Author | Federico Infascelli |
  | Author | Alfredo Di Lucrezia |
  | Author | Pietro Lombardi |
  | Abstract | This study explored a possible relationship between the circulating oxytocin, cortisol, and the willingness of dairy cows to engage in social behaviors with humans in an experimental context. The behaviors of twenty-nine cows were recorded during the impossible task paradigm, a procedure aimed at creating a violation of expectancy, in the presence of the caregiver and a stranger. The results showed that serum oxytocin levels were positively correlated with duration and negatively correlated with the latency of the cows' social interactions with the caregiver. This research provides a clear correlation between circulating oxytocin and a willingness to engage in social contact with the caregiver, excluding the possible effect of different cortisol levels on such behavior. |
  | Date | 2022-01-23 |
  | Language | eng |
  | Library Catalogue | PubMed |
  | Volume | 12 |
  | Pages | 276 |
  | Publication | Animals: an open access journal from MDPI |
  | DOI | 10.3390/ani12030276 |
  | Issue | 3 |
  | Journal Abbr | Animals (Basel) |
  | ISSN | 2076-2615 |
  | PMID | 35158600 |
  | PMCID | PMC8833709 |
  | Date Added | 11/07/2025, 11:45:53 |
  | Modified | 11/07/2025, 11:45:59 |

  ### Tags:

  - cortisol
  - oxytocin
  - human–animal interaction
  - behavior
  - dairy cows
  - impossible task

  ### Attachments

  - Full Text
  - PubMed entry
- ## Serum Oxytocin in Cows Is Positively Correlated with Caregiver Interactions in the Impossible Task Paradigm

  |  |  |
  | --- | --- |
  | Item Type | Journal Article |
  | Author | Biagio D'Aniello |
  | Author | Vincenzo Mastellone |
  | Author | Claudia Pinelli |
  | Author | Anna Scandurra |
  | Author | Nadia Musco |
  | Author | Raffaella Tudisco |
  | Author | Maria Elena Pero |
  | Author | Federico Infascelli |
  | Author | Alfredo Di Lucrezia |
  | Author | Pietro Lombardi |
  | Abstract | This study explored a possible relationship between the circulating oxytocin, cortisol, and the willingness of dairy cows to engage in social behaviors with humans in an experimental context. The behaviors of twenty-nine cows were recorded during the impossible task paradigm, a procedure aimed at creating a violation of expectancy, in the presence of the caregiver and a stranger. The results showed that serum oxytocin levels were positively correlated with duration and negatively correlated with the latency of the cows' social interactions with the caregiver. This research provides a clear correlation between circulating oxytocin and a willingness to engage in social contact with the caregiver, excluding the possible effect of different cortisol levels on such behavior. |
  | Date | 2022-01-23 |
  | Language | eng |
  | Library Catalogue | PubMed |
  | Volume | 12 |
  | Pages | 276 |
  | Publication | Animals: an open access journal from MDPI |
  | DOI | 10.3390/ani12030276 |
  | Issue | 3 |
  | Journal Abbr | Animals (Basel) |
  | ISSN | 2076-2615 |
  | PMID | 35158600 |
  | PMCID | PMC8833709 |
  | Date Added | 11/07/2025, 14:21:52 |
  | Modified | 11/07/2025, 14:21:52 |

  ### Tags:

  - cortisol
  - oxytocin
  - human–animal interaction
  - behavior
  - dairy cows
  - impossible task

  ### Attachments

  - Full Text
  - PubMed entry
- ## Associations between Oxytocin Receptor Gene Polymorphisms, Empathy towards Animals and Implicit Associations towards Animals

  |  |  |
  | --- | --- |
  | Item Type | Journal Article |
  | Author | Melanie Connor |
  | Author | Alistair B. Lawrence |
  | Author | Sarah M. Brown |
  | Abstract | Oxytocin has been well researched in association with psychological variables and is widely accepted as a key modulator of human social behaviour. Previous work indicates involvement of oxytocin receptor gene (OXTR) single nucleotide polymorphisms (SNPs) in human-human empathy, however little is known about associations of OXTR SNPs with empathy and affective reactions of humans towards animals. Five OXTR SNPs previously found to associate with human social behaviour were genotyped in 161 students. Empathy towards animals and implicit associations were evaluated. A General Linear Model was used to investigate the OXTR alleles and allelic combinations along with socio-demographic variables and their influence on empathy towards animals. Empathy towards animals showed a significant association with OXTR SNP rs2254298; homozygous G individuals reported higher levels of empathy towards animals than heterozygous (GA). Our preliminary findings show, for the first time, that between allelic variation in OXTR and animal directed empathy in humans maybe associated, suggesting that OXTRs social behaviour role crosses species boundaries, warranting independent replication. |
  | Date | 2018-08-14 |
  | Language | eng |
  | Library Catalogue | PubMed |
  | Volume | 8 |
  | Pages | 140 |
  | Publication | Animals: an open access journal from MDPI |
  | DOI | 10.3390/ani8080140 |
  | Issue | 8 |
  | Journal Abbr | Animals (Basel) |
  | ISSN | 2076-2615 |
  | PMID | 30110949 |
  | PMCID | PMC6116162 |
  | Date Added | 11/07/2025, 11:45:53 |
  | Modified | 11/07/2025, 11:45:58 |

  ### Tags:

  - empathy
  - human-animal-interaction
  - implicit associations
  - OXTR

  ### Attachments

  - Full Text
  - PubMed entry
- ## Exploratory Study of Fecal Cortisol, Weight, and Behavior as Measures of Stress and Welfare in Shelter Cats During Assimilation Into Families of Children With Autism Spectrum Disorder

  |  |  |
  | --- | --- |
  | Item Type | Journal Article |
  | Author | Gretchen K. Carlisle |
  | Author | Rebecca A. Johnson |
  | Author | Colleen S. Koch |
  | Author | Leslie A. Lyons |
  | Author | Ze Wang |
  | Author | Jessica Bibbo |
  | Author | Nancy Cheak-Zamora |
  | Abstract | Background: Cats are a common companion animal (CA) in US households, and many live in families of children with autism spectrum disorder (ASD). The prevalence of ASD is one in 54, and many children have behavior challenges as well as their diagnostic communication disorders. Objective: Benefits of CAs for children with ASD have been identified, but little is known about the welfare of CAs in these homes. This study explored the welfare of cats (N = 10) screened for ideal social and calm temperament using the Feline Temperament Profile (FTP) and adopted by families of children with ASD. Methods: Cat stress was measured using fecal cortisol, weight, and a behavior stress measure (cat stress score). Measures were taken at baseline in the shelter, 2-3 days after adoption, and at weeks 6, 12, and 18. Result: Outcome measures suggested the adopted cats' stress levels did not increase postadoption; however, the small sample size limited analytical power and generalizability. Conclusion: This study provides preliminary evidence for the success of cat adoption by families of children with ASD, when cats have been temperament screened and cat behavior educational information is provided. Further research is warranted to confirm these findings. |
  | Date | 2021 |
  | Language | eng |
  | Library Catalogue | PubMed |
  | Volume | 8 |
  | Pages | 643803 |
  | Publication | Frontiers in Veterinary Science |
  | DOI | 10.3389/fvets.2021.643803 |
  | Journal Abbr | Front Vet Sci |
  | ISSN | 2297-1769 |
  | PMID | 34552969 |
  | PMCID | PMC8450387 |
  | Date Added | 11/07/2025, 14:21:52 |
  | Modified | 11/07/2025, 14:21:52 |

  ### Tags:

  - children
  - autism spectrum disorder
  - cat adoption
  - cat stress
  - shelter cats

  ### Attachments

  - Full Text
  - PubMed entry
- ## Animal Assisted Therapy (AAT) Program As a Useful Adjunct to Conventional Psychosocial Rehabilitation for Patients with Schizophrenia: Results of a Small-scale Randomized Controlled Trial

  |  |  |
  | --- | --- |
  | Item Type | Journal Article |
  | Author | Paula Calvo |
  | Author | Joan R. Fortuny |
  | Author | Sergio Guzmán |
  | Author | Cristina Macías |
  | Author | Jonathan Bowen |
  | Author | María L. García |
  | Author | Olivia Orejas |
  | Author | Ferran Molins |
  | Author | Asta Tvarijonaviciute |
  | Author | José J. Cerón |
  | Author | Antoni Bulbena |
  | Author | Jaume Fatjó |
  | Abstract | Currently, one of the main objectives of human-animal interaction research is to demonstrate the benefits of animal assisted therapy (AAT) for specific profiles of patients or participants. The aim of this study is to assess the effect of an AAT program as an adjunct to a conventional 6-month psychosocial rehabilitation program for people with schizophrenia. Our hypothesis is that the inclusion of AAT into psychosocial rehabilitation would contribute positively to the impact of the overall program on symptomology and quality of life, and that AAT would be a positive experience for patients. To test these hypotheses, we compared pre-program with post-program scores for the Positive and Negative Syndrome Scale (PANSS) and the EuroQoL-5 dimensions questionnaire (EuroQol-5D), pre-session with post-session salivary cortisol and alpha-amylase for the last four AAT sessions, and adherence rates between different elements of the program. We conducted a randomized, controlled study in a psychiatric care center in Spain. Twenty-two institutionalized patients with chronic schizophrenia completed the 6-month rehabilitation program, which included individual psychotherapy, group therapy, a functional program (intended to improve daily functioning), a community program (intended to facilitate community reintegration) and a family program. Each member of the control group (n = 8) participated in one activity from a range of therapeutic activities that were part of the functional program. In place of this functional program activity, the AAT-treatment group (n = 14) participated in twice-weekly 1-h sessions of AAT. All participants received the same weekly total number of hours of rehabilitation. At the end of the program, both groups (control and AAT-treatment) showed significant improvements in positive and overall symptomatology, as measured with PANSS, but only the AAT-treatment group showed a significant improvement in negative symptomatology. Adherence to the AAT-treatment was significantly higher than overall adherence to the control group's functional rehabilitation activities. Cortisol level was significantly reduced after participating in an AAT session, which could indicate that interaction with the therapy dogs reduced stress. In conclusion, the results of this small-scale RCT suggest that AAT could be considered a useful adjunct to conventional psychosocial rehabilitation for people with schizophrenia. |
  | Date | 2016 |
  | Language | eng |
  | Short Title | Animal Assisted Therapy (AAT) Program As a Useful Adjunct to Conventional Psychosocial Rehabilitation for Patients with Schizophrenia |
  | Library Catalogue | PubMed |
  | Volume | 7 |
  | Pages | 631 |
  | Publication | Frontiers in Psychology |
  | DOI | 10.3389/fpsyg.2016.00631 |
  | Journal Abbr | Front Psychol |
  | ISSN | 1664-1078 |
  | PMID | 27199859 |
  | PMCID | PMC4858645 |
  | Date Added | 11/07/2025, 14:21:52 |
  | Modified | 11/07/2025, 14:21:52 |

  ### Tags:

  - animal-assisted therapy
  - salivary cortisol
  - adherence to treatment
  - EuroQol-5 dimensions
  - PANSS
  - psychosocial rehabilitation
  - salivary alpha-amylase
  - schizophrenia

  ### Attachments

  - Full Text
  - PubMed entry
- ## Affective Implications of Human-Animal Relationship on Pig Welfare: Integrating Non-Linear Heart Rate Variability Measures

  |  |  |
  | --- | --- |
  | Item Type | Journal Article |
  | Author | Javiera Calderón-Amor |
  | Author | Belén Zuleta |
  | Author | Maria Camila Ceballos |
  | Author | Daniel Cartes |
  | Author | Christopher J. Byrd |
  | Author | Benjamin Lecorps |
  | Author | Rocío Palomo |
  | Author | Sergio A. Guzmán-Pino |
  | Author | Daniela Siel |
  | Author | Daniela Luna |
  | Abstract | The human-animal relationship is crucial for animal welfare. Gentle handling enhances pigs' comfort while rough handling causes fear and stress. This study examined how different human-animal relationship qualities affect the behavior and heart rate variability (linear and non-linear parameters) of 36 nursery pigs. Over six weeks, pigs experienced positive (n = 12), minimal (n = 12), or negative (n = 12) human handling. Their responses to handlers were then assessed in an experimental arena with four phases: habituation, exposure to the handler standing and sitting, and forced interaction. Pigs subjected to negative handling exhibited increased fear-related behaviors, spending less time in contact with the handler. They also exhibited heightened stress responses, with greater LF/HF ratio and Lmean values compared with positively handled pigs. Conversely, gently handled pigs displayed affiliative behaviors, accepting more strokes, and higher parasympathetic activation, indicated by greater RMSSD/SDNN and SampEn values, suggesting a more positive affective state. Minimally handled pigs exhibited some behavioral similarities to gently handled pigs, although physiological data indicated that the interaction was likely more rewarding for the gently handled pigs. These results emphasize the impact of human-animal relationships on pig welfare and highlight the value of incorporating non-linear heart rate variability parameters in such evaluations. |
  | Date | 2024-07-31 |
  | Language | eng |
  | Short Title | Affective Implications of Human-Animal Relationship on Pig Welfare |
  | Library Catalogue | PubMed |
  | Volume | 14 |
  | Pages | 2217 |
  | Publication | Animals: an open access journal from MDPI |
  | DOI | 10.3390/ani14152217 |
  | Issue | 15 |
  | Journal Abbr | Animals (Basel) |
  | ISSN | 2076-2615 |
  | PMID | 39123743 |
  | PMCID | PMC11310953 |
  | Date Added | 11/07/2025, 11:37:24 |
  | Modified | 11/07/2025, 11:37:24 |

  ### Tags:

  - human–animal interaction
  - animal welfare
  - affective state
  - heart rate variability
  - human handling
  - non-linear heart rate parameters
  - nursery pigs
  - positive affective state
  - stress physiology

  ### Attachments

  - Full Text
  - PubMed entry
- ## Neurobiological underpinnings of dogs' human-like social competence: How interactions between stress response systems and oxytocin mediate dogs' social skills

  |  |  |
  | --- | --- |
  | Item Type | Journal Article |
  | Author | Alicia Phillips Buttner |
  | Abstract | Domestic dogs (Canis familiaris) have been suggested as a natural model for human social cognition, possessing social skills that are in many ways functionally analogous to those of young humans. Researchers have debated the origins of dogs' human-like social competence and the underlying cognitive mechanisms, but only recently have researchers begun to explore their neurobiological underpinnings. In this review, findings from behavioral studies are integrated with what is known about the biological basis of dogs' human-directed social competence, with an emphasis on how stress-mediating systems, particularly the hypothalamic-pituitary-adrenal (HPA) axis, interact with oxytocin and underlying neural systems to facilitate dogs' interspecific social-cognitive abilities. The working model presented in this paper offers a biological explanation for many of the inconsistent findings from past work on social cognition in dogs and generates questions for future research in the field of canine social competence. |
  | Date | 2016-12 |
  | Language | eng |
  | Short Title | Neurobiological underpinnings of dogs' human-like social competence |
  | Library Catalogue | PubMed |
  | Volume | 71 |
  | Pages | 198-214 |
  | Publication | Neuroscience and Biobehavioral Reviews |
  | DOI | 10.1016/j.neubiorev.2016.08.029 |
  | Journal Abbr | Neurosci Biobehav Rev |
  | ISSN | 1873-7528 |
  | PMID | 27593441 |
  | Date Added | 11/07/2025, 11:45:53 |
  | Modified | 11/07/2025, 11:45:57 |

  ### Tags:

  - Oxytocin
  - Dogs
  - Animals
  - Behavior, Animal
  - Humans
  - Human-animal interaction
  - Attachment
  - Dog
  - Domestication
  - HPA axis
  - Social cognition
  - Social competence
  - Social Skills
  - Stress reactivity

  ### Attachments

  - PubMed entry
- ## Oxytocin levels and self-reported anxiety during interactions between humans and cows

  |  |  |
  | --- | --- |
  | Item Type | Journal Article |
  | Author | Bente Berget |
  | Author | Judit Vas |
  | Author | Gunn Pedersen |
  | Author | Kerstin Uvnäs-Moberg |
  | Author | Ruth C. Newberry |
  | Abstract | INTRODUCTION: Positive social interactions with farm animals may have therapeutic benefits on humans by increasing brain oxytocin secretion, as inferred from circulating oxytocin levels. The aim of this observational study was to investigate acute changes in human plasma oxytocin levels and state anxiety associated with interactions with dairy cows. METHODS: Data were collected from 18 healthy female nursing students who performed stroking and brushing of an unfamiliar cow for 15 min. Blood samples were drawn before entering the cowshed (T1, baseline), and after 5 (T2) and 15 (T3) min of interaction with a cow. At T1 and T3, the students filled out the Norwegian version of the Spielberger State-Trait Anxiety Inventory-State Subscale (STAI-SS). RESULTS: Across participants, no significant changes in average plasma oxytocin concentration were detected between time points (p>0.05). There was, however, a modest decline in the STAI-SS scores between T1 and T3 (p=0.015) and a positive correlation between the change in individual level of state anxiety between T1 and T3 and the change in OT concentration of the same individual between T2 and T3 (p = 0.045). DISCUSSION: The results suggest that friendly social interactions with cows are beneficial in lowering state anxiety, but any relationship with release of OT into the circulation was complex and variable across individuals. The acute reduction in state anxiety lends support to the value of interacting with farm animals in the context of Green Care for people with mental health challenges. |
  | Date | 2023 |
  | Language | eng |
  | Library Catalogue | PubMed |
  | Volume | 14 |
  | Pages | 1252463 |
  | Publication | Frontiers in Psychology |
  | DOI | 10.3389/fpsyg.2023.1252463 |
  | Journal Abbr | Front Psychol |
  | ISSN | 1664-1078 |
  | PMID | 37780173 |
  | PMCID | PMC10536144 |
  | Date Added | 11/07/2025, 11:45:53 |
  | Modified | 11/07/2025, 11:45:57 |

  ### Tags:

  - oxytocin
  - human–animal interaction
  - animal-assisted intervention
  - anxiety
  - cow
  - green care

  ### Attachments

  - Full Text
  - PubMed entry
- ## Psychosocial and psychophysiological effects of human-animal interactions: the possible role of oxytocin

  |  |  |
  | --- | --- |
  | Item Type | Journal Article |
  | Author | Andrea Beetz |
  | Author | Kerstin Uvnäs-Moberg |
  | Author | Henri Julius |
  | Author | Kurt Kotrschal |
  | Abstract | During the last decade it has become more widely accepted that pet ownership and animal assistance in therapy and education may have a multitude of positive effects on humans. Here, we review the evidence from 69 original studies on human-animal interactions (HAI) which met our inclusion criteria with regard to sample size, peer-review, and standard scientific research design. Among the well-documented effects of HAI in humans of different ages, with and without special medical, or mental health conditions are benefits for: social attention, social behavior, interpersonal interactions, and mood; stress-related parameters such as cortisol, heart rate, and blood pressure; self-reported fear and anxiety; and mental and physical health, especially cardiovascular diseases. Limited evidence exists for positive effects of HAI on: reduction of stress-related parameters such as epinephrine and norepinephrine; improvement of immune system functioning and pain management; increased trustworthiness of and trust toward other persons; reduced aggression; enhanced empathy and improved learning. We propose that the activation of the oxytocin system plays a key role in the majority of these reported psychological and psychophysiological effects of HAI. Oxytocin and HAI effects largely overlap, as documented by research in both, humans and animals, and first studies found that HAI affects the oxytocin system. As a common underlying mechanism, the activation of the oxytocin system does not only provide an explanation, but also allows an integrative view of the different effects of HAI. |
  | Date | 2012 |
  | Language | eng |
  | Short Title | Psychosocial and psychophysiological effects of human-animal interactions |
  | Library Catalogue | PubMed |
  | Volume | 3 |
  | Pages | 234 |
  | Publication | Frontiers in Psychology |
  | DOI | 10.3389/fpsyg.2012.00234 |
  | Journal Abbr | Front Psychol |
  | ISSN | 1664-1078 |
  | PMID | 22866043 |
  | PMCID | PMC3408111 |
  | Date Added | 11/07/2025, 11:37:24 |
  | Modified | 11/07/2025, 11:37:24 |

  ### Tags:

  - oxytocin
  - human-animal interaction
  - animal-assisted interventions
  - animal-assisted therapy
  - pet ownership
  - stress reduction

  ### Attachments

  - Full Text
  - PubMed entry
- ## Psychosocial and psychophysiological effects of human-animal interactions: the possible role of oxytocin

  |  |  |
  | --- | --- |
  | Item Type | Journal Article |
  | Author | Andrea Beetz |
  | Author | Kerstin Uvnäs-Moberg |
  | Author | Henri Julius |
  | Author | Kurt Kotrschal |
  | Abstract | During the last decade it has become more widely accepted that pet ownership and animal assistance in therapy and education may have a multitude of positive effects on humans. Here, we review the evidence from 69 original studies on human-animal interactions (HAI) which met our inclusion criteria with regard to sample size, peer-review, and standard scientific research design. Among the well-documented effects of HAI in humans of different ages, with and without special medical, or mental health conditions are benefits for: social attention, social behavior, interpersonal interactions, and mood; stress-related parameters such as cortisol, heart rate, and blood pressure; self-reported fear and anxiety; and mental and physical health, especially cardiovascular diseases. Limited evidence exists for positive effects of HAI on: reduction of stress-related parameters such as epinephrine and norepinephrine; improvement of immune system functioning and pain management; increased trustworthiness of and trust toward other persons; reduced aggression; enhanced empathy and improved learning. We propose that the activation of the oxytocin system plays a key role in the majority of these reported psychological and psychophysiological effects of HAI. Oxytocin and HAI effects largely overlap, as documented by research in both, humans and animals, and first studies found that HAI affects the oxytocin system. As a common underlying mechanism, the activation of the oxytocin system does not only provide an explanation, but also allows an integrative view of the different effects of HAI. |
  | Date | 2012 |
  | Language | eng |
  | Short Title | Psychosocial and psychophysiological effects of human-animal interactions |
  | Library Catalogue | PubMed |
  | Volume | 3 |
  | Pages | 234 |
  | Publication | Frontiers in Psychology |
  | DOI | 10.3389/fpsyg.2012.00234 |
  | Journal Abbr | Front Psychol |
  | ISSN | 1664-1078 |
  | PMID | 22866043 |
  | PMCID | PMC3408111 |
  | Date Added | 11/07/2025, 11:45:53 |
  | Modified | 11/07/2025, 11:45:56 |

  ### Tags:

  - oxytocin
  - human-animal interaction
  - animal-assisted interventions
  - animal-assisted therapy
  - pet ownership
  - stress reduction

  ### Attachments

  - Full Text
  - PubMed entry
- ## Psychosocial and psychophysiological effects of human-animal interactions: the possible role of oxytocin

  |  |  |
  | --- | --- |
  | Item Type | Journal Article |
  | Author | Andrea Beetz |
  | Author | Kerstin Uvnäs-Moberg |
  | Author | Henri Julius |
  | Author | Kurt Kotrschal |
  | Abstract | During the last decade it has become more widely accepted that pet ownership and animal assistance in therapy and education may have a multitude of positive effects on humans. Here, we review the evidence from 69 original studies on human-animal interactions (HAI) which met our inclusion criteria with regard to sample size, peer-review, and standard scientific research design. Among the well-documented effects of HAI in humans of different ages, with and without special medical, or mental health conditions are benefits for: social attention, social behavior, interpersonal interactions, and mood; stress-related parameters such as cortisol, heart rate, and blood pressure; self-reported fear and anxiety; and mental and physical health, especially cardiovascular diseases. Limited evidence exists for positive effects of HAI on: reduction of stress-related parameters such as epinephrine and norepinephrine; improvement of immune system functioning and pain management; increased trustworthiness of and trust toward other persons; reduced aggression; enhanced empathy and improved learning. We propose that the activation of the oxytocin system plays a key role in the majority of these reported psychological and psychophysiological effects of HAI. Oxytocin and HAI effects largely overlap, as documented by research in both, humans and animals, and first studies found that HAI affects the oxytocin system. As a common underlying mechanism, the activation of the oxytocin system does not only provide an explanation, but also allows an integrative view of the different effects of HAI. |
  | Date | 2012 |
  | Language | eng |
  | Short Title | Psychosocial and psychophysiological effects of human-animal interactions |
  | Library Catalogue | PubMed |
  | Volume | 3 |
  | Pages | 234 |
  | Publication | Frontiers in Psychology |
  | DOI | 10.3389/fpsyg.2012.00234 |
  | Journal Abbr | Front Psychol |
  | ISSN | 1664-1078 |
  | PMID | 22866043 |
  | PMCID | PMC3408111 |
  | Date Added | 11/07/2025, 14:21:52 |
  | Modified | 11/07/2025, 14:21:52 |

  ### Tags:

  - oxytocin
  - human-animal interaction
  - animal-assisted interventions
  - animal-assisted therapy
  - pet ownership
  - stress reduction

  ### Attachments

  - Full Text
  - PubMed entry
- ## Judgement bias in goats (Capra hircus): investigating the effects of human grooming

  |  |  |
  | --- | --- |
  | Item Type | Journal Article |
  | Author | Luigi Baciadonna |
  | Author | Christian Nawroth |
  | Author | Alan G. McElligott |
  | Abstract | Animal emotional states can be investigated by evaluating their impact on cognitive processes. In this study, we used a judgement bias paradigm to determine if short-term positive human-animal interaction (grooming) induced a positive affective state in goats. We tested two groups of goats and trained them to discriminate between a rewarded and a non-rewarded location over nine training days. During training, the experimental group (n = 9) was gently groomed by brushing their heads and backs for five min over 11 days (nine training days, plus two testing days, total time 55 min). During training, the control group (n = 10) did not experience any direct interaction with the experimenter, but was kept unconstrained next to him for the same period of time. After successful completion of the training, the responses (latency time) of the two groups to reach ambiguous locations situated between the two reference locations (i.e., rewarded/non-rewarded) were compared over two days of testing. There was not a positive bias effect after the animals had been groomed. In a second experiment, 10 goats were tested to investigate whether grooming induced changes in physiological activation (i.e., heart rate and heart rate variability). Heart rate increased when goats were groomed compared to the baseline condition, when the same goats did not receive any contact with the experimenter. Also, subjects did not move away from the experimenter, suggesting that the grooming was positively accepted. The very good care and the regular positive contacts that goats received from humans at the study site could potentially account for the results obtained. Good husbandry outcomes are influenced by animals' perception of the events and this is based on current circumstances, past experiences and individual variables. Taking into account animals' individual characteristics and identifying effective strategies to induce positive emotions could increase the understanding and reliability of using cognitive biases paradigms to investigate and promote animal welfare. |
  | Date | 2016 |
  | Language | eng |
  | Short Title | Judgement bias in goats (Capra hircus) |
  | Library Catalogue | PubMed |
  | Volume | 4 |
  | Pages | e2485 |
  | Publication | PeerJ |
  | DOI | 10.7717/peerj.2485 |
  | Journal Abbr | PeerJ |
  | ISSN | 2167-8359 |
  | PMID | 27761311 |
  | PMCID | PMC5068416 |
  | Date Added | 11/07/2025, 11:37:24 |
  | Modified | 11/07/2025, 11:37:24 |

  ### Tags:

  - Heart rate
  - Cognition
  - Emotions
  - Human-animal interaction
  - Positive affective states
  - RMSSD
  - Stroking

  ### Attachments

  - Full Text PDF
  - PubMed entry
