# Supplementary material for: Evidence of Physiological Comodulation During Human–Animal Interaction: A Systematic Review
Source: Ann N Y Acad Sci. 2026 Jun 4;1560(1):e70299. doi: 10.1111/nyas.70299 (PMC13238372; doi:10.1111/nyas.70299)
Supplement: Supplementary file 2 — Supplementary Materials: Supp2‐Zotero‐Collection.zip [file NYAS-1560-0-s002.zip › Supp2_Zotero_Collection/title screened/Scopus.htm]

Zotero Report


- ## Equine-assisted therapy and its impact on cortisol levels of children and horses: A pilot study and meta-analysis

  |  |  |
  | --- | --- |
  | Item Type | Journal Article |
  | Author | Jan Yorke |
  | Author | William Nugent |
  | Author | Elizabeth Strand |
  | Author | Rebecca Bolen |
  | Author | John New |
  | Author | Cindy Davis |
  | Date | 2013 |
  | URL | https://www.scopus.com/inward/record.uri?eid=2-s2.0-84879534999&doi=10.1080%2f03004430.2012.693486&partnerID=40&md5=ceea909b8bdcffb6cea3e1e39c3e153f |
  | Extra | Type: Article |
  | Volume | 183 |
  | Pages | 874 – 894 |
  | Publication | Early Child Development and Care |
  | DOI | 10.1080/03004430.2012.693486 |
  | Issue | 7 |
  | Date Added | 11/07/2025, 14:08:23 |
  | Modified | 11/07/2025, 14:08:23 |

  ### Notes:

  - Cited by: 45
- ## Are Hair Cortisol Levels of Humans, Cats, and Dogs from the Same Household Correlated?

  |  |  |
  | --- | --- |
  | Item Type | Journal Article |
  | Author | Justyna Wojtaś |
  | Author | Aleksandra Garbiec |
  | Author | Mirosław Karpiński |
  | Author | Patrycja Skowronek |
  | Author | Aneta Strachecka |
  | Date | 2022 |
  | URL | https://www.scopus.com/inward/record.uri?eid=2-s2.0-85131290461&doi=10.3390%2fani12111472&partnerID=40&md5=9d8d741818c814db16a74f49f5616476 |
  | Extra | Type: Article |
  | Volume | 12 |
  | Publication | Animals |
  | DOI | 10.3390/ani12111472 |
  | Issue | 11 |
  | Date Added | 11/07/2025, 14:08:23 |
  | Modified | 11/07/2025, 14:08:23 |

  ### Notes:

  - Cited by: 7; All Open Access, Gold Open Access, Green Open Access
- ## Impacts of COVID-19 on Animals in Zoos: A Longitudinal Multi-Species Analysis

  |  |  |
  | --- | --- |
  | Item Type | Journal Article |
  | Author | Ellen Williams |
  | Author | Anne Carter |
  | Author | Jessica Rendle |
  | Author | Samantha J. Ward |
  | Date | 2021 |
  | URL | https://www.scopus.com/inward/record.uri?eid=2-s2.0-85109318136&doi=10.3390%2fjzbg2020010&partnerID=40&md5=136cdd1319e38768c824bf08b1d1f451 |
  | Extra | Number: 2 Type: Article |
  | Volume | 2 |
  | Pages | 130 – 145 |
  | Publication | Journal of Zoological and Botanical Gardens |
  | DOI | 10.3390/jzbg2020010 |
  | Issue | 2 |
  | Date Added | 28/07/2025, 12:48:52 |
  | Modified | 28/07/2025, 12:48:52 |

  ### Notes:

  - Cited by: 29; All Open Access, Gold Open Access, Green Open Access
- ## Assessing the Visitor and Animal Outcomes of a Zoo Encounter and Guided Tour Program with Ambassador Cheetahs

  |  |  |
  | --- | --- |
  | Item Type | Journal Article |
  | Author | Katherine M. Whitehouse-Tedd |
  | Author | Jairo Lozano-Martinez |
  | Author | Jessica Reeves |
  | Author | Mollie Page |
  | Author | Jaime H. Martin |
  | Author | Heidi Prozesky |
  | Date | 2022 |
  | URL | https://www.scopus.com/inward/record.uri?eid=2-s2.0-85118252092&doi=10.1080%2f08927936.2021.1986263&partnerID=40&md5=94e2e6f6d08e8b4cfdff032c318b08a9 |
  | Extra | Type: Article |
  | Volume | 35 |
  | Pages | 307 – 322 |
  | Publication | Anthrozoos |
  | DOI | 10.1080/08927936.2021.1986263 |
  | Issue | 2 |
  | Date Added | 19/06/2025, 15:40:13 |
  | Modified | 19/06/2025, 15:40:13 |

  ### Notes:

  - Cited by: 11; All Open Access, Hybrid Gold Open Access
- ## The State of Research on Human–Animal Relations: Implications for Human Health

  |  |  |
  | --- | --- |
  | Item Type | Journal Article |
  | Author | Deborah L. Wells |
  | Date | 2019 |
  | URL | https://www.scopus.com/inward/record.uri?eid=2-s2.0-85063194294&doi=10.1080%2f08927936.2019.1569902&partnerID=40&md5=a7fc38433d2a1aec057e106c7421bc16 |
  | Extra | Type: Article |
  | Volume | 32 |
  | Pages | 169 – 181 |
  | Publication | Anthrozoos |
  | DOI | 10.1080/08927936.2019.1569902 |
  | Issue | 2 |
  | Date Added | 20/06/2025, 09:37:47 |
  | Modified | 20/06/2025, 09:37:47 |

  ### Notes:

  - Cited by: 120; All Open Access, Green Open Access, Hybrid Gold Open Access
- ## Cat Foster Program Outcomes: Behavior, Stress, and Cat–Human Interaction

  |  |  |
  | --- | --- |
  | Item Type | Journal Article |
  | Author | Kristyn R. Vitale |
  | Author | Delaney H. Frank |
  | Author | Jocelyn Conroy |
  | Author | Monique A. R. Udell |
  | Date | 2022 |
  | URL | https://www.scopus.com/inward/record.uri?eid=2-s2.0-85137775119&doi=10.3390%2fani12172166&partnerID=40&md5=cacc02638ef386f0d8da7f172598e0d6 |
  | Extra | Type: Article |
  | Volume | 12 |
  | Publication | Animals |
  | DOI | 10.3390/ani12172166 |
  | Issue | 17 |
  | Date Added | 11/07/2025, 14:08:23 |
  | Modified | 11/07/2025, 14:08:23 |

  ### Notes:

  - Cited by: 10; All Open Access, Gold Open Access, Green Open Access
- ## Therapy Dog Support in Pediatric Dentistry: A Social Welfare Intervention for Reducing Anticipatory Anxiety and Situational Fear in Children

  |  |  |
  | --- | --- |
  | Item Type | Journal Article |
  | Author | Aviva Vincent |
  | Author | Masahiro Heima |
  | Author | Kathleen J. Farkas |
  | Date | 2020 |
  | URL | https://www.scopus.com/inward/record.uri?eid=2-s2.0-85091006780&doi=10.1007%2fs10560-020-00701-4&partnerID=40&md5=2303f4050341d2ca27f18c2232c320fb |
  | Extra | Type: Article |
  | Volume | 37 |
  | Pages | 615 – 629 |
  | Publication | Child and Adolescent Social Work Journal |
  | DOI | 10.1007/s10560-020-00701-4 |
  | Issue | 6 |
  | Date Added | 20/06/2025, 09:37:48 |
  | Modified | 20/06/2025, 09:37:48 |

  ### Notes:

  - Cited by: 16
- ## Therapy Dog Support in Pediatric Dentistry: A Social Welfare Intervention for Reducing Anticipatory Anxiety and Situational Fear in Children

  |  |  |
  | --- | --- |
  | Item Type | Journal Article |
  | Author | Aviva Vincent |
  | Author | Masahiro Heima |
  | Author | Kathleen J. Farkas |
  | Date | 2020 |
  | URL | https://www.scopus.com/inward/record.uri?eid=2-s2.0-85091006780&doi=10.1007%2fs10560-020-00701-4&partnerID=40&md5=2303f4050341d2ca27f18c2232c320fb |
  | Extra | Type: Article |
  | Volume | 37 |
  | Pages | 615 – 629 |
  | Publication | Child and Adolescent Social Work Journal |
  | DOI | 10.1007/s10560-020-00701-4 |
  | Issue | 6 |
  | Date Added | 11/07/2025, 14:08:22 |
  | Modified | 11/07/2025, 14:08:22 |

  ### Notes:

  - Cited by: 16
- ## Perceiving emotions in human-human and human-animal interactions: Hemodynamic prefrontal activity (fNIRS) and empathic concern

  |  |  |
  | --- | --- |
  | Item Type | Journal Article |
  | Author | M.E. Vanutelli |
  | Author | M. Balconi |
  | Date | 2015 |
  | Archive | Scopus |
  | URL | https://www.scopus.com/inward/record.uri?eid=2-s2.0-84939619665&doi=10.1016%2fj.neulet.2015.07.020&partnerID=40&md5=de9df2ba90f1c21de1963ff2e38ea7bf |
  | Volume | 605 |
  | Pages | 1-6 |
  | Publication | Neuroscience Letters |
  | DOI | 10.1016/j.neulet.2015.07.020 |
  | Date Added | 11/07/2025, 11:24:11 |
  | Modified | 11/07/2025, 11:24:11 |

  ### Notes:

  - Export Date: 11 July 2025; Cited By: 18
- ## Effects of Essential Animal Visitation Program (AVP) Components on Students’ Salivary α-Amylase and Amylase-to-Cortisol Ratios

  |  |  |
  | --- | --- |
  | Item Type | Journal Article |
  | Author | Jaymie L. Vandagriff |
  | Author | Alexa M. Carr |
  | Author | Stephanie M. Roeter Smith |
  | Author | Patricia Pendry |
  | Date | 2022 |
  | URL | https://www.scopus.com/inward/record.uri?eid=2-s2.0-85119954206&doi=10.1080%2f08927936.2021.1996025&partnerID=40&md5=472ad933641a68de03a8aebea86442d0 |
  | Extra | Type: Article |
  | Volume | 35 |
  | Pages | 443 – 461 |
  | Publication | Anthrozoos |
  | DOI | 10.1080/08927936.2021.1996025 |
  | Issue | 3 |
  | Date Added | 11/07/2025, 14:08:23 |
  | Modified | 11/07/2025, 14:08:23 |

  ### Notes:

  - Cited by: 2
- ## Dogs as an Adjunct to Therapy: Effects of Animal-Assisted Therapy on Rehabilitation Following Spinal Cord Injury

  |  |  |
  | --- | --- |
  | Item Type | Journal Article |
  | Author | Andie M. Thompkins |
  | Author | Sarah J. Adkins |
  | Author | Maggie Leopard |
  | Author | Cameron Spencer |
  | Author | David Bentley |
  | Author | Lauren Bolden |
  | Author | Christina H. Jagielski |
  | Author | Elizabeth Richardson |
  | Author | Adam M. Goodman |
  | Author | David C. Schwebel |
  | Date | 2019 |
  | URL | https://www.scopus.com/inward/record.uri?eid=2-s2.0-85073219099&doi=10.1080%2f08927936.2019.1645513&partnerID=40&md5=3cfde89620394962a2d93050b75b00da |
  | Extra | Type: Article |
  | Volume | 32 |
  | Pages | 679 – 690 |
  | Publication | Anthrozoos |
  | DOI | 10.1080/08927936.2019.1645513 |
  | Issue | 5 |
  | Date Added | 11/07/2025, 14:08:22 |
  | Modified | 11/07/2025, 14:08:22 |

  ### Notes:

  - Cited by: 6
- ## Nasally-administered oxytocin has limited effects on owner-directed attachment behavior in pet dogs (Canis lupus familiaris)

  |  |  |
  | --- | --- |
  | Item Type | Journal Article |
  | Author | Lauren E. Thielke |
  | Author | Giovanna Rosenlicht |
  | Author | Sarina R. Saturn |
  | Author | Monique A.R. Udell |
  | Date | 2017 |
  | URL | https://www.scopus.com/inward/record.uri?eid=2-s2.0-85030165925&doi=10.3389%2ffpsyg.2017.01699&partnerID=40&md5=284f080554c29cae582c823f8c8542b2 |
  | Extra | Type: Article |
  | Volume | 8 |
  | Publication | Frontiers in Psychology |
  | DOI | 10.3389/fpsyg.2017.01699 |
  | Issue | SEP |
  | Date Added | 20/06/2025, 09:37:48 |
  | Modified | 20/06/2025, 09:37:48 |

  ### Notes:

  - Cited by: 28; All Open Access, Gold Open Access, Green Open Access
- ## The role of oxytocin in relationships between dogs and humans and potential applications for the treatment of separation anxiety in dogs

  |  |  |
  | --- | --- |
  | Item Type | Journal Article |
  | Author | Lauren E. Thielke |
  | Author | Monique A. R. Udell |
  | Date | 2017 |
  | URL | https://www.scopus.com/inward/record.uri?eid=2-s2.0-84948150691&doi=10.1111%2fbrv.12235&partnerID=40&md5=335d12e104352e91711d44775740d938 |
  | Extra | Type: Article |
  | Volume | 92 |
  | Pages | 378 – 388 |
  | Publication | Biological Reviews |
  | DOI | 10.1111/brv.12235 |
  | Issue | 1 |
  | Date Added | 20/06/2025, 09:37:47 |
  | Modified | 20/06/2025, 09:37:47 |

  ### Notes:

  - Cited by: 40
- ## Repeated handling of pigs during rearing. I. Refusal of contact by the handler and reactivity to familiar and unfamiliar humans

  |  |  |
  | --- | --- |
  | Item Type | Journal Article |
  | Author | E.M.C. Terlouw |
  | Author | J. Porcher |
  | Date | 2005 |
  | URL | https://www.scopus.com/inward/record.uri?eid=2-s2.0-33645963149&doi=10.2527%2f2005.8371653x&partnerID=40&md5=d6d53bb38fef45fe78864fcef8c8a81b |
  | Extra | Type: Article |
  | Volume | 83 |
  | Pages | 1653 – 1663 |
  | Publication | Journal of Animal Science |
  | DOI | 10.2527/2005.8371653x |
  | Issue | 7 |
  | Date Added | 19/06/2025, 15:40:12 |
  | Modified | 19/06/2025, 15:40:12 |

  ### Notes:

  - Cited by: 38
- ## Brain and heart activity during interactions with pet dogs: A portable electroencephalogram and heart rate variability study

  |  |  |
  | --- | --- |
  | Item Type | Journal Article |
  | Author | Jillian T. Teo |
  | Author | Stuart J. Johnstone |
  | Author | Susan J. Thomas |
  | Date | 2024 |
  | URL | https://www.scopus.com/inward/record.uri?eid=2-s2.0-85201695749&doi=10.1016%2fj.ijpsycho.2024.112412&partnerID=40&md5=45b08128b1ff29a497f8fa63db56aee9 |
  | Extra | Type: Article |
  | Volume | 204 |
  | Publication | International Journal of Psychophysiology |
  | DOI | 10.1016/j.ijpsycho.2024.112412 |
  | Date Added | 19/06/2025, 15:40:13 |
  | Modified | 19/06/2025, 15:40:13 |

  ### Notes:

  - Cited by: 1; All Open Access, Hybrid Gold Open Access
- ## Brain and heart activity during interactions with pet dogs: A portable electroencephalogram and heart rate variability study

  |  |  |
  | --- | --- |
  | Item Type | Journal Article |
  | Author | J.T. Teo |
  | Author | S.J. Johnstone |
  | Author | S.J. Thomas |
  | Date | 2024 |
  | Archive | Scopus |
  | URL | https://www.scopus.com/inward/record.uri?eid=2-s2.0-85201695749&doi=10.1016%2fj.ijpsycho.2024.112412&partnerID=40&md5=45b08128b1ff29a497f8fa63db56aee9 |
  | Volume | 204 |
  | Publication | International Journal of Psychophysiology |
  | DOI | 10.1016/j.ijpsycho.2024.112412 |
  | Date Added | 11/07/2025, 11:22:51 |
  | Modified | 11/07/2025, 11:22:51 |

  ### Notes:

  - Export Date: 11 July 2025; Cited By: 1
- ## THE IMPACT OF HUMAN-HORSE INTERACTIONS ON PHYSICAL AND EMOTIONAL WELLBEING

  |  |  |
  | --- | --- |
  | Item Type | Journal Article |
  | Author | Rūta Šveistienė |
  | Author | Alfonsas Vainoras |
  | Author | Kristina Berškienė |
  | Author | Mantas Landauskas |
  | Date | 2024 |
  | URL | https://www.scopus.com/inward/record.uri?eid=2-s2.0-85212932655&partnerID=40&md5=6ac6dc2dfbfa36595fb73fe39d108127 |
  | Extra | Type: Article |
  | Volume | 82(1) |
  | Pages | 138 – 138 |
  | Publication | Veterinarija ir Zootechnika |
  | Date Added | 19/06/2025, 15:40:13 |
  | Modified | 19/06/2025, 15:40:13 |

  ### Notes:

  - Cited by: 0
- ## A walk on the wild side: How interactions with non-companion animals might help reduce human stress

  |  |  |
  | --- | --- |
  | Item Type | Journal Article |
  | Author | Rachel C. Sumner |
  | Author | Anne E. Goodenough |
  | Date | 2020 |
  | URL | https://www.scopus.com/inward/record.uri?eid=2-s2.0-85106226191&doi=10.1002%2fpan3.10074&partnerID=40&md5=c99802ae60870ec80eca0e4dc1880925 |
  | Extra | Type: Article |
  | Volume | 2 |
  | Pages | 395 – 405 |
  | Publication | People and Nature |
  | DOI | 10.1002/pan3.10074 |
  | Issue | 2 |
  | Date Added | 19/06/2025, 15:40:12 |
  | Modified | 19/06/2025, 15:40:12 |

  ### Notes:

  - Cited by: 9; All Open Access, Gold Open Access, Green Open Access
- ## A walk on the wild side: How interactions with non-companion animals might help reduce human stress

  |  |  |
  | --- | --- |
  | Item Type | Journal Article |
  | Author | Rachel C. Sumner |
  | Author | Anne E. Goodenough |
  | Date | 2020 |
  | URL | https://www.scopus.com/inward/record.uri?eid=2-s2.0-85106226191&doi=10.1002%2fpan3.10074&partnerID=40&md5=c99802ae60870ec80eca0e4dc1880925 |
  | Extra | Type: Article |
  | Volume | 2 |
  | Pages | 395 – 405 |
  | Publication | People and Nature |
  | DOI | 10.1002/pan3.10074 |
  | Issue | 2 |
  | Date Added | 11/07/2025, 14:08:22 |
  | Modified | 11/07/2025, 14:08:22 |

  ### Notes:

  - Cited by: 10; All Open Access, Gold Open Access, Green Open Access
- ## Effect of previous handling experiences on responses of dairy calves to routine husbandry procedures

  |  |  |
  | --- | --- |
  | Item Type | Journal Article |
  | Author | M. Stewart |
  | Author | H.M. Shepherd |
  | Author | J.R. Webster |
  | Author | J.R. Waas |
  | Author | L.M. McLeay |
  | Author | K.E. Schütz |
  | Date | 2013 |
  | URL | https://www.scopus.com/inward/record.uri?eid=2-s2.0-84875737216&doi=10.1017%2fS175173111200225X&partnerID=40&md5=1118d0fc3cb7b02399bca5957025d90b |
  | Extra | Type: Article |
  | Volume | 7 |
  | Pages | 828 – 833 |
  | Publication | Animal |
  | DOI | 10.1017/S175173111200225X |
  | Issue | 5 |
  | Date Added | 19/06/2025, 15:40:13 |
  | Modified | 19/06/2025, 15:40:13 |

  ### Notes:

  - Cited by: 31; All Open Access, Green Open Access, Hybrid Gold Open Access
- ## Effect of previous handling experiences on responses of dairy calves to routine husbandry procedures

  |  |  |
  | --- | --- |
  | Item Type | Journal Article |
  | Author | M. Stewart |
  | Author | H.M. Shepherd |
  | Author | J.R. Webster |
  | Author | J.R. Waas |
  | Author | L.M. McLeay |
  | Author | K.E. Schütz |
  | Date | 2013 |
  | URL | https://www.scopus.com/inward/record.uri?eid=2-s2.0-84875737216&doi=10.1017%2fS175173111200225X&partnerID=40&md5=1118d0fc3cb7b02399bca5957025d90b |
  | Extra | Type: Article |
  | Volume | 7 |
  | Pages | 828 – 833 |
  | Publication | Animal |
  | DOI | 10.1017/S175173111200225X |
  | Issue | 5 |
  | Date Added | 11/07/2025, 14:08:23 |
  | Modified | 11/07/2025, 14:08:23 |

  ### Notes:

  - Cited by: 31; All Open Access, Green Open Access, Hybrid Gold Open Access
- ## Dog–Owner Relationship, Owner Interpretations and Dog Personality Are Connected with the Emotional Reactivity of Dogs

  |  |  |
  | --- | --- |
  | Item Type | Journal Article |
  | Author | Sanni Somppi |
  | Author | Heini Törnqvist |
  | Author | Aija Koskela |
  | Author | Antti Vehkaoja |
  | Author | Katriina Tiira |
  | Author | Heli Väätäjä |
  | Author | Veikko Surakka |
  | Author | Outi Vainio |
  | Author | Miiamaaria V. Kujala |
  | Date | 2022 |
  | URL | https://www.scopus.com/inward/record.uri?eid=2-s2.0-85130795002&doi=10.3390%2fani12111338&partnerID=40&md5=eb300354ae99124a94bd1bebd82481d3 |
  | Extra | Type: Article |
  | Volume | 12 |
  | Publication | Animals |
  | DOI | 10.3390/ani12111338 |
  | Issue | 11 |
  | Date Added | 19/06/2025, 15:40:13 |
  | Modified | 19/06/2025, 15:40:13 |

  ### Notes:

  - Cited by: 21; All Open Access, Gold Open Access, Green Open Access
- ## Best practices for physiological data collection in youth with autism and co-occurring mental health diagnoses: Implications for human-animal intervention research

  |  |  |
  | --- | --- |
  | Item Type | Journal Article |
  | Author | Cory M. Smith |
  | Author | Katharine Weimann |
  | Author | Madison Widick |
  | Author | Tamara Merritt |
  | Author | Hannah Christensen |
  | Author | Matthew Siegel |
  | Author | Zhaoxing Pan |
  | Author | Robin L. Gabriels |
  | Date | 2025 |
  | URL | https://www.scopus.com/inward/record.uri?eid=2-s2.0-105001331365&doi=10.1016%2fj.mex.2025.103284&partnerID=40&md5=df58ce994cefd9b1d728079174184972 |
  | Extra | Type: Article |
  | Volume | 14 |
  | Publication | MethodsX |
  | DOI | 10.1016/j.mex.2025.103284 |
  | Date Added | 19/06/2025, 15:40:13 |
  | Modified | 19/06/2025, 15:40:13 |

  ### Notes:

  - Cited by: 0
- ## Living and Robotic Dogs as Elicitors of Social Communication Behavior and Regulated Emotional Responding in Individuals with Autism and Severe Language Delay: A Preliminary Comparative Study

  |  |  |
  | --- | --- |
  | Item Type | Journal Article |
  | Author | Karine Silva |
  | Author | Mariely Lima |
  | Author | André Santos-Magalhães |
  | Author | Carla Fafiães |
  | Author | Liliana de Sousa |
  | Date | 2019 |
  | URL | https://www.scopus.com/inward/record.uri?eid=2-s2.0-85060337396&doi=10.1080%2f08927936.2019.1550278&partnerID=40&md5=d6a73cf0bb84108c248c09404f6f7c71 |
  | Extra | Type: Article |
  | Volume | 32 |
  | Pages | 23 – 33 |
  | Publication | Anthrozoos |
  | DOI | 10.1080/08927936.2019.1550278 |
  | Issue | 1 |
  | Date Added | 19/06/2025, 15:40:14 |
  | Modified | 19/06/2025, 15:40:14 |

  ### Notes:

  - Cited by: 20
- ## Recognizing and Mitigating Canine Stress in Human–Canine Interaction Research: Proposed Guidelines

  |  |  |
  | --- | --- |
  | Item Type | Journal Article |
  | Author | Simone B. Sidel |
  | Author | Jaci Gandenberger |
  | Author | Kerry Murphy |
  | Author | Kevin N. Morris |
  | Date | 2025 |
  | URL | https://www.scopus.com/inward/record.uri?eid=2-s2.0-105007748501&doi=10.3390%2fani15111665&partnerID=40&md5=aa35210d9d3f696abac5c4c6f272bbc1 |
  | Extra | Type: Article |
  | Volume | 15 |
  | Publication | Animals |
  | DOI | 10.3390/ani15111665 |
  | Issue | 11 |
  | Date Added | 11/07/2025, 14:08:22 |
  | Modified | 11/07/2025, 14:08:22 |

  ### Notes:

  - Cited by: 0
- ## Animal-based measures for welfare assessment

  |  |  |
  | --- | --- |
  | Item Type | Journal Article |
  | Author | Agostino Sevi |
  | Date | 2009 |
  | URL | https://www.scopus.com/inward/record.uri?eid=2-s2.0-80051564705&doi=10.4081%2fijas.2009.s2.904&partnerID=40&md5=8ececf7c1013d3d0df517972073d05e2 |
  | Extra | Type: Article |
  | Volume | 8 |
  | Pages | 904 – 911 |
  | Publication | Italian Journal of Animal Science |
  | DOI | 10.4081/ijas.2009.s2.904 |
  | Issue | SUPPL. 2 |
  | Date Added | 11/07/2025, 14:08:23 |
  | Modified | 11/07/2025, 14:08:23 |

  ### Notes:

  - Cited by: 14; All Open Access, Gold Open Access, Green Open Access
- ## Inside the Interaction: Contact With Familiar Humans Modulates Heart Rate Variability in Horses

  |  |  |
  | --- | --- |
  | Item Type | Journal Article |
  | Author | Chiara Scopa |
  | Author | Alberto Greco |
  | Author | Laura Contalbrigo |
  | Author | Elisabetta Fratini |
  | Author | Antonio Lanatà |
  | Author | Enzo Pasquale Scilingo |
  | Author | Paolo Baragli |
  | Date | 2020 |
  | URL | https://www.scopus.com/inward/record.uri?eid=2-s2.0-85097604207&doi=10.3389%2ffvets.2020.582759&partnerID=40&md5=f06b1eb984b6009708fac139f34477c6 |
  | Extra | Type: Article |
  | Volume | 7 |
  | Publication | Frontiers in Veterinary Science |
  | DOI | 10.3389/fvets.2020.582759 |
  | Date Added | 19/06/2025, 15:40:13 |
  | Modified | 19/06/2025, 15:40:13 |

  ### Notes:

  - Cited by: 18; All Open Access, Gold Open Access, Green Open Access
- ## Minor immediate effects of a dog on children's reading performance and physiology

  |  |  |
  | --- | --- |
  | Item Type | Journal Article |
  | Author | Lisa Schretzmayer |
  | Author | Kurt Kotrschal |
  | Author | Andrea Beetz |
  | Date | 2017 |
  | URL | https://www.scopus.com/inward/record.uri?eid=2-s2.0-85038833692&doi=10.3389%2ffvets.2017.00090&partnerID=40&md5=81fb748e4c5add107a71a7d43772c2c1 |
  | Extra | Type: Article |
  | Volume | 4 |
  | Publication | Frontiers in Veterinary Science |
  | DOI | 10.3389/fvets.2017.00090 |
  | Issue | JUN |
  | Date Added | 19/06/2025, 15:40:12 |
  | Modified | 19/06/2025, 15:40:12 |

  ### Notes:

  - Cited by: 20; All Open Access, Gold Open Access, Green Open Access
- ## Minor immediate effects of a dog on children's reading performance and physiology

  |  |  |
  | --- | --- |
  | Item Type | Journal Article |
  | Author | Lisa Schretzmayer |
  | Author | Kurt Kotrschal |
  | Author | Andrea Beetz |
  | Date | 2017 |
  | URL | https://www.scopus.com/inward/record.uri?eid=2-s2.0-85038833692&doi=10.3389%2ffvets.2017.00090&partnerID=40&md5=81fb748e4c5add107a71a7d43772c2c1 |
  | Extra | Type: Article |
  | Volume | 4 |
  | Publication | Frontiers in Veterinary Science |
  | DOI | 10.3389/fvets.2017.00090 |
  | Issue | JUN |
  | Date Added | 11/07/2025, 14:08:22 |
  | Modified | 11/07/2025, 14:08:22 |

  ### Notes:

  - Cited by: 20; All Open Access, Gold Open Access, Green Open Access
- ## Emerging cardiovascular risk research: Impact of pets on cardiovascular risk prevention

  |  |  |
  | --- | --- |
  | Item Type | Journal Article |
  | Author | Pamela J. Schreiner |
  | Date | 2016 |
  | URL | https://www.scopus.com/inward/record.uri?eid=2-s2.0-85007158524&doi=10.1007%2fs12170-016-0489-2&partnerID=40&md5=3470048445ac7feb36ef60f69135691c |
  | Extra | Type: Article |
  | Volume | 10 |
  | Pages | 1 – 8 |
  | Publication | Current Cardiovascular Risk Reports |
  | DOI | 10.1007/s12170-016-0489-2 |
  | Issue | 2 |
  | Date Added | 19/06/2025, 15:40:12 |
  | Modified | 19/06/2025, 15:40:12 |

  ### Notes:

  - Cited by: 34; All Open Access, Green Open Access
- ## Emerging cardiovascular risk research: Impact of pets on cardiovascular risk prevention

  |  |  |
  | --- | --- |
  | Item Type | Journal Article |
  | Author | Pamela J. Schreiner |
  | Date | 2016 |
  | URL | https://www.scopus.com/inward/record.uri?eid=2-s2.0-85007158524&doi=10.1007%2fs12170-016-0489-2&partnerID=40&md5=3470048445ac7feb36ef60f69135691c |
  | Extra | Type: Article |
  | Volume | 10 |
  | Pages | 1 – 8 |
  | Publication | Current Cardiovascular Risk Reports |
  | DOI | 10.1007/s12170-016-0489-2 |
  | Issue | 2 |
  | Date Added | 20/06/2025, 09:37:47 |
  | Modified | 20/06/2025, 09:37:47 |

  ### Notes:

  - Cited by: 34; All Open Access, Green Open Access
- ## Emerging cardiovascular risk research: Impact of pets on cardiovascular risk prevention

  |  |  |
  | --- | --- |
  | Item Type | Journal Article |
  | Author | Pamela J. Schreiner |
  | Date | 2016 |
  | URL | https://www.scopus.com/inward/record.uri?eid=2-s2.0-85007158524&doi=10.1007%2fs12170-016-0489-2&partnerID=40&md5=3470048445ac7feb36ef60f69135691c |
  | Extra | Type: Article |
  | Volume | 10 |
  | Pages | 1 – 8 |
  | Publication | Current Cardiovascular Risk Reports |
  | DOI | 10.1007/s12170-016-0489-2 |
  | Issue | 2 |
  | Date Added | 11/07/2025, 14:08:22 |
  | Modified | 11/07/2025, 14:08:22 |

  ### Notes:

  - Cited by: 34; All Open Access, Green Open Access
- ## Regular Positive Human Contacts Do Not Improve Pigs' Response to a Lipopolysaccharide Immune Challenge

  |  |  |
  | --- | --- |
  | Item Type | Journal Article |
  | Author | Oceane Schmitt |
  | Author | Christian Knecht |
  | Author | Birgit Sobczak |
  | Author | Hana Volkmann |
  | Author | Ulrike Gimsa |
  | Author | Jean-Loup Rault |
  | Date | 2025 |
  | URL | https://www.scopus.com/inward/record.uri?eid=2-s2.0-105002556740&doi=10.1159%2f000544748&partnerID=40&md5=badd85ca19fd413b0425f1a09a95e40b |
  | Extra | Type: Article |
  | Volume | 32 |
  | Pages | 83 – 93 |
  | Publication | NeuroImmunoModulation |
  | DOI | 10.1159/000544748 |
  | Issue | 1 |
  | Date Added | 11/07/2025, 14:08:22 |
  | Modified | 11/07/2025, 14:08:22 |

  ### Notes:

  - Cited by: 0
- ## Behavioral and physiological responses of crossbred Holstein-Zebu cows and their interaction with the milker in two milking systems

  |  |  |
  | --- | --- |
  | Item Type | Journal Article |
  | Author | H.P. Santos |
  | Author | A.L.O. Aiura |
  | Author | G.A.M. Gonçalves |
  | Author | F.S. Aiura |
  | Author | M. Ferreira |
  | Author | I.C.S. Alves |
  | Author | B.F.C. Cunha |
  | Date | 2019 |
  | URL | https://www.scopus.com/inward/record.uri?eid=2-s2.0-85065479837&doi=10.31893%2f2318-1265jabb.v7n1p1-5&partnerID=40&md5=7bc8e0533338ab11924c22d0a6677b58 |
  | Extra | Type: Article |
  | Volume | 7 |
  | Pages | 1 – 5 |
  | Publication | Journal of Animal Behaviour and Biometeorology |
  | DOI | 10.31893/2318-1265jabb.v7n1p1-5 |
  | Issue | 1 |
  | Date Added | 19/06/2025, 15:40:13 |
  | Modified | 19/06/2025, 15:40:13 |

  ### Notes:

  - Cited by: 4; All Open Access, Bronze Open Access
- ## A Preliminary Assessment of Equine Affect in Equine-Assisted Services

  |  |  |
  | --- | --- |
  | Item Type | Journal Article |
  | Author | Christine Rudd |
  | Author | Emma Pasiuk |
  | Author | Nichole Anderson |
  | Author | Nathan Hall |
  | Author | Robin Foster |
  | Author | Katy Schroeder |
  | Date | 2024 |
  | URL | https://www.scopus.com/inward/record.uri?eid=2-s2.0-85190283884&doi=10.1080%2f08927936.2024.2333163&partnerID=40&md5=c3f9d45c0cdd9ea1c2c073a5e4a34094 |
  | Extra | Type: Article |
  | Volume | 37 |
  | Pages | 501 – 518 |
  | Publication | Anthrozoos |
  | DOI | 10.1080/08927936.2024.2333163 |
  | Issue | 3 |
  | Date Added | 19/06/2025, 15:40:13 |
  | Modified | 19/06/2025, 15:40:13 |

  ### Notes:

  - Cited by: 2
- ## The Effect of Mental Activation of One's Pet Dog on Stress Reactivity

  |  |  |
  | --- | --- |
  | Item Type | Journal Article |
  | Author | Kerri E. Rodriguez |
  | Author | Dan J. Graham |
  | Author | Rachel G. Lucas-Thompson |
  | Date | 2023 |
  | URL | https://www.scopus.com/inward/record.uri?eid=2-s2.0-85176393122&doi=10.3390%2fijerph20216995&partnerID=40&md5=c14c5420d01df42e974dfc9b98a62b80 |
  | Extra | Type: Article |
  | Volume | 20 |
  | Publication | International journal of environmental research and public health |
  | DOI | 10.3390/ijerph20216995 |
  | Issue | 21 |
  | Date Added | 19/06/2025, 15:40:13 |
  | Modified | 19/06/2025, 15:40:13 |

  ### Notes:

  - Cited by: 0; All Open Access, Gold Open Access

  ### Attachments

  - PDF
- ## The effect of a service dog on salivary cortisol awakening response in a military population with posttraumatic stress disorder (PTSD)

  |  |  |
  | --- | --- |
  | Item Type | Journal Article |
  | Author | Kerri E. Rodriguez |
  | Author | Crystal I. Bryce |
  | Author | Douglas A. Granger |
  | Author | Marguerite E. O'Haire |
  | Date | 2018 |
  | URL | https://www.scopus.com/inward/record.uri?eid=2-s2.0-85055629186&doi=10.1016%2fj.psyneuen.2018.04.026&partnerID=40&md5=7a20e619aeb0bb9b504118966eb4787e |
  | Extra | Type: Article |
  | Volume | 98 |
  | Pages | 202 – 210 |
  | Publication | Psychoneuroendocrinology |
  | DOI | 10.1016/j.psyneuen.2018.04.026 |
  | Date Added | 11/07/2025, 14:08:23 |
  | Modified | 11/07/2025, 14:08:23 |

  ### Notes:

  - Cited by: 60; All Open Access, Green Open Access
- ## Validation of the feeding test as an index of fear in farmed blue (Alopex lagopus) and silver foxes (Vulpes vulpes)

  |  |  |
  | --- | --- |
  | Item Type | Journal Article |
  | Author | Teppo Rekilä |
  | Author | Mikko Harri |
  | Author | Leena Ahola |
  | Date | 1997 |
  | URL | https://www.scopus.com/inward/record.uri?eid=2-s2.0-0030804155&doi=10.1016%2fS0031-9384%2897%2900241-2&partnerID=40&md5=974eb1ba743b783c950056a714474a0b |
  | Extra | Type: Article |
  | Volume | 62 |
  | Pages | 805 – 810 |
  | Publication | Physiology and Behavior |
  | DOI | 10.1016/S0031-9384(97)00241-2 |
  | Issue | 4 |
  | Date Added | 11/07/2025, 14:08:22 |
  | Modified | 11/07/2025, 14:08:22 |

  ### Notes:

  - Cited by: 40
- ## Relationship between hyponeophagia and adrenal cortex function in farmed foxes

  |  |  |
  | --- | --- |
  | Item Type | Journal Article |
  | Author | Teppo Rekilä |
  | Author | Mikko Harri |
  | Author | Liisa Jalkanen |
  | Author | Jaakko Mononen |
  | Date | 1998 |
  | URL | https://www.scopus.com/inward/record.uri?eid=2-s2.0-0032448206&doi=10.1016%2fS0031-9384%2898%2900232-7&partnerID=40&md5=278a20b379faf8acd1c4526357cce1b2 |
  | Extra | Type: Article |
  | Volume | 65 |
  | Pages | 779 – 783 |
  | Publication | Physiology and Behavior |
  | DOI | 10.1016/S0031-9384(98)00232-7 |
  | Issue | 4-5 |
  | Date Added | 11/07/2025, 14:08:23 |
  | Modified | 11/07/2025, 14:08:23 |

  ### Notes:

  - Cited by: 16
- ## Grazing intensity and associated frequency of human contact, and horn status, influence activity on pasture, physiological pre-slaughter reactions and meat quality in beef heifers

  |  |  |
  | --- | --- |
  | Item Type | Journal Article |
  | Author | Anna-Maria Reiche |
  | Author | Paolo Silacci |
  | Author | Frigga Dohme-Meier |
  | Author | E.M. Claudia Terlouw |
  | Date | 2024 |
  | URL | https://www.scopus.com/inward/record.uri?eid=2-s2.0-85205594750&doi=10.1016%2fj.livsci.2024.105578&partnerID=40&md5=baab58b0b4d07cacecab181805ebbc37 |
  | Extra | Type: Article |
  | Volume | 289 |
  | Publication | Livestock Science |
  | DOI | 10.1016/j.livsci.2024.105578 |
  | Date Added | 19/06/2025, 15:40:12 |
  | Modified | 19/06/2025, 15:40:12 |

  ### Notes:

  - Cited by: 0; All Open Access, Hybrid Gold Open Access
- ## Behavioural and physiological assessment of positive and negative emotion in sheep

  |  |  |
  | --- | --- |
  | Item Type | Journal Article |
  | Author | Nadine Reefmann |
  | Author | Beat Wechsler |
  | Author | Lorenz Gygax |
  | Date | 2009 |
  | URL | https://www.scopus.com/inward/record.uri?eid=2-s2.0-69249206508&doi=10.1016%2fj.anbehav.2009.06.015&partnerID=40&md5=7694670c63f1c82cb75fc8274049398e |
  | Extra | Type: Article |
  | Volume | 78 |
  | Pages | 651 – 659 |
  | Publication | Animal Behaviour |
  | DOI | 10.1016/j.anbehav.2009.06.015 |
  | Issue | 3 |
  | Date Added | 19/06/2025, 15:40:12 |
  | Modified | 19/06/2025, 15:40:12 |

  ### Notes:

  - Cited by: 133
- ## Housing induced mood modulates reactions to emotional stimuli in sheep

  |  |  |
  | --- | --- |
  | Item Type | Journal Article |
  | Author | Nadine Reefmann |
  | Author | Thomas Muehlemann |
  | Author | Beat Wechsler |
  | Author | Lorenz Gygax |
  | Date | 2012 |
  | URL | https://www.scopus.com/inward/record.uri?eid=2-s2.0-84856121131&doi=10.1016%2fj.applanim.2011.12.007&partnerID=40&md5=94ab7151d68a5c8e393bec4638970523 |
  | Extra | Number: 2-4 Type: Article |
  | Volume | 136 |
  | Pages | 146 – 155 |
  | Publication | Applied Animal Behaviour Science |
  | DOI | 10.1016/j.applanim.2011.12.007 |
  | Issue | 2-4 |
  | Date Added | 28/07/2025, 12:48:52 |
  | Modified | 28/07/2025, 12:48:52 |

  ### Notes:

  - Cited by: 30
- ## Behavioural and physiological assessment of positive and negative emotion in sheep

  |  |  |
  | --- | --- |
  | Item Type | Journal Article |
  | Author | Nadine Reefmann |
  | Author | Beat Wechsler |
  | Author | Lorenz Gygax |
  | Date | 2009 |
  | URL | https://www.scopus.com/inward/record.uri?eid=2-s2.0-69249206508&doi=10.1016%2fj.anbehav.2009.06.015&partnerID=40&md5=7694670c63f1c82cb75fc8274049398e |
  | Extra | Number: 3 Type: Article |
  | Volume | 78 |
  | Pages | 651 – 659 |
  | Publication | Animal Behaviour |
  | DOI | 10.1016/j.anbehav.2009.06.015 |
  | Issue | 3 |
  | Date Added | 28/07/2025, 12:48:52 |
  | Modified | 28/07/2025, 12:48:52 |

  ### Notes:

  - Cited by: 133
- ## The effect of contact with conspecifics and humans on calves' behaviour and stress responses

  |  |  |
  | --- | --- |
  | Item Type | Journal Article |
  | Author | Satu Raussi |
  | Author | B.J. Lensink |
  | Author | A. Boissy |
  | Author | M. Pyykkönen |
  | Author | I. Veissier |
  | Date | 2003 |
  | URL | https://www.scopus.com/inward/record.uri?eid=2-s2.0-0037738857&partnerID=40&md5=5aff574efbc79aec3aafb72ab9866462 |
  | Extra | Type: Article |
  | Volume | 12 |
  | Pages | 191 – 203 |
  | Publication | Animal Welfare |
  | Issue | 2 |
  | Date Added | 19/06/2025, 15:40:12 |
  | Modified | 19/06/2025, 15:40:12 |

  ### Notes:

  - Cited by: 39
- ## The effect of contact with conspecifics and humans on calves' behaviour and stress responses

  |  |  |
  | --- | --- |
  | Item Type | Journal Article |
  | Author | Satu Raussi |
  | Author | B.J. Lensink |
  | Author | A. Boissy |
  | Author | M. Pyykkönen |
  | Author | I. Veissier |
  | Date | 2003 |
  | URL | https://www.scopus.com/inward/record.uri?eid=2-s2.0-0037738857&partnerID=40&md5=5aff574efbc79aec3aafb72ab9866462 |
  | Extra | Type: Article |
  | Volume | 12 |
  | Pages | 191 – 203 |
  | Publication | Animal Welfare |
  | Issue | 2 |
  | Date Added | 11/07/2025, 14:08:22 |
  | Modified | 11/07/2025, 14:08:22 |

  ### Notes:

  - Cited by: 39
- ## Gentle abdominal stroking (‘belly rubbing’) of pigs by a human reduces EEG total power and increases EEG frequencies

  |  |  |
  | --- | --- |
  | Item Type | Journal Article |
  | Author | J.-L. Rault |
  | Author | S. Truong |
  | Author | L. Hemsworth |
  | Author | M. Le Chevoir |
  | Author | S. Bauquier |
  | Author | A. Lai |
  | Date | 2019 |
  | Archive | Scopus |
  | URL | https://www.scopus.com/inward/record.uri?eid=2-s2.0-85064855069&doi=10.1016%2fj.bbr.2019.04.006&partnerID=40&md5=b159d0e297e2e0b92a358daaea7515eb |
  | Volume | 374 |
  | Publication | Behavioural Brain Research |
  | DOI | 10.1016/j.bbr.2019.04.006 |
  | Date Added | 11/07/2025, 11:22:51 |
  | Modified | 11/07/2025, 11:22:51 |

  ### Notes:

  - Export Date: 11 July 2025; Cited By: 13
- ## Ground-based adaptive horsemanship lessons for veterans with post-traumatic stress disorder: a randomized controlled pilot study

  |  |  |
  | --- | --- |
  | Item Type | Journal Article |
  | Author | Ellen M. Rankins |
  | Author | Andrea Quinn |
  | Author | Kenneth H. McKeever |
  | Author | Karyn Malinowski |
  | Date | 2024 |
  | URL | https://www.scopus.com/inward/record.uri?eid=2-s2.0-85195577643&doi=10.3389%2ffpsyt.2024.1390212&partnerID=40&md5=0acc3fb9def08aefc6789e1d542e0c04 |
  | Extra | Type: Article |
  | Volume | 15 |
  | Publication | Frontiers in Psychiatry |
  | DOI | 10.3389/fpsyt.2024.1390212 |
  | Date Added | 20/06/2025, 09:37:48 |
  | Modified | 20/06/2025, 09:37:48 |

  ### Notes:

  - Cited by: 1; All Open Access, Gold Open Access, Green Open Access
- ## Ground-based adaptive horsemanship lessons for veterans with post-traumatic stress disorder: a randomized controlled pilot study

  |  |  |
  | --- | --- |
  | Item Type | Journal Article |
  | Author | Ellen M. Rankins |
  | Author | Andrea Quinn |
  | Author | Kenneth H. McKeever |
  | Author | Karyn Malinowski |
  | Date | 2024 |
  | URL | https://www.scopus.com/inward/record.uri?eid=2-s2.0-85195577643&doi=10.3389%2ffpsyt.2024.1390212&partnerID=40&md5=0acc3fb9def08aefc6789e1d542e0c04 |
  | Extra | Type: Article |
  | Volume | 15 |
  | Publication | Frontiers in Psychiatry |
  | DOI | 10.3389/fpsyt.2024.1390212 |
  | Date Added | 11/07/2025, 14:08:22 |
  | Modified | 11/07/2025, 14:08:22 |

  ### Notes:

  - Cited by: 1; All Open Access, Gold Open Access, Green Open Access
- ## Ground-based adaptive horsemanship lessons for veterans with post-traumatic stress disorder: a randomized controlled pilot study

  |  |  |
  | --- | --- |
  | Item Type | Journal Article |
  | Author | Ellen M. Rankins |
  | Author | Andrea Quinn |
  | Author | Kenneth H. McKeever |
  | Author | Karyn Malinowski |
  | Date | 2024 |
  | URL | https://www.scopus.com/inward/record.uri?eid=2-s2.0-85195577643&doi=10.3389%2ffpsyt.2024.1390212&partnerID=40&md5=0acc3fb9def08aefc6789e1d542e0c04 |
  | Extra | Type: Article |
  | Volume | 15 |
  | Publication | Frontiers in Psychiatry |
  | DOI | 10.3389/fpsyt.2024.1390212 |
  | Date Added | 28/07/2025, 12:48:52 |
  | Modified | 28/07/2025, 12:48:52 |

  ### Notes:

  - Cited by: 1; All Open Access, Gold Open Access, Green Open Access
- ## Neural Basis of Categorical Representations of Animal Body Silhouettes

  |  |  |
  | --- | --- |
  | Item Type | Journal Article |
  | Author | Y. Pu |
  | Author | S. Han |
  | Date | 2025 |
  | Archive | Scopus |
  | URL | https://www.scopus.com/inward/record.uri?eid=2-s2.0-85199531216&doi=10.1007%2fs12264-024-01268-1&partnerID=40&md5=f7363b1f1408f3535d7460bf3c25b475 |
  | Volume | 41 |
  | Pages | 211-223 |
  | Publication | Neuroscience Bulletin |
  | DOI | 10.1007/s12264-024-01268-1 |
  | Issue | 2 |
  | Date Added | 11/07/2025, 11:22:51 |
  | Modified | 11/07/2025, 11:22:51 |

  ### Notes:

  - Export Date: 11 July 2025; Cited By: 0
- ## Effects of Human–Dog Interactions on Salivary Oxytocin Concentrations and Heart Rate Variability: A Four-Condition Cross-Over Trial

  |  |  |
  | --- | --- |
  | Item Type | Journal Article |
  | Author | Lauren Powell |
  | Author | Kate M. Edwards |
  | Author | Scott Michael |
  | Author | Paul McGreevy |
  | Author | Adrian Bauman |
  | Author | Adam J. Guastella |
  | Author | Bradley Drayton |
  | Author | Emmanuel Stamatakis |
  | Date | 2020 |
  | URL | https://www.scopus.com/inward/record.uri?eid=2-s2.0-85077978460&doi=10.1080%2f08927936.2020.1694310&partnerID=40&md5=da7f3f4219e70c7464677432a178b7f4 |
  | Extra | Type: Article |
  | Volume | 33 |
  | Pages | 37 – 52 |
  | Publication | Anthrozoos |
  | DOI | 10.1080/08927936.2020.1694310 |
  | Issue | 1 |
  | Date Added | 19/06/2025, 15:40:13 |
  | Modified | 19/06/2025, 15:40:13 |

  ### Notes:

  - Cited by: 25
- ## Canine endogenous oxytocin responses to dog-walking and affiliative human–dog interactions

  |  |  |
  | --- | --- |
  | Item Type | Journal Article |
  | Author | Lauren Powell |
  | Author | Kate M. Edwards |
  | Author | Adrian Bauman |
  | Author | Adam J. Guastella |
  | Author | Bradley Drayton |
  | Author | Emmanuel Stamatakis |
  | Author | Paul McGreevy |
  | Date | 2019 |
  | URL | https://www.scopus.com/inward/record.uri?eid=2-s2.0-85063459692&doi=10.3390%2fani9020051&partnerID=40&md5=afa91ee2895ce4e5fddbcfdfcb98773b |
  | Extra | Type: Article |
  | Volume | 9 |
  | Publication | Animals |
  | DOI | 10.3390/ani9020051 |
  | Issue | 2 |
  | Date Added | 20/06/2025, 09:37:47 |
  | Modified | 20/06/2025, 09:37:47 |

  ### Notes:

  - Cited by: 20; All Open Access, Gold Open Access, Green Open Access
- ## Effects of Human–Dog Interactions on Salivary Oxytocin Concentrations and Heart Rate Variability: A Four-Condition Cross-Over Trial

  |  |  |
  | --- | --- |
  | Item Type | Journal Article |
  | Author | Lauren Powell |
  | Author | Kate M. Edwards |
  | Author | Scott Michael |
  | Author | Paul McGreevy |
  | Author | Adrian Bauman |
  | Author | Adam J. Guastella |
  | Author | Bradley Drayton |
  | Author | Emmanuel Stamatakis |
  | Date | 2020 |
  | URL | https://www.scopus.com/inward/record.uri?eid=2-s2.0-85077978460&doi=10.1080%2f08927936.2020.1694310&partnerID=40&md5=da7f3f4219e70c7464677432a178b7f4 |
  | Extra | Type: Article |
  | Volume | 33 |
  | Pages | 37 – 52 |
  | Publication | Anthrozoos |
  | DOI | 10.1080/08927936.2020.1694310 |
  | Issue | 1 |
  | Date Added | 20/06/2025, 09:37:48 |
  | Modified | 20/06/2025, 09:37:48 |

  ### Notes:

  - Cited by: 25
- ## Exploring the Domestication Syndrome Hypothesis in Dogs: Pigmentation Does Not Predict Cortisol Levels

  |  |  |
  | --- | --- |
  | Item Type | Journal Article |
  | Author | JoAnna M. Platzer |
  | Author | Lisa M. Gunter |
  | Author | Erica N. Feuerbacher |
  | Date | 2023 |
  | URL | https://www.scopus.com/inward/record.uri?eid=2-s2.0-85173842893&doi=10.3390%2fani13193095&partnerID=40&md5=94f99639ea3b2ed97dbe3d98b4c53b2a |
  | Extra | Type: Article |
  | Volume | 13 |
  | Publication | Animals |
  | DOI | 10.3390/ani13193095 |
  | Issue | 19 |
  | Date Added | 11/07/2025, 14:08:23 |
  | Modified | 11/07/2025, 14:08:23 |

  ### Notes:

  - Cited by: 0; All Open Access, Gold Open Access, Green Open Access
- ## Serum Oxytocin, Cortisol and Social Behavior in Calves: A Study in the Impossible Task Paradigm

  |  |  |
  | --- | --- |
  | Item Type | Journal Article |
  | Author | Claudia Pinelli |
  | Author | Anna Scandurra |
  | Author | Vincenzo Mastellone |
  | Author | Piera Iommelli |
  | Author | Nadia Musco |
  | Author | Maria Elena Pero |
  | Author | Alfredo Di Lucrezia |
  | Author | Daria Lotito |
  | Author | Raffaella Tudisco |
  | Author | Biagio D’Aniello |
  | Author | Federico Infascelli |
  | Author | Pietro Lombardi |
  | Date | 2023 |
  | URL | https://www.scopus.com/inward/record.uri?eid=2-s2.0-85148884561&doi=10.3390%2fani13040646&partnerID=40&md5=21efc5a7eaeadbe2a87e379dfb01bc51 |
  | Extra | Type: Article |
  | Volume | 13 |
  | Publication | Animals |
  | DOI | 10.3390/ani13040646 |
  | Issue | 4 |
  | Date Added | 20/06/2025, 09:37:48 |
  | Modified | 20/06/2025, 09:37:48 |

  ### Notes:

  - Cited by: 1; All Open Access, Gold Open Access, Green Open Access
- ## Serum Oxytocin, Cortisol and Social Behavior in Calves: A Study in the Impossible Task Paradigm

  |  |  |
  | --- | --- |
  | Item Type | Journal Article |
  | Author | Claudia Pinelli |
  | Author | Anna Scandurra |
  | Author | Vincenzo Mastellone |
  | Author | Piera Iommelli |
  | Author | Nadia Musco |
  | Author | Maria Elena Pero |
  | Author | Alfredo Di Lucrezia |
  | Author | Daria Lotito |
  | Author | Raffaella Tudisco |
  | Author | Biagio D’Aniello |
  | Author | Federico Infascelli |
  | Author | Pietro Lombardi |
  | Date | 2023 |
  | URL | https://www.scopus.com/inward/record.uri?eid=2-s2.0-85148884561&doi=10.3390%2fani13040646&partnerID=40&md5=21efc5a7eaeadbe2a87e379dfb01bc51 |
  | Extra | Type: Article |
  | Volume | 13 |
  | Publication | Animals |
  | DOI | 10.3390/ani13040646 |
  | Issue | 4 |
  | Date Added | 11/07/2025, 14:08:23 |
  | Modified | 11/07/2025, 14:08:23 |

  ### Notes:

  - Cited by: 1; All Open Access, Gold Open Access, Green Open Access
- ## Dog–Owner Relationship and Its Association with Social Cognition in French Bulldogs

  |  |  |
  | --- | --- |
  | Item Type | Journal Article |
  | Author | Lara Peterca |
  | Author | Elena Gobbo |
  | Author | Manja Zupan Šemrov |
  | Date | 2025 |
  | URL | https://www.scopus.com/inward/record.uri?eid=2-s2.0-85214464513&doi=10.3390%2fani15010017&partnerID=40&md5=2df9125faa1b13efa5d1317b2d818c82 |
  | Extra | Type: Article |
  | Volume | 15 |
  | Publication | Animals |
  | DOI | 10.3390/ani15010017 |
  | Issue | 1 |
  | Date Added | 20/06/2025, 09:37:47 |
  | Modified | 20/06/2025, 09:37:47 |

  ### Notes:

  - Cited by: 0
- ## Animal Visitation Program (AVP) Reduces Cortisol Levels of University Students: A Randomized Controlled Trial

  |  |  |
  | --- | --- |
  | Item Type | Journal Article |
  | Author | Patricia Pendry |
  | Author | Jaymie L. Vandagriff |
  | Date | 2019 |
  | URL | https://www.scopus.com/inward/record.uri?eid=2-s2.0-85069723263&doi=10.1177%2f2332858419852592&partnerID=40&md5=b4fd1322caac9f1ffe9bee36d6ba91bd |
  | Extra | Type: Article |
  | Volume | 5 |
  | Publication | AERA Open |
  | DOI | 10.1177/2332858419852592 |
  | Issue | 2 |
  | Date Added | 11/07/2025, 14:08:23 |
  | Modified | 11/07/2025, 14:08:23 |

  ### Notes:

  - Cited by: 70; All Open Access, Gold Open Access
- ## Limited Short-Term Effects of Tactile Stimulation on the Welfare of Newborn Nellore Calves

  |  |  |
  | --- | --- |
  | Item Type | Journal Article |
  | Author | Mariana Parra Cerezo |
  | Author | Victor Brusin |
  | Author | Pedro Henrique Esteves Trindade |
  | Author | Adalinda Hernández |
  | Author | Jens Jung |
  | Author | Charlotte Berg |
  | Author | Mateus José Rodrigues Paranhos da Costa |
  | Date | 2025 |
  | URL | https://www.scopus.com/inward/record.uri?eid=2-s2.0-105003694860&doi=10.3390%2fvetsci12040393&partnerID=40&md5=d743c10f6a2311e7719736ac473a99c6 |
  | Extra | Type: Article |
  | Volume | 12 |
  | Publication | Veterinary Sciences |
  | DOI | 10.3390/vetsci12040393 |
  | Issue | 4 |
  | Date Added | 19/06/2025, 15:40:13 |
  | Modified | 19/06/2025, 15:40:13 |

  ### Notes:

  - Cited by: 0
- ## Ethological and physiological parameters assessment in donkeys used in animal assisted interventions

  |  |  |
  | --- | --- |
  | Item Type | Journal Article |
  | Author | Michele Panzera |
  | Author | Daniela Alberghina |
  | Author | Alessandra Statelli |
  | Date | 2020 |
  | URL | https://www.scopus.com/inward/record.uri?eid=2-s2.0-85092541771&doi=10.3390%2fani10101867&partnerID=40&md5=01eeaf71b4802ed5c519ab136a25412f |
  | Extra | Type: Article |
  | Volume | 10 |
  | Pages | 1 – 24 |
  | Publication | Animals |
  | DOI | 10.3390/ani10101867 |
  | Issue | 10 |
  | Date Added | 19/06/2025, 15:40:13 |
  | Modified | 19/06/2025, 15:40:13 |

  ### Notes:

  - Cited by: 10; All Open Access, Green Open Access
- ## Replication pilot trial of therapeutic horseback riding and cortisol collection with children on the autism spectrum

  |  |  |
  | --- | --- |
  | Item Type | Journal Article |
  | Author | Zhaoxing Pan |
  | Author | Douglas A. Granger |
  | Author | Noémie A. Guérin |
  | Author | Amy Shoffner |
  | Author | Robin L. Gabriels |
  | Date | 2019 |
  | URL | https://www.scopus.com/inward/record.uri?eid=2-s2.0-85060229883&doi=10.3389%2ffvets.2018.00312&partnerID=40&md5=8ef233ebb0360acad5318ecea4668983 |
  | Extra | Type: Article |
  | Volume | 5 |
  | Publication | Frontiers in Veterinary Science |
  | DOI | 10.3389/fvets.2018.00312 |
  | Issue | JAN |
  | Date Added | 11/07/2025, 14:08:23 |
  | Modified | 11/07/2025, 14:08:23 |

  ### Notes:

  - Cited by: 34; All Open Access, Gold Open Access, Green Open Access
- ## Electrocardiogram Measurement and Emotion Estimation of Working Dogs

  |  |  |
  | --- | --- |
  | Item Type | Journal Article |
  | Author | Kazunori Ohno |
  | Author | Kotaro Sato |
  | Author | Ryunosuke Hamada |
  | Author | Takatomi Kubo |
  | Author | Kazushi Ikeda |
  | Author | Miho Nagasawa |
  | Author | Takefumi Kikusui |
  | Author | Sandeep Kumar Nayak |
  | Author | Shotaro Kojima |
  | Author | Satoshi Tadokoro |
  | Date | 2022 |
  | URL | https://www.scopus.com/inward/record.uri?eid=2-s2.0-85123683280&doi=10.1109%2fLRA.2022.3145590&partnerID=40&md5=2d7db48c05ae37bb11a88247d9873eda |
  | Extra | Type: Article |
  | Volume | 7 |
  | Pages | 4047 – 4054 |
  | Publication | IEEE Robotics and Automation Letters |
  | DOI | 10.1109/LRA.2022.3145590 |
  | Issue | 2 |
  | Date Added | 19/06/2025, 15:40:13 |
  | Modified | 19/06/2025, 15:40:13 |

  ### Notes:

  - Cited by: 7; All Open Access, Hybrid Gold Open Access
- ## Influence of Interactive Behaviors Induced by a Therapy Dog and Her Handler on the Physiology of Residents in Nursing Homes: An Exploratory Study

  |  |  |
  | --- | --- |
  | Item Type | Journal Article |
  | Author | Anne Nilsson |
  | Author | Lena Lidfors |
  | Author | Anette Wichman |
  | Author | Linda Handlin |
  | Author | Maria Petersson |
  | Author | Kerstin Uvnäs-Moberg |
  | Date | 2024 |
  | URL | https://www.scopus.com/inward/record.uri?eid=2-s2.0-85178181292&doi=10.1080%2f08927936.2023.2280374&partnerID=40&md5=8902422c7b89bd27b70d880b8ff08a78 |
  | Extra | Type: Article |
  | Volume | 37 |
  | Pages | 323 – 342 |
  | Publication | Anthrozoos |
  | DOI | 10.1080/08927936.2023.2280374 |
  | Issue | 2 |
  | Date Added | 19/06/2025, 15:40:12 |
  | Modified | 19/06/2025, 15:40:12 |

  ### Notes:

  - Cited by: 1
- ## Human-animal interactions in dairy buffalo farms

  |  |  |
  | --- | --- |
  | Item Type | Journal Article |
  | Author | Fabio Napolitano |
  | Author | Francesco Serrapica |
  | Author | Ada Braghieri |
  | Author | Felicia Masucci |
  | Author | Emilio Sabia |
  | Author | Giuseppe De Rosa |
  | Date | 2019 |
  | URL | https://www.scopus.com/inward/record.uri?eid=2-s2.0-85068514155&doi=10.3390%2fani9050246&partnerID=40&md5=4cdd4f929ee6abc1e57480b3161d8ebd |
  | Extra | Type: Article |
  | Volume | 9 |
  | Publication | Animals |
  | DOI | 10.3390/ani9050246 |
  | Issue | 5 |
  | Date Added | 20/06/2025, 09:37:47 |
  | Modified | 20/06/2025, 09:37:47 |

  ### Notes:

  - Cited by: 31; All Open Access, Gold Open Access, Green Open Access
- ## Attachment-like behavioral expressions to humans in puppies are related to oxytocin and cortisol: A comparative study of Akitas and Labrador Retrievers

  |  |  |
  | --- | --- |
  | Item Type | Journal Article |
  | Author | Miho Nagasawa |
  | Author | Sakiko Tomori |
  | Author | Kazutaka Mogi |
  | Author | Takefumi Kikusui |
  | Date | 2024 |
  | URL | https://www.scopus.com/inward/record.uri?eid=2-s2.0-85191350502&doi=10.1016%2fj.peptides.2024.171224&partnerID=40&md5=e34dcb3d1077438168e95c83926106c5 |
  | Extra | Type: Article |
  | Volume | 177 |
  | Publication | Peptides |
  | DOI | 10.1016/j.peptides.2024.171224 |
  | Date Added | 20/06/2025, 09:37:47 |
  | Modified | 20/06/2025, 09:37:47 |

  ### Notes:

  - Cited by: 3; All Open Access, Hybrid Gold Open Access
- ## Attachment-like behavioral expressions to humans in puppies are related to oxytocin and cortisol: A comparative study of Akitas and Labrador Retrievers

  |  |  |
  | --- | --- |
  | Item Type | Journal Article |
  | Author | Miho Nagasawa |
  | Author | Sakiko Tomori |
  | Author | Kazutaka Mogi |
  | Author | Takefumi Kikusui |
  | Date | 2024 |
  | URL | https://www.scopus.com/inward/record.uri?eid=2-s2.0-85191350502&doi=10.1016%2fj.peptides.2024.171224&partnerID=40&md5=e34dcb3d1077438168e95c83926106c5 |
  | Extra | Type: Article |
  | Volume | 177 |
  | Publication | Peptides |
  | DOI | 10.1016/j.peptides.2024.171224 |
  | Date Added | 11/07/2025, 14:08:23 |
  | Modified | 11/07/2025, 14:08:23 |

  ### Notes:

  - Cited by: 3; All Open Access, Bronze Open Access
- ## Environmental enrichment and social rank affects the fear and stress response to regular handling of dairy goats

  |  |  |
  | --- | --- |
  | Item Type | Journal Article |
  | Author | Genaro C. Miranda-de la Lama |
  | Author | Rocío Pinal |
  | Author | Katrin Fuchs |
  | Author | Hugo H. Montaldo |
  | Author | Andrés Ducoing |
  | Author | Francisco Galindo |
  | Date | 2013 |
  | URL | https://www.scopus.com/inward/record.uri?eid=2-s2.0-84883453082&doi=10.1016%2fj.jveb.2013.03.001&partnerID=40&md5=59282105813c4dd261827ecb0a6a8693 |
  | Extra | Type: Article |
  | Volume | 8 |
  | Pages | 342 – 348 |
  | Publication | Journal of Veterinary Behavior: Clinical Applications and Research |
  | DOI | 10.1016/j.jveb.2013.03.001 |
  | Issue | 5 |
  | Date Added | 11/07/2025, 14:08:22 |
  | Modified | 11/07/2025, 14:08:22 |

  ### Notes:

  - Cited by: 24
- ## A shoulder to cry on: Heart rate variability and empathetic behavioral responses to crying and laughing in dogs.

  |  |  |
  | --- | --- |
  | Item Type | Journal Article |
  | Author | Julia E. Meyers-Manor |
  | Author | Marijo L. Botten |
  | Date | 2020 |
  | URL | https://www.scopus.com/inward/record.uri?eid=2-s2.0-85094116133&doi=10.1037%2fcep0000225&partnerID=40&md5=22def67bdcf8ae70dd59f9da90a4fd27 |
  | Extra | Type: Article |
  | Volume | 74 |
  | Pages | 235 – 243 |
  | Publication | Canadian Journal of Experimental Psychology |
  | DOI | 10.1037/cep0000225 |
  | Issue | 3 |
  | Date Added | 19/06/2025, 15:40:14 |
  | Modified | 19/06/2025, 15:40:14 |

  ### Notes:

  - Cited by: 6
- ## Can you spare 15 min? The measurable positive impact of a 15-min petting session on shelter dog well-being

  |  |  |
  | --- | --- |
  | Item Type | Journal Article |
  | Author | Ragen T.S. McGowan |
  | Author | Cynthia Bolte |
  | Author | Hallie R. Barnett |
  | Author | Gerardo Perez-Camargo |
  | Author | François Martin |
  | Date | 2018 |
  | URL | https://www.scopus.com/inward/record.uri?eid=2-s2.0-85044154068&doi=10.1016%2fj.applanim.2018.02.011&partnerID=40&md5=5a256e8284ed0c705aa0b0c47e2b8876 |
  | Extra | Type: Article |
  | Volume | 203 |
  | Pages | 42 – 54 |
  | Publication | Applied Animal Behaviour Science |
  | DOI | 10.1016/j.applanim.2018.02.011 |
  | Date Added | 19/06/2025, 15:40:13 |
  | Modified | 19/06/2025, 15:40:13 |

  ### Notes:

  - Cited by: 35; All Open Access, Hybrid Gold Open Access
- ## Can you spare 15 min? The measurable positive impact of a 15-min petting session on shelter dog well-being

  |  |  |
  | --- | --- |
  | Item Type | Journal Article |
  | Author | Ragen T.S. McGowan |
  | Author | Cynthia Bolte |
  | Author | Hallie R. Barnett |
  | Author | Gerardo Perez-Camargo |
  | Author | François Martin |
  | Date | 2018 |
  | URL | https://www.scopus.com/inward/record.uri?eid=2-s2.0-85044154068&doi=10.1016%2fj.applanim.2018.02.011&partnerID=40&md5=5a256e8284ed0c705aa0b0c47e2b8876 |
  | Extra | Type: Article |
  | Volume | 203 |
  | Pages | 42 – 54 |
  | Publication | Applied Animal Behaviour Science |
  | DOI | 10.1016/j.applanim.2018.02.011 |
  | Date Added | 11/07/2025, 14:08:23 |
  | Modified | 11/07/2025, 14:08:23 |

  ### Notes:

  - Cited by: 35; All Open Access, Hybrid Gold Open Access
- ## INTERACTION OF EMOTIONAL CONNECTION BETWEEN HORSE AND HUMAN

  |  |  |
  | --- | --- |
  | Item Type | Journal Article |
  | Author | Otilija Mataitė |
  | Author | Vilma Vilienė |
  | Date | 2024 |
  | URL | https://www.scopus.com/inward/record.uri?eid=2-s2.0-85212925920&partnerID=40&md5=bfad83920bb67cd2cfb6b1320373a4bd |
  | Extra | Type: Article |
  | Volume | 82(1) |
  | Pages | 155 – 155 |
  | Publication | Veterinarija ir Zootechnika |
  | Date Added | 19/06/2025, 15:40:13 |
  | Modified | 19/06/2025, 15:40:13 |

  ### Notes:

  - Cited by: 0
- ## Effects of contact with a dog on prefrontal brain activation in patients in a minimally conscious state: A controlled crossover trial

  |  |  |
  | --- | --- |
  | Item Type | Journal Article |
  | Author | Rahel Marti |
  | Author | Milena Petignat |
  | Author | Valentine L. Marcar |
  | Author | Jan Hattendorf |
  | Author | Martin Wolf |
  | Author | Margret Hund-Georgiadis |
  | Author | Karin Hediger |
  | Date | 2025 |
  | URL | https://www.scopus.com/inward/record.uri?eid=2-s2.0-105005653323&doi=10.1016%2fj.neuroscience.2025.05.014&partnerID=40&md5=781ab7de1aa74b2384e74dea4cd2699f |
  | Extra | Type: Article |
  | Volume | 577 |
  | Pages | 175 – 189 |
  | Publication | Neuroscience |
  | DOI | 10.1016/j.neuroscience.2025.05.014 |
  | Date Added | 19/06/2025, 15:40:13 |
  | Modified | 19/06/2025, 15:40:13 |

  ### Notes:

  - Cited by: 0
- ## Effects of contact with a dog on prefrontal brain activation in patients in a minimally conscious state: A controlled crossover trial

  |  |  |
  | --- | --- |
  | Item Type | Journal Article |
  | Author | R. Marti |
  | Author | M. Petignat |
  | Author | V.L. Marcar |
  | Author | J. Hattendorf |
  | Author | M. Wolf |
  | Author | M. Hund-Georgiadis |
  | Author | K. Hediger |
  | Date | 2025 |
  | Archive | Scopus |
  | URL | https://www.scopus.com/inward/record.uri?eid=2-s2.0-105005653323&doi=10.1016%2fj.neuroscience.2025.05.014&partnerID=40&md5=781ab7de1aa74b2384e74dea4cd2699f |
  | Volume | 577 |
  | Pages | 175-189 |
  | Publication | Neuroscience |
  | DOI | 10.1016/j.neuroscience.2025.05.014 |
  | Date Added | 11/07/2025, 11:22:51 |
  | Modified | 11/07/2025, 11:22:51 |

  ### Notes:

  - Export Date: 11 July 2025; Cited By: 0
- ## Effects of contact with a dog on prefrontal brain activation in patients in a minimally conscious state: A controlled crossover trial

  |  |  |
  | --- | --- |
  | Item Type | Journal Article |
  | Author | R. Marti |
  | Author | M. Petignat |
  | Author | V.L. Marcar |
  | Author | J. Hattendorf |
  | Author | M. Wolf |
  | Author | M. Hund-Georgiadis |
  | Author | K. Hediger |
  | Date | 2025 |
  | Archive | Scopus |
  | URL | https://www.scopus.com/inward/record.uri?eid=2-s2.0-105005653323&doi=10.1016%2fj.neuroscience.2025.05.014&partnerID=40&md5=781ab7de1aa74b2384e74dea4cd2699f |
  | Volume | 577 |
  | Pages | 175-189 |
  | Publication | Neuroscience |
  | DOI | 10.1016/j.neuroscience.2025.05.014 |
  | Date Added | 11/07/2025, 11:24:11 |
  | Modified | 11/07/2025, 11:24:11 |

  ### Notes:

  - Export Date: 11 July 2025; Cited By: 0
- ## A note on the effect of gestation housing environment on approach test measures in gilts

  |  |  |
  | --- | --- |
  | Item Type | Journal Article |
  | Author | Jeremy N Marchant |
  | Author | R. Harry Bradshaw |
  | Author | Ruth M. Marchant-Forde |
  | Author | Donald M. Broom |
  | Date | 2003 |
  | URL | https://www.scopus.com/inward/record.uri?eid=2-s2.0-0037362335&doi=10.1016%2fS0168-1591%2802%2900229-0&partnerID=40&md5=4cc68116f9cb5b103edecb8f6984eced |
  | Extra | Type: Article |
  | Volume | 80 |
  | Pages | 287 – 296 |
  | Publication | Applied Animal Behaviour Science |
  | DOI | 10.1016/S0168-1591(02)00229-0 |
  | Issue | 4 |
  | Date Added | 19/06/2025, 15:40:14 |
  | Modified | 19/06/2025, 15:40:14 |

  ### Notes:

  - Cited by: 21
- ## A note on the effect of gestation housing environment on approach test measures in gilts

  |  |  |
  | --- | --- |
  | Item Type | Journal Article |
  | Author | Jeremy N Marchant |
  | Author | R. Harry Bradshaw |
  | Author | Ruth M. Marchant-Forde |
  | Author | Donald M. Broom |
  | Date | 2003 |
  | URL | https://www.scopus.com/inward/record.uri?eid=2-s2.0-0037362335&doi=10.1016%2fS0168-1591%2802%2900229-0&partnerID=40&md5=4cc68116f9cb5b103edecb8f6984eced |
  | Extra | Number: 4 Type: Article |
  | Volume | 80 |
  | Pages | 287 – 296 |
  | Publication | Applied Animal Behaviour Science |
  | DOI | 10.1016/S0168-1591(02)00229-0 |
  | Issue | 4 |
  | Date Added | 28/07/2025, 12:48:52 |
  | Modified | 28/07/2025, 12:48:52 |

  ### Notes:

  - Cited by: 21
- ## Piglet- and stockperson-directed sow aggression after farrowing and the relationship with a pre-farrowing, human approach test

  |  |  |
  | --- | --- |
  | Item Type | Journal Article |
  | Author | Jeremy N Marchant |
  | Date | 2002 |
  | URL | https://www.scopus.com/inward/record.uri?eid=2-s2.0-0037011841&doi=10.1016%2fS0168-1591%2801%2900170-8&partnerID=40&md5=5c13e131ffa1940bc78cf005ee287f13 |
  | Extra | Type: Article |
  | Volume | 75 |
  | Pages | 115 – 132 |
  | Publication | Applied Animal Behaviour Science |
  | DOI | 10.1016/S0168-1591(01)00170-8 |
  | Issue | 2 |
  | Date Added | 19/06/2025, 15:40:12 |
  | Modified | 19/06/2025, 15:40:12 |

  ### Notes:

  - Cited by: 67
- ## Piglet- and stockperson-directed sow aggression after farrowing and the relationship with a pre-farrowing, human approach test

  |  |  |
  | --- | --- |
  | Item Type | Journal Article |
  | Author | Jeremy N Marchant |
  | Date | 2002 |
  | URL | https://www.scopus.com/inward/record.uri?eid=2-s2.0-0037011841&doi=10.1016%2fS0168-1591%2801%2900170-8&partnerID=40&md5=5c13e131ffa1940bc78cf005ee287f13 |
  | Extra | Number: 2 Type: Article |
  | Volume | 75 |
  | Pages | 115 – 132 |
  | Publication | Applied Animal Behaviour Science |
  | DOI | 10.1016/S0168-1591(01)00170-8 |
  | Issue | 2 |
  | Date Added | 28/07/2025, 12:48:52 |
  | Modified | 28/07/2025, 12:48:52 |

  ### Notes:

  - Cited by: 67
- ## Comparative analysis of stress responses in dogs and cats during the covid-19 pandemic: a focus on cortisol, total leukocytes, eosinophils, and behavioral changes; [Análise comparativa das respostas ao estresse em cães e gatos durante a pandemia de covid-19: foco no cortisol, nos leucócitos totais, nos eosinófilos e nas alterações comportamentais]

  |  |  |
  | --- | --- |
  | Item Type | Journal Article |
  | Author | R.N. Malancus |
  | Author | V.N. Arsenoaia |
  | Author | M. Ghita |
  | Date | 2024 |
  | URL | https://www.scopus.com/inward/record.uri?eid=2-s2.0-85198094069&doi=10.1590%2f1678-4162-13153&partnerID=40&md5=b4b3f8e2591bb0f3f24e6588fd910ec9 |
  | Extra | Type: Article |
  | Volume | 76 |
  | Publication | Arquivo Brasileiro de Medicina Veterinaria e Zootecnia |
  | DOI | 10.1590/1678-4162-13153 |
  | Issue | 3 |
  | Date Added | 11/07/2025, 14:08:23 |
  | Modified | 11/07/2025, 14:08:23 |

  ### Notes:

  - Cited by: 0; All Open Access, Gold Open Access
- ## Validation of salivary oxytocin and vasopressin as biomarkers in domestic dogs

  |  |  |
  | --- | --- |
  | Item Type | Journal Article |
  | Author | Evan L. MacLean |
  | Author | Laurence R. Gesquiere |
  | Author | Nancy Gee |
  | Author | Kerinne Levy |
  | Author | W. Lance Martin |
  | Author | C. Sue Carter |
  | Date | 2018 |
  | URL | https://www.scopus.com/inward/record.uri?eid=2-s2.0-85029673405&doi=10.1016%2fj.jneumeth.2017.08.033&partnerID=40&md5=94e8c0ee898d7f5d2f1aa6f1fe899f57 |
  | Extra | Type: Article |
  | Volume | 293 |
  | Pages | 67 – 76 |
  | Publication | Journal of Neuroscience Methods |
  | DOI | 10.1016/j.jneumeth.2017.08.033 |
  | Date Added | 20/06/2025, 09:37:48 |
  | Modified | 20/06/2025, 09:37:48 |

  ### Notes:

  - Cited by: 86; All Open Access, Green Open Access
- ## Effects of affiliative human-animal interaction on dog salivary and plasma oxytocin and vasopressin

  |  |  |
  | --- | --- |
  | Item Type | Journal Article |
  | Author | Evan L. MacLean |
  | Author | Laurence R. Gesquiere |
  | Author | Nancy R. Gee |
  | Author | Kerinne Levy |
  | Author | W. Lance Martin |
  | Author | C. Sue Carter |
  | Date | 2017 |
  | URL | https://www.scopus.com/inward/record.uri?eid=2-s2.0-85029612806&doi=10.3389%2ffpsyg.2017.01606&partnerID=40&md5=909650d266523e6955abb0bd70bf8590 |
  | Extra | Type: Article |
  | Volume | 8 |
  | Publication | Frontiers in Psychology |
  | DOI | 10.3389/fpsyg.2017.01606 |
  | Issue | SEP |
  | Date Added | 20/06/2025, 09:37:48 |
  | Modified | 20/06/2025, 09:37:48 |

  ### Notes:

  - Cited by: 74; All Open Access, Gold Open Access, Green Open Access
- ## Human-animal interaction, stress, and embryo production in Bos indicus embryo donors under tropical conditions

  |  |  |
  | --- | --- |
  | Item Type | Journal Article |
  | Author | Gustavo Guerino Macedo |
  | Author | Carmem Estefânia Serra Neto Zúccari |
  | Author | Urbano Gomes Pinto de Abreu |
  | Author | João Alberto Negrão |
  | Author | Eliane Vianna da Costa e Silva |
  | Date | 2011 |
  | URL | https://www.scopus.com/inward/record.uri?eid=2-s2.0-79958851427&doi=10.1007%2fs11250-011-9820-6&partnerID=40&md5=ca64951044c08f1d07e3457c86293b95 |
  | Extra | Type: Article |
  | Volume | 43 |
  | Pages | 1175 – 1182 |
  | Publication | Tropical Animal Health and Production |
  | DOI | 10.1007/s11250-011-9820-6 |
  | Issue | 6 |
  | Date Added | 11/07/2025, 14:08:22 |
  | Modified | 11/07/2025, 14:08:22 |

  ### Notes:

  - Cited by: 30
- ## Salivary oxytocin in pigs, cattle, and goats during positive human-animal interactions

  |  |  |
  | --- | --- |
  | Item Type | Journal Article |
  | Author | Stephanie Lürzel |
  | Author | Laura Bückendorf |
  | Author | Susanne Waiblinger |
  | Author | Jean-Loup Rault |
  | Date | 2020 |
  | URL | https://www.scopus.com/inward/record.uri?eid=2-s2.0-85081034867&doi=10.1016%2fj.psyneuen.2020.104636&partnerID=40&md5=db45f2a018e270075d73f781f0f70f0b |
  | Extra | Type: Article |
  | Volume | 115 |
  | Publication | Psychoneuroendocrinology |
  | DOI | 10.1016/j.psyneuen.2020.104636 |
  | Date Added | 20/06/2025, 09:37:48 |
  | Modified | 20/06/2025, 09:37:48 |

  ### Notes:

  - Cited by: 42
- ## Does training style affect the human-horse relationship? Asking the horse in a separation–reunion experiment with the owner and a stranger

  |  |  |
  | --- | --- |
  | Item Type | Journal Article |
  | Author | Paulina Lundberg |
  | Author | Elke Hartmann |
  | Author | Lina S.V. Roth |
  | Date | 2020 |
  | URL | https://www.scopus.com/inward/record.uri?eid=2-s2.0-85094201421&doi=10.1016%2fj.applanim.2020.105144&partnerID=40&md5=512e2eb0170aafb1124f1dad87566cc9 |
  | Extra | Type: Article |
  | Volume | 233 |
  | Publication | Applied Animal Behaviour Science |
  | DOI | 10.1016/j.applanim.2020.105144 |
  | Date Added | 19/06/2025, 15:40:14 |
  | Modified | 19/06/2025, 15:40:14 |

  ### Notes:

  - Cited by: 20; All Open Access, Green Open Access, Hybrid Gold Open Access
- ## Early human contact and housing for pigs – part 3: ability to cope with the environment

  |  |  |
  | --- | --- |
  | Item Type | Journal Article |
  | Author | M.E. Lucas |
  | Author | L.M. Hemsworth |
  | Author | K.L. Butler |
  | Author | R.S. Morrison |
  | Author | A.J. Tilbrook |
  | Author | J.N. Marchant |
  | Author | J.-L. Rault |
  | Author | R.Y. Galea |
  | Author | P.H. Hemsworth |
  | Date | 2024 |
  | URL | https://www.scopus.com/inward/record.uri?eid=2-s2.0-85193578464&doi=10.1016%2fj.animal.2024.101166&partnerID=40&md5=f6fb6fa038921f039ffed80b17ad77cf |
  | Extra | Type: Article |
  | Volume | 18 |
  | Publication | Animal |
  | DOI | 10.1016/j.animal.2024.101166 |
  | Issue | 6 |
  | Date Added | 11/07/2025, 14:08:22 |
  | Modified | 11/07/2025, 14:08:22 |

  ### Notes:

  - Cited by: 2; All Open Access, Gold Open Access
- ## Early human contact and housing for pigs – part 2: resilience to routine husbandry practices

  |  |  |
  | --- | --- |
  | Item Type | Journal Article |
  | Author | M.E. Lucas |
  | Author | L.M. Hemsworth |
  | Author | K.L. Butler |
  | Author | R.S. Morrison |
  | Author | A.J. Tilbrook |
  | Author | J.N. Marchant |
  | Author | J.-L. Rault |
  | Author | R.Y. Galea |
  | Author | P.H. Hemsworth |
  | Date | 2024 |
  | URL | https://www.scopus.com/inward/record.uri?eid=2-s2.0-85193633524&doi=10.1016%2fj.animal.2024.101165&partnerID=40&md5=785e78cf9371848144a00510eb38b459 |
  | Extra | Type: Article |
  | Volume | 18 |
  | Publication | Animal |
  | DOI | 10.1016/j.animal.2024.101165 |
  | Issue | 6 |
  | Date Added | 11/07/2025, 14:08:22 |
  | Modified | 11/07/2025, 14:08:22 |

  ### Notes:

  - Cited by: 3; All Open Access, Gold Open Access
- ## Early human contact and housing for pigs − part 1: responses to humans, novelty and isolation

  |  |  |
  | --- | --- |
  | Item Type | Journal Article |
  | Author | M.E. Lucas |
  | Author | L.M. Hemsworth |
  | Author | K.L. Butler |
  | Author | R.S. Morrison |
  | Author | A.J. Tilbrook |
  | Author | J.N. Marchant |
  | Author | J.-L. Rault |
  | Author | R.Y. Galea |
  | Author | P.H. Hemsworth |
  | Date | 2024 |
  | URL | https://www.scopus.com/inward/record.uri?eid=2-s2.0-85193445159&doi=10.1016%2fj.animal.2024.101164&partnerID=40&md5=d4e8238acda58f3cc0cbfd855644a6a6 |
  | Extra | Type: Article |
  | Volume | 18 |
  | Publication | Animal |
  | DOI | 10.1016/j.animal.2024.101164 |
  | Issue | 6 |
  | Date Added | 11/07/2025, 14:08:23 |
  | Modified | 11/07/2025, 14:08:23 |

  ### Notes:

  - Cited by: 5; All Open Access, Gold Open Access
- ## Changes in salivary oxytocin after stroking in dogs: Validation of two assays for its assessment

  |  |  |
  | --- | --- |
  | Item Type | Journal Article |
  | Author | Marina López-Arjona |
  | Author | Sandra V. Mateo |
  | Author | José J. Cerón |
  | Author | Silvia Martínez-Subiela |
  | Date | 2021 |
  | URL | https://www.scopus.com/inward/record.uri?eid=2-s2.0-85104445364&doi=10.1016%2fj.rvsc.2021.04.007&partnerID=40&md5=2439370fa19dd68fe0b3f961992b9a1a |
  | Extra | Type: Article |
  | Volume | 136 |
  | Pages | 527 – 534 |
  | Publication | Research in Veterinary Science |
  | DOI | 10.1016/j.rvsc.2021.04.007 |
  | Date Added | 20/06/2025, 09:37:48 |
  | Modified | 20/06/2025, 09:37:48 |

  ### Notes:

  - Cited by: 10
- ## Human-animal interactions and safety during dairy cattle handling-Comparing moving cows to milking and hoof trimming

  |  |  |
  | --- | --- |
  | Item Type | Journal Article |
  | Author | C. Lindahl |
  | Author | S. Pinzke |
  | Author | A. Herlin |
  | Author | L.J. Keeling |
  | Date | 2016 |
  | URL | https://www.scopus.com/inward/record.uri?eid=2-s2.0-84957839565&doi=10.3168%2fjds.2014-9210&partnerID=40&md5=f6ba746c6bde7c7340343c66603cc3bf |
  | Extra | Type: Article |
  | Volume | 99 |
  | Pages | 2131 – 2141 |
  | Publication | Journal of Dairy Science |
  | DOI | 10.3168/jds.2014-9210 |
  | Issue | 3 |
  | Date Added | 19/06/2025, 15:40:13 |
  | Modified | 19/06/2025, 15:40:13 |

  ### Notes:

  - Cited by: 55; All Open Access, Bronze Open Access, Green Open Access
- ## Evaluating changes in salivary oxytocin and cortisol following positive reinforcement training in two adult male western lowland gorillas (Gorilla gorilla gorilla)

  |  |  |
  | --- | --- |
  | Item Type | Journal Article |
  | Author | Austin Leeds |
  | Author | Julie Good |
  | Author | Mandi W. Schook |
  | Author | Patricia M. Dennis |
  | Author | Tara S. Stoinski |
  | Author | Mark A. Willis |
  | Author | Kristen E. Lukas |
  | Date | 2020 |
  | URL | https://www.scopus.com/inward/record.uri?eid=2-s2.0-85075214313&doi=10.1002%2fzoo.21524&partnerID=40&md5=d0c1c04b9d74a9065ded2f196ca28043 |
  | Extra | Type: Article |
  | Volume | 39 |
  | Pages | 51 – 55 |
  | Publication | Zoo Biology |
  | DOI | 10.1002/zoo.21524 |
  | Issue | 1 |
  | Date Added | 20/06/2025, 09:37:48 |
  | Modified | 20/06/2025, 09:37:48 |

  ### Notes:

  - Cited by: 6
- ## Evaluating changes in salivary oxytocin and cortisol following positive reinforcement training in two adult male western lowland gorillas (Gorilla gorilla gorilla)

  |  |  |
  | --- | --- |
  | Item Type | Journal Article |
  | Author | Austin Leeds |
  | Author | Julie Good |
  | Author | Mandi W. Schook |
  | Author | Patricia M. Dennis |
  | Author | Tara S. Stoinski |
  | Author | Mark A. Willis |
  | Author | Kristen E. Lukas |
  | Date | 2020 |
  | URL | https://www.scopus.com/inward/record.uri?eid=2-s2.0-85075214313&doi=10.1002%2fzoo.21524&partnerID=40&md5=d0c1c04b9d74a9065ded2f196ca28043 |
  | Extra | Type: Article |
  | Volume | 39 |
  | Pages | 51 – 55 |
  | Publication | Zoo Biology |
  | DOI | 10.1002/zoo.21524 |
  | Issue | 1 |
  | Date Added | 11/07/2025, 14:08:23 |
  | Modified | 11/07/2025, 14:08:23 |

  ### Notes:

  - Cited by: 6
- ## Talking to Cows: Reactions to Different Auditory Stimuli During Gentle Human-Animal Interactions

  |  |  |
  | --- | --- |
  | Item Type | Journal Article |
  | Author | Annika Lange |
  | Author | Lisa Bauer |
  | Author | Andreas Futschik |
  | Author | Susanne Waiblinger |
  | Author | Stephanie Lürzel |
  | Date | 2020 |
  | URL | https://www.scopus.com/inward/record.uri?eid=2-s2.0-85094681274&doi=10.3389%2ffpsyg.2020.579346&partnerID=40&md5=3ffd628275ae0436220bf79f573e406f |
  | Extra | Type: Article |
  | Volume | 11 |
  | Publication | Frontiers in Psychology |
  | DOI | 10.3389/fpsyg.2020.579346 |
  | Date Added | 19/06/2025, 15:40:12 |
  | Modified | 19/06/2025, 15:40:12 |

  ### Notes:

  - Cited by: 14; All Open Access, Gold Open Access, Green Open Access
- ## Effects of restraint on heifers during gentle human-animal interactions

  |  |  |
  | --- | --- |
  | Item Type | Journal Article |
  | Author | Annika Lange |
  | Author | Susanne Waiblinger |
  | Author | Regien van Hasselt |
  | Author | Roger Mundry |
  | Author | Andreas Futschik |
  | Author | Stephanie Lürzel |
  | Date | 2021 |
  | URL | https://www.scopus.com/inward/record.uri?eid=2-s2.0-85115414374&doi=10.1016%2fj.applanim.2021.105445&partnerID=40&md5=bef25323bec39bc7024f5eae5f019467 |
  | Extra | Type: Article |
  | Volume | 243 |
  | Publication | Applied Animal Behaviour Science |
  | DOI | 10.1016/j.applanim.2021.105445 |
  | Date Added | 19/06/2025, 15:40:12 |
  | Modified | 19/06/2025, 15:40:12 |

  ### Notes:

  - Cited by: 7; All Open Access, Hybrid Gold Open Access
- ## Effects of different stroking styles on behaviour and cardiac parameters in heifers

  |  |  |
  | --- | --- |
  | Item Type | Journal Article |
  | Author | Annika Lange |
  | Author | Sandra Franzmayr |
  | Author | Vera Wisenöcker |
  | Author | Andreas Futschik |
  | Author | Susanne Waiblinger |
  | Author | Stephanie Lürzel |
  | Date | 2020 |
  | URL | https://www.scopus.com/inward/record.uri?eid=2-s2.0-85081170863&doi=10.3390%2fani10030426&partnerID=40&md5=e81b2b323f385bc74b958e757047fe79 |
  | Extra | Type: Article |
  | Volume | 10 |
  | Publication | Animals |
  | DOI | 10.3390/ani10030426 |
  | Issue | 3 |
  | Date Added | 19/06/2025, 15:40:14 |
  | Modified | 19/06/2025, 15:40:14 |

  ### Notes:

  - Cited by: 23; All Open Access, Gold Open Access, Green Open Access
- ## Behavioral and cardiac responses by dogs to physical human-dog contact

  |  |  |
  | --- | --- |
  | Item Type | Journal Article |
  | Author | Franziska Kuhne |
  | Author | Johanna C. Hößler |
  | Author | Rainer Struwe |
  | Date | 2014 |
  | URL | https://www.scopus.com/inward/record.uri?eid=2-s2.0-84899634573&doi=10.1016%2fj.jveb.2014.02.006&partnerID=40&md5=4aa8fdac2e9a696cfb52c2402b93cacd |
  | Extra | Type: Article |
  | Volume | 9 |
  | Pages | 93 – 97 |
  | Publication | Journal of Veterinary Behavior: Clinical Applications and Research |
  | DOI | 10.1016/j.jveb.2014.02.006 |
  | Issue | 3 |
  | Date Added | 19/06/2025, 15:40:14 |
  | Modified | 19/06/2025, 15:40:14 |

  ### Notes:

  - Cited by: 54
- ## Veterans and Shelter Dogs: Examining the Impact of a Dog-Walking Intervention on Physiological and Post-Traumatic Stress Symptoms

  |  |  |
  | --- | --- |
  | Item Type | Journal Article |
  | Author | Cheryl A. Krause-Parello |
  | Author | Erika Friedmann |
  | Author | Kelly Blanchard |
  | Author | Megan Payton |
  | Author | Nancy R. Gee |
  | Date | 2020 |
  | URL | https://www.scopus.com/inward/record.uri?eid=2-s2.0-85081891890&doi=10.1080%2f08927936.2020.1719763&partnerID=40&md5=0da00f53aa0bd1b2b6b949f0cb60934b |
  | Extra | Type: Article |
  | Volume | 33 |
  | Pages | 225 – 241 |
  | Publication | Anthrozoos |
  | DOI | 10.1080/08927936.2020.1719763 |
  | Issue | 2 |
  | Date Added | 19/06/2025, 15:40:12 |
  | Modified | 04/01/2026, 11:26:09 |

  ### Tags:

  - veteran
  - heart rate variability
  - dog
  - animal experiment
  - article
  - female
  - male
  - nonhuman
  - physiological stress
  - human
  - hydrocortisone
  - adult
  - controlled study
  - posttraumatic stress disorder
  - crossover procedure
  - animal model
  - mental stress
  - outcome assessment
  - amylase
  - endogenous compound
  - gene expression
  - protein expression
  - sample size
  - walking

  ### Notes:

  - Cited by: 10
  - Cited by: 10

  ### Attachments

  - Full Text (HTML)
  - Full Text (HTML)
  - Full Text (HTML)
- ## Behavioral and emotional co-modulation during dog–owner interaction measured by heart rate variability and activity

  |  |  |
  | --- | --- |
  | Item Type | Journal Article |
  | Author | Aija Koskela |
  | Author | Heini Törnqvist |
  | Author | Sanni Somppi |
  | Author | Katriina Tiira |
  | Author | Virpi-Liisa Kykyri |
  | Author | Laura Hänninen |
  | Author | Jan Kujala |
  | Author | Miho Nagasawa |
  | Author | Takefumi Kikusui |
  | Author | Miiamaaria V. Kujala |
  | Date | 2024 |
  | URL | https://www.scopus.com/inward/record.uri?eid=2-s2.0-85207349814&doi=10.1038%2fs41598-024-76831-x&partnerID=40&md5=a71885bb730a94e974976bdcb5687808 |
  | Extra | Type: Article |
  | Volume | 14 |
  | Publication | Scientific Reports |
  | DOI | 10.1038/s41598-024-76831-x |
  | Issue | 1 |
  | Date Added | 19/06/2025, 15:40:13 |
  | Modified | 19/06/2025, 15:40:13 |

  ### Notes:

  - Cited by: 0; All Open Access, Gold Open Access
- ## Equine behaviour and heart rate in temperament tests with or without rider or handler

  |  |  |
  | --- | --- |
  | Item Type | Journal Article |
  | Author | U. König von Borstel |
  | Author | S. Euent |
  | Author | P. Graf |
  | Author | S. König |
  | Author | M. Gauly |
  | Date | 2011 |
  | URL | https://www.scopus.com/inward/record.uri?eid=2-s2.0-79959857368&doi=10.1016%2fj.physbeh.2011.05.010&partnerID=40&md5=290e71bbb3c1fc6f6e29770f05cc09b2 |
  | Extra | Type: Article |
  | Volume | 104 |
  | Pages | 454 – 463 |
  | Publication | Physiology and Behavior |
  | DOI | 10.1016/j.physbeh.2011.05.010 |
  | Issue | 3 |
  | Date Added | 19/06/2025, 15:40:13 |
  | Modified | 19/06/2025, 15:40:13 |

  ### Notes:

  - Cited by: 52
- ## Social bonding between humans, animals, and robots: Dogs outperform AIBOs, their robotic replicas, as social companions

  |  |  |
  | --- | --- |
  | Item Type | Journal Article |
  | Author | Stella Klumpe |
  | Author | Kelsey C. Mitchell |
  | Author | Emma Cox |
  | Author | Jeffrey S. Katz |
  | Author | Lucia Lazarowski |
  | Author | Gopikrishna Deshpande |
  | Author | Jonathan Gratch |
  | Author | Ewart J. de Visser |
  | Author | Hasan Ayaz |
  | Author | Xingnan Li |
  | Author | Adrian A. Franke |
  | Author | Frank Krueger |
  | Date | 2025 |
  | URL | https://www.scopus.com/inward/record.uri?eid=2-s2.0-105007059245&doi=10.1371%2fjournal.pone.0324312&partnerID=40&md5=1d6e940322902018d3d494bf5d455486 |
  | Extra | Type: Article |
  | Volume | 20 |
  | Publication | PLoS ONE |
  | DOI | 10.1371/journal.pone.0324312 |
  | Issue | 6 June |
  | Date Added | 20/06/2025, 09:37:48 |
  | Modified | 20/06/2025, 09:37:48 |

  ### Notes:

  - Cited by: 0
- ## Soybean oil supplement induces increased approaching behavior to humans and alters serotonin concentrations in horses

  |  |  |
  | --- | --- |
  | Item Type | Journal Article |
  | Author | Seongmin Kim |
  | Author | Yeonju Choi |
  | Author | Junyoung Kim |
  | Author | Carissa L. Wickens |
  | Author | Minjung Yoon |
  | Date | 2025 |
  | URL | https://www.scopus.com/inward/record.uri?eid=2-s2.0-85215797341&doi=10.1016%2fj.jevs.2025.105361&partnerID=40&md5=b6c887b217427818ae0d861c77205be6 |
  | Extra | Type: Article |
  | Volume | 146 |
  | Publication | Journal of Equine Veterinary Science |
  | DOI | 10.1016/j.jevs.2025.105361 |
  | Date Added | 11/07/2025, 14:08:22 |
  | Modified | 11/07/2025, 14:08:22 |

  ### Notes:

  - Cited by: 0
- ## The effect of serotonin and oxytocin on equine docility and friendliness to humans

  |  |  |
  | --- | --- |
  | Item Type | Journal Article |
  | Author | Junyoung Kim |
  | Author | Minjung Yoon |
  | Date | 2022 |
  | URL | https://www.scopus.com/inward/record.uri?eid=2-s2.0-85125515818&doi=10.1016%2fj.jveb.2022.01.004&partnerID=40&md5=789847e4827514796f2cf6697f7901d5 |
  | Extra | Type: Article |
  | Volume | 50 |
  | Pages | 18 – 22 |
  | Publication | Journal of Veterinary Behavior |
  | DOI | 10.1016/j.jveb.2022.01.004 |
  | Date Added | 20/06/2025, 09:37:48 |
  | Modified | 20/06/2025, 09:37:48 |

  ### Notes:

  - Cited by: 5
- ## Children's relationship with their pet dogs and OXTR genotype predict child-pet interaction in an experimental setting

  |  |  |
  | --- | --- |
  | Item Type | Journal Article |
  | Author | Darlene A. Kertes |
  | Author | Nathan Hall |
  | Author | Samarth S. Bhatt |
  | Date | 2018 |
  | URL | https://www.scopus.com/inward/record.uri?eid=2-s2.0-85053041882&doi=10.3389%2ffpsyg.2018.01472&partnerID=40&md5=b0a1ae416a7b9141e5ffeb0dccec6029 |
  | Extra | Type: Article |
  | Volume | 9 |
  | Publication | Frontiers in Psychology |
  | DOI | 10.3389/fpsyg.2018.01472 |
  | Issue | SEP |
  | Date Added | 20/06/2025, 09:37:48 |
  | Modified | 20/06/2025, 09:37:48 |

  ### Notes:

  - Cited by: 11; All Open Access, Gold Open Access, Green Open Access
- ## Pet dogs: Does their presence influence preadolescents' emotional responses to a social stressor?

  |  |  |
  | --- | --- |
  | Item Type | Journal Article |
  | Author | Kathryn A. Kerns |
  | Author | Kaela L. Stuart-Parrigon |
  | Author | Karin G. Coifman |
  | Author | Manfred H. M. van Dulmen |
  | Author | Amanda Koehn |
  | Date | 2018 |
  | URL | https://www.scopus.com/inward/record.uri?eid=2-s2.0-85040713658&doi=10.1111%2fsode.12246&partnerID=40&md5=c3b158300a4f880d5c655c2982f91619 |
  | Extra | Type: Article |
  | Volume | 27 |
  | Pages | 34 – 44 |
  | Publication | Social Development |
  | DOI | 10.1111/sode.12246 |
  | Issue | 1 |
  | Date Added | 19/06/2025, 15:40:12 |
  | Modified | 19/06/2025, 15:40:12 |

  ### Notes:

  - Cited by: 42; All Open Access, Green Open Access
- ## Flight distance at pasture and stress response during shearing in alpacas

  |  |  |
  | --- | --- |
  | Item Type | Journal Article |
  | Author | Joanna Kapustka |
  | Author | Monika Budzyńska |
  | Author | Olga Witkowska – Piłaszewicz |
  | Author | Julia Fabjanowska |
  | Date | 2023 |
  | URL | https://www.scopus.com/inward/record.uri?eid=2-s2.0-85152130256&doi=10.1016%2fj.jveb.2023.03.002&partnerID=40&md5=66bb76717315e3ef6dd73b5e2880680d |
  | Extra | Type: Article |
  | Volume | 63 |
  | Pages | 1 – 9 |
  | Publication | Journal of Veterinary Behavior |
  | DOI | 10.1016/j.jveb.2023.03.002 |
  | Date Added | 11/07/2025, 14:08:23 |
  | Modified | 11/07/2025, 14:08:23 |

  ### Notes:

  - Cited by: 2
- ## Positive Reinforcement Training for Blood Collection in Grizzly Bears (Ursus arctos horribilis) Results in Undetectable Elevations in Serum Cortisol Levels: A Preliminary Investigation

  |  |  |
  | --- | --- |
  | Item Type | Journal Article |
  | Author | Nicole M. Joyce-Zuniga |
  | Author | Ruth C. Newberry |
  | Author | Charles T. Robbins |
  | Author | Jasmine V. Ware |
  | Author | Heiko T. Jansen |
  | Author | O. Lynne Nelson |
  | Date | 2016 |
  | URL | https://www.scopus.com/inward/record.uri?eid=2-s2.0-84961216733&doi=10.1080%2f10888705.2015.1126523&partnerID=40&md5=ea0b56f08f6aa328a4810d64e525bebe |
  | Extra | Type: Article |
  | Volume | 19 |
  | Pages | 210 – 215 |
  | Publication | Journal of Applied Animal Welfare Science |
  | DOI | 10.1080/10888705.2015.1126523 |
  | Issue | 2 |
  | Date Added | 11/07/2025, 14:08:23 |
  | Modified | 11/07/2025, 14:08:23 |

  ### Notes:

  - Cited by: 24
- ## Interaction with Caged Budgerigars (Melopsittacus Undulatus) Enhances Human Affect

  |  |  |
  | --- | --- |
  | Item Type | Journal Article |
  | Author | Autumn G. Jones |
  | Author | Alexander J. Skolnick |
  | Author | Matthew J. Anderson |
  | Date | 2021 |
  | URL | https://www.scopus.com/inward/record.uri?eid=2-s2.0-85100706862&doi=10.1080%2f08927936.2021.1874113&partnerID=40&md5=85a44cebe7a6ae6d15027a511374c104 |
  | Extra | Type: Article |
  | Volume | 34 |
  | Pages | 127 – 138 |
  | Publication | Anthrozoos |
  | DOI | 10.1080/08927936.2021.1874113 |
  | Issue | 1 |
  | Date Added | 19/06/2025, 15:40:12 |
  | Modified | 19/06/2025, 15:40:12 |

  ### Notes:

  - Cited by: 0
- ## Exploring women’s oxytocin responses to interactions with their pet cats

  |  |  |
  | --- | --- |
  | Item Type | Journal Article |
  | Author | Elizabeth A. Johnson |
  | Author | Arianna Portillo |
  | Author | Nikki E. Bennett |
  | Author | Peter B. Gray |
  | Date | 2021 |
  | URL | https://www.scopus.com/inward/record.uri?eid=2-s2.0-85119353880&doi=10.7717%2fpeerj.12393&partnerID=40&md5=e4106a93cd10816b069656b961ecb2c3 |
  | Extra | Type: Article |
  | Volume | 9 |
  | Publication | PeerJ |
  | DOI | 10.7717/peerj.12393 |
  | Date Added | 20/06/2025, 09:37:47 |
  | Modified | 20/06/2025, 09:37:47 |

  ### Notes:

  - Cited by: 11; All Open Access, Gold Open Access, Green Open Access
- ## Can dogs serve as stress mediators to decrease salivary cortisol levels in a population of liberal arts college undergraduate students?

  |  |  |
  | --- | --- |
  | Item Type | Journal Article |
  | Author | Ana Gabriela Jimenez |
  | Author | Luke Calderaro |
  | Author | Sophia Clark |
  | Author | David Elacqua |
  | Author | Emily Hazen |
  | Author | Vanessa Lam |
  | Author | Grace S. Leightheiser |
  | Date | 2023 |
  | URL | https://www.scopus.com/inward/record.uri?eid=2-s2.0-85136245566&doi=10.1016%2fj.explore.2022.08.007&partnerID=40&md5=cca16433dc61ffb54b5eb26fbff0a87d |
  | Extra | Type: Article |
  | Volume | 19 |
  | Pages | 283 – 289 |
  | Publication | Explore |
  | DOI | 10.1016/j.explore.2022.08.007 |
  | Issue | 3 |
  | Date Added | 11/07/2025, 14:08:23 |
  | Modified | 11/07/2025, 14:08:23 |

  ### Notes:

  - Cited by: 2
- ## Effect of good handling on stress indicators and behaviour in beef cattle; [Efeito de boas práticas de manejo sobre indicadores de estresse e comportamento em bovinos de corte]

  |  |  |
  | --- | --- |
  | Item Type | Journal Article |
  | Author | Carla Comerlato Jardim |
  | Author | Isabella Dias Barbosa Silveira |
  | Author | João Restle |
  | Author | Fábio Souza Mendonça |
  | Author | Javier Alexander Bethancourt-Garcia |
  | Author | Roberson Macedo de Oliveira |
  | Author | Renata Espindola de Moraes |
  | Author | Nathália Pasi Reis |
  | Author | Ricardo Zambarda Vaz |
  | Date | 2022 |
  | URL | https://www.scopus.com/inward/record.uri?eid=2-s2.0-85144526648&doi=10.5433%2f1679-0359.2022v43n6p2517&partnerID=40&md5=acb1bcf5075b47327e1dd5f16b5573e4 |
  | Extra | Type: Article |
  | Volume | 43 |
  | Pages | 2517 – 2530 |
  | Publication | Semina:Ciencias Agrarias |
  | DOI | 10.5433/1679-0359.2022v43n6p2517 |
  | Issue | 6 |
  | Date Added | 11/07/2025, 14:08:23 |
  | Modified | 11/07/2025, 14:08:23 |

  ### Notes:

  - Cited by: 2; All Open Access, Gold Open Access
- ## Horses form cross-modal representations of adults and children

  |  |  |
  | --- | --- |
  | Item Type | Journal Article |
  | Author | Plotine Jardat |
  | Author | Monamie Ringhofer |
  | Author | Shinya Yamamoto |
  | Author | Chloé Gouyet |
  | Author | Rachel Degrande |
  | Author | Céline Parias |
  | Author | Fabrice Reigner |
  | Author | Ludovic Calandreau |
  | Author | Léa Lansade |
  | Date | 2023 |
  | URL | https://www.scopus.com/inward/record.uri?eid=2-s2.0-85135827488&doi=10.1007%2fs10071-022-01667-9&partnerID=40&md5=99d4df3489f383527eefae3ccaf51026 |
  | Extra | Type: Article |
  | Volume | 26 |
  | Pages | 369 – 377 |
  | Publication | Animal Cognition |
  | DOI | 10.1007/s10071-022-01667-9 |
  | Issue | 2 |
  | Date Added | 19/06/2025, 15:40:14 |
  | Modified | 19/06/2025, 15:40:14 |

  ### Notes:

  - Cited by: 6
- ## Companion animals as buffer against the impact of stress on affect: An experience sampling study

  |  |  |
  | --- | --- |
  | Item Type | Journal Article |
  | Author | Mayke Janssens |
  | Author | Erik Janssens |
  | Author | Jannes Eshuis |
  | Author | Johan Lataster |
  | Author | Marianne Simons |
  | Author | Jennifer Reijnders |
  | Author | Nele Jacobs |
  | Date | 2021 |
  | URL | https://www.scopus.com/inward/record.uri?eid=2-s2.0-85110566151&doi=10.3390%2fani11082171&partnerID=40&md5=da89645febe65e184ce9c6e7c3c82c9e |
  | Extra | Type: Article |
  | Volume | 11 |
  | Publication | Animals |
  | DOI | 10.3390/ani11082171 |
  | Issue | 8 |
  | Date Added | 20/06/2025, 09:37:47 |
  | Modified | 20/06/2025, 09:37:47 |

  ### Notes:

  - Cited by: 24; All Open Access, Gold Open Access, Green Open Access
- ## Physiological Effect of Gentle Stroking in Lambs

  |  |  |
  | --- | --- |
  | Item Type | Journal Article |
  | Author | Kamila Janicka |
  | Author | Patrycja Masier |
  | Author | Paulina Nazar |
  | Author | Patrycja Staniszewska |
  | Author | Grzegorz Zięba |
  | Author | Aneta Strachecka |
  | Author | Iwona Rozempolska-Rucińska |
  | Date | 2024 |
  | URL | https://www.scopus.com/inward/record.uri?eid=2-s2.0-85188713214&doi=10.3390%2fani14060887&partnerID=40&md5=358f764dab399440110de5f94a4170b7 |
  | Extra | Type: Article |
  | Volume | 14 |
  | Publication | Animals |
  | DOI | 10.3390/ani14060887 |
  | Issue | 6 |
  | Date Added | 19/06/2025, 15:40:12 |
  | Modified | 19/06/2025, 15:40:12 |

  ### Notes:

  - Cited by: 1; All Open Access, Gold Open Access, Green Open Access
- ## Physiological Effect of Gentle Stroking in Lambs

  |  |  |
  | --- | --- |
  | Item Type | Journal Article |
  | Author | Kamila Janicka |
  | Author | Patrycja Masier |
  | Author | Paulina Nazar |
  | Author | Patrycja Staniszewska |
  | Author | Grzegorz Zięba |
  | Author | Aneta Strachecka |
  | Author | Iwona Rozempolska-Rucińska |
  | Date | 2024 |
  | URL | https://www.scopus.com/inward/record.uri?eid=2-s2.0-85188713214&doi=10.3390%2fani14060887&partnerID=40&md5=358f764dab399440110de5f94a4170b7 |
  | Extra | Type: Article |
  | Volume | 14 |
  | Publication | Animals |
  | DOI | 10.3390/ani14060887 |
  | Issue | 6 |
  | Date Added | 11/07/2025, 14:08:22 |
  | Modified | 11/07/2025, 14:08:22 |

  ### Notes:

  - Cited by: 1; All Open Access, Gold Open Access, Green Open Access
- ## Atypical experiences of captive chimpanzees (Pan troglodytes) are associated with higher hair cortisol concentrations as adults

  |  |  |
  | --- | --- |
  | Item Type | Journal Article |
  | Author | S.L. Jacobson |
  | Author | H.D. Freeman |
  | Author | R.M. Santymire |
  | Author | S.R. Ross |
  | Date | 2017 |
  | URL | https://www.scopus.com/inward/record.uri?eid=2-s2.0-85038597170&doi=10.1098%2frsos.170932&partnerID=40&md5=2462809a44a3e0fe2d9f18399bd18a81 |
  | Extra | Type: Article |
  | Volume | 4 |
  | Publication | Royal Society Open Science |
  | DOI | 10.1098/rsos.170932 |
  | Issue | 12 |
  | Date Added | 11/07/2025, 14:08:22 |
  | Modified | 11/07/2025, 14:08:22 |

  ### Notes:

  - Cited by: 12; All Open Access, Gold Open Access, Green Open Access
- ## An aversive milker causes fear, but does not influence milk yield of holstein cows; [Influência de um ordenhador aversivo sobre a produção leiteira de vacas da raça holandesa]

  |  |  |
  | --- | --- |
  | Item Type | Journal Article |
  | Author | Maria José Hötzel |
  | Author | Luiz Carlos Pinheiro Machado Filho |
  | Author | Maria Cristina Yunes |
  | Author | Marcela Cristina A. C. Da Silveira |
  | Date | 2005 |
  | URL | https://www.scopus.com/inward/record.uri?eid=2-s2.0-31544454406&doi=10.1590%2fs1516-35982005000400024&partnerID=40&md5=dfcd8040c092f544ee59978e4c2652c1 |
  | Extra | Type: Article |
  | Volume | 34 |
  | Pages | 1278 – 1284 |
  | Publication | Revista Brasileira de Zootecnia |
  | DOI | 10.1590/s1516-35982005000400024 |
  | Issue | 4 |
  | Date Added | 20/06/2025, 09:37:47 |
  | Modified | 20/06/2025, 09:37:47 |

  ### Notes:

  - Cited by: 11; All Open Access, Gold Open Access
- ## Exploring the Dynamics of Canine-Assisted Interactions: A Wearable Approach to Understanding Interspecies Well-Being

  |  |  |
  | --- | --- |
  | Item Type | Journal Article |
  | Author | Timothy R. N. Holder |
  | Author | Colt Nichols |
  | Author | Emily Summers |
  | Author | David L. Roberts |
  | Author | Alper Bozkurt |
  | Date | 2024 |
  | URL | https://www.scopus.com/inward/record.uri?eid=2-s2.0-85213375550&doi=10.3390%2fani14243628&partnerID=40&md5=f5eb4ca70a3271d1bbba5f9c56a68b0c |
  | Extra | Type: Article |
  | Volume | 14 |
  | Publication | Animals |
  | DOI | 10.3390/ani14243628 |
  | Issue | 24 |
  | Date Added | 19/06/2025, 15:40:13 |
  | Modified | 19/06/2025, 15:40:13 |

  ### Notes:

  - Cited by: 1; All Open Access, Gold Open Access

  ### Attachments

  - PDF
- ## Oxytocin effects on the behavior in historical and experimental domesticated animals

  |  |  |
  | --- | --- |
  | Item Type | Journal Article |
  | Author | Yury E. Herbeck |
  | Author | Alisa A. Mаlyavko |
  | Author | Darya V. Shepeleva |
  | Date | 2022 |
  | URL | https://www.scopus.com/inward/record.uri?eid=2-s2.0-85132665157&doi=10.20333%2f25000136-2022-2-110&partnerID=40&md5=a41afc9b6edbb4f0b125a4e398bd615f |
  | Extra | Type: Article |
  | Volume | 2022 |
  | Pages | 110 |
  | Publication | Siberian Medical Review |
  | DOI | 10.20333/25000136-2022-2-110 |
  | Issue | 2 |
  | Date Added | 20/06/2025, 09:37:47 |
  | Modified | 20/06/2025, 09:37:47 |

  ### Notes:

  - Cited by: 0; All Open Access, Gold Open Access
- ## Relationships between human-animal interactions and productivity of commercial dairy cows

  |  |  |
  | --- | --- |
  | Item Type | Journal Article |
  | Author | P.H. Hemsworth |
  | Author | G.J. Coleman |
  | Author | J.L. Barnett |
  | Author | S. Borg |
  | Date | 2000 |
  | URL | https://www.scopus.com/inward/record.uri?eid=2-s2.0-0034328467&doi=10.2527%2f2000.78112821x&partnerID=40&md5=8970ba83f1dcc7c8fa974b93c444dab4 |
  | Extra | Type: Article |
  | Volume | 78 |
  | Pages | 2821 – 2831 |
  | Publication | Journal of Animal Science |
  | DOI | 10.2527/2000.78112821x |
  | Issue | 11 |
  | Date Added | 11/07/2025, 14:08:22 |
  | Modified | 11/07/2025, 14:08:22 |

  ### Notes:

  - Cited by: 272
- ## Relationships between handling, behaviour and stress in lambs at abattoirs

  |  |  |
  | --- | --- |
  | Item Type | Journal Article |
  | Author | P.H. Hemsworth |
  | Author | M. Rice |
  | Author | S. Borg |
  | Author | L.E. Edwards |
  | Author | E.N. Ponnampalam |
  | Author | G.J. Coleman |
  | Date | 2019 |
  | URL | https://www.scopus.com/inward/record.uri?eid=2-s2.0-85055483570&doi=10.1017%2fS1751731118002744&partnerID=40&md5=0d661ea540c9213361b1e03965223f1c |
  | Extra | Type: Article |
  | Volume | 13 |
  | Pages | 1287 – 1296 |
  | Publication | Animal |
  | DOI | 10.1017/S1751731118002744 |
  | Issue | 6 |
  | Date Added | 11/07/2025, 14:08:23 |
  | Modified | 11/07/2025, 14:08:23 |

  ### Notes:

  - Cited by: 23; All Open Access, Hybrid Gold Open Access
- ## Human-animal interactions at abattoirs: Relationships between handling and animal stress in sheep and cattle

  |  |  |
  | --- | --- |
  | Item Type | Journal Article |
  | Author | Paul H. Hemsworth |
  | Author | Maxine Rice |
  | Author | Marcus G. Karlen |
  | Author | Lisa Calleja |
  | Author | John L. Barnett |
  | Author | Judy Nash |
  | Author | Grahame J. Coleman |
  | Date | 2011 |
  | URL | https://www.scopus.com/inward/record.uri?eid=2-s2.0-82555165797&doi=10.1016%2fj.applanim.2011.09.007&partnerID=40&md5=cb41fb428bbfd4407913dddcffe5ba84 |
  | Extra | Type: Article |
  | Volume | 135 |
  | Pages | 24 – 33 |
  | Publication | Applied Animal Behaviour Science |
  | DOI | 10.1016/j.applanim.2011.09.007 |
  | Issue | 1-2 |
  | Date Added | 11/07/2025, 14:08:23 |
  | Modified | 11/07/2025, 14:08:23 |

  ### Notes:

  - Cited by: 145
- ## Exploratory investigation of infrared thermography for measuring gorilla emotional responses to interactions with familiar humans

  |  |  |
  | --- | --- |
  | Item Type | Journal Article |
  | Author | Matthew R. Heintz |
  | Author | Grace Fuller |
  | Author | Stephanie Allard |
  | Date | 2019 |
  | URL | https://www.scopus.com/inward/record.uri?eid=2-s2.0-85073324156&doi=10.3390%2fani9090604&partnerID=40&md5=650bc7acb1fc296393b3dadf25c06b55 |
  | Extra | Type: Article |
  | Volume | 9 |
  | Publication | Animals |
  | DOI | 10.3390/ani9090604 |
  | Issue | 9 |
  | Date Added | 20/06/2025, 09:37:48 |
  | Modified | 20/06/2025, 09:37:48 |

  ### Notes:

  - Cited by: 18; All Open Access, Gold Open Access, Green Open Access
- ## Exploratory investigation of infrared thermography for measuring gorilla emotional responses to interactions with familiar humans

  |  |  |
  | --- | --- |
  | Item Type | Journal Article |
  | Author | Matthew R. Heintz |
  | Author | Grace Fuller |
  | Author | Stephanie Allard |
  | Date | 2019 |
  | URL | https://www.scopus.com/inward/record.uri?eid=2-s2.0-85073324156&doi=10.3390%2fani9090604&partnerID=40&md5=650bc7acb1fc296393b3dadf25c06b55 |
  | Extra | Type: Article |
  | Volume | 9 |
  | Publication | Animals |
  | DOI | 10.3390/ani9090604 |
  | Issue | 9 |
  | Date Added | 11/07/2025, 14:08:23 |
  | Modified | 11/07/2025, 14:08:23 |

  ### Notes:

  - Cited by: 20; All Open Access, Gold Open Access, Green Open Access
- ## Effects of positive human contact during gestation on the behaviour, physiology and reproductive performance of sows

  |  |  |
  | --- | --- |
  | Item Type | Journal Article |
  | Author | Megan E. Hayes |
  | Author | Lauren M. Hemsworth |
  | Author | Rebecca S. Morrison |
  | Author | Kym L. Butler |
  | Author | Maxine Rice |
  | Author | Jean-Loup Rault |
  | Author | Paul H. Hemsworth |
  | Date | 2021 |
  | URL | https://www.scopus.com/inward/record.uri?eid=2-s2.0-85100057209&doi=10.3390%2fani11010214&partnerID=40&md5=817b484cba6bb58ea28730d40f61f61c |
  | Extra | Type: Article |
  | Volume | 11 |
  | Pages | 1 – 16 |
  | Publication | Animals |
  | DOI | 10.3390/ani11010214 |
  | Issue | 1 |
  | Date Added | 11/07/2025, 14:08:22 |
  | Modified | 11/07/2025, 14:08:22 |

  ### Notes:

  - Cited by: 12; All Open Access, Gold Open Access, Green Open Access
- ## Positive human contact and housing systems impact the responses of piglets to various stressors

  |  |  |
  | --- | --- |
  | Item Type | Journal Article |
  | Author | Megan E. Hayes |
  | Author | Lauren M. Hemsworth |
  | Author | Rebecca S. Morrison |
  | Author | Alan J. Tilbrook |
  | Author | Paul H. Hemsworth |
  | Date | 2021 |
  | URL | https://www.scopus.com/inward/record.uri?eid=2-s2.0-85106722041&doi=10.3390%2fani11061619&partnerID=40&md5=854ddd3010ce8f52874cbed474b20e02 |
  | Extra | Type: Article |
  | Volume | 11 |
  | Publication | Animals |
  | DOI | 10.3390/ani11061619 |
  | Issue | 6 |
  | Date Added | 11/07/2025, 14:08:23 |
  | Modified | 11/07/2025, 14:08:23 |

  ### Notes:

  - Cited by: 17; All Open Access, Gold Open Access, Green Open Access
- ## Assessing Whether Household Pets Buffer Responses to a Remote Stress Induction

  |  |  |
  | --- | --- |
  | Item Type | Journal Article |
  | Author | Helen M. K. Harvie |
  | Author | Alejandro Rodrigo |
  | Author | Ryan J. Giuliano |
  | Date | 2025 |
  | URL | https://www.scopus.com/inward/record.uri?eid=2-s2.0-105002370519&doi=10.1080%2f08927936.2025.2482328&partnerID=40&md5=2958562633142f8a893c0d9aa83abdf1 |
  | Extra | Type: Article |
  | Volume | 38 |
  | Pages | 545 – 564 |
  | Publication | Anthrozoos |
  | DOI | 10.1080/08927936.2025.2482328 |
  | Issue | 3 |
  | Date Added | 19/06/2025, 15:40:12 |
  | Modified | 19/06/2025, 15:40:12 |

  ### Notes:

  - Cited by: 0
- ## The Effects of a Therapy Dog on the Blood Pressure and Heart Rate of Older Residents in a Nursing Home

  |  |  |
  | --- | --- |
  | Item Type | Journal Article |
  | Author | Linda Handlin |
  | Author | Anne Nilsson |
  | Author | Lena Lidfors |
  | Author | Maria Petersson |
  | Author | Kerstin Uvnäs-Moberg |
  | Date | 2018 |
  | URL | https://www.scopus.com/inward/record.uri?eid=2-s2.0-85053562177&doi=10.1080%2f08927936.2018.1505268&partnerID=40&md5=f5df10df24102b3cfc38ca8ab400a307 |
  | Extra | Type: Article |
  | Volume | 31 |
  | Pages | 567 – 576 |
  | Publication | Anthrozoos |
  | DOI | 10.1080/08927936.2018.1505268 |
  | Issue | 5 |
  | Date Added | 19/06/2025, 15:40:13 |
  | Modified | 19/06/2025, 15:40:13 |

  ### Notes:

  - Cited by: 17; All Open Access, Bronze Open Access, Green Open Access
- ## Evaluating the effects of a temporary fostering program on shelter dog welfare

  |  |  |
  | --- | --- |
  | Item Type | Journal Article |
  | Author | Lisa M. Gunter |
  | Author | Erica N. Feuerbacher |
  | Author | Rachel J. Gilchrist |
  | Author | Clive D.L. Wynne |
  | Date | 2019 |
  | URL | https://www.scopus.com/inward/record.uri?eid=2-s2.0-85063676946&doi=10.7717%2fpeerj.6620&partnerID=40&md5=79a281911c2ca9f6d034841aacdc6ee2 |
  | Extra | Type: Article |
  | Volume | 2019 |
  | Publication | PeerJ |
  | DOI | 10.7717/peerj.6620 |
  | Issue | 3 |
  | Date Added | 19/06/2025, 15:40:13 |
  | Modified | 19/06/2025, 15:40:13 |

  ### Notes:

  - Cited by: 61; All Open Access, Gold Open Access, Green Open Access
- ## Evaluating the effects of a temporary fostering program on shelter dog welfare

  |  |  |
  | --- | --- |
  | Item Type | Journal Article |
  | Author | Lisa M. Gunter |
  | Author | Erica N. Feuerbacher |
  | Author | Rachel J. Gilchrist |
  | Author | Clive D.L. Wynne |
  | Date | 2019 |
  | URL | https://www.scopus.com/inward/record.uri?eid=2-s2.0-85063676946&doi=10.7717%2fpeerj.6620&partnerID=40&md5=79a281911c2ca9f6d034841aacdc6ee2 |
  | Extra | Type: Article |
  | Volume | 2019 |
  | Publication | PeerJ |
  | DOI | 10.7717/peerj.6620 |
  | Issue | 3 |
  | Date Added | 11/07/2025, 14:08:22 |
  | Modified | 11/07/2025, 14:08:22 |

  ### Notes:

  - Cited by: 61; All Open Access, Gold Open Access, Green Open Access
- ## Investigating the impact of brief outings on the welfare of dogs living in us shelters

  |  |  |
  | --- | --- |
  | Item Type | Journal Article |
  | Author | Lisa M. Gunter |
  | Author | Rachel J. Gilchrist |
  | Author | Emily M. Blade |
  | Author | Rebecca T. Barber |
  | Author | Erica N. Feuerbacher |
  | Author | Joanna M. Platzer |
  | Author | Clive D. L. Wynne |
  | Date | 2021 |
  | URL | https://www.scopus.com/inward/record.uri?eid=2-s2.0-85100947131&doi=10.3390%2fani11020548&partnerID=40&md5=4dadfe5d6e9a3936ba770c395c2486ea |
  | Extra | Type: Article |
  | Volume | 11 |
  | Pages | 1 – 15 |
  | Publication | Animals |
  | DOI | 10.3390/ani11020548 |
  | Issue | 2 |
  | Date Added | 11/07/2025, 14:08:23 |
  | Modified | 11/07/2025, 14:08:23 |

  ### Notes:

  - Cited by: 12; All Open Access, Gold Open Access, Green Open Access
- ## Investigating the impact of brief outings on the welfare of dogs living in us shelters

  |  |  |
  | --- | --- |
  | Item Type | Journal Article |
  | Author | Lisa M. Gunter |
  | Author | Rachel J. Gilchrist |
  | Author | Emily M. Blade |
  | Author | Rebecca T. Barber |
  | Author | Erica N. Feuerbacher |
  | Author | Joanna M. Platzer |
  | Author | Clive D. L. Wynne |
  | Date | 2021 |
  | URL | https://www.scopus.com/inward/record.uri?eid=2-s2.0-85100947131&doi=10.3390%2fani11020548&partnerID=40&md5=4dadfe5d6e9a3936ba770c395c2486ea |
  | Extra | Number: 2 Type: Article |
  | Volume | 11 |
  | Pages | 1 – 15 |
  | Publication | Animals |
  | DOI | 10.3390/ani11020548 |
  | Issue | 2 |
  | Date Added | 28/07/2025, 12:48:52 |
  | Modified | 28/07/2025, 12:48:52 |

  ### Notes:

  - Cited by: 12; All Open Access, Gold Open Access, Green Open Access
- ## The Influence of Brief Outing and Temporary Fostering Programs on Shelter Dog Welfare

  |  |  |
  | --- | --- |
  | Item Type | Journal Article |
  | Author | Lisa M. Gunter |
  | Author | Emily M. Blade |
  | Author | Rachel J. Gilchrist |
  | Author | Betsy J. Nixon |
  | Author | Jenifer L. Reed |
  | Author | Joanna M. Platzer |
  | Author | Ingrid C. Wurpts |
  | Author | Erica N. Feuerbacher |
  | Author | Clive D. L. Wynne |
  | Date | 2023 |
  | URL | https://www.scopus.com/inward/record.uri?eid=2-s2.0-85178340951&doi=10.3390%2fani13223528&partnerID=40&md5=d2594f7101e11e7dccd2383c70bfa634 |
  | Extra | Number: 22 Type: Article |
  | Volume | 13 |
  | Publication | Animals |
  | DOI | 10.3390/ani13223528 |
  | Issue | 22 |
  | Date Added | 28/07/2025, 12:48:52 |
  | Modified | 28/07/2025, 12:48:52 |

  ### Notes:

  - Cited by: 1; All Open Access, Gold Open Access, Green Open Access
- ## A wearable system for the evaluation of the human-horse interaction: A preliminary study

  |  |  |
  | --- | --- |
  | Item Type | Journal Article |
  | Author | Andrea Guidi |
  | Author | Antonio Lanata |
  | Author | Paolo Baragli |
  | Author | Gaetano Valenza |
  | Author | Enzo Pasquale Scilingo |
  | Date | 2016 |
  | URL | https://www.scopus.com/inward/record.uri?eid=2-s2.0-84991698546&doi=10.3390%2felectronics5040063&partnerID=40&md5=be2f249cfcbf5995397e1aac3cffb6b8 |
  | Extra | Type: Article |
  | Volume | 5 |
  | Publication | Electronics (Switzerland) |
  | DOI | 10.3390/electronics5040063 |
  | Issue | 4 |
  | Date Added | 19/06/2025, 15:40:13 |
  | Modified | 19/06/2025, 15:40:13 |

  ### Notes:

  - Cited by: 35; All Open Access, Gold Open Access, Green Open Access

  ### Attachments

  - PDF
- ## Assessing the Relationship Between Emotional States of Dogs and Their Human Handlers, Using Simultaneous Behavioral and Cardiac Measures

  |  |  |
  | --- | --- |
  | Item Type | Journal Article |
  | Author | Emma K. Grigg |
  | Author | Serene Liu |
  | Author | Denise G. Dempsey |
  | Author | Kylee Wong |
  | Author | Melissa Bain |
  | Author | John J. Sollers |
  | Author | Rani Haddock |
  | Author | Lori R. Kogan |
  | Author | Jennifer A. Barnhard |
  | Author | Ashley A. Tringali |
  | Author | Abigail P. Thigpen |
  | Author | Lynette A. Hart |
  | Date | 2022 |
  | URL | https://www.scopus.com/inward/record.uri?eid=2-s2.0-85134911057&doi=10.3389%2ffvets.2022.897287&partnerID=40&md5=dc1c7db926a688d9248b3adefe433302 |
  | Extra | Type: Article |
  | Volume | 9 |
  | Publication | Frontiers in Veterinary Science |
  | DOI | 10.3389/fvets.2022.897287 |
  | Date Added | 19/06/2025, 15:40:14 |
  | Modified | 19/06/2025, 15:40:14 |

  ### Notes:

  - Cited by: 4

  ### Attachments

  - PDF
- ## Do animals perceive human developmental disabilities? Guinea pigs’ behaviour with children with autism spectrum disorders and children with typical development. A pilot study

  |  |  |
  | --- | --- |
  | Item Type | Journal Article |
  | Author | Marine Grandgeorge |
  | Author | Elodie Dubois |
  | Author | Zarrin Alavi |
  | Author | Yannig Bourreau |
  | Author | Martine Hausberger |
  | Date | 2019 |
  | URL | https://www.scopus.com/inward/record.uri?eid=2-s2.0-85070654281&doi=10.3390%2fani9080522&partnerID=40&md5=2da4f6e654d8f48d94bf75878749ce0e |
  | Extra | Type: Article |
  | Volume | 9 |
  | Publication | Animals |
  | DOI | 10.3390/ani9080522 |
  | Issue | 8 |
  | Date Added | 19/06/2025, 15:40:12 |
  | Modified | 19/06/2025, 15:40:12 |

  ### Notes:

  - Cited by: 10
- ## Habituation of crossbred beef calves to corral handling reduces their reactivity and improves performance

  |  |  |
  | --- | --- |
  | Item Type | Journal Article |
  | Author | Joseph K. Grajales-Cedeño |
  | Author | Mateus J.R. Paranhos da Costa |
  | Date | 2024 |
  | URL | https://www.scopus.com/inward/record.uri?eid=2-s2.0-85198045138&doi=10.1016%2fj.applanim.2024.106343&partnerID=40&md5=78b880cbcce203142fc29978faff6c0b |
  | Extra | Type: Article |
  | Volume | 277 |
  | Publication | Applied Animal Behaviour Science |
  | DOI | 10.1016/j.applanim.2024.106343 |
  | Date Added | 19/06/2025, 15:40:13 |
  | Modified | 19/06/2025, 15:40:13 |

  ### Notes:

  - Cited by: 1
- ## Effects of human-animal interaction on salivary and urinary oxytocin in children and dogs

  |  |  |
  | --- | --- |
  | Item Type | Journal Article |
  | Author | Gitanjali E. Gnanadesikan |
  | Author | Katherine M. King |
  | Author | Elizabeth Carranza |
  | Author | Abigail C. Flyer |
  | Author | Gianna Ossello |
  | Author | Paige G. Smith |
  | Author | Netzin G. Steklis |
  | Author | H. Dieter Steklis |
  | Author | C. Sue Carter |
  | Author | Jessica J. Connelly |
  | Author | Melissa Barnett |
  | Author | Nancy Gee |
  | Author | Stacey R. Tecot |
  | Author | Evan L. MacLean |
  | Date | 2024 |
  | URL | https://www.scopus.com/inward/record.uri?eid=2-s2.0-85199938687&doi=10.1016%2fj.psyneuen.2024.107147&partnerID=40&md5=678a7fea07f2a7f3ab186bbb2a1167ab |
  | Extra | Type: Article |
  | Volume | 169 |
  | Publication | Psychoneuroendocrinology |
  | DOI | 10.1016/j.psyneuen.2024.107147 |
  | Date Added | 20/06/2025, 09:37:47 |
  | Modified | 20/06/2025, 09:37:47 |

  ### Notes:

  - Cited by: 2

  ### Attachments

  - PDF
- ## Glucocorticoid response to naturalistic interactions between children and dogs

  |  |  |
  | --- | --- |
  | Item Type | Journal Article |
  | Author | Gitanjali E. Gnanadesikan |
  | Author | Elizabeth Carranza |
  | Author | Katherine M. King |
  | Author | Abigail C. Flyer |
  | Author | Gianna Ossello |
  | Author | Paige G. Smith |
  | Author | Netzin G. Steklis |
  | Author | H. Dieter Steklis |
  | Author | Jessica J. Connelly |
  | Author | Melissa Barnett |
  | Author | Nancy Gee |
  | Author | Stacey Tecot |
  | Author | Evan L. MacLean |
  | Date | 2024 |
  | URL | https://www.scopus.com/inward/record.uri?eid=2-s2.0-85187689343&doi=10.1016%2fj.yhbeh.2024.105523&partnerID=40&md5=5dcb2e880f486ae8ce6ba65e652f9667 |
  | Extra | Type: Article |
  | Volume | 161 |
  | Publication | Hormones and Behavior |
  | DOI | 10.1016/j.yhbeh.2024.105523 |
  | Date Added | 11/07/2025, 14:08:22 |
  | Modified | 11/07/2025, 14:08:22 |

  ### Notes:

  - Cited by: 3
- ## Observing live fish improves perceptions of mood, relaxation and anxiety, but does not consistently alter heart rate or heart rate variability

  |  |  |
  | --- | --- |
  | Item Type | Journal Article |
  | Author | Nancy R. Gee |
  | Author | Taylor Reed |
  | Author | April Whiting |
  | Author | Erika Friedmann |
  | Author | Donna Snellgrove |
  | Author | Katherine A. Sloman |
  | Date | 2019 |
  | URL | https://www.scopus.com/inward/record.uri?eid=2-s2.0-85071608592&doi=10.3390%2fijerph16173113&partnerID=40&md5=042b668e6ad559b9cf52ea20737b5d99 |
  | Extra | Type: Article |
  | Volume | 16 |
  | Publication | International Journal of Environmental Research and Public Health |
  | DOI | 10.3390/ijerph16173113 |
  | Issue | 17 |
  | Date Added | 19/06/2025, 15:40:12 |
  | Modified | 19/06/2025, 15:40:12 |

  ### Notes:

  - Cited by: 14; All Open Access, Gold Open Access, Green Open Access
- ## Observing live fish improves perceptions of mood, relaxation and anxiety, but does not consistently alter heart rate or heart rate variability

  |  |  |
  | --- | --- |
  | Item Type | Journal Article |
  | Author | Nancy R. Gee |
  | Author | Taylor Reed |
  | Author | April Whiting |
  | Author | Erika Friedmann |
  | Author | Donna Snellgrove |
  | Author | Katherine A. Sloman |
  | Date | 2019 |
  | URL | https://www.scopus.com/inward/record.uri?eid=2-s2.0-85071608592&doi=10.3390%2fijerph16173113&partnerID=40&md5=042b668e6ad559b9cf52ea20737b5d99 |
  | Extra | Number: 17 Type: Article |
  | Volume | 16 |
  | Publication | International Journal of Environmental Research and Public Health |
  | DOI | 10.3390/ijerph16173113 |
  | Issue | 17 |
  | Date Added | 28/07/2025, 12:48:52 |
  | Modified | 28/07/2025, 12:48:52 |

  ### Notes:

  - Cited by: 14; All Open Access, Gold Open Access, Green Open Access
- ## Visitor effects on the welfare of captive Sumatran orangutans (Pongo abelii) during the pandemic lockdowns

  |  |  |
  | --- | --- |
  | Item Type | Journal Article |
  | Author | Ezekiel F. Gading |
  | Author | Valerie Am Schoof |
  | Author | Maria Franke |
  | Author | Suzanne E. MacDonald |
  | Abstract | The COVID-19 pandemic led to unprecedented lockdowns with rippling impacts on the lives of humans and animals alike. Since zoos were among the first institutions to close during the pandemic, the lockdowns presented the opportunity to conduct a natural experiment examining the relationship between visitor presence and the welfare of zoo-housed animals. In this study, we assessed the welfare of six Sumatran orangutans (Pongo abelii) at Toronto Zoo both during and following the pandemic lockdowns. We compared behavioural and physiological indicators of welfare during a lockdown and after visitors were reintroduced. Specifically, if the orangutans' welfare was affected by the visitor re-introduction phase we predicted there would be an increase in the following measures: (1) use of exhibit areas away from visitors; (2) behavioural measures (hiding, self-directed behaviours, agonistic behaviours, agitated movement, and idiosyncratic object-directed behaviours [head slamming, and fabric tearing]); and (3) physiological measures (faecal consistency and glucocorticoid metabolites) when compared to the lockdown. We also measured changes in activity levels such as foraging and inactivity. We found that orangutan exhibit space use did not change when visitors were reintroduced. In fact, the orangutans hid less when visitors were introduced than during the lockdown. Foraging, inactivity, and other behavioural indicators of stress did not change when visitors were introduced. Similarly, neither faecal consistency nor glucocorticoid metabolites changed across the study phases. Our data show that visitor re-introduction did not negatively affect the welfare of the Toronto Zoo orangutans. However, the presence of keepers was found to affect the behaviour of the orangutans and warrants further study. |
  | Date | 2025 |
  | Language | eng |
  | Library Catalogue | PubMed |
  | Volume | 34 |
  | Pages | e15 |
  | Publication | Animal Welfare (South Mimms, England) |
  | DOI | 10.1017/awf.2025.9 |
  | Journal Abbr | Anim Welf |
  | ISSN | 2054-1538 |
  | PMID | 40071106 |
  | PMCID | PMC11894407 |
  | Date Added | 11/07/2025, 11:33:21 |
  | Modified | 04/01/2026, 11:26:06 |

  ### Tags:

  - human-animal interaction
  - stress
  - animal welfare
  - behaviour
  - COVID-19
  - primates
  - agitation
  - animal behavior
  - animal experiment
  - article
  - Canada
  - coronavirus disease 2019
  - defecation
  - female
  - foraging
  - glucocorticoid
  - lockdown
  - locomotion
  - male
  - metabolite
  - nonhuman
  - orangutan
  - pandemic
  - physiological stress
  - play
  - Pongo abelii
  - primate
  - social interaction
  - zoo animal

  ### Notes:

  - Cited by: 0; All Open Access, Gold Open Access

  ### Attachments

  - Full Text (HTML)
  - PubMed entry
- ## Human physiological responses to different types of human-dog interactions: A randomised crossover study

  |  |  |
  | --- | --- |
  | Item Type | Journal Article |
  | Author | Lene Høeg Fuglsang-Damgaard |
  | Author | Sigrid Juhl Lunde |
  | Author | Janne Winther Christensen |
  | Author | Lene Vase |
  | Author | Poul B. Videbech |
  | Author | Karen Thodberg |
  | Date | 2024 |
  | URL | https://www.scopus.com/inward/record.uri?eid=2-s2.0-85202544805&doi=10.1016%2fj.ctcp.2024.101899&partnerID=40&md5=5eb899bfaca4a95092a52a52f5306297 |
  | Extra | Type: Article |
  | Volume | 57 |
  | Publication | Complementary Therapies in Clinical Practice |
  | DOI | 10.1016/j.ctcp.2024.101899 |
  | Date Added | 19/06/2025, 15:40:12 |
  | Modified | 19/06/2025, 15:40:12 |

  ### Notes:

  - Cited by: 0; All Open Access, Hybrid Gold Open Access
- ## Human physiological responses to different types of human-dog interactions: A randomised crossover study

  |  |  |
  | --- | --- |
  | Item Type | Journal Article |
  | Author | Lene Høeg Fuglsang-Damgaard |
  | Author | Sigrid Juhl Lunde |
  | Author | Janne Winther Christensen |
  | Author | Lene Vase |
  | Author | Poul B. Videbech |
  | Author | Karen Thodberg |
  | Date | 2024 |
  | URL | https://www.scopus.com/inward/record.uri?eid=2-s2.0-85202544805&doi=10.1016%2fj.ctcp.2024.101899&partnerID=40&md5=5eb899bfaca4a95092a52a52f5306297 |
  | Extra | Type: Article |
  | Volume | 57 |
  | Publication | Complementary Therapies in Clinical Practice |
  | DOI | 10.1016/j.ctcp.2024.101899 |
  | Date Added | 11/07/2025, 14:08:22 |
  | Modified | 11/07/2025, 14:08:22 |

  ### Notes:

  - Cited by: 0; All Open Access, Hybrid Gold Open Access
- ## Physiology of human-horse interactions during substance withdrawal within psychotherapy participants

  |  |  |
  | --- | --- |
  | Item Type | Journal Article |
  | Author | M.M. Friend |
  | Author | M.C. Nicodemus |
  | Author | C.A. Cavinder |
  | Author | C.O. Lemley |
  | Author | P. Prince |
  | Author | K. Holtcamp |
  | Author | R.M. Swanson |
  | Date | 2023 |
  | URL | https://www.scopus.com/inward/record.uri?eid=2-s2.0-85184573882&doi=10.1163%2f17552559-20230023&partnerID=40&md5=3cfbd9dddf274b88c9ffdc1b980da655 |
  | Extra | Type: Article |
  | Volume | 20 |
  | Pages | 55 – 68 |
  | Publication | Comparative Exercise Physiology |
  | DOI | 10.1163/17552559-20230023 |
  | Issue | 1 |
  | Date Added | 11/07/2025, 14:08:23 |
  | Modified | 11/07/2025, 14:08:23 |

  ### Notes:

  - Cited by: 5
- ## A Leash on Life: An Exploratory Study on the Effects of a Shelter-Dog Walking Program for Veterans on Dogs’ Stress

  |  |  |
  | --- | --- |
  | Item Type | Journal Article |
  | Author | Erika Friedmann |
  | Author | Cheryl A. Krause-Parello |
  | Author | Megan Payton |
  | Author | Kelly Blanchard |
  | Author | Asha Storm |
  | Author | Erik Barr |
  | Author | Nancy R. Gee |
  | Date | 2022 |
  | URL | https://www.scopus.com/inward/record.uri?eid=2-s2.0-85110585971&doi=10.1080%2f08927936.2021.1944559&partnerID=40&md5=9a499194d5c08209585b568bb7ee4ffd |
  | Extra | Type: Article |
  | Volume | 35 |
  | Pages | 23 – 36 |
  | Publication | Anthrozoos |
  | DOI | 10.1080/08927936.2021.1944559 |
  | Issue | 1 |
  | Date Added | 19/06/2025, 15:40:14 |
  | Modified | 19/06/2025, 15:40:14 |

  ### Notes:

  - Cited by: 2
- ## The Power of Discourse: Associations between Trainers’ Speech and the Responses of Socialized Wolves and Dogs to Training

  |  |  |
  | --- | --- |
  | Item Type | Journal Article |
  | Author | Melissa Gabriela Bravo Fonseca |
  | Author | Heron Oliveira Hilário |
  | Author | Kurt Kotrschal |
  | Author | Friederike Range |
  | Author | Zsófia Virányi |
  | Author | Marina Henriques Lage Duarte |
  | Author | Laryssa Cristina Gomes Pereira |
  | Author | Angélica da Silva Vasconcellos |
  | Date | 2023 |
  | URL | https://www.scopus.com/inward/record.uri?eid=2-s2.0-85151383593&doi=10.3390%2fani13061071&partnerID=40&md5=beaa7f89a9d80dba5f9fa28db6fd6513 |
  | Extra | Type: Article |
  | Volume | 13 |
  | Publication | Animals |
  | DOI | 10.3390/ani13061071 |
  | Issue | 6 |
  | Date Added | 11/07/2025, 14:08:23 |
  | Modified | 11/07/2025, 14:08:23 |

  ### Notes:

  - Cited by: 2; All Open Access, Gold Open Access, Green Open Access
- ## The Effect of Therapy Dogs on Preoperative Anxiety

  |  |  |
  | --- | --- |
  | Item Type | Journal Article |
  | Author | Preston Foerder |
  | Author | Morgan Royer |
  | Date | 2021 |
  | URL | https://www.scopus.com/inward/record.uri?eid=2-s2.0-85105350660&doi=10.1080%2f08927936.2021.1914440&partnerID=40&md5=dbc977010369b20dea94273acd0da2ed |
  | Extra | Type: Article |
  | Volume | 34 |
  | Pages | 659 – 670 |
  | Publication | Anthrozoos |
  | DOI | 10.1080/08927936.2021.1914440 |
  | Issue | 5 |
  | Date Added | 19/06/2025, 15:40:13 |
  | Modified | 19/06/2025, 15:40:13 |

  ### Notes:

  - Cited by: 4
- ## Development of an evidence-based welfare approach for cheetahs (Acinonyx jubatus) under human care

  |  |  |
  | --- | --- |
  | Item Type | Journal Article |
  | Author | B. Fischer |
  | Author | M. Flint |
  | Author | K. Cole |
  | Author | K.A. George |
  | Date | 2021 |
  | URL | https://www.scopus.com/inward/record.uri?eid=2-s2.0-85159875330&doi=10.7120%2f09627286.30.3.006&partnerID=40&md5=2a839e8190d9710e36f15f77899a4a79 |
  | Extra | Type: Article |
  | Volume | 30 |
  | Pages | 295 – 306 |
  | Publication | Animal Welfare |
  | DOI | 10.7120/09627286.30.3.006 |
  | Issue | 3 |
  | Date Added | 11/07/2025, 14:08:23 |
  | Modified | 11/07/2025, 14:08:23 |

  ### Notes:

  - Cited by: 1
- ## Short-term equine interaction for reducing test anxiety and facilitating coping skill development in college students during examination periods: A preliminary study

  |  |  |
  | --- | --- |
  | Item Type | Journal Article |
  | Author | K. Everett |
  | Author | M.M. Friend |
  | Author | E. Farnlacher |
  | Author | A. Hilliard |
  | Author | M.C. Nicodemus |
  | Author | C.A. Cavinder |
  | Author | K. Holtcamp |
  | Author | D. Jousan |
  | Date | 2024 |
  | URL | https://www.scopus.com/inward/record.uri?eid=2-s2.0-85192457536&doi=10.1016%2fj.jevs.2024.105091&partnerID=40&md5=0a5a45f8009be84e7b0bd695d3136fa4 |
  | Extra | Type: Article |
  | Volume | 137 |
  | Publication | Journal of Equine Veterinary Science |
  | DOI | 10.1016/j.jevs.2024.105091 |
  | Date Added | 19/06/2025, 15:40:13 |
  | Modified | 19/06/2025, 15:40:13 |

  ### Notes:

  - Cited by: 0
- ## Short-term equine interaction for reducing test anxiety and facilitating coping skill development in college students during examination periods: A preliminary study

  |  |  |
  | --- | --- |
  | Item Type | Journal Article |
  | Author | K. Everett |
  | Author | M.M. Friend |
  | Author | E. Farnlacher |
  | Author | A. Hilliard |
  | Author | M.C. Nicodemus |
  | Author | C.A. Cavinder |
  | Author | K. Holtcamp |
  | Author | D. Jousan |
  | Date | 2024 |
  | URL | https://www.scopus.com/inward/record.uri?eid=2-s2.0-85192457536&doi=10.1016%2fj.jevs.2024.105091&partnerID=40&md5=0a5a45f8009be84e7b0bd695d3136fa4 |
  | Extra | Type: Article |
  | Volume | 137 |
  | Publication | Journal of Equine Veterinary Science |
  | DOI | 10.1016/j.jevs.2024.105091 |
  | Date Added | 11/07/2025, 14:08:23 |
  | Modified | 11/07/2025, 14:08:23 |

  ### Notes:

  - Cited by: 0
- ## Short-term equine interaction for reducing test anxiety and facilitating coping skill development in college students during examination periods: A preliminary study

  |  |  |
  | --- | --- |
  | Item Type | Journal Article |
  | Author | K. Everett |
  | Author | M.M. Friend |
  | Author | E. Farnlacher |
  | Author | A. Hilliard |
  | Author | M.C. Nicodemus |
  | Author | C.A. Cavinder |
  | Author | K. Holtcamp |
  | Author | D. Jousan |
  | Date | 2024 |
  | URL | https://www.scopus.com/inward/record.uri?eid=2-s2.0-85192457536&doi=10.1016%2fj.jevs.2024.105091&partnerID=40&md5=0a5a45f8009be84e7b0bd695d3136fa4 |
  | Extra | Type: Article |
  | Volume | 137 |
  | Publication | Journal of Equine Veterinary Science |
  | DOI | 10.1016/j.jevs.2024.105091 |
  | Date Added | 28/07/2025, 12:48:52 |
  | Modified | 28/07/2025, 12:48:52 |

  ### Notes:

  - Cited by: 0
- ## The Effect of Dog Videos on Subjective and Physiological Responses to Stress

  |  |  |
  | --- | --- |
  | Item Type | Journal Article |
  | Author | Natalie Ein |
  | Author | Maureen J. Reed |
  | Author | Kristin Vickers |
  | Date | 2022 |
  | URL | https://www.scopus.com/inward/record.uri?eid=2-s2.0-85119422477&doi=10.1080%2f08927936.2021.1999606&partnerID=40&md5=06a9073a562dea6247d075f64c3fab96 |
  | Extra | Type: Article |
  | Volume | 35 |
  | Pages | 463 – 482 |
  | Publication | Anthrozoos |
  | DOI | 10.1080/08927936.2021.1999606 |
  | Issue | 3 |
  | Date Added | 19/06/2025, 15:40:12 |
  | Modified | 19/06/2025, 15:40:12 |

  ### Notes:

  - Cited by: 7
- ## Effects on Wellbeing of Exposure to Dog Videos Before a Stressor

  |  |  |
  | --- | --- |
  | Item Type | Journal Article |
  | Author | Natalie Ein |
  | Author | Julia Gervasio |
  | Author | Maureen J. Reed |
  | Author | Kristin Vickers |
  | Date | 2023 |
  | URL | https://www.scopus.com/inward/record.uri?eid=2-s2.0-85144045934&doi=10.1080%2f08927936.2022.2149925&partnerID=40&md5=cc384dc74c08d11ab4beaa6d1cd5b8b3 |
  | Extra | Type: Article |
  | Volume | 36 |
  | Pages | 349 – 367 |
  | Publication | Anthrozoos |
  | DOI | 10.1080/08927936.2022.2149925 |
  | Issue | 3 |
  | Date Added | 19/06/2025, 15:40:14 |
  | Modified | 19/06/2025, 15:40:14 |

  ### Notes:

  - Cited by: 4
- ## The Interface of Caretaker and Animal Well-being As a Critical Component of Sustainability

  |  |  |
  | --- | --- |
  | Item Type | Journal Article |
  | Author | Lily N. Edwards-Callaway |
  | Author | Paxton A. Sullivan |
  | Date | 2024 |
  | URL | https://www.scopus.com/inward/record.uri?eid=2-s2.0-105007196381&doi=10.22175%2fmmb.18196&partnerID=40&md5=4ebf4796d8a23fac09430b5678706f6d |
  | Extra | Type: Article |
  | Volume | 8 |
  | Publication | Meat and Muscle Biology |
  | DOI | 10.22175/mmb.18196 |
  | Issue | 1 |
  | Date Added | 19/06/2025, 15:40:12 |
  | Modified | 19/06/2025, 15:40:12 |

  ### Notes:

  - Cited by: 1
- ## The Interface of Caretaker and Animal Well-being As a Critical Component of Sustainability

  |  |  |
  | --- | --- |
  | Item Type | Journal Article |
  | Author | Lily N. Edwards-Callaway |
  | Author | Paxton A. Sullivan |
  | Date | 2024 |
  | URL | https://www.scopus.com/inward/record.uri?eid=2-s2.0-105007196381&doi=10.22175%2fmmb.18196&partnerID=40&md5=4ebf4796d8a23fac09430b5678706f6d |
  | Extra | Number: 1 Type: Article |
  | Volume | 8 |
  | Publication | Meat and Muscle Biology |
  | DOI | 10.22175/mmb.18196 |
  | Issue | 1 |
  | Date Added | 28/07/2025, 12:48:52 |
  | Modified | 28/07/2025, 12:48:52 |

  ### Notes:

  - Cited by: 1
- ## Fecal cortisol metabolites in dairy cows: A cross-sectional exploration of associations with animal, stockperson, and farm characteristics

  |  |  |
  | --- | --- |
  | Item Type | Journal Article |
  | Author | Asja Ebinghaus |
  | Author | Ute Knierim |
  | Author | Christel Simantke |
  | Author | Rupert Palme |
  | Author | Silvia Ivemeyer |
  | Date | 2020 |
  | URL | https://www.scopus.com/inward/record.uri?eid=2-s2.0-85091914650&doi=10.3390%2fani10101787&partnerID=40&md5=c932c221ad862357b72cc4e06c39034e |
  | Extra | Type: Article |
  | Volume | 10 |
  | Pages | 1 – 17 |
  | Publication | Animals |
  | DOI | 10.3390/ani10101787 |
  | Issue | 10 |
  | Date Added | 11/07/2025, 14:08:22 |
  | Modified | 11/07/2025, 14:08:22 |

  ### Notes:

  - Cited by: 20; All Open Access, Gold Open Access, Green Open Access
- ## Assessing the effect of counterconditioning to reduce neophobic response in horses (Equus ferus Caballus)

  |  |  |
  | --- | --- |
  | Item Type | Journal Article |
  | Author | Francisca Droguett |
  | Author | Gonzalo Miguez |
  | Author | Vanetza E. Quezada-Scholz |
  | Author | Tamara Tadich |
  | Author | Mario A. Laborda |
  | Date | 2024 |
  | URL | https://www.scopus.com/inward/record.uri?eid=2-s2.0-85209728389&doi=10.1016%2fj.applanim.2024.106453&partnerID=40&md5=ed48b1900de2ec88002fa98b93a56d2a |
  | Extra | Type: Article |
  | Volume | 281 |
  | Publication | Applied Animal Behaviour Science |
  | DOI | 10.1016/j.applanim.2024.106453 |
  | Date Added | 19/06/2025, 15:40:13 |
  | Modified | 19/06/2025, 15:40:13 |

  ### Notes:

  - Cited by: 0
- ## Methods of collection for salivary cortisol measurement in dogs

  |  |  |
  | --- | --- |
  | Item Type | Journal Article |
  | Author | Nancy A. Dreschel |
  | Author | Douglas A. Granger |
  | Date | 2009 |
  | URL | https://www.scopus.com/inward/record.uri?eid=2-s2.0-58149129683&doi=10.1016%2fj.yhbeh.2008.09.010&partnerID=40&md5=689fd8329a8e5d671fd85c701f16b0d3 |
  | Extra | Type: Article |
  | Volume | 55 |
  | Pages | 163 – 168 |
  | Publication | Hormones and Behavior |
  | DOI | 10.1016/j.yhbeh.2008.09.010 |
  | Issue | 1 |
  | Date Added | 11/07/2025, 14:08:23 |
  | Modified | 11/07/2025, 14:08:23 |

  ### Notes:

  - Cited by: 96
- ## Oxytocin in the human-dog bond: Review of the literature and analysis of future investigation fields; [La oxitocina en el vínculo humano-perro: Revisión bibliográfica y análisis de futuras áreas de investigación]

  |  |  |
  | --- | --- |
  | Item Type | Journal Article |
  | Author | Marcos Díaz Videla |
  | Author | Pablo Adrián López |
  | Date | 2017 |
  | URL | https://www.scopus.com/inward/record.uri?eid=2-s2.0-85030679726&partnerID=40&md5=5fbabb487d206c5141963a554de02e32 |
  | Extra | Type: Article |
  | Volume | 34 |
  | Pages | 73 – 90 |
  | Publication | Interdisciplinaria |
  | Issue | 1 |
  | Date Added | 20/06/2025, 09:37:48 |
  | Modified | 20/06/2025, 09:37:48 |

  ### Notes:

  - Cited by: 5
- ## Oxytocin in the human-dog bond: Review of the literature and analysis of future investigation fields; [La oxitocina en el vínculo humano-perro: Revisión bibliográfica y análisis de futuras áreas de investigación]

  |  |  |
  | --- | --- |
  | Item Type | Journal Article |
  | Author | Marcos Díaz Videla |
  | Author | Pablo Adrián López |
  | Date | 2017 |
  | URL | https://www.scopus.com/inward/record.uri?eid=2-s2.0-85030679726&partnerID=40&md5=5fbabb487d206c5141963a554de02e32 |
  | Extra | Type: Article |
  | Volume | 34 |
  | Pages | 73 – 90 |
  | Publication | Interdisciplinaria |
  | Issue | 1 |
  | Date Added | 11/07/2025, 14:08:22 |
  | Modified | 11/07/2025, 14:08:22 |

  ### Notes:

  - Cited by: 5
- ## The power of interspecific sociality: how humans provide social buffering for horses

  |  |  |
  | --- | --- |
  | Item Type | Journal Article |
  | Author | Alfredo Di Lucrezia |
  | Author | Anna Scandurra |
  | Author | Daria Lotito |
  | Author | Valeria Iervolino |
  | Author | Biagio D’Aniello |
  | Author | Vincenzo Mastellone |
  | Author | Pietro Lombardi |
  | Author | Claudia Pinelli |
  | Date | 2025 |
  | URL | https://www.scopus.com/inward/record.uri?eid=2-s2.0-105000068935&doi=10.1007%2fs10071-025-01942-5&partnerID=40&md5=c9aca2814db26104c028300f725eb326 |
  | Extra | Type: Article |
  | Volume | 28 |
  | Publication | Animal Cognition |
  | DOI | 10.1007/s10071-025-01942-5 |
  | Issue | 1 |
  | Date Added | 19/06/2025, 15:40:13 |
  | Modified | 19/06/2025, 15:40:13 |

  ### Notes:

  - Cited by: 1; All Open Access, Gold Open Access
- ## The role of human presence as social buffer for dairy cattle

  |  |  |
  | --- | --- |
  | Item Type | Journal Article |
  | Author | Alfredo Di Lucrezia |
  | Author | Samuel Bagnato |
  | Author | Valeria Iervolino |
  | Author | Biagio D'Aniello |
  | Author | Claudia Pinelli |
  | Author | Pietro Lombardi |
  | Author | Maria Elena Pero |
  | Author | Vincenzo Mastellone |
  | Date | 2025 |
  | URL | https://www.scopus.com/inward/record.uri?eid=2-s2.0-105009589650&doi=10.1016%2fj.applanim.2025.106739&partnerID=40&md5=cec7a405dd860df5412a4fccb12fb20b |
  | Extra | Type: Article |
  | Volume | 291 |
  | Publication | Applied Animal Behaviour Science |
  | DOI | 10.1016/j.applanim.2025.106739 |
  | Date Added | 11/07/2025, 14:08:23 |
  | Modified | 11/07/2025, 14:08:23 |

  ### Notes:

  - Cited by: 0
- ## The power of interspecific sociality: how humans provide social buffering for horses

  |  |  |
  | --- | --- |
  | Item Type | Journal Article |
  | Author | Alfredo Di Lucrezia |
  | Author | Anna Scandurra |
  | Author | Daria Lotito |
  | Author | Valeria Iervolino |
  | Author | Biagio D’Aniello |
  | Author | Vincenzo Mastellone |
  | Author | Pietro Lombardi |
  | Author | Claudia Pinelli |
  | Date | 2025 |
  | URL | https://www.scopus.com/inward/record.uri?eid=2-s2.0-105000068935&doi=10.1007%2fs10071-025-01942-5&partnerID=40&md5=c9aca2814db26104c028300f725eb326 |
  | Extra | Type: Article |
  | Volume | 28 |
  | Publication | Animal Cognition |
  | DOI | 10.1007/s10071-025-01942-5 |
  | Issue | 1 |
  | Date Added | 11/07/2025, 14:08:23 |
  | Modified | 11/07/2025, 14:08:23 |

  ### Notes:

  - Cited by: 2
- ## Effects of brushing dairy calves on behaviour, physiology and genes expression

  |  |  |
  | --- | --- |
  | Item Type | Journal Article |
  | Author | Karolini Tenffen De-Sousa |
  | Author | Catiúcia Oliveira Miranda |
  | Author | João Alberto Negrão |
  | Author | Aníbal Eugênio Vercesi Filho |
  | Author | Marcia Saladini Vieira Salles |
  | Author | Flavia Fernanda Simili |
  | Author | Lenira El Faro |
  | Date | 2025 |
  | URL | https://www.scopus.com/inward/record.uri?eid=2-s2.0-85212080145&doi=10.1016%2fj.applanim.2024.106456&partnerID=40&md5=f314ae65470bc35c59c4a8ecae6229d5 |
  | Extra | Type: Article |
  | Volume | 282 |
  | Publication | Applied Animal Behaviour Science |
  | DOI | 10.1016/j.applanim.2024.106456 |
  | Date Added | 20/06/2025, 09:37:47 |
  | Modified | 20/06/2025, 09:37:47 |

  ### Notes:

  - Cited by: 0
- ## Effects of brushing dairy calves on behaviour, physiology and genes expression

  |  |  |
  | --- | --- |
  | Item Type | Journal Article |
  | Author | Karolini Tenffen De-Sousa |
  | Author | Catiúcia Oliveira Miranda |
  | Author | João Alberto Negrão |
  | Author | Aníbal Eugênio Vercesi Filho |
  | Author | Marcia Saladini Vieira Salles |
  | Author | Flavia Fernanda Simili |
  | Author | Lenira El Faro |
  | Date | 2025 |
  | URL | https://www.scopus.com/inward/record.uri?eid=2-s2.0-85212080145&doi=10.1016%2fj.applanim.2024.106456&partnerID=40&md5=f314ae65470bc35c59c4a8ecae6229d5 |
  | Extra | Type: Article |
  | Volume | 282 |
  | Publication | Applied Animal Behaviour Science |
  | DOI | 10.1016/j.applanim.2024.106456 |
  | Date Added | 11/07/2025, 14:08:22 |
  | Modified | 11/07/2025, 14:08:22 |

  ### Notes:

  - Cited by: 0
- ## Serum Oxytocin in Cows Is Positively Correlated with Caregiver Interactions in the Impossible Task Paradigm

  |  |  |
  | --- | --- |
  | Item Type | Journal Article |
  | Author | Biagio D’aniello |
  | Author | Vincenzo Mastellone |
  | Author | Claudia Pinelli |
  | Author | Anna Scandurra |
  | Author | Nadia Musco |
  | Author | Raffaella Tudisco |
  | Author | Maria Elena Pero |
  | Author | Federico Infascelli |
  | Author | Alfredo Di Lucrezia |
  | Author | Pietro Lombardi |
  | Date | 2022 |
  | URL | https://www.scopus.com/inward/record.uri?eid=2-s2.0-85123119655&doi=10.3390%2fani12030276&partnerID=40&md5=b089463c7426bd436d469e11171743c5 |
  | Extra | Type: Article |
  | Volume | 12 |
  | Publication | Animals |
  | DOI | 10.3390/ani12030276 |
  | Issue | 3 |
  | Date Added | 20/06/2025, 09:37:48 |
  | Modified | 20/06/2025, 09:37:48 |

  ### Notes:

  - Cited by: 6
- ## Serum Oxytocin in Cows Is Positively Correlated with Caregiver Interactions in the Impossible Task Paradigm

  |  |  |
  | --- | --- |
  | Item Type | Journal Article |
  | Author | Biagio D’aniello |
  | Author | Vincenzo Mastellone |
  | Author | Claudia Pinelli |
  | Author | Anna Scandurra |
  | Author | Nadia Musco |
  | Author | Raffaella Tudisco |
  | Author | Maria Elena Pero |
  | Author | Federico Infascelli |
  | Author | Alfredo Di Lucrezia |
  | Author | Pietro Lombardi |
  | Date | 2022 |
  | URL | https://www.scopus.com/inward/record.uri?eid=2-s2.0-85123119655&doi=10.3390%2fani12030276&partnerID=40&md5=b089463c7426bd436d469e11171743c5 |
  | Extra | Type: Article |
  | Volume | 12 |
  | Publication | Animals |
  | DOI | 10.3390/ani12030276 |
  | Issue | 3 |
  | Date Added | 11/07/2025, 14:08:23 |
  | Modified | 11/07/2025, 14:08:23 |

  ### Notes:

  - Cited by: 7
- ## Cortisol levels of shelter dogs in animal assisted interventions in a prison: An exploratory study

  |  |  |
  | --- | --- |
  | Item Type | Journal Article |
  | Author | Danila D’angelo |
  | Author | Serenella D’ingeo |
  | Author | Francesca Ciani |
  | Author | Michele Visone |
  | Author | Luigi Sacchettino |
  | Author | Luigi Avallone |
  | Author | Angelo Quaranta |
  | Date | 2021 |
  | URL | https://www.scopus.com/inward/record.uri?eid=2-s2.0-85099911592&doi=10.3390%2fani11020345&partnerID=40&md5=5177e8dd95b070515bb9b08ff9a3b402 |
  | Extra | Type: Article |
  | Volume | 11 |
  | Pages | 1 – 11 |
  | Publication | Animals |
  | DOI | 10.3390/ani11020345 |
  | Issue | 2 |
  | Date Added | 11/07/2025, 14:08:22 |
  | Modified | 11/07/2025, 14:08:22 |

  ### Notes:

  - Cited by: 26; All Open Access, Gold Open Access, Green Open Access
- ## Human-lamb bonding: Oxytocin, cortisol and behavioural responses of lambs to human contacts and social separation

  |  |  |
  | --- | --- |
  | Item Type | Journal Article |
  | Author | Marjorie Coulon |
  | Author | Raymond Nowak |
  | Author | Stéphane Andanson |
  | Author | Christine Ravel |
  | Author | Pierre Guy Marnet |
  | Author | Alain Boissy |
  | Author | Xavier Boivin |
  | Date | 2013 |
  | URL | https://www.scopus.com/inward/record.uri?eid=2-s2.0-84875051578&doi=10.1016%2fj.psyneuen.2012.07.008&partnerID=40&md5=8e529f9359782ec59114ebf291a8252e |
  | Extra | Type: Article |
  | Volume | 38 |
  | Pages | 499 – 508 |
  | Publication | Psychoneuroendocrinology |
  | DOI | 10.1016/j.psyneuen.2012.07.008 |
  | Issue | 4 |
  | Date Added | 20/06/2025, 09:37:48 |
  | Modified | 20/06/2025, 09:37:48 |

  ### Notes:

  - Cited by: 40
- ## Human-lamb bonding: Oxytocin, cortisol and behavioural responses of lambs to human contacts and social separation

  |  |  |
  | --- | --- |
  | Item Type | Journal Article |
  | Author | Marjorie Coulon |
  | Author | Raymond Nowak |
  | Author | Stéphane Andanson |
  | Author | Christine Ravel |
  | Author | Pierre Guy Marnet |
  | Author | Alain Boissy |
  | Author | Xavier Boivin |
  | Date | 2013 |
  | URL | https://www.scopus.com/inward/record.uri?eid=2-s2.0-84875051578&doi=10.1016%2fj.psyneuen.2012.07.008&partnerID=40&md5=8e529f9359782ec59114ebf291a8252e |
  | Extra | Type: Article |
  | Volume | 38 |
  | Pages | 499 – 508 |
  | Publication | Psychoneuroendocrinology |
  | DOI | 10.1016/j.psyneuen.2012.07.008 |
  | Issue | 4 |
  | Date Added | 11/07/2025, 14:08:23 |
  | Modified | 11/07/2025, 14:08:23 |

  ### Notes:

  - Cited by: 40
- ## Associations between oxytocin receptor gene polymorphisms, empathy towards animals and implicit associations towards animals

  |  |  |
  | --- | --- |
  | Item Type | Journal Article |
  | Author | Melanie Connor |
  | Author | Alistair B. Lawrence |
  | Author | Sarah M. Brown |
  | Date | 2018 |
  | URL | https://www.scopus.com/inward/record.uri?eid=2-s2.0-85052755994&doi=10.3390%2fani8080140&partnerID=40&md5=031534230c1e89df6591fbd0ff61c1c8 |
  | Extra | Type: Article |
  | Volume | 8 |
  | Publication | Animals |
  | DOI | 10.3390/ani8080140 |
  | Issue | 8 |
  | Date Added | 20/06/2025, 09:37:48 |
  | Modified | 20/06/2025, 09:37:48 |

  ### Notes:

  - Cited by: 9; All Open Access, Gold Open Access, Green Open Access
- ## Do Workplace Fish Tanks Influence Employee Wellbeing and Cognitive Performance? An Embedded Mixed-Methods Study

  |  |  |
  | --- | --- |
  | Item Type | Journal Article |
  | Author | Heather Clements |
  | Author | Stephanie Valentin |
  | Author | Nicholas Jenkins |
  | Author | Jean Rankin |
  | Author | Nancy R. Gee |
  | Author | Donna Snellgrove |
  | Author | Katherine A. Sloman |
  | Date | 2024 |
  | URL | https://www.scopus.com/inward/record.uri?eid=2-s2.0-85183833012&doi=10.1080%2f08927936.2024.2303227&partnerID=40&md5=f04db22e11141a5ff78db77b88a39bae |
  | Extra | Type: Article |
  | Volume | 37 |
  | Pages | 459 – 478 |
  | Publication | Anthrozoos |
  | DOI | 10.1080/08927936.2024.2303227 |
  | Issue | 3 |
  | Date Added | 19/06/2025, 15:40:12 |
  | Modified | 19/06/2025, 15:40:12 |

  ### Notes:

  - Cited by: 2
- ## The effects of owner-cat interaction on oxytocin secretion in pet cats with different attachment styles

  |  |  |
  | --- | --- |
  | Item Type | Journal Article |
  | Author | Hao Chang |
  | Author | Jie Zhang |
  | Author | Haitao Huang |
  | Author | Edgar O. Aviles-Rosa |
  | Author | Huiwen Huang |
  | Author | Yan Guo |
  | Author | Zaili Xiao |
  | Author | Qingshen Liu |
  | Author | Baichuan Deng |
  | Author | Lingna Zhang |
  | Date | 2025 |
  | URL | https://www.scopus.com/inward/record.uri?eid=2-s2.0-85215252826&doi=10.1016%2fj.applanim.2025.106524&partnerID=40&md5=e3c9c66d44039deae14de80ec18ed5d3 |
  | Extra | Type: Article |
  | Volume | 283 |
  | Publication | Applied Animal Behaviour Science |
  | DOI | 10.1016/j.applanim.2025.106524 |
  | Date Added | 20/06/2025, 09:37:47 |
  | Modified | 20/06/2025, 09:37:47 |

  ### Notes:

  - Cited by: 0
- ## Investigating the relationship between human-animal interactions, reactivity, stress response and reproductive performance in Nellore heifers

  |  |  |
  | --- | --- |
  | Item Type | Journal Article |
  | Author | Maria C. Ceballos |
  | Author | Aline C. Sant'Anna |
  | Author | Karen Camille R. Góis |
  | Author | Antonio S. Ferraudo |
  | Author | Joao A. Negrao |
  | Author | Mateus J.R. Paranhos da Costa |
  | Date | 2018 |
  | URL | https://www.scopus.com/inward/record.uri?eid=2-s2.0-85054359172&doi=10.1016%2fj.livsci.2018.08.001&partnerID=40&md5=82e93c038e5e1e1a81dae9c2488e523e |
  | Extra | Type: Article |
  | Volume | 217 |
  | Pages | 65 – 75 |
  | Publication | Livestock Science |
  | DOI | 10.1016/j.livsci.2018.08.001 |
  | Date Added | 11/07/2025, 14:08:23 |
  | Modified | 11/07/2025, 14:08:23 |

  ### Notes:

  - Cited by: 19
- ## Buffalo behavioural response to machine milking in early lactation; [Risposta comportamentale della bufala durante la mungitura meccanica nelle prime fasi della lattazione]

  |  |  |
  | --- | --- |
  | Item Type | Journal Article |
  | Author | Roberta Cavallina |
  | Author | Cristina Roncoroni |
  | Author | Maria Concetta Campagna |
  | Author | Michela Minero |
  | Author | Elisabetta Canali |
  | Date | 2008 |
  | URL | https://www.scopus.com/inward/record.uri?eid=2-s2.0-84994469164&partnerID=40&md5=7cd9996205bc343b98094500c346ae4c |
  | Extra | Type: Article |
  | Volume | 7 |
  | Pages | 131 – 140 |
  | Publication | Italian Journal of Animal Science |
  | Issue | 3 |
  | Date Added | 20/06/2025, 09:37:48 |
  | Modified | 20/06/2025, 09:37:48 |

  ### Notes:

  - Cited by: 29
- ## Relationship between different livestock managements and stress response in dairy ewes

  |  |  |
  | --- | --- |
  | Item Type | Journal Article |
  | Author | Vincenzo Carcangiu |
  | Author | Francesca Arfuso |
  | Author | Sebastiano Luridiana |
  | Author | Claudia Giannetto |
  | Author | Maria Rizzo |
  | Author | Pier Paolo Bini |
  | Author | Giuseppe Piccione |
  | Date | 2018 |
  | URL | https://www.scopus.com/inward/record.uri?eid=2-s2.0-85040913809&doi=10.5194%2faab-61-37-2018&partnerID=40&md5=b98e79616520c2981a3d51b4712a077e |
  | Extra | Type: Article |
  | Volume | 61 |
  | Pages | 37 – 41 |
  | Publication | Archives Animal Breeding |
  | DOI | 10.5194/aab-61-37-2018 |
  | Issue | 1 |
  | Date Added | 11/07/2025, 14:08:23 |
  | Modified | 11/07/2025, 14:08:23 |

  ### Notes:

  - Cited by: 9; All Open Access, Gold Open Access, Green Open Access
- ## Animal assisted therapy (AAT) program as a useful adjunct to conventional psychosocial rehabilitation for patients with schizophrenia: Results of a small-scale randomized controlled trial

  |  |  |
  | --- | --- |
  | Item Type | Journal Article |
  | Author | Paula Calvo |
  | Author | Joan R. Fortuny |
  | Author | Sergio Guzmán |
  | Author | Cristina Macías |
  | Author | Jonathan Bowen |
  | Author | María L. García |
  | Author | Olivia Orejas |
  | Author | Ferran Molins |
  | Author | Asta Tvarijonaviciute |
  | Author | José J. Cerón |
  | Author | Antoni Bulbena |
  | Author | Jaume Fatjó |
  | Date | 2016 |
  | URL | https://www.scopus.com/inward/record.uri?eid=2-s2.0-84974829681&doi=10.3389%2ffpsyg.2016.00631&partnerID=40&md5=b04ea470f7f840ce133c80bfcb7c3dae |
  | Extra | Type: Article |
  | Volume | 7 |
  | Publication | Frontiers in Psychology |
  | DOI | 10.3389/fpsyg.2016.00631 |
  | Issue | MAY |
  | Date Added | 11/07/2025, 14:08:22 |
  | Modified | 11/07/2025, 14:08:22 |

  ### Notes:

  - Cited by: 44; All Open Access, Gold Open Access, Green Open Access
- ## Affective Implications of Human–Animal Relationship on Pig Welfare: Integrating Non-Linear Heart Rate Variability Measures

  |  |  |
  | --- | --- |
  | Item Type | Journal Article |
  | Author | Javiera Calderón-Amor |
  | Author | Belén Zuleta |
  | Author | Maria Camila Ceballos |
  | Author | Daniel Cartes |
  | Author | Christopher J. Byrd |
  | Author | Benjamin Lecorps |
  | Author | Rocío Palomo |
  | Author | Sergio A. Guzmán-Pino |
  | Author | Daniela Siel |
  | Author | Daniela Luna |
  | Date | 2024 |
  | URL | https://www.scopus.com/inward/record.uri?eid=2-s2.0-85200733875&doi=10.3390%2fani14152217&partnerID=40&md5=d7d0b177a9780d095edcb1916d778d79 |
  | Extra | Type: Article |
  | Volume | 14 |
  | Publication | Animals |
  | DOI | 10.3390/ani14152217 |
  | Issue | 15 |
  | Date Added | 19/06/2025, 15:40:13 |
  | Modified | 19/06/2025, 15:40:13 |

  ### Notes:

  - Cited by: 2; All Open Access, Gold Open Access, Green Open Access
- ## Early life adversity in dogs produces altered physiological and behavioral responses during a social stress-buffering paradigm

  |  |  |
  | --- | --- |
  | Item Type | Journal Article |
  | Author | Alicia P. Buttner |
  | Author | Samantha L. Awalt |
  | Author | Rosemary Strasser |
  | Date | 2023 |
  | URL | https://www.scopus.com/inward/record.uri?eid=2-s2.0-85159681936&doi=10.1002%2fjeab.856&partnerID=40&md5=cfba92d9251113907883c473cd80739f |
  | Extra | Type: Article |
  | Volume | 120 |
  | Pages | 6 – 20 |
  | Publication | Journal of the Experimental Analysis of Behavior |
  | DOI | 10.1002/jeab.856 |
  | Issue | 1 |
  | Date Added | 11/07/2025, 14:08:22 |
  | Modified | 11/07/2025, 14:08:22 |

  ### Notes:

  - Cited by: 10; All Open Access, Hybrid Gold Open Access
- ## Extreme life histories are associated with altered social behavior and cortisol levels in shelter dogs

  |  |  |
  | --- | --- |
  | Item Type | Journal Article |
  | Author | Alicia Phillips Buttner |
  | Author | Rosemary Strasser |
  | Date | 2022 |
  | URL | https://www.scopus.com/inward/record.uri?eid=2-s2.0-85133781460&doi=10.1016%2fj.applanim.2022.105693&partnerID=40&md5=f044f708a0f87cc9bd564effe5c87724 |
  | Extra | Type: Article |
  | Volume | 256 |
  | Publication | Applied Animal Behaviour Science |
  | DOI | 10.1016/j.applanim.2022.105693 |
  | Date Added | 11/07/2025, 14:08:22 |
  | Modified | 11/07/2025, 14:08:22 |

  ### Notes:

  - Cited by: 5
- ## Biopsychosocial Factors and Cognitive Function in Cat Ownership and Attachment in Community-dwelling Older Adults

  |  |  |
  | --- | --- |
  | Item Type | Journal Article |
  | Author | Sandra M. Branson |
  | Author | Lisa Boss |
  | Author | Nikhil S. Padhye |
  | Author | Nancy R. Gee |
  | Author | Thea T. Trötscher |
  | Date | 2019 |
  | URL | https://www.scopus.com/inward/record.uri?eid=2-s2.0-85063226178&doi=10.1080%2f08927936.2019.1569908&partnerID=40&md5=13b5b7eb3d49b559821e0a58b35544e9 |
  | Extra | Type: Article |
  | Volume | 32 |
  | Pages | 267 – 282 |
  | Publication | Anthrozoos |
  | DOI | 10.1080/08927936.2019.1569908 |
  | Issue | 2 |
  | Date Added | 11/07/2025, 14:08:22 |
  | Modified | 11/07/2025, 14:08:22 |

  ### Notes:

  - Cited by: 20; All Open Access, Hybrid Gold Open Access
- ## Oxytocin levels and self-reported anxiety during interactions between humans and cows

  |  |  |
  | --- | --- |
  | Item Type | Journal Article |
  | Author | Bente Berget |
  | Author | Judit Vas |
  | Author | Gunn Pedersen |
  | Author | Kerstin Uvnäs-Moberg |
  | Author | Ruth C. Newberry |
  | Date | 2023 |
  | URL | https://www.scopus.com/inward/record.uri?eid=2-s2.0-85172998589&doi=10.3389%2ffpsyg.2023.1252463&partnerID=40&md5=0387a3edae6bfee2a88514bc6a6690a6 |
  | Extra | Type: Article |
  | Volume | 14 |
  | Publication | Frontiers in Psychology |
  | DOI | 10.3389/fpsyg.2023.1252463 |
  | Date Added | 20/06/2025, 09:37:47 |
  | Modified | 20/06/2025, 09:37:47 |

  ### Notes:

  - Cited by: 2; All Open Access, Gold Open Access, Green Open Access
- ## Heart rate variability and saliva cortisol assessment in shelter dog: Human-animal interaction effects

  |  |  |
  | --- | --- |
  | Item Type | Journal Article |
  | Author | Luciana Bergamasco |
  | Author | Maria Cristina Osella |
  | Author | Paolo Savarino |
  | Author | Giuseppe Larosa |
  | Author | Laura Ozella |
  | Author | Monica Manassero |
  | Author | Paola Badino |
  | Author | Rosangela Odore |
  | Author | Raffaella Barbero |
  | Author | Giovanni Re |
  | Date | 2010 |
  | URL | https://www.scopus.com/inward/record.uri?eid=2-s2.0-77953326843&doi=10.1016%2fj.applanim.2010.03.002&partnerID=40&md5=7a8106161443cacd7561da7200e3d09e |
  | Extra | Type: Article |
  | Volume | 125 |
  | Pages | 56 – 68 |
  | Publication | Applied Animal Behaviour Science |
  | DOI | 10.1016/j.applanim.2010.03.002 |
  | Issue | 1-2 |
  | Date Added | 19/06/2025, 15:40:13 |
  | Modified | 19/06/2025, 15:40:13 |

  ### Notes:

  - Cited by: 142; All Open Access, Green Open Access
- ## Heart rate variability and saliva cortisol assessment in shelter dog: Human-animal interaction effects

  |  |  |
  | --- | --- |
  | Item Type | Journal Article |
  | Author | Luciana Bergamasco |
  | Author | Maria Cristina Osella |
  | Author | Paolo Savarino |
  | Author | Giuseppe Larosa |
  | Author | Laura Ozella |
  | Author | Monica Manassero |
  | Author | Paola Badino |
  | Author | Rosangela Odore |
  | Author | Raffaella Barbero |
  | Author | Giovanni Re |
  | Date | 2010 |
  | URL | https://www.scopus.com/inward/record.uri?eid=2-s2.0-77953326843&doi=10.1016%2fj.applanim.2010.03.002&partnerID=40&md5=7a8106161443cacd7561da7200e3d09e |
  | Extra | Type: Article |
  | Volume | 125 |
  | Pages | 56 – 68 |
  | Publication | Applied Animal Behaviour Science |
  | DOI | 10.1016/j.applanim.2010.03.002 |
  | Issue | 1-2 |
  | Date Added | 11/07/2025, 14:08:23 |
  | Modified | 11/07/2025, 14:08:23 |

  ### Notes:

  - Cited by: 143; All Open Access, Green Open Access
- ## Exploratory study of Stress-Buffering response patterns from interaction with a therapy dog

  |  |  |
  | --- | --- |
  | Item Type | Journal Article |
  | Author | Sandra B. Barker |
  | Author | Janet S. Knisely |
  | Author | Nancy L. McCain |
  | Author | Christine M. Schubert |
  | Author | Anand K. Pandurangi |
  | Date | 2010 |
  | URL | https://www.scopus.com/inward/record.uri?eid=2-s2.0-75949130357&doi=10.2752%2f175303710X12627079939341&partnerID=40&md5=72de4c7a3b4e2d16d6d39dab5280585d |
  | Extra | Type: Article |
  | Volume | 23 |
  | Pages | 79 – 91 |
  | Publication | Anthrozoos |
  | DOI | 10.2752/175303710X12627079939341 |
  | Issue | 1 |
  | Date Added | 19/06/2025, 15:40:12 |
  | Modified | 19/06/2025, 15:40:12 |

  ### Notes:

  - Cited by: 83
- ## Exploratory study of Stress-Buffering response patterns from interaction with a therapy dog

  |  |  |
  | --- | --- |
  | Item Type | Journal Article |
  | Author | Sandra B. Barker |
  | Author | Janet S. Knisely |
  | Author | Nancy L. McCain |
  | Author | Christine M. Schubert |
  | Author | Anand K. Pandurangi |
  | Date | 2010 |
  | URL | https://www.scopus.com/inward/record.uri?eid=2-s2.0-75949130357&doi=10.2752%2f175303710X12627079939341&partnerID=40&md5=72de4c7a3b4e2d16d6d39dab5280585d |
  | Extra | Type: Article |
  | Volume | 23 |
  | Pages | 79 – 91 |
  | Publication | Anthrozoos |
  | DOI | 10.2752/175303710X12627079939341 |
  | Issue | 1 |
  | Date Added | 11/07/2025, 14:08:22 |
  | Modified | 11/07/2025, 14:08:22 |

  ### Notes:

  - Cited by: 84
- ## Exploratory study of Stress-Buffering response patterns from interaction with a therapy dog

  |  |  |
  | --- | --- |
  | Item Type | Journal Article |
  | Author | Sandra B. Barker |
  | Author | Janet S. Knisely |
  | Author | Nancy L. McCain |
  | Author | Christine M. Schubert |
  | Author | Anand K. Pandurangi |
  | Date | 2010 |
  | URL | https://www.scopus.com/inward/record.uri?eid=2-s2.0-75949130357&doi=10.2752%2f175303710X12627079939341&partnerID=40&md5=72de4c7a3b4e2d16d6d39dab5280585d |
  | Extra | Number: 1 Type: Article |
  | Volume | 23 |
  | Pages | 79 – 91 |
  | Publication | Anthrozoos |
  | DOI | 10.2752/175303710X12627079939341 |
  | Issue | 1 |
  | Date Added | 28/07/2025, 12:48:52 |
  | Modified | 28/07/2025, 12:48:52 |

  ### Notes:

  - Cited by: 83
- ## Hemodynamic (fNIRS) and EEG (N200) correlates of emotional inter-species interactions modulated by visual and auditory stimulation

  |  |  |
  | --- | --- |
  | Item Type | Journal Article |
  | Author | M. Balconi |
  | Author | M.E. Vanutelli |
  | Date | 2016 |
  | Archive | Scopus |
  | URL | https://www.scopus.com/inward/record.uri?eid=2-s2.0-84961182321&doi=10.1038%2fsrep23083&partnerID=40&md5=be679cddfa73b9ae4b725cc257b9b2f0 |
  | Volume | 6 |
  | Publication | Scientific Reports |
  | DOI | 10.1038/srep23083 |
  | Date Added | 11/07/2025, 11:22:51 |
  | Modified | 11/07/2025, 11:22:51 |

  ### Notes:

  - Export Date: 11 July 2025; Cited By: 34
- ## Hemodynamic (fNIRS) and EEG (N200) correlates of emotional inter-species interactions modulated by visual and auditory stimulation

  |  |  |
  | --- | --- |
  | Item Type | Journal Article |
  | Author | M. Balconi |
  | Author | M.E. Vanutelli |
  | Date | 2016 |
  | Archive | Scopus |
  | URL | https://www.scopus.com/inward/record.uri?eid=2-s2.0-84961182321&doi=10.1038%2fsrep23083&partnerID=40&md5=be679cddfa73b9ae4b725cc257b9b2f0 |
  | Volume | 6 |
  | Publication | Scientific Reports |
  | DOI | 10.1038/srep23083 |
  | Date Added | 11/07/2025, 11:24:11 |
  | Modified | 11/07/2025, 11:24:11 |

  ### Notes:

  - Export Date: 11 July 2025; Cited By: 34
- ## Judgement bias in goats (Capra hircus): Investigating the effects of human grooming

  |  |  |
  | --- | --- |
  | Item Type | Journal Article |
  | Author | Luigi Baciadonna |
  | Author | Christian Nawroth |
  | Author | Alan G. McElligott |
  | Date | 2016 |
  | URL | https://www.scopus.com/inward/record.uri?eid=2-s2.0-84992036363&doi=10.7717%2fpeerj.2485&partnerID=40&md5=fdf89522a5356d99e2dd47bd642d7dc2 |
  | Extra | Type: Article |
  | Volume | 2016 |
  | Publication | PeerJ |
  | DOI | 10.7717/peerj.2485 |
  | Issue | 10 |
  | Date Added | 19/06/2025, 15:40:12 |
  | Modified | 19/06/2025, 15:40:12 |

  ### Notes:

  - Cited by: 19; All Open Access, Gold Open Access, Green Open Access
- ## Infrared thermography as a tool in welfare assessment of equines handled in paddock and stall; [Termografia infravermelha como ferramenta na avaliação do bem-estar de equinos manejados soltos ou estabulados]

  |  |  |
  | --- | --- |
  | Item Type | Journal Article |
  | Author | D.R.O.E. Azevedo |
  | Author | B.S.L. Dallago |
  | Author | A.C. Silva |
  | Author | J.R.M. Pereira |
  | Author | R.F. Ferreira |
  | Author | T.S. Borges |
  | Author | T.B. Bisol |
  | Author | L.V. Sobrinho |
  | Author | T.O. Fernandes |
  | Author | F.E.M. Bernal |
  | Date | 2024 |
  | URL | https://www.scopus.com/inward/record.uri?eid=2-s2.0-85198053495&doi=10.1590%2f1678-4162-13037&partnerID=40&md5=c5ea81505ecf888863a274dccf07577c |
  | Extra | Type: Article |
  | Volume | 76 |
  | Publication | Arquivo Brasileiro de Medicina Veterinaria e Zootecnia |
  | DOI | 10.1590/1678-4162-13037 |
  | Issue | 4 |
  | Date Added | 11/07/2025, 14:08:22 |
  | Modified | 11/07/2025, 14:08:22 |

  ### Notes:

  - Cited by: 0
- ## Impact of Human–Animal Interactions on Psychological and Physiological Factors Associated With Veterinary School Students and Donkeys

  |  |  |
  | --- | --- |
  | Item Type | Journal Article |
  | Author | Elpida Artemiou |
  | Author | Pippa Hutchison |
  | Author | Marcus Machado |
  | Author | Daria Ellis |
  | Author | Jennifer Bradtke |
  | Author | Mary Mauldin Pereira |
  | Author | Julia Carter |
  | Author | Don Bergfelt |
  | Date | 2021 |
  | URL | https://www.scopus.com/inward/record.uri?eid=2-s2.0-85114425689&doi=10.3389%2ffvets.2021.701302&partnerID=40&md5=34afa24b552f98b2a23c53e6c9cbc589 |
  | Extra | Type: Article |
  | Volume | 8 |
  | Publication | Frontiers in Veterinary Science |
  | DOI | 10.3389/fvets.2021.701302 |
  | Date Added | 11/07/2025, 14:08:22 |
  | Modified | 11/07/2025, 14:08:22 |

  ### Notes:

  - Cited by: 3
- ## The Health Benefits of Visiting a Zoo, Park, and Aquarium for Older Japanese

  |  |  |
  | --- | --- |
  | Item Type | Journal Article |
  | Author | Junko Akiyama |
  | Author | Taketo Sakagami |
  | Author | Hidehiko Uchiyama |
  | Author | Mitsuaki Ohta |
  | Date | 2021 |
  | URL | https://www.scopus.com/inward/record.uri?eid=2-s2.0-85102961446&doi=10.1080%2f08927936.2021.1898211&partnerID=40&md5=3f5e866611daca5977ceca800d7100cb |
  | Extra | Type: Article |
  | Volume | 34 |
  | Pages | 463 – 473 |
  | Publication | Anthrozoos |
  | DOI | 10.1080/08927936.2021.1898211 |
  | Issue | 3 |
  | Date Added | 20/06/2025, 09:37:48 |
  | Modified | 20/06/2025, 09:37:48 |

  ### Notes:

  - Cited by: 3
- ## The Health Benefits of Visiting a Zoo, Park, and Aquarium for Older Japanese

  |  |  |
  | --- | --- |
  | Item Type | Journal Article |
  | Author | Junko Akiyama |
  | Author | Taketo Sakagami |
  | Author | Hidehiko Uchiyama |
  | Author | Mitsuaki Ohta |
  | Date | 2021 |
  | URL | https://www.scopus.com/inward/record.uri?eid=2-s2.0-85102961446&doi=10.1080%2f08927936.2021.1898211&partnerID=40&md5=3f5e866611daca5977ceca800d7100cb |
  | Extra | Type: Article |
  | Volume | 34 |
  | Pages | 463 – 473 |
  | Publication | Anthrozoos |
  | DOI | 10.1080/08927936.2021.1898211 |
  | Issue | 3 |
  | Date Added | 11/07/2025, 14:08:23 |
  | Modified | 11/07/2025, 14:08:23 |

  ### Notes:

  - Cited by: 3
