# Supplementary material for: Evidence of Physiological Comodulation During Human–Animal Interaction: A Systematic Review
Source: Ann N Y Acad Sci. 2026 Jun 4;1560(1):e70299. doi: 10.1111/nyas.70299 (PMC13238372; doi:10.1111/nyas.70299)
Supplement: Supplementary file 4 — Supplementary Materials: Supp4‐Data‐Dictionary.pdf [file NYAS-1560-0-s009.pdf]

# Evidence of Physiological Co-Modulation During Human-Animal Interaction: A Systematic Review - Data Dictionary (S4)

---

This Data Dictionary contains detailed definitions for each Data Collection Form item.

**Items:**

- **Name:** citation of the paper in APA format
- **Year:** publication year
- **Animal species involved:** list of animal species (other than human)
- **Number of human and animal participants:** sample sizes for both humans and animals.
- **Non healthy (human) / non wild-type (animal) participants:** description of non-healthy human participants and/or genetically mutated animals present in the study
- **Interaction Context:** describes the interaction paradigm as:
  - *Animal-Assisted Therapy/Intervention (AAT/AAI):* studies have been labelled as animal-assisted therapy (AAT, i.e. a planned, structured therapeutic intervention with specific clinical or functional goals (e.g., reduce depression, improve motor skills, enhance socio-emotional functioning)) only when authors explicitly used this term. All other animal-based programs meeting inclusion criteria are described under the broader animal-assisted intervention umbrella (AAI, i. e. Umbrella term for all goal-oriented, structured interventions that intentionally include animals in health, education, or human services for therapeutic benefit). Although all eligible programs were encompassed within the broader AAI framework, the AAT label was retained when explicitly used by authors to preserve intervention-specific detail and avoid unnecessary loss of granularity [1,2].
  - *companionship:* interactions involving humans and animals characterized by social presence and mutual engagement, without therapeutic intent, formal intervention, or performance-oriented activities in sports or working contexts.
  - *competitive sport:* organized sports involving formal competition e.g. dog agility competition.
  - *non-competitive sport:* sports or sports-related activities performed without involving formal competition e.g. race horse training.
  - *working animals:* work activities involving dogs e.g. SAR (Search And Rescue) activities.
- **Behavioural measures:** list of behavioural measures and questionnaires
- **Measured physiological parameter(s):** describes the sampled physiological feature(s) as: heart rate, cortisol, oxytocin, EEG (electroencephalogram), breathing rate, testosterone, CgA (chromogranin A), ACTH (adrenocorticotropic hormone), b-endorphin (beta-endorphin), epinephrine (adrenaline), norepinephrine (noradrenaline), T3 (triiodothyronine), T4 (thyroxine), Na (sodium), K (potassium), CREA (creatinine), urea, TP (total protein), ALB (albumin), Mg (magnesium), AP (alkaline phosphatase), CK (creatine kinase), ALT (alanine aminotransferase), AST (aspartate aminotransferase), WBC (white blood cells), RBC (red blood cells), HGB (haemoglobin), HCT (haematocrit), MCV (mean corpuscular volume), MCH (mean corpuscular haemoglobin), MCHC (mean corpuscular haemoglobin concentration), PLT (platelets), LYM (lymphocytes), GRA (granulocytes).

- **Sampling:** brief description of the instrumentation used for sampling, includes temporal characteristics for hormonal sampling.
- **Index used:** measure used to perform synchrony analysis
- **Analysis description:** brief description of the statistical techniques used to assess synchronization.
- **Data analysis category:** classifies the analysis category as: Time-Series Coupling, Structural Equation Modelling, Generalized Linear Mixed Models, Generalized Linear Models, Linear Regressions, Cross-correlation, Time series correlation, Discrete-Time Correlation Analysis.
- **Outcome:** co-modulation outcomes, classifies as: significant, partial (e.g., limited to specific conditions or subgroups), or absent; based on the authors' reporting of their statistical analyses.

## References


[1] A. H. Fine, S. J. Weaver, The Human–Animal Bond and Animal-Assisted Intervention, Oxford University Press, 2018. doi:10.1093/med/9780198725916.003.0028.

[2] F. L. Green, M. L. Dahlman, A. Lomness, J.-T. Binfet, For the love of acronyms: An analysis of terminology and acronyms used in AAI research 2013–2023, Human-Animal Interactions (Jul. 2024). doi:10.1079/hai.2024.0024.
