# Supplementary material for: Evidence of Physiological Comodulation During Human–Animal Interaction: A Systematic Review
Source: Ann N Y Acad Sci. 2026 Jun 4;1560(1):e70299. doi: 10.1111/nyas.70299 (PMC13238372; doi:10.1111/nyas.70299)
Supplement: Supplementary file 6 — Supplementary Materials: Supp6‐Molecular‐Sampling‐Table.pdf [file NYAS-1560-0-s006.pdf]

Evidence of Physiological Co-Modulation During Human-Animal Interaction: A Systematic Review - Molecular Sampling Table (S9)

Molecular parameter	Salivary sampling	Plasmatic sampling	urinary sampling	Hair sampling
Cortisol (n = 17)	13 studies	2 studies		2 studies
Oxytocin (n = 4)	2 studies [ab]	2 studies [a]	2 studies [b]	
Testosterone (n = 2)	2 studies			
Chromogranin A (n = 1)	1 study			
Other hormonal, biochemical and haematological parameters (n = 1)		1 study		

Molecular Sampling Table:
The table illustrates the different sampling categories of molecular physiological parameters.

[a] Salivary (only humans) and plasmatic (only animals) samples collected in the same study.

[b] Salivary and urinary samples collected in the same study.
