# Supplementary material for: Evidence of Physiological Comodulation During Human–Animal Interaction: A Systematic Review
Source: Ann N Y Acad Sci. 2026 Jun 4;1560(1):e70299. doi: 10.1111/nyas.70299 (PMC13238372; doi:10.1111/nyas.70299)
Supplement: Supplementary file 7 — Supplementary Materials: Supp7‐Summary‐of‐Individual‐Studies.pdf [file NYAS-1560-0-s008.pdf]

Evidence of Physiological Co-Modulation During Human-Animal Interaction: A Systematic Review - Summary of Individual Studies (S12)

Contents

1 Time-Series Coupling Analysis 1
- 1.0.1 works that found evidence of coupling 1
- 1.0.2 works that found evidence of coupling for subsets of data 3
- 1.0.3 works that didn't find evidence of coupling 4

2 Regression Analysis 4
- 2.1 Structural Equation Modelling (SEM) 4
  - 2.1.1 works that found evidence of association 4
- 2.2 Generalized Linear Mixed Models (GLMMs) 5
  - 2.2.1 works that found evidence of association 5
  - 2.2.2 works that found evidence of associations for subsets of data 5
  - 2.2.3 works that didn't find evidence of associations 6
- 2.3 Generalized Linear Models (GLMs) 6
  - 2.3.1 works that found evidence of association 6
- 2.4 Linear Regressions 6
  - 2.4.1 works that found evidence of association 6
  - 2.4.2 works that found evidence of association for subsets of data 7

3 Correlation analysis 8
- 3.1 Cross-Correlation analysis 8
  - 3.1.1 works that found evidence of cross-correlation 8
- 3.2 Time Series Correlation analysis 8
  - 3.2.1 works that found evidence of correlation 8
- 3.3 Discrete-Time Correlation Analysis 9
  - 3.3.1 works that found evidence of correlation 9
  - 3.3.2 works that found evidence of correlation for subsets of data 10
  - 3.3.3 works that didn't find evidence of correlation 14

1. Time-Series Coupling Analysis

1.0.1. works that found evidence of coupling

- **Quantitative Heartbeat Coupling Measures in Human-Horse Interaction**[1]

  Eleven subjects and one mare were involved in a study examining cardiovascular coupling across three interaction phases: resting, visual/olfactory contact, and grooming. ECG signals were recorded at 250 Hz and processed to extract HRV time series using filtering, R-peak detection (Pan-Tompkins for humans, a custom method for horses), and spline interpolation at 10 Hz.
  Three coupling metrics—Magnitude Squared Coherence (MSC), Mean Phase Coherence (MPC), and Dynamic Time Warping (DTW)—were computed to assess signal similarity. MSC evaluates frequency-domain correlation, MPC measures phase synchronization via Hilbert transform, and DTW quantifies temporal alignment. Statistical comparisons using Wilcoxon signed rank tests with Benjamini-Hochberg correction revealed MSC could distinguish all phases, while MPC and DTW showed significant differences only between grooming and the other phases.
  A Nearest Mean Classifier applied to one-minute feature windows achieved over 70% accuracy in phase classification, suggesting the feasibility of quantifying human-horse interaction dynamics through cardiovascular coupling.
  The risk of bias ranking for this paper is "High": despite rigorous methodology and analysis, the use of a single horse and small sample size suggests a high overall risk of bias.

- **The Role of Nonlinear Coupling in Human-Horse Interaction: a Preliminary Study**[2]

  Eleven participants and one mare took part in a study investigating nonlinear cardiovascular coupling during three phases of human-horse interaction: resting (P1), visual/olfactory contact (P2), and grooming (P3). ECG signals were recorded using textile-based systems at 250 Hz and processed to extract HRV

time series through filtering, R-peak detection (Pan-Tompkins for humans, a custom method for horses), and spline interpolation at 10 Hz.
The analysis employed Information Theoretic Learning (ITL) metrics to capture nonlinear dependencies: Cross Information Potential (CIP), Cross-Correntropy (CCORR), and Correntropy Coefficient (CC). These measures were derived using Gaussian kernel functions and Renyi's entropy formulations, allowing the quantification of higher-order statistical relationships between HRV signals.
Statistical comparisons across phases were conducted using Wilcoxon signed rank tests with Benjamini-Hochberg correction. CIP and CCORR significantly distinguished all three phases, while CC showed significant differences only between P1–P2 and P2–P3. These trends suggest a decrease in HRV similarity from P1 to P3, possibly reflecting divergent physiological responses to increasing interaction intensity.
A Support Vector Machine classifier with radial basis kernel was implemented using a Leave-One-Subject-Out cross-validation scheme. The model achieved an overall classification accuracy of 90.9%, indicating strong discriminative power of the nonlinear features. These findings support the use of ITL-based metrics for quantifying and classifying human-horse interaction dynamics.
The risk of bias ranking for this paper is "High": despite strong methodology and analysis, the limited and unbalanced sample (especially the use of a single horse) suggests a high overall risk of bias.

- **A Wearable System for the Evaluation of the Human-Horse Interaction: A Preliminary Study**[3]

Fourteen subjects participated in an experiment consisting of three four-minute phases. In the first phase, the human and the horse were in separate stalls, with the human seated and the horse free to move. In the second phase, the horse joined the human, who remained seated while the horse explored. In the third phase, both stood and engaged in grooming.
ECG signals were filtered using a zero-phase Butterworth band-pass filter (0.5–40 Hz). R-peaks were detected via energy-signal thresholding, and RR intervals were interpolated with a cubic spline at 10 Hz to account for heart rate variability. Dynamic Time Warping (DTW) was used to assess similarity between human and horse HRV across phases, with statistical comparisons made using the Wilcoxon signed rank test and Benjamini-Hochberg correction. Phase classification was performed using a support vector machine with a radial kernel.
All phase comparisons showed statistically significant differences. In 12 of 14 subjects, DTW was lower or equal in P1 compared to P2, and higher in P3 than in the other phases. For horses, the P2–P3 transition was more significant than P1–P2, while for humans, P1–P3 showed the greatest difference. These findings suggest that P3 elicited the strongest response in both species, and DTW effectively distinguished all phases.
The risk of bias ranking for this paper is "Moderate to High": despite strong methodology and signal validation, the use of a single horse and small sample size in the interaction study suggests a moderate overall risk of bias.

- **Unveiling directional physiological coupling in human-horse interactions**[4]

Simultaneous ECG recordings were collected from 20 horses interacting with 20 human pairs, each consisting of one familiar and one unfamiliar individual. The protocol included three sessions: baseline (S1) with horse and human separated, exploration (S2) with the horse free to approach a stationary human, and grooming (S3) on both sides of the horse.
Time-varying bivariate autoregressive models (TV-BVAR) were applied to HRV signals using 60-second windows and 1-second steps. Directed Coherence (DC) was computed across 0–0.2 Hz to assess causal interactions. Statistical significance was evaluated using bootstrap and causal shuffling with cluster correction. Spearman correlations between DC in LF and HF bands and behavioural data were tested with permutation-based p-values and Bonferroni correction.
Significant heartbeat synchronization emerged in S2, absent in S1. In familiar pairs, synchronization occurred earlier and bidirectionally, especially at the end of S2. In unfamiliar pairs, it was predominantly from horse to human. Synchronization involved both LF and HF bands.
In S3, familiarity modulated directionality. In familiar pairs, during left-side grooming (S3L), interactions were unidirectional from horse to human (LF band only). In unfamiliar pairs, S3L showed bidirectional interactions, while right-side grooming (S3R) was dominated by horse-to-human directionality. Human-to-horse synchronizations occurred only in the LF band, whereas horse-to-human synchronizations spanned both LF and HF bands.
The risk of bias ranking for this paper is "Moderate": despite methodological rigour, the moderate sample size, potential unmeasured confounders, and repeated use of individuals suggest a moderate overall risk of bias.

- **Disrupted Human–Dog Interbrain Neural Coupling in Autism-Associated Shank3 Mutant**

Dogs[5]

This study simultaneously recorded non-invasive wireless EEG from laboratory beagles and unfamiliar humans during social interactions across five consecutive days, under three conditions: no interaction in separate rooms, co-presence without interaction, and full interaction. EEG signals were preprocessed using Matlab and FieldTrip, including low-pass filtering (250 Hz), artifact removal, wavelet transformation, and neural network-based cleaning. EEG power in the 4–30 Hz range (theta, alpha, beta) was analysed. Interbrain coupling was assessed via Pearson correlation and Generalized Partial Directed Coherence (GPDC), with statistical testing through Friedman, Mann–Whitney U, two-way ANOVA, and Holm–Šídák correction.
Interbrain correlation in frontal and parietal regions significantly increased over the five days, with a positive linear relationship between interaction duration and correlation. Logistic growth curve analysis in three additional dogs showed a plateau in interbrain coupling by day seven. Mutual gaze or petting alone induced significantly lower interbrain correlation than full interaction, and their combined effect was less than full interaction.
GPDC analysis revealed directional coupling, increasing from day one to five, particularly from human to dog in frontal and parietal regions. This suggests human-initiated behaviours (e.g., gaze, petting) drive neural synchronization.
In dogs with Shank3 mutations (ASD model), no significant increase in interbrain correlation or relationship with interaction time was observed. However, LSD treatment restored interbrain coupling, with significant increases in frontal and parietal GPDC values from human to dog over five days. This study demonstrates directed interbrain neural coupling in human–dog interactions, its disruption by autism-associated mutations, and its restoration via LSD-induced reopening of the social learning critical period.
The risk of bias ranking for this paper is "Moderate": the study is methodologically rigorous and innovative, but limitations in sample diversity and lack of pre-registration suggest a moderate overall risk of bias.

- **The relationship of early life adversity and physiological synchrony within the therapeutic triad in horse-assisted therapy**[6]

The study hypothesized that stronger HRV synchronization between the riding therapist and therapy horse would enhance HRV synchronization in the other dyads of the therapeutic triad. Heart rates of the participant, therapist, and horse were recorded using Polar chest belts (Polar 2022) connected to iPhones/iPads, with data collected via the HRV Logger App (Altini 2013). The interaction protocol included five phases: contact, grooming, riding, lying on the horse, and farewell.
Due to incomplete therapist data, only 42 triads were included in the statistical analysis (9 controls, 33 patients). RR-interval data were processed in R using customized lab scripts. Cross-wavelet power analysis was applied, categorizing HRV into four frequency bands: upper/lower high frequency (UHF/LHF) and upper/lower low frequency (ULF/LLF). Data were z-transformed for normalization.
A reference model predicting HF-HRV synchronization between horse and participant included random intercepts/slopes, covariates (BMI, baseline HRV, mental health), and design variables (minute, interval, frequency, interactions). Model fit significantly improved with the addition of HF-HRV synchronization between horse and therapist ($\chi^2 = 1770.83$, $p < .01$). Findings suggest that physiological coherence in the triad is not a linear cause-effect process but an emergent property of mutual influence among all dyads.
The risk of bias ranking for this paper is "Moderate": this is a methodologically rigorous study with innovative analysis and thoughtful interpretation. Some limitations in sample diversity and unmeasured confounders suggest a moderate overall risk of bias.

1.0.2. works that found evidence of coupling for subsets of data

- Physiological and Behavioral Benefits for People and Horses during Guided Interactions at an Assisted Living Residence[7]

Twenty-four human participants aged 55 or older took part in four guided sessions of stroking one of three horses for 10 minutes, over a period of 4 to 6 weeks. HRV was recorded before, during, and after each session. Human baseline HRV was measured for 5 minutes using a Zephyr Bioharness BT placed snugly around the chest, with an Inner Balance HRV monitor on the earlobe as backup. Horse baseline HRV was recorded using a Polar Equine RS800CX belt around the girth.
HRV was analysed using SDRR (standard deviation of interbeat intervals) and RMSSD (root mean square of successive differences), reflecting overall variability and parasympathetic activity, respectively. Frequency domain analysis divided HRV into VLF (0.003–0.04 Hz), LF (0.05–0.15 Hz), and HF (0.15–0.4 Hz) bands. Kubios HRV Standard software was used to extract parameters and compare peak oscillatory frequencies between humans and horses.

Results showed that 8 of 13 morning participants and 8 of 11 evening participants exhibited HRV frequency coupling with their horses in at least two sessions, with matches up to three or four decimal places. Some participants showed coupling with more than one horse. Additionally, 3 morning and 3 evening participants showed coupling in only one session. Most coupling frequencies were in the VLF range, and their presence was independent of whether the average %VLF increased or decreased during interaction compared to baseline.
The risk of bias ranking for this paper is "Moderate to High": the study is well-conducted and transparent, but the lack of a control group and potential selection bias limit causal inference, as well as the strong limitations of the peak-frequency analysis. The repeated measures design and robust physiological methods strengthen internal validity.

- Effects of Equine Interaction on Mutual Autonomic Nervous System Responses and Interoception in a Learning Program for Older Adults[8]

Subjects (humans n = 24, horses n = 3) participated in mindful grooming during which they slowed their breathing and brushed a horse while noticing sensations in their body and watching the horse's reactions. The subject's and horse's HRV were recorded simultaneously before, during, and after mindful grooming. For control, the same subjects performed mindful grooming with a plush simulation horse.
Heart Rate Variability has been measured as SDRR (standard deviation of the interbeat interval) and RMSSD (root mean square of successive differences). The frequency of the HRV oscillations is divided into: very low frequency (VLF: 0.003–0.04 Hz), low frequency (LF: 0.05–0.15 Hz), and high frequency (HF: 0.15–0.4 Hz). Analysis of the data was performed using the Kubios HRV Standard software program to obtain HRV parameters (the detrending/smoothing function was off, keeping the VLF rhythms in the data).
To determine whether there was coupling of HRV frequency peaks between horse-human pairs, Fast Fourier Transform analysis was performed by the Kubios software to access the HRV frequency domain. Using this analysis, the peak HRV oscillatory frequencies for the horse and human recordings could be compared.
In 10 out of 24 human/horse pairs, matching HRV oscillation frequencies occurred during the interaction that were not present during pre-interaction measures. These data support the concept that oscillations of HRV may become synchronous between horse and human during mindful grooming.
The risk of bias ranking for this paper is "Moderate to High": the paper lacks a rigorous peak-frequency analysis, a clearly defined sample selection process, and full randomization, despite employing strong measurement practices.

1.0.3. works that didn't find evidence of coupling

- **Exploring the Dynamics of Canine-Assisted Interactions: A Wearable Approach to Understanding Interspecies Well-Being[9]**

Holder et al. (2024) developed a custom made system to collect simultaneous and continuous physiological data from both the human and the dog in the context of canine-assisted interaction (CAI).
The study included a sample of 8 adolescents/young adults and 4 canines, the interactions were unstructured (talking to, touching, grooming, toy play, treat giving, commands, etc.).
The preprocessing included; check and removal of outliers, bandpass Butterworth filtering, normalization, and temporal alignment. ECG waveform R peaks has been performed to extract the interbeat interval (IBI), that has been used as the basis for all the other ECG metrics (standard deviation of the IBI of normal sinus beats (SDNN), root mean square of successive differences between normal heartbeats (RMSSD), quotient of SDNN and RMSSD. All the measures have been calculated with two different approaches: average metric by 10 s epoch and rolling window average by epoch; the latter, performed by using a 60 s window shifting with a 0.1 s pace (10 Hz), has been used for correlational analyses of synchrony only.
Pearson's correlation was calculated for the three key ECG metrics (e.g., HR, SDNN, and RMSSD) of human and canine ECG data. Authors further tested dyadic signals via the dynamic time-warping (DTW) methodology, to track these key time series' data alignment in general and when assuming temporal asynchrony.
Both correlation and dynamic time warping results show no clear patterning across subjects.
The risk of bias ranking for this paper is "Moderate": despite strong methodology and transparency, the small sample size and lack of randomization suggest a moderate overall risk of bias.

2. Regression Analysis

2.1. Structural Equation Modelling (SEM)

2.1.1. works that found evidence of association

- **Evidence for a synchronization of hormonal states between humans and dogs during competition[10].**

Buttner et al. (2015) examined hormonal changes in human-dog dyads during agility competitions. Fifty-eight handler-dog dyads participated. Saliva samples were collected from handlers at three time points: baseline (upon enrolment), pre-competition (5 minutes post-run), and post-competition (20 minutes post-run). Dogs' saliva was sampled at baseline and 20 minutes post-run. Human samples were assayed for cortisol and testosterone; dog samples for cortisol. Hormone distributions were positively skewed and normalized via square root transformation. Percent change in cortisol ($\Delta CORT$) and testosterone (ΔT) was calculated. Multivariate normality was confirmed; no outliers were detected. Structural equation modelling (SEM) was used to assess associations among hormonal changes, observed affiliative and punitive behaviours, and handlers' self-rated performance. Handlers' ($\Delta CORT$) significantly predicted dogs' ($\Delta CORT$). Handlers' behaviour did not mediate this association. Performance ratings predicted affiliative and punitive behaviours but not ($\Delta CORT$). An alternative model, positing dogs' ($\Delta CORT$) as the predictor of handlers' ratings and subsequent hormonal and behavioural changes, showed inferior fit (χ^2 difference test, p $\leq$ .05).
Repeated measures ANOVA, Pearson correlations, and t-tests were used to examine hormone levels and behavioural variables. Model fit indices included χ^2, CFI, RMSEA, and SRMR.
The authors report that "handlers' and their dogs' changes in cortisol levels are generally mirrored, and this effect was not accounted for by the humans' behaviour towards the dog that we observed." Additionally, "male handlers' dogs experienced greater elevations in cortisol levels following competitions relative to their baseline levels, whereas dogs belonging to female handlers showed no changes.
The risk of bias ranking for this paper is "Moderate": the study is methodologically sound and innovative, but the naturalistic setting and lack of behavioural stress measures in dogs introduce moderate risk of bias.

2.2. Generalized Linear Mixed Models (GLMMs)

2.2.1. works that found evidence of association

- **Dog's breath rhythm was drawn into owner's breath rhythm[11]**

This study examined whether changes in human breathing influence canine respiration. Six dog-human dyads were monitored using a high-sensitivity magnetic sensor for dogs and a thermocouple for humans. The protocol included three conditions: a baseline rest (rest1), a stress-inducing video (video condition), and a paced breathing task (metronome condition). Each condition was followed by a rest period, and dogs were instructed to lie down while humans sat quietly.
Respiratory peaks were detected using MATLAB's findpeaks() function and manually verified to exclude artifacts. The key variable was the delta-breathing-interval (delta-BI), defined as the difference in mean breathing interval between rest1 and each subsequent condition.
A generalized linear mixed model (GLMM) was used to analyze the dog's delta-BI, with human delta-BI and condition as fixed effects, and dyad identity as a random effect. The analysis revealed that both human breathing changes and the metronome condition significantly affected dog breathing. Specifically, dogs tended to breathe more as their human partners increased their breathing rate.
These findings suggest a physiological coupling in respiration between dogs and humans, even in the absence of direct interaction, highlighting the sensitivity of dogs to human autonomic changes.
The risk of bias ranking for this paper is "Moderate": the study is methodologically sound and innovative, but limited by small sample size, lack of pre-registration, and untested generalizability beyond owner–dog pairs.

2.2.2. works that found evidence of associations for subsets of data

- **Effects of human-animal interaction on salivary and urinary oxytocin in children and dogs[12]**

Children (N=55) completed three laboratory sessions—Pet Dog (PD), Unfamiliar Dog (UD) and nonsocial Control (CT)—with simultaneous endocrine sampling; dogs were evaluated in PD (each child's pet dog) and in UD (a single unfamiliar dog). Salivary oxytocin was assayed at baseline (T1) and +15 min (T2), while urinary oxytocin was assayed at T1 and +50 min (T3; urine only).
Analyses used Bayesian Gaussian models with identity link, four MCMC chains (2000 iterations each), weakly regularizing priors for fixed effects and Student-*t* priors for random intercepts; outcome/predictor variables were standardized, salivary oxytocin was log-transformed, and urine measures were processed with specific-gravity standardization (including an optimized *z*-coefficient) and, where appropriate, Yeo–Johnson transformation; plate effects were modeled via random intercepts.
Baseline salivary and urinary oxytocin concentrations were uncorrelated in both species, and changes from baseline in saliva versus urine showed no strong association. At the dyadic level, salivary oxytocin responses of children and dogs showed a *moderate positive* association in UD (with minor uncertainty), a *weak negative* association in PD (with considerable

uncertainty), and for urinary measures minimal evidence of association in UD but a *moderate* correlation in PD.
Limitations include reliance on a single unfamiliar dog, absence of an acclimation period, and limited demographic diversity; inter-assay variability was addressed by modeling plate as a random effect. Overall, we judge risk of bias as **Moderate**.

2.2.3. works that didn't find evidence of associations

- **Does stress run through the leash? An examination of stress transmission between owners and dogs during a walk**[13]

Harvie et al. (2021) investigated physiological stress transmission in 68 owner–dog dyads during a 15-minute walk conducted at the participants' residences. Dyads were randomly assigned to either a Control or Experimental group. Owners in the Experimental group were informed that their walking performance would be evaluated by dog obedience experts, while Control group participants were told the walk would be recorded for archival purposes. Salivary cortisol samples were collected from owners and dogs before and after the walk. Heart rate (HR) was recorded continuously during the walk using Polar® monitors. Cortisol difference scores were calculated by subtracting baseline from post-walk values. HR difference scores were computed across three timepoints: Baseline to first 60 seconds (Timepoint One), first 60 seconds to midpoint (Timepoint Two), and midpoint to final 60 seconds (Timepoint Three). Linear regressions assessed whether owner HR influenced dog HR and whether owner cortisol influenced dog cortisol.
The owner's HR did not significantly influence their dog's HR for either the Control or the Experimental groups during Timepoint One, Timepoint Two or Timepoint Three. As there were no significant differences between groups, the authors ran an additional linear regression on the HR data, which included all dyads.
The analysis showed that the owner's HR did not significantly influence their dog's HR during Timepoint One, Timepoint Two or Timepoint Three.
They performed two separate linear regressions on the salivary cortisol difference scores between owners and their dogs for both the Control and Experimental groups. The analysis did not show a significant linear relationship for the dog/owner dyads in the Experimental group or the Control group. As there were no significant differences between groups, they ran an additional linear regression on the salivary cortisol data, which included all dyads.
The analysis showed that owner's cortisol levels have a marginally significant influence dog's cortisol levels.
The risk of bias ranking for this paper is "Moderate": the study was well-designed and executed, but limitations in sample representativeness and potential unmeasured confounders suggest a moderate overall risk of bias.

2.3. Generalized Linear Models (GLMs)

2.3.1. works that found evidence of association

- **Long-term stress levels are synchronized in dogs and their owners**[14]

This study investigated long-term stress synchronization in 58 dog-human dyads by analysing hair cortisol concentrations (HCC) from summer and winter periods. Dogs' physical activity was continuously monitored for one week using a cloud-based collar, and owners reported daily routines to control for activity-related cortisol variation.
Statistical analyses were conducted using generalized linear models (GLMs). Summer dog HCC was modelled with normal identity; winter HCC with gamma distribution and log link. Models included human HCC (covariate), dog sex, breed, and lifestyle (fixed factors), and their two-way interactions. Additional models assessed the influence of owner and dog personality traits.
Human HCC significantly predicted dog HCC in both seasons. In summer, interactions were found between human HCC and dog lifestyle, and between human HCC and dog sex. While human HCC affected both pet and competing dogs, and both male and female dogs, the effect was stronger in competing and female dogs.
Findings indicate that long-term cortisol levels are synchronized between dogs and their owners, independent of physical activity or training intensity. Owner personality traits were significantly associated with dog HCC, suggesting dogs mirror their owners' stress levels rather than vice versa.
The risk of bias ranking for this paper is "Moderate": this is a well-controlled, statistically robust study with thoughtful interpretation. Minor limitations in sample diversity and potential unmeasured confounders suggest a moderate overall risk of bias.
It is worth noting that the regression analysis presented in this work reflects associations between long-term hormonal parameters across all dog–owner dyads, rather than tracking changes within each individual dyad over time.

2.4. Linear Regressions

2.4.1. works that found evidence of association

- **Empathy or Apathy? Investigating the influence of owner stress on canine stress in a novel environment**[15]

This study investigated whether changes in owner stress levels, measured via heart rate (HR) and heart rate variability (HRV), influenced their dogs' physiological responses. Owners were divided into two groups—one exposed to stress-inducing stimuli, the other to stress-reducing stimuli—validated through changes in HR, HRV, and STAIS-5 scores. Owners were instructed not to interact with their dogs during the 15-minute experiment to avoid behavioural influence.
Both human and canine ECGs were recorded using Polar H10 monitors. RMSSD was chosen as the HRV metric for its sensitivity to short-term R-R interval variation and was verified manually. HR data were segmented into pre-intervention, intervention, and post-intervention phases, and mean HR values were calculated for each.
A two-way mixed ANOVA assessed changes in mean HR across time and groups. Dog HR data violated sphericity assumptions, so Greenhouse-Geisser correction was applied. Bonferroni-adjusted post hoc tests followed. A linear regression was performed to evaluate the predictive relationship between changes in owner and dog HR, calculated as the difference between pre-intervention and intervention mean HR. The regression revealed that changes in owner HR significantly predicted changes in dog HR. Since the intervention group did not significantly affect HR outcomes, the regression was applied to the entire sample without group stratification. These findings suggest that dogs physiologically respond to changes in their owners' stress levels, even in the absence of direct interaction.
The risk of bias ranking for this paper is "Moderate": the study is well-designed and methodologically sound, but limitations in sample size, generalizability, and potential confounders suggest a moderate overall risk of bias.

- **Behavioral and emotional comodulation during dog–owner interaction measured by heart rate variability and activity**[16]

The study involved 25 healthy pet dogs and their owners. Each dyad participated in a 53-minute protocol conducted in a controlled room, with researchers observing remotely via video and audio. Task instructions were displayed on a computer screen throughout the session.
Electrocardiogram (ECG) signals were recorded simultaneously from dogs and owners using Bittium Faros 180™ devices, synchronized via eMotion Faros Manager 2.3.0. Human ECG data were corrected for artifacts using Kubios Scientific 4.0.3. Due to species-specific differences in heart rate variability (HRV), particularly respiratory sinus arrhythmia in dogs, a beta version of Kubios software was developed in collaboration with the company to enable canine HRV analysis.
Valid ECG segments (1–3 min per task) were selected, and RMSSD (root mean square of successive differences) was extracted for each task. Overall RMSSD values were calculated by averaging across tasks. Shapiro–Wilk tests indicated non-normal distributions in portions of RMSSD and activity data; Spearman correlations were used to assess associations between dog and owner RMSSD and activity. To test for pseudocorrelation, RMSSD values were re-analysed using randomized dog–(non)owner dyads. Multivariate linear regression (stepwise method) was applied to predict owner RMSSD, with inclusion and exclusion criteria set at $p < 0.05$ and $p > 0.10$, respectively. Independent variables included demographic, behavioural, and psychological measures.
The authors report: "The overall RMSSD of dogs and their owners across all tasks correlated statistically significantly." Additionally, "the overall RMSSD of dogs and the overall RMSSD of randomized owners were not correlated statistically significantly," and "part of the overall HRV correlation in the dog–owner dyads is accounted by emotional co-modulation between dogs and owners characteristics for attachment relationships."
The risk of bias ranking for this paper is "Moderate": despite strong methodology and analysis, the limited sample diversity and potential selection bias suggest a moderate overall risk of bias.

2.4.2. *works that found evidence of association for subsets of data*

- Interspecies hormonal interactions between man and the domestic dog[17]

This study investigated the relationship between male handlers' testosterone (T) levels and their behaviour toward dogs following competition, and whether this behaviour influenced dogs' cortisol responses. Of 184 handler/dog teams, 83 male handlers were analysed; disqualified teams were excluded. Salivary samples were collected from both handlers and dogs 90 minutes before and 20 minutes after competition results were posted.
Change scores for T and cortisol were computed using regression residuals, controlling for pre-competition levels—conceptually equivalent to ANCOVA. Among losing teams, higher pre-competition T in handlers significantly predicted greater increases in dogs' cortisol, accounting for 50.9% of the variance. Additionally, greater decreases in handlers' T post-competition were associated with larger increases in dogs' cortisol, though this explained less variance than pre-

competition T. Among winners, neither pre-competition T nor T change predicted dogs' cortisol changes.
Poisson regression showed no significant difference in prior win rates between winners and losers, ruling out performance history as an explanation for T differences.
These findings suggest that handlers' hormonal states, particularly following loss, influence dogs' stress responses, while such effects are absent in winning contexts.
The risk of bias ranking for this paper is "Moderate": despite strong methodology and analysis, the lack of randomization and limited control over contextual variables in a naturalistic setting suggest a moderate overall risk of bias.

3. Correlation analysis

3.1. Cross-Correlation analysis

3.1.1. works that found evidence of cross-correlation

- **Equine-assisted therapy and its impact on cortisol levels of children and horses: a pilot study and meta-analysis [18]**

The phases of this single case design are as follows. The first baseline (A) phase involved the collection of three samples of cortisol a day, one day a week, for three weeks (horses and children were tested on the same day). In the B phase, children came to the location for the therapeutic riding programme, and their cortisol levels were measured every 20–30 minutes. The C phase was the intervention: participants groomed a horse for approximately 15 minutes, rode the horse for approximately 30 minutes and then groomed the horse for another 15 minutes. Cortisol was collected during this phase every 20–30 minutes. The second B phase and the concluding A phase of the study were analogous to the first ones.
A meta-analytic technique was used to estimate the mean cross-correlation between child/horse cortisol levels during the riding phase. In addition, graphs of moving averages (four point) or 'smoothing out' of the data are used to compare both horse and child cortisol levels across the 12 days of the intervention. A lag-zero cross-correlation was calculated for each child/horse pair. Then, the weighted mean cross-correlation for the four child/horse pairs was calculated, along with the standard error of this sample mean cross-correlation, 'pre-whitening' of the time series had been previously performed using the autocorrelation and partial autocorrelation functions.
Overall the weighted mean and the Z-statistic which calculates effect size indicated that there was a mild to moderate symmetry collectively between the child–horse pairs. This could be random, the result of the short period of time the pairs spent together, or a product of child–horse matching.
The risk of bias ranking for this paper is "High": this is a valuable exploratory study with innovative methodology, but the small sample size, lack of randomization, and limited control of confounding variables suggest high overall risk of bias.

3.2. Time Series Correlation analysis

3.2.1. works that found evidence of correlation

- **Emotional Contagion From Humans to Dogs Is Facilitated by Duration of Ownership [19]**

Katayama et al. (2019) investigated emotional comodulation between dogs and owners by analysing heart rate variability (HRV) during a stress-inducing task. Thirty-four owner–dog dyads participated in both Control and Stress (Trier Social Stress Test, TSST) conditions. ECG signals were recorded at 1000 Hz using TSND121 and TS-EMG01 devices. HRV parameters—standard deviation of normal-to-normal R-R intervals (SDNN), root mean square of successive differences (RMSSD), and mean R-R intervals (RRI)—were calculated in 15-second time bins. Due to motion artifacts, 20 dyads were excluded from further analysis.
To test whether shared environment and bonding influenced emotional contagion, Spearman's rank correlation coefficients were calculated between dogs' and owners' HRV parameters. Variables included duration of ownership, daily time spent together, dog's age, sex, body weight, gaze behaviour, and physical contact. Due to multicollinearity ($r = 0.991$), only duration of ownership was retained in statistical models. Generalized linear models (GLM) were used to assess predictors of HRV correlation coefficients.
The authors report: "Correlation coefficients of RRI in the stress condition tended to be higher than that of the control condition." They further state: "There was a significant positive correlations found between the duration of the ownership with the correlation coefficients of RMSSD in the stress condition." GLM revealed that "5 out of 6 correlation coefficients of HRV parameters were positively influenced by the duration of the ownership." Additionally, "female dogs showed stronger correlation coefficients as compared to male," and "owner's gaze time at the dog and dog's gaze time at the owner had negative influences on the correlation coefficients of HRV parameters."
The risk of bias ranking for this paper is "Moderate": despite strong methodology and analysis, the small final sample and high exclusion rate suggest a moderate overall risk of bias.

- **Psychophysiological effects of equine-facilitated psychotherapy on Veterans with PTSD and their horse partners**[20]

The study involved 16 Veterans diagnosed with PTSD, each paired with one of eight therapy mares, participating in Equine-Assisted Therapy sessions. Baseline assessments were conducted prior to each session. Salivary cortisol and oxytocin levels were measured in both humans and horses, with additional plasmatic oxytocin analysis for horses, using ELISA kits. Heart rate variability (HRV) was recorded via Polar monitors and analysed using Kubios HRV Standard software before interaction, during grooming, and post-session.
Five-minute HRV segments were simultaneously collected from both species. Kubios applied medium artifact correction. HRV analysis included time-domain metrics (mean RR, mean HR, RMSSD, SDNN), frequency-domain metrics (LF/HF ratio: LF 0.01–0.07 Hz, HF 0.07–0.60 Hz), and non-linear indices (SNS and PNS Index). Bland–Altman plots assessed agreement in physiological changes between human–horse dyads, focusing on SNS/PNS ratios. Results indicated consistent agreement in autonomic balance shifts during grooming and walking phases.
The risk of bias ranking for this paper is "Moderate": despite strong methodology and transparent reporting, the lack of a control group and potential confounding variables suggest a moderate overall risk of bias.

- **Dogs showed lower parasympathetic activity during mutual gazing while owners did not**[21]

Nagasawa et al. (2023) investigated autonomic responses and emotional co-modulation between dogs and humans during the Strange Situation Test (SST). Twenty-two owner–dog dyads participated. Eleven unfamiliar female participants served as "strangers." ECG signals were recorded using Faros 360° sensors at 1000 Hz. HRV parameters—mean R-R intervals (RRI), standard deviation of normal-to-normal intervals (SDNN), and root mean square of successive differences (rMSSD)—were calculated in 15-second bins.
Correlation coefficients of HRV parameters were computed per episode to assess emotional synchronization. Median HRV values were also calculated. For gaze analysis, ECG data were clipped 6 seconds before and after dog gaze events lasting $\leq$ 6 seconds and averaged every 2 seconds. Wilcoxon signed-rank tests compared dog behaviours and HRV parameters between owner (OW) and stranger (ST) episodes. Linear mixed models (LMMs) assessed the influence of dog behaviour, ownership duration, dog sex, and episode type on HRV and correlation coefficients.
The authors report: "The correlation coefficient of mean RRI was higher in OW episodes." However, "authors did not find any significant effect of dog behaviour on the correlation coefficient HRVs." From gaze episodes, 19 data points from OW episodes of 13 pairs and 19 from ST episodes of 15 pairs were obtained. LMM analysis showed "no changes in HRV were found in both dogs and human participants during the 12 s." Nonetheless, "mean RRI, SDNN, and rMSSD were significantly lower when they gazed at their owners than when they gazed at strangers." Additionally, "SDNN and rMSSD were significantly lower in owners than in strangers."
The risk of bias ranking for this paper is "High": the study is well-conducted for exploratory research, but limitations exist across all domains, especially in sampling, measurement and correlation assessment.

3.3. Discrete-Time Correlation Analysis

3.3.1. works that found evidence of correlation

- **Oxytocin-gaze positive loop and the coevolution of human-dog bonds**[22]

The authors tested the hypothesis that an oxytocin-mediated positive loop exists between humans and dogs, facilitated by mutual gaze. In experiment 1, 30-minute interactions were conducted between owners and dogs, and between owners and hand-raised wolves. Urinary oxytocin concentrations were measured before and after the interaction. Dog behaviours —– gaze at owner, talking, and touching —– were recorded. Dogs were assigned to long-gaze or short-gaze groups.
Dogs in the long-gaze group exhibited the highest duration of gaze toward owners. Wolves rarely showed mutual gaze. After interaction, only owners in the long-gaze group showed a significant increase in urinary oxytocin concentrations and the highest change ratio. The oxytocin change ratio in owners was significantly correlated with that of dogs, the duration of dog-to-owner gaze, and dog-touching. Multiple linear regression analysis revealed that "only the duration of dog-to-owner gaze significantly explained the oxytocin change ratio in owners." Dog-touching showed a trend toward significance. Similarly, dogs in the long-gaze group showed a significantly higher oxytocin change ratio than those in the short-gaze group. Dog-to-owner gaze also significantly explained the oxytocin change ratio in dogs.
In wolves, "the duration of wolf-to-owner gaze did not correlate with the oxytocin change ratio in either owners or wolves." In experiment 2, intranasal oxytocin administration increased gazing behaviour in female dogs and urinary oxytocin in their owners.

No significant oxytocin change was observed in dogs under limited interaction. The authors conclude that "dog-to-owner gaze as a form of social communications probably evolved during domestication."
The risk of bias ranking for this paper is "Moderate": the study is innovative and well-conducted, but limitations in confounding control, sample size, and lack of pre-registration suggest a moderate overall risk of bias.

3.3.2. *works that found evidence of correlation for subsets of data*

- **Associations between the Psychological Characteristics of the Human–Dog Relationship and Oxytocin and Cortisol Levels**[23]

Ten blood samples were collected from each dog owner and her dog during a 60-minute interaction. Blood samples (0, 1, 3, 5, 15, 30, and 60 minutes) were analysed for oxytocin and cortisol by Enzyme Immuno Assay (EIA) and mean values of oxytocin and cortisol were calculated in both owners and dogs. The MDORS scores obtained were correlated with basal and mean oxytocin and cortisol levels. The correlation analysis revealed some relationships between the scores of items in the MDORS that reflect the character of the dog–owner-relationship and the owners' hormone levels.
The non-parametric Spearman rank coefficient was used to calculate correlations because the number of participants in the present study was relatively small and a normal distribution of data could not be assumed. Correlations were calculated between MDORS scores and the mean and basal levels of the owners' and the dogs' oxytocin and cortisol levels. Correlations were also calculated between the owners' and the dogs' oxytocin and cortisol levels at each individual time point of the interaction experiment. The statistical significance level was set at $p < 0.05$.
The owners' mean oxytocin levels at each time point and their mean oxytocin level between 0 and 60 minutes correlated positively, some of which were statistically significant, with the mean oxytocin level of the dogs at 60 minutes.
The higher the owners' oxytocin levels were during the interaction experiment, the higher the dogs' oxytocin levels were at the end of the experiment (for rs- and p-values, see Table 5).
In addition, there was a significant positive correlation between the dogs' and the owners' oxytocin levels at 15 minutes. No significant correlations were found between the owners' and the dogs' cortisol levels.
The risk of bias ranking for this paper is "Moderate to High": while the physiological methods were sound, the small, homogeneous sample and correlational design limit the strength and generalizability of the conclusions.

- **Emotional reactions of horses and trainers during natural method training**[24]

Janczarek et al. (2013) investigated emotional reactions in horse–trainer dyads during natural method training. Forty three-year-old purebred Arabian horses (20 colts, 20 fillies) were randomly assigned to two female trainers (each trained 20 horses). The study was conducted on the first day of training, aiming to have the horse accept a rider. Five training elements were analysed: deconcentration, concentration, desensitizing, putting on the lungeing surcingle, and saddling.
Heart rate (HR) was continuously recorded every 60 seconds using Polar S810 telemetric devices, synchronized for simultaneous analysis. Only data collected while horses and trainers were motionless were analysed. Variance analysis and Pearson correlations were used to assess the effects of horse sex and trainer identity on HR, and to evaluate emotional responses.
"Significant correlations within three training elements were recorded when analysing the associations between the colt and trainer's HR values. A negative coefficient characterized deconcentration and concentration, while a positive one described the desensitizing. The latter also indicated some positive dependencies between the filly and the trainer's HR levels. The filly group had the only case of statistically significant dependence."
Three significant correlations were found for trainer identity: trainer No 1 showed negative HR correlation during concentration and positive during desensitizing; trainer No 2 showed positive correlation during desensitizing. The authors conclude: "Our results do not fully agree with the assumption that there is a clear interaction between the emotional and physiological status of a horse and a person."
The risk of bias ranking for this paper is "Moderate to High": the study is methodologically sound and transparent, but the small number of trainers and limited control over psychological and environmental variables suggest a moderate overall risk of bias.

- **Salivary Cortisol Levels in Horses and their Riders During Three-Day-Events**[25]

The group of 36 warm-blooded half-bred horses (18 stallions and 18 mares) and their riders (20 men and 16 women), were selected for the study. Saliva samples were collected after each phase of the three-day competition. The cortisol concentration was determined using an immunoassay method.

The following factors were considered: type of competition, horse sex, and rider gender.
For each horse, three saliva samples were collected: 1) 5-7 min after the dressage, on the 1st d of the events, 2) 5-7 min after the cross-country, on the 2nd d of the events, and 3) 5-7 min after the show jumping, on the 3rd d of the events.
The Pearson correlation coefficient was also assessed to compare the analysed data. The statistical significance was accepted at the level of $P \leq 0.05$.
The analysis of the horse-rider interaction suggests an interesting correlation between the male rider and his horse salivary cortisol levels for the dressage, and separately between female rider and her horse for the cross-country. This finding suggests that the human-horse interaction can be influenced by the rider gender and by the type of competition.
The risk of bias ranking for this paper is "Moderate": the study is methodologically sound and contributes valuable data, but limitations in sample diversity and control of confounding variables suggest a moderate overall risk of bias, as well as the use of Pearson correlation in absence of data normality assessment.

- **Exploring Synchronicity in the Heart Rates of Familiar and Unfamiliar Pairs of Horses and Humans Undertaking an In-Hand Task**[26]

  The authors investigated heart rate (HR) synchronicity between horses and humans during an in-hand obstacle course, comparing familiar and unfamiliar pairings. Seventeen horses were each led twice—once by a familiar handler and once by an unfamiliar one—in a cross-over design. HR was recorded every 5 seconds using Polar RS800 monitors, and synchronicity was assessed via Spearman correlations at 16 set points. A significant order effect was found: "higher horse heart rates were seen the first time around the course regardless of whether a familiar or unfamiliar handler was leading". However, "horses' mean heart rates for each course were significantly higher with the unfamiliar handler than with the familiar handler", suggesting greater relaxation with familiar humans. In contrast, "human heart rates were higher when paired with a familiar horse compared with an unfamiliar horse", likely reflecting performance anxiety. Significant HR correlations were found in six of 34 pairings: four familiar and two unfamiliar. The authors note: "Our findings indicate that the relationship between horse and human heart rates during interactions is not straightforward or consistent between horses and humans." They conclude that familiarity affects HR differently in horses and humans, and that HR synchronicity is rare in in-hand tasks, possibly due to limited physical contact compared to ridden work.
  The risk of bias ranking for this paper is "Moderate": the study was well-conducted and transparent, but limitations in sample size, potential confounders, and generalizability suggest a moderate overall risk of bias.

- **Influence of Horse and Rider on Stress during Horse-riding Lesson Program**[27] The present study aims to confirm the influence of a horse-riding lesson program (HRLP) on the stress level of horses and riders by respectively analysing their salivary cortisol concentration. Twenty-four healthy horses and 23 riders participated in this study. The horses were randomly classified into two groups for the horse riding lesson program: Class 1 (for the beginner lesson) and Class 2 (for the intermediate lesson).
  Salivettes cotton wool swabs were used for saliva collection and the saliva analyses were conducted using a two-way analysis of variance for repeated measures with SAS version. "Saliva samples were collected four times: at rest (baseline), before the HRLP (T1), after the HRLP (T2), and 1 hour after the HRLP (T3).
  As for the results, the average salivary cortisol concentration of all horses before HRLP significantly increased compared to the baseline while it decreased after the HRLP. The results of the salivary cortisol concentration of the riders were similar to the horses' results. However, there was no difference during the HRLP between Class 1 and Class 2 in the horse or rider groups. The results suggest that the HRLP did not influence the stress level of the horses or riders.
  The results of the correlation between the salivary cortisol concentration of the horses and riders showed that both (Horses' T1 ; riders' T2) and (Horses' T2 ; riders' T3) were positively related.
  The risk of bias ranking for this paper is "Moderate": the study was well-conducted and transparent, but the limited sample diversity and lack of control for some confounding variables suggest a moderate overall risk of bias.

- Physiological Indicators of Attachment in Domestic Dogs (Canis familiaris) and Their Owners in the Strange Situation Test[28]

  The authors investigated physiological indicators of attachment in 29 owner–dog dyads using a modified Ainsworth's Strange Situation Test (SST). Dogs experienced separation and reunion episodes with their owners and interactions with a stranger. Saliva samples were collected from both dogs and owners before and after the SST to measure cortisol (CORT) and chromogranin A (CgA). Data transformations were applied to correct for skewness: square root for door scratching frequency and log10 for CORT and CgA

concentrations. Means were back-transformed for interpretation.
Mixed GLM ANOVAs were used to assess the influence of owner-reported separation anxiety (SA) on proximity and hormonal measures. Independent samples t-tests and partial correlations were also conducted. Dogs spent more time near owners, initiated more contact, and spent less time near the door compared to episodes with the stranger. Dogs that initiated more contact with owners had lower CgA, while their owners had higher CgA. Dogs and owners spent more time near each other when owner CgA was low, owner CORT was high, and the dog had SA. Dogs with higher CORT spent more time with the stranger.
Dogs' initial CgA levels were correlated with their owner's initial CORT levels, and dog final CORT levels were correlated with their owners' final CORT levels. All dyads were classified as securely attached. The authors conclude that "dogs may respond to owner hormonal state and/or behaviour," and that "individual differences in responses to a behavioural challenge reflect the stress physiology of both dogs and their owners."
The risk of bias ranking for this paper is "Moderate": the study is methodologically rigorous and well-controlled, but limited by sample homogeneity and potential unmeasured confounders, suggesting a moderate overall risk of bias.

- Salivary Cortisol Interactions in Search and Rescue Dogs and Their Handlers [29]

Wojtaś et al. (2020) investigated salivary cortisol interactions between search and rescue (SAR) dogs and their handlers during official field and rubble certification exams. Forty-one SAR teams participated, yielding 164 saliva samples—two from each dog and handler before and after the exam. Cortisol levels in handlers were normally distributed; in dogs, they were not, prompting use of nonparametric tests. Spearman's rank-order correlation and Pearson's r were used to assess relationships between cortisol levels and sex.
The authors report: "The level of cortisol during the examination was found to increase significantly in the SAR dogs. This increase, however, was not as substantial as in the handlers." A significant positive correlation was found between dog and handler cortisol levels before the exam ($R_s = 0.34$, $p = 0.032$), suggesting "the dog senses the stress in the handler." Stronger correlations were observed in female dog–female handler dyads ($r = 0.72$, $p = 0.033$), with a trend in male dyads ($r = 0.81$, $p = 0.096$).
"The correlation in the case of the exam with a negative result is clearly stronger than the correlation of the cortisol level before the exam with a positive result." Dogs whose handlers made mistakes had lower cortisol levels than those whose dogs failed. The authors note: "Analysis of the results of examinations were carried out based on a small size of the sample (only 13 failed exams). Therefore, authors treat this part of the analysis as preliminary research."
The risk of bias ranking for this paper is "Moderate": this is a well-conducted observational study with thoughtful interpretation. Some limitations in sample diversity and control of external stressors suggest a moderate overall risk of bias.

- **Assessing the Relationship Between Emotional States of Dogs and Their Human Handlers, Using Simultaneous Behavioral and Cardiac Measures [30]**

The authors investigated emotional contagion between dogs and unfamiliar human handlers during routine handling in a veterinary clinic setting. Forty adult dog–owner pairs completed three handling sets across two sessions. Prior to handling, human participants engaged in either a mindfulness meditation or a control activity. A mild verbal stressor was introduced before the third handling set to assess its impact on emotional state.
Dogs and humans were fitted with Polar H10 cardiac sensors, and heart rate (HR), RMSSD, and HF (log-transformed) were analysed using Kubios HRV. "For all HRV variables, a within-subject approach (difference from baseline measurement for each participant and session date) was used." Pearson's correlations were run to assess relationships between behavioural stress index (SI) and cardiac parameters.
"No significant correlations (Pearson) were found between the human and canine cardiac activity parameters during session 1; during session 2, canine RMSSD was significantly correlated with human HF." Canine HR decreased and RMSSD increased across handling sets, suggesting reduced stress with increased familiarity. "We did not find consistent support for emotional contagion between the dogs and their handlers in this study, perhaps due to the brief time that the dogs spent with the handlers." The authors conclude: "Even short-term familiarity with a previously-unfamiliar handler when low-stress handling protocols are employed... is associated with reduced stress in dogs in a veterinary setting." They recommend consistent personnel and low-stress techniques to improve welfare, noting individual variation and limitations in detecting emotional contagion.
The risk of bias ranking for this paper is "Moderate": despite strong methodology and transparent reporting, limitations in sample diversity, stressor strength, and individual variation, as well as the use

of Pearson's correlation coefficient, suggest a moderate overall risk of bias.

- **Are Hair Cortisol Levels of Humans, Cats, and Dogs from the Same Household Correlated?** [31]

The authors investigated correlations in hair cortisol levels (HCL) among humans, dogs, and cats living in the same household. The study involved 25 women, 45 dogs, and 55 cats. Cortisol was extracted from hair samples and analysed using ELISA. Due to non-normal distributions (Shapiro–Wilk test), Spearman's rank correlation was used. Differences in HCL based on relationship strength were assessed using Mann–Whitney U and Kruskal–Wallis tests.
"There was no significant correlation between the hair cortisol level of the owner and dog or the owner and the cat and between dogs and cats living together." However, "significant positive correlations between human and animal hair cortisol levels (HCL)" were found in specific subgroups: cats never kissed, dogs groomed weekly, dogs whose owners confide in them, and dogs seen as a reason to wake up. In contrast, "significant negative correlations" were found between owners and male cats, cats seen as a reason to wake up, and dogs whose owners deny being observed by them.
The authors suggest: "If a human is strongly emotionally connected with a dog, then we observe a different hormonal relationship... than in the case of a human strongly emotionally connected with a cat." They hypothesize that increased attention may be more stressful for cats.
The risk of bias ranking for this paper is "Moderate": this is a carefully conducted exploratory study with appropriate methodology. Some limitations in sample diversity and unmeasured confounders suggest a moderate overall risk of bias.

- **Physiology of human-horse interactions during substance withdrawal within psychotherapy participants** [32]

This study examined the physiological effects of Psychotherapy Incorporating Equine interaction (PIE) on individuals undergoing substance withdrawal, with a focus on salivary cortisol concentrations and heart rate (HR) as biomarkers of stress. Additionally, the study explored potential human-horse physiological coupling by measuring the same parameters in therapy horses. Data were collected from 18 patients and 4 horses across weeks one, two, and four of a residential treatment program. Human HR was recorded every minute using Inspire 2 Fitbits, while horse HR was recorded every second using Polar Equine monitors. Salivary cortisol samples were obtained before and after equine interaction sessions.
Statistical analyses included Shapiro-Wilk tests for normality, revealing nonparametric distributions. Cortisol data were analysed using the Wilcoxon Signed Rank test. Mixed-effects models (SAS MIXED procedure) assessed fixed effects of age, sex, drug classification, treatment, and week, with backward stepwise selection for significant covariates. HR data were log-transformed and analysed using repeated measures. Spearman correlations assessed human-horse physiological synchrony.
Results indicated a tendency toward moderate negative correlation in cortisol changes between humans and horses across treatment. No significant correlations were observed in week one. However, week two showed a strong negative correlation, with human cortisol decreasing and horse cortisol increasing. Week four revealed a similar trend, with both species showing cortisol reductions, though more pronounced in humans. No significant HR correlations were found across any treatment weeks. These findings suggest potential temporal coupling in stress biomarkers during PIE, warranting further investigation into interspecies physiological dynamics in therapeutic contexts.
The risk of bias ranking for this paper is "Moderate": despite strong methodology and transparent reporting, the small sample size, self-selection, and limited control of confounders suggest a moderate overall risk of bias.

- **Heart rate and salivary cortisol as indicators of arousal and synchrony in clients, therapy horses and therapist in equine-assisted therapy** [33]

Naber et al. (2025) investigated physiological synchronization and stress-buffering effects in equine-assisted therapy (EAT) involving one therapist, four therapy horses, and ten female clients with mild (N = 5) to moderate (N = 5) intellectual disability. Heart rate (HR), heart rate variability (HRV: SDNN, RMSSD, SD1), and salivary cortisol were measured before, during, and after standardized therapy and control sessions. Cardiovascular activity was recorded using Polar® V800 and analysed in 5-min segments via Kubios HRV software. Spearman correlations assessed synchronization between horse, client, and therapist.
The authors report: "The results on synchronization showed no significant relation between HRV, cortisol concentration, and HR of the horse and client during the entire standardized EAT session." However, "a significant correlation between the HR of clients and horses when the interaction took place with a familiar horse ($r = 0.38$, $p = 0.007$)" was found, com-

pared to no significant correlation with unfamiliar horses. Similarly, “there was a significant correlation between the HR of the therapist and the client during the experimental condition as well as during the control condition,” and “a significant correlation for HR between the therapist and the horse,” which was stronger with familiar horses.
No significant changes in HRV or cortisol were found across conditions. However, “clients showed a significantly lower HR in the presence of a horse during the challenge phase.” The authors conclude: “relationship intensity is an important factor for the synchronization process,” and suggest future research should explore oxytocin and include unfamiliar therapist-client pairings.
The risk of bias ranking for this paper is "Moderate": despite rigorous methodology and transparent reporting, the small sample size, lack of blinding, and potential environmental confounds suggest a moderate overall risk of bias.

- **The Effect of Victory and Defeat on the Correlations of Stress Parameters Between the Horse and Rider in Kök-Börü Equestrian Teams**[34]

The authors investigated physiological correlations between horses and riders in the traditional equestrian sport Kök-Börü, focusing on the effects of competitive outcomes. Blood samples were collected from 40 horses and 40 riders before and after two matches, and analysed for hormonal (cortisol, ACTH, beta-endorphin, epinephrine, norepinephrine, T3, T4), biochemical, and haematological parameters using species-specific ELISA kits and automated analysers. Statistical analysis included Shapiro-Wilk tests for normality, followed by nonparametric methods (Mann-Whitney U, Wilcoxon) and Spearman correlation analysis.
The authors report that “positive correlations were observed between lymphocyte percentages (LYM%) and MID/MID%, haemoglobin (HGB) and red cell distribution width (RDW-CV), and between mean platelet volume (MPV) and platelet distribution width (PDW%)” before the game in winning teams. Post-game, “positive correlations strengthened, particularly between lymphocytes (LYM) and red blood cells (RBC), haematocrit (HCT%), and RDW-CV,” suggesting synchronized haematological responses to exertion.
Biochemical correlations pre-game included “AST and total protein (TP), alkaline phosphatase (AP) with TP and potassium (K), and magnesium (Mg) with AST and AP,” while post-game, “positive correlations emerged between ALT and AP, CREA and AST, and Mg and AST,” indicating muscular and metabolic adaptation.
Hormonal correlations were minimal in winners, with only a pre-game “negative association between ACTH and T4.” In contrast, losing teams showed complex pre-game hormonal interactions, such as “positive correlations between cortisol and beta-endorphin, epinephrine and cortisol,” and “negative correlations between cortisol and T3,” reflecting heightened anticipatory stress.
The risk of bias ranking for this paper is "Moderate": the study is well-designed and methodologically sound, but limitations in sample diversity, potential confounders, and interpretation of complex physiological data suggest a moderate overall risk of bias.

3.3.3. works that didn’t find evidence of correlation

- **Effects of Owner–Dog Relationship and Owner Personality on Cortisol Modulation in Human–Dog Dyads**[35]

The authors investigated cortisol modulation in human–dog dyads, examining how relational and personality factors influence physiological responses across species. Twenty-two dyads, composed of intact male dogs and their male or female owners, participated in three experimental sessions involving behavioural tasks and salivary cortisol sampling. Saliva samples were collected at 20-minute intervals during meetings and on control days, allowing for assessment of both basal and reactive cortisol levels.
Cortisol concentrations were analysed using enzyme immunoassays, and statistical procedures included general linear models and nonparametric tests. The study explored associations between dyadic attachment dimensions, relationship quality, and personality traits with cortisol levels. Salivary cortisol values were compared across time points and conditions, including routine interactions and structured challenge tasks.
The authors report that “no correlations between owner and dog morning salivary cortisol values were found over control days”, and that “owners and their dogs did not differ in their reactions to the four test situations,” with no significant changes in cortisol levels before and after tasks such as the “Picture,” “Veterinarian Check,” “Bridge,” and “Threat”. However, during the initial home visit, both dogs and male owners exhibited elevated cortisol responses, suggesting a shared physiological reaction to social intrusion.
The risk of bias ranking for this paper is "Moderate to High": the study is methodologically sound and innovative, but limited by small, homogeneous samples and exploratory design, suggesting a moderate overall risk of bias.

- **Psychobiological Factors Affecting Cortisol Variability in Human-Dog Dyads** [36]

The authors investigated psychobiological factors influencing intra-individual cortisol variability in human–dog dyads, using salivary cortisol as a biomarker of stress regulation. A total of 132 owner-dog pairs participated in two laboratory sessions involving standardized social and environmental challenges. Saliva samples were collected from both partners before and after each task, and on a control day, to calculate the individual coefficient of variation (iCV) of cortisol as an index of adaptive stress modulation.
Cortisol concentrations were analysed using enzyme immunoassays, and statistical models assessed the influence of personality traits, attachment styles, and dyadic gender combinations on cortisol variability. The authors report that "the owner's individual coefficient of variation of cortisol (iCV) did not correlate with the dog's iCV", indicating no direct physiological coupling across dyads. However, dogs of owners scoring high in Neuroticism exhibited lower cortisol variability, and dogs of owners with insecure-ambivalent attachment styles or separation anxiety toward humans also showed reduced iCV.
While cortisol responses varied across tasks, no consistent pattern of synchronized reactivity emerged. Instead, individual and relational factors appeared to modulate stress physiology independently within each species. The authors conclude that "both owner and dog social characteristics influence dyadic cortisol variability, with the human partner being more influential than the dog," suggesting that relational dynamics may shape physiological stress regulation without necessitating direct hormonal coupling.
The risk of bias ranking for this paper is "Moderate": this is a well-designed and statistically robust study with thoughtful interpretation. Minor limitations in sample diversity and environmental control suggest a moderate overall risk of bias.

- **Heart rate variability responses of horses and veterans with post-traumatic stress disorder to ground-based adaptive horsemanship lessons: a pilot study** [37]

The authors investigated heart rate variability (HRV) responses in human–horse dyads during ground-based adaptive horsemanship (AH) sessions, focusing on veterans with post-traumatic stress disorder (PTSD). Electrocardiogram (ECG) data were collected from veterans and horses during weekly 30-minute AH lessons over an eight-week period, as well as during pre- and post-intervention resting and human interaction tasks. HRV metrics included heart rate (HR), root mean square of successive differences (RMSSD), and low frequency to high frequency power ratios (LF/HF), processed using the Pan-Tompkins algorithm and spectral analysis.
To assess potential physiological co-modulation, Pearson correlation coefficients were calculated weekly for HR and HRV measures between veterans and horses. The authors report that "no significant correlations were observed between AH horse and veteran heart rates or HRV measures (LF/HF, RMSSD) during any week or time point after applying a Bonferroni correction" . HR was lowest in both species during the initial 10 minutes of sessions, likely reflecting low-intensity grooming activity. RMSSD and LF/HF varied across weeks in veterans, suggesting transient shifts in autonomic balance, but no consistent trends were observed in horses.
Resting LF/HF ratios were significantly higher in veterans compared to non-veterans, indicating sustained sympathetic activation. However, no changes in HRV were detected across pre- and post-intervention time points. The absence of significant physiological coupling between horses and veterans suggests that, under the conditions tested, HRV responses were modulated independently within each species.
The risk of bias ranking for this paper is "Moderate": despite strong methodology and transparent reporting, the small sample size, lack of pre-registration, and limited control of confounders suggest a moderate overall risk of bias.

References

[1] A. Lanata, A. Guidi, G. Valenza, P. Baragli, E. P. Scilingo, Quantitative heartbeat coupling measures in human-horse interaction, in: 2016 38th Annual International Conference of the IEEE Engineering in Medicine and Biology Society (EMBC), IEEE, IEEE, Orlando, FL, USA, 2016. doi:10.1109/EMBC.2016.7591286.

[2] A. Lanata, A. Guidi, G. Valenza, P. Baragli, E. P. Scilingo, The role of nonlinear coupling in Human-Horse Interaction: A preliminary study, in: 2017 39th Annual International Conference of the IEEE Engineering in Medicine and Biology Society (EMBC), IEEE, IEEE, Seogwipo, 2017. doi:10.1109/EMBC.2017.8037075.

[3] A. Guidi, A. Lanata, P. Baragli, G. Valenza, E. Scilingo, A Wearable System for the Evaluation of the Human-Horse Interaction: A Preliminary Study, Electronics (Sep. 2016). doi:10.3390/electronics5040063.

[4] A. L. Callara, C. Scopa, L. Contalbrigo, A. Lanatà, E. P. Scilingo, P. Baragli, A. Greco, Unveiling directional physiological coupling in human-horse interactions, iScience (Sep. 2024). doi:10.1016/j.isci.2024.110857.

[5] W. Ren, S. Yu, K. Guo, C. Lu, Y. Q. Zhang, Disrupted Human–Dog Interbrain Neural Coupling in Autism-Associated *Shank3* Mutant Dogs, Advanced Science (Nov. 2024). doi:10.1002/advs.202402493.

[6] S. Wienhold, L. Bär, Z. Ringleb, V. Zirpel, A. Gomolla, B. F. Denk, N. Volkmer, R. J. Gaertner, E. S. C. Klink, J. C. Pruessner, The relationship of early life adversity and

physiological synchrony within the therapeutic triad in horse-assisted therapy, Journal of neural transmission (May 2025). doi:10.1007/s00702-025-02947-7.

[7] A. Baldwin, B. Rector, A. Alden, Physiological and Behavioral Benefits for People and Horses during Guided Interactions at an Assisted Living Residence, Behavioral Sciences (Sep. 2021). doi:10.3390/bs11100129.

[8] A. L. Baldwin, L. Walters, B. K. Rector, A. C. Alden, Effects of Equine Interaction on Mutual Autonomic Nervous System Responses and Interoception in a Learning Program for Older Adults (2023).
URL https://docs.lib.purdue.edu/paij/vol6/iss1/3/

[9] T. R. N. Holder, C. Nichols, E. Summers, D. L. Roberts, A. Bozkurt, Exploring the Dynamics of Canine-Assisted Interactions: A Wearable Approach to Understanding Interspecies Well-Being, Animals (2024). doi:10.3390/ani14243628.

[10] A. P. Buttner, B. Thompson, R. Strasser, J. Santo, Evidence for a synchronization of hormonal states between humans and dogs during competition, Physiology & Behavior (Aug. 2015). doi:10.1016/j.physbeh.2015.04.010.

[11] K. Nomoto, T. Hashimoto, M. Nagasawa, T. Kikusui, Dog's breath rhythm was drawn into owner's breath rhythm, Advanced Robotics (Jul. 2024). doi:10.1080/01691864.2024.2369795.

[12] G. E. Gnanadesikan, K. M. King, E. Carranza, A. C. Flyer, G. Ossello, P. G. Smith, N. G. Steklis, H. D. Steklis, C. S. Carter, J. J. Connelly, M. Barnett, N. Gee, S. R. Tecot, E. L. MacLean, Effects of human-animal interaction on salivary and urinary oxytocin in children and dogs, Psychoneuroendocrinology (2024). doi:10.1016/j.psyneuen.2024.107147.

[13] H. Harvie, A. Rodrigo, C. Briggs, S. Thiessen, D. M. Kelly, Does stress run through the leash? An examination of stress transmission between owners and dogs during a walk, Animal cognition (Mar. 2021). doi:10.1007/s10071-020-01460-6.

[14] A.-S. Sundman, E. Van Poucke, A.-C. Svensson Holm, Å. Faresjö, E. Theodorsson, P. Jensen, L. S. V. Roth, Long-term stress levels are synchronized in dogs and their owners, Scientific Reports (Jun. 2019). doi:10.1038/s41598-019-43851-x.

[15] A. Byrne, G. Arnott, Empathy or Apathy? Investigating the influence of owner stress on canine stress in a novel environment, Applied Animal Behaviour Science (Oct. 2024). doi:10.1016/j.applanim.2024.106403.

[16] A. Koskela, H. Törnqvist, S. Somppi, K. Tiira, V.-L. Kykyri, L. Hänninen, J. Kujala, M. Nagasawa, T. Kikusui, M. V. Kujala, Behavioral and emotional co-modulation during dog–owner interaction measured by heart rate variability and activity, Scientific reports (Oct. 2024). doi:10.1038/s41598-024-76831-x.

[17] A. C. Jones, R. A. Josephs, Interspecies hormonal interactions between man and the domestic dog (Canis familiaris), Hormones and Behavior (Sep. 2006). doi:10.1016/j.yhbeh.2006.04.007.

[18] J. Yorke, W. Nugent, E. Strand, R. Bolen, J. New, C. Davis, Equine-assisted therapy and its impact on cortisol levels of children and horses: a pilot study and meta-analysis, Early Child Development and Care (Jul. 2013). doi:10.1080/03004430.2012.693486.

[19] M. Katayama, T. Kubo, T. Yamakawa, K. Fujiwara, K. Nomoto, K. Ikeda, K. Mogi, M. Nagasawa, T. Kikusui, Emotional Contagion From Humans to Dogs Is Facilitated by Duration of Ownership, Frontiers in Psychology (Jul. 2019). doi:10.3389/fpsyg.2019.01678.

[20] L. A. McDuffee, W. J. Montelpare, C. LeBlanc, Psychophysiological effects of equine-facilitated psychotherapy on Veterans with PTSD and their horse partners, Journal of Military, Veteran and Family Health (Jun. 2024). doi:10.3138/jmvfh-2023-0063.

[21] M. Nagasawa, M. Saito, H. Hirasawa, K. Mogi, T. Kikusui, Dogs showed lower parasympathetic activity during mutual gazing while owners did not, The Journal of Physiological Sciences (2023). doi:10.1186/s12576-023-00863-7.

[22] M. Nagasawa, S. Mitsui, S. En, N. Ohtani, M. Ohta, Y. Sakuma, T. Onaka, K. Mogi, T. Kikusui, Oxytocin-gaze positive loop and the coevolution of human-dog bonds (2015). doi:10.1126/science.1261022.

[23] L. Handlin, K. Uvnäs-Moberg, E. Hydbring-Sandberg, M. Ejdebäck, A. Nilsson, Associations between the Psychological Characteristics of the Human–Dog Relationship and Oxytocin and Cortisol Levels, Anthrozoös (Jun. 2012). doi:10.2752/175303712X13316289505468.

[24] I. Janczarek, W. Kędzierski, A. Stachurska, I. Wilk, Emotional reactions of horses and trainers during natural method training / Reakcje emocjonalne koni i trenerów podczas treningu metodami naturalnymi, Annals of Animal Science (Mar. 2013). doi:10.2478/aoas-2013-0008.

[25] K. Strzelec, W. Kędzierski, A. Bereznowski, I. Janczarek, K. Bocian, M. Radosz, Salivary Cortisol Levels in Horses and their Riders During Three-Day-Events, Bulletin of the Veterinary Institute in Pulawy (Jun. 2013). doi:10.2478/bvip-2013-0042.

[26] J. Hockenhull, T. J. Young, S. E. Redgate, L. Birke, Exploring Synchronicity in the Heart Rates of Familiar and Unfamiliar Pairs of Horses and Humans Undertaking an In-Hand Task, Anthrozoös (Sep. 2015). doi:10.1080/08927936.2015.1052284.

[27] O.-D. Kang, Y.-M. Yun, Influence of Horse and Rider on Stress during Horse-riding Lesson Program, Asian Australas. J. Anim. Sci (Mar. 2016). doi:10.5713/ajas.15.1068.

[28] M. G. Ryan, A. E. Storey, R. E. Anderson, C. J. Walsh, Physiological Indicators of Attachment in Domestic Dogs (Canis familiaris) and Their Owners in the Strange Situation Test, Frontiers in Behavioral Neuroscience (Jul. 2019). doi:10.3389/fnbeh.2019.00162.

[29] J. Wojtaś, M. Karpiński, P. Czyżowski, Salivary Cortisol Interactions in Search and Rescue Dogs and Their Handlers, Animals (Apr. 2020). doi:10.3390/ani10040595.

[30] E. K. Grigg, S. Liu, D. G. Dempsey, K. Wong, M. Bain, J. J. Sollers, R. Haddock, L. R. Kogan, J. A. Barnhard, A. A. Tringali, A. P. Thigpen, L. A. Hart, Assessing the Relationship Between Emotional States of Dogs and Their Human Handlers, Using Simultaneous Behavioral and Cardiac Measures, Frontiers in Veterinary Science (2022). doi:10.3389/fvets.2022.897287.

[31] J. Wojtaś, A. Garbiec, M. Karpiński, P. Skowronek, A. Strachecka, Are Hair Cortisol Levels of Humans, Cats, and Dogs from the Same Household Correlated?, Animals (Jun. 2022). doi:10.3390/ani12111472.

[32] M. Friend, M. Nicodemus, C. Cavinder, C. Lemley, P. Prince, K. Holtcamp, R. Swanson, Physiology of human-horse interactions during substance withdrawal within psychotherapy participants (Oct. 2023). doi:10.1163/17552559-20230023.

[33] A. Naber, L. Kreuzer, R. Zink, E. Millesi, R. Palme, K. Hediger, L. Glenk, Heart rate and salivary cortisol as indicators of arousal and synchrony in clients, therapy horses and therapist in equine-assisted therapy, Complementary Therapies in Clinical Practice (May 2025). doi:10.1016/j.ctcp.2025.101937.

[34] A. Rişvanli, İ. Şen, K. Canuzakov, A. Tulobayev, A. Taş, R. Salykov, N. Ceylan, Ü. Türkçapar, U. Alimov, A. Kazakbayeva, A. Cunuşova, N. Abdimnap Uulu, B. F. Yüksel, M. Turanli, M. Uz, M. Bayraktar, N. Ruzikulov, The effect of victory and defeat on the correlations of stress parameters between the horse and rider in kök-börü equestrian teams, Veterinary Medicine & Science (May 2025). doi:10.1002/vms3.70356.

[35] I. Schöberl, M. Wedl, B. Bauer, J. Day, E. Möstl, K. Kotrschal, Effects of Owner–Dog Relationship and Owner Personality on Cortisol Modulation in Human–Dog Dyads, Anthrozoös (Jun. 2012). doi:10.2752/175303712X13316289505422.

[36] I. Schöberl, M. Wedl, A. Beetz, K. Kotrschal, Psychobiological Factors Affecting Cortisol Variability in Human-Dog Dyads, PLoS ONE (Feb. 2017). doi:10.1371/journal.pone.0170707.

[37] E. M. Rankins, B. E. Faremi, K. Hartmann, A. Quinn, H. F. Posada-Quintero, K. H. McKeever, K. Malinowski, Heart rate variability responses of horses and veterans with post-traumatic stress disorder to ground-based adaptive horsemanship lessons: a pilot study, Translational Animal Science (Jan. 2025). doi:10.1093/tas/txaf019.
